# Genotyping of selected germline adaptive immune system loci using short-read sequencing data

**DOI:** 10.1101/gr.280314.124

**Published:** 2025-09

**Authors:** Michael K.B. Ford, Ananth Hari, Meredith Yeager, Lisa Mirabello, Stephen Chanock, Ibrahim Numanagić, COVNET Consortium, S. Cenk Sahinalp

**Affiliations:** 1 National Cancer Institute, National Institutes of Health, Bethesda, Maryland 20892, USA;; 2 Department of Electrical Engineering, University of Maryland, College Park, Maryland 20742, USA;; 3 Department of Computer Science, University of Victoria, British Columbia V8P 5C2, Canada

## Abstract

As we enter the age of personalized medicine, healthcare is increasingly focused on tailoring diagnoses and treatments based on patients’ genetic and environmental circumstances. A critical component of a person's physiological makeup is their immune system, but individual genetic variation in many immune system genes has remained resistant to analysis using classical whole-genome or targeted sequencing approaches. In particular, germline adaptive immune system genes, like immunoglobulin (*IG*) and T cell receptor (*TR*) genes, are particularly hard to genotype using classic reference-based methods owing to their highly repetitive and homologous nature. In this paper, we present ImmunoTyper2, a new computational toolkit for genotyping the variable genes of the *IG* lambda and kappa, and the *TR* loci with short-read whole genome sequence data, using an integer linear programming formulation, as an update to the ImmunoTyper-SR suite, which focused on *IGHV* region only. We evaluate its genotyping performance using Mendelian concordance analysis in 590 trios from the 1000 Genomes Project, benchmarking 40 samples against HPRC assembly-derived genotypes, and assessing robustness through sequencing depth analysis and parameter sensitivity tests. We introduce allele call confidence metrics to help quantify reliability. We also perform a prospective disease association study, applying ImmunoTyper2 to a WGS data set from a cohort of 461 COVID-19 patients from the COVNET Consortium to demonstrate how it can be applied to investigate genetic associations with disease.

Identifying the genetic determinants of disease has been one of the foremost endeavors of the genomic era. Led by genome-wide association studies (GWAS), there have been numerous discoveries of phenotype-associated genetic traits, in areas ranging from cancer (Liang et al. 2020) to drug metabolism (Motsinger-Reif et al. 2013). GWAS typically use single-nucleotide polymorphisms (SNPs) in genomes to relate to various phenotypes, including disease associations, using statistical methods. However, there are challenges with this approach; impactful rare variants might not be included in the analysis, whereas the common variants that are the main focus may not have any true functional relevance (Koboldt et al. 2013). This has led to a shift in the focus of disease association studies toward intentionally targeting functionally relevant loci. GWAS can also be challenged by loci that are missing entirely, as some areas of the genome are too poorly characterized for inclusion in SNP arrays, the primary data-generation method for GWAS. Even using whole-genome sequence (WGS) to call variants can fail in complex, repetitive, or poorly characterized loci.

The human immune system contains many loci that are both functionally relevant and difficult to characterize. Immunoglobulin (*IG*)-like loci—such as the *IG* heavy chain, light chains, and T cell receptor (*TR*) loci—encode the genes responsible for antibodies/B cell receptors as well as *TR*s. However, these contain many gene copies, are highly repetitive, and are host to a variety of structural variants (Watson and Breden 2012; Watson et al. 2013, 2017; Gadala-Maria et al. 2019; Rodriguez et al. 2020; for graphical depictions of the locus organization, see Lefranc and Lefranc 2001a,b). These features make germline genotyping with classic array or short-read-based approaches very challenging.

Although short-read WGS approaches for *IG*-like genotyping are problematic, there are alternative methods available. Germline genotype inference using adaptive immune receptor repertoire sequencing (AIRR-Seq) is an established approach, with many published bespoke tools (Corcoran et al. 2016; Gadala-Maria et al. 2019; Peres et al. 2019; Ralph and Matsen IV 2019). Another option is probe-based target sequencing, with either long reads and de novo assembly, as utilized in the IGenotyper approach (Rodriguez et al. 2020, 2022; Gibson et al. 2023; Engelbrecht et al. 2024), or short reads (Lin et al. 2022). Studies using these approaches have successfully found that germline *IG* variants affect immune receptor repertoire dynamics (Rodriguez et al. 2023) and have association to a wide variety of disease such as cancer (Cui et al. 2021), autoimmune disorders (Cho et al. 2003; Parks et al. 2017; Johnson et al. 2021), and infectious diseases/vaccine responses (Avnir et al. 2016; Yacoob et al. 2016; Yeung et al. 2016; Lee et al. 2021). However, these tools require specialized, and therefore resource-intensive, sequencing and analysis protocols and cannot be applied to the extensive available databases of short-read WGS data. As a result, the human immune receptor loci have largely been ignored by disease association studies (Collins et al. 2020).

Germline genotyping of the human immune system's receptor genes is challenged by the complexity and homology of these loci. The *IG* loci include the kappa (*IGK*) and lambda (*IGL*) light-chain variable genes, whereas the *TR* loci comprise the alpha (*TRA*), beta (*TRB*), delta (*TRD*), and gamma (*TRG*) variable genes. All these loci share the same fundamental structure, containing multiple copies of different classes of short genes, which are ultimately recombined through V(D)J recombination to encode the receptor protein. Of these genes, the variable class is a primary determinant of receptor binding structural diversity. These genes are highly duplicated and contain many pseudogenes, in some cases located on other chromosomes, as summarized in Table 1. These duplications, in conjunction with high sequence similarity and short length (on average, 280–300 bp), make these important genes challenging to genotype with short-read WGS data.

**Table 1.** Summary of immunoglobulin and T cell receptor variable gene loci

	Genes		Alleles	
Locus	Total	Functional	Total	Functional
*IGH*	203	56	677	327
*IGK*	109	41	170	79
*IGL*	80	33	175	91
*TRA*	61	45	135	98
*TRB*	77	48	168	112
*TRD*	3	3	6	6
*TRG*	14	6	24	11

One approach employed to genotype complex and repetitive loci is to use combinatorial optimization to assign reads and determine genotypes. This has been used in pharmacogenetics with tools like Cypiripi (Numanagić et al. 2015) and ALDY (Numanagić et al. 2018; Hari et al. 2023), HLA typing using OptiType (Szolek et al. 2014), killer cell *IG*-like receptor (KIR) genotyping with Geny (Zhou et al. 2024), and a variety of polymorphic loci with Locityper (these loci include the *TRBV9* gene; however, the remaining 47 functional *TRBV* genes and 29 pseudogenes have not been investigated by Locityper) (Prodanov et al. 2024). These methods leverage techniques like integer linear programming (ILP) and clique enumeration to resolve ambiguous read assignments across highly similar gene sequences. Our previous work employed a similar framework to genotype germline *IG* heavy-chain variable (*IGHV*) genes using long-read WGS (Ford et al. 2020) and short-read WGS (Ford et al. 2022), implemented in the ImmunoTyper-SR tool. However, to date there are no equivalent tools for the *IG* light-chain (*IGLV*, *IGKV*) or *TR* (*TRAV*, *TRBV*, *TRGV*, *TRDV*) variable gene loci.

In this paper, we present ImmunoTyper2, expanding upon our previous work to enable genotyping of the variable genes across all *IG* light-chain (*IGK*, *IGL*) and *TR* (*TRA*, *TRB*, *TRD*, *TRG*) loci using short-read WGS. This comprehensive suite facilitates the use of widely available WGS data for immunogenomic association studies. We performed extensive validation, including (1) analyzing Mendelian concordance in 590 1000 Genomes Project (1kGP) trios (Byrska-Bishop et al. 2022), (2) comparing against orthogonal long-read assembly data for *IGLV* and *TRAV* alleles in 1 kGP samples, (3) benchmarking 40 1kGP samples using Human Pangenome Reference Consortium (HPRC) assemblies as the ground truth, (4) assessing the impact of sequencing depth (10×, 20×, 30×) on accuracy using downsampled HPRC data, (5) evaluating parameter sensitivity to confirm robustness, (6) developing a novel allele call confidence metric based on ILP solution stability, and (7) providing detailed case studies of read assignments. Finally, we demonstrate its application in a disease association context using a cohort of 461 COVID-19 patients from the COVNET Consortium.

## Results

### Mendelian concordance analysis reveals strong concordance across immune loci

**Figure 1.**
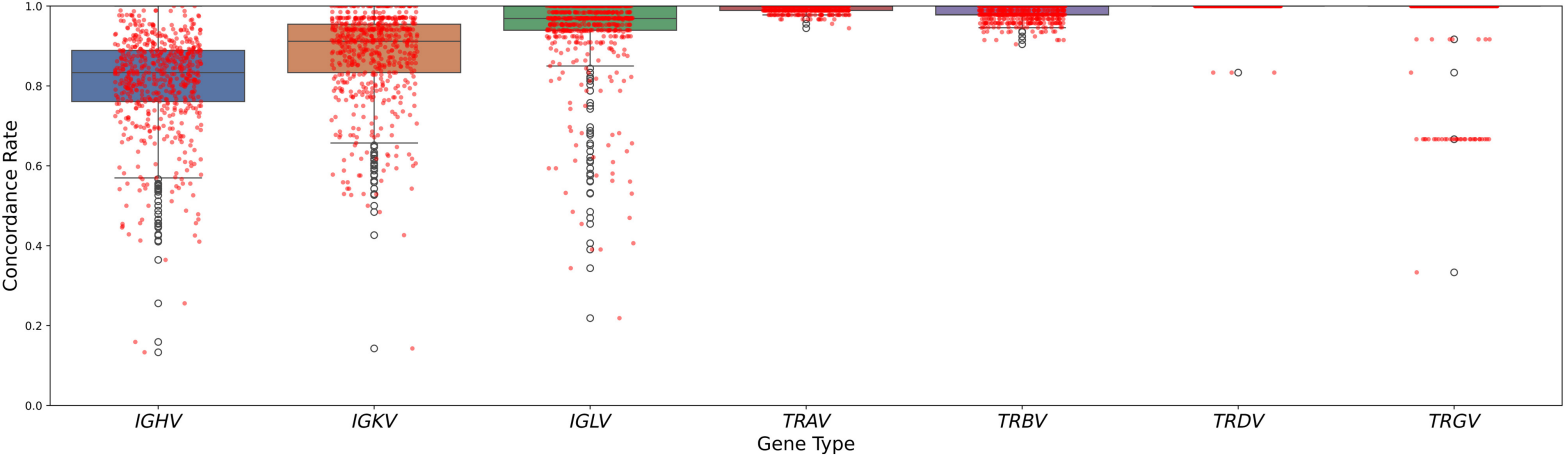
Distribution of Mendelian concordance rates for functional variable gene rates across immune loci. Box plots show the median, quartiles, and outliers for each locus type. Note the distinct clustering of trio concordance in *TRGV* is because that locus has only six genes; for example, a single two-copy gene discordance causes a concordance value of 5/6.

We applied ImmunoTyper2 to genotype 590 parent–child trios from the 1kGP. Processing times varied by locus, ranging from 12 min for *TRAV* and *TRBV* to 63 min for *IGHV*, with read extraction and mapping steps accounting for the majority of computational time. To evaluate genotyping accuracy, we analyzed Mendelian inheritance patterns across all trios (Fig. 1). *TR* loci demonstrated exceptionally high mean concordance rates: *TRAV*, 0.99; *TRBV*, 0.98; *TRGV*, 0.98; and *TRDV*, 1.0. *IG* loci showed more variation in concordance rates: *IGLV*, 0.94; *IGKV*, 0.88; and *IGHV*, 0.74. The lower concordance rate observed for *IGHV* is consistent with the known complexity and prevalence of duplications in this region, which makes the problem of resolving ambiguous read mappings more challenging.

**Figure 2.**
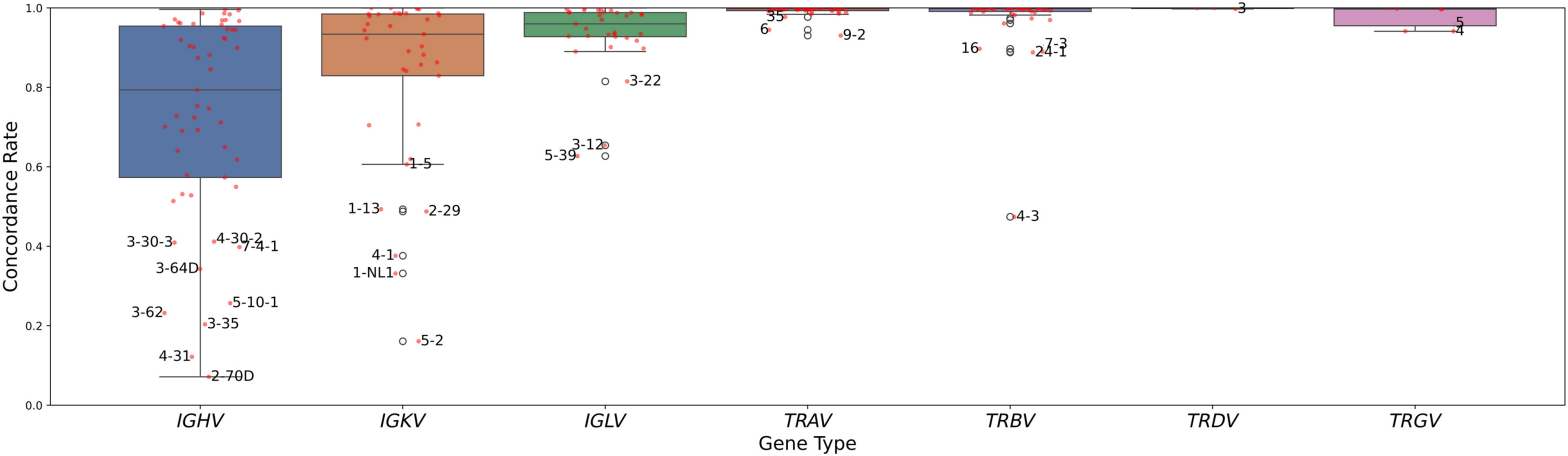
Mean gene-level concordance distribution across all trios, stratified by locus type, in which each point represents a particular gene. Genes with mean concordance rates of one or more SD from the mean are labeled.

**Figure 3.**
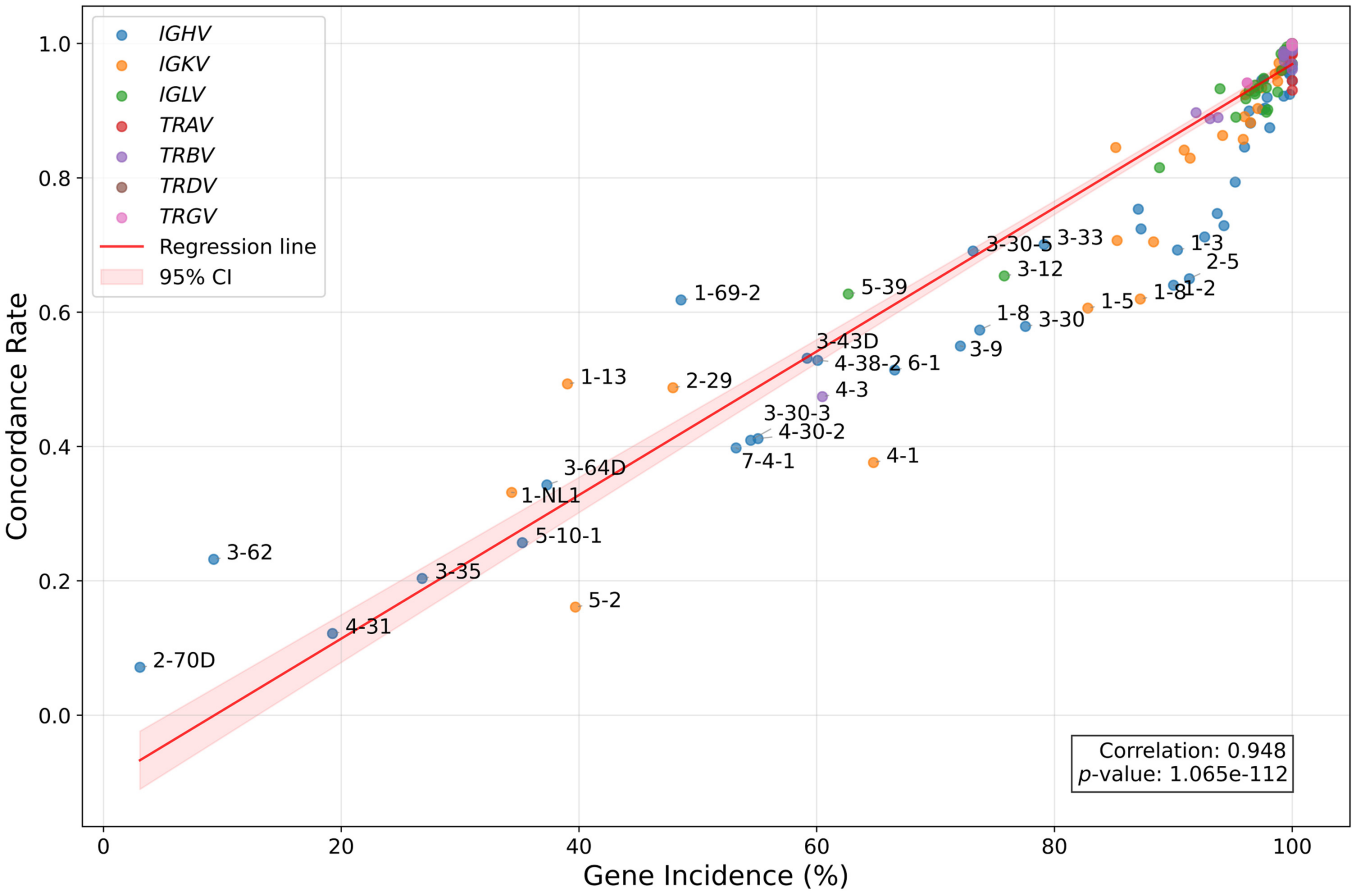
Relationship between mean concordance across all trios for each gene and the fraction of trios in which the gene is present. Linear regression reveals a strong association, indicating rare alleles have lower concordance across trios. Labeled genes have mean concordance rate values at least 1 SD below the mean (calculated across all gene types).

Analysis at the individual gene level revealed substantial variation in concordance rates (Fig. 2), with most genes showing high concordance but with notable outliers occurring primarily in *IG* loci. These outlier genes typically exhibited low prevalence across the trio samples (Fig. 3). Indeed, we observed a strong correlation (*r* = 0.948, *P* = 1.065 × 10^−112^) between a gene's prevalence across trios and its concordance within individual trios, suggesting that genotyping rarer alleles presents additional challenges in these complex genomic regions, potentially impacting concordance.

### Impact of potential *IG* rearrangements in lymphoblastoid cell lines on genotyping concordance

An important consideration in interpreting the concordance rates is the nature of the samples analyzed. The 1kGP samples are lymphoblastoid cell lines (LCLs) derived from B cells, which undergo V(D)J rearrangement, leading to read dropout in *IG* regions (Rodriguez et al. 2021; Lin et al. 2025). To investigate this effect, we examined the relationship between trio concordance and the maximum difference in recruited reads (normalized by sequencing depth) between the child and the two parents within each trio for *IGHV* (Fig. 5, below), *IGLV*, and *IGKV* (Supplemental Fig. 1). We found a statistically significant correlation in all three *IG* variable gene types, which suggests the presence of read dropout owing to V(D)J rearrangement. This read dropout is likely to reduce the accuracy of ImmunoTyper2, which enforces a read-depth-consistency constraint in its read-assignment-optimization model.

### Genotyping concordance varies according to population

**Figure 4.**
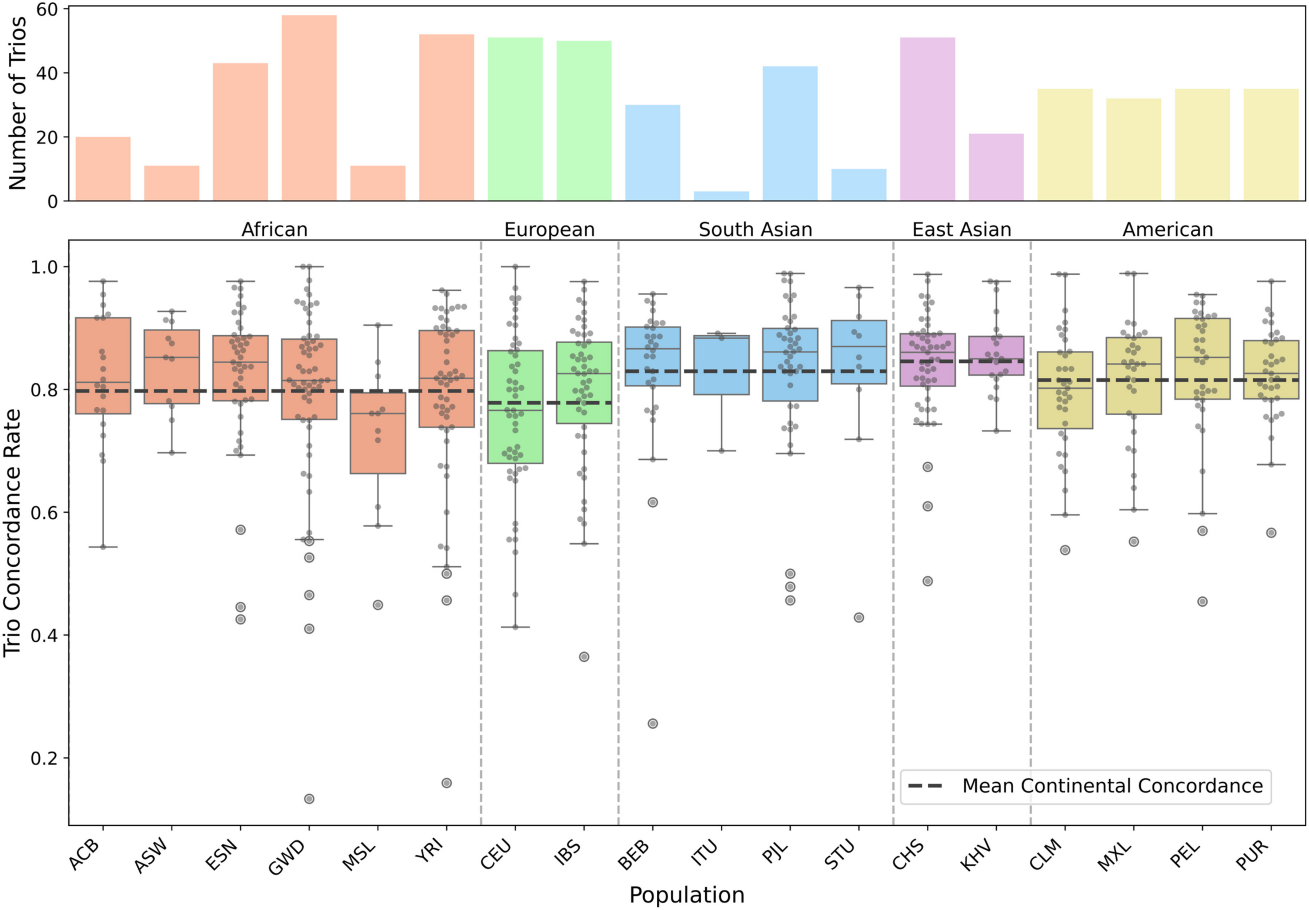
*IGHV* concordance rates across populations and continents, demonstrating significant variation in genotyping accuracy among different ethnic groups (for population codes, see Supplemental Table 1).

We observed significant variation in concordance rates across different ethnic populations (Fig. 4; for population codes, see Supplemental Table 1). A Kruskal–Wallis test confirmed a statistically significant association (*P* = 1.97 × 10^−2^) between concordance rates and population groups, although the biological mechanisms driving this variation remain to be determined. This observation suggests potential population-specific differences in immune locus architecture or complexity that warrant further investigation.

### Comparison with long-read assembly data supports performance in *TRA* and *IGL* loci

To further validate these concordance rates, we compared our genotyping results against high-quality assembly contigs generated from Pacific Biosciences (PacBio) sequencing (Rodriguez et al. 2020). Following our previous evaluation approach for *IGHV* (Ford et al. 2022), we assessed allele presence calls in 15 samples for *IGLV* and 12 samples for *TRAV*, respectively. Because the assembly contigs contain overlapping sequence, their CNV calls for given gene copies may not be accurate. As a result, we evaluated allele-presence accuracy, which does not take into account copy-number variation (CNV) of any given gene. The precision and recall values obtained from this comparison (Table 2) closely mirror the concordance rates observed in our 1kGP trio analysis, providing independent support for our method's performance using orthogonal data.

**Table 2.** Summary of allele presence call accuracy on 1000 Genomes samples (*IGLV*: *n* = 15; *TRAV*: *n* = 12) using orthogonal long-read assembly data as the ground truth

	Mean		Median	
Gene type	Precision	Recall	Precision	Recall
*IGLV*	0.928	0.934	0.971	0.971
*TRAV*	0.916	0.991	0.925	1.0

For accuracy figures with copy-number estimates for each allele, see the Supplemental Figures.

### Genotyping accuracy on HPRC samples

To further evaluate ImmunoTyper2 performance, we computed allele-level precision and recall against Digger-derived genotypes for 40 HPRC samples (Table 3). ImmunoTyper2 demonstrated strong performance across most loci when benchmarked against Digger-derived genotypes, with median precision exceeding 0.79 for all gene types except *TRDV* (Table 3). The notably low precision observed in *TRDV* resulted from the Digger annotation tool providing only *TRDV2* allele annotations, whereas ImmunoTyper2 identified alleles across *TRDV1*, *TRDV2*, and *TRDV3* regions.

**Table 3.** Comparison of ImmunoTyper2 accuracy on HPRC samples as different sequencing depths

Gene type	Full 30×		20× Downsampling		10× Downsampling	
	Precision	Recall	Precision	Recall	Precision	Recall
*IGHV*	0.836	0.807	0.822	0.800	0.803	0.766
*IGLV*	0.954	0.899	0.955	0.899	0.956	0.888
*IGKV*	0.809	0.722	0.814	0.720	0.814	0.700
*TRAV*	0.787	0.929	0.781	0.924	0.779	0.906
*TRBV*	0.872	0.930	0.873	0.929	0.875	0.923
*TRGV*	0.922	0.832	0.922	0.832	0.931	0.837
*TRDV*	0.293	0.983	0.289	0.983	0.299	0.983

Values are the mean; 30× is complete sequence, and 20× and 10× are downsampled.

Detailed analysis of challenging cases highlighted instances in which alignment (and/or assembly) complexities, for example, involving pseudogenes, significantly impacted genotyping accuracy. For example, as demonstrated in Supplemental Figure 2, the *IGLV2-14* gene from sample HG00438 exhibited substantial divergence between the five reported assembly sequences and their corresponding IMGT allele database references, complicating accurate genotype calling and benchmarking. This divergence may be a result of the noise created by V(D)J rearrangement as discussed in the section “Impact of potential *IG* rearrangement in lymphoblastoid cell lines on genotyping concordance.” ImmunoTyper2 called two alleles for this gene: *01, declared a true positive by Digger, and *04, declared a false positive. As demonstrated in Supplemental Figures 3 and 4, a read alignment pileup of reads assigned to allele *04 showed stronger apparent alignment than the allele *01; alignment to the true allele showed distinct prefix and suffix noise. There a several potential sources of the discordance between the ImmunoTyper2 calls and the Digger annotation of the HPRC reference; the significant sequence divergence between the assembly sequences and IMGT reference sequences makes the inference of the best allele sequence challenging for ImmunoTyper2. Furthermore, the potential presence of the additional novel pseudogenes recognized by Digger may be contributing reads to the read assignment performed by ImmunoTyper2, confounding the correct call. Finally, there could be some potential issues with the assembly sequences themselves. These observations underscore the challenges of accurate genotyping in regions containing multiple closely related pseudogene copies, complicating both genotype calls and benchmarking.

### Empirically optimizing ImmunoTyper2 parameters on HPRC samples

To comprehensively assess the robustness of ImmunoTyper2's genotyping accuracy, we performed a parameter sweep, evaluating the impact of various parameter settings on accuracy. Specifically, we varied the number of landmark groups (four, six, and eight), the number of landmarks per group (four, six, and eight), and the standard deviation depth scaling factor (one, 1.5, and two). The default values used in ImmunoTyper2 are six landmark groups, six landmarks per group, and a standard deviation depth scaling factor of 1.5. We observed that genotyping accuracy was robust across all gene types for these parameter variations. The largest observed difference in F1 score between the default and best-performing parameter set was an increase of 0.018 for *TRDV*. For all other gene types, the differences were considerably smaller, with improvements not exceeding 0.003 when comparing the default parameter values to the best-performing parameter values. These results indicate that ImmunoTyper2 maintains stable accuracy across a wide range of parameter settings and that the default parameters provide robust performance suitable for general applications. The difference in accuracy between the default parameter set and the best-performing parameter set for each gene type is summarized in Supplemental Table 4.

### Genotyping accuracy at lower sequencing depths

Table 3 summarizes the accuracy of ImmunoTyper2 genotyping at the reduced sequencing depths. Precision and recall remained robust across most loci at 20× coverage, showing only slight declines compared with the original 30× sequencing. Notably, *IGLV* and *TRBV* retained high precision and recall even at the reduced coverage levels, with minimal changes observed. At 10× coverage, genotyping accuracy declined for certain loci, especially *IGKV* and *IGHV*, which demonstrated reduced recall compared with at 20× and 30× coverage. The *TRDV* locus consistently exhibited high recall but low precision across all coverage depths owing to Digger annotation being restricted to *TRDV2* alleles, whereas ImmunoTyper2 identifies alleles across the *TRDV1*, *TRDV2*, and *TRDV3* regions. These findings illustrate ImmunoTyper2 maintains robust performance even at lower sequencing depths, enabling applications in lower-cost WGS data sets.

### Genotype call confidence metric improves precision

We evaluated the effectiveness our confidence metric (see Methods section “Confidence metric for allele calls”) by analyzing its performance on HPRC assembly samples described in the section “Genotyping accuracy on HPRC samples.” Specifically, we applied the prefix consistency metric to (copy-number insensitive) allele calls from these samples, grouping them by gene. For each gene, we assessed the proportion of allele calls meeting or exceeding various prefix consistency threshold values. An allele call passed the threshold test if its prefix consistency value was equal to or greater than the given threshold.

To quantify the effectiveness of these thresholds, we calculated the positive predictive value (PPV), the proportion of allele calls that were true positives among those that passed each threshold. We then identified optimal prefix consistency thresholds for each gene by maximizing the F-beta score with β set to 0.5. This approach prioritizes precision (PPV) over recall, aligning with scenarios in which high confidence in positive allele identification is crucial.

The results summarized in Table 4 demonstrate improvements in precision compared with a naive approach. Across the evaluated loci, mean PPVs at optimal thresholds ranged widely, with particularly high values for the *IGLV* (mean PPV = 0.9633) and *TRGV* loci (mean PPV = 0.9342). The proportion of allele calls passing the optimal threshold varied by locus; it was notably lower for *IGHV*, likely because of the increased complexity of the locus, but was generally very high for other loci, lending additional improvements in precision (*TRDV* was excluded owing to the incomplete assembly annotations detailed in the section “Genotyping accuracy on HPRC samples”).

**Table 4.** Mean PPV and proportion of calls passing optimal prefix consistency thresholds by gene type

	PPV		Passing proportion	
Gene type	Mean	Median	Mean	Median
*IGHV*	0.8771	0.9310	0.3390	0.3359
*IGLV*	0.9633	1.0000	0.9621	0.9756
*IGKV*	0.8203	0.8966	0.6982	0.7692
*TRAV*	0.8263	1.0000	0.8786	0.9615
*TRGV*	0.9342	0.9762	0.9880	1.0000

Optimal thresholds selected via F-beta (*β* = 0.5), prioritizing precision. A lower passing proportion in *IGHV* reflects locus complexity.

In the ImmunoTyper2 outputs, we provide allele consistency metrics for each allele call, along with gene-level mean PPV and mean passing proportions at each prefix consistency threshold. This information enables users to perform their own filtering based on desired confidence levels for allele calls.

### Application to COVID-19 disease association study

To demonstrate the utility of our ImmunoTyper2 for disease association studies, we applied logistic regression analysis of the *TRAV*, *IGKV*, and *IGHV* allele genotypes from a cohort of 461 COVID-19 patients. Because of data availability constraints, our analysis was necessarily limited to these specific loci, serving primarily as a proof of concept for our genotyping methodology. Following rigorous statistical approaches, we applied best practices including covariate adjustment for sex, age, and population stratification via principal component analysis. Our initial analysis revealed several alleles with nominally significant associations with COVID-19 clinical outcomes. Specifically, we identified three *IGKV*, four *TRAV*, and six *IGHV* alleles that showed statistically significant associations (*P* < 0.05) when examined individually. However, upon implementing the Benjamini–Hochberg false-discovery rate (FDR) correction to control for multiple hypothesis testing, these associations were no longer statistically significant (*α* = 0.05) (for table of statistics, see Supplemental Table 2). As a result, these prospective associations should be interpreted with caution. Although these findings are constrained by the available genetic data and do not represent a comprehensive investigation into germline immune receptor genetic variation and COVID-19, they do serve to demonstrate the utility and application of ImmunoTyper2 to disease association studies.

## Discussion

*IG* and *TR* loci represent some of the most repetitive regions in the human genome. Their inherent complexity, characterized by extensive duplication, structural variation, and high sequence similarity between gene copies, has historically made them challenging targets for genotyping studies using standard short-read WGS approaches. Despite their critical functional role in adaptive immunity and their documented associations with various diseases, these loci have been notably underutilized in large-scale WGS-based disease association studies precisely because suitable genotyping tools were lacking. This underutilization represents a significant gap in our understanding of genetic contributions to immune-related diseases and responses. Therefore, although ImmunoTyper2 does not achieve perfect accuracy, its ability to genotype these loci from ubiquitous short-read data constitutes a significant advance. The accuracy achieved (typically 80%–99% depending on the locus and validation metric) provides researchers with vastly more genetic information for these critical regions than was previously accessible without specialized, resource-intensive sequencing protocols, thereby enabling new avenues of investigation.

Building upon our previous work on *IGHV* genotyping (Ford et al. 2022), we have expanded our methodology to encompass the complete set of adaptive immune receptor variable gene loci. Our analysis of 590 parent–child trios from the 1kGP demonstrates the robustness of our approach, with high Mendelian concordance rates across most loci. The particularly high concordance observed in *TR* loci (>0.98) suggests these regions may be more amenable to short-read genotyping than previously assumed. The somewhat lower concordance in *IG* loci, especially *IGHV*, likely reflects both their increased structural complexity and the impact of V(D)J rearrangement in the LCL samples analyzed. The strong association between gene incidence and concordance accuracy would suggest that these are rare genes that are underrepresented in the established allele database. Several studies from both germline and repertoire data have discussed the limitations of the IMGT allele database or have identified previously uncharacterized novel alleles (Gadala-Maria et al. 2019; Rodriguez et al. 2020; Mikocziova et al. 2021; Peng et al. 2021); the additional evidence of low-incidence genes having lower accuracy motivates future work to utilize alternative allele database sets, such as ORGDB (Christley et al. 2024; Collins et al. 2024). However, some of these low-incidence genes are found in regions without known structural variants and, as a result, would be expected to commonly be present in two copies. In these cases, the low call incidence could be attributed to V(D)J rearrangement, as discussed above, or to some other factor confounding the read-assignment processes.

**Figure 5.**
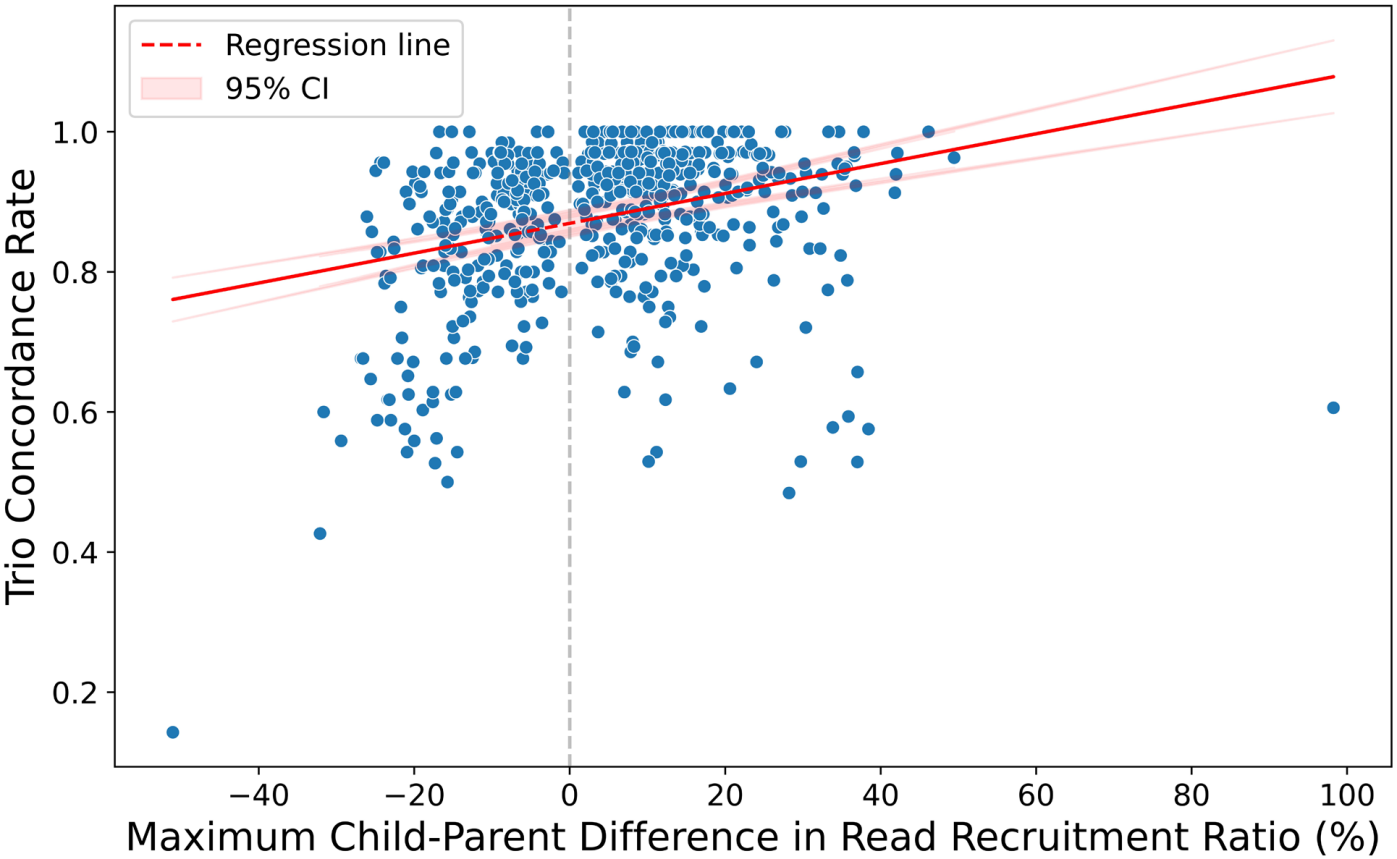
Correlation between trio concordance and maximum difference in *IGHV* read recruitment, normalized depth between child and parent. Each point represents a trio, demonstrating the relationship between read dropout and inheritance pattern consistency. The regression line indicates that a lower read recruitment in any one parent results in a lower trio concordance, with a correlation of 0.357 (*P* = 4.79 × 10^−19^). Similar correlations of 0.222 (*P* = 5.40 × 10^−8^) and 0.301 (*P* = 9.34 × 10^14^) were found with *IGLV* and *IGKV*, respectively (see Supplemental Fig. 1), suggesting the presence of *IG* read dropout, likely owing to rearrangement.

The observed correlation between concordance rates and read recruitment differences within trios (Fig. 5; Supplemental Fig. 1) provides valuable insight into the technical challenges of genotyping *IG* regions from commonly used benchmark data sets. Crucially, all benchmarking in this paper was performed using LCL samples derived from B cells. As discussed in the Results (see section “Impact of potential *IG* rearrangement in lymphoblastoid cell lines on genotyping concordance”) and as documented previously (Rodriguez et al. 2021; Lin et al. 2025), V(D)J rearrangement in these cells leads to somatic deletion and systematic read dropout in *IG* loci. This inherent characteristic of LCLs negatively impacts the genotyping accuracy achievable for *IG* genes within these specific data sets, acting as a limitation of the benchmark samples themselves, not solely the genotyping methodology. The much higher accuracy and concordance rates we consistently observe for *TR* loci (Figs. 1, 2; Tables 2, 3), which do not undergo rearrangement in LCLs, strongly support this interpretation. We anticipate that applying ImmunoTyper2 to more suitable sample types for germline analysis, such as whole blood or non–B cell tissues, would yield higher accuracy for *IG* loci, potentially approaching the excellent levels seen for *TR* loci. This highlights the importance of sample selection for maximizing accuracy in future immunogenomic studies using this tool.

One of the persistent challenges in developing tools for these complex regions has been the limited availability of comprehensive benchmarking resources. The field has historically lacked reliable geno- and haplotyping tools, making it difficult to establish ground-truth data sets for tool validation. Our approach of combining Mendelian concordance analysis (1kGP trios), validation against high-quality assembly contigs (1kGP samples), and benchmarking against Digger calls on HPRC samples provides a multifaceted framework for accuracy assessment. The general consistency across these independent validation approaches supports the reliability of our method. However, the HPRC benchmarking and detailed case studies also highlighted specific challenges, such as discrepancies arising from pseudogenes or limitations in reference annotations (e.g., *TRDV*), underscoring the complexities of both genotyping and validation in these loci. Although our validation is extensive, it is important to note the inherent limitations of our Mendelian calculation approach owing to unphased calls: It does not enforce full haplotype inheritance, only that a pair of alleles in the child can be sourced from the parents. Future work enabling copy phasing would allow for a more comprehensive Mendelian benchmarking scheme and potentially address these current limitations.

The significant variation in concordance rates across populations warrants further investigation. Although our analysis confirms the existence of population-specific differences, understanding the underlying biological or technical factors driving these differences remains an important area for future research. This variation could reflect true population-specific differences in immune locus architecture, or it might point to biases in our current understanding of these regions, in particular the reference sequence data sets, that need to be addressed.

The analysis of COVID-19 outcomes in relation to immune receptor genetic variation, although inconclusive in this study, highlights both the potential and methodological considerations for future disease association studies. Although we identified several nominally significant associations, their disappearance after FDR correction underscores the critical importance of properly accounting for multiple hypothesis testing in genetic-association studies, a consideration that becomes increasingly important as the number of tested alleles grows. This methodological framework, demonstrated here with COVID-19, establishes a foundation for investigating the role of immune receptor genetic variation in a broad spectrum of diseases. Particularly promising candidates for future studies include autoimmune conditions in which germline variation in immune receptors may play a crucial role in disease susceptibility and progression. The high concordance rates achieved by our method enable the retrospective analysis of existing WGS data sets, eliminating the need for specialized sequencing and its associated costs. This is especially valuable given the vast quantity of existing short-read WGS data available across diverse patient cohorts. By providing both the computational tools and analytical framework for immune receptor genotyping from short-read sequencing data, this work opens new avenues for large-scale genetic-association studies that have previously been technically challenging to pursue. Future studies with larger cohorts and more comprehensive genetic data may reveal significant associations that were beyond the scope of our limited data set.

Our results also highlight areas in which further development would be valuable. The lower concordance observed in some highly complex regions, particularly in the *IGHV* locus, suggests that additional strategies may be needed to fully resolve these areas. To help address the inherent uncertainty in challenging regions, we introduced a novel confidence metric based on ILP solution stability, allowing users to filter allele calls based on their desired level of evidence and improving precision, especially when prioritizing high-confidence results. Furthermore, the impact of V(D)J rearrangement on read recruitment indicates that specialized approaches might be needed when working with B cell–derived samples.

In conclusion, this work represents a significant step toward making these traditionally challenging immune loci more accessible to large-scale genetic studies. By enabling reliable genotyping from standard short-read WGS data, our method helps address the historical underutilization of these functionally critical regions in disease association studies, and our application to COVID-19 can serve as a guide for future studies. The comprehensive validation approach we present also provides a framework for evaluating future tools in this space. Furthermore, even when specific allele calls face ambiguity inherent to the locus complexity or sample type, ImmunoTyper2 provides detailed outputs, including read alignments to allele sequences, offering researchers transparency and a basis for deeper investigation when needed, a substantial improvement over standard whole-genome alignment approaches for these regions. The ability to leverage existing WGS data sets for immunogenetic studies, combined with the provided confidence metrics and proper statistical frameworks, opens new possibilities for understanding the role of immune receptor variation in human health and disease. As the field moves forward, this approach can be applied to a wide range of conditions in which immune receptor genetics may play a crucial role. Future studies using appropriate sample types, larger cohorts, and comprehensive genetic data will be well positioned to uncover significant associations that advance our understanding of how immune receptor genetics influence disease susceptibility and progression.

## Methods

### Genotyping of *IG* and *TR* variable genes

ImmunoTyper2 performs germline genotyping of *IG* and *TR* genes in three main steps: (1) *read recruitment* for those reads that may originate from a copy of the gene of interest; (2) *read mapping, assignment, and allele calling* to map the reads to a database of known allele sequences; and (3) *resolving read mapping ambiguity* so that the resultant read assignments are consistent with expected read coverage. Each step is described in detail below.

#### Read recruitment

ImmunoTyper2 takes as input a BAM or CRAM file generated by mapping WGS reads to the reference genome. Reads that have been mapped to the locus relevant to the gene of interest are then extracted. However, it is well known that some *IG* and *TR* loci are highly similar in sequence composition with other loci in the genome; these loci are known as orphons. This creates two possible problematic scenarios: Reads extracted so far may have originated from these orphons, and also, reads that originate from a gene interest may have been erroneously mapped to such orphons in the input BAM or CRAM file. This can lead to an incomplete or erroneous recruitment of reads, which can confound the read coverage consistency we would like to achieve for the output set of alleles.

Although some of the orphon regions have been well characterized in the literature, we have extended their scope by carefully searching for regions of similarity to all loci of interest, namely, *IG* light-chain (*IGKV*, *IGLV*) and *TR* (*TRAV*, *TRBV*, *TRDV*, *TRGV*) genes. For that, we first simulated 100 bp reads at 30× from the IMGT allele databases (Lefranc 2003) for each of these loci using the ART Illumina tool (Huang et al. 2012). We then mapped these reads to the reference genomes using Bowtie 2 (Langmead and Salzberg 2012) and merged all regions in the genome that had contiguous read mappings. We filtered out regions shorter than our expected WGS read length (∼150 bp) and included the remainder in the list of orphons to be used by ImmunoTyper2 for read recruitment. Specifically, ImmunoTyper2 recruits all reads that have been mapped to each orphon region to complement the reads it has recruited from the loci of interest. Currently, ImmunoTyper2 only supports mappings to GRCh38 for all loci (GRCh37 is implemented for *IGHV*, *IGLV* and *TRAV*) and handles all standard chromosome and alt chromosome identifiers.

#### Read mapping, assignment, and allele calling

Read assignment and allele calling generally follows the ImmunoTyper-SR methodology (Ford et al. 2022).

ImmunoTyper2 uses an expanded the IMGT allele database, which now includes uncharacterized pseudogenes from orphon regions in GRCh38, derived from the orphon detection method mentioned earlier; we have also added flanking N's to every allele sequence to aid in clipped mapping.

As a first step, ImmunoTyper2 calculates the expected coverage depth and variance of the data set on Chromosome 1.

Next, each of the recruited reads (as described above) is mapped to the expanded IMGT allele database using Bowtie 2 (Langmead and Salzberg 2012) with the -a ‐‐end-to-end ‐‐very-sensitive -f ‐‐n-ceil C,100,0 ‐‐np 0 as well as ‐‐ignore-quals ‐‐mp 2,2 ‐‐score-min C,-50,0 -L 10 parameters. Notably, these parameters enforce “all-to-all” mapping of the reads to the allele database; as a result of this, each read may end up with more than one mapping allele (these alleles include those of the pseudogenes and the orphons), and some or all of these mappings may be truly ambiguous because of identical mapping quality.

#### Resolving read mapping ambiguity

The ambiguous mappings of the reads are now resolved using an ILP optimization strategy, solved via the Gurobi (Gurobi Optimization 2021) optimization package. The objective of the ILP is to minimize the total edit distance between reads and their allele assignments, with constraints on the read assignments that guarantee that, on each chosen allele, the depth of coverage and variance values of the unambiguously assigned reads *approximately* (within a user defined, additive error tolerance) match the calculated values from Chromosome 1. To speed up computation, the read coverage for each allele is calculated at only a few (uniformly spaced) “landmark” positions. Each read is allowed to be discarded with some fixed additive penalty for the following reason. Although we have expanded the IMGT allele database by adding confounding sequences from GRCh38, it is possible that the sample genome harbors some novel or uncharacterized orphon regions. However, despite not being present in the expanded allele database, reads originating from these regions may still be recruited as they may have been aligned to the recruitment loci in the input file owing to sequence similarity. Our formulation allows these reads to be discarded, reducing their confounding effect on read assignment. We present a more detailed description of the ILP formulation in the Supplemental Materials.

### Confidence metric for allele calls

Allele calls derived from ImmunoTyper2 can exhibit varying degrees of accuracy because of the highly similar sequences present within immunogenomic loci (in particular, *IGHV*), leading to potential ambiguity in read assignment. To assess and quantify the accuracy of individual allele calls, we developed a confidence metric based on the stability of allele presence across alternative optimal solutions of the ILP model. This method leverages the capability of the Gurobi solver to generate near-optimal solutions by iteratively constraining the ILP objective value to be progressively less optimal, creating a pseudobootstrap method to investigate allele call consistency.

Specifically, we first obtained the optimal ILP solution and its corresponding objective value. We then generated alternative solutions by iteratively adding constraints requiring the objective value to be at least 2%, 4%, 6%, and 8% worse than the original optimal value. Each constrained ILP model was solved independently, producing a series of alternative solutions.

Allele call consistency across these solutions was quantified through a metric termed *prefix consistency*. This metric is calculated by sequentially evaluating allele presence, starting from the optimal solution through each progressively constrained alternative solution. The prefix consistency value for an allele was defined as the count of consecutive solution bands (2%, 4%, 6%, etc.) in which the allele was present, starting from the optimal solution. The count was terminated upon encountering the first solution in which the allele was absent. Thus, alleles only present in the optimal solution were assigned a prefix consistency value of zero.

To classify allele calls as either high or low confidence, we empirically determined an appropriate prefix consistency cutoff using allele consistency data from the HPRC validation samples. Alleles with prefix consistency values equal to or greater than this threshold were classified as high confidence, whereas those below the threshold were considered lower-confidence calls.

### Mendelian concordance analysis

To validate the biological accuracy of our genotyping approach, we performed Mendelian inheritance analysis using parent–child trios from the 1kGP (Byrska-Bishop et al. 2022). This data set includes 602 trios, representing 1803 WGS samples that we downloaded from the NCBI Sequence Read Archive (SRA; https://www.ncbi.nlm.nih.gov/sra) using Globus. Of these samples, 12 had corrupted CRAM files that were not readable, resulting in a complete data set of 590 trios.

We performed genotyping across all *IG* and *TR* loci (*IGKV*, *IGLV*, *TRAV*, *TRBV*, *TRDV*, and *TRGV*) using ImmunoTyper2 with default parameters. For each trio, we evaluated Mendelian concordance by examining the inheritance patterns of alleles across each gene. Our concordance calculation methodology was designed to accommodate the complexity of *IG* and *TR* loci, including cases of variable copy number and missing data.

For each gene, concordance was established when the child's alleles could be explained by parental inheritance patterns. When two copies of the gene are called in the child, the gene was considered concordant if the allele from one copy was found in the maternal genotype calls and the allele from the other was found in the paternal. In cases in which a child possessed more than two copies, we considered the inheritance pattern concordant if we could identify a pair of alleles in which one was inherited from each parent. For children with a single allele, concordance was determined by the presence of that allele in at least one parent's genotype. Nonconcordance was recorded in cases in which a child's alleles could not be explained by parental genotypes or in which parental genotype data were missing for the gene in question.

### Comparing allele calls with assembly contigs

Although the Mendelian concordance analysis provides a robust validation of our genotyping approach, we also performed an additional confirmatory step by comparing our allele calls to those derived from long-read assembly contigs. For this ground truth, we use assembly contigs generated with IGenotyper (Rodriguez et al. 2020, 2022; Gibson et al. 2023). We first annotated the contigs by identifying all *IGLV* or *TRAV* allele copies harbored by each contig by mapping the allele sequences from the respective allele database to that contig using Bowtie 2 (Langmead and Salzberg 2012).

Note the contigs provided for this analysis are unphased and unpolished; as a result, they contain duplicate sequences that are not necessarily reflective of true genomic duplication. This can result in an abnormally high CNV count for many of the genes, which could only be fixed by resolving the contig assembly, a task that is beyond the scope of our work. As a result, we look primarily at allele presence and exclude CNVs when comparing our results to the assembly-derived genotype (for a reference, see CNV-sensitive results in Supplemental Table 3). Accuracy was measured using precision and recall for the called alleles.

### Additional genotyping validation with HPRC samples

To further validate ImmunoTyper2, we assessed its performance on 40 HPRC samples with 30× short-read Illumina WGS data from 1000 Genomes Phase 3. Germline genotype calls were initially generated using Digger (Lees et al. 2024). ImmunoTyper2 was then run with default parameters, and its results were compared directly against Digger-derived genotypes. Accuracy metrics, including allele-level precision and recall, were calculated using Digger results as the reference, addressing concerns about systematic biases and confirming ImmunoTyper2's capability in resolving closely related alleles.

### Downsampling of HPRC samples and ground truth assessment

To assess the impact of sequencing depth on genotyping accuracy, we downsampled the HPRC data sets to generate lower coverage samples. Specifically, HPRC samples originally sequenced at approximately 30× coverage were randomly downsampled to two-thirds (20×) and one-third (10×) of their original read counts using SAMtools v1.17, producing data sets of roughly 20× and 10× coverage, respectively. ImmunoTyper2 was then applied to these downsampled data sets using default parameters. Genotyping accuracy at these lower coverage levels was assessed by comparing allele presence calls to the high-coverage (30×) ground-truth allele annotations generated by Digger.

### Association with COVID-19 disease severity

We additionally performed *TRAV*, *IGKV*, and *IGHV* genotyping on short-read WGS collected from a cohort of 461 COVID-19 patients as part of the COVNET Consortium (we were unable to perform the complete *IG* and *TR* genotyping on this data set as we did not have access to those genomic loci at time of writing). Clinical outcomes of the patients were binarized by combining the “moderate” and “severe” categories together as the effect group (*n* = 176) and using the “mild” category as the control group (*n* = 285), and allele genotypes were binarized by presence and absence. Alleles were filtered for low prevalence by ensuring both presence and absence occurred in at least 5% of samples (Hosmer Jr et al. 2013) and complete separation and quasi-separation (Albert and Anderson 1984). We then performed logistic regression (using the statsmodels package) (Seabold and Perktold 2010) for each allele, with presence as the independent variable, binary COVID outcome as the dependent variable, and sex and age included as covariates. We also included the top three SNP-based principal components as covariates to control for population stratification. For each gene type, we performed FDR correction with an alpha value of 0.05 to control for multiple hypothesis testing error (Benjamini and Hochberg 1995).

### Software availability

ImmunoTyper2 is available at GitHub (https://github.com/algo-cancer/ImmunoTyper2). All methods, including scripts to download data, are available at GitHub (https://github.com/michael-ford/ImmunoTyper2-methods) and as Supplemental Code.

## Supplemental Material

Supplement 1

Supplement 2

## References

[GR280314FORC1] Albert A, Anderson JA. 1984. On the existence of maximum likelihood estimates in logistic regression models. Biometrika 71: 1–10. 10.1093/biomet/71.1.1

[GR280314FORC2] Avnir Y, Watson CT, Glanville J, Peterson EC, Tallarico AS, Bennett AS, Qin K, Fu Y, Huang CY, Beigel JH, 2016. IGHV1-69 polymorphism modulates anti-influenza antibody repertoires, correlates with IGHV utilization shifts and varies by ethnicity. Sci Rep 6: 20842. 10.1038/srep20842 26880249 PMC4754645

[GR280314FORC3] Benjamini Y, Hochberg Y. 1995. Controlling the false discovery rate: a practical and powerful approach to multiple testing. J R Stat Soc B (Methodol) 57: 289–300. 10.1111/j.2517-6161.1995.tb02031.x

[GR280314FORC4] Byrska-Bishop M, Evani US, Zhao X, Basile AO, Abel HJ, Regier AA, Corvelo A, Clarke WE, Musunuri R, Nagulapalli K, 2022. High-coverage whole-genome sequencing of the expanded 1000 Genomes Project cohort including 602 trios. Cell 185: 3426–3440.e19. 10.1016/j.cell.2022.08.004 36055201 PMC9439720

[GR280314FORC5] Cho ML, Chen PP, Seo YI, Hwang SY, Kim WU, Min DJ, Park SH, Cho CS. 2003. Association of homozygous deletion of the Humhv3005 and the VH3-30.3 genes with renal involvement in systemic lupus erythematosus. Lupus 12: 400–405. 10.1191/0961203303lu385oa 12765304

[GR280314FORC6] Christley S, Breden F, Burns K, Corrie B, Lees W, Overton J, Peters B, Richardson E, Roskin K, Vita R, 2024. Adaptive immune receptor repertoire knowledge commons: large-scale knowledge for research and innovation (https://airr-knowledge.org). J Immunol 212: 1522_5717–1522_5717. 10.4048/jimmunol.212.supp.1522.5717

[GR280314FORC7] Collins AM, Yaari G, Shepherd AJ, Lees W, Watson CT. 2020. Germline immunoglobulin genes: disease susceptibility genes hidden in plain sight? Curr Opin Syst Biol 24: 100–108. 10.1016/j.coisb.2020.10.011 37008538 PMC10062056

[GR280314FORC8] Collins AM, Ohlin M, Corcoran M, Heather JM, Ralph D, Law M, Martínez-Barnetche J, Ye J, Richardson E, Gibson WS, 2024. AIRR-C IG reference sets: curated sets of immunoglobulin heavy and light chain germline genes. Front Immunol 14: 1330153. 10.3389/fimmu.2023.1330153 38406579 PMC10884231

[GR280314FORC9] Corcoran MM, Phad GE, Bernat NV, Stahl-Hennig C, Sumida N, Persson MA, Martin M, Hedestam GBK. 2016. Production of individualized V gene databases reveals high levels of immunoglobulin genetic diversity. Nat Commun 7: 13642. 10.1038/ncomms13642 27995928 PMC5187446

[GR280314FORC10] Cui M, Huang J, Zhang S, Liu Q, Liao Q, Qiu X. 2021. Immunoglobulin expression in cancer cells and its critical roles in tumorigenesis. Front Immunol 12: 893. 10.3389/fimmu.2021.613530 PMC8024581 33841396

[GR280314FORC11] Engelbrecht E, Rodriguez OL, Shields K, Schultze S, Tieri D, Jana U, Yaari G, Lees WD, Smith ML, Watson CT. 2024. Resolving haplotype variation and complex genetic architecture in the human immunoglobulin kappa chain locus in individuals of diverse ancestry. Genes Immun 25: 297–306. 10.1038/s41435-024-00279-2 38844673 PMC11327106

[GR280314FORC12] Ford M, Haghshenas E, Watson CT, Sahinalp SC. 2020. Genotyping and copy number analysis of immunoglobin heavy chain variable genes using long reads. iScience 23: 100883. 10.1016/j.isci.2020.100883 32109676 PMC7044747

[GR280314FORC13] Ford MK, Hari A, Rodriguez O, Xu J, Lack J, Oguz C, Zhang Y, Oler AJ, Delmonte OM, Weber SE, 2022. ImmunoTyper-SR: a computational approach for genotyping immunoglobulin heavy chain variable genes using short-read data. Cell Syst 13: 808–816.e5. 10.1016/j.cels.2022.08.008 36265467 PMC10084889

[GR280314FORC14] Gadala-Maria D, Gidoni M, Marquez S, Vander Heiden JA, Kos JT, Watson CT, O'Connor KC, Yaari G, Kleinstein SH. 2019. Identification of subject-specific immunoglobulin alleles from expressed repertoire sequencing data. Front Immunol 10: 129. 10.3389/fimmu.2019.00129 30814994 PMC6381938

[GR280314FORC15] Gibson WS, Rodriguez OL, Shields K, Silver CA, Dorgham A, Emery M, Deikus G, Sebra R, Eichler EE, Bashir A, 2023. Characterization of the immunoglobulin lambda chain locus from diverse populations reveals extensive genetic variation. Genes Immun 24: 21–31. 10.1038/s41435-022-00188-2 36539592 PMC10041605

[GR280314FORC16] Gurobi Optimization. 2021. Gurobi optimizer reference manual.

[GR280314FORC17] Hari A, Zhou Q, Gonzaludo N, Harting J, Scott SA, Qin X, Scherer S, Sahinalp SC, Numanagić I. 2023. An efficient genotyper and star-allele caller for pharmacogenomics. Genome Res 33: 61–70. 10.1101/gr.277075.122 36657977 PMC9977157

[GR280314FORC18] Hosmer DW Jr, Lemeshow S, Sturdivant RX. 2013. Applied logistic regression. John Wiley & Sons, Hoboken, NJ.

[GR280314FORC19] Huang W, Li L, Myers JR, Marth GT. 2012. ART: a next-generation sequencing read simulator. Bioinformatics 28: 593–594. 10.1093/bioinformatics/btr708 22199392 PMC3278762

[GR280314FORC20] Johnson TA, Mashimo Y, Wu JY, Yoon D, Hata A, Kubo M, Takahashi A, Tsunoda T, Ozaki K, Tanaka T, 2021. Association of an IGHV3-66 gene variant with Kawasaki disease. J Hum Genet 66: 475–489. 10.1038/s10038-020-00864-z 33106546 PMC7585995

[GR280314FORC21] Koboldt DC, Steinberg KM, Larson DE, Wilson RK, Mardis ER. 2013. The next-generation sequencing revolution and its impact on genomics. Cell 155: 27–38. 10.1016/j.cell.2013.09.006 24074859 PMC3969849

[GR280314FORC22] Langmead B, Salzberg SL. 2012. Fast gapped-read alignment with Bowtie 2. Nat Methods 9: 357–359. 10.1038/nmeth.1923 22388286 PMC3322381

[GR280314FORC23] Lee JH, Toy L, Kos JT, Safonova Y, Schief WR, Havenar-Daughton C, Watson CT, Crotty S. 2021. Vaccine genetics of IGHV1-2 VRC01-class broadly neutralizing antibody precursor naive human B cells. NPJ Vaccines 6: 113. 10.1038/s41541-021-00376-7 34489473 PMC8421370

[GR280314FORC24] Lees WD, Saha S, Yaari G, Watson CT. 2024. Digger: directed annotation of immunoglobulin and T cell receptor V, D, and J gene sequences and assemblies. Bioinformatics 40: btae144. 10.1093/bioinformatics/btae144 38478393 PMC10957512

[GR280314FORC25] Lefranc M. 2003. IMGT databases, web resources and tools for immunoglobulin and T cell receptor sequence analysis, http://www.imgt.org. Leukemia 17: 260–266. 10.1038/sj.leu.2402637 12529691

[GR280314FORC26] Lefranc MP, Lefranc G. 2001a. The immunoglobulin factsbook. Academic Press, London.

[GR280314FORC27] Lefranc MP, Lefranc G. 2001b. The T cell receptor factsbook. Academic Press, London.

[GR280314FORC28] Liang B, Ding H, Huang L, Luo H, Zhu X. 2020. GWAS in cancer: progress and challenges. Mol Genet Genomics 295: 537–561. 10.1007/s00438-020-01647-z 32048005

[GR280314FORC29] Lin MJ, Lin YC, Chen NC, Luo AC, Lai SK, Hsu CL, Hsu JS, Chen CY, Yang WS, Chen PL. 2022. Profiling genes encoding the adaptive immune receptor repertoire with gAIRR Suite. Front Immunol 13: 922513. 10.3389/fimmu.2022.922513 36159868 PMC9496171

[GR280314FORC30] Lin MJ, Langmead B, Safonova Y. 2025. IGLoo enables comprehensive analysis and assembly of immunoglobulin heavy-chain loci in lymphoblastoid cell lines using PacBio high-fidelity reads. Cell Rep Methods 5: 101033. 10.1016/j.crmeth.2025.101033 40315852 PMC12146632

[GR280314FORC31] Mikocziova I, Peres A, Gidoni M, Greiff V, Yaari G, Sollid LM. 2021. Germline polymorphisms and alternative splicing of human immunoglobulin light chain genes. iScience 24: 103192. 10.1016/j.isci.2021.103192 34693229 PMC8517844

[GR280314FORC32] Motsinger-Reif AA, Jorgenson E, Relling MV, Kroetz DL, Weinshilboum R, Cox NJ, Roden DM. 2013. Genome-wide association studies in pharmacogenomics: successes and lessons. Pharmacogenet Genomics 23: 383–394. 10.1097/FPC.0b013e32833d7b45 20639796 PMC3003940

[GR280314FORC33] Numanagić I, Malikić S, Pratt VM, Skaar TC, Flockhart DA, Sahinalp SC. 2015. Cypiripi: exact genotyping of CYP2D6 using high-throughput sequencing data. Bioinformatics 31: i27–i34. 10.1093/bioinformatics/btv232 26072492 PMC4542776

[GR280314FORC34] Numanagić I, Malikić S, Ford M, Qin X, Toji L, Radovich M, Skaar TC, Pratt VM, Berger B, Scherer S, 2018. Allelic decomposition and exact genotyping of highly polymorphic and structurally variant genes. Nat Commun 9: 828. 10.1038/s41467-018-03273-1 29483503 PMC5826927

[GR280314FORC35] Parks T, Mirabel MM, Kado J, Auckland K, Nowak J, Rautanen A, Mentzer AJ, Marijon E, Jouven X, Perman ML, 2017. Association between a common immunoglobulin heavy chain allele and rheumatic heart disease risk in Oceania. Nat Commun 8: 14946. 10.1038/ncomms14946 28492228 PMC5437274

[GR280314FORC36] Peng K, Safonova Y, Shugay M, Popejoy AB, Rodriguez OL, Breden F, Brodin P, Burkhardt AM, Bustamante C, Cao-Lormeau VM, 2021. Diversity in immunogenomics: the value and the challenge. Nat Methods 18: 588–591. 10.1038/s41592-021-01169-5 34002093 PMC8842483

[GR280314FORC37] Peres A, Gidoni M, Polak P, Yaari G. 2019. RAbHIT: R antibody haplotype inference tool. Bioinformatics 35: 4840–4842. 10.1093/bioinformatics/btz481 31173062

[GR280314FORC38] Prodanov T, Plender EG, Seebohm G, Meuth SG, Eichler EE, Marschall T. 2024. Locityper: targeted genotyping of complex polymorphic genes. bioRxiv 10.1101/2024.05.03.592358 PMC12597825 41107551

[GR280314FORC39] Ralph DK, Matsen FA IV. 2019. Per-sample immunoglobulin germline inference from B cell receptor deep sequencing data. PLoS Comput Biol 15: e1007133. 10.1371/journal.pcbi.1007133 31329576 PMC6675132

[GR280314FORC40] Rodriguez OL, Gibson WS, Parks T, Emery M, Powell J, Strahl M, Deikus G, Auckland K, Eichler EE, Marasco WA, 2020. A novel framework for characterizing genomic haplotype diversity in the human immunoglobulin heavy chain locus. Front Immunol 11: 2136. 10.3389/fimmu.2020.02136 33072076 PMC7539625

[GR280314FORC41] Rodriguez OL, Sharp AJ, Watson CT. 2021. Limitations of lymphoblastoid cell lines for establishing genetic reference datasets in the immunoglobulin loci. PLoS One 16: e0261374. 10.1371/journal.pone.0261374 34898642 PMC8668129

[GR280314FORC42] Rodriguez OL, Silver CA, Shields K, Smith ML, Watson CT. 2022. Targeted long-read sequencing facilitates phased diploid assembly and genotyping of the human T cell receptor alpha, delta, and beta loci. Cell Genomics 2: 100228. 10.1016/j.xgen.2022.100228 36778049 PMC9903726

[GR280314FORC43] Rodriguez OL, Safonova Y, Silver CA, Shields K, Gibson WS, Kos JT, Tieri D, Ke H, Jackson KJ, Boyd SD, 2023. Genetic variation in the immunoglobulin heavy chain locus shapes the human antibody repertoire. Nat Commun 14: 4419. 10.1038/s41467-023-40070-x 37479682 PMC10362067

[GR280314FORC44] Seabold S, Perktold J. 2010. statsmodels: Econometric and statistical modeling with Python. In 9th Python in Science Conference.

[GR280314FORC45] Szolek A, Schubert B, Mohr C, Sturm M, Feldhahn M, Kohlbacher O. 2014. OptiType: precision HLA typing from next-generation sequencing data. Bioinformatics 30: 3310–3316. 10.1093/bioinformatics/btu548 25143287 PMC4441069

[GR280314FORC46] Watson CT, Breden F. 2012. The immunoglobulin heavy chain locus: genetic variation, missing data, and implications for human disease. Genes Immun 13: 363–373. 10.1038/gene.2012.12 22551722

[GR280314FORC47] Watson CT, Steinberg KM, Huddleston J, Warren RL, Malig M, Schein J, Willsey AJ, Joy JB, Scott JK, Graves TA, 2013. Complete haplotype sequence of the human immunoglobulin heavy-chain variable, diversity, and joining genes and characterization of allelic and copy-number variation. Am J Hum Genet 92: 530–546. 10.1016/j.ajhg.2013.03.004 23541343 PMC3617388

[GR280314FORC48] Watson CT, Glanville J, Marasco WA. 2017. The individual and population genetics of antibody immunity. Trends Immunol 38: 459–470. 10.1016/j.it.2017.04.003 28539189 PMC5656258

[GR280314FORC49] Yacoob C, Pancera M, Vigdorovich V, Oliver BG, Glenn JA, Feng J, Sather DN, McGuire AT, Stamatatos L. 2016. Differences in allelic frequency and CDRH3 region limit the engagement of HIV Env immunogens by putative VRC01 neutralizing antibody precursors. Cell Rep 17: 1560–1570. 10.1016/j.celrep.2016.10.017 27806295 PMC5207042

[GR280314FORC50] Yeung YA, Foletti D, Deng X, Abdiche Y, Strop P, Glanville J, Pitts S, Lindquist K, Sundar PD, Sirota M, 2016. Germline-encoded neutralization of a *Staphylococcus aureus* virulence factor by the human antibody repertoire. Nat Commun 7: 13376. 10.1038/ncomms13376 27857134 PMC5120205

[GR280314FORC51] Zhou Q, Ghezelji M, Hari A, Ford MK, Holley C, Sahinalp SC, Numanagić I. 2024. Geny: a genotyping tool for allelic decomposition of killer cell immunoglobulin-like receptor genes. Front Immunol 15: 1494995. 10.3389/fimmu.2024.1494995 39763645 PMC11701374

