# Supplementary material for: Genotyping of selected germline adaptive immune system loci using short-read sequencing data
Source: Genome Res. 2025 Sep;35(9):2076–86. doi: 10.1101/gr.280314.124 (PMC12401057; doi:10.1101/gr.280314.124)
Supplement: Supplement 1 [file Supplemental_Code.zip › ImmunoTyper2-methods/HPRC-assembly-benchmarking/digger/docs/_build/html/docker.html]

Docker Image — Digger 0.5.0 documentation


Digger

Getting Started

- Overview
- digger
- dig-sequence
- Docker Image
- Installation
- Release Notes
- Changes in 0.7.5
- Changes in 0.7.4
- Changes in 0.7.3

Examples

- Annotating the human IGH locus
- Annotating the rhesus macaque IGH locus
- Targeted Annotation
- Additional Examples

Usage Documentation

- Commandline Usage
- Anotation format

Digger

- Docker Image
- View page source

---

# Docker Image

The Docker image contains a working installation of Digger and its dependencies. The examples described in this documentation
are installed in the image at /digger/tests and can conveniently be run from the container.

The image is available in Docker Hub at williamlees/digger.

```
# Pull the latest version
$ docker pull williamlees/digger:latest
```

To use Digger from the command line, please use the following command in Linux/Mac/Windows:

```
$ docker run williamlees/digger:latest digger --help
```

To run commands within the container, please use the following command in Linux/Mac:

```
$ docker run -it -v $(pwd):/scratch williamlees/digger:latest bash
```

Or at the Windows command prompt:

```
> docker run -it -v %cd%:/scratch  williamlees/digger:latest bash
```

This will start a bash shell in the container, with the current directory mounted at /scratch. You can then run Digger commands as described in the documentation.

Previous
Next

---

© Copyright 2023, William Lees.

Built with Sphinx using a
theme
provided by Read the Docs.
