# Supplementary material for: Genotyping of selected germline adaptive immune system loci using short-read sequencing data
Source: Genome Res. 2025 Sep;35(9):2076–86. doi: 10.1101/gr.280314.124 (PMC12401057; doi:10.1101/gr.280314.124)
Supplement: Supplement 1 [file Supplemental_Code.zip › ImmunoTyper2-methods/HPRC-assembly-benchmarking/digger/docs/_build/html/examples/human_igh.html]

Annotating the human IGH locus — Digger 0.5.0 documentation


Digger

Getting Started

- Overview
- digger
- dig-sequence
- Docker Image
- Installation
- Release Notes
- Changes in 0.7.5
- Changes in 0.7.4
- Changes in 0.7.3

Examples

- Annotating the human IGH locus
  - Data
  - Annotating the Assembly
  - Comparing the output to IMGT’s annotation
  - References
- Annotating the rhesus macaque IGH locus
- Targeted Annotation
- Additional Examples

Usage Documentation

- Commandline Usage
- Anotation format

Digger

- Annotating the human IGH locus
- View page source

---

# Annotating the human IGH locus

The IGHV locus in the human reference assembly GRCh38.p12 has been annotated by IMGT. In this example, we will annotate with Digger and compare results.
The comparison, and a script to reproduce it using the steps below, can be found in digger’s GitHub repository.
Digger’s annotation can also be found in that directory.

## Data

The locus and IMGT annotations can be downloaded and parsed with parse\_imgt\_annotations:

```
> parse_imgt_annotations \
    --save_sequence IMGT000035.fasta \
        "http://www.imgt.org/ligmdb/view?format=IMGT&id=BK063799" \
        IMGT000035_genes.csv \
        IGH
```

This will create two files: `IMGT000035.fasta`, containing the assembly sequence, and `IMGT000035_genes.csv`, containing the co-ordinates of the IMGT-annotated genes, and sequences of their flanking regions.

The human IGH reference set can be downloaded from IMGT with the receptor\_utils command `extract_refs` (receptor\_utils is installed as part of Digger’s installation):

```
> extract_refs -L IGH "Homo sapiens"
```

This creates the reference sets `Homo_sapiens_IGHV_gapped.fasta`, `Homo_sapiens_IGHV.fasta`, `Homo_sapiens_IGHD.fasta`, `Homo_sapiens_IGHJ.fasta`, `Homo_sapiens_CH.fasta`

Lastly, we need to make a combined file containing all the IGH V, D and J reference genes:

```
> cat Homo_sapiens_IGHV.fasta Homo_sapiens_IGHD.fasta Homo_sapiens_IGHJ.fasta \
    > Homo_sapiens_IGHVDJ.fasta
```

## Annotating the Assembly

With the data in place, we can instruct digger to perform the annotation:

```
> digger IMGT000035.fasta \
    -v_ref Homo_sapiens_IGHV.fasta \
        -d_ref Homo_sapiens_IGHD.fasta \
        -j_ref Homo_sapiens_IGHJ.fasta \
        -v_ref_gapped Homo_sapiens_IGHV_gapped.fasta \
        -ref imgt,Homo_sapiens_IGHVDJ.fasta \
        -species human \
        -locus IGH \
        IMGT000035.csv
```

`-v_ref, -d_ref, -j_ref, -v_ref_gapped` provide the germline reference sequences.

`-ref imgt,Homo_sapiens_IGHVDJ.fasta` instructs Digger to compare any sequences it identifies in the assembly with those in the combined reference
set. It will create columns in the output assembly containing the nearest reference sequence found, % identity and so on. These columns will be prefixed `imgt` as specified in this argument. Multiple `-ref` arguments can be
passed, allowing comparison with multiple reference sets.

`-species` tells Digger to use its internal position-weighted matrices for human loci, and `-locus` specifies the locus.

The output file will be `IMGT000035.csv`. A sample copy of this file will be found in the GitHub repository.

Digger will summarise progress as it runs. It will call the following tools:

- makeblastdb to create BLAST databases for the reference genes
- blastn to run these databases against the reference sequence
- blastresults\_to\_csv to convert the BLAST output to a simpler format
- find\_alignments to annotate gene alignments found in the assembly

## Comparing the output to IMGT’s annotation

compare\_annotations compares the output of a digger run with a summary annotation file. Here we use it to compare the results of digger’s annotation with IMGT’s:

```
> compare_annotations IMGT000035.csv IMGT000035_genes.csv forward comparison_results
```

The final argument specifies that the results should be put into files named `comparison_results`. Three files are produced with differing extensions: a summary graphic (.jpg), a text file listing differences
in sequences annotated as functional (.txt), and a detailed line-by-line comparison (.csv).

comparison\_results.jpg summarises functional annotations found by digger and IMGT, according to which,
digger annotated as functional all genes so annotated by IMGT, and annotated an additional 2 V-genes and one J-gene as functional.

comparison\_results.txt lists the differences in functional analysis in detail.
comparison\_results\_notes.txt adds some commentary: of the two additional
V-genes annotated by digger as functional, one has unusual variations in the RSS, causing IMGT to annotate it as ORF. The other is annotated as ORF on the grounds that it has not been
seen rearranged. The additional J-gene is currently annotated by IMGT in the assembly as ORF, although it is listed in the IMGT gene table as functional.

There is not, at present, a clear set of universally accepted criteria for categorisation of functionality, and minor differences of this nature are to be expected. Over the next few years, we expect to see comparisons of genomic
sequencing of the loci with the expressed repertoire across multiple subjects, and this should allow a deeper understanding to develop. Overall, the comparison of digger results with the manually supervised curation
at IMGT shows a good level of agreement. It is possible that results may change from those noted here, as they are based on downloaded data which may be revised over time.

## References

Lefranc et al., 2015, IMGT®, the international ImMunoGeneTics information system® 25 years on. *Nucleic Acids Res.* doi: 10.1093/nar/gku1056.

Watson et al., 2013, Complete haplotype sequence of the human immunoglobulin heavy-chain variable, diversity, and joining genes and characterization of allelic and copy-number variation. *Am J Hum Genet* doi: 10.1016/j.ajhg.2013.03.004

Schneider et al., 2017, Evaluation of GRCh38 and de novo haploid genome assemblies demonstrates the enduring quality of the reference assembly. *Genome Research* doi: 10.1101/gr.213611.116

Previous
Next

---

© Copyright 2023, William Lees.

Built with Sphinx using a
theme
provided by Read the Docs.
