# Supplementary material for: Genotyping of selected germline adaptive immune system loci using short-read sequencing data
Source: Genome Res. 2025 Sep;35(9):2076–86. doi: 10.1101/gr.280314.124 (PMC12401057; doi:10.1101/gr.280314.124)
Supplement: Supplement 1 [file Supplemental_Code.zip › ImmunoTyper2-methods/HPRC-assembly-benchmarking/digger/docs/_build/html/examples/rhesus_igh.html]

Annotating the rhesus macaque IGH locus — Digger 0.5.0 documentation


Digger

Getting Started

- Overview
- digger
- dig-sequence
- Docker Image
- Installation
- Release Notes
- Changes in 0.7.5
- Changes in 0.7.4
- Changes in 0.7.3

Examples

- Annotating the human IGH locus
- Annotating the rhesus macaque IGH locus
  - Data
  - Preparing position-weighted matrices
  - Annotating the Assembly
  - Comparing the output to the study’s annotation
  - References
- Targeted Annotation
- Additional Examples

Usage Documentation

- Commandline Usage
- Anotation format

Digger

- Annotating the rhesus macaque IGH locus
- View page source

---

# Annotating the rhesus macaque IGH locus

In this example we will see how to:

- Create position weight matrices from existing IMGT annotations
- Use the underlying commands that digger calls, and understand how they might be useful when annotating multiple contigs or scaffolds

IMGT has identified scaffolds in the 2006 rhesus macaque reference assembly, Mmul\_051212, which lie within the IGH locus. Here we will bring them together in a single file and annotate them with motifs derived from the current reference assembly, rhemac10 (Mmul\_10).
While this example is somewhat artificial, in that the scaffolds could equally well be annotated using the single digger command, the example serves to show how the individual commands in the package can be used. This provides some additional flexibility, for example in tuning the blast searches, and also illustrates how they work together.
The comparison with IMGT’s annotation of Mmul\_051212, and a script to reproduce this example using the steps below, can be found in digger’s Git repository.

## Data

As in the previous example, the rhesus IGH germline reference set can be downloaded from IMGT with the receptor\_utils command
`extract_refs` (receptor\_utils is installed as part of digger’s installation). However, the rhesus IG gapped V-genes provided by IMGT contain additional inserted codons relative to
the conventional IMGT alignment. As digger (alongside other tools) expects the conventional alignment, a further step is needed to realign the gapped sequences, using the receptor\_utils
tool `fix_macaque_gaps`. The following commands will prepare the reference data:

```
> extract_refs -L IGH "Macaca mulatta"
> fix_macaque_gaps Macaca_mulatta_IGHV_gapped.fasta \
    Macaca_mulatta_IGHV_gapped_fixed.fasta IGH
> cat Macaca_mulatta_IGHV.fasta Macaca_mulatta_IGHD.fasta Macaca_mulatta_IGHJ.fasta \
    > Macaca_mulatta_IGHVDJ.fasta
```

The Mmul\_51212 scaffolds can be downloaded from IMGT as follows:

```
> parse_imgt_annotations --save_sequence NW_001157919.fasta \
   "https://www.imgt.org/ligmdb/view.action?format=IMGT&id=NW_001157919" \
       NW_001157919_genes.csv IGH
> parse_imgt_annotations --save_sequence NW_001122023.fasta \
   "https://www.imgt.org/ligmdb/view.action?format=IMGT&id=NW_001122023" \
   NW_001122023_genes.csv IGH
> parse_imgt_annotations --save_sequence NW_001122024.fasta \
   "https://www.imgt.org/ligmdb/view.action?format=IMGT&id=NW_001122024" \
       NW_001122024_genes.csv IGH
> parse_imgt_annotations --save_sequence NW_001121239.fasta \
   "https://www.imgt.org/ligmdb/view.action?format=IMGT&id=NW_001121239" \
       NW_001121239_genes.csv IGH
> parse_imgt_annotations --save_sequence NW_001121240.fasta \
   "https://www.imgt.org/ligmdb/view.action?format=IMGT&id=NW_001121240" \
       NW_001121240_genes.csv IGH

>cat NW_001157919.fasta NW_001122023.fasta NW_001122024.fasta \
       NW_001121239.fasta NW_001121240.fasta > Mmul_051212.fasta
```

## Preparing position-weighted matrices

Digger already has PWMs for rhesus IGH, but for the purpose of this example, we will create a set using the features listed in IMGT’s annotation of the rhemac10 IGH locus, which
has the IMGT accession number IMGT000064. The following commands download the annotation, determine the features, and calculate the PWMs from
features of functional annotations:

```
> mkdir motifs
> cd motifs
> parse_imgt_annotations \
        "http://www.imgt.org/ligmdb/view?format=IMGT&id=IMGT000064" \
        IMGT000064_genes.csv IGH
> calc_motifs IGH IMGT000064_genes.csv
```

`calc_motifs` will create 10 motif files in the directory. Additionally, it prepares `motif_params.json`, which contains some further locus-specific motif parameters.
These are descrbed further in calc\_motifs.

The motifs directory may optionally contain a FASTA file `conserved_motifs.fasta` defining strongly-conserved nucleotides in the RSS and leader. Only those features
with conserved nucleotides need to be listed in the file. The names follow the filenames used for the PWMs.
The following sequences were derived from Figure 3 of Ngoune et al. (2022) and will be used in this example:

```
>V-HEPTAMER
CAC---G
>V-NONAMER
-----AACC
>5'D-HEPTAMER
----GTG
>5'D-NONAMER
---T-----
>3'D-HEPTAMER
C-C---G
>3'D-NONAMER
-C----A--
>J-HEPTAMER
C--TGTG
>J-NONAMER
-GTT--TG-
```

Again, this file is provided for download at the location provided near the top of this example.

While the presence or absence of conserved nucleotides can be a useful guide to the likely functionality of a sequence, please bear in mind that it is a guide only:
exceptions can be expected, particularly where the definitions have been built on limited data.

## Annotating the Assembly

The digger command is not able to handle a FASTA file containing multiple contigs, so we will call the underlying tools directly. We start by creating the blast databases and querying against the assembly,
using the reference genes determined in the study:

```
> makeblastdb -in Macaca_mulatta_IGHV.fasta -dbtype nucl
> makeblastdb -in Macaca_mulatta_IGHD.fasta -dbtype nucl
> makeblastdb -in Macaca_mulatta_IGHJ.fasta -dbtype nucl

> blastn -db Macaca_mulatta_IGHV.fasta -query Mmul_051212.fasta -out mmul_IGHV.out \
   -outfmt 7 -gapopen 5 -gapextend 5 -penalty -1 -word_size 11
> blastn -db Macaca_mulatta_IGHD.fasta -query Mmul_051212.fasta -out mmul_IGHD.out \
   -outfmt 7 -gapopen 5 -gapextend 5 -penalty -1 -word_size 7 -evalue 100
> blastn -db Macaca_mulatta_IGHJ.fasta -query Mmul_051212.fasta -out mmul_IGHJ.out \
   -outfmt 7 -gapopen 5 -gapextend 5 -penalty -1 -word_size 7
```

Note that a higher evalue is used for the D genes, as they can be quite short.

Next we call `blastresults_to_csv` to convert to a more convenient format:

```
> blastresults_to_csv mmul_IGHV.out mmul_ighvdj_
> blastresults_to_csv mmul_IGHD.out mmul_ighvdj_ -a
> blastresults_to_csv mmul_IGHJ.out mmul_ighvdj_ -a
```

The commands instruct the tool to create merged files containing V,D and J hits. This is achieved by specifying the same prefix on each command `(mmul_ighvdj_)` and using the `-a` (append) option.
The records created by blastn contain the name of the contig in which a hit was found. `blastresults_to_csv` will create one file per contig. The names contain the ID of the contig in
`Mmul_051212.fasta`, except that they are modified where necessary to ensure file system compatibility.

We now call `find_alignments` to process the annotations:

```
> find_alignments Macaca_mulatta_IGHVDJ.fasta \
       Mmul_051212.fasta \
       "mmul_ighvdj_nw_*.csv" \
       -ref imgt,Macaca_mulatta_IGHVDJ.fasta \
       -align Macaca_mulatta_IGHV_gapped_fixed.fasta \
       -motif_dir motifs \
       Mmul_051212.csv
```

Note that the third argument, `"mmul_ighvdj_nw_*.csv"`, contains a wildcard that will match all the files produced in the previous step. It is quoted to avoid expansion by the shell.
V-genes in the annotation will be annotated and gapped using the IMGT set as a template (with fixed gaps).
`find_alignments` will attempt to deduce the sense in which to annotate each segment. This is helpful in this case as the contigs vary in their orientation. Note that we are
specifying the location of the motifs directory created in the previous step rather than the species and locus, which would cause digger to use the built-in tables.

## Comparing the output to the study’s annotation

`compare_annotations` is not capable of handling the output from multiple sequences in the same file, so unfortunately we need to split the results up for the comparison:

> > head -n 1 Mmul\_051212.csv > mmul\_header.csv
>
> > cp mmul\_header.csv NW\_001157919\_digger.csv
> > grep NW\_001157919 Mmul\_051212.csv >> NW\_001157919\_digger.csv
>
> > cp mmul\_header.csv NW\_001122023\_digger.csv
> > grep NW\_001122023 Mmul\_051212.csv >> NW\_001122023\_digger.csv
>
> > cp mmul\_header.csv NW\_001122024\_digger.csv
> > grep NW\_001122024 Mmul\_051212.csv >> NW\_001122024\_digger.csv
>
> > cp mmul\_header.csv NW\_001121239\_digger.csv
> > grep NW\_001121239 Mmul\_051212.csv >> NW\_001121239\_digger.csv
>
> > cp mmul\_header.csv NW\_001121240\_digger.csv
> > grep NW\_001121240 Mmul\_051212.csv >> NW\_001121240\_digger.csv
>
> > compare\_annotations NW\_001157919\_digger.csv NW\_001157919\_genes.csv forward NW\_001157919\_comp
> > compare\_annotations NW\_001122023\_digger.csv NW\_001122023\_genes.csv forward NW\_001122023\_comp
> > compare\_annotations NW\_001122024\_digger.csv NW\_001122024\_genes.csv forward NW\_001122024\_comp
> > compare\_annotations NW\_001121239\_digger.csv NW\_001121239\_genes.csv forward NW\_001121239\_comp
> > compare\_annotations NW\_001121240\_digger.csv NW\_001121240\_genes.csv forward NW\_001121240\_comp

Scaffold-by-scaffold comparisons are provided in Github.
and an overall comparison is provided here.

Digger identified a total of 13 potentially functional D-genes not annotated by IMGT, across four of the five scaffolds, while IMGT annotated D-genes only in NW\_001121239. The macaque IGHD genes are known
to occupy a small, distinct, region towards the 3’ end of the IGH locus. It would therefore be reasonable to expect them to be located in a single scaffold, and to be
distinct from the V-genes. However, given the sequencing technology available for sequencing and assembly when the scaffolds were created, and bearing
in mind the short length of the D-genes, it is possible that the D-locus was not correctly assembled. Another reason for suspecting this is that two of the D-sequences
identified by Digger are extremely short, at 3nt and 1nt, and yet appear to be flanked by functional RSS. In contrast, in an
annotation of the rhemac10 assembly, Digger identified only one D-gene
not annotated by IMGT (this was also outside the D locus).

## References

Ngoune et al., 2022, IMGT® Biocuration and Analysis of the Rhesus Monkey IG Loci. *Vaccines* doi: 10.3390/vaccines10030394.

Warren et al., 2020, Sequence Diversity Analyses of an Improved Rhesus Macaque Genome Enhance Its Biomedical Utility. *Science* doi: 10.1126/science.abc6617.

Gibbs et al., 2007, Evolutionary and biomedical insights from the rhesus macaque genome. *Science* doi: 10.1126/science.1139247.

Previous
Next

---

© Copyright 2023, William Lees.

Built with Sphinx using a
theme
provided by Read the Docs.
