# Supplementary material for: Genotyping of selected germline adaptive immune system loci using short-read sequencing data
Source: Genome Res. 2025 Sep;35(9):2076–86. doi: 10.1101/gr.280314.124 (PMC12401057; doi:10.1101/gr.280314.124)
Supplement: Supplement 1 [file Supplemental_Code.zip › ImmunoTyper2-methods/HPRC-assembly-benchmarking/digger/docs/_build/html/examples/targeted_annotation.html]

Targeted Annotation — Digger 0.5.0 documentation


Digger

Getting Started

- Overview
- digger
- dig-sequence
- Docker Image
- Installation
- Release Notes
- Changes in 0.7.5
- Changes in 0.7.4
- Changes in 0.7.3

Examples

- Annotating the human IGH locus
- Annotating the rhesus macaque IGH locus
- Targeted Annotation
- Additional Examples

Usage Documentation

- Commandline Usage
- Anotation format

Digger

- Targeted Annotation
- View page source

---

# Targeted Annotation

In the examples covered so far, digger has been used to identify as many receptor genes as possible,
using wide-ranging BLAST searches. A companion tool, dig\_sequence, can be used to identify and annotate the closest match to a single specified
sequence.

As an example of its use, an online BLAST search identifies a 100% sequence match to the human receptor gene IGHV1-18\*04 at
Genbank accession number KC713938. This can be annotated by the `dig_sequence single` command:

```
>dig_sequence single -align Homo_sapiens_IGHV_gapped.fasta -species human IGHV1-18*01 Homo_sapiens_IGHV.fasta KC713938
Using motif files from C:\Users\William\miniconda3\envs\digby_genomics310\lib\site-packages\digger\motifs\human\IGH
target_allele: IGHV1-18*01
genbank_acc: KC713938
genbank_seq: length: 935nt
gene_seq: ATGGACTGGACCTGGAGCATCCTTTTCTTGGTGGCAGCAGCAACAGGTAACGGACTCCCCAGTCCCAGGGCTGAGAGAGAAACCAGGCCAGTCATGTGAGACTTCACCCACTCCTGTGTCCTCTCCACAGGTGCCCACTCCCAGGTTCAGCTGGTGCAGTCTGGAGCTGAGGTGAAGAAGCCTGGGGCCTCAGTGAAGGTCTCCTGCAAGGCTTCTGGTTACACCTTTACCAGCTACGGTATCAGCTGGGTGCGACAGGCCCCTGGACAAGGGCTTGAGTGGATGGGATGGATCAGCGCTTACAATGGTAACACAAACTATGCACAGAAGCTCCAGGGCAGAGTCACCATGACCACAGACACATCCACGAGCACAGCCTACATGGAGCTGAGGAGCCTGAGATCTGACGACACGGCCGTGTATTACTGTGCGAGAGACACAGTGTGAAAACCCACATCCTGAGGGTTTCAGAAACC
seq: CAGGTTCAGCTGGTGCAGTCTGGAGCTGAGGTGAAGAAGCCTGGGGCCTCAGTGAAGGTCTCCTGCAAGGCTTCTGGTTACACCTTTACCAGCTACGGTATCAGCTGGGTGCGACAGGCCCCTGGACAAGGGCTTGAGTGGATGGGATGGATCAGCGCTTACAATGGTAACACAAACTATGCACAGAAGCTCCAGGGCAGAGTCACCATGACCACAGACACATCCACGAGCACAGCCTACATGGAGCTGAGGAGCCTGAGATCTGACGACACGGCCGTGTATTACTGTGCGAGAGA
alignment_score: 99.7
nt_diff: 1
snps: _t111c
start: 392
end: 687
sense: +
functional: Functional
notes:
l_part1: ATGGACTGGACCTGGAGCATCCTTTTCTTGGTGGCAGCAGCAACAG
l_part2: GTGCCCACTCC
v_heptamer: CACAGTG
v_nonamer: TCAGAAACC
```

The command takes as arguments the id of the target sequence to search for (in this case IGHV1-18\*01), the fasta file in
which the sequence can be found, and the Genbank ID to search. Optional arguments include the species (otherwise human
is assumed) and a file of gapped reference sequences, which is used as a guide to gap V sequences. If an output file
is specified
with the `-out_file` argument, the output is written there in CSV format, with information matching that provided by
`digger`. Otherwise a summary is provided to standard output.

The command searches the specified Genbank accession for the closest match to the target sequenece (in this case
differing by a single nucleotide), and returns annotation details. These will include regulatory regions where they are
available. Only a single closest-match sequence is annotated.

Variants of dig\_sequence allow the sequence to be specified directly rather than by Genbank accession number, and
allow multiple searches to be specified in a csv file.

Previous
Next

---

© Copyright 2023, William Lees.

Built with Sphinx using a
theme
provided by Read the Docs.
