# Supplementary material for: Genotyping of selected germline adaptive immune system loci using short-read sequencing data
Source: Genome Res. 2025 Sep;35(9):2076–86. doi: 10.1101/gr.280314.124 (PMC12401057; doi:10.1101/gr.280314.124)
Supplement: Supplement 1 [file Supplemental_Code.zip › ImmunoTyper2-methods/HPRC-assembly-benchmarking/digger/docs/_build/html/genindex.html]

Index — Digger 0.5.0 documentation


Digger

Getting Started

- Overview
- digger
- dig-sequence
- Docker Image
- Installation
- Release Notes
- Changes in 0.7.5
- Changes in 0.7.4
- Changes in 0.7.3

Examples

- Annotating the human IGH locus
- Annotating the rhesus macaque IGH locus
- Targeted Annotation
- Additional Examples

Usage Documentation

- Commandline Usage
- Anotation format

Digger

- Index

---

# Index

---

© Copyright 2023, William Lees.

Built with Sphinx using a
theme
provided by Read the Docs.
