# Supplementary material for: Genotyping of selected germline adaptive immune system loci using short-read sequencing data
Source: Genome Res. 2025 Sep;35(9):2076–86. doi: 10.1101/gr.280314.124 (PMC12401057; doi:10.1101/gr.280314.124)
Supplement: Supplement 1 [file Supplemental_Code.zip › ImmunoTyper2-methods/HPRC-assembly-benchmarking/digger/docs/_build/html/index.html]

Digger: tools for annotating genomic assemblies of the IG/TR receptor loci — Digger 0.5.0 documentation


Digger

Getting Started

- Overview
- digger
- dig-sequence
- Docker Image
- Installation
- Release Notes
- Changes in 0.7.5
- Changes in 0.7.4
- Changes in 0.7.3

Examples

- Annotating the human IGH locus
- Annotating the rhesus macaque IGH locus
- Targeted Annotation
- Additional Examples

Usage Documentation

- Commandline Usage
- Anotation format

Digger

- Digger: tools for annotating genomic assemblies of the IG/TR receptor loci
- View page source

---

# Digger: tools for annotating genomic assemblies of the IG/TR receptor loci

Digger is a toolkit for the automatic annotation of unrearranged V,D and J genes in B- and T- cell immunoglobulin receptor genomic loci. It can be used to annotate both entire assemblies, large fragments of a locus,
or small fragments. It annotates all features of the gene (e.g. leader, RSS) excluding UTR, if the features are present. It attempts to classify the gene as Functional,
ORF, or pseudogene following IMGT practce.

Getting Started

- Overview
- digger
- dig-sequence
- Docker Image
- Installation
- Release Notes
- Changes in 0.7.5
- Changes in 0.7.4
- Changes in 0.7.3

Examples

- Annotating the human IGH locus
- Annotating the rhesus macaque IGH locus
- Targeted Annotation
- Additional Examples

Usage Documentation

- Commandline Usage
- Anotation format

# Indices and tables

- Index
- Module Index
- Search Page

# Citation

If you use Digger in your research, please cite the following paper:
Digger: directed annotation of immunoglobulin and T cell receptor V, D and J gene sequences and assemblies.
Bioinformatics, 2024. DOI: 10.1093/bioinformatics/btae144.

Next

---

© Copyright 2023, William Lees.

Built with Sphinx using a
theme
provided by Read the Docs.
