# Supplementary material for: Genotyping of selected germline adaptive immune system loci using short-read sequencing data
Source: Genome Res. 2025 Sep;35(9):2076–86. doi: 10.1101/gr.280314.124 (PMC12401057; doi:10.1101/gr.280314.124)
Supplement: Supplement 1 [file Supplemental_Code.zip › ImmunoTyper2-methods/HPRC-assembly-benchmarking/digger/docs/_build/html/install.html]

Installation — Digger 0.5.0 documentation


Digger

Getting Started

- Overview
- digger
- dig-sequence
- Docker Image
- Installation
- Release Notes
- Changes in 0.7.5
- Changes in 0.7.4
- Changes in 0.7.3

Examples

- Annotating the human IGH locus
- Annotating the rhesus macaque IGH locus
- Targeted Annotation
- Additional Examples

Usage Documentation

- Commandline Usage
- Anotation format

Digger

- Installation
- View page source

---

# Installation

The latest stable version of Digger may be downloaded from PyPI. Digger requires Python 3.9 or above.

Development versions are available from GitHub.

Digger requires BLAST to be installed. If you use conda/anaconda on Linux or Mac we recommend installing BLAST with conda:

> > conda install bioconda::blast

Alternatively, please follow the link for installation instructions and see the note below. No BLAST databases are needed: just the executable.

The installation instructions at NCBI ask you to browse to the link https://ftp.ncbi.nlm.nih.gov/blast/executables/LATEST/. Browsers
are ceasing support for FTP, and you may find that your browser won’t open the page. The easiest approach in this case is to use curl to download the file. curl is included
in all Windows 10 and 11 installations.

> > curl https://ftp.ncbi.nlm.nih.gov/blast/executables/LATEST/

will list the latest versions available for download. For Windows, for example, you will see a filename similar to ncbi-blast-2.12.0+-win64.exe.

> > curl https://ftp.ncbi.nlm.nih.gov/blast/executables/LATEST/ncbi-blast-2.12.0+-win64.exe -o ncbi-blast-2.12.0+-win64.exe

will download the file to your current directory. You will need to modify the filename to reflect the listed version.

Once BLAST has been installed, following NCBI’s instructions, please verify by typing `blastn -help` at the command line: if everything is ok, it should provide
usage instructions. On Windows you will need to open a new command window to run the command.

If you encounter the error `Cannot allocate memory`, this is coming from BLAST makeblastdb. Please set the environment variable `BLASTDB_LMDB_MAP_SIZE=100000000`.

If you encounter the error `Error while loading shared libraries: libnsl.so.1`, the shared library `libnsl.so.1` needs to be installed. The precise instructions to do so are platform specific, but you should be able to obtain guidance through a Google search.

The easiest way to install Digger itself is with pip:

```
> pip install receptor-digger --user
```

Digger requires the Python packages receptor-utils and biopython. These should be installed automatically by pip.

Previous
Next

---

© Copyright 2023, William Lees.

Built with Sphinx using a
theme
provided by Read the Docs.
