# Supplementary material for: Genotyping of selected germline adaptive immune system loci using short-read sequencing data
Source: Genome Res. 2025 Sep;35(9):2076–86. doi: 10.1101/gr.280314.124 (PMC12401057; doi:10.1101/gr.280314.124)
Supplement: Supplement 1 [file Supplemental_Code.zip › ImmunoTyper2-methods/HPRC-assembly-benchmarking/digger/docs/_build/html/news.html]

Release Notes — Digger 0.5.0 documentation


Digger

Getting Started

- Overview
- digger
- dig-sequence
- Docker Image
- Installation
- Release Notes
- Changes in 0.7.5
- Changes in 0.7.4
- Changes in 0.7.3
  - Changes in 0.7.2
  - Changes in 0.7.1
  - Changes in 0.7.0
  - Changes in 0.6.9
  - Changes in 0.6.8
  - Changes in 0.6.7
  - Changes in 0.6.7
  - Changes in 0.6.6
  - Changes in 0.6.4
  - Changes in 0.6.3:
  - Changes in 0.6.2:
  - Changes in 0.6.1:
  - Changes in 0.6.0:
  - Changes in v 0.5.10:
  - Changes in v 0.5.9:
  - Changes in v 0.5.8:
  - Changes in v 0.5.7:
  - Changes in v 0.5.6:
  - Changes in v 0.5.5:
  - Changes in V 0.5.4:
  - Changes in V 0.5.3:
  - Changes in V 0.5.2:
  - Changes in V 0.5.1:
  - Version 0.5: April 2023

Examples

- Annotating the human IGH locus
- Annotating the rhesus macaque IGH locus
- Targeted Annotation
- Additional Examples

Usage Documentation

- Commandline Usage
- Anotation format

Digger

- Release Notes
- View page source

---

# Release Notes

# Changes in 0.7.5

- further salmonid name fix

# Changes in 0.7.4

- fix problem with salmonid names, e.g. TRB3V

# Changes in 0.7.3

- fix crash in compare\_annotations.py

## Changes in 0.7.2

- digger will default to using the starting set of reference genes for comparison, if no sets are specified with -ref\_comp.

## Changes in 0.7.1

- digger will now copy reference set files to the work directory if necessary, as blast’s makedb requires the files to be local.
- digger can now handle multiple sequences (e.g. contigs) in the assembly file.

## Changes in 0.7.0

- modify motif handling to allow multiple motifs in those cases where the length can be variable
- add variable length motifs for l-part1 and l-part2
- motifs are now based on the analysis in tests/utr
- add identification of TATA\_BOX and OCTAMER in promoter regions

## Changes in 0.6.9

- bump dependency on receptor-utils to fix an issue with naming of D novel alleles

## Changes in 0.6.8

- improve documentation, add additional examples in tests, add Dockerised version
- include TRG motif files in the package

## Changes in 0.6.7

- include motif files for TRG

## Changes in 0.6.7

- better error handling and explanation in parse\_imgt\_annotations
- Docker image added

## Changes in 0.6.6

- fix crash in dig\_sequence if annotating V-gene without specifying a gapped reference file
- update the package to include missing motif files

## Changes in 0.6.4

- fix issue in dig\_sequence

## Changes in 0.6.3:

- add support for multiple definitions of the J motif for a locus
- move J-TRP and other motif definitions to a file in the motifs directory

## Changes in 0.6.2:

- require the user to supply an email address for requests to GenBank.

## Changes in 0.6.1:

- bump required version of receptor-utils

## Changes in 0.6.0:

- added support for TRA, TRB, TRD, TRG and test cases and motifs for human TR loci

## Changes in v 0.5.10:

- fixed unintended breakpoint in find\_alignments which could fire under some circumstances

## Changes in v 0.5.9:

- fixed erroneous reporting of leader1 sequence and co-ordinates when leader1 was not included in the query sequence

## Changes in v 0.5.8:

- fixed various issues with end effects leading to the reporting of negative coordinates
- fixed an issue with targeted annotation which caused negative sense not to be properly reported
- changed IMGT urls to use https as http is no longer utilised

## Changes in v 0.5.7:

- modified reporting of nt\_diff so that it reports the number of nt differences between the query and the reference, including any length differences.
- updated the code to use Bio.Align rather than Bio.pairwise2, which is deprecated.

## Changes in v 0.5.6:

- compatibility update for receptor\_utils 0.0.40

## Changes in v 0.5.5:

- speed optimisation for dig\_sequence
- allow find\_alignments to be called without a -ref parameter

## Changes in V 0.5.4:

- Minor fixes to handling of non-functional sequences

## Changes in V 0.5.3:

- Fixes to annotation in reverse-sense: false positives were not being filtered correctly

## Changes in V 0.5.2:

- Added dig\_sequence command, which allows a sequence stored locally or in Genbank to be searched for a specific allele. The closest match will be annotated.

## Changes in V 0.5.1:

- Refactored elements of the code to make it more modular and easier to maintain.

## Version 0.5: April 2023

First public version.

Previous
Next

---

© Copyright 2023, William Lees.

Built with Sphinx using a
theme
provided by Read the Docs.
