# Supplementary material for: Genotyping of selected germline adaptive immune system loci using short-read sequencing data
Source: Genome Res. 2025 Sep;35(9):2076–86. doi: 10.1101/gr.280314.124 (PMC12401057; doi:10.1101/gr.280314.124)
Supplement: Supplement 1 [file Supplemental_Code.zip › ImmunoTyper2-methods/HPRC-assembly-benchmarking/digger/docs/_build/html/overview.html]

Overview — Digger 0.5.0 documentation


Digger

Getting Started

- Overview
- digger
  - Scope and Features
  - Pipeline
- dig-sequence
- Docker Image
- Installation
- Release Notes
- Changes in 0.7.5
- Changes in 0.7.4
- Changes in 0.7.3

Examples

- Annotating the human IGH locus
- Annotating the rhesus macaque IGH locus
- Targeted Annotation
- Additional Examples

Usage Documentation

- Commandline Usage
- Anotation format

Digger

- Overview
- View page source

---

# Overview

There are two principal tools: `digger` itself, and `dig-sequence`. `digger` performs de-novo annotation, searching for all germline sequences that are similar
to those in a reference set. `dig_sequence` searches for the single sequence that is most similar to a given reference sequence. Both tools use the same
underlying code. `dig_sequence` can be useful for determining the full annotation of a genomic sequence you have identified, or for verifying a sequence
discovered in GenBank. `digger` is slower, but performs the full annotation of a genomic sequence or assembly.

# digger

## Scope and Features

`digger` identifies immunoglobulin receptor germline sequences in a genomic sequence or assembly. It requires two inputs: the sequence or assembly, and a set of reference genes to use as a starting point. An existing
reference set for the species under study is an ideal starting point, but if one is not available, good results can be obtained by using the reference set for another species, preferably one that is
reasonably closely related. The package is intended to be straightforward to use, but some familiarity with command-line tools is expected.

`digger` starts by searching the sequence for approximate matches to sequences in the reference set. Where these are found, the match is extended to the full length of the matched sequence. A window
at either end is then checked for the expected flanking sequences (e.g. leader, RSS). These are identified by means of position weight matrices (PWMs). The flanking sequences have a well-established
‘canonical’ form that is conserved between species. `digger` contains PWMs for human IG/TR and rhesus macaque IG loci, and these can be used as a starting point for other species. However, as some variation
is observed between species, The toolkit also contains tools for deriving tailored PWMs for a species of interest. These can be obtained from an existing annotation of the locus, or from an initial
annotation conducted with the human or rhesus PWMs. Digger’s built-in PWM’s are based on analysis of selected IMGT annotations.

## Pipeline

A brief summary of the tools contained in the package is given below. In most cases, annotation can be performed with the digger tool. This will call subsidiary tools as necessary. Please refer
to the Examples section for more information. The first example, Annotating the human IGH locus, explains straightforward use. The second example, Annotating the rhesus macaque IGH locus, shows how
to generate PWMs for a locus or species that is not already covered by the package. It also shows how to call the underlying tools directly.

# dig-sequence

`dig_sequence` identifies and annotates the closest match to a single specified reference sequence. It provides three modes: one in which the sequence to search
is provided in a FASTA file, one in which it is specified as a GenBank accession number, and a third in which multiple GenBank accession numbers to search are provided
in a file. Its use is described in dig\_sequence.

| Tool | Description |
| --- | --- |
| blastresults\_to\_csv | Convert the result of a BLAST search to a simple tabular format |
| calc\_motifs | Determine PWMs from a list of gene features |
| compare\_annotations | Compare two sets of annotations |
| digger | Run the annotation pipeline |
| dig\_sequence | Targeted annotation of specific sequences |
| find\_alignments | Produce the annotation file, given tabulated BLAST results and other inputs |
| parse\_imgt\_annotations | Download and parse an annotation file from IMGT |

Previous
Next

---

© Copyright 2023, William Lees.

Built with Sphinx using a
theme
provided by Read the Docs.
