# Supplementary material for: Genotyping of selected germline adaptive immune system loci using short-read sequencing data
Source: Genome Res. 2025 Sep;35(9):2076–86. doi: 10.1101/gr.280314.124 (PMC12401057; doi:10.1101/gr.280314.124)
Supplement: Supplement 1 [file Supplemental_Code.zip › ImmunoTyper2-methods/HPRC-assembly-benchmarking/digger/docs/_build/html/tools/annotation.html]

Anotation format — Digger 0.5.0 documentation


Digger

Getting Started

- Overview
- digger
- dig-sequence
- Docker Image
- Installation
- Release Notes
- Changes in 0.7.5
- Changes in 0.7.4
- Changes in 0.7.3

Examples

- Annotating the human IGH locus
- Annotating the rhesus macaque IGH locus
- Targeted Annotation
- Additional Examples

Usage Documentation

- Commandline Usage
- Anotation format
  - Columns in the Annotation File
  - Functionality

Digger

- Anotation format
- View page source

---

# Anotation format

This page describes the annotation file produced by digger / find\_alignments

## Columns in the Annotation File

In addition to the columns in the first table, the file contains the columns in the second table, prefixed by the reference name, for each reference specified with a -ref argument.

| Column Name | Meaning |
| --- | --- |
| contig | ID of the sequence in which the gene or pseudogene was found |
| start | start co-ord of the coding region |
| end | end co-ord of the coding region |
| start\_rev | start co-ord in the reverse-primed sequence |
| end\_rev | end co-ord in the reverse-primed sequence |
| sense | sense (relative to the input sequence) |
| gene\_type | gene type (e.g. IGHV) |
| gene\_start start co-ord of the entire gene including flanking regions |  |
| gene\_end end co-ord of the entire gene including flanking regions |  |
| gene\_start start co-ord of the entire gene including flanking regions in the reverse-primed sequence |  |
| gene\_end end co-ord of the entire gene including flanking regions in the reverse-primed sequence |  |
| likelihood | likelihood that the RSS is that of a functional gene (compared to a random sequence) |
| l\_part1 | leader part 1 equence |
| l\_part2 | leader part 2 sequence |
| v\_heptamer | v-heptamer sequence |
| v\_nonamer | v-nonamer sequence |
| j\_heptamer | j-heptamer sequence |
| j\_nonamer | j-nonamer sequence |
| j\_frame | coding frame of the first nucleotide of the j region (0, 1 or 2) |
| d\_3\_heptamer | 3-prime d-heptamer sequence |
| d\_3\_nonamer | 3-prime d-nonamer sequence |
| d\_5\_heptamer | 5-prime d-heptamer sequence |
| d\_5\_nonamer | 5-prime d-nonamer sequence |
| functional | functionality (see below) |
| notes | annotation notes |
| aa | amino acid translation of the coding region |
| v-gene\_aligned\_aa | IMGT-gapped amino acid translation of the coding sequence (for V-genes) |
| seq | sequence of the coding region |
| seq\_gapped | IMGT-gapped sequence of the coding region (V-genes only) |
| 5\_rss\_start | co-ordinates of the 5-prime RSS |
| 5\_rss\_start\_rev |  |
| 5\_rss\_end |  |
| 5\_rss\_end\_rev |  |
| 3\_rss\_start | co-ordinates of the 3-prime RSS |
| 3\_rss\_start\_rev |  |
| 3\_rss\_end |  |
| 3\_rss\_end\_rev |  |
| l\_part1\_start | co-ordinates of the leader part 1 |
| l\_part1\_start\_rev |  |
| l\_part1\_end |  |
| l\_part1\_end\_rev |  |
| l\_part2\_start | co-ordinates of the leader part 2 |
| l\_part2\_start\_rev |  |
| l\_part2\_end |  |
| l\_part2\_end\_rev |  |
| matches | number of matches to this start/end region that were produced in the BLAST analysis |
| blast\_match | gene in the reference file with the highest match score in this start/end region |
| blast\_score | the highest BLAST match score in this start/end region |
| blast\_nt\_diffs | the number of nucleotides differing from the most highly scoring reference sequence in this BLAST match |
| evalue | evalue of the most highly scoring BLAST match in this start/end region |

Columns provided for each -ref:

| Column Name | Meaning |
| --- | --- |
| \_match | ID of the closest matching reference gene |
| \_score | score of the closest match |
| \_nt\_diffs | number of nucleotides differing from the closest reference sequence |

## Functionality

Functionality is assigned as follows:

Functional

- RSS and leader meet or exceed position-weighted matrix threshold
- Highly-conserved nucleotides agree with the definition for the locus, if a definition has been specified
- If a V-gene, leader starts with ATG, and spliced leader has no stop codons
- If a V-gene, coding region has no stop codons before the cysteine at IMGT position 104
- If a V-gene, conserved nucleotides are at the expected locations
- If a J-gene, donor splice is as expected and coding region has no stop codons

ORF

- One or more of the above conditions are not met, but no stop codon has been detected
- If a V-gene, leader starts with ATG

Pseudo

- Coding region contains stop codon(s)
- Leader does not start with ATG

Previous

---

© Copyright 2023, William Lees.

Built with Sphinx using a
theme
provided by Read the Docs.
