# Supplementary material for: Genotyping of selected germline adaptive immune system loci using short-read sequencing data
Source: Genome Res. 2025 Sep;35(9):2076–86. doi: 10.1101/gr.280314.124 (PMC12401057; doi:10.1101/gr.280314.124)
Supplement: Supplement 1 [file Supplemental_Code.zip › ImmunoTyper2-methods/HPRC-assembly-benchmarking/digger/docs/_build/html/tools/blastresults_to_csv.html]

blastresults\_to\_csv — Digger 0.5.0 documentation


Digger

Getting Started

- Overview
- digger
- dig-sequence
- Docker Image
- Installation
- Release Notes
- Changes in 0.7.5
- Changes in 0.7.4
- Changes in 0.7.3

Examples

- Annotating the human IGH locus
- Annotating the rhesus macaque IGH locus
- Targeted Annotation
- Additional Examples

Usage Documentation

- Commandline Usage
  - blastresults\_to\_csv
    - Positional Arguments
    - Named Arguments
  - calc\_motifs
  - compare\_annotations
  - digger
  - dig\_sequence
  - find\_alignments
  - parse\_imgt\_annotations
- Anotation format

Digger

- Commandline Usage
- blastresults\_to\_csv
- View page source

---

# blastresults\_to\_csv

`blastresults_to_csv` converts the output of a blast search in ‘format 7’ into csv format. If there were multiple query sequences, results are split into separate files.
Please refer to Annotating the rhesus macaque IGH locus for example usage of this and the other ‘individual’ commands.

Convert blast file format 7 to one or more CSVs

```
usage: blastresults_to_csv [-h] [-a] infile out_prefix
```

## Positional Arguments

`infile`
:   the blast file

`out_prefix`
:   prefix for csv files

## Named Arguments

`-a, --append`
:   append to existing output files

    Default: False

Previous
Next

---

© Copyright 2023, William Lees.

Built with Sphinx using a
theme
provided by Read the Docs.
