# Supplementary material for: Genotyping of selected germline adaptive immune system loci using short-read sequencing data
Source: Genome Res. 2025 Sep;35(9):2076–86. doi: 10.1101/gr.280314.124 (PMC12401057; doi:10.1101/gr.280314.124)
Supplement: Supplement 1 [file Supplemental_Code.zip › ImmunoTyper2-methods/HPRC-assembly-benchmarking/digger/docs/_build/html/tools/calc_motifs.html]

calc\_motifs — Digger 0.5.0 documentation


Digger

Getting Started

- Overview
- digger
- dig-sequence
- Docker Image
- Installation
- Release Notes
- Changes in 0.7.5
- Changes in 0.7.4
- Changes in 0.7.3

Examples

- Annotating the human IGH locus
- Annotating the rhesus macaque IGH locus
- Targeted Annotation
- Additional Examples

Usage Documentation

- Commandline Usage
  - blastresults\_to\_csv
  - calc\_motifs
    - Positional Arguments
  - compare\_annotations
  - digger
  - dig\_sequence
  - find\_alignments
  - parse\_imgt\_annotations
- Anotation format

Digger

- Commandline Usage
- calc\_motifs
- View page source

---

# calc\_motifs

`calc_motifs` creates motif files for RSS and leader fields, based on the features produced by parse\_imgt\_annotations. Only annotations
of sequences marked as ‘functional’ are considered.

`calc_motifs` also creates a file containing definitions of other motif parameters for the locus. These are copied from the values used for
human locus, but may be modified if necessary. The following values are included:

| Parameter | Definition |
| --- | --- |
| J\_TRP\_MOTIF | The J amino acid motif to search for. May be a single value, or a Python list, e.g. [“FGXG”, “WGXG”] |
| J\_TRP\_OFFSET | The number of codons between and including the start of the J motif and the final codon before the J-SPLICE |
| J\_SPLICE | The expected nt sequence of the J-SPLICE |
| V\_RSS\_SPACING | The expected length of the V-SPACER |
| J\_RSS\_SPACING | The expected length of the J-SPACER |
| D\_5\_RSS\_SPACING | The expected length of the 5’ D-SPACER (0 if this is not a heavy chain) |
| D\_3\_RSS\_SPACING | The expected length of the 3’ D-SPACER (0 if this is not a heavy chain) |

Please refer to Annotating the rhesus macaque IGH locus for example usage of this and the other ‘individual’ commands.

When calc\_motifs runs, it provides a summary of the sequences it has processed. Please check these for sanity: for example check that the consensus
heptamer is 7nt long, and so on. Errors in the motif table will impact annotation.

Given a set of gene features, create motif matrices

```
usage: calc_motifs [-h] locus feat_file
```

## Positional Arguments

`locus`
:   locus (e.g. IGH, TRA)

`feat_file`
:   feature file, created, for example, by parse\_imgt\_annotations

Previous
Next

---

© Copyright 2023, William Lees.

Built with Sphinx using a
theme
provided by Read the Docs.
