# Supplementary material for: Genotyping of selected germline adaptive immune system loci using short-read sequencing data
Source: Genome Res. 2025 Sep;35(9):2076–86. doi: 10.1101/gr.280314.124 (PMC12401057; doi:10.1101/gr.280314.124)
Supplement: Supplement 1 [file Supplemental_Code.zip › ImmunoTyper2-methods/HPRC-assembly-benchmarking/digger/docs/_build/html/tools/compare_annotations.html]

compare\_annotations — Digger 0.5.0 documentation


Digger

Getting Started

- Overview
- digger
- dig-sequence
- Docker Image
- Installation
- Release Notes
- Changes in 0.7.5
- Changes in 0.7.4
- Changes in 0.7.3

Examples

- Annotating the human IGH locus
- Annotating the rhesus macaque IGH locus
- Targeted Annotation
- Additional Examples

Usage Documentation

- Commandline Usage
  - blastresults\_to\_csv
  - calc\_motifs
  - compare\_annotations
    - Positional Arguments
    - Named Arguments
  - digger
  - dig\_sequence
  - find\_alignments
  - parse\_imgt\_annotations
- Anotation format

Digger

- Commandline Usage
- compare\_annotations
- View page source

---

# compare\_annotations

`compare_annotations` compares annotations produced by digger with IMGT’s annotations as summarised by parse\_annotations. Three files are produced:
- a .jpg showing Venn diagrams of identified functional annotations
- a .txt file summarising the specific functional sequences that were only identified in one annotation as opposed to both
- a .csv file listing agreements and differences of all sequences annotated by either mmethod
Please refer to Annotating the human IGH locus for example usage.

Compare digger results to an IMGT annotation

```
usage: compare_annotations [-h] [-nc] [--filter_annot FILTER_ANNOT] [--comp_name COMP_NAME] [--target_locus TARGET_LOCUS] digger_results annotation_file sense outfile
```

## Positional Arguments

`digger_results`
:   Digger results file

`annotation_file`
:   IMGT annotation produced by parse\_imgt\_assembly\_x.py

`sense`
:   Sense of annotation compared to digger results (forward or reverse)

`outfile`
:   Output file name (will create .csv, .jpg, .txt

## Named Arguments

`-nc`
:   include sequences for leader and rss

    Default: False

`--filter_annot`
:   filter IMGT annotations by sense (forward or reverse)

`--comp_name`
:   name to use for comparison (default IMGT)

`--target_locus`
:   Only consider IMGT matches to the target locus (used for TRA/TRD)

Previous
Next

---

© Copyright 2023, William Lees.

Built with Sphinx using a
theme
provided by Read the Docs.
