# Supplementary material for: Genotyping of selected germline adaptive immune system loci using short-read sequencing data
Source: Genome Res. 2025 Sep;35(9):2076–86. doi: 10.1101/gr.280314.124 (PMC12401057; doi:10.1101/gr.280314.124)
Supplement: Supplement 1 [file Supplemental_Code.zip › ImmunoTyper2-methods/HPRC-assembly-benchmarking/digger/docs/_build/html/tools/dig_sequence.html]

dig\_sequence — Digger 0.5.0 documentation


Digger

Getting Started

- Overview
- digger
- dig-sequence
- Docker Image
- Installation
- Release Notes
- Changes in 0.7.5
- Changes in 0.7.4
- Changes in 0.7.3

Examples

- Annotating the human IGH locus
- Annotating the rhesus macaque IGH locus
- Targeted Annotation
- Additional Examples

Usage Documentation

- Commandline Usage
  - blastresults\_to\_csv
  - calc\_motifs
  - compare\_annotations
  - digger
  - dig\_sequence
    - Sub-commands
      - fasta
      - single
      - multi
      - multi\_seq
  - find\_alignments
  - parse\_imgt\_annotations
- Anotation format

Digger

- Commandline Usage
- dig\_sequence
- View page source

---

# dig\_sequence

`dig_sequence` provides targeted annotation of genomic sequences. The sequences can be as small as a single coding region,
or as large as an entire locus. The tool will search for the sequence that best matches a specified target sequence, and
annotate just that best match.

Options are available to annotate a single sequence in a FASTA file, a single genbank ID (the sequence will be fetched from
Genbank), or a list of sequences or Genbank IDs specified in a CSV file. For GenBank requests, an email address must be
provided, as this is a requirement of the GenBank API.

Example usage is described in Targeted Annotation.

Annotate a genomic sequence representing the nominated receptor gene

```
usage: dig_sequence [-h] {fasta,single,multi,multi_seq} ...
```

## Sub-commands

### fasta

Annotate a single genomic sequence in a FASTA file

```
dig_sequence fasta [-h] [-align ALIGN] [-species SPECIES] [-motif_dir MOTIF_DIR] [-out_file OUT_FILE] [-debug] target germline_file query_file
```

#### Positional Arguments

`target`
:   Name of nominated sequence in reference set

`germline_file`
:   ungapped reference set containing the nominated sequence (FASTA)

`query_file`
:   file containing the sequence to annotate (FASTA)

#### Named Arguments

`-align`
:   gapped reference set to use for V gene alignments (required for V gene analysis

`-species`
:   use motifs for the specified species provided with the package

`-motif_dir`
:   use motif probability files present in the specified directory

`-out_file`
:   output file (CSV)

`-debug`
:   produce parsing\_errors file with debug information

    Default: False

### single

Annotate a single sequence given its genbank accession number

```
dig_sequence single [-h] [-align ALIGN] [-species SPECIES] [-motif_dir MOTIF_DIR] [-out_file OUT_FILE] target germline_file genbank_acc email_addr
```

#### Positional Arguments

`target`
:   Name of nominated sequence

`germline_file`
:   ungapped reference set containing the nominated sequence (FASTA)

`genbank_acc`
:   genbank accession number of the sequence to annotate

`email_addr`
:   email address to provide to genbank

#### Named Arguments

`-align`
:   gapped reference set to use for V gene alignments (required for V gene analysis

`-species`
:   use motifs for the specified species provided with the package

`-motif_dir`
:   use motif probability files present in the specified directory

`-out_file`
:   output file (CSV)

### multi

Read allele names and corresponding genbank accession numbers from a CSV file

```
dig_sequence multi [-h] [-align ALIGN] [-species SPECIES] [-motif_dir MOTIF_DIR] [-out_file OUT_FILE] locus germline_file query_file email_addr
```

#### Positional Arguments

`locus`
:   Locus of nominated sequences

`germline_file`
:   ungapped reference set containing the nominated sequence (FASTA)

`query_file`
:   File containing list of targets and associated genbank accession numbers (CSV)

`email_addr`
:   email address to provide to genbank

#### Named Arguments

`-align`
:   gapped reference set to use for V gene alignments (required for V gene analysis

`-species`
:   use motifs for the specified species provided with the package

`-motif_dir`
:   use motif probability files present in the specified directory

`-out_file`
:   output file (CSV)

### multi\_seq

Read allele names and genomic sequences from a CSV file

```
dig_sequence multi_seq [-h] [-align ALIGN] [-species SPECIES] [-motif_dir MOTIF_DIR] [-out_file OUT_FILE] locus germline_file query_file
```

#### Positional Arguments

`locus`
:   Locus of nominated sequences

`germline_file`
:   ungapped reference set containing the nominated sequence (FASTA)

`query_file`
:   File containing list of targets and genomic sequences (CSV)

#### Named Arguments

`-align`
:   gapped reference set to use for V gene alignments (required for V gene analysis

`-species`
:   use motifs for the specified species provided with the package

`-motif_dir`
:   use motif probability files present in the specified directory

`-out_file`
:   output file (CSV)

Previous
Next

---

© Copyright 2023, William Lees.

Built with Sphinx using a
theme
provided by Read the Docs.
