# Supplementary material for: Genotyping of selected germline adaptive immune system loci using short-read sequencing data
Source: Genome Res. 2025 Sep;35(9):2076–86. doi: 10.1101/gr.280314.124 (PMC12401057; doi:10.1101/gr.280314.124)
Supplement: Supplement 1 [file Supplemental_Code.zip › ImmunoTyper2-methods/HPRC-assembly-benchmarking/digger/docs/_build/html/tools/digger.html]

digger — Digger 0.5.0 documentation


Digger

Getting Started

- Overview
- digger
- dig-sequence
- Docker Image
- Installation
- Release Notes
- Changes in 0.7.5
- Changes in 0.7.4
- Changes in 0.7.3

Examples

- Annotating the human IGH locus
- Annotating the rhesus macaque IGH locus
- Targeted Annotation
- Additional Examples

Usage Documentation

- Commandline Usage
  - blastresults\_to\_csv
  - calc\_motifs
  - compare\_annotations
  - digger
    - Positional Arguments
    - Named Arguments
  - dig\_sequence
  - find\_alignments
  - parse\_imgt\_annotations
- Anotation format

Digger

- Commandline Usage
- digger
- View page source

---

# digger

`digger` annotates the sequences contained in a FASTA file, using BLAST to search for potential germline sequences. It requires an initial reference set
for BLAST to use: this could come from a similar species, or a former annotation.
Please refer to Annotating the human IGH locus for example usage.

Find functional and nonfunctional genes in an assembly sequence or contigs

```
usage: digger [-h] [-species SPECIES] [-motif_dir MOTIF_DIR] [-locus LOCUS] [-v_ref V_REF] [-d_ref D_REF] [-j_ref J_REF] [-v_ref_gapped V_REF_GAPPED] [-ref_comp REF_COMP] [-sense SENSE] [-keepwf]
              assembly_file output_file
```

## Positional Arguments

`assembly_file`
:   File containing one or more sequences to search

`output_file`
:   output file (csv)

## Named Arguments

`-species`
:   use motifs for the specified species provided with the package

`-motif_dir`
:   pathname to directory containing motif probability files

`-locus`
:   locus (default is IGH)

`-v_ref`
:   set of V reference genes to use as starting point for search

`-d_ref`
:   set of D reference genes to use as starting point for search

`-j_ref`
:   set of J reference genes to use as starting point for search

`-v_ref_gapped`
:   IMGT-gapped v-reference set used to determine alignment of novel sequences

`-ref_comp`
:   ungapped reference set(s) to compare to: name and reference file separated by comma eg mouse,mouse.fasta (may be repeated multiple times)

`-sense`
:   sense in which to read the assembly (forward or reverse) (if omitted will select automatically)

`-keepwf`
:   keep working files after processing has completed

    Default: False

At least one file containing reference genes must be provided. You can, for example, supply `v_ref`, `d_ref` and `j_ref`, or just `v_ref`.
These reference files should just contain the sequence of the core coding region (in IMGT terminology, the V-, D- or J- REGION). They should not
include the V leader sequence, or any RSS. Digger will annotate whatever genes are discovered with the corresponding set(s).
In practice, the sets do not have to be that good a match: BLAST will identify partial matches, and Digger’s logic will extend the match to a full gene, including canonical RSS and leader (using the `motif` folder).

Digger requires a set of postion-weighted matrices, to identify RSS and leader. It is also possible to specify conserved locations of motifs. This motif data should be stored in a `motif` folder. Motifs for
human and rhesus macaque IG are built in to the package, and may be used with `-species` by specifying either `human` or `rhesus_macaque`. The species is used in conjunction with `-locus` to determine
the correct motifs. Alternatively, `-motif_dir` can be used to specify custom motifs created outside of the package. Please refer to calc\_motifs and to Annotating the rhesus macaque IGH locus for further details
on custom motifs.

`v_ref_gapped` is used to gep v-sequences correctly in order to identify conserved codons and so on. Again these sequences do not need to be that good a match in practice. The sequences **must be IMGT aligned with
no extraneous codons**. Note in particular that IMGT has introduced insertions into macaque alignments in recent years. **Sets with these insertions should not be used**.

`ref_comp` allows you to specify that you would like annotated sequences to be compared with sequences in a set. You can include as many different sets as you wish. The output file will contain columns
for each of these, listing the closest sequence found and the proximity (%, and number of nucleotides).

If you choose not to specify the `sense`, Digger will select the sense that elicits the highest number of hits and the highest evalue (results are shown in the output so that you can decide whether it has made the right choice,
and whether you wish to annotate in both senses)

Previous
Next

---

© Copyright 2023, William Lees.

Built with Sphinx using a
theme
provided by Read the Docs.
