# Supplementary material for: Genotyping of selected germline adaptive immune system loci using short-read sequencing data
Source: Genome Res. 2025 Sep;35(9):2076–86. doi: 10.1101/gr.280314.124 (PMC12401057; doi:10.1101/gr.280314.124)
Supplement: Supplement 1 [file Supplemental_Code.zip › ImmunoTyper2-methods/HPRC-assembly-benchmarking/digger/docs/_build/html/tools/find_alignments.html]

find\_alignments — Digger 0.5.0 documentation


Digger

Getting Started

- Overview
- digger
- dig-sequence
- Docker Image
- Installation
- Release Notes
- Changes in 0.7.5
- Changes in 0.7.4
- Changes in 0.7.3

Examples

- Annotating the human IGH locus
- Annotating the rhesus macaque IGH locus
- Targeted Annotation
- Additional Examples

Usage Documentation

- Commandline Usage
  - blastresults\_to\_csv
  - calc\_motifs
  - compare\_annotations
  - digger
  - dig\_sequence
  - find\_alignments
    - Positional Arguments
    - Named Arguments
  - parse\_imgt\_annotations
- Anotation format

Digger

- Commandline Usage
- find\_alignments
- View page source

---

# find\_alignments

`find_alignments` takes the output of a blast search (as formatted by blastresults\_to\_csv.py), checks each identified location for the presence of a gene, and annotates if found.
Please refer to Annotating the rhesus macaque IGH locus for example usage of this and the other ‘individual’ commands.

Find valid genes in a contig given blast matches

```
usage: find_alignments [-h] [-species SPECIES] [-motif_dir MOTIF_DIR] [-ref REF] [-align ALIGN] [-locus LOCUS] [-sense SENSE] [-debug] germline_file assembly_file blast_file output_file
```

## Positional Arguments

`germline_file`
:   reference set used to produce the blast matches

`assembly_file`
:   assembly or contig provided to blast

`blast_file`
:   results from blast in the format provided by blastresults\_to\_csv (can contain wildcards if there are multiple files, will be matched by glob)

`output_file`
:   output file (csv)

## Named Arguments

`-species`
:   use motifs for the specified species provided with the package

`-motif_dir`
:   use motif probability files present in the specified directory

`-ref`
:   ungapped reference to compare to: name and reference file separated by comma eg mouse,mouse.fasta (may be repeated multiple times)

`-align`
:   gapped reference file to use for V gene alignments (should contain V genes only), otherwise de novo alignment will be attempted

`-locus`
:   locus (default is IGH)

`-sense`
:   sense in which to read the assembly (forward or reverse) (will select automatically)

`-debug`
:   produce parsing\_errors file with debug information

    Default: False

Previous
Next

---

© Copyright 2023, William Lees.

Built with Sphinx using a
theme
provided by Read the Docs.
