# Supplementary material for: Genotyping of selected germline adaptive immune system loci using short-read sequencing data
Source: Genome Res. 2025 Sep;35(9):2076–86. doi: 10.1101/gr.280314.124 (PMC12401057; doi:10.1101/gr.280314.124)
Supplement: Supplement 1 [file Supplemental_Code.zip › ImmunoTyper2-methods/HPRC-assembly-benchmarking/digger/docs/_build/html/tools/parse_imgt_annotations.html]

parse\_imgt\_annotations — Digger 0.5.0 documentation


Digger

Getting Started

- Overview
- digger
- dig-sequence
- Docker Image
- Installation
- Release Notes
- Changes in 0.7.5
- Changes in 0.7.4
- Changes in 0.7.3

Examples

- Annotating the human IGH locus
- Annotating the rhesus macaque IGH locus
- Targeted Annotation
- Additional Examples

Usage Documentation

- Commandline Usage
  - blastresults\_to\_csv
  - calc\_motifs
  - compare\_annotations
  - digger
  - dig\_sequence
  - find\_alignments
  - parse\_imgt\_annotations
    - Positional Arguments
    - Named Arguments
- Anotation format

Digger

- Commandline Usage
- parse\_imgt\_annotations
- View page source

---

# parse\_imgt\_annotations

`parse_imgt_annotations` downloads an IMGT annotation file or or uses a file already downloaded. It parses the file to provide a list of annotated features.
Optionally it will also store the file downloaded, and create a FASTA file containing the annotated assembly.
Please refer to Annotating the human IGH locus for example usage.

Given a set of IMGT annotations, build a CSV file containing gene names and co-ordinates

```
usage: parse_imgt_annotations [-h] [--save_download SAVE_DOWNLOAD] [--save_sequence SAVE_SEQUENCE] [--save_imgt_annots SAVE_IMGT_ANNOTS] imgt_url outfile locus
```

## Positional Arguments

`imgt_url`
:   URL of IMGT annotation, e.g. http://www.imgt.org/ligmdb/view?format=IMGT&id=IMGT000064, or name of text file containing its contents

`outfile`
:   Output file (CSV)

`locus`
:   one of IGH, IGK, IGL, TRA, TRB, TRD, TRG

## Named Arguments

`--save_download`
:   Save contents of annotation to specified file

`--save_sequence`
:   Save sequence to specified file

`--save_imgt_annots`
:   Save IMGT annotations to specified file

Previous
Next

---

© Copyright 2023, William Lees.

Built with Sphinx using a
theme
provided by Read the Docs.
