# Supplementary material for: Genotyping of selected germline adaptive immune system loci using short-read sequencing data
Source: Genome Res. 2025 Sep;35(9):2076–86. doi: 10.1101/gr.280314.124 (PMC12401057; doi:10.1101/gr.280314.124)
Supplement: Supplement 1 [file Supplemental_Code.zip › ImmunoTyper2-methods/HPRC-assembly-benchmarking/digger/docs/pipeline.pptx]

## Slide 1
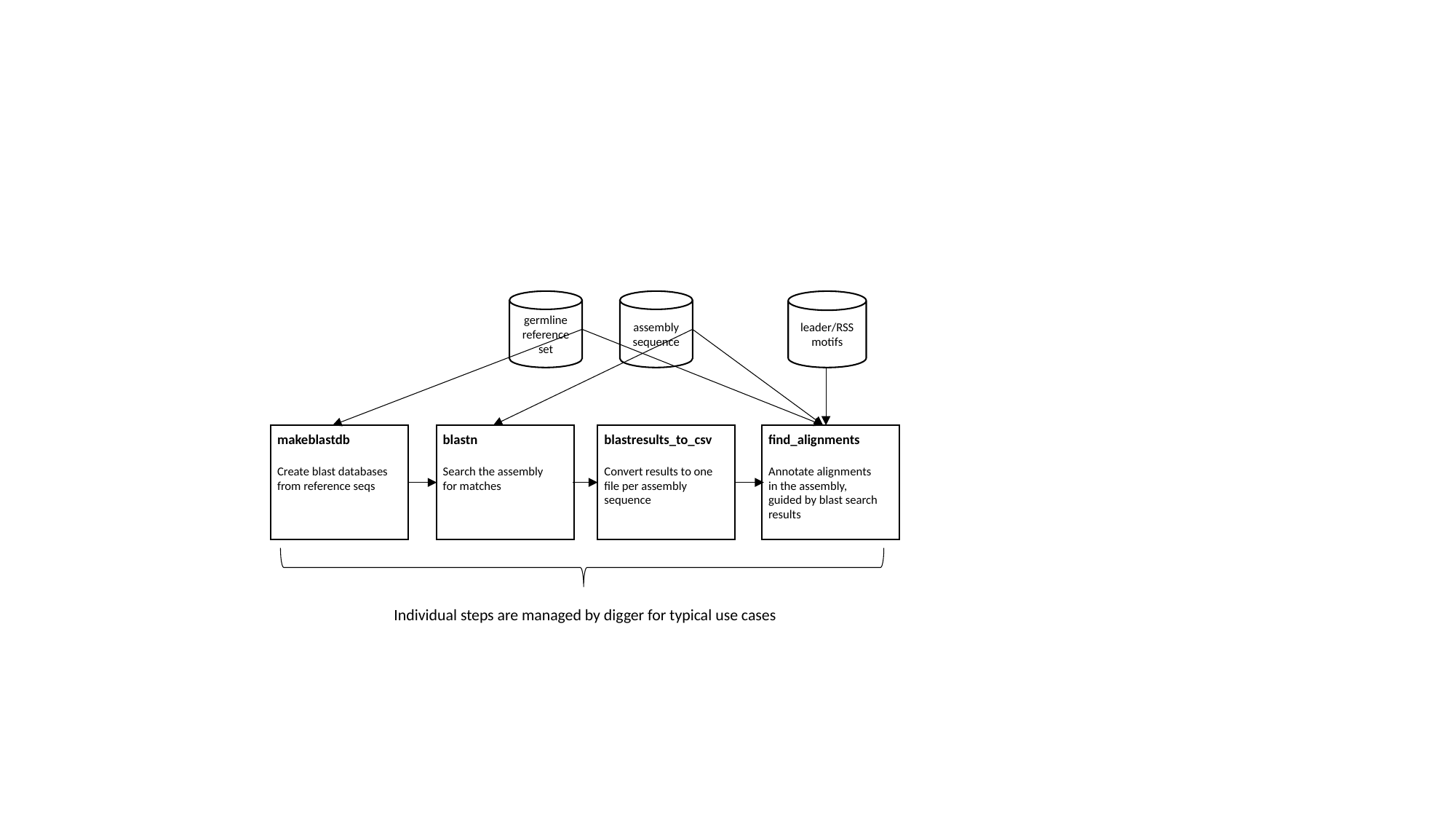

germline
reference
set
assembly
sequence
leader/RSS
motifs
makeblastdb
Create blast databases
from reference seqs
blastn
Search the assembly
for matches
blastresults_to_csv
Convert results to one
file per assembly
sequence
find_alignments
Annotate alignments
in the assembly,
guided by blast search
results
Individual steps are managed by digger for typical use cases
