# Supplementary figures and images for: Genotyping of selected germline adaptive immune system loci using short-read sequencing data
Source: Genome Res. 2025 Sep;35(9):2076–86. doi: 10.1101/gr.280314.124 (PMC12401057; doi:10.1101/gr.280314.124)

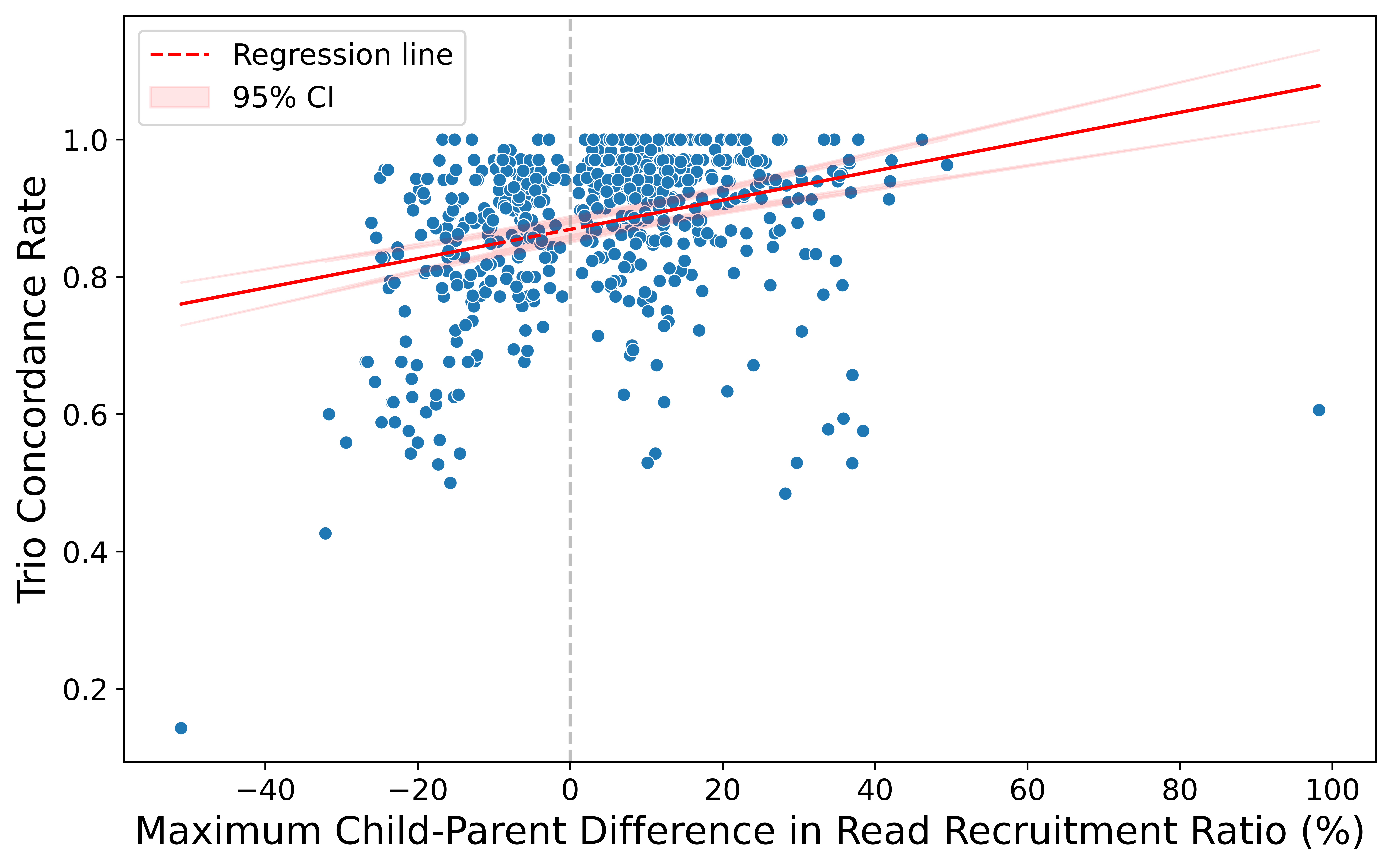

Supplement: Supplement 1 [file Supplemental_Code.zip › ImmunoTyper2-methods/1KGP_Trios/immunotyper-analysis/figures/read_recruitment_change_vs_concordance.png]

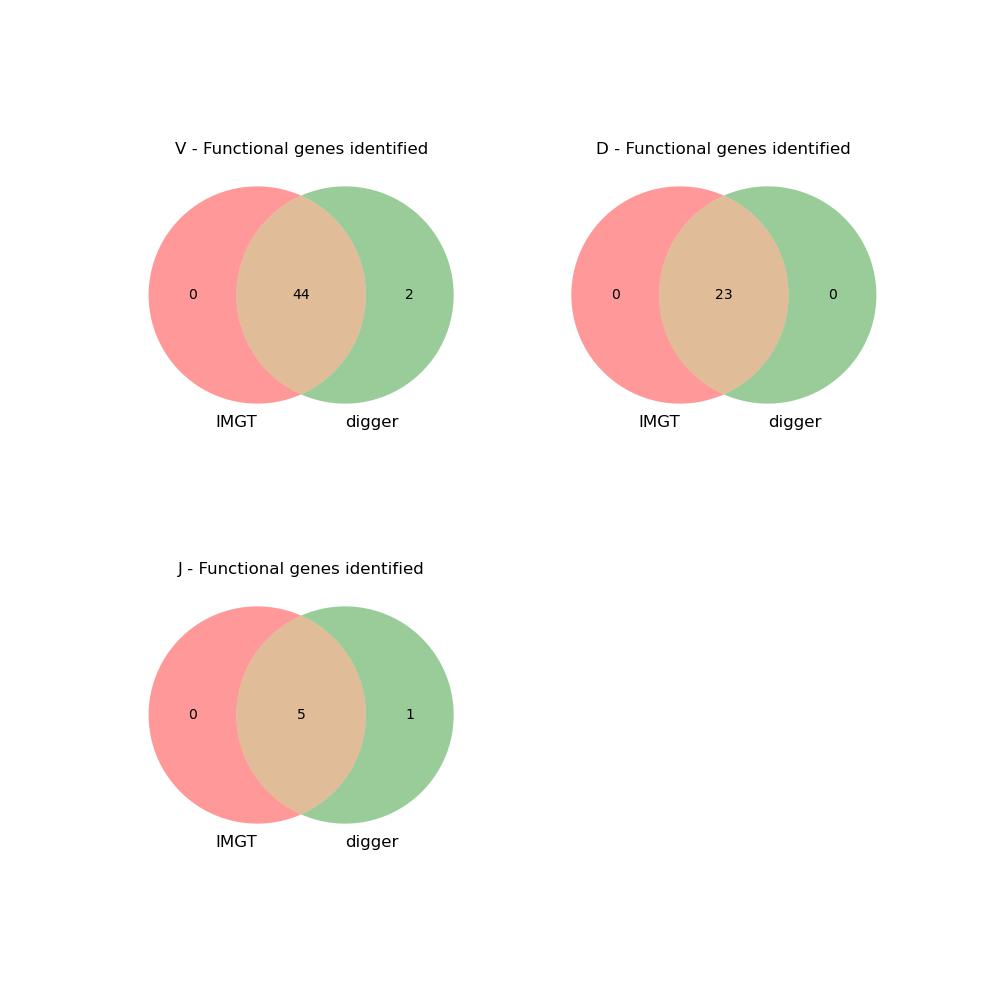

Supplement: Supplement 1 [file Supplemental_Code.zip › ImmunoTyper2-methods/HPRC-assembly-benchmarking/digger/docs/_build/html/_images/igh_results.jpg]

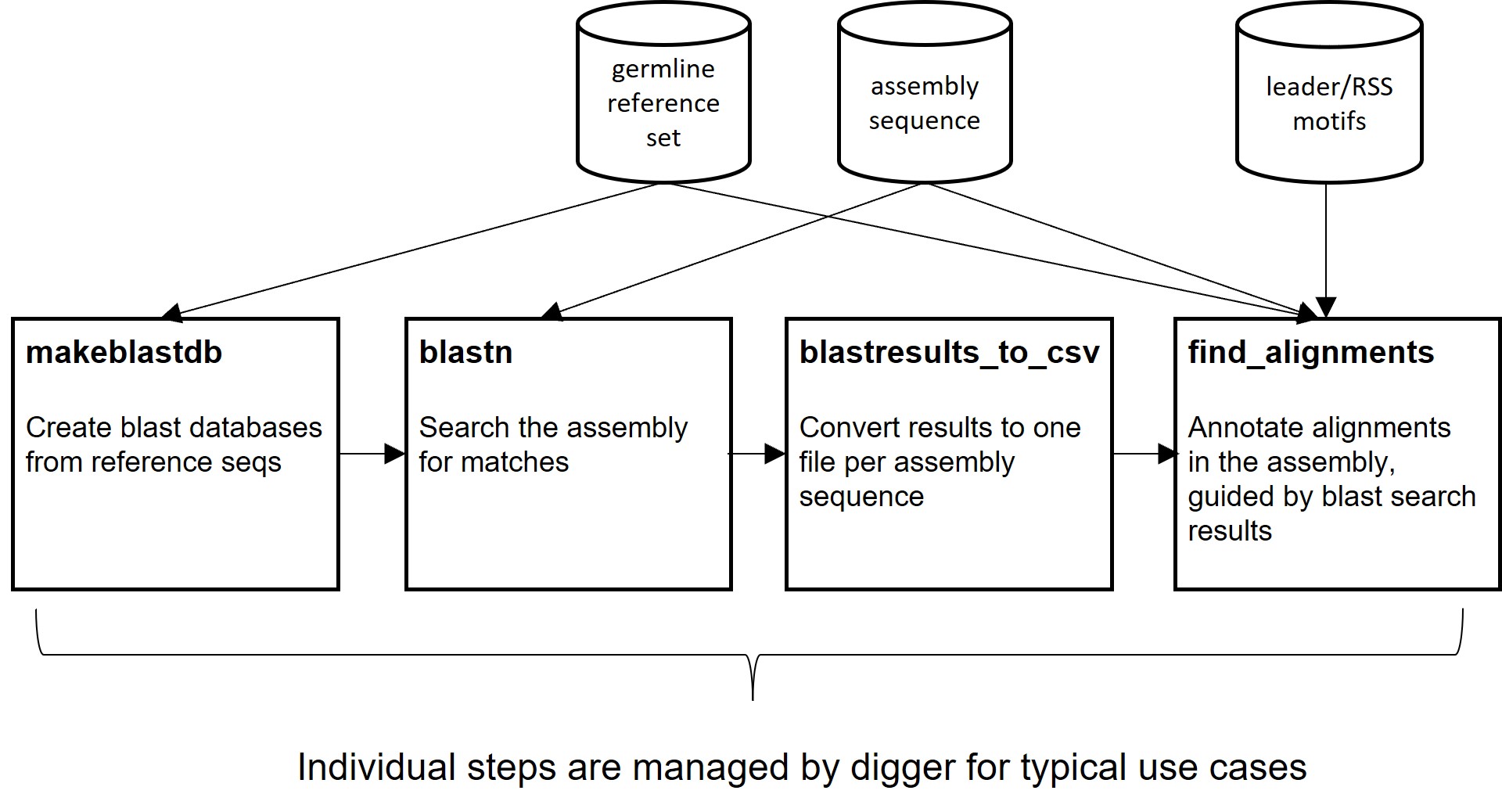

Supplement: Supplement 1 [file Supplemental_Code.zip › ImmunoTyper2-methods/HPRC-assembly-benchmarking/digger/docs/_build/html/_images/pipeline.jpg]

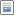

Supplement: Supplement 1 [file Supplemental_Code.zip › ImmunoTyper2-methods/HPRC-assembly-benchmarking/digger/docs/_build/html/_static/file.png]

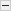

Supplement: Supplement 1 [file Supplemental_Code.zip › ImmunoTyper2-methods/HPRC-assembly-benchmarking/digger/docs/_build/html/_static/minus.png]

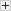

Supplement: Supplement 1 [file Supplemental_Code.zip › ImmunoTyper2-methods/HPRC-assembly-benchmarking/digger/docs/_build/html/_static/plus.png]
