# Supplementary material for: Genotyping of selected germline adaptive immune system loci using short-read sequencing data
Source: Genome Res. 2025 Sep;35(9):2076–86. doi: 10.1101/gr.280314.124 (PMC12401057; doi:10.1101/gr.280314.124)
Supplement: Supplement 2 [file Supplemental_Materials.pdf]

Supplemental Material – Genotyping of selected germline adaptive immune system loci using short-read sequencing data

1 ILP Formulation

Definitions We start with some notation and definitions.

We denote by $\mathbf{R} = \{r_i\}_{i=1}^{n}$ the set of all WGS reads in the sample.

Let $\mathbf{A} = \{a_j\}_{j=1}^{m}$ be the set of database alleles and let $\mathbf{C}_i$ be the set of candidate alleles of read r_i.

Now let $\mathbf{L}_{j,g}$ be a set of landmark nucleotide positions in a_j that are part of landmark group g, where g is a subset of all positions in a_j. $\mathbf{L}_j$ is the set of all landmark positions in allele a_j.

We denote by $covers(r_i, a_j, l)$ the set of reads that have a_j as candidate/mapping and cover landmark l.

Let $\mu_{l,j}$ and $\sigma_{l,j}$ be the expected mean and standard deviation (SD) of read coverage of allele a_j for a given landmark $l \in \mathbf{L}_j$ for a single copy of the allele. (The mean coverage and the standard deviation depends on the locus of the landmark within the allele. A landmark in the center of the allele will typically have the mean coverage and standard deviation that we have observed in Chromosome 1. A landmark closer to the margins of the allele will have a lower coverage and higher relative standard deviation.)

Let λ = user-provided upper bound on the how much the actual coverage can deviate from the expected coverage in terms of the standard deviation calculated for the read coverage of any reference allele. The default is 1.5 standard deviations.

Let min_cov = user-defined minimum allowed coverage of any landmark position, defined as the proportion of the estimated mean read coverage of that landmark position. The default is 0.3.

Let $e(r_i, a_j)$ = edit distance for the best mapping/alignment of r_i on a_j.

Let $expected\_errors(r_i)$ = sequencing error rate $\times$ length of the primary mapping of the read r_i to the allele database.

Let $discard\_penalty\_multiplier$ = user-provided penalty for discarding a read, as a multiple for the number expected number of read errors for a read. The default is 2, i.e., discarding the read will have twice the penalty than aligning the read to an allele with edit distance equal to the expected number of sequencing errors.

Variables

$$\text{Let } D_i^j = \begin{cases} 1 & \text{if } r_i \text{ has been assigned to } a_j \\ 0 & \text{otherwise} \end{cases}$$

$$\text{Let } X_i = \begin{cases} 1 & \text{if } r_i \text{ is discarded} \\ 0 & \text{otherwise} \end{cases}$$

$$\text{Let } C_{c,j} = \begin{cases} 1 & \text{if } a_j \text{ has } c \text{ copies called} \\ 0 & \text{otherwise,} \end{cases}$$

here $c \in \{0, \ldots, K\}$ where K is the maximum allowed number of copies per allele.

Constraints

$$\forall a_j, \quad \sum_c C_{c,j} = 1 \tag{1}$$

$$\forall r_i, \quad \sum_{a_j \in \mathbf{C}_i} D_i^j = 1 - X_i \tag{2}$$

$$\forall a_j, \quad n \cdot \sum_c C_{c,j} \geq \sum_{r_i} D_i^j \text{ where } n \text{ is the total number of reads} \tag{3}$$

$$\forall a_j, \quad \sum_{r_i} D_i^j \geq \sum_c c \cdot C_{c,j} \tag{4}$$

$$\forall a_j, \forall l \in \mathbf{L}_{j,g}, \quad \sum_{r_i \in covers(r_i, a_j, l)} D_i^j \geq min\_cov \cdot \mu_{l,j} \cdot \sum_c c \cdot C_{c,j} \tag{5}$$

$$\forall \mathbf{L}_{j,g}, \quad \sum_{l \in \mathbf{L}_{j,g}} \left| \sum_c \mu_{l,j} \cdot c \cdot C_{c,j} - \sum_{r_i \in covers(r_i, a_j, l)} D_i^j \right| \leq \sum_{l \in \mathbf{L}_{j,g}} \sum_c \lambda \cdot \sigma_{l,j} \cdot \sqrt{c} \cdot C_{c,j} \tag{6}$$

The constraints above can be interpreted as follows.

(1) Each allele has a single copy number value (e.g. the copy number can not be both 1 and 2).

(2) A discarded read can not be assigned to an allele; a non-discarded read can be assigned to only one allele (among its candidates $\mathbf{C}_i$).

(3) If at least one read is assigned to allele a_j, then *at least* one copy of that allele must be called. (This is implied by constraint (3) if n is large enough, maybe slightly redundant?)

(4) If $C_{c,j} = 1$ (allele a_j is called and has $c > 0$ copies), there must be *at least* c reads assigned to a_j, to ensure there is no allele copy with zero reads assigned.

(5) Each landmark position in $\mathbf{L}_{j,g}$ should have a minimum read coverage in proportion to the expected coverage for each copy of the allele a_j, provided allele a_j is called.

(6) If c copies of allele a_j are called, the deviation of read coverage of a group of landmark positions away from the estimated mean is bounded.

Objective Function

Minimize:

$$\sum_{r_i \in \mathbf{R}, a_j \in \mathbf{A}} D_i^j \cdot e(r_i, a_j) + \sum_{r_i \in \mathbf{R}} \Big(X_i \cdot expected\_errors(r_i) \cdot discard\_penalty\_multiplier \Big)$$

Here the first term represents the total edit distance between reads and their assigned alleles, and the second term is proportional to the sum of the expected sequencing errors across all discarded reads.

The output of this last step is a set of functional genes and their alleles that are present, as well as their copy number. (The pseudogenes and orphons in the extended IMGT database to which some reads are assigned are not considered any further.)

2 Supplemental Figures

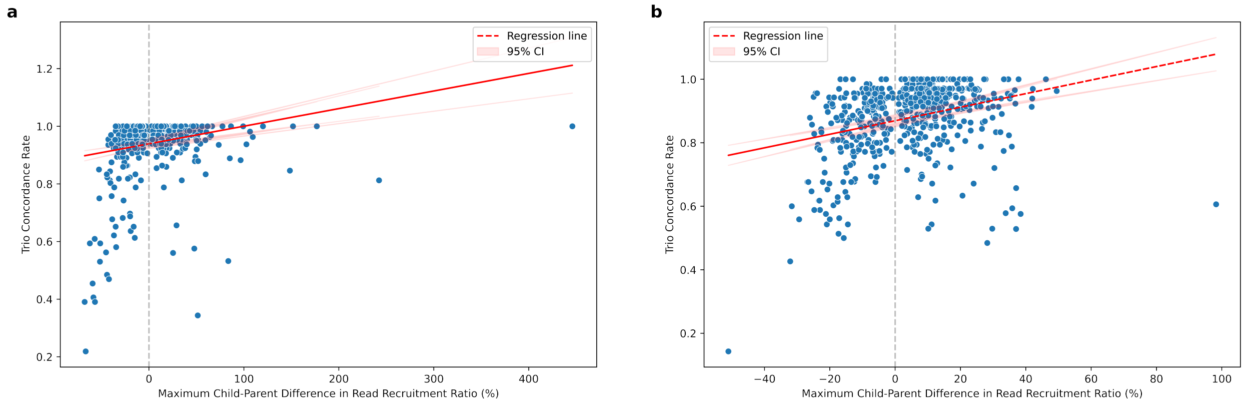

Figure 1: Correlation between trio concordance and maximum difference in IGLV (a) and IGKV (b) read recruitment, normalized depth between child and parent. Each point represents a trio, demonstrating the relationship between read dropout and inheritance pattern consistency. The regression line indicates that a lower read recruitment in any one parent results in a lower trio concordance.

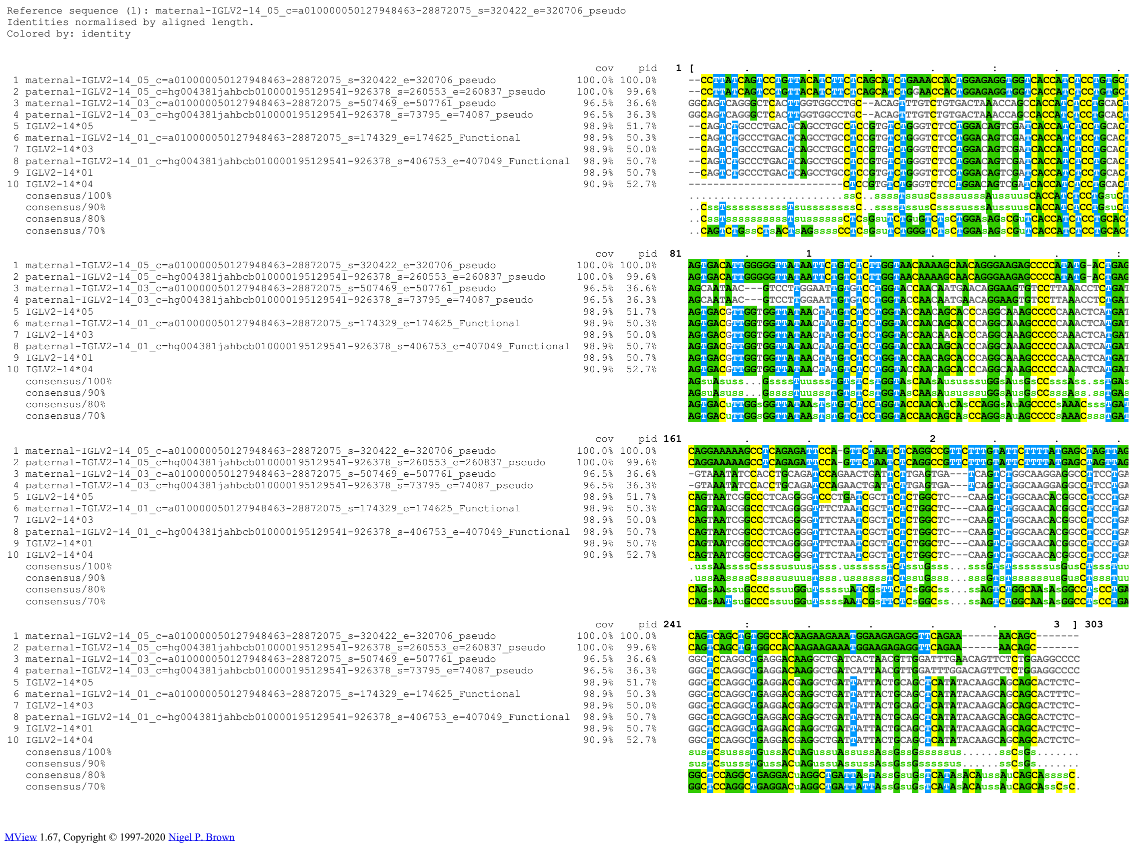

Figure 2: Multiple Sequence alignment of HPRC assembly sequences from sample HG00438 identified by Digger as copies of IGLV2-14, with their annotated allele id, including IMGT allele database reference sequences for *01, *03, *04, *05. The id fields for the assembly sequence correspond as follows: c= is the contig string, s=, e= are the start and end position respectively, and the suffix of the id string indicates whether Digger has labeled the sequence as functional, pseudogene or ORF. Note that 3/5 assembly sequences are labeled as pseudogene, and all sequences diverge significantly from their IMGT reference sequence genotype, which complicates both genoptype calling and benchmarking accuracy. Functional copies are listed as 2 copies of *01, while ImmunoTyper2 calls 1 copy of *01 and 1 copy of *04. Generated with Clustal Omega [3] and Mview [1] [2]

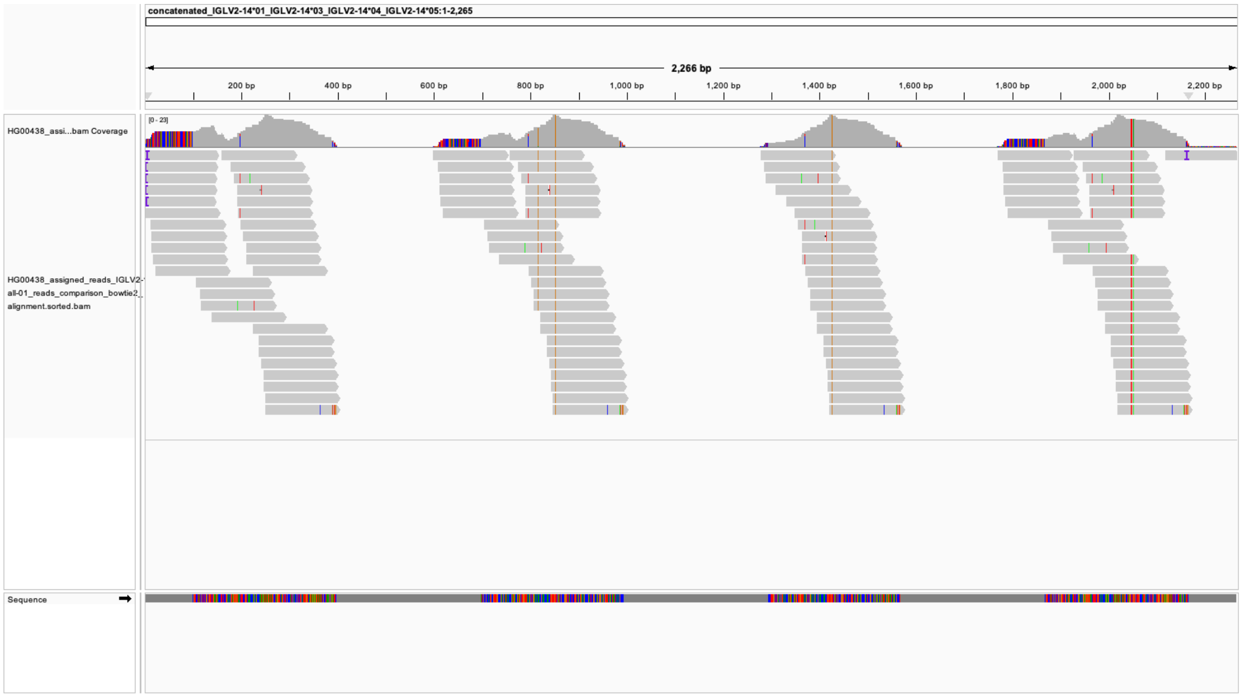

Figure 3: Multi mapping pileup of reads assigned to IGLV2-14*04, which is a false positive (corresponding false negative *01) from HPRC sample HG00438. Reference sequences composed of *01, *03, *04, *05, concatenated with 100bp Ns in between each sequence, aligned with `bowtie2 -a --end-to-end --very-sensitive --n-ceil C,100,0 --np 0 --ignore-quals --mp 2,2 --score-min C,-50,0 -L 10`, which corresponds to the command used in ImmunoTyper. Note the best alignment appears to be with *04, rather than the annotated ground truth value of *01. This could be due to other complicating pseudogene copies (see Supplementary Figure 2). See Supplementary Figure 4 for an example of alignment to a true positive instance of this gene in the same sample.

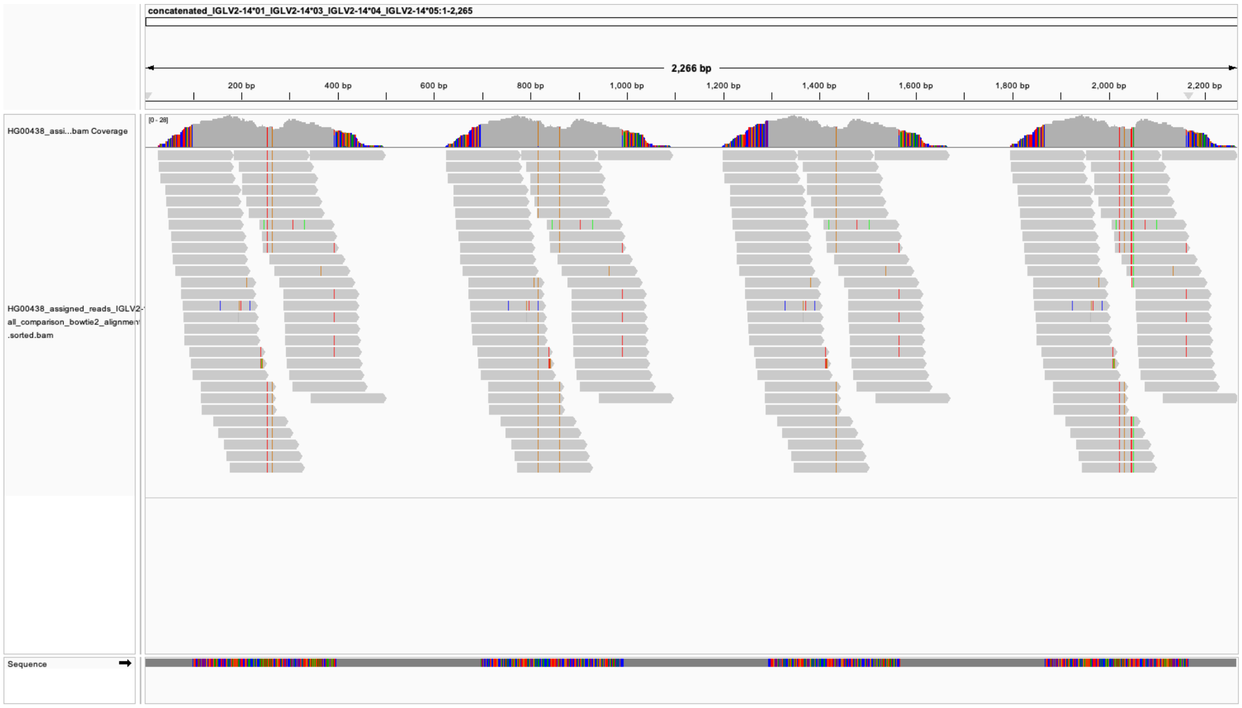

Figure 4: Multi mapping pileup of reads assigned to IGLV2-14*01, which is a true positive from HPRC sample HG00438. Reference sequences composed of *01, *03, *04, *05, concatenated with 100bp Ns in between each sequence, aligned with `bowtie2 -a --end-to-end --very-sensitive --n-ceil C,100,0 --np 0 --ignore-quals --mp 2,2 --score-min C,-50,0 -L 10`, which corresponds to the command used in ImmunoTyper. Note the best alignment corresponds to *01, despite the prefix noise found in alignments to all sequences. This is due to variants in the underlying genome (see Supplementary Figure 2) as well as the difficulty in prefix and suffix reads as they are clipped due to the ends of the reference sequence, and therefor have less nucleotides to align. See Supplementary Figure 3 for an example of alignment to a false positive instance of this gene in the same sample.

3 Supplemental Data

Group	Code	Description
African	YRI	Yoruba in Ibadan, Nigeria
	ESN	Esan in Nigeria
	GWD	Mandinka in The Gambia
	MSL	Mende in Sierra Leone
	ACB	African Caribbean in Barbados
	ASW	Americans of African Ancestry in SW USA
European	CEU	Utah Residents with Northern and Western European ancestry
	IBS	Iberian population in Spain
South Asian	PJL	Punjabi from Lahore, Pakistan
	BEB	Bengali from Bangladesh
	STU	Sri Lankan Tamil from the UK
	ITU	Indian Telugu from the UK
East Asian	CHS	Southern Han Chinese
	KHV	Kinh in Ho Chi Minh City, Vietnam
American	PUR	Puerto Rican from Puerto Rico
	CLM	Colombian from Medellin, Colombia
	PEL	Peruvian from Lima, Peru
	MXL	Mexican Ancestry from Los Angeles USA

Table 1: Population groups and codes used in the analysis of 1000 Genomes Trios.

Allele	Odds Ratio	Standard Error	P value	N	BH Corrected P value
TRAV29/DV5*01	2.4481	0.3510	0.0107	400	0.3175
TRAV1-1*02	0.5965	0.2159	0.0167	270	0.3175
TRAV12-2*02	1.7191	0.2283	0.0176	303	0.3175
TRAV13-1*02	1.9564	0.3147	0.0329	395	0.4247
IGKV1-8*02	0.5225	0.2591	0.0122	114	0.4188
IGKV1-5*04	0.5831	0.2388	0.0239	141	0.4188
IGKV1-9*03	0.6226	0.2235	0.0340	173	0.4188
IGHV3-66*02	1.7379	0.2282	0.0154	143	0.6313
IGHV1-46*01	0.5193	0.2803	0.0194	386	0.6313
IGHV4-28*02	0.4364	0.3866	0.0320	50	0.6313
IGHV5-51*01	1.6935	0.2478	0.0335	313	0.6313
IGHV3-7*03	0.5910	0.2546	0.0389	119	0.6313
IGHV3-66*03	0.6312	0.2255	0.0413	247	0.6313

Table 2: Combined analysis of TRAV, IGKV, and IGHV alleles on COVNET dataset which have potential impact on COVID-19 severity prior to Benjamini-Hochberg (BH) Multiple-Hypothesis Correction. N is the number of samples (out of a total 461 samples) with allele presence. Statistical significance was assessed through logistic regression where age, sex and the top three principal components for genome wide SNP variation were added as covariates. After BH Correction, none of the alleles appear to be statistically significant.

Table 3: Summary of Copy Number sensitive allele call accuracy on 1000 Genomes samples (IGLV: $n = 15$; TRAV: $n = 12$) using orthogonal long read assembly data as ground truth. Note that the "ground truth" is sourced from un-phased assembled contigs which may have noisy Copy Number estimates.

Gene type	Precision (mean)	Recall (mean)	Precision (median)	Recall (median)
IGLV	0.902	0.758	0.937	0.762
TRAV	0.883	0.875	0.874	0.882

Table 4: Difference in F1 Scores Between Default and Best-Performing Parameter Sets Across Gene Types. Default parameters are defined as 6 landmark groups (LG), 6 landmarks per group (LPG), and a standard deviation depth scaling factor (Stdev) of 1.5.

Gene Type	Default F1 Score	Best Parameters (LG, LPG, Stdev)	Best F1 Score	F1 Score Difference
IGHV	0.813	(4, 8, 2.0)	0.814	+0.001
TRDV	0.519	(4, 4, 2.0)	0.537	+0.018
IGLV	0.926	(8, 8, 2.0)	0.929	+0.003
TRGV	0.875	(4, 4, 2.0)	0.878	+0.003
TRAV	0.852	(4, 4, 2.0)	0.854	+0.002
TRBV	0.900	(4, 4, 2.0)	0.901	+0.001
IGKV	0.763	(4, 4, 2.0)	0.763	-0.000

References

[1] N. P. Brown, C. Leroy, and C. Sander. "MView: A Web compatible database search or multiple alignment viewer". In: *Bioinformatics* 14.4 (1998). [PubMed], pp. 380–381.

[2] Fábio Madeira et al. "The EMBL-EBI Job Dispatcher sequence analysis tools framework in 2024". In: *Nucleic acids research* 52.W1 (July 2024), W521–W525. ISSN: 0305-1048. DOI: 10.1093/nar/gkae241.

[3] Fabian Sievers et al. "Fast, scalable generation of high-quality protein multiple sequence alignments using Clustal Omega". In: *Molecular Systems Biology* 7 (Oct. 2011), p. 539. DOI: 10.1038/msb.2011.75.
