# Supplementary material for: Genotyping of selected germline adaptive immune system loci using short-read sequencing data
Source: Genome Res. 2025 Sep;35(9):2076–86. doi: 10.1101/gr.280314.124 (PMC12401057; doi:10.1101/gr.280314.124)
Supplement: Supplement 1 [file Supplemental_Code.zip › ImmunoTyper2-methods/HPRC-assembly-benchmarking/digger/tests/utr_motifs/IGH/test_seqs/IMGT000035.html]

IMGT/LIGM-DB


# WELCOME! to IMGT/LIGM-DB

##### THE INTERNATIONAL IMMUNOGENETICS INFORMATION SYSTEM�

Database contains
  
251473 sequences
  
from 361 species

# IMGT/LIGM-DB sequence

Version:
:   1.2.11

Database release:
:   LIGMDB\_V12

Citing IMGT/LIGM-DB:
:   Giudicelli, V., Duroux P., Ginestoux C., Folch G., Jabado-Michaloud J., Chaume D., Lefranc M.-P., Nucleic Acids Res., 34:D781-D784 (2006).
    PMID:16381979
    Abstract
    Full

|  |  |
| --- | --- |
| Views | - Annotation - IMGT - EMBL - FASTA - Translation - Dump - IMGT/BlastSearch - IMGT/V-QUEST |

## BK063799 - IMGT


```
ID   BK063799; SV 1; linear; gDNA; STD; HUM; 1293408 BP.
XX
AC   BK063799; IMGT000035;
XX
DT   06-SEP-2023 (Rel. 202336-3, arrived in LIGM-DB)
DT   05-DEC-2023 (Rel. 202349-2, Last updated, Version 7)
XX
DE   TPA: Homo sapiens IGH locus.
XX
KW   antigen receptor; Immunoglobulin superfamily (IgSF); immunoglobulin (IG);
KW   IG-Heavy; IG-Heavy-Mu; IG-Heavy-Delta; IG-Heavy-Alpha; IG-Heavy-Epsilon;
KW   constant; IG-Heavy-Alpha-1-IGHA1; IG-Heavy-Alpha-2-IGHA2;
KW   IG-Heavy-Gamma-3-IGHG3; variable; diversity; joining; pseudogene;
KW   IG-Heavy-Gamma-1-IGHG1; IG-Heavy-Gamma-2-IGHG2; IMGT reference sequence;
KW   regular; gDNA; germline; undefined; functional; ORF; V-gene; D-gene;
KW   J-gene; C-gene.
XX
OS   Homo sapiens (human)
OC   cellular organisms; Eukaryota; Opisthokonta; Metazoa; Eumetazoa; Bilateria;
OC   Deuterostomia; Chordata; Craniata; Vertebrata; Gnathostomata; Teleostomi;
OC   Euteleostomi; Sarcopterygii; Dipnotetrapodomorpha; Tetrapoda; Amniota;
OC   Mammalia; Theria; Eutheria; Boreoeutheria; Euarchontoglires; Primates;
OC   Haplorrhini; Simiiformes; Catarrhini; Hominoidea; Hominidae; Homininae;
OC   Homo; Homo sapiens.
XX
RN   [1]
RP   1-1293408
RX   PUBMED; 34875068.
RA   Manso,T., Folch,G., Giudicelli,V., Jabado-Michaloud,J., Kushwaha,A.,
RA   Nguefack Ngoune,V., Georga,M., Papadaki,A., Debbagh,C., Pegorier,P.,
RA   Bertignac,M., Hadi-Saljoqi,S., Chentli,I., Cherouali,K., Aouinti,S.,
RA   El Hamwi,A., Albani,A., Elazami Elhassani,M., Viart,B., Goret,A., Tran,A.,
RA   Sanou,G., Rollin,M., Duroux,P., Kossida,S.;
RT   "IMGT(R) databases, related tools and web resources through three main axes
RT   of research and development";
RL   Nucleic Acids Res 50(D1):D1262-D1272(2022).
XX
RN   [2]
RP   1-1293408
RA   Albani A., Maria G., Papadaki A., Folch G., Jabado-Michaloud J.,
RA   Giudicelli V., Kossida S.;
RT   ;
RL   Submitted (12-SEP-2023) to the INSDC.
RL   IMGT, the international ImMunoGeneTics information system, Institute of
RL   Human Genetics, IGH, CNRS, University of Montpellier, 141 rue de la
RL   Cardonille, Montpellier 34396, France
XX
DR   GenBank; BK063799.
XX
CC   IMGT/LIGM-DB annotation level: by annotators
CC   
CC   
CC   THIRD PARTY DATABASE: This TPA record uses data from
CC   DDBJ/EMBL/GenBank entries AL928742.3, AL928761.2, AL928769.1,
CC   AL928765.1, AL928767.1, AL928762.2, AL901608.1, AL928768.1,
CC   AL122127.6, AC244452.3, AC247036.3, AC245023.2, AC245166.2,
CC   AC244226.3, AC246787.2, AC245369.4 and ABBA01040607.1.
XX
FH   Key                 Location/Qualifiers
FH   
FT   V-D-J-C-CLUSTER     1..1293408
FT                       /chromosome="14"
FT                       /db_xref="taxon:9606"
FT                       /mol_type="genomic DNA"
FT                       /organism="Homo sapiens"
FT   IMGT-LOCUS-UNIT     1..1293408
FT                       /IMGT_locus_3prime_gene="IGHA2"
FT                       /IMGT_locus_5prime_gene="IGHV(III)-82"
FT                       /IMGT_locus_length="1293 kb"
FT                       /IMGT_locus_name="Homo sapiens, IGH"
FT                       /IMGT_locus_orientation="reverse (REV)"
FT                       /IMGT_locus_positions="chromosome 14: 
FT                       105586437-106879844 in genome assembly GRCh38.p12"
FT                       /IMGT_locus_type="IGH"
FT   V-GENE              1..2579
FT                       /IMGT_allele="IGHV(III)-82*01"
FT                       /IMGT_gene="IGHV(III)-82"
FT                       /pseudo="frameshifts in V-REGION: several insertions 
FT                       and deletions"
FT   V-REGION            <11..292
FT                       /partial
FT                       /CDR_length="[8.X.2]"
FT                       /FR_length="[X.17.30.G]"
FT                       /IMGT_allele="IGHV(III)-82*01"
FT                       /IMGT_gene="IGHV(III)-82"
FT                       /IMGT_note="partial gene in 5', restored translation 
FT                       in CDR1-IMGT, FR2-IMGT, CDR2-IMGT"
FT                       /out_of_frame
FT                       /pseudo="frameshifts in V-REGION: several insertions 
FT                       and deletions"
FT   FR1-IMGT            <11..86
FT                       /partial
FT                       /IMGT_note="frameshifts in FR1-IMGT: deletion of 1 nt 
FT                       and insertion of 2 nt"
FT                       /out_of_frame
FT   CDR1-IMGT           87..110
FT                       /translation="DFTCTGYS"
FT                       /AA_IMGT="AA 27 to 38, AA 31, 32, 33 and 34 are 
FT                       missing"
FT                       /out_of_frame
FT                       /restored_translation
FT   FR2-IMGT            111..161
FT                       /AA_IMGT="AA 39 to 55"
FT                       /out_of_frame
FT                       /restored_translation
FT   CDR2-IMGT           162..196
FT                       /IMGT_note="frameshift in CDR2-IMGT: insertion of 11 
FT                       nt"
FT                       /out_of_frame
FT                       /restored_translation
FT   FR3-IMGT            197..286
FT                       /translation="KLTVPSNTLSSAVYLKMTNVKAKDKTLYYC"
FT                       /IMGT_note="deletions in FR3-IMGT: 3 AA are missing, 
FT                       deletions in FR3-IMGT: 5 AA are missing"
FT                       /in_frame
FT   3'V-REGION          284..292
FT                       /in_frame
FT   2nd-CYS             284..286
FT   CDR3-IMGT           287..292
FT                       /AA_IMGT="AA 105 to 106"
FT                       /IMGT_note="STOP-CODON in CDR3-IMGT: position 106"
FT                       /in_frame
FT   3'UTR               293..2579
FT   V-RS                293..332
FT   V-HEPTAMER          293..299
FT                       /IMGT_note="noncanonical V-HEPTAMER: catagga instead 
FT                       of cacagtg"
FT   V-SPACER            300..323
FT   V-NONAMER           324..332
FT   V-GENE              2580..5981
FT                       /IMGT_allele="IGHV7-81*01"
FT                       /IMGT_gene="IGHV7-81"
FT                       /ORF="noncanonical V-HEPTAMER: caccatg instead of 
FT                       cacagtg, noncanonical V-NONAMER: tcagaaatc instead of 
FT                       acacaaacc"
FT   5'UTR               2580..4827
FT   L-V-GENE-UNIT       4828..5303
FT                       /IMGT_allele="IGHV7-81*01"
FT                       /IMGT_gene="IGHV7-81"
FT                       /ORF="noncanonical V-HEPTAMER: caccatg instead of 
FT                       cacagtg, noncanonical V-NONAMER: tcagaaatc instead of 
FT                       acacaaacc"
FT   L-INTRON-L          4828..4968
FT   L-PART1             4828..4873
FT                       /translation="MDWTWSILFLVAAAT"
FT   INIT-CODON          4828..4830
FT   DONOR-SPLICE        4873..4875
FT   V-INTRON            4874..4957
FT   ACCEPTOR-SPLICE     4955..4959
FT   V-EXON              4958..5264
FT                       /codon_start=3
FT                       /translation="TYSQVQLVQSGHEVKQPGASVKVSCKASGYSFTTYGMNWVP
FT                       QAPGQGLEWMGWFNTYTGNPTYAQGFTGRFVFSMDTSASTAYLQISSLKAEDMAM
FT                       YYCAR"
FT   L-PART2             4958..4968
FT                       /codon_start=3
FT                       /translation="TYS"
FT   V-REGION            4969..5264
FT                       /translation="QVQLVQSGHEVKQPGASVKVSCKASGYSFTTYGMNWVPQAP
FT                       GQGLEWMGWFNTYTGNPTYAQGFTGRFVFSMDTSASTAYLQISSLKAEDMAMYYC
FT                       AR"
FT                       /CDR_length="[8.8.2]"
FT                       /FR_length="[25.17.38.G]"
FT                       /IMGT_allele="IGHV7-81*01"
FT                       /IMGT_gene="IGHV7-81"
FT                       /ORF="noncanonical V-HEPTAMER: caccatg instead of 
FT                       cacagtg, noncanonical V-NONAMER: tcagaaatc instead of 
FT                       acacaaacc"
FT   FR1-IMGT            4969..5043
FT                       /translation="QVQLVQSGHEVKQPGASVKVSCKAS"
FT                       /AA_IMGT="AA 1 to 26, AA 10 is missing"
FT   1st-CYS             5032..5034
FT   CDR1-IMGT           5044..5067
FT                       /translation="GYSFTTYG"
FT                       /AA_IMGT="AA 27 to 38, AA 31, 32, 33 and 34 are 
FT                       missing"
FT   FR2-IMGT            5068..5118
FT                       /translation="MNWVPQAPGQGLEWMGW"
FT                       /AA_IMGT="AA 39 to 55"
FT   CONSERVED-TRP       5074..5076
FT   CDR2-IMGT           5119..5142
FT                       /translation="FNTYTGNP"
FT                       /AA_IMGT="AA 56 to 65, AA 60 and 61 are missing"
FT   FR3-IMGT            5143..5256
FT                       /translation="TYAQGFTGRFVFSMDTSASTAYLQISSLKAEDMAMYYC"
FT                       /AA_IMGT="AA 66 to 104, AA 73 is missing"
FT   3'V-REGION          5254..5264
FT                       /translation="CAR"
FT   2nd-CYS             5254..5256
FT   CDR3-IMGT           5257..5264
FT                       /translation="AR"
FT                       /AA_IMGT="AA 105 to 106"
FT   3'UTR               5265..5981
FT   V-RS                5265..5303
FT   V-HEPTAMER          5265..5271
FT                       /IMGT_note="noncanonical V-HEPTAMER: caccatg instead 
FT                       of cacagtg"
FT   V-SPACER            5272..5294
FT   V-NONAMER           5295..5303
FT                       /IMGT_note="noncanonical V-NONAMER: tcagaaatc instead 
FT                       of acacaaacc"
FT   V-GENE              5982..9411
FT                       /IMGT_allele="IGHV4-80*02"
FT                       /IMGT_gene="IGHV4-80"
FT                       /pseudo="STOP-CODON in V-REGION: position 75"
FT   5'UTR               5982..6658
FT   L-INTRON-L          6659..6798
FT   L-PART1             6659..6704
FT                       /translation="MKNLWFFLPLVAAPR"
FT   INIT-CODON          6659..6661
FT   DONOR-SPLICE        6704..6706
FT   V-INTRON            6705..6787
FT   ACCEPTOR-SPLICE     6785..6789
FT   V-EXON              6788..7062
FT                       /codon_start=3
FT   L-PART2             6788..6798
FT                       /codon_start=3
FT                       /translation="VLS"
FT   V-REGION            6799..>7062
FT                       /partial
FT                       /CDR_length="[9.7.X]"
FT                       /FR_length="[25.17.30.G]"
FT                       /IMGT_allele="IGHV4-80*02"
FT                       /IMGT_gene="IGHV4-80"
FT                       /IMGT_note="partial gene in 3'"
FT                       /pseudo="STOP-CODON in V-REGION: position 75"
FT   FR1-IMGT            6799..6873
FT                       /translation="QVQLQEWGPGLVKPSETLSLTCAVS"
FT                       /AA_IMGT="AA 1 to 26, AA 10 is missing"
FT   1st-CYS             6862..6864
FT   CDR1-IMGT           6874..6900
FT                       /translation="GDSISSGNW"
FT                       /AA_IMGT="AA 27 to 38, AA 32, 33 and 34 are missing"
FT   FR2-IMGT            6901..6951
FT                       /translation="WSWVRQSPGKGLEWIGY"
FT                       /AA_IMGT="AA 39 to 55"
FT   CONSERVED-TRP       6907..6909
FT   CDR2-IMGT           6952..6972
FT                       /translation="IYYSGRS"
FT                       /AA_IMGT="AA 56 to 65, AA 60, 61 and 62 are missing"
FT   FR3-IMGT            6973..7062
FT                       /IMGT_note="STOP-CODON in FR3-IMGT: position 75"
FT   3'UTR               7063..9411
FT   V-GENE              9412..13092
FT                       /IMGT_allele="IGHV3-79*02"
FT                       /IMGT_gene="IGHV3-79"
FT                       /pseudo="frameshift in L-PART1: deletion of 1 nt 
FT                       causing defectuous DONOR-SPLICE, frameshift in 
FT                       V-REGION: deletion of 1 nt in FR1-IMGT"
FT   5'UTR               9412..11752
FT   L-V-GENE-UNIT       11753..12235
FT                       /IMGT_allele="IGHV3-79*02"
FT                       /IMGT_gene="IGHV3-79"
FT                       /pseudo="frameshift in L-PART1: deletion of 1 nt 
FT                       causing defectuous DONOR-SPLICE, frameshift in 
FT                       V-REGION: deletion of 1 nt in FR1-IMGT"
FT   L-INTRON-L          11753..11901
FT   L-PART1             11753..11797
FT                       /IMGT_note="frameshift in L-PART1: deletion of 1 nt 
FT                       causing defectuous DONOR-SPLICE"
FT                       /out_of_frame
FT   INIT-CODON          11753..11755
FT   DONOR-SPLICE        11797..11799
FT   V-INTRON            11798..11890
FT   ACCEPTOR-SPLICE     11888..11892
FT                       /IMGT_note="noncanonical ACCEPTOR-SPLICE: nggnn 
FT                       instead of nagnn"
FT   V-EXON              11891..12197
FT                       /codon_start=3
FT                       /out_of_frame
FT   L-PART2             11891..11901
FT                       /codon_start=3
FT                       /translation="VQC"
FT   V-REGION            11902..12197
FT                       /CDR_length="[8.8.3]"
FT                       /FR_length="[X.17.38.G]"
FT                       /IMGT_allele="IGHV3-79*02"
FT                       /IMGT_gene="IGHV3-79"
FT                       /IMGT_note="restored translation in CDR1-IMGT, 
FT                       FR2-IMGT, CDR2-IMGT, FR3-IMGT, 3'V-REGION and 
FT                       CDR3-IMGT"
FT                       /out_of_frame
FT                       /pseudo="frameshift in L-PART1: deletion of 1 nt 
FT                       causing defectuous DONOR-SPLICE, frameshift in 
FT                       V-REGION: deletion of 1 nt in FR1-IMGT"
FT   FR1-IMGT            11902..11975
FT                       /IMGT_note="frameshift in FR1-IMGT: deletion of 1 nt"
FT                       /out_of_frame
FT   CDR1-IMGT           11976..11999
FT                       /AA_IMGT="AA 27 to 38, AA 31, 32, 33 and 34 are 
FT                       missing"
FT                       /out_of_frame
FT                       /restored_translation
FT   FR2-IMGT            12000..12050
FT                       /AA_IMGT="AA 39 to 55"
FT                       /out_of_frame
FT                       /restored_translation
FT   CDR2-IMGT           12051..12074
FT                       /translation="IQCDGSQI"
FT                       /AA_IMGT="AA 56 to 65, AA 60 and 61 are missing"
FT                       /out_of_frame
FT                       /restored_translation
FT   FR3-IMGT            12075..12188
FT                       /translation="CYAQSVTSRFTVSKENAKNSLYLQMNSLRAEGTAVCYC"
FT                       /AA_IMGT="AA 66 to 104, AA 73 is missing"
FT                       /out_of_frame
FT                       /restored_translation
FT   3'V-REGION          12186..12197
FT                       /out_of_frame
FT                       /restored_translation
FT   CDR3-IMGT           12189..12197
FT                       /AA_IMGT="AA 105 to 107"
FT                       /out_of_frame
FT                       /restored_translation
FT   3'UTR               12198..13092
FT   V-RS                12198..12235
FT   V-HEPTAMER          12198..12204
FT                       /IMGT_note="noncanonical V-HEPTAMER: accaggt instead 
FT                       of cacagtg"
FT   V-SPACER            12205..12226
FT   V-NONAMER           12227..12235
FT                       /IMGT_note="noncanonical V-NONAMER: agacacaga instead 
FT                       of acacaaacc"
FT   V-GENE              13093..21500
FT                       /IMGT_allele="IGHV(II)-78-1*02"
FT                       /IMGT_gene="IGHV(II)-78-1"
FT                       /pseudo="truncated pseudogene not assigned to 
FT                       subgroups with functional genes"
FT   5'UTR               13093..13949
FT   V-REGION            <13950..14224
FT                       /partial
FT                       /codon_start=2
FT                       /CDR_length="[X.7.4]"
FT                       /FR_length="[X.X.37.G]"
FT                       /IMGT_allele="IGHV(II)-78-1*02"
FT                       /IMGT_gene="IGHV(II)-78-1"
FT                       /IMGT_note="partial gene in 5'"
FT                       /pseudo="truncated pseudogene not assigned to 
FT                       subgroups with functional genes"
FT                       /putative_limit="5'"
FT   FR1-IMGT            <13950..13998
FT                       /partial
FT                       /codon_start=2
FT                       /translation="GLVKPSQTLSLTCAVS"
FT                       /putative_limit="5'"
FT   1st-CYS             13987..13989
FT   FR2-IMGT            14029..14080
FT                       /IMGT_note="STOP-CODON in FR2-IMGT: position 39"
FT   CONSERVED-TRP       14035..14037
FT   CDR2-IMGT           14081..14101
FT                       /translation="IGHEGST"
FT                       /AA_IMGT="AA 56 to 65, AA 60, 61 and 62 are missing"
FT   FR3-IMGT            14102..14212
FT                       /translation="HYHPLLKCPVTIPRSVSKKAVLPTAELHEQQAHSHVF"
FT                       /AA_IMGT="AA 66 to 104, AA 73 and 82 is missing"
FT                       /IMGT_note="no 2nd-CYS: Phe instead of Cys"
FT   3'V-REGION          14210..14224
FT                       /translation="FLSQR"
FT   CDR3-IMGT           14213..14224
FT                       /translation="LSQR"
FT                       /AA_IMGT="AA 105 to 108"
FT   3'UTR               14225..21500
FT   V-RS                14225..14263
FT   V-HEPTAMER          14225..14231
FT   V-SPACER            14232..14254
FT   V-NONAMER           14255..14263
FT   V-GENE              21501..38316
FT                       /IMGT_allele="IGHV5-78*01"
FT                       /IMGT_gene="IGHV5-78"
FT                       /pseudo="no V-RS"
FT   5'UTR               21501..28298
FT   L-INTRON-L          28299..28438
FT   L-PART1             28299..28344
FT                       /translation="MGSTVILSLVLAVLQ"
FT   INIT-CODON          28299..28301
FT   DONOR-SPLICE        28344..28346
FT   V-INTRON            28345..28427
FT   ACCEPTOR-SPLICE     28425..28429
FT   V-EXON              28428..28732
FT                       /codon_start=3
FT                       /translation="VFAEVQLLQSAAEVKRPGESLRISCKTSGYSFTSYWIHWVR
FT                       QMPGKELEWMGSIYPGNSDTRYSPSFQGHVTISADSSSSTAYLQWSSLKASDAAM
FT                       YYCVR"
FT                       /putative_limit="3'"
FT   L-PART2             28428..28438
FT                       /codon_start=3
FT                       /translation="VFA"
FT   V-REGION            28439..28732
FT                       /translation="EVQLLQSAAEVKRPGESLRISCKTSGYSFTSYWIHWVRQMP
FT                       GKELEWMGSIYPGNSDTRYSPSFQGHVTISADSSSSTAYLQWSSLKASDAAMYYC
FT                       VR"
FT                       /CDR_length="[8.8.2]"
FT                       /FR_length="[25.17.38.G]"
FT                       /IMGT_allele="IGHV5-78*01"
FT                       /IMGT_gene="IGHV5-78"
FT                       /pseudo="no V-RS"
FT                       /putative_limit="3'"
FT   FR1-IMGT            28439..28513
FT                       /translation="EVQLLQSAAEVKRPGESLRISCKTS"
FT                       /AA_IMGT="AA 1 to 26, AA 10 is missing"
FT   1st-CYS             28502..28504
FT   CDR1-IMGT           28514..28537
FT                       /translation="GYSFTSYW"
FT                       /AA_IMGT="AA 27 to 38, AA 31, 32, 33 and 34 are 
FT                       missing"
FT   FR2-IMGT            28538..28588
FT                       /translation="IHWVRQMPGKELEWMGS"
FT                       /AA_IMGT="AA 39 to 55"
FT   CONSERVED-TRP       28544..28546
FT   CDR2-IMGT           28589..28612
FT                       /translation="IYPGNSDT"
FT                       /AA_IMGT="AA 56 to 65, AA 60 and 61 are missing"
FT   FR3-IMGT            28613..28726
FT                       /translation="RYSPSFQGHVTISADSSSSTAYLQWSSLKASDAAMYYC"
FT                       /AA_IMGT="AA 66 to 104, AA 73 is missing"
FT   3'V-REGION          28724..28732
FT                       /translation="CVR"
FT                       /putative_limit="3'"
FT   2nd-CYS             28724..28726
FT   CDR3-IMGT           28727..28732
FT                       /translation="VR"
FT                       /AA_IMGT="AA 105 to 106"
FT                       /putative_limit="3'"
FT   3'UTR               28733..38316
FT   V-GENE              38317..49846
FT                       /IMGT_allele="IGHV(III)-76-1*01"
FT                       /IMGT_gene="IGHV(III)-76-1"
FT                       /pseudo="truncated pseudogene not assigned to 
FT                       subgroups with functional genes"
FT   5'UTR               38317..47794
FT   V-REGION            <47795..48082
FT                       /partial
FT                       /CDR_length="[8.7.2]"
FT                       /FR_length="[22.17.40.G]"
FT                       /IMGT_allele="IGHV(III)-76-1*01"
FT                       /IMGT_gene="IGHV(III)-76-1"
FT                       /IMGT_note="partial gene in 5'"
FT                       /pseudo="truncated pseudogene not assigned to 
FT                       subgroups with functional genes"
FT                       /putative_limit="5'"
FT   FR1-IMGT            <47795..47860
FT                       /partial
FT                       /translation="LVESGREMIQPNCSQSLFYKAS"
FT                       /IMGT_note="no 1st-CYS: Tyr instead of Cys"
FT                       /putative_limit="5'"
FT   CDR1-IMGT           47861..47884
FT                       /translation="EFTFIDSS"
FT                       /AA_IMGT="AA 27 to 38, AA 31, 32, 33 and 34 are 
FT                       missing"
FT   FR2-IMGT            47885..47935
FT                       /translation="MSSAQQASGQGLVWEAK"
FT                       /AA_IMGT="AA 39 to 55"
FT                       /IMGT_note="no CONSERVED-TRP: Ser instead of Trp"
FT   CDR2-IMGT           47936..47956
FT                       /translation="ARDSSGI"
FT                       /AA_IMGT="AA 56 to 65, AA 60, 61 and 62 are missing"
FT   FR3-IMGT            47957..48076
FT                       /IMGT_note="STOP-CODON in FR3-IMGT: position 67"
FT   3'V-REGION          48074..48082
FT                       /translation="CER"
FT   2nd-CYS             48074..48076
FT   CDR3-IMGT           48077..48082
FT                       /translation="ER"
FT                       /AA_IMGT="AA 105 to 106"
FT   3'UTR               48083..49846
FT   V-RS                48083..48089
FT                       /IMGT_note="partial V-RS: no V-NONAMER"
FT   V-HEPTAMER          48083..48089
FT                       /IMGT_note="noncanonical V-HEPTAMER: cacagga instead 
FT                       of cacagtg"
FT   V-GENE              49847..53861
FT                       /IMGT_allele="IGHV3-76*01"
FT                       /IMGT_gene="IGHV3-76"
FT                       /pseudo="several STOP-CODON in V-REGION, no 
FT                       CONSERVED-TRP: STOP-CODON instead of Trp, frameshift 
FT                       in V-REGION: deletion of 2 nt in FR2-IMGT"
FT   5'UTR               49847..51534
FT   L-V-GENE-UNIT       51535..52023
FT                       /IMGT_allele="IGHV3-76*01"
FT                       /IMGT_gene="IGHV3-76"
FT                       /pseudo="several STOP-CODON in V-REGION, no 
FT                       CONSERVED-TRP: STOP-CODON instead of Trp, frameshift 
FT                       in V-REGION: deletion of 2 nt in FR2-IMGT"
FT   L-INTRON-L          51535..51692
FT   L-PART1             51535..51580
FT                       /translation="MELGMRWVFFVVVLK"
FT   INIT-CODON          51535..51537
FT   DONOR-SPLICE        51580..51582
FT                       /IMGT_note="noncanonical DONOR-SPLICE: ngc instead of 
FT                       ngt"
FT   V-INTRON            51581..51681
FT   ACCEPTOR-SPLICE     51679..51683
FT   V-EXON              51682..51984
FT                       /codon_start=3
FT                       /out_of_frame
FT   L-PART2             51682..51692
FT                       /codon_start=3
FT                       /translation="VQC"
FT   V-REGION            51693..51984
FT                       /CDR_length="[8.X.2]"
FT                       /FR_length="[25.X.38.G]"
FT                       /IMGT_allele="IGHV3-76*01"
FT                       /IMGT_gene="IGHV3-76"
FT                       /IMGT_note="restored translation in CDR2-IMGT, 
FT                       FR3-IMGT, 3'V-REGION and CDR3-IMGT"
FT                       /out_of_frame
FT                       /pseudo="several STOP-CODON in V-REGION, no 
FT                       CONSERVED-TRP: STOP-CODON instead of Trp, frameshift 
FT                       in V-REGION: deletion of 2 nt in FR2-IMGT"
FT   FR1-IMGT            51693..51767
FT                       /AA_IMGT="AA 1 to 26, AA 10 is missing"
FT                       /IMGT_note="several STOP-CODON in FR1-IMGT: pos 3 and 
FT                       20"
FT   1st-CYS             51756..51758
FT   CDR1-IMGT           51768..51791
FT                       /translation="GYTFSNYG"
FT                       /AA_IMGT="AA 27 to 38, AA 31, 32, 33 and 34 are 
FT                       missing"
FT   FR2-IMGT            51792..51842
FT                       /AA_IMGT="AA 39 to 55"
FT                       /IMGT_note="frameshift in FR2-IMGT: deletion of 2 nt"
FT                       /out_of_frame
FT   CDR2-IMGT           51843..51864
FT                       /out_of_frame
FT                       /restored_translation
FT   FR3-IMGT            51865..51978
FT                       /translation="YYSDSVKGRLTISRENTKNSLYLQINSFIADTMAVYYC"
FT                       /AA_IMGT="AA 66 to 104, AA 73 is missing"
FT                       /out_of_frame
FT                       /restored_translation
FT   3'V-REGION          51976..51984
FT                       /translation="CKR"
FT                       /out_of_frame
FT                       /restored_translation
FT   CDR3-IMGT           51979..51984
FT                       /translation="KR"
FT                       /AA_IMGT="AA 105 to 106"
FT                       /out_of_frame
FT                       /restored_translation
FT   3'UTR               51985..53861
FT   V-RS                51985..52023
FT   V-HEPTAMER          51985..51991
FT   V-SPACER            51992..52014
FT   V-NONAMER           52015..52023
FT   V-GENE              53862..57271
FT                       /IMGT_allele="IGHV3-75*01"
FT                       /IMGT_gene="IGHV3-75"
FT                       /pseudo="no DONOR-SPLICE, frameshifts in V-REGION: 
FT                       several insertions and deletions"
FT   5'UTR               53862..55697
FT   L-V-GENE-UNIT       55698..56206
FT                       /IMGT_allele="IGHV3-75*01"
FT                       /IMGT_gene="IGHV3-75"
FT                       /pseudo="no DONOR-SPLICE, frameshifts in V-REGION: 
FT                       several insertions and deletions"
FT   L-INTRON-L          55698..55857
FT   L-PART1             55698..55743
FT                       /translation="MGFVLTWDLLVTILK"
FT                       /IMGT_note="no DONOR-SPLICE"
FT   INIT-CODON          55698..55700
FT   V-INTRON            55744..55846
FT   ACCEPTOR-SPLICE     55844..55848
FT                       /IMGT_note="noncanonical ACCEPTOR-SPLICE: nggnn 
FT                       instead of nagnn"
FT   V-EXON              55847..56167
FT                       /codon_start=3
FT                       /out_of_frame
FT   L-PART2             55847..55857
FT                       /codon_start=3
FT                       /translation="VQC"
FT   V-REGION            55858..56167
FT                       /CDR_length="[8.8.2]"
FT                       /FR_length="[X.17.X.G]"
FT                       /IMGT_allele="IGHV3-75*01"
FT                       /IMGT_gene="IGHV3-75"
FT                       /IMGT_note="restored translation in CDR1-IMGT, 
FT                       FR2-IMGT, CDR2-IMGT, FR3-IMGT, 3'V-REGION and 
FT                       CDR3-IMGT"
FT                       /out_of_frame
FT                       /pseudo="no DONOR-SPLICE, frameshifts in V-REGION: 
FT                       several insertions and deletions"
FT   FR1-IMGT            55858..55945
FT                       /translation="EAQLLEPGGDLVQPGAVGVGVPETVLCSL"
FT                       /IMGT_note="frameshift in FR1-IMGT: insertion of 13 nt"
FT                       /out_of_frame
FT   CDR1-IMGT           55946..55969
FT                       /translation="GFAFSSHW"
FT                       /AA_IMGT="AA 27 to 38, AA 31, 32, 33 and 34 are 
FT                       missing"
FT                       /out_of_frame
FT                       /restored_translation
FT   FR2-IMGT            55970..56020
FT                       /translation="MHWACQAPGNRLEWVSC"
FT                       /AA_IMGT="AA 39 to 55"
FT                       /out_of_frame
FT                       /restored_translation
FT   CDR2-IMGT           56021..56044
FT                       /translation="ISTDGSSI"
FT                       /AA_IMGT="AA 56 to 65, AA 60 and 61 are missing"
FT                       /out_of_frame
FT                       /restored_translation
FT   FR3-IMGT            56045..56159
FT                       /IMGT_note="frameshift in FR3-IMGT: insertion of 1 nt"
FT                       /out_of_frame
FT                       /restored_translation
FT   3'V-REGION          56157..56167
FT                       /translation="CAG"
FT                       /out_of_frame
FT                       /restored_translation
FT   CDR3-IMGT           56160..56167
FT                       /translation="AG"
FT                       /AA_IMGT="AA 105 to 106"
FT                       /out_of_frame
FT                       /restored_translation
FT   3'UTR               56168..57271
FT   V-RS                56168..56206
FT   V-HEPTAMER          56168..56174
FT   V-SPACER            56175..56197
FT   V-NONAMER           56198..56206
FT   V-GENE              57272..64000
FT                       /IMGT_allele="IGHV(II)-74-1*01"
FT                       /IMGT_gene="IGHV(II)-74-1"
FT                       /pseudo="truncated pseudogene not assigned to 
FT                       subgroups with functional genes"
FT   5'UTR               57272..58434
FT   ACCEPTOR-SPLICE     58432..58436
FT   V-EXON              58435..58628
FT                       /codon_start=3
FT                       /out_of_frame
FT                       /putative_limit="3'"
FT   L-PART2             58435..58445
FT                       /codon_start=3
FT                       /translation="ALS"
FT   V-REGION            58446..>58628
FT                       /partial
FT                       /CDR_length="[10.7.2]"
FT                       /FR_length="[X.16.37.G]"
FT                       /IMGT_allele="IGHV(II)-74-1*01"
FT                       /IMGT_gene="IGHV(II)-74-1"
FT                       /IMGT_note="partial gene in 3', restored translation 
FT                       in CDR1-IMGT, FR2-IMGT, CDR2-IMGT, FR3-IMGT, 
FT                       3'V-REGION and CDR3-IMGT"
FT                       /out_of_frame
FT                       /pseudo="truncated pseudogene not assigned to 
FT                       subgroups with functional genes"
FT                       /putative_limit="3'"
FT   FR1-IMGT            58446..58519
FT                       /IMGT_note="frameshift in FR1-IMGT: deletion of 1 nt"
FT                       /out_of_frame
FT   CDR1-IMGT           58520..58549
FT                       /translation="GHYITNSAYD"
FT                       /AA_IMGT="AA 27 to 38, AA 32 and 33 are missing"
FT                       /out_of_frame
FT                       /restored_translation
FT   FR2-IMGT            58550..58597
FT                       /IMGT_note="deletions in FR2-IMGT: 1 AA is missing"
FT                       /out_of_frame
FT                       /restored_translation
FT   CDR2-IMGT           58598..58618
FT                       /translation="IGSDGGG"
FT                       /AA_IMGT="AA 56 to 65, AA 60, 61 and 62 are missing"
FT                       /out_of_frame
FT                       /restored_translation
FT   REPEAT_UNIT         58629..58737
FT                       /rpt_family="LINE1"
FT   3'UTR               58738..64000
FT   V-GENE              64001..73068
FT                       /functional
FT                       /IMGT_allele="IGHV3-74*01"
FT                       /IMGT_gene="IGHV3-74"
FT   5'UTR               64001..68949
FT   L-V-GENE-UNIT       68950..69444
FT                       /functional
FT                       /IMGT_allele="IGHV3-74*01"
FT                       /IMGT_gene="IGHV3-74"
FT   L-INTRON-L          68950..69109
FT   L-PART1             68950..68995
FT                       /translation="MEFGLSWVFLVAILK"
FT   INIT-CODON          68950..68952
FT   DONOR-SPLICE        68995..68997
FT   V-INTRON            68996..69098
FT   ACCEPTOR-SPLICE     69096..69100
FT   V-EXON              69099..69405
FT                       /codon_start=3
FT                       /translation="VQCEVQLVESGGGLVQPGGSLRLSCAASGFTFSSYWMHWVR
FT                       QAPGKGLVWVSRINSDGSSTSYADSVKGRFTISRDNAKNTLYLQMNSLRAEDTAV
FT                       YYCAR"
FT   L-PART2             69099..69109
FT                       /codon_start=3
FT                       /translation="VQC"
FT   V-REGION            69110..69405
FT                       /translation="EVQLVESGGGLVQPGGSLRLSCAASGFTFSSYWMHWVRQAP
FT                       GKGLVWVSRINSDGSSTSYADSVKGRFTISRDNAKNTLYLQMNSLRAEDTAVYYC
FT                       AR"
FT                       /CDR_length="[8.8.2]"
FT                       /FR_length="[25.17.38.G]"
FT                       /functional
FT                       /IMGT_allele="IGHV3-74*01"
FT                       /IMGT_gene="IGHV3-74"
FT   FR1-IMGT            69110..69184
FT                       /translation="EVQLVESGGGLVQPGGSLRLSCAAS"
FT                       /AA_IMGT="AA 1 to 26, AA 10 is missing"
FT   1st-CYS             69173..69175
FT   CDR1-IMGT           69185..69208
FT                       /translation="GFTFSSYW"
FT                       /AA_IMGT="AA 27 to 38, AA 31, 32, 33 and 34 are 
FT                       missing"
FT   FR2-IMGT            69209..69259
FT                       /translation="MHWVRQAPGKGLVWVSR"
FT                       /AA_IMGT="AA 39 to 55"
FT   CONSERVED-TRP       69215..69217
FT   CDR2-IMGT           69260..69283
FT                       /translation="INSDGSST"
FT                       /AA_IMGT="AA 56 to 65, AA 60 and 61 are missing"
FT   FR3-IMGT            69284..69397
FT                       /translation="SYADSVKGRFTISRDNAKNTLYLQMNSLRAEDTAVYYC"
FT                       /AA_IMGT="AA 66 to 104, AA 73 is missing"
FT   3'V-REGION          69395..69405
FT                       /translation="CAR"
FT   2nd-CYS             69395..69397
FT   CDR3-IMGT           69398..69405
FT                       /translation="AR"
FT                       /AA_IMGT="AA 105 to 106"
FT   3'UTR               69406..73068
FT   V-RS                69406..69444
FT   V-HEPTAMER          69406..69412
FT   V-SPACER            69413..69435
FT   V-NONAMER           69436..69444
FT   V-GENE              73069..83000
FT                       /functional
FT                       /IMGT_allele="IGHV3-73*02"
FT                       /IMGT_gene="IGHV3-73"
FT   5'UTR               73069..76691
FT   L-V-GENE-UNIT       76692..77192
FT                       /functional
FT                       /IMGT_allele="IGHV3-73*02"
FT                       /IMGT_gene="IGHV3-73"
FT   L-INTRON-L          76692..76851
FT   L-PART1             76692..76737
FT                       /translation="MEFGLSWVFLVAILK"
FT   INIT-CODON          76692..76694
FT   DONOR-SPLICE        76737..76739
FT   V-INTRON            76738..76840
FT   ACCEPTOR-SPLICE     76838..76842
FT   V-EXON              76841..77153
FT                       /codon_start=3
FT                       /translation="VQCEVQLVESGGGLVQPGGSLKLSCAASGFTFSGSAMHWVR
FT                       QASGKGLEWVGRIRSKANSYATAYAASVKGRFTISRDDSKNTAYLQMNSLKTEDT
FT                       AVYYCTR"
FT   L-PART2             76841..76851
FT                       /codon_start=3
FT                       /translation="VQC"
FT   V-REGION            76852..77153
FT                       /translation="EVQLVESGGGLVQPGGSLKLSCAASGFTFSGSAMHWVRQAS
FT                       GKGLEWVGRIRSKANSYATAYAASVKGRFTISRDDSKNTAYLQMNSLKTEDTAVY
FT                       YCTR"
FT                       /CDR_length="[8.10.2]"
FT                       /FR_length="[25.17.38.G]"
FT                       /functional
FT                       /IMGT_allele="IGHV3-73*02"
FT                       /IMGT_gene="IGHV3-73"
FT   FR1-IMGT            76852..76926
FT                       /translation="EVQLVESGGGLVQPGGSLKLSCAAS"
FT                       /AA_IMGT="AA 1 to 26, AA 10 is missing"
FT   1st-CYS             76915..76917
FT   CDR1-IMGT           76927..76950
FT                       /translation="GFTFSGSA"
FT                       /AA_IMGT="AA 27 to 38, AA 31, 32, 33 and 34 are 
FT                       missing"
FT   FR2-IMGT            76951..77001
FT                       /translation="MHWVRQASGKGLEWVGR"
FT                       /AA_IMGT="AA 39 to 55"
FT   CONSERVED-TRP       76957..76959
FT   CDR2-IMGT           77002..77031
FT                       /translation="IRSKANSYAT"
FT                       /AA_IMGT="AA 56 to 65"
FT   FR3-IMGT            77032..77145
FT                       /translation="AYAASVKGRFTISRDDSKNTAYLQMNSLKTEDTAVYYC"
FT                       /AA_IMGT="AA 66 to 104, AA 73 is missing"
FT   3'V-REGION          77143..77153
FT                       /translation="CTR"
FT   2nd-CYS             77143..77145
FT   CDR3-IMGT           77146..77153
FT                       /translation="TR"
FT                       /AA_IMGT="AA 105 to 106"
FT   3'UTR               77154..83000
FT   V-RS                77154..77192
FT   V-HEPTAMER          77154..77160
FT   V-SPACER            77161..77183
FT   V-NONAMER           77184..77192
FT   V-GENE              83001..96709
FT                       /functional
FT                       /IMGT_allele="IGHV3-72*01"
FT                       /IMGT_gene="IGHV3-72"
FT   5'UTR               83001..88691
FT   L-V-GENE-UNIT       88692..89192
FT                       /functional
FT                       /IMGT_allele="IGHV3-72*01"
FT                       /IMGT_gene="IGHV3-72"
FT   L-INTRON-L          88692..88851
FT   L-PART1             88692..88737
FT                       /translation="MEFGLSWVFLVVILQ"
FT   INIT-CODON          88692..88694
FT   DONOR-SPLICE        88737..88739
FT   V-INTRON            88738..88840
FT   ACCEPTOR-SPLICE     88838..88842
FT   V-EXON              88841..89153
FT                       /codon_start=3
FT                       /translation="VQCEVQLVESGGGLVQPGGSLRLSCAASGFTFSDHYMDWVR
FT                       QAPGKGLEWVGRTRNKANSYTTEYAASVKGRFTISRDDSKNSLYLQMNSLKTEDT
FT                       AVYYCAR"
FT   L-PART2             88841..88851
FT                       /codon_start=3
FT                       /translation="VQC"
FT   V-REGION            88852..89153
FT                       /translation="EVQLVESGGGLVQPGGSLRLSCAASGFTFSDHYMDWVRQAP
FT                       GKGLEWVGRTRNKANSYTTEYAASVKGRFTISRDDSKNSLYLQMNSLKTEDTAVY
FT                       YCAR"
FT                       /CDR_length="[8.10.2]"
FT                       /FR_length="[25.17.38.G]"
FT                       /functional
FT                       /IMGT_allele="IGHV3-72*01"
FT                       /IMGT_gene="IGHV3-72"
FT   FR1-IMGT            88852..88926
FT                       /translation="EVQLVESGGGLVQPGGSLRLSCAAS"
FT                       /AA_IMGT="AA 1 to 26, AA 10 is missing"
FT   1st-CYS             88915..88917
FT   CDR1-IMGT           88927..88950
FT                       /translation="GFTFSDHY"
FT                       /AA_IMGT="AA 27 to 38, AA 31, 32, 33 and 34 are 
FT                       missing"
FT   FR2-IMGT            88951..89001
FT                       /translation="MDWVRQAPGKGLEWVGR"
FT                       /AA_IMGT="AA 39 to 55"
FT   CONSERVED-TRP       88957..88959
FT   CDR2-IMGT           89002..89031
FT                       /translation="TRNKANSYTT"
FT                       /AA_IMGT="AA 56 to 65"
FT   FR3-IMGT            89032..89145
FT                       /translation="EYAASVKGRFTISRDDSKNSLYLQMNSLKTEDTAVYYC"
FT                       /AA_IMGT="AA 66 to 104, AA 73 is missing"
FT   3'V-REGION          89143..89153
FT                       /translation="CAR"
FT   2nd-CYS             89143..89145
FT   CDR3-IMGT           89146..89153
FT                       /translation="AR"
FT                       /AA_IMGT="AA 105 to 106"
FT   3'UTR               89154..96709
FT   V-RS                89154..89192
FT   V-HEPTAMER          89154..89160
FT   V-SPACER            89161..89183
FT   V-NONAMER           89184..89192
FT   V-GENE              96710..106776
FT                       /IMGT_allele="IGHV3-71*01"
FT                       /IMGT_gene="IGHV3-71"
FT                       /pseudo="STOP-CODON in V-REGION: position 67"
FT   5'UTR               96710..104226
FT   L-V-GENE-UNIT       104227..104727
FT                       /IMGT_allele="IGHV3-71*01"
FT                       /IMGT_gene="IGHV3-71"
FT                       /pseudo="STOP-CODON in V-REGION: position 67"
FT   L-INTRON-L          104227..104386
FT   L-PART1             104227..104272
FT                       /translation="MESWLSWVFLAAILK"
FT   INIT-CODON          104227..104229
FT   DONOR-SPLICE        104272..104274
FT   V-INTRON            104273..104375
FT   ACCEPTOR-SPLICE     104373..104377
FT   V-EXON              104376..104688
FT                       /codon_start=3
FT   L-PART2             104376..104386
FT                       /codon_start=3
FT                       /translation="VQC"
FT   V-REGION            104387..104688
FT                       /CDR_length="[8.10.2]"
FT                       /FR_length="[25.17.38.G]"
FT                       /IMGT_allele="IGHV3-71*01"
FT                       /IMGT_gene="IGHV3-71"
FT                       /pseudo="STOP-CODON in V-REGION: position 67"
FT   FR1-IMGT            104387..104461
FT                       /translation="EVQLVESGGGLVQPGGSLRLSCAAS"
FT                       /AA_IMGT="AA 1 to 26, AA 10 is missing"
FT   1st-CYS             104450..104452
FT   CDR1-IMGT           104462..104485
FT                       /translation="GFTFSDYY"
FT                       /AA_IMGT="AA 27 to 38, AA 31, 32, 33 and 34 are 
FT                       missing"
FT   FR2-IMGT            104486..104536
FT                       /translation="MSWVRQAPGKGLEWVGF"
FT                       /AA_IMGT="AA 39 to 55"
FT   CONSERVED-TRP       104492..104494
FT   CDR2-IMGT           104537..104566
FT                       /translation="IRNKANGGTT"
FT                       /AA_IMGT="AA 56 to 65"
FT   FR3-IMGT            104567..104680
FT                       /AA_IMGT="AA 66 to 104, AA 73 is missing"
FT                       /IMGT_note="STOP-CODON in FR3-IMGT: position 67"
FT   3'V-REGION          104678..104688
FT                       /translation="CAR"
FT   2nd-CYS             104678..104680
FT   CDR3-IMGT           104681..104688
FT                       /translation="AR"
FT                       /AA_IMGT="AA 105 to 106"
FT   3'UTR               104689..106776
FT   V-RS                104689..104727
FT   V-HEPTAMER          104689..104695
FT   V-SPACER            104696..104718
FT   V-NONAMER           104719..104727
FT   V-GENE              106777..113311
FT                       /functional
FT                       /IMGT_allele="IGHV2-70*01"
FT                       /IMGT_gene="IGHV2-70"
FT   5'UTR               106777..108824
FT   L-V-GENE-UNIT       108825..109307
FT                       /functional
FT                       /IMGT_allele="IGHV2-70*01"
FT                       /IMGT_gene="IGHV2-70"
FT   L-INTRON-L          108825..108967
FT   L-PART1             108825..108870
FT                       /translation="MDILCSTLLLLTVPS"
FT   INIT-CODON          108825..108827
FT   DONOR-SPLICE        108870..108872
FT   V-INTRON            108871..108956
FT   ACCEPTOR-SPLICE     108954..108958
FT   V-EXON              108957..109268
FT                       /codon_start=3
FT                       /translation="VLSQVTLRESGPALVKPTQTLTLTCTFSGFSLSTSGMCVSW
FT                       IRQPPGKALEWLALIDWDDDKYYSTSLKTRLTISKDTSKNQVVLTMTNMDPVDTA
FT                       TYYCARI"
FT   L-PART2             108957..108967
FT                       /codon_start=3
FT                       /translation="VLS"
FT   V-REGION            108968..109268
FT                       /translation="QVTLRESGPALVKPTQTLTLTCTFSGFSLSTSGMCVSWIRQ
FT                       PPGKALEWLALIDWDDDKYYSTSLKTRLTISKDTSKNQVVLTMTNMDPVDTATYY
FT                       CARI"
FT                       /CDR_length="[10.7.3]"
FT                       /FR_length="[25.17.38.G]"
FT                       /functional
FT                       /IMGT_allele="IGHV2-70*01"
FT                       /IMGT_gene="IGHV2-70"
FT   FR1-IMGT            108968..109042
FT                       /translation="QVTLRESGPALVKPTQTLTLTCTFS"
FT                       /AA_IMGT="AA 1 to 26, AA 10 is missing"
FT   1st-CYS             109031..109033
FT   CDR1-IMGT           109043..109072
FT                       /translation="GFSLSTSGMC"
FT                       /AA_IMGT="AA 27 to 38, AA 32 and 33 are missing"
FT   FR2-IMGT            109073..109123
FT                       /translation="VSWIRQPPGKALEWLAL"
FT                       /AA_IMGT="AA 39 to 55"
FT   CONSERVED-TRP       109079..109081
FT   CDR2-IMGT           109124..109144
FT                       /translation="IDWDDDK"
FT                       /AA_IMGT="AA 56 to 65, AA 60, 61 and 62 are missing"
FT   FR3-IMGT            109145..109258
FT                       /translation="YYSTSLKTRLTISKDTSKNQVVLTMTNMDPVDTATYYC"
FT                       /AA_IMGT="AA 66 to 104, AA 73 is missing"
FT   3'V-REGION          109256..109268
FT                       /translation="CARI"
FT   2nd-CYS             109256..109258
FT   CDR3-IMGT           109259..109268
FT                       /translation="ARI"
FT                       /AA_IMGT="AA 105 to 107"
FT   3'UTR               109269..113311
FT   V-RS                109269..109307
FT   V-HEPTAMER          109269..109275
FT   V-SPACER            109276..109298
FT   V-NONAMER           109299..109307
FT   V-GENE              113312..123455
FT                       /functional
FT                       /IMGT_allele="IGHV1-69D*01"
FT                       /IMGT_gene="IGHV1-69D"
FT   5'UTR               113312..117314
FT   L-V-GENE-UNIT       117315..117792
FT                       /functional
FT                       /IMGT_allele="IGHV1-69D*01"
FT                       /IMGT_gene="IGHV1-69D"
FT   L-INTRON-L          117315..117457
FT   L-PART1             117315..117360
FT                       /translation="MDWTWRFLFVVAAAT"
FT   INIT-CODON          117315..117317
FT   DONOR-SPLICE        117360..117362
FT   V-INTRON            117361..117446
FT   ACCEPTOR-SPLICE     117444..117448
FT   V-EXON              117447..117753
FT                       /codon_start=3
FT                       /translation="VQSQVQLVQSGAEVKKPGSSVKVSCKASGGTFSSYAISWVR
FT                       QAPGQGLEWMGGIIPIFGTANYAQKFQGRVTITADESTSTAYMELSSLRSEDTAV
FT                       YYCAR"
FT   L-PART2             117447..117457
FT                       /codon_start=3
FT                       /translation="VQS"
FT   V-REGION            117458..117753
FT                       /translation="QVQLVQSGAEVKKPGSSVKVSCKASGGTFSSYAISWVRQAP
FT                       GQGLEWMGGIIPIFGTANYAQKFQGRVTITADESTSTAYMELSSLRSEDTAVYYC
FT                       AR"
FT                       /CDR_length="[8.8.2]"
FT                       /FR_length="[25.17.38.G]"
FT                       /functional
FT                       /IMGT_allele="IGHV1-69D*01"
FT                       /IMGT_gene="IGHV1-69D"
FT   FR1-IMGT            117458..117532
FT                       /translation="QVQLVQSGAEVKKPGSSVKVSCKAS"
FT                       /AA_IMGT="AA 1 to 26, AA 10 is missing"
FT   1st-CYS             117521..117523
FT   CDR1-IMGT           117533..117556
FT                       /translation="GGTFSSYA"
FT                       /AA_IMGT="AA 27 to 38, AA 31, 32, 33 and 34 are 
FT                       missing"
FT   FR2-IMGT            117557..117607
FT                       /translation="ISWVRQAPGQGLEWMGG"
FT                       /AA_IMGT="AA 39 to 55"
FT   CONSERVED-TRP       117563..117565
FT   CDR2-IMGT           117608..117631
FT                       /translation="IIPIFGTA"
FT                       /AA_IMGT="AA 56 to 65, AA 60 and 61 are missing"
FT   FR3-IMGT            117632..117745
FT                       /translation="NYAQKFQGRVTITADESTSTAYMELSSLRSEDTAVYYC"
FT                       /AA_IMGT="AA 66 to 104, AA 73 is missing"
FT   3'V-REGION          117743..117753
FT                       /translation="CAR"
FT   2nd-CYS             117743..117745
FT   CDR3-IMGT           117746..117753
FT                       /translation="AR"
FT                       /AA_IMGT="AA 105 to 106"
FT   3'UTR               117754..123455
FT   V-RS                117754..117792
FT   V-HEPTAMER          117754..117760
FT   V-SPACER            117761..117783
FT   V-NONAMER           117784..117792
FT   V-GENE              123456..134000
FT                       /IMGT_allele="IGHV1-68D*02"
FT                       /IMGT_gene="IGHV1-68D"
FT                       /pseudo="frameshift in L-PART1: deletion of 1 nt 
FT                       causing defectuous DONOR-SPLICE"
FT   5'UTR               123456..129117
FT   L-V-GENE-UNIT       129118..129597
FT                       /IMGT_allele="IGHV1-68D*02"
FT                       /IMGT_gene="IGHV1-68D"
FT                       /pseudo="frameshift in L-PART1: deletion of 1 nt 
FT                       causing defectuous DONOR-SPLICE"
FT   L-INTRON-L          129118..129262
FT   L-PART1             129118..129162
FT                       /IMGT_note="frameshift in L-PART1: deletion of 1 nt 
FT                       causing defectuous DONOR-SPLICE"
FT                       /out_of_frame
FT   INIT-CODON          129118..129120
FT   DONOR-SPLICE        129162..129164
FT   V-INTRON            129163..129251
FT   ACCEPTOR-SPLICE     129249..129253
FT                       /IMGT_note="noncanonical ACCEPTOR-SPLICE: nggnn 
FT                       instead of nagnn"
FT   V-EXON              129252..129558
FT                       /codon_start=3
FT                       /translation="VCFQVQLGQSEAEVKKPGASVKVSCKASGYTFTYCSLHWLQ
FT                       QAPGQGLERMRWITLYNGNINYAKKFQSRVTITRDMSLRTAYIELSSLRSEDSAV
FT                       YYWAR"
FT   L-PART2             129252..129262
FT                       /codon_start=3
FT                       /translation="VCF"
FT   V-REGION            129263..129558
FT                       /translation="QVQLGQSEAEVKKPGASVKVSCKASGYTFTYCSLHWLQQAP
FT                       GQGLERMRWITLYNGNINYAKKFQSRVTITRDMSLRTAYIELSSLRSEDSAVYYW
FT                       AR"
FT                       /CDR_length="[8.8.2]"
FT                       /FR_length="[25.17.38.G]"
FT                       /IMGT_allele="IGHV1-68D*02"
FT                       /IMGT_gene="IGHV1-68D"
FT                       /pseudo="frameshift in L-PART1: deletion of 1 nt 
FT                       causing defectuous DONOR-SPLICE"
FT   FR1-IMGT            129263..129337
FT                       /translation="QVQLGQSEAEVKKPGASVKVSCKAS"
FT                       /AA_IMGT="AA 1 to 26, AA 10 is missing"
FT   1st-CYS             129326..129328
FT   CDR1-IMGT           129338..129361
FT                       /translation="GYTFTYCS"
FT                       /AA_IMGT="AA 27 to 38, AA 31, 32, 33 and 34 are 
FT                       missing"
FT   FR2-IMGT            129362..129412
FT                       /translation="LHWLQQAPGQGLERMRW"
FT                       /AA_IMGT="AA 39 to 55"
FT   CONSERVED-TRP       129368..129370
FT   CDR2-IMGT           129413..129436
FT                       /translation="ITLYNGNI"
FT                       /AA_IMGT="AA 56 to 65, AA 60 and 61 are missing"
FT   FR3-IMGT            129437..129550
FT                       /translation="NYAKKFQSRVTITRDMSLRTAYIELSSLRSEDSAVYYW"
FT                       /AA_IMGT="AA 66 to 104, AA 73 is missing"
FT                       /IMGT_note="no 2nd-CYS: Trp instead of Cys"
FT   3'V-REGION          129548..129558
FT                       /translation="WAR"
FT   CDR3-IMGT           129551..129558
FT                       /translation="AR"
FT                       /AA_IMGT="AA 105 to 106"
FT   3'UTR               129559..134000
FT   V-RS                129559..129597
FT   V-HEPTAMER          129559..129565
FT   V-SPACER            129566..129588
FT   V-NONAMER           129589..129597
FT                       /IMGT_note="noncanonical V-NONAMER: tcaggaacc instead 
FT                       of acacaaacc"
FT   V-GENE              134001..139387
FT                       /IMGT_allele="IGHV(III)-67-4D*02"
FT                       /IMGT_gene="IGHV(III)-67-4D"
FT                       /pseudo="no L-PART1, frameshift in V-REGION: insertion 
FT                       of 1 nt in FR1-IMGT"
FT   5'UTR               134001..137979
FT   ACCEPTOR-SPLICE     137977..137981
FT   V-EXON              137980..138285
FT                       /codon_start=3
FT                       /out_of_frame
FT   L-PART2             137980..137990
FT                       /codon_start=3
FT                       /translation="VLY"
FT   V-REGION            137991..138285
FT                       /CDR_length="[8.8.2]"
FT                       /FR_length="[X.17.38.G]"
FT                       /IMGT_allele="IGHV(III)-67-4D*02"
FT                       /IMGT_gene="IGHV(III)-67-4D"
FT                       /IMGT_note="restored translation in CDR1-IMGT, 
FT                       FR2-IMGT, CDR2-IMGT, FR3-IMGT, 3'V-REGION and 
FT                       CDR3-IMGT"
FT                       /out_of_frame
FT                       /pseudo="no L-PART1, frameshift in V-REGION: insertion 
FT                       of 1 nt in FR1-IMGT"
FT   FR1-IMGT            137991..138066
FT                       /IMGT_note="frameshift in FR1-IMGT: insertion of 1 nt"
FT                       /out_of_frame
FT   CDR1-IMGT           138067..138090
FT                       /translation="GFTFAGNS"
FT                       /AA_IMGT="AA 27 to 38, AA 31, 32, 33 and 34 are 
FT                       missing"
FT                       /out_of_frame
FT                       /restored_translation
FT   FR2-IMGT            138091..138141
FT                       /AA_IMGT="AA 39 to 55"
FT                       /out_of_frame
FT                       /restored_translation
FT   CDR2-IMGT           138142..138165
FT                       /translation="VSQQVGVL"
FT                       /AA_IMGT="AA 56 to 65, AA 60 and 61 are missing"
FT                       /out_of_frame
FT                       /restored_translation
FT   FR3-IMGT            138166..138279
FT                       /AA_IMGT="AA 66 to 104, AA 73 is missing"
FT                       /out_of_frame
FT                       /restored_translation
FT   3'V-REGION          138277..138285
FT                       /translation="CEV"
FT                       /out_of_frame
FT                       /restored_translation
FT   CDR3-IMGT           138280..138285
FT                       /translation="EV"
FT                       /AA_IMGT="AA 105 to 106"
FT                       /out_of_frame
FT                       /restored_translation
FT   3'UTR               138286..139387
FT   V-RS                138286..138325
FT   V-HEPTAMER          138286..138292
FT                       /IMGT_note="noncanonical V-HEPTAMER: cacagga instead 
FT                       of cacagtg"
FT   V-SPACER            138293..138316
FT   V-NONAMER           138317..138325
FT   V-GENE              139388..141530
FT                       /IMGT_allele="IGHV(III)-67-3D*02"
FT                       /IMGT_gene="IGHV(III)-67-3D"
FT                       /pseudo="truncated pseudogene not assigned to 
FT                       subgroups with functional genes"
FT   5'UTR               139388..140449
FT   V-REGION            <140450..140724
FT                       /partial
FT                       /CDR_length="[8.8.2]"
FT                       /FR_length="[13.18.X.G]"
FT                       /IMGT_allele="IGHV(III)-67-3D*02"
FT                       /IMGT_gene="IGHV(III)-67-3D"
FT                       /IMGT_note="partial gene in 5', restored translation 
FT                       in 3'V-REGION and CDR3-IMGT"
FT                       /out_of_frame
FT                       /pseudo="truncated pseudogene not assigned to 
FT                       subgroups with functional genes"
FT                       /putative_limit="5'"
FT   FR1-IMGT            <140450..140488
FT                       /partial
FT                       /translation="QPGCFLSLFCKAS"
FT                       /putative_limit="5'"
FT   1st-CYS             140477..140479
FT   CDR1-IMGT           140489..140512
FT                       /translation="RFTFTDYS"
FT                       /AA_IMGT="AA 27 to 38, AA 31, 32, 33 and 34 are 
FT                       missing"
FT   FR2-IMGT            140513..140566
FT                       /IMGT_note="no CONSERVED-TRP: STOP-CODON instead of 
FT                       Trp"
FT   CDR2-IMGT           140567..140590
FT                       /translation="IDSSGSSQ"
FT                       /AA_IMGT="AA 56 to 65, AA 60 and 61 are missing"
FT   FR3-IMGT            140591..140718
FT                       /IMGT_note="frameshifts in FR3-IMGT: insertion of 1 nt 
FT                       and insertion of 13 nt"
FT                       /out_of_frame
FT   3'V-REGION          140715..140724
FT                       /out_of_frame
FT                       /restored_translation
FT   CDR3-IMGT           140719..140724
FT                       /translation="ER"
FT                       /AA_IMGT="AA 105 to 106"
FT                       /out_of_frame
FT                       /restored_translation
FT   3'UTR               140725..141530
FT   V-RS                140725..140762
FT   V-HEPTAMER          140725..140731
FT                       /IMGT_note="noncanonical V-HEPTAMER: tacagcg instead 
FT                       of cacagtg"
FT   V-SPACER            140732..140753
FT   V-NONAMER           140754..140762
FT   V-GENE              141531..147002
FT                       /functional
FT                       /IMGT_allele="IGHV1-69-2*01"
FT                       /IMGT_gene="IGHV1-69-2"
FT   5'UTR               141531..142297
FT   L-V-GENE-UNIT       142298..142774
FT                       /functional
FT                       /IMGT_allele="IGHV1-69-2*01"
FT                       /IMGT_gene="IGHV1-69-2"
FT   L-INTRON-L          142298..142439
FT   L-PART1             142298..142343
FT                       /translation="MDCTWRILLLVAAAT"
FT   INIT-CODON          142298..142300
FT   DONOR-SPLICE        142343..142345
FT   V-INTRON            142344..142428
FT   ACCEPTOR-SPLICE     142426..142430
FT   V-EXON              142429..142735
FT                       /codon_start=3
FT                       /translation="THAEVQLVQSGAEVKKPGATVKISCKVSGYTFTDYYMHWVQ
FT                       QAPGKGLEWMGLVDPEDGETIYAEKFQGRVTITADTSTDTAYMELSSLRSEDTAV
FT                       YYCAT"
FT   L-PART2             142429..142439
FT                       /codon_start=3
FT                       /translation="THA"
FT   V-REGION            142440..142735
FT                       /translation="EVQLVQSGAEVKKPGATVKISCKVSGYTFTDYYMHWVQQAP
FT                       GKGLEWMGLVDPEDGETIYAEKFQGRVTITADTSTDTAYMELSSLRSEDTAVYYC
FT                       AT"
FT                       /CDR_length="[8.8.2]"
FT                       /FR_length="[25.17.38.G]"
FT                       /functional
FT                       /IMGT_allele="IGHV1-69-2*01"
FT                       /IMGT_gene="IGHV1-69-2"
FT   FR1-IMGT            142440..142514
FT                       /translation="EVQLVQSGAEVKKPGATVKISCKVS"
FT                       /AA_IMGT="AA 1 to 26, AA 10 is missing"
FT   1st-CYS             142503..142505
FT   CDR1-IMGT           142515..142538
FT                       /translation="GYTFTDYY"
FT                       /AA_IMGT="AA 27 to 38, AA 31, 32, 33 and 34 are 
FT                       missing"
FT   FR2-IMGT            142539..142589
FT                       /translation="MHWVQQAPGKGLEWMGL"
FT                       /AA_IMGT="AA 39 to 55"
FT   CONSERVED-TRP       142545..142547
FT   CDR2-IMGT           142590..142613
FT                       /translation="VDPEDGET"
FT                       /AA_IMGT="AA 56 to 65, AA 60 and 61 are missing"
FT   FR3-IMGT            142614..142727
FT                       /translation="IYAEKFQGRVTITADTSTDTAYMELSSLRSEDTAVYYC"
FT                       /AA_IMGT="AA 66 to 104, AA 73 is missing"
FT   3'V-REGION          142725..142735
FT                       /translation="CAT"
FT   2nd-CYS             142725..142727
FT   CDR3-IMGT           142728..142735
FT                       /translation="AT"
FT                       /AA_IMGT="AA 105 to 106"
FT   3'UTR               142736..147002
FT   V-RS                142736..142774
FT   V-HEPTAMER          142736..142742
FT   V-SPACER            142743..142765
FT   V-NONAMER           142766..142774
FT   V-GENE              147003..153774
FT                       /IMGT_allele="IGHV3-69-1*01"
FT                       /IMGT_gene="IGHV3-69-1"
FT                       /pseudo="no INIT-CODON: Lys instead of Met"
FT   5'UTR               147003..151229
FT   L-V-GENE-UNIT       151230..151721
FT                       /IMGT_allele="IGHV3-69-1*01"
FT                       /IMGT_gene="IGHV3-69-1"
FT                       /pseudo="no INIT-CODON: Lys instead of Met"
FT   L-INTRON-L          151230..151389
FT   L-PART1             151230..151275
FT                       /translation="KESWLSWLFLVAILK"
FT                       /IMGT_note="no INIT-CODON: Lys instead of Met"
FT   DONOR-SPLICE        151275..151277
FT   V-INTRON            151276..151378
FT   ACCEPTOR-SPLICE     151376..151380
FT   V-EXON              151379..151682
FT                       /codon_start=3
FT                       /translation="VQCEVQLVESGGGLVKPGGSLRLSCAASGFTFSDYYMNWVR
FT                       QAPGKGLEWVSSISSSSTIYYADSVKGRFTISRDNAKNSLYLQMNSLRAEDTAVY
FT                       YCAR"
FT   L-PART2             151379..151389
FT                       /codon_start=3
FT                       /translation="VQC"
FT   V-REGION            151390..151682
FT                       /translation="EVQLVESGGGLVKPGGSLRLSCAASGFTFSDYYMNWVRQAP
FT                       GKGLEWVSSISSSSTIYYADSVKGRFTISRDNAKNSLYLQMNSLRAEDTAVYYCA
FT                       R"
FT                       /CDR_length="[8.7.2]"
FT                       /FR_length="[25.17.38.G]"
FT                       /IMGT_allele="IGHV3-69-1*01"
FT                       /IMGT_gene="IGHV3-69-1"
FT                       /pseudo="no INIT-CODON: Lys instead of Met"
FT   FR1-IMGT            151390..151464
FT                       /translation="EVQLVESGGGLVKPGGSLRLSCAAS"
FT                       /AA_IMGT="AA 1 to 26, AA 10 is missing"
FT   1st-CYS             151453..151455
FT   CDR1-IMGT           151465..151488
FT                       /translation="GFTFSDYY"
FT                       /AA_IMGT="AA 27 to 38, AA 31, 32, 33 and 34 are 
FT                       missing"
FT   FR2-IMGT            151489..151539
FT                       /translation="MNWVRQAPGKGLEWVSS"
FT                       /AA_IMGT="AA 39 to 55"
FT   CONSERVED-TRP       151495..151497
FT   CDR2-IMGT           151540..151560
FT                       /translation="ISSSSTI"
FT                       /AA_IMGT="AA 56 to 65, AA 60, 61 and 62 are missing"
FT   FR3-IMGT            151561..151674
FT                       /translation="YYADSVKGRFTISRDNAKNSLYLQMNSLRAEDTAVYYC"
FT                       /AA_IMGT="AA 66 to 104, AA 73 is missing"
FT   3'V-REGION          151672..151682
FT                       /translation="CAR"
FT   2nd-CYS             151672..151674
FT   CDR3-IMGT           151675..151682
FT                       /translation="AR"
FT                       /AA_IMGT="AA 105 to 106"
FT   3'UTR               151683..153774
FT   V-RS                151683..151721
FT   V-HEPTAMER          151683..151689
FT   V-SPACER            151690..151712
FT   V-NONAMER           151713..151721
FT   V-GENE              153775..160517
FT                       /functional
FT                       /IMGT_allele="IGHV2-70D*04"
FT                       /IMGT_gene="IGHV2-70D"
FT   5'UTR               153775..155827
FT   L-V-GENE-UNIT       155828..156310
FT                       /functional
FT                       /IMGT_allele="IGHV2-70D*04"
FT                       /IMGT_gene="IGHV2-70D"
FT   L-INTRON-L          155828..155970
FT   L-PART1             155828..155873
FT                       /translation="MDILCSTLLLLTVPS"
FT   INIT-CODON          155828..155830
FT   DONOR-SPLICE        155873..155875
FT   V-INTRON            155874..155959
FT   ACCEPTOR-SPLICE     155957..155961
FT   V-EXON              155960..156271
FT                       /codon_start=3
FT                       /translation="VLSQVTLKESGPALVKPTQTLTLTCTFSGFSLSTSGMRVSW
FT                       IRQPPGKALEWLARIDWDDDKFYSTSLKTRLTISKDTSKNQVVLTMTNMDPVDTA
FT                       TYYCARI"
FT   L-PART2             155960..155970
FT                       /codon_start=3
FT                       /translation="VLS"
FT   V-REGION            155971..156271
FT                       /translation="QVTLKESGPALVKPTQTLTLTCTFSGFSLSTSGMRVSWIRQ
FT                       PPGKALEWLARIDWDDDKFYSTSLKTRLTISKDTSKNQVVLTMTNMDPVDTATYY
FT                       CARI"
FT                       /CDR_length="[10.7.3]"
FT                       /FR_length="[25.17.38.G]"
FT                       /functional
FT                       /IMGT_allele="IGHV2-70D*04"
FT                       /IMGT_gene="IGHV2-70D"
FT   FR1-IMGT            155971..156045
FT                       /translation="QVTLKESGPALVKPTQTLTLTCTFS"
FT                       /AA_IMGT="AA 1 to 26, AA 10 is missing"
FT   1st-CYS             156034..156036
FT   CDR1-IMGT           156046..156075
FT                       /translation="GFSLSTSGMR"
FT                       /AA_IMGT="AA 27 to 38, AA 32 and 33 are missing"
FT   FR2-IMGT            156076..156126
FT                       /translation="VSWIRQPPGKALEWLAR"
FT                       /AA_IMGT="AA 39 to 55"
FT   CONSERVED-TRP       156082..156084
FT   CDR2-IMGT           156127..156147
FT                       /translation="IDWDDDK"
FT                       /AA_IMGT="AA 56 to 65, AA 60, 61 and 62 are missing"
FT   FR3-IMGT            156148..156261
FT                       /translation="FYSTSLKTRLTISKDTSKNQVVLTMTNMDPVDTATYYC"
FT                       /AA_IMGT="AA 66 to 104, AA 73 is missing"
FT   3'V-REGION          156259..156271
FT                       /translation="CARI"
FT   2nd-CYS             156259..156261
FT   CDR3-IMGT           156262..156271
FT                       /translation="ARI"
FT                       /AA_IMGT="AA 105 to 107"
FT   3'UTR               156272..160517
FT   V-RS                156272..156310
FT   V-HEPTAMER          156272..156278
FT   V-SPACER            156279..156301
FT   V-NONAMER           156302..156310
FT   V-GENE              160518..170380
FT                       /functional
FT                       /IMGT_allele="IGHV1-69*06"
FT                       /IMGT_gene="IGHV1-69"
FT   5'UTR               160518..164724
FT   L-V-GENE-UNIT       164725..165202
FT                       /functional
FT                       /IMGT_allele="IGHV1-69*06"
FT                       /IMGT_gene="IGHV1-69"
FT   L-INTRON-L          164725..164867
FT   L-PART1             164725..164770
FT                       /translation="MDWTWRFLFVVAAAT"
FT   INIT-CODON          164725..164727
FT   DONOR-SPLICE        164770..164772
FT   V-INTRON            164771..164856
FT   ACCEPTOR-SPLICE     164854..164858
FT   V-EXON              164857..165163
FT                       /codon_start=3
FT                       /translation="VQSQVQLVQSGAEVKKPGSSVKVSCKASGGTFSSYAISWVR
FT                       QAPGQGLEWMGGIIPIFGTANYAQKFQGRVTITADKSTSTAYMELSSLRSEDTAV
FT                       YYCAR"
FT   L-PART2             164857..164867
FT                       /codon_start=3
FT                       /translation="VQS"
FT   V-REGION            164868..165163
FT                       /translation="QVQLVQSGAEVKKPGSSVKVSCKASGGTFSSYAISWVRQAP
FT                       GQGLEWMGGIIPIFGTANYAQKFQGRVTITADKSTSTAYMELSSLRSEDTAVYYC
FT                       AR"
FT                       /CDR_length="[8.8.2]"
FT                       /FR_length="[25.17.38.G]"
FT                       /functional
FT                       /IMGT_allele="IGHV1-69*06"
FT                       /IMGT_gene="IGHV1-69"
FT   FR1-IMGT            164868..164942
FT                       /translation="QVQLVQSGAEVKKPGSSVKVSCKAS"
FT                       /AA_IMGT="AA 1 to 26, AA 10 is missing"
FT   1st-CYS             164931..164933
FT   CDR1-IMGT           164943..164966
FT                       /translation="GGTFSSYA"
FT                       /AA_IMGT="AA 27 to 38, AA 31, 32, 33 and 34 are 
FT                       missing"
FT   FR2-IMGT            164967..165017
FT                       /translation="ISWVRQAPGQGLEWMGG"
FT                       /AA_IMGT="AA 39 to 55"
FT   CONSERVED-TRP       164973..164975
FT   CDR2-IMGT           165018..165041
FT                       /translation="IIPIFGTA"
FT                       /AA_IMGT="AA 56 to 65, AA 60 and 61 are missing"
FT   FR3-IMGT            165042..165155
FT                       /translation="NYAQKFQGRVTITADKSTSTAYMELSSLRSEDTAVYYC"
FT                       /AA_IMGT="AA 66 to 104, AA 73 is missing"
FT   3'V-REGION          165153..165163
FT                       /translation="CAR"
FT   2nd-CYS             165153..165155
FT   CDR3-IMGT           165156..165163
FT                       /translation="AR"
FT                       /AA_IMGT="AA 105 to 106"
FT   3'UTR               165164..170380
FT   V-RS                165164..165202
FT   V-HEPTAMER          165164..165170
FT   V-SPACER            165171..165193
FT   V-NONAMER           165194..165202
FT   V-GENE              170381..180000
FT                       /IMGT_allele="IGHV1-68*01"
FT                       /IMGT_gene="IGHV1-68"
FT                       /pseudo="frameshift in L-PART1: deletion of 1 nt 
FT                       causing defectuous DONOR-SPLICE"
FT   5'UTR               170381..175558
FT   L-V-GENE-UNIT       175559..176038
FT                       /IMGT_allele="IGHV1-68*01"
FT                       /IMGT_gene="IGHV1-68"
FT                       /pseudo="frameshift in L-PART1: deletion of 1 nt 
FT                       causing defectuous DONOR-SPLICE"
FT   L-INTRON-L          175559..175703
FT   L-PART1             175559..175603
FT                       /IMGT_note="frameshift in L-PART1: deletion of 1 nt 
FT                       causing defectuous DONOR-SPLICE"
FT                       /out_of_frame
FT   INIT-CODON          175559..175561
FT   DONOR-SPLICE        175603..175605
FT   V-INTRON            175604..175692
FT   ACCEPTOR-SPLICE     175690..175694
FT                       /IMGT_note="noncanonical ACCEPTOR-SPLICE: nggnn 
FT                       instead of nagnn"
FT   V-EXON              175693..175999
FT                       /codon_start=3
FT                       /translation="VCSQVQLGQSEAEVKKPGASVKVSCKASGYTFTCCSLHWLQ
FT                       QAPGQGLERMRWITLYNGNTNYAKKFQGRVTITRDMSLRTAYIELSSLRSEDSAV
FT                       YYWAR"
FT   L-PART2             175693..175703
FT                       /codon_start=3
FT                       /translation="VCS"
FT   V-REGION            175704..175999
FT                       /translation="QVQLGQSEAEVKKPGASVKVSCKASGYTFTCCSLHWLQQAP
FT                       GQGLERMRWITLYNGNTNYAKKFQGRVTITRDMSLRTAYIELSSLRSEDSAVYYW
FT                       AR"
FT                       /CDR_length="[8.8.2]"
FT                       /FR_length="[25.17.38.G]"
FT                       /IMGT_allele="IGHV1-68*01"
FT                       /IMGT_gene="IGHV1-68"
FT                       /pseudo="frameshift in L-PART1: deletion of 1 nt 
FT                       causing defectuous DONOR-SPLICE"
FT   FR1-IMGT            175704..175778
FT                       /translation="QVQLGQSEAEVKKPGASVKVSCKAS"
FT                       /AA_IMGT="AA 1 to 26, AA 10 is missing"
FT   1st-CYS             175767..175769
FT   CDR1-IMGT           175779..175802
FT                       /translation="GYTFTCCS"
FT                       /AA_IMGT="AA 27 to 38, AA 31, 32, 33 and 34 are 
FT                       missing"
FT   FR2-IMGT            175803..175853
FT                       /translation="LHWLQQAPGQGLERMRW"
FT                       /AA_IMGT="AA 39 to 55"
FT   CONSERVED-TRP       175809..175811
FT   CDR2-IMGT           175854..175877
FT                       /translation="ITLYNGNT"
FT                       /AA_IMGT="AA 56 to 65, AA 60 and 61 are missing"
FT   FR3-IMGT            175878..175991
FT                       /translation="NYAKKFQGRVTITRDMSLRTAYIELSSLRSEDSAVYYW"
FT                       /AA_IMGT="AA 66 to 104, AA 73 is missing"
FT                       /IMGT_note="no 2nd-CYS: Trp instead of Cys"
FT   3'V-REGION          175989..175999
FT                       /translation="WAR"
FT   CDR3-IMGT           175992..175999
FT                       /translation="AR"
FT                       /AA_IMGT="AA 105 to 106"
FT   3'UTR               176000..180000
FT   V-RS                176000..176038
FT   V-HEPTAMER          176000..176006
FT   V-SPACER            176007..176029
FT   V-NONAMER           176030..176038
FT                       /IMGT_note="noncanonical V-NONAMER: tcaggaacc instead 
FT                       of acacaaacc"
FT   V-GENE              180001..185874
FT                       /IMGT_allele="IGHV(III)-67-4*01"
FT                       /IMGT_gene="IGHV(III)-67-4"
FT                       /pseudo="frameshifts in V-REGION: several insertions 
FT                       and deletions"
FT   5'UTR               180001..184324
FT   L-V-GENE-UNIT       184325..184793
FT                       /IMGT_allele="IGHV(III)-67-4*01"
FT                       /IMGT_gene="IGHV(III)-67-4"
FT                       /pseudo="frameshifts in V-REGION: several insertions 
FT                       and deletions"
FT   L-INTRON-L          184325..184458
FT   L-PART1             184325..184370
FT                       /translation="MESALIWVSLLITLH"
FT   INIT-CODON          184325..184327
FT   DONOR-SPLICE        184370..184372
FT                       /IMGT_note="noncanonical DONOR-SPLICE: ntt instead of 
FT                       ngt"
FT   V-INTRON            184371..184447
FT   ACCEPTOR-SPLICE     184445..184449
FT   V-EXON              184448..184753
FT                       /codon_start=3
FT                       /out_of_frame
FT   L-PART2             184448..184458
FT                       /codon_start=3
FT                       /translation="VLY"
FT   V-REGION            184459..184753
FT                       /IMGT_allele="IGHV(III)-67-4*01"
FT                       /IMGT_gene="IGHV(III)-67-4"
FT                       /out_of_frame
FT                       /pseudo="frameshifts in V-REGION: several insertions 
FT                       and deletions"
FT   3'UTR               184754..185874
FT   V-RS                184754..184793
FT   V-HEPTAMER          184754..184760
FT                       /IMGT_note="noncanonical V-HEPTAMER: cacagga instead 
FT                       of cacagtg"
FT   V-SPACER            184761..184784
FT   V-NONAMER           184785..184793
FT   V-GENE              185875..189957
FT                       /IMGT_allele="IGHV(III)-67-3*01"
FT                       /IMGT_gene="IGHV(III)-67-3"
FT                       /pseudo="truncated pseudogene not assigned to 
FT                       subgroups with functional genes"
FT   5'UTR               185875..186956
FT   V-REGION            <186957..187231
FT                       /partial
FT                       /CDR_length="[8.8.2]"
FT                       /FR_length="[13.18.X.G]"
FT                       /IMGT_allele="IGHV(III)-67-3*01"
FT                       /IMGT_gene="IGHV(III)-67-3"
FT                       /IMGT_note="partial gene in 5', restored translation 
FT                       in 3'V-REGION and CDR3-IMGT"
FT                       /out_of_frame
FT                       /pseudo="truncated pseudogene not assigned to 
FT                       subgroups with functional genes"
FT                       /putative_limit="5'"
FT   FR1-IMGT            <186957..186995
FT                       /partial
FT                       /translation="QPGCSLSLFCKAS"
FT                       /putative_limit="5'"
FT   1st-CYS             186984..186986
FT   CDR1-IMGT           186996..187019
FT                       /translation="RFTFTDYS"
FT                       /AA_IMGT="AA 27 to 38, AA 31, 32, 33 and 34 are 
FT                       missing"
FT   FR2-IMGT            187020..187073
FT                       /IMGT_note="insertions in FR2-IMGT: 1 AA is added"
FT   CDR2-IMGT           187074..187097
FT                       /translation="IDSSGSSQ"
FT                       /AA_IMGT="AA 56 to 65, AA 60 and 61 are missing"
FT   FR3-IMGT            187098..187225
FT                       /IMGT_note="frameshifts in FR3-IMGT: insertion of 1 nt 
FT                       and insertion of 13 nt"
FT                       /out_of_frame
FT   3'V-REGION          187223..187231
FT                       /translation="CER"
FT                       /out_of_frame
FT                       /restored_translation
FT   CDR3-IMGT           187226..187231
FT                       /translation="ER"
FT                       /AA_IMGT="AA 105 to 106"
FT                       /out_of_frame
FT                       /restored_translation
FT   3'UTR               187232..189957
FT   V-RS                187232..187269
FT   V-HEPTAMER          187232..187238
FT   V-SPACER            187239..187260
FT   V-NONAMER           187261..187269
FT   V-GENE              189958..192982
FT                       /IMGT_allele="IGHV(III)-67-2*01"
FT                       /IMGT_gene="IGHV(III)-67-2"
FT                       /pseudo="truncated pseudogene not assigned to 
FT                       subgroups with functional genes"
FT   5'UTR               189958..192644
FT   V-REGION            <192645..192743
FT                       /partial
FT                       /codon_start=3
FT                       /CDR_length="[X.X.3]"
FT                       /FR_length="[X.X.X.G]"
FT                       /IMGT_allele="IGHV(III)-67-2*01"
FT                       /IMGT_gene="IGHV(III)-67-2"
FT                       /IMGT_note="partial gene in 5'"
FT                       /pseudo="truncated pseudogene not assigned to 
FT                       subgroups with functional genes"
FT                       /putative_limit="5'"
FT   FR3-IMGT            <192645..192733
FT                       /partial
FT                       /codon_start=3
FT                       /IMGT_note="STOP-CODON in FR3-IMGT: position 95"
FT   3'V-REGION          192731..192743
FT                       /translation="CARG"
FT   2nd-CYS             192731..192733
FT   CDR3-IMGT           192734..192743
FT                       /translation="ARG"
FT                       /AA_IMGT="AA 105 to 107"
FT   3'UTR               192744..192982
FT   V-RS                192744..192781
FT   V-HEPTAMER          192744..192750
FT   V-SPACER            192751..192772
FT   V-NONAMER           192773..192781
FT   V-GENE              192983..194500
FT                       /IMGT_allele="IGHV(II)-67-1*01"
FT                       /IMGT_gene="IGHV(II)-67-1"
FT                       /pseudo="truncated pseudogene not assigned to 
FT                       subgroups with functional genes"
FT   5'UTR               192983..193302
FT   ACCEPTOR-SPLICE     193300..193304
FT   V-EXON              193303..193452
FT                       /codon_start=3
FT                       /out_of_frame
FT                       /putative_limit="3'"
FT   L-PART2             193303..193313
FT                       /codon_start=3
FT                       /translation="VLF"
FT   V-REGION            193314..>193452
FT                       /partial
FT                       /CDR_length="[10.X.X]"
FT                       /FR_length="[X.X.X.G]"
FT                       /IMGT_allele="IGHV(II)-67-1*01"
FT                       /IMGT_gene="IGHV(II)-67-1"
FT                       /IMGT_note="partial gene in 3'"
FT                       /out_of_frame
FT                       /pseudo="truncated pseudogene not assigned to 
FT                       subgroups with functional genes"
FT                       /putative_limit="3'"
FT   FR1-IMGT            193314..193379
FT                       /IMGT_note="frameshifts in FR1-IMGT: deletion of 1 nt, 
FT                        deletion of 7 nt and deletion of 1 nt"
FT   CDR1-IMGT           193380..193409
FT                       /AA_IMGT="AA 27 to 38, AA 32 and 33 are missing"
FT                       /IMGT_note="STOP-CODON in CDR1-IMGT: position 37"
FT   FR2-IMGT            193410..193452
FT                       /translation="WTWICQSPGNGLNG"
FT   CONSERVED-TRP       193416..193418
FT   3'UTR               193453..194500
FT   GENE                194501..198000
FT                       /IMGT_gene="SLC20A1P1"
FT                       /pseudo
FT   5'UTR               194501..195495
FT   GENE-UNIT           195496..197106
FT                       /IMGT_gene="SLC20A1P1"
FT                       /pseudo
FT   3'UTR               197107..198000
FT   V-GENE              198001..201830
FT                       /IMGT_allele="IGHV1-67*01"
FT                       /IMGT_gene="IGHV1-67"
FT                       /pseudo="frameshift in V-REGION: insertion of 4 nt in 
FT                       CDR1-IMGT"
FT   5'UTR               198001..198802
FT   L-V-GENE-UNIT       198803..199281
FT                       /IMGT_allele="IGHV1-67*01"
FT                       /IMGT_gene="IGHV1-67"
FT                       /pseudo="frameshift in V-REGION: insertion of 4 nt in 
FT                       CDR1-IMGT"
FT   L-INTRON-L          198803..198942
FT   L-PART1             198803..198848
FT                       /translation="MEWTWRILILVVVAA"
FT   INIT-CODON          198803..198805
FT   DONOR-SPLICE        198848..198850
FT   V-INTRON            198849..198931
FT   ACCEPTOR-SPLICE     198929..198933
FT   V-EXON              198932..199242
FT                       /codon_start=3
FT                       /out_of_frame
FT   L-PART2             198932..198942
FT                       /codon_start=3
FT                       /translation="AQS"
FT   V-REGION            198943..199242
FT                       /CDR_length="[X.8.2]"
FT                       /FR_length="[25.17.38.G]"
FT                       /IMGT_allele="IGHV1-67*01"
FT                       /IMGT_gene="IGHV1-67"
FT                       /IMGT_note="restored translation in FR2-IMGT, 
FT                       CDR2-IMGT, FR3-IMGT, 3'V-REGION and CDR3-IMGT"
FT                       /out_of_frame
FT                       /pseudo="frameshift in V-REGION: insertion of 4 nt in 
FT                       CDR1-IMGT"
FT   FR1-IMGT            198943..199017
FT                       /translation="QVQLVQSGDEMKKAGASVKVSCKTC"
FT                       /AA_IMGT="AA 1 to 26, AA 10 is missing"
FT   1st-CYS             199006..199008
FT   CDR1-IMGT           199018..199045
FT                       /translation="GYTYLHQLL"
FT                       /IMGT_note="frameshift in CDR1-IMGT: insertion of 4 nt"
FT                       /out_of_frame
FT   FR2-IMGT            199046..199096
FT                       /AA_IMGT="AA 39 to 55"
FT                       /out_of_frame
FT                       /restored_translation
FT   CDR2-IMGT           199097..199120
FT                       /translation="MCPSDGSI"
FT                       /AA_IMGT="AA 56 to 65, AA 60 and 61 are missing"
FT                       /out_of_frame
FT                       /restored_translation
FT   FR3-IMGT            199121..199234
FT                       /translation="SYAEKFQGRVTMTRDTSTSTAYMELSSLRSEDTAMYYC"
FT                       /AA_IMGT="AA 66 to 104, AA 73 is missing"
FT                       /out_of_frame
FT                       /restored_translation
FT   3'V-REGION          199232..199242
FT                       /translation="CGR"
FT                       /out_of_frame
FT                       /restored_translation
FT   CDR3-IMGT           199235..199242
FT                       /translation="GR"
FT                       /AA_IMGT="AA 105 to 106"
FT                       /out_of_frame
FT                       /restored_translation
FT   3'UTR               199243..201830
FT   V-RS                199243..199281
FT   V-HEPTAMER          199243..199249
FT   V-SPACER            199250..199272
FT   V-NONAMER           199273..199281
FT                       /IMGT_note="noncanonical V-NONAMER: tcagtaacc instead 
FT                       of acacaaacc"
FT   V-GENE              201831..206293
FT                       /functional
FT                       /IMGT_allele="IGHV3-66*03"
FT                       /IMGT_gene="IGHV3-66"
FT   5'UTR               201831..204379
FT   L-V-GENE-UNIT       204380..204869
FT                       /functional
FT                       /IMGT_allele="IGHV3-66*03"
FT                       /IMGT_gene="IGHV3-66"
FT   L-INTRON-L          204380..204537
FT   L-PART1             204380..204425
FT                       /translation="MEFGLSWVFLVAILK"
FT   INIT-CODON          204380..204382
FT   DONOR-SPLICE        204425..204427
FT   V-INTRON            204426..204526
FT   ACCEPTOR-SPLICE     204524..204528
FT   V-EXON              204527..204830
FT                       /codon_start=3
FT                       /translation="VQCEVQLVESGGGLIQPGGSLRLSCAASGFTVSSNYMSWVR
FT                       QAPGKGLEWVSVIYSCGSTYYADSVKGRFTISRDNSKNTLYLQMNSLRAEDTAVY
FT                       YCAR"
FT   L-PART2             204527..204537
FT                       /codon_start=3
FT                       /translation="VQC"
FT   V-REGION            204538..204830
FT                       /translation="EVQLVESGGGLIQPGGSLRLSCAASGFTVSSNYMSWVRQAP
FT                       GKGLEWVSVIYSCGSTYYADSVKGRFTISRDNSKNTLYLQMNSLRAEDTAVYYCA
FT                       R"
FT                       /CDR_length="[8.7.2]"
FT                       /FR_length="[25.17.38.G]"
FT                       /functional
FT                       /IMGT_allele="IGHV3-66*03"
FT                       /IMGT_gene="IGHV3-66"
FT   FR1-IMGT            204538..204612
FT                       /translation="EVQLVESGGGLIQPGGSLRLSCAAS"
FT                       /AA_IMGT="AA 1 to 26, AA 10 is missing"
FT   1st-CYS             204601..204603
FT   CDR1-IMGT           204613..204636
FT                       /translation="GFTVSSNY"
FT                       /AA_IMGT="AA 27 to 38, AA 31, 32, 33 and 34 are 
FT                       missing"
FT   FR2-IMGT            204637..204687
FT                       /translation="MSWVRQAPGKGLEWVSV"
FT                       /AA_IMGT="AA 39 to 55"
FT   CONSERVED-TRP       204643..204645
FT   CDR2-IMGT           204688..204708
FT                       /translation="IYSCGST"
FT                       /AA_IMGT="AA 56 to 65, AA 60, 61 and 62 are missing"
FT   FR3-IMGT            204709..204822
FT                       /translation="YYADSVKGRFTISRDNSKNTLYLQMNSLRAEDTAVYYC"
FT                       /AA_IMGT="AA 66 to 104, AA 73 is missing"
FT   3'V-REGION          204820..204830
FT                       /translation="CAR"
FT   2nd-CYS             204820..204822
FT   CDR3-IMGT           204823..204830
FT                       /translation="AR"
FT                       /AA_IMGT="AA 105 to 106"
FT   3'UTR               204831..206293
FT   V-RS                204831..204869
FT   V-HEPTAMER          204831..204837
FT   V-SPACER            204838..204860
FT   V-NONAMER           204861..204869
FT   V-GENE              206294..210712
FT                       /IMGT_allele="IGHV(II)-65-1*01"
FT                       /IMGT_gene="IGHV(II)-65-1"
FT                       /pseudo="no L-PART1, frameshifts in V-REGION: several 
FT                       insertions and deletions"
FT   5'UTR               206294..207760
FT   ACCEPTOR-SPLICE     207758..207762
FT   V-EXON              207761..208073
FT                       /codon_start=3
FT                       /out_of_frame
FT   L-PART2             207761..207771
FT                       /codon_start=3
FT                       /translation="LVH"
FT   V-REGION            207772..208073
FT                       /CDR_length="[10.X.2]"
FT                       /FR_length="[X.X.38.G]"
FT                       /IMGT_allele="IGHV(II)-65-1*01"
FT                       /IMGT_gene="IGHV(II)-65-1"
FT                       /IMGT_note="restored translation in CDR1-IMGT, 
FT                       FR2-IMGT, CDR2-IMGT"
FT                       /out_of_frame
FT                       /pseudo="no L-PART1, frameshifts in V-REGION: several 
FT                       insertions and deletions"
FT   FR1-IMGT            207772..207848
FT                       /IMGT_note="frameshift in FR1-IMGT: insertion of 2 nt"
FT                       /out_of_frame
FT   CDR1-IMGT           207849..207878
FT                       /translation="AFSITTSASC"
FT                       /AA_IMGT="AA 27 to 38, AA 32 and 33 are missing"
FT                       /out_of_frame
FT                       /restored_translation
FT   FR2-IMGT            207879..207931
FT                       /translation="WSCIHHPPQGRDWSESG"
FT                       /IMGT_note="frameshift in FR2-IMGT: insertion of 2 nt"
FT                       /out_of_frame
FT                       /restored_translation
FT   CDR2-IMGT           207932..207951
FT                       /translation="TGHEGV"
FT                       /IMGT_note="frameshift in CDR2-IMGT: deletion of 1 nt"
FT                       /out_of_frame
FT                       /restored_translation
FT   FR3-IMGT            207952..208065
FT                       /AA_IMGT="AA 66 to 104, AA 73 is missing"
FT                       /IMGT_note="no 2nd-CYS: Phe instead of Cys, STOP-CODON 
FT                       in FR3-IMGT: position 90"
FT                       /in_frame
FT   3'V-REGION          208063..208073
FT                       /translation="NTK"
FT                       /in_frame
FT   CDR3-IMGT           208066..208073
FT                       /translation="TK"
FT                       /AA_IMGT="AA 105 to 106"
FT                       /in_frame
FT   3'UTR               208074..210712
FT   V-RS                208074..208112
FT   V-HEPTAMER          208074..208080
FT                       /IMGT_note="noncanonical V-HEPTAMER: cacaacg instead 
FT                       of cacagtg"
FT   V-SPACER            208081..208103
FT   V-NONAMER           208104..208112
FT   V-GENE              210713..217735
FT                       /IMGT_allele="IGHV3-65*02"
FT                       /IMGT_gene="IGHV3-65"
FT                       /pseudo="frameshift in V-REGION: insertion of 1 nt in 
FT                       CDR1-IMGT"
FT   5'UTR               210713..213312
FT   L-V-GENE-UNIT       213313..213807
FT                       /IMGT_allele="IGHV3-65*02"
FT                       /IMGT_gene="IGHV3-65"
FT                       /pseudo="frameshift in V-REGION: insertion of 1 nt in 
FT                       CDR1-IMGT"
FT   L-INTRON-L          213313..213465
FT   L-PART1             213313..213358
FT                       /translation="MKSGLSRFFLVAILK"
FT   INIT-CODON          213313..213315
FT   DONOR-SPLICE        213358..213360
FT                       /IMGT_note="noncanonical DONOR-SPLICE: nat instead of 
FT                       ngt"
FT   V-INTRON            213359..213454
FT   ACCEPTOR-SPLICE     213452..213456
FT   V-EXON              213455..213768
FT                       /codon_start=3
FT                       /out_of_frame
FT   L-PART2             213455..213465
FT                       /codon_start=3
FT                       /translation="VQC"
FT   V-REGION            213466..213768
FT                       /CDR_length="[X.10.2]"
FT                       /FR_length="[25.17.38.G]"
FT                       /IMGT_allele="IGHV3-65*02"
FT                       /IMGT_gene="IGHV3-65"
FT                       /IMGT_note="restored translation in FR2-IMGT, 
FT                       CDR2-IMGT, FR3-IMGT, 3'V-REGION and CDR3-IMGT"
FT                       /out_of_frame
FT                       /pseudo="frameshift in V-REGION: insertion of 1 nt in 
FT                       CDR1-IMGT"
FT   FR1-IMGT            213466..213540
FT                       /translation="EVQLVESGGGLVQPGGSLRLSCAAS"
FT                       /AA_IMGT="AA 1 to 26, AA 10 is missing"
FT   1st-CYS             213529..213531
FT   CDR1-IMGT           213541..213565
FT                       /IMGT_note="frameshift in CDR1-IMGT: insertion of 1 nt"
FT                       /out_of_frame
FT   FR2-IMGT            213566..213616
FT                       /translation="MQWICRAPGKGLEWVGF"
FT                       /AA_IMGT="AA 39 to 55"
FT                       /out_of_frame
FT                       /restored_translation
FT   CDR2-IMGT           213617..213646
FT                       /translation="TKNKTNRGTT"
FT                       /AA_IMGT="AA 56 to 65"
FT                       /out_of_frame
FT                       /restored_translation
FT   FR3-IMGT            213647..213760
FT                       /translation="EYAASVKGRFTISSDDSKSIAYLQMNSLKTEDTAVYYY"
FT                       /AA_IMGT="AA 66 to 104, AA 73 is missing"
FT                       /out_of_frame
FT                       /restored_translation
FT   3'V-REGION          213758..213768
FT                       /translation="YTR"
FT                       /out_of_frame
FT                       /restored_translation
FT   CDR3-IMGT           213761..213768
FT                       /translation="TR"
FT                       /AA_IMGT="AA 105 to 106"
FT                       /out_of_frame
FT                       /restored_translation
FT   3'UTR               213769..217735
FT   V-RS                213769..213807
FT   V-HEPTAMER          213769..213775
FT   V-SPACER            213776..213798
FT   V-NONAMER           213799..213807
FT   V-GENE              217736..224663
FT                       /functional
FT                       /IMGT_allele="IGHV3-64*02"
FT                       /IMGT_gene="IGHV3-64"
FT   5'UTR               217736..221666
FT   L-V-GENE-UNIT       221667..222161
FT                       /functional
FT                       /IMGT_allele="IGHV3-64*02"
FT                       /IMGT_gene="IGHV3-64"
FT   L-INTRON-L          221667..221826
FT   L-PART1             221667..221712
FT                       /translation="MEFGLSWVFLVAIFK"
FT   INIT-CODON          221667..221669
FT   DONOR-SPLICE        221712..221714
FT   V-INTRON            221713..221815
FT   ACCEPTOR-SPLICE     221813..221817
FT   V-EXON              221816..222122
FT                       /codon_start=3
FT                       /translation="VQCEVQLVESGEGLVQPGGSLRLSCAASGFTFSSYAMHWVR
FT                       QAPGKGLEYVSAISSNGGSTYYADSVKGRFTISRDNSKNTLYLQMGSLRAEDMAV
FT                       YYCAR"
FT   L-PART2             221816..221826
FT                       /codon_start=3
FT                       /translation="VQC"
FT   V-REGION            221827..222122
FT                       /translation="EVQLVESGEGLVQPGGSLRLSCAASGFTFSSYAMHWVRQAP
FT                       GKGLEYVSAISSNGGSTYYADSVKGRFTISRDNSKNTLYLQMGSLRAEDMAVYYC
FT                       AR"
FT                       /CDR_length="[8.8.2]"
FT                       /FR_length="[25.17.38.G]"
FT                       /functional
FT                       /IMGT_allele="IGHV3-64*02"
FT                       /IMGT_gene="IGHV3-64"
FT   FR1-IMGT            221827..221901
FT                       /translation="EVQLVESGEGLVQPGGSLRLSCAAS"
FT                       /AA_IMGT="AA 1 to 26, AA 10 is missing"
FT   1st-CYS             221890..221892
FT   CDR1-IMGT           221902..221925
FT                       /translation="GFTFSSYA"
FT                       /AA_IMGT="AA 27 to 38, AA 31, 32, 33 and 34 are 
FT                       missing"
FT   FR2-IMGT            221926..221976
FT                       /translation="MHWVRQAPGKGLEYVSA"
FT                       /AA_IMGT="AA 39 to 55"
FT   CONSERVED-TRP       221932..221934
FT   CDR2-IMGT           221977..222000
FT                       /translation="ISSNGGST"
FT                       /AA_IMGT="AA 56 to 65, AA 60 and 61 are missing"
FT   FR3-IMGT            222001..222114
FT                       /translation="YYADSVKGRFTISRDNSKNTLYLQMGSLRAEDMAVYYC"
FT                       /AA_IMGT="AA 66 to 104, AA 73 is missing"
FT   3'V-REGION          222112..222122
FT                       /translation="CAR"
FT   2nd-CYS             222112..222114
FT   CDR3-IMGT           222115..222122
FT                       /translation="AR"
FT                       /AA_IMGT="AA 105 to 106"
FT   3'UTR               222123..224663
FT   V-RS                222123..222161
FT   V-HEPTAMER          222123..222129
FT   V-SPACER            222130..222152
FT   V-NONAMER           222153..222161
FT   V-GENE              224664..228180
FT                       /IMGT_allele="IGHV3-63*02"
FT                       /IMGT_gene="IGHV3-63"
FT                       /pseudo="several STOP-CODON in V-REGION"
FT   5'UTR               224664..227164
FT   L-V-GENE-UNIT       227165..227661
FT                       /IMGT_allele="IGHV3-63*02"
FT                       /IMGT_gene="IGHV3-63"
FT                       /pseudo="several STOP-CODON in V-REGION"
FT   L-INTRON-L          227165..227324
FT   L-PART1             227165..227210
FT                       /translation="MGFELTRIFLVAILK"
FT   INIT-CODON          227165..227167
FT   DONOR-SPLICE        227210..227212
FT   V-INTRON            227211..227313
FT   ACCEPTOR-SPLICE     227311..227315
FT   V-EXON              227314..227622
FT                       /codon_start=3
FT   L-PART2             227314..227324
FT                       /codon_start=3
FT                       /translation="VQC"
FT   V-REGION            227325..227622
FT                       /CDR_length="[8.8.3]"
FT                       /FR_length="[25.17.38.G]"
FT                       /IMGT_allele="IGHV3-63*02"
FT                       /IMGT_gene="IGHV3-63"
FT                       /pseudo="several STOP-CODON in V-REGION"
FT   FR1-IMGT            227325..227399
FT                       /translation="EVELIESIEGLRQLGKFLRLSCVAS"
FT                       /AA_IMGT="AA 1 to 26, AA 10 is missing"
FT   1st-CYS             227388..227390
FT   CDR1-IMGT           227400..227423
FT                       /AA_IMGT="AA 27 to 38, AA 31, 32, 33 and 34 are 
FT                       missing"
FT   FR2-IMGT            227424..227474
FT                       /translation="MSWVNETLGKGLEGVID"
FT                       /AA_IMGT="AA 39 to 55"
FT                       /IMGT_note="STOP-CODON in FR2-IMGT: position 39"
FT   CONSERVED-TRP       227430..227432
FT   CDR2-IMGT           227475..227498
FT                       /translation="VKYDGSQI"
FT                       /AA_IMGT="AA 56 to 65, AA 60 and 61 are missing"
FT   FR3-IMGT            227499..227612
FT                       /translation="YHADSVKGRFTISKDNAKNSPYLQTNSLRAEDMTMHGC"
FT                       /AA_IMGT="AA 66 to 104, AA 73 is missing"
FT   3'V-REGION          227610..227622
FT   2nd-CYS             227610..227612
FT   CDR3-IMGT           227613..227622
FT                       /AA_IMGT="AA 105 to 107"
FT                       /IMGT_note="STOP-CODON in CDR3-IMGT: position 106"
FT   3'UTR               227623..228180
FT   V-RS                227623..227661
FT   V-HEPTAMER          227623..227629
FT                       /IMGT_note="noncanonical V-HEPTAMER: ccaagtg instead 
FT                       of cacagtg"
FT   V-SPACER            227630..227652
FT   V-NONAMER           227653..227661
FT   V-GENE              228181..232814
FT                       /IMGT_allele="IGHV(II)-62-1*02"
FT                       /IMGT_gene="IGHV(II)-62-1"
FT                       /pseudo="truncated pseudogene not assigned to 
FT                       subgroups with functional genes"
FT   5'UTR               228181..229056
FT   V-REGION            <229057..229329
FT                       /partial
FT                       /CDR_length="[10.7.2]"
FT                       /FR_length="[16.X.X.G]"
FT                       /IMGT_allele="IGHV(II)-62-1*02"
FT                       /IMGT_gene="IGHV(II)-62-1"
FT                       /IMGT_note="partial gene in 5', restored translation 
FT                       in CDR2-IMGT, FR3-IMGT, 3'V-REGION and CDR3-IMGT"
FT                       /out_of_frame
FT                       /pseudo="truncated pseudogene not assigned to 
FT                       subgroups with functional genes"
FT                       /putative_limit="5'"
FT   FR1-IMGT            <229057..229104
FT                       /partial
FT                       /translation="GLVKPSQTLSLMCVIS"
FT                       /putative_limit="5'"
FT   1st-CYS             229093..229095
FT   CDR1-IMGT           229105..229134
FT                       /translation="AFSITTSASS"
FT                       /AA_IMGT="AA 27 to 38, AA 32 and 33 are missing"
FT   FR2-IMGT            229135..229187
FT                       /translation="WSCIHQPLSGREWSGLG"
FT                       /IMGT_note="frameshift in FR2-IMGT: insertion of 2 nt"
FT                       /out_of_frame
FT   CDR2-IMGT           229188..229208
FT                       /translation="IGHEGST"
FT                       /AA_IMGT="AA 56 to 65, AA 60, 61 and 62 are missing"
FT                       /out_of_frame
FT                       /restored_translation
FT   FR3-IMGT            229209..229321
FT                       /IMGT_note="frameshift in FR3-IMGT: deletion of 1 nt"
FT                       /out_of_frame
FT                       /restored_translation
FT   3'V-REGION          229319..229329
FT                       /out_of_frame
FT                       /restored_translation
FT   CDR3-IMGT           229322..229329
FT                       /translation="AK"
FT                       /AA_IMGT="AA 105 to 106"
FT                       /out_of_frame
FT                       /restored_translation
FT   3'UTR               229330..232814
FT   V-RS                229330..229368
FT   V-HEPTAMER          229330..229336
FT   V-SPACER            229337..229359
FT   V-NONAMER           229360..229368
FT   V-GENE              232815..238500
FT                       /IMGT_allele="IGHV3-62*03"
FT                       /IMGT_gene="IGHV3-62"
FT                       /pseudo="STOP-CODON in V-REGION: position 51"
FT   5'UTR               232815..236259
FT   L-V-GENE-UNIT       236260..236751
FT                       /IMGT_allele="IGHV3-62*03"
FT                       /IMGT_gene="IGHV3-62"
FT                       /pseudo="STOP-CODON in V-REGION: position 51"
FT   L-INTRON-L          236260..236419
FT   L-PART1             236260..236305
FT                       /translation="MEFGLSWVFLVAILR"
FT   INIT-CODON          236260..236262
FT   DONOR-SPLICE        236305..236307
FT   V-INTRON            236306..236408
FT   ACCEPTOR-SPLICE     236406..236410
FT   V-EXON              236409..236715
FT                       /codon_start=3
FT   L-PART2             236409..236419
FT                       /codon_start=3
FT                       /translation="VQC"
FT   V-REGION            236420..236715
FT                       /CDR_length="[8.8.2]"
FT                       /FR_length="[25.17.38.G]"
FT                       /IMGT_allele="IGHV3-62*03"
FT                       /IMGT_gene="IGHV3-62"
FT                       /pseudo="STOP-CODON in V-REGION: position 51"
FT   FR1-IMGT            236420..236494
FT                       /translation="EVQLVESGEGLVQPGGSLRLSCAAS"
FT                       /AA_IMGT="AA 1 to 26, AA 10 is missing"
FT   1st-CYS             236483..236485
FT   CDR1-IMGT           236495..236518
FT                       /translation="GFTFSSSA"
FT                       /AA_IMGT="AA 27 to 38, AA 31, 32, 33 and 34 are 
FT                       missing"
FT   FR2-IMGT            236519..236569
FT                       /AA_IMGT="AA 39 to 55"
FT                       /IMGT_note="STOP-CODON in FR2-IMGT: position 51"
FT   CONSERVED-TRP       236525..236527
FT   CDR2-IMGT           236570..236593
FT                       /translation="ISTSGDTV"
FT                       /AA_IMGT="AA 56 to 65, AA 60 and 61 are missing"
FT   FR3-IMGT            236594..236707
FT                       /translation="LYTDSVKGRFTISRDNAQNSLYLQMNSLRADDMAVYYC"
FT                       /AA_IMGT="AA 66 to 104, AA 73 is missing"
FT   3'V-REGION          236705..236715
FT                       /translation="CVK"
FT   2nd-CYS             236705..236707
FT   CDR3-IMGT           236708..236715
FT                       /translation="VK"
FT                       /AA_IMGT="AA 105 to 106"
FT   3'UTR               236716..238500
FT   V-RS                236716..236751
FT   V-HEPTAMER          236716..236722
FT   V-SPACER            236723..236742
FT   V-NONAMER           236743..236751
FT   V-GENE              238501..241318
FT                       /functional
FT                       /IMGT_allele="IGHV4-61*01"
FT                       /IMGT_gene="IGHV4-61"
FT   5'UTR               238501..240288
FT   L-V-GENE-UNIT       240289..240765
FT                       /functional
FT                       /IMGT_allele="IGHV4-61*01"
FT                       /IMGT_gene="IGHV4-61"
FT   L-INTRON-L          240289..240427
FT   L-PART1             240289..240334
FT                       /translation="MKHLWFFLLLVAAPR"
FT   INIT-CODON          240289..240291
FT   DONOR-SPLICE        240334..240336
FT   V-INTRON            240335..240416
FT   ACCEPTOR-SPLICE     240414..240418
FT   V-EXON              240417..240726
FT                       /codon_start=3
FT                       /translation="VLSQVQLQESGPGLVKPSETLSLTCTVSGGSVSSGSYYWSW
FT                       IRQPPGKGLEWIGYIYYSGSTNYNPSLKSRVTISVDTSKNQFSLKLSSVTAADTA
FT                       VYYCAR"
FT   L-PART2             240417..240427
FT                       /codon_start=3
FT                       /translation="VLS"
FT   V-REGION            240428..240726
FT                       /translation="QVQLQESGPGLVKPSETLSLTCTVSGGSVSSGSYYWSWIRQ
FT                       PPGKGLEWIGYIYYSGSTNYNPSLKSRVTISVDTSKNQFSLKLSSVTAADTAVYY
FT                       CAR"
FT                       /CDR_length="[10.7.2]"
FT                       /FR_length="[25.17.38.G]"
FT                       /functional
FT                       /IMGT_allele="IGHV4-61*01"
FT                       /IMGT_gene="IGHV4-61"
FT   FR1-IMGT            240428..240502
FT                       /translation="QVQLQESGPGLVKPSETLSLTCTVS"
FT                       /AA_IMGT="AA 1 to 26, AA 10 is missing"
FT   1st-CYS             240491..240493
FT   CDR1-IMGT           240503..240532
FT                       /translation="GGSVSSGSYY"
FT                       /AA_IMGT="AA 27 to 38, AA 32 and 33 are missing"
FT   FR2-IMGT            240533..240583
FT                       /translation="WSWIRQPPGKGLEWIGY"
FT                       /AA_IMGT="AA 39 to 55"
FT   CONSERVED-TRP       240539..240541
FT   CDR2-IMGT           240584..240604
FT                       /translation="IYYSGST"
FT                       /AA_IMGT="AA 56 to 65, AA 60, 61 and 62 are missing"
FT   FR3-IMGT            240605..240718
FT                       /translation="NYNPSLKSRVTISVDTSKNQFSLKLSSVTAADTAVYYC"
FT                       /AA_IMGT="AA 66 to 104, AA 73 is missing"
FT   3'V-REGION          240716..240726
FT                       /translation="CAR"
FT   2nd-CYS             240716..240718
FT   CDR3-IMGT           240719..240726
FT                       /translation="AR"
FT                       /AA_IMGT="AA 105 to 106"
FT   3'UTR               240727..241318
FT   V-RS                240727..240765
FT   V-HEPTAMER          240727..240733
FT   V-SPACER            240734..240756
FT   V-NONAMER           240757..240765
FT   V-GENE              241319..245249
FT                       /IMGT_allele="IGHV(II)-60-1*01"
FT                       /IMGT_gene="IGHV(II)-60-1"
FT                       /pseudo="truncated pseudogene not assigned to 
FT                       subgroups with functional genes"
FT   5'UTR               241319..241871
FT   V-REGION            <241872..242140
FT                       /partial
FT                       /CDR_length="[10.7.1]"
FT                       /FR_length="[16.17.38.G]"
FT                       /IMGT_allele="IGHV(II)-60-1*01"
FT                       /IMGT_gene="IGHV(II)-60-1"
FT                       /IMGT_note="partial gene in 5'"
FT                       /pseudo="truncated pseudogene not assigned to 
FT                       subgroups with functional genes"
FT                       /putative_limit="5'"
FT   FR1-IMGT            <241872..241919
FT                       /partial
FT                       /translation="GLVEPSQTLSLTCAVS"
FT                       /putative_limit="5'"
FT   1st-CYS             241908..241910
FT   CDR1-IMGT           241920..241949
FT                       /translation="GFSITTSVSC"
FT                       /AA_IMGT="AA 27 to 38, AA 32 and 33 are missing"
FT   FR2-IMGT            241950..242000
FT                       /translation="WSWIHESTWEGLEWTRR"
FT                       /AA_IMGT="AA 39 to 55"
FT   CONSERVED-TRP       241956..241958
FT   CDR2-IMGT           242001..242021
FT                       /translation="TRHEGSK"
FT                       /AA_IMGT="AA 56 to 65, AA 60, 61 and 62 are missing"
FT   FR3-IMGT            242022..242135
FT                       /AA_IMGT="AA 66 to 104, AA 73 is missing"
FT                       /IMGT_note="STOP-CODON in FR3-IMGT: position 103, no 
FT                       2nd-CYS: Arg instead of Cys"
FT   3'V-REGION          242133..242140
FT                       /translation="RR"
FT   CDR3-IMGT           242136..242140
FT                       /translation="R"
FT                       /AA_IMGT="AA 105"
FT   3'UTR               242141..245249
FT   V-RS                242141..242179
FT   V-HEPTAMER          242141..242147
FT   V-SPACER            242148..242170
FT   V-NONAMER           242171..242179
FT   V-GENE              245250..250000
FT                       /IMGT_allele="IGHV3-60*02"
FT                       /IMGT_gene="IGHV3-60"
FT                       /pseudo="frameshift in V-REGION: insertion of 1 nt in 
FT                       FR1-IMGT"
FT   5'UTR               245250..248191
FT   L-V-GENE-UNIT       248192..248684
FT                       /IMGT_allele="IGHV3-60*02"
FT                       /IMGT_gene="IGHV3-60"
FT                       /pseudo="frameshift in V-REGION: insertion of 1 nt in 
FT                       FR1-IMGT"
FT   L-INTRON-L          248192..248351
FT   L-PART1             248192..248237
FT                       /translation="MESGLSWIFLVAVLK"
FT   INIT-CODON          248192..248194
FT   DONOR-SPLICE        248237..248239
FT   V-INTRON            248238..248340
FT   ACCEPTOR-SPLICE     248338..248342
FT   V-EXON              248341..248648
FT                       /codon_start=3
FT                       /out_of_frame
FT   L-PART2             248341..248351
FT                       /codon_start=3
FT                       /translation="VQC"
FT   V-REGION            248352..248648
FT                       /CDR_length="[8.8.2]"
FT                       /FR_length="[X.17.38.G]"
FT                       /IMGT_allele="IGHV3-60*02"
FT                       /IMGT_gene="IGHV3-60"
FT                       /IMGT_note="restored translation in CDR1-IMGT, 
FT                       FR2-IMGT, CDR2-IMGT, FR3-IMGT, 3'V-REGION and 
FT                       CDR3-IMGT"
FT                       /out_of_frame
FT                       /pseudo="frameshift in V-REGION: insertion of 1 nt in 
FT                       FR1-IMGT"
FT   FR1-IMGT            248352..248427
FT                       /translation="EVQLVESGGGLVKTGGVSETLLCSL"
FT                       /IMGT_note="frameshift in FR1-IMGT: insertion of 1 nt"
FT                       /out_of_frame
FT   CDR1-IMGT           248428..248451
FT                       /translation="GFTFSSSA"
FT                       /AA_IMGT="AA 27 to 38, AA 31, 32, 33 and 34 are 
FT                       missing"
FT                       /out_of_frame
FT                       /restored_translation
FT   FR2-IMGT            248452..248502
FT                       /translation="MHWVHQAPGKGLEWVSV"
FT                       /AA_IMGT="AA 39 to 55"
FT                       /out_of_frame
FT                       /restored_translation
FT   CDR2-IMGT           248503..248526
FT                       /translation="ISTSGDTV"
FT                       /AA_IMGT="AA 56 to 65, AA 60 and 61 are missing"
FT                       /out_of_frame
FT                       /restored_translation
FT   FR3-IMGT            248527..248640
FT                       /AA_IMGT="AA 66 to 104, AA 73 is missing"
FT                       /out_of_frame
FT                       /restored_translation
FT   3'V-REGION          248638..248648
FT                       /translation="CVK"
FT                       /out_of_frame
FT                       /restored_translation
FT   CDR3-IMGT           248641..248648
FT                       /translation="VK"
FT                       /AA_IMGT="AA 105 to 106"
FT                       /out_of_frame
FT                       /restored_translation
FT   3'UTR               248649..250000
FT   V-RS                248649..248684
FT   V-HEPTAMER          248649..248655
FT   V-SPACER            248656..248675
FT   V-NONAMER           248676..248684
FT   V-GENE              250001..254843
FT                       /functional
FT                       /IMGT_allele="IGHV4-59*01"
FT                       /IMGT_gene="IGHV4-59"
FT   5'UTR               250001..252164
FT   L-V-GENE-UNIT       252165..252635
FT                       /functional
FT                       /IMGT_allele="IGHV4-59*01"
FT                       /IMGT_gene="IGHV4-59"
FT   L-INTRON-L          252165..252303
FT   L-PART1             252165..252210
FT                       /translation="MKHLWFFLLLVAAPR"
FT   INIT-CODON          252165..252167
FT   DONOR-SPLICE        252210..252212
FT   V-INTRON            252211..252292
FT   ACCEPTOR-SPLICE     252290..252294
FT   V-EXON              252293..252596
FT                       /codon_start=3
FT                       /translation="VLSQVQLQESGPGLVKPSETLSLTCTVSGGSISSYYWSWIR
FT                       QPPGKGLEWIGYIYYSGSTNYNPSLKSRVTISVDTSKNQFSLKLSSVTAADTAVY
FT                       YCAR"
FT   L-PART2             252293..252303
FT                       /codon_start=3
FT                       /translation="VLS"
FT   V-REGION            252304..252596
FT                       /translation="QVQLQESGPGLVKPSETLSLTCTVSGGSISSYYWSWIRQPP
FT                       GKGLEWIGYIYYSGSTNYNPSLKSRVTISVDTSKNQFSLKLSSVTAADTAVYYCA
FT                       R"
FT                       /CDR_length="[8.7.2]"
FT                       /FR_length="[25.17.38.G]"
FT                       /functional
FT                       /IMGT_allele="IGHV4-59*01"
FT                       /IMGT_gene="IGHV4-59"
FT   FR1-IMGT            252304..252378
FT                       /translation="QVQLQESGPGLVKPSETLSLTCTVS"
FT                       /AA_IMGT="AA 1 to 26, AA 10 is missing"
FT   1st-CYS             252367..252369
FT   CDR1-IMGT           252379..252402
FT                       /translation="GGSISSYY"
FT                       /AA_IMGT="AA 27 to 38, AA 31, 32, 33 and 34 are 
FT                       missing"
FT   FR2-IMGT            252403..252453
FT                       /translation="WSWIRQPPGKGLEWIGY"
FT                       /AA_IMGT="AA 39 to 55"
FT   CONSERVED-TRP       252409..252411
FT   CDR2-IMGT           252454..252474
FT                       /translation="IYYSGST"
FT                       /AA_IMGT="AA 56 to 65, AA 60, 61 and 62 are missing"
FT   FR3-IMGT            252475..252588
FT                       /translation="NYNPSLKSRVTISVDTSKNQFSLKLSSVTAADTAVYYC"
FT                       /AA_IMGT="AA 66 to 104, AA 73 is missing"
FT   3'V-REGION          252586..252596
FT                       /translation="CAR"
FT   2nd-CYS             252586..252588
FT   CDR3-IMGT           252589..252596
FT                       /translation="AR"
FT                       /AA_IMGT="AA 105 to 106"
FT   3'UTR               252597..254843
FT   V-RS                252597..252635
FT   V-HEPTAMER          252597..252603
FT   V-SPACER            252604..252626
FT   V-NONAMER           252627..252635
FT   V-GENE              254844..259012
FT                       /functional="noncanonical DONOR-SPLICE: nat instead of 
FT                       ngt, noncanonical V-NONAMER: tcagaaacg instead of 
FT                       acacaaacc, however this V-GENE has been seen 
FT                       rearranged in vivo"
FT                       /IMGT_allele="IGHV1-58*01"
FT                       /IMGT_gene="IGHV1-58"
FT   5'UTR               254844..257050
FT   L-V-GENE-UNIT       257051..257527
FT                       /functional="noncanonical DONOR-SPLICE: nat instead of 
FT                       ngt, noncanonical V-NONAMER: tcagaaacg instead of 
FT                       acacaaacc, however this V-GENE has been seen 
FT                       rearranged in vivo"
FT                       /IMGT_allele="IGHV1-58*01"
FT                       /IMGT_gene="IGHV1-58"
FT   L-INTRON-L          257051..257192
FT   L-PART1             257051..257096
FT                       /translation="MDWIWRILFLVGAAT"
FT   INIT-CODON          257051..257053
FT   DONOR-SPLICE        257096..257098
FT                       /IMGT_note="noncanonical DONOR-SPLICE: nat instead of 
FT                       ngt"
FT   V-INTRON            257097..257181
FT   ACCEPTOR-SPLICE     257179..257183
FT   V-EXON              257182..257488
FT                       /codon_start=3
FT                       /translation="AHSQMQLVQSGPEVKKPGTSVKVSCKASGFTFTSSAVQWVR
FT                       QARGQRLEWIGWIVVGSGNTNYAQKFQERVTITRDMSTSTAYMELSSLRSEDTAV
FT                       YYCAA"
FT   L-PART2             257182..257192
FT                       /codon_start=3
FT                       /translation="AHS"
FT   V-REGION            257193..257488
FT                       /translation="QMQLVQSGPEVKKPGTSVKVSCKASGFTFTSSAVQWVRQAR
FT                       GQRLEWIGWIVVGSGNTNYAQKFQERVTITRDMSTSTAYMELSSLRSEDTAVYYC
FT                       AA"
FT                       /CDR_length="[8.8.2]"
FT                       /FR_length="[25.17.38.G]"
FT                       /functional="noncanonical DONOR-SPLICE: nat instead of 
FT                       ngt, noncanonical V-NONAMER: tcagaaacg instead of 
FT                       acacaaacc, however this V-GENE has been seen 
FT                       rearranged in vivo"
FT                       /IMGT_allele="IGHV1-58*01"
FT                       /IMGT_gene="IGHV1-58"
FT   FR1-IMGT            257193..257267
FT                       /translation="QMQLVQSGPEVKKPGTSVKVSCKAS"
FT                       /AA_IMGT="AA 1 to 26, AA 10 is missing"
FT   1st-CYS             257256..257258
FT   CDR1-IMGT           257268..257291
FT                       /translation="GFTFTSSA"
FT                       /AA_IMGT="AA 27 to 38, AA 31, 32, 33 and 34 are 
FT                       missing"
FT   FR2-IMGT            257292..257342
FT                       /translation="VQWVRQARGQRLEWIGW"
FT                       /AA_IMGT="AA 39 to 55"
FT   CONSERVED-TRP       257298..257300
FT   CDR2-IMGT           257343..257366
FT                       /translation="IVVGSGNT"
FT                       /AA_IMGT="AA 56 to 65, AA 60 and 61 are missing"
FT   FR3-IMGT            257367..257480
FT                       /translation="NYAQKFQERVTITRDMSTSTAYMELSSLRSEDTAVYYC"
FT                       /AA_IMGT="AA 66 to 104, AA 73 is missing"
FT   3'V-REGION          257478..257488
FT                       /translation="CAA"
FT   2nd-CYS             257478..257480
FT   CDR3-IMGT           257481..257488
FT                       /translation="AA"
FT                       /AA_IMGT="AA 105 to 106"
FT   3'UTR               257489..259012
FT   V-RS                257489..257527
FT   V-HEPTAMER          257489..257495
FT   V-SPACER            257496..257518
FT   V-NONAMER           257519..257527
FT                       /IMGT_note="noncanonical V-NONAMER: tcagaaacg instead 
FT                       of acacaaacc"
FT   V-GENE              259013..265500
FT                       /IMGT_allele="IGHV3-57*02"
FT                       /IMGT_gene="IGHV3-57"
FT                       /pseudo="no L-PART1, frameshifts in V-REGION: several 
FT                       insertions and deletions"
FT   5'UTR               259013..260630
FT   ACCEPTOR-SPLICE     260628..260632
FT   V-EXON              260631..260936
FT                       /codon_start=3
FT                       /out_of_frame
FT   L-PART2             260631..260641
FT                       /codon_start=3
FT                       /translation="VLC"
FT   V-REGION            260642..260936
FT                       /IMGT_allele="IGHV3-57*02"
FT                       /IMGT_gene="IGHV3-57"
FT                       /out_of_frame
FT                       /pseudo="no L-PART1, frameshifts in V-REGION: several 
FT                       insertions and deletions"
FT   3'UTR               260937..265500
FT   V-RS                260937..260976
FT   V-HEPTAMER          260937..260943
FT                       /IMGT_note="noncanonical V-HEPTAMER: cacagga instead 
FT                       of cacagtg"
FT   V-SPACER            260944..260967
FT   V-NONAMER           260968..260976
FT   V-GENE              265501..271708
FT                       /IMGT_allele="IGHV7-56*02"
FT                       /IMGT_gene="IGHV7-56"
FT                       /pseudo="STOP-CODON in V-REGION: position 28, 
FT                       frameshifts in V-REGION: several insertions and 
FT                       deletions"
FT   5'UTR               265501..269648
FT   L-V-GENE-UNIT       269649..270122
FT                       /IMGT_allele="IGHV7-56*02"
FT                       /IMGT_gene="IGHV7-56"
FT                       /pseudo="STOP-CODON in V-REGION: position 28, 
FT                       frameshifts in V-REGION: several insertions and 
FT                       deletions"
FT   L-INTRON-L          269649..269789
FT   L-PART1             269649..269694
FT                       /translation="MDWTWSILFLVAAAT"
FT   INIT-CODON          269649..269651
FT   DONOR-SPLICE        269694..269696
FT   V-INTRON            269695..269778
FT   ACCEPTOR-SPLICE     269776..269780
FT   V-EXON              269779..270083
FT                       /codon_start=3
FT                       /out_of_frame
FT   L-PART2             269779..269789
FT                       /codon_start=3
FT                       /translation="AHS"
FT   V-REGION            269790..270083
FT                       /CDR_length="[8.X.2]"
FT                       /FR_length="[25.17.X.G]"
FT                       /IMGT_allele="IGHV7-56*02"
FT                       /IMGT_gene="IGHV7-56"
FT                       /IMGT_note="restored translation in FR3-IMGT, 
FT                       3'V-REGION and CDR3-IMGT"
FT                       /out_of_frame
FT                       /pseudo="STOP-CODON in V-REGION: position 28, 
FT                       frameshifts in V-REGION: several insertions and 
FT                       deletions"
FT   FR1-IMGT            269790..269864
FT                       /translation="RVQLVQSGPEVKQPGASAKVSCKVS"
FT                       /AA_IMGT="AA 1 to 26, AA 10 is missing"
FT   1st-CYS             269853..269855
FT   CDR1-IMGT           269865..269888
FT                       /AA_IMGT="AA 27 to 38, AA 31, 32, 33 and 34 are 
FT                       missing"
FT                       /IMGT_note="STOP-CODON in CDR1-IMGT: position 28"
FT   FR2-IMGT            269889..269939
FT                       /translation="MNWIRQTPGQGLEWMGW"
FT                       /AA_IMGT="AA 39 to 55"
FT   CONSERVED-TRP       269895..269897
FT   CDR2-IMGT           269940..269962
FT                       /IMGT_note="frameshifts in CDR2-IMGT: deletion of 2 nt 
FT                       and insertion of 1 nt"
FT                       /out_of_frame
FT   FR3-IMGT            269963..270075
FT                       /IMGT_note="frameshift in FR3-IMGT: deletion of 1 nt"
FT                       /out_of_frame
FT                       /restored_translation
FT   3'V-REGION          270073..270083
FT                       /translation="CVR"
FT                       /out_of_frame
FT                       /restored_translation
FT   CDR3-IMGT           270076..270083
FT                       /translation="VR"
FT                       /AA_IMGT="AA 105 to 106"
FT                       /out_of_frame
FT                       /restored_translation
FT   3'UTR               270084..271708
FT   V-RS                270084..270122
FT   V-HEPTAMER          270084..270090
FT   V-SPACER            270091..270113
FT   V-NONAMER           270114..270122
FT                       /IMGT_note="noncanonical V-NONAMER: ttagaaacc instead 
FT                       of acacaaacc"
FT   V-GENE              271709..275910
FT                       /IMGT_allele="IGHV4-55*01"
FT                       /IMGT_gene="IGHV4-55"
FT                       /pseudo="STOP-CODON in V-REGION: position 39"
FT   5'UTR               271709..273293
FT   L-V-GENE-UNIT       273294..273768
FT                       /IMGT_allele="IGHV4-55*01"
FT                       /IMGT_gene="IGHV4-55"
FT                       /pseudo="STOP-CODON in V-REGION: position 39"
FT   L-INTRON-L          273294..273433
FT   L-PART1             273294..273339
FT                       /translation="MKHLWFFLLLVAAPR"
FT   INIT-CODON          273294..273296
FT   DONOR-SPLICE        273339..273341
FT   V-INTRON            273340..273422
FT   ACCEPTOR-SPLICE     273420..273424
FT   V-EXON              273423..273729
FT                       /codon_start=3
FT   L-PART2             273423..273433
FT                       /codon_start=3
FT                       /translation="VLS"
FT   V-REGION            273434..273729
FT                       /CDR_length="[9.7.2]"
FT                       /FR_length="[25.17.38.G]"
FT                       /IMGT_allele="IGHV4-55*01"
FT                       /IMGT_gene="IGHV4-55"
FT                       /pseudo="STOP-CODON in V-REGION: position 39"
FT   FR1-IMGT            273434..273508
FT                       /translation="QVQLQESGPGLVKPSETLSLICAVS"
FT                       /AA_IMGT="AA 1 to 26, AA 10 is missing"
FT   1st-CYS             273497..273499
FT   CDR1-IMGT           273509..273535
FT                       /translation="GDSISSGNW"
FT                       /AA_IMGT="AA 27 to 38, AA 32, 33 and 34 are missing"
FT   FR2-IMGT            273536..273586
FT                       /AA_IMGT="AA 39 to 55"
FT                       /IMGT_note="STOP-CODON in FR2-IMGT: position 39"
FT   CONSERVED-TRP       273542..273544
FT   CDR2-IMGT           273587..273607
FT                       /translation="IHHSGST"
FT                       /AA_IMGT="AA 56 to 65, AA 60, 61 and 62 are missing"
FT   FR3-IMGT            273608..273721
FT                       /translation="YYNPSLKSRITMSVDTSKNQFYLKLSSVTAADTAVYYC"
FT                       /AA_IMGT="AA 66 to 104, AA 73 is missing"
FT   3'V-REGION          273719..273729
FT                       /translation="CAR"
FT   2nd-CYS             273719..273721
FT   CDR3-IMGT           273722..273729
FT                       /translation="AR"
FT                       /AA_IMGT="AA 105 to 106"
FT   3'UTR               273730..275910
FT   V-RS                273730..273768
FT   V-HEPTAMER          273730..273736
FT   V-SPACER            273737..273759
FT   V-NONAMER           273760..273768
FT   V-GENE              275911..279202
FT                       /IMGT_allele="IGHV3-54*03"
FT                       /IMGT_gene="IGHV3-54"
FT                       /pseudo="frameshift in V-REGION: insertion of 8 nt in 
FT                       FR1-IMGT"
FT   5'UTR               275911..278052
FT   L-V-GENE-UNIT       278053..278548
FT                       /IMGT_allele="IGHV3-54*03"
FT                       /IMGT_gene="IGHV3-54"
FT                       /pseudo="frameshift in V-REGION: insertion of 8 nt in 
FT                       FR1-IMGT"
FT   L-INTRON-L          278053..278203
FT   L-PART1             278053..278098
FT                       /translation="MELGLSWVFTVAVLK"
FT   INIT-CODON          278053..278055
FT   DONOR-SPLICE        278098..278100
FT   V-INTRON            278099..278192
FT   ACCEPTOR-SPLICE     278190..278194
FT   V-EXON              278193..278507
FT                       /codon_start=3
FT                       /out_of_frame
FT   L-PART2             278193..278203
FT                       /codon_start=3
FT                       /translation="VQC"
FT   V-REGION            278204..278507
FT                       /CDR_length="[8.8.2]"
FT                       /FR_length="[X.17.38.G]"
FT                       /IMGT_allele="IGHV3-54*03"
FT                       /IMGT_gene="IGHV3-54"
FT                       /IMGT_note="restored translation in CDR1-IMGT, 
FT                       FR2-IMGT, CDR2-IMGT, FR3-IMGT, 3'V-REGION and 
FT                       CDR3-IMGT"
FT                       /out_of_frame
FT                       /pseudo="frameshift in V-REGION: insertion of 8 nt in 
FT                       FR1-IMGT"
FT   FR1-IMGT            278204..278286
FT                       /IMGT_note="frameshift in FR1-IMGT: insertion of 8 nt"
FT                       /out_of_frame
FT   CDR1-IMGT           278287..278310
FT                       /AA_IMGT="AA 27 to 38, AA 31, 32, 33 and 34 are 
FT                       missing"
FT                       /out_of_frame
FT                       /restored_translation
FT   FR2-IMGT            278311..278361
FT                       /AA_IMGT="AA 39 to 55"
FT                       /out_of_frame
FT                       /restored_translation
FT   CDR2-IMGT           278362..278385
FT                       /AA_IMGT="AA 56 to 65, AA 60 and 61 are missing"
FT                       /out_of_frame
FT                       /restored_translation
FT   FR3-IMGT            278386..278499
FT                       /translation="CYAQSVKSRFTISKENAKNSLCLQMNSLRAEGTAVCYC"
FT                       /AA_IMGT="AA 66 to 104, AA 73 is missing"
FT                       /out_of_frame
FT                       /restored_translation
FT   3'V-REGION          278497..278507
FT                       /out_of_frame
FT                       /restored_translation
FT   CDR3-IMGT           278500..278507
FT                       /AA_IMGT="AA 105 to 106"
FT                       /out_of_frame
FT                       /restored_translation
FT   3'UTR               278508..279202
FT   V-RS                278508..278548
FT   V-HEPTAMER          278508..278514
FT                       /IMGT_note="noncanonical V-HEPTAMER: caccagg instead 
FT                       of cacagtg"
FT   V-SPACER            278515..278539
FT   V-NONAMER           278540..278548
FT                       /IMGT_note="noncanonical V-NONAMER: acacagaat instead 
FT                       of acacaaacc"
FT   V-GENE              279203..283474
FT                       /IMGT_allele="IGHV(II)-53-1*02"
FT                       /IMGT_gene="IGHV(II)-53-1"
FT                       /pseudo="truncated pseudogene not assigned to 
FT                       subgroups with functional genes"
FT   5'UTR               279203..279879
FT   ACCEPTOR-SPLICE     279877..279881
FT   V-EXON              279880..280190
FT                       /codon_start=3
FT   L-PART2             279880..279893
FT                       /codon_start=3
FT                       /translation="PVHA"
FT   V-REGION            279894..280190
FT                       /CDR_length="[10.X.2]"
FT                       /FR_length="[25.17.38.G]"
FT                       /IMGT_allele="IGHV(II)-53-1*02"
FT                       /IMGT_gene="IGHV(II)-53-1"
FT                       /IMGT_note="restored translation in FR3-IMGT, 
FT                       3'V-REGION and CDR3-IMGT"
FT                       /out_of_frame
FT                       /pseudo="truncated pseudogene not assigned to 
FT                       subgroups with functional genes"
FT   FR1-IMGT            279894..279968
FT                       /translation="NNCVSQCPLGLVKPSQTLSLTCAVS"
FT                       /AA_IMGT="AA 1 to 26, AA 10 is missing"
FT   1st-CYS             279957..279959
FT   CDR1-IMGT           279969..279998
FT                       /translation="GFPITTSASC"
FT                       /AA_IMGT="AA 27 to 38, AA 32 and 33 are missing"
FT   FR2-IMGT            279999..280049
FT                       /AA_IMGT="AA 39 to 55"
FT                       /IMGT_note="no CONSERVED-TRP: Cys instead of Trp, 
FT                       STOP-CODON in FR2-IMGT: position 52"
FT   CDR2-IMGT           280050..280069
FT                       /translation="TGHEGV"
FT                       /IMGT_note="frameshift in CDR2-IMGT: deletion of 1 nt"
FT                       /out_of_frame
FT   FR3-IMGT            280070..280183
FT                       /AA_IMGT="AA 66 to 104, AA 73 is missing"
FT                       /out_of_frame
FT                       /restored_translation
FT   3'V-REGION          280181..280190
FT                       /out_of_frame
FT                       /restored_translation
FT   CDR3-IMGT           280184..280190
FT                       /translation="QK"
FT                       /AA_IMGT="AA 105 to 106"
FT                       /out_of_frame
FT                       /restored_translation
FT   3'UTR               280191..283474
FT   V-RS                280191..280229
FT   V-HEPTAMER          280191..280197
FT   V-SPACER            280198..280220
FT   V-NONAMER           280221..280229
FT   V-GENE              283475..290113
FT                       /functional
FT                       /IMGT_allele="IGHV3-53*02"
FT                       /IMGT_gene="IGHV3-53"
FT   5'UTR               283475..286718
FT   L-V-GENE-UNIT       286719..287208
FT                       /functional
FT                       /IMGT_allele="IGHV3-53*02"
FT                       /IMGT_gene="IGHV3-53"
FT   L-INTRON-L          286719..286876
FT   L-PART1             286719..286764
FT                       /translation="MEFWLSWVFLVAISK"
FT   INIT-CODON          286719..286721
FT   DONOR-SPLICE        286764..286766
FT   V-INTRON            286765..286865
FT   ACCEPTOR-SPLICE     286863..286867
FT   V-EXON              286866..287169
FT                       /codon_start=3
FT                       /translation="VQCEVQLVETGGGLIQPGGSLRLSCAASGFTVSSNYMSWVR
FT                       QAPGKGLEWVSVIYSGGSTYYADSVKGRFTISRDNSKNTLYLQMNSLRAEDTAVY
FT                       YCAR"
FT   L-PART2             286866..286876
FT                       /codon_start=3
FT                       /translation="VQC"
FT   V-REGION            286877..287169
FT                       /translation="EVQLVETGGGLIQPGGSLRLSCAASGFTVSSNYMSWVRQAP
FT                       GKGLEWVSVIYSGGSTYYADSVKGRFTISRDNSKNTLYLQMNSLRAEDTAVYYCA
FT                       R"
FT                       /CDR_length="[8.7.2]"
FT                       /FR_length="[25.17.38.G]"
FT                       /functional
FT                       /IMGT_allele="IGHV3-53*02"
FT                       /IMGT_gene="IGHV3-53"
FT   FR1-IMGT            286877..286951
FT                       /translation="EVQLVETGGGLIQPGGSLRLSCAAS"
FT                       /AA_IMGT="AA 1 to 26, AA 10 is missing"
FT   1st-CYS             286940..286942
FT   CDR1-IMGT           286952..286975
FT                       /translation="GFTVSSNY"
FT                       /AA_IMGT="AA 27 to 38, AA 31, 32, 33 and 34 are 
FT                       missing"
FT   FR2-IMGT            286976..287026
FT                       /translation="MSWVRQAPGKGLEWVSV"
FT                       /AA_IMGT="AA 39 to 55"
FT   CONSERVED-TRP       286982..286984
FT   CDR2-IMGT           287027..287047
FT                       /translation="IYSGGST"
FT                       /AA_IMGT="AA 56 to 65, AA 60, 61 and 62 are missing"
FT   FR3-IMGT            287048..287161
FT                       /translation="YYADSVKGRFTISRDNSKNTLYLQMNSLRAEDTAVYYC"
FT                       /AA_IMGT="AA 66 to 104, AA 73 is missing"
FT   3'V-REGION          287159..287169
FT                       /translation="CAR"
FT   2nd-CYS             287159..287161
FT   CDR3-IMGT           287162..287169
FT                       /translation="AR"
FT                       /AA_IMGT="AA 105 to 106"
FT   3'UTR               287170..290113
FT   V-RS                287170..287208
FT   V-HEPTAMER          287170..287176
FT   V-SPACER            287177..287199
FT   V-NONAMER           287200..287208
FT   V-GENE              290114..294161
FT                       /IMGT_allele="IGHV3-52*01"
FT                       /IMGT_gene="IGHV3-52"
FT                       /pseudo="STOP-CODON in V-REGION: position 9"
FT   5'UTR               290114..293018
FT   L-V-GENE-UNIT       293019..293508
FT                       /IMGT_allele="IGHV3-52*01"
FT                       /IMGT_gene="IGHV3-52"
FT                       /pseudo="STOP-CODON in V-REGION: position 9"
FT   L-INTRON-L          293019..293173
FT   L-PART1             293019..293064
FT                       /translation="MESGLSWVFLVAILK"
FT   INIT-CODON          293019..293021
FT   DONOR-SPLICE        293064..293066
FT   V-INTRON            293065..293162
FT   ACCEPTOR-SPLICE     293160..293164
FT   V-EXON              293163..293469
FT                       /codon_start=3
FT   L-PART2             293163..293173
FT                       /codon_start=3
FT                       /translation="VQC"
FT   V-REGION            293174..293469
FT                       /CDR_length="[8.8.2]"
FT                       /FR_length="[25.17.38.G]"
FT                       /IMGT_allele="IGHV3-52*01"
FT                       /IMGT_gene="IGHV3-52"
FT                       /pseudo="STOP-CODON in V-REGION: position 9"
FT   FR1-IMGT            293174..293248
FT                       /AA_IMGT="AA 1 to 26, AA 10 is missing"
FT                       /IMGT_note="STOP-CODON in FR1-IMGT: position 9"
FT   1st-CYS             293237..293239
FT   CDR1-IMGT           293249..293272
FT                       /translation="GFTFSSSW"
FT                       /AA_IMGT="AA 27 to 38, AA 31, 32, 33 and 34 are 
FT                       missing"
FT   FR2-IMGT            293273..293323
FT                       /translation="MHWVCQAPEKGLEWVAD"
FT                       /AA_IMGT="AA 39 to 55"
FT   CONSERVED-TRP       293279..293281
FT   CDR2-IMGT           293324..293347
FT                       /translation="IKCDGSEK"
FT                       /AA_IMGT="AA 56 to 65, AA 60 and 61 are missing"
FT   FR3-IMGT            293348..293461
FT                       /translation="YYVDSVKGRLTISRDNAKNSLYLQVNSLRAEDMTVYYC"
FT                       /AA_IMGT="AA 66 to 104, AA 73 is missing"
FT   3'V-REGION          293459..293469
FT                       /translation="CVR"
FT   2nd-CYS             293459..293461
FT   CDR3-IMGT           293462..293469
FT                       /translation="VR"
FT                       /AA_IMGT="AA 105 to 106"
FT   3'UTR               293470..294161
FT   V-RS                293470..293508
FT   V-HEPTAMER          293470..293476
FT   V-SPACER            293477..293499
FT   V-NONAMER           293500..293508
FT   V-GENE              294162..295455
FT                       /IMGT_allele="IGHV(II)-51-2*01"
FT                       /IMGT_gene="IGHV(II)-51-2"
FT                       /pseudo="truncated pseudogene not assigned to 
FT                       subgroups with functional genes"
FT   5'UTR               294162..294868
FT   V-REGION            <294869..295131
FT                       /partial
FT                       /CDR_length="[10.6.2]"
FT                       /FR_length="[16.X.38.G]"
FT                       /IMGT_allele="IGHV(II)-51-2*01"
FT                       /IMGT_gene="IGHV(II)-51-2"
FT                       /IMGT_note="partial gene in 5', restored translation 
FT                       in CDR2-IMGT, FR3-IMGT, 3'V-REGION and CDR3-IMGT"
FT                       /out_of_frame
FT                       /pseudo="truncated pseudogene not assigned to 
FT                       subgroups with functional genes"
FT                       /putative_limit="5'"
FT   FR1-IMGT            <294869..294916
FT                       /partial
FT                       /translation="GLVRPSQTPSLTCAVS"
FT                       /putative_limit="5'"
FT   1st-CYS             294905..294907
FT   CDR1-IMGT           294917..294946
FT                       /translation="GFSITTSASC"
FT                       /AA_IMGT="AA 27 to 38, AA 32 and 33 are missing"
FT   FR2-IMGT            294947..294993
FT                       /translation="WSWIRQSSVKGVDQG"
FT                       /IMGT_note="frameshift in FR2-IMGT: deletion of 4 nt"
FT                       /out_of_frame
FT   CDR2-IMGT           294994..295011
FT                       /translation="RSRGST"
FT                       /AA_IMGT="AA 56 to 65, AA 59, 60, 61 and 62 are 
FT                       missing"
FT                       /out_of_frame
FT                       /restored_translation
FT   FR3-IMGT            295012..295125
FT                       /AA_IMGT="AA 66 to 104, AA 73 is missing"
FT                       /out_of_frame
FT                       /restored_translation
FT   3'V-REGION          295123..295131
FT                       /translation="CER"
FT                       /out_of_frame
FT                       /restored_translation
FT   CDR3-IMGT           295126..295131
FT                       /translation="ER"
FT                       /AA_IMGT="AA 105 to 106"
FT                       /out_of_frame
FT                       /restored_translation
FT   3'UTR               295132..295455
FT   V-RS                295132..295168
FT   V-HEPTAMER          295132..295138
FT                       /IMGT_note="noncanonical V-HEPTAMER: aacagaa instead 
FT                       of cacagtg"
FT   V-SPACER            295139..295159
FT   V-NONAMER           295160..295168
FT   V-GENE              295456..298472
FT                       /IMGT_allele="IGHV8-51-1*02"
FT                       /IMGT_gene="IGHV8-51-1"
FT                       /ORF="noncanonical V-HEPTAMER: catcgtg instead of 
FT                       cacagtg, noncanonical V-NONAMER: agacagact instead of 
FT                       acacaaacc"
FT   5'UTR               295456..295741
FT   L-V-GENE-UNIT       295742..296383
FT                       /IMGT_allele="IGHV8-51-1*02"
FT                       /IMGT_gene="IGHV8-51-1"
FT                       /ORF="noncanonical V-HEPTAMER: catcgtg instead of 
FT                       cacagtg, noncanonical V-NONAMER: agacagact instead of 
FT                       acacaaacc"
FT   L-INTRON-L          295742..296048
FT   L-PART1             295742..295781
FT                       /translation="MLVCVLLYSFRLF"
FT   INIT-CODON          295742..295744
FT   DONOR-SPLICE        295781..295783
FT   V-INTRON            295782..296037
FT   ACCEPTOR-SPLICE     296035..296039
FT   V-EXON              296038..296344
FT                       /codon_start=3
FT                       /translation="IQGEAQLTESGGDLVHLEGPLRLSCAASWFTFSIYEIHWVC
FT                       QASGKGLEWVAVIWRGESHQYNADYVRGRLTTSRDNTKYMLYMQMISLRTQNMAA
FT                       FNCAG"
FT   L-PART2             296038..296048
FT                       /codon_start=3
FT                       /translation="IQG"
FT   V-REGION            296049..296344
FT                       /translation="EAQLTESGGDLVHLEGPLRLSCAASWFTFSIYEIHWVCQAS
FT                       GKGLEWVAVIWRGESHQYNADYVRGRLTTSRDNTKYMLYMQMISLRTQNMAAFNC
FT                       AG"
FT                       /CDR_length="[8.8.2]"
FT                       /FR_length="[25.17.38.G]"
FT                       /IMGT_allele="IGHV8-51-1*02"
FT                       /IMGT_gene="IGHV8-51-1"
FT                       /ORF="noncanonical V-HEPTAMER: catcgtg instead of 
FT                       cacagtg, noncanonical V-NONAMER: agacagact instead of 
FT                       acacaaacc"
FT   FR1-IMGT            296049..296123
FT                       /translation="EAQLTESGGDLVHLEGPLRLSCAAS"
FT                       /AA_IMGT="AA 1 to 26, AA 10 is missing"
FT   1st-CYS             296112..296114
FT   CDR1-IMGT           296124..296147
FT                       /translation="WFTFSIYE"
FT                       /AA_IMGT="AA 27 to 38, AA 31, 32, 33 and 34 are 
FT                       missing"
FT   FR2-IMGT            296148..296198
FT                       /translation="IHWVCQASGKGLEWVAV"
FT                       /AA_IMGT="AA 39 to 55"
FT   CONSERVED-TRP       296154..296156
FT   CDR2-IMGT           296199..296222
FT                       /translation="IWRGESHQ"
FT                       /AA_IMGT="AA 56 to 65, AA 60 and 61 are missing"
FT   FR3-IMGT            296223..296336
FT                       /translation="YNADYVRGRLTTSRDNTKYMLYMQMISLRTQNMAAFNC"
FT                       /AA_IMGT="AA 66 to 104, AA 73 is missing"
FT   3'V-REGION          296334..296344
FT                       /translation="CAG"
FT   2nd-CYS             296334..296336
FT   CDR3-IMGT           296337..296344
FT                       /translation="AG"
FT                       /AA_IMGT="AA 105 to 106"
FT   3'UTR               296345..298472
FT   V-RS                296345..296383
FT   V-HEPTAMER          296345..296351
FT                       /IMGT_note="noncanonical V-HEPTAMER: catcgtg instead 
FT                       of cacagtg"
FT   V-SPACER            296352..296374
FT   V-NONAMER           296375..296383
FT                       /IMGT_note="noncanonical V-NONAMER: agacagact instead 
FT                       of acacaaacc"
FT   V-GENE              298473..307000
FT                       /functional
FT                       /IMGT_allele="IGHV5-51*01"
FT                       /IMGT_gene="IGHV5-51"
FT   5'UTR               298473..300667
FT   L-V-GENE-UNIT       300668..301141
FT                       /functional
FT                       /IMGT_allele="IGHV5-51*01"
FT                       /IMGT_gene="IGHV5-51"
FT   L-INTRON-L          300668..300807
FT   L-PART1             300668..300713
FT                       /translation="MGSTAILALLLAVLQ"
FT   INIT-CODON          300668..300670
FT   DONOR-SPLICE        300713..300715
FT   V-INTRON            300714..300796
FT   ACCEPTOR-SPLICE     300794..300798
FT   V-EXON              300797..301103
FT                       /codon_start=3
FT                       /translation="VCAEVQLVQSGAEVKKPGESLKISCKGSGYSFTSYWIGWVR
FT                       QMPGKGLEWMGIIYPGDSDTRYSPSFQGQVTISADKSISTAYLQWSSLKASDTAM
FT                       YYCAR"
FT   L-PART2             300797..300807
FT                       /codon_start=3
FT                       /translation="VCA"
FT   V-REGION            300808..301103
FT                       /translation="EVQLVQSGAEVKKPGESLKISCKGSGYSFTSYWIGWVRQMP
FT                       GKGLEWMGIIYPGDSDTRYSPSFQGQVTISADKSISTAYLQWSSLKASDTAMYYC
FT                       AR"
FT                       /CDR_length="[8.8.2]"
FT                       /FR_length="[25.17.38.G]"
FT                       /functional
FT                       /IMGT_allele="IGHV5-51*01"
FT                       /IMGT_gene="IGHV5-51"
FT   FR1-IMGT            300808..300882
FT                       /translation="EVQLVQSGAEVKKPGESLKISCKGS"
FT                       /AA_IMGT="AA 1 to 26, AA 10 is missing"
FT   1st-CYS             300871..300873
FT   CDR1-IMGT           300883..300906
FT                       /translation="GYSFTSYW"
FT                       /AA_IMGT="AA 27 to 38, AA 31, 32, 33 and 34 are 
FT                       missing"
FT   FR2-IMGT            300907..300957
FT                       /translation="IGWVRQMPGKGLEWMGI"
FT                       /AA_IMGT="AA 39 to 55"
FT   CONSERVED-TRP       300913..300915
FT   CDR2-IMGT           300958..300981
FT                       /translation="IYPGDSDT"
FT                       /AA_IMGT="AA 56 to 65, AA 60 and 61 are missing"
FT   FR3-IMGT            300982..301095
FT                       /translation="RYSPSFQGQVTISADKSISTAYLQWSSLKASDTAMYYC"
FT                       /AA_IMGT="AA 66 to 104, AA 73 is missing"
FT   3'V-REGION          301093..301103
FT                       /translation="CAR"
FT   2nd-CYS             301093..301095
FT   CDR3-IMGT           301096..301103
FT                       /translation="AR"
FT                       /AA_IMGT="AA 105 to 106"
FT   3'UTR               301104..307000
FT   V-RS                301104..301141
FT   V-HEPTAMER          301104..301110
FT   V-SPACER            301111..301132
FT   V-NONAMER           301133..301141
FT   V-GENE              307001..314664
FT                       /IMGT_allele="IGHV3-50*01"
FT                       /IMGT_gene="IGHV3-50"
FT                       /pseudo="no INIT-CODON: Ile instead of Met, no 
FT                       DONOR-SPLICE, STOP-CODON in L-PART2, frameshift in 
FT                       V-REGION: deletion of 1 nt in FR1-IMGT"
FT   5'UTR               307001..313289
FT   L-V-GENE-UNIT       313290..313784
FT                       /IMGT_allele="IGHV3-50*01"
FT                       /IMGT_gene="IGHV3-50"
FT                       /pseudo="no INIT-CODON: Ile instead of Met, no 
FT                       DONOR-SPLICE, STOP-CODON in L-PART2, frameshift in 
FT                       V-REGION: deletion of 1 nt in FR1-IMGT"
FT   L-INTRON-L          313290..313448
FT   L-PART1             313290..313335
FT                       /translation="IELGLSWVSLVIILK"
FT                       /IMGT_note="no INIT-CODON: Ile instead of Met, no 
FT                       DONOR-SPLICE"
FT   V-INTRON            313336..313437
FT   ACCEPTOR-SPLICE     313435..313439
FT   V-EXON              313438..313745
FT                       /codon_start=3
FT                       /out_of_frame
FT   L-PART2             313438..313448
FT                       /codon_start=3
FT                       /IMGT_note="STOP-CODON in L-PART2"
FT   V-REGION            313449..313745
FT                       /CDR_length="[8.8.3]"
FT                       /FR_length="[X.17.38.G]"
FT                       /IMGT_allele="IGHV3-50*01"
FT                       /IMGT_gene="IGHV3-50"
FT                       /IMGT_note="restored translation in CDR1-IMGT, 
FT                       FR2-IMGT, CDR2-IMGT, FR3-IMGT, 3'V-REGION and 
FT                       CDR3-IMGT"
FT                       /out_of_frame
FT                       /pseudo="no INIT-CODON: Ile instead of Met, no 
FT                       DONOR-SPLICE, STOP-CODON in L-PART2, frameshift in 
FT                       V-REGION: deletion of 1 nt in FR1-IMGT"
FT   FR1-IMGT            313449..313522
FT                       /IMGT_note="frameshift in FR1-IMGT: deletion of 1 nt"
FT                       /out_of_frame
FT   CDR1-IMGT           313523..313546
FT                       /AA_IMGT="AA 27 to 38, AA 31, 32, 33 and 34 are 
FT                       missing"
FT                       /out_of_frame
FT                       /restored_translation
FT   FR2-IMGT            313547..313597
FT                       /AA_IMGT="AA 39 to 55"
FT                       /out_of_frame
FT                       /restored_translation
FT   CDR2-IMGT           313598..313621
FT                       /translation="IMYDGSQT"
FT                       /AA_IMGT="AA 56 to 65, AA 60 and 61 are missing"
FT                       /out_of_frame
FT                       /restored_translation
FT   FR3-IMGT            313622..313735
FT                       /AA_IMGT="AA 66 to 104, AA 73 is missing"
FT                       /out_of_frame
FT                       /restored_translation
FT   3'V-REGION          313733..313745
FT                       /out_of_frame
FT                       /restored_translation
FT   CDR3-IMGT           313736..313745
FT                       /translation="VKG"
FT                       /AA_IMGT="AA 105 to 107"
FT                       /out_of_frame
FT                       /restored_translation
FT   3'UTR               313746..314664
FT   V-RS                313746..313784
FT   V-HEPTAMER          313746..313752
FT                       /IMGT_note="noncanonical V-HEPTAMER: ccaaatg instead 
FT                       of cacagtg"
FT   V-SPACER            313753..313775
FT   V-NONAMER           313776..313784
FT   V-GENE              314665..319000
FT                       /IMGT_allele="IGHV(II)-49-1*01"
FT                       /IMGT_gene="IGHV(II)-49-1"
FT                       /pseudo="truncated pseudogene not assigned to 
FT                       subgroups with functional genes"
FT   5'UTR               314665..315246
FT   V-REGION            <315247..315517
FT                       /partial
FT                       /CDR_length="[X.X.2]"
FT                       /FR_length="[16.X.38.G]"
FT                       /IMGT_allele="IGHV(II)-49-1*01"
FT                       /IMGT_gene="IGHV(II)-49-1"
FT                       /IMGT_note="partial gene in 5', restored translation 
FT                       in FR2-IMGT, FR3-IMGT, 3'V-REGION and CDR3-IMGT"
FT                       /out_of_frame
FT                       /pseudo="truncated pseudogene not assigned to 
FT                       subgroups with functional genes"
FT                       /putative_limit="5'"
FT   FR1-IMGT            <315247..315294
FT                       /partial
FT                       /translation="GLAKPSQTLSLICAVS"
FT                       /putative_limit="5'"
FT   1st-CYS             315283..315285
FT   CDR1-IMGT           315295..315323
FT                       /translation="ASPSQPVLP"
FT                       /IMGT_note="frameshift in CDR1-IMGT: deletion of 1 nt"
FT                       /out_of_frame
FT   FR2-IMGT            315324..315375
FT                       /translation="WSWIPQLPREGTGVNQV"
FT                       /IMGT_note="frameshift in FR2-IMGT: insertion of 1 nt"
FT                       /out_of_frame
FT                       /restored_translation
FT   CONSERVED-TRP       315330..315332
FT   CDR2-IMGT           315376..315395
FT                       /translation="TGHEGE"
FT                       /IMGT_note="frameshift in CDR2-IMGT: deletion of 1 nt"
FT                       /out_of_frame
FT   FR3-IMGT            315396..315509
FT                       /translation="QRNPRLKSPVTISRSTSKTQFLLQLSYLSNEYTTMNFY"
FT                       /AA_IMGT="AA 66 to 104, AA 73 is missing"
FT                       /out_of_frame
FT                       /restored_translation
FT   3'V-REGION          315507..315517
FT                       /translation="YTK"
FT                       /out_of_frame
FT                       /restored_translation
FT   CDR3-IMGT           315510..315517
FT                       /translation="TK"
FT                       /AA_IMGT="AA 105 to 106"
FT                       /out_of_frame
FT                       /restored_translation
FT   3'UTR               315518..319000
FT   V-RS                315518..315556
FT   V-HEPTAMER          315518..315524
FT                       /IMGT_note="noncanonical V-HEPTAMER: tacagca instead 
FT                       of cacagtg"
FT   V-SPACER            315525..315547
FT   V-NONAMER           315548..315556
FT   V-GENE              319001..332500
FT                       /functional
FT                       /IMGT_allele="IGHV3-49*04"
FT                       /IMGT_gene="IGHV3-49"
FT   5'UTR               319001..322447
FT   L-V-GENE-UNIT       322448..322948
FT                       /functional
FT                       /IMGT_allele="IGHV3-49*04"
FT                       /IMGT_gene="IGHV3-49"
FT   L-INTRON-L          322448..322607
FT   L-PART1             322448..322493
FT                       /translation="MEFGLSWVFLVAILK"
FT   INIT-CODON          322448..322450
FT   DONOR-SPLICE        322493..322495
FT   V-INTRON            322494..322596
FT   ACCEPTOR-SPLICE     322594..322598
FT   V-EXON              322597..322909
FT                       /codon_start=3
FT                       /translation="VQCEVQLVESGGGLVQPGRSLRLSCTASGFTFGDYAMSWVR
FT                       QAPGKGLEWVGFIRSKAYGGTTEYAASVKGRFTISRDDSKSIAYLQMNSLKTEDT
FT                       AVYYCTR"
FT   L-PART2             322597..322607
FT                       /codon_start=3
FT                       /translation="VQC"
FT   V-REGION            322608..322909
FT                       /translation="EVQLVESGGGLVQPGRSLRLSCTASGFTFGDYAMSWVRQAP
FT                       GKGLEWVGFIRSKAYGGTTEYAASVKGRFTISRDDSKSIAYLQMNSLKTEDTAVY
FT                       YCTR"
FT                       /CDR_length="[8.10.2]"
FT                       /FR_length="[25.17.38.G]"
FT                       /functional
FT                       /IMGT_allele="IGHV3-49*04"
FT                       /IMGT_gene="IGHV3-49"
FT   FR1-IMGT            322608..322682
FT                       /translation="EVQLVESGGGLVQPGRSLRLSCTAS"
FT                       /AA_IMGT="AA 1 to 26, AA 10 is missing"
FT   1st-CYS             322671..322673
FT   CDR1-IMGT           322683..322706
FT                       /translation="GFTFGDYA"
FT                       /AA_IMGT="AA 27 to 38, AA 31, 32, 33 and 34 are 
FT                       missing"
FT   FR2-IMGT            322707..322757
FT                       /translation="MSWVRQAPGKGLEWVGF"
FT                       /AA_IMGT="AA 39 to 55"
FT   CONSERVED-TRP       322713..322715
FT   CDR2-IMGT           322758..322787
FT                       /translation="IRSKAYGGTT"
FT                       /AA_IMGT="AA 56 to 65"
FT   FR3-IMGT            322788..322901
FT                       /translation="EYAASVKGRFTISRDDSKSIAYLQMNSLKTEDTAVYYC"
FT                       /AA_IMGT="AA 66 to 104, AA 73 is missing"
FT   3'V-REGION          322899..322909
FT                       /translation="CTR"
FT   2nd-CYS             322899..322901
FT   CDR3-IMGT           322902..322909
FT                       /translation="TR"
FT                       /AA_IMGT="AA 105 to 106"
FT   3'UTR               322910..332500
FT   V-RS                322910..322948
FT   V-HEPTAMER          322910..322916
FT   V-SPACER            322917..322939
FT   V-NONAMER           322940..322948
FT   V-GENE              332501..345170
FT                       /functional
FT                       /IMGT_allele="IGHV3-48*03"
FT                       /IMGT_gene="IGHV3-48"
FT   5'UTR               332501..341579
FT   L-V-GENE-UNIT       341580..342074
FT                       /functional
FT                       /IMGT_allele="IGHV3-48*03"
FT                       /IMGT_gene="IGHV3-48"
FT   L-INTRON-L          341580..341739
FT   L-PART1             341580..341625
FT                       /translation="MELGLCWVFLVAILE"
FT   INIT-CODON          341580..341582
FT   DONOR-SPLICE        341625..341627
FT   V-INTRON            341626..341728
FT   ACCEPTOR-SPLICE     341726..341730
FT   V-EXON              341729..342035
FT                       /codon_start=3
FT                       /translation="VQCEVQLVESGGGLVQPGGSLRLSCAASGFTFSSYEMNWVR
FT                       QAPGKGLEWVSYISSSGSTIYYADSVKGRFTISRDNAKNSLYLQMNSLRAEDTAV
FT                       YYCAR"
FT   L-PART2             341729..341739
FT                       /codon_start=3
FT                       /translation="VQC"
FT   V-REGION            341740..342035
FT                       /translation="EVQLVESGGGLVQPGGSLRLSCAASGFTFSSYEMNWVRQAP
FT                       GKGLEWVSYISSSGSTIYYADSVKGRFTISRDNAKNSLYLQMNSLRAEDTAVYYC
FT                       AR"
FT                       /CDR_length="[8.8.2]"
FT                       /FR_length="[25.17.38.G]"
FT                       /functional
FT                       /IMGT_allele="IGHV3-48*03"
FT                       /IMGT_gene="IGHV3-48"
FT   FR1-IMGT            341740..341814
FT                       /translation="EVQLVESGGGLVQPGGSLRLSCAAS"
FT                       /AA_IMGT="AA 1 to 26, AA 10 is missing"
FT   1st-CYS             341803..341805
FT   CDR1-IMGT           341815..341838
FT                       /translation="GFTFSSYE"
FT                       /AA_IMGT="AA 27 to 38, AA 31, 32, 33 and 34 are 
FT                       missing"
FT   FR2-IMGT            341839..341889
FT                       /translation="MNWVRQAPGKGLEWVSY"
FT                       /AA_IMGT="AA 39 to 55"
FT   CONSERVED-TRP       341845..341847
FT   CDR2-IMGT           341890..341913
FT                       /translation="ISSSGSTI"
FT                       /AA_IMGT="AA 56 to 65, AA 60 and 61 are missing"
FT   FR3-IMGT            341914..342027
FT                       /translation="YYADSVKGRFTISRDNAKNSLYLQMNSLRAEDTAVYYC"
FT                       /AA_IMGT="AA 66 to 104, AA 73 is missing"
FT   3'V-REGION          342025..342035
FT                       /translation="CAR"
FT   2nd-CYS             342025..342027
FT   CDR3-IMGT           342028..342035
FT                       /translation="AR"
FT                       /AA_IMGT="AA 105 to 106"
FT   3'UTR               342036..345170
FT   V-RS                342036..342074
FT   V-HEPTAMER          342036..342042
FT   V-SPACER            342043..342065
FT   V-NONAMER           342066..342074
FT   V-GENE              345171..354781
FT                       /IMGT_allele="IGHV(III)-47-1*01"
FT                       /IMGT_gene="IGHV(III)-47-1"
FT                       /pseudo="no L-PART1, several STOP-CODON in V-REGION, 
FT                       frameshift in V-REGION: insertion of 2 nt in FR2-IMGT"
FT   5'UTR               345171..348408
FT   ACCEPTOR-SPLICE     348406..348410
FT   V-EXON              348409..348712
FT                       /codon_start=3
FT                       /out_of_frame
FT   L-PART2             348409..348419
FT                       /codon_start=3
FT                       /translation="VPC"
FT   V-REGION            348420..348712
FT                       /CDR_length="[8.8.2]"
FT                       /FR_length="[24.X.38.G]"
FT                       /IMGT_allele="IGHV(III)-47-1*01"
FT                       /IMGT_gene="IGHV(III)-47-1"
FT                       /IMGT_note="restored translation in CDR2-IMGT, 
FT                       FR3-IMGT, 3'V-REGION and CDR3-IMGT"
FT                       /out_of_frame
FT                       /pseudo="no L-PART1, several STOP-CODON in V-REGION, 
FT                       frameshift in V-REGION: insertion of 2 nt in FR2-IMGT"
FT   FR1-IMGT            348420..348491
FT                       /translation="EVNYWSLSEKYYRPRILRLFWKPC"
FT                       /IMGT_note="no 1st-CYS: Trp instead of Cys"
FT   CDR1-IMGT           348492..348515
FT                       /translation="GFTFTENS"
FT                       /AA_IMGT="AA 27 to 38, AA 31, 32, 33 and 34 are 
FT                       missing"
FT   FR2-IMGT            348516..348568
FT                       /IMGT_note="frameshift in FR2-IMGT: insertion of 2 nt"
FT                       /out_of_frame
FT   CDR2-IMGT           348569..348592
FT                       /translation="SNSSGSSQ"
FT                       /AA_IMGT="AA 56 to 65, AA 60 and 61 are missing"
FT                       /out_of_frame
FT                       /restored_translation
FT   FR3-IMGT            348593..348706
FT                       /AA_IMGT="AA 66 to 104, AA 73 is missing"
FT                       /out_of_frame
FT                       /restored_translation
FT   3'V-REGION          348704..348712
FT                       /translation="CEG"
FT                       /out_of_frame
FT                       /restored_translation
FT   CDR3-IMGT           348707..348712
FT                       /translation="EG"
FT                       /AA_IMGT="AA 105 to 106"
FT                       /out_of_frame
FT                       /restored_translation
FT   3'UTR               348713..354781
FT   V-RS                348713..348751
FT   V-HEPTAMER          348713..348719
FT   V-SPACER            348720..348742
FT   V-NONAMER           348743..348751
FT   V-GENE              354782..362476
FT                       /IMGT_allele="IGHV3-47*01"
FT                       /IMGT_gene="IGHV3-47"
FT                       /pseudo="no L-PART1"
FT   5'UTR               354782..360962
FT   ACCEPTOR-SPLICE     360960..360964
FT   V-EXON              360963..361266
FT                       /codon_start=3
FT                       /translation="VQCEDQLVESGGGLVQPGGSLRPSCAASGFAFSSYALHWVR
FT                       RAPGKGLEWVSAIGTGGDTYYADSVMGRFTISRDNAKKSLYLHMNSLIAEDMAVY
FT                       YCAR"
FT   L-PART2             360963..360973
FT                       /codon_start=3
FT                       /translation="VQC"
FT   V-REGION            360974..361266
FT                       /translation="EDQLVESGGGLVQPGGSLRPSCAASGFAFSSYALHWVRRAP
FT                       GKGLEWVSAIGTGGDTYYADSVMGRFTISRDNAKKSLYLHMNSLIAEDMAVYYCA
FT                       R"
FT                       /CDR_length="[8.7.2]"
FT                       /FR_length="[25.17.38.G]"
FT                       /IMGT_allele="IGHV3-47*01"
FT                       /IMGT_gene="IGHV3-47"
FT                       /pseudo="no L-PART1"
FT   FR1-IMGT            360974..361048
FT                       /translation="EDQLVESGGGLVQPGGSLRPSCAAS"
FT                       /AA_IMGT="AA 1 to 26, AA 10 is missing"
FT   1st-CYS             361037..361039
FT   CDR1-IMGT           361049..361072
FT                       /translation="GFAFSSYA"
FT                       /AA_IMGT="AA 27 to 38, AA 31, 32, 33 and 34 are 
FT                       missing"
FT   FR2-IMGT            361073..361123
FT                       /translation="LHWVRRAPGKGLEWVSA"
FT                       /AA_IMGT="AA 39 to 55"
FT   CONSERVED-TRP       361079..361081
FT   CDR2-IMGT           361124..361144
FT                       /translation="IGTGGDT"
FT                       /AA_IMGT="AA 56 to 65, AA 60, 61 and 62 are missing"
FT   FR3-IMGT            361145..361258
FT                       /translation="YYADSVMGRFTISRDNAKKSLYLHMNSLIAEDMAVYYC"
FT                       /AA_IMGT="AA 66 to 104, AA 73 is missing"
FT   3'V-REGION          361256..361266
FT                       /translation="CAR"
FT   2nd-CYS             361256..361258
FT   CDR3-IMGT           361259..361266
FT                       /translation="AR"
FT                       /AA_IMGT="AA 105 to 106"
FT   3'UTR               361267..362476
FT   V-RS                361267..361305
FT   V-HEPTAMER          361267..361273
FT   V-SPACER            361274..361296
FT   V-NONAMER           361297..361305
FT   V-GENE              362477..366000
FT                       /IMGT_allele="IGHV(II)-46-1*01"
FT                       /IMGT_gene="IGHV(II)-46-1"
FT                       /pseudo="truncated pseudogene not assigned to 
FT                       subgroups with functional genes"
FT   5'UTR               362477..363851
FT   ACCEPTOR-SPLICE     363849..363853
FT                       /IMGT_note="noncanonical ACCEPTOR-SPLICE: nacnn 
FT                       instead of nagnn"
FT   V-EXON              363852..364034
FT                       /codon_start=3
FT                       /out_of_frame
FT                       /putative_limit="3'"
FT   L-PART2             363852..363862
FT                       /codon_start=3
FT                       /translation="ALS"
FT   V-REGION            363863..>364034
FT                       /partial
FT                       /CDR_length="[10.6.X]"
FT                       /FR_length="[X.X.X.G]"
FT                       /IMGT_allele="IGHV(II)-46-1*01"
FT                       /IMGT_gene="IGHV(II)-46-1"
FT                       /IMGT_note="partial gene in 3', restored translation 
FT                       in CDR1-IMGT, FR2-IMGT, CDR2-IMGT"
FT                       /out_of_frame
FT                       /pseudo="truncated pseudogene not assigned to 
FT                       subgroups with functional genes"
FT                       /putative_limit="3'"
FT   FR1-IMGT            363863..363936
FT                       /translation="QEQLQESALDLNSTHLPSASPTLL"
FT                       /IMGT_note="frameshift in FR1-IMGT: deletion of 1 nt"
FT                       /out_of_frame
FT   CDR1-IMGT           363937..363966
FT                       /AA_IMGT="AA 27 to 38, AA 32 and 33 are missing"
FT                       /out_of_frame
FT                       /restored_translation
FT   FR2-IMGT            363967..364016
FT                       /IMGT_note="frameshift in FR2-IMGT: deletion of 1 nt"
FT                       /out_of_frame
FT                       /restored_translation
FT   CDR2-IMGT           364017..364034
FT                       /translation="IASGGG"
FT                       /AA_IMGT="AA 56 to 65, AA 59, 60, 61 and 62 are 
FT                       missing"
FT                       /out_of_frame
FT                       /restored_translation
FT   3'UTR               364035..366000
FT   V-GENE              366001..370591
FT                       /functional
FT                       /IMGT_allele="IGHV1-46*01"
FT                       /IMGT_gene="IGHV1-46"
FT   5'UTR               366001..368292
FT   L-V-GENE-UNIT       368293..368770
FT                       /functional
FT                       /IMGT_allele="IGHV1-46*01"
FT                       /IMGT_gene="IGHV1-46"
FT   L-INTRON-L          368293..368434
FT   L-PART1             368293..368338
FT                       /translation="MDWTWRVFCLLAVAP"
FT   INIT-CODON          368293..368295
FT   DONOR-SPLICE        368338..368340
FT   V-INTRON            368339..368423
FT   ACCEPTOR-SPLICE     368421..368425
FT   V-EXON              368424..368730
FT                       /codon_start=3
FT                       /translation="AHSQVQLVQSGAEVKKPGASVKVSCKASGYTFTSYYMHWVR
FT                       QAPGQGLEWMGIINPSGGSTSYAQKFQGRVTMTRDTSTSTVYMELSSLRSEDTAV
FT                       YYCAR"
FT   L-PART2             368424..368434
FT                       /codon_start=3
FT                       /translation="AHS"
FT   V-REGION            368435..368730
FT                       /translation="QVQLVQSGAEVKKPGASVKVSCKASGYTFTSYYMHWVRQAP
FT                       GQGLEWMGIINPSGGSTSYAQKFQGRVTMTRDTSTSTVYMELSSLRSEDTAVYYC
FT                       AR"
FT                       /CDR_length="[8.8.2]"
FT                       /FR_length="[25.17.38.G]"
FT                       /functional
FT                       /IMGT_allele="IGHV1-46*01"
FT                       /IMGT_gene="IGHV1-46"
FT   FR1-IMGT            368435..368509
FT                       /translation="QVQLVQSGAEVKKPGASVKVSCKAS"
FT                       /AA_IMGT="AA 1 to 26, AA 10 is missing"
FT   1st-CYS             368498..368500
FT   CDR1-IMGT           368510..368533
FT                       /translation="GYTFTSYY"
FT                       /AA_IMGT="AA 27 to 38, AA 31, 32, 33 and 34 are 
FT                       missing"
FT   FR2-IMGT            368534..368584
FT                       /translation="MHWVRQAPGQGLEWMGI"
FT                       /AA_IMGT="AA 39 to 55"
FT   CONSERVED-TRP       368540..368542
FT   CDR2-IMGT           368585..368608
FT                       /translation="INPSGGST"
FT                       /AA_IMGT="AA 56 to 65, AA 60 and 61 are missing"
FT   FR3-IMGT            368609..368722
FT                       /translation="SYAQKFQGRVTMTRDTSTSTVYMELSSLRSEDTAVYYC"
FT                       /AA_IMGT="AA 66 to 104, AA 73 is missing"
FT   3'V-REGION          368720..368730
FT                       /translation="CAR"
FT   2nd-CYS             368720..368722
FT   CDR3-IMGT           368723..368730
FT                       /translation="AR"
FT                       /AA_IMGT="AA 105 to 106"
FT   3'UTR               368731..370591
FT   V-RS                368731..368770
FT   V-HEPTAMER          368731..368737
FT   V-SPACER            368738..368761
FT   V-NONAMER           368762..368770
FT   V-GENE              370592..379176
FT                       /functional
FT                       /IMGT_allele="IGHV1-45*02"
FT                       /IMGT_gene="IGHV1-45"
FT   5'UTR               370592..372411
FT   L-V-GENE-UNIT       372412..372888
FT                       /functional
FT                       /IMGT_allele="IGHV1-45*02"
FT                       /IMGT_gene="IGHV1-45"
FT   L-INTRON-L          372412..372553
FT   L-PART1             372412..372457
FT                       /translation="MDWTWRILFLVAAVT"
FT   INIT-CODON          372412..372414
FT   DONOR-SPLICE        372457..372459
FT   V-INTRON            372458..372542
FT   ACCEPTOR-SPLICE     372540..372544
FT   V-EXON              372543..372849
FT                       /codon_start=3
FT                       /translation="AYSQMQLVQSGAEVKKTGSSVKVSCKASGYTFTYRYLHWVR
FT                       QAPGQALEWMGWITPFNGNTNYAQKFQDRVTITRDRSMSTAYMELSSLRSEDTAM
FT                       YYCAR"
FT   L-PART2             372543..372553
FT                       /codon_start=3
FT                       /translation="AYS"
FT   V-REGION            372554..372849
FT                       /translation="QMQLVQSGAEVKKTGSSVKVSCKASGYTFTYRYLHWVRQAP
FT                       GQALEWMGWITPFNGNTNYAQKFQDRVTITRDRSMSTAYMELSSLRSEDTAMYYC
FT                       AR"
FT                       /CDR_length="[8.8.2]"
FT                       /FR_length="[25.17.38.G]"
FT                       /functional
FT                       /IMGT_allele="IGHV1-45*02"
FT                       /IMGT_gene="IGHV1-45"
FT   FR1-IMGT            372554..372628
FT                       /translation="QMQLVQSGAEVKKTGSSVKVSCKAS"
FT                       /AA_IMGT="AA 1 to 26, AA 10 is missing"
FT   1st-CYS             372617..372619
FT   CDR1-IMGT           372629..372652
FT                       /translation="GYTFTYRY"
FT                       /AA_IMGT="AA 27 to 38, AA 31, 32, 33 and 34 are 
FT                       missing"
FT   FR2-IMGT            372653..372703
FT                       /translation="LHWVRQAPGQALEWMGW"
FT                       /AA_IMGT="AA 39 to 55"
FT   CONSERVED-TRP       372659..372661
FT   CDR2-IMGT           372704..372727
FT                       /translation="ITPFNGNT"
FT                       /AA_IMGT="AA 56 to 65, AA 60 and 61 are missing"
FT   FR3-IMGT            372728..372841
FT                       /translation="NYAQKFQDRVTITRDRSMSTAYMELSSLRSEDTAMYYC"
FT                       /AA_IMGT="AA 66 to 104, AA 73 is missing"
FT   3'V-REGION          372839..372849
FT                       /translation="CAR"
FT   2nd-CYS             372839..372841
FT   CDR3-IMGT           372842..372849
FT                       /translation="AR"
FT                       /AA_IMGT="AA 105 to 106"
FT   3'UTR               372850..379176
FT   V-RS                372850..372888
FT   V-HEPTAMER          372850..372856
FT   V-SPACER            372857..372879
FT   V-NONAMER           372880..372888
FT   V-GENE              379177..387907
FT                       /IMGT_allele="IGHV(II)-44-2*01"
FT                       /IMGT_gene="IGHV(II)-44-2"
FT                       /pseudo="truncated pseudogene not assigned to 
FT                       subgroups with functional genes"
FT   5'UTR               379177..385463
FT   V-REGION            <385464..385700
FT                       /partial
FT                       /codon_start=3
FT                       /CDR_length="[X.7.2]"
FT                       /FR_length="[X.X.38.G]"
FT                       /IMGT_allele="IGHV(II)-44-2*01"
FT                       /IMGT_gene="IGHV(II)-44-2"
FT                       /IMGT_note="partial gene in 5'"
FT                       /out_of_frame
FT                       /pseudo="truncated pseudogene not assigned to 
FT                       subgroups with functional genes"
FT                       /putative_limit="5'"
FT   FR2-IMGT            <385518..385558
FT                       /partial
FT                       /codon_start=3
FT                       /translation="ELNPLARFHGLSG"
FT   CDR2-IMGT           385559..385579
FT                       /translation="THCKGDT"
FT                       /AA_IMGT="AA 56 to 65, AA 60, 61 and 62 are missing"
FT   FR3-IMGT            385580..385693
FT                       /AA_IMGT="AA 66 to 104, AA 73 is missing"
FT   3'V-REGION          385690..385700
FT                       /translation="CAR"
FT   2nd-CYS             385690..385692
FT   CDR3-IMGT           385693..385700
FT                       /translation="AR"
FT                       /AA_IMGT="AA 105 to 106"
FT   3'UTR               385701..387907
FT   V-RS                385701..385739
FT   V-HEPTAMER          385701..385707
FT                       /IMGT_note="noncanonical V-HEPTAMER: aacagtg instead 
FT                       of cacagtg"
FT   V-SPACER            385708..385730
FT   V-NONAMER           385731..385739
FT   V-GENE              387908..396000
FT                       /IMGT_allele="IGHV(IV)-44-1*02"
FT                       /IMGT_gene="IGHV(IV)-44-1"
FT                       /pseudo="frameshifts in V-REGION: several insertions 
FT                       and deletions"
FT   5'UTR               387908..390074
FT   L-V-GENE-UNIT       390075..390542
FT                       /IMGT_allele="IGHV(IV)-44-1*02"
FT                       /IMGT_gene="IGHV(IV)-44-1"
FT                       /pseudo="frameshifts in V-REGION: several insertions 
FT                       and deletions"
FT   L-INTRON-L          390075..390214
FT   L-PART1             390075..390120
FT                       /translation="MAPPLLLLCLLAALC"
FT   INIT-CODON          390075..390077
FT   DONOR-SPLICE        390120..390122
FT   V-INTRON            390121..390203
FT   ACCEPTOR-SPLICE     390201..390205
FT   V-EXON              390204..390503
FT                       /codon_start=3
FT                       /out_of_frame
FT   L-PART2             390204..390214
FT                       /codon_start=3
FT                       /translation="VHS"
FT   V-REGION            390215..390503
FT                       /CDR_length="[7.X.2]"
FT                       /FR_length="[26.17.X.G]"
FT                       /IMGT_allele="IGHV(IV)-44-1*02"
FT                       /IMGT_gene="IGHV(IV)-44-1"
FT                       /IMGT_note="restored translation in FR3-IMGT, 
FT                       3'V-REGION and CDR3-IMGT"
FT                       /out_of_frame
FT                       /pseudo="frameshifts in V-REGION: several insertions 
FT                       and deletions"
FT   FR1-IMGT            390215..390292
FT                       /translation="EDHLVQWEEEVVVPLVMLSLTYAAYA"
FT                       /AA_IMGT="AA 1 to 26"
FT                       /IMGT_note="no 1st-CYS: Tyr instead of Cys"
FT   CDR1-IMGT           390293..390313
FT                       /translation="HSISEHS"
FT                       /AA_IMGT="AA 27 to 38, AA 31, 32, 33, 34 and 35 are 
FT                       missing"
FT   FR2-IMGT            390314..390364
FT                       /translation="VSWIHHLPSKGLQCVGV"
FT                       /AA_IMGT="AA 39 to 55"
FT   CONSERVED-TRP       390320..390322
FT   CDR2-IMGT           390365..390388
FT                       /translation="IWVKGNTK"
FT                       /AA_IMGT="AA 56 to 65, AA 60 and 61 are missing"
FT                       /IMGT_note="frameshift in CDR2-IMGT: deletion of 14 nt"
FT                       /out_of_frame
FT   FR3-IMGT            390389..390495
FT                       /IMGT_note="frameshift in FR3-IMGT: insertion of 1 nt"
FT                       /out_of_frame
FT                       /restored_translation
FT   3'V-REGION          390493..390503
FT                       /translation="CAQ"
FT                       /out_of_frame
FT                       /restored_translation
FT   CDR3-IMGT           390496..390503
FT                       /translation="AQ"
FT                       /AA_IMGT="AA 105 to 106"
FT                       /out_of_frame
FT                       /restored_translation
FT   3'UTR               390504..396000
FT   V-RS                390504..390542
FT   V-HEPTAMER          390504..390510
FT   V-SPACER            390511..390533
FT   V-NONAMER           390534..390542
FT   V-GENE              396001..404249
FT                       /IMGT_allele="IGHV(III)-44*01"
FT                       /IMGT_gene="IGHV(III)-44"
FT                       /pseudo="truncated pseudogene not assigned to 
FT                       subgroups with functional genes"
FT   5'UTR               396001..401451
FT   L-INTRON-L          401452..401607
FT   L-PART1             401452..401497
FT                       /translation="MLFGLSWAFLVTILR"
FT   INIT-CODON          401452..401454
FT   DONOR-SPLICE        401497..401499
FT   V-INTRON            401498..401596
FT   ACCEPTOR-SPLICE     401594..401598
FT   V-EXON              401597..401628
FT                       /codon_start=3
FT                       /translation="VQYEVQLVES"
FT                       /putative_limit="3'"
FT   L-PART2             401597..401607
FT                       /codon_start=3
FT                       /translation="VQY"
FT   V-REGION            401608..>401628
FT                       /partial
FT                       /translation="EVQLVES"
FT                       /IMGT_allele="IGHV(III)-44*01"
FT                       /IMGT_gene="IGHV(III)-44"
FT                       /IMGT_note="partial gene in 3'"
FT                       /pseudo="truncated pseudogene not assigned to 
FT                       subgroups with functional genes"
FT                       /putative_limit="3'"
FT   3'UTR               401629..404249
FT   V-GENE              404250..408000
FT                       /IMGT_allele="IGHV(II)-43-1*02"
FT                       /IMGT_gene="IGHV(II)-43-1"
FT                       /pseudo="truncated pseudogene not assigned to 
FT                       subgroups with functional genes"
FT   5'UTR               404250..406844
FT   V-REGION            <406845..>407054
FT                       /partial
FT                       /CDR_length="[10.7.X]"
FT                       /FR_length="[1.17.X.G]"
FT                       /IMGT_allele="IGHV(II)-43-1*02"
FT                       /IMGT_gene="IGHV(II)-43-1"
FT                       /IMGT_note="partial gene in 5' and 3'"
FT                       /out_of_frame
FT                       /pseudo="truncated pseudogene not assigned to 
FT                       subgroups with functional genes"
FT                       /putative_limit="3'"
FT                       /putative_limit="5'"
FT   FR1-IMGT            <406845..>406847
FT                       /partial
FT                       /putative_limit="5'"
FT   CDR1-IMGT           406848..406877
FT                       /translation="GFPNRTSASC"
FT                       /AA_IMGT="AA 27 to 38, AA 32 and 33 are missing"
FT   FR2-IMGT            406878..406928
FT                       /translation="WSWIHQPPGKGLEWVRC"
FT                       /AA_IMGT="AA 39 to 55"
FT   CONSERVED-TRP       406884..406886
FT   CDR2-IMGT           406929..406949
FT                       /translation="TGHEGST"
FT                       /AA_IMGT="AA 56 to 65, AA 60, 61 and 62 are missing"
FT   FR3-IMGT            406950..407054
FT                       /IMGT_note="frameshift in FR3-IMGT: deletion of 1 nt"
FT                       /out_of_frame
FT   3'UTR               407055..408000
FT   V-GENE              408001..412887
FT                       /functional
FT                       /IMGT_allele="IGHV3-43*01"
FT                       /IMGT_gene="IGHV3-43"
FT   5'UTR               408001..409124
FT   L-V-GENE-UNIT       409125..409621
FT                       /functional
FT                       /IMGT_allele="IGHV3-43*01"
FT                       /IMGT_gene="IGHV3-43"
FT   L-INTRON-L          409125..409284
FT   L-PART1             409125..409170
FT                       /translation="MEFGLSWVFLVAILK"
FT   INIT-CODON          409125..409127
FT   DONOR-SPLICE        409170..409172
FT   V-INTRON            409171..409273
FT   ACCEPTOR-SPLICE     409271..409275
FT   V-EXON              409274..409582
FT                       /codon_start=3
FT                       /translation="VQCEVQLVESGGVVVQPGGSLRLSCAASGFTFDDYTMHWVR
FT                       QAPGKGLEWVSLISWDGGSTYYADSVKGRFTISRDNSKNSLYLQMNSLRTEDTAL
FT                       YYCAKD"
FT   L-PART2             409274..409284
FT                       /codon_start=3
FT                       /translation="VQC"
FT   V-REGION            409285..409582
FT                       /translation="EVQLVESGGVVVQPGGSLRLSCAASGFTFDDYTMHWVRQAP
FT                       GKGLEWVSLISWDGGSTYYADSVKGRFTISRDNSKNSLYLQMNSLRTEDTALYYC
FT                       AKD"
FT                       /CDR_length="[8.8.3]"
FT                       /FR_length="[25.17.38.G]"
FT                       /functional
FT                       /IMGT_allele="IGHV3-43*01"
FT                       /IMGT_gene="IGHV3-43"
FT   FR1-IMGT            409285..409359
FT                       /translation="EVQLVESGGVVVQPGGSLRLSCAAS"
FT                       /AA_IMGT="AA 1 to 26, AA 10 is missing"
FT   1st-CYS             409348..409350
FT   CDR1-IMGT           409360..409383
FT                       /translation="GFTFDDYT"
FT                       /AA_IMGT="AA 27 to 38, AA 31, 32, 33 and 34 are 
FT                       missing"
FT   FR2-IMGT            409384..409434
FT                       /translation="MHWVRQAPGKGLEWVSL"
FT                       /AA_IMGT="AA 39 to 55"
FT   CONSERVED-TRP       409390..409392
FT   CDR2-IMGT           409435..409458
FT                       /translation="ISWDGGST"
FT                       /AA_IMGT="AA 56 to 65, AA 60 and 61 are missing"
FT   FR3-IMGT            409459..409572
FT                       /translation="YYADSVKGRFTISRDNSKNSLYLQMNSLRTEDTALYYC"
FT                       /AA_IMGT="AA 66 to 104, AA 73 is missing"
FT   3'V-REGION          409570..409582
FT                       /translation="CAKD"
FT   2nd-CYS             409570..409572
FT   CDR3-IMGT           409573..409582
FT                       /translation="AKD"
FT                       /AA_IMGT="AA 105 to 107"
FT   3'UTR               409583..412887
FT   V-RS                409583..409621
FT   V-HEPTAMER          409583..409589
FT   V-SPACER            409590..409612
FT   V-NONAMER           409613..409621
FT   V-GENE              412888..426445
FT                       /IMGT_allele="IGHV3-42*03"
FT                       /IMGT_gene="IGHV3-42"
FT                       /pseudo="no L-PART1, frameshift in V-REGION: insertion 
FT                       of 1 nt in FR1-IMGT"
FT   5'UTR               412888..416277
FT   ACCEPTOR-SPLICE     416275..416279
FT   V-EXON              416278..416591
FT                       /codon_start=3
FT                       /out_of_frame
FT   L-PART2             416278..416288
FT                       /codon_start=3
FT                       /translation="VQR"
FT   V-REGION            416289..416591
FT                       /CDR_length="[8.10.2]"
FT                       /FR_length="[X.17.38.G]"
FT                       /IMGT_allele="IGHV3-42*03"
FT                       /IMGT_gene="IGHV3-42"
FT                       /IMGT_note="restored translation in CDR1-IMGT, 
FT                       FR2-IMGT, CDR2-IMGT, FR3-IMGT, 3'V-REGION and 
FT                       CDR3-IMGT"
FT                       /out_of_frame
FT                       /pseudo="no L-PART1, frameshift in V-REGION: insertion 
FT                       of 1 nt in FR1-IMGT"
FT   FR1-IMGT            416289..416364
FT                       /translation="EAQLVESGGGLVQPGWVPETLICSF"
FT                       /IMGT_note="frameshift in FR1-IMGT: insertion of 1 nt"
FT                       /out_of_frame
FT   CDR1-IMGT           416365..416388
FT                       /AA_IMGT="AA 27 to 38, AA 31, 32, 33 and 34 are 
FT                       missing"
FT                       /out_of_frame
FT                       /restored_translation
FT   FR2-IMGT            416389..416439
FT                       /translation="MHWIRQASGKGLEWVGR"
FT                       /AA_IMGT="AA 39 to 55"
FT                       /out_of_frame
FT                       /restored_translation
FT   CDR2-IMGT           416440..416469
FT                       /translation="IRTKRNSYTT"
FT                       /AA_IMGT="AA 56 to 65"
FT                       /out_of_frame
FT                       /restored_translation
FT   FR3-IMGT            416470..416583
FT                       /translation="ECAASVKGRFTISRDDSKNTLYLQVNTLKTEYTAIYYC"
FT                       /AA_IMGT="AA 66 to 104, AA 73 is missing"
FT                       /out_of_frame
FT                       /restored_translation
FT   3'V-REGION          416581..416591
FT                       /translation="CTR"
FT                       /out_of_frame
FT                       /restored_translation
FT   CDR3-IMGT           416584..416591
FT                       /translation="TR"
FT                       /AA_IMGT="AA 105 to 106"
FT                       /out_of_frame
FT                       /restored_translation
FT   3'UTR               416592..426445
FT   V-RS                416592..416629
FT   V-HEPTAMER          416592..416598
FT                       /IMGT_note="noncanonical V-HEPTAMER: cagtgag instead 
FT                       of cacagtg"
FT   V-SPACER            416599..416620
FT   V-NONAMER           416621..416629
FT   V-GENE              426446..445000
FT                       /IMGT_allele="IGHV3-41*02"
FT                       /IMGT_gene="IGHV3-41"
FT                       /pseudo="STOP-CODON in V-REGION: position 52"
FT   5'UTR               426446..436260
FT   L-V-GENE-UNIT       436261..436753
FT                       /IMGT_allele="IGHV3-41*02"
FT                       /IMGT_gene="IGHV3-41"
FT                       /pseudo="STOP-CODON in V-REGION: position 52"
FT   L-INTRON-L          436261..436420
FT   L-PART1             436261..436306
FT                       /translation="MESWLSWVFLAAILK"
FT   INIT-CODON          436261..436263
FT   DONOR-SPLICE        436306..436308
FT   V-INTRON            436307..436409
FT   ACCEPTOR-SPLICE     436407..436411
FT   V-EXON              436410..436714
FT                       /codon_start=3
FT   L-PART2             436410..436420
FT                       /codon_start=3
FT                       /translation="VQC"
FT   V-REGION            436421..436714
FT                       /CDR_length="[8.8.2]"
FT                       /FR_length="[25.17.38.G]"
FT                       /IMGT_allele="IGHV3-41*02"
FT                       /IMGT_gene="IGHV3-41"
FT                       /pseudo="STOP-CODON in V-REGION: position 52"
FT   FR1-IMGT            436421..436495
FT                       /translation="EVQLVESGGGLVQPGGSLRLSCAAS"
FT                       /AA_IMGT="AA 1 to 26, AA 10 is missing"
FT   1st-CYS             436484..436486
FT   CDR1-IMGT           436496..436519
FT                       /translation="GFSFSSYG"
FT                       /AA_IMGT="AA 27 to 38, AA 31, 32, 33 and 34 are 
FT                       missing"
FT   FR2-IMGT            436520..436570
FT                       /AA_IMGT="AA 39 to 55"
FT                       /IMGT_note="STOP-CODON in FR2-IMGT: position 52"
FT   CONSERVED-TRP       436526..436528
FT   CDR2-IMGT           436571..436594
FT                       /translation="IWNDGSQK"
FT                       /AA_IMGT="AA 56 to 65, AA 60 and 61 are missing"
FT   FR3-IMGT            436595..436708
FT                       /translation="YYADSVKGRFTISRDNSKSMLYLQMDSLKAKDTAMYYC"
FT                       /AA_IMGT="AA 66 to 104, AA 73 is missing"
FT   3'V-REGION          436706..436714
FT                       /translation="CTR"
FT   2nd-CYS             436706..436708
FT   CDR3-IMGT           436709..436714
FT                       /translation="TR"
FT                       /AA_IMGT="AA 105 to 106"
FT   3'UTR               436715..445000
FT   V-RS                436715..436753
FT   V-HEPTAMER          436715..436721
FT   V-SPACER            436722..436744
FT   V-NONAMER           436745..436753
FT   V-GENE              445001..456000
FT                       /IMGT_allele="IGHV7-40*03"
FT                       /IMGT_gene="IGHV7-40"
FT                       /pseudo="truncated pseudogene not assigned to 
FT                       subgroups with functional genes"
FT   5'UTR               445001..454283
FT   V-REGION            <454284..454486
FT                       /partial
FT                       /CDR_length="[X.8.2]"
FT                       /FR_length="[X.17.38.G]"
FT                       /IMGT_allele="IGHV7-40*03"
FT                       /IMGT_gene="IGHV7-40"
FT                       /IMGT_note="partial gene in 5'"
FT                       /pseudo="truncated pseudogene not assigned to 
FT                       subgroups with functional genes"
FT                       /putative_limit="5'"
FT   CDR1-IMGT           <454284..454289
FT                       /partial
FT                       /translation="YG"
FT                       /AA_IMGT="AA 27 to 38, AA 28, 29, 30, 31, 32, 33, 34, 
FT                       35, 36 and 37 are missing"
FT   FR2-IMGT            454290..454340
FT                       /AA_IMGT="AA 39 to 55"
FT                       /IMGT_note="no CONSERVED-TRP: Ser instead of Trp, 
FT                       STOP-CODON in FR2-IMGT: position 43"
FT   CDR2-IMGT           454341..454364
FT                       /translation="IITYTGNP"
FT                       /AA_IMGT="AA 56 to 65, AA 60 and 61 are missing"
FT   FR3-IMGT            454365..454478
FT                       /translation="TYTNGFTGRFLFSMDTSVSMAYLQISSLKAEDTAVYDC"
FT                       /AA_IMGT="AA 66 to 104, AA 73 is missing"
FT   3'V-REGION          454476..454486
FT                       /translation="CMR"
FT   2nd-CYS             454476..454478
FT   CDR3-IMGT           454479..454486
FT                       /translation="MR"
FT                       /AA_IMGT="AA 105 to 106"
FT   3'UTR               454487..456000
FT   V-RS                454487..454525
FT   V-HEPTAMER          454487..454493
FT   V-SPACER            454494..454516
FT   V-NONAMER           454517..454525
FT   V-GENE              456001..459792
FT                       /functional
FT                       /IMGT_allele="IGHV4-39*01"
FT                       /IMGT_gene="IGHV4-39"
FT   5'UTR               456001..457697
FT   L-V-GENE-UNIT       457698..458175
FT                       /functional
FT                       /IMGT_allele="IGHV4-39*01"
FT                       /IMGT_gene="IGHV4-39"
FT   L-INTRON-L          457698..457837
FT   L-PART1             457698..457743
FT                       /translation="MKHLWFFLLLVAAPR"
FT   INIT-CODON          457698..457700
FT   DONOR-SPLICE        457743..457745
FT   V-INTRON            457744..457826
FT   ACCEPTOR-SPLICE     457824..457828
FT   V-EXON              457827..458136
FT                       /codon_start=3
FT                       /translation="VLSQLQLQESGPGLVKPSETLSLTCTVSGGSISSSSYYWGW
FT                       IRQPPGKGLEWIGSIYYSGSTYYNPSLKSRVTISVDTSKNQFSLKLSSVTAADTA
FT                       VYYCAR"
FT   L-PART2             457827..457837
FT                       /codon_start=3
FT                       /translation="VLS"
FT   V-REGION            457838..458136
FT                       /translation="QLQLQESGPGLVKPSETLSLTCTVSGGSISSSSYYWGWIRQ
FT                       PPGKGLEWIGSIYYSGSTYYNPSLKSRVTISVDTSKNQFSLKLSSVTAADTAVYY
FT                       CAR"
FT                       /CDR_length="[10.7.2]"
FT                       /FR_length="[25.17.38.G]"
FT                       /functional
FT                       /IMGT_allele="IGHV4-39*01"
FT                       /IMGT_gene="IGHV4-39"
FT   FR1-IMGT            457838..457912
FT                       /translation="QLQLQESGPGLVKPSETLSLTCTVS"
FT                       /AA_IMGT="AA 1 to 26, AA 10 is missing"
FT   1st-CYS             457901..457903
FT   CDR1-IMGT           457913..457942
FT                       /translation="GGSISSSSYY"
FT                       /AA_IMGT="AA 27 to 38, AA 32 and 33 are missing"
FT   FR2-IMGT            457943..457993
FT                       /translation="WGWIRQPPGKGLEWIGS"
FT                       /AA_IMGT="AA 39 to 55"
FT   CONSERVED-TRP       457949..457951
FT   CDR2-IMGT           457994..458014
FT                       /translation="IYYSGST"
FT                       /AA_IMGT="AA 56 to 65, AA 60, 61 and 62 are missing"
FT   FR3-IMGT            458015..458128
FT                       /translation="YYNPSLKSRVTISVDTSKNQFSLKLSSVTAADTAVYYC"
FT                       /AA_IMGT="AA 66 to 104, AA 73 is missing"
FT   3'V-REGION          458126..458136
FT                       /translation="CAR"
FT   2nd-CYS             458126..458128
FT   CDR3-IMGT           458129..458136
FT                       /translation="AR"
FT                       /AA_IMGT="AA 105 to 106"
FT   3'UTR               458137..459792
FT   V-RS                458137..458175
FT   V-HEPTAMER          458137..458143
FT   V-SPACER            458144..458166
FT   V-NONAMER           458167..458175
FT   V-GENE              459793..465384
FT                       /IMGT_allele="IGHV(III)-38-1*01"
FT                       /IMGT_gene="IGHV(III)-38-1"
FT                       /pseudo="STOP-CODON in V-REGION: position 4, 
FT                       frameshifts in V-REGION: several insertions and 
FT                       deletions"
FT   5'UTR               459793..461408
FT   L-V-GENE-UNIT       461409..461866
FT                       /IMGT_allele="IGHV(III)-38-1*01"
FT                       /IMGT_gene="IGHV(III)-38-1"
FT                       /pseudo="STOP-CODON in V-REGION: position 4, 
FT                       frameshifts in V-REGION: several insertions and 
FT                       deletions"
FT   L-INTRON-L          461409..461545
FT   L-PART1             461409..461454
FT   INIT-CODON          461409..461411
FT   DONOR-SPLICE        461454..461456
FT                       /IMGT_note="noncanonical DONOR-SPLICE: ntt instead of 
FT                       ngt"
FT   V-INTRON            461455..461534
FT   ACCEPTOR-SPLICE     461532..461536
FT   V-EXON              461535..461827
FT                       /codon_start=3
FT                       /out_of_frame
FT   L-PART2             461535..461545
FT                       /codon_start=3
FT                       /translation="VLC"
FT   V-REGION            461546..461827
FT                       /CDR_length="[8.X.2]"
FT                       /FR_length="[24.17.X.G]"
FT                       /IMGT_allele="IGHV(III)-38-1*01"
FT                       /IMGT_gene="IGHV(III)-38-1"
FT                       /IMGT_note="restored translation in FR3-IMGT"
FT                       /out_of_frame
FT                       /pseudo="STOP-CODON in V-REGION: position 4, 
FT                       frameshifts in V-REGION: several insertions and 
FT                       deletions"
FT   FR1-IMGT            461546..461617
FT                       /IMGT_note="deletions in FR1-IMGT: 1 AA is missing"
FT   1st-CYS             461606..461608
FT   CDR1-IMGT           461618..461635
FT                       /translation="GFTEKR"
FT   FR2-IMGT            461636..461686
FT                       /translation="TSLVQQDSWQVLVREIT"
FT                       /AA_IMGT="AA 39 to 55"
FT                       /IMGT_note="no CONSERVED-TRP: Leu instead of Trp"
FT   CDR2-IMGT           461687..461709
FT                       /translation="VIQVEVL"
FT                       /IMGT_note="frameshift in CDR2-IMGT: deletion of 1 nt"
FT                       /out_of_frame
FT   FR3-IMGT            461710..461821
FT                       /IMGT_note="frameshift in FR3-IMGT: deletion of 2 nt"
FT                       /out_of_frame
FT                       /restored_translation
FT   CDR1-IMGT           461712..461735
FT                       /translation="GLAFSTKK"
FT                       /AA_IMGT="AA 27 to 38, AA 31, 32, 33 and 34 are 
FT                       missing"
FT                       /in_frame
FT   3'V-REGION          461819..461827
FT                       /translation="CEG"
FT                       /in_frame
FT   2nd-CYS             461819..461821
FT   CDR3-IMGT           461822..461827
FT                       /translation="EG"
FT                       /AA_IMGT="AA 105 to 106"
FT                       /in_frame
FT   3'UTR               461828..465384
FT   V-RS                461828..461866
FT   V-HEPTAMER          461828..461834
FT   V-SPACER            461835..461857
FT   V-NONAMER           461858..461866
FT   V-GENE              465385..476000
FT                       /IMGT_allele="IGHV3-38*02"
FT                       /IMGT_gene="IGHV3-38"
FT                       /ORF="this gene has not been found rearranged."
FT   5'UTR               465385..468901
FT   L-V-GENE-UNIT       468902..469390
FT                       /IMGT_allele="IGHV3-38*02"
FT                       /IMGT_gene="IGHV3-38"
FT                       /ORF="this gene has not been found rearranged."
FT   L-INTRON-L          468902..469061
FT   L-PART1             468902..468947
FT                       /translation="MQFVLSWVFLVGILK"
FT   INIT-CODON          468902..468904
FT   DONOR-SPLICE        468947..468949
FT   V-INTRON            468948..469050
FT   ACCEPTOR-SPLICE     469048..469052
FT   V-EXON              469051..469353
FT                       /codon_start=3
FT                       /translation="VQCEVQLVESGGGLVQPRGSLRLSCAASGFTVSSNEMSWIR
FT                       QAPGKGLEWVSSISGGSTYYADSRKGRFTISRDNSKNTLYLQMNNLRAEGTAVYY
FT                       CARY"
FT   L-PART2             469051..469061
FT                       /codon_start=3
FT                       /translation="VQC"
FT   V-REGION            469062..469353
FT                       /translation="EVQLVESGGGLVQPRGSLRLSCAASGFTVSSNEMSWIRQAP
FT                       GKGLEWVSSISGGSTYYADSRKGRFTISRDNSKNTLYLQMNNLRAEGTAVYYCAR
FT                       Y"
FT                       /CDR_length="[8.6.3]"
FT                       /FR_length="[25.17.38.G]"
FT                       /IMGT_allele="IGHV3-38*02"
FT                       /IMGT_gene="IGHV3-38"
FT                       /ORF="this gene has not been found rearranged."
FT   FR1-IMGT            469062..469136
FT                       /translation="EVQLVESGGGLVQPRGSLRLSCAAS"
FT                       /AA_IMGT="AA 1 to 26, AA 10 is missing"
FT   1st-CYS             469125..469127
FT   CDR1-IMGT           469137..469160
FT                       /translation="GFTVSSNE"
FT                       /AA_IMGT="AA 27 to 38, AA 31, 32, 33 and 34 are 
FT                       missing"
FT   FR2-IMGT            469161..469211
FT                       /translation="MSWIRQAPGKGLEWVSS"
FT                       /AA_IMGT="AA 39 to 55"
FT   CONSERVED-TRP       469167..469169
FT   CDR2-IMGT           469212..469229
FT                       /translation="ISGGST"
FT                       /AA_IMGT="AA 56 to 65, AA 59, 60, 61 and 62 are 
FT                       missing"
FT   FR3-IMGT            469230..469343
FT                       /translation="YYADSRKGRFTISRDNSKNTLYLQMNNLRAEGTAVYYC"
FT                       /AA_IMGT="AA 66 to 104, AA 73 is missing"
FT   3'V-REGION          469341..469353
FT                       /translation="CARY"
FT   2nd-CYS             469341..469343
FT   CDR3-IMGT           469344..469353
FT                       /translation="ARY"
FT                       /AA_IMGT="AA 105 to 107"
FT   3'UTR               469354..476000
FT   V-RS                469354..469390
FT   V-HEPTAMER          469354..469360
FT   V-SPACER            469361..469381
FT   V-NONAMER           469382..469390
FT   V-GENE              476001..484916
FT                       /IMGT_allele="IGHV3-37*02"
FT                       /IMGT_gene="IGHV3-37"
FT                       /pseudo="frameshifts in V-REGION: several insertions 
FT                       and deletions"
FT   5'UTR               476001..482730
FT   L-V-GENE-UNIT       482731..483219
FT                       /IMGT_allele="IGHV3-37*02"
FT                       /IMGT_gene="IGHV3-37"
FT                       /pseudo="frameshifts in V-REGION: several insertions 
FT                       and deletions"
FT   L-INTRON-L          482731..482886
FT   L-PART1             482731..482776
FT                       /translation="MLFGLSWAFLFTILR"
FT   INIT-CODON          482731..482733
FT   DONOR-SPLICE        482776..482778
FT   V-INTRON            482777..482875
FT   ACCEPTOR-SPLICE     482873..482877
FT   V-EXON              482876..483180
FT                       /codon_start=3
FT                       /out_of_frame
FT   L-PART2             482876..482886
FT                       /codon_start=3
FT                       /translation="VQY"
FT   V-REGION            482887..483180
FT                       /CDR_length="[8.8.X]"
FT                       /FR_length="[X.17.X.G]"
FT                       /IMGT_allele="IGHV3-37*02"
FT                       /IMGT_gene="IGHV3-37"
FT                       /IMGT_note="restored translation in CDR1-IMGT, 
FT                       FR2-IMGT, CDR2-IMGT, FR3-IMGT"
FT                       /out_of_frame
FT                       /pseudo="frameshifts in V-REGION: several insertions 
FT                       and deletions"
FT   FR1-IMGT            482887..482971
FT                       /translation="EVQLVESGGDLVQLWWGWGCPETLMCSL"
FT                       /IMGT_note="frameshift in FR1-IMGT: insertion of 10 nt"
FT                       /out_of_frame
FT   CDR1-IMGT           482972..482995
FT                       /translation="GFILRSND"
FT                       /AA_IMGT="AA 27 to 38, AA 31, 32, 33 and 34 are 
FT                       missing"
FT                       /out_of_frame
FT                       /restored_translation
FT   FR2-IMGT            482996..483046
FT                       /translation="MNWAHRASRKGLEWVSY"
FT                       /AA_IMGT="AA 39 to 55"
FT                       /out_of_frame
FT                       /restored_translation
FT   CDR2-IMGT           483047..483070
FT                       /translation="ISASGGSL"
FT                       /AA_IMGT="AA 56 to 65, AA 60 and 61 are missing"
FT                       /out_of_frame
FT                       /restored_translation
FT   FR3-IMGT            483071..483180
FT                       /IMGT_note="frameshift in FR3-IMGT: deletion of 2 nt"
FT                       /out_of_frame
FT                       /restored_translation
FT   3'UTR               483181..484916
FT   V-RS                483181..483219
FT   V-HEPTAMER          483181..483187
FT   V-SPACER            483188..483210
FT   V-NONAMER           483211..483219
FT   V-GENE              484917..488639
FT                       /IMGT_allele="IGHV3-36*02"
FT                       /IMGT_gene="IGHV3-36"
FT                       /pseudo="several STOP-CODON in V-REGION, frameshift in 
FT                       V-REGION: deletion of 1 nt in FR1-IMGT, no V-NONAMER"
FT   5'UTR               484917..486613
FT   L-INTRON-L          486614..486775
FT   L-PART1             486614..486659
FT                       /translation="MEFGLSWVFLVANVK"
FT   INIT-CODON          486614..486616
FT   DONOR-SPLICE        486659..486661
FT   V-INTRON            486660..486764
FT   ACCEPTOR-SPLICE     486762..486766
FT   V-EXON              486765..487076
FT                       /codon_start=3
FT                       /out_of_frame
FT   L-PART2             486765..486775
FT                       /codon_start=3
FT                       /translation="VQC"
FT   V-REGION            486776..487076
FT                       /CDR_length="[11.8.1]"
FT                       /FR_length="[X.17.38.G]"
FT                       /IMGT_allele="IGHV3-36*02"
FT                       /IMGT_gene="IGHV3-36"
FT                       /IMGT_note="restored translation in CDR1-IMGT, 
FT                       FR2-IMGT, CDR2-IMGT, FR3-IMGT, 3'V-REGION and 
FT                       CDR3-IMGT"
FT                       /out_of_frame
FT                       /pseudo="several STOP-CODON in V-REGION, frameshift in 
FT                       V-REGION: deletion of 1 nt in FR1-IMGT, no V-NONAMER"
FT   FR1-IMGT            486776..486849
FT                       /IMGT_note="frameshift in FR1-IMGT: deletion of 1 nt"
FT                       /out_of_frame
FT   CDR1-IMGT           486850..486882
FT                       /translation="GFTFSTFIRYW"
FT                       /AA_IMGT="AA 27 to 38, AA 33 is missing"
FT                       /out_of_frame
FT                       /restored_translation
FT   FR2-IMGT            486883..486933
FT                       /AA_IMGT="AA 39 to 55"
FT                       /out_of_frame
FT                       /restored_translation
FT   CDR2-IMGT           486934..486957
FT                       /translation="MSCCVGST"
FT                       /AA_IMGT="AA 56 to 65, AA 60 and 61 are missing"
FT                       /out_of_frame
FT                       /restored_translation
FT   FR3-IMGT            486958..487071
FT                       /translation="SYADSVKGRFTLSRDDAKKSLYLQMNSVRAEDRSVYYC"
FT                       /AA_IMGT="AA 66 to 104, AA 73 is missing"
FT                       /out_of_frame
FT                       /restored_translation
FT   3'V-REGION          487069..487076
FT                       /translation="CG"
FT                       /out_of_frame
FT                       /restored_translation
FT   CDR3-IMGT           487072..487076
FT                       /translation="G"
FT                       /AA_IMGT="AA 105"
FT                       /out_of_frame
FT                       /restored_translation
FT   3'UTR               487077..488639
FT   V-RS                487077..487083
FT                       /IMGT_note="partial V-RS: no V-NONAMER"
FT   V-HEPTAMER          487077..487083
FT                       /IMGT_note="noncanonical V-HEPTAMER: cattgtg instead 
FT                       of cacagtg"
FT   V-GENE              488640..496000
FT                       /functional
FT                       /IMGT_allele="IGHV3-35*01"
FT                       /IMGT_gene="IGHV3-35"
FT   5'UTR               488640..490001
FT   L-V-GENE-UNIT       490002..490494
FT                       /functional
FT                       /IMGT_allele="IGHV3-35*01"
FT                       /IMGT_gene="IGHV3-35"
FT   L-INTRON-L          490002..490159
FT   L-PART1             490002..490047
FT                       /translation="MEFGLSWVFLAAILK"
FT   INIT-CODON          490002..490004
FT   DONOR-SPLICE        490047..490049
FT   V-INTRON            490048..490148
FT   ACCEPTOR-SPLICE     490146..490150
FT   V-EXON              490149..490455
FT                       /codon_start=3
FT                       /translation="VQCEVQLVESGGGLVQPGGSLRLSCAASGFTFSNSDMNWVH
FT                       QAPGKGLEWVSGVSWNGSRTHYADSVKGRFIISRDNSRNTLYLQTNSLRAEDTAV
FT                       YYCVR"
FT   L-PART2             490149..490159
FT                       /codon_start=3
FT                       /translation="VQC"
FT   V-REGION            490160..490455
FT                       /translation="EVQLVESGGGLVQPGGSLRLSCAASGFTFSNSDMNWVHQAP
FT                       GKGLEWVSGVSWNGSRTHYADSVKGRFIISRDNSRNTLYLQTNSLRAEDTAVYYC
FT                       VR"
FT                       /CDR_length="[8.8.2]"
FT                       /FR_length="[25.17.38.G]"
FT                       /functional
FT                       /IMGT_allele="IGHV3-35*01"
FT                       /IMGT_gene="IGHV3-35"
FT   FR1-IMGT            490160..490234
FT                       /translation="EVQLVESGGGLVQPGGSLRLSCAAS"
FT                       /AA_IMGT="AA 1 to 26, AA 10 is missing"
FT   1st-CYS             490223..490225
FT   CDR1-IMGT           490235..490258
FT                       /translation="GFTFSNSD"
FT                       /AA_IMGT="AA 27 to 38, AA 31, 32, 33 and 34 are 
FT                       missing"
FT   FR2-IMGT            490259..490309
FT                       /translation="MNWVHQAPGKGLEWVSG"
FT                       /AA_IMGT="AA 39 to 55"
FT   CONSERVED-TRP       490265..490267
FT   CDR2-IMGT           490310..490333
FT                       /translation="VSWNGSRT"
FT                       /AA_IMGT="AA 56 to 65, AA 60 and 61 are missing"
FT   FR3-IMGT            490334..490447
FT                       /translation="HYADSVKGRFIISRDNSRNTLYLQTNSLRAEDTAVYYC"
FT                       /AA_IMGT="AA 66 to 104, AA 73 is missing"
FT   3'V-REGION          490445..490455
FT                       /translation="CVR"
FT   2nd-CYS             490445..490447
FT   CDR3-IMGT           490448..490455
FT                       /translation="VR"
FT                       /AA_IMGT="AA 105 to 106"
FT   3'UTR               490456..496000
FT   V-RS                490456..490494
FT   V-HEPTAMER          490456..490462
FT   V-SPACER            490463..490485
FT   V-NONAMER           490486..490494
FT   V-GENE              496001..504000
FT                       /IMGT_allele="IGHV7-34-1*01"
FT                       /IMGT_gene="IGHV7-34-1"
FT                       /pseudo="STOP-CODON in L-PART1, several STOP-CODON in 
FT                       V-REGION"
FT   5'UTR               496001..502111
FT   L-V-GENE-UNIT       502112..502584
FT                       /IMGT_allele="IGHV7-34-1*01"
FT                       /IMGT_gene="IGHV7-34-1"
FT                       /pseudo="STOP-CODON in L-PART1, several STOP-CODON in 
FT                       V-REGION"
FT   L-INTRON-L          502112..502252
FT   L-PART1             502112..502157
FT                       /IMGT_note="STOP-CODON in L-PART1"
FT   INIT-CODON          502112..502114
FT   DONOR-SPLICE        502157..502159
FT   V-INTRON            502158..502241
FT   ACCEPTOR-SPLICE     502239..502243
FT   V-EXON              502242..502545
FT                       /codon_start=3
FT   L-PART2             502242..502252
FT                       /codon_start=3
FT                       /translation="AHS"
FT   V-REGION            502253..502545
FT                       /CDR_length="[8.8.2]"
FT                       /FR_length="[24.17.38.G]"
FT                       /IMGT_allele="IGHV7-34-1*01"
FT                       /IMGT_gene="IGHV7-34-1"
FT                       /pseudo="STOP-CODON in L-PART1, several STOP-CODON in 
FT                       V-REGION"
FT   FR1-IMGT            502253..502324
FT                       /translation="LQLVQSGPEVKKPGASVKVSYKSS"
FT                       /IMGT_note="deletions in FR1-IMGT: 1 AA is missing"
FT   CDR1-IMGT           502325..502348
FT                       /translation="GYTFTIYG"
FT                       /AA_IMGT="AA 27 to 38, AA 31, 32, 33 and 34 are 
FT                       missing"
FT   FR2-IMGT            502349..502399
FT                       /AA_IMGT="AA 39 to 55"
FT                       /IMGT_note="several STOP-CODON in FR2-IMGT: pos 43, 44 
FT                       and 54"
FT   CONSERVED-TRP       502355..502357
FT   CDR2-IMGT           502400..502423
FT                       /translation="IITYTGNP"
FT                       /AA_IMGT="AA 56 to 65, AA 60 and 61 are missing"
FT   FR3-IMGT            502424..502537
FT                       /translation="TYTHGFTGWFVFSMDTSVSTACLQISSLKAEDTAEYYC"
FT                       /AA_IMGT="AA 66 to 104, AA 73 is missing"
FT   3'V-REGION          502535..502545
FT                       /translation="CAK"
FT   2nd-CYS             502535..502537
FT   CDR3-IMGT           502538..502545
FT                       /translation="AK"
FT                       /AA_IMGT="AA 105 to 106"
FT   3'UTR               502546..504000
FT   V-RS                502546..502584
FT   V-HEPTAMER          502546..502552
FT   V-SPACER            502553..502575
FT   V-NONAMER           502576..502584
FT                       /IMGT_note="noncanonical V-NONAMER: tcagaaagc instead 
FT                       of acacaaacc"
FT   V-GENE              504001..508261
FT                       /functional
FT                       /IMGT_allele="IGHV4-34*01"
FT                       /IMGT_gene="IGHV4-34"
FT   5'UTR               504001..505751
FT   L-V-GENE-UNIT       505752..506223
FT                       /functional
FT                       /IMGT_allele="IGHV4-34*01"
FT                       /IMGT_gene="IGHV4-34"
FT   L-INTRON-L          505752..505891
FT   L-PART1             505752..505797
FT                       /translation="MKHLWFFLLLVAAPR"
FT   INIT-CODON          505752..505754
FT   DONOR-SPLICE        505797..505799
FT   V-INTRON            505798..505880
FT   ACCEPTOR-SPLICE     505878..505882
FT   V-EXON              505881..506184
FT                       /codon_start=3
FT                       /translation="VLSQVQLQQWGAGLLKPSETLSLTCAVYGGSFSGYYWSWIR
FT                       QPPGKGLEWIGEINHSGSTNYNPSLKSRVTISVDTSKNQFSLKLSSVTAADTAVY
FT                       YCAR"
FT   L-PART2             505881..505891
FT                       /codon_start=3
FT                       /translation="VLS"
FT   V-REGION            505892..506184
FT                       /translation="QVQLQQWGAGLLKPSETLSLTCAVYGGSFSGYYWSWIRQPP
FT                       GKGLEWIGEINHSGSTNYNPSLKSRVTISVDTSKNQFSLKLSSVTAADTAVYYCA
FT                       R"
FT                       /CDR_length="[8.7.2]"
FT                       /FR_length="[25.17.38.G]"
FT                       /functional
FT                       /IMGT_allele="IGHV4-34*01"
FT                       /IMGT_gene="IGHV4-34"
FT   FR1-IMGT            505892..505966
FT                       /translation="QVQLQQWGAGLLKPSETLSLTCAVY"
FT                       /AA_IMGT="AA 1 to 26, AA 10 is missing"
FT   1st-CYS             505955..505957
FT   CDR1-IMGT           505967..505990
FT                       /translation="GGSFSGYY"
FT                       /AA_IMGT="AA 27 to 38, AA 31, 32, 33 and 34 are 
FT                       missing"
FT   FR2-IMGT            505991..506041
FT                       /translation="WSWIRQPPGKGLEWIGE"
FT                       /AA_IMGT="AA 39 to 55"
FT   CONSERVED-TRP       505997..505999
FT   CDR2-IMGT           506042..506062
FT                       /translation="INHSGST"
FT                       /AA_IMGT="AA 56 to 65, AA 60, 61 and 62 are missing"
FT   FR3-IMGT            506063..506176
FT                       /translation="NYNPSLKSRVTISVDTSKNQFSLKLSSVTAADTAVYYC"
FT                       /AA_IMGT="AA 66 to 104, AA 73 is missing"
FT   3'V-REGION          506174..506184
FT                       /translation="CAR"
FT   2nd-CYS             506174..506176
FT   CDR3-IMGT           506177..506184
FT                       /translation="AR"
FT                       /AA_IMGT="AA 105 to 106"
FT   3'UTR               506185..508261
FT   V-RS                506185..506223
FT   V-HEPTAMER          506185..506191
FT   V-SPACER            506192..506214
FT   V-NONAMER           506215..506223
FT   V-GENE              508262..511303
FT                       /IMGT_allele="IGHV3-33-2*01"
FT                       /IMGT_gene="IGHV3-33-2"
FT                       /pseudo="STOP-CODON in L-PART2, several STOP-CODON in 
FT                       V-REGION"
FT   5'UTR               508262..510298
FT   L-V-GENE-UNIT       510299..510788
FT                       /IMGT_allele="IGHV3-33-2*01"
FT                       /IMGT_gene="IGHV3-33-2"
FT                       /pseudo="STOP-CODON in L-PART2, several STOP-CODON in 
FT                       V-REGION"
FT   L-INTRON-L          510299..510449
FT   L-PART1             510299..510344
FT                       /translation="MELGLSWVFTVAVLK"
FT   INIT-CODON          510299..510301
FT   DONOR-SPLICE        510344..510346
FT   V-INTRON            510345..510438
FT   ACCEPTOR-SPLICE     510436..510440
FT   V-EXON              510439..510747
FT                       /codon_start=3
FT   L-PART2             510439..510449
FT                       /codon_start=3
FT                       /IMGT_note="STOP-CODON in L-PART2"
FT   V-REGION            510450..510747
FT                       /CDR_length="[8.8.3]"
FT                       /FR_length="[25.17.38.G]"
FT                       /IMGT_allele="IGHV3-33-2*01"
FT                       /IMGT_gene="IGHV3-33-2"
FT                       /pseudo="STOP-CODON in L-PART2, several STOP-CODON in 
FT                       V-REGION"
FT   FR1-IMGT            510450..510524
FT                       /translation="EVQLVESGEDPRQPGGSLRLSCADS"
FT                       /AA_IMGT="AA 1 to 26, AA 10 is missing"
FT   1st-CYS             510513..510515
FT   CDR1-IMGT           510525..510548
FT                       /AA_IMGT="AA 27 to 38, AA 31, 32, 33 and 34 are 
FT                       missing"
FT                       /IMGT_note="STOP-CODON in CDR1-IMGT: position 38"
FT   FR2-IMGT            510549..510599
FT                       /AA_IMGT="AA 39 to 55"
FT                       /IMGT_note="no CONSERVED-TRP: Ser instead of Trp, 
FT                       STOP-CODON in FR2-IMGT: position 52"
FT   CDR2-IMGT           510600..510623
FT                       /translation="IQCDGSQI"
FT                       /AA_IMGT="AA 56 to 65, AA 60 and 61 are missing"
FT   FR3-IMGT            510624..510737
FT                       /translation="CYAQSVKSKFTISKENAKNSLYLQMNSLRAEGTAVCYC"
FT                       /AA_IMGT="AA 66 to 104, AA 73 is missing"
FT   3'V-REGION          510735..510747
FT   2nd-CYS             510735..510737
FT   CDR3-IMGT           510738..510747
FT                       /AA_IMGT="AA 105 to 107"
FT                       /IMGT_note="STOP-CODON in CDR3-IMGT: position 106"
FT   3'UTR               510748..511303
FT   V-RS                510748..510788
FT   V-HEPTAMER          510748..510754
FT                       /IMGT_note="noncanonical V-HEPTAMER: ccaggta instead 
FT                       of cacagtg"
FT   V-SPACER            510755..510779
FT   V-NONAMER           510780..510788
FT                       /IMGT_note="noncanonical V-NONAMER: acagtttcc instead 
FT                       of acacaaacc"
FT   V-GENE              511304..516047
FT                       /IMGT_allele="IGHV(II)-33-1*01"
FT                       /IMGT_gene="IGHV(II)-33-1"
FT                       /pseudo="truncated pseudogene not assigned to 
FT                       subgroups with functional genes"
FT   5'UTR               511304..512139
FT   ACCEPTOR-SPLICE     512137..512141
FT   V-EXON              512140..512454
FT                       /codon_start=3
FT   L-PART2             512140..512153
FT                       /codon_start=3
FT                       /translation="PVAA"
FT   V-REGION            512154..512454
FT                       /CDR_length="[10.7.2]"
FT                       /FR_length="[25.X.38.G]"
FT                       /IMGT_allele="IGHV(II)-33-1*01"
FT                       /IMGT_gene="IGHV(II)-33-1"
FT                       /IMGT_note="restored translation in CDR2-IMGT, 
FT                       FR3-IMGT, 3'V-REGION and CDR3-IMGT"
FT                       /out_of_frame
FT                       /pseudo="truncated pseudogene not assigned to 
FT                       subgroups with functional genes"
FT   FR1-IMGT            512154..512228
FT                       /translation="NNCISRCTLGLVKPSQNLSLTCAIS"
FT                       /AA_IMGT="AA 1 to 26, AA 10 is missing"
FT   1st-CYS             512217..512219
FT   CDR1-IMGT           512229..512258
FT                       /translation="AFSITTSVSF"
FT                       /AA_IMGT="AA 27 to 38, AA 32 and 33 are missing"
FT   FR2-IMGT            512259..512311
FT                       /translation="WSCIHQRPHGRYWSGSG"
FT                       /IMGT_note="frameshift in FR2-IMGT: insertion of 2 nt"
FT                       /out_of_frame
FT   CDR2-IMGT           512312..512332
FT                       /translation="IGHGGST"
FT                       /AA_IMGT="AA 56 to 65, AA 60, 61 and 62 are missing"
FT                       /out_of_frame
FT                       /restored_translation
FT   FR3-IMGT            512333..512446
FT                       /translation="DYSPLLKSPVTISRSMSKKYFFLQVNYVSNKHIAMYFR"
FT                       /AA_IMGT="AA 66 to 104, AA 73 is missing"
FT                       /out_of_frame
FT                       /restored_translation
FT   3'V-REGION          512444..512454
FT                       /translation="RAK"
FT                       /out_of_frame
FT                       /restored_translation
FT   CDR3-IMGT           512447..512454
FT                       /translation="AK"
FT                       /AA_IMGT="AA 105 to 106"
FT                       /out_of_frame
FT                       /restored_translation
FT   3'UTR               512455..516047
FT   V-RS                512455..512493
FT   V-HEPTAMER          512455..512461
FT   V-SPACER            512462..512484
FT   V-NONAMER           512485..512493
FT   V-GENE              516048..521000
FT                       /functional
FT                       /IMGT_allele="IGHV3-33*01"
FT                       /IMGT_gene="IGHV3-33"
FT   5'UTR               516048..519600
FT   L-V-GENE-UNIT       519601..520093
FT                       /functional
FT                       /IMGT_allele="IGHV3-33*01"
FT                       /IMGT_gene="IGHV3-33"
FT   L-INTRON-L          519601..519758
FT   L-PART1             519601..519646
FT                       /translation="MEFGLSWVFLVALLR"
FT   INIT-CODON          519601..519603
FT   DONOR-SPLICE        519646..519648
FT   V-INTRON            519647..519747
FT   ACCEPTOR-SPLICE     519745..519749
FT   V-EXON              519748..520054
FT                       /codon_start=3
FT                       /translation="VQCQVQLVESGGGVVQPGRSLRLSCAASGFTFSSYGMHWVR
FT                       QAPGKGLEWVAVIWYDGSNKYYADSVKGRFTISRDNSKNTLYLQMNSLRAEDTAV
FT                       YYCAR"
FT   L-PART2             519748..519758
FT                       /codon_start=3
FT                       /translation="VQC"
FT   V-REGION            519759..520054
FT                       /translation="QVQLVESGGGVVQPGRSLRLSCAASGFTFSSYGMHWVRQAP
FT                       GKGLEWVAVIWYDGSNKYYADSVKGRFTISRDNSKNTLYLQMNSLRAEDTAVYYC
FT                       AR"
FT                       /CDR_length="[8.8.2]"
FT                       /FR_length="[25.17.38.G]"
FT                       /functional
FT                       /IMGT_allele="IGHV3-33*01"
FT                       /IMGT_gene="IGHV3-33"
FT   FR1-IMGT            519759..519833
FT                       /translation="QVQLVESGGGVVQPGRSLRLSCAAS"
FT                       /AA_IMGT="AA 1 to 26, AA 10 is missing"
FT   1st-CYS             519822..519824
FT   CDR1-IMGT           519834..519857
FT                       /translation="GFTFSSYG"
FT                       /AA_IMGT="AA 27 to 38, AA 31, 32, 33 and 34 are 
FT                       missing"
FT   FR2-IMGT            519858..519908
FT                       /translation="MHWVRQAPGKGLEWVAV"
FT                       /AA_IMGT="AA 39 to 55"
FT   CONSERVED-TRP       519864..519866
FT   CDR2-IMGT           519909..519932
FT                       /translation="IWYDGSNK"
FT                       /AA_IMGT="AA 56 to 65, AA 60 and 61 are missing"
FT   FR3-IMGT            519933..520046
FT                       /translation="YYADSVKGRFTISRDNSKNTLYLQMNSLRAEDTAVYYC"
FT                       /AA_IMGT="AA 66 to 104, AA 73 is missing"
FT   3'V-REGION          520044..520054
FT                       /translation="CAR"
FT   2nd-CYS             520044..520046
FT   CDR3-IMGT           520047..520054
FT                       /translation="AR"
FT                       /AA_IMGT="AA 105 to 106"
FT   3'UTR               520055..521000
FT   V-RS                520055..520093
FT   V-HEPTAMER          520055..520061
FT   V-SPACER            520062..520084
FT   V-NONAMER           520085..520093
FT   GENE                521001..523000
FT                       /IMGT_gene="GOLGA4P1"
FT                       /pseudo
FT   5'UTR               521001..522047
FT   GENE-UNIT           522048..522561
FT                       /IMGT_gene="GOLGA4P1"
FT                       /pseudo
FT   3'UTR               522562..523000
FT   V-GENE              523001..524419
FT                       /IMGT_allele="IGHV3-32*01"
FT                       /IMGT_gene="IGHV3-32"
FT                       /pseudo="STOP-CODON in L-PART1, several STOP-CODON in 
FT                       V-REGION"
FT   5'UTR               523001..523242
FT   L-V-GENE-UNIT       523243..523739
FT                       /IMGT_allele="IGHV3-32*01"
FT                       /IMGT_gene="IGHV3-32"
FT                       /pseudo="STOP-CODON in L-PART1, several STOP-CODON in 
FT                       V-REGION"
FT   L-INTRON-L          523243..523402
FT   L-PART1             523243..523288
FT                       /IMGT_note="STOP-CODON in L-PART1"
FT   INIT-CODON          523243..523245
FT   DONOR-SPLICE        523288..523290
FT   V-INTRON            523289..523391
FT   ACCEPTOR-SPLICE     523389..523393
FT   V-EXON              523392..523700
FT                       /codon_start=3
FT   L-PART2             523392..523402
FT                       /codon_start=3
FT                       /translation="VQC"
FT   V-REGION            523403..523700
FT                       /CDR_length="[8.8.3]"
FT                       /FR_length="[25.17.38.G]"
FT                       /IMGT_allele="IGHV3-32*01"
FT                       /IMGT_gene="IGHV3-32"
FT                       /pseudo="STOP-CODON in L-PART1, several STOP-CODON in 
FT                       V-REGION"
FT   FR1-IMGT            523403..523477
FT                       /translation="EVELIESIEDLRQPGKFLRLSCVAS"
FT                       /AA_IMGT="AA 1 to 26, AA 10 is missing"
FT   1st-CYS             523466..523468
FT   CDR1-IMGT           523478..523501
FT                       /AA_IMGT="AA 27 to 38, AA 31, 32, 33 and 34 are 
FT                       missing"
FT                       /IMGT_note="STOP-CODON in CDR1-IMGT: position 38"
FT   FR2-IMGT            523502..523552
FT                       /AA_IMGT="AA 39 to 55"
FT                       /IMGT_note="no CONSERVED-TRP: Arg instead of Trp, 
FT                       STOP-CODON in FR2-IMGT: position 52"
FT   CDR2-IMGT           523553..523576
FT                       /translation="IKDDGSQI"
FT                       /AA_IMGT="AA 56 to 65, AA 60 and 61 are missing"
FT   FR3-IMGT            523577..523690
FT                       /translation="HHADSVKGRFSISKDNAKNSLYLQMNTQRAEDVAVYGY"
FT                       /AA_IMGT="AA 66 to 104, AA 73 is missing"
FT                       /IMGT_note="no 2nd-CYS: Tyr instead of Cys"
FT   3'V-REGION          523688..523700
FT   CDR3-IMGT           523691..523700
FT                       /AA_IMGT="AA 105 to 107"
FT                       /IMGT_note="STOP-CODON in CDR3-IMGT: position 106"
FT   3'UTR               523701..524419
FT   V-RS                523701..523739
FT   V-HEPTAMER          523701..523707
FT                       /IMGT_note="noncanonical V-HEPTAMER: ccaagtg instead 
FT                       of cacagtg"
FT   V-SPACER            523708..523730
FT   V-NONAMER           523731..523739
FT                       /IMGT_note="noncanonical V-NONAMER: acacaacat instead 
FT                       of acacaaacc"
FT   V-GENE              524420..527775
FT                       /IMGT_allele="IGHV(II)-30-21*01"
FT                       /IMGT_gene="IGHV(II)-30-21"
FT                       /pseudo="truncated pseudogene not assigned to 
FT                       subgroups with functional genes"
FT   5'UTR               524420..525125
FT   V-REGION            <525126..525381
FT                       /partial
FT                       /CDR_length="[10.7.2]"
FT                       /FR_length="[16.17.X.G]"
FT                       /IMGT_allele="IGHV(II)-30-21*01"
FT                       /IMGT_gene="IGHV(II)-30-21"
FT                       /IMGT_note="partial gene in 5', restored translation 
FT                       in 3'V-REGION and CDR3-IMGT"
FT                       /out_of_frame
FT                       /pseudo="truncated pseudogene not assigned to 
FT                       subgroups with functional genes"
FT                       /putative_limit="5'"
FT   FR1-IMGT            <525126..525173
FT                       /partial
FT                       /translation="WLETSSQTLSLTCTVS"
FT                       /putative_limit="5'"
FT   1st-CYS             525162..525164
FT   CDR1-IMGT           525174..525203
FT                       /translation="GFPIITSVSF"
FT                       /AA_IMGT="AA 27 to 38, AA 32 and 33 are missing"
FT   FR2-IMGT            525204..525254
FT                       /AA_IMGT="AA 39 to 55"
FT                       /IMGT_note="STOP-CODON in FR2-IMGT: position 39, no 
FT                       CONSERVED-TRP: Cys instead of Trp"
FT   CDR2-IMGT           525255..525275
FT                       /translation="MGCEGST"
FT                       /AA_IMGT="AA 56 to 65, AA 60, 61 and 62 are missing"
FT   FR3-IMGT            525276..525373
FT                       /IMGT_note="frameshift in FR3-IMGT: deletion of 7 nt, 
FT                       deletions in FR3-IMGT: 2 AA are missing, deletions in 
FT                       FR3-IMGT: 1 AA is missing"
FT                       /out_of_frame
FT   3'V-REGION          525371..525381
FT                       /translation="EAK"
FT                       /out_of_frame
FT                       /restored_translation
FT   CDR3-IMGT           525374..525381
FT                       /translation="AK"
FT                       /AA_IMGT="AA 105 to 106"
FT                       /out_of_frame
FT                       /restored_translation
FT   3'UTR               525382..527775
FT   V-RS                525382..525420
FT   V-HEPTAMER          525382..525388
FT                       /IMGT_note="noncanonical V-HEPTAMER: tgcaatg instead 
FT                       of cacagtg"
FT   V-SPACER            525389..525411
FT   V-NONAMER           525412..525420
FT   V-GENE              527776..532806
FT                       /functional
FT                       /IMGT_allele="IGHV4-30-2*01"
FT                       /IMGT_gene="IGHV4-30-2"
FT   5'UTR               527776..530124
FT   L-V-GENE-UNIT       530125..530601
FT                       /functional
FT                       /IMGT_allele="IGHV4-30-2*01"
FT                       /IMGT_gene="IGHV4-30-2"
FT   L-INTRON-L          530125..530263
FT   L-PART1             530125..530170
FT                       /translation="MKHLWFFLLLVAAPR"
FT   INIT-CODON          530125..530127
FT   DONOR-SPLICE        530170..530172
FT   V-INTRON            530171..530252
FT   ACCEPTOR-SPLICE     530250..530254
FT   V-EXON              530253..530562
FT                       /codon_start=3
FT                       /translation="VLSQLQLQESGSGLVKPSQTLSLTCAVSGGSISSGGYSWSW
FT                       IRQPPGKGLEWIGYIYHSGSTYYNPSLKSRVTISVDRSKNQFSLKLSSVTAADTA
FT                       VYYCAR"
FT   L-PART2             530253..530263
FT                       /codon_start=3
FT                       /translation="VLS"
FT   V-REGION            530264..530562
FT                       /translation="QLQLQESGSGLVKPSQTLSLTCAVSGGSISSGGYSWSWIRQ
FT                       PPGKGLEWIGYIYHSGSTYYNPSLKSRVTISVDRSKNQFSLKLSSVTAADTAVYY
FT                       CAR"
FT                       /CDR_length="[10.7.2]"
FT                       /FR_length="[25.17.38.G]"
FT                       /functional
FT                       /IMGT_allele="IGHV4-30-2*01"
FT                       /IMGT_gene="IGHV4-30-2"
FT   FR1-IMGT            530264..530338
FT                       /translation="QLQLQESGSGLVKPSQTLSLTCAVS"
FT                       /AA_IMGT="AA 1 to 26, AA 10 is missing"
FT   1st-CYS             530327..530329
FT   CDR1-IMGT           530339..530368
FT                       /translation="GGSISSGGYS"
FT                       /AA_IMGT="AA 27 to 38, AA 32 and 33 are missing"
FT   FR2-IMGT            530369..530419
FT                       /translation="WSWIRQPPGKGLEWIGY"
FT                       /AA_IMGT="AA 39 to 55"
FT   CONSERVED-TRP       530375..530377
FT   CDR2-IMGT           530420..530440
FT                       /translation="IYHSGST"
FT                       /AA_IMGT="AA 56 to 65, AA 60, 61 and 62 are missing"
FT   FR3-IMGT            530441..530554
FT                       /translation="YYNPSLKSRVTISVDRSKNQFSLKLSSVTAADTAVYYC"
FT                       /AA_IMGT="AA 66 to 104, AA 73 is missing"
FT   3'V-REGION          530552..530562
FT                       /translation="CAR"
FT   2nd-CYS             530552..530554
FT   CDR3-IMGT           530555..530562
FT                       /translation="AR"
FT                       /AA_IMGT="AA 105 to 106"
FT   3'UTR               530563..532806
FT   V-RS                530563..530601
FT   V-HEPTAMER          530563..530569
FT   V-SPACER            530570..530592
FT   V-NONAMER           530593..530601
FT   V-GENE              532807..536015
FT                       /IMGT_allele="IGHV3-30-2*01"
FT                       /IMGT_gene="IGHV3-30-2"
FT                       /pseudo="STOP-CODON in L-PART2, several STOP-CODON in 
FT                       V-REGION"
FT   5'UTR               532807..535011
FT   L-V-GENE-UNIT       535012..535497
FT                       /IMGT_allele="IGHV3-30-2*01"
FT                       /IMGT_gene="IGHV3-30-2"
FT                       /pseudo="STOP-CODON in L-PART2, several STOP-CODON in 
FT                       V-REGION"
FT   L-INTRON-L          535012..535162
FT   L-PART1             535012..535057
FT                       /translation="MELGLSWVFTVAVLK"
FT   INIT-CODON          535012..535014
FT   DONOR-SPLICE        535057..535059
FT   V-INTRON            535058..535151
FT   ACCEPTOR-SPLICE     535149..535153
FT   V-EXON              535152..535460
FT                       /codon_start=3
FT   L-PART2             535152..535162
FT                       /codon_start=3
FT                       /IMGT_note="STOP-CODON in L-PART2"
FT   V-REGION            535163..535460
FT                       /CDR_length="[8.8.3]"
FT                       /FR_length="[25.17.38.G]"
FT                       /IMGT_allele="IGHV3-30-2*01"
FT                       /IMGT_gene="IGHV3-30-2"
FT                       /pseudo="STOP-CODON in L-PART2, several STOP-CODON in 
FT                       V-REGION"
FT   FR1-IMGT            535163..535237
FT                       /translation="EVQLVESGEDPRQPGGSLRLSCADS"
FT                       /AA_IMGT="AA 1 to 26, AA 10 is missing"
FT   1st-CYS             535226..535228
FT   CDR1-IMGT           535238..535261
FT                       /AA_IMGT="AA 27 to 38, AA 31, 32, 33 and 34 are 
FT                       missing"
FT                       /IMGT_note="STOP-CODON in CDR1-IMGT: position 38"
FT   FR2-IMGT            535262..535312
FT                       /AA_IMGT="AA 39 to 55"
FT                       /IMGT_note="no CONSERVED-TRP: Ser instead of Trp, 
FT                       STOP-CODON in FR2-IMGT: position 52"
FT   CDR2-IMGT           535313..535336
FT                       /translation="IQCDGSQI"
FT                       /AA_IMGT="AA 56 to 65, AA 60 and 61 are missing"
FT   FR3-IMGT            535337..535450
FT                       /AA_IMGT="AA 66 to 104, AA 73 is missing"
FT                       /IMGT_note="STOP-CODON in FR3-IMGT: position 69"
FT   3'V-REGION          535448..535460
FT   2nd-CYS             535448..535450
FT   CDR3-IMGT           535451..535460
FT                       /AA_IMGT="AA 105 to 107"
FT                       /IMGT_note="STOP-CODON in CDR3-IMGT: position 106"
FT   3'UTR               535461..536015
FT   V-RS                535461..535497
FT   V-HEPTAMER          535461..535467
FT                       /IMGT_note="noncanonical V-HEPTAMER: ccaggta instead 
FT                       of cacagtg"
FT   V-SPACER            535468..535488
FT   V-NONAMER           535489..535497
FT                       /IMGT_note="noncanonical V-NONAMER: agacacagt instead 
FT                       of acacaaacc"
FT   V-GENE              536016..540758
FT                       /IMGT_allele="IGHV(II)-30-1*02"
FT                       /IMGT_gene="IGHV(II)-30-1"
FT                       /pseudo="truncated pseudogene not assigned to 
FT                       subgroups with functional genes"
FT   5'UTR               536016..536850
FT   ACCEPTOR-SPLICE     536848..536852
FT   V-EXON              536851..537165
FT                       /codon_start=3
FT   L-PART2             536851..536864
FT                       /codon_start=3
FT                       /translation="PVAA"
FT   V-REGION            536865..537165
FT                       /CDR_length="[10.7.2]"
FT                       /FR_length="[25.X.38.G]"
FT                       /IMGT_allele="IGHV(II)-30-1*02"
FT                       /IMGT_gene="IGHV(II)-30-1"
FT                       /IMGT_note="restored translation in CDR2-IMGT, 
FT                       FR3-IMGT, 3'V-REGION and CDR3-IMGT"
FT                       /out_of_frame
FT                       /pseudo="truncated pseudogene not assigned to 
FT                       subgroups with functional genes"
FT   FR1-IMGT            536865..536939
FT                       /translation="NNCISRCTLGLVKPSQNLSLTCAIS"
FT                       /AA_IMGT="AA 1 to 26, AA 10 is missing"
FT   1st-CYS             536928..536930
FT   CDR1-IMGT           536940..536969
FT                       /translation="AFSITTSVSF"
FT                       /AA_IMGT="AA 27 to 38, AA 32 and 33 are missing"
FT   FR2-IMGT            536970..537022
FT                       /translation="WSCIHQRPHGRYWSGSG"
FT                       /IMGT_note="frameshift in FR2-IMGT: insertion of 2 nt"
FT                       /out_of_frame
FT   CDR2-IMGT           537023..537043
FT                       /translation="IGHGGST"
FT                       /AA_IMGT="AA 56 to 65, AA 60, 61 and 62 are missing"
FT                       /out_of_frame
FT                       /restored_translation
FT   FR3-IMGT            537044..537157
FT                       /AA_IMGT="AA 66 to 104, AA 73 is missing"
FT                       /out_of_frame
FT                       /restored_translation
FT   3'V-REGION          537155..537165
FT                       /translation="RAK"
FT                       /out_of_frame
FT                       /restored_translation
FT   CDR3-IMGT           537158..537165
FT                       /translation="AK"
FT                       /AA_IMGT="AA 105 to 106"
FT                       /out_of_frame
FT                       /restored_translation
FT   3'UTR               537166..540758
FT   V-RS                537166..537204
FT   V-HEPTAMER          537166..537172
FT   V-SPACER            537173..537195
FT   V-NONAMER           537196..537204
FT   V-GENE              540759..546000
FT                       /functional
FT                       /IMGT_allele="IGHV3-30*18"
FT                       /IMGT_gene="IGHV3-30"
FT   5'UTR               540759..544311
FT   L-V-GENE-UNIT       544312..544804
FT                       /functional
FT                       /IMGT_allele="IGHV3-30*18"
FT                       /IMGT_gene="IGHV3-30"
FT   L-INTRON-L          544312..544469
FT   L-PART1             544312..544357
FT                       /translation="MEFGLSWVFLVALLR"
FT   INIT-CODON          544312..544314
FT   DONOR-SPLICE        544357..544359
FT   V-INTRON            544358..544458
FT   ACCEPTOR-SPLICE     544456..544460
FT   V-EXON              544459..544765
FT                       /codon_start=3
FT                       /translation="VQCQVQLVESGGGVVQPGRSLRLSCAASGFTFSSYGMHWVR
FT                       QAPGKGLEWVAVISYDGSNKYYADSVKGRFTISRDNSKNTLYLQMNSLRAEDTAV
FT                       YYCAK"
FT   L-PART2             544459..544469
FT                       /codon_start=3
FT                       /translation="VQC"
FT   V-REGION            544470..544765
FT                       /translation="QVQLVESGGGVVQPGRSLRLSCAASGFTFSSYGMHWVRQAP
FT                       GKGLEWVAVISYDGSNKYYADSVKGRFTISRDNSKNTLYLQMNSLRAEDTAVYYC
FT                       AK"
FT                       /CDR_length="[8.8.2]"
FT                       /FR_length="[25.17.38.G]"
FT                       /functional
FT                       /IMGT_allele="IGHV3-30*18"
FT                       /IMGT_gene="IGHV3-30"
FT   FR1-IMGT            544470..544544
FT                       /translation="QVQLVESGGGVVQPGRSLRLSCAAS"
FT                       /AA_IMGT="AA 1 to 26, AA 10 is missing"
FT   1st-CYS             544533..544535
FT   CDR1-IMGT           544545..544568
FT                       /translation="GFTFSSYG"
FT                       /AA_IMGT="AA 27 to 38, AA 31, 32, 33 and 34 are 
FT                       missing"
FT   FR2-IMGT            544569..544619
FT                       /translation="MHWVRQAPGKGLEWVAV"
FT                       /AA_IMGT="AA 39 to 55"
FT   CONSERVED-TRP       544575..544577
FT   CDR2-IMGT           544620..544643
FT                       /translation="ISYDGSNK"
FT                       /AA_IMGT="AA 56 to 65, AA 60 and 61 are missing"
FT   FR3-IMGT            544644..544757
FT                       /translation="YYADSVKGRFTISRDNSKNTLYLQMNSLRAEDTAVYYC"
FT                       /AA_IMGT="AA 66 to 104, AA 73 is missing"
FT   3'V-REGION          544755..544765
FT                       /translation="CAK"
FT   2nd-CYS             544755..544757
FT   CDR3-IMGT           544758..544765
FT                       /translation="AK"
FT                       /AA_IMGT="AA 105 to 106"
FT   3'UTR               544766..546000
FT   V-RS                544766..544804
FT   V-HEPTAMER          544766..544772
FT   V-SPACER            544773..544795
FT   V-NONAMER           544796..544804
FT   GENE                546001..548000
FT                       /IMGT_gene="GOLGA4P2"
FT                       /pseudo
FT   5'UTR               546001..546763
FT   GENE-UNIT           546764..547609
FT                       /IMGT_gene="GOLGA4P2"
FT                       /pseudo
FT   3'UTR               547610..548000
FT   V-GENE              548001..549475
FT                       /IMGT_allele="IGHV3-29*01"
FT                       /IMGT_gene="IGHV3-29"
FT                       /pseudo="STOP-CODON in L-PART1, several STOP-CODON in 
FT                       V-REGION"
FT   5'UTR               548001..548281
FT   L-V-GENE-UNIT       548282..548778
FT                       /IMGT_allele="IGHV3-29*01"
FT                       /IMGT_gene="IGHV3-29"
FT                       /pseudo="STOP-CODON in L-PART1, several STOP-CODON in 
FT                       V-REGION"
FT   L-INTRON-L          548282..548441
FT   L-PART1             548282..548327
FT                       /IMGT_note="STOP-CODON in L-PART1"
FT   INIT-CODON          548282..548284
FT   DONOR-SPLICE        548327..548329
FT   V-INTRON            548328..548430
FT   ACCEPTOR-SPLICE     548428..548432
FT   V-EXON              548431..548739
FT                       /codon_start=3
FT   L-PART2             548431..548441
FT                       /codon_start=3
FT                       /translation="VQC"
FT   V-REGION            548442..548739
FT                       /CDR_length="[8.8.3]"
FT                       /FR_length="[25.17.38.G]"
FT                       /IMGT_allele="IGHV3-29*01"
FT                       /IMGT_gene="IGHV3-29"
FT                       /pseudo="STOP-CODON in L-PART1, several STOP-CODON in 
FT                       V-REGION"
FT   FR1-IMGT            548442..548516
FT                       /translation="EVELIEPTEDLRQPGKFLRLSCVAS"
FT                       /AA_IMGT="AA 1 to 26, AA 10 is missing"
FT   1st-CYS             548505..548507
FT   CDR1-IMGT           548517..548540
FT                       /AA_IMGT="AA 27 to 38, AA 31, 32, 33 and 34 are 
FT                       missing"
FT                       /IMGT_note="STOP-CODON in CDR1-IMGT: position 38"
FT   FR2-IMGT            548541..548591
FT                       /AA_IMGT="AA 39 to 55"
FT                       /IMGT_note="no CONSERVED-TRP: Pro instead of Trp, 
FT                       STOP-CODON in FR2-IMGT: position 52"
FT   CDR2-IMGT           548592..548615
FT                       /translation="IKDDGSQI"
FT                       /AA_IMGT="AA 56 to 65, AA 60 and 61 are missing"
FT   FR3-IMGT            548616..548729
FT                       /translation="HHADSVKGRFSISKDNAKNSLYLQMNSQRTEDMAVYGC"
FT                       /AA_IMGT="AA 66 to 104, AA 73 is missing"
FT   3'V-REGION          548727..548739
FT   2nd-CYS             548727..548729
FT   CDR3-IMGT           548730..548739
FT                       /AA_IMGT="AA 105 to 107"
FT                       /IMGT_note="STOP-CODON in CDR3-IMGT: position 106"
FT   3'UTR               548740..549475
FT   V-RS                548740..548778
FT   V-HEPTAMER          548740..548746
FT                       /IMGT_note="noncanonical V-HEPTAMER: ccaagtg instead 
FT                       of cacagtg"
FT   V-SPACER            548747..548769
FT   V-NONAMER           548770..548778
FT   V-GENE              549476..552807
FT                       /IMGT_allele="IGHV(II)-28-1*03"
FT                       /IMGT_gene="IGHV(II)-28-1"
FT                       /pseudo="truncated pseudogene not assigned to 
FT                       subgroups with functional genes"
FT   5'UTR               549476..550157
FT   V-REGION            <550158..550429
FT                       /partial
FT                       /CDR_length="[10.7.2]"
FT                       /FR_length="[16.17.X.G]"
FT                       /IMGT_allele="IGHV(II)-28-1*03"
FT                       /IMGT_gene="IGHV(II)-28-1"
FT                       /IMGT_note="partial gene in 5', restored translation 
FT                       in 3'V-REGION and CDR3-IMGT"
FT                       /out_of_frame
FT                       /pseudo="truncated pseudogene not assigned to 
FT                       subgroups with functional genes"
FT                       /putative_limit="5'"
FT   FR1-IMGT            <550158..550205
FT                       /partial
FT                       /translation="WLETSLQTLSLTCTVS"
FT                       /putative_limit="5'"
FT   1st-CYS             550194..550196
FT   CDR1-IMGT           550206..550235
FT                       /translation="GFPIITSVSC"
FT                       /AA_IMGT="AA 27 to 38, AA 32 and 33 are missing"
FT   FR2-IMGT            550236..550286
FT                       /AA_IMGT="AA 39 to 55"
FT                       /IMGT_note="STOP-CODON in FR2-IMGT: position 39, no 
FT                       CONSERVED-TRP: Cys instead of Trp"
FT   CDR2-IMGT           550287..550307
FT                       /translation="MGCEGST"
FT                       /AA_IMGT="AA 56 to 65, AA 60, 61 and 62 are missing"
FT   FR3-IMGT            550308..550421
FT                       /AA_IMGT="AA 66 to 104, AA 73 is missing"
FT                       /IMGT_note="frameshift in FR3-IMGT: deletion of 13 nt, 
FT                       deletions in FR3-IMGT: 1 AA is missing"
FT                       /out_of_frame
FT   3'V-REGION          550419..550429
FT                       /out_of_frame
FT                       /restored_translation
FT   CDR3-IMGT           550422..550429
FT                       /translation="RA"
FT                       /AA_IMGT="AA 105 to 106"
FT                       /out_of_frame
FT                       /restored_translation
FT   3'UTR               550430..552807
FT   V-RS                550430..550452
FT   V-HEPTAMER          550430..550436
FT                       /IMGT_note="noncanonical V-HEPTAMER: cattgtg instead 
FT                       of cacagtg"
FT   V-SPACER            550437..550443
FT                       /IMGT_note="unexpected V-SPACER length: 7 nt instead 
FT                       of 23 nt"
FT   V-NONAMER           550444..550452
FT   V-GENE              552808..558619
FT                       /functional
FT                       /IMGT_allele="IGHV4-28*07"
FT                       /IMGT_gene="IGHV4-28"
FT   5'UTR               552808..555156
FT   L-V-GENE-UNIT       555157..555630
FT                       /functional
FT                       /IMGT_allele="IGHV4-28*07"
FT                       /IMGT_gene="IGHV4-28"
FT   L-INTRON-L          555157..555295
FT   L-PART1             555157..555202
FT                       /translation="MKHLWFFLLLVAAPR"
FT   INIT-CODON          555157..555159
FT   DONOR-SPLICE        555202..555204
FT   V-INTRON            555203..555284
FT   ACCEPTOR-SPLICE     555282..555286
FT   V-EXON              555285..555591
FT                       /codon_start=3
FT                       /translation="VLSQVQLQESGPGLVKPSDTLSLTCAVSGYSISSSNWWGWI
FT                       RQPPGKGLEWIGYIYYSGSTYYNPSLKSRVTMSVDTSKNQFSLKLSSVTAVDTAV
FT                       YYCAR"
FT   L-PART2             555285..555295
FT                       /codon_start=3
FT                       /translation="VLS"
FT   V-REGION            555296..555591
FT                       /translation="QVQLQESGPGLVKPSDTLSLTCAVSGYSISSSNWWGWIRQP
FT                       PGKGLEWIGYIYYSGSTYYNPSLKSRVTMSVDTSKNQFSLKLSSVTAVDTAVYYC
FT                       AR"
FT                       /CDR_length="[9.7.2]"
FT                       /FR_length="[25.17.38.G]"
FT                       /functional
FT                       /IMGT_allele="IGHV4-28*07"
FT                       /IMGT_gene="IGHV4-28"
FT   FR1-IMGT            555296..555370
FT                       /translation="QVQLQESGPGLVKPSDTLSLTCAVS"
FT                       /AA_IMGT="AA 1 to 26, AA 10 is missing"
FT   1st-CYS             555359..555361
FT   CDR1-IMGT           555371..555397
FT                       /translation="GYSISSSNW"
FT                       /AA_IMGT="AA 27 to 38, AA 32, 33 and 34 are missing"
FT   FR2-IMGT            555398..555448
FT                       /translation="WGWIRQPPGKGLEWIGY"
FT                       /AA_IMGT="AA 39 to 55"
FT   CONSERVED-TRP       555404..555406
FT   CDR2-IMGT           555449..555469
FT                       /translation="IYYSGST"
FT                       /AA_IMGT="AA 56 to 65, AA 60, 61 and 62 are missing"
FT   FR3-IMGT            555470..555583
FT                       /translation="YYNPSLKSRVTMSVDTSKNQFSLKLSSVTAVDTAVYYC"
FT                       /AA_IMGT="AA 66 to 104, AA 73 is missing"
FT   3'V-REGION          555581..555591
FT                       /translation="CAR"
FT   2nd-CYS             555581..555583
FT   CDR3-IMGT           555584..555591
FT                       /translation="AR"
FT                       /AA_IMGT="AA 105 to 106"
FT   3'UTR               555592..558619
FT   V-RS                555592..555630
FT   V-HEPTAMER          555592..555598
FT   V-SPACER            555599..555621
FT   V-NONAMER           555622..555630
FT   V-GENE              558620..563483
FT                       /IMGT_allele="IGHV7-27*01"
FT                       /IMGT_gene="IGHV7-27"
FT                       /pseudo="STOP-CODON in V-REGION: position 43, no V-RS"
FT   5'UTR               558620..561608
FT   L-INTRON-L          561609..561749
FT   L-PART1             561609..561654
FT                       /translation="MYWTWRILFLVAAAT"
FT   INIT-CODON          561609..561611
FT   DONOR-SPLICE        561654..561656
FT   V-INTRON            561655..561738
FT   ACCEPTOR-SPLICE     561736..561740
FT   V-EXON              561739..562044
FT                       /codon_start=3
FT                       /putative_limit="3'"
FT   L-PART2             561739..561749
FT                       /codon_start=3
FT                       /translation="VHS"
FT   V-REGION            561750..562044
FT                       /CDR_length="[8.8.2]"
FT                       /FR_length="[25.17.38.G]"
FT                       /IMGT_allele="IGHV7-27*01"
FT                       /IMGT_gene="IGHV7-27"
FT                       /pseudo="STOP-CODON in V-REGION: position 43, no V-RS"
FT                       /putative_limit="3'"
FT   FR1-IMGT            561750..561824
FT                       /translation="QVQLVQSGPEVKKPGASLKVSCKAS"
FT                       /AA_IMGT="AA 1 to 26, AA 10 is missing"
FT   1st-CYS             561813..561815
FT   CDR1-IMGT           561825..561848
FT                       /translation="GYTFTSYA"
FT                       /AA_IMGT="AA 27 to 38, AA 31, 32, 33 and 34 are 
FT                       missing"
FT   FR2-IMGT            561849..561899
FT                       /AA_IMGT="AA 39 to 55"
FT                       /IMGT_note="STOP-CODON in FR2-IMGT: position 43"
FT   CONSERVED-TRP       561855..561857
FT   CDR2-IMGT           561900..561923
FT                       /translation="INTNTGNL"
FT                       /AA_IMGT="AA 56 to 65, AA 60 and 61 are missing"
FT   FR3-IMGT            561924..562037
FT                       /translation="TYAQGFTGRFVFSMDTSVSMAYLHISSLKAEDTCKRHS"
FT                       /AA_IMGT="AA 66 to 104, AA 73 is missing"
FT                       /IMGT_note="no 2nd-CYS: Ser instead of Cys"
FT   3'V-REGION          562035..562044
FT                       /translation="SVE"
FT                       /putative_limit="3'"
FT   CDR3-IMGT           562038..562044
FT                       /translation="VE"
FT                       /AA_IMGT="AA 105 to 106"
FT                       /putative_limit="3'"
FT   3'UTR               562045..563483
FT   V-GENE              563484..567650
FT                       /IMGT_allele="IGHV(II)-26-2*01"
FT                       /IMGT_gene="IGHV(II)-26-2"
FT                       /pseudo="no L-PART1, frameshifts in V-REGION: several 
FT                       insertions and deletions"
FT   5'UTR               563484..564884
FT   ACCEPTOR-SPLICE     564882..564886
FT   V-EXON              564885..565197
FT                       /codon_start=3
FT                       /out_of_frame
FT   L-PART2             564885..564895
FT                       /codon_start=3
FT                       /translation="VLS"
FT   V-REGION            564896..565197
FT                       /CDR_length="[X.10.2]"
FT                       /FR_length="[X.17.38.G]"
FT                       /IMGT_allele="IGHV(II)-26-2*01"
FT                       /IMGT_gene="IGHV(II)-26-2"
FT                       /IMGT_note="restored translation in CDR1-IMGT, 
FT                       FR2-IMGT, CDR2-IMGT, FR3-IMGT, 3'V-REGION and 
FT                       CDR3-IMGT"
FT                       /out_of_frame
FT                       /pseudo="no L-PART1, frameshifts in V-REGION: several 
FT                       insertions and deletions"
FT   FR1-IMGT            564896..564968
FT                       /translation="QVQLQESGPGPMRPSDTALCLCCV"
FT                       /IMGT_note="frameshift in FR1-IMGT: deletion of 2 nt"
FT                       /out_of_frame
FT   CDR1-IMGT           564969..564996
FT                       /translation="GFFFLPWIL"
FT                       /IMGT_note="frameshift in CDR1-IMGT: insertion of 1 nt"
FT                       /out_of_frame
FT                       /restored_translation
FT   FR2-IMGT            564997..565047
FT                       /AA_IMGT="AA 39 to 55"
FT                       /out_of_frame
FT                       /restored_translation
FT   CDR2-IMGT           565048..565077
FT                       /AA_IMGT="AA 56 to 65"
FT                       /out_of_frame
FT                       /restored_translation
FT   FR3-IMGT            565078..565191
FT                       /AA_IMGT="AA 66 to 104, AA 73 is missing"
FT                       /out_of_frame
FT                       /restored_translation
FT   3'V-REGION          565189..565197
FT                       /translation="CER"
FT                       /out_of_frame
FT                       /restored_translation
FT   CDR3-IMGT           565192..565197
FT                       /translation="ER"
FT                       /AA_IMGT="AA 105 to 106"
FT                       /out_of_frame
FT                       /restored_translation
FT   3'UTR               565198..567650
FT   V-RS                565198..565236
FT   V-HEPTAMER          565198..565204
FT   V-SPACER            565205..565227
FT   V-NONAMER           565228..565236
FT   V-GENE              567651..574242
FT                       /IMGT_allele="IGHV(III)-26-1*02"
FT                       /IMGT_gene="IGHV(III)-26-1"
FT                       /pseudo="truncated pseudogene not assigned to 
FT                       subgroups with functional genes"
FT   5'UTR               567651..570180
FT   V-REGION            <570181..570439
FT                       /partial
FT                       /CDR_length="[8.3.2]"
FT                       /FR_length="[13.X.X.G]"
FT                       /IMGT_allele="IGHV(III)-26-1*02"
FT                       /IMGT_gene="IGHV(III)-26-1"
FT                       /IMGT_note="partial gene in 5', restored translation 
FT                       in CDR2-IMGT, FR3-IMGT, 3'V-REGION and CDR3-IMGT"
FT                       /out_of_frame
FT                       /pseudo="truncated pseudogene not assigned to 
FT                       subgroups with functional genes"
FT                       /putative_limit="5'"
FT   FR1-IMGT            <570181..570219
FT                       /partial
FT                       /translation="QPSSSLRLSCKAS"
FT                       /putative_limit="5'"
FT   1st-CYS             570208..570210
FT   CDR1-IMGT           570220..570243
FT                       /translation="GFGFTGCS"
FT                       /AA_IMGT="AA 27 to 38, AA 31, 32, 33 and 34 are 
FT                       missing"
FT   FR2-IMGT            570244..570293
FT                       /translation="MSLVQHTSQQELVWVA"
FT                       /IMGT_note="frameshift in FR2-IMGT: deletion of 16 nt"
FT                       /out_of_frame
FT   CDR2-IMGT           570294..570302
FT                       /translation="SER"
FT                       /AA_IMGT="AA 56 to 65, AA 58, 59, 60, 61, 62, 63 and 
FT                       64 are missing"
FT                       /out_of_frame
FT                       /restored_translation
FT   FR3-IMGT            570303..570433
FT                       /IMGT_note="frameshift in FR3-IMGT: insertion of 17 nt"
FT                       /out_of_frame
FT                       /restored_translation
FT   3'V-REGION          570431..570439
FT                       /translation="SEG"
FT                       /out_of_frame
FT                       /restored_translation
FT   CDR3-IMGT           570434..570439
FT                       /translation="EG"
FT                       /AA_IMGT="AA 105 to 106"
FT                       /out_of_frame
FT                       /restored_translation
FT   3'UTR               570440..574242
FT   V-RS                570440..570479
FT   V-HEPTAMER          570440..570446
FT   V-SPACER            570447..570470
FT   V-NONAMER           570471..570479
FT   V-GENE              574243..582000
FT                       /functional
FT                       /IMGT_allele="IGHV2-26*01"
FT                       /IMGT_gene="IGHV2-26"
FT   5'UTR               574243..578006
FT   L-V-GENE-UNIT       578007..578489
FT                       /functional
FT                       /IMGT_allele="IGHV2-26*01"
FT                       /IMGT_gene="IGHV2-26"
FT   L-INTRON-L          578007..578149
FT   L-PART1             578007..578052
FT                       /translation="MDTLCYTLLLLTTPS"
FT   INIT-CODON          578007..578009
FT   DONOR-SPLICE        578052..578054
FT   V-INTRON            578053..578138
FT   ACCEPTOR-SPLICE     578136..578140
FT   V-EXON              578139..578450
FT                       /codon_start=3
FT                       /translation="VLSQVTLKESGPVLVKPTETLTLTCTVSGFSLSNARMGVSW
FT                       IRQPPGKALEWLAHIFSNDEKSYSTSLKSRLTISKDTSKSQVVLTMTNMDPVDTA
FT                       TYYCARI"
FT   L-PART2             578139..578149
FT                       /codon_start=3
FT                       /translation="VLS"
FT   V-REGION            578150..578450
FT                       /translation="QVTLKESGPVLVKPTETLTLTCTVSGFSLSNARMGVSWIRQ
FT                       PPGKALEWLAHIFSNDEKSYSTSLKSRLTISKDTSKSQVVLTMTNMDPVDTATYY
FT                       CARI"
FT                       /CDR_length="[10.7.3]"
FT                       /FR_length="[25.17.38.G]"
FT                       /functional
FT                       /IMGT_allele="IGHV2-26*01"
FT                       /IMGT_gene="IGHV2-26"
FT   FR1-IMGT            578150..578224
FT                       /translation="QVTLKESGPVLVKPTETLTLTCTVS"
FT                       /AA_IMGT="AA 1 to 26, AA 10 is missing"
FT   1st-CYS             578213..578215
FT   CDR1-IMGT           578225..578254
FT                       /translation="GFSLSNARMG"
FT                       /AA_IMGT="AA 27 to 38, AA 32 and 33 are missing"
FT   FR2-IMGT            578255..578305
FT                       /translation="VSWIRQPPGKALEWLAH"
FT                       /AA_IMGT="AA 39 to 55"
FT   CONSERVED-TRP       578261..578263
FT   CDR2-IMGT           578306..578326
FT                       /translation="IFSNDEK"
FT                       /AA_IMGT="AA 56 to 65, AA 60, 61 and 62 are missing"
FT   FR3-IMGT            578327..578440
FT                       /translation="SYSTSLKSRLTISKDTSKSQVVLTMTNMDPVDTATYYC"
FT                       /AA_IMGT="AA 66 to 104, AA 73 is missing"
FT   3'V-REGION          578438..578450
FT                       /translation="CARI"
FT   2nd-CYS             578438..578440
FT   CDR3-IMGT           578441..578450
FT                       /translation="ARI"
FT                       /AA_IMGT="AA 105 to 107"
FT   3'UTR               578451..582000
FT   V-RS                578451..578489
FT   V-HEPTAMER          578451..578457
FT   V-SPACER            578458..578480
FT   V-NONAMER           578481..578489
FT   V-GENE              582001..588350
FT                       /IMGT_allele="IGHV(III)-25-1*02"
FT                       /IMGT_gene="IGHV(III)-25-1"
FT                       /pseudo="truncated pseudogene not assigned to 
FT                       subgroups with functional genes"
FT   5'UTR               582001..586037
FT   ACCEPTOR-SPLICE     586035..586039
FT   V-EXON              586038..586328
FT                       /codon_start=3
FT                       /out_of_frame
FT                       /putative_limit="3'"
FT   L-PART2             586038..586048
FT                       /codon_start=3
FT                       /translation="VLC"
FT   V-REGION            586049..>586328
FT                       /partial
FT                       /CDR_length="[8.8.X]"
FT                       /FR_length="[X.17.36.G]"
FT                       /IMGT_allele="IGHV(III)-25-1*02"
FT                       /IMGT_gene="IGHV(III)-25-1"
FT                       /IMGT_note="partial gene in 3', restored translation 
FT                       in CDR1-IMGT, FR2-IMGT, CDR2-IMGT, FR3-IMGT"
FT                       /out_of_frame
FT                       /pseudo="truncated pseudogene not assigned to 
FT                       subgroups with functional genes"
FT                       /putative_limit="3'"
FT   FR1-IMGT            586049..586121
FT                       /IMGT_note="frameshifts in FR1-IMGT: deletion of 1 nt 
FT                       and deletion of 1 nt"
FT                       /out_of_frame
FT   CDR1-IMGT           586122..586145
FT                       /translation="GFTFTDCS"
FT                       /AA_IMGT="AA 27 to 38, AA 31, 32, 33 and 34 are 
FT                       missing"
FT                       /out_of_frame
FT                       /restored_translation
FT   FR2-IMGT            586146..586196
FT                       /translation="ISLVQQAPGPGLMWAAT"
FT                       /AA_IMGT="AA 39 to 55"
FT                       /out_of_frame
FT                       /restored_translation
FT   CDR2-IMGT           586197..586220
FT                       /AA_IMGT="AA 56 to 65, AA 60 and 61 are missing"
FT                       /out_of_frame
FT                       /restored_translation
FT   FR3-IMGT            586221..586328
FT                       /AA_IMGT="AA 66 to 104, AA 73, 81 and 82 are missing"
FT                       /out_of_frame
FT                       /restored_translation
FT   3'UTR               586329..588350
FT   V-GENE              588351..596860
FT                       /IMGT_allele="IGHV3-25*03"
FT                       /IMGT_gene="IGHV3-25"
FT                       /pseudo="STOP-CODON in V-REGION: position 103"
FT   5'UTR               588351..590365
FT   L-V-GENE-UNIT       590366..590858
FT                       /IMGT_allele="IGHV3-25*03"
FT                       /IMGT_gene="IGHV3-25"
FT                       /pseudo="STOP-CODON in V-REGION: position 103"
FT   L-INTRON-L          590366..590523
FT   L-PART1             590366..590411
FT                       /translation="MEFVLSWVFLVAILK"
FT   INIT-CODON          590366..590368
FT   DONOR-SPLICE        590411..590413
FT   V-INTRON            590412..590512
FT   ACCEPTOR-SPLICE     590510..590514
FT   V-EXON              590513..590819
FT                       /codon_start=3
FT   L-PART2             590513..590523
FT                       /codon_start=3
FT                       /translation="IQC"
FT   V-REGION            590524..590819
FT                       /CDR_length="[8.8.2]"
FT                       /FR_length="[25.17.38.G]"
FT                       /IMGT_allele="IGHV3-25*03"
FT                       /IMGT_gene="IGHV3-25"
FT                       /pseudo="STOP-CODON in V-REGION: position 103"
FT   FR1-IMGT            590524..590598
FT                       /translation="EMQLVESGGGLAKPAWSPRLSCAAS"
FT                       /AA_IMGT="AA 1 to 26, AA 10 is missing"
FT   1st-CYS             590587..590589
FT   CDR1-IMGT           590599..590622
FT                       /translation="QFTFSSYY"
FT                       /AA_IMGT="AA 27 to 38, AA 31, 32, 33 and 34 are 
FT                       missing"
FT   FR2-IMGT            590623..590673
FT                       /translation="MNCVRQAPGNGLELVGQ"
FT                       /AA_IMGT="AA 39 to 55"
FT                       /IMGT_note="no CONSERVED-TRP: Cys instead of Trp"
FT   CDR2-IMGT           590674..590697
FT                       /translation="VNPNGGST"
FT                       /AA_IMGT="AA 56 to 65, AA 60 and 61 are missing"
FT   FR3-IMGT            590698..590811
FT                       /AA_IMGT="AA 66 to 104, AA 73 is missing"
FT                       /IMGT_note="STOP-CODON in FR3-IMGT: position 103"
FT   3'V-REGION          590809..590819
FT                       /translation="CTR"
FT   2nd-CYS             590809..590811
FT   CDR3-IMGT           590812..590819
FT                       /translation="TR"
FT                       /AA_IMGT="AA 105 to 106"
FT   3'UTR               590820..596860
FT   V-RS                590820..590858
FT   V-HEPTAMER          590820..590826
FT   V-SPACER            590827..590849
FT   V-NONAMER           590850..590858
FT   V-GENE              596861..607061
FT                       /functional="noncanonical DONOR-SPLICE: ngc instead of 
FT                       ngt, however this V-GENE has been seen rearranged in 
FT                       vivo"
FT                       /IMGT_allele="IGHV1-24*01"
FT                       /IMGT_gene="IGHV1-24"
FT   5'UTR               596861..602861
FT   L-V-GENE-UNIT       602862..603338
FT                       /functional="noncanonical DONOR-SPLICE: ngc instead of 
FT                       ngt, however this V-GENE has been seen rearranged in 
FT                       vivo"
FT                       /IMGT_allele="IGHV1-24*01"
FT                       /IMGT_gene="IGHV1-24"
FT   L-INTRON-L          602862..603003
FT   L-PART1             602862..602907
FT                       /translation="MDCTWRILFLVAAAT"
FT   INIT-CODON          602862..602864
FT   DONOR-SPLICE        602907..602909
FT                       /IMGT_note="noncanonical DONOR-SPLICE: ngc instead of 
FT                       ngt"
FT   V-INTRON            602908..602992
FT   ACCEPTOR-SPLICE     602990..602994
FT   V-EXON              602993..603299
FT                       /codon_start=3
FT                       /translation="THAQVQLVQSGAEVKKPGASVKVSCKVSGYTLTELSMHWVR
FT                       QAPGKGLEWMGGFDPEDGETIYAQKFQGRVTMTEDTSTDTAYMELSSLRSEDTAV
FT                       YYCAT"
FT   L-PART2             602993..603003
FT                       /codon_start=3
FT                       /translation="THA"
FT   V-REGION            603004..603299
FT                       /translation="QVQLVQSGAEVKKPGASVKVSCKVSGYTLTELSMHWVRQAP
FT                       GKGLEWMGGFDPEDGETIYAQKFQGRVTMTEDTSTDTAYMELSSLRSEDTAVYYC
FT                       AT"
FT                       /CDR_length="[8.8.2]"
FT                       /FR_length="[25.17.38.G]"
FT                       /functional="noncanonical DONOR-SPLICE: ngc instead of 
FT                       ngt, however this V-GENE has been seen rearranged in 
FT                       vivo"
FT                       /IMGT_allele="IGHV1-24*01"
FT                       /IMGT_gene="IGHV1-24"
FT   FR1-IMGT            603004..603078
FT                       /translation="QVQLVQSGAEVKKPGASVKVSCKVS"
FT                       /AA_IMGT="AA 1 to 26, AA 10 is missing"
FT   1st-CYS             603067..603069
FT   CDR1-IMGT           603079..603102
FT                       /translation="GYTLTELS"
FT                       /AA_IMGT="AA 27 to 38, AA 31, 32, 33 and 34 are 
FT                       missing"
FT   FR2-IMGT            603103..603153
FT                       /translation="MHWVRQAPGKGLEWMGG"
FT                       /AA_IMGT="AA 39 to 55"
FT   CONSERVED-TRP       603109..603111
FT   CDR2-IMGT           603154..603177
FT                       /translation="FDPEDGET"
FT                       /AA_IMGT="AA 56 to 65, AA 60 and 61 are missing"
FT   FR3-IMGT            603178..603291
FT                       /translation="IYAQKFQGRVTMTEDTSTDTAYMELSSLRSEDTAVYYC"
FT                       /AA_IMGT="AA 66 to 104, AA 73 is missing"
FT   3'V-REGION          603289..603299
FT                       /translation="CAT"
FT   2nd-CYS             603289..603291
FT   CDR3-IMGT           603292..603299
FT                       /translation="AT"
FT                       /AA_IMGT="AA 105 to 106"
FT   3'UTR               603300..607061
FT   V-RS                603300..603338
FT   V-HEPTAMER          603300..603306
FT   V-SPACER            603307..603329
FT   V-NONAMER           603330..603338
FT   V-GENE              607062..613184
FT                       /functional
FT                       /IMGT_allele="IGHV3-23*04"
FT                       /IMGT_gene="IGHV3-23"
FT   5'UTR               607062..610783
FT   L-V-GENE-UNIT       610784..611278
FT                       /functional
FT                       /IMGT_allele="IGHV3-23*04"
FT                       /IMGT_gene="IGHV3-23"
FT   L-INTRON-L          610784..610943
FT   L-PART1             610784..610829
FT                       /translation="MEFGLSWLFLVAILK"
FT   INIT-CODON          610784..610786
FT   DONOR-SPLICE        610829..610831
FT   V-INTRON            610830..610932
FT   ACCEPTOR-SPLICE     610930..610934
FT   V-EXON              610933..611239
FT                       /codon_start=3
FT                       /translation="VQCEVQLVESGGGLVQPGGSLRLSCAASGFTFSSYAMSWVR
FT                       QAPGKGLEWVSAISGSGGSTYYADSVKGRFTISRDNSKNTLYLQMNSLRAEDTAV
FT                       YYCAK"
FT   L-PART2             610933..610943
FT                       /codon_start=3
FT                       /translation="VQC"
FT   V-REGION            610944..611239
FT                       /translation="EVQLVESGGGLVQPGGSLRLSCAASGFTFSSYAMSWVRQAP
FT                       GKGLEWVSAISGSGGSTYYADSVKGRFTISRDNSKNTLYLQMNSLRAEDTAVYYC
FT                       AK"
FT                       /CDR_length="[8.8.2]"
FT                       /FR_length="[25.17.38.G]"
FT                       /functional
FT                       /IMGT_allele="IGHV3-23*04"
FT                       /IMGT_gene="IGHV3-23"
FT   FR1-IMGT            610944..611018
FT                       /translation="EVQLVESGGGLVQPGGSLRLSCAAS"
FT                       /AA_IMGT="AA 1 to 26, AA 10 is missing"
FT   1st-CYS             611007..611009
FT   CDR1-IMGT           611019..611042
FT                       /translation="GFTFSSYA"
FT                       /AA_IMGT="AA 27 to 38, AA 31, 32, 33 and 34 are 
FT                       missing"
FT   FR2-IMGT            611043..611093
FT                       /translation="MSWVRQAPGKGLEWVSA"
FT                       /AA_IMGT="AA 39 to 55"
FT   CONSERVED-TRP       611049..611051
FT   CDR2-IMGT           611094..611117
FT                       /translation="ISGSGGST"
FT                       /AA_IMGT="AA 56 to 65, AA 60 and 61 are missing"
FT   FR3-IMGT            611118..611231
FT                       /translation="YYADSVKGRFTISRDNSKNTLYLQMNSLRAEDTAVYYC"
FT                       /AA_IMGT="AA 66 to 104, AA 73 is missing"
FT   3'V-REGION          611229..611239
FT                       /translation="CAK"
FT   2nd-CYS             611229..611231
FT   CDR3-IMGT           611232..611239
FT                       /translation="AK"
FT                       /AA_IMGT="AA 105 to 106"
FT   3'UTR               611240..613184
FT   V-RS                611240..611278
FT   V-HEPTAMER          611240..611246
FT   V-SPACER            611247..611269
FT   V-NONAMER           611270..611278
FT   V-GENE              613185..>615688
FT                       /partial
FT                       /IMGT_allele="IGHV(III)-22-2*01"
FT                       /IMGT_gene="IGHV(III)-22-2"
FT                       /pseudo="Truncated pseudogenes not assigned to 
FT                       subgroups with functional genes."
FT   5'UTR               613185..614961
FT   L-INTRON-L          614962..615129
FT   L-PART1             614962..615016
FT                       /translation="MAHHRVRAELGFLVAILK"
FT   INIT-CODON          614962..614964
FT   DONOR-SPLICE        615016..615018
FT                       /IMGT_note="noncanonical DONOR-SPLICE: ngc instead of 
FT                       ngt"
FT   V-INTRON            615017..615118
FT   ACCEPTOR-SPLICE     615116..615120
FT   V-EXON              615119..>615159
FT                       /partial
FT                       /codon_start=3
FT   L-PART2             615119..615129
FT                       /codon_start=3
FT                       /IMGT_note="STOP-CODON in L-PART2"
FT   V-REGION            615130..>615159
FT                       /partial
FT                       /translation="EVQVVECRGE"
FT                       /IMGT_allele="IGHV(III)-22-2*01"
FT                       /IMGT_gene="IGHV(III)-22-2"
FT                       /pseudo="Truncated pseudogenes not assigned to 
FT                       subgroups with functional genes."
FT   3'UTR               615160..615688
FT   V-GENE              615689..619073
FT                       /IMGT_allele="IGHV(II)-22-1*01"
FT                       /IMGT_gene="IGHV(II)-22-1"
FT                       /pseudo="truncated pseudogene not assigned to 
FT                       subgroups with functional genes"
FT   5'UTR               615689..616216
FT   V-REGION            <616217..616485
FT                       /partial
FT                       /codon_start=3
FT                       /CDR_length="[9.X.2]"
FT                       /FR_length="[X.17.38.G]"
FT                       /IMGT_allele="IGHV(II)-22-1*01"
FT                       /IMGT_gene="IGHV(II)-22-1"
FT                       /IMGT_note="partial gene in 5', restored translation 
FT                       in FR3-IMGT, 3'V-REGION and CDR3-IMGT"
FT                       /out_of_frame
FT                       /pseudo="truncated pseudogene not assigned to 
FT                       subgroups with functional genes"
FT                       /putative_limit="5'"
FT   FR1-IMGT            <616217..616266
FT                       /partial
FT                       /codon_start=3
FT                       /translation="PGEALTDPPPGLWHLC"
FT                       /IMGT_note="no 1st-CYS: Trp instead of Cys"
FT                       /putative_limit="5'"
FT   CDR1-IMGT           616267..616293
FT                       /translation="FSITTSASC"
FT                       /AA_IMGT="AA 27 to 38, AA 32, 33 and 34 are missing"
FT   FR2-IMGT            616294..616344
FT                       /translation="WSWIRHPPGKGLERIRC"
FT                       /AA_IMGT="AA 39 to 55"
FT   CONSERVED-TRP       616300..616302
FT   CDR2-IMGT           616345..616364
FT                       /translation="TGHEGV"
FT                       /IMGT_note="frameshift in CDR2-IMGT: deletion of 4 nt"
FT                       /out_of_frame
FT   FR3-IMGT            616365..616478
FT                       /translation="QSNPLLKSPVTISRSTSKKQFLLQLSYLNKEYTTMIFI"
FT                       /AA_IMGT="AA 66 to 104, AA 73 is missing"
FT                       /out_of_frame
FT                       /restored_translation
FT   3'V-REGION          616476..616485
FT                       /translation="IQK"
FT                       /out_of_frame
FT                       /restored_translation
FT   CDR3-IMGT           616479..616485
FT                       /translation="QK"
FT                       /AA_IMGT="AA 105 to 106"
FT                       /out_of_frame
FT                       /restored_translation
FT   3'UTR               616486..619073
FT   V-RS                616486..616525
FT   V-HEPTAMER          616486..616492
FT   V-SPACER            616493..616516
FT   V-NONAMER           616517..616525
FT                       /IMGT_note="noncanonical V-NONAMER: acactctac instead 
FT                       of acacaaacc"
FT   V-GENE              619074..633000
FT                       /IMGT_allele="IGHV3-22*02"
FT                       /IMGT_gene="IGHV3-22"
FT                       /pseudo="STOP-CODON in V-REGION: position 67"
FT   5'UTR               619074..621621
FT   L-V-GENE-UNIT       621622..622122
FT                       /IMGT_allele="IGHV3-22*02"
FT                       /IMGT_gene="IGHV3-22"
FT                       /pseudo="STOP-CODON in V-REGION: position 67"
FT   L-INTRON-L          621622..621781
FT   L-PART1             621622..621667
FT                       /translation="MESWLSWVFLAAILK"
FT   INIT-CODON          621622..621624
FT   DONOR-SPLICE        621667..621669
FT   V-INTRON            621668..621770
FT   ACCEPTOR-SPLICE     621768..621772
FT   V-EXON              621771..622083
FT                       /codon_start=3
FT   L-PART2             621771..621781
FT                       /codon_start=3
FT                       /translation="VQC"
FT   V-REGION            621782..622083
FT                       /CDR_length="[8.10.2]"
FT                       /FR_length="[25.17.38.G]"
FT                       /IMGT_allele="IGHV3-22*02"
FT                       /IMGT_gene="IGHV3-22"
FT                       /pseudo="STOP-CODON in V-REGION: position 67"
FT   FR1-IMGT            621782..621856
FT                       /translation="EVQLVESGGGLVQPGGSLRLSCAAS"
FT                       /AA_IMGT="AA 1 to 26, AA 10 is missing"
FT   1st-CYS             621845..621847
FT   CDR1-IMGT           621857..621880
FT                       /translation="GFTFSYYY"
FT                       /AA_IMGT="AA 27 to 38, AA 31, 32, 33 and 34 are 
FT                       missing"
FT   FR2-IMGT            621881..621931
FT                       /translation="MSGVRQAPGKGLEWVGF"
FT                       /AA_IMGT="AA 39 to 55"
FT                       /IMGT_note="no CONSERVED-TRP: Gly instead of Trp"
FT   CDR2-IMGT           621932..621961
FT                       /translation="IRNKANGGTT"
FT                       /AA_IMGT="AA 56 to 65"
FT   FR3-IMGT            621962..622075
FT                       /AA_IMGT="AA 66 to 104, AA 73 is missing"
FT                       /IMGT_note="STOP-CODON in FR3-IMGT: position 67"
FT   3'V-REGION          622073..622083
FT                       /translation="CSR"
FT   2nd-CYS             622073..622075
FT   CDR3-IMGT           622076..622083
FT                       /translation="SR"
FT                       /AA_IMGT="AA 105 to 106"
FT   3'UTR               622084..633000
FT   V-RS                622084..622122
FT   V-HEPTAMER          622084..622090
FT   V-SPACER            622091..622113
FT   V-NONAMER           622114..622122
FT   V-GENE              633001..655000
FT                       /functional
FT                       /IMGT_allele="IGHV3-21*01"
FT                       /IMGT_gene="IGHV3-21"
FT   5'UTR               633001..644329
FT   L-V-GENE-UNIT       644330..644822
FT                       /functional
FT                       /IMGT_allele="IGHV3-21*01"
FT                       /IMGT_gene="IGHV3-21"
FT   L-INTRON-L          644330..644487
FT   L-PART1             644330..644375
FT                       /translation="MELGLRWVFLVAILE"
FT   INIT-CODON          644330..644332
FT   DONOR-SPLICE        644375..644377
FT   V-INTRON            644376..644476
FT   ACCEPTOR-SPLICE     644474..644478
FT   V-EXON              644477..644783
FT                       /codon_start=3
FT                       /translation="VQCEVQLVESGGGLVKPGGSLRLSCAASGFTFSSYSMNWVR
FT                       QAPGKGLEWVSSISSSSSYIYYADSVKGRFTISRDNAKNSLYLQMNSLRAEDTAV
FT                       YYCAR"
FT   L-PART2             644477..644487
FT                       /codon_start=3
FT                       /translation="VQC"
FT   V-REGION            644488..644783
FT                       /translation="EVQLVESGGGLVKPGGSLRLSCAASGFTFSSYSMNWVRQAP
FT                       GKGLEWVSSISSSSSYIYYADSVKGRFTISRDNAKNSLYLQMNSLRAEDTAVYYC
FT                       AR"
FT                       /CDR_length="[8.8.2]"
FT                       /FR_length="[25.17.38.G]"
FT                       /functional
FT                       /IMGT_allele="IGHV3-21*01"
FT                       /IMGT_gene="IGHV3-21"
FT   FR1-IMGT            644488..644562
FT                       /translation="EVQLVESGGGLVKPGGSLRLSCAAS"
FT                       /AA_IMGT="AA 1 to 26, AA 10 is missing"
FT   1st-CYS             644551..644553
FT   CDR1-IMGT           644563..644586
FT                       /translation="GFTFSSYS"
FT                       /AA_IMGT="AA 27 to 38, AA 31, 32, 33 and 34 are 
FT                       missing"
FT   FR2-IMGT            644587..644637
FT                       /translation="MNWVRQAPGKGLEWVSS"
FT                       /AA_IMGT="AA 39 to 55"
FT   CONSERVED-TRP       644593..644595
FT   CDR2-IMGT           644638..644661
FT                       /translation="ISSSSSYI"
FT                       /AA_IMGT="AA 56 to 65, AA 60 and 61 are missing"
FT   FR3-IMGT            644662..644775
FT                       /translation="YYADSVKGRFTISRDNAKNSLYLQMNSLRAEDTAVYYC"
FT                       /AA_IMGT="AA 66 to 104, AA 73 is missing"
FT   3'V-REGION          644773..644783
FT                       /translation="CAR"
FT   2nd-CYS             644773..644775
FT   CDR3-IMGT           644776..644783
FT                       /translation="AR"
FT                       /AA_IMGT="AA 105 to 106"
FT   3'UTR               644784..655000
FT   V-RS                644784..644822
FT   V-HEPTAMER          644784..644790
FT   V-SPACER            644791..644813
FT   V-NONAMER           644814..644822
FT   MISC_FEATURE        655001..667500
FT                       /IMGT_note="the gene IGHV(II)-20-1 is only represented 
FT                       by a V-RS. Its V-REGION is replaced by the Alu 
FT                       Homo-sapiens_IGH_Alu-20-1 preceded by an undetermined 
FT                       region and the Alu Homo-sapiens_IGH_Alu-20-3"
FT   V-GENE              667501..675857
FT                       /IMGT_allele="IGHV3-20*02"
FT                       /IMGT_gene="IGHV3-20"
FT                       /ORF="no 1st-CYS: Phe instead of Cys"
FT   5'UTR               667501..668453
FT   L-V-GENE-UNIT       668454..668948
FT                       /IMGT_allele="IGHV3-20*02"
FT                       /IMGT_gene="IGHV3-20"
FT                       /ORF="no 1st-CYS: Phe instead of Cys"
FT   L-INTRON-L          668454..668613
FT   L-PART1             668454..668499
FT                       /translation="MEFGLSWVFLVAILK"
FT   INIT-CODON          668454..668456
FT   DONOR-SPLICE        668499..668501
FT   V-INTRON            668500..668602
FT   ACCEPTOR-SPLICE     668600..668604
FT   V-EXON              668603..668909
FT                       /codon_start=3
FT                       /translation="VQCEVQLVESGGGVVRPGGSLRLSFAASGFTFDDYGMSWVR
FT                       QAPGKGLEWVSGINWNGGSTGYADSVKGRFTISRDNAKNSLYLQMNSLRAEDTAL
FT                       YHCAR"
FT   L-PART2             668603..668613
FT                       /codon_start=3
FT                       /translation="VQC"
FT   V-REGION            668614..668909
FT                       /translation="EVQLVESGGGVVRPGGSLRLSFAASGFTFDDYGMSWVRQAP
FT                       GKGLEWVSGINWNGGSTGYADSVKGRFTISRDNAKNSLYLQMNSLRAEDTALYHC
FT                       AR"
FT                       /CDR_length="[8.8.2]"
FT                       /FR_length="[25.17.38.G]"
FT                       /IMGT_allele="IGHV3-20*02"
FT                       /IMGT_gene="IGHV3-20"
FT                       /ORF="no 1st-CYS: Phe instead of Cys"
FT   FR1-IMGT            668614..668688
FT                       /translation="EVQLVESGGGVVRPGGSLRLSFAAS"
FT                       /AA_IMGT="AA 1 to 26, AA 10 is missing"
FT                       /IMGT_note="no 1st-CYS: Phe instead of Cys"
FT   CDR1-IMGT           668689..668712
FT                       /translation="GFTFDDYG"
FT                       /AA_IMGT="AA 27 to 38, AA 31, 32, 33 and 34 are 
FT                       missing"
FT   FR2-IMGT            668713..668763
FT                       /translation="MSWVRQAPGKGLEWVSG"
FT                       /AA_IMGT="AA 39 to 55"
FT   CONSERVED-TRP       668719..668721
FT   CDR2-IMGT           668764..668787
FT                       /translation="INWNGGST"
FT                       /AA_IMGT="AA 56 to 65, AA 60 and 61 are missing"
FT   FR3-IMGT            668788..668901
FT                       /translation="GYADSVKGRFTISRDNAKNSLYLQMNSLRAEDTALYHC"
FT                       /AA_IMGT="AA 66 to 104, AA 73 is missing"
FT   3'V-REGION          668899..668909
FT                       /translation="CAR"
FT   2nd-CYS             668899..668901
FT   CDR3-IMGT           668902..668909
FT                       /translation="AR"
FT                       /AA_IMGT="AA 105 to 106"
FT   3'UTR               668910..675857
FT   V-RS                668910..668948
FT   V-HEPTAMER          668910..668916
FT   V-SPACER            668917..668939
FT   V-NONAMER           668940..668948
FT   V-GENE              675858..689000
FT                       /IMGT_allele="IGHV3-19*01"
FT                       /IMGT_gene="IGHV3-19"
FT                       /pseudo="no L-PART1, no L-PART2"
FT   5'UTR               675858..682851
FT   V-REGION            682852..683147
FT                       /translation="TVQLVESGGGLVEPGGSLRLSCAASGFTFSNSDMNWVRQAP
FT                       GKGLEWVSGVSWNGSRTHYADSVKGRFIISRDNSRNFLYQQMNSLRPEDMAVYYC
FT                       VR"
FT                       /CDR_length="[8.8.2]"
FT                       /FR_length="[25.17.38.G]"
FT                       /IMGT_allele="IGHV3-19*01"
FT                       /IMGT_gene="IGHV3-19"
FT                       /pseudo="no L-PART1, no L-PART2"
FT                       /putative_limit="5'"
FT   FR1-IMGT            682852..682926
FT                       /translation="TVQLVESGGGLVEPGGSLRLSCAAS"
FT                       /AA_IMGT="AA 1 to 26, AA 10 is missing"
FT                       /putative_limit="5'"
FT   1st-CYS             682915..682917
FT   CDR1-IMGT           682927..682950
FT                       /translation="GFTFSNSD"
FT                       /AA_IMGT="AA 27 to 38, AA 31, 32, 33 and 34 are 
FT                       missing"
FT   FR2-IMGT            682951..683001
FT                       /translation="MNWVRQAPGKGLEWVSG"
FT                       /AA_IMGT="AA 39 to 55"
FT   CONSERVED-TRP       682957..682959
FT   CDR2-IMGT           683002..683025
FT                       /translation="VSWNGSRT"
FT                       /AA_IMGT="AA 56 to 65, AA 60 and 61 are missing"
FT   FR3-IMGT            683026..683139
FT                       /translation="HYADSVKGRFIISRDNSRNFLYQQMNSLRPEDMAVYYC"
FT                       /AA_IMGT="AA 66 to 104, AA 73 is missing"
FT   3'V-REGION          683137..683147
FT                       /translation="CVR"
FT   2nd-CYS             683137..683139
FT   CDR3-IMGT           683140..683147
FT                       /translation="VR"
FT                       /AA_IMGT="AA 105 to 106"
FT   3'UTR               683148..689000
FT   V-RS                683148..683186
FT   V-HEPTAMER          683148..683154
FT   V-SPACER            683155..683177
FT   V-NONAMER           683178..683186
FT   V-GENE              689001..697500
FT                       /functional
FT                       /IMGT_allele="IGHV1-18*04"
FT                       /IMGT_gene="IGHV1-18"
FT   5'UTR               689001..694509
FT   L-V-GENE-UNIT       694510..694985
FT                       /functional
FT                       /IMGT_allele="IGHV1-18*04"
FT                       /IMGT_gene="IGHV1-18"
FT   L-INTRON-L          694510..694650
FT   L-PART1             694510..694555
FT                       /translation="MDWTWSILFLVAAAT"
FT   INIT-CODON          694510..694512
FT   DONOR-SPLICE        694555..694557
FT   V-INTRON            694556..694639
FT   ACCEPTOR-SPLICE     694637..694641
FT   V-EXON              694640..694946
FT                       /codon_start=3
FT                       /translation="AHSQVQLVQSGAEVKKPGASVKVSCKASGYTFTSYGISWVR
FT                       QAPGQGLEWMGWISAYNGNTNYAQKLQGRVTMTTDTSTSTAYMELRSLRSDDTAV
FT                       YYCAR"
FT   L-PART2             694640..694650
FT                       /codon_start=3
FT                       /translation="AHS"
FT   V-REGION            694651..694946
FT                       /translation="QVQLVQSGAEVKKPGASVKVSCKASGYTFTSYGISWVRQAP
FT                       GQGLEWMGWISAYNGNTNYAQKLQGRVTMTTDTSTSTAYMELRSLRSDDTAVYYC
FT                       AR"
FT                       /CDR_length="[8.8.2]"
FT                       /FR_length="[25.17.38.G]"
FT                       /functional
FT                       /IMGT_allele="IGHV1-18*04"
FT                       /IMGT_gene="IGHV1-18"
FT   FR1-IMGT            694651..694725
FT                       /translation="QVQLVQSGAEVKKPGASVKVSCKAS"
FT                       /AA_IMGT="AA 1 to 26, AA 10 is missing"
FT   1st-CYS             694714..694716
FT   CDR1-IMGT           694726..694749
FT                       /translation="GYTFTSYG"
FT                       /AA_IMGT="AA 27 to 38, AA 31, 32, 33 and 34 are 
FT                       missing"
FT   FR2-IMGT            694750..694800
FT                       /translation="ISWVRQAPGQGLEWMGW"
FT                       /AA_IMGT="AA 39 to 55"
FT   CONSERVED-TRP       694756..694758
FT   CDR2-IMGT           694801..694824
FT                       /translation="ISAYNGNT"
FT                       /AA_IMGT="AA 56 to 65, AA 60 and 61 are missing"
FT   FR3-IMGT            694825..694938
FT                       /translation="NYAQKLQGRVTMTTDTSTSTAYMELRSLRSDDTAVYYC"
FT                       /AA_IMGT="AA 66 to 104, AA 73 is missing"
FT   3'V-REGION          694936..694946
FT                       /translation="CAR"
FT   2nd-CYS             694936..694938
FT   CDR3-IMGT           694939..694946
FT                       /translation="AR"
FT                       /AA_IMGT="AA 105 to 106"
FT   3'UTR               694947..697500
FT   V-RS                694947..694985
FT   V-HEPTAMER          694947..694953
FT   V-SPACER            694954..694976
FT   V-NONAMER           694977..694985
FT   GENE                697501..703000
FT                       /IMGT_gene="SLC20A1P2"
FT                       /pseudo
FT   5'UTR               697501..699352
FT   GENE-UNIT           699353..700960
FT                       /IMGT_gene="SLC20A1P2"
FT                       /pseudo
FT   3'UTR               700961..703000
FT   V-GENE              703001..707135
FT                       /IMGT_allele="IGHV1-17*02"
FT                       /IMGT_gene="IGHV1-17"
FT                       /pseudo="frameshift in V-REGION: deletion of 1 nt in 
FT                       FR2-IMGT"
FT   5'UTR               703001..705066
FT   L-V-GENE-UNIT       705067..705542
FT                       /IMGT_allele="IGHV1-17*02"
FT                       /IMGT_gene="IGHV1-17"
FT                       /pseudo="frameshift in V-REGION: deletion of 1 nt in 
FT                       FR2-IMGT"
FT   L-INTRON-L          705067..705208
FT   L-PART1             705067..705112
FT                       /translation="MDCTWGILFLVAAAT"
FT   INIT-CODON          705067..705069
FT   DONOR-SPLICE        705112..705114
FT   V-INTRON            705113..705197
FT   ACCEPTOR-SPLICE     705195..705199
FT   V-EXON              705198..705503
FT                       /codon_start=3
FT                       /out_of_frame
FT   L-PART2             705198..705208
FT                       /codon_start=3
FT                       /translation="VHS"
FT   V-REGION            705209..705503
FT                       /CDR_length="[8.8.2]"
FT                       /FR_length="[25.X.38.G]"
FT                       /IMGT_allele="IGHV1-17*02"
FT                       /IMGT_gene="IGHV1-17"
FT                       /IMGT_note="restored translation in CDR2-IMGT, 
FT                       FR3-IMGT, 3'V-REGION and CDR3-IMGT"
FT                       /out_of_frame
FT                       /pseudo="frameshift in V-REGION: deletion of 1 nt in 
FT                       FR2-IMGT"
FT   FR1-IMGT            705209..705283
FT                       /translation="QVQLLQPGAEVKKPASSVKVSCQAS"
FT                       /AA_IMGT="AA 1 to 26, AA 10 is missing"
FT   1st-CYS             705272..705274
FT   CDR1-IMGT           705284..705307
FT                       /translation="RYTFTKYF"
FT                       /AA_IMGT="AA 27 to 38, AA 31, 32, 33 and 34 are 
FT                       missing"
FT   FR2-IMGT            705308..705357
FT                       /translation="TQWVRQGPGQGHSGWD"
FT                       /IMGT_note="frameshift in FR2-IMGT: deletion of 1 nt"
FT                       /out_of_frame
FT   CDR2-IMGT           705358..705381
FT                       /translation="INPYNDNT"
FT                       /AA_IMGT="AA 56 to 65, AA 60 and 61 are missing"
FT                       /out_of_frame
FT                       /restored_translation
FT   FR3-IMGT            705382..705495
FT                       /translation="HYAQKFRGRVTITSDRSVSTAYMELSSLRSEDMVVYSC"
FT                       /AA_IMGT="AA 66 to 104, AA 73 is missing"
FT                       /out_of_frame
FT                       /restored_translation
FT   3'V-REGION          705493..705503
FT                       /translation="CVR"
FT                       /out_of_frame
FT                       /restored_translation
FT   CDR3-IMGT           705496..705503
FT                       /translation="VR"
FT                       /AA_IMGT="AA 105 to 106"
FT                       /out_of_frame
FT                       /restored_translation
FT   3'UTR               705504..707135
FT   V-RS                705504..705542
FT   V-HEPTAMER          705504..705510
FT   V-SPACER            705511..705533
FT   V-NONAMER           705534..705542
FT   V-GENE              707136..711663
FT                       /IMGT_allele="IGHV(III)-16-1*02"
FT                       /IMGT_gene="IGHV(III)-16-1"
FT                       /pseudo="no L-PART1, truncated pseudogene not assigned 
FT                       to subgroups with functional genes"
FT   5'UTR               707136..708803
FT   V-REGION            <708804..709099
FT                       /partial
FT                       /IMGT_allele="IGHV(III)-16-1*02"
FT                       /IMGT_gene="IGHV(III)-16-1"
FT                       /IMGT_note="partial gene in 5'"
FT                       /pseudo="no L-PART1, truncated pseudogene not assigned 
FT                       to subgroups with functional genes"
FT                       /putative_limit="5'"
FT   3'UTR               709100..711663
FT   V-RS                709100..709139
FT   V-HEPTAMER          709100..709106
FT                       /IMGT_note="noncanonical V-HEPTAMER: cacagga instead 
FT                       of cacagtg"
FT   V-SPACER            709107..709130
FT   V-NONAMER           709131..709139
FT                       /IMGT_note="noncanonical V-NONAMER: acagaaaaa instead 
FT                       of acacaaacc"
FT   V-GENE              711664..715346
FT                       /IMGT_allele="IGHV3-16*02"
FT                       /IMGT_gene="IGHV3-16"
FT                       /ORF="noncanonical V-HEPTAMER: tcctgtg instead of 
FT                       cacagtg"
FT   5'UTR               711664..714188
FT   L-V-GENE-UNIT       714189..714681
FT                       /IMGT_allele="IGHV3-16*02"
FT                       /IMGT_gene="IGHV3-16"
FT                       /ORF="noncanonical V-HEPTAMER: tcctgtg instead of 
FT                       cacagtg"
FT   L-INTRON-L          714189..714346
FT   L-PART1             714189..714234
FT                       /translation="MEFGLSWVFLAGILK"
FT   INIT-CODON          714189..714191
FT   DONOR-SPLICE        714234..714236
FT   V-INTRON            714235..714335
FT   ACCEPTOR-SPLICE     714333..714337
FT   V-EXON              714336..714642
FT                       /codon_start=3
FT                       /translation="VQCEVQLVESGGGLVQPGGSLRLSCAASGFTFSNSDMNWAR
FT                       KAPGKGLEWVSGVSWNGSRTHYVDSVKRRFIISRDNSRNSLYLQKNRRRAEDMAV
FT                       YYCVR"
FT   L-PART2             714336..714346
FT                       /codon_start=3
FT                       /translation="VQC"
FT   V-REGION            714347..714642
FT                       /translation="EVQLVESGGGLVQPGGSLRLSCAASGFTFSNSDMNWARKAP
FT                       GKGLEWVSGVSWNGSRTHYVDSVKRRFIISRDNSRNSLYLQKNRRRAEDMAVYYC
FT                       VR"
FT                       /CDR_length="[8.8.2]"
FT                       /FR_length="[25.17.38.G]"
FT                       /IMGT_allele="IGHV3-16*02"
FT                       /IMGT_gene="IGHV3-16"
FT                       /ORF="noncanonical V-HEPTAMER: tcctgtg instead of 
FT                       cacagtg"
FT   FR1-IMGT            714347..714421
FT                       /translation="EVQLVESGGGLVQPGGSLRLSCAAS"
FT                       /AA_IMGT="AA 1 to 26, AA 10 is missing"
FT   1st-CYS             714410..714412
FT   CDR1-IMGT           714422..714445
FT                       /translation="GFTFSNSD"
FT                       /AA_IMGT="AA 27 to 38, AA 31, 32, 33 and 34 are 
FT                       missing"
FT   FR2-IMGT            714446..714496
FT                       /translation="MNWARKAPGKGLEWVSG"
FT                       /AA_IMGT="AA 39 to 55"
FT   CONSERVED-TRP       714452..714454
FT   CDR2-IMGT           714497..714520
FT                       /translation="VSWNGSRT"
FT                       /AA_IMGT="AA 56 to 65, AA 60 and 61 are missing"
FT   FR3-IMGT            714521..714634
FT                       /translation="HYVDSVKRRFIISRDNSRNSLYLQKNRRRAEDMAVYYC"
FT                       /AA_IMGT="AA 66 to 104, AA 73 is missing"
FT   3'V-REGION          714632..714642
FT                       /translation="CVR"
FT   2nd-CYS             714632..714634
FT   CDR3-IMGT           714635..714642
FT                       /translation="VR"
FT                       /AA_IMGT="AA 105 to 106"
FT   3'UTR               714643..715346
FT   V-RS                714643..714681
FT   V-HEPTAMER          714643..714649
FT                       /IMGT_note="noncanonical V-HEPTAMER: tcctgtg instead 
FT                       of cacagtg"
FT   V-SPACER            714650..714672
FT   V-NONAMER           714673..714681
FT   V-GENE              715347..721058
FT                       /IMGT_allele="IGHV(II)-15-1*01"
FT                       /IMGT_gene="IGHV(II)-15-1"
FT                       /pseudo="truncated pseudogene not assigned to 
FT                       subgroups with functional genes"
FT   5'UTR               715347..716040
FT   V-REGION            <716041..716311
FT                       /partial
FT                       /CDR_length="[10.X.2]"
FT                       /FR_length="[16.17.38.G]"
FT                       /IMGT_allele="IGHV(II)-15-1*01"
FT                       /IMGT_gene="IGHV(II)-15-1"
FT                       /IMGT_note="partial gene in 5', restored translation 
FT                       in FR3-IMGT, 3'V-REGION and CDR3-IMGT"
FT                       /out_of_frame
FT                       /pseudo="truncated pseudogene not assigned to 
FT                       subgroups with functional genes"
FT                       /putative_limit="5'"
FT   FR1-IMGT            <716041..716088
FT                       /partial
FT                       /translation="VLVKPSETLSFTCAVS"
FT                       /putative_limit="5'"
FT   1st-CYS             716077..716079
FT   CDR1-IMGT           716089..716118
FT                       /translation="GFPIITSTSS"
FT                       /AA_IMGT="AA 27 to 38, AA 32 and 33 are missing"
FT   FR2-IMGT            716119..716169
FT                       /translation="WSCICQPPGKKLKWVRC"
FT                       /AA_IMGT="AA 39 to 55"
FT                       /IMGT_note="no CONSERVED-TRP: Cys instead of Trp"
FT   CDR2-IMGT           716170..716189
FT                       /translation="VGHEET"
FT                       /IMGT_note="frameshift in CDR2-IMGT: deletion of 1 nt"
FT                       /out_of_frame
FT   FR3-IMGT            716190..716303
FT                       /AA_IMGT="AA 66 to 104, AA 73 is missing"
FT                       /out_of_frame
FT                       /restored_translation
FT   3'V-REGION          716301..716311
FT                       /translation="YTK"
FT                       /out_of_frame
FT                       /restored_translation
FT   CDR3-IMGT           716304..716311
FT                       /translation="TK"
FT                       /AA_IMGT="AA 105 to 106"
FT                       /out_of_frame
FT                       /restored_translation
FT   3'UTR               716312..721058
FT   V-RS                716312..716354
FT   V-HEPTAMER          716312..716318
FT                       /IMGT_note="noncanonical V-HEPTAMER: cacagaa instead 
FT                       of cacagtg"
FT   V-SPACER            716319..716345
FT   V-NONAMER           716346..716354
FT                       /IMGT_note="noncanonical V-NONAMER: aacctccct instead 
FT                       of acacaaacc"
FT   V-GENE              721059..729965
FT                       /functional
FT                       /IMGT_allele="IGHV3-15*01"
FT                       /IMGT_gene="IGHV3-15"
FT   5'UTR               721059..725761
FT   L-V-GENE-UNIT       725762..726262
FT                       /functional
FT                       /IMGT_allele="IGHV3-15*01"
FT                       /IMGT_gene="IGHV3-15"
FT   L-INTRON-L          725762..725921
FT   L-PART1             725762..725807
FT                       /translation="MEFGLSWIFLAAILK"
FT   INIT-CODON          725762..725764
FT   DONOR-SPLICE        725807..725809
FT   V-INTRON            725808..725910
FT   ACCEPTOR-SPLICE     725908..725912
FT   V-EXON              725911..726223
FT                       /codon_start=3
FT                       /translation="VQCEVQLVESGGGLVKPGGSLRLSCAASGFTFSNAWMSWVR
FT                       QAPGKGLEWVGRIKSKTDGGTTDYAAPVKGRFTISRDDSKNTLYLQMNSLKTEDT
FT                       AVYYCTT"
FT   L-PART2             725911..725921
FT                       /codon_start=3
FT                       /translation="VQC"
FT   V-REGION            725922..726223
FT                       /translation="EVQLVESGGGLVKPGGSLRLSCAASGFTFSNAWMSWVRQAP
FT                       GKGLEWVGRIKSKTDGGTTDYAAPVKGRFTISRDDSKNTLYLQMNSLKTEDTAVY
FT                       YCTT"
FT                       /CDR_length="[8.10.2]"
FT                       /FR_length="[25.17.38.G]"
FT                       /functional
FT                       /IMGT_allele="IGHV3-15*01"
FT                       /IMGT_gene="IGHV3-15"
FT   FR1-IMGT            725922..725996
FT                       /translation="EVQLVESGGGLVKPGGSLRLSCAAS"
FT                       /AA_IMGT="AA 1 to 26, AA 10 is missing"
FT   1st-CYS             725985..725987
FT   CDR1-IMGT           725997..726020
FT                       /translation="GFTFSNAW"
FT                       /AA_IMGT="AA 27 to 38, AA 31, 32, 33 and 34 are 
FT                       missing"
FT   FR2-IMGT            726021..726071
FT                       /translation="MSWVRQAPGKGLEWVGR"
FT                       /AA_IMGT="AA 39 to 55"
FT   CONSERVED-TRP       726027..726029
FT   CDR2-IMGT           726072..726101
FT                       /translation="IKSKTDGGTT"
FT                       /AA_IMGT="AA 56 to 65"
FT   FR3-IMGT            726102..726215
FT                       /translation="DYAAPVKGRFTISRDDSKNTLYLQMNSLKTEDTAVYYC"
FT                       /AA_IMGT="AA 66 to 104, AA 73 is missing"
FT   3'V-REGION          726213..726223
FT                       /translation="CTT"
FT   2nd-CYS             726213..726215
FT   CDR3-IMGT           726216..726223
FT                       /translation="TT"
FT                       /AA_IMGT="AA 105 to 106"
FT   3'UTR               726224..729965
FT   V-RS                726224..726262
FT   V-HEPTAMER          726224..726230
FT   V-SPACER            726231..726253
FT   V-NONAMER           726254..726262
FT   V-GENE              729966..735618
FT                       /IMGT_allele="IGHV1-14*02"
FT                       /IMGT_gene="IGHV1-14"
FT                       /pseudo="no L-PART1, no L-PART2, frameshifts in 
FT                       V-REGION: several insertions and deletions"
FT   5'UTR               729966..733667
FT   V-REGION            733668..733963
FT                       /CDR_length="[8.8.2]"
FT                       /FR_length="[X.17.38.G]"
FT                       /IMGT_allele="IGHV1-14*02"
FT                       /IMGT_gene="IGHV1-14"
FT                       /out_of_frame
FT                       /pseudo="no L-PART1, no L-PART2, frameshifts in 
FT                       V-REGION: several insertions and deletions"
FT                       /putative_limit="5'"
FT   FR1-IMGT            733668..733742
FT                       /translation="MTHPASDDSPVASVHGLSEGVLQSS"
FT                       /AA_IMGT="AA 1 to 26, AA 10 is missing"
FT                       /IMGT_note="frameshifts in FR1-IMGT: deletion of 2 nt 
FT                       and deletion of 1 nt"
FT                       /putative_limit="5'"
FT   1st-CYS             733732..733734
FT   CDR1-IMGT           733743..733766
FT                       /translation="GYTFASYD"
FT                       /AA_IMGT="AA 27 to 38, AA 31, 32, 33 and 34 are 
FT                       missing"
FT   FR2-IMGT            733767..733817
FT                       /AA_IMGT="AA 39 to 55"
FT                       /IMGT_note="several STOP-CODON in FR2-IMGT: pos 43 and 
FT                       48, no CONSERVED-TRP: Cys instead of Trp"
FT   CDR2-IMGT           733818..733841
FT                       /translation="SYSGNGNT"
FT                       /AA_IMGT="AA 56 to 65, AA 60 and 61 are missing"
FT   FR3-IMGT            733842..733955
FT                       /translation="GYAQKFQGRVTMTRDTSTSTAYMELSSQRSEDIDVYYC"
FT                       /AA_IMGT="AA 66 to 104, AA 73 is missing"
FT   3'V-REGION          733953..733963
FT                       /translation="CAR"
FT   2nd-CYS             733953..733955
FT   CDR3-IMGT           733956..733963
FT                       /translation="AR"
FT                       /AA_IMGT="AA 105 to 106"
FT   3'UTR               733964..735618
FT   V-RS                733964..734002
FT   V-HEPTAMER          733964..733970
FT   V-SPACER            733971..733993
FT   V-NONAMER           733994..734002
FT                       /IMGT_note="noncanonical V-NONAMER: tcagaaatc instead 
FT                       of acacaaacc"
FT   V-GENE              735619..743805
FT                       /IMGT_allele="IGHV(III)-13-1*02"
FT                       /IMGT_gene="IGHV(III)-13-1"
FT                       /pseudo="no L-PART1, frameshifts in V-REGION: several 
FT                       insertions and deletions"
FT   5'UTR               735619..737260
FT   ACCEPTOR-SPLICE     737258..737262
FT   V-EXON              737261..737559
FT                       /codon_start=3
FT                       /out_of_frame
FT   L-PART2             737261..737271
FT                       /codon_start=3
FT                       /translation="VPC"
FT   V-REGION            737272..737559
FT                       /CDR_length="[8.9.2]"
FT                       /FR_length="[X.X.38.G]"
FT                       /IMGT_allele="IGHV(III)-13-1*02"
FT                       /IMGT_gene="IGHV(III)-13-1"
FT                       /IMGT_note="restored translation in CDR1-IMGT, 
FT                       FR2-IMGT"
FT                       /out_of_frame
FT                       /pseudo="no L-PART1, frameshifts in V-REGION: several 
FT                       insertions and deletions"
FT   FR1-IMGT            737272..737341
FT                       /IMGT_note="frameshift in FR1-IMGT: deletion of 5 nt"
FT                       /out_of_frame
FT   CDR1-IMGT           737342..737365
FT                       /translation="GFTFTENS"
FT                       /AA_IMGT="AA 27 to 38, AA 31, 32, 33 and 34 are 
FT                       missing"
FT                       /out_of_frame
FT                       /restored_translation
FT   FR2-IMGT            737366..737412
FT                       /IMGT_note="frameshift in FR2-IMGT: deletion of 1 nt"
FT                       /out_of_frame
FT                       /restored_translation
FT   CDR2-IMGT           737413..737439
FT                       /translation="NNNSNRSSQ"
FT                       /AA_IMGT="AA 56 to 65, AA 61 is missing"
FT                       /in_frame
FT   FR3-IMGT            737440..737553
FT                       /AA_IMGT="AA 66 to 104, AA 73 is missing"
FT                       /IMGT_note="several STOP-CODON in FR3-IMGT: pos 70 and 
FT                       75, no 2nd-CYS: Phe instead of Cys"
FT                       /in_frame
FT   3'V-REGION          737551..737559
FT                       /translation="FEG"
FT                       /in_frame
FT   CDR3-IMGT           737554..737559
FT                       /translation="EG"
FT                       /AA_IMGT="AA 105 to 106"
FT                       /in_frame
FT   3'UTR               737560..743805
FT   V-RS                737560..737598
FT   V-HEPTAMER          737560..737566
FT   V-SPACER            737567..737589
FT   V-NONAMER           737590..737598
FT   V-GENE              743806..751450
FT                       /functional
FT                       /IMGT_allele="IGHV3-13*05"
FT                       /IMGT_gene="IGHV3-13"
FT   5'UTR               743806..749852
FT   L-V-GENE-UNIT       749853..750344
FT                       /functional
FT                       /IMGT_allele="IGHV3-13*05"
FT                       /IMGT_gene="IGHV3-13"
FT   L-INTRON-L          749853..750012
FT   L-PART1             749853..749898
FT                       /translation="MELGLSWVFLVAILE"
FT   INIT-CODON          749853..749855
FT   DONOR-SPLICE        749898..749900
FT   V-INTRON            749899..750001
FT   ACCEPTOR-SPLICE     749999..750003
FT   V-EXON              750002..750305
FT                       /codon_start=3
FT                       /translation="VQCEVQLVESGGGLVQPGGSLRLSCAASGFTFSSYDMHWVR
FT                       QATGKGLEWVSAIGTAGDPYYPGSVKGRFTISRENAKNSLYLQMNSLRAGDTAVY
FT                       YCAR"
FT   L-PART2             750002..750012
FT                       /codon_start=3
FT                       /translation="VQC"
FT   V-REGION            750013..750305
FT                       /translation="EVQLVESGGGLVQPGGSLRLSCAASGFTFSSYDMHWVRQAT
FT                       GKGLEWVSAIGTAGDPYYPGSVKGRFTISRENAKNSLYLQMNSLRAGDTAVYYCA
FT                       R"
FT                       /CDR_length="[8.7.2]"
FT                       /FR_length="[25.17.38.G]"
FT                       /functional
FT                       /IMGT_allele="IGHV3-13*05"
FT                       /IMGT_gene="IGHV3-13"
FT   FR1-IMGT            750013..750087
FT                       /translation="EVQLVESGGGLVQPGGSLRLSCAAS"
FT                       /AA_IMGT="AA 1 to 26, AA 10 is missing"
FT   1st-CYS             750076..750078
FT   CDR1-IMGT           750088..750111
FT                       /translation="GFTFSSYD"
FT                       /AA_IMGT="AA 27 to 38, AA 31, 32, 33 and 34 are 
FT                       missing"
FT   FR2-IMGT            750112..750162
FT                       /translation="MHWVRQATGKGLEWVSA"
FT                       /AA_IMGT="AA 39 to 55"
FT   CONSERVED-TRP       750118..750120
FT   CDR2-IMGT           750163..750183
FT                       /translation="IGTAGDP"
FT                       /AA_IMGT="AA 56 to 65, AA 60, 61 and 62 are missing"
FT   FR3-IMGT            750184..750297
FT                       /translation="YYPGSVKGRFTISRENAKNSLYLQMNSLRAGDTAVYYC"
FT                       /AA_IMGT="AA 66 to 104, AA 73 is missing"
FT   3'V-REGION          750295..750305
FT                       /translation="CAR"
FT   2nd-CYS             750295..750297
FT   CDR3-IMGT           750298..750305
FT                       /translation="AR"
FT                       /AA_IMGT="AA 105 to 106"
FT   3'UTR               750306..751450
FT   V-RS                750306..750344
FT   V-HEPTAMER          750306..750312
FT   V-SPACER            750313..750335
FT   V-NONAMER           750336..750344
FT   V-GENE              751451..754994
FT                       /IMGT_allele="IGHV(II)-12-1*01"
FT                       /IMGT_gene="IGHV(II)-12-1"
FT                       /pseudo="frameshifts in V-REGION: several insertions 
FT                       and deletions, no V-RS"
FT   5'UTR               751451..752408
FT   L-INTRON-L          752409..752551
FT   L-PART1             752409..752454
FT                       /translation="MALLCLLLCLVMAPP"
FT   INIT-CODON          752409..752411
FT   DONOR-SPLICE        752454..752456
FT                       /IMGT_note="noncanonical DONOR-SPLICE: ngg instead of 
FT                       ngt"
FT   V-INTRON            752455..752540
FT   ACCEPTOR-SPLICE     752538..752542
FT                       /IMGT_note="noncanonical ACCEPTOR-SPLICE: nacnn 
FT                       instead of nagnn"
FT   V-EXON              752541..752735
FT                       /codon_start=3
FT                       /putative_limit="3'"
FT   L-PART2             752541..752551
FT                       /codon_start=3
FT                       /translation="ALS"
FT   V-REGION            752552..752735
FT                       /IMGT_allele="IGHV(II)-12-1*01"
FT                       /IMGT_gene="IGHV(II)-12-1"
FT                       /pseudo="frameshifts in V-REGION: several insertions 
FT                       and deletions, no V-RS"
FT                       /putative_limit="3'"
FT   REPEAT_UNIT         752736..752848
FT                       /rpt_family="LINE1"
FT   3'UTR               752849..754994
FT   V-GENE              754995..758407
FT                       /IMGT_allele="IGHV1-12*02"
FT                       /IMGT_gene="IGHV1-12"
FT                       /pseudo="In frame STOP_CODON and Truncated V-GENE with 
FT                       completely divergent 3' region."
FT   5'UTR               754995..757135
FT   L-INTRON-L          757136..757277
FT   L-PART1             757136..757181
FT                       /translation="MGWTCKILFLVAAAT"
FT   INIT-CODON          757136..757138
FT   DONOR-SPLICE        757181..757183
FT   V-INTRON            757182..757266
FT   ACCEPTOR-SPLICE     757264..757268
FT   V-EXON              757267..757427
FT                       /codon_start=3
FT                       /putative_limit="3'"
FT   L-PART2             757267..757277
FT                       /codon_start=3
FT                       /translation="AHS"
FT   V-REGION            757278..757427
FT                       /CDR_length="[8.8.2]"
FT                       /FR_length="[25.17.38.G]"
FT                       /IMGT_allele="IGHV1-12*02"
FT                       /IMGT_gene="IGHV1-12"
FT                       /pseudo="In frame STOP_CODON and Truncated V-GENE with 
FT                       completely divergent 3' region"
FT                       /putative_limit="3'"
FT   FR1-IMGT            757278..757352
FT                       /translation="QVQLVQSGAEVKKPGASVKVSCKAS"
FT                       /AA_IMGT="AA 1 to 26, AA 10 is missing"
FT   1st-CYS             757341..757343
FT   CDR1-IMGT           757353..757376
FT                       /translation="GYTFTYCY"
FT                       /AA_IMGT="AA 27 to 38, AA 31, 32, 33 and 34 are 
FT                       missing"
FT   FR2-IMGT            757377..757427
FT                       /AA_IMGT="AA 39 to 55"
FT                       /IMGT_note="STOP-CODON in FR2-IMGT: position 43"
FT   CONSERVED-TRP       757383..757385
FT   3'UTR               757574..758407
FT   V-GENE              758408..761225
FT                       /IMGT_allele="IGHV(III)-11-1*01"
FT                       /IMGT_gene="IGHV(III)-11-1"
FT                       /pseudo="truncated pseudogene not assigned to 
FT                       subgroups with functional genes"
FT   5'UTR               758408..759369
FT   ACCEPTOR-SPLICE     759367..759371
FT   V-EXON              759370..759584
FT                       /codon_start=3
FT                       /out_of_frame
FT                       /putative_limit="3'"
FT   L-PART2             759370..759380
FT                       /codon_start=3
FT                       /translation="VLC"
FT   V-REGION            759381..>759584
FT                       /partial
FT                       /CDR_length="[X.7.X]"
FT                       /FR_length="[X.17.11.G]"
FT                       /IMGT_allele="IGHV(III)-11-1*01"
FT                       /IMGT_gene="IGHV(III)-11-1"
FT                       /IMGT_note="partial gene in 3', restored translation 
FT                       in CDR1-IMGT"
FT                       /out_of_frame
FT                       /pseudo="truncated pseudogene not assigned to 
FT                       subgroups with functional genes"
FT                       /putative_limit="3'"
FT   FR1-IMGT            759381..759456
FT                       /IMGT_note="frameshifts in FR1-IMGT: deletion of 1 nt 
FT                       and insertion of 2 nt"
FT                       /out_of_frame
FT   CDR1-IMGT           759457..759479
FT                       /translation="GSPSLIA"
FT                       /IMGT_note="frameshift in CDR1-IMGT: deletion of 1 nt"
FT                       /out_of_frame
FT                       /restored_translation
FT   FR2-IMGT            759480..759530
FT                       /translation="ISLVQQAPGPELAWAAT"
FT                       /AA_IMGT="AA 39 to 55"
FT                       /IMGT_note="no CONSERVED-TRP: Leu instead of Trp"
FT                       /in_frame
FT   CDR2-IMGT           759531..759551
FT                       /translation="GRNSRGS"
FT                       /AA_IMGT="AA 56 to 65, AA 60, 61 and 62 are missing"
FT                       /in_frame
FT   FR3-IMGT            759552..759584
FT                       /translation="SYMHPYVHGLT"
FT                       /in_frame
FT   3'UTR               759585..761225
FT   V-GENE              761226..767343
FT                       /functional
FT                       /IMGT_allele="IGHV3-11*06"
FT                       /IMGT_gene="IGHV3-11"
FT   5'UTR               761226..762760
FT   L-V-GENE-UNIT       762761..763249
FT                       /functional
FT                       /IMGT_allele="IGHV3-11*06"
FT                       /IMGT_gene="IGHV3-11"
FT   L-INTRON-L          762761..762914
FT   L-PART1             762761..762806
FT                       /translation="MEFGLSWVFLVAIIK"
FT   INIT-CODON          762761..762763
FT   DONOR-SPLICE        762806..762808
FT   V-INTRON            762807..762903
FT   ACCEPTOR-SPLICE     762901..762905
FT   V-EXON              762904..763210
FT                       /codon_start=3
FT                       /translation="VQCQVQLVESGGGLVKPGGSLRLSCAASGFTFSDYYMSWIR
FT                       QAPGKGLEWVSYISSSSSYTNYADSVKGRFTISRDNAKNSLYLQMNSLRAEDTAV
FT                       YYCAR"
FT   L-PART2             762904..762914
FT                       /codon_start=3
FT                       /translation="VQC"
FT   V-REGION            762915..763210
FT                       /translation="QVQLVESGGGLVKPGGSLRLSCAASGFTFSDYYMSWIRQAP
FT                       GKGLEWVSYISSSSSYTNYADSVKGRFTISRDNAKNSLYLQMNSLRAEDTAVYYC
FT                       AR"
FT                       /CDR_length="[8.8.2]"
FT                       /FR_length="[25.17.38.G]"
FT                       /functional
FT                       /IMGT_allele="IGHV3-11*06"
FT                       /IMGT_gene="IGHV3-11"
FT   FR1-IMGT            762915..762989
FT                       /translation="QVQLVESGGGLVKPGGSLRLSCAAS"
FT                       /AA_IMGT="AA 1 to 26, AA 10 is missing"
FT   1st-CYS             762978..762980
FT   CDR1-IMGT           762990..763013
FT                       /translation="GFTFSDYY"
FT                       /AA_IMGT="AA 27 to 38, AA 31, 32, 33 and 34 are 
FT                       missing"
FT   FR2-IMGT            763014..763064
FT                       /translation="MSWIRQAPGKGLEWVSY"
FT                       /AA_IMGT="AA 39 to 55"
FT   CONSERVED-TRP       763020..763022
FT   CDR2-IMGT           763065..763088
FT                       /translation="ISSSSSYT"
FT                       /AA_IMGT="AA 56 to 65, AA 60 and 61 are missing"
FT   FR3-IMGT            763089..763202
FT                       /translation="NYADSVKGRFTISRDNAKNSLYLQMNSLRAEDTAVYYC"
FT                       /AA_IMGT="AA 66 to 104, AA 73 is missing"
FT   3'V-REGION          763200..763210
FT                       /translation="CAR"
FT   2nd-CYS             763200..763202
FT   CDR3-IMGT           763203..763210
FT                       /translation="AR"
FT                       /AA_IMGT="AA 105 to 106"
FT   3'UTR               763211..767343
FT   V-RS                763211..763249
FT   V-HEPTAMER          763211..763217
FT   V-SPACER            763218..763240
FT   V-NONAMER           763241..763249
FT   V-GENE              767344..778078
FT                       /functional
FT                       /IMGT_allele="IGHV5-10-1*03"
FT                       /IMGT_gene="IGHV5-10-1"
FT   5'UTR               767344..771437
FT   L-V-GENE-UNIT       771438..771911
FT                       /functional
FT                       /IMGT_allele="IGHV5-10-1*03"
FT                       /IMGT_gene="IGHV5-10-1"
FT   L-INTRON-L          771438..771577
FT   L-PART1             771438..771483
FT                       /translation="MGSTAILALLLAVLQ"
FT   INIT-CODON          771438..771440
FT   DONOR-SPLICE        771483..771485
FT   V-INTRON            771484..771566
FT   ACCEPTOR-SPLICE     771564..771568
FT   V-EXON              771567..771873
FT                       /codon_start=3
FT                       /translation="VCAEVQLVQSGAEVKKPGESLRISCKGSGYSFTSYWISWVR
FT                       QMPGKGLEWMGRIDPSDSYTNYSPSFQGHVTISADKSISTAYLQWSSLKASDTAM
FT                       YYCAR"
FT   L-PART2             771567..771577
FT                       /codon_start=3
FT                       /translation="VCA"
FT   V-REGION            771578..771873
FT                       /translation="EVQLVQSGAEVKKPGESLRISCKGSGYSFTSYWISWVRQMP
FT                       GKGLEWMGRIDPSDSYTNYSPSFQGHVTISADKSISTAYLQWSSLKASDTAMYYC
FT                       AR"
FT                       /CDR_length="[8.8.2]"
FT                       /FR_length="[25.17.38.G]"
FT                       /functional
FT                       /IMGT_allele="IGHV5-10-1*03"
FT                       /IMGT_gene="IGHV5-10-1"
FT   FR1-IMGT            771578..771652
FT                       /translation="EVQLVQSGAEVKKPGESLRISCKGS"
FT                       /AA_IMGT="AA 1 to 26, AA 10 is missing"
FT   1st-CYS             771641..771643
FT   CDR1-IMGT           771653..771676
FT                       /translation="GYSFTSYW"
FT                       /AA_IMGT="AA 27 to 38, AA 31, 32, 33 and 34 are 
FT                       missing"
FT   FR2-IMGT            771677..771727
FT                       /translation="ISWVRQMPGKGLEWMGR"
FT                       /AA_IMGT="AA 39 to 55"
FT   CONSERVED-TRP       771683..771685
FT   CDR2-IMGT           771728..771751
FT                       /translation="IDPSDSYT"
FT                       /AA_IMGT="AA 56 to 65, AA 60 and 61 are missing"
FT   FR3-IMGT            771752..771865
FT                       /translation="NYSPSFQGHVTISADKSISTAYLQWSSLKASDTAMYYC"
FT                       /AA_IMGT="AA 66 to 104, AA 73 is missing"
FT   3'V-REGION          771863..771873
FT                       /translation="CAR"
FT   2nd-CYS             771863..771865
FT   CDR3-IMGT           771866..771873
FT                       /translation="AR"
FT                       /AA_IMGT="AA 105 to 106"
FT   3'UTR               771874..778078
FT   V-RS                771874..771911
FT   V-HEPTAMER          771874..771880
FT   V-SPACER            771881..771902
FT   V-NONAMER           771903..771911
FT   V-GENE              778079..787933
FT                       /IMGT_allele="IGHV(III)-10-2*01"
FT                       /IMGT_gene="IGHV(III)-10-2"
FT                       /pseudo="truncated pseudogene not assigned to 
FT                       subgroups with functional genes"
FT   5'UTR               778079..784301
FT   V-REGION            <784302..784551
FT                       /partial
FT                       /CDR_length="[8.9.2]"
FT                       /FR_length="[9.X.38.G]"
FT                       /IMGT_allele="IGHV(III)-10-2*01"
FT                       /IMGT_gene="IGHV(III)-10-2"
FT                       /IMGT_note="partial gene in 5', restored translation 
FT                       in CDR2-IMGT, FR3-IMGT, 3'V-REGION and CDR3-IMGT"
FT                       /out_of_frame
FT                       /pseudo="truncated pseudogene not assigned to 
FT                       subgroups with functional genes"
FT                       /putative_limit="5'"
FT   FR1-IMGT            <784302..784328
FT                       /partial
FT                       /translation="SQILFYKAS"
FT                       /IMGT_note="no 1st-CYS: Tyr instead of Cys"
FT                       /putative_limit="5'"
FT   CDR1-IMGT           784329..784352
FT                       /translation="EFTFIDSI"
FT                       /AA_IMGT="AA 27 to 38, AA 31, 32, 33 and 34 are 
FT                       missing"
FT   FR2-IMGT            784353..784404
FT                       /IMGT_note="frameshift in FR2-IMGT: insertion of 1 nt"
FT                       /out_of_frame
FT   CDR2-IMGT           784405..784431
FT                       /AA_IMGT="AA 56 to 65, AA 61 is missing"
FT                       /out_of_frame
FT                       /restored_translation
FT   FR3-IMGT            784432..784545
FT                       /translation="WYPPSIQRRIIILRDTLVSTVSLKMTNLRAENKVTCYC"
FT                       /AA_IMGT="AA 66 to 104, AA 73 is missing"
FT                       /out_of_frame
FT                       /restored_translation
FT   3'V-REGION          784543..784551
FT                       /translation="CER"
FT                       /out_of_frame
FT                       /restored_translation
FT   CDR3-IMGT           784546..784551
FT                       /translation="ER"
FT                       /AA_IMGT="AA 105 to 106"
FT                       /out_of_frame
FT                       /restored_translation
FT   3'UTR               784552..787933
FT   V-RS                784552..784593
FT   V-HEPTAMER          784552..784558
FT   V-SPACER            784559..784584
FT   V-NONAMER           784585..784593
FT   V-GENE              787934..794139
FT                       /functional
FT                       /IMGT_allele="IGHV3-64D*06"
FT                       /IMGT_gene="IGHV3-64D"
FT   5'UTR               787934..791271
FT   L-V-GENE-UNIT       791272..791762
FT                       /functional
FT                       /IMGT_allele="IGHV3-64D*06"
FT                       /IMGT_gene="IGHV3-64D"
FT   L-INTRON-L          791272..791427
FT   L-PART1             791272..791317
FT                       /translation="MEFWLSWVLLVAILK"
FT   INIT-CODON          791272..791274
FT   DONOR-SPLICE        791317..791319
FT   V-INTRON            791318..791416
FT   ACCEPTOR-SPLICE     791414..791418
FT   V-EXON              791417..791723
FT                       /codon_start=3
FT                       /translation="VQCEVQLVESGGGLVQPGGSLRLSCSASGFTFSSYAMHWVR
FT                       QAPGKGLEYVSAISSNGGSTYYADSVKGRFTISRDNSKNTLYLQMSSLRAEDTAV
FT                       YYCVK"
FT   L-PART2             791417..791427
FT                       /codon_start=3
FT                       /translation="VQC"
FT   V-REGION            791428..791723
FT                       /translation="EVQLVESGGGLVQPGGSLRLSCSASGFTFSSYAMHWVRQAP
FT                       GKGLEYVSAISSNGGSTYYADSVKGRFTISRDNSKNTLYLQMSSLRAEDTAVYYC
FT                       VK"
FT                       /CDR_length="[8.8.2]"
FT                       /FR_length="[25.17.38.G]"
FT                       /functional
FT                       /IMGT_allele="IGHV3-64D*06"
FT                       /IMGT_gene="IGHV3-64D"
FT   FR1-IMGT            791428..791502
FT                       /translation="EVQLVESGGGLVQPGGSLRLSCSAS"
FT                       /AA_IMGT="AA 1 to 26, AA 10 is missing"
FT   1st-CYS             791491..791493
FT   CDR1-IMGT           791503..791526
FT                       /translation="GFTFSSYA"
FT                       /AA_IMGT="AA 27 to 38, AA 31, 32, 33 and 34 are 
FT                       missing"
FT   FR2-IMGT            791527..791577
FT                       /translation="MHWVRQAPGKGLEYVSA"
FT                       /AA_IMGT="AA 39 to 55"
FT   CONSERVED-TRP       791533..791535
FT   CDR2-IMGT           791578..791601
FT                       /translation="ISSNGGST"
FT                       /AA_IMGT="AA 56 to 65, AA 60 and 61 are missing"
FT   FR3-IMGT            791602..791715
FT                       /translation="YYADSVKGRFTISRDNSKNTLYLQMSSLRAEDTAVYYC"
FT                       /AA_IMGT="AA 66 to 104, AA 73 is missing"
FT   3'V-REGION          791713..791723
FT                       /translation="CVK"
FT   2nd-CYS             791713..791715
FT   CDR3-IMGT           791716..791723
FT                       /translation="VK"
FT                       /AA_IMGT="AA 105 to 106"
FT   3'UTR               791724..794139
FT   V-RS                791724..791762
FT   V-HEPTAMER          791724..791730
FT   V-SPACER            791731..791753
FT   V-NONAMER           791754..791762
FT   V-GENE              794140..797659
FT                       /IMGT_allele="IGHV3-7-2*01"
FT                       /IMGT_gene="IGHV3-7-2"
FT                       /pseudo="STOP-CODON in L-PART1, several STOP-CODON in 
FT                       V-REGION, frameshift in V-REGION: insertion of 1 nt in 
FT                       FR3-IMGT"
FT   5'UTR               794140..796515
FT   L-V-GENE-UNIT       796516..797003
FT                       /IMGT_allele="IGHV3-7-2*01"
FT                       /IMGT_gene="IGHV3-7-2"
FT                       /pseudo="STOP-CODON in L-PART1, several STOP-CODON in 
FT                       V-REGION, frameshift in V-REGION: insertion of 1 nt in 
FT                       FR3-IMGT"
FT   L-INTRON-L          796516..796665
FT   L-PART1             796516..796561
FT                       /IMGT_note="STOP-CODON in L-PART1"
FT   INIT-CODON          796516..796518
FT   DONOR-SPLICE        796561..796563
FT   V-INTRON            796562..796654
FT   ACCEPTOR-SPLICE     796652..796656
FT   V-EXON              796655..796964
FT                       /codon_start=3
FT                       /out_of_frame
FT   L-PART2             796655..796665
FT                       /codon_start=3
FT                       /translation="VQC"
FT   V-REGION            796666..796964
FT                       /CDR_length="[8.8.3]"
FT                       /FR_length="[25.17.X.G]"
FT                       /IMGT_allele="IGHV3-7-2*01"
FT                       /IMGT_gene="IGHV3-7-2"
FT                       /IMGT_note="restored translation in 3'V-REGION and 
FT                       CDR3-IMGT"
FT                       /out_of_frame
FT                       /pseudo="STOP-CODON in L-PART1, several STOP-CODON in 
FT                       V-REGION, frameshift in V-REGION: insertion of 1 nt in 
FT                       FR3-IMGT"
FT   FR1-IMGT            796666..796740
FT                       /translation="EVQLIESIEDLRQSGKFLRLSCLAS"
FT                       /AA_IMGT="AA 1 to 26, AA 10 is missing"
FT   1st-CYS             796729..796731
FT   CDR1-IMGT           796741..796764
FT                       /AA_IMGT="AA 27 to 38, AA 31, 32, 33 and 34 are 
FT                       missing"
FT                       /IMGT_note="STOP-CODON in CDR1-IMGT: position 38"
FT   FR2-IMGT            796765..796815
FT                       /AA_IMGT="AA 39 to 55"
FT                       /IMGT_note="STOP-CODON in FR2-IMGT: position 52"
FT   CONSERVED-TRP       796771..796773
FT   CDR2-IMGT           796816..796839
FT                       /translation="IKYDGSQI"
FT                       /AA_IMGT="AA 56 to 65, AA 60 and 61 are missing"
FT   FR3-IMGT            796840..796954
FT                       /IMGT_note="frameshift in FR3-IMGT: insertion of 1 nt"
FT                       /out_of_frame
FT   3'V-REGION          796952..796964
FT                       /out_of_frame
FT                       /restored_translation
FT   CDR3-IMGT           796955..796964
FT                       /AA_IMGT="AA 105 to 107"
FT                       /out_of_frame
FT                       /restored_translation
FT   3'UTR               796965..797659
FT   V-RS                796965..797003
FT   V-HEPTAMER          796965..796971
FT                       /IMGT_note="noncanonical V-HEPTAMER: ccaagtg instead 
FT                       of cacagtg"
FT   V-SPACER            796972..796994
FT   V-NONAMER           796995..797003
FT   V-GENE              797660..807954
FT                       /IMGT_allele="IGHV(II)-7-1*01"
FT                       /IMGT_gene="IGHV(II)-7-1"
FT                       /pseudo="no L-PART1, no L-PART2, frameshifts in 
FT                       V-REGION: several insertions and deletions"
FT   5'UTR               797660..798351
FT   V-REGION            798352..798629
FT                       /CDR_length="[X.X.2]"
FT                       /FR_length="[25.16.29.G]"
FT                       /IMGT_allele="IGHV(II)-7-1*01"
FT                       /IMGT_gene="IGHV(II)-7-1"
FT                       /IMGT_note="restored translation in FR2-IMGT, 
FT                       CDR2-IMGT"
FT                       /out_of_frame
FT                       /pseudo="no L-PART1, no L-PART2, frameshifts in 
FT                       V-REGION: several insertions and deletions"
FT                       /putative_limit="5'"
FT   FR1-IMGT            798352..798426
FT                       /translation="QQLCFSAHFWLETSLQTLSLTCTVS"
FT                       /AA_IMGT="AA 1 to 26, AA 10 is missing"
FT                       /putative_limit="5'"
FT   1st-CYS             798415..798417
FT   CDR1-IMGT           798427..798455
FT                       /translation="GFPIITSAS"
FT                       /IMGT_note="frameshift in CDR1-IMGT: insertion of 5 nt"
FT                       /out_of_frame
FT   FR2-IMGT            798456..798503
FT                       /translation="LNYICLPLDGQEWIRC"
FT                       /IMGT_note="deletions in FR2-IMGT: 1 AA is missing"
FT                       /out_of_frame
FT                       /restored_translation
FT   CDR2-IMGT           798504..798534
FT                       /translation="MGCEGRTNYN"
FT                       /IMGT_note="frameshift in CDR2-IMGT: insertion of 7 nt"
FT                       /out_of_frame
FT                       /restored_translation
FT   FR3-IMGT            798535..798621
FT                       /IMGT_note="deletions in FR3-IMGT: 6 AA are missing, 
FT                       deletions in FR3-IMGT: 2 AA are missing, deletions in 
FT                       FR3-IMGT: 1 AA is missing"
FT                       /in_frame
FT   3'V-REGION          798619..798629
FT                       /in_frame
FT   CDR3-IMGT           798622..798629
FT                       /translation="AK"
FT                       /AA_IMGT="AA 105 to 106"
FT                       /in_frame
FT   3'UTR               798630..807954
FT   V-RS                798630..798668
FT   V-HEPTAMER          798630..798636
FT   V-SPACER            798637..798659
FT   V-NONAMER           798660..798668
FT   V-GENE              807955..820791
FT                       /functional
FT                       /IMGT_allele="IGHV3-7*03"
FT                       /IMGT_gene="IGHV3-7"
FT   5'UTR               807955..817240
FT   L-V-GENE-UNIT       817241..817735
FT                       /functional
FT                       /IMGT_allele="IGHV3-7*03"
FT                       /IMGT_gene="IGHV3-7"
FT   L-INTRON-L          817241..817400
FT   L-PART1             817241..817286
FT                       /translation="MELGLSWVFLVAILE"
FT   INIT-CODON          817241..817243
FT   DONOR-SPLICE        817286..817288
FT   V-INTRON            817287..817389
FT   ACCEPTOR-SPLICE     817387..817391
FT   V-EXON              817390..817696
FT                       /codon_start=3
FT                       /translation="VQCEVQLVESGGGLVQPGGSLRLSCAASGFTFSSYWMSWVR
FT                       QAPGKGLEWVANIKQDGSEKYYVDSVKGRFTISRDNAKNSLYLQMNSLRAEDTAV
FT                       YYCAR"
FT   L-PART2             817390..817400
FT                       /codon_start=3
FT                       /translation="VQC"
FT   V-REGION            817401..817696
FT                       /translation="EVQLVESGGGLVQPGGSLRLSCAASGFTFSSYWMSWVRQAP
FT                       GKGLEWVANIKQDGSEKYYVDSVKGRFTISRDNAKNSLYLQMNSLRAEDTAVYYC
FT                       AR"
FT                       /CDR_length="[8.8.2]"
FT                       /FR_length="[25.17.38.G]"
FT                       /functional
FT                       /IMGT_allele="IGHV3-7*03"
FT                       /IMGT_gene="IGHV3-7"
FT   FR1-IMGT            817401..817475
FT                       /translation="EVQLVESGGGLVQPGGSLRLSCAAS"
FT                       /AA_IMGT="AA 1 to 26, AA 10 is missing"
FT   1st-CYS             817464..817466
FT   CDR1-IMGT           817476..817499
FT                       /translation="GFTFSSYW"
FT                       /AA_IMGT="AA 27 to 38, AA 31, 32, 33 and 34 are 
FT                       missing"
FT   FR2-IMGT            817500..817550
FT                       /translation="MSWVRQAPGKGLEWVAN"
FT                       /AA_IMGT="AA 39 to 55"
FT   CONSERVED-TRP       817506..817508
FT   CDR2-IMGT           817551..817574
FT                       /translation="IKQDGSEK"
FT                       /AA_IMGT="AA 56 to 65, AA 60 and 61 are missing"
FT   FR3-IMGT            817575..817688
FT                       /translation="YYVDSVKGRFTISRDNAKNSLYLQMNSLRAEDTAVYYC"
FT                       /AA_IMGT="AA 66 to 104, AA 73 is missing"
FT   3'V-REGION          817686..817696
FT                       /translation="CAR"
FT   2nd-CYS             817686..817688
FT   CDR3-IMGT           817689..817696
FT                       /translation="AR"
FT                       /AA_IMGT="AA 105 to 106"
FT   3'UTR               817697..820791
FT   V-RS                817697..817735
FT   V-HEPTAMER          817697..817703
FT   V-SPACER            817704..817726
FT   V-NONAMER           817727..817735
FT   V-GENE              820792..829162
FT                       /IMGT_allele="IGHV3-6*02"
FT                       /IMGT_gene="IGHV3-6"
FT                       /pseudo="frameshift in V-REGION: deletion of 1 nt in 
FT                       FR1-IMGT"
FT   5'UTR               820792..823846
FT   L-V-GENE-UNIT       823847..824340
FT                       /IMGT_allele="IGHV3-6*02"
FT                       /IMGT_gene="IGHV3-6"
FT                       /pseudo="frameshift in V-REGION: deletion of 1 nt in 
FT                       FR1-IMGT"
FT   L-INTRON-L          823847..824006
FT   L-PART1             823847..823892
FT                       /translation="MELGLRWVFLAAILK"
FT   INIT-CODON          823847..823849
FT   DONOR-SPLICE        823892..823894
FT   V-INTRON            823893..823995
FT   ACCEPTOR-SPLICE     823993..823997
FT   V-EXON              823996..824301
FT                       /codon_start=3
FT                       /out_of_frame
FT   L-PART2             823996..824006
FT                       /codon_start=3
FT                       /translation="VQC"
FT   V-REGION            824007..824301
FT                       /CDR_length="[8.8.2]"
FT                       /FR_length="[X.17.38.G]"
FT                       /IMGT_allele="IGHV3-6*02"
FT                       /IMGT_gene="IGHV3-6"
FT                       /IMGT_note="restored translation in CDR1-IMGT, 
FT                       FR2-IMGT, CDR2-IMGT, FR3-IMGT, 3'V-REGION and 
FT                       CDR3-IMGT"
FT                       /out_of_frame
FT                       /pseudo="frameshift in V-REGION: deletion of 1 nt in 
FT                       FR1-IMGT"
FT   FR1-IMGT            824007..824080
FT                       /translation="EMQLVESGANLTKPGCPRDSPVQP"
FT                       /IMGT_note="frameshift in FR1-IMGT: deletion of 1 nt"
FT                       /out_of_frame
FT   CDR1-IMGT           824081..824104
FT                       /translation="GFTFSSHS"
FT                       /AA_IMGT="AA 27 to 38, AA 31, 32, 33 and 34 are 
FT                       missing"
FT                       /out_of_frame
FT                       /restored_translation
FT   FR2-IMGT            824105..824155
FT                       /translation="THWVPQAPGKGLQWVPV"
FT                       /AA_IMGT="AA 39 to 55"
FT                       /out_of_frame
FT                       /restored_translation
FT   CDR2-IMGT           824156..824179
FT                       /translation="ISGSGSTM"
FT                       /AA_IMGT="AA 56 to 65, AA 60 and 61 are missing"
FT                       /out_of_frame
FT                       /restored_translation
FT   FR3-IMGT            824180..824293
FT                       /translation="YYADSVKGRFTISRDNTKNSLYLQMNRLRAEDAAAYDS"
FT                       /AA_IMGT="AA 66 to 104, AA 73 is missing"
FT                       /out_of_frame
FT                       /restored_translation
FT   3'V-REGION          824291..824301
FT                       /translation="SVR"
FT                       /out_of_frame
FT                       /restored_translation
FT   CDR3-IMGT           824294..824301
FT                       /translation="VR"
FT                       /AA_IMGT="AA 105 to 106"
FT                       /out_of_frame
FT                       /restored_translation
FT   3'UTR               824302..829162
FT   V-RS                824302..824340
FT   V-HEPTAMER          824302..824308
FT                       /IMGT_note="noncanonical V-HEPTAMER: tacggta instead 
FT                       of cacagtg"
FT   V-SPACER            824309..824331
FT   V-NONAMER           824332..824340
FT   V-GENE              829163..837182
FT                       /IMGT_allele="IGHV(III)-5-2*01"
FT                       /IMGT_gene="IGHV(III)-5-2"
FT                       /pseudo="truncated pseudogene not assigned to 
FT                       subgroups with functional genes"
FT   5'UTR               829163..833984
FT   V-REGION            <833985..834245
FT                       /partial
FT                       /codon_start=3
FT                       /CDR_length="[8.9.2]"
FT                       /FR_length="[X.17.X.G]"
FT                       /IMGT_allele="IGHV(III)-5-2*01"
FT                       /IMGT_gene="IGHV(III)-5-2"
FT                       /IMGT_note="partial gene in 5', restored translation 
FT                       in 3'V-REGION and CDR3-IMGT"
FT                       /out_of_frame
FT                       /pseudo="truncated pseudogene not assigned to 
FT                       subgroups with functional genes"
FT                       /putative_limit="5'"
FT   FR1-IMGT            <833985..834022
FT                       /partial
FT                       /codon_start=3
FT                       /translation="PGYSLSFFCKAS"
FT                       /putative_limit="5'"
FT   1st-CYS             834011..834013
FT   CDR1-IMGT           834023..834046
FT                       /translation="RFTFTDCS"
FT                       /AA_IMGT="AA 27 to 38, AA 31, 32, 33 and 34 are 
FT                       missing"
FT   FR2-IMGT            834047..834097
FT                       /translation="IYWALMAGGEGLEWVVA"
FT                       /AA_IMGT="AA 39 to 55"
FT   CONSERVED-TRP       834053..834055
FT   CDR2-IMGT           834098..834124
FT                       /translation="MSDPSGSSQ"
FT                       /AA_IMGT="AA 56 to 65, AA 61 is missing"
FT   FR3-IMGT            834125..834239
FT                       /IMGT_note="frameshift in FR3-IMGT: insertion of 1 nt"
FT                       /out_of_frame
FT   3'V-REGION          834237..834245
FT                       /translation="CER"
FT                       /out_of_frame
FT                       /restored_translation
FT   CDR3-IMGT           834240..834245
FT                       /translation="ER"
FT                       /AA_IMGT="AA 105 to 106"
FT                       /out_of_frame
FT                       /restored_translation
FT   3'UTR               834246..837182
FT   V-RS                834246..834283
FT   V-HEPTAMER          834246..834252
FT   V-SPACER            834253..834274
FT   V-NONAMER           834275..834283
FT   V-GENE              837183..840857
FT                       /IMGT_allele="IGHV(III)-5-1*01"
FT                       /IMGT_gene="IGHV(III)-5-1"
FT                       /pseudo="truncated pseudogene not assigned to 
FT                       subgroups with functional genes"
FT   5'UTR               837183..840080
FT   V-REGION            <840081..840179
FT                       /partial
FT                       /codon_start=3
FT                       /translation="FIISRDNVKKMLFLQMGNLQTKDTSLHYCARE"
FT                       /CDR_length="[X.X.3]"
FT                       /FR_length="[X.X.X.G]"
FT                       /IMGT_allele="IGHV(III)-5-1*01"
FT                       /IMGT_gene="IGHV(III)-5-1"
FT                       /IMGT_note="partial gene in 5'"
FT                       /pseudo="truncated pseudogene not assigned to 
FT                       subgroups with functional genes"
FT                       /putative_limit="5'"
FT   FR3-IMGT            <840081..840169
FT                       /partial
FT                       /codon_start=3
FT                       /translation="FIISRDNVKKMLFLQMGNLQTKDTSLHYC"
FT   3'V-REGION          840167..840179
FT                       /translation="CARE"
FT   2nd-CYS             840167..840169
FT   CDR3-IMGT           840170..840179
FT                       /translation="ARE"
FT                       /AA_IMGT="AA 105 to 107"
FT   3'UTR               840180..840857
FT   V-RS                840180..840212
FT   V-HEPTAMER          840180..840186
FT   V-SPACER            840187..840203
FT                       /IMGT_note="unexpected V-SPACER length: 17 nt instead 
FT                       of 23 nt"
FT   V-NONAMER           840204..840212
FT   V-GENE              840858..848000
FT                       /functional="noncanonical V-HEPTAMER: cacaaag instead 
FT                       of cacagtg, however this V-GENE has been seen 
FT                       rearranged in vivo"
FT                       /IMGT_allele="IGHV2-5*02"
FT                       /IMGT_gene="IGHV2-5"
FT   5'UTR               840858..841499
FT   L-V-GENE-UNIT       841500..841982
FT                       /functional="noncanonical V-HEPTAMER: cacaaag instead 
FT                       of cacagtg, however this V-GENE has been seen 
FT                       rearranged in vivo"
FT                       /IMGT_allele="IGHV2-5*02"
FT                       /IMGT_gene="IGHV2-5"
FT   L-INTRON-L          841500..841642
FT   L-PART1             841500..841545
FT                       /translation="MDTLCSTLLLLTIPS"
FT   INIT-CODON          841500..841502
FT   DONOR-SPLICE        841545..841547
FT   V-INTRON            841546..841631
FT   ACCEPTOR-SPLICE     841629..841633
FT   V-EXON              841632..841943
FT                       /codon_start=3
FT                       /translation="VLSQITLKESGPTLVKPTQTLTLTCTFSGFSLSTSGVGVGW
FT                       IRQPPGKALEWLALIYWDDDKRYSPSLKSRLTITKDTSKNQVVLTMTNMDPVDTA
FT                       TYYCAHR"
FT   L-PART2             841632..841642
FT                       /codon_start=3
FT                       /translation="VLS"
FT   V-REGION            841643..841943
FT                       /translation="QITLKESGPTLVKPTQTLTLTCTFSGFSLSTSGVGVGWIRQ
FT                       PPGKALEWLALIYWDDDKRYSPSLKSRLTITKDTSKNQVVLTMTNMDPVDTATYY
FT                       CAHR"
FT                       /CDR_length="[10.7.3]"
FT                       /FR_length="[25.17.38.G]"
FT                       /functional="noncanonical V-HEPTAMER: cacaaag instead 
FT                       of cacagtg, however this V-GENE has been seen 
FT                       rearranged in vivo"
FT                       /IMGT_allele="IGHV2-5*02"
FT                       /IMGT_gene="IGHV2-5"
FT   FR1-IMGT            841643..841717
FT                       /translation="QITLKESGPTLVKPTQTLTLTCTFS"
FT                       /AA_IMGT="AA 1 to 26, AA 10 is missing"
FT   1st-CYS             841706..841708
FT   CDR1-IMGT           841718..841747
FT                       /translation="GFSLSTSGVG"
FT                       /AA_IMGT="AA 27 to 38, AA 32 and 33 are missing"
FT   FR2-IMGT            841748..841798
FT                       /translation="VGWIRQPPGKALEWLAL"
FT                       /AA_IMGT="AA 39 to 55"
FT   CONSERVED-TRP       841754..841756
FT   CDR2-IMGT           841799..841819
FT                       /translation="IYWDDDK"
FT                       /AA_IMGT="AA 56 to 65, AA 60, 61 and 62 are missing"
FT   FR3-IMGT            841820..841933
FT                       /translation="RYSPSLKSRLTITKDTSKNQVVLTMTNMDPVDTATYYC"
FT                       /AA_IMGT="AA 66 to 104, AA 73 is missing"
FT   3'V-REGION          841931..841943
FT                       /translation="CAHR"
FT   2nd-CYS             841931..841933
FT   CDR3-IMGT           841934..841943
FT                       /translation="AHR"
FT                       /AA_IMGT="AA 105 to 107"
FT   3'UTR               841944..848000
FT   V-RS                841944..841982
FT   V-HEPTAMER          841944..841950
FT                       /IMGT_note="noncanonical V-HEPTAMER: cacaaag instead 
FT                       of cacagtg"
FT   V-SPACER            841951..841973
FT   V-NONAMER           841974..841982
FT   V-GENE              848001..856179
FT                       /functional
FT                       /IMGT_allele="IGHV7-4-1*01"
FT                       /IMGT_gene="IGHV7-4-1"
FT   5'UTR               848001..854263
FT   L-V-GENE-UNIT       854264..854739
FT                       /functional
FT                       /IMGT_allele="IGHV7-4-1*01"
FT                       /IMGT_gene="IGHV7-4-1"
FT   L-INTRON-L          854264..854404
FT   L-PART1             854264..854309
FT                       /translation="MDWTWRILFLVAAAT"
FT   INIT-CODON          854264..854266
FT   DONOR-SPLICE        854309..854311
FT   V-INTRON            854310..854393
FT   ACCEPTOR-SPLICE     854391..854395
FT   V-EXON              854394..854700
FT                       /codon_start=3
FT                       /translation="AHSQVQLVQSGSELKKPGASVKVSCKASGYTFTSYAMNWVR
FT                       QAPGQGLEWMGWINTNTGNPTYAQGFTGRFVFSLDTSVSTAYLQICSLKAEDTAV
FT                       YYCAR"
FT   L-PART2             854394..854404
FT                       /codon_start=3
FT                       /translation="AHS"
FT   V-REGION            854405..854700
FT                       /translation="QVQLVQSGSELKKPGASVKVSCKASGYTFTSYAMNWVRQAP
FT                       GQGLEWMGWINTNTGNPTYAQGFTGRFVFSLDTSVSTAYLQICSLKAEDTAVYYC
FT                       AR"
FT                       /CDR_length="[8.8.2]"
FT                       /FR_length="[25.17.38.G]"
FT                       /functional
FT                       /IMGT_allele="IGHV7-4-1*01"
FT                       /IMGT_gene="IGHV7-4-1"
FT   FR1-IMGT            854405..854479
FT                       /translation="QVQLVQSGSELKKPGASVKVSCKAS"
FT                       /AA_IMGT="AA 1 to 26, AA 10 is missing"
FT   1st-CYS             854468..854470
FT   CDR1-IMGT           854480..854503
FT                       /translation="GYTFTSYA"
FT                       /AA_IMGT="AA 27 to 38, AA 31, 32, 33 and 34 are 
FT                       missing"
FT   FR2-IMGT            854504..854554
FT                       /translation="MNWVRQAPGQGLEWMGW"
FT                       /AA_IMGT="AA 39 to 55"
FT   CONSERVED-TRP       854510..854512
FT   CDR2-IMGT           854555..854578
FT                       /translation="INTNTGNP"
FT                       /AA_IMGT="AA 56 to 65, AA 60 and 61 are missing"
FT   FR3-IMGT            854579..854692
FT                       /translation="TYAQGFTGRFVFSLDTSVSTAYLQICSLKAEDTAVYYC"
FT                       /AA_IMGT="AA 66 to 104, AA 73 is missing"
FT   3'V-REGION          854690..854700
FT                       /translation="CAR"
FT   2nd-CYS             854690..854692
FT   CDR3-IMGT           854693..854700
FT                       /translation="AR"
FT                       /AA_IMGT="AA 105 to 106"
FT   3'UTR               854701..856179
FT   V-RS                854701..854739
FT   V-HEPTAMER          854701..854707
FT   V-SPACER            854708..854730
FT   V-NONAMER           854731..854739
FT   V-GENE              856180..862719
FT                       /IMGT_allele="IGHV(II)-4-2*01"
FT                       /IMGT_gene="IGHV(II)-4-2"
FT                       /pseudo="no L-PART1, frameshifts in V-REGION: several 
FT                       insertions and deletions"
FT   5'UTR               856180..857558
FT   ACCEPTOR-SPLICE     857556..857560
FT   V-EXON              857559..857912
FT                       /codon_start=3
FT                       /out_of_frame
FT   L-PART2             857559..857569
FT                       /codon_start=3
FT                       /translation="VLS"
FT   V-REGION            857570..857912
FT                       /CDR_length="[10.X.2]"
FT                       /FR_length="[X.17.X.G]"
FT                       /IMGT_allele="IGHV(II)-4-2*01"
FT                       /IMGT_gene="IGHV(II)-4-2"
FT                       /IMGT_note="restored translation in CDR1-IMGT, 
FT                       FR2-IMGT, CDR2-IMGT, 3'V-REGION and CDR3-IMGT"
FT                       /out_of_frame
FT                       /pseudo="no L-PART1, frameshifts in V-REGION: several 
FT                       insertions and deletions"
FT   FR1-IMGT            857570..857642
FT                       /translation="QVQLQESGPGPMRPSDTALHLCCV"
FT                       /IMGT_note="frameshift in FR1-IMGT: deletion of 2 nt"
FT                       /out_of_frame
FT   CDR1-IMGT           857643..857672
FT                       /translation="GFFFFTGGYY"
FT                       /AA_IMGT="AA 27 to 38, AA 32 and 33 are missing"
FT                       /out_of_frame
FT                       /restored_translation
FT   FR2-IMGT            857673..857723
FT                       /AA_IMGT="AA 39 to 55"
FT                       /out_of_frame
FT                       /restored_translation
FT   CDR2-IMGT           857724..857782
FT                       /IMGT_note="frameshift in CDR2-IMGT: insertion of 38 
FT                       nt"
FT                       /out_of_frame
FT                       /restored_translation
FT   FR3-IMGT            857783..857906
FT                       /IMGT_note="frameshift in FR3-IMGT: insertion of 10 nt"
FT                       /out_of_frame
FT   3'V-REGION          857904..857914
FT                       /translation="CER"
FT                       /out_of_frame
FT                       /restored_translation
FT   CDR3-IMGT           857907..857914
FT                       /translation="ER"
FT                       /AA_IMGT="AA 105 to 106"
FT                       /out_of_frame
FT                       /restored_translation
FT   3'UTR               857913..862719
FT   V-RS                857913..857951
FT   V-HEPTAMER          857913..857919
FT   V-SPACER            857920..857942
FT   V-NONAMER           857943..857951
FT   V-GENE              862720..871137
FT                       /functional
FT                       /IMGT_allele="IGHV4-4*02"
FT                       /IMGT_gene="IGHV4-4"
FT   5'UTR               862720..867488
FT   L-V-GENE-UNIT       867489..867962
FT                       /functional
FT                       /IMGT_allele="IGHV4-4*02"
FT                       /IMGT_gene="IGHV4-4"
FT   L-INTRON-L          867489..867627
FT   L-PART1             867489..867534
FT                       /translation="MKHLWFFLLLVAAPR"
FT   INIT-CODON          867489..867491
FT   DONOR-SPLICE        867534..867536
FT   V-INTRON            867535..867616
FT   ACCEPTOR-SPLICE     867614..867618
FT   V-EXON              867617..867923
FT                       /codon_start=3
FT                       /translation="VLSQVQLQESGPGLVKPSGTLSLTCAVSGGSISSSNWWSWV
FT                       RQPPGKGLEWIGEIYHSGSTNYNPSLKSRVTISVDKSKNQFSLKLSSVTAADTAV
FT                       YYCAR"
FT   L-PART2             867617..867627
FT                       /codon_start=3
FT                       /translation="VLS"
FT   V-REGION            867628..867923
FT                       /translation="QVQLQESGPGLVKPSGTLSLTCAVSGGSISSSNWWSWVRQP
FT                       PGKGLEWIGEIYHSGSTNYNPSLKSRVTISVDKSKNQFSLKLSSVTAADTAVYYC
FT                       AR"
FT                       /CDR_length="[9.7.2]"
FT                       /FR_length="[25.17.38.G]"
FT                       /functional
FT                       /IMGT_allele="IGHV4-4*02"
FT                       /IMGT_gene="IGHV4-4"
FT   FR1-IMGT            867628..867702
FT                       /translation="QVQLQESGPGLVKPSGTLSLTCAVS"
FT                       /AA_IMGT="AA 1 to 26, AA 10 is missing"
FT   1st-CYS             867691..867693
FT   CDR1-IMGT           867703..867729
FT                       /translation="GGSISSSNW"
FT                       /AA_IMGT="AA 27 to 38, AA 32, 33 and 34 are missing"
FT   FR2-IMGT            867730..867780
FT                       /translation="WSWVRQPPGKGLEWIGE"
FT                       /AA_IMGT="AA 39 to 55"
FT   CONSERVED-TRP       867736..867738
FT   CDR2-IMGT           867781..867801
FT                       /translation="IYHSGST"
FT                       /AA_IMGT="AA 56 to 65, AA 60, 61 and 62 are missing"
FT   FR3-IMGT            867802..867915
FT                       /translation="NYNPSLKSRVTISVDKSKNQFSLKLSSVTAADTAVYYC"
FT                       /AA_IMGT="AA 66 to 104, AA 73 is missing"
FT   3'V-REGION          867913..867923
FT                       /translation="CAR"
FT   2nd-CYS             867913..867915
FT   CDR3-IMGT           867916..867923
FT                       /translation="AR"
FT                       /AA_IMGT="AA 105 to 106"
FT   3'UTR               867924..871137
FT   V-RS                867924..867962
FT   V-HEPTAMER          867924..867930
FT   V-SPACER            867931..867953
FT   V-NONAMER           867954..867962
FT   V-GENE              871138..876358
FT                       /functional
FT                       /IMGT_allele="IGHV1-3*01"
FT                       /IMGT_gene="IGHV1-3"
FT   5'UTR               871138..874312
FT   L-V-GENE-UNIT       874313..874789
FT                       /functional
FT                       /IMGT_allele="IGHV1-3*01"
FT                       /IMGT_gene="IGHV1-3"
FT   L-INTRON-L          874313..874454
FT   L-PART1             874313..874358
FT                       /translation="MDWTWRILFLVAAAT"
FT   INIT-CODON          874313..874315
FT   DONOR-SPLICE        874358..874360
FT   V-INTRON            874359..874443
FT   ACCEPTOR-SPLICE     874441..874445
FT   V-EXON              874444..874750
FT                       /codon_start=3
FT                       /translation="AHSQVQLVQSGAEVKKPGASVKVSCKASGYTFTSYAMHWVR
FT                       QAPGQRLEWMGWINAGNGNTKYSQKFQGRVTITRDTSASTAYMELSSLRSEDTAV
FT                       YYCAR"
FT   L-PART2             874444..874454
FT                       /codon_start=3
FT                       /translation="AHS"
FT   V-REGION            874455..874750
FT                       /translation="QVQLVQSGAEVKKPGASVKVSCKASGYTFTSYAMHWVRQAP
FT                       GQRLEWMGWINAGNGNTKYSQKFQGRVTITRDTSASTAYMELSSLRSEDTAVYYC
FT                       AR"
FT                       /CDR_length="[8.8.2]"
FT                       /FR_length="[25.17.38.G]"
FT                       /functional
FT                       /IMGT_allele="IGHV1-3*01"
FT                       /IMGT_gene="IGHV1-3"
FT   FR1-IMGT            874455..874529
FT                       /translation="QVQLVQSGAEVKKPGASVKVSCKAS"
FT                       /AA_IMGT="AA 1 to 26, AA 10 is missing"
FT   1st-CYS             874518..874520
FT   CDR1-IMGT           874530..874553
FT                       /translation="GYTFTSYA"
FT                       /AA_IMGT="AA 27 to 38, AA 31, 32, 33 and 34 are 
FT                       missing"
FT   FR2-IMGT            874554..874604
FT                       /translation="MHWVRQAPGQRLEWMGW"
FT                       /AA_IMGT="AA 39 to 55"
FT   CONSERVED-TRP       874560..874562
FT   CDR2-IMGT           874605..874628
FT                       /translation="INAGNGNT"
FT                       /AA_IMGT="AA 56 to 65, AA 60 and 61 are missing"
FT   FR3-IMGT            874629..874742
FT                       /translation="KYSQKFQGRVTITRDTSASTAYMELSSLRSEDTAVYYC"
FT                       /AA_IMGT="AA 66 to 104, AA 73 is missing"
FT   3'V-REGION          874740..874750
FT                       /translation="CAR"
FT   2nd-CYS             874740..874742
FT   CDR3-IMGT           874743..874750
FT                       /translation="AR"
FT                       /AA_IMGT="AA 105 to 106"
FT   3'UTR               874751..876358
FT   V-RS                874751..874789
FT   V-HEPTAMER          874751..874757
FT   V-SPACER            874758..874780
FT   V-NONAMER           874781..874789
FT   V-GENE              876359..886000
FT                       /IMGT_allele="IGHV(III)-2-1*02"
FT                       /IMGT_gene="IGHV(III)-2-1"
FT                       /pseudo="no L-PART1, frameshifts in V-REGION: several 
FT                       insertions and deletions"
FT   5'UTR               876359..877927
FT   ACCEPTOR-SPLICE     878007..878011
FT                       /IMGT_note="noncanonical ACCEPTOR-SPLICE: nggnn 
FT                       instead of nagnn"
FT   V-EXON              878010..878311
FT                       /codon_start=3
FT                       /out_of_frame
FT   L-PART2             878010..878020
FT                       /codon_start=3
FT                       /translation="VLC"
FT   V-REGION            878021..878311
FT                       /codon_start=3
FT                       /CDR_length="[8.X.2]"
FT                       /FR_length="[X.X.X.G]"
FT                       /IMGT_allele="IGHV(III)-2-1*02"
FT                       /IMGT_gene="IGHV(III)-2-1"
FT                       /out_of_frame
FT                       /pseudo="no L-PART1, frameshifts in V-REGION: several 
FT                       insertions and deletions"
FT   FR1-IMGT            878021..878091
FT                       /codon_start=3
FT                       /translation="SLLMESEGEKFYSPVVRLSCKAS"
FT   1st-CYS             878080..878082
FT   CDR1-IMGT           878092..878115
FT                       /translation="GFTFTGYS"
FT                       /AA_IMGT="AA 27 to 38, AA 31, 32, 33 and 34 are 
FT                       missing"
FT   FR2-IMGT            878116..878183
FT   CDR2-IMGT           878184..878211
FT   FR3-IMGT            878212..878305
FT   3'V-REGION          878303..878311
FT   2nd-CYS             878303..878305
FT   CDR3-IMGT           878306..878311
FT                       /AA_IMGT="AA 105 to 106"
FT   3'UTR               878312..886000
FT   V-RS                878312..878350
FT   V-HEPTAMER          878312..878318
FT   V-SPACER            878319..878341
FT   V-NONAMER           878342..878350
FT   V-GENE              886001..897000
FT                       /functional
FT                       /IMGT_allele="IGHV1-2*04"
FT                       /IMGT_gene="IGHV1-2"
FT   5'UTR               886001..892825
FT   L-V-GENE-UNIT       892826..893302
FT                       /functional
FT                       /IMGT_allele="IGHV1-2*04"
FT                       /IMGT_gene="IGHV1-2"
FT   L-INTRON-L          892826..892967
FT   L-PART1             892826..892871
FT                       /translation="MDWTWRILFLVAAAT"
FT   INIT-CODON          892826..892828
FT   DONOR-SPLICE        892871..892873
FT   V-INTRON            892872..892956
FT   ACCEPTOR-SPLICE     892954..892958
FT   V-EXON              892957..893263
FT                       /codon_start=3
FT                       /translation="AHSQVQLVQSGAEVKKPGASVKVSCKASGYTFTGYYMHWVR
FT                       QAPGQGLEWMGWINPNSGGTNYAQKFQGWVTMTRDTSISTAYMELSRLRSDDTAV
FT                       YYCAR"
FT   L-PART2             892957..892967
FT                       /codon_start=3
FT                       /translation="AHS"
FT   V-REGION            892968..893263
FT                       /translation="QVQLVQSGAEVKKPGASVKVSCKASGYTFTGYYMHWVRQAP
FT                       GQGLEWMGWINPNSGGTNYAQKFQGWVTMTRDTSISTAYMELSRLRSDDTAVYYC
FT                       AR"
FT                       /CDR_length="[8.8.2]"
FT                       /FR_length="[25.17.38.G]"
FT                       /functional
FT                       /IMGT_allele="IGHV1-2*04"
FT                       /IMGT_gene="IGHV1-2"
FT   FR1-IMGT            892968..893042
FT                       /translation="QVQLVQSGAEVKKPGASVKVSCKAS"
FT                       /AA_IMGT="AA 1 to 26, AA 10 is missing"
FT   1st-CYS             893031..893033
FT   CDR1-IMGT           893043..893066
FT                       /translation="GYTFTGYY"
FT                       /AA_IMGT="AA 27 to 38, AA 31, 32, 33 and 34 are 
FT                       missing"
FT   FR2-IMGT            893067..893117
FT                       /translation="MHWVRQAPGQGLEWMGW"
FT                       /AA_IMGT="AA 39 to 55"
FT   CONSERVED-TRP       893073..893075
FT   CDR2-IMGT           893118..893141
FT                       /translation="INPNSGGT"
FT                       /AA_IMGT="AA 56 to 65, AA 60 and 61 are missing"
FT   FR3-IMGT            893142..893255
FT                       /translation="NYAQKFQGWVTMTRDTSISTAYMELSRLRSDDTAVYYC"
FT                       /AA_IMGT="AA 66 to 104, AA 73 is missing"
FT   3'V-REGION          893253..893263
FT                       /translation="CAR"
FT   2nd-CYS             893253..893255
FT   CDR3-IMGT           893256..893263
FT                       /translation="AR"
FT                       /AA_IMGT="AA 105 to 106"
FT   3'UTR               893264..897000
FT   V-RS                893264..893302
FT   V-HEPTAMER          893264..893270
FT   V-SPACER            893271..893293
FT   V-NONAMER           893294..893302
FT   GENE                897001..904500
FT                       /IMGT_gene="RPS8P1"
FT                       /pseudo
FT   5'UTR               897001..900675
FT   GENE-UNIT           900676..901377
FT                       /IMGT_gene="RPS8P1"
FT                       /pseudo
FT   3'UTR               901378..904500
FT   GENE                904501..922000
FT                       /IMGT_gene="ADAM6"
FT                       /pseudo
FT   5'UTR               904501..907575
FT   GENE-UNIT           907576..910116
FT                       /IMGT_gene="ADAM6"
FT                       /pseudo
FT   3'UTR               910117..922000
FT   V-GENE              922001..937159
FT                       /IMGT_allele="IGHV(II)-1-1*01"
FT                       /IMGT_gene="IGHV(II)-1-1"
FT                       /pseudo="no L-PART1, no ACCEPTOR-SPLICE, frameshift in 
FT                       V-REGION: deletion of 52 nt in FR1-IMGT"
FT   5'UTR               922001..934390
FT   V-EXON              934391..934637
FT                       /codon_start=3
FT                       /out_of_frame
FT   L-PART2             934391..934401
FT                       /codon_start=3
FT                       /translation="VLH"
FT                       /IMGT_note="no ACCEPTOR-SPLICE"
FT   V-REGION            934402..934637
FT                       /CDR_length="[X.7.2]"
FT                       /FR_length="[X.14.38.G]"
FT                       /IMGT_allele="IGHV(II)-1-1*01"
FT                       /IMGT_gene="IGHV(II)-1-1"
FT                       /IMGT_note="restored translation in FR2-IMGT, 
FT                       CDR2-IMGT, FR3-IMGT, 3'V-REGION and CDR3-IMGT"
FT                       /out_of_frame
FT                       /pseudo="no L-PART1, no ACCEPTOR-SPLICE, frameshift in 
FT                       V-REGION: deletion of 52 nt in FR1-IMGT"
FT   FR1-IMGT            934402..934454
FT                       /translation="QMQLLESCPGLVRSSQT"
FT                       /IMGT_note="frameshift in FR1-IMGT: deletion of 52 nt"
FT                       /out_of_frame
FT   FR2-IMGT            934455..934496
FT                       /translation="WAWTQQPSGKALGH"
FT                       /IMGT_note="deletions in FR2-IMGT: 3 AA are missing"
FT                       /out_of_frame
FT                       /restored_translation
FT   CDR2-IMGT           934497..934517
FT                       /translation="LSSRGST"
FT                       /AA_IMGT="AA 56 to 65, AA 60, 61 and 62 are missing"
FT                       /out_of_frame
FT                       /restored_translation
FT   FR3-IMGT            934518..934631
FT                       /translation="HFSPAFLGQLSICRDTSKAQLSLQLSSVMAKGRAAHSR"
FT                       /AA_IMGT="AA 66 to 104, AA 73 is missing"
FT                       /out_of_frame
FT                       /restored_translation
FT   3'V-REGION          934629..934637
FT                       /translation="RGR"
FT                       /out_of_frame
FT                       /restored_translation
FT   CDR3-IMGT           934632..934637
FT                       /translation="GR"
FT                       /AA_IMGT="AA 105 to 106"
FT                       /out_of_frame
FT                       /restored_translation
FT   3'UTR               934638..937159
FT   V-RS                934638..934674
FT   V-HEPTAMER          934638..934644
FT                       /IMGT_note="noncanonical V-HEPTAMER: cagaatg instead 
FT                       of cacagtg"
FT   V-SPACER            934645..934665
FT   V-NONAMER           934666..934674
FT   V-GENE              937160..950000
FT                       /functional
FT                       /IMGT_allele="IGHV6-1*01"
FT                       /IMGT_gene="IGHV6-1"
FT   5'UTR               937160..939643
FT   L-V-GENE-UNIT       939644..940130
FT                       /functional
FT                       /IMGT_allele="IGHV6-1*01"
FT                       /IMGT_gene="IGHV6-1"
FT   L-INTRON-L          939644..939786
FT   L-PART1             939644..939692
FT                       /translation="MSVSFLIFLPVLGLPW"
FT   INIT-CODON          939644..939646
FT   DONOR-SPLICE        939692..939694
FT   V-INTRON            939693..939775
FT   ACCEPTOR-SPLICE     939773..939777
FT   V-EXON              939776..940091
FT                       /codon_start=3
FT                       /translation="VLSQVQLQQSGPGLVKPSQTLSLTCAISGDSVSSNSAAWNW
FT                       IRQSPSRGLEWLGRTYYRSKWYNDYAVSVKSRITINPDTSKNQFSLQLNSVTPED
FT                       TAVYYCAR"
FT   L-PART2             939776..939786
FT                       /codon_start=3
FT                       /translation="VLS"
FT   V-REGION            939787..940091
FT                       /translation="QVQLQQSGPGLVKPSQTLSLTCAISGDSVSSNSAAWNWIRQ
FT                       SPSRGLEWLGRTYYRSKWYNDYAVSVKSRITINPDTSKNQFSLQLNSVTPEDTAV
FT                       YYCAR"
FT                       /CDR_length="[10.9.2]"
FT                       /FR_length="[25.17.38.G]"
FT                       /functional
FT                       /IMGT_allele="IGHV6-1*01"
FT                       /IMGT_gene="IGHV6-1"
FT   FR1-IMGT            939787..939861
FT                       /translation="QVQLQQSGPGLVKPSQTLSLTCAIS"
FT                       /AA_IMGT="AA 1 to 26, AA 10 is missing"
FT   1st-CYS             939850..939852
FT   CDR1-IMGT           939862..939891
FT                       /translation="GDSVSSNSAA"
FT                       /AA_IMGT="AA 27 to 38, AA 32 and 33 are missing"
FT   FR2-IMGT            939892..939942
FT                       /translation="WNWIRQSPSRGLEWLGR"
FT                       /AA_IMGT="AA 39 to 55"
FT   CONSERVED-TRP       939898..939900
FT   CDR2-IMGT           939943..939969
FT                       /translation="TYYRSKWYN"
FT                       /AA_IMGT="AA 56 to 65, AA 61 is missing"
FT   FR3-IMGT            939970..940083
FT                       /translation="DYAVSVKSRITINPDTSKNQFSLQLNSVTPEDTAVYYC"
FT                       /AA_IMGT="AA 66 to 104, AA 73 is missing"
FT   3'V-REGION          940081..940091
FT                       /translation="CAR"
FT   2nd-CYS             940081..940083
FT   CDR3-IMGT           940084..940091
FT                       /translation="AR"
FT                       /AA_IMGT="AA 105 to 106"
FT   3'UTR               940092..950000
FT   V-RS                940092..940130
FT   V-HEPTAMER          940092..940098
FT   V-SPACER            940099..940121
FT   V-NONAMER           940122..940130
FT   D-GENE              950001..961666
FT                       /functional
FT                       /IMGT_allele="IGHD1-1*01"
FT                       /IMGT_gene="IGHD1-1"
FT   5'UTR               950001..960326
FT   D-GENE-UNIT         960299..960371
FT                       /functional
FT                       /IMGT_allele="IGHD1-1*01"
FT                       /IMGT_gene="IGHD1-1"
FT   5'D-RS              960299..960326
FT   5'D-NONAMER         960299..960307
FT   5'D-SPACER          960308..960319
FT   5'D-HEPTAMER        960320..960326
FT   D-REGION            960327..960343
FT                       /translation="GTTGT"
FT                       /functional
FT                       /IMGT_allele="IGHD1-1*01"
FT                       /IMGT_gene="IGHD1-1"
FT   3'UTR               960344..961666
FT   3'D-RS              960344..960371
FT   3'D-HEPTAMER        960344..960350
FT   3'D-SPACER          960351..960362
FT   3'D-NONAMER         960363..960371
FT   D-GENE              961667..964237
FT                       /functional
FT                       /IMGT_allele="IGHD2-2*01"
FT                       /IMGT_gene="IGHD2-2"
FT   5'UTR               961667..962988
FT   D-GENE-UNIT         962961..963047
FT                       /functional
FT                       /IMGT_allele="IGHD2-2*01"
FT                       /IMGT_gene="IGHD2-2"
FT   5'D-RS              962961..962988
FT   5'D-NONAMER         962961..962969
FT   5'D-SPACER          962970..962981
FT   5'D-HEPTAMER        962982..962988
FT   D-REGION            962989..963019
FT                       /functional
FT                       /IMGT_allele="IGHD2-2*01"
FT                       /IMGT_gene="IGHD2-2"
FT   3'UTR               963020..964237
FT   3'D-RS              963020..963047
FT   3'D-HEPTAMER        963020..963026
FT   3'D-SPACER          963027..963038
FT   3'D-NONAMER         963039..963047
FT   D-GENE              964238..966047
FT                       /functional
FT                       /IMGT_allele="IGHD3-3*01"
FT                       /IMGT_gene="IGHD3-3"
FT   5'UTR               964238..965455
FT   D-GENE-UNIT         965428..965514
FT                       /functional
FT                       /IMGT_allele="IGHD3-3*01"
FT                       /IMGT_gene="IGHD3-3"
FT   5'D-RS              965428..965455
FT   5'D-NONAMER         965428..965436
FT   5'D-SPACER          965437..965448
FT   5'D-HEPTAMER        965449..965455
FT   D-REGION            965456..965486
FT                       /translation="VLRFLEWLLY"
FT                       /functional
FT                       /IMGT_allele="IGHD3-3*01"
FT                       /IMGT_gene="IGHD3-3"
FT   3'UTR               965487..966047
FT   3'D-RS              965487..965514
FT   3'D-HEPTAMER        965487..965493
FT   3'D-SPACER          965494..965505
FT   3'D-NONAMER         965506..965514
FT   D-GENE              966048..967096
FT                       /functional
FT                       /IMGT_allele="IGHD4-4*01"
FT                       /IMGT_gene="IGHD4-4"
FT   5'UTR               966048..966607
FT   D-GENE-UNIT         966580..966651
FT                       /functional
FT                       /IMGT_allele="IGHD4-4*01"
FT                       /IMGT_gene="IGHD4-4"
FT   5'D-RS              966580..966607
FT   5'D-NONAMER         966580..966588
FT   5'D-SPACER          966589..966600
FT   5'D-HEPTAMER        966601..966607
FT   D-REGION            966608..966623
FT                       /functional
FT                       /IMGT_allele="IGHD4-4*01"
FT                       /IMGT_gene="IGHD4-4"
FT   3'UTR               966624..967096
FT   3'D-RS              966624..966651
FT   3'D-HEPTAMER        966624..966630
FT   3'D-SPACER          966631..966642
FT   3'D-NONAMER         966643..966651
FT   D-GENE              967097..968503
FT                       /functional
FT                       /IMGT_allele="IGHD5-5*01"
FT                       /IMGT_gene="IGHD5-5"
FT   5'UTR               967097..967568
FT   D-GENE-UNIT         967541..967616
FT                       /functional
FT                       /IMGT_allele="IGHD5-5*01"
FT                       /IMGT_gene="IGHD5-5"
FT   5'D-RS              967541..967568
FT   5'D-NONAMER         967541..967549
FT   5'D-SPACER          967550..967561
FT   5'D-HEPTAMER        967562..967568
FT   D-REGION            967569..967588
FT                       /translation="VDTAMV"
FT                       /functional
FT                       /IMGT_allele="IGHD5-5*01"
FT                       /IMGT_gene="IGHD5-5"
FT   3'UTR               967589..968503
FT   3'D-RS              967589..967616
FT   3'D-HEPTAMER        967589..967595
FT   3'D-SPACER          967596..967607
FT   3'D-NONAMER         967608..967616
FT   D-GENE              968504..969678
FT                       /functional
FT                       /IMGT_allele="IGHD6-6*01"
FT                       /IMGT_gene="IGHD6-6"
FT   5'UTR               968504..969417
FT   D-GENE-UNIT         969390..969463
FT                       /functional
FT                       /IMGT_allele="IGHD6-6*01"
FT                       /IMGT_gene="IGHD6-6"
FT   5'D-RS              969390..969417
FT   5'D-NONAMER         969390..969398
FT   5'D-SPACER          969399..969410
FT   5'D-HEPTAMER        969411..969417
FT   D-REGION            969418..969435
FT                       /translation="EYSSSS"
FT                       /functional
FT                       /IMGT_allele="IGHD6-6*01"
FT                       /IMGT_gene="IGHD6-6"
FT   3'UTR               969436..969678
FT   3'D-RS              969436..969463
FT   3'D-HEPTAMER        969436..969442
FT   3'D-SPACER          969443..969454
FT   3'D-NONAMER         969455..969463
FT   D-GENE              969679..971271
FT                       /functional
FT                       /IMGT_allele="IGHD1-7*01"
FT                       /IMGT_gene="IGHD1-7"
FT   5'UTR               969679..969921
FT   D-GENE-UNIT         969894..969966
FT                       /functional
FT                       /IMGT_allele="IGHD1-7*01"
FT                       /IMGT_gene="IGHD1-7"
FT   5'D-RS              969894..969921
FT   5'D-NONAMER         969894..969902
FT   5'D-SPACER          969903..969914
FT   5'D-HEPTAMER        969915..969921
FT   D-REGION            969922..969938
FT                       /translation="GITGT"
FT                       /functional
FT                       /IMGT_allele="IGHD1-7*01"
FT                       /IMGT_gene="IGHD1-7"
FT   3'UTR               969939..971271
FT   3'D-RS              969939..969966
FT   3'D-HEPTAMER        969939..969945
FT   3'D-SPACER          969946..969957
FT   3'D-NONAMER         969958..969966
FT   D-GENE              971272..973884
FT                       /functional
FT                       /IMGT_allele="IGHD2-8*01"
FT                       /IMGT_gene="IGHD2-8"
FT   5'UTR               971272..972603
FT   D-GENE-UNIT         972576..972662
FT                       /functional
FT                       /IMGT_allele="IGHD2-8*01"
FT                       /IMGT_gene="IGHD2-8"
FT   5'D-RS              972576..972603
FT   5'D-NONAMER         972576..972584
FT   5'D-SPACER          972585..972596
FT   5'D-HEPTAMER        972597..972603
FT   D-REGION            972604..972634
FT                       /functional
FT                       /IMGT_allele="IGHD2-8*01"
FT                       /IMGT_gene="IGHD2-8"
FT   3'UTR               972635..973884
FT   3'D-RS              972635..972662
FT   3'D-HEPTAMER        972635..972641
FT   3'D-SPACER          972642..972653
FT   3'D-NONAMER         972654..972662
FT   D-GENE              973885..975241
FT                       /functional
FT                       /IMGT_allele="IGHD3-9*01"
FT                       /IMGT_gene="IGHD3-9"
FT   5'UTR               973885..975133
FT   D-GENE-UNIT         975106..975192
FT                       /functional
FT                       /IMGT_allele="IGHD3-9*01"
FT                       /IMGT_gene="IGHD3-9"
FT   5'D-RS              975106..975133
FT   5'D-NONAMER         975106..975114
FT   5'D-SPACER          975115..975126
FT   5'D-HEPTAMER        975127..975133
FT   D-REGION            975134..975164
FT                       /functional
FT                       /IMGT_allele="IGHD3-9*01"
FT                       /IMGT_gene="IGHD3-9"
FT   3'UTR               975165..975241
FT   3'D-RS              975165..975192
FT   3'D-HEPTAMER        975165..975171
FT   3'D-SPACER          975172..975183
FT   3'D-NONAMER         975184..975192
FT   D-GENE              975242..975781
FT                       /functional
FT                       /IMGT_allele="IGHD3-10*01"
FT                       /IMGT_gene="IGHD3-10"
FT   5'UTR               975242..975317
FT   D-GENE-UNIT         975290..975376
FT                       /functional
FT                       /IMGT_allele="IGHD3-10*01"
FT                       /IMGT_gene="IGHD3-10"
FT   5'D-RS              975290..975317
FT   5'D-NONAMER         975290..975298
FT   5'D-SPACER          975299..975310
FT   5'D-HEPTAMER        975311..975317
FT   D-REGION            975318..975348
FT                       /functional
FT                       /IMGT_allele="IGHD3-10*01"
FT                       /IMGT_gene="IGHD3-10"
FT   3'UTR               975349..975781
FT   3'D-RS              975349..975376
FT   3'D-HEPTAMER        975349..975355
FT   3'D-SPACER          975356..975367
FT   3'D-NONAMER         975368..975376
FT   D-GENE              975782..976701
FT                       /IMGT_allele="IGHD4-11*01"
FT                       /IMGT_gene="IGHD4-11"
FT                       /ORF="noncanonical 5'D-HEPTAMER: tgctgtg instead of 
FT                       cactgtg"
FT   5'UTR               975782..976213
FT   D-GENE-UNIT         976186..976257
FT                       /IMGT_allele="IGHD4-11*01"
FT                       /IMGT_gene="IGHD4-11"
FT                       /ORF="noncanonical 5'D-HEPTAMER: tgctgtg instead of 
FT                       cactgtg"
FT   5'D-RS              976186..976213
FT   5'D-NONAMER         976186..976194
FT   5'D-SPACER          976195..976206
FT   5'D-HEPTAMER        976207..976213
FT                       /IMGT_note="noncanonical 5'D-HEPTAMER: tgctgtg instead 
FT                       of cactgtg"
FT   D-REGION            976214..976229
FT                       /IMGT_allele="IGHD4-11*01"
FT                       /IMGT_gene="IGHD4-11"
FT                       /ORF="noncanonical 5'D-HEPTAMER: tgctgtg instead of 
FT                       cactgtg"
FT   3'UTR               976230..976701
FT   3'D-RS              976230..976257
FT   3'D-HEPTAMER        976230..976236
FT   3'D-SPACER          976237..976248
FT   3'D-NONAMER         976249..976257
FT   D-GENE              976702..977939
FT                       /functional
FT                       /IMGT_allele="IGHD5-12*01"
FT                       /IMGT_gene="IGHD5-12"
FT   5'UTR               976702..977173
FT   D-GENE-UNIT         977146..977224
FT                       /functional
FT                       /IMGT_allele="IGHD5-12*01"
FT                       /IMGT_gene="IGHD5-12"
FT   5'D-RS              977146..977173
FT   5'D-NONAMER         977146..977154
FT   5'D-SPACER          977155..977166
FT   5'D-HEPTAMER        977167..977173
FT   D-REGION            977174..977196
FT                       /translation="VDIVATI"
FT                       /functional
FT                       /IMGT_allele="IGHD5-12*01"
FT                       /IMGT_gene="IGHD5-12"
FT   3'UTR               977197..977939
FT   3'D-RS              977197..977224
FT   3'D-HEPTAMER        977197..977203
FT   3'D-SPACER          977204..977215
FT   3'D-NONAMER         977216..977224
FT   D-GENE              977940..978947
FT                       /functional
FT                       /IMGT_allele="IGHD6-13*01"
FT                       /IMGT_gene="IGHD6-13"
FT   5'UTR               977940..978682
FT   D-GENE-UNIT         978655..978731
FT                       /functional
FT                       /IMGT_allele="IGHD6-13*01"
FT                       /IMGT_gene="IGHD6-13"
FT   5'D-RS              978655..978682
FT   5'D-NONAMER         978655..978663
FT   5'D-SPACER          978664..978675
FT   5'D-HEPTAMER        978676..978682
FT   D-REGION            978683..978703
FT                       /translation="GYSSSWY"
FT                       /functional
FT                       /IMGT_allele="IGHD6-13*01"
FT                       /IMGT_gene="IGHD6-13"
FT   3'UTR               978704..978947
FT   3'D-RS              978704..978731
FT   3'D-HEPTAMER        978704..978710
FT   3'D-SPACER          978711..978722
FT   3'D-NONAMER         978723..978731
FT   D-GENE              978948..980532
FT                       /IMGT_allele="IGHD1-14*01"
FT                       /IMGT_gene="IGHD1-14"
FT                       /ORF="noncanonical 5'D-HEPTAMER: cacagcg instead of 
FT                       cactgtg, noncanonical 3'D-HEPTAMER: cactgtc instead of 
FT                       cacagtg"
FT   5'UTR               978948..979190
FT   D-GENE-UNIT         979163..979235
FT                       /IMGT_allele="IGHD1-14*01"
FT                       /IMGT_gene="IGHD1-14"
FT                       /ORF="noncanonical 5'D-HEPTAMER: cacagcg instead of 
FT                       cactgtg, noncanonical 3'D-HEPTAMER: cactgtc instead of 
FT                       cacagtg"
FT   5'D-RS              979163..979190
FT   5'D-NONAMER         979163..979171
FT   5'D-SPACER          979172..979183
FT   5'D-HEPTAMER        979184..979190
FT                       /IMGT_note="noncanonical 5'D-HEPTAMER: cacagcg instead 
FT                       of cactgtg"
FT   D-REGION            979191..979207
FT                       /translation="GITGT"
FT                       /IMGT_allele="IGHD1-14*01"
FT                       /IMGT_gene="IGHD1-14"
FT                       /ORF="noncanonical 5'D-HEPTAMER: cacagcg instead of 
FT                       cactgtg, noncanonical 3'D-HEPTAMER: cactgtc instead of 
FT                       cacagtg"
FT   3'UTR               979208..980532
FT   3'D-RS              979208..979235
FT   3'D-HEPTAMER        979208..979214
FT                       /IMGT_note="noncanonical 3'D-HEPTAMER: cactgtc instead 
FT                       of cacagtg"
FT   3'D-SPACER          979215..979226
FT   3'D-NONAMER         979227..979235
FT   D-GENE              980533..983031
FT                       /functional
FT                       /IMGT_allele="IGHD2-15*01"
FT                       /IMGT_gene="IGHD2-15"
FT   5'UTR               980533..981857
FT   D-GENE-UNIT         981830..981916
FT                       /functional
FT                       /IMGT_allele="IGHD2-15*01"
FT                       /IMGT_gene="IGHD2-15"
FT   5'D-RS              981830..981857
FT   5'D-NONAMER         981830..981838
FT   5'D-SPACER          981839..981850
FT   5'D-HEPTAMER        981851..981857
FT   D-REGION            981858..981888
FT                       /functional
FT                       /IMGT_allele="IGHD2-15*01"
FT                       /IMGT_gene="IGHD2-15"
FT   3'UTR               981889..983031
FT   3'D-RS              981889..981916
FT   3'D-HEPTAMER        981889..981895
FT   3'D-SPACER          981896..981907
FT   3'D-NONAMER         981908..981916
FT   D-GENE              983032..984766
FT                       /functional
FT                       /IMGT_allele="IGHD3-16*02"
FT                       /IMGT_gene="IGHD3-16"
FT   5'UTR               983032..984174
FT   D-GENE-UNIT         984147..984239
FT                       /functional
FT                       /IMGT_allele="IGHD3-16*02"
FT                       /IMGT_gene="IGHD3-16"
FT   5'D-RS              984147..984174
FT   5'D-NONAMER         984147..984155
FT   5'D-SPACER          984156..984167
FT   5'D-HEPTAMER        984168..984174
FT   D-REGION            984175..984211
FT                       /functional
FT                       /IMGT_allele="IGHD3-16*02"
FT                       /IMGT_gene="IGHD3-16"
FT   3'UTR               984212..984766
FT   3'D-RS              984212..984239
FT   3'D-HEPTAMER        984212..984218
FT   3'D-SPACER          984219..984230
FT   3'D-NONAMER         984231..984239
FT   D-GENE              984767..985810
FT                       /functional
FT                       /IMGT_allele="IGHD4-17*01"
FT                       /IMGT_gene="IGHD4-17"
FT   5'UTR               984767..985321
FT   D-GENE-UNIT         985294..985365
FT                       /functional
FT                       /IMGT_allele="IGHD4-17*01"
FT                       /IMGT_gene="IGHD4-17"
FT   5'D-RS              985294..985321
FT   5'D-NONAMER         985294..985302
FT   5'D-SPACER          985303..985314
FT   5'D-HEPTAMER        985315..985321
FT   D-REGION            985322..985337
FT                       /functional
FT                       /IMGT_allele="IGHD4-17*01"
FT                       /IMGT_gene="IGHD4-17"
FT   3'UTR               985338..985810
FT   3'D-RS              985338..985365
FT   3'D-HEPTAMER        985338..985344
FT   3'D-SPACER          985345..985356
FT   3'D-NONAMER         985357..985365
FT   D-GENE              985811..987214
FT                       /functional
FT                       /IMGT_allele="IGHD5-18*01"
FT                       /IMGT_gene="IGHD5-18"
FT   5'UTR               985811..986283
FT   D-GENE-UNIT         986256..986331
FT                       /functional
FT                       /IMGT_allele="IGHD5-18*01"
FT                       /IMGT_gene="IGHD5-18"
FT   5'D-RS              986256..986283
FT   5'D-NONAMER         986256..986264
FT   5'D-SPACER          986265..986276
FT   5'D-HEPTAMER        986277..986283
FT   D-REGION            986284..986303
FT                       /translation="VDTAMV"
FT                       /functional
FT                       /IMGT_allele="IGHD5-18*01"
FT                       /IMGT_gene="IGHD5-18"
FT   3'UTR               986304..987214
FT   3'D-RS              986304..986331
FT   3'D-HEPTAMER        986304..986310
FT   3'D-SPACER          986311..986322
FT   3'D-NONAMER         986323..986331
FT   D-GENE              987215..988392
FT                       /functional
FT                       /IMGT_allele="IGHD6-19*01"
FT                       /IMGT_gene="IGHD6-19"
FT   5'UTR               987215..988125
FT   D-GENE-UNIT         988098..988174
FT                       /functional
FT                       /IMGT_allele="IGHD6-19*01"
FT                       /IMGT_gene="IGHD6-19"
FT   5'D-RS              988098..988125
FT   5'D-NONAMER         988098..988106
FT   5'D-SPACER          988107..988118
FT   5'D-HEPTAMER        988119..988125
FT   D-REGION            988126..988146
FT                       /translation="GYSSGWY"
FT                       /functional
FT                       /IMGT_allele="IGHD6-19*01"
FT                       /IMGT_gene="IGHD6-19"
FT   3'UTR               988147..988392
FT   3'D-RS              988147..988174
FT   3'D-HEPTAMER        988147..988153
FT   3'D-SPACER          988154..988165
FT   3'D-NONAMER         988166..988174
FT   D-GENE              988393..989960
FT                       /functional
FT                       /IMGT_allele="IGHD1-20*01"
FT                       /IMGT_gene="IGHD1-20"
FT   5'UTR               988393..988637
FT   D-GENE-UNIT         988610..988682
FT                       /functional
FT                       /IMGT_allele="IGHD1-20*01"
FT                       /IMGT_gene="IGHD1-20"
FT   5'D-RS              988610..988637
FT   5'D-NONAMER         988610..988618
FT   5'D-SPACER          988619..988630
FT   5'D-HEPTAMER        988631..988637
FT   D-REGION            988638..988654
FT                       /translation="GITGT"
FT                       /functional
FT                       /IMGT_allele="IGHD1-20*01"
FT                       /IMGT_gene="IGHD1-20"
FT   3'UTR               988655..989960
FT   3'D-RS              988655..988682
FT   3'D-HEPTAMER        988655..988661
FT   3'D-SPACER          988662..988673
FT   3'D-NONAMER         988674..988682
FT   D-GENE              989961..992539
FT                       /functional
FT                       /IMGT_allele="IGHD2-21*02"
FT                       /IMGT_gene="IGHD2-21"
FT   5'UTR               989961..991266
FT   D-GENE-UNIT         991239..991322
FT                       /functional
FT                       /IMGT_allele="IGHD2-21*02"
FT                       /IMGT_gene="IGHD2-21"
FT   5'D-RS              991239..991266
FT   5'D-NONAMER         991239..991247
FT   5'D-SPACER          991248..991259
FT   5'D-HEPTAMER        991260..991266
FT   D-REGION            991267..991294
FT                       /functional
FT                       /IMGT_allele="IGHD2-21*02"
FT                       /IMGT_gene="IGHD2-21"
FT   3'UTR               991295..992539
FT   3'D-RS              991295..991322
FT   3'D-HEPTAMER        991295..991301
FT   3'D-SPACER          991302..991313
FT   3'D-NONAMER         991314..991322
FT   D-GENE              992540..994385
FT                       /functional
FT                       /IMGT_allele="IGHD3-22*01"
FT                       /IMGT_gene="IGHD3-22"
FT   5'UTR               992540..993783
FT   D-GENE-UNIT         993756..993842
FT                       /functional
FT                       /IMGT_allele="IGHD3-22*01"
FT                       /IMGT_gene="IGHD3-22"
FT   5'D-RS              993756..993783
FT   5'D-NONAMER         993756..993764
FT   5'D-SPACER          993765..993776
FT   5'D-HEPTAMER        993777..993783
FT   D-REGION            993784..993814
FT                       /functional
FT                       /IMGT_allele="IGHD3-22*01"
FT                       /IMGT_gene="IGHD3-22"
FT   3'UTR               993815..994385
FT   3'D-RS              993815..993842
FT   3'D-HEPTAMER        993815..993821
FT   3'D-SPACER          993822..993833
FT   3'D-NONAMER         993834..993842
FT   D-GENE              994386..995449
FT                       /IMGT_allele="IGHD4-23*01"
FT                       /IMGT_gene="IGHD4-23"
FT                       /ORF="noncanonical 5'D-HEPTAMER: tgctgtg instead of 
FT                       cactgtg"
FT   5'UTR               994386..994956
FT   D-GENE-UNIT         994929..995003
FT                       /IMGT_allele="IGHD4-23*01"
FT                       /IMGT_gene="IGHD4-23"
FT                       /ORF="noncanonical 5'D-HEPTAMER: tgctgtg instead of 
FT                       cactgtg"
FT   5'D-RS              994929..994956
FT   5'D-NONAMER         994929..994937
FT   5'D-SPACER          994938..994949
FT   5'D-HEPTAMER        994950..994956
FT                       /IMGT_note="noncanonical 5'D-HEPTAMER: tgctgtg instead 
FT                       of cactgtg"
FT   D-REGION            994957..994975
FT                       /IMGT_allele="IGHD4-23*01"
FT                       /IMGT_gene="IGHD4-23"
FT                       /ORF="noncanonical 5'D-HEPTAMER: tgctgtg instead of 
FT                       cactgtg"
FT   3'UTR               994976..995449
FT   3'D-RS              994976..995003
FT   3'D-HEPTAMER        994976..994982
FT   3'D-SPACER          994983..994994
FT   3'D-NONAMER         994995..995003
FT   D-GENE              995450..997115
FT                       /IMGT_allele="IGHD5-24*01"
FT                       /IMGT_gene="IGHD5-24"
FT                       /ORF="noncanonical 5'D-HEPTAMER: ggccgtg instead of 
FT                       cactgtg"
FT   5'UTR               995450..995922
FT   D-GENE-UNIT         995895..995970
FT                       /IMGT_allele="IGHD5-24*01"
FT                       /IMGT_gene="IGHD5-24"
FT                       /ORF="noncanonical 5'D-HEPTAMER: ggccgtg instead of 
FT                       cactgtg"
FT   5'D-RS              995895..995922
FT   5'D-NONAMER         995895..995903
FT   5'D-SPACER          995904..995915
FT   5'D-HEPTAMER        995916..995922
FT                       /IMGT_note="noncanonical 5'D-HEPTAMER: ggccgtg instead 
FT                       of cactgtg"
FT   D-REGION            995923..995942
FT                       /translation="VEMATI"
FT                       /IMGT_allele="IGHD5-24*01"
FT                       /IMGT_gene="IGHD5-24"
FT                       /ORF="noncanonical 5'D-HEPTAMER: ggccgtg instead of 
FT                       cactgtg"
FT   3'UTR               995943..997115
FT   3'D-RS              995943..995970
FT   3'D-HEPTAMER        995943..995949
FT   3'D-SPACER          995950..995961
FT   3'D-NONAMER         995962..995970
FT   D-GENE              997116..998549
FT                       /functional
FT                       /IMGT_allele="IGHD6-25*01"
FT                       /IMGT_gene="IGHD6-25"
FT   5'UTR               997116..998288
FT   D-GENE-UNIT         998261..998334
FT                       /functional
FT                       /IMGT_allele="IGHD6-25*01"
FT                       /IMGT_gene="IGHD6-25"
FT   5'D-RS              998261..998288
FT   5'D-NONAMER         998261..998269
FT   5'D-SPACER          998270..998281
FT   5'D-HEPTAMER        998282..998288
FT   D-REGION            998289..998306
FT                       /translation="GYSSGY"
FT                       /functional
FT                       /IMGT_allele="IGHD6-25*01"
FT                       /IMGT_gene="IGHD6-25"
FT   3'UTR               998307..998549
FT   3'D-RS              998307..998334
FT   3'D-HEPTAMER        998307..998313
FT   3'D-SPACER          998314..998325
FT   3'D-NONAMER         998326..998334
FT   D-GENE              998550..1006485
FT                       /functional
FT                       /IMGT_allele="IGHD1-26*01"
FT                       /IMGT_gene="IGHD1-26"
FT   5'UTR               998550..998791
FT   D-GENE-UNIT         998764..998839
FT                       /functional
FT                       /IMGT_allele="IGHD1-26*01"
FT                       /IMGT_gene="IGHD1-26"
FT   5'D-RS              998764..998791
FT   5'D-NONAMER         998764..998772
FT   5'D-SPACER          998773..998784
FT   5'D-HEPTAMER        998785..998791
FT   D-REGION            998792..998811
FT                       /translation="GIVGAT"
FT                       /functional
FT                       /IMGT_allele="IGHD1-26*01"
FT                       /IMGT_gene="IGHD1-26"
FT   3'UTR               998812..1006485
FT   3'D-RS              998812..998839
FT   3'D-HEPTAMER        998812..998818
FT   3'D-SPACER          998819..998830
FT   3'D-NONAMER         998831..998839
FT   J-GENE              1006486..1014238
FT                       /IMGT_allele="IGHJ1P*02"
FT                       /IMGT_gene="IGHJ1P"
FT                       /pseudo="no DONOR-SPLICE"
FT   5'UTR               1006486..1014167
FT   J-GENE-UNIT         1014131..1014221
FT                       /IMGT_allele="IGHJ1P*02"
FT                       /IMGT_gene="IGHJ1P"
FT                       /pseudo="no DONOR-SPLICE"
FT   J-RS                1014131..1014167
FT   J-NONAMER           1014131..1014139
FT                       /IMGT_note="noncanonical J-NONAMER: gtgttttgg instead 
FT                       of ggtttttgt"
FT   J-SPACER            1014140..1014160
FT   J-HEPTAMER          1014161..1014167
FT                       /IMGT_note="noncanonical J-HEPTAMER: cactggc instead 
FT                       of cactgtg"
FT   J-REGION            1014168..1014221
FT                       /translation="KGAGGPWTRPALETAATS"
FT                       /IMGT_allele="IGHJ1P*02"
FT                       /IMGT_gene="IGHJ1P"
FT                       /pseudo="no DONOR-SPLICE"
FT   5'J-REGION          1014168..1014188
FT                       /translation="KGAGGPW"
FT   J-MOTIF             1014186..1014197
FT                       /translation="WTRP"
FT                       /IMGT_note="noncanonical J-MOTIF: WTXP instead of WGXG"
FT   J-TRP               1014186..1014188
FT   3'UTR               1014222..1014238
FT   D-GENE              1014239..1014335
FT                       /functional
FT                       /IMGT_allele="IGHD7-27*01"
FT                       /IMGT_gene="IGHD7-27"
FT   5'UTR               1014239..1014283
FT   D-GENE-UNIT         1014256..1014322
FT                       /functional
FT                       /IMGT_allele="IGHD7-27*01"
FT                       /IMGT_gene="IGHD7-27"
FT   5'D-RS              1014256..1014283
FT   5'D-NONAMER         1014256..1014264
FT   5'D-SPACER          1014265..1014276
FT   5'D-HEPTAMER        1014277..1014283
FT   D-REGION            1014284..1014294
FT                       /translation="LTG"
FT                       /functional
FT                       /IMGT_allele="IGHD7-27*01"
FT                       /IMGT_gene="IGHD7-27"
FT   3'UTR               1014295..1014335
FT   3'D-RS              1014295..1014322
FT   3'D-HEPTAMER        1014295..1014301
FT   3'D-SPACER          1014302..1014313
FT   3'D-NONAMER         1014314..1014322
FT   J-GENE              1014336..1014497
FT                       /functional
FT                       /IMGT_allele="IGHJ1*01"
FT                       /IMGT_gene="IGHJ1"
FT   5'UTR               1014336..1014386
FT   J-GENE-UNIT         1014349..1014438
FT                       /functional
FT                       /IMGT_allele="IGHJ1*01"
FT                       /IMGT_gene="IGHJ1"
FT   J-RS                1014349..1014386
FT   J-NONAMER           1014349..1014357
FT   J-SPACER            1014358..1014379
FT   J-HEPTAMER          1014380..1014386
FT   J-REGION            1014387..1014438
FT                       /translation="AEYFQHWGQGTLVTVSS"
FT                       /functional
FT                       /IMGT_allele="IGHJ1*01"
FT                       /IMGT_gene="IGHJ1"
FT   5'J-REGION          1014387..1014407
FT                       /translation="AEYFQHW"
FT   J-MOTIF             1014405..1014416
FT                       /translation="WGQG"
FT   J-TRP               1014405..1014407
FT   DONOR-SPLICE        1014438..1014440
FT   3'UTR               1014439..1014497
FT   J-GENE              1014498..1014801
FT                       /IMGT_allele="IGHJ2*01"
FT                       /IMGT_gene="IGHJ2"
FT                       /ORF="noncanonical J-HEPTAMER: ggctgtg instead of 
FT                       cactgtg"
FT   5'UTR               1014498..1014593
FT   J-GENE-UNIT         1014556..1014646
FT                       /IMGT_allele="IGHJ2*01"
FT                       /IMGT_gene="IGHJ2"
FT                       /ORF="noncanonical J-HEPTAMER: ggctgtg instead of 
FT                       cactgtg"
FT   J-RS                1014556..1014593
FT   J-NONAMER           1014556..1014564
FT   J-SPACER            1014565..1014586
FT   J-HEPTAMER          1014587..1014593
FT                       /IMGT_note="noncanonical J-HEPTAMER: ggctgtg instead 
FT                       of cactgtg"
FT   J-REGION            1014594..1014646
FT                       /codon_start=2
FT                       /translation="YWYFDLWGRGTLVTVSS"
FT                       /IMGT_allele="IGHJ2*01"
FT                       /IMGT_gene="IGHJ2"
FT                       /ORF="noncanonical J-HEPTAMER: ggctgtg instead of 
FT                       cactgtg"
FT   5'J-REGION          1014594..1014615
FT                       /codon_start=2
FT                       /translation="YWYFDLW"
FT   J-MOTIF             1014613..1014624
FT                       /translation="WGRG"
FT   J-TRP               1014613..1014615
FT   DONOR-SPLICE        1014646..1014648
FT   3'UTR               1014647..1014801
FT   J-GENE              1014802..1015112
FT                       /IMGT_allele="IGHJ2P*02"
FT                       /IMGT_gene="IGHJ2P"
FT                       /pseudo="no DONOR-SPLICE"
FT   5'UTR               1014802..1014992
FT   J-GENE-UNIT         1014955..1015054
FT                       /IMGT_allele="IGHJ2P*02"
FT                       /IMGT_gene="IGHJ2P"
FT                       /pseudo="no DONOR-SPLICE"
FT   J-RS                1014955..1014992
FT   J-NONAMER           1014955..1014963
FT                       /IMGT_note="noncanonical J-NONAMER: tgttcatgt instead 
FT                       of ggtttttgt"
FT   J-SPACER            1014964..1014985
FT   J-HEPTAMER          1014986..1014992
FT                       /IMGT_note="noncanonical J-HEPTAMER: tagtgtg instead 
FT                       of cactgtg"
FT   J-REGION            1014993..1015054
FT                       /codon_start=3
FT                       /translation="YKCLEHWGQGSPATVSLGTS"
FT                       /IMGT_allele="IGHJ2P*02"
FT                       /IMGT_gene="IGHJ2P"
FT                       /pseudo="no DONOR-SPLICE"
FT   5'J-REGION          1014993..1015015
FT                       /codon_start=3
FT                       /translation="YKCLEHW"
FT   J-MOTIF             1015013..1015024
FT                       /translation="WGQG"
FT   J-TRP               1015013..1015015
FT   3'UTR               1015055..1015112
FT   J-GENE              1015113..1015401
FT                       /functional
FT                       /IMGT_allele="IGHJ3*02"
FT                       /IMGT_gene="IGHJ3"
FT   5'UTR               1015113..1015208
FT   J-GENE-UNIT         1015170..1015258
FT                       /functional
FT                       /IMGT_allele="IGHJ3*02"
FT                       /IMGT_gene="IGHJ3"
FT   J-RS                1015170..1015208
FT   J-NONAMER           1015170..1015178
FT   J-SPACER            1015179..1015201
FT   J-HEPTAMER          1015202..1015208
FT   J-REGION            1015209..1015258
FT                       /codon_start=2
FT                       /translation="DAFDIWGQGTMVTVSS"
FT                       /functional
FT                       /IMGT_allele="IGHJ3*02"
FT                       /IMGT_gene="IGHJ3"
FT   5'J-REGION          1015209..1015227
FT                       /codon_start=2
FT                       /translation="DAFDIW"
FT   J-MOTIF             1015225..1015236
FT                       /translation="WGQG"
FT   J-TRP               1015225..1015227
FT   DONOR-SPLICE        1015258..1015260
FT   3'UTR               1015259..1015401
FT   J-GENE              1015402..1015787
FT                       /functional
FT                       /IMGT_allele="IGHJ4*02"
FT                       /IMGT_gene="IGHJ4"
FT   5'UTR               1015402..1015582
FT   J-GENE-UNIT         1015544..1015630
FT                       /functional
FT                       /IMGT_allele="IGHJ4*02"
FT                       /IMGT_gene="IGHJ4"
FT   J-RS                1015544..1015582
FT   J-NONAMER           1015544..1015552
FT   J-SPACER            1015553..1015575
FT   J-HEPTAMER          1015576..1015582
FT   J-REGION            1015583..1015630
FT                       /codon_start=3
FT                       /translation="YFDYWGQGTLVTVSS"
FT                       /functional
FT                       /IMGT_allele="IGHJ4*02"
FT                       /IMGT_gene="IGHJ4"
FT   5'J-REGION          1015583..1015599
FT                       /codon_start=3
FT                       /translation="YFDYW"
FT   J-MOTIF             1015597..1015608
FT                       /translation="WGQG"
FT   J-TRP               1015597..1015599
FT   DONOR-SPLICE        1015630..1015632
FT   3'UTR               1015631..1015787
FT   J-GENE              1015788..1016187
FT                       /functional
FT                       /IMGT_allele="IGHJ5*02"
FT                       /IMGT_gene="IGHJ5"
FT   5'UTR               1015788..1015980
FT   J-GENE-UNIT         1015942..1016031
FT                       /functional
FT                       /IMGT_allele="IGHJ5*02"
FT                       /IMGT_gene="IGHJ5"
FT   J-RS                1015942..1015980
FT   J-NONAMER           1015942..1015950
FT   J-SPACER            1015951..1015973
FT   J-HEPTAMER          1015974..1015980
FT   J-REGION            1015981..1016031
FT                       /codon_start=3
FT                       /translation="NWFDPWGQGTLVTVSS"
FT                       /functional
FT                       /IMGT_allele="IGHJ5*02"
FT                       /IMGT_gene="IGHJ5"
FT   5'J-REGION          1015981..1016000
FT                       /codon_start=3
FT                       /translation="NWFDPW"
FT   J-MOTIF             1015998..1016009
FT                       /translation="WGQG"
FT   J-TRP               1015998..1016000
FT   DONOR-SPLICE        1016031..1016033
FT   3'UTR               1016032..1016187
FT   J-GENE              1016188..1016489
FT                       /IMGT_allele="IGHJ3P*02"
FT                       /IMGT_gene="IGHJ3P"
FT                       /pseudo="STOP-CODON in J-REGION"
FT   5'UTR               1016188..1016379
FT   J-GENE-UNIT         1016342..1016429
FT                       /IMGT_allele="IGHJ3P*02"
FT                       /IMGT_gene="IGHJ3P"
FT                       /pseudo="STOP-CODON in J-REGION"
FT   J-RS                1016342..1016379
FT   J-NONAMER           1016342..1016350
FT   J-SPACER            1016351..1016372
FT   J-HEPTAMER          1016373..1016379
FT                       /IMGT_note="noncanonical J-HEPTAMER: ccgtctg instead 
FT                       of cactgtg"
FT   J-REGION            1016380..1016429
FT                       /codon_start=2
FT                       /IMGT_allele="IGHJ3P*02"
FT                       /IMGT_gene="IGHJ3P"
FT                       /pseudo="STOP-CODON in J-REGION"
FT   5'J-REGION          1016380..1016398
FT                       /codon_start=2
FT                       /translation="LQLDFP"
FT   J-MOTIF             1016396..1016407
FT                       /translation="PGRQ"
FT                       /IMGT_note="no J-TRP: Pro instead of Trp, noncanonical 
FT                       J-MOTIF: PGXR instead of WGXG"
FT   DONOR-SPLICE        1016429..1016431
FT                       /IMGT_note="noncanonical DONOR-SPLICE: ngg instead of 
FT                       ngt"
FT   3'UTR               1016430..1016489
FT   J-GENE              1016490..1020878
FT                       /functional
FT                       /IMGT_allele="IGHJ6*03"
FT                       /IMGT_gene="IGHJ6"
FT   5'UTR               1016490..1016584
FT   J-GENE-UNIT         1016547..1016647
FT                       /functional
FT                       /IMGT_allele="IGHJ6*03"
FT                       /IMGT_gene="IGHJ6"
FT   J-RS                1016547..1016584
FT   J-NONAMER           1016547..1016555
FT   J-SPACER            1016556..1016577
FT   J-HEPTAMER          1016578..1016584
FT   J-REGION            1016585..1016647
FT                       /codon_start=3
FT                       /translation="YYYYYYMDVWGKGTTVTVSS"
FT                       /functional
FT                       /IMGT_allele="IGHJ6*03"
FT                       /IMGT_gene="IGHJ6"
FT   5'J-REGION          1016585..1016616
FT                       /codon_start=3
FT                       /translation="YYYYYYMDVW"
FT   J-MOTIF             1016614..1016625
FT                       /translation="WGKG"
FT   J-TRP               1016614..1016616
FT   DONOR-SPLICE        1016647..1016649
FT   3'UTR               1016648..1020878
FT   C-GENE              1020879..1031000
FT                       /functional
FT                       /IMGT_allele="IGHM*04"
FT                       /IMGT_gene="IGHM"
FT   5'UTR               1020879..1023627
FT   ACCEPTOR-SPLICE     1023625..1023629
FT   C-GENE-UNIT         1023628..1027879
FT                       /functional
FT                       /IMGT_allele="IGHM*04"
FT                       /IMGT_gene="IGHM"
FT   CH1                 1023628..1023939
FT                       /codon_start=3
FT                       /translation="SASAPTLFPLVSCENSPSDTSSVAVGCLAQDFLPDSITFSW
FT                       KYKNNSDISSTRGFPSVLRGGKYAATSQVLLPSKDVMQGTDEHVVCKVQHPNGNK
FT                       EKNVPLP"
FT   DONOR-SPLICE        1023939..1023941
FT   INTRON              1023940..1024029
FT   ACCEPTOR-SPLICE     1024027..1024031
FT   CH2                 1024030..1024365
FT                       /codon_start=3
FT                       /translation="IAELPPKVSVFVPPRDGFFGNPRKSKLICQATGFSPRQIQV
FT                       SWLREGKQVGSGVTTDQVQAEAKESGPTTYKVTSTLTIKESDWLGQSMFTCRVDH
FT                       RGLTFQQNASSMCVP"
FT                       /splice-expected-nt="g"
FT                       /splice-expectedcodon="gtg[V]"
FT                       /splicing-frame="sf1"
FT   DONOR-SPLICE        1024365..1024367
FT   INTRON              1024366..1024610
FT   ACCEPTOR-SPLICE     1024608..1024612
FT   CH3                 1024611..1024928
FT                       /codon_start=3
FT                       /translation="QDTAIRVFAIPPSFASIFLTKSTKLTCLVTDLTTYDSVTIS
FT                       WTRQNGEAVKTHTNISESHPNATFSAVGEASICEDDWNSGERFTCTVTHTDLPSP
FT                       LKQTISRPK"
FT                       /splice-expected-nt="g"
FT                       /splice-expectedcodon="gat[D]"
FT                       /splicing-frame="sf1"
FT   DONOR-SPLICE        1024928..1024930
FT   INTRON              1024929..1025107
FT   ACCEPTOR-SPLICE     1025105..1025109
FT   CH4-CHS             1025108..1025499
FT                       /codon_start=3
FT                       /translation="VALHRPDVYLLPPAREQLNLRESATITCLVTGFSPADVFVQ
FT                       WMQRGQPLSPEKYVTSAPMPEPQAPGRYFAHSILTVSEEEWNTGETYTCVVAHEA
FT                       LPNRVTERTVDKSTGKPTLYNVSLVMSDTAGTCY"
FT                       /splice-expected-nt="g"
FT                       /splice-expectedcodon="ggg[G]"
FT                       /splicing-frame="sf1"
FT   CH4                 1025108..1025439
FT                       /codon_start=3
FT                       /translation="VALHRPDVYLLPPAREQLNLRESATITCLVTGFSPADVFVQ
FT                       WMQRGQPLSPEKYVTSAPMPEPQAPGRYFAHSILTVSEEEWNTGETYTCVVAHEA
FT                       LPNRVTERTVDKST"
FT   CHS                 1025440..1025499
FT                       /translation="GKPTLYNVSLVMSDTAGTCY"
FT                       /IMGT_note="for complete translation nucleotide g at 
FT                       position 1025440 has been included in CHS"
FT   INT-DONOR-SPLICE    1025440..1025442
FT   INTRON              1025500..1027581
FT   STOP-CODON          1025500..1025502
FT   POLYA_SIGNAL        1025605..1025610
FT   ACCEPTOR-SPLICE     1027579..1027583
FT   M1                  1027582..1027697
FT                       /codon_start=3
FT                       /translation="GEVSADEEGFENLWATASTFIVLFLLSLFYSTTVTLFK"
FT                       /splice-expected-nt="g"
FT                       /splice-expectedcodon="gag[E]"
FT                       /splicing-frame="sf1"
FT   CONNECTING-REGION   1027582..1027619
FT   3'UTR               1027602..1031000
FT   TM-PART1            1027620..1027697
FT                       /translation="LWATASTFIVLFLLSLFYSTTVTLFK"
FT   DONOR-SPLICE        1027697..1027699
FT   INTRON              1027698..1027870
FT   ACCEPTOR-SPLICE     1027868..1027872
FT   M2                  1027871..1027876
FT                       /translation="VK"
FT                       /splicing-frame="sf0"
FT   TM-PART2            1027871..1027873
FT   CYTOPLASMIC-REGION  1027874..1027876
FT   STOP-CODON          1027877..1027879
FT   POLYA_SIGNAL        1028113..1028118
FT   C-GENE              1031001..1075000
FT                       /functional
FT                       /IMGT_allele="IGHD*02"
FT                       /IMGT_gene="IGHD"
FT   5'UTR               1031001..1034167
FT   ACCEPTOR-SPLICE     1034165..1034169
FT   C-GENE-UNIT         1034168..1042694
FT                       /functional
FT                       /IMGT_allele="IGHD*02"
FT                       /IMGT_gene="IGHD"
FT   CH1                 1034168..1034470
FT                       /codon_start=3
FT                       /translation="PTKAPDVFPIISGCRHPKDNSPVVLACLITGYHPTSVTVTW
FT                       YMGTQSQPQRTFPEIQRRDSYYMTSSQLSTPLQQWRQGEYKCVVQHTASKSKKEI
FT                       FRWP"
FT   DONOR-SPLICE        1034470..1034472
FT   INTRON              1034471..1034918
FT   ACCEPTOR-SPLICE     1034916..1034920
FT   H1                  1034919..1035020
FT                       /codon_start=3
FT                       /translation="SPKAQASSVPTAQPQAEGSLAKATTAPATTRNT"
FT                       /splice-expected-nt="g"
FT                       /splice-expectedcodon="gag[E]"
FT                       /splicing-frame="sf1"
FT   DONOR-SPLICE        1035020..1035022
FT   INTRON              1035021..1037812
FT   ACCEPTOR-SPLICE     1037810..1037814
FT   H2                  1037813..1037884
FT                       /codon_start=3
FT                       /translation="RGGEEKKKEKEKEEQEERETKTP"
FT                       /splice-expected-nt="g"
FT                       /splice-expectedcodon="gga[G]"
FT                       /splicing-frame="sf1"
FT   DONOR-SPLICE        1037884..1037886
FT   INTRON              1037885..1038620
FT   ACCEPTOR-SPLICE     1038618..1038622
FT   CH2                 1038621..1038944
FT                       /codon_start=3
FT                       /translation="CPSHTQPLGVYLLTPAVQDLWLRDKATFTCFVVGSDLKDAH
FT                       LTWEVAGKVPTGGVEEGLLERHSNGSQSQHSRLTLPRSLWNAGTSVTCTLNHPSL
FT                       PPQRLMALREP"
FT                       /splice-expected-nt="g"
FT                       /splice-expectedcodon="gag[E]"
FT                       /splicing-frame="sf1"
FT   DONOR-SPLICE        1038944..1038946
FT   INTRON              1038945..1039154
FT   ACCEPTOR-SPLICE     1039152..1039156
FT   CH3                 1039155..1039478
FT                       /codon_start=3
FT                       /translation="AQAPVKLSLNLLASSDPPEAASWLLCEVSGFSPPNILLMWL
FT                       EDQREVNTSGFAPARPPPQPRSTTFWAWSVLRVPAPPSPQPATYTCVVSHEDSRT
FT                       LLNASRSLEVS"
FT                       /splice-expected-nt="g"
FT                       /splice-expectedcodon="gct[A]"
FT                       /splicing-frame="sf1"
FT   DONOR-SPLICE        1039478..1039480
FT   INTRON              1039479..1041415
FT   ACCEPTOR-SPLICE     1041413..1041417
FT   CHS                 1041416..1041441
FT                       /splice-expected-nt="t"
FT                       /splice-expectedcodon="tat[Y]"
FT                       /splicing-frame="sf1"
FT   INTRON              1041442..1042243
FT   STOP-CODON          1041442..1041444
FT   POLYA_SIGNAL        1041509..1041514
FT   ACCEPTOR-SPLICE     1042241..1042245
FT   M1                  1042244..1042401
FT                       /codon_start=3
FT                       /translation="LAMTPLIPQSKDENSDDYTTFDDVGSLWTTLSTFVALFILT
FT                       LLYSGIVTFIK"
FT                       /splice-expected-nt="t"
FT                       /splice-expectedcodon="tac[Y]"
FT                       /splicing-frame="sf1"
FT   CONNECTING-REGION   1042244..1042323
FT   TM-PART1            1042324..1042401
FT                       /translation="LWTTLSTFVALFILTLLYSGIVTFIK"
FT   DONOR-SPLICE        1042401..1042403
FT   INTRON              1042402..1042685
FT   ACCEPTOR-SPLICE     1042683..1042687
FT   M2                  1042686..1042691
FT                       /translation="VK"
FT                       /splicing-frame="sf0"
FT   TM-PART2            1042686..1042688
FT   CYTOPLASMIC-REGION  1042689..1042691
FT   3'UTR               1042692..1075000
FT   STOP-CODON          1042692..1042694
FT   POLYA_SIGNAL        1043359..1043364
FT   C-GENE              1075001..1125000
FT                       /functional
FT                       /IMGT_allele="IGHG3*10"
FT                       /IMGT_gene="IGHG3"
FT   5'UTR               1075001..1108439
FT   ACCEPTOR-SPLICE     1108437..1108441
FT   C-GENE-UNIT         1108440..1113931
FT                       /functional
FT                       /IMGT_allele="IGHG3*10"
FT                       /IMGT_gene="IGHG3"
FT   CH1                 1108440..1108733
FT                       /codon_start=3
FT                       /translation="STKGPSVFPLAPCSRSTSGGTAALGCLVKDYFPEPVTVSWN
FT                       SGALTSGVHTFPAVLQSSGLYSLSSVVTVPSSSLGTQTYTCNVNHKPSNTKVDKR
FT                       V"
FT   DONOR-SPLICE        1108733..1108735
FT   INTRON              1108734..1109125
FT   ACCEPTOR-SPLICE     1109123..1109127
FT   H1                  1109126..1109176
FT                       /codon_start=3
FT                       /translation="LKTPLGDTTHTCPRCP"
FT                       /splice-expected-nt="g"
FT                       /splice-expectedcodon="gag[E]"
FT                       /splicing-frame="sf1"
FT   DONOR-SPLICE        1109176..1109178
FT   INTRON              1109177..1109319
FT   ACCEPTOR-SPLICE     1109317..1109321
FT   H2                  1109320..1109364
FT                       /codon_start=3
FT                       /translation="PKSCDTPPPCPRCP"
FT                       /splice-expected-nt="g"
FT                       /splice-expectedcodon="gag[E]"
FT                       /splicing-frame="sf1"
FT   DONOR-SPLICE        1109364..1109366
FT   INTRON              1109365..1109507
FT   ACCEPTOR-SPLICE     1109505..1109509
FT   H3                  1109508..1109552
FT                       /codon_start=3
FT                       /translation="PKSCDTPPPCPRCP"
FT                       /splice-expected-nt="g"
FT                       /splice-expectedcodon="gag[E]"
FT                       /splicing-frame="sf1"
FT   DONOR-SPLICE        1109552..1109554
FT   INTRON              1109553..1109695
FT   ACCEPTOR-SPLICE     1109693..1109697
FT   H4                  1109696..1109740
FT                       /codon_start=3
FT                       /translation="PKSCDTPPPCPRCP"
FT                       /splice-expected-nt="g"
FT                       /splice-expectedcodon="gag[E]"
FT                       /splicing-frame="sf1"
FT   DONOR-SPLICE        1109740..1109742
FT   INTRON              1109741..1109858
FT   ACCEPTOR-SPLICE     1109856..1109860
FT   CH2                 1109859..1110188
FT                       /codon_start=3
FT                       /translation="PELLGGPSVFLFPPKPKDTLMISRTPEVTCVVVDVSHEDPE
FT                       VQFKWYVDGVEVHNAKTKPREEQYNSTFRVVSVLTVLHQDWLNGKEYKCKVSNKA
FT                       LPAPIEKTISKTK"
FT                       /splice-expected-nt="g"
FT                       /splice-expectedcodon="gca[A]"
FT                       /splicing-frame="sf1"
FT   DONOR-SPLICE        1110188..1110190
FT   INTRON              1110189..1110285
FT   ACCEPTOR-SPLICE     1110283..1110287
FT   CH3-CHS             1110286..1110605
FT                       /codon_start=3
FT                       /translation="QPREPQVYTLPPSREEMTKNQVSLTCLVKGFYPSDIAVEWE
FT                       SSGQPENNYNTTPPMLDSDGSFFLYSKLTVDKSRWQQGNIFSCSVMHEALHNRFT
FT                       QKSLSLSPGK"
FT                       /splice-expected-nt="g"
FT                       /splice-expectedcodon="gga[G]"
FT                       /splicing-frame="sf1"
FT   CH3                 1110286..1110599
FT                       /codon_start=3
FT                       /translation="QPREPQVYTLPPSREEMTKNQVSLTCLVKGFYPSDIAVEWE
FT                       SSGQPENNYNTTPPMLDSDGSFFLYSKLTVDKSRWQQGNIFSCSVMHEALHNRFT
FT                       QKSLSLSP"
FT   CHS                 1110600..1110605
FT                       /translation="GK"
FT                       /IMGT_note="for complete translation nucleotide g at 
FT                       position 1110600 has been included in CHS"
FT   INT-DONOR-SPLICE    1110600..1110602
FT   INTRON              1110606..1111901
FT   STOP-CODON          1110606..1110608
FT   POLYA_SIGNAL        1110709..1110714
FT   ACCEPTOR-SPLICE     1111899..1111903
FT   M1                  1111902..1112032
FT                       /codon_start=3
FT                       /translation="LQLEESCAEAQDGELDGLWTTITIFITLFLLSVCYSATVTF
FT                       FK"
FT                       /splice-expected-nt="g"
FT                       /splice-expectedcodon="gag[E]"
FT                       /splicing-frame="sf1"
FT   CONNECTING-REGION   1111902..1111954
FT                       /translation="SCNWRRAVRRRRTGSWT"
FT   TM-PART1            1111955..1112032
FT                       /translation="LWTTITIFITLFLLSVCYSATVTFFK"
FT   DONOR-SPLICE        1112032..1112034
FT   INTRON              1112033..1113847
FT   ACCEPTOR-SPLICE     1113845..1113849
FT   M2                  1113848..1113928
FT                       /translation="VKWIFSSVVDLKQTIIPDYRNMIGQGA"
FT                       /splicing-frame="sf0"
FT   TM-PART2            1113848..1113850
FT   CYTOPLASMIC-REGION  1113851..1113928
FT                       /translation="KWIFSSVVDLKQTIIPDYRNMIGQGA"
FT   3'UTR               1113929..1125000
FT   STOP-CODON          1113929..1113931
FT   POLYA_SIGNAL        1115316..1115321
FT   C-GENE              1125001..1150000
FT                       /functional
FT                       /IMGT_allele="IGHG1*05"
FT                       /IMGT_gene="IGHG1"
FT   5'UTR               1125001..1136774
FT   ACCEPTOR-SPLICE     1136772..1136776
FT   C-GENE-UNIT         1136775..1142083
FT                       /functional
FT                       /IMGT_allele="IGHG1*05"
FT                       /IMGT_gene="IGHG1"
FT   CH1                 1136775..1137068
FT                       /codon_start=3
FT                       /translation="STKGPSVFPLAPSSKSTSGGTAALGCLVKDYFPEPVTVSWN
FT                       SGALTSGVHTFPAVLQSSGLYSLSSVVTVPSSSLGTQTYICNVNHKPSNTKVDKK
FT                       V"
FT   DONOR-SPLICE        1137068..1137070
FT   INTRON              1137069..1137459
FT   ACCEPTOR-SPLICE     1137457..1137461
FT   H                   1137460..1137504
FT                       /codon_start=3
FT                       /translation="PKSCDKTHTCPPCP"
FT                       /splice-expected-nt="g"
FT                       /splice-expectedcodon="gag[E]"
FT                       /splicing-frame="sf1"
FT   DONOR-SPLICE        1137504..1137506
FT   INTRON              1137505..1137622
FT   ACCEPTOR-SPLICE     1137620..1137624
FT   CH2                 1137623..1137952
FT                       /codon_start=3
FT                       /translation="PELLGGPSVFLFPPKPKDTLMISRTPEVTCVVVDVSHEDPE
FT                       VKFNWYVDGVEVHNAKTKPREEQYNSTYRVVSVLTVLHQDWLNGKEYKCKVSNKA
FT                       LPAPIEKTISKAK"
FT                       /splice-expected-nt="g"
FT                       /splice-expectedcodon="gca[A]"
FT                       /splicing-frame="sf1"
FT   DONOR-SPLICE        1137952..1137954
FT   INTRON              1137953..1138049
FT   ACCEPTOR-SPLICE     1138047..1138051
FT   CH3-CHS             1138050..1138369
FT                       /codon_start=3
FT                       /translation="QPREPQVYTLPPSRDELTKNQVSLTCLVKGFYPSDIAVEWE
FT                       SNGQPENNYKTTPPVLDSDGSFFLYSKLTVDKSRWQQGNVFSCSVMHEALHNHYT
FT                       QKSLSLSPGK"
FT                       /splice-expected-nt="g"
FT                       /splice-expectedcodon="ggg[G]"
FT                       /splicing-frame="sf1"
FT   CH3                 1138050..1138363
FT                       /codon_start=3
FT                       /translation="QPREPQVYTLPPSRDELTKNQVSLTCLVKGFYPSDIAVEWE
FT                       SNGQPENNYKTTPPVLDSDGSFFLYSKLTVDKSRWQQGNVFSCSVMHEALHNHYT
FT                       QKSLSLSP"
FT   CHS                 1138364..1138369
FT                       /translation="GK"
FT                       /IMGT_note="for complete translation nucleotide g at 
FT                       position 1138364 has been included in CHS"
FT   INT-DONOR-SPLICE    1138364..1138366
FT   INTRON              1138370..1139651
FT   STOP-CODON          1138370..1138372
FT   POLYA_SIGNAL        1138473..1138478
FT   ACCEPTOR-SPLICE     1139649..1139651
FT   M1                  1139652..1139782
FT                       /codon_start=3
FT                       /translation="LQLEESCAEAQDGELDGLWTTITIFITLFLLSVCYSATVTF
FT                       FK"
FT                       /splice-expected-nt="g"
FT                       /splice-expectedcodon="gag[E]"
FT                       /splicing-frame="sf1"
FT   CONNECTING-REGION   1139652..1139704
FT                       /translation="SCNWRRAVRRRRTGSWT"
FT   TM-PART1            1139705..1139782
FT                       /translation="LWTTITIFITLFLLSVCYSATVTFFK"
FT   DONOR-SPLICE        1139782..1139784
FT   INTRON              1139783..1141999
FT   ACCEPTOR-SPLICE     1141997..1142001
FT   M2                  1142000..1142080
FT                       /translation="VKWIFSSVVDLKQTIIPDYRNMIGQGA"
FT                       /splicing-frame="sf0"
FT   TM-PART2            1142000..1142002
FT   CYTOPLASMIC-REGION  1142003..1142080
FT                       /translation="KWIFSSVVDLKQTIIPDYRNMIGQGA"
FT   3'UTR               1142081..1150000
FT   STOP-CODON          1142081..1142083
FT   POLYA_SIGNAL        1143476..1143481
FT   C-GENE              1150001..1165500
FT                       /IMGT_allele="IGHEP1*04"
FT                       /IMGT_gene="IGHEP1"
FT                       /pseudo="no CH1, no CH2"
FT   5'UTR               1150001..1157312
FT   ACCEPTOR-SPLICE     1157310..1157314
FT   CH3                 1157313..1157636
FT                       /codon_start=3
FT                       /translation="FNPRGVSAYLSRPSPFDLFIRKSPTITCLVVDLAPSKWTVN
FT                       LTWSRASGKPVNHSTRKEEKQRNGTLTVTSTLPVGTRDWIEGETYQCRVTHPHLP
FT                       RALVRSTTKTS"
FT                       /IMGT_note="noncanonical ACCEPTOR-SPLICE: ntgnn 
FT                       instead of nagnn"
FT   DONOR-SPLICE        1157636..1157638
FT   INTRON              1157637..1157719
FT   ACCEPTOR-SPLICE     1157717..1157721
FT   CH4-CHS             1157720..1158048
FT                       /codon_start=3
FT                       /translation="PRAAPEVYAFATPEWLGSRDKRTLTCLIQNFMPEDISVQWL
FT                       HNEVQLPDARHSTTQPRKTKGSGFFIFSRLEVTRAEWEQKDEFICRAVHEAASPS
FT                       QTVQRAVSVNPGK"
FT                       /splice-expected-nt="g"
FT                       /splice-expectedcodon="ggc[G]"
FT                       /splicing-frame="sf1"
FT   CH4                 1157720..1158042
FT                       /codon_start=3
FT                       /translation="PRAAPEVYAFATPEWLGSRDKRTLTCLIQNFMPEDISVQWL
FT                       HNEVQLPDARHSTTQPRKTKGSGFFIFSRLEVTRAEWEQKDEFICRAVHEAASPS
FT                       QTVQRAVSVNP"
FT   CHS                 1158043..1158048
FT                       /translation="GK"
FT                       /IMGT_note="for complete translation nucleotide g at 
FT                       position 1158043 has been included in CHS"
FT   INT-DONOR-SPLICE    1158043..1158045
FT   INTRON              1158049..1160133
FT   STOP-CODON          1158049..1158051
FT   POLYA_SIGNAL        1158157..1158162
FT   ACCEPTOR-SPLICE     1160131..1160135
FT   M1                  1160134..1160255
FT                       /codon_start=3
FT                       /translation="LDVCVEEAEGEAPWTWTGLCIFAALFLLSVSYSAAITLLM"
FT                       /splice-expected-nt="g"
FT                       /splice-expectedcodon="gag[E]"
FT                       /splicing-frame="sf1"
FT   CONNECTING-REGION   1160134..1160177
FT                       /translation="SWTCAWRRPRARRR"
FT   TM-PART1            1160178..1160255
FT                       /translation="TWTGLCIFAALFLLSVSYSAAITLLM"
FT   DONOR-SPLICE        1160255..1160257
FT   INTRON              1160256..1160339
FT   ACCEPTOR-SPLICE     1160337..1160341
FT   M2                  1160340..1160420
FT                       /translation="VQRFLSATRQGRPQTSLDYTNVLQPHA"
FT                       /splicing-frame="sf0"
FT   TM-PART2            1160340..1160342
FT   CYTOPLASMIC-REGION  1160343..1160420
FT                       /translation="QRFLSATRQGRPQTSLDYTNVLQPHA"
FT   3'UTR               1160421..1165500
FT   STOP-CODON          1160421..1160423
FT   C-GENE              1165501..1193000
FT                       /functional
FT                       /IMGT_allele="IGHA1*03"
FT                       /IMGT_gene="IGHA1"
FT   5'UTR               1165501..1171180
FT   ACCEPTOR-SPLICE     1171178..1171182
FT   C-GENE-UNIT         1171181..1175457
FT                       /functional
FT                       /IMGT_allele="IGHA1*03"
FT                       /IMGT_gene="IGHA1"
FT   CH1                 1171181..1171486
FT                       /codon_start=3
FT                       /translation="SPTSPKVFPLSLCSTQPDGNVVIACLVQGFFPQEPLSVTWS
FT                       ESGQGVTARNFPPSQDASGDLYTTSSQLTLPATQCLAGKSVTCHVKHYTNPSQDV
FT                       TVPCP"
FT   DONOR-SPLICE        1171486..1171488
FT   INTRON              1171487..1171700
FT   ACCEPTOR-SPLICE     1171698..1171702
FT   H-CH2               1171701..1172060
FT                       /codon_start=3
FT                       /translation="PSTPPTPSPSTPPTPSPSCCHPRLSLHRPALEDLLLGSEAN
FT                       LTCTLTGLRDASGVTFTWTPSSGKSAVQGPPERDLCGCYSVSSVLPGCAEPWNHG
FT                       KTFTCTAAYPESKTPLTATLSKS"
FT                       /splice-expected-nt="g"
FT                       /splice-expectedcodon="gtt[V]"
FT                       /splicing-frame="sf1"
FT   H                   1171701..1171756
FT                       /codon_start=3
FT                       /translation="PSTPPTPSPSTPPTPSPS"
FT   CH2                 1171757..1172060
FT                       /codon_start=3
FT   DONOR-SPLICE        1172060..1172062
FT   INTRON              1172061..1172282
FT   ACCEPTOR-SPLICE     1172280..1172284
FT   CH3-CHS             1172283..1172674
FT                       /codon_start=3
FT                       /translation="NTFRPEVHLLPPPSEELALNELVTLTCLARGFSPKDVLVRW
FT                       LQGSQELPREKYLTWASRQEPSQGTTTFAVTSILRVAAEDWKKGDTFSCMVGHEA
FT                       LPLAFTQKTIDRLAGKPTHVNVSVVMAEVDGTCY"
FT                       /splice-expected-nt="g"
FT                       /splice-expectedcodon="gga[G]"
FT                       /splicing-frame="sf1"
FT   CH3                 1172283..1172614
FT                       /codon_start=3
FT                       /translation="NTFRPEVHLLPPPSEELALNELVTLTCLARGFSPKDVLVRW
FT                       LQGSQELPREKYLTWASRQEPSQGTTTFAVTSILRVAAEDWKKGDTFSCMVGHEA
FT                       LPLAFTQKTIDRLA"
FT   CHS                 1172615..1172674
FT                       /translation="GKPTHVNVSVVMAEVDGTCY"
FT                       /IMGT_note="for complete translation nucleotide g at 
FT                       position 1172615 has been included in CHS"
FT   INT-DONOR-SPLICE    1172615..1172617
FT   INTRON              1172675..1175242
FT   STOP-CODON          1172675..1172677
FT   POLYA_SIGNAL        1172702..1172707
FT   ACCEPTOR-SPLICE     1175240..1175244
FT   M                   1175243..1175454
FT                       /codon_start=3
FT                       /translation="SCCVADWQMPPPYVVLDLPQETLEEETPGANLWPTTITFLT
FT                       LFLLSLFYSTALTVTSVRGPSGNREGPQY"
FT                       /splice-expected-nt="g"
FT                       /splice-expectedcodon="ggc[G]"
FT                       /splicing-frame="sf1"
FT   CONNECTING-REGION   1175244..1175337
FT                       /translation="LLLCCRLADAASLCGAGLAAGDPGGGDPRRQ"
FT   TRANSMEMBRANE-REGION1175338..1175418
FT                       /translation="LWPTTITFLTLFLLSLFYSTALTVTSV"
FT   CYTOPLASMIC-REGION  1175419..1175454
FT                       /translation="RGPSGNREGPQY"
FT   3'UTR               1175455..1193000
FT   STOP-CODON          1175455..1175457
FT   POLYA_SIGNAL        1175863..1175868
FT   C-GENE              1193001..1225000
FT                       /IMGT_allele="IGHGP*03"
FT                       /IMGT_gene="IGHGP"
FT                       /pseudo="frameshift in CH3: insertion of 1 nt"
FT   5'UTR               1193001..1210000
FT   ACCEPTOR-SPLICE     1209998..1210002
FT   C-GENE-UNIT         1210001..1215219
FT                       /IMGT_allele="IGHGP*03"
FT                       /IMGT_gene="IGHGP"
FT                       /pseudo="frameshift in CH3: insertion of 1 nt"
FT   CH1                 1210001..1210294
FT                       /codon_start=3
FT                       /translation="STKGPSVFPLVPSSRSVSEGTAALGCLVKDYFPEPVTVSWN
FT                       SGALTRSVHTFPAVLQSSGLYSLSSVVTVPSSSLGTQTYTCNVDHKPSNTKVDKT
FT                       V"
FT   DONOR-SPLICE        1210294..1210296
FT   INTRON              1210295..1210683
FT   ACCEPTOR-SPLICE     1210681..1210685
FT   H                   1210684..1210734
FT                       /codon_start=3
FT                       /translation="PKTPCCDTTHTCPPCA"
FT                       /splice-expected-nt="g"
FT                       /splice-expectedcodon="gag[E]"
FT                       /splicing-frame="sf1"
FT   DONOR-SPLICE        1210734..1210736
FT   INTRON              1210735..1210852
FT   ACCEPTOR-SPLICE     1210850..1210854
FT   CH2                 1210853..1211182
FT                       /codon_start=3
FT                       /translation="TEPLGGPSVFLFPPKPKDTLMISRTPEVTCVVVDVSHEDPE
FT                       VKFNWYVDGVEVHNAKTKPWEEQYNSTYHVVSVLTVVHQNWLNGKEYKCKVSNKG
FT                       LPAPIEKTISKTK"
FT                       /splice-expected-nt="a"
FT                       /splice-expectedcodon="aca[T]"
FT                       /splicing-frame="sf1"
FT   DONOR-SPLICE        1211182..1211184
FT   INTRON              1211183..1211279
FT   ACCEPTOR-SPLICE     1211277..1211281
FT   CH3-CHS             1211280..1211597
FT                       /codon_start=3
FT                       /splice-expected-nt="g"
FT                       /splice-expectedcodon="ggg[G]"
FT                       /splicing-frame="sf1"
FT   CH3                 1211280..1211591
FT                       /codon_start=3
FT                       /translation="QPREPQVYTLPPSQKMTKNQVTLTCLVKGFYPSDIAVEWES
FT                       NGQPENNYKTTPPMLDSNGSFFLYSKLTVDKSRWQQGNVFSCSVMHEGLQNHYTQ
FT                       KSLSLSP"
FT                       /IMGT_note="frameshift in CH3: insertion of 1 nt"
FT                       /out_of_frame
FT   CHS                 1211592..1211597
FT                       /translation="GK"
FT                       /IMGT_note="for complete translation nucleotide g at 
FT                       position 1211592 has been included in CHS"
FT   INT-DONOR-SPLICE    1211592..1211594
FT   INTRON              1211598..1212878
FT   STOP-CODON          1211598..1211600
FT   POLYA_SIGNAL        1211701..1211706
FT   ACCEPTOR-SPLICE     1212876..1212880
FT   M1                  1212879..1213009
FT                       /codon_start=3
FT                       /translation="LQLEESCAEAQDGELDGLWTTITIFITLFLLSVCYSATVTF
FT                       FK"
FT                       /splice-expected-nt="g"
FT                       /splice-expectedcodon="gag[E]"
FT                       /splicing-frame="sf1"
FT   CONNECTING-REGION   1212880..1212931
FT                       /translation="AATGGELCGGAGRGAGR"
FT   TM-PART1            1212932..1213009
FT                       /translation="LWTTITIFITLFLLSVCYSATVTFFK"
FT   DONOR-SPLICE        1213009..1213011
FT   INTRON              1213010..1215135
FT   ACCEPTOR-SPLICE     1215133..1215137
FT   M2                  1215136..1215216
FT                       /translation="VKWIFSSVVDLKQTIVPDYRNMIGQGA"
FT                       /splicing-frame="sf0"
FT   TM-PART2            1215136..1215138
FT   CYTOPLASMIC-REGION  1215139..1215216
FT                       /translation="KWIFSSVVDLKQTIVPDYRNMIGQGA"
FT   3'UTR               1215217..1225000
FT   STOP-CODON          1215217..1215219
FT   POLYA_SIGNAL        1216601..1216606
FT   C-GENE              1225001..1246500
FT                       /functional
FT                       /IMGT_allele="IGHG2*06"
FT                       /IMGT_gene="IGHG2"
FT   5'UTR               1225001..1235055
FT   ACCEPTOR-SPLICE     1235053..1235057
FT   C-GENE-UNIT         1235056..1238880
FT                       /functional
FT                       /IMGT_allele="IGHG2*06"
FT                       /IMGT_gene="IGHG2"
FT   CH1                 1235056..1235349
FT                       /codon_start=3
FT                       /translation="STKGPSVFPLAPCSRSTSESTAALGCLVKDYFPEPVTVSWN
FT                       SGALTSGVHTFPAVLQSSGLYSLSSVVTVPSSNFGTQTYTCNVDHKPSNTKVDKT
FT                       V"
FT   DONOR-SPLICE        1235349..1235351
FT   INTRON              1235350..1235741
FT   ACCEPTOR-SPLICE     1235739..1235743
FT   H                   1235742..1235777
FT                       /codon_start=3
FT                       /translation="RKCCVECPPCP"
FT                       /splice-expected-nt="g"
FT                       /splice-expectedcodon="gag[E]"
FT                       /splicing-frame="sf1"
FT   DONOR-SPLICE        1235777..1235779
FT   INTRON              1235778..1235895
FT   ACCEPTOR-SPLICE     1235893..1235897
FT   CH2                 1235896..1236222
FT                       /codon_start=3
FT                       /translation="PPVAGPSVFLFPPKPKDTLMISRTPEVTCVVVDVSHEDPEV
FT                       QFNWYVDGVEVHNAKTKPREEQFNSTFRVVSVLTVVHQDWLNGKEYKCKVSNKGL
FT                       PAPIEKTISKTK"
FT                       /splice-expected-nt="a"
FT                       /splice-expectedcodon="gca[A]"
FT                       /splicing-frame="sf1"
FT   DONOR-SPLICE        1236222..1236224
FT   INTRON              1236223..1236319
FT   ACCEPTOR-SPLICE     1236317..1236321
FT   CH3-CHS             1236320..1236639
FT                       /codon_start=3
FT                       /translation="QPREPQVYTLPPSREEMTKNQVSLTCLVKGFYPSDISVEWE
FT                       SNGQPENNYKTTPPMLDSDGSFFLYSKLTVDKSRWQQGNVFSCSVMHEALHNHYT
FT                       QKSLSLSPGK"
FT                       /splice-expected-nt="g"
FT                       /splice-expectedcodon="ggg[G]"
FT                       /splicing-frame="sf1"
FT   CH3                 1236320..1236633
FT                       /codon_start=3
FT                       /translation="QPREPQVYTLPPSREEMTKNQVSLTCLVKGFYPSDISVEWE
FT                       SNGQPENNYKTTPPMLDSDGSFFLYSKLTVDKSRWQQGNVFSCSVMHEALHNHYT
FT                       QKSLSLSP"
FT   CHS                 1236634..1236639
FT                       /translation="GK"
FT                       /IMGT_note="for complete translation nucleotide g at 
FT                       position 1236634 has been included in CHS"
FT   INT-DONOR-SPLICE    1236634..1236636
FT   INTRON              1236640..1237935
FT   STOP-CODON          1236640..1236642
FT   POLYA_SIGNAL        1236743..1236748
FT   ACCEPTOR-SPLICE     1237933..1237937
FT   M1                  1237936..1238066
FT                       /codon_start=3
FT                       /translation="LQLEESCAEAQDGELDGLWTTITIFITLFLLSVCYSATITF
FT                       FK"
FT                       /splice-expected-nt="g"
FT                       /splice-expectedcodon="gag[E]"
FT                       /splicing-frame="sf1"
FT   CONNECTING-REGION   1237936..1237988
FT                       /translation="SCNWRRAVRRRRTGSWT"
FT   TM-PART1            1237989..1238066
FT                       /translation="LWTTITIFITLFLLSVCYSATITFFK"
FT   DONOR-SPLICE        1238066..1238068
FT   INTRON              1238067..1238796
FT   ACCEPTOR-SPLICE     1238794..1238798
FT   M2                  1238797..1238877
FT                       /translation="VKWIFSSVVDLKQTIVPDYRNMIRQGA"
FT                       /splicing-frame="sf0"
FT   TM-PART2            1238797..1238799
FT   CYTOPLASMIC-REGION  1238800..1238877
FT                       /translation="KWIFSSVVDLKQTIVPDYRNMIRQGA"
FT   3'UTR               1238878..1246500
FT   STOP-CODON          1238878..1238880
FT   POLYA_SIGNAL        1240260..1240265
FT   C-GENE              1246501..1268000
FT                       /functional
FT                       /IMGT_allele="IGHG4*04"
FT                       /IMGT_gene="IGHG4"
FT   5'UTR               1246501..1253779
FT   ACCEPTOR-SPLICE     1253777..1253781
FT   C-GENE-UNIT         1253780..1257933
FT                       /functional
FT                       /IMGT_allele="IGHG4*04"
FT                       /IMGT_gene="IGHG4"
FT   CH1                 1253780..1254073
FT                       /codon_start=3
FT                       /translation="STKGPSVFPLAPCSRSTSESTAALGCLVKDYFPEPVTVSWN
FT                       SGALTSGVHTFPAVLQSSGLYSLSSVVTVPSSSLGTKTYTCNVDHKPSNTKVDKR
FT                       V"
FT   DONOR-SPLICE        1254073..1254075
FT   INTRON              1254074..1254465
FT   ACCEPTOR-SPLICE     1254463..1254467
FT   H                   1254466..1254501
FT                       /codon_start=3
FT                       /translation="SKYGPPCPSCP"
FT                       /splice-expected-nt="g"
FT                       /splice-expectedcodon="gag[E]"
FT                       /splicing-frame="sf1"
FT   DONOR-SPLICE        1254501..1254503
FT   INTRON              1254502..1254619
FT   ACCEPTOR-SPLICE     1254617..1254621
FT   CH2                 1254620..1254949
FT                       /codon_start=3
FT                       /translation="PEFLGGPSVFLFPPKPKDTLMISRTPEVTCVVVDVSQEDPE
FT                       VQFNWYVDGVEVHNAKTKPREEQFNSTYRVVSVLTVLHQDWLNGKEYKCKVSNKG
FT                       LPSSIEKTISKAK"
FT                       /splice-expected-nt="g"
FT                       /splice-expectedcodon="gca[A]"
FT                       /splicing-frame="sf1"
FT   DONOR-SPLICE        1254949..1254951
FT   INTRON              1254950..1255046
FT   ACCEPTOR-SPLICE     1255044..1255048
FT   CH3-CHS             1255047..1255366
FT                       /codon_start=3
FT                       /translation="QPREPQVYTLPPSQEEMTKNQVSLTCLVKGFYPSDIAVEWE
FT                       SNGQPENNYKTTPPVLDSDGSFFLYSRLTVDKSRWQEGNVFSCSVMHEALHNHYT
FT                       QKSLSLSLGK"
FT                       /splice-expected-nt="g"
FT                       /splice-expectedcodon="ggg[G]"
FT                       /splicing-frame="sf1"
FT   CH3                 1255047..1255360
FT                       /codon_start=3
FT                       /translation="QPREPQVYTLPPSQEEMTKNQVSLTCLVKGFYPSDIAVEWE
FT                       SNGQPENNYKTTPPVLDSDGSFFLYSRLTVDKSRWQEGNVFSCSVMHEALHNHYT
FT                       QKSLSLSL"
FT   CHS                 1255361..1255366
FT                       /translation="GK"
FT                       /IMGT_note="for complete translation nucleotide g at 
FT                       position 1255361 has been included in CHS"
FT   INT-DONOR-SPLICE    1255361..1255363
FT   INTRON              1255367..1256646
FT   STOP-CODON          1255367..1255369
FT   POLYA_SIGNAL        1255470..1255475
FT   ACCEPTOR-SPLICE     1256644..1256648
FT   M1                  1256647..1256777
FT                       /codon_start=3
FT                       /translation="LQLEESCAEAQDGELDGLWTTITIFITLFLLSVCYSATVTF
FT                       FK"
FT                       /splice-expected-nt="g"
FT                       /splice-expectedcodon="gag[E]"
FT                       /splicing-frame="sf1"
FT   CONNECTING-REGION   1256647..1256699
FT                       /translation="SCNWRRAVRRRRTGSWT"
FT   TM-PART1            1256700..1256777
FT                       /translation="LWTTITIFITLFLLSVCYSATVTFFK"
FT   DONOR-SPLICE        1256777..1256779
FT   INTRON              1256778..1257849
FT   ACCEPTOR-SPLICE     1257847..1257851
FT   M2                  1257850..1257930
FT                       /translation="VKWIFSSVVDLKQTIVPDYRNMIRQGA"
FT                       /splicing-frame="sf0"
FT   TM-PART2            1257850..1257852
FT   CYTOPLASMIC-REGION  1257853..1257930
FT                       /translation="KWIFSSVVDLKQTIVPDYRNMIRQGA"
FT   3'UTR               1257931..1268000
FT   STOP-CODON          1257931..1257933
FT   POLYA_SIGNAL        1259313..1259318
FT   C-GENE              1268001..1286500
FT                       /functional
FT                       /IMGT_allele="IGHE*04"
FT                       /IMGT_gene="IGHE"
FT   5'UTR               1268001..1278117
FT   ACCEPTOR-SPLICE     1278115..1278119
FT   C-GENE-UNIT         1278118..1282154
FT                       /functional
FT                       /IMGT_allele="IGHE*04"
FT                       /IMGT_gene="IGHE"
FT   CH1                 1278118..1278426
FT                       /codon_start=3
FT                       /translation="STQSPSVFPLTRCCKNIPSNATSVTLGCLATGYFPEPVMVT
FT                       WDTGSLNGTTMTLPATTLTLSGHYATISLLTVSGAWAKQMFTCRVAHTPSSTDWV
FT                       DNKTFS"
FT   DONOR-SPLICE        1278426..1278428
FT   INTRON              1278427..1278633
FT   ACCEPTOR-SPLICE     1278631..1278635
FT   CH2                 1278634..1278954
FT                       /codon_start=3
FT                       /translation="CSRDFTPPTVKILQSSCDGGGHFPPTIQLLCLVSGYTPGTI
FT                       NITWLEDGQVMDVDLSTASTTQEGELASTQSELTLSQKHWLSDRTYTCQVTYQGH
FT                       TFEDSTKKCA"
FT                       /splice-expected-nt="g"
FT                       /splice-expectedcodon="gtc[V]"
FT                       /splicing-frame="sf1"
FT   DONOR-SPLICE        1278954..1278956
FT   INTRON              1278955..1279040
FT   ACCEPTOR-SPLICE     1279038..1279042
FT   CH3                 1279041..1279364
FT                       /codon_start=3
FT                       /translation="SNPRGVSAYLSRPSPFDLFIRKSPTITCLVVDLAPSKGTVN
FT                       LTWSRASGKPVNHSTRKEEKQRNGTLTVTSTLPVGTRDWIEGETYQCRVTHPHLP
FT                       RALMRSTTKTS"
FT                       /splice-expected-nt="g"
FT                       /splice-expectedcodon="gat[D]"
FT                       /splicing-frame="sf1"
FT   DONOR-SPLICE        1279364..1279366
FT   INTRON              1279365..1279447
FT   ACCEPTOR-SPLICE     1279445..1279449
FT   CH4-CHS             1279448..1279776
FT                       /codon_start=3
FT                       /translation="PRAAPEVYAFATPEWPGSRDKRTLACLIQNFMPEDISVQWL
FT                       HNEVQLPDARHSTTQPRKTKGSGFFVFSRLEVTRAEWEQKDEFICRAVHEAASPS
FT                       QTVQRAVSVNPGK"
FT                       /splice-expected-nt="g"
FT                       /splice-expectedcodon="ggc[G]"
FT                       /splicing-frame="sf1"
FT   CH4                 1279448..1279770
FT                       /codon_start=3
FT                       /translation="PRAAPEVYAFATPEWPGSRDKRTLACLIQNFMPEDISVQWL
FT                       HNEVQLPDARHSTTQPRKTKGSGFFVFSRLEVTRAEWEQKDEFICRAVHEAASPS
FT                       QTVQRAVSVNP"
FT   CHS                 1279771..1279776
FT                       /translation="GK"
FT                       /IMGT_note="for complete translation nucleotide g at 
FT                       position 1279771 has been included in CHS"
FT   INT-DONOR-SPLICE    1279771..1279773
FT   INTRON              1279777..1281864
FT   STOP-CODON          1279777..1279779
FT   POLYA_SIGNAL        1279885..1279890
FT   ACCEPTOR-SPLICE     1281862..1281866
FT   M1                  1281865..1281986
FT                       /codon_start=3
FT                       /translation="LDVCVEEAEGEAPWTWTGLCIFAALFLLSVSYSAAITLLM"
FT                       /splice-expected-nt="g"
FT                       /splice-expectedcodon="gag[E]"
FT                       /splicing-frame="sf1"
FT   CONNECTING-REGION   1281865..1281908
FT                       /translation="SWTCAWRRPRARRR"
FT   TM-PART1            1281909..1281986
FT                       /translation="TWTGLCIFAALFLLSVSYSAAITLLM"
FT   DONOR-SPLICE        1281986..1281988
FT   INTRON              1281987..1282070
FT   ACCEPTOR-SPLICE     1282068..1282072
FT   M2                  1282071..1282151
FT                       /translation="VQRFLSATRQGRPQTSLDYTNVLQPHA"
FT                       /splicing-frame="sf0"
FT   TM-PART2            1282071..1282073
FT   CYTOPLASMIC-REGION  1282074..1282151
FT                       /translation="QRFLSATRQGRPQTSLDYTNVLQPHA"
FT   3'UTR               1282152..1286500
FT   STOP-CODON          1282152..1282154
FT   C-GENE              1286501..1293408
FT                       /functional="functionality defined for the available 
FT                       sequence of the gene by homology with allele IGHA2*02"
FT                       /IMGT_allele="IGHA2*02"
FT                       /IMGT_gene="IGHA2"
FT                       /IMGT_note="partial gene in 3'"
FT   5'UTR               1286501..1291450
FT   ACCEPTOR-SPLICE     1291448..1291452
FT   CH1                 1291451..1291756
FT                       /codon_start=3
FT                       /translation="SPTSPKVFPLSLDSTPQDGNVVVACLVQGFFPQEPLSVTWS
FT                       ESGQNVTARNFPPSQDASGDLYTTSSQLTLPATQCPDGKSVTCHVKHYTNSSQDV
FT                       TVPCR"
FT   DONOR-SPLICE        1291756..1291758
FT   INTRON              1291757..1291970
FT   ACCEPTOR-SPLICE     1291968..1291972
FT   H-CH2               1291971..1292291
FT                       /codon_start=3
FT                       /translation="PPPPPCCHPRLSLHRPALEDLLLGSEANLTCTLTGLRDASG
FT                       ATFTWTPSSGKSAVQGPPERDLCGCYSVSSVLPGCAQPWNHGETFTCTAAHPELK
FT                       TPLTANITKS"
FT                       /splice-expected-nt="g"
FT                       /splice-expectedcodon="gtt[V]"
FT                       /splicing-frame="sf1"
FT   H                   1291971..1291987
FT                       /codon_start=3
FT                       /translation="PPPPP"
FT   CH2                 1291988..1292291
FT                       /codon_start=3
FT   DONOR-SPLICE        1292291..1292293
FT   INTRON              1292292..1292513
FT   ACCEPTOR-SPLICE     1292511..1292515
FT   CH3-CHS             1292514..1292905
FT                       /codon_start=3
FT                       /translation="NTFRPEVHLLPPPSEELALNELVTLTCLARGFSPKDVLVRW
FT                       LQGSQELPREKYLTWASRQEPSQGTTTYAVTSILRVAAEDWKKGETFSCMVGHEA
FT                       LPLAFTQKTIDRMAGKPTHINVSVVMAEADGTCY"
FT                       /splice-expected-nt="g"
FT                       /splice-expectedcodon="gga[G]"
FT                       /splicing-frame="sf1"
FT   CH3                 1292514..1292845
FT                       /codon_start=3
FT                       /translation="NTFRPEVHLLPPPSEELALNELVTLTCLARGFSPKDVLVRW
FT                       LQGSQELPREKYLTWASRQEPSQGTTTYAVTSILRVAAEDWKKGETFSCMVGHEA
FT                       LPLAFTQKTIDRMA"
FT   CHS                 1292846..1292905
FT                       /translation="GKPTHINVSVVMAEADGTCY"
FT                       /IMGT_note="for complete translation nucleotide g at 
FT                       position 1292846 has been included in CHS"
FT   DONOR-SPLICE        1292846..1292848
FT   INT-DONOR-SPLICE    1292846..1292848
FT   3'UTR               1292906..1293408
FT   STOP-CODON          1292906..1292908
FT   POLYA_SIGNAL        1292933..1292938
XX
SQ   Sequence 1293408 BP; 363979 A; 292393 C; 291994 G; 345042 T; 0 other;
     aagtcctgtt gaagcttact gatggagtca gagggggaaa cattgtacag cccagcggtt        60
     ctcacagact ctcctgcaaa gcctctgatt tcacttgtac tggctacagc atgagcttgg       120
     tccagcaggc ttcatgacag ggatttgtgt gggtggcaac agtgagttat cagggttact       180
     ctccatgagt acaagtaaat taacagtccc aagcaacacc ctttcaagtg cagtctacct       240
     taaaatgacc aatgtgaaag ccaaggacaa gaccttgtat tactgtgagt gacataggag       300
     caggaacatc tgcgtgagcc cagacacaaa atcctctgca gggagacagg agggaatcgc       360
     atggtagatg ctgattggaa ctaccatggg tcgctcagaa ctaccaggag gtactcagaa       420
     ccactagggg gcgctcagga caccagggga cgctcaggac aaccaggggg tgttcaggac       480
     accagggggt gctccgagcc accagggggc actcaggaca ccaggggacg ctcagaacca       540
     ttacttcctt gtaaatccat gatttcttta caaatgttac ttccaaaaca ttaacttaga       600
     accgggaatt tttttttttt tgacaaagtc tccctcttgt cgcacaggct ggagtgcagt       660
     ggcatgatct tggctcatgg caacctccaa ctcccgggtt caagcaattc tcctgcctca       720
     tacttctgag tagctgccac cacgtgcctg ccaccatgcc tggctaatgt ttgtactttt       780
     agtagagacg gggtttcacc atgttgacca ggctagtctc gaattcatga cctcaggtga       840
     tctgccccgc taggcttccc aaagtgctgg gattacaggc gtgagccacc atgcccagcc       900
     tatttgaagt tttaatgctg cgtgttttct gagtaacgct agcaatgatc tgtcagaaca       960
     atttttaaaa taggttattt atatatttta ttaaaatact attattaaaa gagtaaaatt      1020
     gtaaaaattg cacctgcaat ctcagcactt tgggaggttg aggcaggcag atcacttgag      1080
     ctcaggagtt ttagaacagc ctggcaacac aatgaaatcc tttctataca aaaaaaatag      1140
     ctgggtgtga tgctgcctgt ggtcccagat acttggaagg ctgaggtggg gggatccctt      1200
     aatcctggga ggttgaggct gtagtgagat gtgattgtac cactgcactc cagcctggtt      1260
     gacagagtga gaccctgtct taaaaaatgt gtatcctcga tacacattgt ttccatgaat      1320
     agagtttagt gtttttgtgc tgtgatactc aaacaattat atatgttttc taacattaac      1380
     tcaccatatg catggtgttt tgtttctttt tctttcatca gctgaaagtg gaatggaaat      1440
     taaacaccac tttaaaggct cttgttctcc actttggttg gctcctggtg tcctgttttt      1500
     cagactattt ctccatcttc ccttttactt taaaagtctt ttatcttcct cagtcaccat      1560
     gcaggaaata aaaattccct ttactttctg tcctccaagt ctggtgaaca gtttccttct      1620
     cttcataata actgaagcca accaagttta ggaggataac agttctcctt agaatatgct      1680
     catctacctg cagactctct gccctcctca cccttttcta aggtcctgca gacatcaccc      1740
     tcattccatt ccctcccttc cctaagtacg acagagtggg ctctgcagct cctgctgcct      1800
     tctgtgtgct cagccctggg gctccctagt gcttttgttg caggacgatc aaagatggga      1860
     gagaccaaac agagttcagg agagtccttt attaaaaggt gatcacctgg ctcagttgga      1920
     ctagcatcca ggaaagtctg agccctggac aaagaaagca gccacctttt acacagtcag      1980
     tggctgggag ctacatgatg caggaagcgt aattacagaa gagagaacaa aggcagttat      2040
     ttgttacttc atgtcttaca tccttgggaa tacatggttc tgtaacatat acttaacaat      2100
     cttatgacct tgcagctgtg ctggggaggt aagcaagaac cctctgagcc tcaaggaatg      2160
     tgaaaccaat gagaacagat aaggctcact gagcacagaa ggaaaaacag gcagttagta      2220
     ttctctaact tagactacgg gggaggggct acacactaca gtaagccctg aggggaaagt      2280
     taattttctg gctttacata ttgtaaaatt cataaattac ttcttcactt tgatgatgaa      2340
     gcccaaatcc ccatgtgctt acactctctc agaccaccct ctaggaagct gccattgtga      2400
     gtgagcccta aaaagcatgg gctgggttta gcttcatatt gctggatgtt ctctattata      2460
     aaggcatgtt ggcaaataaa gactacagtt tgtattgaat attcatgcca aagaagtttt      2520
     tttcaaaact tttcaagtaa aaaattttat cttgcctagt ttgaaaatta ccatctaaat      2580
     tcaacaaata aggtaataca gttttaaaag tgatgtttgt cttattagtt attcaattta      2640
     ttaacaacag actgatattt aaaataaata ccattgcaca tttaagtgcc atactgttct      2700
     gggatttttt aaggaatcag agagaccgac tctgttcagg aggatattta ttatttaggt      2760
     tcaggaggat atttattatt taggtgcacc ggccaagtcg aattaacatc caaaggactg      2820
     agcccagaac agagttcagt taccttttaa gcattttgtg gggtgggaga ggggacatct      2880
     gtgcagggtg aagcatacta cagaagtgag aaacaaagac agttattcaa ttgaaacatg      2940
     tattacatca tttcttcctt ttcaaggaaa aacatgtttt gcgacttgag tttatctttc      3000
     tagtgacctt gcagctacac tgctagggaa tcagggtctt caaaatgcct gagaagggag      3060
     gagaggtaag gctcattagc cacagaaaaa caggcagtta gtattttaaa ggactccagc      3120
     tctttctctt tttcagggag aattgggttt tcttacatac aactgagttt ctgcttacac      3180
     attctttaat ttcttttaat tcctgtttca atacttgaca agaatggcat ttacatacag      3240
     ttttaccaaa acatgtattt aaatatattt gtctttttaa tattggaata ggcagacata      3300
     cacgtagatc agcattattt tgtactaaaa tctcaaactg caaacacaat ttaaattcaa      3360
     ttaaataatt agaataatat gaaacaaatg ggtgtgttgt tttggtgttt acgtatgcat      3420
     tcacttttgc atgggcacat gtatgagtct ttgctgggct gttgtgcacg tatgtgtgtt      3480
     tgtatgacca ggaggttttc aaatacatca ttaaattaca tagttatatt aatcttggca      3540
     aggcacttgt attctgtttt ctttaattct gtttgcagaa agtagacaca tattcagtct      3600
     tagttccagt gtagggagtg cttttcatga gaaaaatacc agaaaaaagg gcaaacatgg      3660
     ggcccactaa tgtaaaaatt agccacaatg tgtatgtgtg tgtgtgtgtg tgtgtgtgtg      3720
     tgtctgagtt gaatagtaga gttggagtgg gcttctatcc acatgcacct gcgcctacag      3780
     gtattatcag gtacaataat caactgcaga accctaaagg aaataagagt ccccccaaac      3840
     ccctgaagag tgtttgggtt caccatgtgt ccaatgattc agtgcctctc gagctccagg      3900
     aaacggctcc ctggtgatgc gtgagatctt ttcttggggt gtccctgcag agttcgctgg      3960
     gtttcctaag gctgattcac tatttcaaaa gatggtgtga gaagcatatg gtgtaaataa      4020
     agcagaattc tgagccaggg cacagccact ttatactggg ctagagacac tggtaggaat      4080
     atactctgtc agctcagata gaaacctccc tgcagggtgg gggcagggct gcagggggcg      4140
     ctcaggacac atcgagcaca gtcttccgcc ccagagcagg tgcacatgag gctggggaga      4200
     ggttcctctc agggcctggg acttccttta aaaatatcta aaataagtat ttcacaagga      4260
     ctgctgatgt ttgtataaat atcctattca attgtgagca tttatcaaac tggatgttgt      4320
     aatgagaacc acttttataa tggcgatttc aaactctgct agttatctta ataatagcag      4380
     ctggaggtca ggaagagatt attacttata aataagtgca atttttggag agacacactc      4440
     attcccaaaa taacgcattc acatattaag gtctagaaat ggttcacgtt gcccctgaga      4500
     cattcaaatg tgggttcaaa gtgaggtgct gtcctcgggg agttgttccc cagtggagga      4560
     agctctgtca acacagagtt cagggatggg taggggatgc atggcctcta acaggattac      4620
     gactcgaacc ctcagcttct acaattgtgt cgtccgtgtg tcatggattt tctctttctc      4680
     atactgggtc aggaattggt ctattaaata gcatccttca tgaatatgca aataactgag      4740
     gggaatatag tatctctgta ccctgaaagc atcacccaac aacaacatcc ctccttggga      4800
     gaatccccta gagcacagct cctcaccatg gactggacct ggagcatcct cttcttggtg      4860
     gcagcagcaa caggtaaggg gctccccagt ctcagggctg aggaagaaac caggccactc      4920
     aagtgaggct ttacccaccc ctgtgtcctc tccacaggta cctactccca ggtgcagctg      4980
     gtgcagtctg gccatgaggt gaagcagcct ggggcctcag tgaaggtctc ctgcaaggct      5040
     tctggttaca gtttcaccac ctatggtatg aattgggtgc cacaggcccc tggacaaggg      5100
     cttgagtgga tgggatggtt caacacctac actgggaacc caacatatgc ccagggcttc      5160
     acaggacggt ttgtcttctc catggacacc tctgccagca cagcatacct gcagatcagc      5220
     agcctaaagg ctgaggacat ggccatgtat tactgtgcga gatacaccat gtggaaaccc      5280
     acatcctgag agtgtcagaa atcctgatgt gggaggcagc tgtgctgagc tgaggcagtg      5340
     atgcagcagt ttccttaact tccatcttat ctcattttgc atcgacatcc acctttatat      5400
     tagccaagaa tgtggaatag acgggtgctc ataagagatc cttaacttgc ccattttaat      5460
     gggttttcca gaagatgtga gaagccactt tgttagcaaa acatgccaaa gccatgccct      5520
     gctccagaca catgtgagcc catttcctgc tctttgctta actgacaagc tctcatcagt      5580
     gcacctgggc taatttcaca tcaggtacag gaatatgttc taaaggaaag ctaattttat      5640
     aatagcaatt cctgcttaat aaccttcagc ttcattgttt ttgtgtaatc tatcaacaaa      5700
     ttatgttagt tcaaggttct caatgggagt ttctaataaa tagaagggat gtatagaagt      5760
     tcccctaatt aaaacaattg tgaacacaat cttggtattc agctgtgtct ccacccttct      5820
     taccattcac cacaaagtaa ttctcacttc tggaagctgg gttcattttc aaattagttt      5880
     tttttttcat tttaatatct caaggttatt gtatgtgact attttagcag aaattgaatt      5940
     ttgggaaatt gaactaacca actgaaaata aattcacaac taaataacaa gatgccagaa      6000
     tataattggc tccaggcttt gttaattcag cagtttatct acctaggcat tatatttaca      6060
     catcttcatg ctatttttac agtcaactcc cattatgtaa gaaatggtca tacaccattt      6120
     cttacatatg gtaagatatc tacgatggat attcagaact acgataaaca ttggatgcat      6180
     ccaggtttgt ctgaactcac tgagattatt acactcagct gctgtttcaa tgtgtctgaa      6240
     ggtataaatg acaattcgaa tgacttgggt gtgtggatgg ttttacatga gtctttaagt      6300
     gtagaacaat attgaccctg ttccaaaatc tgtccttgat ccatgatcac actcatctcc      6360
     cagaacagct ccttcaacac atttcctatc tggatgaaga ggactctgtg cttggtgagg      6420
     ggaggccaca ggtagagaac tgagttctca gagggcacag ccagcatcct cctcccaggg      6480
     tgaacccgaa agactgcggc ctccctcatc ccttttcatc tctccataca aaggcaccac      6540
     ccacatgcaa atcctcactt aagcgcccac aggaaaccac cacacatttc cttaaattcg      6600
     gcgttctgct tacacgggaa atgctttctg agagtcatgg acctcgtgtg aaaagaacat      6660
     gaaaaacctg tggttcttcc tccccctggt ggcagctccc agaagtgagt gtctccagga      6720
     tgcagatatg aagatatggg aggctgcatc tgagcccagg gctcacagtg ggtctctgtg      6780
     ttcacagggg tcctgtccca ggtgcagctg caggagtggg gcccaggact ggtgaagcct      6840
     tcggagaccc tgtccctcac atgcgctgtc tctggtgact ccatcagcag tggtaactgg      6900
     tggagctggg tccgccagtc cccagggaag gggctggaat ggattggata catctattat      6960
     agtgggagga gctactacac cccgtccctc aggagttgag tcaccatgtc aatagaaacg      7020
     tccaagaacc agttttccct gaagctgagc tctgtgaccg cagggaggcg gagggggcgg      7080
     gcgcaggtgc cgctcaggac cagcaggggg cgcgcggggc ccacagagca tgaggccggg      7140
     tcaggagcag gtgcagggag ggcggggctt cctcatctgc tcagggctct ccctcctcgc      7200
     cagcacctca gctgtctcca gggctcctct ttctttctta tctgtggttc tgcttcctca      7260
     catccttgtg gcaggcaaga aaggaggacg acaggcctgg tgcggtggtg tatgcctgta      7320
     atccccgcac tttgggaggc cgaggcgggt ggatcacctg acgtctggag ttcgagacca      7380
     gcctgaccaa catggagaaa ccccgtctct actaaaagta caaaattagc caggcgtggt      7440
     ggcgcatgcc tgtaatccca gctactccgg aggctgaggc aggagaacgg cttgaacctg      7500
     gggggcggag gttgctgtga gccgagatcg tgccattgca ctccagcctg ggcaacaaga      7560
     gcaaaactcc gtctaaaaaa aaataaaagg aagaagacaa attttcctct tacagtcgaa      7620
     gtatcaacaa tactaggaac tttcctacga gttcctgaat ggcccctttt ttccttcttg      7680
     attaaaaaaa agttctaatg cttctcacca tctctttatt tgtgtcgtca attgaattgt      7740
     gccctctttg aaattcatat actgaaatct tgaatccagt ggatctatat tggaatttta      7800
     agtatataat taaggttaaa tgtggtcata cgtgtgagac cctaaagcaa tagacatgtt      7860
     gtctttataa gaagaggaag agacaccaga gacctctcac ttttcacgtg cacatagaaa      7920
     ggaggccatg tggacacaca gtggactaga aggtggccct gggcaagcca ggaagaagcc      7980
     gcaccaagaa ccaccctgcc agcaccgtga tcttggacat tcagactgca gaattgtaag      8040
     aaagtcagta tttgttgttt atgccaccca ctcctgttgt cttcttgtga agacccacac      8100
     agactaatac tacataactc tgttagcttt gtctcctaga aggagaagca ggcccctgag      8160
     gctgggcaca tctcagattt ccatataaag taggcaaaaa tagtaactct tataaaaaaa      8220
     tgtgtcaagg ccctttcttt cttttttttc tttgagacag agtctcgctc tgtcaccagg      8280
     ctggagtgca gtggcacgat ctcggctcac tacaacctcc acctccaggg ttcaagtgat      8340
     tctccttcct cagcctccca agtacctggg actacaggca cccgccacca accctggcta      8400
     attttttgta tttttagtag agatggggtt tcaccatgtt agcaaggatg gtctcgatct      8460
     cctgacctcg tgatcctccc gcctcacctc ccaaagtgct gggattacag gtgtgagcca      8520
     ccattggcca tttttggtgg aaaggtctct gaaaccaacc ctggtgtctg tgccacaaaa      8580
     gttgtctttt atcttttatt tggacataac aaatggacag tgagtaccac ctggatggag      8640
     actgaccact gaccatcttc tgctgtctcc taagtatgtc aaagaaaacc acatcgacat      8700
     cactattttt cttcaacctc ctcgaatgaa ctatagaaat gatccctgaa agtatagtct      8760
     attccttcaa ccttctaaat ttgcactaaa tctcttccta aatgaggagc tacagggggt      8820
     ctgagtttta ttcccttctt tccagttttc cccaagtaca aagagcagaa tagactttca      8880
     gtgaaatttg gctgacaatg cccctaacac cacatcactt tctaaaaccc atatcctgcc      8940
     tccatccttc tctggacacc cctcatcggg ctacccagga atggccagaa gctggcatca      9000
     gcttctgggc tgaggccacg agctatacac acatgtgatt tcagttacac agactctact      9060
     gcaggaccac acctgtgttc tgaggcactc aggcacctgc tgatctcagt cattctctaa      9120
     taaattacac acctcttatt aataaaggtc caaatggtcc catcagctgc agagcagtgg      9180
     agtaaagctc atgggtgggt ccgtcaggca gaagtcagac aatgggatgg gtaggttggt      9240
     cacctccatt gtcactgagg ttcccagtgg attttaacag aataaaacaa tattaacaac      9300
     aaagggatct tgaaagtatt taaggtccag gaattttgtt tggggggcta gagaaagaca      9360
     tttttaaaca gaaagcccta aagcacatac agcagctgac agagtggcca ctgtgcacat      9420
     gacggctgag gagacagatg gtaggggaca ttcccccaag atgtccctgg gtgtgtgatg      9480
     gttggactcc tcatgcatat gaaaataaga gctggactca ggtagaaaca agggccatac      9540
     cccataggaa aggaagaaaa aaacagatgg gcaaccctgg agagagctca ttagatttgg      9600
     taggtttgag atgaaaatta cttccatgga ttttaatgtt ctaagctaag taagaatctc      9660
     ttcaataaag tgagaattaa aaggagaatg gagctaggag ttgagagagg caacaaataa      9720
     tgagagagca gaaagcaaat ccacaaaaaa ctgtcacatg acagaggcca gaatggagct      9780
     gatgcagctg cgtcatttcc tacagaccta gttgaccatg tggagaagag gcttgaacaa      9840
     atggggacgt tctccaacct tccaaatcag ccttcttctt aggcttgagt agtaatcatg      9900
     gttctgaggg tgaccttcca aattttcctg tctgtacctc ttccaccagg ggtagagtgt      9960
     cttcccaacc ataatggttt ctctccagtg tcctcagctt ctcctccttt gtacttaccc     10020
     tgcagattaa ggatttcttc tagatggagt ttcctgggaa tcctctgttt tcttttgttt     10080
     ctgttgactc caccacaccc cataggtcac aggtttgatt ggatttcccc tggagacagt     10140
     ggaggtggat ccaggcgttc aacagtcctc gctgttcttc ccttcctgtc agcaccacag     10200
     gacagcagat aagggagttg actgtggatt tttcaaattc ctgggaaaag cctgcaagga     10260
     ctagtagatt cacactctaa taccattagc acatgcatcc aaaaaaaatc ctcactaaat     10320
     atttccaggt tagcctgttc ctctctcaat gccatccagt ggcacctgcc ctgggttcac     10380
     taacatgtgg gccccagtcc tctctgcagg cctctctctc ctcacatttc agtcttcttg     10440
     ctttctctgt gaaactaact cacaaatgtt acaggttttt cttcttcctt tattcacagt     10500
     ttttcaggtt tgttgttaat gaggacagaa taagatcata gtttcctcat ttttcacatt     10560
     cccacactga gtagatttct atataaaagc cagaaactaa gagaacaaat caaatatcca     10620
     tatccattac aggtgaatgt taaacaattt gacataagat tatgaagtaa agtacaatgc     10680
     agcattagaa tcaaggcatc ctcattcaca taaagtcatg gctacattca caaataactg     10740
     cgctgagtgg aagtagctaa agtattaaca gtgcacttca taaacttcta tttgtataaa     10800
     ctgtagaagg taaaactatt ctaaagtaac agagaagaga atttggtgag aattgggtgt     10860
     tgaaagtaat ggggtgttga gatgaaatta cagagaagtg acagaaagat ttagaggtta     10920
     acttaattgc acacgactta attgaaaccc tgattaaagg gttgcacaca tacattaaca     10980
     tattccaaat tgtgcagtat atatttgaat taattatttg ttaattttgt acttaaaaaa     11040
     gcagtaacaa agaaacaaat gaatgtgttg gttgaggagg agcaaaaaat agatggctat     11100
     taacacttga aacatcgctg acttttgaaa gtacacatgt atgaacactg gttctctcta     11160
     tatatatttc agataaacaa cagaatacac taaaagaaaa cagagatatc ctggcagggg     11220
     tggaatcctg caaacctcac taggcatgtc ccacactgcc ctggagttgt ctcaggggag     11280
     cagtcttctc tagtggtcag aggcacaggc atggataatg ggactaattc tgtccagctg     11340
     tgtgatcttg accatattgt acagccactc tgttctgtgt gtaatgtaaa ttccttaaaa     11400
     tgtaacattg acacttgcat taaatgtatt ctgcaaatat gtagaaatga aataagatga     11460
     tgactgctaa atagttatca aggcatgatc acataatata aaatgatatt ttcctgagtg     11520
     ataagatgac taccaatctc ccccaaggca ctttgtctgc tctgacccct gcccctcctc     11580
     aggattccca tcccagagct tgctatacag taggagacat gcaaacaggt ttctcccttt     11640
     gttgatgaaa accagcccag tcctgacccc gtaggtttgg gagagaagcc ccagccctgg     11700
     gattcccagg ggtttccgtt ttgtgatcag gactaaagac agaggaccca tcatggagtc     11760
     tgggctgagc tgggtttcac agttgttatt ttaaaaggta aaccagagaa atagagtgtg     11820
     agtgaatata tttgagagaa acagtggata tgtcaggcag tttctcacca ggatctctac     11880
     gagtttacgg gtgtccagtg tgaggtacag ctggtggagt ctggagagga cccaagacaa     11940
     cctggggttc cctgagactc tcctgtgcag cctctggatt aaccttcagt agctactgaa     12000
     tgagctcggt ttcccaggct ccagggaagg ggctggagtg agtagtagat atacagtgtg     12060
     atggaagtca gatatgttat gcacaatctg tgacgagcag attcaccgtc tccaaagaaa     12120
     atgccaagaa ctcactgtat ttgcaaatga acagtctgag agcagagggc acagctgtgt     12180
     gttactgtat gtgaggcacc aggtaagaag acatcagtgt gaacacagac acagaggttc     12240
     ctgtaatgat aagggaggag gctgggataa agggagcact caagacccac agaaaacagg     12300
     ggaagctcta gggcaggtgc agacggtcgt catgggctac tttccttcag ggtctgtggc     12360
     tttctctgca tccaacagtt cccctgggag cctcttgaca tttatgtttc tgtgcccatc     12420
     cctgaggtct ctggattaga aaaatttatt ataagaagtg gaaacattct catttgtccc     12480
     aaaggcagat gtaagtaatg gaggcataaa aatgcacagg aggccaggga ggctgtagac     12540
     actgccaccc cagaatgtca atctcaccac tagtgctgga gaagagtggg agtttgatgg     12600
     agcttaccta aataccctgg gtccaagcta agttcagcga ggccactggt gcctcccaga     12660
     gctcagttgt ccatcaggaa tctcccatgt gtcccagcag cagccatgcc tcagtacttc     12720
     cgctgtgcac agccattgtc tgggaggagc tcccaggttg ggtgtctttg gcacacacca     12780
     ggtgacaggt gttagagtgc agtgcagcag ctggctgcct ggtctattgg gctccctgat     12840
     gttggagaga tgggaggtgg attctcaggg ccagcaccca gtttgtgaat ttttatataa     12900
     aaaccatgat tttacttcat tttctcagat gacatagata attaagaaca gagtctggtc     12960
     cggacgtggt ggctgatgcc tgtaatccca gcactttggg aggccaaggc gggcagatca     13020
     cctgaggtcg agagttcgag accagcctga ccaatatgga aaaatccatg tctactaaca     13080
     atacaaaatt agccaggcat ggtagcatat gcctgtaatc ccagctacta gggaggctga     13140
     ggcaggagaa tcgcttgaac ccgggaggtg aaggttatgg tgagccaaga tcacaccatt     13200
     acactccagc ctgggcaaca agagtgagac tccatcaaaa aaaaaaaaca acaaaaaaaa     13260
     aacagacttt gcaaaataat gttcaacctt atcccaaatt cattgtttct taattctgtg     13320
     cagcatccag acatattatt accttcctca tgatgaattg ttctgtttaa actgagatca     13380
     gttttttctc atattctttg gtttctgtct aagaacagag atcttgatta cagtaaggtt     13440
     ggttctttcc tcatactaac cctcacctcc cccagagaaa gagcagagat tgtcctcact     13500
     ctgagtctga gggaggagct gttcctgtac aactcagagc ctgcagagac ccccaggtgc     13560
     agcttcagtg agtcagacat ttctccatgt gggtgacctc cactgccagt gattgctgct     13620
     gaggtctaat tgtgggttaa taattaggac acccttcagg ttgtcacatc ccaatcctat     13680
     tctgaacatc accattatca tagacagaga tagttcaatg cccattctac tgacataagt     13740
     ttctctttat aatttggttc caactatgga gaaaaacgtg accctatatt tgtctgaatc     13800
     taacctcaga atctgctgca ttgctctagg agattcacaa attgaacaca agtgagctgt     13860
     ttattctcat aaaaatgtac atatttgaga atttcaatgt attgcccaga acctgtgcat     13920
     gccaacaact gtgtttctca gcgcactctt ggcctggtga agccctcaca gaccctctcc     13980
     cttacctgcg ctgtgtctgt gttccccatg ataacgaggt cttcctgctg aagctggatc     14040
     catcagcccc ccagggaagg gaatggagtg gattgggtgc ataggtcatg aagggagcac     14100
     acattaccac ccactcctca agtgtccagt caccatcccc agatccgtgt ccaaaaaagc     14160
     agttcttcct acagctgagc tacatgagca acaagcacat agccatgtat ttttaagcca     14220
     aagacacagt gagggaatca cagtgtgagc tcacacccaa acctccctgt gggggtgcat     14280
     aggacagaag gggttgctca ggtccccagg gggctctcag gacaccaagg ggcactcaag     14340
     accattgtag aggcacgcag gtcagccggg ggctctcagg aaccatcgag gaaaatcagg     14400
     acaccagagg gtgctcggta cagcaggggg ctcaggacca ttgtgaggac tcagaaagag     14460
     caggttcaag gctcagcctt agggcaggtg aagctgggat gaaaaggggc tggatgaggg     14520
     gttttgtgtc accatcatat ttcaccacta gacaccctcc actacatctg ttctaatgca     14580
     tgttctaaca catgtgagtg tttgtatgac tagaaaatga tattttgggg ccaggtgcag     14640
     tgactcatgc ctgtaatccc agcactttgg gaggctgagg cgggcagatc acctgaggtc     14700
     aggagttcaa gactagcctg gccaacatga tgaaactccg tttctactaa aaatacaaat     14760
     attagccagg cccagtggca cacgcctgta atcccagcta ctcgggaggc tgaggcagga     14820
     gaatcgcttg aacctgggag gcagaggttg cgttgagcca agatgtgcca ctgcactcca     14880
     gcctgggcaa cagagcgaga ctccattaaa aaaaagagag aaaaaaagaa aatgatattt     14940
     atatcaatat ataaccatag ctaggtgtgt caagttgtcc tctccatcta tgtcagcctt     15000
     gtccataagg actaattccc tgtaatttct tgaggacctc ataaactgtg gtcaattatg     15060
     taatattcct ctcttttttc tgccttcctt cctgccttct ctctctcaca cagaaactga     15120
     cgtacaccca ccccacaaca cacataaatc tatacctttt attacctgat gtatttaata     15180
     aacttgatta atgtgcagct tttcaagctt agttgcttgt gaggttgtaa gaataagaac     15240
     cattttttca aagggaatgc ttccagtttt tgcccattca gtatgatatt ggctgtgggt     15300
     ttgtcataga tagctcttat tattttgaaa tacgtcccat cgatacttaa tttattgaga     15360
     gtttttagca tgaagggttg ttgaattttg tcaaaggctt tttctgcatc tattgagata     15420
     atcatgtggt ttttgtcttt ggctttgttt atatgctgga ttacatttat tgatttgcgt     15480
     atattgaacc agccttgcat cccagggatg aagcccactt gatcatggtg gataagcttt     15540
     ttgatgtgct gctggattcg ttttgccagt attttattga ggttttttgc atcaatgttc     15600
     atctgacaaa gggctaatat ccagaatcta caatgaactc caacaaatct acaagaaaaa     15660
     aaaaacaacc ccatcaaaaa gtgggcgaag gacatgaaca gacacttctc aaaagaagac     15720
     atttatgcag ccaaaaaaca catgaaaaaa tgctcatcat cactggccat cagagaaatg     15780
     caaatcaaaa ccacaatgag ataccatctc acaccagtta gaatggcaat cattaaaaag     15840
     ttaggaaaca acaggtgctg gagaggatgt ggagaaatag gaacattttt acactgttgg     15900
     tgggactgta aactagttca accattatgg aagtcagtgt ggcaattcct cagggatctg     15960
     gaactggaaa taccatttga cccagccatc ccattactgg gtatataccc aaaggactat     16020
     aaatcatgct gctataaaga cacatgcaca cgtatgttta ttgcggcatt attcacaata     16080
     gcaaagactt ggaaccaacc caaatgtcca aaaatgatag actggattaa gaaaatgtgg     16140
     cacatataca ccatggaata ctatgcagcc ataaaaaatg atgagttcat gtcctttgta     16200
     gggacatggg tgaaattgga aatcatcatt ctcagtaaac tatcgcaaga acaaaaaacc     16260
     aaacaccgca tattctccct cataggtggg aattgaacaa tgagatcaca tggacacagg     16320
     aaggggaata tcacactctg gggactgttg tggggtgggg ggagggggga gagatagcat     16380
     cgggagatat acctaatgct agatgacgag ttagtgggtg cagcacacca gcatggcaca     16440
     tgtatacata tgtaactaac ctgcacaatg tgcacatgta ccctaaaact taaagtataa     16500
     taaacaaaaa aagaaaggtg agggagatac tgtgagtaaa gagctcgctt ggggtctgca     16560
     atgtagtatg gcttagtcag tgtgttagtg ctctgtcctc ttctttctga aatcacagtt     16620
     caccatcacc tccttatttt ctagaaacac ctggaaatag tgaaactttt ttacttccct     16680
     tatatttcat attagtgcta tacccaggga tcatcagtta accacaatga cagtgacaaa     16740
     aagctgcctt catattttct cccaggagaa ttttggctcc aagaatcaag gctttaatag     16800
     gattcaggag aagtcagtga tcacagggca gatgcctcag gtttaaagtc acatcaaacc     16860
     gagtgtgaac cttctgtaac tcgcaactgt gctaactttt ggaggtttct tgaaaaattc     16920
     tagtcagttt gggttaaaga atggtttcag taatgtcaca ctcagggtca tgactgctca     16980
     actggtcatt agccctggga agtaacttcc gctaacattg gcttacctca taggagctta     17040
     taaattgcca tcatattgat tatctcatta ggtcatcagg atcctgaaga atgatctttt     17100
     aagaaaacaa tctttttatc aggcttattt atcattgctt ctatatgctg ctcttgtcct     17160
     gcagaggagg cagcctgggg tcaatcaaga tactgggtat ttgggttcat actttaagcc     17220
     ccccactctc tcatggtcta acttcagaca agtcacctct gctttttgag cttcactttt     17280
     ctcagccacc aaatggatgt agagataata tttgcttgcc caattttgaa atgtaggacg     17340
     gggcatcatt aattaattag ttcttttgtc cacaaacatt tacccagctc cagcatgagc     17400
     ccagcctatg cttggtcttc tgcagaacag agaaatgcct tagaaggaga ttctgactct     17460
     agtcatccac gctgtagtta aagaactgtg agctgggagg gtggggcagg cgagggggcc     17520
     ttctgatatt tatgtctcat ctttccctga aaaaatactg tgtttctact ttgctgcttg     17580
     tattcattcc caacctatta ctgttcataa aatgcataag gtttactttg actccaagtt     17640
     tatttttatt aattaaaata ttccatgttt atcataatga gggtgattct cattaaaact     17700
     cttttcattg ccttcctgac taaaagcaag tacattttta ttttaataaa cataagtaac     17760
     acaataaaaa aaaaagaacc atgtgtttcc cagctgtgta cctctctaag ctgagtagca     17820
     tcagcttata atacccagca tgaaaaacaa ctcaaatgtc agtcatcagc ttaattggta     17880
     aagaaattga ggaaaactca tccattggca tattacccac tactacaagc aactaatgtt     17940
     ggctactgtc aacagcatgg caaaattcac aagtagttct cataagtaaa atgagccaaa     18000
     caaataaaag tacttacata agatacaact tttataaatt tcataaaatg aaaagtaatc     18060
     taaacttaca caaaaatcag tagctcactg tgaatattgt aggaaaaggg aaggtctagg     18120
     aaggagaaat tacagaacaa gagaaaattt taaggaaatt gacttgttat ctacgttgat     18180
     aatgattatg tttgggacaa tattagtaaa attgaacact tcataaagat attttttaat     18240
     ttaacctaat taaagatagc actaattata acatgtataa tttggtagaa aaggagttag     18300
     acagagataa ataaaataca tgaaatgtca gagacacctg aaatacacag caacgagccc     18360
     taggcatctc tgtattttta gagaaacatt agaatacagc aaaataatgg actaattcta     18420
     cgacattaag aaagtttatc aaacccacca ggcatgttgt attagttcat tttcacactc     18480
     ctataaagaa ctacgtgaga ctgggtagtt tatgaaaaaa gaggtttagt tgactcacag     18540
     ttctgcacag ctggggaagt cacaggaaac ttacaatcat ggtggaaggt gaaggggaag     18600
     caaggcacgt ctcagatggc agcaggtgag agagagagag agagaagtga catacacttt     18660
     taaaccctca ggtcttgtga gaactcacta tcatgagaac aacatgggga agctgccccc     18720
     gtgatccaat cacctctcac ttggtccctc ccttgacatg tggggattac aatttgagat     18780
     gatatttagg tggggacaca aagccaatgc atatcatatg tccagctcta tcctggagtt     18840
     gtttcaggaa tccagtgtgt cctgttgata gaaaccgtga cactgagctc acagcatcag     18900
     tagtatttga caccatgcaa agtcaagaga tctcaactga gatttagtgt ggatgttttg     18960
     tctgatgaag tcgtacactc agagcaagtg aatatggaga ggtttattat ctgcactgta     19020
     gaggtgtctg ctgagagcag ggcagttctc tcaggactgt ctaaaatggc ttggtaaagg     19080
     aggaatggag actgactcag ggtttttata atggtttggt ggtgggggca gagtgaggct     19140
     tcccactcac agaaaggggt ttatagggtt tgaaactccc aatggcatca catgaagaag     19200
     ctcctgtgat tgcaaactag attcaccttg tttgacaaaa gaggagatga tagaggagtg     19260
     agccttaagt cctcagcagt catgcatcaa aaatattgtg acaacttatt ctatgtagca     19320
     agaataaaaa taatgaataa gcaaaaagat aagggttcaa tgcaggtgga caacatggag     19380
     gtctacagga atgcaatgac tttataaata taagcaaata atattgagaa aaaggaggag     19440
     gggttgggga attagagagg ggtcctggtc taatgtcttg ggtagaagct tctcacaaca     19500
     aaggactatc agcttattct gcaggtctta ggccagctat ctgcttaaaa acatccaaaa     19560
     ccccagagaa tctataagga tgctcagttt aatatctcct atttgagtag ctttacagtt     19620
     gtgtggaatt ctttattgat tcttttttat ttgtttttag agacaggctc tcaccctgtt     19680
     gcacagtttg aagaacagtt gtatgatcaa agctcactgt aattttgaac acctggctca     19740
     cataatcttc ccatctttgc ttcctgagta tctagaagta gaggggaacg ccacccctca     19800
     cctgcttatt ctttaaaata ttttttcaga gaaataacat ctctttatgt tgctcaggtt     19860
     ggcttcgatt gcctcgtctc acgggatttc cctcattttt gcttctgaaa gtggtgtgat     19920
     tatagcgatg atccactgca tctggcctga atttattctt tagttgtaaa acatgaaccc     19980
     aacaattaac tgcctgaata ttttctgcag cgagttagtt aaaagtatct gataagattc     20040
     cttccaatat gattcaagag gagtattttc tgctgatgtt ccttccaatt tccttgctga     20100
     agatcacaag agtctgtgga aaagatgtaa caaaaaggca gcctcaaact cttcatgatt     20160
     ggagtggata tcacacagga atcactttca gtattttgta acacatgcat gcaataggac     20220
     aaagatgatc tctgggggta aactctataa acgtatgggc cttttagctg ccaagtcata     20280
     gggtaataac tgatgcaccc tgaggagtgg aacatgattc catagtgcta gtgggagaac     20340
     ctttggccaa gggagtttta cattttatta aagatttgat catttttatg tagagatgcc     20400
     atttttaaaa cattcctaga agattgttga gtaggattca ttctgtctta tatgaacaat     20460
     gataatgctc tccatggtta gataatatta taaactagat tgagctagag cgtttggtgt     20520
     atttaatcac tttttttagc ttccattagc atttggatta aatgaaaatc ataacaaaca     20580
     gtctctcaga ccacagtgcg atcaaattag aactcagaat taagaaactc attcaaaacc     20640
     acacaactac atggaaactg aacaacctgc ttctgaatga ctactgggta aataatgaaa     20700
     ttaaggcaga aataaacaag ttctttgaaa ccaatgagaa caaagacaca acataccaga     20760
     atctctcgga cacagctaaa gcagtgttta gagggaaact tataggacta aatgctcatg     20820
     agagaaagca agaaagatct gaaatcgaca ccctaacatc acagttaata gaactagaga     20880
     agcaagagca aaccaattca aaaactagca gaagacaaga aataacgaag atcagagcag     20940
     aactgaagga gatagagaca ggaaaaaccc ttcaaaaatc aatgaatcca ggagatagtt     21000
     ttttgaaaag attaacaaaa tagatagact gctagccata ctaataaaga agaaatgaga     21060
     gaagaatcaa atggacacaa taaaaaaatg ataaagagga catcaccact gatcccactg     21120
     aaatacaaac taccatcagg gaatactata aacacctcta tgcatataaa ctagaaaatc     21180
     tagaagaaat ggataaattt ttggacacat acaccctccc aagactaaac caggaagaaa     21240
     tagaatccct gaatagatga ataacaagtt ctgaaattaa gtcagtaatt aataatagcc     21300
     tactaataat attattaata gtaattaata atagccaact aaaaaaagac cactaccagg     21360
     tggattcaca gctgaattct accaggggta caaagaggag ctggtaccat tcctcctgaa     21420
     actattccta acaatagaaa aagagggact cctccctaac tcattttatg aggccagcat     21480
     caccctgata ccaaaacctg tcagagacac aacaaaaaaa gaaaatttca ggccaatatc     21540
     cctgaagaac atcgatgcaa aaatcctcaa taaaatactg gcaaaccgaa cccagaagca     21600
     catcaaaaag ctcatccaac aggatcaagt cggcttcatc cctgggatac aagggtggtt     21660
     tgacatatgc aaaacaataa acataatcca tcacgtaaac agaaccaatg acaaaaacca     21720
     catgattatc tcaatagatg cagaaaaggc ctttgataaa atccaacacc acttcatgct     21780
     aaaacctctg aataaaccag gtattgatgg aaagtatctc aaaataataa gagctattta     21840
     tgacaaaccc acagccaata tcatactgaa caggcaaaag ctggaagcat tccctttgaa     21900
     aactggcaca agacaagggt gctctctctc accactccta ttcaacatag tattggaagt     21960
     tctggccagg gcaatcaggc aagagaaaga aataaagggt actcaaaata ggaaaagagg     22020
     aagtcatatt gtctctgttt gcagatgaca tgattttata tttagaaaac cccatcgtct     22080
     cagcccaaaa actccttaag ctgataagca acttcagcaa agtcccagga tataaagtca     22140
     atgtgcaaaa atcacaagca ttcctataca ccaataatag acaaaaagag agccaaatca     22200
     tgagtgaact cccattcaaa attgctacaa agagaataaa atacctggga atacaactta     22260
     caagggatgt gaagaacccc ttcaaggaga actacaaacc actgctcaac aaaataagag     22320
     aggacatgaa caaatggaaa aacattccat gctcatgaat aggaagaatc aatattgcca     22380
     aatggccata cggcccaaag taatttatag attcaatgct attcccatca agctaccatt     22440
     gacttccttc acagaattag gaaaagctac ttaaatttca tatgggacca aaaaagatcc     22500
     cgtatagcca agacaatcct gagcaaaaag aacaaaactg gaggcatcac gctacctgac     22560
     ttcaaactat actacaaggc tacagtaacc aaaacagcat ggtacttgta ccaaaacaga     22620
     catatagacc aatagaacag aacagaggcc tcagaaataa catcacacag ctacaaccat     22680
     ctgatctttg aaaaacctga caaaaacagc aatggggaaa ggattcccta ttttataaac     22740
     agtgctggga aaactggcta gccatatgta gaaagttaaa actggatccc ttccttagac     22800
     cttatataaa aattaactca agatggatta aagacttaaa cataagacct aaaaccatac     22860
     aaaccctaga agaaaaccta ggcagtacca ttcaggacat aggcatgggc aaagacttca     22920
     tgactaaaac atcaaaggca atggcaacaa aagccaaaat tgacaaatgg gatctaatta     22980
     aactaaggag cttctgcaca gcaaaagaaa ctaccatcag agtgaacagg caacctacag     23040
     actgggagaa tgtttgtgca atctatctat ctgacaaaag ggctaatatc cagaatctac     23100
     aaagaactta aatttacaag gaaaagacaa acaatcccat caaaaagtgg gcgaaacata     23160
     tgaacacttg cttctcaaaa gaagacacat gaaaaaaaaa gctcatcctt actggtcatt     23220
     agaggaatgc aaatcaaaac cacaatgaga taccatctca caccagttag aatggccatc     23280
     attaaaaagt caggaaacaa cagatgctgg aaaggatgtg gagaaatagg aacgctttta     23340
     cactgttggt gggagtgtaa attggttcaa ctattgtgga agacagtgtg gcgattcctc     23400
     aagcatcaag aaccagaaat tccatttgac ccagcaatcc cattactggg tatatcagca     23460
     aaggattata aatcattcta ctgtgtctta cgtgacacat gaacacatat gtttattgca     23520
     gcactgttca taattgcaaa gacttggaaa caacccttat gcccatcagt gatagactgg     23580
     ataaagaaaa tgtggaaaat atacaccatg gaatactctg cagccataaa aaggatgagt     23640
     tcatgtcatt ttcagggaca tggataaagc tggaaaccgt cattctcagc aaactaacac     23700
     aagaacagaa aatcaaacac tgtatgttct cactcaaaag tgggaattga acaatgagaa     23760
     cacatggaca cagggagggg aacatcacac acaggggcct gtcacggggt ggggaagcta     23820
     ggggagggat agcattagga gaaataccaa atgtagatga tgggttgatg agtgcagcaa     23880
     accaccatgg cacatatata cctatgtaac aaacctgtac attctgcaca tgtatcccat     23940
     aacttaaagt ataataaaaa atagtaactc aaaaaattat gttaatcagt tctctagtag     24000
     gtagaaattc atctgggagt ttcttctctt gtccattttg ataggatctg cagaagacat     24060
     aagaaccctc tctgttaaaa atattcctaa gttgtacacc atctagaaac atatatactt     24120
     aattataatt tttaatttat taaaaagctt taataagtgc aatgttttct gccttctgag     24180
     ttgatttcat aacacataga aggatatatc ctgaatgaaa ctttgtacta gtaatacaaa     24240
     ttaaaggtta ataacctcta cttttattat tgaggtatta cccaccaata tataatctta     24300
     aatcagtgct ctcaatgggg cttttaccta aataatataa aaaatatttt cctgatcttg     24360
     acacaaaata gatgtgaaca cattcttcat attcagccat gtctcctgtc tattacatta     24420
     tgaaccacat gctaactttg atttacttgg gacttgttct aatttcaaac tagttatttt     24480
     ttatcttcat gcagctagat tattatgtgt gagtatttta tcagagagtg ataaagataa     24540
     tttaaacaag taattgtgtg tctcaggact cttcttgggt tctgagacaa gaaatgccat     24600
     tactacctga aggattgaga caactttgaa aatgtcagag gaatcatctc taattgcttc     24660
     taaatttcag ttttatcaat tcttatgctt tctgaattat ttttgatttt agttattaaa     24720
     ggattctttc tttttctctt aatctagcta agaatttgtc cttttttaag aaaaaactct     24780
     taggtttgtt gatttttcta ttgcttgtaa agtctttttt cacttatttc atttactcta     24840
     aagcctcata aacacattgg tgacaggtag atggagaact tgattgtaaa tgccctgttt     24900
     ttgttagacc cagctgatca tgtcaggaag ccccctgagg ggtctaggag ctgactgtgg     24960
     ctcccaccac tggacaagga tgagaacctc catgctttgg ggatgggagg acatgcaggg     25020
     cagtgtgagg ggaggagggt tgctggcagg tccagcattg cttcccctgg ctgcaccact     25080
     catgtccttc ctcactcaga gattcagtca tttcttccca ggtggaaaaa tcagttaact     25140
     ttatctttga tatatggatg ggagcccaag atagaggctt gtcagtcatt atttctgtgc     25200
     ctgctgcctt gggaatgtct gaatttctca tgctctgtcc tgggtctata ccccagtggc     25260
     tcccctactc catggcaccc acctgtgcct gtgcatggcc cctgatcaca gcctccgtga     25320
     aacatctggt ttggctcagg aagaaccctc cctgccttgt tttcctgctg tccctggtcc     25380
     acatccttct ccatccacag gctctcagtg gtgcctccca tccacactga ccctacgtgg     25440
     gttcatatct tgtccaccac ttcctctctc ttttcttctg catcttgctt tttattccaa     25500
     cagcctgttt ccacctcacc tggacttatg tacaggaaag tgtttactga gcaggcttgt     25560
     gttgtgcaaa ccctgaagat tactgaaaaa gtgctttctc aagccacatc catattcagc     25620
     tcctgggaga tgatatctgc ttttttggca tactcctcct ggaaatattt ttaaaccatt     25680
     ttattaaggt aaggattaca tctataagac tatacgttat ttaacatact taattcattg     25740
     agttaagtat gcaaagttac catcaccatc aaggtcataa acatagcaat gacttccaaa     25800
     acattcctct ttattctttt tattcttagt attatgatta ttattattag ttgaaatgta     25860
     tttttgcttg tgtgtctctg tctctgtgtg tgtagcctgg gtgcacacta tgctagctgt     25920
     ttccataatg acttatgggg aatgcctatt attgctgaag gggccagcgt ctgagcactg     25980
     gaggtcagtt atgcaggtgc cacacacatc tgtgatgggc cccaataaac agcactgggc     26040
     accagcactc aggtgagctt ccctggtgga caatgcttca cacgtattgt catgcatcac     26100
     tgctgggaga actggggaca agagacttct cagggaaaga acacctggga gcttgtgcct     26160
     ggattctcag ggccttccca ctggttcctt tgctcatttt aattcatatt catttgctat     26220
     aataaagctg cacccacgag aataacagct tgtcttgagt cctttatttg tactgattat     26280
     ggggcctgag gatggtcttg ggacactcaa cacaattaca tcagaggtgt aaattgctag     26340
     aaagaccctg actactgaca catgtctata gcatttataa tatcaaagga tgagaaagtg     26400
     taggataaaa gacattcagt gcctggatgg ctatagaatc acatggcatg agatgatgtg     26460
     tgcactccaa taaggagcag gaagtgaaag taatccatgg aatttagaaa cgacggacac     26520
     agctccctag gagttgttcc ctgaacaaga acaagtaaaa ataatgaaga acaacctgag     26580
     tatgcaactc atatctgtga tagctaaaat aatagtaaaa agatgcacat ccagccctgc     26640
     agtgtgccct gtgagccaag gctgctagac tcccattcac tgccaaaggg tacagttgtc     26700
     cagaaacaac acacaacaca caagagctac acagatggta gcccagggtt tggactgggg     26760
     aggggaatcc acttgacaac tagaacaggg aggatagtgt ggaaagtggc atcattttgt     26820
     gatatgaatg atatcattaa tagatatcat cagatcgtga acaacattag ccaatggact     26880
     aggagactga ctgatgtagc agattctctt tggcttcatg tgttgcataa gggaggagca     26940
     tgtttgggaa gatgctggac ccacagctca caatagacac atcacagatg gctctagctg     27000
     atcccaacct caagtaggat gcttctgatg ggacagcttt cctcaagcac tggatcccga     27060
     tcatgtaaag tttctctacc ctgagaatga gactgccctt tctctcttta aaatgcaaag     27120
     tgggctctcc agatgaggag cccaatgctg tatatggaag ccatagtgct atgtgtttat     27180
     gttgataccg acggtcattg cttagcatgc atcttatcca ggtcctgctg ctgtgtgttt     27240
     ctgttgatgc tgctggacat tgcttcgcat gcaccttacc taggtcatgg cctgtgctgt     27300
     gataaagaga agtccttttg cttctgcacc ccaggtacaa ctcctgtgag caatctggca     27360
     aatattttag aaactgtgta gtttgtattg ccttgtcttt tcatttgaat cttttagatg     27420
     ataaggaaat gagagtggtt cacaagataa tggggcagac acaggggata gttaagggcc     27480
     tcctccaagc aatgtggata tcataaaatg aatgttaaaa aatgaaagaa ctaaacaaga     27540
     aggtgctggg gttgacacaa aggacttaaa acagaactac caggagttgg gtgcagcaat     27600
     ggcaatccct tcagttccat cagcaattca gagacctaaa caaatattcc ctatttatgt     27660
     tgaattggca gactgtagaa atgtagaaga caaaatgacc agaaaaaatg tgtgatttgg     27720
     catgggtgaa ggtctggcaa gttaatcaag ttgaggactg ttgaagccct agggtggtcc     27780
     tgcctttgct gggtgcccag accataatgg cactcatggg aaattgccag caagtagaaa     27840
     ctaggtgttt tttttttttt tgtagttctt attatcaggc atatgtgcat ttgaattttg     27900
     tcttcatagc atttcctggt tctatttctc attttttaaa acacacgtga tttcatctag     27960
     atttatcacc ttcacagggt cacagagaag ggtaggaaga agggaggccc tggtttgggt     28020
     cttgtaggac catggacaag agtgcggagg gacaacaggg ggttgtaaga cattattaag     28080
     accttattct gcctctccca agaggctcag ttcagccgtt tttctgcagt tgcggttctg     28140
     ggttataaac cttgtagact cttcccttca ggccagggcg gcaactatgc aaattcaagt     28200
     gggggcctcc ccacttaaac ccagggctct cctccacagt gagtctcctt caccacccag     28260
     ctgggatctc agtgctttct tttctgtcct cctccaggat ggggtcaacc gtcatccttt     28320
     ccctcgtcct ggctgttctc caaggtcagt cctgcccagg gtttgaggtc acagagaaga     28380
     atgggcagaa gggagcccct gatgcaaatt ttgtgtctcc cacacaggtg tctttgccga     28440
     ggtgcagctg ttgcagtctg cagcagaggt gaaaagaccc ggggagtctc tgaggatctc     28500
     ctgtaagact tctggataca gctttaccag ctactggatc cactgggtgc gccagatgcc     28560
     cgggaaagaa ctggagtgga tggggagcat ctatcctggg aactctgata ccagatacag     28620
     cccatccttc caaggccacg tcaccatctc agccgacagc tccagcagca ccgcctacct     28680
     gcagtggagc agcctgaagg cctcggacgc cgccatgtat tattgtgtga gagggaccat     28740
     ctaaaacctt ccgcggtgca ggtgcagagt gagctgccag acacaccctc cccaggggcc     28800
     tctctattca tccggggagg aaacactggc tgtttgtgtc ctcaggagca aaaaccagag     28860
     aacaacatgg gagcgttcct aacccctaag gcaactggat gggagacctg acccatccag     28920
     ttctctgagg gggctcttgt gtgttctaca aggttgttca tggtgtatat tacatggtta     28980
     acatcaaaag gctgcctaat aggcacctct tcaatataat agtcttttaa ttagtgaaaa     29040
     ttttacacag ttcatcattg cttgcttgcc ttcctccctt ctgtccgctc ttactccctc     29100
     cttcttttat tttctactta cttttccaaa atcatttaac ccctttttgt accattaata     29160
     agttatcttg tttttgttgt tgttttcctt ttaacaatat gcactgaata attcatcttt     29220
     gtgccaattc gtaaatattt tgatataaca aagacttctg tcataaatag atttcctgtg     29280
     agtaatcttg caaatatttt agaacctgtt tggttaagaa taaattaaaa taaatagaca     29340
     aatttttaaa gacatacaac ctatcaaaac tgaaccacca agaaataaaa aatctgggct     29400
     gggcgtggtt gctcatatgt gtattctcag cactttggga gaccgatgcg ggtggatcac     29460
     ctcaggtcag gagttcaaga tcagcctggc caacatggca aaaccccgtc tctactaaaa     29520
     atacacagat tagctgggtg tggtggtgtt cacccgcagt cccatagtcc cggctactcg     29580
     ggaggctgag gtaggagaat gacttgaacc tgggaggcag aggttgcaga aagccaaaat     29640
     cgagccactg cactccagac tgagcaacag agtgagactc caattaaaaa aaatctgaac     29700
     agaccaatga gtaaagagat cgagtcagtg atttttccaa catctcgaat caaagtccag     29760
     aacttcatgg cttcactact gaattttatc aaaaattaaa aaaaacctag aatctctact     29820
     caaatttcca acaaaataaa gagaaagaaa tatatccaag cttattttgg aaggccctat     29880
     ttccaaagca aggaaaagac actacaagta aataaaacta caggccaata tccctgatta     29940
     tcatagtttc aaaaactctc aagagtgatg agatgacaaa caaaattaaa cagcagatta     30000
     agaatagaat tcaccattat ctgtgtctcc catacccaga gaggctgctg cttgccctgc     30060
     acaaggatac tctgagcaga aacctacctg gcccagccct caccttgctt tatgcagcca     30120
     tctgccctgg cagcttaaca caaaggacag catcttttgg gagatacatg gccacaccca     30180
     ctgcctgaga atccacagta gcccctattc ctgggcaaca taagccaagc aaaaacccca     30240
     ctgttaatac tgtagctggt gctctttgga aagcaccatc tccaagcagg aagccaataa     30300
     acagtccatc acagtatctc ctggtagact atcactgcac ccagaaagga gaaaatgact     30360
     gtgtgatctc agctatgacc agtgcctgca ccactctgga tgaccaggag gtcctgagtc     30420
     tgtccatgtg aacagttcat taccgctata accagcattc aagaaagcca gtacacaaag     30480
     gctatcaata tctaaggaat ttcagtctcc atcactcccc tgctgcttcc atcagacctg     30540
     gttcactgct cactgctgaa aaattgagga cacatcacac caccggatcc ctcacataca     30600
     ttccccaaca ccagcctgaa gtgtgtcaac ttcactgggc ggctagacct agagaagcaa     30660
     caacactcac agtagtatgg ctcccaggtt ctcctgctcc taggggaagg gagtgcacca     30720
     cattgaggga acaccctgtg ggacaggaga atctggatgg caggccttga acatcaatca     30780
     gatccctcca ttggtgggaa acttctttta gtagaggcag ttacagtgct gagctcagca     30840
     gagaaagtct tcagctctgt ctcaacagtc agatatccct ggtgcatgtg aaaaacttga     30900
     agaagaggaa gtctttcccc cttgtacacc actgcaggca cacttggggt ctctcccata     30960
     agagctcagt acgggtgcaa ctatagacag catttctgga acacatcatg ttgactgcat     31020
     cccccagaaa gagcactctc caggttcagg ttttggccag acacagagtc acaattcctc     31080
     tgtatttaac acttcaactt ttgtacagag gaaaagacat gcctgtctga tctgaatagc     31140
     tggaatcctg ggatacggga gtgtcttaga ggtgcataac tttcctacag acctggcaag     31200
     acagctgagg tggctccaac ccttccccca gataagacct cagtgtgact cactaaaagc     31260
     tcctccagcc aactctgtca aggctgggac ttcatttacc acctgattta gccacagctg     31320
     gtttctatcc agggacaagc cccctactgg tctgaagcct gaaccatcaa actagcaaat     31380
     aaatcactgg gggaaaataa ataaatacgt gcatgccaca gaggaatgaa ctaaggttta     31440
     atagacctca accattctaa ccccatagaa gacagtgaac ttgctcacat actgagcaga     31500
     tttctactgc aatcaacaca taggaaagcc atcatacaaa gattctccat aaccacggaa     31560
     ctcttacaga gtcttcaccc ctctaaggac caaaaaccaa attagactgt aattaattat     31620
     aaccattaaa gtctcattct taagggaaaa aataaattta aaaacaaaca aaagaaacag     31680
     ttaaaacaaa cataatttca atactagtta gaagaaatag tctaccaaaa taagaggaaa     31740
     tgagaaaaat aactcaagta atatgacaaa acagggtgct ataacatccc aaaaaggtca     31800
     cactaggtct ccagcaataa actttaacca aaatgaagtc tttaaaatac cagataagaa     31860
     attcagaatg ttgattatta agctactcaa gaagatataa taagaaggtg aatatcatca     31920
     taaacaaatg tataaaagca gttcagaata taaatacaaa tttttataga gagatagata     31980
     tcataaagag aaaccaatca gaacttctga aaataaaaga caaaatgcag ggaaatagta     32040
     agggaattac aaaatgccat ggaaagtttt aaaaatagac tagaaaaaag tagaagaaaa     32100
     aataaacaag agactagaaa aagtacaaaa taaaaagttg aagtaaaaaa ttagatgaaa     32160
     aaagaagaag aataaagtag aaataagaaa aaagaagaaa taaaaaagac tatttttgaa     32220
     ataatcaaat cagagaaaaa taaatttaga aagaaccaaa agaagtaaac acagtctcca     32280
     ggaaatatgg gattatgtaa aatgaccaaa cctcagcata attggtgttc ctgaagaaga     32340
     aaaaaaataa tatgtttgga aaacttattt gagagaaaaa ttgagaaata cattcctggc     32400
     ctgcctagag acctagatat ccaaatccaa gaagttcaaa gaactcctgg gaaatgtatt     32460
     gcaaaactgc cttcaccaag cttatagtca tcaggctacc gaaagttaac atgaaggaaa     32520
     taattctaag agcagcaata caaaacaaaa caaaaaaccc atcagactaa tggcagactt     32580
     ctcagcagaa acttcaccag ccagatggat taagcttcta tcttcaaatt ccttaaatag     32640
     aacaactttc aatcaagaat tttgtatcct gccaaactgt ttgataaatg aaaaagaagt     32700
     aaagtcattt tcagaatcac cagtgctcag ggaattcgtc actaccaaac cagcagtaca     32760
     agaaatgcta aaaggagttg caaaccttga aacaaaagcc cagtaagcac ataaagggaa     32820
     cctcttaaga gcctgaaact cacaggccta taaaacaata acacagtgaa aaaaaaaaac     32880
     taggtaagaa ttaacatggc aaagaaaaca gtaactcata tctaaatatt cacattgaat     32940
     gtaaatggcc taaatgctcc atataaaaga tacacaatgg cagaatagat taaaaaatca     33000
     caatggaaat atctgatgtc tctaaaagat ccccctaaca cacaaagatt tacataaact     33060
     caaggtaaaa ggttggaaga aggtatttca caaaaatgga aaccaagagt gagcaggagt     33120
     agctattttg atatcagaca aaatggactt caaagcaaca acagtaaaaa aaagacatag     33180
     atgatcacta tacaattaca aaaggatcaa ttcaataaga aaatactaca attctaaatt     33240
     tataggcacc aaacaccaga ggaactagat tcataaaaca agtactatta gagatttaaa     33300
     aaaatgagag cgaaacaatc agagtgggag actttaatac aatcataaca gcactagaca     33360
     gatcttcgag actgaaagtc aacagagaaa aaatgcactt aaataactca ctgaaacaaa     33420
     tggatgtagc agatgtgtac agaacattct gtccaagatc tgcagaataa acattcttgt     33480
     aatcagtaca tgcaacattc tacaagatag aacatataat aggtcacaaa acaagtctca     33540
     atgaattttt aaaacagtga aatcatatca agtatcttct cagaccacag gagaataaaa     33600
     ctaaaaatga actctcaaag aaacccttac aactgaacaa atacatagaa attaagaaat     33660
     ctgctcctga gtaatatctg ggttaacaat ggcataagat gaaaatttaa aaattacctt     33720
     aattaaatgg taacaagtta ttgaaacctt aagaatacag caaaagcagt ggtaagagga     33780
     aagtttatag tgactactgc ctacgtgaaa aagccggaaa gagcacaagt tgtcaaccta     33840
     atgttgacac gagaaaaaag aaaaaattaa agaaagaaaa tataaatcaa aaaagagaaa     33900
     gaaacaagct gcctgtgtta ggctgactca cgtcaaaggc agtaacacgc aaagtttcaa     33960
     tgatgttatc taagggccgg agctcagagg aatgggctct gaagactctc ccagcactcc     34020
     ctcagcataa ggatgaaaag aggtaagttt tcctatctct cctttagtgt aagtgaactt     34080
     ccctgtcgaa tctcatcccc tctgctatgt aaactatacc ttgcctcttg ctctgtacgt     34140
     tttaggagtt tctgtttttc ctgtagttaa tgattgcagg ttcctgcttc tgcatctaag     34200
     cagtacagcc tgagcaggtc tggcttgtag ccagctacat gccatggtgg gggtggcagg     34260
     atgagtcttt gtgagactcc tttgaactaa gcaggtaatg accatctggg ctgcatggga     34320
     aggagtattg ctaaccctga gttacaagcc tggctttgtt tgattaacgg cctctgtctt     34380
     gcctctgtac gtgcgcgttc gtgccactta ggaggaggtg tataagcaaa accttgtctt     34440
     tgttcggggc ccagttgttt ggacgttgag tcccttggga cagagtgcac tcagtgaaga     34500
     ccctcctgct atgccccgag gtgtcccttg ccctcctgat gttccgcaac ctttcttgcg     34560
     agccagcagc caggaggaga agagaggaca gattggctgt ctcctttgcc ggagggtctg     34620
     gggctctggt cgcggcgatc tgtgacccca gggcggcctc ggggagcact tcagcccgga     34680
     ggggagaccg gctctccagg cccccggggc ccctcccctg acagcgcaaa ggagcgcgga     34740
     aagggctgcg ggatgatccc agaagcagcg ctaatgaacc gcggtcggct tttggggccc     34800
     aaggcaggac ccgtcccacg aggacggagg ggagccccat cacctgccgg gctgtgcgtc     34860
     tagtccgacc cagggaggct gcggcggcga gagggggtcg ctcactcggg taccgcagac     34920
     gccgcaaccg acgcggcaca ggagagaggc ccgccaggcc ggttaggaaa gggaactggg     34980
     gaggggaagt gggtgaaagg gcgtgcaaga gacggctgca ggaggggccg gtgcggggag     35040
     tgacgtggga ggcgcagatc tcagcgtgga ctgggcgctc ccagcgaagc ctggggccga     35100
     cctgggatag cggcgaagcg gctggtagga gctgccccgt ggcagcagcg gccgtgctgg     35160
     gaatagggga ctctcctagc caggcggccc cgaaaactcc tgtaatagga gacgctctgc     35220
     tcggtaaaaa aggaagtcag agtgagtgtg cgtcgtagag ggaggaaatg ggaggaaaat     35280
     cgtggaaacc cactccactg gaatgtatac taaagaattt taagaaaggt tttgcagcgg     35340
     attatggaat taagctgacc ccctgaagtt gaaaactctc tgtgaatcag aatggccttc     35400
     tttaggggtc ggctgccggc cgagggtgct atatagatag ggaggtgatt ggccgggtgt     35460
     ttaaggtggt gaccggggtg ggaggacagc cagggtaccc agatcaattt ccctatatcg     35520
     attcatggct caatgttata caaaactgac cagcgtggtt gcacccccca gcagcatatt     35580
     gtaagacgcc tgtggcccga gcagccgaag acaaaatgaa agtaaaatca gccatgctgg     35640
     cagctgcaga gaaaaaggga aagtcacagg aaagccgaga taaaccagtt ctgcgggcac     35700
     ctcaggagga aacagaattt cctcctcctc ctcatgttcc aagccacccc cctttgccga     35760
     gaccaactgc tccctctgca agttcagctg tgaggccagc agccccagct gtccatgggg     35820
     agttaaatcc gggagagtat actcccccag cctcaactaa gaaggaggaa ttggagctgc     35880
     aggaagttag agtggaaagc ccagaaagtc aggcaggcca tctcaggtct ggccgcacct     35940
     gagtcctgca aatgcgtctc agggagacga ggggacctgt ctattatgac cagcatggcc     36000
     acatccagag gggagagtga actttcgttt accagccctt ttcaaccact gacctcctaa     36060
     agtggaagca tcatattccc tcttacatgg agaagccaca ggctctcgtt gacttgatgc     36120
     agtccgtttt tcagacacac aatccaacgt gaccagattg aaaacagctt cttttgaccc     36180
     tgtttaatat ggaggaatgg cagagagtga cacaggcagc cctccagtgg ctaaaactaa     36240
     tgcacctgca gacacagtta atgcccagac atatgcatat ttgaccctgt ttaatatgga     36300
     ggaatggcag agggtgacac aggcagccct ccggtggcta aaactaatgc acctgcagac     36360
     acagttaatg cccaggcata tgcacgggtc agttccctga aacaaacccc aattgggacc     36420
     caaatgatgc aacccagttg cagcacctgc agcggtactg agaggcaatc tttcatgggc     36480
     tgaaggatgg tgggagaaaa gcagttaata taggaaaaat ctcggaggtg ctccagggag     36540
     cagttgagag cccaagccag ttgtattaga gactttgtga ggcttatcgg ctgtacaccc     36600
     tgttcaaccc tgagactact gagaatcagc atgtggtaaa tatgtcattt gtaggtcagg     36660
     cccagggtga tatcaggcaa aatttgcaga agatggaagg tttcgcgggt atgaatgcta     36720
     ctcagcttat tgaggtagcc accaaggtgt acattaacaa tgatcaggag gcaaagaggg     36780
     aggctgattg aaggcttaag aagaaggctg atttactggc agctgccctt acgggaaggg     36840
     aagctggttt tgcaagggga tgcgggtatg gatgagggca tggaagaggc cggactggat     36900
     ggggatttga gagtcagctg agactagaaa gagatcaatg tgtgcagtgc aaaaggaaag     36960
     gacactggaa gaatgagtgt ccagagaata acaaggagaa tggtcagggc tgtggtatgg     37020
     gaaggccacc agcagggggc tgccgcacct gggaggaacc agacactgac ctaatcggac     37080
     tggccagggc tgaaggatat gaggactagg acagaccggg ctctttctct ttgggcaccc     37140
     aagagcctat gttcagatta gaagtggagg ccaactagtg gactttatgg tagatactgg     37200
     ggctgagcac tcagcagtga cccgacctat agggccgcta tccaagaact gtgcaactat     37260
     tgcaggagcc actggagtct cagagaagag gcctttctgt cagtcaaaga ggcgtgttat     37320
     aggaggacaa ggagtccaac aagaattcct gtacctccca aattgtccag ttccttcgct     37380
     ggaaagagac ccatttcaaa aactgcaggc acagattaca tttgggccac aaggggatgt     37440
     aactttaaat ctagctcact cagaggctat ggtgttaacc cttactgtcc cacgggctga     37500
     ggaatggaga ctatatgcag gaaagactcc agaaccagga gtaaattaaa tgcgtgggtt     37560
     acttactaaa attactggag tttgggctga aagtaacctg ccggaactgg cagtaaatcc     37620
     ggcaccaatt gtagtggagt taaaactggg agcgactctg gttcatgtcc gtcagtaccc     37680
     gcttctccct gaggccatac gggagtccac aaacacttag attgacttca caggtgagga     37740
     accatagtca aatgccagtc actatggaac acccctctct tgccagtgtg gaagccgtct     37800
     ggagaatata ggccagtgca ggacttgtgt gcagtaaacc aggccacggt gaccatccac     37860
     ccagtgctgc caaacctgta tactttaatg ggacatattc cagtgagtgc cacttggttt     37920
     acagtcctag atttgaagga cacatttttc tgtctccagc tggcaccgat tagtcagcct     37980
     gtttttgctt tgcaatgggg tgaatcacag tatacttgaa caagactgcc acagggattt     38040
     aagactctcc cacaatcttt gaagaggcac tggcctcaga ccttaaagcc tacaccccac     38100
     caagcagtga ctgtgtcttg ctctagtaca ttgatgatct tcttctagca gccccaacct     38160
     ggaaggactg ctttcaggag acccaagacc tcctgcacct gttatggaag gcaggatata     38220
     aaggcaggat ataaagtgtc agggaaaaag gatcaaatct gctctgaaag tgtccagtat     38280
     ttaggcttct atataagcga agggaaaaga ttgcttggta gtgaatgaaa gcaggctttt     38340
     gtgtgcttcc cactccaacc acctggtgac aaatgagaga gttactaaag gcagctgggt     38400
     tctgccacat ctggatccca tgtttctcac tcatgggtaa gccattgtat gaagccacaa     38460
     agaggggaaa gaaggagccc ctcctctggg aggccaccca ggaaaaggcc ttttaataaa     38520
     tcaaaggaga cttgactgaa gctccagctt tagggttgcc agatctaact aagcttttct     38580
     ttttatatgc ccatgagtga aaaggaatgg tcataggggt cctaactcaa gtcatagggt     38640
     catggcacca cccggtggca tatttatcca ggcaattaga tactgtggca cttgcatgga     38700
     cccttgttta aggcattagc tgctactgct tcactggcac aggaagctaa taaactgact     38760
     ttgggacggc aactgataat ccaggtacca tactcggtta taactttaat ggacaaaagg     38820
     gggcaccaat ggttataaaa tccgagaatg atttgatatc aagagctctt atgtgaaaat     38880
     ccctgcataa ctttagagac tgtaaatacc ctaaacctgg ctaccttgcc cccttcattg     38940
     ctgtgtggac gtagcagatg aagtgttctc aagccagaga gatttgacag atcggcccct     39000
     cggggacccg gacattgaat atttcactga tgggagcagc ttcatcctag aggggcccgc     39060
     cgagcagggt atgcagtagt gactttggac tcagtagcag aggcatagtc tttgcctaca     39120
     ggaacttctg ctcagaaagc agagctaata gctctgacaa gggctcattg gctagcaaaa     39180
     gaccaaaaga ccaatattca cacaaactct aaatatgctt ttgccatttt gaatgttcat     39240
     ggggctattt acaaagaaag aggactcttg actgatggag ggaaagaaag aaagtacaag     39300
     gaagaaattc tacagccctt agatgctgtg tgggccccga agcaggtggc tgtaatgcac     39360
     tgtagggggc acctgagggc aggaacatca gaggtgaaag gaaacagaaa ggcagacaag     39420
     aggcaaagca gactgcagtg gtgactctgc cttctaaaga ggaagccttg gctatgcctc     39480
     tccttccaga aattcccctc ccagagaccc caagctacac tccaaatgaa agggcttttt     39540
     ttgcccaaga aaatgggagc tacgttgaag gaggatggtg gaaattctcc aatgggaggc     39600
     tagccataac tgaaatggtg gcccccagat ttgtaaagca gttccaccaa gggactcaca     39660
     tgggaaaaac ggcactagag acattattag gttgtcactt ctatgtgtcc cagcttacgg     39720
     ctatcacttg agcagtttgt gaacggtgtt taacttgtgc tcataacaac cctcgacaag     39780
     ggcccacttg accccccagg aattcaggaa acaagagcca tccctgtgaa aacctgctta     39840
     tgggcttcac caaactgtcc tgagcagggg gctatcagta catgttggtg cttgtctgca     39900
     ccttttcagg atgggtcgag gccttctcca cccagacaaa gaaggcatga gaggcgacca     39960
     aggtattgtt aagagacgtt attcccagat ttggactacc cctgactctg gggttagaca     40020
     atggactggc atttgtggct gaagtagtgc aggacttaac tcgactatta ggaataaaat     40080
     ggaaattata tacagcctgt aggctgcaga gctcaggtaa agtggagcac atgaactgga     40140
     cactaaaaca gctactgaag aaattttgtg aggaaactca cctgaggtgg gatcaggtct     40200
     tacccgtggt cctcctccaa gtcgggtgca cccgcacaaa acaaaccggg tgttcaccct     40260
     atgagatttt attcggccgg ccacccccaa tcacaggtca aattaaaggt gatctctgtg     40320
     agttaggaga actctaagga agcaaatgca ggctttaggg ctagctatgc agaaagtgca     40380
     tggctgggtg caagaaagaa tgcccattgg tctgacagac ccagtacacc ctttcaaacc     40440
     tggggacctc gttggggtta agaaatggaa tcataactct gggacccata tgtgatgggc     40500
     cccatgttgt aattttgtcc actcccactg ctgttaaagt tcagaaatcg tgccttggat     40560
     ccaccacgac tggctgaaac cggcagccca ggaccagagg accagctagc aggacccaga     40620
     ccatccgacc cagctgaccc tgcgacgaga ctgggttccc agcggagagg acaacagccc     40680
     ggctctgctt acttcggaag ctgaccagtc tatgcatggt ggaagcttga gaaaacagca     40740
     agctctgctc tagtcactct ggaagctgac tagtttacgc atggcagaaa gcttgagtcg     40800
     tcatcatcag atgagtaaat gtggccagag atcttaggtc cagtaatctt atttgtatta     40860
     ttaattatat tactgctgtg ctattgcttc aacaaatctc ctccaccctg cccatgctag     40920
     gtgtaagaat gctacacctt gccttgtttc tgtcgatccc catatctgtc ctaggagagg     40980
     aagagcctat agaggaatgt ccacattgca tgtgtactac gtggataggt aatacactag     41040
     ttaagacttt attgtaccat acatactatg aacgcacagg aacccctctg gggacttgta     41100
     tgtacaacca aacgaactat tcagtttgtg acccagggga tagctagctt tatgtatact     41160
     atgaccctaa gcttttacct gggcctggtt tgagattcgc actgggtcaa gggaaggaag     41220
     tcttttaaac aagaccgaag tttctctctc ccacaaggga gtcacatctt tgtattttga     41280
     tatttgccag ataacatcca tagactcact ctttcccgta atccacagtt ccatggagta     41340
     ctataatagc tgccataaaa gtaagtgtgc atcccctgct tgctccaccg gttccctggt     41400
     aacaacttgc tggggctttg caacatggcc tactaaccaa caatcactag ggctaatcat     41460
     gcttaccaaa ataccttcag aaccagattg taaaactaac atttgcaatc ctgtgaatct     41520
     taccaccttg gagccaaatt ggctcgtatg gactacaggt ttaaaagcac cactgagggt     41580
     acaagccagc agccaagaaa tagactcaag agtctatgtc tatattatat acataaaaaa     41640
     aaacctgaat ccttcttgcc cagcagcagt tccaagtctt taagtcattc aatgagcact     41700
     ttaagcagga ggtacctgaa gctcctcctc ctatagctgt agatctgttt gcacggttgg     41760
     ctgaaaacag ctggcagctt aggtgtttcc tcatgctttg tctgtggagg aacaaatatg     41820
     ggaaaccagt ggccttggga aacaaaagag ctatcgcctc aagaaaatta cactttaacc     41880
     ctttcatccc cagagccggc actcacgagc tcaagtgtct ggctgctaaa atcttctgtc     41940
     attggaagat tctgcattgc ccactgggga aaagccttta caaacccagt aggggaatta     42000
     gtctgcctag gacagcaata ttacaatgag acattaggga aaactttatg gcggggcaat     42060
     aataagcaag gtaaagatac aaccaggttg ccatgccata atccattttc ccattttcac     42120
     tctttaaacc actcctggta tcaactagag gcaccaaatg aacacctagc aagcaccttc     42180
     tggcctcgac tggatctgtg ggccatgagc ttattggcaa ttacctgcca attggacagg     42240
     ggcctgtgtg ttggggacaa taagaccatc tttcttcctt gacccgttac aacagggaga     42300
     ggttttaggg tatcctatct atgatgaaat taaggggagg agcaaaagga atatagacat     42360
     aaaaaaagat ataaagatag gagattggaa ggacaccgat tggcctcctg aaagaataat     42420
     ttaatactat gggccagcta catgggcaca agatgggatg cagggatact gtacccctat     42480
     ttacatgctc aacagcatca taaggttgca ggcagtactt gaaatcatca ccaatgaaac     42540
     tgcaaatgca ttagacctgc tggcccagca agccacaaag atgagaaatg ccatttatca     42600
     aaataggcta ggcttggact acctcctagc ccaggaagga ggcataggcg ggaagtttaa     42660
     tctgacaaat tgttgtttag aaattgatga taacagaaag gccatcatgg aaataactgc     42720
     aagaatgcaa aagctagctc atgtcccagt tcagacctgg aagccaatat ggtctctaga     42780
     ttcccttttt ggaggttggt tttcaggttt tggcagtttt aaaacattaa tagggatagt     42840
     actagctata ttaggaggct gtttaatact cccctgcctc ttacccctcc ttatcagggg     42900
     catccaatca actatagacg ccgtagttga caaaaccatc accacttgac taatggctct     42960
     aactaaatac caagctgtac caaacgaaga aaacttgcct tatcatgaag aacaaagttg     43020
     tagtgatgac ttctattaaa atttcataca taggaggcat caaaggcagg gtatgagatg     43080
     ggaaaaaaga aaaaaattaa gaaaaaaaga aaatagaaat taaaaaagag aaagaaacaa     43140
     gctgcctgtg ttaggctgac tcatgtcaaa ggcagtaaca cgcaaagttt caataatgta     43200
     atttaagggc cagagctcag aggaatgtgc tctgaagact ctcccagcac tccctcaaca     43260
     taaggatgtg aagagataag ttttcctatc tctcctttag tgtaagtaaa cttccccgtc     43320
     gaatctcatc ccctctgcta tgtaaactat atcttgctcc ttgctctgta agtttttgga     43380
     gtttctgttt ttcctacagt taatgattgt aggttcctgc ttctgcatct aagcagtata     43440
     gcctgagcag gtctggctag tagccagcta gacgccatag tgggggtcgc aagatgagtc     43500
     tttgtgagac tcctttgaac taagcagata atgaccatct gggcagcata gtaaggagta     43560
     tactaaactt gagttataag cctgtcttag tttgtttaac ggcctttgtc ttgcctctgt     43620
     acattcgcgt ttgcaccact taggagtagg tatataagca aaaccttgtc tttgttcggg     43680
     gcccagtttt ttggacgttg agtccactga gacagagtgc actcaataaa ggatcctcct     43740
     gctataccct gaggtctccc ttgccctcct aaatttctgc aacaatgtca cacctcaaga     43800
     aactggagaa ataagaacaa actaaaccta gatccagaag aagaaaagaa ataacaaaga     43860
     tcagagcaga actaaatgaa aatcaaacaa aaaacacaaa aaaccaatga aataaaaagt     43920
     tggttatttg aaaaaataat caaattcatg gaccattagc tatattaacc aagaaaagaa     43980
     gagcaaagat ccagataagt tcaattagaa atgaaatgag acaatacaat ctacactgct     44040
     aatatataaa ataatttgag actactatgt acaacttcat gtgcacaatc taggagactt     44100
     acaggaaatg gaaaaattcc tagaaacata caactctcct agattaaatc aagaagaaac     44160
     agttactttg aatagatgaa taataaacaa tgagattgaa tcagtaattc aagaattgcc     44220
     aacaatacca acaacaaaat agggccaggt gaattcacat ttgaagtcta cacaaaattc     44280
     aaagaaaaat tgctaccatt ttttctgaaa ctatttttta aatttagaaa aaaagaatcc     44340
     tccctaaatt attctctaaa gctactataa cccagatacc caaaccagga aaacacacac     44400
     acacacacac acacacacac acacacacac tctctacata cgaattttcc tggtaaatat     44460
     agatgcaaaa atactcaaaa aatagctacc tgagtccaac agcacatcca aaagataatt     44520
     catcatgata aaagtaggtt tcaactcaga aatggaagga ttatttgaac atacacaagt     44580
     caataaatgt aacacatcac ataaacagaa ctaaaaacaa aaactatagg attatctcaa     44640
     tagatgcaga aaaagcattt aacaaaattc agcgttttta attataaaaa ttctaagtca     44700
     ggtaggcata gaaggaactg acctcaaact aataaaggtt atataggaaa aactcacacc     44760
     caacatcatg ctgaatgtga aaaagttaaa agcattcccc ctgggaacag aaacaataga     44820
     agaatgtcca tgttcaccag ttctattcaa catagtgcta gaagttctac ccagagcaat     44880
     taatcagaag aaaaaaataa agggcatcca tattgagaaa gagaaagtca aactatcact     44940
     gtttacagat gaagtgatta tatacctaga aaaccctaaa ctcccccaaa atactgttag     45000
     ttttagtaaa tgaattcact tatgtctcag gttacaaaat aaatgtacac aaattggtag     45060
     cactgctata catcaacaac aacaaagctg cgaatcaaat caagaactcc atttctttta     45120
     cagaagctac aaaaagatat ttaggaatat acttaaccaa aaaggtaaaa gatttctaca     45180
     aggagaacta caaaacactg ctgaaagaaa tcatagatga cacaaacaaa tggaaatgaa     45240
     tcccatgctc atggattgga agaagcaata ttgtgaacat gaccgtaatg cccaaagcaa     45300
     tctacagatt cagtgcaatt cttatgaaaa tacccacatt attttttaca gaattaaaaa     45360
     ataataatgt taaaattcat atgaaaccaa aaaagcccaa atagccaaat aaatcctaat     45420
     aagaaaaaaa tggagccatc acattactga acttcaaatt ataccacaag gctgcagtta     45480
     cctaaacaac atggtactga tataaagtag gttcatagac cactggaaca aaatagagaa     45540
     cccagaaata aagccaaata tgtaaagcca actgatgttt tacaaaacat acaaaaatat     45600
     aaattggaaa atagacacca tatttaataa atggtactgg gaaaactagg aagccacatg     45660
     taaaagaata agactggatc tctatctgtc accatataaa aaacaactcc cattcaaaac     45720
     ttcaaagaaa tgcatttaca taaattggaa aaaaactctt tataatttgt atggctgggg     45780
     tgattgaccc aggctatcaa gatggaatca tactactact ccacaatgga ggtaagaaat     45840
     agtatgtttg gaatacaggg ggatttctta gggcatctct tagcattact ttgccatgtg     45900
     attaaggtca atgggaaact acaacaacct aatccaggca ggcatacaga tggctcagat     45960
     tcttcaagag taaaagttta ggtcactcca ccaggtaaac aaacaaacaa atgaacaaga     46020
     aacaaaactg accagccaag gtaactgctg aaggccaaaa aatacagaat gggtagatac     46080
     aagaaagttg ttatcaatac caggtatgac catcaaccag aaatgaggac tggaattgtc     46140
     atgagtatgt ctttcttatc ttcttaagaa tatatttctg catgtatata actgtactaa     46200
     gaaaatgttt tctttttgtt tttcatgtaa cataagattt atcgacttca tattagcatt     46260
     tgagagttgt taactgtata tagtggcatt taggttaagg attagtgcat tttcagttgt     46320
     atgaaggata gctgtattat gttaggcata attatgacct tactattgtc tttatttgga     46380
     gatcgagcat gatttcaggt gatatgtagg gatgctaagt tgacaacaag ggattggcct     46440
     gtgatagtta atattaggtt taacttgatt ggattaaagc atgcttagat ggctgggaaa     46500
     gtattgtttc tttgtgtgtc tcttgggttt tgccagagga ggttggcatt tgactaagta     46560
     gcctgcaaga gaaagaccca gccccaatat ggaagggcac catccactca gctgccagca     46620
     cagctagaat aaagcaggtg gaagaagagg agataacatt gcttgctgcg ccttctgttt     46680
     ttgttttgtt ttgctttgtt ttccttgctg gctgctttct tctgctcttc ctacccttgg     46740
     acataagatt tcacgttctt tggcagttga actatggggc gtgaagcagg ggcttgctga     46800
     gggttcttgg gcccttggtc agagaatgaa gtgtgcactg ttggcttttc tggttttgag     46860
     acttctggac ttagactgag ccactaccag tttctctctc ttcccaagct tttagaaagc     46920
     ctatcatgga actttcattt tgtaattttg tgagaaaatt ctaataaact atatttccta     46980
     tatacatatg ttatattagt tctatttctc tagagaactc tgggtaatac agctttgata     47040
     caaaaaacaa cattgttgta ttcacaaaaa tgtatcctaa gataagccaa cacataaaag     47100
     tgcataaagt atgattctat ttctaggaat tataaaaaat gaaaatgaat ctaaagtaac     47160
     aaaaaaagat cagtagttgc ctaaggacag attggaaaat tatatttttc caaatgctac     47220
     aaatgatact ctcacctact atgagcattg ctctctcctc aggaatttca cccaagaacc     47280
     gactctatag tcggagacat gcaaacaggt tcctgttcat gtacgaactc ttcctaacac     47340
     tttcttttca tgtacttcct tgacttattt gttgatgaag gttgatacca taaaaataac     47400
     taaaccaggc tcacattaga gaaaaagaag agagtgatgg aaagattaag ccggagcatc     47460
     cagtttcagt gattctttga ccctgccctc cctgaagtct agaggcagag ggatgagccc     47520
     tgctgagcag ttcacacttg tccacaggga gaaagaggtg taaatgtgcc ccccatccat     47580
     gctcataaaa tggcccatgg acagcttatc ccctgtccat gaaggggatg ccacctgatg     47640
     tcggtgccct tcctcagtgt ccacacctta gggtctctgc taatctgggg ttctcttctt     47700
     attctcagag gtagggtccc tgtggatcaa gcctagctgt ggctgcttct cagcctgttc     47760
     cctctgtgtt tgtaagagtc ctacaagaag ttcactggtg gaatctggaa gagaaatgat     47820
     acagcctaac tgttctcaga gcctttttta caaagcctct gaatttacct tcattgactc     47880
     tagcatgagc tcagcccagc aggcttcagg acaggggttg gtgtgggagg caaaagcgag     47940
     ggattcaagt ggaatttgtt agtggtaccc tccatcaata caaagaaaaa tcataatcct     48000
     cagggacacc cttgtcagca cagtctccct caaaatgacc aacctgagag ccgaggagaa     48060
     ggccatgtat tactgtgaga gacacaggag atatccgtgt ggcaacctaa cacaggggac     48120
     acctgtattt gtgtctgagc ccagacacaa acctccctgc agggagacag gaggggaccg     48180
     tgtgacagac actgctcaga accaccaagg ggagctcaga accagcagga ggcagtgggg     48240
     acacaaagga agactcagga gccaccaggg ggcgctcaga accacctggg ggtggtgagg     48300
     acaccagggg gcgctgagga cacaagggga ggatcaggac accagggtcg gctcaggacg     48360
     cgaggaggtg ctcaggacac cagggtctgc tcagaaccac cagggggcac tcaggaacca     48420
     ccaggagagg ctcagccacc accagggggc actcaggaca tcaggagctg ctcagaatca     48480
     ccagacggcg gctgggcgcg gtggctcacg tctgtaatcc cagcactttg ggaggccgag     48540
     gcgggcggat cacgaggtca ggagatcgag accatcccgg ctaaaacggt gaaaccccgt     48600
     ctctactaaa aatacaaaaa attagccagg cgtagtggcg ggcgcctgta gtcccagcta     48660
     cttgggaggc tgaggcagga gaatggcgtg aacccgggtg gcggagcttg cagtgagccg     48720
     agatcccgcc actgcactcc agcctggggt gacagagcga gactccgtct caaaaaaaaa     48780
     aaaaaaaaaa aaaagaatca ccagacggcg cacaggacat gaggtggcgc acaggacatg     48840
     aggtggcact caggacacaa gggggagctc agaaccacct gggggtggtg aggacaccag     48900
     gggatgctga ggacacaagg ggaggatcag gacaccaggg tctgctcagc aaccaccagg     48960
     gggcgctcag gacatcagga gctgctcaga atcaccagac gtcgctcagg acatgaggtg     49020
     gcactcagga cacaaggggg agctcagaac caccaggggg cgctcaggac atgaggtggc     49080
     gctcaggaca caagggggag ctcagaacca acagggggcg ctcgggacac tagggggtgc     49140
     taagaacacc aaggggcggt caggacatca gggttctctt aggaagcagt gcatcatcag     49200
     gtccctgggg agggtgggtt tctttattta ggccttgtga ttcctgacct ggtaaagtga     49260
     aactcttctc agcatctttt gccatttctt ccttggaatc catgtttctc ttacctacaa     49320
     aatcctaact tagaacagga attcaattaa acttttaatt ctttatattt ttgtaataat     49380
     actagtaata atatctcagt acaattttaa aatgaagaaa ttgtgtatgc ttacttcaaa     49440
     ctatttacac agatttttgt tactgtgctg tgtcagtcac acttttgtaa atgttttcta     49500
     acaataactc agcatatgca tggtgttgag tttctttttc ttccatttgc tgattgtgaa     49560
     gatgaaaccc cactttaagg gctcctgtcc tccactttgg tggcccctgg tgttctgccc     49620
     ctcagtctgt ttctccacct tcccttcttc tttccaaacc ttttataatc ctcaatttcc     49680
     attcaaggaa tcagcaagcc cgtttattat gtctcatcca tgtctggtga atcagttcac     49740
     ttccctttat gatcactgaa gccaaccaag tataggcagg caacagttct aagaatatgc     49800
     tcatctacct gcagactctc tgccctcctc acccctttca acggtcctgc agacatcacc     49860
     ccaccccatc ctctatcttc cctaagtacc acagagtggg ctctgcagct cctgctgccc     49920
     actgtgtgct cagccctggg gctcactagt gctttgatga taaagcctaa atccctaacg     49980
     tgtttgcaca cttcctgacc accctctagc aagttgtcat tgtgagtgaa tcctgggaac     50040
     cctgtgctga tattaattct atatttatgg atattctcta gcatccagga atattgtcaa     50100
     ataacctatc gagttgatgt taaaaattca tgtgaagcca ttgcaaaaga aaaaagtctt     50160
     taaactggaa attgctattt tttaatactt aatatttttt taatttaata tttaaatgct     50220
     atttaaatta gaaaaaggtg atgccttttg taaagtacaa tgttatttca accaatcgtg     50280
     taataaaatt ttaatagaga tgcttgcctt attgattatt atatttattt ataacattaa     50340
     atgcattaaa acatttattt aaaaaatatg ctattagtaa aaatgggaaa taagctagca     50400
     cagagtatgt aaagaatacc attgtatttt tcaatgatag atttgtcaat aatggcagtt     50460
     acagtaagtt ttattaaaac atgcatttac atatatttct taatttacat tggaatagac     50520
     agacttactc atagaagaat gttactttgg actaaaacct taaactataa atacaaataa     50580
     atccatataa attaaatata ataacacaga aacaaatgta tgtcactttg atgtttggaa     50640
     atgcatttat ttttgcctgt gcacatatat gtttcattgc tgggtgtgtg tgtctgtgtc     50700
     tgtatatatg ggtgtgactg tgaagtttca aaatacatta ttaacttgta tagttatata     50760
     aatctaagtt tttttatgtt aaaagatagt aaaaaaacac acacaaaaat attctagaga     50820
     gcatttgcaa taactaagga gtattcaggt gttgtattaa taatgaaaaa ctagccaaaa     50880
     tgtttttaaa cttttatttt aggttcagaa gtacttgtgc aagtgttgtt ttataggcaa     50940
     attgtgagtt ctgttggttt gatgtacaga ttattttgtc accaaggaaa taagcatagt     51000
     acctggtagg tagtttttca attctcaccc tcctcccacc ctccacactc aagtaggacc     51060
     tagtgtctgt tgttttcttt gtgtcaatat gattagctcc cacttataca tgagaacaga     51120
     tggtttgtaa ttttctgtta ctgtgttact ttccttagga taatgacctc cagctccatc     51180
     tatgttgctg caaataacat gatcttattc ttcttatgac tgtgttgtat tccattctga     51240
     aatatacata aatcagtaaa tgtgattcat cacacacaca caatcatctc attgatcaat     51300
     attaactttc aatgtaaatc tcccaggaat ctttcatctt ctcttgaccc tcccctctcc     51360
     tcaggcatcc caaaccatag ctttctatat attaggatac atgaaaatag ggccctctct     51420
     cagctgatga aaaccagccc agccctgact ctgcagctct gggagacaag ccccagccgt     51480
     gcagtaccaa ggtgcttcca ttcagtgatt agcactgaac actgaagaca tcccatggag     51540
     ttaggaatgc gctgggtttt ctttgttgtt gttctaaaag gcaatcaaga agaactagaa     51600
     atattgtctg tgagtgatat gagtgagaga aacagtggat ctgtgtggca gtttctgaca     51660
     agaatgtctc tgtgtttgca ggtgtccaat gtgaagtgta gctggtagag tctgggggag     51720
     gcttggtaca gcttgggggg tccatgtgac tctcctgtgc agcctctgga tacactttca     51780
     gtaactatgg catgcactag gtggaccaag ctccagggaa gggactggag tgggtcttac     51840
     attaatgcta gtggtggagg catatactac tcagactctg tgaagggccg gttgaccatc     51900
     tccagagaaa acaccaagaa ctcactgtat ctgcaaataa acagtttcat tgctgacacc     51960
     atggccgtct attactgtaa gagacacagt gaggagaagt cagtgtgagc ccagtcacaa     52020
     acctcctaca ggaacgctgg gaggaaaatc agctacaggg ctcactcaag gcccactgat     52080
     cagagtccac tccagaggga ggttcacatg gaggttaggg gctggttttc tgtcaggaca     52140
     ttggacttca tcatcttctt ccattacttt ctttagggag cctcttcatt ttagaattct     52200
     gtgtgtacta ttgttatata tacatatata tatatatata tatataaaat aatcatagta     52260
     atgcgaggac atattttctc atgcacaaaa tgtagattga cacttacaga gaagaaaaat     52320
     tatcaaccaa tgtcaaaaac atcagagtcc tggaaaatct taagtgttct ggtgggtcat     52380
     ctctaatcag acccagaaca gtaacctcag tgaaattcca tgattcagat actatttttg     52440
     gattctgatc ataaccaaga cagagtctgg accagggtca gtgtcatata gaacctcaca     52500
     gcttccatat atgacccttc tcttgacagt gaaatatgaa attcagtatc agcagtgatt     52560
     tgatttttct tttgctttcc gtattctttc tttttagttg ttgttgttta gttatttctt     52620
     ttgctatatc cattttcttt ttttttagtt gtgcctttca cttttggctt tcacttttcc     52680
     atttgctttt cctgcttcct gataaaggga gatgtaatcc ctgtggtcta aattccaggg     52740
     ctcaagcctt ttcctgggag ctcaggtggg tctcaggatg tggctcctgc agccacgtga     52800
     aagaggctga tgggactttc tttccttctc atgactcagg cattccacgg tgttgtgtgg     52860
     agactcatct gagattgcac gtggccatta gtagtgaagg ggatgagatg tgggtgtgga     52920
     ctgatcctgt gctgtgcaaa ttgtcattag tagtgaaggg gatgagatgt ggatgtggac     52980
     tgatcctgtg ctgtgcaaat tgtcattagt agtgaagggg atgagatgtg ggtgtggact     53040
     gatcctgtgc tgtgcaaatt gtcattagta gtgaagggga tgagatgtgg gtgtggactg     53100
     atcctgtgct gtgcaaattg tcattagtag tgaaggggat gagatgtggg tgtggactga     53160
     tcctgtgctg tgcaaattgt cattagtagt gaaggggatg agatgtgggt gtggactgat     53220
     cctgtgctgt gcaaattgtc atggagtcac ttttctcacc atagtgttag aaagagactg     53280
     taaaagttgt cagaatcaaa ctgcaacaat ttgtgttaaa acctgggaaa tggagctggg     53340
     aaggccatga gggagggttc tcatacacat gccttccata acaagaactg tcataaatag     53400
     attctgcaaa accacaacct tgcacaaagg ccagcacaac cttaaaataa ttacttctcc     53460
     aagtacatct gcccagaggt gcctgtgcaa tattacactg atgccaccct ggttattgat     53520
     cattttatcc caggttaagt gtctcaaaac cgcatatgtg gccctcctca ttttttcttt     53580
     aaaacacttt ccttatttac ctaccgaaat gtgcccgtgc acattcccat tgcaatgcta     53640
     attctcaaat aaataccact ttactttaga gtttctttct gtctgatatt caggtttgac     53700
     aagctgcaga tggacttgcc aattttctgt gggatacagg gtataataat tatgagagat     53760
     gtgtaggcat gttcaatgtt cataagagca accgtaaata aatgcaggct ggaagtctca     53820
     gagagagctg aatgtgctga aacaccatac tattttatct tctcccattc ttttctgatt     53880
     ggaatcagac ccaaccaggt tatcaatgcc aatctaccta agctagagcc aactgattac     53940
     ggtatcaaca gcaacaatag ctttttattt tctgcttggc tttgtattgt taccttgttc     54000
     catcctctag agtatctatt tcagcccttc ggataccggt ttcagaggct ttcccctaaa     54060
     gtgtaatttc ttagttttgt agtggtgata agggaggagg tgggtctgga tgtgttttag     54120
     aagttcaggc tgtcattctc tcccagacag acactttagt acagaacgat ttatgtggtt     54180
     gtcccatttc tacaggaaag atattcaaga gaataagaaa gctcttcagt atttggtccc     54240
     tgagaatttt acactagagc acattcacca ctcttgactt taagcaacct aattaatgta     54300
     tttgaagcct tatcttgaaa tagactatat ttcatatggc agactctgcc tcaggtaatc     54360
     tcataccctg actttgttac tctctacaag aacttgtctc tccctaaatt tcagatttgt     54420
     tgtttgtctt aaaatttcga ttatctgaag tatgaaagac aatttgccaa gtttcttatt     54480
     ttctgttcat ctgttattgt tgatgttgtt gttgtaaaat acaaaaatat atatatttct     54540
     tatctaagta cttctgcaag ctgagtagca gatatttttg gtaaaaccca gaataacaga     54600
     agagtccaca tattaattag cttcttaata ggaaaaatca ttatagtaaa tttgttcact     54660
     ggactgttac ccatcattta taatgtacaa atatctgata cacacaacaa actaaaatat     54720
     ctaggtattt atgctgagta aaataagcca aaaaaataag tatatatgct atattattcc     54780
     atttctataa attctggaaa atgaaaacta acctacaata atttaatgaa gaacagttgt     54840
     tgcctgagga aatggtagaa gaagggaaag ggataggagg aaatgtggaa caggagaaaa     54900
     aattgaggcg aattcacttg tccactttct tgataatggt gatagttatg tcatgtttat     54960
     caatgtatac tttaagtata tgaagtttat tatctctcaa ttagacctta taaagtttat     55020
     tacaagcaaa caaataaaaa attagacaaa aagaactgat agaaaatggg taacatatgt     55080
     taaatgccag ttacctgaaa atttatctgc gtgaacctta gttctctcca taattttagg     55140
     tgaacgctgg agtgtaacag agtcacaata ttttcattaa ggcagtaagt tctgcaagcc     55200
     acactagaca tgtcccgctc tgtcctggag ttagttcagg gaacacagtg atgaggaaca     55260
     cgggctcaga taccggaact cactcatctc agacatgagc acttagacac atacataggc     55320
     actctgtgtg tgggtttact ttcacatccg tacatggaga aacattgagt ctcacagaca     55380
     taatttacac acatatttaa aatataataa ggtgtttgat gctgtttatc acagcacaat     55440
     ttcctcgtaa aagtgcaaat cttctaaata ccataagtta ccaaactcct gcaggacacc     55500
     atcatcctct ctcggcaatg ccttctcctc acgtgtccca ccctagagtt ttttatgtag     55560
     ttggaaatat gcaaaggacc ctccttcttt ggtgaaaacc agccctgccc tcaccctaca     55620
     gttctgggat gggagaccca gccctggaat ttcaaggttt ttccacatgg tgattagcac     55680
     tgaacagaga actcaccatg gggtttgtgc tgacttggga tttacttgtt actattttaa     55740
     aagtagattc agggtgaact agagattttg agtgtgagtg gataacagtt aaggaaacac     55800
     tgaaaatatg tgccagtttc tgaccaggat gcccctgtgt ttttgggtgt ccagtgtgag     55860
     gcgcagttgt tggagcctgg gggagacttg gtacagcctg gcgcggtggg ggtgggggtc     55920
     cctgagactg tcctgtgcag cctctggatt tgccttcagt agccactgga tgcactgggc     55980
     ctgccaggct ccagggaaca gactggagtg ggtctcatgt attagtactg atgggagtag     56040
     catacgctat gcacattctg ggaagttctg attccccatc tccagaaaca atgccaagaa     56100
     cacactctta tctggaaatg aacagtttga gagctgagta cacagccacg tattactgtg     56160
     ctggagacgc agtgaaggga agtcaggttg agcccagaca caaacctctc tgcagaaaca     56220
     catgaagtaa atcagctacg ggggcgctca ggacccactg atcagattca acctcagagg     56280
     cagatgcaga tacagggtag gagctggttt tctcatagtg tttgtaactt ccctctccaa     56340
     ctgacagttt cccagagaac ctcactaatt tagagttctg tgcctaccaa aaaaatctcc     56400
     aaatatacac cttttaaaag gtagtaatat aaaggcctag tctcacatgc acaaaatgca     56460
     gatcaccact tccaggcatg agaagttctc aaccacggtc accaagatca gagtcctgag     56520
     gaagctcagg ggtgccttgt gggtcttctc caatcacatt cgggacagga aactaagttt     56580
     aattccctga ttaaaaaagg ctataattcc ctgatttaaa acgtttcatt ccctgattaa     56640
     aaaaggctgg aatttttctg attccagcca agaaagaggc tgggccagag tcagtgtcac     56700
     gtacaagctc acaggtttca cgtctgcccc ttctcctgat atgagactat gcaaagcagt     56760
     atcagcactc atctgctgca ctttttgctg tcaatccatt ttctttcttt tcagctgttg     56820
     tcactttttc gttttctttt ctgctccctg aaaaagaaag atgtggtcca tgcggtctaa     56880
     attccaaggc tcaagctctt ccgctggagc tcaggtgggg ctcaggctgt ggctcctgca     56940
     gccgcatggg agaggctgat ggggctttct tctttcccca gtgcttggga ccctccaccg     57000
     tgtttcatgg ggactcatgt ggaaatttga gtggcccagg ggaactgaag ggcataagct     57060
     tgtttggtga gagtgaaacg tgactgtgga attgattctt tgttgtgcaa accagaagaa     57120
     attttgaatt ttgtcagaat caaaacggag tcacttttgt taaaactttt gcaaatggga     57180
     ctgggaaagg ccatagaagc agggttctca tacacttaca cttggtaaca agaactacca     57240
     caaatagagc ctaaaaaaca caaccttgca caaaatccac cacaacttta ggaaaaagct     57300
     acccccacaa ggatatgtcc tcatgtactg tgagtccaac tttccactgt tgccaccctt     57360
     gttattaatc tttgcagccc aggaaaattg tctcaaagca acttagcaac ttacttagcc     57420
     cttcttattt taactttaac tgcctaagaa tttataccgt gaagtaattg cttcatttag     57480
     tgttaatatt aatgcccttt tgacaatctt tgaagctctt tttttctgat atttcttcca     57540
     acataaagaa tgggagcttt ttcaccatat aaagaatttt taggaagata tgatggaaag     57600
     ggaaactcaa tgtattgttc agtgtttctg cctaacacat caatctctta aaatattttg     57660
     ctgtgtctgc atgtgataaa gcagatgcta acactggaga taaaagaaaa aaatgcacag     57720
     accttccaca ttccaaatca taaagtggta ctggatgtgt ttctgatgaa gtgaaccatg     57780
     ggtcataagt gctagtgcaa gtagctttgg aaaatggagt tcatagcttc ttaaagtttt     57840
     gctagtttta atttaagaat gttatttact atttcttttt agtacaagct ttccagaaga     57900
     ttcttgatgg taatggaagt gtcgcattta agaattacaa atgactcaca gagttacttt     57960
     gtaatttttt ctgtagggtg tacatgtcag tcacttgaca taaaagctca tatgtcacag     58020
     atagaaaaat gaaaattgag ccaattttaa taactgcagt ggaaatagtt tttggcaatg     58080
     agtgcttcaa gacaaccaca aaataaatcg gcaaaatatt aaaatacagc aggaatctga     58140
     tctcagtttc tctgccttat aagaacaagg ctgtcccaca tcaaaattat tattcaagct     58200
     ctaggaaaaa atacactttt ggattctgga agctaaaagc tctctcctta gatagtgcta     58260
     aagtacttgt gaaatgcaga gttgtgttca tgaaaaagat gactttattg tgattttttt     58320
     tttttttttt ttgcctgttg acaactcacc aatctgtctc agatgtaaag ccctgctcta     58380
     tgggttgaat gtggttgcac atgactggca gagactgatt attcatatct ccaggtgccc     58440
     tgtctttgga gcagctacag gagtcaggcc cagactagaa gagcccacac tgaccttctg     58500
     catcacttat gctctctctg gccactacat cacaaacagt gcttatgact gggcctgaat     58560
     ctgccagccc aggaaagggc tggaatggat ggggtgcatt ggtagtgatg gtgggggtcc     58620
     tactagcctg tggcaaatgg aagcatctct tttttatcag actgaataat attgtagtgt     58680
     tttcttatac cacatttact tcatcccttt gtgcattaac acttaggttg tttttatata     58740
     ttggctgcca taaatagagc tgcaattaat ataagagcac aggtgtcttt acaagttcat     58800
     gattttattt cctttccctg tatttccaga gaagcgtttg ctgagtcata ttgtatttcc     58860
     atttttgatt tatttatgag ctgctatact gtttttcata atggtgcaac taaaaaatgg     58920
     tgtagtcatc atgaaaaaca gttttagttt ttgacataat ccaaacacaa tgtttgaatc     58980
     tggttatcta taggagtttc tagtaaaatt ggtgaatgta aattaaaaag cagtaagaaa     59040
     aatctgcaat tcagaaatag tgtacccaga tggtctttct ctccgaaatg aaagagaaat     59100
     aaactatttc tcaggtgaga taaactaaga tattttcttc acacttgacc agtcatacaa     59160
     gaaatattca agggaatttt tgtataaaag ccagcatatg aaaaccatca gcttggaaaa     59220
     aaacacacaa aacctatatt tcaatggtag aaacaattaa aaaaggagaa aagaaacaaa     59280
     cattattact acaaaacaac atcaaatcat aaaaccaaac agaaagagaa taaacaaaga     59340
     tagagatata tacatatata caaacaatca taaaacaata aagaaaataa tttgagtaaa     59400
     tacttataaa taataacctt atgtaataaa attcctcaat taaaaataca gtataaaaat     59460
     tgattttatt aaagtcccaa ctctatgctg cttacaagag actcaaatca tctgagaatc     59520
     tacacacaga ctgaaagtga gtgggtggaa aaaagatatg ctacaaaaat agaaaccaac     59580
     agcacaaata gctctactta tatcagacac aaattttaag tcaaatgctg taaagggaga     59640
     ccaaaatgga cattatataa taaaaaatga tcaatgtagc aagaatacgt aacaactgta     59700
     aatatacata tatacatata tgttatatat atgtaacaag aacacataat tgtatatata     59760
     tacatatata cacacacaca tatatacata tgtacccaca cctgagaact caaatatgta     59820
     aagcaaatct attacatagt aagggataga tactaacctt atacaattat agttggagaa     59880
     tttaacacgt tatttagcat tggatatgtt acctagccaa aaattaacag acaaacattg     59940
     gatttaaact gcactatatt ccaaatggac ctgaatttac agaccattgc accaaacaac     60000
     ttcagaacac atattctttt ttttttaaga attaaatgga tgtgtttatt tatctttttt     60060
     tattattatt atactttaag ttttagggta catgtgcaca atgtgcaggt tagttacata     60120
     tgtatacatg tgccatgctg gtgtgctgca cccatcaact cgtcatttag cattaggtat     60180
     atctcctaaa gctatccctc ccctctcccc ccaccccaca acagtcccca gagtgtgatg     60240
     ttccccttcc tgtgtccatg tgttctcatt gttcaattcc cacctgtgag tgagaatatg     60300
     tttggttttt tgttcttgcg atagtttact gagaatgatg atttccagtt tcatccatgt     60360
     ccctacaaag gacatgaact catcattttt tgtggctgca tagtattcca tggtgtatat     60420
     gtgccacatt ttcttaatcc agtctatcat tgttggacat gtgagttggt tccaagtctt     60480
     tgctattgtg aatagtgccg caataaacat acgtgtgcat gtgtctttat agcagcatga     60540
     tttatagtcc tttgggtata tacccagtaa tgggatggct gggtcaaatg gtatttccag     60600
     ttctagatcc ctgaggaatg gccacactga cttccacaat ggttgaacta gtttacagtc     60660
     ccaccaacag tgtcaaagtg ttcctatttc tccacatcct ctccagcacc tgttgtttcc     60720
     tgacttttta atgattgcca ttctaactgg tgtgagatgg tatctcattg tggttttgat     60780
     tcgcatttct ctggtggcca gtgatggtga gcattttttc atgcagaaca catattcttt     60840
     tcttcagcgc atgtgatact cttcagagtt gatcatatat taatacagaa aatgtatcaa     60900
     aaaatttaaa taaaattata ccgacgtttt atctgactaa gatagagtca aagatggaat     60960
     caaactaaac atcagtaact ggaggaactt cttttttttt tttttttgag atagactctt     61020
     tctctgtatc ccaggctgga gtgcagtgac agaatctcgg ctcactacaa cctccacctt     61080
     ccgagttcaa accattctcc tgcctcagcc tcctgataag ctgaaattac aggcacctgc     61140
     caccactcct ggctaatttt ggtatgttta gtagagaggg ggtttcacca tgttggccag     61200
     gctggtcttg aaatcctgac ctaaagtcat ccaccagcct cggcctccaa agtgccggga     61260
     ctacaggcat gagccaccac ccagcctgga gaaaatttca aaaaaatata aacacaaaaa     61320
     ttaaacaaat cactcttaca tggccaatgg gtgaaagaag acattaagaa gaaaataaaa     61380
     aatgtatgaa acaaacaaag aaacacaaca taccaaatct tatggcgttt agtcaaatca     61440
     ctattatgag gcaagtttag agcaataaat gcccacatca aaaagtagaa atatcttaat     61500
     taatcaaaat aacaatgcat gttagagaac ttgaagaaca agaaaaagct aaattcaaaa     61560
     ttatttttaa aaacaataaa gagcagatta gatataaatg gaattaagac taaaaatacc     61620
     agaaaatatt aaaatagcaa tagtcttttt aaaaaagata agcaaaattg aaagccatta     61680
     gctgggctaa caaaaaaaat ataaagagct aagacatgaa taaatgaggt agaaacagaa     61740
     gatgtctcaa ctgatatccc agaattaaaa taaattagga aatactataa taaattatgt     61800
     gataaatgtg aaaacctaga ggaattggat acattcctgg ccatatacta cctgacaaga     61860
     ttgaaaaagg aaaaaataga aagccttaac agaccaataa aaagtaatga gagggactca     61920
     cttaaaaaat ctatatccta ttgaacataa ataaaaaaac ccaaattact agcacagcaa     61980
     atcaaacagc atttaaatac taatactaat actaatacag catgacaaag tagaattcaa     62040
     cccagggatt caattatgct ttaatataca caagtgaata aacatgatat atcacgtata     62100
     tacgacaagg atgaaaaaca tatttttact ttaatagatg cagaataagt actttataaa     62160
     actcaaaacc tcttcatgaa aaaaactctc aattatgtgt agaaagaaca acacaataag     62220
     accacatgac aagttcatag ctaacatcat acttaacaga aaaaaagttt aatgcttttc     62280
     ttctgaaaac tggaaaagac aaggaagcca actctcagca cttattcaac acagtactga     62340
     aggtactagc cagagcagtt agacaagaga aagaaataaa ggggattaca attagaaaaa     62400
     aggtagccaa attacctcta atttcagatg acataatcat atatatacta aagactcttg     62460
     taaatgctct tagagctgat gaatttagta aagtttcaag atacaatatc aacatagaaa     62520
     tctcagtggg gtttctatac acaaacaata aactaggtaa aaaaaatcaa gaaatcccat     62580
     tttcagtaac tacaaaaact attaaacacc taggaataca tttcaccaag gaggtgaaaa     62640
     tccgtaaatg aacaaactat gaaacactga tataattaat tgaatgagac aaacacaaaa     62700
     taagaaggac aatctgtatt catgaactgg aataatttat attgttagaa tgaccataat     62760
     gcacatgaca atctacatat tcaatgtcat ctctatgaaa ataccaatgg cattattcac     62820
     agaaatatta aaaagcatta ttaaaatttg tatacaattg aaaatacccc aaatgactaa     62880
     tgcagtcttg tggataaaaa acagaggtgg aggtattaac aaccagattt caaaaaaaat     62940
     tataaaactg taataacaaa aacagcgtag tgctggcata aaaatccatg gaaaaaagta     63000
     gagaaaccaa gacattaatc catatattta tagataactt atttgcaaca gaggcaccaa     63060
     aaatatttat tgtgaaaaga aaagtatcct cagtaaattg tgtagggaaa aactgaatat     63120
     ccatatgcag atgaatgaac ttacattccc acatttaact gtatataaaa atgaaataaa     63180
     aataagttaa atactcaaat ggaagaccca aaactataaa gctgctctta aaaacttaga     63240
     aataatgctt catgaaatct gtctggaaaa caattttata aataagacct caaaagtaca     63300
     gtcaactaaa taaaacacaa acgaattaaa ttttatcaaa ctaaacagtg tttacacagt     63360
     aaaataatcc aaatattgaa tagataacat aaaagtggta aaatgtattt taaactatta     63420
     attaaacagg ctattaatat ccagaatata ccagaaaaat aaatgtctca accacaaaaa     63480
     tataaataat cttattttat aacagacaat taacccaaat aagcagacgt ttctcaaaga     63540
     aacacataca agatgccaga aaatatttga gaaaaaatgc tcaccatcgc taataatcag     63600
     gaaagcacaa atttaaacta caatgaaata ttatctcacc ccagacagaa tagccatatt     63660
     caaatacata aaaatagtgc taatgtggat gaagataaaa tgggacatgt atacactgtt     63720
     gatgagaatg tacactcata cagccactat ggagagacgt atagaagtta gtaaaaccac     63780
     aaatgcaaca atcatatgat acagaaattt tacgactgaa catttgtccc aaggacagga     63840
     aattaatata ttgaagagga aaatcaatat tttgatgaga tttcttcact cccatgttta     63900
     ttgcagccct gttcacagta gccaagtaat tgaatcaacc gaagtgttca atataaggtg     63960
     agtagagaaa gatgatgtgg aatatgtaca taatggaatt ctacacagcc atgaaagatt     64020
     aaattgtgtc actcatagaa acttgcatgt aacatataag actctatgtt aattgaaaga     64080
     gccaggaaca gaaagctaaa caccacatgt tttcactccc ataaaatagt caagaaatat     64140
     ccataaaaca taaataattg aaattggaca tgaactatag aaacaataat tgtcacctga     64200
     atatttattt tccttcaaat aatatataat tcaatgatag taagaagagt cctgataaat     64260
     aaatcatgtt gagataaaga gctactgaat tgtgcattcc acactaaatg gaaacaatgc     64320
     cataagatcc agtgttgtgt aggatctgtt taccttttta cctcaaatac tacaagtcac     64380
     tcccttgttc taatcacccc gcaaaactga tattttgttg ctttatgtta tgtcagtaaa     64440
     aatgattcca cattggaagc ttcatgtgtt gctcagaaat caccctgttg ataaaatgag     64500
     cacaactaca gttacaacct ttctgtagat attttctgca tatgatcctc actttttatt     64560
     tgttgttttt gttcacagtt tctttaagca ctcagagtta tatagcatat tttcagcata     64620
     ttttaattat tttccattgt tatggtcagt gctcctgtgg gctcaagagt caggtgttgt     64680
     gtgactgcca gttgacttca ctgccacctt tacctacaca aaacactgca agatgaacca     64740
     tgatgtgtaa gaacccatcc acaaagtgct tcaaataaag acaaggtgac ttgtgtatag     64800
     atttagtgag actggggaac agttcacggt gttatgttac agaggagcag aaaggtgaat     64860
     ctctctcgga gttattgagg gtgtaagagc tgcagttttg gagggtggtg aaaggcactt     64920
     ttgagtggga agccctagag catgtgtgaa agctgaagga atgatcacca tgcacatgga     64980
     cgctgatgag gcatgtggta aggtccattc cctccagatg ttcctggatg ggcggttgta     65040
     ggatggctca tgtatatgaa aatgtgggca gggctgaaat aggaacaagg ccatcatccc     65100
     acgggaatgc gagaaagcaa aacagagatg gacacccatg aagagagctc attagacctg     65160
     gtgaaggaaa gataggaatc tcttccaaag gctccgctgt tcttggctaa atatgaattg     65220
     tggtgataga ctgaaaataa ggaggggaag ggagatatga ctcaagagag gaaaaaccct     65280
     aagacagcat aaaccaaatc tacacagaac aatcacatgg tagatgccag ggtggggctg     65340
     aaacaggagc ttcaatctac tgggtaccta gttgagatcc tttgacattt cttgtgaggt     65400
     aattctagtg atgatgaatt tcctcagttt tcctttgtct gggaagtatt tctctatcct     65460
     tcatttcata agagatgttt tatcaatttt aggttcttgg ttggctgctt tttcccccca     65520
     gaaatttaaa tccagttatt tgctgcataa caaagtttct gccagtaaca gatggtggtt     65580
     ccacggtgct atacttcctt acttatctat atctctttat gtttagatac acaaataatt     65640
     actactatgt ttccatggcc aacagtattc actacggttg tttctgagtc actggggtgt     65700
     tgctttttct ggccagaacc ctctgtggcc agtggcactt ttgcctgagt tcttgtccta     65760
     catgtggaaa gaatgaagta tgcagccatg tggagggtaa gcaagacaaa gcctagcttc     65820
     attaagcatt agagcatctc agaggagacc cccagtgagt agctcctctc tgtaggcagg     65880
     tagactggtt gagtgttcag ctctaagcag agagggtagc tcctccctgc agccggtcgt     65940
     cctgtggtct tcccagttct caggagagag ggtagctcct ccctgcagct ggtcgtcctg     66000
     tggtcttccc atttctcagg agagagggta gctcctccct gcagctggtc gtcctgtcgt     66060
     ctctccatcc tctgtcctgc tccagctgag gccaggggtt tttacggacc tccgagggga     66120
     ggaagtgcaa gcccattggt ccatgggtgc tcatgggtgg gccgtaggaa gcaccacaag     66180
     tcccgactct ggtctgagga tctgggagcc ccggcccagc cttcaggccc ttcctgggga     66240
     cccgcccctg tgcacaggaa tcttcctgcc tcctgttgta gttcatggtc cctggggctc     66300
     cgccctgact ttgctccaag atcagagcag gcaccagcag caggaagaag ccaggaagtg     66360
     ggagcaggca cttcggagca tgcaagagca cagggctttt cctggggacc ccaagagtgc     66420
     aggggcgcct gaggccgcag cccccggttt gcggagcagg gcttctgccg gcttcatgga     66480
     gcgagaggcc cgtgtcttca gccgcagttt gggaggctgc agccacagcc ggggaggcag     66540
     ggctccttcc tgttcctcga cctccaagag cacagagtcc gagcccactg ccctggtttg     66600
     cgcagctgga gccgcacctg agagatgaga gctgctgcct gctcctggct cccaccggct     66660
     ccgaggagca tcagcccctc cacccctcct tgcagcctgg gcaggggctc ctgatcctct     66720
     ctgggccctg gccggcgtcc agggcaggag tgacatctcc acaagctccc cgcattgccc     66780
     cggtgctcag ggaggcccgg gcagagctga tgacgacccc ggcccgtagt cgggagtggc     66840
     ggcctccatg gtcaccctga catgcggctg aaccctgggg acgcggccca agcagcctgc     66900
     acagaacctc ccaaggccca ggaaccccgc accctaggcg gggtgggcac agcggctgct     66960
     ccgctgcccg ggttttcaag gaggcgccac ttccacttcc ctccctggaa ccccgaagtt     67020
     tgacatgggg gctcctttct gccttgctcc gcagctcccc ttcccccgga gtggagcccc     67080
     ctgtttccct ggcgttccct cccacagctg cagtgttctc catcggtgtc atcaccttcc     67140
     agctctgctg ccctgctctg cagactaagg ctctgattcc gtaagagagg ggagctgctt     67200
     cccagtagaa ccttgctggg gagctctgtt cccatctcag ttcctgaggg gttaaaccag     67260
     tgcatttagg atactggttt tggtggtttg cccctgttga gtaatttctt agttctctag     67320
     tgggtgtaag agacttggct ctggaagcat ttcagaagtg tgggctctga tacaccccag     67380
     acagacactt tgggaaggga agatttttgt gactattctc attataaggg aaaggcattc     67440
     aaaagaatag aaaaactctc cagtatgtgg ttcctgagaa tttctcacta aaaacatgct     67500
     tatcacactc gactcaaaac agttcaatgt atattagtgt attttatctt gtactagcct     67560
     ttattgcatt tggtgaactc tgccccagtt cagctcatac tctagctttg attctcccta     67620
     caaaaacttg tctctctcta gatttcagat ttgttgattg tcttaaaatt tcaatgatct     67680
     gaagtattaa agaaaatttg caaaagtcca ttttctctga ttaacagtta ttgttgattt     67740
     tattcttgtt gtaaaaaaaa gaaattctca tctatgtaca tttcaaacct gaataacaaa     67800
     atttttatta acaccaaaaa taataaaaga atccaaatat ttatcagctg cctaatagaa     67860
     aaacaaatca tggtaacatt gttcgctgga atattaccca tcattcataa taagggaatg     67920
     tctgatacac aaaataagaa gataaaatta tcaagtattt aaattgagta aaataagcca     67980
     aacaaataag agtatgtatg attctatttt taaaaattct ggaaaatgaa aactgatcta     68040
     aagtaatata aagaagatta gtagtttcct gggaatatgt tggcagaagg gaaggagaaa     68100
     ggataaggaa atagaaatag gaagtagaag gacagaaaag aagttgaggg aatttcactt     68160
     gtccaccttc cttataatgg taatagttat gccatgatta tcagttttac actttaaata     68220
     tgtaaagttt ataatctgtc aatcaaatct tataaaatgt attatgagga aacaagttga     68280
     aaattagaca atgtaggagt gacagaaaga tagatatgag tatgttgaat gtcagagata     68340
     cctgaaagtt tatctacctg aaccctagtt ctctccatag tttaaggtaa acaggagagt     68400
     gcaggaaaat catccatatt ctgattaggc agtggcttct gcaaaccaca ctaggcctgg     68460
     ccggctgtgt cctggagttg gctaagggag gagtcagggc cagtggtgag aagtgcaggc     68520
     ccagatacca gaactcactc atcccagaca tgagctctta gatacacaga gagcccatcc     68580
     atgtgtggat ttatcttaca tctgtaagta gagaacattg actcttacag aacataattt     68640
     acacacatag gtaaatctga aataaggtga tcagtgtgaa gattttatca cagcacagtt     68700
     tcataataag cacaatttct caaatcccat tgttgtcacc catcttcctc aggacacttt     68760
     catctgccct gggtcctgct ctttcttcag gtgtctcacc ccagagcttg atatatagta     68820
     ggagacatgc aaatagggcc ctcactctgc tgaagaaaac cagccctgca gctctgggag     68880
     aggagcccca gccctgggat tcccagctgt ttctgcttgc tgatcaggac tgcacacaga     68940
     gaactcacca tggagtttgg gctgagctgg gttttccttg ttgctatttt aaaaggtgat     69000
     tcatggagaa ctggagatat ggagtgtgaa tggacatgag tgagataagc agtggatgtg     69060
     tgtggcagtt tctgaccagg gtgtctctgt gtttgcaggt gtccagtgtg aggtgcagct     69120
     ggtggagtcc gggggaggct tagttcagcc tggggggtcc ctgagactct cctgtgcagc     69180
     ctctggattc accttcagta gctactggat gcactgggtc cgccaagctc cagggaaggg     69240
     gctggtgtgg gtctcacgta ttaatagtga tgggagtagc acaagctacg cggactccgt     69300
     gaagggccga ttcaccatct ccagagacaa cgccaagaac acgctgtatc tgcaaatgaa     69360
     cagtctgaga gccgaggaca cggctgtgta ttactgtgca agagacacag tgaggggaag     69420
     tcaatgtgag cccagacaca aacctcgctg caggggcatc tgagaccacg agggggtgtc     69480
     ctgggccctg tgaactgggc tgctctccgt ggcagcggct ggtggtgcta aaggctgatt     69540
     ttctctcagc atctggggct gattcatcaa gtttcctcag agaacctttc agatttacaa     69600
     ttctgtactt acgtttaatg tctctgaatg tgacactttc cttccctggt gtgtctttgt     69660
     ttttgtgaca agaggacaca ttctcacctc cacagaagcc cgagtgtcac tttggggaca     69720
     gaaatgaccc tgccctggtc accagaatca gagtcccgag gaagcccagg aggacctggg     69780
     aagtgttttt caatcagact cagggcaggc gtctccgtgg gaatctctga ttggaacagg     69840
     ctttgggatt cagattggga ccaagaggga ggctcaccca gggccagggt ccttagaatc     69900
     ctgacagttt tcacagtaac cccatcgtcc tttaaaactg aacatctaac tcagaactga     69960
     cccatttggt cctttctctg taatccattt tccttttctc taggcttcat tcttacactt     70020
     cccttttcac cttcattctg aaaatggagg gtgtgcttcc tgtggtctaa accacaggcc     70080
     tcagatgcat tacctggaac tcaggtgtgg ctctggctat ggctcctgtg gacctggcag     70140
     gctgagggat ctttctcatt ccctggtgcc tgcatgcccc tgctgtcttc tatgcgtgga     70200
     tgcatttggg aaatgcaagt ggacactcat agtcgtttcc tcaaatggga tactgttgta     70260
     gagctgatct tgtgcttctc accctgtcac agagccccca ctctcacttg tggatttttg     70320
     ggagagctga ggatggacac tttattgggc tgtgagctct gcatgatggc aatcgtgagg     70380
     tctgggtggg cacagccaga gccaatggag ctggccaaaa ggaaagacag gacggaattc     70440
     ctgggaagtc ctacagctgc tgtctaccat agagttccat tgtcttctcc tctgctagga     70500
     ttaaaccaga ccgaccaagt tcatctagga caatcttcct gacctagaga agtgatgaca     70560
     ggctttaata acacctgtat tatacatcag agcaacacct agattagtgt ttgattgaat     70620
     aattgagact atgtcctagt caaggtgaca cacaaaatca attattacca tgattaatat     70680
     tttatattga ttaatatttt aatactaata ttaatcaatg caatattgat ttaatattaa     70740
     ttaatatttt atatttgaca atagaatcag actcatgtta taaataattt tgcaaatata     70800
     tgtatattat tattggtctt ctgagcataa atctccatta gccgattaag tgtgcatgca     70860
     ttggtcgaag gagggcatcc acccttttga gggaaaatgc atggagggta aatgaggctg     70920
     ggaagctgat ggcatatgat ggaagcctgt ccctgagtga aggagagagg gaggcaggat     70980
     tgggtggaac tttcctacat ttctgtgctg tgcaaagaaa ctccagatta tcactgagtc     71040
     tcctgcaagt caaagttgcc cctcaggaaa cccccatcac tcccagcaat gactctgccc     71100
     cagatgagtg caaggctcac tctattcctg agaaaggagc acaggatatg ggatttagca     71160
     tgagccacgt catggacgtc agagagcagg agctgggtgc atgatccaag tgcactgttc     71220
     tgcctgtagg tggagagagg aaggtgcatt cccagagcta acacactgtg gatttttata     71280
     cagaatacac gttttcacct gatttattgg aaggcatatg aaaaaatgtg cagtccacaa     71340
     acaattgtaa tttccaaatt taccacaatt gcattatttc ttcattctgc atgatgtcct     71400
     ggaacagagt tacatttttc atgggtattg tttcatggat tgaggacatg aatttcacac     71460
     ctgccttttt agccatgtgc agagaccttg agtagagcat gtctggacct catctacact     71520
     atctctcatg ccccaaggga aagagtggag acttgactga ctgcaaactt tggggtggga     71580
     gctgctcctg cacacctcag aggctgcaga gaccccaagt gcagttttat tgagttgggg     71640
     tgtctttcta tgtgggggaa ttatgggctg ccccactcct ggtgattgct ggtcagctct     71700
     aaagacgggt tcagaatgag gtcacctggt aacctttctc actgcagctc cattatgtaa     71760
     attaccacta ccagattcca ggtaataatg caagctctat ttttgcgacc tcattttctt     71820
     atcttttttg ttcataagtt tggttataga aaatgttacc acctagtttc ctgattcaaa     71880
     tcctataatt tgctgaacta ctcaggatgt tttttaatgt gcccagtatt tgggaaatat     71940
     aaatgaacag ccatgtgtgg ttcactgagt ggacatagtc agttaagtac attagtaaaa     72000
     acatgattgc tgaattgtat agtgaaacta caattagatt tgcagaaaat tgcctccagt     72060
     aatgtctgaa gggagagcca cccagccaat cccgtctctg ggcttggagg aagctcacta     72120
     cacaggccag gtcacatggg tgatatccaa ggtgcagctc ccagcgttac tgtgctattt     72180
     tccaggaacc agacatacaa cttggcatct cagaagaaga tctccaagat ttcctttacc     72240
     tggacttcat gggcagcaca ggaccagcag gagatgctca gaactcaccc aaggcagaac     72300
     cccagggaag agcaggtgcg gagaggaacc agccagggaa cctctgacca ccatctaggg     72360
     ttgccttctt ttccttcaca gcatggagcc ctcaggagac cacccactag gttgtctgta     72420
     actcacgtcc ttcttgtctt gaagcaaaac agacataaat atgaatttgc agttagcccc     72480
     ttgaacctgg gtcaattttt tctgagaatg ccaacttaat cgtcaacatc atgggcatta     72540
     tatcaaaatc gagatggata tttagtcacc aggaagacag tttgtctcat ggtagaagtg     72600
     cttattcaat taaaaattac attttaatga aaaggtctct aagtgaggac tgttcaaata     72660
     ataaagtatt agtgaagtat cagaaaaata aatggcacca ttatctttgg tatccagtta     72720
     atctataaag catatcaata ttcatgttgt aagtggccta ggacaagtta tggcagtatt     72780
     taggctataa ggagacattg ctttccttct tagataggaa tgtccttttt tatcatactt     72840
     taagttctgg ggtacatgtg cagaatgtgc atgtttgtta cataggtata caagtgccat     72900
     ggtggtttgc tgcactcatc aacccgtcat cgacattagg tattactcct aatgctatcc     72960
     ctcccctagc cccccacccc ccaacaggcc ccagtgtgtg atgttcccct ccctgtgtcc     73020
     atgtgttctc attcttcaac tcccacttat gagtgagaac atgcagtgtt tggttttctg     73080
     ttcctgtgtt agtttgctga gaatgatggt ttccagcttc atcaatgtcc ctgcaaagga     73140
     catgaacagg aaattccttc taaatgaact gtgttttgac aaatattatc cagtgactct     73200
     tcctacatgg ggtttctaca tgatctattt ttcttttcca aaaacaaatc actcaagttt     73260
     ctcatctcct gagtgagaaa aattttcccc aaacacaatc tcagtctagg tagacacctt     73320
     catgaatgag ccttcattct cagacaggca cacactgtcc ccatgtggat gcttctctca     73380
     gacaggcaca catatcccca catggttggg atcatctgct gggactttgc cccatgggga     73440
     tctctattat cactggggca agttagcacc tcgatcctca tccacactgt ggcctttgat     73500
     gtctgtgacc tgccacaccc tgtctgtccc tcagtgtcta gaatggtgaa gtagtccacc     73560
     ccagggatgg aggaagatcc cagattttcc tgtgaaggag acagaatgcc aatcacagag     73620
     atggaaatct ctcacatccc tcctgggtca tattctgggc agggtctctg aagtcacccc     73680
     tggatcagtg tcctaagatg tctccctcta tttccttggg aagaacccat agaaagacaa     73740
     ataagttttt ctattagggc atcgtagaac ttgtgctttc ttgtgtgaca aatataggta     73800
     ttttgactat tttaaaaatt agatataata ttaagtctac caaatatgta aatatataag     73860
     ctttataatg tatccctaca gggatgtctc ctggaattga gaattgaata tatatatata     73920
     tatatatata tatatatatt ccaagataaa tcttggtgaa cattataaaa ttgactctaa     73980
     tatttatgtt caagatgatc atttcaaaaa agttttaaca acagagacca gatgtatctt     74040
     tccaccagaa ataactgtaa aacacagtga aattttttaa acatgatttt aaggtatttt     74100
     acatcaaata acaaggacag tgattgctga ggtggggaaa atatacaaaa ggagccctct     74160
     gatgaacgca tgctttgcta ctttttaaag agagtttcca ggccacagtg ctgaagagga     74220
     accaggtaga tttgaagttg aggaaaagga gctgagatcc cagacaggcc aagaagtgtg     74280
     gagatcacag actagacagt ggatagcaga acactgttca gagagtgagc cccgtagatg     74340
     gccggagaga gacccagcct tggtagagtg tggatcagtg tatgggaacc ctggaaagtt     74400
     gtacatgaga acacacctta ctctaggggg cttgccttaa aatctaaaat taccctaaaa     74460
     tttaaacata atcagttaca aattttaaaa cataaagaaa gacaccatgt taaagaaaga     74520
     taaaggaaag gtggatttgc aatattaaaa tataaaaagt gtatttctga acaaatgata     74580
     atactgatga ggggggacaa gtttattcca tcacggcaaa tgatttaaga gaagaacatt     74640
     ccaaatgctt atggccctaa tgaggaagct tcaatcacga atataaatag agttatctca     74700
     ttaccagtgt gatggtgtga gaaacttcaa gatttccaaa gagacaacct aatctggtaa     74760
     aaattataga aacccaacct gtactatcaa ggaaaattat tctgagaaca cacagtaaat     74820
     gaaatccatt gactgacata aagtcctcct gttgactgaa gggttatgac cccccacaag     74880
     gcatatgttg gaatctcaac tccatcgtga cggtggcagg aggtggggcc tttggtctcc     74940
     tttgaaaggt gttgaggctc cgtttcatga atggaatcag cgtcctgata caatacccca     75000
     gagagctccc tctttccttc caccaggtgt ggtcacagta ggaaggtggc aatctataaa     75060
     taaagaattg ggacatcacc agataccaat ctttaagtca tctttatctt gaacttccca     75120
     gccttgaaaa ttgggacaaa tgcatttatg ttatttctga gccacccagt ccatgatatc     75180
     ctcttatagc aactcaaaca gtcttagcaa gtgcagtgag gatcacaata cttgagagaa     75240
     gagcattacc tcttctttac atctcaggat cacagaatct tgaccttggg ctgaactgac     75300
     aagaaggtgg tgcttccctc tcccccagat ctcagttgag gtcatgatgt cagatcttta     75360
     tggtggagac taaattcctg cctgtgattt gatggatgcc tttctcctcc cagcctcact     75420
     cacaagacat agtttccatg tcaggtgtta tagaccaaga acaggtggac cccgattatt     75480
     ctcaccccag ctcacttgta gggcagaggt tctgggagct tcctgggaga ggaacagtga     75540
     gatcatacaa gtccatcact tccatgcagt tttctgtact taaagcagga gtgtccttct     75600
     gagctgcttg aaccctttct tctgcaccaa ggtagagact ggcatgtttt tctagtgata     75660
     cagtcagggc ataaaaacag tgatagaaag ctctctctaa agggcgtgac cagatttgca     75720
     gtagaatgag aatagtttca accctaaagt tcatctaaaa tgttagtggt tttcatggta     75780
     agtaattaat agaaacctga cagctccatg acagcaatat agaaatcaat agtccactca     75840
     ttttaacaga gagaattaga aaacctataa tttaagaata accagctggg cactgtggct     75900
     ctgcctgcag ttgaatttac ttgggagttt gaggtgagag gatcacttga gcccaggatt     75960
     tcaagcgcag cctggaaaca tagctagact taatttcttg aaaaaaatca gtgctatttt     76020
     ggggtcagaa caatcctcaa aaatggtccc cggaaagaag cccaaattta attgcaccag     76080
     attgttgaac gatttatgct acaaaacagt gtcacatgtc agaaatgagc acagtgtaac     76140
     atctacatgg tttgttaaga gacacaaatg gtcaagtaga acaatcaggt aattaggctg     76200
     tcgaaggcaa cactgccaaa tgacagcatt tccgtggaaa ctgcgtgtac atccaggact     76260
     gcacctgtga acgatgacat catacccttc acagtgtcga ggaaagagac atcactcaaa     76320
     cagacaagcc aagggacttc agaaaatata aggggaaata cagtgtgcaa atatgtaaaa     76380
     aatgcaataa gatgattact cctaaatgaa tatcaagaca caatcacata atatgaaatt     76440
     aaattttcct gaatgatagg attactacca atcacccccc aggacaccct catctactct     76500
     gtgcacagcc ttctcgtcag gcgtcccagc ccagaccttg ctatgtagca gaagacatgc     76560
     aaataagacc cccctttttg ctgatgaaaa gcagcccagc cctgaccctg cagctctggg     76620
     agaggagctc cagccttggg attcccagct gtctccactc ggtgatcggc actgaataca     76680
     ggagactcac catggagttt gggctgagct gggttttcct tgttgctatt ttaaaaggtg     76740
     attcatgggg aactagagat actgagtgtg agtggacatg agtgagagaa acagtggacg     76800
     tgtgtggcac tttctgacca gggtgtctct gtgtttgcag gtgtccagtg tgaggtgcag     76860
     ctggtggagt ccgggggagg cttggtccag cctggggggt ccctgaaact ctcctgtgca     76920
     gcctctgggt tcaccttcag tggctctgct atgcactggg tccgccaggc ttccgggaaa     76980
     gggctggagt gggttggccg tattagaagc aaagctaaca gttacgcgac agcatatgct     77040
     gcgtcggtga aaggcaggtt caccatctcc agagatgatt caaagaacac ggcgtatctg     77100
     caaatgaaca gcctgaaaac cgaggacacg gccgtgtatt actgtactag acacacagtg     77160
     aggggaggtc agtgtgagcc cggacacaaa cctccctgca ggggcgcgcg gggctaccag     77220
     ggggcgctcg ggactcactg agggcgggac aggtcccagg aacaggtgca gcggtaggtt     77280
     ttctttctcc tcagctggag aagtcaggtt tgtgttttca gaactctgga gtcttacagg     77340
     ttgctacatt ttcatacagt tattagtatg tatttattat cattggtatt taagttttaa     77400
     taattttaaa ccttttatgt agtgttattt tttaaaactg tttactttca tttgcagtta     77460
     ttcttccaga gtttcattaa catctattgc tatgagcaac tacatagcta tgagagcata     77520
     aatttacacc tgtagacgta ggtctaaatg ccacaaacct gtgcataaat gtatagtgac     77580
     ttatatttaa cattataata agataatttt taaaatatat cctaaacgat caaacttact     77640
     gatgaactaa atataaatta tcagagtaat gcataattga tttcaataat tttatattgt     77700
     ttatattaat taatatctat ttctttactg aaacataata tattggtcat ttcaaaatag     77760
     ctacgataca tttcaaatgg ccttgatgct aataatgaaa agattttgag gtgattaata     77820
     tgctaattag ttatatttaa ttattccata ttgtattaat atatcaaaac attgctttgt     77880
     acctcataaa tagatacagt cgtaatttgt caattttcaa gacagttatt taatccatcc     77940
     tgggtcataa tctttttttc ccctgtccat ggctgattgg attgtcctga taaacccatc     78000
     catacgcctg cctcccgcag gcttctgaga cgggctccat gaaggccaga agcaggcacc     78060
     ctgtgagagt ccacacgacc tggagcctcc ctctccttgg attaggccat ctcctcggga     78120
     tcgcagggct cttcattatc ctcaccccct tgttgtacca aacaagcaac atcacacttt     78180
     aattcatcac gctttgcttt aattttcccc ccaaaaatca aggtaataat tttagcaata     78240
     aacatcacaa cctactatga gtaagtcctt tccaacgtac taaggtttct tctgaggact     78300
     ttacatgtat tacacagttt aaatttcttt atgaaatgag ataccaatat ctctatatta     78360
     gagattgttt ttctaagtcg tttctgacaa ttaatttgtc caagggcctg taaggattct     78420
     gttagaaatc caggataaaa tttgaaattt cagcagtaat atctggaaaa acattgagac     78480
     tatatttaac ttcgtacacg tgctctcaaa tatccttatt tccctaaagg ttgttctcag     78540
     ttattatttt acacatgata tctataatta atactaataa cacaggttaa aatattatct     78600
     caattattta acgacttttg ataataacaa aatacatatt ccacatattt ttgaccagtt     78660
     tgttttctct atgaaacacg tgtttattaa cttattatta tcagatgtca cccttatctt     78720
     tctgatttct gggatttaat tctagcaggt atcgtttagt atattttatt aattcatatt     78780
     atattgttca cattttcagt atgattttat tatttgcatt gtttattgaa tttgaaactt     78840
     tcttacataa ttttttaaat tttttatttt catgagatgt aatttcaata taagtaataa     78900
     tgcatagatc tgcaatgtac cattacagga tttctggcaa atgtaaacac acttgtcccc     78960
     aacagccaag gttggatgta gaggaggccc attcccccag cacaagcgac cttctctgtg     79020
     cttccagtca gctcccacat ttaacacaat gttttgattt ctgccactgt agattcactg     79080
     ttcatgtttt gattttcata taaatggatt caaacattat agaccccttt tctggtaagg     79140
     ggctactttt gttatttttg acgtttattt atgctatttc atatgtaaaa ttgcatctgt     79200
     atattgtcat ttattcactt aacagactgc ctctgatata aaatctcaaa gttttgatat     79260
     atatggataa agagagaggg agagagagag agggagaaat tttatacttg agtcagtgca     79320
     tttaacaata aacgacagta agctgatcca tttccctgtc aatgcacttt taatttgttt     79380
     ctggtttatt aacatcataa gcaaagctgc tgcacatatt ttttgttttt ctatttacct     79440
     tttctcattt ttattgagaa agtatgtgga aatatccatt tcttcatcat aaaagtaaac     79500
     ttcatttgtt cagctttatg gaactataat tgacaataaa attgtatata tttgaggtgc     79560
     accacttgat attttggtat aaattgtgaa attttcacta tgaccaagga gttagcatgt     79620
     ctgttaccct acatttgcca ttttcttcct ttaatttata tgtgtgtgtc caatgagagc     79680
     acctgagatc tatcccttta ccagagatca agtttgcaat acagtgttga tagctatagt     79740
     cccattgctt tacattagac ctccagaacc cattcaacct gcacaactga aactttatgc     79800
     ccttgacaag aatcattcta tttccactcc tcccagccct gggaaccact tttctactct     79860
     ctgcttctaa gaatttgaat attttagatt ccccatataa atgagataat gcagcatttg     79920
     tctttctgtg tctggcttat tccacttagc ataatgttct ccaggcccat ccatgttgtt     79980
     ggaaatgtta taatttcctt atttttagag gtcacataat attccattgt atgtgtatac     80040
     atttgcttta tacacccatt tacctacggt cactaacttt aaaaaaattc taggccatta     80100
     tgaataatct tgtaataaac atatttttta cttactgatt ttatttcctt tgaatacgca     80160
     gccagaagtg ggatcaccag atcacgtaat agttttactt tcaatttata aaataacttt     80220
     tactaccact ccactgaata attctctttt caccagctaa ctgtcagcat tttgttatct     80280
     tttgtgtctg tgataatagt catggtaatg aacgtgaggt gatatctcat tgtggttttg     80340
     atttggattc ccctgatgat tagcaatgtg gaacaccttt ccatgtcctg ctggccatgc     80400
     atatgtcttc ccttgaatac aatgtctatg tacagttttg ccctcttaaa aatcaggggt     80460
     tttttttgct attgtatggg ttctttacac atttagatag atccctttgt cagatatata     80520
     gcttcaagta atttatttgt ctatgatttt ggtgtcgaat ccaaataaat cattttccag     80580
     atcaactctg gaaagcttct tttttccctg ttttcttcta tgagtttaca attttagata     80640
     tcatattata tccaagcctt gagtacattt ttaggtgatt tttgtatatg agatgagatg     80700
     aggtctcatt tttttctgca tatggatgct cagtttttac accatttgtt aaaaaggcaa     80760
     tcttttcctt attgtgtgtc cttggaacaa aaaatctggg agatacacaa aagaggaaaa     80820
     tcttagacca atattcctga tgatagacct aaaaatcttc aagaaaatac taggaaattg     80880
     aatatagcag cacattaaaa agttattaca ttatgatcca gtagatttta ctcctttcat     80940
     gcaaggctag ttcaacatac acaaatcaat aaaggtgctc caccacataa gcagaatcaa     81000
     aagcaaaaac tatataatta tctcaataga tccagagaaa ctttcaataa gattcaacat     81060
     tcttcatgat aaaaaccttc aacagactag acatcaaaga aacttagctt aaaatgataa     81120
     gagccatgta tgacaaaccc acaactgaca ccatactgaa tcaacaaaag ctcaaatcat     81180
     tccccttcat aactggaaca aaacaagaat acccactctc accactccta ttcaacatag     81240
     tagtggatgt tctagccaga gaaatcaaac aagagaaaga aataaaaggc atccccacaa     81300
     gtaaataaga attcaaacta tctatttttg ctgttgatag gatcctatgc atagaaaatt     81360
     ctatagactc tgccaaaaga atcctagaat tgataaacaa ctttagtaat gtttcaggat     81420
     acagaataaa tatacgaaaa tcagtagctt ttttatacac caacgacatc caggcataga     81480
     gaaaaataaa tgacacgatc ccacttacaa catctacaaa aaaagaaata cctaggaata     81540
     cagctaacca aggaggtgaa agatctctac aagaagaact acaacctctc cagcacatgt     81600
     tgtttcctga ctttttaatg atcgccattc tagctggtgt gagatggtat ctcattgtgg     81660
     ttttgatttg catttctctg atggcgagtg atgatgagca gtttttcatg tgtctgttga     81720
     ctgcataaat gtcttctttc gagaagtgtc tgttcatatc cttcacccac tttttgatgg     81780
     ggttgtttgt ttttttcttg taaatttgtt tgagttcatt gtagattctg gacattagcc     81840
     ctttgtcaga tgagtagatt gcaaacattt tctcccattc gttaggttgc ttgttcactc     81900
     tgttggtagt ttcttttgct gtgcagaagc tctttagttt aattagatcc catttgtcaa     81960
     ttttggcttg tgttgccatt gcttttggtg ttttagacat gaagtccttg cccatgctta     82020
     tgtcctgaat ggtattgcct agattttctt ctagggtttc tatggtttta ggtctaacat     82080
     ttaagttttt aatccatctt gaattaattt ttctataagg tgtaaggaag agatccagtt     82140
     tcagctttct acatatggct agccagtttt cccagcacca tttattaaat agggaatcct     82200
     ttccccattt cttgtttttg tcaggtttgt caaagatcag atagttgtag atatgtggca     82260
     ttatttctga gggctctgtt ctgttccatt ggtctatatc tctggtttgg taccagtacc     82320
     atgctgtttt ggttactgta gccttggaaa caacaggtaa caacaggtgc tggagaggat     82380
     gtggagaaat aggaacactt ttacactgtt ggtgggactg taaactagtt caaacattgt     82440
     ggaagtcagt atggcaattt ctcagggatc tagaactaga aataccattt gacccagcca     82500
     ttccattact gggtatatac caaaaggatt ataaaccatg ctgctataaa aacacatgca     82560
     cacgtatgtt tattgtggca ctattcacaa tagcaaagac ttggaaccaa ctcagatgtc     82620
     caacaatgat agactggatt aagaaaatgt ggcacatata caccatggaa tactatgcag     82680
     ccataaaaaa tgatgagttc atgtcctttg tagggacatg gatgaagctg gaaaccatca     82740
     ttctcagcaa actatcgcaa ggacaaaaaa ccaaacaccg catattctca ctcataggtg     82800
     agaattgaac aatgagaaca catggacaca ggaaggggaa catcacacac cggggcctgt     82860
     tgtggggtgg ggggaggagg gagggatagc attaggagat acacgtaatg ctaaatgatg     82920
     agttaatggg tgcagcagac caacatggca catgtataca tatgtaacaa acctgcacat     82980
     tgtgcacatg taccctaaaa cttaaagtat aattaaaaaa aacaattaca acgaaaatcc     83040
     ttaatgtctc ttgagtaggt agagaacata tattttatct aagtaacttg ctctaaactg     83100
     caaaccaaaa taagatgatt caataaagta ttttgtaaac tagtctttag caaaaaaaaa     83160
     aaaagaacta caaaacactg cagaaagaaa tcagaaagta aacaaataaa ttgaaaaaaa     83220
     aattccatgc tcatggaatg ggagaaataa tattgcaaaa atggcaatat tatccttcaa     83280
     tatttatcga ttcaatgtta ttatcaaaat accagtttta ttcttctaga aatataagaa     83340
     aagtattcta aaatttgtat gtaaccaaaa aagaacaaaa acagccaaag taatcctcag     83400
     ccaaaagaac aatgccaggg acatcacact atttgacttc aaactctact ataaagccac     83460
     agtaacaaaa actgtttggt acttgtacaa gaataaacac atagacaaat gaaatagaat     83520
     agaaaactca gaaataaagc tgcacaccca caaccatctg ttttttgaca accctgaaaa     83580
     aaataaacaa gcaatgggga aaagacaacc tagtcaataa atggtgctgg gataacttgc     83640
     tagccatatg cagaagatgg gaacttgact cgtgactatc accatatgaa aaagaaaatt     83700
     aactcaaaat ggattatagc tttaactaag acttcaaact acaaaaagct aggaagacaa     83760
     cctaggaata ctcttctctg cattggcctt ggcaaagaat tactggctct gagaccaggc     83820
     attttttgta acagtgattt attttctggg gtacatatca tggagtggga ttgctgattg     83880
     aacgatagat ctattttagt tgcttgataa gcctttacaa tttttttcac ataggttgca     83940
     ctaatttaca ttaccaacaa cagtgtataa atgcattttc tctgaattct catccatatc     84000
     tataattttt tgacttcata ataataggca ctttgactga tgtaaagttg tgtctcactg     84060
     ttgttttaat attaatttat ctaatgatta atgttgttca gctttttatt tccatatgct     84120
     ttttggccat ttgaatatct tcttcacaaa atgtctgttg atgtcttttg cccacatttt     84180
     aatggtgtaa tttatttatt tgttgttgtt gctgagttgt ctgtgttctt gttagattct     84240
     agatgttagt tatttattgc acagtaatgt aataaatcta aaagtcagga aacaacaggt     84300
     gctggagagg atgtggagaa ataggaacac ttttacactg ttggtgggac tgtaaactag     84360
     ttcaaccatt gtggaagtca gtgtggcgat tcctcaggga tctagaacta gaaatatcat     84420
     ttgacccagc aatcccatta ctgagtatat accatcccat gactgggtat atacccaaag     84480
     gattataaat catgctgcta taaagacaca tgcacacgta tgtttattgt agcactattc     84540
     acaatagcaa agacttggaa ccaacccaag tgtccaacaa tgatagactg gattaagaaa     84600
     atgtggcaca tatacaccat ggaatactat gcagccataa aagatgatga gttcacgtcc     84660
     tttgtaggga catggatgaa gctggaaacc atcactctca gcaaactatc gcaaggacaa     84720
     aaaaccaaac accacatgtt ctcactcata ggtgggaatt gaacaatgag agcacatgga     84780
     cacaggaagg ggaacatcac acactggggc ctgttgtggg gttggagggg cggtggggag     84840
     ggatagcatt aggagaaata cctaatgtta aatgatgagt taatgggtgc agcacaccaa     84900
     catggcacac atatacatat gcaacaaacc tgcacattgt gcacacatac cctaaaactt     84960
     aaagtgtaat aaaaaaaata aaattaaaaa aatcaattgc aaaaaaactg ttgaaactat     85020
     gaaaatatga ggtaattaaa caacacaatt ttgaccaatc attggctcac caataaaatt     85080
     aagatattaa tttttaaaaa tacaaagaaa aatgaagcca caacatacca aaccctctga     85140
     gatacaggaa gagcagtact aagagcttat agttctaaat gtctatgaaa aaatagaaag     85200
     attataaact agcaatctat tgatttactt caaggaacta gatattttag aacaaaccaa     85260
     ccccagagct agaagaagaa aataaataaa gatcatggca gatttcaaaa atattgaaac     85320
     aaaaaagcaa aatataagga atcaacaaaa caaaatgtgg ttattttaaa acatgaacac     85380
     aattgataga gcactaacta ggtcaacaaa aaaaaaaaga cagaagattc aagttagcac     85440
     aagcagaaat gataaaggtg gtattataac tgacccaatg gaaatacaaa agattatcaa     85500
     gacatactga atatgtctat gctcacaaac tagaaaatct agaggaaatc aataaatttc     85560
     tggaaatatt caactacccc ccaaaattga accaggaagg aaaagaaatc ctaaatagac     85620
     caatgcgagt aataagactg aatcagaaac tgaaaatctc ccaaccccaa aagcccagaa     85680
     ccagataaat tcaaggtcta attttaccag atgtaccatt cttactggaa ctattccaaa     85740
     ggattaaaga gaagagattc tttcccacct ttgtctaaaa tactcgtatc acctaatacc     85800
     aaaatcaggc aaggatacaa ctacaacaac aaaaagctac agggaaatat ccctaatgaa     85860
     catagataca aaatagtaaa cagaatacta gctagctgaa accaacagca caataaaaga     85920
     taatacatca tgaccaattg ggttttattc cagggatgca aagcttgtct aaaataaaca     85980
     catcaataaa tatgattcaa catataacat aattaaaaac agaaaacata agaccatctc     86040
     aatgaatgca aaaaagtcat tcaataaaat tcaatatccc ttcatgaaaa attcctcaac     86100
     aagcttagca tcaaagaaac ctacctcaaa actataaaag ccacatacaa caaccaacaa     86160
     aaaactctta aataaatcaa tagaacaggg atcttcttaa atttagtaaa aacctatgca     86220
     actgatttat tcaatatgct ggacttggta gtgaagaaat gaatgacttt aactaaaaaa     86280
     aaaagccaca tataacaaaa caacattata tggaatgaga aaaagttgca aaaatattcc     86340
     tgaagaacgt gagcaagaat gcacactttc accactccta ctggacgtat tactgaaagt     86400
     cctagccaga ataatcagaa aggaaaaaag aaatgtatgt ttcaagttcc aagctaagga     86460
     atccaggagt ggcagaccca gagacttgtt ttttaatcta tgaataacat ccaaacccct     86520
     gaccgatccc ttggaacaca ggatttaggg gtgattgagg ctctttcttt tgggttaaat     86580
     gaaagttgct aggtggaggg tgttaagtaa aaattactac ataagctgca tagttttgtc     86640
     aaatggttgt agttttcttg tccagcctgc tgttcctgaa ccaccctata tgaaagccct     86700
     caataaacta tatgtcttgt tcactggttt ggcctgttgt acacgatgcc agtcctattg     86760
     gagtccatat tggtctggcg tgacatccag tcaacctggt aaaaggtcca aaaaaaatct     86820
     catgagcacc cagacaatgg aatctaaaaa gaggagaccc ctgcagaaac cttgggcagg     86880
     ctgttaattt ctacgtgggg cccaacagcc gttatccatg atggatgggg cccagtgcct     86940
     gagtatgggg ataccgccac aatgccaaaa gttctggaag aacctcttca gtgggtgcat     87000
     cttacaaagt gaaagtcaga tgtccaggct gttgcattat caggtgagtg gttcttcctg     87060
     actgcactct gagaagccac tgagacagtg ctcactgtgt atgctacagt gtgtcagtca     87120
     caagaagagc tactcctaga aaaacatgcc agactagctc agactaatca gagacctttc     87180
     tgtcctgcct gtggaagcag aatggcaaat tggggaccgc cgcttgtgga gcagcacatt     87240
     tgaaggattt ctggccatca catgaattag ttacggctgt gatgactaaa ctgtcatggg     87300
     accctaagac ctagttccca taaagagttc tagggggagg aggatgaagt ttggggtaag     87360
     cccatggggg ttcctgtcct ctctacacat ctggtggcta ccaccaaggt aaaaatgggc     87420
     caactgcacc agcagaggcg agccccagag acatttccca cctgaaataa ggaaacacac     87480
     cacaatgtgg gattccacca ctgcggaatt gctgacactt agaaatgggt tcagacagaa     87540
     gaagagagag ttgatcacag ggtggcttct tcatctgtga ggcatgcggg aggagggcat     87600
     tatactccct gaactcaaga ttagtaaagt gacatccatc acaaaccatc tgggcctgag     87660
     gcagcacctc gatggtgtca gtcatggaga ttggaccatc cctctcctta gctgggtggt     87720
     tgcagactgc aagggaccct ggtgaaacga gggagatgtc cgctttatta gtgtgttctc     87780
     gtattgctag aaagaaatac ctgaaactga gtaattcaca tagaaaatag gtttaattta     87840
     ctcatgattc tgctggctgt atgggaagca tgatgaaaaa tctgttcagc ttctggagag     87900
     gcctcaggaa atttacaatg atagtgaaag gcaaaagggg agcagccact ttatatggtg     87960
     ggagcaggag caaagaaaga gtgagggtgg aggtcccaca cactgttaaa caaccaaatc     88020
     tcatgagaac tcatttacta tcatagcaag gggaccgtgc taaccattat tcataaatcc     88080
     ggacccacga tacaaccacc gggccccgcc tcaaacatta gggaagcaaa atcacataca     88140
     gttgttatga caggtgggac atcctgaaaa cctctctagg catgtcccac acggccctgg     88200
     agctgtctca ggggagcagt ctcctccagt gtttagacgc acaggcacgg ataatagggc     88260
     taagtctggc cagatgtgtg atattcaaca cattgcacaa ctgctctgtt ctgtatgtaa     88320
     tttatcttct ctacaaatgt aacattgatg gttgcattaa atatattctg caaatatgta     88380
     aaaataaaat aagatgatga ttgctaaatg attatcaagg cacaatcaca taatccgaag     88440
     ttacaatttc ctgagagata ggattactgc ccatgcttac caggacactc acatctgctc     88500
     tgggcactgc cttctcctca ggcgtcccac cccagagctt gctatatagt aggagacatg     88560
     caaatagggt cctccctctg ctgatgaaaa ccagcccagc cctgaccctg cagctctgag     88620
     agcggagccc cagccccaga attcccaggt gttttcattt ggtgatcagc actgaacaca     88680
     gaggactcac catggagttt gggctgagct gggttttcct tgttgttatt ttacaaggtg     88740
     atttatggag aactagagat gttaagtgtg agtggacgtg agtgagagaa acagtggatt     88800
     tgtgtgacag tttctgacca gggtgtctct gtgtttgcag gtgtccagtg tgaggtgcag     88860
     ctggtggagt ctgggggagg cttggtccag cctggagggt ccctgagact ctcctgtgca     88920
     gcctctggat tcaccttcag tgaccactac atggactggg tccgccaggc tccagggaag     88980
     gggctggagt gggttggccg tactagaaac aaagctaaca gttacaccac agaatacgcc     89040
     gcgtctgtga aaggcagatt caccatctca agagatgatt caaagaactc actgtatctg     89100
     caaatgaaca gcctgaaaac cgaggacacg gccgtgtatt actgtgctag agacacagcg     89160
     aggggaggtc agtgtgagcc cggacacaaa cctccctgca ggggcgcgtg gggccaccag     89220
     ggggcggtcg ggaaccactg aggacgggac aggtcccagg agcaggtgca gggggcggtt     89280
     tcctgtcagc tgcaggaggc gggtttgttt ttgcaggact ctggagcctt atgaggttgc     89340
     actattttat tatggttatt tatcatgtgt tttttattga tatttgtgtt ttagtaattt     89400
     ttacatttat atgtaggggt attttttaaa aatacataac ttcaagaaat aattcttcct     89460
     agtaatttac acctattcct ctaaagtttt attaacacct gttgatatca gcaactacat     89520
     agctatgcaa cattaattta catctataaa catatgtgtg aatacatgaa actatgcata     89580
     catgtgtagt catttatatt taacattata ataaaataat aaattaaata aaataattga     89640
     attaaattaa aagaattaaa cttaatgatg aactaaatat aaattagagt aatccataat     89700
     tgatttcaat ccttctctat ggtttacata aatcgatgtc tacttgtaag cttaagtata     89760
     gtgaattggt cattttaaaa tagccaagaa caaatttcaa atgttcttgt caccaaaaaa     89820
     tgataaggat ttgaagattt gaggtgatat atatgtcaat tagctcgatt caattattcc     89880
     acattgcatt cataaatcat aacatagctt tgtaccccat cagtatatgg ggtacaacca     89940
     aaatttctca atttccaatg aaattttaat tatatatttt ttaatttgat gcctctcttt     90000
     ggatcaggcc acctcctcag gattacagtg ctgttcagtt ttctcaacat gctgttgtac     90060
     cagataagca caaaacattt aatttcatta tgctttgctt tagtttttca aaacaacata     90120
     aaggtaataa ttttcccaac aaacatatta cagcctattc tatacttttt tattttttgt     90180
     ttttatgaga taaaatttac atgtaagaaa aaatttatag atctgaaatg tgtcactaga     90240
     gagtttctgg caaatgtgaa tacccttgtc ccgaacacct aaggtagact aaagagcaag     90300
     tccataccca caacatgcgt ctttctctgt gcctgcggtc aacccctaca tggggaagtg     90360
     ttttgatttc tgacaccata gatccatttt tttctgtttt gagctttata taaatggaat     90420
     caaacattat agaccttttt ttggtaaggg actactttgc tattttttag gttaattcat     90480
     gctatttaat gtatgaaatt agatcatcat agtgtcattt actcaattaa tgggctgact     90540
     ctgatatgaa acctcaaagt attcatgtat atatggagag agagagaggg aggaaggaca     90600
     gagattttat atctgagaca gtccattgag taaatatatg acaatatatt gatctatttg     90660
     ttgtcgatgg attttacatt tgtttccagt atatgaatat tataagcaaa gctgttacaa     90720
     atacattagt gtaagttttt ctatctatgt tgtctcaatt ttgttgagaa aatatgaagt     90780
     atgtttttct ttattgtaag aggaagttta atttttttca gttttattga ggtaaaaata     90840
     aaatattttt taaaatgtat aggtttaagg tgccccactt gacgttttga tgttgtatta     90900
     gtccattctc acactgctat aaaaaatgcc tgagactgag taatttgaaa aaaaatatgt     90960
     ttaattggct cacggttctg caggctgtac aggaagtgca gtggcttctc cttctgggga     91020
     gagtcaggaa agttaggatc atggcagaag acaaagggga gcaggtgtgt cacatggcca     91080
     gagcggaagc cacaggaagc ggaccggggt gtggggggac tgcacactgt tagacaacta     91140
     gatcttgtga gaactcaggc actgtagtga gaacagcacc aggagtcggt gctgaaccac     91200
     tcatgaaaga cccaccccac gacccagtca tctcccacca ggtcccaccc caggactgag     91260
     gattataata taacatgaga tttggtccaa gacacagatc caaaccatat catatgcaca     91320
     ttgtgaaatg ctcatcatgg ttaaggtaat ttgtatattc atcttctcac tattttttgt     91380
     ttgatttttg agacagagtt ttgctcttgt cgtccaggct ggagttcaat gtcaccatct     91440
     cagctcactg caacctccaa cccctggatt caagggattc tcctgcctca gcttcccgag     91500
     tagctgggat tacaggagct caacaccaca ccccgctatt tttttttttt tttggtactt     91560
     ttagtaaaga cagagtttcg ccacgttggc caggctggcc tcgaacagct gacctcaaga     91620
     gatctgtccg cctcggcctc ccaaagtgct gggattacag atgcaagcca cagcgcccag     91680
     ccaattttat tattttaaaa atgtactgca tgtgagtgtg tgcgtgtgtg tgtgtttcat     91740
     gagaacacct aagatctacc cttttagcaa aaatcacttt tacagtacag tattaaatac     91800
     aggaaaattg gcgtaggtta gatctccaga actcattcac cctgcacagc tgaaactctg     91860
     taccctctga ccaacttcac ccaatttccc tctcctccca ggtcctggga cccactattc     91920
     tactctctgc tttcaagagc ttgaatattt cagatcccac acgtaaatga gatcatgcag     91980
     catttgtctt tctgcatctg gcttattcca ctcagcgtca tgtcctccag gcccatccat     92040
     gttgcaaaag ccagaacttc cttcttttca aagctaaata aaattcagtt ttatgtgtac     92100
     acattttctt taaacattct ttaatctatg gtcattaaac tctttcacaa atctacacta     92160
     ttataaataa tctggcagtg aacatgtgtt taacataata attttatttc atttgaatat     92220
     ataacaagaa gtgggatcac cagatcgtac gataacttta tttttcaatt tattgagtaa     92280
     caagtcctac cacaccatat catgatttcc tttcccaaca ttcttgtgaa cacttgttat     92340
     attttgtctt gctgataata gtcattttaa gtggtttgag gcaatatttc atcgtgcttt     92400
     agatttgaat ttctctgaaa attagtaatg ttgggaacct ttttaggctc tgtttttcat     92460
     gagtgggttt tctgaaaaaa agtctatcca ggttttttcc cttttatcag gtcgtttgta     92520
     atttgcacat agtttttttt cctgtgcttt tggtattaaa ttcaaataaa tcatttctga     92580
     atcaatgaca ggaagttttt ccatgctgtc tatgagtttg tggtttcagg ttttgctcat     92640
     ttttagttga tttttgtaca tggtgtgaga gaaggtctaa ttttatttct tctgcatatg     92700
     gatgtccagt tatacatcat ttattgagga gactgtcttt cttcactgtg tctttttggc     92760
     atacaaaatc aggaagagac ataattaaaa aagaaaatat tagatgaata ttcgtgatga     92820
     acatagacct aaaagttctc aacaaaatat tagcaaatag aatccagaag cactttaaaa     92880
     tgtaacacat catgatcaag tgggctttac cctgggatgc aaagttcatt caacatccac     92940
     aaatcaataa ctgtgattca ttgtgtaagc agaataaaag caaaaaccat atgttatctc     93000
     aatagatgct gagaaagctt ttgatatgct ccaacactca ctcataataa aaaccctcag     93060
     caaactaggc atcaaaaaac atacctcaaa ataataagag ccatctatga ccaacccaca     93120
     gtcaacatta tactgagtga gcaaaagctc aaaccccatg agaattggaa caagacaagg     93180
     atgccctctc accactccta ttgaacatag cactgaaaat cctagtcagg gcaaccaggc     93240
     aatagcgaaa ataaaacgca gccgatatgg tttgtactag tgccccattc aaatttcatg     93300
     tcaaattgta attcccaggg ttggggctgg ggtctggtga agggtgattc tatcatgagg     93360
     atgggtctcc gtctcttgtg ctgctctcgt gatggtcctc aggagatctg gtttcaaagt     93420
     atgtgtcatc tccctcctct ctcttcctcc tgctcctgcc ctgtaagaca tgatgcttcc     93480
     ctttctgtca tgattgtcat gttcctgagg cccccccaag agccataatt cctgtacagc     93540
     ctgcagaacc ataagacaat taaaaacctt tatacattac acagtctcag gtgtttcttt     93600
     agagtgtgcg aatggactaa tacagaatca aaataggaaa agaagtcaat gtatctgtcc     93660
     aaattgatga tataattcta tacctagaaa attctaaaga ctctgccaaa ataattctag     93720
     aatagataaa caactttggt aaagtgtcgg gatacaaaat caatgtacaa aaatcactag     93780
     catttctgta ccccaactac atccaagctg agagtgaaat caagaacaca attctatccc     93840
     acttacaata tccacaaaga aaaggaaatg cctgggaaca cagataacaa ataagatgga     93900
     agatccctgc aagatgaatg acaaaacaca gcagaaataa gtcataaatg acacaaaaaa     93960
     tgggaaaaca tttcatgctc atgattggaa taatcaatat tgtaaaaatt gtcatactgc     94020
     caatagaaat ttacagattc aatactattc acataaaact atcaccatca ttcttcacag     94080
     aattagaaaa aaaagtatat atattctaaa agttatatgg aaccaaaaag cccctgagta     94140
     gccaaagcaa ttctaagcag aaagaaccat gacagaggca acatgatacc tgactttaaa     94200
     ctacaccata aagtcacagt aaccaaaaca gcttggaagt ggtacatgag cagacatgca     94260
     gaaaaatgga acaagagaga aaacagaaat aaacctgcac acataaacca tctgatattt     94320
     gataaggctg acgaaaacaa gcaatgggga taaaactctg tattcactaa acggtgctgg     94380
     gataactgga ttctgtcaga tatacagaat gcaaaagtga aggaggctgg gcagcttcga     94440
     cttagatttc agaggatatg tagagggccc tgcaggccca gcaggaggcc ggctgcagca     94500
     gtgcagccac cacagacagc ctctactagg acagtgccaa agatggggaa agagagggtt     94560
     ggagccccaa ttcacaatcc ccactagggc attttacact aaagctgtgg gaatgtggcc     94620
     acgaccctca aggtccctga atggtagaac caaaggcagc ttgcactctc agcctggaaa     94680
     aacttctggt actcgactct aacctatgaa tgcaggccaa taagctgtgc tcagctataa     94740
     ggatgaaaag cctgaggttt ctgggactcc ttcaacccag ggtgccctgg ttgaagtaca     94800
     tcaagtgaag cgagattatt ttgtagcttt aagatttaat atctgcccag acaggcttta     94860
     ggtgtgtggg gggcctattg gccattcctt tggacatttt tttccttttg tattaacaat     94920
     atttaaccaa tatctgtggc agtcttgcat gttggaagta aataactctt taaaacattt     94980
     tgccagctca tagctggagg gaaattgcct taagactcgg ataagacttt ggaattttca     95040
     gttggaggtt gaccaagtta agacttttgg aaactattaa aaaaaaagat cattttgcaa     95100
     cctctgaagg acgtaagatc agggggcaca agagtgaaat gatatagttt agatgatggt     95160
     cccctccaaa tctcatggcg aaatgtgctc cacaatattt gaggtggggc cgactgggag     95220
     attttgggac atggggaaaa atccttcagg aatggctcaa taaccaccct atggcagtag     95280
     gtgaattatt cctgtattaa ctactgggag atctgattat taaaaggagt ctgatgaccc     95340
     tcctcccctc tcacgtccac ccctcaccat atgacaccgc ctgcttcccc tttgccttcc     95400
     accataactg taagcttcct gaggccctca caagaagcag atgctggtgc catgctcctc     95460
     acacagcctg ccgaactcta agccaaatga gcctctttta tttgtaaatt acttggcctc     95520
     aggcattaat ttagagcaat gcaaaataga ctaaaacaca ctgcccaaag caatatacag     95580
     agtcaatgca gtttccatca aataaccaat ataattaagt acattttttt taaatgtcct     95640
     aaaatttata tggaagcaaa aaagagcctt ttgattccat ataatagcaa aagccgtcct     95700
     aagacaagga aacaaagctg aaagccccac attccctgac ttcaaattat actacacagt     95760
     tataggaaga aagacagcac ggtactggta ccaaaacaaa acaaaaaata ttagaccagg     95820
     tgtgcctgta atcccagcac tttaggtggc agaagctggt ggatcacctg aggtcaggag     95880
     ctcaagacca gcctgaccaa catggtgaac ccctgtctct agtaaaaata tgaaaaacta     95940
     gccaggccta gtggcccata cctgtaatct cagctacatc ggaagctgag gcaggagaat     96000
     cacttaaacc cgggaggtgg aggtttcagt gagctgagat cttgccattg cactccagcc     96060
     tgggtgacaa gagcaaaact ccatctcaaa aaaaaaatta atgtaacaga atagagagcc     96120
     cagaaagaaa gccaaatatc tacaaccaac tattctttga caaaactgac aaaaatatac     96180
     actggagaaa tagtcctcta ttcagtaagt cgtgctggga aaattggatg gccactcata     96240
     gaagaataaa accaggcttc tatctcacca cagacaaaaa gtaactgaat atggattcaa     96300
     taattaaatg tataaactga aactataaaa atacttgaag aaaatgtagg gagaattctc     96360
     gggacattgg cctaggcaga taaaatatga ctaagacttc aaaagcaaat gcaacgaaaa     96420
     caaaaataga tgaagaggac ttaattgaac taaagatctt ctgcagagaa aaagaaataa     96480
     tcaacatggt gaacaaacag cctacacaat ggtagaaggt atcacttcat ctcagttaag     96540
     agggctctta tcaaaaaaga aaaaaaacag ccaggcgtgg tggctcatgc ctgtaatccc     96600
     agcactttgc gaggccgagg caggtggatc acttgagatc aggagtttaa gaccaacttg     96660
     tccaacatgg caaaacccta tccctcctaa aaatgcaaaa attagctggg cgtgttggcc     96720
     agcacctgta gccccagcta ctcggtaggt taaggcaaga taatcgattg aacctgggag     96780
     ttggaggttg cagtgaggtg agatcacgcc actacactcc agcctgggtg acaggagtga     96840
     aactctgtct caaaaagaag gaaggaagga aggaaggaag gaaggaagga aggaaggaag     96900
     gaagggaggg agggagggag ggagggaggg agggagggag gaagggaaag aaagaaagaa     96960
     agaacgaaag aaagaaagaa agaaaaagaa attaagaaag aaaaaaatta agaaagaaaa     97020
     gaaagaaaga aaaagaagga aagagagaaa aaagaaagag agagagaaaa aaggaaggaa     97080
     ggatggaagg acagaaggag ggatagaagg aagaaaagga aaggagaaag aaagaaaaaa     97140
     gagaaagaga taaaaagaaa taaagagaga aagaaagaaa actaacaaat gctggtaagg     97200
     ttggggagaa ggtaaactag tacagtcagt cactatggag agcagcgtgg aggtacctca     97260
     aaaaactaca aatacaacta ccaaacgacc cagcaattcc actactgcaa atgtatccaa     97320
     acgcatgaaa ataattatat cagagatttc ttactcccag tgtatatggc aggacctttc     97380
     acaatggcca agatatgaaa tcaaaccagg tgtcagagag cagatgaatg gttaaagatt     97440
     atgtggtgca tatacatgat gagatgctca tcatccacag gaaaggaaat cctgttgttt     97500
     ttggcaatga agacggaact ggaggacatt atgtgaaatg aaataaacca ggaacttcaa     97560
     gtaatacacc ttgttttctc actcatacgt ggaaggaaaa agaaaatttg tctcatagaa     97620
     ataaatagta gaacagagga tactagagtc tgggaagtgc aggggaagag ataggcagag     97680
     atttgtgaag gaatacatta gagttaggtg ggaggaatag attataatgt tctttaccac     97740
     tgtgggataa atgtatttaa caattatatg tagtttcaaa cagttagaag aaggatattg     97800
     agtgttccca acaaaaagaa aatgtttcag atgacggata tattaattat ccttatctgt     97860
     cccctacaca ctgtttcaca acatcgctat gtacctcatg aatatttaca atcattattt     97920
     gtctattaaa attatattag aaaacacaaa attcactgga agtaacataa atggaggctc     97980
     tgggatagac ctggagataa cggagggtct gggatacatt acaggcctag cactcaatgg     98040
     tcagaggagc tcccattagc tttttgtatt tgtagcactg agtaaatcta acatttctca     98100
     aagcttccct ttgcaataga gacgtgaggc ctagggagat cttgcagata gtcagatatg     98160
     cagagggaag aagcaggggt agagggttgc tggcccattc aacaatagtt ggaatgataa     98220
     ttacgcatgt agagaagcca acattgattt aaataagttg catcactgaa agccatgaaa     98280
     aaaaatccaa aggaaatctt taaataaaat tgagagtgaa actgttttgg gtgaagaaat     98340
     gttcactgaa gttaggtgtg ccagtaaagc tgttaagaac tacagtaacc actggattgg     98400
     attcagcttt gtctgaattc agtgtgatta ttactggctc aggcaggggt cagaaatcaa     98460
     catggatagg tggtagcaac acgaaaaagc cagggaccag aaagattcac agccgaattt     98520
     tttcagatgt ataaagaagt gttgctacca atcctagtgt aactatttcc caaaaatgag     98580
     gataaaggtc tcctccctaa attattccat gaagccagca ttattctgat aacaaaacct     98640
     ggcagagact taataaaaaa acatgacttt agaccaatat acctgatgca aaaatgctca     98700
     atgaaatact cacaaactga atacagcagc acatcaaaaa gcctaaccta ccaacacttt     98760
     gttcctggga tgcaaggttg gttcaacata tgcaaatcag gaaatgtgat tcatcacata     98820
     aacagaacga aaaacaaaaa ccatgttaac cggcagggca cggtgactca tgcctgtaat     98880
     cccagcactt tgggaggctg aggcgggcgg atcataaggt caggagctcg agaccatcct     98940
     ggttaacatg gtgaaacccc gtctctacta aaaatacaaa aaaattagct gggcatggtg     99000
     gcgggctcct gtagtcccag ctactcggga ggttgaggca ggagaatggc gtgagtccag     99060
     gaggtggagc ttgcagtgag ccgagatcac gccactgcag ttcagcctgg gcgacagagt     99120
     gagactctgt ctcaaaaaaa aaaaaaaaaa aagaaagaaa acgttaacca tagacacaga     99180
     aaaggttttg aaaaaacttt aagattcttt catgttaaaa accatcaacc aggcatgaag     99240
     gaatacacca caaaataata agggccatct atgacaaact cacagccaac attatactga     99300
     ctgagcaaaa cctggaagca ttttcccctg aaaactagaa caagaaaagg acacccattc     99360
     taaccactcc tgttcatcat tgtcatggaa gtcccagcca gagcatgcag gcaagagaaa     99420
     gaaataaaaa gcatcaagtc aggaagagag ggtgtcaaac tatccctgca gatgaaatga     99480
     tcacattcct gagtctgtag tttggtgact gacattaatt taaaatattg tcagtcatca     99540
     ttgtttaaat atttcttctt agcctttctc tatttttttc tatttcccat tatgtgcatg     99600
     ttatacctct gtcactgtct caccattctt gggtaagctg ttcttctttt gtatatatgt     99660
     gtgtgtgtgt atatatatat atgtatatat gtgtgtatat atgtatatat atatgtatat     99720
     atatgtgtgt atatatgtat atatgtgtat atatgtatat atgtgtatat atgtgtgtgt     99780
     gtgtgtctgt gtgtgtgtat atttgtattt gtgtgtttac tgttctaaca atatattttt     99840
     tgtttttgct tttcagtttg ggaaatttct attgagatag ttttaagttc agatttttgt     99900
     ccgcagctat gccccatcta catataagtc cattgaaagc attacttaat tctgttacaa     99960
     aattcttgat atctactttt ttttcctttt ttggaatttg cttcattcta cttgcatgac    100020
     acagctgttc cagcatgctg tctgcttaat tcattacaga ccttagcata ttaatcacag    100080
     tggtcttaaa ttctttgttt ggtaattcca aactaaatgg catggctgaa tctcatttta    100140
     atgcttgttt tgtcttgtca cactatgata tatttctttt agtatatatc atgattttta    100200
     tagggagaaa ttatgtatca ggtgaaagaa actgctgtaa agaggccttt agtgatatca    100260
     ttgtaagttg tggagagaaa cctgtttcat aatcctctgg ccatggcttc atctaggaag    100320
     cttaaggcct ttgaaaataa atccttacat gaacgtgaaa agataagcca cagatatgga    100380
     gaatttcctc acaaatcaaa tatttggaaa aggactttta tcaggaataa taaaaattac    100440
     cttacagttg aatgacaaaa ccacatgatc taattttaaa atagacaaag acctgtatag    100500
     aaagaagcct catctaaaaa taaatataaa aagtgatcag catgattttt aggtatatgt    100560
     atgtttaata ttcaaaaaat actatatcct gttagaatgg gtaaaataca cattttagac    100620
     agtaccaaat ggtgatgagg aagttgaaaa acaggaacat tcactccttg tgggtgggaa    100680
     ggggatgctg aagaggcaca tacacaacta cagttagtac tttattgtat atttcagaat    100740
     agctagaaga taagatttag aatgttctta acacaaagaa gtaatcagtg atggaggtga    100800
     gggatatccc aggtatctgg atttgatcat tgcagataac atgcttttat caaaatatca    100860
     tgcgtatccc ttaaaggttt tgaactgtta tgtatctata taaattaaac atttttaaaa    100920
     aaacggtgca ggggaatcca gctaattgca gattcccatt agatgggatg tgctgcataa    100980
     tctgtaaact aatcagagtc tgtgggtttg taaaggactt tagaaatcaa ctggactact    101040
     tgtttctaaa gataagtctc gcaagtttaa ttattttctt aatgggtggc gattttgaac    101100
     atagaagaaa tgttaacagt tgtttctcaa gttttcttct agtttcagaa gaaaaggttt    101160
     gcagggagga atctgggacc atctttcaag tctcaacttc aatgtgatta tctcagtaaa    101220
     tattttctga tacccctgag cagggtaagt ctcatatatg tcctagaaac tgcaagtaga    101280
     attctaggca ccccaactga ctgaagggaa cttttctctt caacgaggag ttccatggac    101340
     actagaaaac ctagtttagg ccgtgtcaga aatggaggat cagaaatccc tcattatacc    101400
     ctcctccctt tggaattcaa gcacaagtaa ccagcatttt ccttaaaaca gatttaaggc    101460
     tcagaaaaca gattccgtgt agcagtaaaa caccaaattc taacctgact caagaatagc    101520
     atcacatgac agagaaaagg ccttggaagg aatcaaagtc ttttacccta aaatatatgt    101580
     ctttgacata tttttaaatc accctgcaca gttatctttt tgcaagagaa atttacattc    101640
     tgtagagaat cgcttccctt tgaaggtgtt tgtgttatac tgaggtatcg ggggaaattc    101700
     agccagatac tgggagaaat tcacccccga tatttcacgt agattctttt ctattttcct    101760
     taagtgtcag ccggtctgag aaataaaggg acaaagtaca aaagagagaa attttaaagc    101820
     tgggtgtctg ggggagacat cacagttcgg caggttctgt gatgccccct gagccgcaaa    101880
     accagcaggt ttttattagt gattttcaaa aggggaggga gtgtacgaat agggtgtggg    101940
     tcacagagat cactgcttca caaggtaata gaatatcaca aggccaatgg aggcagggcg    102000
     agatcacagg accacaggac cagggcgaga ttaaaattgc taatgaagtt tcaggcacgc    102060
     attgtcatcg ataacatctt atcaggagac agggtttgag agcagacaac cggtctgacc    102120
     aaaatttatt aggcgggaat ttcctcgtcc taataagcct gggagcgcta cgggagactg    102180
     gggcttattt catccccccg ctgcgaccgt aaaggacagc cgccccccga agcggccatt    102240
     ttagaggcgt ttagagcgtt tctgtcttct ctcccagttt ctacgagttc ctccagtgcc    102300
     tgtaacacag agctgttagt ggctttgccc ctgcctgtta attctttggt catgtaatgg    102360
     tggtgaggga gctgggtctg gatgcatttc agccacagct gctgttccac ttccccacac    102420
     agaaccatct cgggaggaag aggttggaga tttttctgat gtgtcctcaa tcctggcaga    102480
     aaaggtttca atagcaatag gattccctca gttgtatgtc ctctgataat ttaaacaata    102540
     acttctttat tatactcaac tctaaacggt ctaaagagta tgtctaagtt aaccctgcat    102600
     tcgcttatat gacatttgct ctaccccaga taaggtcata ttctctttct gtttgttcct    102660
     gcaagtcact gactttcaca agatgtaagg tcccttgttt gtcctataac atcaataatc    102720
     tgatgtttta aagaaaatat gctaatttgc agctcagtaa gtttagttgt aaaaataata    102780
     catttttttc tgagatgcct acatctccat gctgagttgg agctttattt gtaacactca    102840
     gaaactggaa ataacaaaaa tattaaggag ctttataaat agtacgggca cactcattaa    102900
     ctggaattct gttcctcatt acaaggaaca tattctttat acacaaccaa actactgact    102960
     cacaagtaat tgtgctgagc aagaagccac acaaatgaaa tggaaactgt aaggtttcac    103020
     tttaaccaaa ttttgaaaaa tagacctgaa gtagcaaaca tgagagactg actcagtgta    103080
     gtgagcggaa aatgactggg aggcaggaat tagaggaaac tagggaaacc ttgaagtgta    103140
     attcattgtt atcttgattg ttattttgga tgcacaggta agcacatgtg aaactgcatt    103200
     ttttcctttg gagacatggt cttgctttgt catccaggct ggagctcagt ggtgagatca    103260
     tagctctctg cagcctcaga ccctgcgtct ccaccaatct tcctccctct gccatacgta    103320
     gctgggacta ctggtatgta agacaatact tggcttcagt gcttattaaa atgcacactt    103380
     taattaagca gtatttgtta tacagcaatc atgcctcaat aagagtattg caaataagtg    103440
     gatagataat ttgttcaata aagattgatg gaaagataga tgctaacatg agaaaatgca    103500
     tgacacccaa gaaaataaca ctgtaggaaa catgcttttc tttacaattg ttaggtaatc    103560
     acaacagagc atacacatca caccatgttc tcgttacaga gaaaaggttc tgcaaacctc    103620
     actagctgca acccctgtgt gctgggcttg gttcagggag aagtcaggtc cagtggtgag    103680
     aagcacaggc ccagataacc aagctcactc tgaccgaatg tgagccctgg gtacattgta    103740
     caacccatct gtgcttctgc tggtaatttt tcatctgtaa catggaaatg acattgatac    103800
     tatataccat gtttcctctg catatgtaaa aataaaaggt gattggtgct aactttaaat    103860
     atacacagtt tatgtacatt gattgcacct caatacaaca gttttaaata gatattacaa    103920
     cattataagt tttataggtt taatatttta tcacagaaca aactttcaat aagaaaccac    103980
     aatttccaaa tgctatcaat atcacaaatc tcccccagga cactctcaca tgctctgagc    104040
     cccactctct cctcaggcgt cccatcccag agcttggcat gtagtaggag acatgcaaat    104100
     agagccctcc ctcggcttat gaaaaccagc ccagccctga ccctgcagca ctggaagagg    104160
     agccccagcc ctgggatttt caggtgtttt catttggtga tcaggactga acacagagga    104220
     ctcaccatgg agtcatggct gagctgggtt tttcttgccg ctattttaaa aggtaattca    104280
     ttgagaacta ttgaaattga gtgtgagtgg ataagagtga gagaaacagt ggatttgtgt    104340
     ggcagtttct gaccagggtt tctttttgtt tgcaggtgtc cagtgtgagg tgcagctggt    104400
     ggagtccggg ggaggcttgg tccagcctgg ggggtccctg agactctcct gtgcagcctc    104460
     tggattcacc ttcagtgact actacatgag ctgggtccgc caggctcccg ggaaggggct    104520
     ggagtgggta ggtttcatta gaaacaaagc taatggtggg acaacagaat agaccacgtc    104580
     tgtgaaaggc agattcacaa tctcaagaga tgattccaaa agcatcacct atctgcaaat    104640
     gaacagcctg agagccgagg acacggccgt gtattactgt gcgagagaca cagtgagggg    104700
     aagtcagtgt gagcccggac acaaacctcc ctgcaggggt ccccaggacc accagggggc    104760
     gccagggaca ctgtgcacgg ggctgtctcc agggcaggtg caggtgctgc tgagggctgg    104820
     cttcctgtga tggtctgggg ctgcctcatt gtcaaatttc cccagggaac ttctccagat    104880
     ttacaatcct gtactaacat ttcatgtctc taaatgcaaa acttttttgt tctttctgta    104940
     tttttgtttt tgtaacagga ggacacattc tcgcctccac agaagccaca gtgtcacttt    105000
     gggggcagat gatccttctg tagtcagcag gatgaaagtg cagaggaatc tcagtggaac    105060
     ctgggaagtc ttttccagtt agactcaggg cagagacctc catgggaatc tctgattaga    105120
     acaggctttg agttctgata ggagccaaga gagaggctca cccagggtca gagtccttaa    105180
     aacctgacgg ttttcacagc tatctcacct cgtcttgaaa aactgtgcac atctgactca    105240
     cactaattca gttgacctta tttctgctaa tctattttcc ttctctgcag acttgattct    105300
     cacagttccc tttcttcttc tcttgcctga aaacagagga tgtgttttct gtagtcaaaa    105360
     tcccagagat caggtctgca ggacctgggt aggctgaggg gactttctca ctcaccatag    105420
     cctggtgaca ctcctgctgt attttgtatg tggaggtgtt tggaaaacga agtgaacatt    105480
     agtcatgaag ggaataatac tagttttctc caaagggatg tggatgtaga gctgatcttg    105540
     tgcttctcac actgtcacag aatttatgct ctcccctgtg actttaggag agctgaggat    105600
     ggacactcca ttgtgctgtg agctctggta atagtaatta tagggtctcg ctaggcagcc    105660
     taaggtcaat actgctggcc ttcgggaaag gcaggctggg attcctagga aaacctgcat    105720
     ctgccatcca gcatggagtc ccatcgtctt ctgttatgct ctgattgaat caggcacacc    105780
     taggttatct agaacactct tcgtgacttg ggaaaaatta gtggcaggct ctactaagac    105840
     ctgcattatg ccatgggagc aacacctagg ctagtgtgtg attgagtagg tgagactatg    105900
     gtctagtcaa ggtgacaggg aaaattgttg ccattattat gttttatttt atatttggca    105960
     atatagtcat gctcatatta caaatatctt tctacatttt ttgtgtcaga agcttttgaa    106020
     caagagcaac ttcatcttga atagaggcta ggaaaaataa gactgagacc tgctgggcta    106080
     cattcccagt aagttaaggc gttcttagtc acaggatgag ataggaggtc tgcacacgat    106140
     ccaggttata aagaccttgc tggtaaaggt tacagtaaag aagctggcca cagcccacca    106200
     aaaccaggat ggcaaaaaag tgatctctgt tcgtcctcac tgctcattat aatgccttaa    106260
     catgctgaaa gccactcccc tcagcgccat gacggtttac acatgccatg gcaacatcag    106320
     gaaggttccc tagatggtgt acaaaggagg ggaggacacc tcagcttcgg gaattgccca    106380
     ggggactcat gaataatcca tccgttgtga gaaatacatt catccctttg agagaaaatg    106440
     cacagggggt ggaatgaggc tgggaagccg atggcacatg gtggaagcct gtccctgagt    106500
     gaaagagagg gggaagctgg attgggtgga aggtccctat atttctgtgc tgtgcaaggg    106560
     aggggcaaaa tataattgag tctcttgtaa gtcagtgttt cctctcaggg gatccccatg    106620
     actcccagaa ataatggctc tgcttaggaa tccctgcaga gtcactcctt tcattagagt    106680
     agaccacagg acatgggcct cagcacacgc catgccacag atgtcagaaa gcagctgctg    106740
     ggaacctgac ccacctgcat tttgctgcct gtaggaggag ggagggacgt gcattctcag    106800
     ggccaacaca ctgtttttgg atttttatag agaacacctg cttttacttt attttttgga    106860
     aaaatatatg aacaaatgtg cacctgcaaa caattgtaat tttcacattt attttaattg    106920
     catttgttta taattgtgca tgaggtcctg gtgcagagtt gagtttttca tgggaattgt    106980
     tccaagaatc aagaacatcc attttccact ttcactggtt tttccccatg tgcagagacc    107040
     ttgagtagag cacatctggc ccttatccac actatctctt gtgtccccgg gaaagagcag    107100
     ggatttgcct gactgcagaa tctagggtag gagtctgcac acgtctgagc ctgcagagaa    107160
     gcccagtgaa gtttaatgaa gtcagagagc tttcccgtgt ggggaacctc tagcttcctc    107220
     actctcagag attgctggtc agctgtaatt gaggggttaa gaattagagc acctgggagc    107280
     ctgtctcatc acagctccat tctgtaactt accaccatca gactgcaggt aataactcaa    107340
     tcttgatttt tctgacccca tcttattcat ttgtaagttt ggttatacaa aatgttacat    107400
     tttagtttct gattcaaacc tcagaatttt gtgaattcat caggaataat taaaaatgtg    107460
     tccagttttt gggaaatgta aattaatgtc catactgttt atgtgtgtgt gcgtgtgtgt    107520
     gtggggggat atgtcagtca cttacacccc agtaaaaatg attccttaat tatatagtcg    107580
     aactgcaatt agatttgctg aaaattgtct acagtaattt ctgtgaagct gaccttgcca    107640
     ccttgctgtg aggatgctca gtgcacaggt gaggtctcac aggagagatc caaggttcag    107700
     cccccaggac tggctgagct cctggcttgg agccagacat aggactgggc atctcagaag    107760
     cagatcccct ggccctcctt tatctgggct gtatgggcag cagaggacaa gcagaagaaa    107820
     ctcagaattc agccaagaca gagccccatt taggagcagg tgcatcaagg aaccagccag    107880
     agaacctctg actacaatct agggttgtct tcttttgctt cacagcatcc agtcctaagg    107940
     agaccaccca ctatatcatc tgtaactcag aactttcttg ttacaaagga aaacacaaat    108000
     gaatataact ctgcagttaa cttcttgaac ctggattgat gagtttccac ggccttatga    108060
     gcacagagac cacatccagt gcaggtgcag agcaaggctg caacagtcag cctgctgctc    108120
     cctcaggagt cttccaattc ccttgtccaa agcatccgct cattcatggg ctgagaaaag    108180
     ggaagtcatt catgcagtct actctctcca cagaactgac ggggcacaag gacaacatcg    108240
     atttttcatg gaacatgcct ctaggaatgc agctgtgtgc acacacactg ctaagcacac    108300
     acttctttac ataattactt gtaactgtat tttcttattt attctctcca atttttttac    108360
     acaaattcat cacttttccc cataatcaaa gaggattttg atcagaatgc ttgtggggag    108420
     ccccttgccc cccagatgcc caccatcatt attcttgaag ggaggaggaa cggcagctct    108480
     cttgatttct actctaatcc tctaggacta aaaccagaag gttgcatgtc cggtgcggga    108540
     gcatcgaaga agatcctgtc tgtagaagca ggagcgtcaa gacttgactg agagccatgg    108600
     tgctgaaatg agatagattc cctgatggag agcacacgtg gacccccaca cctgagggct    108660
     cactgctcct caccacagat gcactcccct actgagtcct gagacctgag tgcaccccat    108720
     agagtagggc tcagatgagg ggatgcaaat ctccaccagc tccaccctcc cctgggttca    108780
     aaagacgagg acagggcctc gctcagtgaa tcctgctctc caccatggac atactttgtt    108840
     ccacgctcct gctactgact gtcccgtcct gtgagtgctg tggtcaggta gtacttcaga    108900
     agcaaaaaat ctattctctc ctttgtgggc ttcatcttct tatgtcttct ccacaggggt    108960
     cttatcccag gtcaccttga gggagtctgg tcctgcgctg gtgaaaccca cacagaccct    109020
     cacactgacc tgcaccttct ctgggttctc actcagcact agtggaatgt gtgtgagctg    109080
     gatccgtcag cccccaggga aggccctgga gtggcttgca ctcattgatt gggatgatga    109140
     taaatactac agcacatctc tgaagaccag gctcaccatc tccaaggaca cctccaaaaa    109200
     ccaggtggtc cttacaatga ccaacatgga ccctgtggac acagccacgt attactgtgc    109260
     acggatacca cagagacaca gcccagggcg cctcctgtac aagaacccag gctgcttctc    109320
     agtggtgctc cctccccacc tctgcagaac aggatagtgt ggctgagatg ccatttcctg    109380
     ccagggcctg cgtttcccat ccccatctga ctcagagcct tgttttcctc cctcttcttt    109440
     actaataaat ggcatgtccc ctgttagtgg ttcatgcaag cagaagctgt atcctgtttg    109500
     acaaagattc agcatgaaag gtcctgctac ctaaaaaaaa atagacagat gagacttaat    109560
     taacctaaat aatttttttc acaacaacag agtgaatacg caatttacag aatgacagaa    109620
     aacttttgca cactttgcct gtgacaggga actaatatga agaatttgca aggaactcaa    109680
     acaactctac aacaacaaca gcaacaagaa ccaaataact ccgttaaaat gagcaaagga    109740
     catgagtaga cattttcaaa agaacacata gaaatggata ataaatatat aaacaatact    109800
     caacatcact aaccatcagg gaaatgcaaa ttaaaaccac aataagatat catcttccac    109860
     cagtcacaat gactattact aaaaactcaa ataatatcag atgttgctga ggatgggaaa    109920
     tgaaggcaac tcttagacat tgttgatgag gatgtagatg agtacaacct ctgtggaaaa    109980
     tggtatggag atttcccaga aaactagaaa tagaactgtc atttggtcca gcaatcccac    110040
     tactgggtaa ctacccaaag gaaaataaac tattatttca aaaagatacc caccttctat    110100
     gcttaccata aaactactct caatagcaca tatgtcaaac tgagtgtctg ccaaccgatg    110160
     attttataaa agaatatagc atgtatgcac aattcaatac tagtcagcca caataaggaa    110220
     tgaaactgtg tcttttgcag caagatgcat agaagtgggg gacaatataa ttagtgaact    110280
     aactcacaaa cagaatgtca catgtcacac attattactt gtaagtggga ggtaaacagc    110340
     gtgtacacaa ggatttgtag agagaaatta cacacattgg agacttacaa ggatgggcgg    110400
     gcagaaggtg ggagcatgat gagtcattac ataacaggca caatataaaa taattaagaa    110460
     ttgaccaatg atcttaaaat taaaatgtag aatatgatca ataaatgaac ttgatattag    110520
     ttgacctcat taaatttaaa aactttttct actcaaatga ctgtaagaaa atgaatgccc    110580
     ggttacagat gagaaactgt ttgcgagtca aataaccacc aatgtaacta taataagaaa    110640
     cttcagaact caactgtgaa taaaaaagaa acaactgatg gataaattag gcaagggttt    110700
     ctacagacat ttcgtcaaag aagatgtgca gatgacactg aagcatataa acaggatctc    110760
     aacaggattt tccgttagag aaattcaaat caagcccgca aagagacacc actgtacact    110820
     ttttaaaatg gctgaaatta agaagaaata cagataacat caatgctggt gagcatacca    110880
     ggttgctaga ggctaaaaca ttgctaacag gaatgcaaaa tgaaacagat actcaggaaa    110940
     ataattttta gttttctcta aaatcaaaca tacccttaac acctgaatat ttgcatcaga    111000
     gaaaaacaat cttacattca cgcataactt ctattcaaat attcaagata tcgtgtgtat    111060
     gtgtgttaga aagtaaaaat aacataaatg tctcaaaatt tgaataggtg aagaactagg    111120
     aagcatctat aaattgaata ccaccagcaa taaaaaaata acaagtgacc gatacataaa    111180
     ctattacagg tgaactccag acattgtgct aagtgagaga agccagtctc aaagatcaaa    111240
     gggacacagc tgtaagcacc acggtcatcc tcaggtgtca gtggtttggg ctggactttc    111300
     tgtgtctctt tcctgaccag acccagatat tgagctccac cacttgcaga tggaaaatcc    111360
     tattttcaac catgcagtga ggtttgaact gcttcacaga ctgaacgaaa caaacacggg    111420
     ctcctttgaa cagcgtccgg catttgttcc aaccacaaga gaacgtccct cagctctccc    111480
     acctcctcgg ttctctcctg caagccagca gccctgcagt ttagcctgca tctcccgtgc    111540
     atccacccat ctccctccaa gcaccttccc ccacaccctc cactgtttct gagagcacag    111600
     gcaggctttg aacttttccg cattctgttg ttattgaagt taggatgttt aggaccaact    111660
     taaggatcat attttatgac tgaattccag tgccccttct ctcctgggac agagtgcata    111720
     accaagtttc tgcaggtgga gacgaagttg agcttttttc ttcctcagcc taggagatga    111780
     gcgctaattg gagggttggg cagaagcttc ccaccatccc agcactttgg ttctggtggg    111840
     gcggaatcgg tgccataggg cagagctaga aaccgcggac tgaatgttcc cagtggcact    111900
     ggacccaggg cagagcctcc atccacgagt ggggctctat ggaagaagtg agtctctggc    111960
     tctcagtagc tctcgtccag cactgaacct cagcatcatg tgctgtgtgc agggtcagag    112020
     ggccaacgta ctggcccctg ggaaagcgtt tcctctggtg ggagttggta gaaggtgtcc    112080
     tgtcttcttg gctgcatctg tccgcagtgg agtttacatc atgctgagct gggatgtgga    112140
     aggaaggaag agcatcttag atcaaatatg atgactggcc ttactgagtt ttctagattt    112200
     tcctgaataa atgtttcttc actcactgtg tgctgttaga gtctttccaa acctgtaatt    112260
     tcccaaaata attttcactg gtctcatgag ggcatggatt cattgagccc ctcatgctgt    112320
     caaagagaaa tagaactgtt tttttttttc acttcatagc gaacatccat gggttatcaa    112380
     ataatgggct ggcttttctt ccaacacttt acagacacca tcaattttct tcttgcttat    112440
     aaggttttaa ccagaagaat gctgtcatgg tcttttctgt tcttttggaa ggaatgcccc    112500
     ctctactcac ctccacttgt ctgcctgtat ttctatttgt ctttggtttt caacaatttt    112560
     aataagattt acctaaatgt gtgtgggggg agcatggggt gttattctgc tgttctgtgt    112620
     tctctgagat gcatggattc accatttact ctgtctccat ttttgtgaaa acaattagaa    112680
     aaaaagtcag tatgagccca gaaacaagcc tccctgaagt gggcacagga ccacctgggg    112740
     gcgctcagga cccactgaac acaagagcca gccccagggc aggtgcagat gcgggttaag    112800
     ttctggtttc ctgtcaaccc tgtggcttcc tctccataaa acagtttcct ttgtggcata    112860
     tctctggatt ccttatcctg ttcttcctgt gaagtctctg aagaagaaac atttgtcgta    112920
     acaagagaaa aactttctca catgcaccaa aggcagagtc acctacagtc acttactcct    112980
     gtttctcaat gtcaataagt taccaatgct tctgaagtta atcagctaaa tctataaaag    113040
     gtgcggtgtt taactcagca ttacagccca gctcaacaga actccaaagg tcagccagca    113100
     gcagccagga aaaagtgcat gctgggcatt ggggcagagg gagttaccat ccagtgcaag    113160
     tagccttata attaggtgaa cttcggaatt taatcttcag ttgtgttaaa taaaaaaaaa    113220
     aaacacaatc tacaatttag gaaagtgaga gtttattttt atcaagggtt acagccatcc    113280
     catatcctgg aaagcatagc tcttggtaaa gacgagagaa aggcactccc aggaagaagg    113340
     ggttgggcag aagctttatg ctgaagggtt tggctaaaca gacataatca acaggttaca    113400
     ggaggggcta tagatgttca tggaggtggt cctgacacat gcatactgaa caaacatgtc    113460
     tgtaacatat gacccctgtt cacttaccag tggagcctta gcatttaaat tcattccagt    113520
     gaggccctat gtgcaaacag cacaagcaga gacacaaagg cactcagtgt gcagcttctg    113580
     taaacggcca gagccaggcc atggtcagtg gtcttggatc aggagaaagt tcctgatatc    113640
     actctagtgt tcaatcaaag ctggggttat ggtttgcgga acagggggtc agttcatcag    113700
     ggggtgggct gcaattgtct tcatagtgct tgtctcagtg ccggtgctta ctgagccact    113760
     agagaaaaag gtttaattga gcttctttaa aatcaacatt ttgaatgatt tatcagacat    113820
     ttcaaatatg tcatgttgtt tagattctat tgctggagag ttaaggtgat atttggggtt    113880
     ttgtgactct gttttttcat acttcctgaa ttgcttatct gtttgctttt cattagctaa    113940
     actatcgctt cttcttattt tttaattcat tctgattttg atgaatattt aattcccttt    114000
     agaatgtgaa tataatgtac attgtgtggg tattttgatt ttggttcttg gtttacttag    114060
     tggcaaagac tctgtaagag ttccttgtct atagatagcc attatttagt ggctttctga    114120
     aatggtggtt ttagtaccaa agtactggac ttgtgagtag gctcactgcc cgctgcaggt    114180
     cctagatagt ggaggcctca ggaactgttt ctcatttgga atgcctttgt ttcagcagat    114240
     tttgtgttgg gttgttaagt tcaccctcca cattagtaga tgtcttacag attagagctg    114300
     actctggtag aagcagttga gtacatgctt gatatctgtg cacagggaga agctctctgt    114360
     tgcctcaggc gatggactgg tctatgaaat gcacagtgac ctgagttccc tgctcagccc    114420
     ctgagaggtg gaccaagctg gacacacatg agccaccgag cctggcaagc aaaagcgcca    114480
     gccttgatgg aaatggcgag ctgaggggca tctactcagt gtggtttctt ttgttattaa    114540
     gagctttagt gtggtggctt tttcaaattc ccgttgtagt ggtaatatac tgggtatgtg    114600
     agcaggcccg tggtcttttg cggggttgga atcaccgaag taatgagaag ctaatctcat    114660
     tttcaactgc tgtacactgg tggtattgag tttgtatgag gtcatgcagt ttgaacgtca    114720
     ggccagtagg tggtgctcgc aggtaagagc cggctatggt ggcagcagaa gggtttatgc    114780
     tttaccggtg gttaaagtgg gaaacttgtc gtgttccaga tcttagagaa aagactttta    114840
     gttatttctc attcaacctg atactacctg aaagtctctc gaatgtaact tttattttgt    114900
     cgcgatgggt tctttctata cccatttttt atggtttttt tatgaaagga tgttgtttca    114960
     tcaaatgcgt tttcagcatc aattgaaaaa aattatatgt tgattaaaga tcaaaatgta    115020
     taacctaaca ctataaaacc tctggataat aacctaggaa acagaattta ggaggtaaga    115080
     actgacaaag gttttataat gaaaatgcta gaagtagttg caacaaaatt gaaaattgac    115140
     aaatgggacc taagtaaatt aaagagcttc tgtacagcaa aagatactat caacagagaa    115200
     aacaggcaac ctacagaatg ggaaataaaa tatttgcagc ctatacatct gacaaaggtc    115260
     cgatacttag tatatacatg gaaatttaac aaacattcaa gaaataaaaa gtgaccaaag    115320
     gacatgaaaa gacacttcaa aaaagaccta catgtggcca acaagcatag gaaaaagtgc    115380
     tgaatatcac tatcattaga gaaatacata tcaaaacctc aatgaggtac cgtctcacat    115440
     cagtcaggat ggctaatctt aaaaaaaaaa taacagattt ttaaggttac agaaaaaagg    115500
     ggaaatttat acacttctgg cgggaatata aatgagttca accattgtgg aaagcagtgt    115560
     ggtgatccct ccaataacct aaaacagaag tttcatttga cccaacaatc ctacaactgg    115620
     acatatacct aaaggaatat aaacatgtag gttcactgca gcactatcca caatagcata    115680
     gacatggaat ttacctaaat ccccatcact ggcagaatga tagagaaaaa tgtggtacat    115740
     acaaccatgg aatactatgc agctaaggaa agaatgaaac tatgtccttt gtaggaacat    115800
     gatggaactg gcagtcaata ctcttagaaa actaattcag gaacagaaaa ccagatatta    115860
     tatattctcc cttatttgtt ggagataaat aaaagcaaat attcttccag ggcctgagtc    115920
     ttccttattc aacaagtcat tctaaattaa gtgttcagca agttgctgat actcatctaa    115980
     atattctatt tcatctgggc cacttacatc actcaaaaag caatgagagc tatatttcta    116040
     aggggggttc taggataata aatacctgaa tagtgagaat atgaaggata tggaaactgg    116100
     gccacttata tcactcaaaa aggaatgaga gctatattta taaggggggt tctaggataa    116160
     taaatacctg aatagtgaga atatgaagga tatggaaact gggccactta tatcactcaa    116220
     aaagcaagga gagctatatt tctaagtggg gttctaggat aataaatatc tgaatagtga    116280
     gaatatgaag gatatggatg gttttttttt aattcaatgg gcacataact gtgggagaca    116340
     ctatattcct atgaagaagg tattcagact tcagagataa gtaaagtttc ctacgttgtg    116400
     tttgtgactt ggaagcagtg gattgaagag tgtgataggt gcccagacca agcggaacag    116460
     aaatcaacat gtaaagatga tgatctatgg atatgatcta aaaccatgaa aatacttcaa    116520
     ataattctat ttaatgcagt ttgaaataaa acacaaactt attcaaaata caaattactt    116580
     ggtaattatt ttgggagcta tgagttcatc aagaaactca aattcctatt tctacttcaa    116640
     cccctgattc ctactgtcaa tgggagggaa gtctcagaac caatcacaca tcagacggca    116700
     aatctgtcaa ccaagagtct ttcccttgaa ggcctgggag gtcaggaccc tcaggaaagt    116760
     gctggggact ctgtcttggg agtgtccagc agatctcaga actctccatg ggtcctgctg    116820
     gacactcatg tagggtaacg agtggccacc ttttcagtgt taccagtgag ctctgagtgt    116880
     tcctaatggg accaggatgg gtctaggtgc ctgctcaatg tcagagacag caatggtccc    116940
     acaaaaaacc caggtaatct ttaggccaat aaaatgtggg ttcacagtga ggagtgcatc    117000
     ctggggttgg ggtttgttct gcagcgggaa gagcgctgtg cacagaaagc ttagaaatgg    117060
     ggcaagagat gcttttcctc aggcaggatt tagggcttgg tctctcagca tcccacactt    117120
     gtacagctga tgtggcatct gtgttttctt tctcatcgta gatcaggctt tgagctgtga    117180
     aataccctgc ctcatgcata tgcaaataac ctgaggtctt ctgagataaa tatagatata    117240
     ttggtgccct gagagcatca cataacaacc acattcctcc tctaaagaag cccctgggag    117300
     cacagctcat caccatggac tggacctgga ggttcctctt tgtggtggca gcagctacag    117360
     gtaaggggct tcctagtcct aaggctgagg aagggatcct ggtttagtta aagaggattt    117420
     tattcacccc tgtgtcctct ccacaggtgt ccagtcccag gtgcagctgg tgcagtctgg    117480
     ggctgaggtg aagaagcctg ggtcctcggt gaaggtctcc tgcaaggctt ctggaggcac    117540
     cttcagcagc tatgctatca gctgggtgcg acaggcccct ggacaagggc ttgagtggat    117600
     gggagggatc atccctatct ttggtacagc aaactacgca cagaagttcc agggcagagt    117660
     cacgattacc gcggacgaat ccacgagcac agcctacatg gagctgagca gcctgagatc    117720
     tgaggacacg gccgtgtatt actgtgcgag agacacagtg tgaaaaccca catcctgaga    117780
     gtgtcagaaa ccctgaggga gaaggcagct gtgccgggct gaggagatga cagggtttat    117840
     taggtttaag gctgtttaca aaatgggtta tatatttgag aaaaaaagaa cagtagaaac    117900
     aagtacatac tcctctaatt ttaagataat tattccattc aagagtcgta atataagcca    117960
     gattcacaga gtggaaaagg ccacactcta taaagttgat acaaacattc catgaaggtg    118020
     ctactgtgaa caagttttca aattggatga atacatgatt tggagcaagg ttatttgatc    118080
     atgtggtgag actaagaatg attcttaaaa agtgccaaaa gtttccttca aatgtttctg    118140
     tcactcctta tcataaagtt tattttacag cagttttagg attacaaaga aattgcacag    118200
     gaggcgtgag aattcccatg actccctgcc ctacacaggc acagcctcct ccactacgac    118260
     catcctgcac cgcagtcaca aatcagttac aatggaggaa tctccaagga cgcttggttc    118320
     tttctttttc tggtgatctc ctaatataac aagcctaagt atctcaagat tccacggttt    118380
     tttcagtgtt ttctagaact gatattagtc agagggaaag tgggtaaggc tattactatt    118440
     tgaactcttt cttccaaaat ccacaaaata tatattaatt tagagcttat cttacttctg    118500
     gtttacaatg ctccttccca gacagtaaga tttttttaag cttttagagg ccagttcatg    118560
     tctctaaaag accagaaagt tctggggaat cccataatga acatcctttc atgagaattg    118620
     gagaccctgg caatgagaga ctccatgtat aatgccctag agttggatta gatgctctgt    118680
     aagctcttgg gtggtgagtc tgagtaaggg ggtttgtgca gcaaacgcaa acacatgcat    118740
     gggacccagg taggaacaaa agcttccctc tacaaaggga gtgtgcacct gaagcagccc    118800
     tcacagaggt gggcactgct cacccttgat gagtgcacat tagccagagg catgatcatg    118860
     attggtcttg cagacaaaga gcaccactga ggtcataggt tatgaaaatg tttgtcatcc    118920
     tccagctgag caagtccatc tgcttgtttg tgggtgtcaa ctccatggag ggtgcacttt    118980
     gggagatgac aagatgcaca caaacctcct ctcactaatt atccactacc acacactcaa    119040
     aacaaccctg tgctccagaa agggatacgt gcctgccgaa acaaacagag cttaagggtt    119100
     ttattacctg gtaaatatac tgcccaaagc cacacgtttc aggaagatta gctcagaaat    119160
     gttcaccaat tgactgaagg gcagtggcgg gtgaggtgat gggacagcct cagggctgca    119220
     catgaggagg gctccctccc ccatgcaggc ttttctccag gagcttcacc aggaactcaa    119280
     gggggatcag ggagaattct gagaacaccc tactgtggag ctgcctagag aggaagaata    119340
     aatgatgaaa aataaaactc tgagtaacgt atgggcattt gttaatgaaa actgtttttc    119400
     tgaaagcttg tgaaggtctt gaaatgcccc tgagtagctg agggcaaata tttaaaccct    119460
     ccttccacag ggagttcaag caggctggat gtgtccttct atggatgatc ttccccaacc    119520
     ccttcctctt cccagatcat ccctggctct ctgtgtaaac agttctcatc agtgtaatgt    119580
     gattgatgaa gtgaggtctt caatttcctc atcttcttgg tggtcatgtt attttttttc    119640
     atctgagggt taaaaactca cctgcatgca gcacgtgaca gcctaaaatc tcttgtggac    119700
     aaaacagtaa caaaggcacc cacgagggtt gagcacccgt gttgctgaca gcgacaacca    119760
     ggggtcaacg tcctctccaa aatcctgtgt cagagcatca ctggaatgat ttcattagca    119820
     acttcccagg agaatcagct taaaaaatac ttgtcccatt ttccatgcag atataaccca    119880
     tcccttttcc tgaagaaaca gagctcaata ctaaatacac tgaatgttgt ttttgctggt    119940
     tttgtaagtt tgtgacttta tcactttcta atttctgagt taggtggacc actctacata    120000
     tttctctcat gggtgtgacc agccttctgg atgtcaaata taactgactt tgttcgtgta    120060
     aatgtcaaca caagctcttc atggtttcgg tgctcactga ctactctaaa cttacacatg    120120
     tgtttcttta ttagctttgt tttctggtgt ctctacttgg cttattcatc atgtccattt    120180
     tgtgttattt ctacaggtaa tgattgttat ttttaaaatt tactgttgct tactttaatt    120240
     aaataagaag gcaatagctt agaatagaaa acaaggtaaa gtaaaagtac atgcagtaat    120300
     atttaatata agtaagcagg cagctgaaag caagggaaat ctactctgct gtgtaaaagg    120360
     gtatggagac ctcatagtat gatgatcttc tgcagtttaa tccatactgc tacataagtg    120420
     acagcatcca ctctgtttat atcagagctt tccctaatat gtataatgaa gactgagccc    120480
     aggtacctcc aaccagactg accagcacct tttaccaagt gcctgaactg acagcaatag    120540
     agatcacgtt tgtctaaaaa aaaactcaca ctgtaaggat acccaccagt catatggaga    120600
     caatttgaaa aaattaaagc ccaagcctga taggtagtaa tttatactta gaagaattaa    120660
     cttttatctc cacatttttt taatcataga gaaactacta gagtcactgt ccaagttact    120720
     gaatatctta ttccctagaa tgagacattg cacaatgtgt tctgacacga gggaatgtag    120780
     ctgtagttaa gggacaggag atgtgatcac gggaacatga gtctgcgatc cagcagttct    120840
     tgctccttca catttgccca gaaacaaaga ccccagcaga acaatggaag cattgactta    120900
     ggtctccaca gtctgatgtg agataaacag caggtagttt atgcctctgt tttatagcag    120960
     gaaatttaat ctctaaagaa gggccatttt gaggataata ctattctcca ggatgcttac    121020
     atgcccccaa acagagagca ctacagtgcc ccctggccag gagctacaac acctgggtct    121080
     ggaaaccact ggacagaaat atgagtttct cttaccacat gtatgatgga aatttgaaaa    121140
     atgtttcctt ccttccccat aatcataggc tgtgatggac tggaggtccc agtacataaa    121200
     aaaactgtgt ctgcattgga tacagaatta gtctcataaa ataattgtgc tacggtggtg    121260
     cccacactgt tggaccagcg gaccgagaaa gtgttgtaag agctgcagtg tctactgatt    121320
     cctatcatat ttggggctac attcacatct atcaagggaa agagagataa gaaaatctct    121380
     ctaggccggg cgcggtggct cacgcctgta atcccagcac tttgtggggc caaggtgggc    121440
     ggatcacgag gtcaggagat ccggaccatc ctggccaaca tggtgaaacc ccgtctctcc    121500
     taaaaataca aaaaattagc tgggcatggt ggtgggcgcc tgtagtccca gctactcagg    121560
     agactgaggc aggagaatgg cgtgaacccg gcaggcggag cttgcagtga gccgagattg    121620
     tgccacttca actccagcct gggggacaga gctagactcc ataaaaaaag aaagaaagga    121680
     agaaagaaag aaagaaagaa agaaagaaag aaagaaagaa agaaagaaag aaagaaagga    121740
     gagagaaaga gagagagaga aagaaagaaa gaaagaagga agaaagaagg aaggaagaag    121800
     gaaggaagga aagaaagaaa gaagaaagaa agaaggaagg aaggaaggaa gaaggaagga    121860
     aggaaggaaa gaaagaaaga aagagaaaga aagaaagaag gaaggaagga agaaggaagg    121920
     aagtaaggaa agaaagaaag aaaaagaaag aaagaaagaa aaagaaaaga aagaaagaga    121980
     gagaaagaaa atctggccta gatagagtct acatatttat tttcatttct tcaaatgagg    122040
     taaaaaaatc gcatcaacta aaatggggac gatgatgaag tgatcaagga caacagccac    122100
     tcagtgtttc aggtgtgttt taacccaaag tcacacaacg tgtgagaacc tgaactccgg    122160
     agccaggggc atctctgagg agaatgtgga aggtgggcgg gaggaaacag tgattcatgt    122220
     ctagggaaat ttcagggctg cctgctctag gggaaaggaa agcctgagcc tctcccacag    122280
     tgtgctctgt taaaattgtt gggtgactta ttgtattctt cgacacaaat atttttgttt    122340
     agtcctctct taaatttttt tatctctatt aaaattctca ctttgttctt gcatgctgcc    122400
     actagcttac taaacatgtt tctaatattt acttcaaatt ttctgccaga acatcaatat    122460
     gttttatatt cattgatgcc agattctgga cctctatgtt gtctgtctgt ttggaccatg    122520
     ttcccgtgtt tcttcatttt ccttgacggt ctttcttgct atctgttcat cataacaaac    122580
     agctctttgc tcatcataac aaagagccac actcttcaaa gactagggtc atagagacca    122640
     ccctcaccaa tcaccccagt caataagtgt ggatctctca aaaattcata ataacttcat    122700
     aatattcaaa ctgctgcctc tgttcttagt ggcccccagg tttagagaac attgggttgt    122760
     gccaggactc ccaagtagga gaggtagaaa ctatttactc aagcagactc ttgaaaaggt    122820
     gtaccattag acacagtttt attattgagt tatgattcat caaaataatg ttagttcaag    122880
     tttctttcaa ccagtcatct aacattgaaa tgttataatt tcttacttgg atctctctgg    122940
     ggtttatctt tgggatattt aggtaaaatt acaaactagt ataaagctgc ctacttgaca    123000
     aaattatctg ctgcagaatt ttcatcttcc atgtatctgt ttttgcacct aaatctaagt    123060
     ttttattagt caagtatcca gaatatagaa atatgtctta aagttccttc actttttttt    123120
     aagtttatgg ggtttccaga agaggtgaga acgttcttta tatataaaat acctgattcc    123180
     acattgctgg atagtttctt ctttaattta tctcctcctc tcacattata ctatattaag    123240
     caaggagaaa tcaaaacaca ccatccacac tttgctagaa atccccaaag ctaaatttta    123300
     agtatcactt actaattcta attttaccca ttgtaactta attaacataa aatctataac    123360
     aaggttacac atactttccc gttctaatac catgttcgtg acagaaacaa agcaggtgtc    123420
     ttctctgcaa tgtggtcatc tgaggtctga gatgaggggt cacacaggta ttttataata    123480
     tttaagggtt tggacaacag tagtgataac aattctgaac agcactggct cctaattgaa    123540
     aggttatagg taaatttaat gctgcaattt tatattaaca ccttgtccga atgaagctca    123600
     gaggaagatg ccacatccca ggtcacacag tgaggaggaa tggagctgcg ctctgctctc    123660
     cacaccacct acaaatccca ggccccagcc caggttccac tggcgcacgg cacctagagc    123720
     acagttccca ggggatgatc tcagggcacc tgcttcctcg ggcagggcgc tgccttctct    123780
     caattgtgca cttgctcctc ctgcaggttc tgtagtgagc acttgtattg tcgtgattta    123840
     tacatgttct ctcccctaat atggaaaaaa atatttatta aattttcaac tcattttctc    123900
     tgatgcacac tgcattagca gaaaggaata aatcacattt cctatcctcc cataaagcca    123960
     aagattcccg aagacagact gatgtgatgt actcataggt ggatctctgc ccctcagggg    124020
     aggccttggt cttcaagttt cagtaattct aggaagcgaa ggacacctac atctcctgct    124080
     ccctgctctg tagctcacct gagaacagct ttctcattgg aatgtcttgt gtttaaggaa    124140
     taagagtcca tgtttcaggt tcgggagccc aggtgcacct actggatgca gcccaggatt    124200
     ggagacactt tccagaagac aacatcacct gagacatgac cagtcccact gtttcacttt    124260
     cacaatttca acttcctcag aagaaaatta aaattgctga gacttgttca taagtgttct    124320
     gccatgtcct tactctgttt tcttgcctgt tcatttatgt cataccaggt gcctgttata    124380
     cgtaataaga tcagaattct gcctccagta acacatcaat ggagaccttt gattgtactt    124440
     ttggtttatg cactgacaca tagattatga tgttcatcac attcattttt atgtcaaagg    124500
     aaatctgcat aatctgaatg ccaatacttt ttggaatctg ctaagtaact gaaattgaaa    124560
     aaaaaatacc cactcaagaa cctggataga cagccatgtc cagaatggca gttgacactt    124620
     gtttaactgg aagagaatct acagaagcca caagttgttg agggcactta catgataagc    124680
     actatagttg tcttaaagac agatgtggac tcagtaaatg tgactgttcc agagggtctt    124740
     atacttctat gttttatgga ctttctcacc agaaacctcc agattctaaa aaatactatc    124800
     caaatacatt tcctatttgt cagatcggag aagattaatt actgaaaaat attacgggag    124860
     actttttcaa aagcttctac atggaaggac ttttcgagaa ccttgtccta tgtaaaggaa    124920
     gacaattctc ccattccaga tttctctccc attcttccat tattatacac atgagcaaag    124980
     ttagccaata ggggtaagat gtaagtaaat agtccagggg cactgaaacc acaaaaggga    125040
     gtaatggcca aagtagcttt tcccctggag attcctggtc aaagtcacag cccagaagag    125100
     gaagccgatc gcgtctctag gtttccattg tcaaaacagg cagtgcttgc ctgcactgca    125160
     caatccattc taaccagtgt gatagctctg gattaaagat ggaagtgtgg caatgcacag    125220
     actctatgtg agaagaacac tggaaaacta aaggacaaag gcagagagta ggacaaggac    125280
     gacaaagcaa tctgaagcct ctgacatcac catttttaag atcaaggtct tggaaactcc    125340
     ctcattgacc tttagatctc taggagaaaa aagtcagata cctgggccta gtgtcagtgt    125400
     aggaggaatt ttctataggc taggcattag gaagaaggga aaattcttcc ttattaggaa    125460
     gttattgtta tagtgttata gtgttattgg aatagtggat atggagcggg ctttcatctc    125520
     gatcaatctg cacctgctgg tattttccaa tgctacattc acctgcaaga gcccaatgaa    125580
     gaaagaaggc actcccaaat cttttgcaag tttttgtatt cactgtgggt ccactgctta    125640
     agtgcatctg gaacttcaga gaaggggctc tgtcctgtgt catagaaccc ttgctttgag    125700
     tctcatggca gagttcaatc tgtttagtaa agttgatcaa ttatttcaag agatggtgtc    125760
     accagcatat ggtgtcactg agggagtatt ctacactagc acacagccat ttcacgctgg    125820
     gctagagaag ctggggggaa atgctttgtg agccccaaca agaacctcct tgcaaggcaa    125880
     gggctgggcg ggagggggca ctcaggagcc attcagcacg ggttccagcc ctgcagctgg    125940
     tgcacaggag gctgctgaga aggtttcctc tcaggggctg ggtcttcctt tgggagaaaa    126000
     aagctaaaat tcaataagtt gctggtgtgc ccttaaatat tctatcacat ctgagctgtt    126060
     cccacaattc aaggcgatga gaactatcct tataaatatt ctatcacatc tgagctgctc    126120
     ccacaattca agaccatgag aactatcttt ataaatattc tatcacatct gagctgctcc    126180
     cacaattcaa ggcgatgaga actatctttt taaatattct atcacatctg agctgctccc    126240
     aaaattcaag gcaatgggaa ctatcttttt aaatattcta tcacatctga gctgttccca    126300
     caattcaaga ccatgagaac tatcttttta aatattctat cacatctgag ctgctcccac    126360
     aattcaaggc gatgagaact atctttttaa atattctatc acatctgagc tgttcccaca    126420
     attcaagacc atgagaacta tctttttaaa tattctatca catctgagct gctcccacaa    126480
     ttcaagatca tgagaactat ctttttaaat attctatcac atctgagctg ctcccacaat    126540
     tcaaggcgat gagaactatc tttttaaata ttctatcaca tctgagctgc tcccacaatt    126600
     caagaccatg agaactatct ttataaatat tctatcacat ctgagctgct cccacaattc    126660
     aaggcgatga gaactatctt tttaaatatt ctatcacatc tgagctgctc ccacaattca    126720
     aggcgatgag gactatcttt ttaaatattc tatcacatct gagctgctcc cacaattcaa    126780
     ggaatgagaa ctatcttttt aaatattcta tcacatctga gctgctccca caattcaaga    126840
     caaggagaac tatcttttta aatattctat cacatctgag ctgctcccac aattcaagac    126900
     aaggagaact atctttttaa atattctatc acatctgagc tgctcccaca attcaaggca    126960
     atgagaacta tctttttaaa tattctatca catctgagct gctcccacaa ttcaagacaa    127020
     ggagaactat ctttttaaat attctatcac atctgagctg ctcccacaat tcaagaccag    127080
     gagaactatc tttttaaata ttctatcaca tctgagctgc tcccacaatt caagaccagg    127140
     agaactatct ttttaaatat tctatcacat ctgagctgct cccacaattc aaggcaatga    127200
     gaactatctc tttaaatatt ctatcacatc tgagctgctc ccacaattca agacaaggag    127260
     aactatcttt ttaaatattc tatcacatct gagctgctcc cacaattcaa ggcgatgaga    127320
     actatcttta taaatattct atcacatctg agctgctccc acaattcaag gcgatgagaa    127380
     ctatcttttt aaatattcta tcacatctga gctgctccca caattcaagg cgatgagaac    127440
     tatcttttta aatattctat cacatctgag ctgctcccac aattcaaggc gatgagaact    127500
     atctttttaa atattctatc acatctgagc tgctcccaca attcaagaca aggagaacta    127560
     tctttttaaa tattctatca catctgagct gctcccacaa ttcaagacca ggagaactat    127620
     ctttttaaat attctgtcac atctgagctg ctcccacaat tcaaggcgat gagaactatc    127680
     tttttaaata ttctatcaca tctgagctgc tcccacaatt caagacaagg agaactatct    127740
     ttttaaagtc ggcttctaag attataaata ccttaatagt gagaatatga agaataggga    127800
     tggtcttact aattcaatgc agagagaatt atgggagtca ctatatttct atgaataata    127860
     atttcagatt tcaggctggg gctggtggct catgcctgta atgccagcac tttgcgaggc    127920
     tgaggtgagg gggtcacgag gtcaggagct ggagaccatc ctggctaaca ccatgaaatc    127980
     ctgtctctat tcaaaataca aaaaattagc caggtggggt ggcatgaacc tgtagtccca    128040
     gctactcggg aggctgaggc aggagattta atcctacaag acctacagcc tcagacaatg    128100
     ctgctggcag tgatcgtggg aagcagagtt tgcagtgagc tgagattgcg ccactgcact    128160
     ccagcttggg caacaaagcg agattccatc tccaaaaaaa aaattcagat ttcagtgtta    128220
     agtaaagttg cctacattgt gtgagtgaca gggcagtggt ggatccgaga gtgtggatct    128280
     gagagtgagt cagaaatcag catgtaaaga tgaggatcta tgcacatgaa ctgaaagtat    128340
     gtaaacagtt catgaaattc taataaatcc agtaggaaat aaaacccaaa cttatccaaa    128400
     cacaaattcc cttgaaatta ttatgggagc atgagttcat aaagaactcc taactcctgt    128460
     ttcaacttct gaatcctagt gtccatgaca taagaaaatc atctccaatt atgcatcaca    128520
     gggcaaatct gtaaactaag agtttttctg ttgacgatcc tgaggaatca ggacaccaag    128580
     aaggttctgg agaaactgtc tcaggagcgc cccagggatc tcagaagaac gtgctggcca    128640
     ctcacgtggg acatcagcgt cacttgctca gagtcatcag tgagctgtgc tggtgtctga    128700
     cgggtccagc atagggccaa ggcacctgct ctgtgtcatg gaccgagatg gtccccagaa    128760
     tgatccaagt ggtctctgtg ctaatctaat gtagtttcac agtgaggggc cgttctgagg    128820
     gggcttcttc ttcagtgaaa ggacctctgt ccacaaatat tcctaaatgg acaggggcat    128880
     gcatttcctc aagcaggatt agggcttgga ccatcagcat ctcactcttg caaggctgat    128940
     gtgtcatttg tcttcccttt cttatcatgg atcaggcttt gagctatgaa atgccctgtc    129000
     tcatgaatat gtaaatacct gagatccact gaggtaaata tggtctgtgc cctgagagcg    129060
     tcacccaaca atcacatccc gcctctagag aatcctccga gagcatggct cctcaccatg    129120
     gacaggacct agagctaatc ttcctggtgg cagcagctac aggtaaggtg ctcccaagtc    129180
     ccagtgatga gaaggggatt gagtacagtc aaggaggctt tcatccactc ctgtgtccca    129240
     ccctccaatg ggtgtctgct tccaggtgca gctggggcag tctgaggctg aggtgaagaa    129300
     gcctggggcc tcagtgaagg tctcctgcaa ggcttccgga tacaccttca cctactgctc    129360
     cttgcactgg ttgcaacagg cccctggaca agggcttgaa aggatgagat ggatcacact    129420
     ttacaatggt aacatcaact atgcaaagaa gttccagagc agagtcacca ttaccaggga    129480
     catgtccctg aggacagcct acatagagct gagcagcctg agatctgagg actcggctgt    129540
     gtattactgg gcaagataca cggtgcgaga acccacatcc tgagagagtc aggaacccca    129600
     gggaggaggc agctgtgctg gcatggagga gatgacaaag attattagat tgaagacttt    129660
     cttagaaaat agcattaagt catttacaaa aaggaacaat ataaatgtgt atttgagaaa    129720
     ttgtaattat ttgagagatt tcttatgcaa catttattct gtaagcaaat tctagggatt    129780
     ggagaattaa tcaaattaat aaagctgaca tagaaattcc tctgaaggta tctttgtaaa    129840
     catcaatttc tgaatcagtg ttgtaaatgt tttggaacag agacgcaaga tcacatttta    129900
     actctacttt tgtctctata aaaaatgcca aaaagagtct cattttgacc atgtacctct    129960
     ttgaattccc accatcaatg aatgattgtt cttgggtttc cacattatat taccatttat    130020
     cattatgaga attgtgtgtt ttaaccatta taataggtga gtaatggtat ctaatttttg    130080
     cttaaatgca catgacccta ataaaattca tatttaacaa ttttcatata atttttggtt    130140
     agatgcctct cctgatattt ggttcatttt caatagcact gttttctttt gattagttgt    130200
     aagtttactt gcatattgat tataaaagtt aattaacaaa ttaaaagaat tcatttaaca    130260
     aatatgtgac ttggaagttg caggggtttg tcctgcacac cctgacacaa tgacgaatga    130320
     ataaagtgca ctgacacaaa gatattctgc tttgccagct cgactgagca tctgggccac    130380
     ttagtcacag ccaccgcctc gatcagtcag ggagacttac atttattcag taaagattaa    130440
     ttgacaaagg ccgtgagtaa acaccactag aaggtaattg acattgtgga cctcttgagt    130500
     ggaaagcaat taagcacccg cggtagatca aaggttagtc ttaggaacac atgagtaaac    130560
     aagctagtta gataagctcc tcacattcct ttgtttctac tctactttat gtaactaaag    130620
     gtaaggggac taggctgcct tcagccagat ttattaccaa agttatgcaa actcacaggc    130680
     cttccaagag ggtttgtggc tattagaact aaaatgtttc ccaccagcct gactggacca    130740
     ccacatctcc ccattttttg ttttctacat caggtctttt ggattggagc gtgcagatat    130800
     gcgcagcaac atgtctgtca ggtgtggcag tcattgctct tattctggct ttgcatccta    130860
     gaattagcaa ataacataaa acaatcatga gtagaattag caacattctt ttccagtcag    130920
     agtgaccccc aggagcgggg tctaaccagg agagatgatc tcacacaccc ttccatatga    130980
     ctgtttgttg ggggtgtaga tctattgcgt gaagagattc taaaatttta gtttgaactt    131040
     actttacgtc tgctgttaaa ttgtcatgaa aggttcccca gaggtattgt tttacctcat    131100
     cctaactatg tattgataga ttccgtggta gagaagtgat acagatatgt tcatgttccc    131160
     agtcacagtt taaatgctgt cagaatgcca ctgcatcttg tcgctccctg acatattcta    131220
     aagcagcctc aagggctagc agacgtgtga taattttttg atctatacct tgctgtaaga    131280
     gaagttcatt agacacattt ttggccagat tatctaaaaa ggcagcagtt tacactaagt    131340
     caataataca tgcaacagca acactagctg ttgccaggat gactatggct gagactataa    131400
     aggccataag tgtaactatg aatcttttgt gtctgacctg ggacagggca cgttctaagg    131460
     ttgcaagggc ataggaacct tgccaattgc atgccagatt gactggtaag aatgcctcag    131520
     attttctctt taataccatg gcgctagtaa tttttaagtt agatatactg tgattagtga    131580
     tacatgaggc aaaccaagcc tgtccctgca tccaggtcac aaacgtggag ttttgaggtg    131640
     taatggaaat gttagttccc ataaggaaat cgtatggatg ggtagtgcaa attaggcact    131700
     gatcagtgtg attatgaata aagcttatag catagttgca acgggaaata tggtatgtcc    131760
     catgccaggt gttaagggag gtgctaagat gtcccagaca ccataaagta ccttggggcg    131820
     gcatggactt tacttggggc cttggatgtg ccatcccccc attggcacaa attatagggg    131880
     aaaagacatg gctatgaaac tgattgatgc tctgatggat gaaggtatta gtaaggctgc    131940
     cctgcaatca gccgttgggt ctccagtcta agatgttata attacctcac tggaggctat    132000
     gggctcgtct cccatgacag acctcccagc taaagtcgaa ccgattactg tcccagcttt    132060
     gttccttagc acaggaagga atgtttggga aagaagcagc ggcatggatt gcattgctca    132120
     gtttgaggct atctgcagct aagaatgtta aggcatttcc tttgctatga tgtaaccata    132180
     cttgtttttg ggcaggtaca cagtaagggt tagagtcttt ataacttaca cacactggga    132240
     ggataatagt ggattgacat atagtgttat ctgccacctt agtccaatgt gtgccattat    132300
     tgagggaccc cacgtggggt aaatctatcc ctcctagcca agcagtcacg ttactatagg    132360
     ctgggaaggg agtgtcttcc catttggcag ggtgaaagaa aggtgtgtct aagatagagg    132420
     cccaatagag tgtagcaggt acaggttgca gacaaagtga gagcataaaa aggattaata    132480
     ccctacatga gttgcaatgt acaacagaaa gcatagcacc aaacacatta tctggagtaa    132540
     atggtgtctg catttggagc aggattcgtt cagcctcctg agttgtcttt ttcagtatcc    132600
     cccaggtaat gtctggggat tgtgttgttc acagaagctg catcgtccgg ggctgtaggt    132660
     cttgtagggt taactccttc atttctggta ctgggttggg tcctagcaat gtcctggtat    132720
     ggtttgatgc gtcgtgctgg aacccaaaga ggacccgagg gggtgtggat acaagcatac    132780
     cctcttcccc acattaataa ttcacttgga ccacaccatt cattactgtt tacatctttc    132840
     cataaaactg caggtttcat gtattgggaa attttagcaa agtgcttttc tacagcggat    132900
     tcaaatttgt cttctaaatt aaaaaaaaat aaggcttgtg ccaaaagtgt tgcagggtct    132960
     gtacccatac tcccacgttt ttgttttttg tgcatatttt aagggtggag ttggcacgtt    133020
     ccactattgc ttgtccttgg gggttatatg ggatacctgt ggaatgtcgt atattccaca    133080
     tgtgaaaaaa ttgttgaaat tgtgagctgg cattacctgg acaattatcg gttctaattt    133140
     ttgtgggttg ttccataaat gcaaaagata aaagaagatg tttaatgaca tatcgagtag    133200
     actctccagc cagagtatgt gcactaatta aatgagtgtt gatatcaaag gatacatgta    133260
     cctatctaag ttttccaaat tcagggatgt gtgtaacatc tgtttgacac aacagattag    133320
     gttctagggt taatacctgt tgaaggaaga gacgtacctg tgagctggcg atctgggcat    133380
     tgtaagataa tttgtttagc cagtctctgt gtaagttgga attgtttaga taagtttttc    133440
     caattttggt ggaaaaattg gtgcaattgg gtggcttggt caagcagtga tatcataact    133500
     tgaaggtcgg cttgattatt gtcataacct aatggaccac gcagtgagct gtgggctgga    133560
     atgtgtgtaa taaaaatagg atgtgtactt tgatttagca attgctgaaa tcagagaaaa    133620
     agagtccaca gggctggctc cagagttgac ttaattaggg ctgtaaaagt tctctagacc    133680
     caatattaag gactcaactt tagctttctg agtgctagta aacccagatt gagtgaatga    133740
     attgtgtggt gtccaccaga ctgctgcttt tccatgttta ccagatctat cagtaaacag    133800
     tgctaaagca ttaaatatgg gggagtaaac tatttttgta ggtaacacgt taactaggcc    133860
     ttttgggaat gatgaaaatg ggtaaattct aagggctgtt agtagaatat tacccataaa    133920
     atgaatactc ttgtagccag gaaacttttt tctactaggg agcaaagctt gcttttaatt    133980
     gctccctaac taacttaggg gctttttgta atgtctctcc ctttagaggt tactgtttta    134040
     cttaaactgg atttggagtc atgttaggaa taagagagag ataacagtgg tcattattag    134100
     aaagaggttt ctcaaatcct tgaattttac ttaactatta gcatagaatt ataatctggg    134160
     atgtagcttg tgtacaagga acataacaaa ttttgctttg ggccgcctgc ggagccagag    134220
     agcggagcag gcgggtccca gggcggctgc cgctttgtgc ttgcttagaa gccacttttc    134280
     gccaccgcgt ttcctctgtg ccagctccct ccccacactg ctcagcagct gctgccggca    134340
     gaggggagct tcctgcactt gctcctgccc ctaatgcctt ttcttaaccc tttgtgtacc    134400
     tgattgcctt gctgatcttg cattactggg cagtctaaga gctccccttt taatgctgct    134460
     tgtcgaagac agggacatat agctgtagtg tatttcctct attttttcta tctattgaaa    134520
     gagggggctc aggcaaaacc tccatttcct ctttgttatt ttgggaagag caggtggtaa    134580
     ggcaagtgac gtttcttcct ccttcccctt tttgggttct tctttgtata atgggactaa    134640
     agccatctct actaaagccc atagtgttaa agatgatgct gggacccgtt gcgcttgtgt    134700
     gtaatgttgt tcaagatttc tccctacttg ttcccagagc tttacttcta gctttccttc    134760
     ttctgggacc acagattatg tgagacaaca gttttcatta ggtgccttaa ttgagcctgc    134820
     gaaactgatg ctcccctagc cttaagcaac tgttacaata cttttatatg ctgtttgtgt    134880
     tgagctgata actgctgtcc catgatgaaa tctcagcctg aacaatctcc cctgaacttg    134940
     gagatcccaa gtgggcacca atgacttact gttttactga cttgaccacg cagtcttcct    135000
     caaccttcgt tttcatgggg tctgtcacgc tccctttgca gcgatcctca catggggcac    135060
     cggctgtggg ggtctgtcct gcaggtcctg acacaacaac gggtgaataa agtacactga    135120
     cacacagata ttctgctttg ccagcttgac tgaccatcca ggtagatcaa aggtgagtct    135180
     taggaccaca tgagtgaaca agctagttag ataaactccc cacattcttt tgtttctact    135240
     caaatttact aaactaaagg taaggggact aggctgcctt cagccagatt tattactgaa    135300
     gttatgcaat ctctcaagcc ttccacgagg atttgtggct attataacta aaatttttcc    135360
     cacgagcctg actgaacccc acaggaagta ctttctccaa gtctgtggtt gccttttact    135420
     cccttatcag taggtatcgc agaaaaatgt gtgtgtttgt gtgtgtgtgt ttgtacaaat    135480
     ttagattgaa aacatatata attttattca ttcatagatc atgtctttgg cattatatct    135540
     gaagtttcat tataaaataa actaatagtc attatttttt ccatatctct aatctcaggc    135600
     cacaatcaac tcatgagtgt ttaaacttca cctgcttgat tggaggacca tcaatctaat    135660
     gtatttggaa tacttctgta aggagatgtg ttcttcttcc tattattttt taattgatca    135720
     tctattaata tcagtattgg ttgatggatg tccattttat actttgaaaa agatccatgc    135780
     tacatcattc atttaattgt tcaaagcacc acagctttat taggtgctgg gagctcattt    135840
     tgtttgaatc ctgcatcctt acagcacacc tcacacctca tttttttgtt tttgaaaact    135900
     tgcgtatttc ctggtattac cataaattct aagcttgttt tctttattac ctttttttta    135960
     catagaatca accactttta taaagattgc atgtttctga tgttaaagaa tagtattaaa    136020
     ataaaacatt gtgatactgg ctctgtgtgt tgttaatgtg gtatgagtac ttctagaacc    136080
     tctcaaacaa cggtcccagt aaacgtgcat gtttatatga acccaagttt atggactcat    136140
     tgaaactatt tatgtatcta atcttctgta accctattac attaaaaatg agaacacact    136200
     ggtctctcca tccaactatg ttagcacaga gacctttcta gtcttctttc cttgagtttc    136260
     cataaccact cactacaaag tgagaaaccc cattccacca tatgcaattt tattacttag    136320
     ctgcacagtt tcaggacaca tgcatagcag tatctaaagt gtaaagctgt accctttttg    136380
     gaaccatgtt tatctattag aatagagtgc ttatgtgcag attttttaca cattaaactt    136440
     atggaatttc ctcgttttct gagttgctta ggtcagcaac ttcattttcc acattcttca    136500
     atgaagtcat ttcaattaca ttgtataatt tcatttattt gaaattccat aaatgcttaa    136560
     actatagtca agtaaacaga tagaggatgt tccaggaatt tagagagtgg gtataaaata    136620
     agcaaaaaaa agtgatgttt aagaaaaata aaactatttt tagtgatatg caatggctga    136680
     gatatgacat aactaatttg tctaagctaa taattttgtg atggaaaata taaacttaaa    136740
     tatattcaat ttaaaaaaat tcagcagttc attaacccca ggattaaatg cagactgtat    136800
     aaaattatcc aataacttat ttggtgagag tggggatgtt atgagataca tgcaacaaag    136860
     aatgaagtaa ttttccttat ttgcatatac aatgtttcca ttcactgaag accttttatt    136920
     ttaaaaaaat caattttcta ccttaccctt gtttttaatt cccgacaagc aaataaccca    136980
     aaggattctt ttctttcatt ggttgagaaa gattttcccc taacttcagc ttagttcagg    137040
     catacactga cctgaatggg catttgccct cagatgggta cacacctgtc aatatgtggc    137100
     ctcttctgtc agacagacac agcttcactc atgtggattc ttccctcaaa cacaaatgtc    137160
     cccacatgga ctatttcctc agactaccac atatgtcctt acatttacac tttcctcaga    137220
     aaacagacat ttcctcatgt ggactcttgt ctcagataag caaacatgtc tcaatgtgaa    137280
     tgaagtcttc actcagataa gtacacatat ttcaacattg actgtttcct gacacaagca    137340
     catatatcca atgttaaact gtcttgcgac aaaatgatct caagataatg ataattataa    137400
     actccaaccc tgaaaacctg tggatctgca ttttgtccat tgtaacataa cttcttctca    137460
     ttgtcagaaa cagtcgtttg cagctataaa tgcactgatt acagtcagat ttccattttc    137520
     tctggaaatg tatttcttat gttcttactg gactaatttg ttgacaatgt ttgctcacat    137580
     gaagatacct gaacagtgtc cacattagag aataagaaag agcaatgggc agattaaccc    137640
     tgtgcatcca gatgcaggaa tcatttgact ctgccttccc tgaaatggag acacagagga    137700
     tggatgagca acgctgagtg gtgcacctac gaccacaaag agaaagacgt ggaaatatgt    137760
     cccatcccct cctcatgaaa ggcagctcat cccctgttcc ttcaggccct ggtgaggagc    137820
     caccccatat ctgtgtcttt cttcagtgtt cacactatgg agtctgcact gatctgggtt    137880
     tcccttctca tcaccctcaa tattagtgtc ccttgtaaat caggtccagc tgtggctgct    137940
     catagggttg ttctcagtct atttcctctg tgttcataga agtcctgtat gaagttcagt    138000
     ggtggagtca gaggggtaaa cgttgtacag cccagtgatt cactgagact ttcatgcaaa    138060
     gtggctggat tcacctttgc tggcaacagc ctgaggttgg tctagcaggc ttcacaacag    138120
     ggattgtggt ggctggcaac agtgagtcag caagtgggag tgctcagggt tactcttcat    138180
     gagtacaaat aaattaactg gtccagcgac accctttcac gtgcactcta ccttacaatg    138240
     actaacctga atgccaagga caaggttgtg taatactgtg aggttcacag gagagggatt    138300
     atctgcacga gcccagacac aaaaatctgc agagacagga gggaactgca tgatagatgc    138360
     tgctcagaag caccagcggg cactcaacac aagggggccc tcaggataca gctgggggcg    138420
     ctcaggacac cagggggtca ctcaggatac agcgggggac gctcagaacc accagggggc    138480
     tcttagggca ccagagggca ttcaggacca ccagggagcc ctcagggcca ccaggaggac    138540
     ctcaggacat gtcagcgtgc tggagccacc agggggcgct gaggcaacca agggacgctc    138600
     tgaaccgcga ggggaaggaa gctcaggacc acgaggggac gctcaggaca ccagggggcg    138660
     ctcagaacac cagtgggcac tcagaacagg aagaatctct tagaaagcag ctccacatca    138720
     ggatcctggg aggctgtgga gaaatgggaa cactcataaa ctgttgagca tttaaattag    138780
     tacaaccgct atagagaaca gcttagagtt tcctcaaaac ctgacgatag ttatcattat    138840
     aatctggcaa tcctgctgct ggtatatacc cctaaaaagg aaatgttttt gcactaccat    138900
     gtctgtaata gcgctgttaa agcactgttc acaacagtca aaatttgaaa gcaacctatg    138960
     tgccgattaa cacatgaatg gataaataaa atatggtaca tataggatat gggttaatat    139020
     tatgccataa aaaaatgaga ttttatcatt tgcaacaaca tggatgaaac tggagttcat    139080
     tatattaatt gaaatgagcc cagaagagaa agacgaaatt cacatgttcc catttgtttg    139140
     tgaaagctaa aaattaagac aattgaactc gtggagatag agagggaaaa gatggtcccc    139200
     agaagctggg aaggtctatg ctagatgggg attattgggg atggtcaatg ggtagaaaag    139260
     gtgattagaa ataataaata acacataata tttgataaca caacagaata agtatagtcc    139320
     tcaataactt aattatagat tttaaaataa aagtttatga atggaatgtt ggtaactcta    139380
     ttaatgcttg agaggatgta taaactattc ttcataatgt gattattatg cagttcatgc    139440
     tgattcagag ttctgtaccc cataaaaata tacatgtact atttacccac aaagattcaa    139500
     aatttaagtg tttatgtgtc taatattctg taactatatt acattaaacc taagaacaca    139560
     ctggctttta cacactactg tcatccacat agacattcat agccttcttt ctctgcttgt    139620
     ccataatccc acactccaaa atgagaaaac ctctcctacc atatgacatt aatttactta    139680
     gtgcacaatt tcaaaataca caaatcgtct ttaggattgt taaactgacc cttgttgaaa    139740
     atatgtttat caactagaat acagtgctta tgtggagctt atttatattt tacaatcgca    139800
     acatcaaatt ctttccaagt ttcttgtgtc caatatttat gccccacctt cttcagggta    139860
     attagttcaa accttctgat tgttgttaga tatttttata cagctgtctt tttttgtgtt    139920
     tcatgacctg ctaaatactt tcgaagttgt atgcatttag gttaacactt tgtctcaaaa    139980
     aatatgagtt ttcagaaatt ctttatgcca tgtgtccatc attgaatatc ataaaaatag    140040
     tttcattggc cgggcatggt ggctcacacc tgtaatccca gcactttggg aggccaaggt    140100
     gggcgggtca cgaggtcagg agatcgagac catcctggct aacacaatga aaccccatct    140160
     ctactaaaaa tacaaaaatt agccaggcgt ggtggcaggc acctgtagtc ccaaatactc    140220
     aggaggctga ggcaggagaa tggcacgaac ccaggaggtg gaggttgctg tgagcacaga    140280
     tcgcaccacc acactccagc ctgggtggca gagtgagact ctgtctcaaa aaaaaaagat    140340
     agcttcattg tgattaaaaa atgtgtttca cctaatccac acaccttctc cccaccttcc    140400
     tgggaaacct caaatgttta ctggatctat atttttatct ttggcaaaac agcctggctg    140460
     ttttcttagc ctcttctgca aagcatcaag attcaccttc actgactaca gcatgaattg    140520
     agtccagatg gctgggggac agaggctgga gagggtggta acagtgattg attcaagtgg    140580
     aagttctcag tgatattctg catcagcaca ataaagattc acaattccca gggacaccaa    140640
     ttaccagcac agtctccctt aaaataatct acttggaagc tgagggggct ctcacagggg    140700
     taggcagtgt attactgtga gagatacagc gagggacatt tctgtgagtc cagacggaaa    140760
     cctccctgca gggagacaag agaggacttt gtgataaatg gtgcttagga caccaggggg    140820
     cactcaggac agcagagggt gctcagcaga gcagggtgcg ctcaggacat ggtgtgtgtt    140880
     gtagggggca ttgctcagga tcaccagggg gctctcagaa ccaccaaggg tcactcagga    140940
     aaccaggggg cgctctggac accaggggga gctcagaacc accaggtggt actcaggaca    141000
     ccagggggcg ctcatgacca gcagggcatg ctcagggcaa cagggggctt gcagaacccc    141060
     agttccaacg ccagtgttgg aagcactttt cattggcaag acactaggaa ggagagcaaa    141120
     ttggaggctt gttactgtgc aaatatgcca ccgtgagtgt gtgtgggggg ggtaggtggg    141180
     gggtggacct tgaaacataa ggtgtgtgcg taaagtgata atctgatgta aacttcgacc    141240
     acagaatcct aaagaaaaaa aaaaaagagg ctgccccaaa gtccccatca gttcctggac    141300
     tcgccatgtg tctggaccat atcagtgcat ctggagctcc agggaagggg ctccctggtg    141360
     gatttagtga ttccttgctt cctgtgccaa ggtctccctg tgggatatgt taggtttcct    141420
     aaggtctatt ttctagtgta agaagtgatg tgagaagtag ttgctgtcac tgaaggagca    141480
     ttcttagcca gggcacagcc aggtcatact gggcttgaga tgctgggaga aaaatgctct    141540
     gtgagcccag aaaagaactt ccctgcaggg caggagctga gctgcagggg gcgctcagga    141600
     tgcacccagc acaggatcca gctctggagc aggtgcacag gaggctgtgg aggggttttc    141660
     tctcagggat tgaatattcc ttatttcaaa gccataatga cataaaattt aaataagaat    141720
     ttagcaagta ctgatgtgtc tttagtattc tcttgcatac acagaggcta ccaaacccaa    141780
     taacttaacg caaaacacat ttaaaaggag aaatgtctag gcctttcaaa tgtatttata    141840
     gttaggaatt gaggggtggt tttattaatt caatgggtgt tactctccgg agacacactc    141900
     atcccagaag ttagatgtgc agagggcaag gcccaggaaa agttcagatt gtcagtgagc    141960
     cataggaaca agaaatcaca gtgaggacaa tgccctgtga gattctgggt ttctgtaaga    142020
     ggagttctgt cttcatggac ttctaagtgt gtcagaggac aaatatcatt aaacaaagtt    142080
     cagggcaggg agctcagtgt cccactgtgg catggtccgt gtgtcaccta tcttcttccc    142140
     cagggtgggg tggcctaagc tatgaaatac ctgcctcatg aatatgcaaa tgcagtggtg    142200
     tctaccgagg taaatacaga tctgtccttg cccagagagc atcacacaac agccacatcc    142260
     ctcccctaca gaagccccca gagagcagca cctcaccatg gactgcacct ggaggatcct    142320
     cctcttggtg gcagcagcta caggtaagag aatccttagt tccagggctg atgaggggac    142380
     tgggtccagt taagtggtgt ctcatccact cctttctctt ctccacaggc acccacgccg    142440
     aggtccagct ggtacagtct ggggctgagg tgaagaagcc tggggctaca gtgaaaatct    142500
     cctgcaaggt ttctggatac accttcaccg actactacat gcactgggtg caacaggccc    142560
     ctggaaaagg gcttgagtgg atgggacttg ttgatcctga agatggtgaa acaatatacg    142620
     cagagaagtt ccagggcaga gtcaccataa ccgcggacac gtctacagac acagcctaca    142680
     tggagctgag cagcctgaga tctgaggaca cggccgtgta ttactgtgca acagacacag    142740
     tgtgaaaacc cacatgctga gagtgtcaga aaccctgagg aaggaggcag ctgtgctggg    142800
     gctgaaaaga tgacagcatt tatgaggttt aaagtgttta gaagatgggt taagtcacag    142860
     agtaaaagta gaaatagaaa gatgtataca ttccaattat ataggaaata atcttttcaa    142920
     ctttcaccct gtaagtaaca ttcacagagt gggaaaggca gcaatcaatg gagtggatgc    142980
     aaacattcct ttgtgaggac ataaatttta aaatcgaatg gataaatcat ttggagcagg    143040
     attgcttgat cacatggtaa gactaaatat aatttctaaa aagtgaccaa catttctaac    143100
     aaaatgtctt tgccactcct tttacattaa ttttatttta aaacattttt aggattgcag    143160
     caaatttgag tagaataaac agcattccca tgtactcctg cccaagacat gcacagactt    143220
     ctccatgatc aacaccctgc gctgaagtaa taaataactt gcaacagatg aacccacatg    143280
     gacaccttga ttgtttcctt tcctggtggc ccctggtata acaagcctaa attaccttga    143340
     agcaccccag gttcttctag tgggttacag gaaatgatgc caggtagagg gaaagtgagt    143400
     ggggatgatc ctctttgcac tgtttcctca agaatccata aaatgtacat tgatttagag    143460
     ctcaactaac tcctggtttt ctatgcccct ttccagaagg taaggtctcc aagaatttac    143520
     agcaggggat ctcctcaaga gtccataaaa tgtacagcgg tttagagctc gtctgactcc    143580
     tggttttata tgcccctacc aagaggataa ggtctccaag cacttacggc aggggtctcc    143640
     aacccctagg cagtgaaccc gtactggtca gcagcctgat agaaagcagg ctgcacagca    143700
     ggagatgaac agggggtgag catcactgcc tgagctccgc ctcctgtcag atcagctgcg    143760
     cattagattt tcctaggagc caaattctat tgtgaactgt gcatgcaagg gatctagggc    143820
     gaccgctccc tatgagaatc taatgcctga tgattgcagg tggaacaggt tcatcccata    143880
     accacctgcc tccctcatcc atggaaaaat tgtcatccat gaaactgttc cctggtggca    143940
     aaaaggttgg ggaccactgg tgtagaggca ggtctgtgca actaaaaggc cagtggcttc    144000
     tgctgaatcc tataattaat gtcctttaat gagaagtgga gactttggtc atgagggctt    144060
     tcgtgaataa tgcccttcag tcggattcaa aacactgtaa gatcttgtgg gtgtttctgg    144120
     ataaagcact ttgggcagaa aatgcaaaca catgcatgag atccaggcag gaaaaacaaa    144180
     gcttccttta caaagtggtt tggcacgtgg aaggagctct cagggttggg cactgtaccc    144240
     tttgctgact acactttagc cagaggcctg agcctgattg ggtcacaggt tacaaaaacg    144300
     tctgtcatcc ttcagctgag caagcatatc tgcgtgcttg tcagtgtcca ccccatgagg    144360
     ggtgcactct ggaagatgac aagatgtaca caaaccttct cccactgatt atccactagc    144420
     acacactcaa ctgcaaccca tgctccagaa agggacaggg gcctgcaggg ataaacagaa    144480
     tggaagtatt ttattacctg ggaaagacac tgccaaatat cacgtttcag gaaggttaac    144540
     tcataagtgt tcaggaagtg actgaagggc agtggtgggt gagctgacgg gactgcctca    144600
     gggctgcaca tgaggagggc tccctcccac acagcctttt cctccaggag ctacaccaga    144660
     aactcataga agatcaggaa ggattctgag aacattcttc tgtggtgctg cctagagggg    144720
     gaaaataaat tataaaaaat aaatcaattc taaacaaagt atgacatttg ttattagaac    144780
     catttctgaa acctgtggga atcctgacat aagccaccat tctctgtgaa taaacatata    144840
     aacactcctt ccatggggaa gtcaacgaag ttggatcggt tctttcatgg ataacatccc    144900
     ccaaagtcct ctccattccc tgagcatccc cgggtctctc agtgacaagt tctcctcagt    144960
     ggaatgtggt tgatgtagtg agatcttcaa ttttcccgtg ttctgtgttc tcatgttctc    145020
     cccttatctg agattaagaa actcgcctgc atgtggtaca tcacagtcta atgtctctca    145080
     tggacaaaat agtggccaca gcaaccaccg gcggccacca tcccctccac agcctgtgtt    145140
     ggaacatcac ctgagagatt gtattagcaa ctttccttca gaatcatctt aaaaactatg    145200
     tgtcccattt cacatggaga tgtcatctat ccattttctc ttcatagaca aatagaagga    145260
     gatgaatacc caatacactt catatctccg gattattcaa atacgattgt cactttatca    145320
     ccttctgatt tctggccttc gcagaacact ctataaattt ttctcactgg tattatacca    145380
     aaacttacaa agtgctggat acagggatta ttttaaccaa cattattcta atgattctaa    145440
     gaatgtagaa ttacaattat cctcttttca tatatggata aactaaccta tgcctttgaa    145500
     aataaaccct tacataagag tgaaaaggta agctatagat ttggagaatt tccttgcaaa    145560
     tccgttattt ggaaaaggga ttttatcaca aatatacaaa ttaacttaca attgaacaac    145620
     aataaaacca caacatttaa ttttaaaatg ggcaaagacc tgaatagaaa ccacatctaa    145680
     aaatatatat aaaaagagat caacttgatt ttcatgaagt acatgtttgt ttaatatttt    145740
     taaaaattct gctaatctat gagaatgggt aaaatacaca atgtagacaa tatcaaacgg    145800
     tgatgaggaa gttgaaaaac aggaactttc actccttgct gctgggaatg ctaaagcgac    145860
     acatagacaa ctccagttaa taatttactg tatgtttcaa aatagctaga agataagatt    145920
     tagagtgcac ataacacaaa aagtaatcaa tgattgagat aaaggctatc ccagttatcc    145980
     agatttgatc attgaacata aaatgctatt atcaaaatat caattgtacc ccctaaagat    146040
     tttgaactat tatgtatcta tataaattaa acattaaaaa aacaccaatg ccaaggaatc    146100
     cagctaattg cagattccca tttattgtgg gatctggcca gcagcctgca atgcaatggg    146160
     gctctctctt tgttcccagg aggatcagca ggttgagaaa taatagacac acacaagata    146220
     gtgtacactg agtccagggg agtcaccgcc ttctggtccc atggtgccaa caatgcactg    146280
     gatataccag catttattat taagtttagt aagggtgggg gtaggttagt gagggattta    146340
     ggatcatttg attatgaggt gagatggtca catcaggatg aagtaattct ttaacataac    146400
     atctgtatgc agaagtacag tacatttgta tgtagaggtt acagagataa gaatttacaa    146460
     tatagtgtgt gcatccataa tttctaacag agccttaaaa cagaaacaca gtctttccat    146520
     aacctgtgat tagcaagata ttaatcagca gtaacagttg cagcaaaagc tggttacaaa    146580
     caatccatga aacaggacgt gaagctagac aacgggttag accggaattt ctcaaaaggg    146640
     agtatgcctt aaccctaaag aggcttagaa gagccgtggc aagatggggg cgtttatagc    146700
     cctatcttat ccatatggac aggtgccccc catgcgtccg tttataggct ctccacaagg    146760
     gtcgcattcc attcccagag ctatgaacat ctgcttttct gggacaggaa tcttggtgat    146820
     gtgaaacctc cctgactgca cgtccattca caggctctct gcagggggaa gcacatcacg    146880
     cgctgttggc ttgttctggc agtccaacct ggcattgtct ttacacaatc ctgcgtgcaa    146940
     ttttatattt acaataatca ggaggatttc atcttttatt ccatagcaat agtttcaggg    147000
     ggtctcccta cacccgttag atgggatgtg ctgcataatc tgtaaactaa tcagagtctg    147060
     tgggtttgta aaggacttta gaaatcaact ggactacttg tttctaaaga taagtctccc    147120
     aagtttaact gttttcttaa tggatggcta ttttgaacat agaagaaatg ttaacatttg    147180
     tttctggtaa gttttcttct tctagtctca ggagaaaaag tttgcaggga agaatctggg    147240
     accatctttc aagtctcaac ttcaatgtga ttatctcgta aatattttct ggtaccccta    147300
     agataaatct catatctgtc ctagaaacta aaattatgat tctaagcatc caactgactg    147360
     gatggaccct tcctcttcac caaggagttc cagagacttg aaaacctagc tcacgccatg    147420
     ttagaaatag aggatcagaa atgcctcatt ataccctccc ccctttggaa ttcaagcaca    147480
     actaaccagc atttttataa aaacagatct taagcctcag aaaacagatt ctttgtagca    147540
     gcaaaatgcc aaattctaac ttgactcaag aatagcatca catgacagag agcagacttt    147600
     gaaaggaatc aaaggctaga atattccttc gacatatttt aaaatcgccc tgcaaagtta    147660
     tcttttgtga gagaaattta tattctgtag agaatctcct tccctttcca ggtgtttttg    147720
     ttatactgag attagctgag agtctagtat cttttaaagg tcttaataga aaacatttcc    147780
     catccactgc ctctaaaagt ggtcacagga cacaggaagg ggaacatcac acaccggggc    147840
     ctgttgtggg ggttggggga ggggggaggg atagcattgg gagatatact taatgtaaat    147900
     gatgagttaa tgggtgcagc acaccaacat ggcacatgta cacatatgta acaaacctgc    147960
     acgttgtgca catgtaccct agaacttaaa gtataataaa aaaatatata tatatgaaaa    148020
     aagagacttc atatacataa taagaatctt gttctccaaa acccgttatc ttaacctaga    148080
     aacgtttttc tactgctttc aggtctttag aaaaaaactt aagtctttca accaattgcc    148140
     aatcagaaaa tctttgagtt cacctgtaag gtgtaatccc ctgcccatac tgactcactt    148200
     ctagcagttt ttctttatgg accaaatcaa cacgtttctc tcatgtattg gttgaagtct    148260
     gtctctctaa gacattaaat ttaagctgcc atcaagccac tttgggaaca tgttctcaag    148320
     atcttctggg gttgtgtcac agggcatggt ccccacattt ggctcagaat aaatctctcc    148380
     aaatattttg caaagttggc tttttcatcg atagttctaa cagaacctac tctgtggtct    148440
     ttgttacaag catgatatta atatagtatt agttatatta gtattagtta tagcacatgt    148500
     tggcaatttc ttatgaagtt atgcatagac taacaatggg atcaagtgta aatgtttcaa    148560
     agcatttatg cagttatttc aaaatctaca ttccacagat tcttctatca ggttcattat    148620
     atcagctaat tatttgtttt atgatttttt tgcaattttt gcagacagat tctttctttt    148680
     cttggccttt attgtagaaa attgtgtgaa actagtcagg gctgcatttg aaagtcattg    148740
     tcacagtggc tcccagaatc taagtgcatt tcctcttcag ccaccagaca ctcagattac    148800
     tctaatgaca ccttaatttt gttttctgct tggcattggg ccttaaattg tgtgttccag    148860
     agagactctg tctttcatct cacccaggtc atcccagagc cacatggacc tgataattgt    148920
     caatgtggtg ggaagggtcg cacagaacgg gcactctctg tccttctgag ggagccacac    148980
     tccaggcaga catcacagtc ctggctgagg acgtgtcctt ccaagtatcc ctgcccctcc    149040
     tgcaggtgtg atgctgatcc atgcatcttc ctcccattcc tgggtagagg gtctcgttgt    149100
     tttttcccag atcttcttcc agcagccact atgtcttcca ctgatgtctt cagcttcctc    149160
     ttttctgccc ttcccagcag gatgaggcta tttttcctga gggaagagag aggaggtggg    149220
     agatgaggct gttattcctg agagaatgga cagagctttg gagcttttct ttcttccttc    149280
     ccagattcta tgagttcctc cagtgcctgt aacacagagg tgttagtggc tttgcccctg    149340
     catgttaatt ctttggtcat gtagtggtgg tgagggaggt gggtctggat gcatttcagc    149400
     cacagctgct gttccgtttc ccacacagaa ccatctcggg agggagaggt tggagatttt    149460
     tctgatgtgt cctcaatcct ggcagaaaag gtttcaatag caataggatt ccctcagtcg    149520
     tatgtcctct gagaataaac aataacttct ttattatact caattttaaa caatccaatg    149580
     aatatgtcta atttaatttt gcattagatt atatgacatt tgcaggcctc tgccccagat    149640
     aaggtcatat tctcttctgt ttgttcctgc aagtcactga ttttctcaag atgtaaggtc    149700
     tcttgattgt cctataacat caataatctg atttattaaa gaaaatgtgt taatttgcat    149760
     gtcagtaaag ttaattgttg ttgtaaaaat aatatatttt atgggatgcc tacatctccc    149820
     agctgagtag aagctttatt tgtaatactc agaaactgga aattatgcaa atattaagga    149880
     gatttctaaa taaatactat ggttgctgtc ttaaactgga atgctcttcc tcatcagaat    149940
     caacacattc tttatacaca accaaattac tgactcacaa gtaattatgc tgtgcaagaa    150000
     gccacacaaa tgaaattgaa aatgtaagat ttcactttaa caaaatctcg aaaaatagat    150060
     ctgaattaac aaagatgaga ggttgactca gtatggtgag agggaaatga ctgggaggca    150120
     ggaatgagag gaacacaagg aagcttttaa gtgtaattca tcgttgtttt tattgtattt    150180
     tggatgcaca ggtgagcaca tgagaaatta catttttttt ttccctttgg agacatggtc    150240
     ttgctttgtc atccaggctg gagctcagtg gtgagatcac agctctctgc agcctcagac    150300
     cctgggtctc cagcaatcct cctccctctg ccctatgtat gcaccaccat gctcagcttc    150360
     agtgcttact acactgcaca ctttaattat gcaatattta ttatacagca ataatgcctc    150420
     aataagggta tcagaaataa gtgaatagat aatttgttag ataaagattg atggaaagac    150480
     agacactgac atgagaaatg tatgacactc aagaaaataa aactgtagga aacgtgcttt    150540
     tcttcacatt tgttaggtaa tcacaacagt gcgtacacat cacaccatat tcttgttaca    150600
     gagaaaaggt tctgcaaacc tcagtaggtg cgacgccctg tgtgctgtgc ttagttcagg    150660
     gagcagtcag gctcggtggt gagaagcaca ggcccagata cccaggtcac cctgaccaaa    150720
     tgtgaactct agggagattg aacaaccgat ctgtgatttt gctggtaatt tttcatctgt    150780
     tacatggaaa taacattgat actacatacc atggtttcac tgcatatgaa aaaataaaag    150840
     atgatttgtt ctaactttaa acatatgcac tttctgttga tctactgtac ctcaatagaa    150900
     ctgttttaaa ataaaaatta caaaattata agatttatag gttttaaggt tttatcacag    150960
     agcagattta ccataagaaa ccacaatttc ccaaatgcta tcaatatcac aaatctcccc    151020
     aggacactgt cacgtgctct gagccccact ctctccaaag gcctctaacc agagagctta    151080
     ctatatagta ggagacatgg aaatagagcc ctccctctgc ttatgaaaac cagcccagcc    151140
     ctgaccctgc agctctggga caggagcccc agccctggga ttttcaggtg ttttcatttg    151200
     gtgatcagga ctgaacacag aggaccacca aggagtcatg gctgagctgg ctttttcttg    151260
     tggctatttt aaaaggtaat tcatggagaa atagaaaaat tgagtgtgag tggataagag    151320
     tgagataaac agtggatttg tgtggaagtt tctgaccagg ttgtctcttt gtttgcaggt    151380
     gtccagtgtg aggtgcagct ggtggagtct gggggaggct tggtaaagcc tggggggtcc    151440
     ctgagactct cctgtgcagc ctctggattc accttcagtg actactacat gaactgggtc    151500
     cgccaggctc cagggaaggg gctggagtgg gtctcatcca ttagtagtag tagtaccata    151560
     tactacgcag actctgtgaa gggccgattc accatctcca gagacaacgc caagaactca    151620
     ctgtatctgc aaatgaacag cctgagagcc gaggacacgg ctgtgtatta ctgtgcgaga    151680
     gacacagtga ggggaagtca gtgtgagccc agacacaaac ctccctgcag gggtccccag    151740
     gaccaccagg gggcgcccgg gacactgtgc acggggctgt ctccagggca ggtgcaggtg    151800
     ctgctgaggc ctggcttccc tgtcatggcc tgggcggcct cgttgtcaaa tttctccagg    151860
     gaacttctcc agatttacaa ttctgtactg acatttcatg tctctaaatg caaaactttt    151920
     ttgttctttt tgtatttttg tttttgtaac aggaggacac accctcacct ccacagaagc    151980
     cacagtgtca ctttgggggc agatgatcct tctgtggtca gcaggatgaa agttccgagg    152040
     aatctcaggg gaacccgaag agtgtttgcc agttagactc agggcagcga cctccacagg    152100
     aatctctgat tagaacaggc tttgagttct gataggagcc aagagagacg ctcacccagg    152160
     gtcagagtcc ttaaaacctt gtggttttca cagcaatccc ccctggtctt gtaaaactgt    152220
     gtacatatga ctcagactga ttcacttgac cccctttctg ctaatccatt ttccttctct    152280
     gcatacttga ttctcacagt tccctttctt cttctctttc ctgaaaacag aggatgtgtt    152340
     ttctgtagtc aaaatcccag agatcaggtc tgcaggacct gggtaggctg aggggacttt    152400
     ctcactcacc ataggctgat gacactcctg ctgtattttg tgtgtggagg tgtttggaaa    152460
     acgaaatgaa cattagtcat gaagggaata atactagttt tctccaaagg gatgtggatg    152520
     tagagctgat cttgtgcttc tcacactgtc acagaattta tgctctcccc tgtgacttta    152580
     ggagagctga ggatggacac tccattgtgc tgtgagctct ggtaatagta attatagggt    152640
     ctggctaggc agcctaaggt caatactgct ggccttcggg aaaggcaggc tgggattcct    152700
     aggaaaacct gcatctgccg tccagcatgg agtcccatcg tcttctgtta tgctctgatt    152760
     gaatcaggcc cacctaggtt atctagaaca ctcttcgtga cttgggcaaa attagtggca    152820
     ggctctacta agacctgcat tatgccatgg gagcaacacc taggctagtg tgtgattgag    152880
     taggtgagac tgtggtctag tcaaggtggc agggaaaatt gttgccatta ttatgtttta    152940
     ttttatattt ggcaatacag tcatgctcat attacaaata cctttctaca tttttcgtgt    153000
     cagaagcttt tgaacaagag caacttcatc ttgaataagg gctagaaaaa ataagactga    153060
     gacctgctgg gctacatttc cagtaagcta aggcatcctt agtcacagga tgagatagga    153120
     ggtctgcaca cgatccaggt tataaagacc ttgctggtaa aggttacagt aaagaagctg    153180
     ggcaaagccc accaaaacca agatggcaac aaaagtgatt tctgttcatc ctcactgctc    153240
     attataatgc cttaacatgc tgaaagccac tcccttcagc gccatgatgg tttacacatg    153300
     ccatggcaac atcaggcagg ttccctagat ggtgtacaaa ggagggagga aacctcagct    153360
     tcgggaattg cccaggggac tcatgaataa tccatccgtt gtgagaaata cattcatccc    153420
     tttgagagaa aatgcacagg gggtggaatg aggctgggaa gctgatggca catggtggaa    153480
     gcctgtccct gagtgaaaga gagagggaag ctggattggg tggaaggtcc ctatatttct    153540
     gtgctgtgca aggaaggtgc aaaatataat tgagtcttgt gcaagtcagt gctgcctctc    153600
     aggggatccc catgactccc agaaataatg gctctgctta ggaatccctg cagagtcact    153660
     cctttcatta gagtagacca caggacatgg gcctcagcac ctgccatgcc acagatgtca    153720
     gaaagcagct gctgggaacc tgacccacct gcattttgct gcctgtagga ggagggaggg    153780
     acgtgcattc tcagggccaa cacgctgttt ttggattttt atagagaaca cctgctttta    153840
     ctttactttt tggaaaaata tatgaacaaa tgtgcacctg caaacaattg taattttcac    153900
     atttatttta attacatttg tttataattg tgcatgaggt cctggtgcag agttgagttt    153960
     ttcatgggaa ttgttccaag aatcaagaac atccattttc cactttcact ggtttttccc    154020
     catgtgcaga gaccttgagt agagcacatc tggcccttat ccacactatc tcttgtgtcc    154080
     ccgggaaaga gcagggattt gcctgactgc agaatctggg gtaggagtct gcacacctct    154140
     gagcctgcag agaagcccag agaagtttta tggagtcaga gagctttccc gtgtggggaa    154200
     cctctagctt cctcactctc agagattgct ggtcagctgt aattgagggg ttaagaatta    154260
     gagcacctgg aagcctgtct catcacagct ccattatgta acttaccacc attagactgc    154320
     aggtaataac tcaatcttga tttttctgac cccatcttat tcatttgtaa gtttggttat    154380
     agaaaatgtt acattttagt ttctgattca aacctcagaa ttttgtgaat tcatcaggaa    154440
     taattaaaaa tgtgtccagt ttttgggaaa tgtaaattaa tgtccatact gtttatgtgt    154500
     gtgtgcgtgt gtgtgtgggg ggggacatgt cagtcactta caccccagta aaaatgattc    154560
     cttaattata tagtcgaact gcaattagat ttgctgaaaa ttgtctacag taatttctgt    154620
     gaagctgacc ttgccacctt gctgtgaggt gtgaggatgc tcagtgcaca ggtgaggtct    154680
     cacaggagag atccaaggtt cagcccccag gactggctga gctcctggct tggagccaga    154740
     cataggactg ggcatctcag aagcagatcc cctggccctc ctttatctgg gctgtatggg    154800
     cagcagagga caagcagaag aaactcagaa ctcagccaag acagagcccc atttaggagc    154860
     aggtgcatca aggaaccagc cagagaacct ctgactacaa tctagggttg tcttcttttg    154920
     cttcacagca tccaacccta aggagaccac ccactatatc atctgtaact cagaactttc    154980
     ttgttacaaa ggaaaacaca aatgaatata actctgcagt taacttcttg aacctggatt    155040
     gatgagtttc catggcctca tgagcacaga gaccacatcc agtgcaggtg cagagcaagg    155100
     ctgcaacagt cagcctgctg ctccctcagg agtcttccaa ttcccttgtc caaagcatcc    155160
     gctcattcat gggctgagaa aagggaagtc attcatgcag tctactctct ccacagaact    155220
     gacggggcac aaggacaaca tcgatttttc atggaacatg cctctaggaa tgcagctgtg    155280
     tgcacacaca ctgctaagca cacacttctt tacataatta cttgtaactg tattttctta    155340
     tttattctct ccaatttttt tacacaaatt catcactttt ccccataatc aaagaggatt    155400
     ttgatcagaa tgcttgtggg gagccccttg cctgccagat gcccaacatc actactcttg    155460
     aagggaggag gaacggcagc tctcttgatt tctactctaa tcctctagga ctaaaaccag    155520
     aaggttgcat gtccagtgcg ggagcatcga agaagatcct gtctgtagaa gcaggagcgt    155580
     caagacttga ctgagagcca tggtgctgaa atgagataga ttccctgatg gagagcacac    155640
     gtggaccccc acacctgagg gctcactgct cctcaccaca gatgcactcc cctactgagt    155700
     cctgagacct gagtgcaccc catagagtag ggctcagatg aggggatgca aatctccacc    155760
     agctccaccc tcccctgggt tcaaaagacg aggacagggc ctcgctcagt gaatcctgct    155820
     ctccaccatg gacatacttt gttccacgct cctgctactg actgtcccgt cctgtgagtg    155880
     ctgtggtcag gtagtacttc agaagcaaaa aatctattct ctcctttgtg ggcttcatct    155940
     tcttatgtct tctccacagg ggtcttatcc caggtcacct tgaaggagtc tggtcctgcg    156000
     ctggtgaaac ccacacagac cctcacactg acctgcacct tctctgggtt ctcactcagc    156060
     actagtggaa tgcgtgtgag ctggatccgt cagcccccag ggaaggccct ggagtggctt    156120
     gcacgcattg attgggatga tgataaattc tacagcacat ctctgaagac caggctcacc    156180
     atctccaagg acacctccaa aaaccaggtg gtccttacaa tgaccaacat ggaccctgtg    156240
     gacacagcca cgtattactg tgcacggata ccacagagac acagcccagg gcgcctcctg    156300
     tacaagaacc caggctgctt ctcagtggtg ctccctcccc acctctgcag aacaggatag    156360
     tgtggctgag atgccatttc ctgccagggc ctgcgtttcc catccccatc tgactcagag    156420
     ccttgttttc ctccctcttc tttactaata aatggcatgt cccctgttag tggttcgtgc    156480
     aagcagaagc tgtatcctgt ttgacaaaga ttcagcatga aaggttcctg ttacctaaaa    156540
     aaaaatagac agatgagact taattaacct aaataatttt tttcacaaca acagagtgaa    156600
     tacacaattt gcagaatgac agaaaacttt tgcacacttt gtctgtgaca gggaactaat    156660
     atgaagaatt tgcaaggaac tcaaacaact ctacaacaac aacagcaacg agaaccaaat    156720
     aaccccatta aaatgagcaa agaacatgag tagacatttt caaaagaaca catagaaatg    156780
     gataataaat atataaacaa tgctcaacat cactaaccat cagggaaatg caaattaaaa    156840
     ccacaataag atatcatctt ccaccagtca caatgactgt tactaaaaac tcaaataata    156900
     tcagatgttg ctgaggatgg gaaataaagg caactcttag acattgttga tgaggatgta    156960
     gacgagtaca acctctgtgg aaaatggtat ggagatttcc cagaaaacta gaaatagaac    157020
     tgccatttgg tccagcaatc ccactactgg gtaactaccc aaaggaaaat aaactattat    157080
     ttcaaaaaga tacccacctt ctatgcttac cataaaacta ctctcaatag cacatatgtc    157140
     aaactgagtg tctgccaaca aatgatttta taaaagaata tagcacgtat gcacaattca    157200
     atactagtca gtcacaataa ggaatgaaac tgtgtctttt gcagcaagat gcatagaagt    157260
     gggggacaat acaattagtg aactaactca caaacagaat gtcacatgtc acatgttatt    157320
     acttgtaagt gggaggtaaa cagtgtgtac acaaggattt gtagagagaa attatacaca    157380
     ttggagactt agaaggatgg gtgggcagaa ggtgggagca tgatgagtca ttacataaca    157440
     ggcacaatat aaaataatta agaattgacc aatgatctta aaattaaaat gtagaatatg    157500
     atcaataaat gaacttgata ttagttgacc tcattaaatt taaaaacttt ttctactcaa    157560
     gtgactgtaa gaaaatgaat gcccggttac agatgagaaa ctgtttgcga gtcatataac    157620
     caccaatgta attataataa gaaccttcag aactcaactg tgaataaaaa agaaacaact    157680
     gatggataaa ttaggcaagg gtttctacag acatttcgtc agagaagatg tgcagatgac    157740
     actgaagcat ataaacaggc tctcaacagg attttccatt agagaaattc aaatcaagcc    157800
     cacaaagaga caccactgta cacttttcaa aatggctgaa attaagaaga aatacagata    157860
     acatcaatgc tggtgagcat accaggttgc tagaggctaa aacattgcta acgggaatgc    157920
     aaaatgaaac agatactcag gaaaataatt tttagttttc tctaaaatca aacataccct    157980
     taacacctga atatttgcat cagagaaaaa caatcttaca ttcacgcata acttctattc    158040
     aaatattcaa gatatcgtgt gtatgtgtgt tagaaagtaa aaataacata aatgtctcaa    158100
     aatttgaata ggtgaagaac taggaagcat ctataaattg aataccacca gcaataaaaa    158160
     aaaaaacaag tgaccaatac ataaaccatt gcaggtgaac tccagacatt gtgctaagtg    158220
     agagaagcca gtctcaaaga tcaaagggac acagctgtaa gcaccacggt catcctcagg    158280
     tgtcagtggt ttgggctgga ctttctgtgt ctctttcctg accagaccca gatgttgagc    158340
     tccaccactt gcagatggaa aatcctattt tcaaccatgc agtgaggttt caactacttc    158400
     acagactgaa cgacacaaac acgggctcct ttgaacagcg tccggcattt gttccaacca    158460
     caagagaacg tccctcagct ctcccccctc ctcggttctc tcctgcaagc cagcagccct    158520
     gcagtttagt ctgcatctcc catgcatcca cccatctccc tccaagcacc ttcccccaca    158580
     ccctccactg tttctgagat cacaggcagg ctttgaactt ttccgcattc tgttgttatt    158640
     gaagttagga tgtttaggac caacttaagg atcatatttt atgactgaat tccagtgccc    158700
     cttctctcct gggacagagt gcataaccaa gtttctgcag gtggagacga agttgagctt    158760
     ttttcttcct cagcctagga gatgagcgct aattggaggg ttgggcagaa gcttcccacc    158820
     atcccagcac tttggttctg gtggggcgga atcggtgcca tagggcagag ctagaaaccg    158880
     cggactgaat gttcccagtg gcactggacc cagggcagag cctccatcca cgagtggggc    158940
     tctatggaag aagtgagtct ctggctctca gtagctctcg tccagcactg aacctcagca    159000
     tcatgtgctg tgtgcagggt cagagggcca acgtactggc ccctgggaaa gcgtttcctc    159060
     tggtgggagt tggtagaagg tgtcctgtct tcttggctgc atctgtccgc agtggagttt    159120
     acatcatgct gagctgggat gtggaaggaa ggaagagcat cttagatcaa atatgatgac    159180
     tggccttact gagttttcta gattttcctg aataaatgtt tcttcactca ctgtgtgctg    159240
     ttagagtctt tccaaacctg taatttccca aaataatttt cactggtctc atgagggcat    159300
     ggattcattg agcccctcat gctgtcaaag agaaatagaa ctgttttttt tttcacttca    159360
     tagcgaacat ccatgggtta tcaaataatg ggctggcttt tcttccaaca ctttacagac    159420
     accatcaatt ttcttcttgc ttataaggtt ttaaccagaa gaatgctgtc atggtctttt    159480
     ctgttctttt ggaaggaatg ccccctctac tcacctccac ttgtctgcct gtatttctat    159540
     ttgtctttgg ttttcaacaa ttttaataag atttacctaa atgtgtgtgg ggggagcatg    159600
     gggtgttatt ctgctgttct gtgttctctg agatgcatgg attcaccatt tactctgtct    159660
     ccatttttgt gaaaacaatt agaaaaaatg tcagtgtgag cctagaaaca agcctccctg    159720
     aagtgggcac aggaccactt gggggcgcac aggacccact gagcacaaga gccaacctca    159780
     gggcaggtgc agatggggtt taaggtctgg tttcctgtca gccctgtggc ttcctctcca    159840
     tgaaacagtt tcctttgtgg catatctctg gattccttat cctgttcttc ctgtgaagtc    159900
     tctgaagaag aaacatttgt cgtaacaaga gaaaaacttt ctcacatgca ccaaaggcag    159960
     agtcacctac agtcacttac tcctgtttct caatgtcaat aagttatcaa tgcttctgaa    160020
     gttaatcagc taaatctata aaggtgcagt gtttaactca gcattgcagc ccagctcaac    160080
     agaactccaa aggccagcca gcagcagcca ggataatgag catgctgggc attggggcag    160140
     aaggagttag catccagtgc aagagaagaa agcccccgtg gtggtcattg tcaggactcc    160200
     aatcccacag ttccaattgt aggtgatgcc aggcaaagga agagagaccc caccaatggt    160260
     tagtgtggat gtcgagtttg atgtttccac actcacactc caggtgaata tgaaaagatt    160320
     tattagctct atttctgagg tgtctgctga gagcagcaca gtcctctcaa gaaattacag    160380
     attggaattt cctcagtaga gcaggaaagg aggctggctc agggctttat aatgatttgg    160440
     tggtggggtc ggcggggggg gggggggcgt ttctactcag gagaaggagc ttgtgtgatt    160500
     taaacctcac actgacatca catgagggag cttccatgat ttcttactag atttcccatg    160560
     tgtgggggac aaggatgagg gagaataaac cttaattcat cagcatcaag gcaccaaaaa    160620
     taggacctga cactttattc tccctagcag cttaagaaaa tgagtgaaaa agagagataa    160680
     gagtccaccc atgtgctgaa aagcatagct cttggtaaag acgagaaaaa ggcactccta    160740
     cgaagaaggg gttgggcaga agctttatgc tgaagggttt ggctaaagag acataatcaa    160800
     caggttacag gaggggctac tgatgttcat ggaggtggtc ctcacacatg catactgaac    160860
     aaacatgtct gtaacgtatg acccctgttc acttaccagt ggagacttag catttaaatt    160920
     cattccagtc aggccctatg tgcaaacagc agaagcagag acacaaaggt actcagggtg    160980
     cagcctctgt gaacggccag agccaggcca tggtcagcgg tctcggatta ggagaaagtt    161040
     cctgatatca ctgtagtgtt caatcaaagc tggggttatg gtttgtggaa caggggtcag    161100
     ttcatcaggg ggtgggctgc aattgtcttc atagtgcttg tctcagtgcc ggtgcttact    161160
     gagccactag agaaaaaggt ttaattgagc ttctttaaaa tcaacatttt gaattattta    161220
     tcagacgttt caaatatgtc atgttgttta gattctattg ctggagagtt aaggtgatat    161280
     ttggggtttt gtaactctgt tttttcatac ttcctgaatt gcttatctgt ttgcttttca    161340
     ttagctaaac tatcgcttct tcttattttt taattcattc tgattttgat gaatatttaa    161400
     ttccctttag aatgtgaata taatgtacat tgtgtgggta ttttgatttt ggttcttggt    161460
     ttacttagtg gcaaagactc tgtaagagtt ccttgtctat agatagccat tatttagtgg    161520
     ctttctgaaa tggtggtttt agtaccaaag tactggactt gtgagtaggc tcactgcccc    161580
     ctgcaggtcc tagatagtgg aggcctcagg aactgtttct catttggaat gcctttgttt    161640
     cagcagattt tgtgttgggt tgttaagttc accctccaca ttagtagatg tccttacaga    161700
     ttagagctga ctctggtaga agcagttgag tgcatgcttg atatctgtgc acagggagaa    161760
     gctctctgtt gcctcaggcg atggactggt ctatgaaatg cacagtgacc tgagttccct    161820
     gctcagcccc tgagaggtgg accaagctgg acacacatga gccaccgagc ctggcaagca    161880
     aaagcgccag ccttgatgga aatggcgagc tgagcggcat ctactcagtg tggtttcttt    161940
     tgttattaag agctttagtg tggtggctgt ttcaaattcc cgttgtagta gtaatatact    162000
     gggtatgtga gcaggcccgt ggtcttttgc ggggttggaa tcaccgaagt aatgagaagc    162060
     taatctcatt ttcaactgct gtacactggt ggtattgagt ttgtatgagg tcatgcagtt    162120
     tgaacgtcag gccagtaggt ggtgctcgca ggtaagagcc ggctatggtg gcagcagaag    162180
     ggtttatgct ttactggtga ttaaagtggg aaacttggcg tgttccagat cttagagaaa    162240
     agatttttag ttatttctca ttcaacctga tactacctga aagtctctcg aatgtaactt    162300
     ttattttgtc gagatgggtt ctttctatac ccatttttta tgtttttttt gtgaaaggat    162360
     gttgtttcat caaatgcgtt ttcagcatca attgaaaaaa gttatatgtg gattaaagat    162420
     caaaatgtaa aacctaacac tataaaacct ctggataata acataggaaa cagaatttag    162480
     gaggtaagaa ctgacaaagg ttttataatg aaaatgctag aagtagttgc aacaaaattg    162540
     aaaattgaca aatgggacct aagtaaatta aagaacttct gtacagcaaa agacactatc    162600
     gacagagtaa acaggcaacc tacagaatgg gaaataaaat atttgcagcc tatacatctg    162660
     acaaaggtcc gacacttagt atatacatgg aaatttaaca aacatacaag aaataaaaag    162720
     tgaccaaagg acatgaaaag acacttcaaa aaagacctac atgtggccaa caagcatagg    162780
     aaaaaatgct gaatatcact atcattagag aaatacatat caaaacctca atgaggtacc    162840
     gtctcacatc agtcaggatg gctaatctta aaaaaaaaat aacagatttt taaggttaca    162900
     gaaaaaaggg gaaatttata cacttttggc gggaatataa atgagttcaa ccattgtgga    162960
     aagcagtgtg gtgatccctc caataaccta aaacagaagt ttcatttgac ccaacaatcc    163020
     tacaactgga catataccta aaggaatata aacatgtagg ttcactgcag cactatccac    163080
     aatagcatag acatggaatt tacctaaatc cccatcactg gcagaatgat agagaaaaat    163140
     gtggtacata caaccatgga atactatgca gctaaggaaa gaatgaaact atgtcctttg    163200
     taggaacatg atggaactgg cagtcaatac tcttagaaaa ctaattcagg aacagaaaac    163260
     cagatattat atattctccc ttatttgttg gagataaata aaagcaaata ttcttccagg    163320
     gcctgagtct tccttattca acaagtcatt ctaaattaag tgttcagcaa gttgctgata    163380
     ctcatctaaa tattctattt catctgggcc acttacatca ctcaaaaagc aatgagagct    163440
     atatttctaa ggggggttct aggataataa atacctgaat agtgagaata tgaaggatat    163500
     ggaaactggg ccacttatat cactcaaaaa ggaatgagag ctatatttat aaggggggtt    163560
     ctaggataat aaatacctga atagtgagaa tatgaaggat atggaaactg ggccacttat    163620
     atcactcaaa aagcaatgaa agctatattt acaagggggg ttctaggata ataaatatct    163680
     gaatagtgag aatatgaagg atatggatgg ttttttttta actcaatggg cacataactg    163740
     tgggagatac tatattccta tgaagaaggt attcagactt cagagataag taatgtttcc    163800
     tacattgtgc ttgtgacttg gaagcagtgg attgaagagt gtgataagtg cccagaccaa    163860
     gcagaacaga aatcagcatg taaagatgat gatctatgga tatgatctaa aaccatgtaa    163920
     atacttcaaa taattctatt taatgcagtt tgaaataaaa cacaaactta ttcaaaatac    163980
     aaattacttg gtaattattt tgggagctat gagttcacca agaaactcaa attcctattt    164040
     ctatttcaac ccctgattcc tactgtcaat gggagggaag tctcagaacc aatcacacat    164100
     cagacggcaa atctgtcaac caagagtctt tccactgaag gacctgggag gtcaggaccc    164160
     tcaggaaagt gctggggacc ctgtcttggg agtgcccagc agatctcaga actctccatg    164220
     ggtcctgctg gacactcatg tagggtaacg agtggccacc ttttcagtgt taccagtgag    164280
     ctctgagtgt tcctaatggg accaggatgg gtctaggtgc ctgctcaatg tcagagacag    164340
     caatggtccc acaaaaaacc caggtaatct ttaggccaat aaaatgtggg ttcacagtga    164400
     ggagtgcatc ctggggttgg ggtttgttct gcagcgggaa gagcgctgtg cacagaaagc    164460
     ttagaaatgg ggcaagagat gcttttcctc aggcaggatt tagggcttgg tctctcagca    164520
     tcccacactt gtacagctga tgtggcatct gtgttttctt tctcatccta gatcaggctt    164580
     tgagctgtga aataccctgc ctcatgcata tgcaaataac ctgaggtctt ctgagataaa    164640
     tatagatata ttggtgccct gagagcatca cataacaacc acattcctcc tctgaagaag    164700
     cccctgggag cacagctcat caccatggac tggacctgga ggttcctctt tgtggtggca    164760
     gcagctacag gtaaggggct tcctagtcct aaggctgagg aagggatcct ggtttagtta    164820
     aagaggattt tattcacccc tgtgtcctct ccacaggtgt ccagtcccag gtgcagctgg    164880
     tgcagtctgg ggctgaggtg aagaagcctg ggtcctcggt gaaggtctcc tgcaaggctt    164940
     ctggaggcac cttcagcagc tatgctatca gctgggtgcg acaggcccct ggacaagggc    165000
     ttgagtggat gggagggatc atccctatct ttggtacagc aaactacgca cagaagttcc    165060
     agggcagagt cacgattacc gcggacaaat ccacgagcac agcctacatg gagctgagca    165120
     gcctgagatc tgaggacacg gccgtgtatt actgtgcgag agacacagtg tgaaaaccca    165180
     catcctgaga gtgtcagaaa ccctgaggga gaaggcagct gtgccgggct gaggagatga    165240
     caggggttat taggtttaag gctgtttaca aaatgggtta tatatttgag aaaaaaagaa    165300
     cagtagaaac aagtacatac tctaatttta agataaatat tccattcaag agtcgtaata    165360
     taagccaaat tcacagagtg gaaaaggcca cactctataa cgttgataca aacattccat    165420
     gaaggtgcta ctgtgaacaa gttttcaaat tggatgaata catgatttgg agcaaggtta    165480
     tttgatcatg tggtgagact aagaatgatt cttaaaaagt gccaaaagtt tccttcaaat    165540
     gtttctgtca ctccttatca taaagtttat tttacagcag ttttaggatt acaaagaaat    165600
     tgcacaggag gcgtgagaat tcccatgact ccctgcccta cacaggcaca gcctcctcca    165660
     ctacgaccat cctgcaccgc agtcacaaat cagttacaat ggaggaatct ccaaggacgc    165720
     ttggttcttt ctttttctgg tgatctccta atataacaag cctaagtatc tcaagattcc    165780
     acggtttttt cagtgttttc tagaactgat attagtcaga gggaaagtgg gtaaggctat    165840
     tactatttga actctttctt ccaaaatcca caaaatatat attaatttag agcttatctt    165900
     acttctggtt tacaatgctc cttcccagag agtaagattt ttaagctttt agaggccagt    165960
     tcatgtctct aaaagaccag aaagttctgg ggaatcccat aatgaacatc ctttcattgg    166020
     aattggagac cctggcaatg agagattcca tgtataatgc cctagagttg gattaggtgc    166080
     actgtgagct cttgggtggt gagtctgagt aaggtggttt gtgcagcaaa tgcaaacaca    166140
     ttcatgggac ccaggtagga acaaaagctt ccctctgcaa agggagtgtg cacctgaagc    166200
     agccctcaca gaggtgggca ctgcttgccc ttgatgagtg cacattagcc agaggcatga    166260
     tcatgattgg tcttgcagat aaagagcacc actgaggtca taggttatga aaatgtttgt    166320
     catcctccag ctgagcaagt ccatctgctt gtttgtgggt gtcaactcca tagagggtgc    166380
     actttgggag atgacaagat gcgcacaaac ctgctctcac taattatcca ctaccacaca    166440
     ctcaagacca acctgtgctc cggaaaggaa tacgtgcctg tggaaataga cagagcttaa    166500
     gtattttgta acctggtgaa catactgccc aaagccacac gtttcaggaa gattagctca    166560
     gaaatgttca ccaattgact gaagggcagt ggtgggtgag gtgatgggac agcctcaggg    166620
     ctgcacatga ggagggctcc ctcccccatg caggcttttc ctccaggagc tgcatcagga    166680
     cctcaaggaa gatcagggag aattctgaga acacccttct gtggagctgc ctagagaaga    166740
     agaataaata tgaaaaaata caactctgag taatgcatgg atatttgttc atgaaaactc    166800
     tctctgaaag cttgtgaagg tctcgaaata cccctgatta gctgaagaca aacatttaaa    166860
     ccctccttcc acagggagtt caagcaggct ggatgtgtcc ttctatggat gatcttcccc    166920
     aaccccttcc tcttcccagc tcatccctgg ctctctgtgt aaacagttct catcagtgga    166980
     atgtggttga tgaagtgacg tcttcaattt cctcatcttc ttggtggtca tgttattttt    167040
     tttcatctga gggttaaaaa ctcacctgca tgcagcacat gacaggctaa aatctcttgt    167100
     ggacaaaacg gtaacaaagg cacccaccag ggttgagcat ccgtgttgct gacaacgacc    167160
     accaggggtc aacgtcctct tcacaatcct gtgtcagagc atcactggaa tgatttcatt    167220
     agcaacttcc caggagaatc agcttaaaaa atacttgtcc cattttccat gcagatataa    167280
     cccatccctt ttcctgaaga aacagagctc aatactaaat acactgaatg ttgtttttgc    167340
     tggttttgta agtttgtgac tttatcactt tctaatttct gagttaggtg gaccactcta    167400
     catatttctc tcatgggtgt gaccagcctt ctggatgtca aatataactg actttgttcg    167460
     tgtaaatgtc aacacaagct cttcatggtt tcggtgctca ctgactactc taaacttaca    167520
     caatgtgttt ctttattagc tttgttttct ggtatctcta cgtggcttat tcatcacgcc    167580
     cattttatgt tacttgtaca ggtaattgat tgttattttt aaaatttact attgcttact    167640
     ttaattaaat aagtaggcaa tagcttagaa tagaaaataa ggtaaagtaa aagtacatgc    167700
     agtaatattt aatataagta agcaggcagc tgaaagcaag ggaaatctac tctgctgtgt    167760
     aaaagggtat ggagacctca cagtatgatg atcttctgca gtttaatcca tactgctaca    167820
     taagtgacaa cgtccactct gtttatatca gagcttcccc taatatgtac aatgaagact    167880
     gagcccaggt acctccaacc agaatggcca gcacctttta ccaagtgcct gaactgacag    167940
     caatagagat cacgtttgtc taaaaaaaaa ctcacactgt aaggataccc accagtcata    168000
     tggagacaat tagaaaaaat taaagcccaa gcctgatagg tagtaatttg tacttagaag    168060
     aattaacttt tatctccaca tgtttttttt ttaatcatag agaaactact agtggtagtt    168120
     ccactgtcca agttactgaa tatcttattc ctagaatgag acactgcaca atgtgttctg    168180
     acacgacaga acgtagctgt agttaaggga caggagatgt gatcacggga acacgagtct    168240
     gcgatccagc agttcttgct ccttcacatt tgcccagaaa caaagacccc agcagaacaa    168300
     tggaagcatt gacttaggtc tccacagtct gatgtgagat aaacagcagg tagtttatgc    168360
     ctctgtttta tagcaggaaa tttaatctct aaagaagggc cattttggag ataatactat    168420
     tctccaggat gcttaaatgc ccccaaacag agagcactgc agagccccgt ggccaggagc    168480
     tacaacacct gggtctggaa accactggac agaaatatga gattctctta ccacatgtat    168540
     gatggaacct tgaagaatgt ttccttcctt ctccataatc acagcctgcg atgaactgga    168600
     ggtcccagta cataaaaaaa catgtgtctg cattggatac agaattagtc ccataaaata    168660
     attgtgctac tgtggtgctc acactgttgg accagctgac caagaaagtg ttgtaagagc    168720
     tgcagtgtct actgattcct accatatttg gggctacatt cacatctatc aagggaaaga    168780
     gagataagaa aatctctcta ggccgggcgc ggtggctcac gcctgtaatc ccagcacttt    168840
     gcggggccaa ggtgggcgga tcacgaggtc aggagatccg gaccatcctg gccaacatgg    168900
     tgaaacctcc atctctccta aaaatacaaa aaattagctg ggcgtggtgg tgggcgcctg    168960
     tagtcccagc tactcgggag gctgaggcag gagaatggca tgaacctggg aggcgtagct    169020
     tgcagtgagc cgagattgtg ccacttcaac tccagcctgg gggacagagc tagactccat    169080
     aaaagaaaga aagaagaaag aaagaaagag agagagagag aaagaaaaat aaagaaagaa    169140
     agaaagaaag aagaaagaaa gaaagagaaa gaaagaaaga aagaaagaaa gaaagaaaga    169200
     aagaaagaaa gaaagaaaga aagaaagaaa gaaaggaaga aagaaagaga aagaaagaaa    169260
     gaaaaaggaa atctggccta gtctattgat ttattttcat ttcttcaaaa tgaggtaaaa    169320
     aaatcgcatc aactaaaatg cggacgatga tgaagtgatc aaagacaaca gccactcagt    169380
     gtttcaggtg tgttttaacc caaagtcaca caacatgtga gaagctgaac tccggagcca    169440
     ggggcatctc tgaggagaat gtggaaggtg ggcgggagga aacagtgatt catgtctagg    169500
     gaaatttcag ggctgcctgc tctaggggaa aggaaagcct gagcctctcc cacagtgtgc    169560
     tctgttaaaa attttggctg acttattgta ttcttcgaca caaatatttt tgtttcgtcc    169620
     tcttttaaat ttttttatct ctattaaaat tctcactttg ttcttgcatg ctgccactag    169680
     cttattaaac atgtttctaa tatttacttc aaattttctg ccaggacatc catatgtttt    169740
     tttattcatt gatgccagat tctggacctc tatgttgtct gtctgtttgg accatgttcc    169800
     cgtgtttctt cattttcctt gacggtcttt cttgctatcc gttcatcata acaaacagct    169860
     ctttgctcat cataacaaag agccacactc ttccaagcct aggctcatag agacaaccct    169920
     caccaatcac tccatcagtg attgcggatc tctcaaaact tcataatgac ttcataatat    169980
     tccaacttct gtctctgttc ttagtggccc ccaggtttag agaacgttgg gttgtgccag    170040
     gactcccaaa taggagaggt agaaactata tactcaagta gcctcttgaa cagtgtacca    170100
     ttagacacag ttttattatt gaggtatgat tcatcaaaat aatgttagtt caagcgcctc    170160
     tcaaccagtc atctaacatt gaaatgttat aatttcttat ttggatctct ctggagttta    170220
     tcttttgaat atttaggtaa aattacaaac tagtataaag ctatttggca aaattatctg    170280
     ctgcagaagt ttcatcttct gtgtatctct ttttgcactt aaatatgttt ttattagtca    170340
     tgtatccaga atatagaaat atgtcttaaa gttccttcat ttttttaagg ttatggggtt    170400
     ttcagaagag gtgagaacct tctttattta taaaatacct gattccacat tgctggatag    170460
     tttcttcttt aatttgtctc ctcctctcac attatactat attaagcaag gagaaatcaa    170520
     aacacaccat ccacactttg cttagaaatc cccaaagcta aattttaagt atcacttact    170580
     aattctaatt ttacccattg taacttaatt aacatgaaat ctataacaag gttacacata    170640
     ctttcccgtt ctaataccat gttcgtgaca gaaacaaagc aggtgtcttc tctgcaatgt    170700
     ggtcatctga ggtctgagat gaggggtcac acaggtattt tataatattt aagggtttgg    170760
     acaacagtag tgataacaat tctgaacagc actggctcct aattgaaagg ttataggtaa    170820
     atttaatgct gcaattttat attaacacct tgtccgaatg aagctcagag gaagatgcca    170880
     catcccaggt cacacagtga ggaggaatgg agctgtgctc tgctctccac accacctaca    170940
     aatcccaggc cccagcccag gttccactgg cgcacggcac ctagagcaca gttcccaggg    171000
     gatgatctca gggcacctgc ttcctcgggc agggcgctgc cttctctcag ttgtgcactt    171060
     gctcctcctg caggttctgt agtgagcact tgcattgccg tgatttatac atgttctctc    171120
     ccctaatatg gaaaaaatat ttattaaatt ttcaactcat tttctctgat gcacactgca    171180
     ttagcagaaa ggaataaatc acattcctta tcctcccata aagccaaaga ttcccgaaga    171240
     cagagctgat gtgatgtact cataggtgga tctctgcccc tcaagggagg ccttggtctt    171300
     caagtttcag taattctagg aagcgaagga cacctacatc tcctgctccc tgctctgtag    171360
     ctcacctgag aacagctttc tcattggaat gtcttgtgtt taaggaataa gagtccatgt    171420
     ttcaggttcg ggagcccagg tgcacctact ggatgcagcc caggattgga gacactttcc    171480
     agaagacaac atcacctgag acatgaccag tcccactgtt tcactttcac aatttcaact    171540
     tcctcagaag aaaattaaaa ttgctgagac ttgttcataa gtgttgtgcc atgtccttac    171600
     tctgttttct tgcctgttca tttatgtcat accaggtgtc tgttatatgt aataagatca    171660
     aaattctgcc tccagtaaca catcaatgga gacctttgat tgtacttttg gtttatgcac    171720
     tgacacatag attatgatgt tcatcacatt catttttatg tcaaaggaaa tctgcataat    171780
     ctgaatgcca atactttttg gaatctgcta agtaactgaa attgaaaaaa aaatacccac    171840
     tcaagaacct ggatagacag ccatgtccag aatggcagtt gacacttgtt taactggaag    171900
     agaatctaca gaagccacaa gttgttgagg gcacttacat gataagcact atagttgtct    171960
     taaagacaga tgtggactca gtaaatgtga ctgttccaga gggtcttata tttctatgtt    172020
     ttatggactt tctcaccaga aacctccaga ttctaaaaaa tactatccaa atacgtttct    172080
     tatttgtcag attggagaag attaattact gaaaaatatt acgggagact ttttcaaaag    172140
     cttctacatg gaaggacttt tcgagaacct tgtcctatgt aaaggaagac aaatctccca    172200
     ttccagattt ctctcccatt cttccattat tatagaaatg agcaaagtta gccaataggg    172260
     gtaagatgta agtaaatagt ccagggacac tgaaaccaca aaagggagta atggccaaag    172320
     tagcttttcc cctggagatt cctggtcaaa gtcacagccc agaagaggaa gccgatcgca    172380
     tctctaggtt tccattgtca aaacaggcag tgcttgcctg cactgcacaa tccattctaa    172440
     ccagtgtgat agctctggat taaagatgga agtgtggcaa tgcacagact ctatgtgaga    172500
     agaacactgg aaaactaaag gacaaaggca gagagtagga caaggacgac aaagcaatct    172560
     gaagcctctg acatcaccat ttttaagatc aaggtcttgg aaactccctc attgaccttt    172620
     agatctctag gagaaaaaag tcagatacct gggcctagtg tcagtgtagg aggaattttc    172680
     tataggctag gcattaggaa gaagggaaaa ttcttcctta ttaggaagtt attgttatag    172740
     tgttatagtg ttattggaat agtggatatg gagtgggctt tcatctcgat caatctgcac    172800
     ctgctggtat tttccaatgc tacattcacc tgcaagagcc caatgaagaa agaaggcact    172860
     cccaaatctt ttgcaagttt ttgtattcac tgtgggtcca ctgcttaagt gcatctggag    172920
     cttcagagaa ggggctctgt cctgtgtcat agaacccttg ctttgagtct cacggcagag    172980
     ttcaatctgt ttagtaaagt tgatcaatta tttcaagaga tggtgtcacc agcatatggt    173040
     gtcactgagg gagtattcta cactagcaca cagccatttc acgctgggct agagaagctg    173100
     gggggaaatg ctttgtgagc cccaacagga acctccttgc aaggcaaggg ctgggcggga    173160
     gggggcactc aggagccact cagcacgggt tccagccctg cagctggtgc acaggaggct    173220
     gctgagaagg tttcctctca ggggctgggt cttcctttgg gagaaaaaag ctaaaattca    173280
     ataagttgct ggtgtgccct taaatattct atcacatctg agctgctccc acaattcaag    173340
     agaaggagaa ctatctttat aaatattcta tcacatctga gctgctccca caattcaagg    173400
     cgatgagaac tgtctttata aatattctat cacatctgag ctgctcccac aattcaaggc    173460
     gatgagaact atcttttcaa atattctatc acatctgagc tgctcccaca attcaaggcg    173520
     atgagaacta tctttttaaa tattctatca catctgagct gctccaacaa ttcaaggtga    173580
     tgagaactat cttttcaaat attctatcac atctgagctg ctcccacagt tcaaggaatg    173640
     agaactatct ttttaaatat tctatcacat ctgagctgct cccgcaattc aagacaatga    173700
     gaactatctt tttaaatatt ctatcacatc tgagctgctc caacaattca aggcaatgag    173760
     aactatcttt ttaaatattc tatcacatct gagctgctcc cacaattcaa ggcgatgaga    173820
     actatctttt taaatattct atctcatctg agctgctccc acaattcaag acaaggagaa    173880
     ctatcttttt aaatattcta tcacatctga gctgctccca caattcaaga caaggagaac    173940
     tatcttttta aatattctat cacatctgag ctgttcccac aattcaaggc gatgagaact    174000
     atctttttaa atatactatc acatctgagc tgctcccaca attcaaggaa tgagaactat    174060
     ctttttaaat attctatcac atctgagctg ctcccacaat tcaaggcaat gataactatc    174120
     tttttaaata ttctatcaca tctgagctgc tcccacaatt caagacaagg agaactatct    174180
     ttttaaagtc ggcttctaag attataaata ccttaatagt gagaatatga agaataggga    174240
     tggtcttact aattcaatgc agagagaatt atgggagtca ctatatttcc atgaataata    174300
     atttcagatt tcaggctggg gctggtggct catgcctgta atcccagcac tttgcgaggc    174360
     tgaggtgagc gggtcacgag gtcaggagct ggagaccatc ctggctaaca ccatgaaacc    174420
     ctgtctctat tcaaaataca aaaaattagc caggtggggt ggcatgaacc tgtagtccca    174480
     gctactcggg aggctgaggc aggagattta atcctacaag acctacagcc tcagacaatg    174540
     ctgctgtcag tgatcgtggg aagcagagtt tgcagtgagc tgagattgtg ccactgcact    174600
     ccagcttggg caacaaagcg agattccatc tccaaaaaaa aaattcagat ttcagtgtta    174660
     agtaaagttg cctacattgt gtgagtgaca gggcagtggt ggatccgaga gtgtggatct    174720
     gagagtgagt cagaaatcag catgtaaaga tgaggatcta tgcacatgaa ctgaaagtat    174780
     gtaaacagtt catgaaattc taataaatcc agtaggaaat aaaacccaaa cttatccaaa    174840
     acacaaattc ccttgaaatt attatgggag catgagttca taaagaactc ctaactcctg    174900
     tttcaacttc tgaatcctag tgtccatgac ataagaaaat catctccaat tatgcatcac    174960
     agggcaaatc tgtaaactaa gagtttttct gttgacgatc ctggggaatc aggacaccag    175020
     gaaggtgctg gagaaactgt ctcaggagcg ccccagggat ctcagaggaa cgtgctggcc    175080
     actcacgtgg gacatcagcg tcacttgctc agagtcatca gtgagctgtg ctggtgtctg    175140
     acgggtccag catagggcca aggcacctgc tctgtgtcat ggaccgagat ggtccccaga    175200
     atgatccaag tggtctctgt gctaatctaa tgtagtttca cagtgagagg ccgttctgag    175260
     ggggcttctt cttcagtgaa aggacctctg tccacaaata ttcctaaatg gacaggggca    175320
     tgcatttcct caagcaggat tagggcttgg accatcagca tctcactctt gcaaggctga    175380
     tgtgtcattt gtcttccctt tcttatcatg gatcaggctt tgagctatga aatgccctgt    175440
     ctcatgaata tgtaaatacc tgagatccac tgaggtaaat atggtctgtg ccctgagagc    175500
     ttcacccaac aatcacatcc cgcctctaga gaatcccccg agagcatggc tcctcaccat    175560
     ggacaggacc tagagctaat cttcctggtg gcagcagcta caggtaaggt gctcccaagt    175620
     cccagtgatg agaaggggat tgagtacagt caaggaggct ttcatccact cctgtgtccc    175680
     accctccaat gggtgtctgc tcccaggtgc agctggggca gtctgaggct gaggtaaaga    175740
     agcctggggc ctcagtgaag gtctcctgca aggcttccgg atacaccttc acttgctgct    175800
     ccttgcactg gttgcaacag gcccctggac aagggcttga aaggatgaga tggatcacac    175860
     tttacaatgg taacaccaac tatgcaaaga agttccaggg cagagtcacc attaccaggg    175920
     acatgtccct gaggacagcc tacatagagc tgagcagcct gagatctgag gactcggctg    175980
     tgtattactg ggcaagatac acggtgcgag aacccacatc ctgagagagt caggaacccc    176040
     agggaggagg cagctgtgct ggcatggagg agatgacaaa gattattaga ttgaagactt    176100
     tcttagaaaa tagcattaag tcatttacga aaaggaacaa tataaatgtg tatttgagaa    176160
     attgtaatta tttgagagat ttcttatgca acatttattc tgtaagcaaa ttctagggat    176220
     tggagaatta atcaaattaa taaagctgac atagaaattc ctctgaaggt atctttgtaa    176280
     acatcaattt ctgaatcagt gttgtaaatg ttttggaaca cagacacaag atcacatttt    176340
     aactctactt ttgtctctat aaaaaatgcc aaaaagagtc tcattttgac catgtacctc    176400
     tttgaattcc caccatcaat gaatgattgt tcttgggttt ccacattata ttaccattta    176460
     tcattatgag aattgtgtgt tttaaccatt ataataggtg agtaatggta tctaattttt    176520
     gcttaaatgc acatgcccct aataaaattc atatttaaca attttcatat aatttttggt    176580
     gagatgcctc tcctgatatt tggttcattt tcaatagcac tgttttcttt tgattagttg    176640
     taagtttact tgcatattga ttataaaagt taattaacaa attaaaagaa ttcatttaac    176700
     aaatatgtga cttggaagtt gcaggggttt gtcctgcaca ccctgacaca atgacgaatg    176760
     aataaagtgc actgacacaa agatattctg ctttgccagc tcgactgagc atctgggcca    176820
     cttagtcaca gccaccgcct cgatcagtca gggagactta catttattca gtaaagatta    176880
     attgacaaag gccgtgagta aacaccacta gaaggtaatt gacattgtgg acctcttgag    176940
     tggaaagcaa ttaagcaccc gcggtagatc aaaggttagt cttaggaaca catgagtaaa    177000
     caagctagtt agataagctc ctcacattcc tttgtttcta ctctacttta tgtaactaaa    177060
     ggtaagggga ctaggctgcc ttcagccaga tttattacca aagttatgca aactcacagg    177120
     ccttccaaga gggtttgtgg ctattagaac taaaatgttt cccaccagcc tgactggacc    177180
     accacatctc cccatttttt gttttctaca tcaggtcttt tggattggag cgtgcagata    177240
     tgcgcagcaa catgtctgtc aggtgtggca gtcattgctc ttattctggc tttgcatcct    177300
     agaattagca aataacataa aacaatcatg agtagaatta gcaacattct tttccagtca    177360
     gagtgacccc caggagcggg gtctaaccag gagagatgat ctcacacacc cttccatatg    177420
     actgtttgtt gggggtgtag atctattgcg tgaagagatt ctaaaatttt agtttgaact    177480
     tactttacgt ctgctgttaa attgtcatga aaggttcccc agaggtattg ttttacctca    177540
     tcctaactat gtattgatag attccgtggt agagaagtga tacagatatg ttcatgttcc    177600
     cagtcacagt ttaaatgctg tcagaatgcc actgcatctt gtcgctccct gacatattct    177660
     aaagcagcct caagggctag cagacgtgtg ataatttttt gatctatacc ttgctgtaag    177720
     agaagttcat tagacacatt tttggccaga ttatctaaaa aggcagcagt ttacactaag    177780
     tcaataatac atgcaacagc aacactagct gttgccagga tgactatggc tgagactata    177840
     aaggccataa gtgtaactat gaatcttttg tgtctgacct gggacagggc acgttctaag    177900
     gttgcaaggg cataggaacc ttgccaattg catgccagat tgactggtaa gaatgcctca    177960
     gattttctct ttaataccat ggcgctagta atttttaagt tagatatact gtgattagtg    178020
     atacatgagg caaaccaagc ctgtccctgc atccaggtca caaacgtgga gttttgaggt    178080
     gtaatggaaa tgttagttcc cataaggaaa tcgtatggat gggtagtgca aattaggcac    178140
     tgatcagtgt gattatgaat aaagcttata gcatagttgc aacgggaaat atggtatgtc    178200
     ccatgccagg tgttaaggga ggtgctaaga tgtcccagac accataaagt accttggggc    178260
     ggcatggact ttacttgggg ccttggatat gccatccccc cttggcacaa attatagggg    178320
     aaaagacatg gctatgaaac tgattgatgc tctgatggat gaaggtatta gtaaggctgc    178380
     cctgcaatca gccgctggga ctccagtcta agatgttata attacctaac tggaggctat    178440
     gggctcgtct cccatgacag acctcccagc taaagtcgaa ccgattactg tcccagcttt    178500
     gttccttagc acaggaagga atgtttggga aagaagcagc ggcatggatt gcattgctca    178560
     gtttgaggct atctgcagct aagaatgtta aggcatttcc tttgctatga tgtaaccata    178620
     cttgtttttg ggcaggtaca cagtaagggt tagagtcttt ataacttaca cacactggga    178680
     ggataatagt ggattgacat atagtgttat ctgccacctt agtccattgt gtgccattat    178740
     tgagggaccc cacgtggggt aaatctatcc ctcctagcca agcagtcacg ttactatagg    178800
     ctgggaaggg agtgtcttcc catttggcag ggtgaaagaa aggtgtgtct aagatagagg    178860
     cccaatagag tgtagcaggt acaggttgca gacaaagtga gagcataaaa aggattaata    178920
     ccctacatga gttgcaatgt acaacagaaa tcatagcacc aaacacatta tctggagtaa    178980
     atggtgtctg catttggagc aggattcgtt cagcctcctg agttgtcctt ttcagtaccc    179040
     cccaggtaat gtctggggat tgtgttgttc acagaagctg catcgtccgg ggctgtaggt    179100
     cttgtagggt taactccttc atttctggta ctgggttggg tcctagcaat gtcctggtat    179160
     ggtttgatgc gtcgtgctgg aacccaaaga cgacccgagg gggtgtggat acaagcgtac    179220
     cctcttcccc acattaataa ttcacttgga ccacaccatt cattactgtt tacatctttc    179280
     cataaaactg caggtttcat gtattgggaa attttagcaa agtgcttttc tacagcggat    179340
     tcaaatttgt cttctaaatt aaaaaaataa ggtttgtgcc aaaagtgttg cagggtctgt    179400
     acccatactc ccctgttttt gttttttgtg catattttaa gggtggagtt ggcaagttct    179460
     actattgctt gtccttgggg tttatatggg atacctgagg aatgtcggat attccacgtg    179520
     tgacaaaatt gttgaaattg tgagctggcc taacctggac aattatcagt cttaattttt    179580
     gcgggttgtt ccataaatgc aaaagataaa agaagatgtt taatgacata tcgagtaaac    179640
     tctccaggcg gagtatgtgc actacttaaa tgagtgttga tatcaaagga tacatgtacc    179700
     tatctaagtt ttccaaattc agggatgtgt gtaacatctg tttgccacaa cagattaggt    179760
     tctagggtta atacctgttg aaggaggaga cgtgcctgtg agctggcgat ctcagagact    179820
     gtttagccag tctctgtata agttgagctg gcgatctcag agactgttta gccagtctct    179880
     atataagttg gaattgttta gataagtttt tccaattttg gtggaaaaat tggtgcaatt    179940
     gggtggcttg gtcaagcagc gatatcataa cttgaaggtt ggcttgatta ttgtcataag    180000
     ctaatggacc aggcagtgag ctgtgggctg gaatgtgtgt aataaaatag gatgtgtact    180060
     ttgatttagc aattgctgaa atcagagaaa aagagtccac agggctggct ccagagttga    180120
     cttaattagg gctgtaaaag ttctctagac ccaatattaa ggactcaact ttagctttct    180180
     gagtgctagt aaacccagat tgagtgaatg aattgtgtgg tgtccaccag actgctgctt    180240
     ttccatattt accagatcta tcagtaaaca gtgctaaagc attaaatatg ggggagtaaa    180300
     ctatttttgt aggtaacaca ttaactaggc cttttgggaa tgatgaaaat gggtaaattc    180360
     taagggctgt tagtagaata ttacccataa aatgaataat cttgtagcca ggaaactttt    180420
     tcctactagg gagcaaagct tgcttttaat tgctccctaa ctaacttagg ggctttttgt    180480
     aatgtctctc cctttagagg ttactgtttt acttaaactg gatttggagt catgttagga    180540
     ataagagaga gataacagtg gccattatta gaaagaggtt tctcaaattc ttaaatttta    180600
     cttaactctt atcatagaat tataatctgg gatgtcgctt gtatacaaag aacataacaa    180660
     attttgcttt gggccgcctg cggagccaga gagcggagca ggtgggtccc agggcggctg    180720
     cggctttgtg cttgcttaga agccactttt cgccaccgcg tttcctctgt gccagctccc    180780
     tccccacact gctcagcagc tgctgccggc agaggggagc ttcctgcact tgctcctgcc    180840
     cctaatgcct tttcttaacc cttcgtgtac ctgattgcct tgctgatctt gcattactgg    180900
     gcagtctaag agctcccctt ttaatgctgc ttgtcgaaga cagggacata tagctgtagt    180960
     gtatttcctc tattttttct atctattgaa agagggggct caggcaaaac ctccgtttcc    181020
     tctttgttat tttgggaaga gcaggtggta aggcaagtga cgtttcttcc tccttcccct    181080
     ttttgggttc ttctttgtat aatgggacta aagccatccc tactaaagcc catagtgtta    181140
     aagatgatgc tgggacccgt tgcgcttgtg tgtaatgctg ttcaagattt ctccctactt    181200
     gttcccagag ctttacttct agctttcctt cttctgggac cacagattat gtgagacaac    181260
     agttttcatt aggtgcctta attgagcctg cgaaactgat gctcccctag ccttaagcaa    181320
     ctgttacaat actttcatat gctgtttgtg ttgagctgat aactgctgtc ccatgatgaa    181380
     atctcagcct gaacaatctc ccctgaactt ggagatccca agtgggcacc aatgacttac    181440
     tgttttactg acttgaccac gcagtcttcc tcaaccttcg ttttcatggg gtctgtcacg    181500
     ctccctttgc agcgatcctc acatggggca ccggctgtgg gggtctgtcc tgcaggtcct    181560
     gacacaacaa cgggtgaata aagtacactg acacacagat attctgcttt gccagcttga    181620
     ctgaccatcc aggtagatca aaggtgagtc ttaggaccac atgagtgaac aagctagtta    181680
     gataaactcc ccacattctt ttgtttctac tcaaatttac taaactaaag gtaaggggac    181740
     taggctgcct tcagccagat ttattactga agttatgcaa tctctcaagc cttccacgag    181800
     gacttgtggc tattataact aaaatttttc ccacgagcct gactgaaccc cacaggaagt    181860
     attttctcca agtctgtggt tgccttttac tcccttatca gtaggtatcg cagaaaaatg    181920
     tgtgtgtttg tgtgtgtgtg tgtttgtaca aatttagatt gaaaacatat ataattttat    181980
     tcattcatag atcatgcctt tggcattata tctgaagttt cattataaaa taaactaata    182040
     gtcattattt tttccatatc tctaatctca ggccacaatc aactcatgag tgtttaaact    182100
     tcacctgctt gattggagga ccatcaatct aatgtatttg gaatacttct gtaaggagat    182160
     gtgttcttct tcctattatt ttttaattaa tcatctatta atatcagtat tggttgatgg    182220
     atgtccattt tatactttga aaaagatcca tgctacatca ttcatttaat tgttcaaagc    182280
     accacagctt tattaggtgc tgggagctca ttttgtttga atcctgcatc cttacagcac    182340
     acctcatatt tttgtttttg aacacttgcg tatttcctgg tattaccata aattctaagc    182400
     ttgttttctt tattaccttt ttttacatag aatcaaccac ttttataaag attgcatgtt    182460
     tctgatgtta aagaatagta ttaaaataaa acattgtgat actggctctg tgtgttgtta    182520
     atgtggtatg agtacttcta gaacctctca aacaatggtc ccagtaaacg tgcatgttta    182580
     tgtgaaccca agtttatgga ctcattgaaa ctatttatgt atctaatctt ctgtaaccat    182640
     atcacattaa aaatgagaac acactggtct ctccacccaa ctatgttagc acatgaacct    182700
     ttctagtctt ctttctttgc gtttccataa ccacccactg caaagtgaga aaccccattc    182760
     caccatatgt aattttatta cttagctgca cagtttcagg acacatgcat agcagtatca    182820
     aaagtgtaaa gctgtgccct tgatggaacc atgtttatct actagaatag agtgcttatg    182880
     tgcagatttt ttacacatta aacttataga atttcttcgt tttctgagtt gcttaggtca    182940
     gcaacttcat tttccacatt cttcaatgaa gtcatttcaa ttacattgta taatttcatt    183000
     tatttgaaat tccataaatg cttagactat agtcaagtaa acagacagag gatgttccag    183060
     gaatttagag agtgggtata aaataagcaa aaaaaaaaat gtggtgttta ggaaaaataa    183120
     aactattttt agtgatatgc aatggttgag atatgatata actaatttgt ctaagctaat    183180
     aattttgtga tagaaaatat aaacttaaac atattcaatt aaaaaaattc agcagttcat    183240
     taaccccagg attaaatgca gactgtataa aattatccaa taacttattt ggtgagagtg    183300
     gggatgttat gagatacatg caacaaagaa tgaagtaatt ttccccattt gcatatacaa    183360
     tgtttccatt cactgaagac cttttatttt aaaaaaatca attttctacc ttacccttgt    183420
     ttttaattcc cgacaagcaa ataacccaaa ggattctttt ctttcattgg ttgagaaaga    183480
     ttttccccta acttcagctt agttcaggca tacgctgacc tgaatgggca tttaccctca    183540
     gatgggtaca cacctgtcaa tatgtggact cttctgtcag acagacacag cttcactcat    183600
     gtggattctt ccctcaaaca caaatgtccc cacatggact atttcctcag actaccacat    183660
     atgtccttac atttacactt tcctcagaaa acagacattt cctcatgtgg actcttgtct    183720
     cagataagca aacatgtctc agtgtgaatg aagtcttcac tcagataagt acacatattt    183780
     caacactgac tgtttcctga cacaagcaca tatgtccaat gttaaactgt cttgcgacaa    183840
     aatgatctca agataatgat aattataaac tccaaccctg aaaatctgta gatctgcatt    183900
     ttgtctattg taacataact tcatctcatt gtcagaaaca gtcgtttgca gctataaatg    183960
     cactgattac agtcagattt ccattttctc tggaaatgta tttcttatgt tcttactgga    184020
     ctaatttgtt gataatgttt gctcacatga agatacctga acagtgtcca cattagagaa    184080
     taaaaaagag caatgggcag attaaccctg tgcatccaga cgcaggaacc atttgactct    184140
     gccttccctg aaatggagac acagaggatg gatgagcaac gctgagtggt gcacctacga    184200
     ccacaaagag aaagacctgg aaatatgtcc catcccctcc tcatgaaagg cagctcatcc    184260
     cctgttcctt caggccctgg tgaggagcca ccccatatct gtgtctttct tcagtgttca    184320
     cactatggag tctgcactga tctgggtttc ccttctcatc accctccata ttagtgtccc    184380
     ttgtaaatca ggtccagctg tggctgctca tagggttgtt ctcagtctat ttcctctgtg    184440
     ttcatagaag tcctgtatga agttcagtgg tggagtcaga ggggtaaacg tagtacagcc    184500
     cagtggttca ctgagacttt cttgcaaagc gtctggattc accttttctg gcaacagcct    184560
     gaggttggtc cagcaggctt cacaacaggg attgtggtgg ctggcaacag tgagtcaaca    184620
     agtgggagtg ctcaggttta ctcttcatga gtacaaataa attaactggt ccagcgacac    184680
     cctttcacgt gcactctacc ttacaatgac taacctgaaa gccaaggaca aggttgtgta    184740
     atactgtgag cttcacagga gagagattat ctgcacaagc ccagacacaa aaatctgcag    184800
     ggagacagga gggaactgca tggtagatgc tgctcagaag caccaggggg cactcaacac    184860
     aagggggcgc tcaggacacc aggcggcact caggatacag ctgggggcgc tcaggacacc    184920
     agggggccac tcaggataca gcggggggtg ctcggatcca ccagggggct cttagggcac    184980
     cagggggcat tcaggaccac tagggagccc tcagggccac caggaggacc tcaggacacg    185040
     tcagcgtgct ggagccaccg gggggggggg gggggggggg cgctgaggaa accaagagac    185100
     gctctgaacc gcgaggggaa ggaagctcag gaccacgagg ggacgctcag gacaccaggg    185160
     ggcgctcaga acaccagtgg gcactcagaa caggaagaat ctcttagaaa gcagctccac    185220
     atcaggatcc tgggaggctg tggagaaatg ggaacactca taaactgttg agcatttaaa    185280
     ttagtacaac cactatagag aacagcttag agtttcctca aaacctgacg atagagttat    185340
     catataatct ggcaatcctg ctgctggtat atacccctaa aaaggaaatg tttttgcact    185400
     accatgtctg taatagcact gttaaagcac tgttcacaac agtcaaaatt tgaaagcaac    185460
     ctatgtgccg attaacacat gaatggataa ataaaatatg gtacatatag gatatgggtt    185520
     aatatcatgc cataaaaaaa tgagattttg tcatttgcag caacatggat gaaactggag    185580
     ttcactatat taattgaaat gagcccagaa cagaaagaca aaattcacat gttcccattt    185640
     atttgtgaaa gctaaaaatt aagacaattg aactcgtgga gatagagagg gaaaagatgg    185700
     tcaccagaag ctgggaaggt ctatggtaga tggggattat tggggatggt caatgggtag    185760
     aaaaagtgat tagaaataat aaataacaca taatatttta taacacaaca gaataaatat    185820
     agtcctcaat aacttaatta tagattttaa aataaaagtt tatgaatgga atgttggtaa    185880
     ctctattaat gcttgagagg atgtataaaa tattcttcat gatgtgatta ttatgcagtt    185940
     catgctgatt cagagtcctg taccccataa atatatacgt gtactattta cccacaaaga    186000
     ttcaaaatta aagtgtttat gtgtctaata ttctgtaact atattacatt aaacctaaga    186060
     acacactggc ttttacacac tactgtcatc cacatagaca ttcatagcct tctttctctg    186120
     cttgtccata atcccacact ccaaaatgag aaaacctctc ctaagatatg acattaattt    186180
     acttagtgca caatttcaaa atacacaaat cgtctttagg attgttaaac tgacccttgt    186240
     tgaaaatatg tttatcaact agaatacagt gcttatgtgg agcttattta tgttttacaa    186300
     tcgcaacatc aaattctttc caagtctctt gtgtccaata tttatgcccc accttcttca    186360
     gtgtaattag ttcaaacctt ctgattgttg ttagatattt ttatacagct gtcttttttt    186420
     gtgtttcatg acctgctaaa tactttcgaa gttgtatgca tttaggttaa cactttgtct    186480
     caaaaaatat gagttttcag aaattcttta tgccatgtgt ccatcattga atatcataaa    186540
     aatagtttca ttggccgggc gtgttggctc acgcctgtaa tcccagcact ttgggaggcc    186600
     gaggtgggcg gatcacgagg tcaggagatc gagaccatcc tggctaacac aatgaaaccc    186660
     catctctact aaaaatacaa aaattagccg ggagtggtgg tgggcacctg tagtcccaac    186720
     tactcgggag gctgaggcag gagaatggcg cgaacccggg aggcagagct tgctatgagc    186780
     agagatcgcg ccactgcact ccagcctggg tgacagagca agactccctc tcaaaaaaaa    186840
     aaaatagttt cattgggatt aaaaagtgtg tgtttcacct aatccacaca ccttctcccc    186900
     accttcctgg gaaacctcaa atgtttactg gatctatatt tttatctttg gcaaaacagc    186960
     ctggctgttc tcttagcctc ttttgcaaag catcaagatt caccttcact gactacagca    187020
     taaattgagc ccagatggct gggggacaga ggctggagtg ggtggtaaca gtgattgatt    187080
     caagtggaag ttctcagtga tattctgcat cagcataatg aagattcaca attcccaggg    187140
     acaccaatta ccagcacagt ctcccttaaa ataatctact tggaagctga gggggctctc    187200
     acaggggtag gcagtgtatt actgtgagag acacagcgag ggacatttct gtgagtccag    187260
     acagaaacct ccctgcaggg agacaagaga ggactttgtg ataaatggtg cttaggacac    187320
     cagggggcac tcaggacagc agagggtgct cagcagagca gggtgcgctc aggacatggt    187380
     gtgtgttgta gggggcattg ctcaggatca ccagggggct ctcagaacca ccaagggtca    187440
     ctcaggaaac cagggggcgc tctggacacc agggggagct cagaaccacc aggtggtact    187500
     caggacacca gggggcgctc atgaccagca gggcatgctt gggactctat gaggcatgca    187560
     ggagaagcag gccgtgctca gggcaacagg gggctcacag aaccacctgg gggtgctcag    187620
     aaccactagg ggtcactcag gacagcagca ggcattcggg acaccatggg gccctcagga    187680
     cctgcagggg gcgctcagaa caccagggga aggtcagaaa gacctggggg cacttggaac    187740
     caccaggggg cactcaggac agcagggggc gctcaggacc actagggggc gctcaggacc    187800
     agcagggggt gctcaggaca ccatggggca tgcaggagca gcaggctgtt ctcagggtaa    187860
     cagggggctc tcagaacaac ctgggggtgc tcagaaccac cagggggcac ccaggacatc    187920
     atgcggccct cagaacctgc agggggcgct caggacacca ggggaagctc agaatgacct    187980
     gggggcactc agaaccacca ggtggcactc aggacagcag ggggcgctca gtaccacggg    188040
     gtgcactcag gacagcaggg ggcgctcaag acacagtggg atgctcagga ccagcaagag    188100
     gcactcagga ccaccagggg gcactcgggg gagcaggagg cactcaggac accagaagag    188160
     gatcagaacc actcgggggc actcagaacc accagggggc gctcaggaca ctgggggcat    188220
     tcagaaccac cagcaggttc tcaggacacc cagggacact caggacagca ggggcgctca    188280
     ggacaccagg gagggtgctc aggacaccag ggggctctca gaaacacctg ggggtgacca    188340
     ggactagcag gggcatgcag gacaccaggg aaggggctca ggacaccagg gggtgctcag    188400
     gacagcaggg ggcacagaat cacgagggtg cattccagac caccaagaaa tactcaggac    188460
     caggggtaac agaaacacct aaagacacac aagaccacca ggaggcactc acgactgctc    188520
     agagtgctga ggacaccaga gcttcaggac gaacaggggg tgctcaggcc accatggggg    188580
     gctcaggaac caccagggta tcactcagac aacagggttc tcttaggagg cagctccaca    188640
     tcaggtgcct ggggagggtc agatttgctt ttagaccttg ctgatccctg atctggtcaa    188700
     gcgaaaacct tcccaggatc tctcaccatt tcttccttgt aacccatggt ttctttcacg    188760
     tacaaatcat taacttagaa caggaattca attcaacttt taaccctcca tatttttaga    188820
     ataacactag cagtaattga atgttttgat aataagaaat tgcgtatgcc taattcaagc    188880
     tctggttcca tgcatgggtt tttgttttct ttgctgtgtc agtcacacta tagtcaatgc    188940
     ttttctacca ataactcagc ataggcatag tgttaagttt ctttttcttt catctgcctt    189000
     ttgtggagat gaaacaccac tttcagggct cagttccccc accttggttg ggttctggtg    189060
     ttctgctcct cacactattt ctccaccttc ccttcttctg tccaagcctt ttgtcttcct    189120
     cagtctccta gcaaggaagc aacaagtccc tttattttgc ctcttccatg tctggtgaat    189180
     ctgttcactt ctcttcatga tcattgaagt caaccaagtt taggaggata acagttctcc    189240
     ttagaatatg ctcatctacc tgcagactct ctgccttcct cacccctttc tagggtcctg    189300
     cagacgtcac ctccaccaca tccactcctt tacctaagta ccacagaata gtctctgcag    189360
     ctcctgctgc tctctgtgtg ctcagcacta aggctcacta gtgctttgat gatgaagttc    189420
     aaatccctaa tgtgttagca attctcagac aaccctgcca ttgtgagtga attttgggga    189480
     tcctgggctg ttttcagttc catattactg gatgttctct agcatccagg agtactggca    189540
     aattaacatc tagaatttgt attgcaaatt tatacaaaat caattgtaga aaaaaatcct    189600
     aatttctaaa taagaacatt tatcctgact tttataaaaa tacaatgtga atttaaccaa    189660
     taatataata caattttaaa agtgatgttt atcttatatg ttattaagtt tattaataat    189720
     tggctggtat tttaaaagta tactgatagc aaaactgaca aataaaccaa cacagattct    189780
     atacataaat accattgtcc atttaattaa tgtgtttggc cagaatggca tttacattca    189840
     gttctaccga ggcatgtctt taaatgtatt tgtttattgg cattaggata gagaaacact    189900
     cacacagaaa agcattattt gggactacta cattatacta taaaagagag taaattcaat    189960
     tcaattaaat aaataaaata aattgaataa attcatgttt tggtttggta tttggatagg    190020
     tgttcatttt tctattttca catgtatatt tcagtactgg gcatatgtgt gtatgtgtat    190080
     gcatgattat gaacttctaa aatacattac tcccctatat agttatataa attgaagttt    190140
     tgcatgttaa gagacagtag aaaccacaaa aggggaaaag ttatagaaag catttggaat    190200
     acctagggcc tacagaggag tcatattaaa atgaaaaata atagctgaat attttggaaa    190260
     tgacacatgc agtaagaaat gtctatatga aattaacagg atattggcac aaaatttaga    190320
     aaaaaataac atgattatgc ccactattgg tctatgatat gcctataata ataacacatt    190380
     ttatttatca tagaaaataa aataattata caaatagcac tctcattgct tgattttagt    190440
     attaggtact tcacttgttt tttgatagaa atgaacattt atcatttttt gatgataatt    190500
     tctatattat cttctaaaat taatgtccat aattatgatt gacatgttat aatttccaga    190560
     aacctatttt atcaattgtg acttgtattt ttatgatagt atttttacat tcatatacat    190620
     ttgattcaga ataattgtag attaacaaaa taatcataaa gttaatacag tgtgttccca    190680
     tctctaagta tctcctaaga tgaacatcct ccacaatcat aaaacatggt gttaacacat    190740
     ttacgttgat aactttaaat atattcaata ccaggcctta tttgagattt accatttttc    190800
     caataatttc ttttgtaaac caaaaataaa attctaagct tcctcatcca actaaatgaa    190860
     ctcaactatc agccaaagat attttaaagt aaactttaaa acttgttcca gcaataatgg    190920
     gaaaatgaga ggtcagatat gcctcattat gtcctcctgt ctttggaatt aaggcacctg    190980
     tggccagcat taacactaaa acaaagaccc ttagactgac aaaactatat tttttctata    191040
     aacactcttc atagtgaaaa gataccaaat tacaatatga ttctagtata gcatcacatg    191100
     acaaacagca acgcctaaaa atctattctc tatgaagtct gctacctgga ggtttcatct    191160
     acataataac aattatgagc tccacaatct cgtatcttaa tccagatact catttgtaat    191220
     gagtgtgtaa catttcaacc aattgccaat cagaaaatgt ttgaattcac gtatgtcctg    191280
     gaagcacttg agtaagctta ttctgccttt ctggactgtg taaatggtat agctcacatg    191340
     tgttgattca tatctgtcta taccttctgt accactaaaa tgtataaaat caagctgtaa    191400
     ccaaaccatt ttggatacgt gtactcagga actcctggag ttgtgtcatg ggccttggcc    191460
     actcatatat atctcagaat aaactgtctt aaataattca cggtttgtct cttttagtgg    191520
     atactgtctt tttctttttt ctttcttttt ttttttttgt tttttgagac aggatctctc    191580
     cttgctgttg tttttgtata tttgtttgtt cccagattcc aacaaggtca tcactttgaa    191640
     ttgagttgtc atgttccttt attctcttct ggcaaatcac agtttgtgtt tctgaagttt    191700
     tctagtttaa tattttatag ataaaaattt atcaagtaga ttgatagtcc tatttaggac    191760
     acttatatct ttacttattt gaacactgct tgtcaatcac aatgagtgtt gatttctccg    191820
     tcttctatac atgttattat attttctgtt ccaaatccat tcattaagta tttttaaatg    191880
     tattttggaa agttgatcct attactttat gtaatatttc tcttgagtcc caaaggtctt    191940
     ctttttatga ttattttgtc ttaaattaac atagctattt aagttttctt ttggtttata    192000
     ttttcatgat atatattttt ccatctttat tttttctatt tctgaatatg taaagaaatt    192060
     ttcttggaga taaataatag ttgggtgttt ttttttagtc aactatgaag aattattttt    192120
     taatgatgtt actcttttgt ccaattattc ttgttttaat ttgtattttt acaataaagt    192180
     ttattgtttc tattaactta ttatttaatc attctgtgac taacatttta tgggttacca    192240
     taaggataac aataaaaagt ttctataatt gaattctaat tcaaaaattt aattcccatt    192300
     catatatact acagagatat tataactcta tatatctaat tcctctttcc tatcccatgt    192360
     tttttatttt ctgtttgtat ctatacatgc tataaaatgt aatattggca tttttataat    192420
     tgctttccac acttacataa tgaacaaaat acaaatgcaa aaactgtatt tcaacctcat    192480
     ttttccattg ttgaccatct tctttgtgca tattttcagg tgttggatct atgtcacatg    192540
     gctattcatc tagagactct gctaaaatgt ctacggaaag attcatgtgc tggaaatagg    192600
     ttttctcagg ttttattggt ccgacagaat atttatttgt ctttgattta ttgtctccag    192660
     agacaatgtc aagaatatgc tatatctgca aatgggcgat ctgtaaacca agaacacatc    192720
     agtatatcac tgtgcaagag gagcacatga gggaaagcca gtgtgtgccc agacataaac    192780
     ctccagggac acatgggcga attagctgca gggggagctt gggacccact gatgagagtc    192840
     gaccccagag ggaggtgcac agaaaggtta agaactgctt tcctgccagt atatttttaa    192900
     aaaaacaaaa cttccatctg acagtttctc cagagaactt ccctaatttt agcattctgt    192960
     gcctaccaat gccatcacta aataggtttt taaaaaatat tgtaatatga ggacatattc    193020
     tcatatggac aaaacgcaaa ttgacattta cagaaatgaa aagtcctcaa ccgtggtcgc    193080
     tagaattgga gacctgtagc agctcagtgg gacctgttga gtcttctcca atcacactca    193140
     gaacagagac cttagtgcat ctccctgact agaataccct ttaggtatcg agataacagc    193200
     ctaaagatgg taaatcatga gagtctcaga tgtccagatg acagacatgg gtctatggga    193260
     atagtactgt aactgacagt aactaatttt ccataccgcc agatgtccta ttcaggagca    193320
     gctacagcag tcatgcctag gtgtgaagat cacacactga cctcacccat gctgtctctg    193380
     gccacttcat cacaaccaat gcttaatatt ggacgtggat ctgccagtcc ccggggaatg    193440
     ggttgaatgg ataaaatgca tggctagtgc tgctggaaac ccattcctac tgtggcaaat    193500
     ggcagcatct cttttaaaag gctaaataat attctattct gtatacatac cacattgcca    193560
     ttatcctttt tgtttgtttg tttgtttgtt tgtttgagat ggagattcac tcactctgtc    193620
     acccaggctg gagtgcagtg gcgtgatttc aggtcactgt aatccctgcc ttctgggctc    193680
     aaacaattct cctgcttcag cctccggagt atctgggatt acaggcatgc accaccactc    193740
     ccggttaatt tttgtatttt ttgtagagat ggtgtttcaa catgtttgtc aggctggtct    193800
     caaactgtga cctcaaaaaa tcaacccacc tcagtctttg aaagtgctca gattgcaggc    193860
     gtgagccata gcatccagcc acttttctgc attttttttt cattgacact tagattattt    193920
     caatatcttg gctcttgtga atagcgctgc agtaaacata ggagtgcaga tatctccaca    193980
     aggtggtgat tttttctatc ttgggtatat tcccagaagg gaggttgctg gtcatacagt    194040
     agttctagtt ctaattgatt taagatcatc ttactgcttt ccatagtggt ggagattcag    194100
     attagtgtag ctattatgaa aaacagtttc aatgttgggg agtgactgaa aaacaaataa    194160
     tgcttgatta tggttctcca tgagagtctc taataaacct aatggaagtt cagaaagttt    194220
     cctcaccttg ttaagaaaac atttgttttg ctcataattc tctttcagcc cagagttggc    194280
     agtatgtaga atggaggctg ttggctcgtt taaggattct gaagcaaaga acagtaatga    194340
     gagatgacaa tagagaaaaa gagagagaag gagatgctga atcatcatgg actcattcca    194400
     cctgcaagcc tttcactgta aagctctatt gctaaggatc ctgaggattc tatactccca    194460
     tgaatgcata atcaggatcc ctcaattcaa attttattgg cagagacaca ctgggacccc    194520
     tctaggatat gtgccctaca ttactggctc aacatgccgc actgagaatc ctgcattaac    194580
     tggctttatt tgtagtggtg agtaaagttc acagttttca gtcatccatg aaggggagat    194640
     tttttttttg gttcatataa tttttgcaga aattcaggta ttagtgaaaa ccaataagat    194700
     tgaggatttc tggatatttt agcaacacta gggatgatgg ccattttgta gaaaaattta    194760
     catttttcaa actgacctac atgttaggct gacataaaaa aatccaacta cacagcaata    194820
     ttatggcaac acttcaatac acttggtggt gaaatacctc agaaatgaaa tgttacacag    194880
     attaattgag ttaatgaaac tgttctgaac tatgataaac atttgtttgc acccagattt    194940
     gcttgaattc aatttgatta ttatattcag ctgctgccct aacttatttg aatgagtaag    195000
     ttactattta aatgaccctg tctgtttgat ttgttatata tacatctgaa ttaaagaact    195060
     taaaggacag gtttgtgcta ttattagaaa gagatattct ggggggccct tgtttgctcc    195120
     aggaacctcc acatttttaa cttagaatta atataaattc cttgtgaatt gggtcagtct    195180
     tcaagagacc taatctctaa aacatatacc cagaaccttc tccagacaga ccatccagag    195240
     gaagaatctt tccagtaatt tatctccatg gtggaaggtc cttcattgtc atgcagaacc    195300
     ctctgcccag gcttccttta ttatgcaaat aattaatatt agccagtagg gtaagattaa    195360
     acaaaaatag accggagatg ctgcagacag ggaaggaagt aatagaccaa aatcagcttt    195420
     attaatggag actgctttta aaggtcacag ccaagaaatg gaagaggata aaatcaatga    195480
     gatgaaattg taagaaactt tgtccagttc aatcaagcca tcagcaacca gatagcttca    195540
     gtggacatta tgtgtattac aacatggcta aagattctag attacacaag gattttctct    195600
     atagtttgca tattgacaaa gtggaagatg gcatgggaga tctggtggca aacacttaag    195660
     gtgcaataat agctattttt cctatatcat gacaatacat ggcatgcctc tatattcatt    195720
     tcattccaaa gaagtccttc agaaggtcaa gtaaatggag aatcttacat gcatggccca    195780
     atgcagtctc caagaagtga ttttgaatgt acagctacac cagttactgc aatgctgtgt    195840
     ctgaccttcc cagcatctaa gatattcatg agtatcaagg gggagatggg tctaggtgaa    195900
     agaaaaggaa gtagtcattc ttgagaagaa tggtatggcc aagataagcc tgaactctct    195960
     atcttcttcc atttgctgca gcttcttatt gcccgattga ggtcactcgc ccatggtggt    196020
     aatgacatca gcaatgcagt acattctctg gtggctttgt atctggttta taatgtggga    196080
     gatgtttcct caagagtggt gacgccagta tggcttctgc tctctggtgg tgatggcatc    196140
     tgcattagcc taggggtttg gagaaagaga gttattcaga ccatggggaa gaatctgaca    196200
     ctgtcacacc gcctggtgcc ttcagtactg gactggctcc tgccctcatt ggtgattgca    196260
     tcaaatattg gcctcctcat cagtacaaca cattgtaaca gtcttctttg tacctggctt    196320
     taatccaaga aagttgttga cttatggctc tttcattata ttgtatggcc tggtttgtca    196380
     caatctttat ttctggaatt atcagtgctg ccatcttggc tgacttcaag tatgtcatcc    196440
     tcacagtttg aagctgcttg acattaacat ttttgtcaat gtttgggact accttacata    196500
     ttccggctct ctgaaagaat gattagcgtt agggaaggct gacactaaat ttgggagcca    196560
     gagaaggaaa gtgttactgt gctacagttg ttttgtgata aatatggtta ggttaaaaat    196620
     gagctatgta atgtagccag gtttccactg agttccactt tgcttcttgg ctgtctattc    196680
     ctgtgcaagt ttctatatat tttgtatcag gcttcaattc cattatgttt taaatgttgt    196740
     ctctaaagat aaacaaagat tttttaaaac taccaaacat gcagccattt gacagagtgt    196800
     gcgctgcatt tttagtctca ccatcttctg ccctaacaag caccgaatct aacaacacaa    196860
     atatcatgga agctttcctt ggagtagctc ctgtaattag aatcattggt attctgccct    196920
     cctgtcagtg gtggaacgtt ctattggcac atgtggaaac ctcttagagg gatgagtttc    196980
     tttgaacaca ataaaatttt aagttaggaa taacttattt gaaagcaatt cattgaaggt    197040
     tattgctaag aagaagttag aagcagtctt ttggcagtca tttcttcaag atgtatggca    197100
     gcgtaggatg gatatatgaa gtggaatagg aacttgggct agttaaatgg aatagcctcc    197160
     aatgttaatc tgtttacctt ttaactgaat gaaaaagcct atagttgtaa gaaaagaaaa    197220
     aacaatacag tgctcccctg tcccacacat taccttctca tcagtataat taatctacat    197280
     tgaagatgag tgtggtaatg cagagactct atttagggag gagaacatag ggaaatgcaa    197340
     agacaatggg agaaaaaagt caaggacagt agagcaattc gaagcctctg ataccaatag    197400
     cttgaggacc aaggtcatga ctcaactttt ggtggacaca gagtcacttc tcttagggaa    197460
     actgcagtat tctaaggtga caatatgcaa acatgatgaa tgtacacttg ctacatctcc    197520
     aattgtgtac tgttttcctt acttctgttt gaagaaggga gtcgtatact caggtctagt    197580
     atcagtgtag ggggtgctcc ctatttgcaa gatactagaa aaaaatggta agttagaggc    197640
     ccattgttat aaaaatctac tagctccgtg tcagtgtgtg tgtgtgtgtc tgtgtgaatg    197700
     tgtgtgttat tgaaacagaa gacatggagt gggcattgat ccacaagcat agatacctac    197760
     aggcactctt agatgtaata tttaactgca ggagtctaaa taaaataaag gattcgccaa    197820
     aagtcttgaa aattttggtg ctgactatgt gtccactgat tcaatgcatc ctgagttcca    197880
     gggaagggaa tccccaattc tttcatagag ttgactgggt ctcctaaggt caatgttttc    197940
     aaacgatggt gtcagtaaca tatggtgtca ctgaaagagc attttaaact agggcatggc    198000
     cacttcctat agccctagag acactgagag gtaaatgccc tgtgagccca gatggaaacc    198060
     tccatgcagg gcagaggcag tgctgcaggg ggcgcccagg acccacccag aacaggctcc    198120
     agccccagag ctggtgcaca ggaggctgca gagggagtct ctcacaagga ctgagtgtta    198180
     ctttactgaa aaccaaaaaa ttataacatg ctaaataaga attttatgag gactatttat    198240
     gtatatttaa ataccctaat gtatttagaa ataaataaat caataattca gtgagaacca    198300
     catttaaaag gcaaaattat tactgctttg agatatttta ctagtaaaaa gataaagaac    198360
     aagaatggtt ttattaaata aaaatgtaca catttcagag acacactggt cccgagagta    198420
     agactgcgga gagcaaatcc tggaaagggc ggaggctgtc catgtgccat gggaacttgg    198480
     gctcacgctg agaaccatgt ccggtgtgag tcagcttctt agcggagaaa ttctcccttc    198540
     acaaatttcc gggcatataa agggaaacat gtcattaaat aagaatgaag atttgtacct    198600
     cagcatccca cagttgtgtt gtccatgtga gatctatttt ctctttctcg tgctggatca    198660
     ggtgtaatgc tatgaagtag tagtcctcat gaatacgcaa atcacctgag gtgaacacta    198720
     cagataactc tgtgccctga gagcatcacc caataaccac atccctcctc tagagaagcc    198780
     cctgagagca cagctcctca ccatggagtg gacttggagg atcctaattt tggtggtcgt    198840
     agctgcaggt aggataattc tcagtcccca ggactaaggt gactgggtcc agtcaaaggg    198900
     ggttttatcc actcccgtgt cctctccaca ggtgcccagt cccaggtgca gctggtgcag    198960
     tctggggatg agatgaagaa ggctggggca tcagtgaaag tctcctgcaa gacttgtgga    199020
     tacacctacc ttcaccagtt actctatgca ctaggtgcgc caggcccatg cacaagggct    199080
     tgagtggatg ggaaggatgt gccctagtga tggcagcata agctacgcag agaagttcca    199140
     gggcagagtc accatgacca gggacacatc cacgagcaca gcctacatgg agctgagcag    199200
     cctgagatct gaagacacgg ccatgtatta ctgtgggaga gacacagtgt gaaaactcat    199260
     atcctgagag tgtcagtaac cctgagggag gaagcagctg tcccagtttt caggatatga    199320
     caggatttat ggggtttaat gttgtttaga aaataggtta tataattgag gaaaagaaga    199380
     aatagaaaga tatatgcatt ctaattatat aagaaatatt cttttcaaat ctcaccctat    199440
     aagcaaaatt aacagagtgg gaaaagcagc aatcaatcaa gctgaaacaa acattcccat    199500
     ggaggatttg tgggggcaga cgttttaaaa ttgaatgggt aaatcattta gggcaagatt    199560
     gcttgatcca atactaagac taaatgtaat ttctgagaaa ctgcctatct ttttatatta    199620
     aatttattat aaagcagttt tagggttaca gcagatgtaa aaagtagaaa cagagagctc    199680
     ccatgtaccc ctgcccaaca catacacagc cctccccatg atcagctccc tgaaacagag    199740
     tcctaactgc gttatgtggg atgaacctac atggacacac tagttctttc ctttcctggt    199800
     ggtcccccgg tataacaaac ataaattatt ttgaagcgcc acgggttctt caagtgggtt    199860
     actgggaatg atgccagtta gagggaaagt gggtggggcc attccttttt gcactctttc    199920
     ttcaggaatc cataaaatgc acattgactt agagctcatc tgactcctgg ttttctatgc    199980
     cccttcccag agggtaagtt ctccaagcgt gggaggaagg tccatttcac taaaaggcca    200040
     gaagcttctg gtgaattcca taatgcacaa ccttttttga gaagtggaga ctttggacat    200100
     gagggatttc atgtatgatg ccctggagtt ggattaaata cagcgtaagc tcttgggggt    200160
     atttctggag gaggcccttt ctgcaggaaa ttagaataca tgcaggggat ccaggcagga    200220
     agaatcactt cctttgacat cgtttggcac ctgaaggaag ccctctcagc attgggcact    200280
     ggtcccactt gctggctgca ctttagccag aggcctgcgc ctgatcattc ttgcagtggg    200340
     agagccttac tggggtcaca ggttataaaa atgcctgtca ctctccagct gagcaagtcc    200400
     atatgcgtgc ttgtcagtgt caaccccatg atgagtgcac tctggaaggt gacaatctgc    200460
     acacaaacct cctcccacta gttacccact actatacact actttaaaaa ttaatactta    200520
     taattatata atataatata tattatataa ctatataaaa atgattatga tgatgacatt    200580
     tggctgttgc aatttcttta ggatctctgc atctctcttc actagacatg taagtcttca    200640
     atttcttgtc cctgtgacac gttgcccagt gtttgaagtc aaagttgtgc cgagtacctg    200700
     agtatgtcct cagagaagat ctcagcaatg gttgattgtt ctacagggac ttttccagga    200760
     gacctgtccc agggagtcat gaggttcctc tggaaatact agcccctgca ggagatgaga    200820
     cggaagcagg agaatcaaga gcaggtgaca acattgtcag caggaggctc cagatgcttt    200880
     ccagacacag tgaaccttca cccacttgcc acactgcaca gggagtttgc aggggcacaa    200940
     tgaggtgcct gtctgtggga tgttggggga caaagtgaaa acctgagctc agcatccagg    201000
     aaagtatgaa ctccagccct gataatatgt agcctgtgtt ttctttcctt ctacgtgaac    201060
     actttgcctt attgttaaga acagaggatt ctagctctaa gtgcacggat tgcagacagc    201120
     tatgactttc ctccgggcca ggctcctctt aatgtacttc ttggacttat ttgttgatga    201180
     aggttggttc cattaaatac caaaacaggg ttcatattaa agaaaaatga aagagtgaca    201240
     ggtagattaa acccgagcat ccagttccag tgattctttg accctgcctt cccaaaaatc    201300
     tccgatatga acaatgacaa tgccctccat ggttagatta tattataaac tagattgagc    201360
     tagagtgttt ggtgtattaa gtcactattt tttttagctt ccctgttagt tttttgtttg    201420
     tgtgttagca tttactttaa aattctatta atcagttctc tagtaggtag aaattcatct    201480
     gagagtttct tctcttgttg tccactttga taagatttcc agaagacata agaacccttt    201540
     ttgtttgcaa aaatattcca aagttttaca ctaactagga acatatatac ttaattctaa    201600
     tttttaattt gtttaaaagc tctaataagt gcactgtttt ctgccttttg agttaatttc    201660
     acaacacata ggagaatata tcctaaatga aagtttgtgc taataataca aattattggt    201720
     aaataacctc tgttttaatt attgaggtat tatccattaa tatataatct taaattgatg    201780
     ttctcaatgg gactcttacc taaagaatat acaaaatatt ttcctgatct tgacataaaa    201840
     tagatgtgaa cacattctta gtattcagcc atgtctcctg tctatcacat tatgaaccac    201900
     atgctaactt tgatttactt gggacttgtt ctaatttcaa actagttatt tttttatctt    201960
     catgcagctg gattattatg tgtggctatt ttatcagaga atgataaaga caattttaac    202020
     aattttcact gcaggcacgt ctaggcaagc cccctgtgca caatgacctt cgtgggttgg    202080
     acattctatg gggactctcc cctgtctgcc taggagagtt atctgcctcc tccctctatc    202140
     attttcctct ttgaataagc gcatctaact gccgttagaa tacagacaaa ggccaacctt    202200
     aactgctccc agctgacagg ggatgctgtt ttgggaagat ctcccttgag gtctgtctaa    202260
     gggacctagt aaaagggagc cattatccca ggcttcactt ggatgaccat ttggagttga    202320
     tgcctgaagg tgagaagaga caaaccaggt tattagaaga catgtatcaa aaccaaacaa    202380
     ggtggtaagg acagtttgaa aaaaaattcc aaggctgctg acacacccag ataactggcg    202440
     gctgtagtta tgcctgctaa gatttgggtg catggggctt ggctttcgtt agctccctag    202500
     gacttatttt cccaaacaaa gaaacctccg ggttaggggg accctattta ttccagtcac    202560
     ctggcatgat ttgcaggata attgctcaga attaaaatat tcgtccagat gtttatatag    202620
     cccatgcctg tgtttcttct gagctgcagc cagagatcat tggttcgttc acagcgataa    202680
     gcagagttag tctaaaatgg aggcaaatac ttaaaacaat tgaagagact gtaatttaaa    202740
     gacaaatgta tgatatgttt tgaaacataa tttttctctc tccagttctg atttttgtca    202800
     gaaactaatc attataggac tgagtgattt gcaaaataaa ctttagtctt gtggttggtc    202860
     tgatcatttg cataaagtgg agcaataata attaataata attctgtagg aaaagcctgc    202920
     aagcaccagg agcttcacag tctaacagta tgagcacatg catcctccag caactcactg    202980
     aatattttca agtcagctgg ttcttagctt aaataacatc cagttggtat ctgtcccagg    203040
     aacactaata tatggttctc tctgcaggcc cctctcgcca cagatttaag gtttattttt    203100
     ttcctctgtg atatcaactc agatatgttg aaggtttttc ccacatttgt ggtttttcag    203160
     gtttgttgtt aataaggtca gaataagatc atagtttact cattttttta cattcccatg    203220
     ctgagtagct acttttctct ataaaatcca ttagctgaga gaaaaaataa cattttccta    203280
     acggtgaaca attaaatagt ttgacatata tttgtgtacc agtatataat gcagcttcga    203340
     atcaaggtgt gcctcaatca taaaaaacat ggctaaattc tcaaagaatt gtgctgagtg    203400
     aaagaagcta aggaattaag agtaaatttt atataattca ttgtagaaat attagaagat    203460
     gccactacca taaattaaaa tgaagaagac ttaaattttt ctgagaaaat ggtgttggga    203520
     atgatgcgga tgtgatttaa gtttcagagg aataaggaaa aagatttagg gattaattta    203580
     attattcaaa agttgattga agtgccgagt gaatggctgc aaacatagct ctacattttt    203640
     caaatcattc cctataaatc tgaattaatt atttatttat tatacttgaa taaagcaata    203700
     acgaagaaat aaatgaatat ttttgctaaa atggagcaat aaaaagactg atattgacag    203760
     aagaaatatg actgacttct gaaaacacac atgaaccatg gttctctctg catatttagg    203820
     tgaattacag aaagttgtca taacagatgg ggaatcctgc agacttcact aggcatggtc    203880
     cacgctgccc tggagttgtc tcaggggagc tgcctcctcc ggtgattaga gcacaggccc    203940
     agataatagg attacatttt tttagatgtg taaacttaga cgcactgcac agctgctgta    204000
     ttctctatgt aaattatctt ctgtaaaata caacattaaa ggctgcatta aatatattgt    204060
     gtaaatatgt aaaaataaaa tcagattatg agagctaaat gttaatcaag gcacaatcac    204120
     ataatataaa attatatttt cctgaatgat ggaattacta ccaatctccc ccaggagact    204180
     tcatctgcac tgggcccggc ctctcctcag atgtcccatc acagagcttg ctatataatg    204240
     ggggacatgc aaatagggcc ctccctctgc tgatgaaaac cagcccagcc ctgaccctgc    204300
     agctctggga gaggagccca gcactgggat tccgaggtgt ttccattcag tgatctgcac    204360
     tgaacacaga ggactcgcca tggagtttgg gctgagctgg gttttccttg ttgctatttt    204420
     aaaaggtgat tcatggagaa ctagagatat tgagtgtgag tgaacacgag tgagagaaac    204480
     agtggatatg tgtggcagtt tctaaccaat gtctctgtgt ttgcaggtgt ccagtgtgag    204540
     gtgcagctgg tggagtctgg aggaggcttg atccagcctg gggggtccct gagactctcc    204600
     tgtgcagcct ctgggttcac cgtcagtagc aactacatga gctgggtccg ccaggctcca    204660
     gggaaggggc tggagtgggt ctcagttatt tatagctgtg gtagcacata ctacgcagac    204720
     tccgtgaagg gccgattcac catctccaga gacaattcca agaacacgct gtatcttcaa    204780
     atgaacagcc tgagagctga ggacacggct gtgtattact gtgcgagaga cacagtgagg    204840
     ggaggccatt gtgcgcccag acacaaacct ccctgcagga acgctggggg aatcagcggc    204900
     agggggcgct caggagccac tgatcagagt cagccccgga ggcaggtgca ggtggaggct    204960
     gtttcctgtc aggatgtggg acttcatctt cttccaacag tttctctaat gaacctctct    205020
     aattttagaa ttctgtggtt cctaatgtca tctctacata ttttcaaaag atcattttaa    205080
     catgaggaca taacctctca tgcaccaaat gcacattgat gcttacaaag atgaaaagtt    205140
     ctcaaccatt gtgaccagga tcgcagtcct gaggaagctc acgggtgtct gatgagtctc    205200
     ctccattcag gcccaggaca gaaacctcag agggattcct ggaccagaac ggcagggatt    205260
     ctgatcacag ccaatagaga ggctgggcca gggtcagtgt cctgtagaag ctcacagggt    205320
     tcaagtctga cccttctcct gaccctaaag ccaatgagca tcagcactga tctggtgctg    205380
     cttttgctcc caatacatgt tctttctttg gagtgtttgt tctccctttt ttatttgctt    205440
     ttctttcttc ctgaaaaaga aacacatggt ctctgtgatc tacactccag ggctcaaggc    205500
     attttcttag aactcaggca aggctcaggc ttggctactc cagccacgtg ggagaggctg    205560
     acgggatttc cttctctctc catattctca gggccctcct ctgtgttgtg tgtagactca    205620
     tctgggaatg caattggctg ttagtagtga aggggatgaa ctcatttgat caaaatggga    205680
     tgtggatgtg gaattaaccc tgttctatgc acactgtcag agtcatcttc ttcagaagta    205740
     gtgttagaaa gagcttgtga aatttatcgg caacaaaatg gatccccttg tgttaaaacc    205800
     ctaatgaatg aagctgggga aggccatgaa ggagggttct aacacatatt cctgataaga    205860
     tgaactgtca taaatagact ctgcacagcc acaacctttt acacggagac caccacagcc    205920
     ttaaaagctt tatttctcca agtaaatctg ccctgcaact gcctgttcaa ccttacaccg    205980
     ctgtctcaaa atagctgctg tcaccctcct catttttcct taattctctt ttttattatt    206040
     ttttaaatta tactttaagt tctagggtac atgtgctcaa agtgcaggtt tgttacatat    206100
     gtatacatgt gccatgttgg tttgctgccc ccattaactc atcatttaca ttaggtattt    206160
     ctcctaatgc tatccctccc ccttcccgcc accccatgac aggccccggt gtgtgatgtt    206220
     tcccaccctg tgtccacatg ttctccttaa ttttttatct tccttttcct acctgaatgt    206280
     acccatacat attttaattg aaatgcacat cctggaaaaa atattattat actttagagt    206340
     ctctttctgt ctgttattca ggttgacaag ctgcagacgg acatgccaca tttctgtgag    206400
     acatagagga tgacagtttt tggagatgtc taggaatctc caatgtccat aagatcagcc    206460
     atcaataaat gcagactgga ggtcccagag agaagtgaag ctgctgaatc gccgtggaat    206520
     ttcattttct ccagttctgc tctgatggaa tcaggcccac caattttatc aatgacaatc    206580
     tacctaactt agagtcaatt gataacagta ttaatatcat ctattaagta aattcataac    206640
     tatatatata gtaagagagg gagagagaga gacagagtct cgctctgtta cccagggtgg    206700
     agtgcaatgt catgatcttg gctcactgca acctctgtct ccggagttca agcagttctc    206760
     ctgtctcagc ctcctcccag tatctggaaa tacaggcaca caccaccata tccggctaat    206820
     ttttgtattt ttagtagaga cagtgtttca ccacattggt caggctggtc tcaaactact    206880
     gacctcaggt gatccacccg cctcagcctc ccaaagtgct gggattacag gtgcaggcca    206940
     ccaagtccgg ctaatttttg tatttttagt agagatgggg tttcacaata ttggtctggc    207000
     tggtctcaaa ctcatgatct cagcctccca aagtgctggg attacagatg tgagctactg    207060
     tgcccggcca acatcaccta tattaatgtt ggattgaata actacaaagt ctaccctaac    207120
     caagtgtacc atcaaactta ccattaccca gagggaagaa tctttaacat gagatcgatt    207180
     tcttaagtgt tttaaggtgc aaaactgacc actgtacaat cacccaatta tgattttgct    207240
     aatgagttgt agaagttgca tgtacatttt ggatattaac actttttcag atgcacagct    207300
     tataattata ttctctcttt ctgtgggttg gaaatttttt ccatgctttg cagcatcttt    207360
     ttgctgtgat gtagttctgc ttgttcaatt ctgcttttat tgtctgtgcc tttaatttga    207420
     aatataggaa atcattctta atattatttt ctgggtgggg gaaattttac aattgcaagc    207480
     catacgttta agtatttcgc ccatttagag taaattttgg tatttaatct aaactaaaat    207540
     tcttaattct ttgcatgtgt agattcagtt tttctaacac cctcttaggg agagactttg    207600
     attcatccat tgtatattgt tagttctcat gctgaaaatc agtttgctgt agatatatta    207660
     gtttatttct gagctctctc tatatattaa tgccaatacc atcctgttat tatttatgga    207720
     tcaaaatgtg catatttgtg aatttcaaaa tgttgttcag aacttgtgca tgccaacaac    207780
     tgtgtttctc ctgcactctt gggctagtga agctctcaca gaccctctct ctcacctgtg    207840
     ctgtctctgc attctccatc acaaccagtg cttcctgctg gagctgcatc catcaccccc    207900
     cgcaaggaag ggactggagc gaatcaggtg cacaggtcat gagggagtgc acattccaac    207960
     ccactcctca agagtccagt cactatctcc agatccacat ccaaaaaaca gtgtttcctg    208020
     tagctgagct acctgagcaa caagtacaca accatgaatt ttaatacaaa agacacaacg    208080
     aggggaagtc attgtgagcc cagatacaaa cctccctgca ggggagctca gaaagagcag    208140
     gaggcactca ggacaccagg gaacactctg gacacatcaa ggcaggtgca atggggggac    208200
     aaggggctgg agatggggtt tggcatcacc atcgtatttc accactggac acccaccacc    208260
     gtgtttattc tcatgtacct gattctgtgt attattagaa aatggcattt atataaatat    208320
     atagccatat gtaggtgcat caagttgtcc tctccatcgt atgtggacct ttccactaag    208380
     actctgagtc cctgtattta tatgagcacc tcataaatta tggtcaattt tgtgggatct    208440
     ctatcttttc tttctccctt cttccctcct tctctctcac tcacaaactc acacacacgc    208500
     aaaattctac aacttcagtt acgtgatgtg ttgaagacag tttataatgt gcagcttttc    208560
     cagtttagct gatgttcata ttgctgtgag aataggaaca ttgtgtttct cagctgtgta    208620
     tttctctaag gtagcagcaa cttttttttt taatttgaca ttttttaaaa attttattat    208680
     tattatactt taagttttag ggtacatgtg cacaaagtgc aggtttgtta catatatata    208740
     catgtgccat gttggtgtgc tgcacccatt aactcgtcat ttagcattag gtatatctcc    208800
     taatgcattt ttttaaatac ccagatactg aaagcaatcc aaatatcaat cagcagctta    208860
     atcataaaca aattgtggta aattcattcc ctggaaaatc acccactgtt acattctggt    208920
     accgctggat acgttcaaca gcaaaggtaa agtcacaagt acatatgatg agtcaaataa    208980
     gacaaataaa tacaagtaca cacatacaat tcctctttta taaattctat aaaattaata    209040
     ccaatctaaa gttacataaa gaaaaccagt agttgactct gggtgtggtg tgagaaggaa    209100
     aggtgtagga acaagaaatt acaggaaaac aagaggaaat tttgagggca cttgagtttt    209160
     ttctgtgttg agaaaggtaa tggttatgtc actatttgtc aaattgtgca cattatggga    209220
     tggctattat ttgctaattt cacctcatta aaatatgcca aatttaaaca catataattt    209280
     ggtagaaaat aagttagaca gagatgaata aaatatatga gaaataaaaa aagaaaatca    209340
     cagaacgttg gcataaggac ttcactcatc aaactgaaga aattttaaat ttctcaacac    209400
     agaattaaag atttaattaa agatatatga gaaatcaaag actcctggat atatacatga    209460
     ataaacccta agcctaccta tatttttagg gaaacactag aatacaacaa aataatgtca    209520
     tgaattcatt acataatggg cgttaatcaa accccaccag gcatgtccag ctgtgtcctg    209580
     gggttgattc agggaacagg tgtgtcctgt ggttaggaga agtggcaaca agctcacagc    209640
     atctgttcta gttgacacca taaaaaggcc aagagatcac aaccaaaatg tagtgtggat    209700
     gtcacatctg tgagtgccgc acactccccc atgtgaatac gaaaaggttg attacctctt    209760
     gaggtgtctg ctgagagtag agctggtctc tcaggaatgt ccaaaatggc tcgatagagc    209820
     aagaaaggag actggctcag gttgctatac cagtttggtg gtggaggcag agtgagggtt    209880
     ctcactcaca gaaatgggtt tgtggggttt gaacctccaa atggcatcaa atgagggagt    209940
     tcttgtgatt cctaactaga tccacctcgt gtcggaaaga aaggagatga tggaggaatg    210000
     agccttaagt tatcagcagt cagacatcaa aacaagtctg atgacttatt ctatgtagca    210060
     actataaata aatgaataaa aatagatcat aatgaattat tcataataaa tgaataaaaa    210120
     tatgaataaa aatagatcag tgtccaatat gggtggacaa aaaccagtct acacaaaaca    210180
     agaagactgc ttataagaat aagcaaatca taatgaaaag gaggaaaatg aggtgaaagg    210240
     cacggggcag gggattacac aagggtcctg ttccaatgtc ttgtgtggag acttttcatt    210300
     acctaaaatc atccgcttgt tctgaatgtc ttaggtgagc tgtctgctcc aaaacatcag    210360
     aagcgccaga ggattcatga ggatattcag tttaatatct tctatttaag gtgccttaca    210420
     atagtgtaaa attcttaatt attattatta taataagaga tagggtctca ctgtgtcaca    210480
     caggttggag tgcagtggca caaccatagc tcactgtaac cttgaacctc tggctcacac    210540
     aatccttctt tataagcctc ctgagtagtg aggacatgag accaagctga ttttttttta    210600
     attctttcat agaagtgggg tctctctatg ttatccaggc tggtcttgac tactagtctt    210660
     atgtgattcc cctggttttg cttctcaaag tggtagaatt atagacatga gccacgatat    210720
     tcggtctgaa ctgattcttt agttgcaaaa tatcaaccca agaattgact cccttaattt    210780
     ttttctgcag tgttttggtt aatattgcct gataaaattt attctaattt gtttcaacag    210840
     cagaattctt cctgacgttt tttccaatat atgtattttc atggttgaag atcacaaaat    210900
     actgttaaaa catgacataa aaaggcaacc ttaaactttt ggtgattgga gttagtataa    210960
     tacaaaaatt acgtccaata ttgtgtaaca cacacatgca aaaagacgaa ggtgagcaat    211020
     gagggtaaac tttctaaatg tatgaatctt ccagctccca agtcatagga tagtaactga    211080
     tgtggcctga gggaggtacc agagtgacac agtgctagtg gcataactct tggccaagag    211140
     actttcaata ttttattaaa gtttttatat ttttttaatt taatgatgcc atttttttca    211200
     acatattcag aatattgtga gtgggattga ttctggatga tatgaacaat gggaatgcct    211260
     tctatgttta gataatatta aggaccaggt tgagattgaa agtttgtaga aatgatgcac    211320
     cataacatta ttctagtttc catactaggt tttttctctt tgcttgcatt tcatttcaaa    211380
     tttatatgtc agatatctag aatgtaataa tttatttaaa aatttctttt cttgtagtcc    211440
     attttgatgg ggcttccaga agatttgaaa acctcttctg cctgcaggac attttaaaat    211500
     tctgcactac agtaaaacat ctgtatttaa tcatggtctt cggtttattg acaagctcta    211560
     gtaagtgcaa taacttctgc cttctcggct gattttacat cagatagagg aatttatctt    211620
     aaatgaaagt ttgtactagc aatataaatt atgagttaat aaccaccacc tttattattt    211680
     aggtgttatt catcaagaaa taatgttaaa tcaaggctct caacgggaat attatctaag    211740
     ggatgcacaa aatgttttgc taatctagat aaaatagatg tgaacacact cttagtatcc    211800
     agtcatgtct cctgtctgtc acattattaa ccacacggta actttgactt cacttcggac    211860
     tcgttctaat tttcaaattc gttacatatt aagcttaatg tctctagact attctagcag    211920
     agtgaagaaa gacaagctag actgacaaac aacctaggat gacaaacttc ttgtcactct    211980
     ctataagaac ttgcttctcc ctgaacttca gacttgtcgc gtgttttgaa acattaatta    212040
     tcaaaagtat taaagaaaat ttgcaaagct acatcttcta tgtttatgtt attgttgatg    212100
     caattgtaaa aaataaggaa aatgtattcc ttttctatgt gcgtttccaa acttaatagc    212160
     agatttttag taagacccag aataataaaa aattcaaata ttgttcagct gcttaacaga    212220
     aaaacaaatt atggtaaatt tgtttgctag aatgctatcc agtatttaga ataaataaat    212280
     gtctgctata cccaactata aggtaaaata tcgaattaca tatgttaagt aaaataagcc    212340
     aaacaaataa gaatatatac tgcattgatt tgtataaatt ttaataaact aaatgactca    212400
     gccgcaatgt atagctcagg agttgccaga gaaatggtag aagaatgaaa gggaaaagga    212460
     ggaagaatgt agaagaacaa aaggaaatgt tgagaattct cttgtccacc ttgataacaa    212520
     tgatggttac attatattta tcaattgtac aatttaaata tgtgaaagtt tattatctgt    212580
     aaactaaaac tctcaaaatt tattacaagc aaacaaatgg aaacttagac aaagaaggag    212640
     tgatagaaag atagaaaaaa tgtatattaa atttcagaaa tacctaagaa cttatctgcc    212700
     tgaaccctag ttctcaccat atttttaggt gaatgctaga atgcagcaaa atcacacgtg    212760
     ttctcactac agaaagtggg ttccacaacc acactaggca cgcccagctc tgtcctggag    212820
     ttggctcagg gagtaattgg ggccagtgat gaggagcaca ggctcaggta ctggggctta    212880
     ctcatccaga cgtgagctct tagacacata cttagccctt tctccatgcg tggttgactt    212940
     ccacatctgt acatggagaa acaattgact ccgacaaaac ataatttgca caaatatgta    213000
     aaaataaaat aggatgatga attcaaaaag tttatcacag cataatttta taataagaca    213060
     gcatattttc tgagtaccat cgttgtcacc aaattcttgc agggcacagt cattttatct    213120
     gggtactgcc ttctcgtcag gcttcccacc ccagagcttg ctatatagta ggagacatgc    213180
     aaatagggcc atctctctgc tgaagaaaac cagcccagcc ctgactctgc agctctggga    213240
     gaggggcccc agccctggga tgcccaggtg tttccacttg gtgatcagca ctgaacaccg    213300
     actaccaacc atatgaagtc tgggctgagc cggtttttcc ttgttgctat tttaaaagat    213360
     aattaatggt gaactagaga tacttagtgt gaggggacat gagtgagaga aacagtggat    213420
     agtgtggcag tttctgatca cgatgtctct ggaggtgtcc agtgtgaggt gcagctggtg    213480
     gagtctgggg gaggcttggt acagcctggg gggtccctga gactctcctg tgcagcctct    213540
     ggattcacct tctgttaact actgcatgca gtggatctgc cgggctccag gaaaggggct    213600
     ggagtgggta ggtttcacta aaaacaaaac taatcgtgga acaacagaat acgccgcgtc    213660
     tgtgaaaggc agattcacca tctcaagcga tgattccaaa agcatcgcct atctgcaaat    213720
     gaacagcctg aaaaccgagg acacggccgt gtattactat accagagaca cagtgagggg    213780
     aggtcagtgt gagcccagac acaaacctcc ctgcaggggc gcgcggggcc accgggggcg    213840
     gtcaaaaccc actgaggaca ggacaggtcc caggagcaga tgctggggta gttttccttt    213900
     ctcatcagct ggagaagtca ggtttgtatt tgcaggactc tgcagcattc taggctgtga    213960
     cattttatca cttgtattta ttatgacttt attatcgtta gtatttaaat tttagtaatt    214020
     attaaaatta tatataagta cattttaaat atgtactttg aagatataaa tattcctaat    214080
     tatttgcact gattcttcca gagttttatt aacatttgtt gacatcagca actacatagc    214140
     tatagggaca taaatttata aacatagaaa tatgtatcgg caagacatgg tggtctgtgc    214200
     ctgtaacccc agcttgggaa gctgaggcag aagtactgct tgaacccaga aggcggaggt    214260
     tgcaatgagc cgtgatcgcg ccactgcact ccagccgggc aactgagact ccatctcaaa    214320
     aaactaaata aataaaaaga aagaaagaaa aaaagatgta caaatacaca gacctatgca    214380
     tatatgtgta ggcattcata ttaaacatta agataaaata attctaaaaa catgtcctga    214440
     agaatcaaaa ttaatgatga actaaatata aattattaga gtaattcata attgattttg    214500
     gttattttta attgtttaca tgttgtgatt ttacacaaaa tattagtcat ttatcctgat    214560
     aaaacaggtc aaaatgttat atcaagagtt tatttacttt gtattactaa taatataaaa    214620
     tgttccacat atttttagcc atgttttact tatctgtgaa atgtgtttat taatttttca    214680
     ttttaatatg tcacctttat ctttctaatt tcctggattt aattccacta cagctgaatg    214740
     cattttaata attattgtaa tcacatttac attttttatt tgaattttca tatatatttt    214800
     attaggattt taatatcaat ataaatattt tcctaccatt gtctattttc tatttttatg    214860
     atataaaatt aacataaaaa tgcttagatt ttcaatgtac agcttgaggg cttctgacaa    214920
     atgtgcgcac aaataagaat tatttgctta ctttttagtg tatactgagg agtgggattg    214980
     ctgggtaaaa tgatatctct gttttaagtt ctttaagaaa tctccagtct gctttccaaa    215040
     ggggcaagat taacttatat tcttctcatc agggtataag tgttctcttt tctccagagt    215100
     cccaccagca ttcattgttt tttgactttt tcgtgatagc cattctgagt ggtgtgttgc    215160
     tgcacaccta caatcatctc atatttgata aggctgatga aaacaagcaa taaggaaggg    215220
     actccctgtt caataaatgg tgctgggaca actggctagc catgtgatga agattgaagc    215280
     tggacgtcta ctttcaacat acataaaatt aactcaaaat tgatgaaaga cttaaatgta    215340
     agacctcaaa ccataaaaat ccttgaagac aacctaggaa atactcttct cgaccccaac    215400
     tttgacgaat aatttttggc tgagtatcca aaagcaaatt caaccaaaac acaaatagat    215460
     gagtgggacc taattaacta aggagtttct gcacagcaaa cccgccagga gatagatgct    215520
     caggacacca gagagaactc agttaaacca gggggcgctc agaacagcag agtgtgctca    215580
     ggacaccagg gggcgctcag aaccaccagc aggagctcag aacagcagag tgtgctcagg    215640
     acaccagggg gcgctcagaa ccaccagcag gagctcagaa cagcagagtg tgctcaggac    215700
     accagggggc gctcagaacc accagcgggc gctcagaaca gcagagtgtg ctcaggacac    215760
     cagggggcgc tcagaaccac cagtgggcgc tcagaacagc agagtgtgct caggacacca    215820
     gggggcgctc agaaccacca gcgggcgctc agaacagcag agtgtgctca ggacaccagg    215880
     gggcgctcag aaccaccagc gggcgctcag aaccaccagg gggcgctcag gacggcaggg    215940
     gtcgctcaga caccacggtt cccttaggag gcagctccac attaggtccc tgggcagggt    216000
     ggggtttcct ttttgaactt gctatttttt gaccttgtaa agcaaaggtc ttccccagga    216060
     tctcttagta tttcttcctt gtaactcatg tcttgtcttg ttttgttttt gtttttgttt    216120
     tttgtttttg gtcatctaca aaaacttaaa cttagaacag ttgattcaga aaggcaggaa    216180
     agcaggcatt cctctaagtc tcccctgaga caaatacata tatgatttct tcctccccac    216240
     tataatttat gtaaaaatga agattcactg agtcagacta aattgtgtat tcagtggaag    216300
     gataataaat gacacaaaag aatgcaacct attgtctctt atcttcttct aacctgcaag    216360
     cccccatttt catttgtcct ggcttacagg aaaaaaaaat gtacatttca catgtattga    216420
     ctgatgtctc atgtctctgt aaaatgtata aaagcaagct gtacttcaat caccttgtgc    216480
     acatgtctca ggacttcctg aggctggtta tggttgggtt cttaactttg gcaaaacaaa    216540
     tgtattagtc tgttctcctg ctgctgataa aaacataatc aagactgggt aacgtataaa    216600
     ggaaagagtt ttaattgact cacatttcca cgtggctggg gacacttcgc aatcatgtca    216660
     gaaaaggaag ggacatctta catggtggaa gactagagag agcttgtgca gatgaattcc    216720
     cctttagaaa accatcagat ctaatgaaac ttattcacta tcatgagttc atcatgggaa    216780
     agacctgccc ctatgattca attacttctc actgggttct tccaatgaca catgggaaat    216840
     gtgggactac aattcaaaat gagttttgga tggggacaca gacaaaccat attaataaac    216900
     ttcctaaatt gactgagacc tgtctcagat atttgttgtt tatcctactc atgtacagcc    216960
     ttcagagttc caaagcctat atcagttttc caggattgtt tccccttttt gttggttatt    217020
     tcctccttta ttttctatgt ttagttccct ttttctccca ctattttttc atcattggga    217080
     tgtgagactt cacaacattt gaaaggtagg taacaatgag ctatcttaac aacatgggac    217140
     ctgtttatct aagagtaatc cattctatca atgaaagata aaacaaaaca ggagaccaga    217200
     aactcatttg gttgtaaaat gcttcctctg aatgattttg aaaaggaaca ggtggggaaa    217260
     atataaaagg aaaataaaaa cttgtgctgt caattcatta tgtcatgagg ggggaaaatc    217320
     ctaaaggatg atccatgcaa gaaactgatt ttcctttcat tcctaagaga atagctacaa    217380
     ataaaaagtt aaatatcttc acagatagct actctttgtt cattttactt tatataaagt    217440
     gctaacttag ttcaggagaa ctacataatt tttctgtttc cctatgtgct tctctctcat    217500
     tacaacatgt aaattttcat actgtccctc tttcccctct atccagcttt cccccctttt    217560
     ttgtattgaa agccctgaaa atcatctttg gggaacggca ctgaccacaa atttttctgt    217620
     gattacttgt attttcattc caggcatgcc ctaactttgg caaaattaat tttaatttga    217680
     ttgagatctg tctcggaaac ctttggttta cactaggaaa gatcccaaat tacgagtcaa    217740
     ttactataaa gctcagcctt cccactgtgt atgtgtgtgt gcatgtgtgt gtgtgtgtgt    217800
     gtacacgtgt gttttaattt ttgtgggctt tgagccatgt agttctctct gtggacatac    217860
     tatttggcgt gatctttgaa tagagaattc tgaaagaaat aagaggctcc tatgagttct    217920
     ctgaaagttt ctggactcac catggatctt gactgtgtca ttgcatctga cagtcccagg    217980
     gaacagactc tctggtggtt tcaaagaatc tgtgcttggg ctccccctgc agtttactgg    218040
     gtacagtaat gttaaatcac tgtttcgaga gacaatttcg aaagcattag atgctgctga    218100
     gagaggattg tgaaccaggg gacagcccct tcattctggg ggagcgacat tgggagaata    218160
     tgctctgtga gcccaaacag catcctcccc tgccgggtga gggcagagct gcaggacagg    218220
     cccagaaacc actcaacaca gatgtcagcc ctggagcagc tgcagaggag tctgaggaga    218280
     aaattttacc agcatctgaa ttacacttat ttcaaaacaa aaatgcaatt aaaaagttaa    218340
     aataagtaat taatgtccag gcacagtggc ttacacctat aatcccagaa atttgggagg    218400
     ctgaggtggg aagatttcca ggagtttgag atcagtctgt gaaacatagt gaggcttcat    218460
     ctttactttt gaaaataaaa taaaataaaa cataaataat ttagcaaaaa ctaccgatgt    218520
     gtcttccata tcccatcgta cttagagcat tgatgtaacc caatgagtca atgagaacca    218580
     aatttgaaag gagaaaattt tagagttttc agatatctta agagttggaa gatgtagaac    218640
     aaaactaatt ttatcaattc aatgtgtgca aacatggaga gacacactca tgccagtagt    218700
     tcaacttgca aagggcaaaa accaaaaaag tttgaagttg ctagtgatcc atttgtaggt    218760
     gagatcattt tgaggatcat gtcctgtgag aggctgtttc tctattagag gagttctgtg    218820
     ctcatgaagt gctggacatg ctaggggaca aatatcagta aacaaacatc agaacttgaa    218880
     tgtcagcttc cccctgctgc attctccgtg tgtcatctct ctgttatttc tcatgctaga    218940
     tcaggtcttt agctatgaaa tattccacct aatttacatg tcaatagctt gaagtctact    219000
     gagtttaatt catatttttt ctatatgtta cagttacagt ttgtccacaa ggacaaggtt    219060
     tgtccacaag tttgtccaca aggttacagt ttgtccacaa ggactcaatc atagaagtgc    219120
     agagtccttt tcgggccaca tttaattcac tttatcagtg cccttcagta tgtggttcct    219180
     gagaatttca catgacaaca catttaccac actggaattt aagcaatcca acacatgttt    219240
     gtagctttat cttgtaatag gctgtatttc atatggcagc ctctgcttca gtttagctaa    219300
     cactatggct ttgtttctct ctacaagaac ttgtttctcc caagatttcc atgtttgtga    219360
     aaggaaaaga aatctctggg acccccaaat cactaagcca aagggaaaag tcaagctgaa    219420
     aactgcttgg ggcaaaccca cctccattct ttccctaaaa tgatagctac taactgtcag    219480
     gcctctgagc ccaagataag ccatcatatc ccctgtgacc tgcacataca catccagatg    219540
     gccggttcct gccttaatgg atgacattcc accacaaaag aagtgaaaat ggcctattcc    219600
     tgccttaact gatgatacta tcttgtgaaa ttccttctcc tggctcatcc tggctcaaaa    219660
     gctcccctac tgagcacctt gtgaccccct ctcctgcccg ccagagaaca accccccttt    219720
     ttcctttacc tacccaaatc ctataaaacg gccccacccc tatctccctt cgctgactct    219780
     cttttcagac tcagcccgcc tgcccccagg tgaaataaac agctttattg ctcacacaaa    219840
     gcctgtttgg tggtctcttc acatggacac gagtgaaact aaggttttaa aaaactacat    219900
     acctccttca aaatttgacc acaaggaaaa accttgtgga ccaacaacag acagagtcat    219960
     ttctctgctc acataagtca aatgcctatc tgattgccac catttctcta ttgttttgct    220020
     aagccagact aaggcctacg tgactattcc tgtaaattgt gcattcagtt aaaggctaat    220080
     cagaaactca agagaatgca agcatttgtc tcaaacctac ctgtgatctg gacgctctct    220140
     ccccacttca gaaagtcctg cctttctgaa ccaaaccaat gtacatctta catatattga    220200
     ttactgtccc atgtctccct aaattgcata aaatcaagct gtgtcccacc accttgggca    220260
     tatatcgtca ggactccctg aggcggtgtc actgccatgt ccttaatctt ggaatacgaa    220320
     cttcctaaat ctattgagat tagtctcagg tactctttgg attacaggtt tgttttttat    220380
     ttcataactt caattatctg aaatactaaa caaaatttgc caaattgtac attctcttgt    220440
     tactgttgtt gcaaaaaata tatttatgta acttatatat aatttatcat ctatgtgtgt    220500
     taccaagctg agtagcagat ttattagtaa gacttagaga gagtaatgaa aaattcaaat    220560
     gccagttagt aacttaatag aaaaacaaat tatgctacat ttgtttgctg aaatgctacc    220620
     cattatttgt aagaaataaa cacatgcaac ataaaagatt taattctcag tatttctatt    220680
     gagaaaaata atccaaatac ataagagcat atactatatt ttttcattct tataaattct    220740
     agaatataaa agctagtcaa aagacatgtg aaaacatcag tagatttata aagaaatggt    220800
     agaagaaggg aaggagaaaa accaaaaaaa tataaaagag caagaggaat gctgagggga    220860
     gttgacttgt caccttctta aaaatagaga ttttattttt caaagtttac tattgtacag    220920
     aataaatatg tgaattttct tatctgtcaa ttaaacctca taaaatttat tacaaaaaaa    220980
     ctgaaatttt agacaaaaag agggtgatag gaaggaacaa ataaatatgt taaatgtcaa    221040
     atatacctaa aaatttattt gtctgacccc tagttttctc cgtattttta ggtaaatgca    221100
     gcaaaatcac acaggttgtc gtggcaggaa gtggattctg caaaccacac taggcccgtt    221160
     tatctctgtc ctagagttgg ttaaaagagc aactgaggcc agctgtgagg agcataggcc    221220
     cgggtactag gactcactca tgccagatat aagcccttag acacatacat agcccctcca    221280
     tgtgtgggtt cacttttaca tctgtacatg aagaaaccac tgattcctaa ataacataat    221340
     ttatacacat aggtaaaaat aattaaaaat gtgatagtta ttaagtgttt atcacacaac    221400
     aatttcacaa taaaacagca ttttcccaaa tgtaatcatt gtcatcgaaa tccccaagga    221460
     cactctcatc tgccctgggc cctgccctct cctcaggcat ctcaccccag agcttgctat    221520
     atagtaggag acatgcaaat aggtccctcc ctctcctgat gaaaaccagc ccagccctga    221580
     ctccgcagct ctgggagagg agcccccgcc ctgggattcc caggtgtttt catttggtga    221640
     tcagcactga acacagaaga gtcatgatgg agtttgggct gagctgggtt ttccttgttg    221700
     ctatttttaa aggtgattca tgaggaaata gagatattga gtgtgagtgg acatgagtga    221760
     gagaaacagt ggatttgtgt ggcagtttct gaccttggtg tctctgtgtt tgcaggtgtc    221820
     cagtgtgagg tgcagctggt ggagtctggg gaaggcttgg tccagcctgg ggggtccctg    221880
     agactctcct gtgcagcctc tggattcacc ttcagtagct atgctatgca ctgggtccgc    221940
     caggctccag ggaagggact ggaatatgtt tcagctatta gtagtaatgg gggtagcaca    222000
     tattatgcag actctgtgaa gggcagattc accatctcca gagacaattc caagaacacg    222060
     ctgtatcttc aaatgggcag cctgagagct gaggacatgg ctgtgtatta ctgtgcgaga    222120
     gacacagtga ggagaagtta atgtgggacc atgcagaaac ctccctgcgg gaacgctggg    222180
     gaaagtcatc tgcagggggc gctcaggagc cactgatcag agtcagcccc agcggcaggt    222240
     gcagatgaag gctgatttcc tgtcacgatg tgggacttca tcttcttaaa gtttctctac    222300
     tgaacctaag ttcggaattc tgtgattact agggtcattt ctacatattt ttaaaatgat    222360
     tgttttaata tgaaaaccta ttctcctatg cagaaaatgc agattgatcc tcacagagga    222420
     gatgaaaagt tctcaaccat ggtcaccact gtcagagtcc tgaggaagct cagggcttcc    222480
     tggtgagtct tctccagtca gactgaggac aggaactcca gaaagaatcc tgactagaac    222540
     tgaggtattg ctcctcacaa gagaactcaa gctgcggggg gtctgttcct gcagacccct    222600
     gacccggtgg cggatgaata aaacgtacac tgacacacag atattctgtt ttgccagttc    222660
     agctgagggt ccgaggcaac ttacagactc caagaagaat cctgtaaaga acggcagcag    222720
     tggccctgag cagctcgcac tccaggcgtg tattgagtat agaattaaca acagaagctc    222780
     tgacaacaca cttgtggata attaacatgg ttaagagagt agttctacga atgattaaag    222840
     ctcagggact gcggtctaaa gtaaatacca ttagggggca ataccctggt tagttaatgg    222900
     aggtagggta accagactta actggggagg cctctattgt ccctagtatt taccctatgc    222960
     ctaacgctct aaggtaagaa ctggccgcct tcagcctgtt caattattac aagctatgta    223020
     acctttcggc cttctaaaaa ggtatatgac tattccctat aactttcctt aatattcccc    223080
     tttaatattt ccgccaccat cctgagtgaa tcccagcaag aacagtcttt ggggattctg    223140
     attacagaaa aaagagaggc tgggccaggg tcagtgtcat gtagaacctc acaggtttct    223200
     cgtaggaccc ttctcctgac actgaagaat gcaaatcaga atcaatactg atctggagct    223260
     tcttttccta attcatttac tgtctttttt agatgttgtt ctcatttttc catttgcttt    223320
     tcctgctttc cgaaaaagga agatgttttt gctgtggtca aaattccaga cctcaagccc    223380
     ttttcttggc gctcaggtgg gtctcaggct gtggctgctg cagtcacgca ggagagtctg    223440
     gtgggacttt cttcactctt cgtcactcag gaccctccac tgtgttgcat ggagactcat    223500
     ctggaaatgc aagttgccag tgggaactga agggacaagc ttgtctggtt aatgtgggat    223560
     gtggatgtgt ttctaatcct gttctgaaaa accgtcacac agtaacgttc ttcactagtg    223620
     gagaaggaag tgggtgtgaa cgttgtcaga ataaaaatgg agccacgtgt gttataatct    223680
     ttacaggcga agctggaaga ggtcatgaat agagggttct catgcacaca tccctgataa    223740
     caagaattac cctaaaaata ctctgcacaa ccacaatctt gaacaaaggc taccacaaca    223800
     ataagagaat taatactgag gggatatctg ccctgcaact ccctgttcag ccttaaactg    223860
     attctaccct tgttattgat tcttctaccc caggataatt gtctcaaaat agctcaggta    223920
     atcttctcat ttatccttca gacacttgtc tttctttacc aacctaaatg tgaccatgga    223980
     taatcccatt gcaatgctca ttttcaaata aatacgattt gattttggag aatctctttc    224040
     tctctgatgt ttaggtttga caagctgcag agggacacac cactttcctg tgagatgtag    224100
     gggatgacag ttttggggga tggctggaaa cgtgcagtat ccttagggtc agccatcagt    224160
     aaatgcaggc tggaaatctt agaaagatct caagctgctt aatcaccacg gagttttacc    224220
     ttctccagat ctgttctgat ggaatcaggg ccaagtttgt tattgatgat aatctaccta    224280
     atattgagtc aactgatcac agttttaata acatctattt aaaaattcac acctggatta    224340
     gtgtttgatc aaataactac aaagtattgc ccaaccaagc ataccatcag gcagactgtt    224400
     acccacagag aaaaacattg aacatgagtt tgaggttctc acattgttaa aggtgtaaaa    224460
     ccgattatgt ttaaattatg ctatttttat tattgttatt gagttgtaga tgtttcattt    224520
     acattttgga tattaacgct ttttcagatt catggcatat tatccaattc tgtgagttgg    224580
     aattattttg ttactttgcg gaatcttttt ttaaatctag tcccacttgt ccaattctgt    224640
     gtttttttat ttgtttgaat gtaaaatcca gaaaaagatt gctaattttt tgagggttgg    224700
     gagttttaca attgcacgtg tttcatttaa gtatttaatg catttagaat taattttttt    224760
     gtttattcta acctaaaatt cttaattctt tgcatgtaaa tatccaattt tcataacacg    224820
     ctctttggaa gacactataa tttagcaatt atatattgat ggttctcacg ctgaaagtca    224880
     gctggccatc aatatgtggg tttatatcta agctctctgt atgaatttat gcgaatgcca    224940
     ttctgattta ttactgtatg tttctaataa atgttgaggc ctggaagtgt aatacctcaa    225000
     gctttattct tgccttgtta cagatattag accaaaatat tctaaaattt tactattgag    225060
     tataataata gctgtggctt ttcttaattg gtttcattat gtacaagttg ttttcttgtc    225120
     ttcctacttt gttcagagtt tttataatga aatcctgaat ttttttcaag tgttttctgt    225180
     gtctgatgaa atgttactga gatatttttt ctttagtttt ttaattaatc aactgaattg    225240
     attgatttga gaatgtcaaa tcatctgtgc atctcagaat aaatttgagt tgatcatggt    225300
     gtatggtctt ctataaaatc tttagaattt attttactat tgttgggggg ttaatttatg    225360
     tctactaatg atattggtct atgattttct tttattgtgg tgcctttgtc tattactggt    225420
     agtactctaa cggtagcctc atagaaagag tttggaaggt gtgttgcaga ctacctttaa    225480
     aatagatttt atcagtggag aaacggtgat agtttttttc ttcagttttc tgttgggaag    225540
     agttttgttt gtttgtttgt tttttgtttt tttgttttgt tttgtttgtt ttttgagaca    225600
     gaatctcgct ctgttgccca ggctggagtg cagtggcaga atctcggctc actgcaagct    225660
     ccgcctcctg ggttcatgcc attctcctgc ctcagccccc aagtagctgg gactacaggc    225720
     gcctgccacc acgcctggct aactttttgt atttttagta gagatggggt ttcaccgtgt    225780
     tagccagaat ggtctcgatc tcctgacctc gtgatccgcc cgcctcggcc tcccaaggga    225840
     agagttttat gttgtttaaa agatattctg aataacttaa cctgcttaat gggcttacat    225900
     aatattcctt tcttccattc tttttaaaga actgcttacc tttcctaatt atttttaggg    225960
     tgattttttt cataaagatt gtgcaataca ttttgaggtg agacttagtg gattttttct    226020
     aatgaattag aaataataaa tcacttaatt gactattgta ttaaggttga tttgttgaat    226080
     atttgctaaa ggccagttct ttaagctgtg ccatgtacta aatccctaac cgactatttt    226140
     attcaggttg gtttgttgaa tatttgttag agaccagttc tttacactat ggcatgtaat    226200
     aaattccaaa tggcagtacg tcattgttta cttagctttt gtgcttatat ttttcagagg    226260
     aaaaaaaata ctgtaaattg taaataccca atacctaaca gtattgtatg caaatctgtg    226320
     actgttggca gtgtcatctc tgagaaacag ataaagttta tttactatat ataaaaaaag    226380
     agtttggaag gtggactcct caccaatttt tgaaagagtt agagaagcat tagcattaat    226440
     tctttaaatg ttaagactaa ttttatgatg taacatataa cctatcatgg agaatgttca    226500
     atgggtgctt gagaaggatg tgtattacat ggctcttggt tggaaggttc tgtaaatgtc    226560
     tttcaggtaa atttgttcaa tactgttgtt caagttcaga ggcttattaa gaattttctt    226620
     tctggatttg ctatacatta ttctaagtga ggtattaagg ttttccctta tttttatata    226680
     gttttctatt tctctctata tatatatctt aaagtttgct taatgcattt atatttgtat    226740
     ttgtactgtg taaaaagtaa aataaaataa taattgctta gtgagtttca tggcacaatc    226800
     acgttatgaa taatcatatt ttcccaaatg ctgtcattgc cactaactcc tccagataca    226860
     tggtatatta tccaattctg tgagttggaa ttatttcgtt gctttgcaga atcttttttt    226920
     gttaatctag ttccacttgt tcaattatgc tcctccggga gtctcacatc tgctctgggc    226980
     actgccttct tctcaggcat cccacactgg agcttgctat agaggaggag gcatgaaaac    227040
     agggccctcc ctctactggt gaaaacaagc ccagacctgg ccctgcagct ctgggagaag    227100
     agccacagca ctgggattcc caggggtttc catgtggtaa tcagggctga acacagagct    227160
     cactatgggg tttgagctaa ccagaatttt tcttgttgct attttaaaag gtgactcata    227220
     gagaaataga gtgagtgaga gtgagtggat ataagtgaga aaaacagtag atgtgtttgg    227280
     cagtttctga ccaggacgtt tgtgtatttt caggtgttca gtgtgaggtg gagctgatag    227340
     agtccataga gggcctgaga caacttggga agttcctgag actctcctgt gtagcctctg    227400
     gattcacctt cagtagctac tgaatgagct gggtcaatga gactctaggg aaggggctgg    227460
     agggagtaat agatgtaaaa tatgatggaa gtcagatata ccatgcagac tctgtgaagg    227520
     gcagattcac catctccaaa gacaatgcta agaactcacc gtatctgcaa acgaacagtc    227580
     tgagagctga ggacatgacc atgcatggct gtacataagg ttccaagtga ggaaacatcg    227640
     gtgtgagtcc agacacaaaa tttcctgcag aaagaagaaa ggattctggg ccgaagggga    227700
     cactcagcac tcacaaaaca ggtggagccc cagggcaggt acagaaaagc agtcaagggc    227760
     tgctgtcctt caggatctgt gctttcctct gcatgtagca gttcccctcg aatcctctgc    227820
     acttttatgt ttctgtgccc accatgaggt ccctggatta caaaacttta atttaaaaga    227880
     ggaaacattc ttatatgtcc caaaaacaaa agtaagtatt agaggcacaa aagtgcacag    227940
     gcggccaggt gaggctgtag acactgccac cccgcaatgc cagtctcaca actagcactg    228000
     gagagtagtg ggcattcagt ggagcttcct acctatcctg tggtccgagc taagtccagc    228060
     aaggccattg gtgccttcca gagcacagta gtccatcagg aatctcccat gtgtcccagc    228120
     agcagccttg cctcagtatc tccactgtgc acagccattg tctgggagga gctcccagga    228180
     tgagtgtctt tggcacacac caggtggcgg gtgttagagt gcagtacagc agctggctgc    228240
     ctggtctatt gggctccctg atgttggagg gatttgaggt gcattctcag gcccagcacc    228300
     ctctttgtga atttttatat aaaaaccctg attttacttc attttctcag atgacataga    228360
     taactaaaaa cagaatctgc aaagaaattg taattttcaa ctttacccca aattcattgt    228420
     ttcttaattc tgtgcaagat ccaaacatat ttttgccttc cacatgagaa attgttctgt    228480
     ttaaactgaa atcatttttt ctcatattct ttctttttgc ccaagtacag agatcttgtt    228540
     gaaaataagt tgggttcttt ccacacacta accctcacct cccctagaga aagagcagag    228600
     attgtcctca ctctgagtct aggggaggag ctgttcctgt acaattcaga gcctgcagag    228660
     acccccaggt gcagcttcag tgagtcagac atttctccat gtgggcgacc tccagtgcct    228720
     gtgactgctt ctcaggccta attgtgggtt aagaattagg acactcttta agttatcaca    228780
     tctgaatcct attctgaaaa tcaccataaa gagagatagt tcaatggcta ttctcctgac    228840
     ataagtttct ctttattact tggttccaag tatggagaaa catgtgaccc tatatttgtc    228900
     tgaatccaac atcagaatcc actgcattac tctgggatac tcacggatcg aacacaggtg    228960
     agctctttat tctcacagaa atgtatgtag ttgggaattt caacatattg tccagaacgt    229020
     gtgcctgcca acaactgtgt ttctcggtgc actcttggcc tggtgaagcc ctcacagacc    229080
     ctctccctca tgtgtgtcat ctctgcattc tccatcacaa ccagtgcttc ctcctggagc    229140
     tgcatccatc agcccctctc tgggagggaa tggagtggat tgggtgcata ggtcatgaag    229200
     ggagcacaca ttactcccct ttcctcaaga gtccagtcac catccccaga tccatgtcca    229260
     aaaacagttc ttcctacagc tgagctacat gagcaacaat cacatagcca tatattttta    229320
     agcaaaagac acagtgaggg aatcacagtg tgaactcaca ccaaaacctc cctgtgggga    229380
     tgcataggac agaaggggtt actccggagc ccagggggct ctcaggacac caaggggcac    229440
     tcaagaccat tgtagaggca cgcaggtagc cgggggctct caggaaccat ggaggaaaat    229500
     caggacacca aagggtgctc ggtacagcga ggggctcagg acaattgtgg ggattcagaa    229560
     agaacaggtt caaggctcag cttcagggca ggtgcagctg gtgtgaaaag gggctggatg    229620
     aggcgttttg tgtcaccatc atgtttcacc accagacacc ctccactaca tctgttctaa    229680
     tgcatgtgtt tgtatgatta gaaaatgata ttgatataaa tatataacca tagctaggtg    229740
     tgtctttttc tgccttccgt cctcccttct tctctctctc tcacacagaa atttacatac    229800
     acccacccca caacacacat aaatctataa cttttattac ctgatgtatt caataaacct    229860
     gattaatgtg ttttgcagct tcattgttta tgctgttgta gcaataaaaa ccatctgttt    229920
     cccagctgtg tacttctcta agctgagtag catctttgtt cataataccc agaatgaaaa    229980
     acaacccaaa tgtcaatcat cagcttaact ggtaaacaca ttgtggaaaa gtcattcatt    230040
     gacatactac ccactactac catcagctaa tgttggctat gctcaaaagc atggttaaat    230100
     tcacaagtac ttcagatgag taaaatgagc ccaagtgata aaagtgcata cataggatac    230160
     aactttcata agttctatag aacaaaaagt aatctaaagt tgcaaaaaaa aaatcagtag    230220
     ttcactgtga atattgtagg agaagggatg gtctaggaag gaggaattat ggaacaagat    230280
     aaaatgttga gggaattgac ttgttatcta tgttgatagt gataatgtct atgacaatat    230340
     ttgcaaaatt gaacacttca tgtggagatt attgttttta gtttagcccc attaaagata    230400
     gtattaatta gaacaggtat aaattggtat caaaggaatt aaagataaat aaaatacatg    230460
     aaaagtcaga gaatcctgaa tatacacatg aatgagcact ggacatctct gtatttttag    230520
     agaagcacta gaatacagca aaataatggc ataattttac atcactaaga aagtttatca    230580
     aacccaccag gcatgttgta ttagttcatt ttcacactgg tataaaaaac tacttgagac    230640
     tgggtagttt acaaggaaaa gaggtatagt tggctcacag ttctgcatgg ctggggaggc    230700
     cacaggaaac ttacaatcat ggtggaaggt gaagaggaag caaggcacgt ctcacagccc    230760
     agcaggagag agatagagga ggggaagtga cacatacttt taaaccatca gcggttgtta    230820
     gaactcactc actatcatga gaacaacatg gggaaactgc ccccatgatc caatcacctc    230880
     tcacctggtc cctcccttga catgtgggga ttagaatttg agatgatatt taggtggggc    230940
     acaaaaccaa tctatatcac atgtccagtt ctgtcctgga gttgtttcag ggatccagtg    231000
     tgtcctgttg atagaaacag tgacaccaag ttcatattat cagttgtagt tgacaccatg    231060
     caaagccaag agatctcaag tgagatttag tgtgtatgtt gtgtctaatg aagtcacaca    231120
     ctcagagcaa gtgaataggg aaaagttcat tatctacact gtagaggtgt ctgctgagtg    231180
     cagggcaggt ctcccaggaa aatctaaaat ggcttgaaag aaaaccaaag gagactggct    231240
     cagggttttt ataatggttt agtggtgggg gcaaagtgaa gcttcccact cacagatggg    231300
     ggtttatagg gtttgaaact cccactggca tcaaatgaag aagctcctgt gagtctgaac    231360
     tagattcatc ttgtgtggca aaaaaggaga cgatggagga gtgagcctta agtaatcagc    231420
     agtcatgcac caaaagatag agtgacaact tattccatgc agcaggaata aaaataatga    231480
     ataagaaaga agacaagggt tcagtatggg tggacaacac ccagatctgc agaaatgaga    231540
     tgactttaga aatgtaagca aagaataatg agaaaaataa gaagtggatg gaaaattaaa    231600
     caggggcctg gtctaatgtc ttgggtagaa gcttttcaca atcaaggact atcagcttat    231660
     tctgcaggtc ttaggtcagc catctgctta aaaacatcag aaacgccaga gggtctatga    231720
     ggatgctcag tttaacatct cctatttgag tagctttaca gttgtgtgga attcttaatt    231780
     gattattttt tgtttgtttt tagggacagg ctctcacact gttccacagc ttaaagtaca    231840
     gttgtgtgat catagctcac cttaaatttg agctcctgac tcacacaacc tttccatctt    231900
     agctttctga gtatctacaa ctagatgggc acaccacccc taccctcctt attttttaaa    231960
     cattttttca tagaaatagc atctctttat gttgctcagg ttggcttcga ttgcctggtc    232020
     tcataggatt tccctcactt tgcttctgga agtggtgtga ttatagggaa gatccactgc    232080
     atctgacctg aatttattct ttagttgtaa aatatgaagc caataattaa ctgcctgaat    232140
     gttttctgca gtgagttagt taaaagcatc tgataaggtt ccttcctatg tgattcacga    232200
     gcagtatttt ccgctgatgt tccttccagt ttccttgctg aagatcacag ggaattgtgg    232260
     aaaatatgta gtaaaaaggc agccttaaac tcttcattgt tagagtggat atcacacagg    232320
     aatcactttc agtattttgt aacacatgca tgcaatagag caaacatgat ctctgggggt    232380
     aaagtctata aacgtatggg ccttttagct accaagtcat agggtaataa ctgatgtacc    232440
     ctgaggactg gaccatgatt ccatagtgct agtggaagaa cccttgacca agggagtttt    232500
     acattttatt aaaatatgta gagatgccac tttttaaaca ttcccagaag gttgtgagtg    232560
     ggattgactc tgtctcgtat gaacaaagac aatgccctcc atgattagat aatattataa    232620
     actagattga gctacagtgt ttgctgtatt gaatcactgt atattttatt ttttagctcc    232680
     atgttagttt tttgtctgcg tgttagcgtt ggctttaaaa tgatattaat cagctctgta    232740
     gtaagtagaa attcatctga gagtttcttt ccttgttttc cagtttgata ggatttccag    232800
     aatacataag aaccctctct gtttgtaaaa atattccagg ccaggccagt ggctcatgcc    232860
     tgtaaaccca gcactttggg aggccgaggt gggggggatc atttcaggtc aggagtttga    232920
     gaccagcctg gccaacatgg tgaaacctcc tttctactaa aaatacaaaa attagccggg    232980
     catggtggtg cttgaggatt gcttgaaccc tggaggctga tgttgcagtg agccgaaatt    233040
     gtgccactgc actccagcct gagagacaca gcgagagtcc atctcaaaat aaaagaaaac    233100
     aaaacattcc aaagttatgc acaatctaga aacatatgta cttaattctc atttttaatt    233160
     tattaaaaag ctctaataag ttcaatgttt tctgccttct cagttgattt cacaacacat    233220
     ataataatat atcctaaata aaagtttgtt ctcttaatac aaattactag ttaataaccc    233280
     ttactttatt attgaggtat tatccatcag tgttcatcaa tgctctcaat gggactctta    233340
     catagagaat atacaaaata ttttcctgat catgacataa aacagatgtg aacacattct    233400
     tagtattcag ccatgtctcc tatcatgtta taaaccacat gctaactttg actttattgg    233460
     aacttgttct aatttcaaac tagttatttt ttatcttcat gcagctggat tattatgtgt    233520
     ggttatttat tccgagagtg ataaagacaa cattaacaat tttcactgca ggcatgtcta    233580
     ggcaaccccc tgtgcacaat gaccttggtg cattggagat tctatgggga ctcttcccta    233640
     cctgcctagg agagttctct gccttctacc tctatcattt tcctctttga agaaatacat    233700
     ctaactgtcg ttagaataga gacaaagaca aatcttaact gcttccagct gacgggggat    233760
     gctgtttggg gaagatctct cttggaggcc tgtctaaggg accccaggaa aagggagcca    233820
     ttatcccagg cttcagttgc atgagcattt ggagtttgat ggtctgaaaa cgagaaaagg    233880
     caaatcagat tattagaaga catgtatcca aacctaacaa ggtggtcaag gtggtaagga    233940
     cagtttgaaa gaaaattcca aggctgctga catgcccaga taactgcggc tgtagttatg    234000
     cctgctaagg tttgggcgca tgaggcttgg cttttgtcag ctccctggga tttattttcc    234060
     caaacaaaga aacctccagg ttaggggcac cctattcatt cccatcacct ggcatgattt    234120
     aaaggataat tgcttagaat taaaatattg atacagattt ttttatattc cccatcgctt    234180
     tttgtttctt ctgggctgta gccagagatc attgattggc gctcaggaat aagcagtcag    234240
     tctaaaatgc aggcaaatac ttaaacaact gaagagatta gaatttaaag atgatcgtat    234300
     gatatgtttt gaaatacaat ttttctcttt ccagttctgg tttttgtcag aatcaagtaa    234360
     ttgtaagact gagttgtttg caaaataaac tttagtctta aacttggcct gattatttgc    234420
     ataaagtgca gcaagaatat taataataat tctgtaggaa aagcctgcaa gcaccaggag    234480
     tttcacagtc taacactatg agcacgtgca tcctcacgca actcgctgaa tatttccaag    234540
     ccagcctgtt cctatcttaa atgccatcca gttgtatctg ccccaggtac actaatatat    234600
     gggtcctgct tctctgcagc ctcctctctc ctcagatttc aggttttgtt tattgtttgt    234660
     tttctctctg atataaactc aaatatgttg aaggtttttt tgtgtgtggt tgttcaggtt    234720
     tgttgttaat gaggtcagaa taagataata ctttactctt tttatttatt tatttattta    234780
     ttttttgaga cggagtctcg ctctgtcacc caggctggag tgcagtggcc ctatctcagc    234840
     tcactgcaag ctccgcctcc cgggttcacg ccattctcct gcctcagcct ccagagtagc    234900
     tgggactaca ggcgcccgcc accatggccg gctaattttt tgtagtttta gtagagacgg    234960
     ggtttcaccg tgttagccag gatggtctcg atctcctgac cttgtgatcc gcccgcctcg    235020
     gcctcccaaa gtgctgggat tacaggcgtg agcccccgcg cccggcccat actttgctcc    235080
     gttttatatt cccatgcttt agtagctgct tttctctatc aaatccatta actgagagaa    235140
     caaatcacat ttagttacag gtgaacaatt aaatagtttg gcatatattt atgtactgga    235200
     acataacgca gcttgaaatc aaggcatgcc tcactcatat aaacaacatg gctaaattct    235260
     caagtaattc tgtttagtga aagaaactaa ggagttaaga gtaaatttta tatgatacat    235320
     ttgtaggaat tttagaagat gccattattg taaattaaca cggagaagat ttgagtttgt    235380
     ctgacaatac gctgttggga gtaatgtgga tgtgagttga atttcagaga aataaaagaa    235440
     agatttaggg attaatttaa ttattcaaaa cttgattgaa gtgctgagca aatggctgca    235500
     aacataggtc aacatttttc aaatccttca ctataaattt gaattaatta cttattaatt    235560
     acacttgaat aaagcaataa cgaagaaacc aataaaataa catttgacta aaatggagca    235620
     ataaacagat cgatgttaac acaaggaata tgactgactt atgaaaacat gcacatgaac    235680
     catggttcac tctacgtatt ttggtaaatt acagaaagtt gtcataacag atggggaatc    235740
     ctgcagactt cactaggcat ggtccacgct gccctggagt tgtctcaggg gagctgcctc    235800
     ctccagtggt tagagcacag gcccaggtaa taggactaat ttttttagat gtgtaatttt    235860
     agacacactg cacaactgct gtgttctctg tgtaaattat ctcctgtaaa atgtaacatt    235920
     gaaacctgca ttaaacatat tgtataaata tgtaagaata aaataagatt atgagagcta    235980
     aatattaatc aaggcacaag cacataatat aaaattatat tttcctgaat gatacaatta    236040
     ttccaatctg tcccaggaca cttcatctgc cctgagccca gcctctcctc agatgtccca    236100
     ccacagagct tgctatatag tgggggacat gcgaataggg ccctccctct gctgatgaaa    236160
     accagcccag ctgaccctgc agctctggga gaggagccca gcactggaag tcggcggtgt    236220
     ttccattccg tgatcagcac tgaacacaga ggactcacca tggagtttgg gctgagctgg    236280
     gttttccttg ttgctatttt aagaggtgat tcatagataa atagagatgt tgagtgggag    236340
     tggacatgag tgagagaaac agtggatgtg tgtggcagtt tctgaccttg gtgtctttgt    236400
     gtttgcaggt gtccagtgtg aggtgcagct ggtggagtct ggggaaggct tggtccagcc    236460
     tggggggtcc ctgagactct cctgtgcagc ctctggattc accttcagta gctctgctat    236520
     gcactgggtc cgccaggctc caagaaaggg tttgtagtgg gtctcagtta ttagtacaag    236580
     tggtgatacc gtactctaca cagactctgt gaagggccga ttcaccatct ccagagacaa    236640
     tgcccagaat tcactgtatc tgcaaatgaa cagcctgaga gccgacgaca tggctgtgta    236700
     ttactgtgtg aaagacgcag tgagaagtca gtgtgagccc agacacaaac ctccctgcag    236760
     ggtacctggg acaaccaggg aaagcctggg acactgtata ctgggctgtc cccaggggca    236820
     agtccaggtg gtataagcct aggtttcctg tcatggtcta gggttgcctt tttagcaact    236880
     taccccagag aacttctcta gatttccaat tctgtaataa catttgatgt cgtctctggc    236940
     tgcaaaatgt cccctcaact ttgtatcttt tttttttttt ttgtaacagg aggacacatc    237000
     ctcaccctgc agaagcctga gtgtcacatt gggggcagaa atgatctgcc ttgatctcac    237060
     tgatcactgt cccgaggaac ataccccaca ggggaccctg atgactccag taatgtctct    237120
     gcctcaaaac cattgaagag tccttccttc cattagagtt gaccacagca cctgggcttc    237180
     agcacaagcc ataccacaga cgtcacaaag cagcagattg acacctgatc caggtgcatt    237240
     ttctcacctt tagaggctga gagaggggtg tattctcaag gtcaacacac tctttgtgga    237300
     tttttacaga gaacacctac ttttacttta tttcatttga agataaatga acaaatgtgc    237360
     agctgcaaat gattgtaatt ttcacattta ttccaattcc aatatttctg aattctgcat    237420
     gatgtcctgg cacagggttg tgttttctac aggtattttt catcaaacca agaaaatgca    237480
     ttttccactt tccgtggttt ttctccatgt gcagaggcct tgagtagagc acgtctgact    237540
     gtgtttgttt tcacgctgct gataaagaga ccttgagtag atcacgtctg actgtgtttg    237600
     tctgttttca cgtggctgat aaagacatac cagagactgg gaaacttaca acagaaagag    237660
     ttttattgga cttacagttc cacgtggctg gaaaggcctc acaatcatgg tggaaggtga    237720
     aaggcacatc tgacatggcg gcagacaaga gaagagagct tgtgctgggt ctcccctttt    237780
     taaaaccatc agatctcatg agactcattc actattaaga gaacagtgca ggagagacac    237840
     aacccccata actcagtcac ctcccactgg tccctcccac aacattgaga attatgggag    237900
     ctacacatca aaatgagatt tgagtgagga cacatccgac ccatatcatt ccaccccagg    237960
     ttccctccca aatctcatgt cctcacattt caaaaccaat catgccttcc caacagctcc    238020
     ccaaaggcta aactcatttc atcattaact cagaagtcca cagtacaaca tttcatctga    238080
     ggcaaggcaa gtcccttccc cttatgagcc tgtaaattca gaagcaagtt acaagcattg    238140
     ggtaaacaca cccattacaa atgacagaaa ttgcccataa caaaaaggcc ccatgcaagt    238200
     ccaaaatcca gcagggcagt caaatcatag agctccacaa tgatcacctg taactcccct    238260
     tctcacatcc gggacatgtt gatgcaatag gtgagttccg atggtcttgg gcagctccac    238320
     ccctgtggct ttgcagggta cagcctccct cctggctgct ttcacaggct ggtgttcagt    238380
     gtctgtggat tttccaggca caaagtgcaa actgtcagtg gatctaccat tccagggtct    238440
     ggaggatagc agccctcttc tcacagctcc actaggcagt gccccagtaa ggactctgtg    238500
     tgggagctct ggccctatat ttcctttcct ccttgcccta gcagaggttc tccatgaatg    238560
     atccaccact gcagccaact tatgactgga catccaggtg ttttcataca tcttctgaaa    238620
     tctaggcaga ggttctcaaa caccaattct tagcactctg tcgataatgg ttcatttttt    238680
     tggcctgttc attactggta tttttcaaag gaatctcact tgaatcttta ctcttttgca    238740
     ttttgtctcc atgacaatgt tgggaagttt tacctccacc atcataacat gatctagtga    238800
     tctcacacat ttgtggcaaa caatacctac aaattcagaa gctctttgct tttctttcca    238860
     tgaaatataa ttctttctgt tctgtgtata agcatatctt agcaactctg tgcacaccca    238920
     catagatgtc cacaagccta tgaattattc tctgtaaata aaaatttata tcaatttccc    238980
     tcaatgttca taattctcct gagggtgagg aagctccttc tcgatctgtt caaacaaaat    239040
     gcccagaaac catctggtag gtaaggagtt cacctggctc tggtgtgggg tctgtctctt    239100
     tccctctgtt gtcacacagg tcagcccagt tgttcaggtc ctaagaagaa agcccaggtt    239160
     tgtcctgatt ttaaaacaca tcaaacttct gatgactctc ctgttaccca catccatgga    239220
     gatagattat ttattatata attcaccaaa ctaatgtcaa atgcccaagt tgcaatacca    239280
     cacatcctag ggtatgttca tgcaattcaa tggaggagaa agtctttcag agacagatgg    239340
     atctgaaatg ataaatatgt gggtaaggac tctgggcttg agtatcattg tccagccatg    239400
     tttcacaagt gtgtcctgtc agggaaggac aagagttcct tgtgttctca gagggaaggg    239460
     gtcacagagt tcctctctgg ttcccaggaa agataatcgc actaatcttc atgatcttca    239520
     tgagactatc ctccagtgct gacctgttat agagtttttg tctgaagttc tcactgcaat    239580
     ccccaatcta catattttca atcagaagtg tttagaggcc aggacatatc ttcacggtca    239640
     cacattgaga aggatgtaga tatgtcccac taccttctcc tgagatctca gacagaatcc    239700
     cagatttcaa aaggacacag aaggacagct ctcaggtgct tttaaaaaat gacccacttc    239760
     cagggacagg gagcttccct ataaccatgg tggatgttct gaactacaat aaacattgga    239820
     tggatccagg attgtttgaa gtcactgtca ttattacatt cagctgctgt ttcaatgtgt    239880
     ctgaagtagt aaatgacaat ttagatgaca atttatatga atcttcaagg gtagaacaat    239940
     attgaccata ttccaaaatc tgtccttgat ccatgatcac actcatctcc cagaccaggt    240000
     ccttcagcac gtctctttac ctgaaagaag aggactctgg gcttggagag gggagacccc    240060
     aagaagacaa ctgagttctc aaagggcaca gccagcatcc tactcccagg gcgagcccaa    240120
     aagactgggg cctccctcct cctttttcac ctctccgtac aaaggcacca cccacatgca    240180
     aatccttact taagcaccca caggaaacca ccacacattt ccttaaattc aggttccagc    240240
     tcacatggga aatactttct gagagtcctg gacctcctgt gcaagaacat gaaacacctg    240300
     tggttcttcc tcctcctggt ggcagctccc agatgtgagt gtctcaggga tccagacatg    240360
     ggggtatggg aggtgcctct gatcccaggg ctcactgtgg gtctctctgt tcacaggggt    240420
     cctgtcccag gtgcagctgc aggagtcggg cccaggactg gtgaagcctt cggagaccct    240480
     gtccctcacc tgcactgtct ctggtggctc cgtcagcagt ggtagttact actggagctg    240540
     gatccggcag cccccaggga agggactgga gtggattggg tatatctatt acagtgggag    240600
     caccaactac aacccctccc tcaagagtcg agtcaccata tcagtagaca cgtccaagaa    240660
     ccagttctcc ctgaagctga gctctgtgac cgctgcggac acggccgtgt attactgtgc    240720
     gagagacaca gtgaggggag gtgagtgtga gcccagacac aaacctccct gcatggacgc    240780
     ggaggggacc ggcgcaggtg ctgctcagga ccagcaggtg gcgcgcgggg cccccagagc    240840
     atgaggccgg gtcaggagca ggtgcaggga gggcggggct tcctcatctg ctcagtggtc    240900
     tccgtcctcg ccagcacctc gctgtcacca gggctcctct ttctttatta tctgtggttc    240960
     tgcttcctca cattcttgtg ccaggaaaga aacgaggaag acaaattttc gtctatagtt    241020
     gaagcttcac caattactag gaacttgcct acaagttcct gcatgaccca ttataactta    241080
     tcgattaaaa aatatatatt ctaatgcttc tcaccatctc ttgatttgta tcatcaactg    241140
     aattgtaccc tctttgaaat tcatatgatg aaaccttaaa ttcaatggat ctatattgga    241200
     attttaatga aataattaag gttaaatgtg gtcataattg taagacccta atgcaataga    241260
     cgtgttgtct ttataagaag aggaagagac accagagacc tctcactttt cacgtgcagg    241320
     cagagaagag gccatgtgga gacatagtgc actagaaggt ggcccagtgc aagccaggaa    241380
     gaagccgcgc caagaaccag ccctgccagc acactaatct tcaacattca gactgcagaa    241440
     ttttaagaaa atcaatattt gttgtttaag ccacccactc ctgttgtctt cttatgaaga    241500
     tccagacaga ctaataccac ataactctgt tagtgctgtc ccctggatgg agaattagcc    241560
     tcctgaggct gggcacatct ctcagatttc cacataaaac aggtaaaaaa tagtagttct    241620
     gatataaaaa cttgtcatgt ccctgttggc caatttctgg gcaaggtctt ttaaataagc    241680
     caagtttgcg gggaaatgga gaccatatgt ttgtgggact ctaaccgtgg aatctactgc    241740
     attgctctag cagaatcaaa aattgaacac cagtgagctc tttattctca ttaaaatgtg    241800
     tgtctttgga aatgttaaca ttctgtccag aatctgtgct cacaaacaac tgtgtttctt    241860
     agtgcactct tggcctggtg gagccctcgc agaccctctc cctcacctgt gctgtctctg    241920
     gattttccat cacaaccagt gtttcctgct ggagctggat ccacgagtcc acatgggaag    241980
     gactggagtg gaccaggcgc acacgtcatg aagggagcaa aaattcccac ccactcctta    242040
     tgaatccagt caccatctcc aaattcgggt ccaaaaaaca cttgttttta cagtggagct    242100
     atgtgagcaa caagctcaca gccatgtttt aaagaagaga cagagtgagg ggaccacggt    242160
     gcgagctcac acccaaacct tcctggaggg gtgcacagga cagcaggagt gccgatgatg    242220
     gaagggggtg gtctggattc cagggcacac gcaagaccat tgtagaggca ctcaggtcac    242280
     ccgggggctc tcaggaacca tcgaggaaaa tcaggacacc agaagagact cggtacagca    242340
     gggggctcag gaccattgtg gggactcaga aacagcaggt tctaggctca gcctcagggc    242400
     aggtgcagat ggggtgaaaa ggggctggat gaggggtttt gtgtcaccat catatttcac    242460
     cactagacac cctccactac atgtgttcta ctgcatatga gtgtatgatt agaaaatcat    242520
     acttctataa atatataacc atacgaaggt gtgtcaagtt ttcctcttca acttctatcg    242580
     gccttgttcg taaggactaa ttccctgtaa ttacttgagg acctcataaa ttgtggtcag    242640
     ttttgtaggt ttcctctctt tccttgtctc cctttcccct tattctctct cacacaaatg    242700
     gacataatcc ccactccaca cgcaaagaaa tctataactt tcattacctg atgtattgaa    242760
     taaaattgat taatgtgcag ctttcccatc tttgttgttg ttcatgctgc tgtaagtata    242820
     agaaccatga gattctcagc tgtgtacttc tccaagctga gtggcagctt tgcttatcat    242880
     actgagatgg ccaacaatcc aaatgtcaat catcagctta actggtaaac aaattgtgga    242940
     aaagtcattc attggcatac tactgactgt gaccacaata tactaatgtt ggatacgctc    243000
     aacagtatgg ttaaattctc atatggtgag taaaatgagc caaacaaata aaagtacaca    243060
     catatgatac cacttttata cattctccca tccaagtact aaccaggccc gaccctgctt    243120
     agcttccgag atcagacgag atcgggcgcg ttcagggtgg tatggccgta gacacacttt    243180
     tatacattct atagaataaa aactaaagtt acataaagaa tcagtagttc actgtgaata    243240
     ttatagaagg gaaagtgtag acaggaggaa ttacagaaaa gagaaaattt taagcataat    243300
     tgacttgtta tctatattga taataatgat gtctatgata atatttgtac aattgaacac    243360
     ttcatgtgaa gatctttttt tttagtttaa catcattaaa gatagtacta actgtaacgt    243420
     gtgtaatttg gtagaaacgg agtcagacag agataaaata cataaaaagt cagagacacc    243480
     tgaatattca catgagtgag cctgcacttc tctatgtttt tatgaaaaca ctacaataca    243540
     gcaaaataat gacatcattt tatgacgtga agggggttta ttaaaccact ccaggcacgt    243600
     tgtattagtt tgttttccca ctgctacaaa gaactacctg agtttgggta acttataaag    243660
     aaaataggtt attgactcat agttctacat ggctggaaag gcctcaggaa acttgcaatc    243720
     aaggcagagt gtgaagggga agcaagagac atctcacatg gcagcaggag aggtggggag    243780
     aaccaccaca cactttaaat tatcagatct cctgagacct caatctgaga cctcaatcat    243840
     gaaaacagca tggaggaacc accccatgag cccatcccct ctcaccaggt ccctccctgg    243900
     acgtgtggca attccaattc gagatcagat ttgggtgggg acacagaacc aaaccatatc    243960
     acatgtccag ttctgtccca gagttggttc aggaatcaga tgtgtcctgt taacaggagc    244020
     tgtgacacca agctcccagc accacttgta gttgacgtca tgcaaaggcc aagatatctc    244080
     aactaagatt tagtgtggat gtgatgttgg atggtgacac accctcagac caagtggata    244140
     tggaagggat aattatctct attctagacg tgtctgctga gagcagggca gttctctcag    244200
     gaaagtgcaa aatggcttga tagaacaggg aaagagaggg cctcagggtt tggtggtggg    244260
     ggcagattga gtctttccac tcacagaaag gggtttatag ggtgggaaac tcccactggc    244320
     ttcaaataag gaagctcctg tgattctgga ccgatattca ccttgtgtgt aaaaaaggag    244380
     gtaatggagg aatgagcctt aagctatcag cagtcacaca ccaaaataca gtgtgacaag    244440
     ttattctatg tagcaactat aaaaataatg aataaaaaac gagataaggg ttcaatatga    244500
     tgggcaacac tcaggtctac agaaaatgag atgagtgttt ataagtcaaa gcaaataata    244560
     atgagaagca ggaggagggt gtgcagaatt agacaggggt ccaggtccag tgacttgggt    244620
     ggaaaccttt tgctattaag gattatcagc ttagtctgaa ggtcttaggt cagctatctg    244680
     tttaaagaca tcagaaacac cagagaatct ttgaggatgc tcagattaac atctcctatt    244740
     ttagtagcct tgcagttgtg tgaaaacctt ttttatctta tttcatttta ttatttattt    244800
     atttatctgt tgagatggaa tttcgctctt gttgcccagg ctggagcgaa atggcacgat    244860
     cttgactcac cgcctcttcc acctcccaga ttcaagcgat tctcctgcct cagcctccca    244920
     gtagctgata ttacaggcat gcaccaccag gcccagctaa ttttgtattt ttagtagaga    244980
     tggggtttct ccacattggt caggctggtc tcaaactctc aacctccggt gacccgccca    245040
     ccttggcctc ccgaagtgct gggattacag gtgtgagcca ctgctccggg ccgaaaatct    245100
     tattcattct tttttgtttg tttttagaga cagagtctct ctctgttgca cgttgaaagt    245160
     ggagtggtgt gataacagct cactgtaaat ttgaactctt ggctcacaga gtatgttcct    245220
     atgtatcgtg caggtgtgtc tgtgtgtgtt tacccggctt ggggtcctct aatttttgga    245280
     tctgtagtta ggtgtttcac attcttaaga tatttttctt aagaaatata tcttaagata    245340
     taagtatatc ttaagatatc attaattctt aagatatttt ctctgtttct aatttctatt    245400
     tttgaaattt gaaatatatc tacactatac tttttgatgt tagttcttaa atttcttcac    245460
     tctttttccc tttgaaattc actttgtgta atttccagta acggagttga agtccctggt    245520
     ttcatttaag agctgagcga attctactgc tgagcgtgcc caaggtatat tcaactttat    245580
     actgcatcac ttttgataat tttatgaatt tccatttgat tctttcttag tatttctctc    245640
     tgtcagtcac ctatctagtc ttgcactgtg tccacatttt ctttcagata cttttaacaa    245700
     attcaattac tttaaatatc ctacttaata agatagttct agtattgtgt catttacaaa    245760
     tatcaattct gaggattttt ccattatgac tttggtgttt tttataactt atatgacttt    245820
     gatgaatgtt ataatttttg ttgataacca gccatatttg gacccttgat attgacgtat    245880
     tattttattt tgtgcatttc tgcctgtatt tgaccacacc ctatctgtgc tgggccttga    245940
     ttgtggagat gtctgtgcat ctcttcagag ctacatttga catttacttt tgcaacggct    246000
     gtagcagttg aagtgtgttc ttctatgtcc actggagact tcagatactc tagtgatacc    246060
     ttgttttcat gcctgcttgg ctttgtctat tcacctcatt ccattctaca gagaatctcc    246120
     ttcacattcc tagggtggat taaagtgtta tatttaactg ccaattgtga aattggcgga    246180
     aggcattaga ctaaaggaag aaactgacct ctcgttgggc cataattcta gaaagccgtt    246240
     gtgatcctga gtctgagtgt gaccttccaa gttttcccga ccctcctgca agtgagatac    246300
     tggtctgtgt gttcttgcct cttcccctgg ggtagagtcc tcctgttttc cccagttgtt    246360
     ccctcccaca gctctcacaa tctctgttgg tgtccccatc ttccagatct gctgccatga    246420
     cctgcagatt aaggctctga ttccataaga aactgagggg agctgcttct caatagatct    246480
     ttgatgggga cctctgttcc catatcagtt catgaggggc tgctccagtg ccctaggatg    246540
     ctgattttca tggcttgctc ctgcagggta atatctgagt ttcatagtgg gaatcagaga    246600
     gttgggtctg gatgcatttc agaagtgtgg gctctcattc tctcccagac agtcactttg    246660
     ggaaggatag attcttgtga ctgtaaaggt tcttcaagag aatagcacaa ctcttcagta    246720
     tgttgtccct tagaatttct cactacaaca cgcttaacac actcgacttc aagcaatgca    246780
     atgtgtattt gtgctccatc ttgtaatggc ctacgttgaa tacgacagac tgtgcctcag    246840
     gtaatttcat atcttgactt tattactctg tactagaact tgcctctccc tagatttcaa    246900
     ttttttttgt tttaaacctt cagttatctg aagcatttaa taaaatttgc gaacttccat    246960
     cttttctgtt tatctgttat tgctgatgtt attgttttaa aaataaatat atatttctca    247020
     ttcatgtaca tttttaagct gagcagcata tttttaagta aaacctggaa taataaaaga    247080
     atccaaacat ttttcagctg ccccaaaaaa accaaattat ggtaaatgtg ttcactggaa    247140
     cactactcat cacttataat aaatatattt ctggtacaca gagcaacaaa gaaaaatatc    247200
     taagtgttta tgctgagtaa aatacgccag acaaataaga atatgtacca tattactcca    247260
     tttatgcaat ttcttttaag tgaaaaagaa tctaaagcaa tatccagaag atcagtagtt    247320
     acctggaaaa agggtagacc aaaggaaggg gaaaggagga aacttacaga agaacaagag    247380
     aaaatgttga ggggagttca cttgtccagc ttggaaatga tgggttacat catgttgatc    247440
     aattgcacac tttaaatatg tgaagtctat tatctgccaa ttaacactcg caaaatttat    247500
     tgcaagcaga caaatgaaaa attagacaaa gaaaggatgg tataaagata gaaaatatat    247560
     attaaatgtc agaaatgtct gagaatttaa ctcctgaccc tagttccgtc cttattttta    247620
     ggtgaatggt agcgtgcacc aaaatcacac acattctcag tacaggaagt gggttccaca    247680
     aagcacacga ggtatgtcca attcttacca agatttggtt cagggagtaa cagtgatgag    247740
     gaatcacagg cccagatacc ggggctcact catctcagac atgacctcgt ggacacacac    247800
     ttagcccctc ctccatgtgt aggttgactt ccacatatgt aaatggagaa accattgact    247860
     cctacagaac ataatttaca gaaatataca aaagataaaa tagtgcaaat acttatcaca    247920
     acaaaatttc ctaataagac agtgtatttt ccaaataccg taattgtcac ccaactcctg    247980
     tggggccgtg tcattttatc tggggtctgc cgtctcctca ggattcccac cccagagctc    248040
     tctatgtagt aggagacaag caaatagggc cctccctctg ctgctgaaaa tcagccaaat    248100
     cctgaccctg cagctctggg agaggagccc ccgccccggg attcccagct gtctccactt    248160
     ggtcatgaac actgaacaca gaagacacac catggagtct gggctgagct ggattttcct    248220
     tgttgcagtt ttaaaaggtg atttatggag aatgagacac actgagtgtg actggacata    248280
     agtgagagaa acagtggatt tgtgtggcag tttctgacca gggtgtctcc gtgtttgcag    248340
     gtgtccagtg tgaggtgcag ctggtggagt ctgggggagg cttagtaaag actggagggg    248400
     tctctgagac tctcctgtgc agcctctgga ttcaccttca gtagctctgc tatgcactgg    248460
     gtccaccagg ctccaggaaa gggtttggag tgggtctcag ttattagtac aagtggtgat    248520
     accgtactct acacagactc tgtgaagggc tgattcacca tctctagaga caatgcccag    248580
     aattcactgt ctctgcaaat gaacagcctg agagccgagg gcacagttgt gtactactgt    248640
     gtgaaagacg cagtgagaag tcagtgtgag cccagacaca aacctcctgc agggtacctg    248700
     ggacaatcag ggaaagcctg ggacactgta tactgggctg tccccagggg caagtccagg    248760
     tgatataagc ctgggtttcc tgtcatgatc tagggttgca ttgttagcaa attaccccag    248820
     ggaccatctc tagatttcca attctgtaat aacatttgat gtcgtctctg actgcacaat    248880
     gtcccctcaa ctttgtatct tttttttttt tgtaacagga ggacacatcc tcaccctgca    248940
     gaagcctgag tgtcactttg ggggcagaaa tgacctgcct tgatcacatt gatcactgtc    249000
     ctgaggaaaa taccccacag gggaccccga tgactccagc aaaggctctg cctcaaaacc    249060
     attgaagagt ccttcctttc attagaattg accacagcac ctgggcttca gcacaagcca    249120
     taccacagac gtcacaaagc agcagcttga cacctgatcc aggtgcattt tctcaccttt    249180
     agaagctgag agaggggtgt attctcaagg tcaacacact ctttgtgggt ttttacacag    249240
     aaaacctgct tttactttat ttcatttgaa gataaatgaa caaatgtgca tctataaatg    249300
     attgtaattt tcacatttat tccaaatcca atatttctga attctgcata atgtcctggc    249360
     acaaggttgt gttttctata ggtatttttc aacagaccaa gaaaatgcat tttccaattt    249420
     ccctgttttt cctccatgtg cagagacctt gagtagagca cgtctgactg tgtttgtctg    249480
     ttttcacgct gctgatgaag acataccaga gacgaggaaa cttacaatag aaagagtttt    249540
     attggactta cagttccacg tggctggaaa ggtctcacaa tcatggtgga aggtgaaagg    249600
     cacatctgac atggcggcag acaagagaag agagcttgtg ctgggtctcc cctttttaaa    249660
     accatcagat ctcatgagac tcattcacta ttaagagaac agtgcaggag agacgcaacc    249720
     cccataactc agtcacctcc cactggtccc tcccacaaca ttgagaatta tgggagttac    249780
     acatcaaagt gagatttgag tgaggacaca tccgacccat atcattccac cccaggttcc    249840
     ctcccaaatc tcacgtcctc acatttcaaa accaatcatg ccttcccaac agctccccaa    249900
     aggctaaact catttcatca ttaacacaga agtccacagt acaacatttc acctgagaca    249960
     aggcaagtcc tttccactta taagcctgta aattcaaaag caagttacag gcattgggta    250020
     aacacaccca ttggaaatga cagaaattgc ccataacaaa aagaccccat gcaagtccaa    250080
     aatccagcag ggcagtcaaa tcataaagct ccacaatgat cacctgtaac tccccttctc    250140
     acatccggga catgttgatg caacaggtga gttcccatgg tcttgggcag ctccacccct    250200
     gtggctttgc agggtacagc ctccctcctg gctgctttca caggctggtg ttcagtgtct    250260
     gtggattttc caggcacaaa gtgcaaactg tcagtggatc taccattcca gggtctggag    250320
     gatagcagcc ctcttctcac agctccacta ggcagtgccc cagtagggac tctgtgtggg    250380
     agctctggcc ctatatttcc tttcctcatt gccctagcag aggttcccca tgaatgagcc    250440
     accactgcag ccaacttgtg actggacatc caggtgtttt catacacctt ctgaaatcta    250500
     ggcagaggtt ctcaaacccc aattcttagc actctttcca taatggttca tttttttggc    250560
     ctgttcatta ctggtatttt tcaaaggaat ctcacttgaa tctttactct tttgcaattt    250620
     gtctccatga caatgttggg aagttttatc tccaccatca taacatgatc tagtgatctc    250680
     acacatttgt ggcaaacaat acctacaaat tcagaagctc tttgcttttc tttccatgaa    250740
     atataattct ttctgttctg tgtataagca tatcttagca accctgcaca cacccacata    250800
     gatgtccaca agcctatgaa ttattctctg taaataaaaa cttatatcaa tttccctcaa    250860
     tgttcataat tctcctgagt gtgaggaagc tccttctcga tctgttcaaa caaaatgccc    250920
     agagaccatc cggtaggtaa ggagttcacc tggctctggt gtggggtctg tctctttccc    250980
     tctgttgtcc cacaggtcag cccagttgtt cacgtcctaa caagaaagcc caggtttgtt    251040
     ctgattttaa aacacttcaa acttctgatg actctcctgt tacccacatc catggagata    251100
     gattatttat tatataattc accaaactaa tgtcaaatgt ccaagttgca ataccacaca    251160
     tcctagggta tgttcatgca attcaatgga ggagaaagtc tttcagagac agatggatct    251220
     gaaatgataa atatgtgggt aaggactctg gacttgagtg tcattgtcca gccatgtttc    251280
     acaagtgtgt cctgtcaggg aaggatcaga gttccttgtg ctctcagagg gaaggggtca    251340
     cagagttcct ctctggttcc caggaaaggt aatcgcacta atcttcatga tcttcatgag    251400
     actatcctcc agtgctgacc tgttatagag tttttgtctg aagttctcac tgcaatcccc    251460
     aatctacata ttttcaatca gaagtgttta gaggccagga cacatcttca aggtcacaca    251520
     ttgagaagga tgtagatatg tcccactacc ttctcctgag atctcagaca gaatcccaga    251580
     tttcaaaagg acacagaagg acagctctca ggtgctttta aaaaatgacc cacttccagg    251640
     gacagggagc ttccctataa ccatggtgga tgttctgaac tacaataaac attggatgga    251700
     tccaggattg tttgaagtca ctgtcattat tacattcagc tgctgtttca atgtgtctga    251760
     agtagtaaat gacaatttag atgacaattt atatgaatct tcaagggtag aacaatattg    251820
     accatattcc aaaatctgtc cttgatccat gatcacactc atctcccaga ccaggtcctt    251880
     cagcacgtct ctttacctga aagaagagga ctctgggctt ggagagggga gaccccaaga    251940
     agacaactga gttctcaaag ggcacagcca gcatcctact cccagggcga gcccaaaaga    252000
     ctggggcctc cctcctcctt tttcacctct ccatacaaag gcaccaccca catgcaaatc    252060
     ctcacttaag cacccacagg aaaccaccac acatttcctt aaattcaggt tccagctcac    252120
     atgggaaata ctttctgaga gtcctggacc tcctgtgcaa gaacatgaaa catctgtggt    252180
     tcttccttct cctggtggca gctcccagat gtgagtatct cagggatcca gacatgggga    252240
     tatgggaggt gcctctgatc ccagggctca ctgtgggtct ctctgttcac aggggtcctg    252300
     tcccaggtgc agctgcagga gtcgggccca ggactggtga agccttcgga gaccctgtcc    252360
     ctcacctgca ctgtctctgg tggctccatc agtagttact actggagctg gatccggcag    252420
     cccccaggga agggactgga gtggattggg tatatctatt acagtgggag caccaactac    252480
     aacccctccc tcaagagtcg agtcaccata tcagtagaca cgtccaagaa ccagttctcc    252540
     ctgaagctga gctctgtgac cgctgcggac acggccgtgt attactgtgc gagagacaca    252600
     gtgaggggag gtgagtgtga gcccagacaa aaacctccgt gcagggaggc ggaggggacc    252660
     ggcgcaggtg ctgctcagcg ccagcagggg gcgcgcgggg cccacagagc aggaggcccg    252720
     gtcaggagca ggtgcaggga gggcggggct tcctcatctg ctcagtggtc tccctcctcg    252780
     ccagcacctc agctgtcccc aggggtcctc tttctttatt atctgtggtt ctgcttcctc    252840
     acattcttgt gccaagaaag aaatgaggaa gacaaatttt cgtctgtagt tgaagtttca    252900
     ccaattacta ggaactttcc tagaagttcc tgcatggccc attatagctt actgattaaa    252960
     aaatatatat tctaacgctt ctcagcatct cttgatttgt gtcatcaact gaattgtgcc    253020
     ctctttgaaa ttcatatgca gaaaccttaa attcaattga tgtatattgg aattttaatg    253080
     aaataattaa ggttaaatgt ggtcataagt gtaagactct aattcaacag acgtgtcgtc    253140
     tttataagaa gaggaagaga caccagagac ctctcacttt tcacgtgcag gcagagaaga    253200
     ggccatgtgg agacgtaatg cactagaagg tggcccagtg caagccagga agaagcctca    253260
     ccaagaacca accctgccag aacattgatc ttcaacattc agactgcaga attttaagaa    253320
     aatcaatatt tgttgtttaa gccacccact cctgttgtct tcttatgaag atccagacag    253380
     actaatacca cataactctg ttagcgctgt cccctggatg cagaatcagc ccgctggggc    253440
     tgggcacatc tctcagattt ccacataaag taggcaaaaa atagtagttc tgatataaaa    253500
     atttgtcatg tccctgttgg ccaatttctg ggcaaggtct tttaaagaag ccctgggggc    253560
     tttgtcacaa aagttgcctt ttatcattta ttaggacata actgatgaac aatgagtacc    253620
     agttggatgg agactgacca ctgaccatct tctgctgtct cctaagtatg ccacagaaaa    253680
     ccacaccaac attactctat gtcttcaact ttctaaattt gcactgattg gtatttaagg    253740
     caggcccagc gttgaataac tcctttagtt tttgcttctc tgggaaaggt cttatctatc    253800
     ctggccttgg tcttcaagtt tcagcaattc tgggaagcca aggacgcctc tatctcctcc    253860
     tccatgctct gcaactcacc tgagaacagc tttctcattg gaatgtcttc tgtttaagga    253920
     ataagagtcc ctgtttcagg cttgggtgcc tgagtacacc tactggatcc agcccaggat    253980
     tggagaaact ttccagaaca catcacctga gaaatgacca gtcacactgt tacactttca    254040
     caatttccgc ttcctcatga gaaaattaaa attgcagaga ctttttcata agcgttgtgc    254100
     catgtccttt cttgttttct tgcctgttca tttatgtcag accaggtgcc acatctatgt    254160
     aatcaggtta gaatcctgcc tccagtaaca catgaaaagg acctatggtt gtacttttgg    254220
     tctttgctcc aaagtgtaaa gattacaaaa gtcatcaccc tcattcttat gccaagagtc    254280
     atctgcacaa tctgatcttc aatacatttt agaatccatc aaatgaatga aattccattt    254340
     tttaaattac caccccaaaa actagagaga tgggcatgtc cagaatagca gttgatggtt    254400
     gcttaactgg aagagaagtt tcagaagcca caagctgttg aaggcactta cgtggttagc    254460
     actatagacg tctgcaagac agatgtggac tagggtgaaa tgacagttcc agagggccgc    254520
     actctcctca gtcttctgga atttccctct agaaatctcc agaatctaaa aaatacaatc    254580
     caaatatgtt tcctatgggt cataactggg gaagtttaat tactgaaaaa tatatcagga    254640
     gccttctcca aaagatccta cagggaagaa cttttccaga acctcatact atgtgaaggg    254700
     aagaaaaatc tgcccattcc agatccctcc ccacttcctc cattattata caaatgagta    254760
     agtttagcca atagggtaag atgtaagcaa atagtccagg gaaactgaag ccacaaaaag    254820
     gagtaaagat gaaaattcag cttttcccct ggagatgcct ggtcaaggtc acagcccaga    254880
     aaaggaatct gattgagtct ctaggtttcc atggtcagaa caagcagtgc tgacccgcac    254940
     tgcacaatcc tttctaacca ggatgatggc tctggattaa atatgagagt gtgccaatgc    255000
     acagtctctg aggagaacat agggacacta aaaaagcaat ggcagggagt tagacaagga    255060
     cagtagagca atatgaagcc tctgacgtga acatttttaa aaacaagatc ttggaaactc    255120
     cctcattcac ctcagcttct tttatcatga agactttcca agattcttaa ctgagacaaa    255180
     caaaataaca acctgcatgc acttccgaag tctccgcttg tatcctgttt gctttagatc    255240
     tctaggagaa aaatgtcaga cacctgggcc tagtgtcaat gtgggaggca ctttctacag    255300
     atgaggcaca agaaggaagg gaaaacgtgt gttattggaa tagtggatat gaagtgtgct    255360
     ctcatctgaa agcatctgca cctgctggaa atctcagatg caacattcaa ctgcaagaac    255420
     caaggcacac ccaaatctcc tgtaagattt tggattcatt atccactgat tcagtgcaac    255480
     tggagcttca gagaaggggc tccctcctgt gtcatagcat ccttgctttg agttcattag    255540
     atttagtaag gctaatcaat tgtttgaaga gatggtgtca gcagcgtatg ctgtcactga    255600
     aggagtattc taaaccagga caaagccatt tcatgttagg cgagggaagc tggtggaaaa    255660
     tgctttatga gccccacagg aacttccttg caaggcaagg gctgggctgg agggggcgcg    255720
     caggagccgc ccagcacagg ttccatcccc ggaggggtgc acaggaggct ggggacgagg    255780
     ttccctctca gcgcctgtgt cttcctttgg caacaaaaaa aatcctaagt gttcaagaag    255840
     ttgctgatgt gtctttaagt atcctgttcc gtcagagcct ttcctataac tgaaggcaac    255900
     cagaactgtg ttttaaagtc ggttccgagg actgcaagta tcttaatagt gaggatataa    255960
     aggataggaa tggttttact aattgaaagg atacagaatt gtggggttcc gaggactgca    256020
     agtatcttaa tagtgaggat ataaaggata ggaatggttt cactaattga aaggatacag    256080
     aattgtggga gtcactatgt tcctatgaat aaaaaattca gatttcagtg ttaagtaatg    256140
     ttgcctacat tgtgtgagtg acagggcagt ggtggatctg agagtgtggc aggtgcacag    256200
     acctagtgag tcagaaatca atatggaaag atgaggatct atggatatga actgaaagta    256260
     agtaaacagt tcatgaaatt ctattaaatg gagtaggaaa taaaacccaa acttatccaa    256320
     aacacaaatt ccttggcgat tattttggga gcagtgagtt catcaggaac cccaaacttc    256380
     tcttacgtct tctgattcct gttgtccatg agatgagaaa ttcagctcta attgtacatc    256440
     acagggcaaa tctgtaaacc aggagtgttt ctattgagga tcatggtgga tcaggattcc    256500
     aggcaggtgc tggagacact gtctcaggag cgcccagatg atctcagggg gacctgctgg    256560
     acactcacgt gggacatcag cagtcacttt ctcagagtaa ccagtgagct gtgctggtgc    256620
     ctgatgggac taggatgggg tcaaggcacc ttctcagtgt catggagagt gattgttcca    256680
     gaaatcatcc aggtggtctc tacgctaatc aaatatgggt tcacagggag gaacatgtgc    256740
     tctgggtgct tggtcttcag tgaaaggacg tctggccacc aaaagtttgt aaatggagca    256800
     gggcatgcat ttcctcaagg aggattaggg cttggagcat cagcatccca ctcttgtaag    256860
     gctgatgtgt catttacctt ccctttctta tcccaaatca gggtcttcag ctatgaaatg    256920
     ctctgactca tgaatatgca aataacctga gatgcactga ggtaaatatg gatatttgtc    256980
     agccctgaga gcatcatcca gaaaccacat ccctccgcta gagaagcccc tgacggcaca    257040
     gttcctcact atggactgga tttggaggat cctcttcttg gtgggagcag cgacaggcaa    257100
     ggagatgcca agtcccagtg atgaggaggg gattgagtcc agtcaaggtg gctttcatcc    257160
     actcctgtgt tctctccaca ggtgcccact cccaaatgca gctggtgcag tctgggcctg    257220
     aggtgaagaa gcctgggacc tcagtgaagg tctcctgcaa ggcttctgga ttcaccttta    257280
     ctagctctgc tgtgcagtgg gtgcgacagg ctcgtggaca acgccttgag tggataggat    257340
     ggatcgtcgt tggcagtggt aacacaaact acgcacagaa gttccaggaa agagtcacca    257400
     ttaccaggga catgtccaca agcacagcct acatggagct gagcagcctg agatccgagg    257460
     acacggccgt gtattactgt gcggcagaca cagtgtgaaa acccacatcc tgagagtgtc    257520
     agaaacgcca ggaaggaggc agctgtactg gcatggaggg gatgacaaag gttattagat    257580
     tgaagatttt cttagaaaac gacttcaagt cattaaagaa gaggaacaac ataaatgtgt    257640
     atttgtgaaa ttttaattga gagatttttc atacaacatt tattctgtaa gctatttcag    257700
     ggattggaat atgaatcaaa ttaataaagc tgatatagac atcctctgaa ggcatcttcg    257760
     taaacatcaa tttctgaatc agtgttgtaa atattttgga acacagacac aagatcacat    257820
     ttttactcta cttttatctc tatttttaaa aaatgccaaa aagaatctta ttttgtgcat    257880
     gccccatttt gaattcccac cgtcaatgca tgatagttct tggttttcct cattcaagtt    257940
     gtcatttgtc attaagagtg ttgtgtgttt taaccattct aataggtgag taacggtatc    258000
     taatttttat ttaaatgcac atgtccctaa aaaattcata tttaacaatt tttatataat    258060
     ttttggtgag atgcctctcg tgatatttgg ttcatttttt aaatgcattt tttatcagtt    258120
     gtaagtatgc ttgcatattg attataaaag tcatttaaca aattaaaata attcatttaa    258180
     caaatatgca tcttggaatt tttttctcca agtctgcagt tgtcttttac tcccttatcc    258240
     ctgtgtattg cagaaaaatg tttgtgtgtg tgtgtgcctg tgtatgtaca aatttagatt    258300
     ttaaaaatgt acatttttat tcatttaaag atcatgtctt tggcagtata tctgaaatct    258360
     cattgtaaaa gacacaatag tcattatttt ttccatgtct ctaatcttag gacacaatca    258420
     actcatgagt gtttaacctt ccctacctga ttggaggact atccacctga gacatttgga    258480
     atacttctgt aaggagatgt gtccttccca ttatttcttt atttagtcat ctattgatgt    258540
     tcctattggt ttatggatgt ctatttcata ctccgaagaa gatccttgtt acattacttg    258600
     ttttattgtt caaaacacca cagctttatt aggtgctggg agctcattta gtttggatcc    258660
     tgcatcctta cagcacacct catctttttg tttttagaca tttccctgtt tccaagtatt    258720
     acaataaatt ctaagcttgt tctctacgtt accttttttg tacatagaat cagccatttt    258780
     tctaaagact gcttgttttt catgttaaag aacagtattg aaatttaaaa atgtgatcct    258840
     gggtatgtgt gttgttaatg tggtatcagt acttcttcta ggatatctcc accaattggc    258900
     ctagtaaatg ggcatgttta taggaaccca agtttatgga cacatcgaaa ctatttatgt    258960
     atctaatcct ctgtaatgtg attacattaa aaatgagaac acactggtgt ctccatccaa    259020
     ctatgctacc atatggatgt ttccagcctt ccttccctca ctgtccataa ccacccactg    259080
     caaagtgagg aaccccatcc caccatatgc cattttatta cttagctgca caatttcagg    259140
     acacatgcat agcagtatca gaaatgtaaa gctcgttgga aacatgttta tctactagaa    259200
     cagactgctt atgtgcagtt tctttacact gtaaacttat agaatttctt cattttcaaa    259260
     gttgcttagg tcagcaactt cattttccac tctcctcagt gaagtcattt caatgacaat    259320
     gtataatgtc atttatttga aattctttca aagccaaaac tatagtccgg taaacatata    259380
     gaggatattc aaggaattta gagagtgggt ataaaataag taaaaatggc actgtttaag    259440
     aagagtaaaa ttgtttttag tgatatacaa tggttgagac acaacacaat taatttggct    259500
     aagctcatac ttttgtgaca gaaaatataa acctaaatat atacaatttt tgtaaaaaaa    259560
     ctttagcagt tcattaaccc cagggttaaa caagactgtg taaaattatc taataactta    259620
     tttgatgagg gtggggatat catgagacat atgcaacaaa gaatgaaggc attttcctca    259680
     tttgcatgta agatgttacc attcactaaa gacctttaat aaaaaaaaat ttctacgtga    259740
     tccagtattt ttttccttct gtcaagcaaa taactcatag gattcttttc tttccttggt    259800
     tgagaaagat tttctacaag cttcagcaca cgtcaggcat acactgtccc tgaatgggca    259860
     tttaccctca gatgggtaca cacacctgtc aacatggggg ctcttctgtc agacaaacac    259920
     acctttactc atgtggattc ttccctcaga caaacacaca tgtccccacg tggactcttt    259980
     cctcagatta caacatttgt ccttacattt actatttcct cagaaaacag acattgcatc    260040
     atgtggtctc ttgtctcaga caagcaaaca tgtcttcagt cagataagtc tgtggatttc    260100
     cacattgact gtttccttac acaagcacct atatccaata tttaactgtt ttgtgagaaa    260160
     tttatccctt ttctctggaa atgtattttt atgttcttac tggacatatt ttttaataat    260220
     gtttgctact atgaagatac ccgaacactg tccatacgag agaataagaa agagtaataa    260280
     gcagattaac cctgtgcatc cagacccagg agtcctttga tcctgccctt ccgaaatgga    260340
     gacacagagg aaggatgagc aatgctgagc agtgcaccca tgaccacaaa aggaaagaca    260400
     tggcaatgtg tcccctcccc tcctcatgaa aggcagctca tcccctgttc cttcaggccc    260460
     tggtgaggag ccaccccatg tatattccct tgatcagtgt ccacaccatg gggtctgcac    260520
     tgatctgggc ttcccttctc atcaccctca atattagtgt cccttgtgaa tcaggtccag    260580
     ctgcggctgt tccacatggg gccgttcttc catttcctca gtgtttgcag aagtcctgtg    260640
     tgaagtttat tgatggagtc agaggcagaa aattgtacag cccagtggtt cactgagact    260700
     ctcctgcaaa gcctctgatt tcacctttac tggctacagc atgagcttgg tccagcaggc    260760
     ttcatgacag ggattggtgt gggtggaaac agtgagtgat caagtgggag ttctcagagt    260820
     tactctccat gagtacaaat aaattaacag tcccaagcga caccttttca tgtgcagtct    260880
     accttacaat gaccaacctg aaagccaagg acaaggctgt gtattactgt gagggacaca    260940
     ggagagggaa tatctgtgtg agcccagaca caaaaatctc tgcagagaga caggagggaa    261000
     ctgcatggta gatgctcctc ataaccacaa aggggcagtc aggaccatca ggaggagctc    261060
     aggacacctg ggggtgctca gaaccatgag gggtgctcag gacatcgggg gctctcagaa    261120
     ccatgagggg tgctcaggac atcagggggc tctcagaacc accagggggc gctcaggaca    261180
     cctgggggcg ctcagaagca tcaggggtgc tcaggatatc agagtgccct cagtaccacg    261240
     agggggcgct catgacacca ggggcactca gaaccaccag ggggcgctca gtacacgggg    261300
     gttctcttag gaagcagctc cacatcagga gcctgagagg ctgtgatttc gttttaaacc    261360
     ttggtgattc ccgacctggt caagcaaaag tcttccccag gatctctcac catttcttct    261420
     ttgtaaatcc atgatttttt tttacctaca aaacattaag ttagaacagg gatttaattc    261480
     aacttttaat tctgcagatt ttccgagtaa tagtagcaat gttccctcag gacaattttt    261540
     aaaattggtt atttatatat tttatttatg taaaatgata ctatcttgaa aatagtaaaa    261600
     tttttaaaat catacctcta atcccagcac tttaggaggt tcgggcaggc agatcagttg    261660
     cgctcaggag ttttagaaca gcctggcaac acatttagat cttttccata caaaaagaaa    261720
     aagaaaaaga aaatagctag gtgtggtgct acctctggta ccagctattt gaaagtctga    261780
     ggtgggagga tcccttaagc ctgggaggtt gatgctgcag tgagctgtga ttgtgccact    261840
     acactccggc ctggttgaca gagtgagacc ctttcttaaa aatacaaact gtttcaataa    261900
     gtagagcttt gtgtctttgt gctgtaataa tcagttgtat atattttcta actgtaactc    261960
     agcatatgca tggtgttctg tttctttttc tttcatttgc tgtttttgga aattaaacac    262020
     ctctttaaac gctcttgttc tcccctttgg ttcgcttcgg gtgtcctgtt tttcagacta    262080
     tttcttcatc ttcccttttt ctttgaaagt ctttttcttt ctcagtttcc atgcaggata    262140
     aagaaagtcc ctttactttc tgtcctccaa gtctggtgaa cagttccctt ctcttcataa    262200
     ccactgaagc caaccaagtt taggaggata acagttctcc ttagaatatg ctcgtctacc    262260
     tgcagactct ctgccctcct cacccttttc tagggtcctg cacacatcac actcatccca    262320
     tcccctccct tccctaagta ccacagagtg ggctctgcag ttcctgcttc cctgtgtgtg    262380
     ctcagccctg gggctcacta gtgctttgat gatgacgttc aaatccccat gtgcttagac    262440
     tctctaagac caccctctag gaagatgcca ttgtgagtga gtcctggaaa tcatgggctg    262500
     tgttcagttt catattgctg gatcttctct attttaaagg aatactggca attaagatta    262560
     gagtttgtat tgaatattca tgccaaaaaa gttttctttc aaaacttttc aaataaaaca    262620
     atttttcctg cctagtttga aaactacaat gtaaattcaa caaataatat aatacaattt    262680
     taaaggtgac gtttgtctta ttggttattc aatttattaa aaacagaaga tatttaaaat    262740
     aaattccatt gcacatttaa gtgatgtatt tgacaagaat ggcatttaca tacattttta    262800
     ccaaaacacg tatttaaata tatttgtctt tttaatattg gtagaggcag acatacacgt    262860
     agaaaagcat cattttgtac tacaatctca aactgtaaac acaatttaaa ttcagttaaa    262920
     taattagaat aatatgaaac aggccgggcg tggtggctca tgcctgtaat cccagcactt    262980
     tgggaggccg aggcgggcgg atcacgaggt caggagatca agaccattct ggctaaaacg    263040
     gtgaaacccc gtctctacta aaaatacaaa aaattagccg ggcgcagtgg cgggcgcctg    263100
     tagtcccagc tacttgggag gctgaggcag gagaatggcg tgaacccggg aggcggagct    263160
     tgcagtgagc cgagatcccg ccactgcact ccagcctggg cgacagagcg agactccgtc    263220
     tcaaaaaaaa aaaaaaaaaa aaaaatgaaa caaataggtg cgttgttttg gtgtttagat    263280
     atacattcac ttttgcatgg gcacatgtat gtgtctttgc tgggctgttg tgtatgtatg    263340
     tgtgcttgta tgaccatcaa gttttcaaat acatcattaa atttcatagt tacattcgtc    263400
     ttggccaggc acggaggctc aggcctgtaa tcccagcatt ttgggaggct taggcaagca    263460
     gatgacttgg agttaagagt tcaagaccag ccttgtcaac atggcaaaac cccatctcta    263520
     cgaaaaatac aaaaattagc caggtgttgt ggcacgtgcc tttagtccca gtgcgacagg    263580
     gaaatgctgg gtcagaaatc ccacacaaag tctctacttg gttaccacct aatggaactg    263640
     taggaagaaa gccaccatcc ttcagaaccc agaatggtag atccactgac agcttgcaca    263700
     gtgtgcctgg aagagccaga aacactcagt gacagcccat gagtgtagcc agaagggaga    263760
     ttgtgccctg caaagccaca agggcagaac tgtccaagac catgggaacc caccagttgc    263820
     atccggtaac ctggatttga gacatggagt caaagaagac tattttggaa cgttaagatt    263880
     tgactgccca gctggatttc agacttgcac gtggcctgca gtttggacca atttctccca    263940
     tttgcaatgg ctgtatttac acaatgcctg aacccccatt gtacctagga agtaacttgc    264000
     ttgtttttta ttttacaggc tcatagatgg aagggagttg ccttgtctca gaaaagacca    264060
     tgggctgtgg acttttgagt tggggctcaa atgagttaag acttcagggg cctgttggaa    264120
     agtcatgaat ggttttgaaa cgtaaggaca ttagatttaa gaggcgccag tgttggaatc    264180
     atatggtttg gctgtgtccc tgcacaaatc tcatcttgaa ttgtatctcc cacaattccc    264240
     acgtgtcgtg ggagaaaccc agtgggaggt gattaaatca tggagggagt tcttctttgt    264300
     gcgttattgt gatagtggat gactctcatg agatctgatg gttttaaaaa acgggagttc    264360
     cctgccctct ctttgtctgc tgccatctcc atgtaagata aaacttgctc ctccttgcct    264420
     tccaccatga tgtggaggcc tcccaagcca tgtgtaactg taagtccatt aaactttttc    264480
     ctctataaat tactcagtct catgtatgtc tttattagca gtgtgaaaac agactaatac    264540
     agcaaatatc aatctcttaa aatattttgt tgttctgcat gtaatatagc acagtctaat    264600
     atgggaggta aaataaatca tccatggacc ttcagatata agtcataggg taattatgcc    264660
     tgtgtccctg aaggagtgaa ctagaggtta tacacactag tggcactacc tttggcaaag    264720
     ggtgttcagg ggtttctgaa agttctgata attttaattt taaaattgaa tttactatgt    264780
     atttcttcat caacctttcc agaagatttt tggcagtaag aacagcctag tattggagta    264840
     atactgatga attagagagt tattttgtaa ttattctcct gagatttgca tgaatcaatt    264900
     gatatagaag ttcgtatgtc ccaagttgaa acatcagaat caagaaggct tcatcaccct    264960
     taatggctgt ggtttttggc aaggcagtac ttcaattcag ccagaaagag agacaacaat    265020
     aaaactttca aatgcagggg agtctgacct cagtctctct cttataaagg caaaggcaaa    265080
     gctgtgccac atcaagattt ttcttcaagc cttcagagtc aacattggga atggaaaagc    265140
     caatcacttt gtccttagag aaggcgaaga tctgagagga atgcagagtt gtgttcatga    265200
     aggtgatgac attgttattt cttctccttt gcccagtgac tgctttcaag ttgagtgttt    265260
     cagatgtcag acctgactta tagggatggt tgtgactgca cgtgactgac agggactgag    265320
     actctatatc tctaagtgtc ctgtcctagg agcagctaca ggagtcagcc ctagactgga    265380
     agtgccctca ctgaccctct gcctcacctg tgcagcctct ggccagttga agaaagtttt    265440
     atccctggaa catcttttgt caaaaccact ccaactagta ttcatgacct atggtccaca    265500
     ccctcaagta cacagacatg atcatgctat gacttggtgt ctagaagatc cctgcacttt    265560
     taattttacc tctaatatta gactccactt tatcacatgc ggacacagca gaatatttta    265620
     agactttgat atgttatgct gaaaaattca atagttcata aaagtctact tcccatcact    265680
     gccagtgttt actactgagt aaggatctgc catcccctag ggaggaacta taatggatgg    265740
     tgtgccctgt tagtggttat gggaatgaat ccatcttgtg gcaaatggca gcatcttctt    265800
     gttttgcaag attaaatagt tttctatggt gtataaatac aacattgtct ttatccattt    265860
     gtctatctac tgacactcag attgtttcca tatattggct aagattaata gtgtttcaat    265920
     aaacatagga ttcaactatc ttaacaaggt ggtaatttca tctgctttgg gtatatttct    265980
     aaaagcagaa tttctggtca tataacattt ccagttttaa ttcatttggt gcctttatat    266040
     tgctttccat aattgtgaaa atatagagtg gtatagccat tatgaaaaac agtttcagtt    266100
     ttgaggtatg atccataaac aaataatgtt tgattatggc tctccatgag aatttctaat    266160
     aaatatgata gaagtcaaga aagcgtctca cattaaagca aggattaatt ttatccctta    266220
     ctccctcaaa agcaaaagat tggaagcatg catgatggag gctgttagca gggttcagaa    266280
     tgatatcata aaaggaaact cagttggata aaaatgttgg gctttcagtc ataggtatgt    266340
     cgatactcaa tatgaaaccg cgaagtcaaa tgaggatcta gaatgtgttc acttgaagca    266400
     acagcacatt ctcaaacgca cctattgctg catcacaagg gtgatgaact tttgaaacaa    266460
     gtaaagtgtg agtgcacagt aagtgataaa gttatcacct ttacatgaag tttgttcaat    266520
     atgccaaagg cttttacaga ttaatcatac acaaatatac acaatgtatt tttgcataca    266580
     cagatgtcta gaggatgatt cttttaggga aaattcttca ggtcacagaa atctgtgtaa    266640
     gttggaggtc ccatgaaaaa gtatctcttt ataaatactg ttctattgct caattaggga    266700
     aaatgatctc tgttggagaa agcttccaat tttgcagatt tcttcattgc ttcttgagtt    266760
     gtaagacatg aaccaaaaaa ttgactggag ttttttggag tttcatggct ggagtttcat    266820
     ggctgtgttg ttaaaaattt cttatacggt ttctttcaat tatttgagat cagtttttct    266880
     gatttttttc tccagtagat aaattttcac aagatctcag gaaaccatgt ctctggtgct    266940
     ttagtttgaa aaactaactt cctaggtaaa gaagcttctg tctaaccatg atgagctcat    267000
     ttcttctgca taccatgaag tttgagaccc cattgtccag aatcatgaaa attttacact    267060
     ccagtttact gaaaatcttg actttgaatt taatattgat tggcattgac aaaaatggtt    267120
     cattcttgga tgtgtcctaa gttgctcacc caacatatca gtggactgag aatcttccat    267180
     tagctccctc tgtccatagc actgagtcta ttgacaatct ttgcagtcct ctatgaagga    267240
     gagtcctgag gttcatcaga ttcttgtaga cactcagatg aaccaaagga attaacggaa    267300
     ttgaggactg ccagccattt ccacaacact aggaataatt accatctaag tgtaaaggtc    267360
     tacatcattc agaataccct gcatgacagg ctgatgcaaa aaaaaatcca accctgcaga    267420
     ggcttcacag caaccctcac agttccttca gggaagaaat aatctccaag ttcagtgagt    267480
     cagtaaagct gttctgagct acagtaaaca ttggattggg cccagttttg tctgagttca    267540
     atgtgattgt tatactcagc tgctgatcct atatggactg agtatggata atttaaatga    267600
     gcctggctgc gtggtttgtt atatgaaaac ctgaactaaa tataaataaa gggcatgtct    267660
     ggactagcat gagggtgaga gattctggga gctccacccc ccttactctt attgcccttt    267720
     cctccaggaa cctccaggtc ctcagggtga gaatccacac agatcccttc atggctctat    267780
     tgccaggaga ccaaatctct gataaaatca ctcagacctt cttcagacag acaacccagg    267840
     gaaagggcat ttttacaccc ttatgtatgt ggggaaaggt aatccatacc cattagaagc    267900
     ccctgccagc agcctaactt tatcatgaaa atgggtaaaa ttagccaata ggggtacatt    267960
     taagaaaatt ttcctgtgat gttccatcca gaaaagagca aaaatcagct tcacacctgg    268020
     agacttcctg tatcgggcac agcccagaga aagaagatca ccacagaatt aaaatgcaac    268080
     tgtaagaaca tgtaatgctt ccgtgttcca cacattatgt ctcaccagtt tagtcaatat    268140
     ggattaaata tgagagcgtg gcaatgcaca aactctatct gaggaggaaa gtcagagaaa    268200
     aaatgttaaa gaaaatatgg caatttgaag cctctgacac cagcaacttc agcaccaagg    268260
     aaatgatttc accctcattg gcctcaaatt tacttttcat ggagcatctt cagggttcca    268320
     aagtgagacc aggtgaattc aatgtgcatg cacttcccgg gtgtccactt gtattttgtt    268380
     tactttactt ctatttgcag aaagtaaaca catactcagc cttagtgtca gtgtagggag    268440
     tgctttccat gacatgaata ccagaaaaaa aggaaaaaca tggggccaat taatgtaaaa    268500
     attagccact gtgtgtgtgt gtgtgtgtgt gtgtgtaggt gtgatttgaa tactagagtt    268560
     ggagtgggct tctatccaca tgcacctgca cctgcaggta gtctcaggtg caataatcaa    268620
     ctgcctgacc ctaaaggaaa caagaatctc cccaaacccc tgaagagtgt ttgggtttac    268680
     cgtgtgtcca gtgattcagt gcctctagag ctccaggaag gggctccccg gtgatgcctg    268740
     agatcttttc ttcaggtctc cctgcagagt tctctgggtt tcctaagggc aattcactat    268800
     ttcaaaagat ggtgtgagaa gcacatgctg tcattaaagg agaattctga gccacggcac    268860
     agccacttta tactgggcta gagacactgg tatgaatata ctctgtaagt ttagacagaa    268920
     agcctcgtgc atggtagggg ctgggctgca gggggtgctc aggataaacg cagcacagtc    268980
     tcccgcccca gagcaggtgc acaggaggct ggggaggggt tcctctcagg gcctgggact    269040
     tcctttgaaa atatctaaaa taagtatttc acaagggctg ctgttgtttg tataaatatc    269100
     ctattcaatt gtgagcattt atcaaactgg atgttgtaat gacaaccact tttacaatgg    269160
     ggatttcaaa ctcccctaga tatcttaata gtaagcagct ggaggtcaag aagagatcct    269220
     ttcttttaaa taagtgcaat ttttggagaa acatactaat tcccaaaata acgcattcac    269280
     atattaaggt ctagaaatgg ctcaagttgt ccctggtgca ttcgaatgtg ggttcaaagt    269340
     gaggtgcgtg tcctgaggga gcttgttctc cagtggagga agctctgtca acacagagtt    269400
     cagggatgtg taggggtcgc atcgcctcta acaggattac ggcttgaacc ctcagcatgt    269460
     acaattgtgt cgtccatctg tcatgtattt gctctatctc atcctggctc aggaattggg    269520
     ctattcaata gcatccttcg tgaatatgca aatcactaag gttaatacag atatctctgt    269580
     gccgtgagag catcacccaa caaccacacc cctccttggg agaatcccct agatcacagc    269640
     tcctcaccat ggactggacc tggagcatcc tcttcttggt ggcagcagca acaggtaagg    269700
     gactccccag tcccagggct gagggagaaa ccaggccagt catgtgagac ttcacccact    269760
     gctgtctcct ctccacaggt gcccactccc gagtgcagct ggtgcagtct gggcctgagg    269820
     tgaagcagcc tggggcctcg gcgaaggtct cctgcaaggt gtctggttaa actgtcatca    269880
     cctatggtat gaattggata cgacagaccc caggacaggg gcttgagtgg atgggatgga    269940
     tcatcctacc ctggtgaacc caatgtatgc ccacagattc acacacggtt tgtcttctcc    270000
     atggacacct ctgtcagcac ggcggatctg cagactagct gcctaaagac tgaggatgca    270060
     gccatttatt actgtgtgag gtacaccgtg tggaaaccca catcccgaga gttttagaaa    270120
     ccctgaggaa ggaggcagct gtgatgagct gaggcagtgg tgcagcatgt ctctaaactt    270180
     ccattttatc taagtttgca ttgagttccg ctttaatatt agccaggaat gtgggataga    270240
     cgggtgctcc taagaggtcc ttaatttgcc cattttgatg ggttttccag aagacgtgag    270300
     aagccacttt gttaacaaag catcccaaag ccatgccctg ctccagaaac acgtgtaccc    270360
     atttcctggt ctttggttaa ctgacaagct ctcatcagcg cacctgggct aatttctcat    270420
     caggtagaaa aatgtgttgt aaagcaaggc taacgttgtg atagcaattc ctgctcaata    270480
     accttcagca tcgttgttgt tgtgttctat caactaatta cttgacttca aggttctcat    270540
     tgggagtgtc ttataaattt aagggatata tagaagttcc cctaattaaa ataaaacaat    270600
     tgtgagcaca acctcagtgt tcaaccatgt ctccaccctt cccaccattc accccaaaga    270660
     aatgttcacc tctcctggaa gtcgggttca ttttcaaatt agttattttt tattttacta    270720
     tatcaagatt attgtatgtg actactgtag cagaaagtga attaggggaa cttgaagtaa    270780
     ccaacgaaag ataaattcag aactaattaa acaagatgtc agaacgtgat tggctctagt    270840
     cttttaaaat tcagcaggtt atgtaaccag gctttaaatt tacacatctt cctgttacct    270900
     tcacggcaca gtcaactccc attatgtaag aaatggcaac tgcattccca agcgtcatcc    270960
     aaaattgtaa aaatagactg ggtgaggtga ggagttgatt gtttaaattc cgctctgaag    271020
     aagcagcatc aactcaacaa accaccgctt ttccctcagt gactagagct atgtcgcagg    271080
     ccacatggac ctaaatatcc ttgatagaga taataggact acataaattg ggctgatcat    271140
     ttttatgctg taaaattaat aggtgagtct gcactccagc ctgggcaaca aaacaagtct    271200
     tgcctgtaaa tacaaaagaa agataaatta ataggtactg actttgacat ttcggataat    271260
     aatattttca taaaccgaat ttaattatac ccacattgtt acctacacct tcactgaaaa    271320
     gttcctagtt atcatgagtt ccatcaacac tccacgtgtt caaatctgga catccaagag    271380
     agtctggaga ataaaatgca atgagggcag tgaaacttgc atatattcag cacctcttaa    271440
     ctcaggagga ctcaatacac cctggaacac tctgcttttc tgaatggctc acaatgactc    271500
     cagctcactc tccaacctcc tcaaacatct ggcttctgtt tgccctaagt tcacgctctg    271560
     ctcttagtct gtgctctgaa gtctttgcaa aggtgaaaat gagctgtcag atggaacttc    271620
     cttctcacct cagcatggaa tttactgttt catttaatga ccactctttc cataatggtt    271680
     gatttctttc agcctgttca ttactggtga ttttcaaggg aatctctatt gaatttttac    271740
     atttttgcat ttttgtctcg gtgacaatgt tgagaagttt ttacctctag catcataaca    271800
     tgatctagtg acctgacaca tttgtggcaa gcaataccta caaattcaga agttctttgg    271860
     ttactttcca caaaatataa ttatttctgg tctgtgtatg agcatatcct agcaaccttg    271920
     tactccacac aggtagatgt ctacaagcct atgaattaat ctctgtaaat aaaaatttat    271980
     ctcaatttct ttcaatgttc ataattcttc tgaggatgag gaagatcttt ctggatctat    272040
     tcagacaata ggcccagaga ccacctggta tgtaaggagc tcacctggct cacctggttc    272100
     cccctgttgt ctcacataag gtcagcccac ttgttcaggt cctaagaaga gagctcagtt    272160
     ttatctgatt ttacaacact cccaatttct gctgactctc ctgttaccca catccatgga    272220
     gatacattat ttattataca attaaccaaa gtaatgtcaa aggcccaatg tgcaatattg    272280
     cacatcctag ggtatgttca tgcaattgaa tcgaggagaa agtctttcag agacagatgg    272340
     atctgaactg gtaaatatgt gtgtaaggac tctgggctta agtgtcattg tccagccatg    272400
     tttcacaggt gtgacctgtc agggaagaac cagagttcct tgtgttctca gaggggaggg    272460
     gtcccagaag tcctctctgg ttcccaggaa aggtaattgc attaatcttg gtgatgagac    272520
     tatcatccag tgatgatgta ctatagagtt tatgtttgaa gttgacactg ctatcgcaat    272580
     ctacatcttt tcacacagaa gtgtttagag gtcaggccac atcttcagga tcccacattg    272640
     agaaggacag agatatattc cactaccttc tcctgagatc tcaggcagaa acccaaattt    272700
     caaaaggtct cagaagggca gctctcaggg gctatttaaa aataacccac ttcgtgggac    272760
     agggagcatc cttctaacca tgatggatgt tctgaactac aataaacatt gcatggatcc    272820
     agggtctgaa ttcactgtga ttattacact ccactgctgt ttcaatgtgt ctgaaggggt    272880
     aaatgacaat ttagatgacc tgggtgtgtg gtttgtttta tataaatctt caaggataga    272940
     acagcattga acctattcca aaatctgtcc ctgatccaag atcacactga tctcccagac    273000
     cagcatcttc agcacatttc cctacctgga agaagaggac tatgggcttg gtaaggggag    273060
     gccacaggaa gagaactgag ttctcagagg gcacagccag cttcctactc ccagggcgag    273120
     cccaaaagac tggggcctcc ctcctccctt ttcacctgtc catacaaagt caccgcccac    273180
     atgcaaatcc tcacttaggc acctacagga aaccagcaca catttcctta aatttgggat    273240
     ccagctcaca tgggaaatac tttctgagac tcatgggcct cctgcacaag aacatgaaac    273300
     acctgtggtt cttcctcctg ctggtggcag ctcccagatg tgagtgcctc agggatccag    273360
     acctgaagat atgagatgct gcctctcatc ccagggctca ccgtggttct ctctgttcac    273420
     aggggtcctg tcccaggtgc agctgcagga gtcgggccca ggactggtga agccttcgga    273480
     gaccctgtcc ctcatctgcg ctgtctctgg tgactccatc agcagtggta actggtgaat    273540
     ctgggtccgc cagcccccag ggaaggggct ggagtggatt ggggaaatcc atcatagtgg    273600
     gagcacctac tacaacccgt ccctcaagag tcgaatcacc atgtccgtag acacgtccaa    273660
     gaaccagttc tacctgaagc tgagctctgt gaccgccgcg gacacggccg tgtattactg    273720
     tgcgagatac acagtgaggg gaggtgagtg tgagcccaga cacaaacctc cctacagata    273780
     ggcagagggg gcgggcacag gtgctgctca ggaccaacag ggggcgcgcg aggccacaga    273840
     gcccgaggcc gggtcaggag caggtgcagg gagggcgggg cttcctcatc agctcagtga    273900
     tctccctcct cgccagcact cagatgtccc cagggctcct gtttctttat tgtctgtggt    273960
     tctgcttcct cacatccttg tggcagacaa gaaaggagga agacaatttt tctgtttact    274020
     gttgaggttt caccaattac taggaacttt cctacaagtt cctgcgtgac tcattttacc    274080
     gtatatgtgt gtgtgtataa atatatacac atacacacac acaccatata tatacaccat    274140
     atatattata tagatacaca ccatatatat tatatatata cacaccatat atattatata    274200
     tacacaccat atatattata tatatacaca cgatatatat aatatatata tacacaccat    274260
     atgtatatta tatataaaca caccgtatat atatatggtt ctcaccatct cttgatttgt    274320
     gtcatcaatg gaattgtgcc tatttgaaat tcatttaccc aaaccttaaa tccaatggat    274380
     ctataccgga attttaatga tgtaattaag gttaaatgtg gtcaaagtgt gagaccctaa    274440
     ttcaataaac cagttgtctt tataagaaga ggaagagaca cccgagacct ctcacttttc    274500
     gcgtgcacac agagaagaag ccgtgaggag acgtagtgca ctagaaggtg gccctgtgca    274560
     agccaggaag aagccgcgct aagaaccaat ttttacagct ccttgatctt ccacattcag    274620
     actgcagaat tgtaagaaaa tcaatatttg ttgtttaacc cacccactcc tgttgtcttc    274680
     ttatgaagat ccaaacagac tgataccacg taattctgtt agctctggtt cgtggaggga    274740
     ggagcagccc cctgaggctg gacacttctc tcagatttcc acgtgaagta ggtaaaaata    274800
     gtagctctca tataaaaatg tgtcatgacc ctgttggcca tttttgagca aggtctctga    274860
     aaccagccct tgtgtgtgtg tcacaaatgt tttcttttat cttttatttg gacataacac    274920
     atagacaagg ggtaccagct ggatggagac tggtcactgc ccatcttctg ttgtctcctt    274980
     agtatgtcac agaaaaccac accaacatca ccaacgtcac tgtttttctt caaccacctc    275040
     aaaccgacta tagaaatgat ccctgcagta tagtctattt cttcaacttt ctaaatttgc    275100
     actgaatctc ttcctaaatg gggagctaca tggggtctga gttttgttcc tttcttccca    275160
     gtcttcccca agtgccaagg acagaataga cttaaaataa aatttggccg tcagtggccc    275220
     caaccccaca tcactttcta aaacccacat cctgcatcca tccttctctg gacacccctc    275280
     atcgggctac ctacgaatgg ccagaagctg ccatcacctt ctgggctgag gccacgagtt    275340
     atacacacgt gtgatttcag tcacacacac tctactgcag gacacacctg tgttctgagg    275400
     cactcaggca cctgctgatc tcagtcattc tctaataaat tacacatctc ttattaataa    275460
     aggtccagat ggtcccatca gctgcagagc agtggagtaa agctcatggg tgggtccgtc    275520
     aggtagaagt cagacaatgg atgggatggc tggtcacttc ccttttcact gatgtcccca    275580
     gtgaatatta atggaataaa accatattaa ctacagaggg acagaaaaga agactagctc    275640
     atcaaggtat ttaaggacca ggaactttat ttggggggaa agtgaaagac acttttaaat    275700
     ggaaagccct aaagcacata caacagctga gagagtggcc actgtgcaca tgaaggctga    275760
     ggagacggat ggcagcgtcc gttcctccaa gatgtccctg ggtgtgtgat ggttggactc    275820
     cttatgcata tgaatataag agctggactc agggagaaaa aagggccata tcccatagga    275880
     aaggaagaca aaaaagcaga tgggcatccc tggagagagc tcattagatt tggtgtgttt    275940
     aagacaaaaa tttcttccaa aaattgtaat gttctaagct aaatatgaac ctcttcaata    276000
     aactgagaat taacaggaga atagagctat gagttgagag aagaaacaaa tcatgagaga    276060
     gcagaaagca aatccacaaa aaactgtcat atgacagaag tcagaatgga gctggggcag    276120
     ctacttcatt attctgaaga cttgttgacc atgtggagaa ggggcttgaa caaatgggga    276180
     cgttctccaa ccttctgaat cagcctcctt cttatgcatg agtaaaaatc atagttctgg    276240
     gggtgaccct cccaattttc ctgtctgtac ctcttccccc aggggtagag tgtcttccca    276300
     accacaatgg tttctcacca gtgtcctcag cttctcctcc attgaactta ccctgcagat    276360
     taagaatttc ttctagatgt agttctttgg gaattttctg ttttctttag tttctgttga    276420
     ctccaccaca ccccataggt cacaggtttg attggatttc ccctggagac agtggaggtg    276480
     gatccaggcg ttcaacagtc ctcgctgttc ctcccttcct gtcagcacca caggacagca    276540
     gataagggag ttgactgtag atttttcgaa ttcttgggaa aatcctgcaa ggactagtag    276600
     attcacactc caataccatt agcacatgca tccaaaaaaa atactcacga aatatttcca    276660
     ggttagcctg ttcctctctc aatgccatcc agtggcacct gccctgggtt caccaacatg    276720
     tgggccccac tcctctctgc tggcatctct ctcctcacat ttcagtcttc tcgttagctc    276780
     tgtgaaagca actcagatat gttaaaaggt tttcttcttc atttattcag tttttcaggt    276840
     ttgttgttaa tgaggtcaga ataagaccat agttttctca tttttcacat tcccacactg    276900
     agtagccact ttctatataa aagccagaaa ctaagggaac aaatcaaata tccatatcca    276960
     ctacaggtga acgttaaaca atttgacata tgattatgaa ctaaagtaca atgcagaatt    277020
     agaatcaagg catcctcatt ctcataaaag catgtctaca ttctcaaata actctgctga    277080
     gtgaaagtag ctgaagaatt aagagtgcaa ttcataaact tctaattgta taaactgcaa    277140
     aaggtccaac tattctaaag taacagagca gatttgaaat ttgtgagaaa cgggtgttga    277200
     aagtaattgg ctggtgagat gaaattacag agaagtgaca gaaagattta ggggttaact    277260
     taattgtaca caacctgatt aaagtttgca cacatacgtt accattttcc aaattgtgca    277320
     gtgtagattt gaattaatta ttaattgtac ttaaaaaaag cagtaacaaa taaacacatg    277380
     aatatgttta ctgaggagga acaaaaaata gatgggtatg aacactggaa acatctcaga    277440
     ctcttgaaag tacacaggct tgaacactgg ttctctccgt atacttccgg taaacggctg    277500
     aatacactaa aagaaaacag agatgtcctg gcaggggtgg aatcctgcag acctcactag    277560
     gtgtgtccca cactgccctg gagttgtctc aggggagcag tctcctctag tggtcagagg    277620
     cacaggctga gataatgggg ttaactctgt ccagctgtgt gactttgaat gcattgtata    277680
     aacactctgt tctgtatgta atttatcttc cttaaaatgc aacattgaca cttacattaa    277740
     atgtattcta caaatatgtc aaaaagaaga tgatgactgc taaatgatta tcaaggcaca    277800
     atcacataat ataatgatat tttcctgagt gataagatga ctaccaatct cgggggcact    277860
     ttgtctgctc tgagccctgc ccctcctcag gattcccatc ccagagcttg ctatacagta    277920
     ggagacatgc aaataggttt ctccctctgc tgatgaccag tcctgacccc atagctctgg    277980
     gagagaagcg ccagccctgg gattcccagg ggtttccatt tggtgatcag gactaaagac    278040
     agaggaccca ccatggagct tgggctgagc tgggttttca ctgttgctgt tttaaaaggt    278100
     gaactagaga gattgagtgt gaatggatac acttgagaga aacagtggat atgtctggaa    278160
     ctttctgacc aggacaccta caaagtttgc aggtgtccag tgtgaggtac agctggtgga    278220
     gtctgaagaa aaccaaagac aacttgacaa cttgggggat ccctgagact ctcctgtgca    278280
     gactctggat taaccttcag tagctactga atgagctcag attcccaggc tccagggaag    278340
     gggctggagt gagtagtaga tatatagtac gatagaagtc agatatgtta tgcacaatct    278400
     gtgaagagca gattcaccat ctccaaagaa aatgccaaga actcactctg tttgcaaatg    278460
     aacagtctga gagcagaggg cacagctgtg tgttactgta tgtgaggcac caggtaagaa    278520
     gacatcagtg tgaacacaga cacagaattt cctgaaataa gggaggagtc tgggctaaaa    278580
     gggcactcag gacccacaga aaacagggga agctctaggg caggtgcaga tggtcatcat    278640
     gggctgcttt ccttgagggt ctgaggcttc ctctgcatct aacagtttcc ctgggagcct    278700
     ctctacattt atgcttctgt ggccacccct gaggtctctg gacattctca tttgttgcaa    278760
     aggcagatgt aagtattgga ggcataaaaa tgcacaggag gccagggagt ctgtagacat    278820
     tgttacccca gaaggtcaat ctcaccacta gtgctggagg agggtgggag tttgatgaag    278880
     ctgccctaag tatcctgtgg tctaagctaa gtccaacgag gccatttgtg cctccctgag    278940
     cacagttgtc catcagagat gtcccatgtg tcccagcagc agccatgtct cagtgtcttc    279000
     actgtgcaca gccattgtct gggaggagct cccaggatgg gtgtctttgg cacacaccag    279060
     gtggcgggtg ttagagtgcg gtgcagcagc tggctgcctg ttctattggg ctccctgatg    279120
     ctggagagat gggaggtgca ttctcaggtc cagcaccctg tttgtgaatt tttatataaa    279180
     accatgattt tacttcattt tctcagatga catagataat taggaacaga acctgcaaag    279240
     aaattgtaat tttcaacttt accccaaatt tattgtttct taattctgtg taagatccag    279300
     acatattatt gccttcctca tgagaaattg ttctatttaa aatgaaatta gttttttctc    279360
     acattctttg tttctgttca agtacagaga tctttattaa agtaagttgg gttctttcca    279420
     cacactaacc ctcacctccc ccagagaaag agcagagatt ttcctcactc tgagtctaag    279480
     ggaggagctg ttcctgcacg actcagagcc tgcagagacc cccccccgcc aggtgcagct    279540
     tcagtgagtc aggtatttct ccttgtgggt gacctccacc gccagtgatt gctgctcagg    279600
     tctaattgtg ggttaagcat taggacaccc ttcaggtgat cacatctcag tcttattctg    279660
     aaaatcacca tgaacagaga tagttcaatg tctattctcc tgacattagt ttctctttat    279720
     tatttggttc caagtatgga gaaaaatgtg acaataaatt tgtcagaatc taacctcagt    279780
     atccactgca ttactctagg agactcacaa attgaacaca aatgagccct ttattctcat    279840
     aaaagtgtat gtatttggga atttcaatgt gttctccaga acctgtgcat gccaacaact    279900
     gtgtttctca gtgcccactt ggcctggtga agccctcaca gaccctctcc ctcacctgtg    279960
     ctgtctctgg attccccatc acaaccagtg cttcctgctg tagctgcatt cataaacccc    280020
     ccaggaaggg actggagtga atccggtgca caggtcatga gggagtgcac attccaaccc    280080
     actcctcaag agtccagtca ccatctccag atccatgtcc aaaaagcagt tcttcctaca    280140
     gccgagctaa gtgagccgca agcacacagc catgtatttt taacaaaaga cacagtaagg    280200
     taaccacagt gggaactcac acccaaacct ccctgtgggg gtgcacagga cagccacagt    280260
     tactcaggac cccaggattc ctcaggacac caaggggcac tcaaggccat tgtagatgcc    280320
     ctcaggtagc caagggttct caggaaacat ggaggaaaac caggacccca aaaggtgctc    280380
     cgtacagcag gggactcagg acaattgtgg ggactcagag caggctcaaa gctcagcttc    280440
     agggcaggtg cagctggggt tgaaaggggc tggatgaggg gttttgtgac accatcatat    280500
     ttcaccacta gacacactcc actttgtcta ttctaacgca tgagagtgta tgattagaaa    280560
     atgatattta tataaataca taaccatagt tagctgtgtc aagttgtcct cttgctaggt    280620
     gtccatagct aggtgcatca gccttgtcca taaggactaa ttccccgcaa ttactggaga    280680
     atctcataaa ttgtggtcaa ttatgtcaga ttcctctctt tttctgcctt cctttctccc    280740
     ttcttctctc tctctcacac agaaacttac atacacccac cccacaacac accaaaatct    280800
     ataactttta ttacctgata tattcaataa acctgattaa tgtgcagctt ttccagcttc    280860
     gttatttatg ctgttgtaac aataagaaca atgtgtttcc tagctgtgta cttctctaag    280920
     ctgagtagca tctttgttta taatacctag aattaaaaac aacccaaaag tcaatcacca    280980
     gcttaactgg taaacaaatt gaggaaaagt cattcattga catactatcc actactacca    281040
     tcaactaatg ttgggtacac tcaacagcat ggttaaattc acaagtactt gtgatgagta    281100
     aaatgagcca aagtaacaaa agtgcataca taagatacaa ctttcataaa ttctatagaa    281160
     caaaaagtaa tctaaagtta cataaaaatc agtagttcac tgtgagtatt gtaggagagg    281220
     ggaaggacta ggaaggagga attatagtac aagacaaaat tttgagggaa ttgacttgtt    281280
     atctatgttg ctagtgatga tgtctatgac ccatttgtaa aattgaacac ttcatatgga    281340
     gattattatt tttaatttaa ctccattaat gacagtacta attatagtag gtataatttg    281400
     gtatcaaaag aattagacag agataaataa aatacatgaa aagtcagaga ctcttgaata    281460
     tacacataaa tgagccctgg gcatctctgt atttttagag aaatgctaga atatagaaaa    281520
     ataatggcat aattttatgt cactaaaaaa gtttatcgaa ctccaccagt catgtggtat    281580
     tagttcattt tcacactggt ataaagaact acctgagact gggtagttta caaggaaaag    281640
     agatttagtt ggctcaccgt tcttcatggc tggggaggcc acaggaaact tacaatcatg    281700
     gtggaaggtg aaggggaaac aaggcacatc tcccagggca gcaggagaga gagagagggg    281760
     ggaagtgaca catactttta aacaatcagt ggttgttaga actcactcac taccatgaga    281820
     acaacatggg gaaactggac ccatgatcca atcacctctc acctggtccc tcccctgaca    281880
     tgtggggatt acaatttgag atgatacttt gatggggata cgaaatcaat ctatatcaca    281940
     tgtccagctc tgtcctggag ttgtttcagg gacccagggt gtccggctga tagaaccagt    282000
     gacaccaagc tcacaccctc agctgtagtt gacaccacac aaagccaaga gattacaact    282060
     aagatttagt ttgaatgtcg tgtctgatga agtcacacac tcagagaaag tgaatatgga    282120
     aaagtttatt atttgcactc tataggtgtc tggtgagtgc agggcaggtc tcccaggaaa    282180
     atctgaaaca gcttgaaaga agaagaaagg agactggctc agcattttta tgatggtttg    282240
     gtcctggggg cagagtgagg cttcccactc acagaaaggg gtttgcaggg tttgaaactc    282300
     cccctggcat cgaatgaaga agctcctgtg atttcaaact agagccacct tgtgtggcaa    282360
     aaaaggagat gatggaggaa tatgctttaa atcatcagca gtcatgcacc caaaaatagt    282420
     gtgacaactt attctatgca gcaggaataa aaataattaa taagaaagaa gataagggtt    282480
     cagtgtgggt ggacaacacg caggtctaca gaaatgagat gactttagaa atataagcaa    282540
     aggataatga aaaaaaggag gggaagggga attaaacagg gtcctggtct gatgtcttgg    282600
     gtagaagctt ctcacaatca aggactacca gctcattctg caggtcttag gtcagccatc    282660
     tgcttaaaaa catcagaaac gccagagaat ctatgaacat gctcagttta acatttccta    282720
     tttgagtagc tttacagtta tgtgaaattc ttaactggtt cttgattttt cttttagata    282780
     caggctctca ccctgtcaca cagtttaaag tgcagtggtg tgatcatagc tcgctgtaat    282840
     tttgaactcc tgactcatat tcttcccatc ttagcctctt gaatatctag aactagaggg    282900
     gcatgccact catcccctcc ttatttttta ttttattttt tcatataaat aaggtctctt    282960
     tgtgttgccc aggctggttt tgattgcctg gtctcatggg atttccctca cttcccttct    283020
     gaaagtggtg tgattataca gatgatccag tgcatctggc ctgaatttat tctttaattg    283080
     taaaatacga acccaataat taactgcctg aatgttttct gcagtgagtt agttaaaagg    283140
     atctgacaag attccttcca atatgattca agagcagtat tgtccactga tgttccttcc    283200
     agtttccttg ttgaagatca caagagtctg tggaaaagag gtagtaaaaa ggccgcctca    283260
     aactcttcgt ggttggagtg ggtaccacac atgcaagcag taggacaagg atgatctctg    283320
     gggtaaagtc tataaacata taggcctttt agctgccaag tcatagggta ataactgatg    283380
     catcctgagg agtggaccat ggtttcatag tgctagtggg agaacccttg gccaagcaag    283440
     ttttacattt tattaaagat ttgataattt taatgtaaag aagacatttt ttaaacgttc    283500
     ccagaagatt gtgagtggta ttgattctgt ctcgtatgaa caatgacagt gctctccacg    283560
     gttagattat gttataaact agaatgaggt agagtgtttg gtgtattaaa tcactatttt    283620
     tttagcttct atgttagttt tttgtttgtg tgttagcatt tgctttaaaa ttctattaat    283680
     cagatctcta gttggtagaa attcatctga aagtttcttc cattgttgtc cattttgata    283740
     ggatttccag aagatgtaag aaccctctct gtttgcaaaa atattccaaa gttgtgcacc    283800
     atctagaaac atagttactt aattctaatt tttaatttat taaaaagttg tgataagtgc    283860
     aaagttttct gccttctgaa ttgatttcat aacacacaga ataatatata ctaaatggaa    283920
     gtttgtacta gtaatacaaa ttactggttc ataacctcta cttttattat tgaggtatta    283980
     tccatcaata tataatctta aatcaatgat ctcagtggga atcttaccta agtaatatac    284040
     aaaatatttt cctgatcttg acataaaata gatgtgaaca cattcttcat attcagccat    284100
     gtctcctgtc tatcacatta tgaaccacat gctaactttg atttacttgg gacttgctct    284160
     aatttcaaac tagttatttt ttatcttcac gcagctggat tattatgtgt ggctatttta    284220
     ccagagtgat aagatacaat actaacaatt ttcactgcag gcatgtctag gcaagcgccc    284280
     tgtgcacaat gaccttagtg ggttggacat tctatgggga ctctcccctg tctgcctagg    284340
     agagttatct gcctcctccc tctatcattt tcctctttga ataagtgcat ctaacccgtt    284400
     agaatacaaa ggccaacctt aactgctccc agctgacagg ggatgctgtt ttgggaagat    284460
     ctcccttgag gtctgtctaa gggacccagt aaaagggagc cattatccca ggcttcactt    284520
     ggatgaccat ttggagttga tgcctgaagg tgagaagaga caaaccgggt tattagaaga    284580
     catgtatcaa aaccaaacaa ggtggtaagg acagtttgaa aaaaaattcc aaggctgctg    284640
     acacacccag ataactggtg gctgtagtta tgcctgctaa gatttgggtg catggggctt    284700
     ggctttcgtt acctcccttg gacttatttt cccaaacaaa gaaacctccg ggttaggggg    284760
     accctattta ttccagtcac ctggcatgat ttgcaggata attgctcaga attaaaatat    284820
     tcgtccagat gtttatatag cccatgcctg tgtttcttct gagctgcagc cagagatcat    284880
     tggttggttc acagcgataa gcagagttag tctaaaatgg aggcaaatac ttaaaactta    284940
     tttcttctct cagttaatgg attctataga gaaaagtagc tactcggcat gggaatgtaa    285000
     aaaaatgagt aaactatgat cttattctga actcattaac aacaaacctg aaaaaccaat    285060
     tgaagagact gtaatttaaa gacaagtgta tgatatgttt tgaaacataa tttttctctc    285120
     tccagttctg atttttgtca gaaactaatc attataggac tgagtgattt gcaaaataaa    285180
     ctttagtctt atggttggtc tgatcatttg cataaagcga agccataata attaataata    285240
     attctgtagg aaaagcctgc aagcacgagg agcttcacag tctaacacta tgagcacatg    285300
     catcctccag caactcactg aatattttca agtcagctgg ttcttagctt aaataacatc    285360
     cagttggtat ctgtcccagg aacactaata tatggttctc tctgcaggcc cctttctcca    285420
     cagattaagg gttttttttt ttctctgtaa tatcaactca gatatgttga atgctttttc    285480
     cttattagtg gtttttcagg tttgttgtta atgatttcag aataagatca ttgtttactc    285540
     atttttttta aattcccgtg ccgagtagct acttttctct atagaatcca ttaactggga    285600
     gaaaaaataa cattttctta tgggtgaaca attaaatagt ttgacatata tttatgtact    285660
     ggtatataat gcagcttgaa atcaaggcat gcctcaatca taaaaatcat ggctaaattc    285720
     tcaaagaatt gtgctgagtg aaagaagcta aggaattaag agtaaatttt atataattca    285780
     ttgtagaaat attagaagat gccactacca taaattaaaa tgaagaagac ttaaattttt    285840
     ctgagaaaat ggtgttggga atgatgcgga tgtgatttaa gtttcagagg aataagaaaa    285900
     agatttaggg attaatttaa ttattcaaaa gttgattgaa gtgccgagtg aatggctgca    285960
     aacatagctc tacatttttc aaatcattcc ctataaattt gaattaatta tttattttta    286020
     tacttgaata aagcaataac aaagaaataa atgaatattt ttgctaaaat ggagcaataa    286080
     aaagactgat attgacagaa gaaatatgac tgacttctga aaatacacac acatgagccg    286140
     tggttctctc tacatattta gataaattac agaaagttgt cataactgat ggggaatcct    286200
     gcagacttca ctaggcatag tccacactgc cctggagttg tctcagggga gctgcctcct    286260
     ccagtggtta gagcacaggc ccaggtaata ggactcattt ttttagatgt gtaattttag    286320
     acacactgca caactgctgt gttctctgtg caaattatct cctgtaaaat gtaacattga    286380
     aacctgcctt aaatatattg tgtaaatatg taaaaataaa atcagattgt gagagctaaa    286440
     tgctaatcaa ggcgcaatca cgtaatatac aattatattt tcctgaatga tggaattaat    286500
     accaatctcc cccaggacac ttcatctgca cggagcccgg cctctcctca gatgtcccac    286560
     cccagagctt gctatatagt cggggacatg caaatagggc cctccctctg ctgatgaaaa    286620
     ccagcccagc tgaccctgca gctctgggag aggagcccag cactgggatt ccgaggtgtt    286680
     tccattcggt gatcagcact gaacacagag gactcaccat ggagttttgg ctgagctggg    286740
     ttttccttgt tgctatttca aaaggtgatt catggagaac tagagatatc gagtgtgagt    286800
     gaacacgagt gagagaaaca gtggatatgt gtggcagttt ctaaccaatg tctctgtgtt    286860
     tgcaggtgtc cagtgtgagg tgcagctggt ggagactgga ggaggcttga tccagcctgg    286920
     ggggtccctg agactctcct gtgcagcctc tgggttcacc gtcagtagca actacatgag    286980
     ctgggtccgc caggctccag ggaaggggct ggagtgggtc tcagttattt atagcggtgg    287040
     tagcacatac tacgcagact ccgtgaaggg ccgattcacc atctccagag acaattccaa    287100
     gaacacgctg tatcttcaaa tgaacagcct gagagccgag gacacggccg tgtattactg    287160
     tgcgagagac acagtgaggg gaggccattg tgcgcccaga cacaaacctc cctgcaggaa    287220
     cgctggggaa atcagcggca gggggcgctc aggagccact gatcagagtc agccccggag    287280
     gcaggtgcag atggaggctg atttcctgtc aggatgtggg actctgtctt cttctgacgg    287340
     ttccccaggg aacctctcta agtttagcat tctgtgccta tgaacgtctt ctctaagtat    287400
     ttgaaagaga ttattttaat atgaagagca gttctcactc gcacaaaatg tggattgatg    287460
     cttactggga tgaaaagtcc tcaaacatgg tcaccacgat cagagtctga gtgagctcag    287520
     ggcttcctgc tgagtctcct cctatcagac caaggacagg gacctcagtg aggttccccg    287580
     tctagaacag tctttatgga tactgattgt gggcggcaag ccacccaggt gccgacgcaa    287640
     gagaccgagg acacgagctg ttccagtaca ataaaatata aaacaagaat agttatacca    287700
     gatatagatc ttagatatga ttatatatga atatcattaa tcattagttg gtagcaatta    287760
     ctctttattc caatattata ataatcctca ctctacaatc ataacctagg aaaagccagg    287820
     ccatacagag ataggagctg aggggacata gtgagaagtg accagaagac aagagtgcga    287880
     gccttctgtt atgcctggac agggcgacca gagggctcct tggtctagca gtaatgccag    287940
     catctgggaa gacgcctgtt gccaagcgga ccatggtcta gtggtagact cagtgtcaag    288000
     gaaaaacacc tgctacttag cagaccagga aagggagtct ccctttcccc ggggagttta    288060
     gagaagactc tgctcctcca cctcctgtgg agggcctgat atcagtcaga cccgcccgca    288120
     cttatccgga ggcctaacag tctccctgtg atgctgtgct tcagtggcca cactcctagt    288180
     cctccttcgt gttccatcct gtacacctgg ctctgccttc tagatagcag tagcaaatca    288240
     gtgaaagtac taacagtctc tgataagcag aaataatatt gtaagctgtt tctctccttc    288300
     tcctctctct ctctgcctca gctgccaggc aggaaagggt cccctgtcca gtggacacgt    288360
     gacccatgtg accttaccta tcattggaga tggctcacac tccttaccct gtccctttgt    288420
     cttatatcca attaatatca gcgcagcctg gcattcaggg ccactactag tctccgcatc    288480
     ttggtggtag tggtcccccg ggcccagctg tcttttcttt tatctctttg tcttgtgtct    288540
     ttatttctat gctctctcgt ctccgcacac ggggagaaac ccactgaccc tgtggggctg    288600
     gtccctacac tgatcacaga caatagaggg taggccagga tcagtgtcat gtaggacatc    288660
     acaggtttca cctctgaacc ttttcctgac actaaatatg caaatcagca tcagcactga    288720
     tctggtgatt cttttgttcc taatccattt aattcctttt tcagtcgttg ttttcatttt    288780
     tccgtttgct tttcctgctt tctgcaaaag gaagattttt ccctgtggtc aaaattccgg    288840
     acctcaagcc ctttcctgac gctcaggtgg gtctcaggct gtggctgctg cagtcacgcg    288900
     ggagaggctg gtgggacttt cttcactcct cgtcactcag ggccctccac tgtgttgcat    288960
     ggagacttat ctggaaatgc aagttgcgac tgagaactga aggggacaag cttgtttggt    289020
     taacatggga tgtggatgtg tttctaattt tgttctgata aactttcaca gagtaacttt    289080
     ctgcactagt catgtgagga agaggatgtg aacgttgtca gaataaaaat agaacaactt    289140
     gtgttataat ctttacaggt gaagctggag aaggtcatga atagagggtt ctcatgcaca    289200
     catccctgat aacaagaact accataaaat tactctgcac aaccacaact ttcaacaaag    289260
     gctaccacaa caataagaga attaatattg tgaggatatc tgccctgcaa ctcccagtac    289320
     aatcttaaac tgattccacc cttgttatta attcttctac ccccaggata attgcctcag    289380
     aacagctcat gtaagtcctc tcatttatcc tttaaaacaa cctttaccaa cctttactaa    289440
     cctgacttcc tttacctacc taaatatgcc cagggataat cccactggaa tgctcatttt    289500
     caaatacata ttatttgatt ttggagaatt tctttctgtc tgatattcag gtgtgacaag    289560
     ctgtagaggg tcacaccact ttcctgtgag atgtagggga tgacaatttg gggggatggc    289620
     tggaaacatc caatatcctc agggtcggcc atcagtaagc gcaggctgga agtctcagaa    289680
     cgagttgaag ctgcttaacc acggaatttt accttctcca gatcagcttt gatggaatca    289740
     gggccaaact ggttatcaat gataatctac ctaacattga gtcaactgat cacagtttta    289800
     ataacctcta ttaaaaattc acaccaacac ttggattagt gtctgatcaa ataactacaa    289860
     agtattttcc agccaagtat accataaaac agaccattac ccatggagaa aaacatttaa    289920
     catgagttct aggtccttac attgttaaag gtgtaaaact gattattttt aaattatgct    289980
     ttttattttt gctattgagt tgtagaagtt tcatttacat tttggatatt aacgcttttt    290040
     tcagatacat gatatattat ccaattctgt gagttggaat tatttcattg ctttgcagaa    290100
     tattttttta atctagtcca acttgttcaa ttctgctttt ttttaaatgt gctttgaatg    290160
     taaaatccag aaaaagattg ctaatttttg aggattggga gttttacagt tgcaggaatt    290220
     tcattgaaat atttaatgca tttaaagtta atttttgtgt ttattctaac ctaaaattct    290280
     taattcttca catgtgaaaa tccagttttc ataacatgct ctttggaaga caccataatt    290340
     tagccattgt atgttgatgt ttctcatgct gaaaatcagt tcgccatcaa tatgtgggtt    290400
     tatatctaag ctctctatat gcatttatgc tgataccatt cggatttatt actctgtgtt    290460
     tgtaacaaat gttgaggact ggaagtgaaa tgcctcaagc tttattcttg ccttattaca    290520
     gatattagac caaaatattc taaccttcta ctagtgagta taataatagc tgtcgctttt    290580
     tttttttttt tgattcagag tttcactctt gttgtgtagg ctggagtgca gtggtgtgat    290640
     ctcagctcac cgcaacctct gcctcccggg ttcaagcgat tctcctgcct cggcctcccg    290700
     agtagctggg attacaggca tgcaccacca cacccggcta attttgtatt ttcagtaaag    290760
     atggggtttc tccatgttgg tcaggctggt cgcgaactcc agacctcagg tgatcctccc    290820
     gcctcagcct cccaaagtgc tgggattaca agcatgagcc actgcaccca gcctcttcat    290880
     ttttttttta ttcatatgtt cattcagcag ccactatgtc ttcccattga tttctttggt    290940
     ttcctcttta ctatcttttt ctttttagta aagctgttac tcctaaggga agatgggagg    291000
     tgggcctgga cagggatttg gtgcattcct ctcttcactc ccagttctta ttgatctctc    291060
     cagtgtctct agaacactgg ttttcctggc attaccgctg cagataattt ctcttgcaat    291120
     gtagtgctga tgaggaggtg tgtctggatg catttcagct atagttgctg ttttgcttac    291180
     cctgacacaa ccgtcccaag gggtagaggc tggagcattt tgtgatgtgt ccccagtact    291240
     gaagaaaaag gcttcaatag caggaggaat tcctcaactg tatacactct gagaatttaa    291300
     acaataactt ctctatcaca ctcaaattta aaccatccaa tgaatatgtc tactttaatc    291360
     gtaacttaaa tgatatttgg cagcctctgt cccagaaaag attatcgtct gctcctgttt    291420
     atttccctgc aagtctttat ctctcttcag atttcagata tcttgtttgt cttataacat    291480
     caaaaatctg atgaatttaa gaaaatgtgc taatttgcag atcagtaagc tttagtagtt    291540
     gtgagaataa taacaaattt ttatgggatg cctacatctc caagctgagt agcatcttta    291600
     tttctaacac tcagaaacta gaaacaatgc aaatatcaag aagatatata gataaagagt    291660
     aatggcatgc taatttacgg taatcatagc catcactaga atcaatacac tgttgatgct    291720
     caatgtggtt gaatcacaag tagttataat gagtgagaag ccacacacat aaaacacata    291780
     ctatataatt cctgtataat aaattcttga aactcaaaac caaagtattc aatatgaagg    291840
     attgactcag aatatggcaa gggaaaaaaa taattgggaa ggaggaattg tagagtaaca    291900
     caaggaaact tttaagtgta atttatttgt tcgttatctg gatggttttt ggggatgcac    291960
     aggtgagcac gagtggaatt acattttgtt gtgtttgttt ttttctgaag agatgtggtc    292020
     ctactctgtg acccaggcag gactgcagtg gtgggatcat agttcaatgt agcttccaac    292080
     ttctggtcac caacgatcct cctgcatctg ccatctaagt agctgaaact acagttgtgt    292140
     gccaccaggc tcagcttgag tacttattaa atcaaacact ttatgcaata tttaatgtat    292200
     ggcaataatg tctcattgag agtattacaa ataaatgaat ggataatttg ttcagtacag    292260
     attgatggaa aatagacact aacatgagga atgtctgaca tttatgaaca tacaactgca    292320
     taaaatgtgt tctcttacat tcattaggta aacacaatag tgcatacaca tcaaaccatg    292380
     ctttcattac aggaaggaag ttctgaaaat gtcactgggg gtgacccacg ctgtgctggg    292440
     cttggttcgg gggcagtcag gcccggtggt gagaagcaca ggcccagata cccaggctta    292500
     ctctgcaaat gtgagctctg gggacattgt accacccatc tgtgcttctg ctggtaattt    292560
     tccatctgta acgtggaaat aacattgata ctacataccg tgatttctcc acatatgtaa    292620
     aaataaaata agatgattgc tgctaagttt aaataagggc agttttcata ggtccattgt    292680
     acctcaataa aattacttta aaataaaaat tacaaataca gttgtaggtt taaagagttt    292740
     atcacagaac aaacttataa taagaaacta tattttcaaa aattgtatca atatctctaa    292800
     actcccccag gacacactca cctgctctgg gctctccact ctcctcagga ttcccacccc    292860
     agagcttgct atatagtagg agacatgcaa acagagccaa acctctgctg atgaaaagca    292920
     gcccagccct gaccctgcag ctctgggaga ggagccccag ctccaggatt cccaggtctt    292980
     tccatttagt cttcagggct gagcacagag gactcaccat ggagtctggg ctgagctggg    293040
     ttttccttgt tgctattttg aaaggtgatt catggggaat gagttgaatg taagtgaata    293100
     tgagtgagag aaacagtgga tgtgtgcggc agtttctgac cagggtgtct ctgtgtttgc    293160
     aggtgtccag tgtgaggtgc agctggtgga gtctgggtga ggcttggtac agcctggagg    293220
     gtccctgaga ctctcctgtg cagcctctgg attcaccttc agtagctcct ggatgcactg    293280
     ggtctgccag gctccggaga aggggctgga gtgggtggcc gacataaagt gtgacggaag    293340
     tgagaaatac tatgtagact ctgtgaaggg ccgattgacc atctccagag acaatgccaa    293400
     gaactccctc tatctgcaag tgaacagcct gagagctgag gacatgaccg tgtattactg    293460
     tgtgagaggc acagtgaggg gaggtcagtg tgagcccaga cacaaacctc ctgcaggggc    293520
     atctggagcc acaagggggc gctcaggata cacagaggac aggggcagcc ccagggcagg    293580
     tgcaggtgga ggtcaagggc tgctctcctt cagggtctgt ggcttcctct catctaacag    293640
     ttccgcaggg agcctcttgt atttacagtg atgtgctact gaggtttcta agtttgtaaa    293700
     gtttattact acaggaggaa ccactatcaa acgcccttaa ggcaggtgtc actaatggag    293760
     aaaggaaagt gcacaggagg ctgggtgagg ctgtggacac tgtctgccta tgattcaagt    293820
     ttcacaagca gtgacggaga aatgggagtt tgatggagct ccctaactac catgtggtct    293880
     aaactaagtc caactaagtc cctgagctct gggtgcccat cagggatccg ccatgtgccc    293940
     ggcagccgcg tgcctttttg tctcctctgc gcccaatcac tgtctgtgat gagcttccag    294000
     gatgtgtgtg tttggcacaa accaggtgat ggacgtcaga cagcagcagc tggtgcccgg    294060
     atcatgggct ccctaatgct ggaggaatga gaggtgcatt ctcagggaca agacattgtt    294120
     gatggatttt tatgtagaaa ccacgatttt acttgcttct ctcaggagac atagaagaag    294180
     caaccatgca gtcagcaaat aattataatt tccacaatta ccccaaatgg ttaatcctta    294240
     attctgcgca gggtcccacc gtagagtcac ctttctcatg aggaatggtt gaatctagaa    294300
     tgagtccaca tgattttcat atttttggct tctgtccatg ttcagagagt tagagtagag    294360
     taagtttgga cctttccaca cactaagcct cacctccccc acagaaagag cagagacttc    294420
     aactattcct gagtgtgggg taggggctgg tcctgcacac ctcagagcct gcagagactg    294480
     ccacgtgcag tgttatagac ttgggtgttt tctcattcag gaggggtgac ctccacggcc    294540
     tccgatttct gctaagatat aactgtgagt gcagaattag gacactatta ggctatcatg    294600
     cctcacttct attctgaaaa tcaccctctt tatagtaaaa gaaaacaatt caatgtccat    294660
     gcccctgaaa gtcatttctt tttaatttgg ttgcgagttt accacatagt gcctgaattt    294720
     attctcagaa tctactgaat tgtttataaa tctcacaaat tgaacaaaag tgaattcttc    294780
     attcttacta aaatgtgtgt atgtaggaat ttcagtgtgt tttccagaag ccatgcacac    294840
     caaccactgt gtttcatggt catttattgg cctggtgagg ccctcacaga ccccctccct    294900
     cacctgtgct gtctctggat tctccatcac aactagtgct tcctgctgga gctggatccg    294960
     ccagtcctca gtcaagggag tggatcaggg cataggtcac gagggagcac aaattctaac    295020
     ccactcctca tgagctcagt caccatctcc agatccacgt ccaagaacca aattttcttt    295080
     tagctgagtt ctgtgaccaa caatgccaca accttgtatt actgtgagag gaacagaaga    295140
     gatgtcagtg tgatcccaga cacaaacttc cctggagagg ggcccaggac caccaaagag    295200
     cactcaggct ccatgaaaac agggcccaag ctggagaacg ggtttcctgt caccctcacg    295260
     tttcaccatt agatactcta cactatgcct atgctgaggt gtgtgtttaa tataattaga    295320
     aaatggtatt tatatacgtg tataatcata tctagtgtaa gaatttgtag tttccacctt    295380
     atgtgaccat taaagacaac atctctcacg ttattcaagg accttataaa gtataatact    295440
     tttacataag atttatctcc ttttttgttt ctccttctgt ctatattctc tctgtctctc    295500
     tcttcctccc tctttctctc tctctctcca tctctctctc tctgtctctc tctcacacac    295560
     aaatacacac acatgcaatc gttgacagta tataggctaa ttaagaatat attcccatat    295620
     gatttaaaaa taacagtttt acaaatattt gcctttaatt tttcaaagaa aattaaatcc    295680
     aaatgccagt gattttttat gtgactttta aaatttaaat tgttaatttt tgttttcatc    295740
     aatgcttgta tgtgtgcttt tgtattcttt cagattattt ggtgccttca cagaattaaa    295800
     catgtgaaac tctccacttg aatttctgtg actcaggact ggtgagcaaa aagttagaag    295860
     tcactctcct gatccttccc tacagctgca gattcctgaa ggtaatgacc tcacagtaga    295920
     ccaaggccct caaaggtgac cttgagttcc agcacatctt gggaggccaa gcggatgttt    295980
     gtgaaagaaa tagtggctat gtgacagttt ctaaccagaa tatctctgtg ttttcaggta    296040
     tccagggtga ggcccagctt acagagtctg ggggagactt ggtacactta gaggggcccc    296100
     tgaggctctc ctgtgcagcc tcttggttca ccttcagtat ctatgagatt cactgggttt    296160
     gccaggcctc agggaagggg ctggaatggg ttgcagttat atggcgtggt gaaagtcatc    296220
     aatacaatgc agactatgtt aggggcagac tcaccacttc cagagacaac accaagtaca    296280
     tgctgtacat gcaaatgatc agcctgagaa cccagaacat ggcagcattt aactgtgcag    296340
     gaaacatcgt gatgggaagt ccacgtgggc tcagagacag actgccatgc aggacacagg    296400
     gggtggcttg gctgaagggg gcactcagca cccacagaag acaggagcag cccagggcag    296460
     gggcaggtgg agttcaaggg ctgctttcct gtcaggttct gtggcttctt ctgcatcaaa    296520
     tgcgttctcc tgggagcctc tctatattta tggttctctg cctaccactg aggcctctgg    296580
     attggaaaag attactacta gaaaaaaaaa ttctcatata cccctgaaga atacatcatt    296640
     aattgaggca gaagatgtca caggaggcca gggaggctgt ggaaactgtg cgacgtggat    296700
     gcacatctga caacaaagac ataaaaaatc agggaacact gataaaacct cctaattatg    296760
     ccatggccct ctctaagcct agtaaagcca ttgatgcctt cctgggccaa acccacccat    296820
     cagggatcac tcctgtgtcc cagcagcaga catgccttag aatctccact gtatgtaatc    296880
     actgtctggg aggagctccc agggcatgtg tctttgcatt aaccaggtga tgggtgtcag    296940
     agaacagcag ctggatgcct ggtctatggg ttccctgatg ttgaagaaat gggaggtgca    297000
     gtctcagagc caatacactg tttatgcatt tttatataga aaccatgatt tttcttgctt    297060
     ctctcagatg acacagagga tcaagaaaac agtctgtaaa caattgtaat gtccccattt    297120
     cttctgaata aatgcattat ttcttaattc tgagtggggg tttgtcagac catgcccttc    297180
     tcctaagcaa ttgttcaatc caggctgaaa ccacatgggg atcacatact ttggtttggt    297240
     tcaggtgcag aggtcttcag tagagcaaat ttggtccttc ccacacagta agcctcacct    297300
     tcctcagaga aagagctgaa attgatccaa ctttgtctga ggtgggagct gcttctccac    297360
     accttggagc ttgcagagac ccctaaatgc agctttattg agtcaggtgt gtctccatgt    297420
     gggggacttc tgctgctagt gatctctgct cagttctaaa tgtgggttca gaattaggac    297480
     acttaggtta tcacgcctca aaaattacca tcattgtaga tagaaataat agtacaatgc    297540
     caattgttgt cacttgtttc ttattattat tggagtataa gtttgaagag acaaattgtt    297600
     accatatatt tgcataaatc taatgtcaga atctcttgaa ttgctcttga acacttacaa    297660
     actggttaga agcagattct tatttttttc tatactagtg tattttggga tgtcaacatt    297720
     ttgtccagaa catgtgagta ccaataactg tgtgtttcag aaatgactgt gagattcagt    297780
     catttctatc gagtgggaaa accagttaac tttgtctttg atatatggat ggaagcccaa    297840
     gatagaggct tgtcagtcat tgtttctgtg ccttctccct tgggaatgtc tgtatttttt    297900
     tatgctctgt cctgggtcta taccccaatg gttcccctac tccatggcac ctgcctgtgc    297960
     ctgtgcatgc ccctgcccac actctccgtg aaacatctgc tttggctcag gaagaacccc    298020
     tcctctgcct tttttcctcc tgcccctggt ccacattctt ctccatccac aggctctcag    298080
     cagcacctcc catccacact aaccctacat ggggtcatac cttgtccacc acttcctctc    298140
     tcttttcttc tgagctttgc cttttattcc aacagcctgt ttccatctca cctggacttg    298200
     tgtacaggaa aggatttacc gagcagtctt gggttgtgca aaccctgaac attcctgaaa    298260
     aagtgctttc tcaagctagg cccatattca gctcctggga gatgatatct gcctttttgg    298320
     catactcctc ctggtagtat tattaaacca cttgattaag ataaggttta catctgtaaa    298380
     actacacatt atttaacata cttaactcat tgagtttgga gtaagtatac atctgcaaag    298440
     ttatccccat catcaaggtc ataaccatag aaatcacttc caaaacattc ctcttatctc    298500
     tcttaatatt gtgattattt tattagttaa aatgtatttt tgtttgtgtt tgtctgtgtc    298560
     tctgtgtgtg tagcctgggt gcacgctata ctagctgttt tcataatgac ttatggtgaa    298620
     tgcctattag tgctcaagag gccggcgtct gagcactgga ggtcaattgt gcaggtgtca    298680
     cacacatctg tgatgggccc aataaacagc cctgggcaca agcactcagg tgagcttccc    298740
     tggtggacaa tgcttcacac atgttgtcat gcatcactgc tgggagaact ggggacaaga    298800
     gactcctcag ggaaagaaca cctgggagct catgcctgga ttctcagggc cttctccctg    298860
     gtgcctttgc tcattttaat tcatattcat tcgctataat aaagctgcac ccatgagaat    298920
     aacagctttt cttgtgtcct ttatttgtac tgattaaggg gcctgaggat ggtcttggga    298980
     cactcaacac aattacatca gagttggaga atgctagaaa gttcctgatt gctgacgcat    299040
     ggctagagga ttttttataa catcaaagga tgagaaagtg cgggataaaa gacattcaat    299100
     gcccagatgg ctacagaatc atatggcatg agatggcatg tacactccaa taaggagcag    299160
     gaagtgaaag taatccatgg aatttagaaa caacagatac agcttcctag gagttgttcc    299220
     ctgaaaaagg aaaagtaaaa ataatgagga acaacccgaa tatgcaattc atatctgtga    299280
     tagctaaaat aatagtaaaa agatgcacat ccagccctgc agtgtgccct gtgagccgag    299340
     gctgctagac tcccattcac caccaaaggg tacagttgtc cagaaacaac acacaacaca    299400
     taagagtgac acagaaggta gcccagagtt tgggctgggg agaggaatcc actagacaac    299460
     tataacaagg aggacagcat ggaaagtggc atcattttgt gatatgattg atatcattaa    299520
     tttatatcat cggattgtga acaaagttag ccaatggact atgagagtga ctgacgtagc    299580
     agcttctctg gcttcgtgtg ctgcagatga gaggagaatg tttgggagga tgctggaccc    299640
     acagctcaca acagacatat gacagacgta gctgatccca acctcaagta ggatgttctt    299700
     gatgagacag cttccctggt gcactggata tcagatcctg tatagtttca gaacttgtgt    299760
     agtttgtgtt gctttggctt tttctctgga tcttttaggt gctaatgaaa tgagagtgct    299820
     tcacaaaaaa atggggcaga caaaggggat agttaagggc ctcctccaag caatgtggat    299880
     atcataaaat aaatgttaaa aaatgaaaga actaaacaag aaggtgctgg ggttgacaca    299940
     aaggacttaa aacagcacta ccaggagttg ggtgcagcaa tgggcatccc ttcaggtcca    300000
     tcagcaagtc agagacctaa acaaatattc cctattcatg ttgaattggc agagtgtaaa    300060
     aatctagaag acaaaatggc caggaaaaat gtgcgatttt gcatgggtga aggtctggca    300120
     agttaatcat cttgaggact gttgaggtcc tgaggtggtc ctgcccttgc ttgatgccca    300180
     ggccgtagtc caactcacac gaaattgcca gggaatagac agtagttttt ttagtagttc    300240
     ttgttatcag gcataagtgc atttgaattt tctcttcatg gcctttcctg gcactatttc    300300
     tcattttttt taacacacat agtttcaact agatttatca ccttcacagg gtcacagaga    300360
     agggtggaag aagggaggcc ctgtatgggt ctcgaagaaa catggaaaag agtggagagg    300420
     gacaatagca gggtgtaagg aattattgag accttactct gcccctccca ggaggctcag    300480
     gccagccttt ttctgcattt gaggttctgg gttataaacg ctgtagactc ctcccttcag    300540
     ggcagggtga caactatgca aatgcaagtg ggggcctccc cacttaaacc cagggctccc    300600
     ctccacagtg agtctccctc actgcccagc tgggatctca gggcttcatt ttctgtcctc    300660
     caccatcatg gggtcaaccg ccatcctcgc cctcctcctg gctgttctcc aaggtcagtc    300720
     ctgccgaggg cttgaggtca cagaggagaa cgggtggaaa ggagcccctg attcaaattt    300780
     tgtgtctccc ccacaggagt ctgtgccgag gtgcagctgg tgcagtctgg agcagaggtg    300840
     aaaaagcccg gggagtctct gaagatctcc tgtaagggtt ctggatacag ctttaccagc    300900
     tactggatcg gctgggtgcg ccagatgccc gggaaaggcc tggagtggat ggggatcatc    300960
     tatcctggtg actctgatac cagatacagc ccgtccttcc aaggccaggt caccatctca    301020
     gccgacaagt ccatcagcac cgcctacctg cagtggagca gcctgaaggc ctcggacacc    301080
     gccatgtatt actgtgcgag acacacagtg agagaaacca gccccgagcc cgtctaaaac    301140
     cctccacacc gcaggtgcag aatgagctgc tagagactca ctccccaggg gcctctctat    301200
     tcatccgggg aggaaacact ggctgtttgt gtcctcagga gcaagaacca gagaacaatg    301260
     tgggagggtt cccagcccct aaggcaactg tataggggac ctgaccatgg gaggtggatt    301320
     ctctgacggg gctcttgtgt gttctacaag gttgttcatg gtgtatatta gatggttaac    301380
     atcaaaaggc tgcctaacag gcacctctcc aatatgacag tattttaatt agtgaaaatt    301440
     ttacacagtt catcattgct tgcttgcctt cctccctcct gtccactctc actcactcct    301500
     tcttttattt tctacttaat tttacaaaat catttaaccc ctttttgaac tattaatagg    301560
     ttatctttgt ttggtgattg ttttcctttc aataatatgt actgaataat tcatctttgt    301620
     gccaattcat aagtattctg gtgtaataaa gacttctttc ataaaaattg gataaattaa    301680
     aataaagata aatttttaaa aacatacgat ctatcaaaac tgaaccataa agaaataaaa    301740
     actctgggtt gggtgtgttt gctcattcct gtaatcccag cactttggga ggccatggcc    301800
     ggtggatcac ctgaggtcag gagttcgata tcggtctggc aaacgtggag aaacgctgtc    301860
     tccactaaaa atacaaaaat tagctggaca tggtggtgct cgcctgtagt cccagatact    301920
     tgggagccta aggccagaga agagattgaa cccgggaggc agaggttgaa ataagccgaa    301980
     atctagccac tgcattccag actgggcaac agagtgagac tccatcccga aaaaaaaaac    302040
     tgaacagacc tatgagtaaa gagattgagt cagtgatttt tcaaacatct caaatcaaag    302100
     aaaagtcaag aacttcatgg cttcactact gaattttatc aaaaatttaa aaaaaaacta    302160
     gaatctctat acaaatttcc aacaaaataa agaggaaaaa atacatgcaa gcttattttg    302220
     gaaggtccta tttccaaagc aaggaaaaga cactccaaat aaataaaatt acaggctaat    302280
     atccctgatt atctagtttc aaaaactctc aagggtggtg agaaaccaaa ttcaacagca    302340
     cattaacaac agaattcacc atgatcaggt gtggtttatc tctaggaagc aatgaagttt    302400
     caacctgcag aaataaatgt gatatatcaa atgaaaatat tgaaggacta aaaccatatg    302460
     caccatatgt ccatgtcaat agatgcagaa agagcctctg tcgaaatcca ctacactcta    302520
     atttttaaaa atctgaacat attatgcata aaacatatat acctcaacat aataaagacc    302580
     acatatcaca agcccacatc taacatcata cacagtgatg aaaattttat tttcctctaa    302640
     gactagaaac tagacaagat gcttcactat caccaatatt attaaacaca gcactgggtg    302700
     gtctagacaa aacaggccag aagaaaaaaa tagaagtcat ccatatagta atgaataaat    302760
     ataaaatata tttttacata ttacatgctc ttatttatac aaagccttaa acactccacc    302820
     aaaaaagatt gaaactaatg aagaaattca ataaagttgc agaatccaaa atcaaactta    302880
     catttcaaga tggcaggtta gaggcattgc tagtatacct cttccacttg gaaggacaaa    302940
     ctgatgtgga gagatgaaca tcgcggactt attttcaaga agcaatgcag gaactgaaca    303000
     gaaacactga aataatctac aaactttctg aaaaagcagg aagctgcagc ctacactgtg    303060
     agtcaggtga agggctggga gtccccagca tttgagggag aacagccaaa aatttcagcc    303120
     agtggtccca agttgaaagt agctctcaac aggggtgtgt aatataacct agggttggga    303180
     cgaactccct tggccagggc ctgggtaggg aagtgttaaa agtgggctct gcaagtttag    303240
     gagccatggg tgcaggagct ggtgccctgc tttgcagcag acaggaaggg gcatggcatg    303300
     aaacccatgg ctgcagtctc catggggaca gcctatgact cctggcattt gcaggtattg    303360
     atcacagatt gactgaaact catctcactg ctgccagtgg aacaccacgg gagtggatca    303420
     gcctcaccaa gtatgtggga actgtgtggg gcctaccacc acctgccact ccccaccccc    303480
     tgcccaaact tctgtgcagc agaggcagcc atggtcccat ctggaacatc atcccagtgg    303540
     cctgagaacc acccctgtcc cccacaccca caggggctgc tgcttgccct gcatacagag    303600
     actcagagcg gaaacccacc tggcccaacc ctcaccttgc tttgtgcagc cacctgccct    303660
     ggcagcttaa cacaaaagac agcatctttt gggagctaca tagccacacc cactgcctaa    303720
     gaatccatag tagcccccat acctgggcaa cacaaggctt gcaaaaatcc caccgctaat    303780
     aatgcagctg gtgctctttt gcaagcacca cctcttagct ggaggccaac caaccatgca    303840
     ttacagcatc tcctagtaga ctaacactgc atcctggaag gagaaaatga ctgtgcgatc    303900
     tcagttatca ccatggcctg caccactttg gatgaccagg agatcctgag tctctccatg    303960
     tgaccagttc attgctacta taaccatcat tcaagaaagc cagtacacaa aggctatcaa    304020
     tatccatgga atttcacaga gtctccttca ctctcctgcc tgccactccc atcagacccg    304080
     gttctgctgc tcactgttga aaaatatgag gacagttcac atcactggat cacttagaga    304140
     catttgccca caccagcctg aagtgtgtca acgtcactgg gcagctagac ctagagaaga    304200
     aacataactc acagtaccat ggctcccagg ttctcctcct cttaggggaa gggagtgcac    304260
     cacattgagg gaacacctca tgggacaaga gaatctggat ggcaggcctt ggacaccaga    304320
     tccctccact ggtgggaaac ttcttctagc agaagtacag ttgcagcact gggctcagct    304380
     gggaaagtct tcagctcttt ctcaacagac agccctgttg cttgtgaaga atcttgaaga    304440
     agaggaagcc tttccccctt gtacaccact gtaggcacac ttggggtctc tcccacaaga    304500
     cctcagcatg ggtgcaacta tagacagcct ttctggaaca catcattgtg actgcatccc    304560
     ccagaaacag cactttctgg attcaggctt gcatgagaca gagagtcaca gttcctctct    304620
     atttggcaca tgaacatttg tacagatgaa aaaacatgcc tgtcttatct gaatagccgg    304680
     aatactggga cagcagtatg tctgagaggt ggataaattt cctactgact tgacaagaga    304740
     gctgaggtgg ttccaaccct tcccaccgat aagacctcag tgggactcac taaaacctct    304800
     ttcagccaac tctgtcaagg ctgggactta atttacccac ctgctttagc cacaactagt    304860
     ttctacccaa ggaaaagtcc tccactgatg tgaagcttga gccatcaagc aaataaataa    304920
     atcactgggg acaaataaaa taaacatgtg catgccacag aggaatgaga taaggtttaa    304980
     gagacctcta ccattctaac cccatagaag acagtgaact tgctcacaga ccgagcagat    305040
     ttctactgca atcaacacat aggaaagcca tcatacaaag attctccata accccggaac    305100
     tcttacagag tcttcacccc tctaaggacc aaaaaccaaa tcaggttgta attaattata    305160
     agcattaaag tctcattctt aagggaaaaa aatccattta aaaacaaaca caagaaacag    305220
     tcaaatcaaa cataaattca ataataatta gaaaatctac caaaatgaga ggaaatgaga    305280
     aaaataactg gggaaaaatg agaaaacagg gtgctgtgcc agtgccaaaa ggtcacaagg    305340
     tcacactacc tctccagcaa taaaccataa caaaaatgga gtctttgaaa tatcagaaaa    305400
     gaaaatcaga atgttgattg ataagctact caagaagata tcagaaaaag gcaaaaatca    305460
     taataaataa atttataaaa ctgttgagaa tatgaataca aatttttaca gagagataga    305520
     catcctaaag agaaaccaat cagaactcct gaaaatttaa aaaaatgcag ggaattggaa    305580
     agggaattac aaaatgccat ggaaagtttg aaaaatagac tagaaaaaag tagcagaaat    305640
     aacaatagac tagaaacagt acaaaataaa agtagaagta aaatattcga ggaaaaagaa    305700
     aaaggaaaca gttaaagaaa aaagtagaaa gaccagacta ttattgaaat aaccaaatca    305760
     gagaaaaata aatttaaaaa gagccaaaag aagtgaaaaa agtctccagg aaatatggga    305820
     ttatgtaaaa caaacaaatt taagaataat tggtgctcct gaagaagaaa aaataataat    305880
     aagtttggaa aacttctctg aaggaataat tgagaactac ttctctggcc tgactaaaga    305940
     cctagatatc caaatccaaa aagttcaagg aactcctggg gaattcattg caaaaatacc    306000
     ttcaccaagc atacagtcat caggctacct aaagtctaca tgaaggaaat aattctaaga    306060
     gcagtaaaac gaagaaaccc atcagaataa tggcagacgt ctcagcagaa acttgacaag    306120
     caagacagat tagggttcta ttttcaaact ccttaaacag aaaaactttc aaccaagaat    306180
     tcttttttat cccgccaaac tgttttataa ataaaaaaga aataaagtca ttttcagaaa    306240
     aacaaatgct gagggaattc atcactatca aaccagcact acaagaaatg ctagaataag    306300
     ttctaaacct tgaaacaaaa ggccaatatg cacaaaaatg gaacctctta aaaattaaaa    306360
     actcacgggg cctataaaac aataacacaa tatcaaagaa taaacaaaat taggtaatga    306420
     catgacaaag agaatagcaa ctcatatcta aatattcaca ttgaatgtaa atcataaaag    306480
     gcatggaaga aggaatttca caaaaataga aaccaggagt gagcaggact agctattttt    306540
     gatctcagac aaaacaggac ttcaaagcaa aaacaattta aaaagacata gatgatcact    306600
     atacaatgat aaaaggatca attcaacaaa aaattacaat tacatattta tatgcaccaa    306660
     acactggagg aactagattc attaaacaag tactgctaga cctaaaaaac tgagagagtt    306720
     agcaaaacaa tcataatggg agattttagt acaatcatga cagtactaaa cagatcttcg    306780
     agacaaaaag tcaacagata aacaatgcac ctaaatgact cactggaaca aatggatgca    306840
     gcagatattt acagaacatt ctatccaaga tctgcagaat atacattctt ctaatcagca    306900
     catgcaacat tctccaaggt agagcatata ctaggccaca aaacaagtct caataaattt    306960
     ttaaaacaat gaaatcatat caagtatctt ctcagaccac agcagaataa aactaaaaat    307020
     catctcgcta aagaactgtc gaaaatgaac aaatacatag aaattaagaa atttgcttct    307080
     gaataatatc tgggttaaca gttacatcaa gatgaaaatt taaaaattat cttaattaaa    307140
     ttataataat gagacaagtt attgaaacct caaaaataaa ggaaaagcag tgataagagg    307200
     aaagtttata gtgccagctg cctgcatcaa aaagtctgaa agagcacaaa ttcacaacct    307260
     aatgtcacac cttgagaaat tggagaaaca agaacaaact aaacatagag ccagaagaag    307320
     aaaagaaata acaaagatca gagcagaact aaatgcaatc caaacaaaaa aaagcaatga    307380
     aacaaacagt tggttatttg aaaaaataaa caaattcatg ggtcattagc tagattaaca    307440
     aagaaaagaa tatcaaagat ccaaataagc tcagaaatga aacgagacat tacaatctac    307500
     accactaata taaaaaataa tttgagacta ctaagttcac cttcatgtgc acaatgtaga    307560
     agacttagag gaaatggaaa aatttctaga aacatacaac actcctagat taaataaaaa    307620
     agaaacagtt actttgaata gacaaataac aaacagtgag attgaatcag taattcaaga    307680
     attgccaaca ataacaacaa tgaaataggg ccaggtgaat tcacagctga attctatcaa    307740
     aaaatttaaa gaagaattgc tgccaatttt gctgcaacta ttttttaaat ttagataaaa    307800
     aagaatcctc cctaaattat tctatgaagc tagtataacc aagataccaa aaccaggaaa    307860
     acacacacac acagacacac acacactcta cagatgaatt tccctgataa atatagatgc    307920
     aaaaatagca aaaaaaaaaa tagctagttg agttcaacag cacatcaaaa agataattcc    307980
     tcatgatcaa gcgtgtttca tctcagaatt gcagggatta tttgaacata ctcaagtcaa    308040
     taaatgtaat acatcacata aacagaatta caaataaaaa ccttacgatt gtctcaatag    308100
     atgcagaaaa agcattcaac aaaattcagc attttttaat gataaaaact gtaaataaat    308160
     gaggcataga agaaacctgc ctcaaactaa taaaagttac atatgaaaaa tccacagcta    308220
     atatcatgct gaatgtgaaa aagttaaaag catttaccct gagaacacaa acaatacaag    308280
     gatgcccaag ttcaccaatt ctattcaaca tagttctgga agttctagcc agagcaatta    308340
     gtcaggagaa aaaaaataag ggtatccaaa tttaaaaaga gaacgtcaga ctatcactgt    308400
     ttacagatca agggattata tatattaaaa ccctagactc ctccaaaata ctaatagttt    308460
     tagtaaatga attcagttaa gtctcagatt acaaaataca tgtacacaaa ttagtagcac    308520
     ggctatatat caacaacagc aatgctgaga gttaaatcaa gaactccatc ccttttacag    308580
     aagctgcgaa aagatagtat atttaccaat atacttaacc aaaagggtaa aagatctcaa    308640
     caaggagaac tacacaacac tgctgaaaga aatcatagat gatacaaaca aatgcaaatg    308700
     catcccctga tcatggattg caagaatcgc tattgtgaaa atgaccataa tgcctaaagc    308760
     aatctacaga atcaatgaaa ttcttattaa aataccaaca ttattttcta cagaattcaa    308820
     aaataataac gctgaaattt atttgaaacc aaaaaaaagc caaaataccc aaagaaatcc    308880
     taataagata aagaaaattg gagccatcac attactgaac ttcatattac accacaaggc    308940
     tgcagttacc aaaacaacat ggcactgaca taatagtagg ctcatagacc actggaacaa    309000
     aatagagaac ccagaaataa agccacatat gtaaatccaa ctgatgcttt gcaaaatgta    309060
     ccaaatttta aattgaaaaa tagacaccac atttaacaaa tggtacaaga aaaactagca    309120
     agccacatgt aaaagaataa aactggattt ctatctgtca ccatataaaa gaccaactca    309180
     agatgaatca ggtactgaaa tataagacat taaactcaaa aattctagaa aacaatattg    309240
     gaaaaacact tttagacatc agggtaggca aagaatttat aactaagacc ccaaaagcaa    309300
     atgcagcaaa aacaaaacta aatttatgga acccatttaa actaaagtgt ttcttcacag    309360
     cacaactaag agtcaatgga gtaaaaagac aactcacaga acgggagaaa atatgtgcaa    309420
     acttcacatc tgacaaaaaa ttggtatatt cagaatctac aaaatactaa aacaagtcgc    309480
     caagaaaaaa caaacgatcc catttgaaag tggacaaggg acataaatag atagttctca    309540
     gaagaagata tacaaatggc caacaaacat atgaaaaact gtgtaacatc actaatcatc    309600
     aggaaaatac aaattaaaac cacaatctaa taacacctaa tcctgcaaga atggccacta    309660
     taaaaagtca aaaaacaaaa gggaatgctt atacactgtt gatgaaaatg taatttagta    309720
     taaccactat ggaaaacact attgagattt cttgaaaagc agaaaggtag atctactatt    309780
     ttttttttct tttttgagat ggagtctctc tcagtcaccc aggctggagt gcagtggtgc    309840
     aatctcatct cattgcaacc tccgcctccc gtgtcaagtg atgctcccac ctcagcctcc    309900
     agagtatctg ggattacagg cacccaccat catgcctggc taattgtttt ttgaattttt    309960
     gtagagaagg ggtttcccca tgttggccag gctggtctcc aactcctgac ctcagctgat    310020
     cctccccgct cggcagccca aattactgaa attacaggcg tgcagcatca cacctggcca    310080
     gatctactgt ttgatccagc aatctcatta ctggagagct acccaaagga aataaagtca    310140
     ttatatgaaa aagacgtgta tatgtatgtt tatagcagca gaattcaaaa ttgaaaatat    310200
     gtgaaaccaa tttaaatgcc cattggccaa tgagttcaga aagaaaatgt gacatcgcct    310260
     gtaatcccag catttcggga ggccgaagag ggcggatcac gagctcagga gatcgacacc    310320
     atcctggcca acatggtgaa atcccgtctc tactaaaaat acaaaaaatt tgccggacct    310380
     gttggcgtgc acctggagtc ccagctactc gggaggctga ggcaggagaa ttgcttgaac    310440
     ccgggaggca gaggctgcag tgaaccgaga ttgcagcact gcactccagc ctgggtgaca    310500
     gagcaagact ctgcctcaaa aaaaaaaaaa aaaaagaaaa gaaaagaaaa gaaaaagaaa    310560
     atttgacata tatacatcat ggaatactac caagccgtta aagggaatgg cataatgtcc    310620
     tttgcagcaa cttggatgaa gctggaggta atgagtataa gtgaagttac acaggagtag    310680
     aaaagcaaaa actgtgtttg cacacttaca agtgggagat gggctatgaa ctggcaaagg    310740
     catacagagt gatataaggg actttggaga ctcagaaggg aaagaatagg atggatacta    310800
     gagagaaaat aactacactt taggtagagt gtacactatt caggtgatga atgtactaaa    310860
     atctcagaat ttattgctat ataattcctc catctaacaa aaaaatattt acccccaaag    310920
     ctattgaaat taaacaaaaa tcaacctaca aaaataagca tcttctgtat acactaaaaa    310980
     atgattattc aaaattaaaa tcaagaaaat aaatctaaat acaacagctt aaaattcata    311040
     aaataaattt taccaaaata aatttaacaa ggatacaaaa tatctgcaca ttgaaaatta    311100
     taatatattg attaaaatgt aagaaaacac aaataaatgg aaggctatct tgtgttcttg    311160
     tgttactgga acatacattg ttaatatgtc catcctaacc taagcattta cagattcaat    311220
     gcaatcccat tcaaaatttc aaagatatgc atttacataa attgaaaaaa aaaacctctc    311280
     tcatttgaat ggctggggtg attgacccgg actatcaatt tgaaatcaga ctactactcc    311340
     acaatggagg tgagacagag tatgtctgga atacaggaga tttcttagga catctcttag    311400
     tattactttg ccatgggatt aagatcaatg ggaaaaaaca acctaattca ggcaggaata    311460
     caaatggccc agatacttca gaaataaaag tttgggtctt cacccagatt aaaaatcctg    311520
     accagccaag gtacttgctg aaggcaaaac aaatacagaa tgggtagtgg aagaaggtag    311580
     ttgtcaatac caggaatgac cacacgacca gaagtgggga ccgtaattgt cgtgagtatc    311640
     tccttaagct gataagcaac ttcagcaaag tctcaggata ttttctccat atcttgttag    311700
     gaatatattt ctgcatgtgt ataactgtac taagaaaata tcttcattgt gccggacacg    311760
     gtggctcaca cctgtaatcc cagcaattta ggaagccaag gtgggtggat tacctgaggt    311820
     ctagaggtcg agaccagctt gaccagtatg gtgaaacccc gtctctacca aaaatacaaa    311880
     aattagcctg gcatggtggc aggtgcctgt aatcccaggc ttctcagaag gctgagacag    311940
     gagaatagct tgaaccctga acccgcgagg cagaggtggc agtgagccaa gattgcacca    312000
     ctgcactcca gcctgggcaa cagactgaga ctccatctaa aaaacaaaca aacaaacaaa    312060
     aatatattaa ttgttttcat tgaatttgtt ttctttttat catgtgtcac aagatttatt    312120
     gacttcctaa cagcatttaa atgttgttaa ctatatgtag tggtatgtat gtaggttaag    312180
     gattagtgca ctttcagttg tatgaaggat agctgtatta tgttaggcat aattatgacc    312240
     ttattattgt ctttatttgg agatcaagca tgattgcagc tagatgtgta ggggtgctaa    312300
     gttgacaaga ggtgggcttt tgatgcttga tactaggtgt caacttgatt ggattaaagg    312360
     atgcctacat ggctgggaaa gtattgtttc ttggtgtgtc tctgggggtt ttgccagagg    312420
     aggctgacat ttgagtcaat ggactggaaa tttgagtttg tcatgttgca tgatcctttt    312480
     aaagtcttat agaacttagt tgactattat tttggtgtag ttcatttgta taaatatttc    312540
     tttgaagttt aattttcttc ctttgtgttt gtctgtcact ggcaacacgg taattgtagc    312600
     cttttagaaa tagcttagaa ggttggcgac tcatcatttt ttaaaaaagt tagagaacag    312660
     ttggcatgac ttttttaaat tctgagagcc attttttatc taacatatag gctatcatga    312720
     agaatattta tagtgtgctt gagaagcatg cgtattacac tgcgcttggt tggaatgttc    312780
     tgtagatgtc tattaggtca gtttgttcaa tagtgttgtt taagtccatg ggcttcctaa    312840
     gaattttttt atggatgttc tatacattat tgagggtgag gtgttgaagt ctcctccttt    312900
     cttattgttt tctatttctc acttcatatc tcttaaactc ttgttaatgc ggttatattt    312960
     ctatttttat aactatgtaa aatagaaata aaataataat tgcttagtaa ttttaatggg    313020
     aaaatcacat aataagaaat tatattttcc caaatgctgc catcaccact aaactcctcc    313080
     aggagtctca catctgctct gggctctgct ctctcctaag gggtcccaca tcagagtttg    313140
     ctacagagga ggagacaaga aaatagggcc ctccctctcc tgatgaaaac cagccctgcc    313200
     ctgaccctgc aactctggga gaagatctct agtccagaat taccaggagt ttggatttga    313260
     tgatcagctc tgtacaaaca tggctcacca tagagttagg gctgagctgg gtttcccttg    313320
     tcattatttt aaaaggcgaa taatggagaa cttgagatat ggagtgtgag tggatatgag    313380
     tgaaaaaaca gtgattctgt gtggcaggtt ctgactcaga tgtctctgtg cttgtaggtg    313440
     tctagtgtgg ggtgcagatg gtggagtctt ggggagagtt ggcacaagct gaatgtgcct    313500
     gagactctgc cgtgcatcct ctgaatccac cttctgtagc tactagatca gctgaatctg    313560
     ccaggctcca ggaaaggggc tgcagtgagt agtagatata atgtacgatg gaagtcagac    313620
     ataatatgca gactctgtga aggtcagatt caccatctcc aaagacaatg ccaagcacag    313680
     gttgtatctg caaatgaaca gtctgagagc tgagaatatg gctctgtatt attgagtcaa    313740
     aggtaccaaa tgaagggaca tcagtgtgaa cccagacaca aaatttcctg cagggaggag    313800
     ggaggaggct gggctgcagt gggcactcag cacacacaaa aggcagggac agttccaggg    313860
     gcaggtacag gtgcaggtga aggcaaaggt ctactttcct tccagatctg tggattcctc    313920
     tgcatccaac agttcgcctg ggcctctgtc tttatggatc tgcgcctacc actgatgtct    313980
     ctgggttagt aaagtttgct actataggag gaaacattgt catttgtcag aaaggcgaat    314040
     aatggagaac aaaagatatt gagtgtgagt ggatatgagt gaaaaaacag tgattatgtg    314100
     tggcaggttc tgaccaaaat gtctctgtgt ttgtaagtgt ctagtgtgag gtgcaggtgg    314160
     tggagtgtag gggagaggca agaggcagaa aagcgcgcag gaggccgggt gaggctgtag    314220
     acattgtcag ctcactatgc caatctcaca acagtgctgg agaaggtggg agtctgatgg    314280
     agcttcccaa caaccctgtg gtctaagcta agtccatcaa ggccgctggt tcctcctgga    314340
     acacagctgt ccatcaggaa tcccccatgt gcccagcagc agccacgcgt tagcatcttc    314400
     actgtgcaca gtcattgttt gggaggagct ccaggatggg tctctttgtc aaaaaccggg    314460
     tgatggtgtc agagagctgt gccgggtgcc tggtccaggg gctccctgac gttggaggta    314520
     taggaggtgc gttctcaggg tgggaacacc ttttatggaa atgtacagaa gaaaacatga    314580
     ttttccttgg tcctctcaga tgacatggag aagcaagcgc gcagcttgca aacaattgta    314640
     atgtccttgt aatttcctac tttatcccaa gttcattgat tcttgtaaac cttggtttct    314700
     gtccaagtac agagatattg attagagtga gtttggttct ttccacacac taaccctcac    314760
     ctcccccaga taaagagcac attgtcctta ctctgagtct gagggaggag ctgttcctgt    314820
     acaactaagg gcctgcagag acccccgtgt acagctttgc agagtcagat ctttccacat    314880
     gtgaggcgac ctccacggcc catgattgct gctcaggtct aattgtggat tcaggattag    314940
     gacatccttt aggctatcac aggtcaatca gattctgaaa aatcactgtt atcatagaca    315000
     gaggtaataa ttcaataccc actcccctga cagcagattc tctttcttat ttggttgaaa    315060
     gtttgaagaa aataagtgac catttattat tcttattcta actttagtat ccactatttg    315120
     ttctagggga ttcacaaatt tcaaaacact gagctcttca ttctcatgaa aatgtgcata    315180
     tttgtgaatt tcaacgtgtt gttcagaacc tgtgcatgcc gacatctgta tttctcgtgc    315240
     gttcttggcc tggcgaagcc ctcacagacc ctctccctca tctgtgctgt ctctgcttct    315300
     ccatcacaac cagtgcttcc tgctggagct ggatccctca gctccccagg gaagggactg    315360
     gagtgaatca ggtgcacagg tcatgaggga gaacacaacg caacccacgc ctcaagagtc    315420
     cagtcaccat ctccagatcc acatccaaaa cacagtttct tctacagctg agctacctga    315480
     gcaacgagta cacaaccatg aatttttaca caaaagatac agcaagggga agtcattgtg    315540
     agcccagaaa caaacctccc tgcagggaag ctcaggaact gcgggaggcc ctcgggacac    315600
     cagggggcgc tcaggacaca catcaaggca ggtgcaagag gaaaaggtgc tggagatggg    315660
     gtttggcatc atcatcatat ttcactgaca cccgccactg tgtttattct catgtacgtg    315720
     attctttgta ttattagaaa atggcgttta tgtaaataaa taaccatatg taggtgcatc    315780
     aagtcgtcct ctcccttttt tttttttttt tttttttttg agtcctgctc tgtcacccag    315840
     gctggagtgc agtggcgcga tctcggctca ctccaacctc cgcctcccga gttcaagcga    315900
     ttctcctgac tcagcctccc gagtagctgg aattacaggt gtgcaccacc gcgcctggct    315960
     aagttttgta tttttagtag agaccggatt tcgccatatt ggccaggctg gcctcaaact    316020
     catgatctca gttgatccgc ccgccttggc ctcccacagt gctgggatta caggtgtgag    316080
     ccaccgcgcc cggcccgtcc tctccatctt atgtggaact tttccattaa gactctgagt    316140
     ccctggattt atttgagaac ctcataaatc atggtcaatt atgtctcttt ctcctctttc    316200
     tcccttcttc actcctctct agctcacaaa ctgaaacaca cacagaattt tctaacttca    316260
     attacctgat gtgttgaaga caattgatta aagcgcagct tttccagttc agccactgtt    316320
     catattgttg taagaataag aacgttgtgt ttctcagctg cgtgcttctc taagatgagt    316380
     agcagctttt ttttaataat acccagaggc tgaaagcaat ctgaatacca atcatcagct    316440
     taatcagtaa acacaaggtg gtaaattcgc tcactggaaa aacaccgact gttacaatca    316500
     ggtaaggctg gatactctca acagcaagtg taaattcaca agtatatatg cttattaaaa    316560
     taagacaaac aaatacaagt acatacatac tgttccactt ttacaaattc tataaaatga    316620
     atgctaatct aaagttacat aaagaaaacc agtagttgac tctggacctg gtgtgaggag    316680
     ggaagggtta ggaaccagaa attgtaggaa atgaagaaga aatgctgacg gtagtggagt    316740
     tttttctatg ttgatgaagg taatagttat gtcactatta atcaaatcgt acacattgtg    316800
     ggatgcctat tatttgtgaa tttcacccta ttaaaatatt acaagttaaa acaagtataa    316860
     ttttgtagaa aattagttag acatagatga ataaaatgta tgataaataa aaaaagaaaa    316920
     tcacagaaca atagcataag gacttcactc atcaaactga agaaatttta aatttctcaa    316980
     cacagaatta aagatttaat tagatataca taagaaatca aagactcccg gatatacaca    317040
     cgaataaacc ctaagtccac ctgtattttt agggaaacgc tagaatacaa gaaaataatg    317100
     tcatgatttc attacataat gggggttaat gaaaccacat caggcatgtc cagctgtgtc    317160
     ctggggttgg ttcagggaac gggtgtgtcc tgtggttagg agacgtggca acaagctcac    317220
     agcatcagtt ctagctgaca ccataaaaag gccaagagat cacaattaaa atgtcatgtg    317280
     gatgtcacat ctgtgggtgc cacacactcc cccatgtgaa tacggaaagg ttaattacac    317340
     tcttcaggtg tctgctgaga gtacagctgg tctctcagga atgtccaaaa tggctcgata    317400
     gagcaagaaa ggagactggc tcagggttcc tgtagtggct tggtggtgtg ggcagagtga    317460
     gggttctcac tcaagaaagg agtttgtggc gtttgaacat ctacatggct tcaaataggg    317520
     taactcctgt gattcctaac tagattcacc tcgtgtggga aagaaaggag atgatgaagg    317580
     aatgagcctt aagctatcag cagtcagata tcaaaacaat agtctgatga cttattctat    317640
     gtagcaccta taaaaatcgt tctggtcacg gtggcctaag tctgtcatcc cagcatttgg    317700
     ggaggctgag gcaggtggat catgaggtca ggagttcaag accagcctgg ccaatatggt    317760
     gaaaccccgt ctctactaaa aatatttaaa aaaaaaatag ttgggttgtg gtggtgggcg    317820
     cctgtaatcc cagctactca ggaggctgag gcaggagaat agcttaatcc cagggggcgt    317880
     aggttgtagt gagctgagat cacaccactg cactgcagcc tgggtgacag agcaagactc    317940
     catctcaaaa aaaaaaaaaa gagtaaaaaa atatcagccc aagatgggtg gacaacaacc    318000
     aggcctacaa aaaaggagaa gactgcttat aagaataagc aaatcataat gaaaattagg    318060
     aggaaaacaa ggagagtgag gtgaaaggca tggggcaggg gattagacaa gggtcctgtt    318120
     ccaatgtctt gtgtggagac ttttcactat ctaaaatcat ccacctattc tgaatgtctt    318180
     aggtgaactg tctgctccaa aacatcagaa ccaccagagg atttttgagg atactcagtt    318240
     taatatctta tatttgaggt gccttacaac tgtgtaaaat gcttagttat tatcagtata    318300
     attagagata gggcctcact gtgtcacaca ggttggagtg cagtggcaca atcatagctc    318360
     actgttaccc tgaacttctg gctcacataa tccttctgtc tcagcctcct gagtagcatg    318420
     gactagagag gtgcaccacc acacacagct gatttaaaaa aaattgtttc atagaaatgg    318480
     ggtctctcta tgttgcccag gctggtcttg aactcctggt ctcacgtgat tcccctggtt    318540
     ttgtttctaa aaatggtgga attaaaggca tgagccacca tgtctgctca gaaatgatta    318600
     tttggttgca aaatattaac ccaagaattg actccctgaa ttttctctgc agcgttttgg    318660
     ttaataatgt ctgataagat ttatttcaat agtttcaaca gcagagtgtt ctcctgatgt    318720
     ttcttccaat agtgataatt tcatggttga agatcacaaa atactattaa aagatgacat    318780
     aaaaaggcaa ccttaaactt ttggtgatgg agtgagtata atacaaaaat tacgttcaat    318840
     attgtgtaac acacgcctgc agtaggagga aggtgagcaa tgagggtaaa atttctaaat    318900
     gtatgaatct tccagctccc aagtcacagg gcagtaactg atgagtcctg agagagggaa    318960
     cgtaatgccg tagtgcttgt gggataactc ttggccaaga gagtttccat attttattaa    319020
     agtggttata atttttaaat ttaaagatgc cattttttca acatatccag aatattgtga    319080
     gtgggattga ttctgtatga tacgaacaat ggcaatgcct tctgtttgga taatattaca    319140
     aaccaggtta agactgagtg tttggtgtaa tgatgcacca gaacattatt ctagcttcca    319200
     tactagattt tgtttttctc tttgtttgca ttagtttcaa atttatatgt aaggtctcta    319260
     gaatgtagga gtttatttga aagtgtcttt tcttgttggc cattttgatg gggcttccag    319320
     gagatttaaa aacctcctct gcctgcagca cagtttaaaa ttctgcacta cagtagaaca    319380
     tctgtattta atcctggtct ttggttaatt gacaagctct agtaagtgca ataacttctg    319440
     ccttctgggc tgattttata tcaggtagag gaatatatcc taaatgaaaa tttgcactag    319500
     taatatatat tattaattaa taaccactac ttttattatt taagtgttat taatcaagaa    319560
     ctaatgttaa atcaagggtc tcaatgggaa tattacctaa gggatacaca agatattttg    319620
     ctaatctaga taaaatagat gtgaacactc tcgtagtatc cagccatgtc tcctgtctgt    319680
     cactttgtta accacacagt aaattcactt ggcacttgtt ctaattatct aattagtcac    319740
     ttacttatct caaagcctcc ggattattat gtgtgactat ttcagcagag agtgaagaaa    319800
     gacaacccag gctgaccaaa caatgaggaa aatcacaacc tgatgaaaca ggaagcctgg    319860
     atgtgagtgg ctccaggatt gaataatttg acagctcatg tgcccaggct gtttcttgtc    319920
     tcaattttcc acactgatac atccagaaag gcagcttcac cccccagggg actcctatca    319980
     tgctgtgaca tggtgacaac attcccaagg ctcacacaca tatgttaaat attggctgga    320040
     aaaggggcag agacatttct ggaattctct tctgaaggac catgaatgcc tcaaccaacc    320100
     atctcccctg cctccatgac cagaaatgca ccacgtgccc acacggatat tcatccctga    320160
     tggggataag actccattga tgaggctgac tattttatca tataaaatta ctaaagactg    320220
     atttcagggt ttcaaaaact aattgaactc tgttgttcta tgtccaccag agattacaaa    320280
     tcttccaatg atgccttctt tgttttttgt ctgcttgact ttgtctcttc aacttgttct    320340
     gtaccccaga gaatctcttt cagctccctc aggtgcattc aattgtttta tttaactgac    320400
     aatttctaaa tcggttaaag acattaggct aaagactcca tattcctagg tccatattcc    320460
     tttttccata ttcctaggaa ggcattgtga cccagagtct gggcatgacc ttgtgagtgt    320520
     tcctgaccct cctccatatg agatgctggt ctgggtgttc ttgccccttt ccctggggta    320580
     gagtcctcct gttttcccca ggtgctccct cccacagctc tagtgttctc agtcagtgtc    320640
     atcaccttcc agatcttctg ccctgccctg cagactaagg ctctgattcc ataagcaaga    320700
     taggggagct gctcctcaat agatctttgg tgaggatctc tgttcccatc tcaattcctg    320760
     tagggttgaa ccagtgttcc taggattctg gtttcagtag cttgtccctg cagagtaaat    320820
     tcttagttct gtagggggat taagggagtt gggtctgaat acattttaga tgtcgaggat    320880
     cttgttctct cccagaaaga cacttcggga aagtaagact ttagtaactg tcccctttct    320940
     tggggaaagg gattcaagag gataggttgc ttttgggcat gtggtccctt aaaatttcac    321000
     actaaaaagc gtttcccaca ctcaatttca agcagcccaa tatatatttg tattttttct    321060
     tggaacagac aatattttat attccagact ctgccttagg taatttcaaa ccctggcttt    321120
     gttactctct acaagaaatt gcttctccat aagcttcaga tttgttgtgt gttttgaaat    321180
     tttatcagaa atattaaaga aaattggcaa aattccatcc tccatgttta tctgttattg    321240
     ttgatgcagt tgtaaaaaat aagaaaaata cattcctttt ttctgtacat ttccaagctt    321300
     agtagcaaat ttttagtaac acccagaata ataaaaaatt caaatattgt ttagctgctt    321360
     aataggaaaa caaattatag taaatttgtt tgctagaatg ctacccagca tttataataa    321420
     gtaaacattt gatataccca actacaaggt aaaatatcca tttatgctaa ctaaaataag    321480
     ccaaacaaat gagaatgtat actatgttat ttcattttta taaattctga aaaattaaaa    321540
     tgaatttgca gcaatgtaaa gatcagtagt tgccagggaa atggtagaag aaagaaagga    321600
     aaaggagaaa gaatacagaa gaacaaaagg aaatgttaag aattctcttg tccaacttga    321660
     taaggatgac ggttacatca tttttatcaa ctgtaatctt taaatatgtg aaagtttatt    321720
     atctgtaaac taaacttata aactttatta caagcaaaaa ttgaaagtta gacaagaagg    321780
     agtgatagaa agagaaaatg tatattaaat ttcagaaata tttaagaatg tatctgcctg    321840
     aaccctagtt ctcaccatat ctttaggtga atgctaaaat gcagcaaaat cacgcatgtt    321900
     ctcactacag aaagtgggtt ctacaaacca cactcggcac atttagcttt gtcctggagt    321960
     tggttcaggg agttattggg gccagtgatg aggagcacag gccaagatac cagcgatcac    322020
     ttatcccaaa catgagctct aacatacaca cttagtccct tttccgtgtg tggtttactt    322080
     ccacatctgt acatggagag accactgact gacaaaatat aatttataca aatatgtaaa    322140
     attaaatagg gtgatcagtt caaggtgttt atcacagcat aattttacaa taagacagca    322200
     tatttcccaa ataccatcat tgtcaccaaa ctccttcaag gcacagtcat cttatctggg    322260
     ccccgtcctc tcctcaggtg tcccacccca gagcttggta tatagtagga gacatgcaaa    322320
     taaggccctc cctctgctga tgaaaatgag cccagccctg accctgcagc tctgggagag    322380
     gagccccagc cgtgagattc ccaggagttt ccacttggtg atcagcactg aacacagacc    322440
     accaaccatg gagtttgggc ttagctgggt tttccttgtt gctattttaa aaggtaattc    322500
     atggtgtact agagatactg agtgtgaggg gacatgagtg gtagaaacag tggatatgtg    322560
     tggcagtttc tgaccttggt gtttctgtgt ttgcaggtgt ccaatgtgag gtgcagctgg    322620
     tggagtctgg gggaggcttg gtacagccag ggcggtccct gagactctcc tgtacagctt    322680
     ctggattcac ctttggtgat tatgctatga gctgggtccg ccaggctcca gggaaggggc    322740
     tggagtgggt aggtttcatt agaagcaaag cttatggtgg gacaacagaa tacgccgcgt    322800
     ctgtgaaagg cagattcacc atctcaagag atgattccaa aagcatcgcc tatctgcaaa    322860
     tgaacagcct gaaaaccgag gacacagccg tgtattactg tactagagac acagtgaggg    322920
     gaggtcagtg tgagcccaga cacaaacctc cctgcagggg cgcacagagc caccaggggg    322980
     cgctagggac cgactgagta cgggacaggt cccaggagca ggtgcagggg gaggtttcct    323040
     ttttccttgg ctggaaaagt cacctttatc ttcccaggac tcgagccttc taggctgtga    323100
     tattttatta cttgtattta ctgttcattt attatcatta gtttttaaat tttggtaatt    323160
     tttacaactc tatggatata tttttaagtg tatactttca agaaataaac attcctaatt    323220
     atttgcactg attctcccag agttttatta acatttgttg acatcagcaa ctacatagct    323280
     atagggacaa acacttttaa cgatagacag ttgtttaggc ctgaaacccc gtttatacta    323340
     aaaatttaca aaaattagcc tggcgtggtg aggggcgcct gtaatcccag ctactcggga    323400
     ggctgagcaa ggagaattgc ttgaatccgg gaagcggaag tcacagtgag tggagtggac    323460
     tgccactaca ctccagcctg gcgacagagc gagactttgt atgaaagaaa gaaaagaaaa    323520
     ggaagaaagg aagaaaggaa ggaaggaagg aaggaaggaa ggaaggaagg aaggaaggaa    323580
     ggaaggaaaa gagagaaaga aagagagagg aaggaaggaa ggaaggaagg aaggaaggaa    323640
     ggaaaagaga gaaagaaaga gagaggaagg aaggaaggaa gaaaggaagg aaggaagaaa    323700
     agaaggaaag aaagaaaaaa gatatataaa cacgcagacc tatgcatata accataggga    323760
     tttatattaa acattacaat aaaataattc taaaaatgtg tcctaaggaa tcaaacataa    323820
     tgatgaagta aatataaatg ttagagtaat ttataattga tttgttattt ttaatcgttt    323880
     acttgaattt atttctattt gttcatttaa aagtagtata ttggtcattt caagagagct    323940
     aacagtaaat ttcagatgtt gttgttacga tatatgataa gaatttgagg tggtgaatga    324000
     taattatctt atttctcaat tatcttagtt attccatatt gtattcacaa atcataacat    324060
     tgcttcttac cttataaata taaacaacca taatttgtga aattacaata attttcttta    324120
     ttttaatttt ttaatttatc ccagatcatt atctttttct tcgcttccag atctcactgg    324180
     atcatctcga gggcccatcc tcacccctgt ctcccgaaga cttctggaga ggctgcagga    324240
     cgggcagaag gaggagcccc gtgtgagtcc acacgacctg gagcctccct ctccttggat    324300
     taggccatct cctcgggatc acagggctct tcattatcct taccccgctg ttgtaccaaa    324360
     caagcaacat cacacttcaa ttcatcaggg tttgctttaa ttttctaaat catcgtgaag    324420
     gtgataattt taacagtaac gtatcacaac caaataggat aagccctttt ccatggaaca    324480
     gggtttctta tcaggatata catgtattat ggattctcaa tttatttgta gatgagatgc    324540
     tattatctcc atattgtaga tgattctgct aagtcatctc ttaaaattaa tttttccaaa    324600
     gactcaaaca aataaatgat ataattacaa atttcaggtg taatagctga gccaacattg    324660
     agattatatt aacatttaga acatgaactt gcaaatattg ttattttcct gtcagctgtc    324720
     cccaattgtg attttataca gaatattaga aatttatcct gataaatcag gttaaaatat    324780
     tatatcacta gtttattaac ttttataact aataatataa aatgttccac atatttttta    324840
     gccatgtttt acttacccgt gatatgtgta tttattaatt tttcatttta agatgccacc    324900
     tttatctttc ttatttctgg gattttattc tagtagatag agctgaatgc attttaataa    324960
     ttatcataat aatcacattt acattttgca ttttaatttt catatacatt ttattaatat    325020
     tttaatttca atataaaata ttttcctaca aatgtcaatt tttgattttt atgagataaa    325080
     attaacctat acaaaataca tatattttca atgtacagtt tgagggtttc tggcaaatgt    325140
     gcacacattt gtctccagca tctaagttat gatgaggagc aggtccatct ccacaacaag    325200
     tgtcctcttc ggtgcttcca gtcagctctc acataaggaa ttttttttca aatttaatat    325260
     agatacagag ggtaaatgtt tggatttgtc acgggggatt attgagtgat gctgaggttt    325320
     ggaatacaga ttccaccatc ctctccctcc actctccagc agtccacagt gtctatcatt    325380
     ctcataatta tgtccatgtg tgctcaatgc tgaggtctta cttaggagaa tatgtggtat    325440
     tcagttttct gctcctacat taatttgttt aggattaagg gccccagctt ctttcatttt    325500
     actgcaaagg acatgatttc attctttttc atggctgtgt agtattatac attgtagatg    325560
     taccgcattt tgtatattca gtctaccaat gatgtgcatc tggtttgatc tatgtcgttg    325620
     ccactgtgaa tagcacagca atgaacatag atgtgtatgt gtctttttgg tagaattatt    325680
     tgcttacttt tcagtgtata ccccgtggtg ggactgctgc gtaaaatgat atctctgttt    325740
     taagttcttt gagaaatctc cagtctgatt tccaaaatgg aaacaccaat ttatattccc    325800
     ctcatcagtg tatgtgttct cttttctcca cagtcccagc agcatccatt gttttttgac    325860
     tttttagtga taaccattct gagtggtgca tggctgcaca cctacagtca tctcatcttt    325920
     gataaggctg atgaaaacaa gcaatgagga agggactccc tgttcaataa atggtgctgg    325980
     gacaactggc taggcatatg atgaagattg aagctggata tctactttca acatgtataa    326040
     aattaacaca aaattcatta aagttttaaa tgtaagacct caaaccataa aaatccttga    326100
     agacaaccta ggaaatactc ttcttgacat caggtttgac aaaaaaatgt tggctgagta    326160
     tccaaaacca attgcaacaa aaagaaaaat agacaagtgg ggcctaatta actgaggagc    326220
     tcctgctcag caaaacaaac aaagaagcaa acaaaactaa cagcacagta ctcagacaac    326280
     ctacagaatg tggaaagata ttcacaaacg ttccatccaa caaagccgta atatccagaa    326340
     tctataggga aattaaacaa atcaagaagc aaaaaataat aataataata accccattaa    326400
     aaatgggctg atacggtttg ctgtgtcccc acccaaatat caacttgaat tgtatctccc    326460
     agaattccca tgtgttgtga gacggaccca gggggaggta attgaaccat gggggcctgt    326520
     ctttcccgtg ctattctctt ggtagtgaat aagtctcacc agatctaatg agacttatca    326580
     ggggtttatc tgatgggttt atcaggggtt tccgcttttg cttcttcctc tttttctctt    326640
     gccatcacca ggtaaggagt gccttttacc tgccaccgtg ttttggaggc cttcacagcc    326700
     atgtggaact gtaattccaa ttaaacctct ttttgttccc agttttgggt gtgtctttgt    326760
     tagcagtttg aaaatggact aatgcgtggg caaatgacac agacacttct caaaggaata    326820
     catacaagtg accagcaaat atatttttaa aatgtttaac atcactaatc atcagagaaa    326880
     tgtaaataga aaaatgttct gatttctgtc actataggtc cattttcttg ttttgaattt    326940
     catatacatg gaataaaata ttatagctca tttttgtaag aagcttttac tatctgtgag    327000
     gttcattcat gtgatagcat ctatcaaggt tttgtcaaca tatgtataaa tatgtatgta    327060
     ctcatacaca tatagatatt tcatatctga atcagtccat tgcattaata aatgacagat    327120
     tattaaataa atcagttcat taagggaata agtgacaata tgtatatcta ttttcctgtt    327180
     gatggaattt aaatttgttt ccaatataaa tatcataaac aaaactgtca tacatatctt    327240
     tgtacaagtt cttctgttta tattcacaca tttttattga taaaatatgt tgaaatataa    327300
     gtatgcatta taccttttca gctttatgga gctatcactg acaaataaaa ttttctgtat    327360
     ttaaggtaca ccaattgatg tattgatatt cttggggaaa tgctcataat gatcaaggta    327420
     attggcatgc ctatcatctc agagagttaa cattttatgc ctttaattta ttgtgtatgt    327480
     gtgatgaaat cacctaatat ctacttgtct ggcaaaagat atgtttataa tgcaacattc    327540
     attagtatag tcacattgct gtaggtttga tctccagaac tatttcaacc tgtgtattag    327600
     tccattctca ccctaatgta aggaactacc tgagactggg aaatttatga agaaaagtgg    327660
     tttagttgac tcacagttct gcaggcttaa cagcaagtat tactaggagg tatcaggaaa    327720
     cttacagtca tcacagaaag tgaaggggaa gcaaggaccg cttcacatgc tggcaggaga    327780
     gagagaaaga gcaaggggag atgcaccacc cttttaaacc ttgagatctt gtgagaactc    327840
     tgtcacaaga acagcaaaag ggaagaccgc ccgcatgatc caatcactcc ccatcagaca    327900
     cctgctacaa cacttgggga ttaaaatttg acatgagatg tgggtgaaaa cacagagcca    327960
     aaccatatca ttccacccat ggtctatcag aaatctcatg tccttctcac attgcaaaat    328020
     atcattatgc cttctcaaca gtcttccagg cttaactcat ttcagcatta tcacaaaaat    328080
     ctatagtcta aagtcacctc tgagacaagg taaatttctt ccccctataa acctgtaaaa    328140
     ttaaaaagaa gttagttatt tccaagacac aatggaggtg catgtactgg gtaaatgctc    328200
     ccattccaaa tgggtgtcat tggccacaga aaaggggcta caggccccat gcaagtccaa    328260
     acaccagtag ggcagccatt aaatgttaaa gctacagcat aatttccttt taccccatgt    328320
     ctcacatcca ggacacactg atacaagggt tgggctccca aggccttagg aagctccacc    328380
     ctgtggctca acgtggtaca gtccccatga ctgctttgat aggctggcat tgagtttcta    328440
     tggctttaca ggcacagcat gcaagctgtt ggtggatcta ccattccggg gtctggagga    328500
     aggtgcccct cttctcacag atccactagg catctagtgc ctagtgccca gtgggtactc    328560
     tgtgtgggag atccaacacc acattttcct tccacactgg cctagtagag gtactccatt    328620
     agggctcagc cctgcataag acttctgcct gaatacccag acattttcat acgtcatctg    328680
     aaatctaaga ggaagctccc aaacctcaac tcttgcctta tgtgcacccg cagactcaac    328740
     accacgtgga agcaaccaaa gcttagggct tgcaccctct gaagcaatgg cctgagctgg    328800
     accttggccc ctttagccat ggctggcagg agagggacag ggatgtccca aggctgcaca    328860
     gagcagtcgg gtcctgggcc tggaccatga aaccattttt ctctactggg cttctgggcc    328920
     tgtgatggga gggaccgcag caaagatctc tgaaatgctc tcaagatgtt tttcccatcg    328980
     ggttcctcat tacttatgca aatttctgca gcccgcttga atttcttccc aggaaatgga    329040
     ttcttcttgt ctaccacatg gtcaggctgc aatttttttc aaccttttat gatctgcttc    329100
     ccttttaaac ataagttcaa atttcagacc atctcttaat gaatgcatat gacttacatt    329160
     ttcagaaaca gccagggcaa atattgaatg ctttgctact tagaaatttt ttctgctaga    329220
     taccttaagt catctctccc tagttcaaca ttgcacagat ctctagggca ggggcaaaat    329280
     gccactagtc tttttgctaa agcatagcaa gtgtgagctt tactccagtt cacaagaagt    329340
     tcttcatctt agcatctgag accacctcag cctggacttt attgtccata tcactatctg    329400
     cattttgttc aaaaccattc agtaagcctc tagaaagttt caaacttttc cacagcttcc    329460
     tgtcttcttg tgagccctcc aagctttacc agcctctgtc cattacccag ttacaaagtc    329520
     acttccacag ttttaggtat cttaatagca gtgcccactc ctagtgcaaa ttttctgtat    329580
     tagttgattc ttacactgct gtaaagaact acctgagact gggtaattta tgaagaaaag    329640
     agtttagtag actcacagtt cttcaggcta aacaggaagc attactggga ggcatcaaga    329700
     cacacaatga tggccgaagg tgaaggggat tcaaaaatct tctttgcaag atggcaggag    329760
     agagacagca agggagacgg gaggtgccac acttttaaac catcagatct cttgagaact    329820
     ttatcaggag aacagcaaag aggaaggcca ccccattacc caatcacttt atattaggcc    329880
     cttccttcaa catgtaggga ttacaatttg gcctgagatt tgagtgggaa cacagagcca    329940
     aactgtatca acctgcataa ctgaaagtta taacctttga ccaacatcag ccaatttttt    330000
     cctcctccca gcccctggga actactattc tactttgctt ccaagagcat gaatatttta    330060
     gattctacat ataaatgagt tcatgcaaca tttgactctc tgtgtctcac tccacttagc    330120
     aaagtgttct ctatgtgttg tacatgttag aatttcctcg tttttaaagg cagaataata    330180
     ttcactttta ggtaggaata agccacattt tatctgttga ttcatagatg gacattgacc    330240
     tattttctat atctaggcta ttatgaataa tctcacaata aacatatatt tgtcacactc    330300
     actttatttt ctctagatgt atactcagaa gtgggtatat tctatgttca attcattgag    330360
     taatcttgat gctgtttttt cataatggct gtactaattt gcattttgtt ccaaaccata    330420
     catggataac tttgtaccac atattcaggt ctttgtttaa gtcttaaatc catttttagc    330480
     tgatttatat gtattgtgtg agataaggtc catttttttt cttccgcata tggatgccca    330540
     gttttcacag cacttgttga agagactgtt ttttctctat tgtgtgttct tggcagttca    330600
     tcaaagatca gtttattggg aagaaattgg tggacttcca gattgtctgt aatgttctgt    330660
     tggattctat gtctgtttaa atgtcagcat tatactgttt tgatttacat agatttgatt    330720
     ttgaaattat agaatatgat atattcagtt atattttacc caaaattatt ttggctattt    330780
     aaagcttttg tatttttaca taaattggag aactttttaa tatttttgta aaaccatgcc    330840
     atggagatta tatatttatt ttataggcat ataatagata tacctaattt atgggaacat    330900
     gtaatatatt gatgcattta taaacgtgta aagatcagat caggattggt atatctatca    330960
     cattaaacgt gtatattttc tttatgctag gcacatttga acaactctct tctggctatt    331020
     ttaacgtata cattagatta tcgttaacta tagtcacctt actcatctat caaacattta    331080
     ggttgtattt ctcctatata actgtatatc tgtcttcagt aatcatcctc tctttatcct    331140
     cttctctctt gtatccaacc aggcttctgg taaacaacaa tctactctct gtcttcagga    331200
     aatccaatgt tttagttgtg acataaaagt aagaagatgc aatatttgtc tttctgtgtt    331260
     tggtttattt aacttaacat tatggcttcc acttccattc atattgttgc aaatgacaag    331320
     atatcaacat ttatggctga ataatattct attgcctaca tcgattatat ttttatatcc    331380
     atttctccac ctacagacac tgaggttgct ttcatatctg ggctattgtg aatagagctg    331440
     caagaacatg gagtgcacat gcctttatga ggcagtgatt ttatcttctt tagaacatac    331500
     tcagaatagg atttgctgag tcatcaggta tttctatttt tcttttattt tggagtcttc    331560
     atacaacttt gcacaatagt ggaaatacaa acggaaaaac cattataaaa agcagtttca    331620
     gttttgaggt atgatccaaa aacacacaac atttcatcat gattctcatt gggagtctct    331680
     aatgaatctg gtggaaaggc aggaagtttt ctaaccttgt taagaaaatt atgagtttgc    331740
     acatttttct ttttgaggct ggaaattggc atgattcaag atgcgtaatg ttagcaggtt    331800
     tcagaatgat atagcgaaaa tcataatgag aaggcaagag agagaataag agaaagacag    331860
     gaaaacaaag aaacaaataa atttcacaag agaagaaact gctggatgtc agtgttgggt    331920
     tttgttccta gacatatctg tactgagtga gaaaccatga agccaaggga agagtctaga    331980
     acttgttcaa gtgaagcatc agcatatttc tccaaggcac ctattgtcac cccatcacaa    332040
     gggtgatgag tttttgaata ccattaaata tgagttccta gtaagtaatc atgtttccat    332100
     gaaattcagt taaaatgcaa agggtgtgtc cagatcactc atgcacaaaa atacaaaatt    332160
     tgcctttgca tttatggctg catagaggta aagtccattg agacttcagc aggttacaga    332220
     gatctgttaa gttttggatt cccataggag agtgcctctt agtgaaggtt ggactattaa    332280
     ttaagtatgt acaaattccc tctatgggag tagtgttcca tttgtgtgga atttttaatt    332340
     ccttattgaa ttgcaaaaca aatcccaagg actgactcca tggagtttta ctgtagtgtt    332400
     cataaaaata tttaataaga ttccttccag tgtttcaagg aaagtttttc tctaaaattt    332460
     tttctaatac ataaattttc ctgcctaaaa ttgaaacagg ctgttgttaa aaagacatac    332520
     tggaatggca gctttaatct gttggtgata ggaatgagca taatacatga atctctttga    332580
     gtgttctgcc tcatccaaat actgtatggc aaaggctagt attggaggta aaatctctaa    332640
     atgtatttgt tccagccatc aagtcatggg gcaataacag atacatcctt gaggcagagg    332700
     accataatgc cacagtgcta gtaggagtac atgtcgccaa gggagttcaa gggttcctta    332760
     aaattgtgat aattttaatg taagaacacc ttattagaga cagggtttca gcatgttgac    332820
     caggcttggt ctagaactct tgacctcttg atctgcccgc cttggcctcc caaactgctg    332880
     atattacagg agtgagtcac tgtgtctagc cagtccattt attttcttct gcacatggat    332940
     acccataaac ttatgtggaa gattgtggga ggttataaca gtgtcatact ttagtatcag    333000
     ccatacctca cagaatcatt ttataatagt tcctttcatg ggtgtacttc actcagttca    333060
     tgcaaaacct ggcaggtcac aggctaaaaa cacaaaatac cgaagtgttt aaccaactga    333120
     aagggctgta gccttttgtc aatgaattat ttcaagacac ggagaacaca tatagattat    333180
     aaataaaata ctttcacatc cccttaaggg tggaaattga ggaaatttta cctaaatgtg    333240
     tccaaagggc cctgtggctt ggtttctgtc taggtcccaa tgatagggtt ctcccaggat    333300
     tttgtggctg tcttgttaca cttcatcaag aattaacctc tgctgtttcc tcaaagtgtt    333360
     taattggata atgaatttgt ctataaattg aagagttgaa atacatcaaa tattaatttg    333420
     taataatctg gcacaaatta tctaagcaaa gtcaataact agatgttttt tcatttattt    333480
     ttatttaaaa tcaggaccta aagttatgct ttaataacat ctgtgaccct ctcagcagtt    333540
     ttctcttctg aggatatgat ctgctgtggc agttttctta gcttcaatgt tacctctttt    333600
     tgcaatgact accgtcttta tatttgccag gaatctggga taaaggagtg cttctaagag    333660
     ttccctaact tgcccatttt ggtgggtgtt ccggaacata tgagatgctc tgttgttaac    333720
     aaagcatccc aaagccatgc actgccctaa aatgtgtttg tttcctagtt tgacaaattg    333780
     gaagttctaa taaatacaat cacttctgcc atctgggctg attttacatc agatagaggg    333840
     ctgtattcca aagaaaagct tacattagta atagcaattt tagtcaaaag cctagagttt    333900
     tatcattgag gtgcaattca taacaaataa tattaggtcg aggttctcag tggcagtgtc    333960
     taaatctctt aggtgtacag ggtcttccct gttaacatga agcatttata agcacagtca    334020
     tagtttccag ccatgcttct ccctgtctca ttatcaccac aaactatggc ctcacctgga    334080
     acttgggtta atttccaaat aagtaatttt ttagtcttta tgcctctaga ttattatgtg    334140
     agaaagttaa cattcagtag aaagttaaaa agaacatttg aactgactaa acaacacaga    334200
     aaatcaagaa taaaattcaa agcctagatg tgagaggctc caggcctgga taatgcaata    334260
     gttcatgtag gcaggcagtt tctttgccca gttctacact gatacaccca gaatgtcagc    334320
     ttcatgccag atttgactcc tattatgtag agacatggca atacattctc aagggtcaca    334380
     tgaaataata tgaaaattgg tgggaatagg ggaggagaca actctgcaat tctcatctga    334440
     aggaccagga aagcctggac agaccatctc cccagcctcc gtgactgcac cacgtgccca    334500
     catggacact catccctgat agggtaagaa gactccattg atggggctga gcattttatg    334560
     atagaaatta ctagagactg acgtggaggt ttcaacaact aatatttata accaaaattt    334620
     aattaccccc acattgttac cattttcttc agtgaaaaat tgcttgccat gattaagttt    334680
     taagtagatt tccaatgttc acaactgagc ttccaagaga gtcttgagaa caaaaacaat    334740
     gagggcagag aaatctatct tttctgtatt caccactaaa ctcaagtgga ctcagcactg    334800
     cctttgatca ctgctgcttc tctgcagagt tcaggtttct acttctcaca attctgacac    334860
     acattctacc tctcctcaaa tgtttggcct ctgcttcttg taaggtcacc ctctgttctt    334920
     aacttcttct ctgagtcatt ttgtgaggtg gtcatgagcc attaaatgga tattttatat    334980
     tttcccaaca tgaatcacat gagtggtcat gaattatact tctgattatg gcagttgatt    335040
     tttcttggca tgttcatgac tagtaatatt tgaagccatt tcattcaaat cttcggggct    335100
     tcgtttttgt tgctatgaca ttttttcttc tattgagtct ttccactagt attataacat    335160
     gacctagtat ccaggctcag ttgtcattaa taataaccac atatgtcaaa aatcatgcat    335220
     tcttttcaca gcagacataa tttcctcttt tctgcagatg aagacacact gctgagctac    335280
     ccccacttac aagaatatat gcacaattat gatatcttca tttatttgac taataagcta    335340
     tatcattctc ccttcaaatt ctttaccccc cagaagtcct ggacaaattt ctgcatctgc    335400
     tcaaaccata aactcagaac tacatggtga gtaaaagtca cctggttctg gatattgggt    335460
     ccatctcttc ccctccaatg tcccagagca cctcagcaca cctgtccagg ttctatcaag    335520
     aaagagtagc tcctgcacac tgaaggaaac aattgagtta agagaggacc tgcagatgat    335580
     agacaatatt gaaaactatt aatatgacaa aggattacta ccaagcatgt gaaataagct    335640
     caacgggtgt ggtggttcat gtctgtagta ccagcaattt gggaggcaag ttgcgcagat    335700
     cacctgaggt taggagctcg acaccagcct gaccaacata aagaacaccc tgtctctact    335760
     aaaagtacaa aattagctgg gcatggtggc atgcgcctgt aatcccagct actcgggagg    335820
     ctgaggcagg agcatcactt gaacctggga ggtggaggtt gcggtgagct gagatggcac    335880
     cattgcactc cagcctgggc aacaagaggg aaactccatc tcaaaaaaaa aaattacaaa    335940
     aaattagctg agcgtggtgg tgggcgcctg tatacccagc tgctagggag actgaggcag    336000
     gagaatcact tgaacccagg aggtgaaggt tgcagtgagc tgagattgcg ccattgcact    336060
     ccatcctggg caacaagagt gaaactccat ctcaaaaaaa aaaaaaaaga gccttgcaaa    336120
     gggcaaatag atcatagaca gacagataga tagatagacc tattagtata catacataca    336180
     tatatataca ctaatattca ggaaaatgca aattcataat gagatgtctt ttcacccttc    336240
     atctctgcta gaaagtttgt tatctgaaaa acaaatacat acatacatac ttattaaaag    336300
     ctggccagga tgcctagaaa gtaaaactca tagaccactg gtggaaatgt aaattagtgc    336360
     agccatcaag ggaaaaaaat agaactacca tatattccag caatccaact gctaagtata    336420
     tatctattta aatatttaaa agaaaaaact aatattgaag agatacctgt acacccatgt    336480
     ttattgcagc actaatcaca atttctaaga tatgaaatca acatatgtgt ccatcaacag    336540
     atgaatggat acataaaatg tgatatattt acacaatgga atattattca gccttaacaa    336600
     tgaaattctg ccatttaaag caacatggat ggaatgggac accactatgt tgagtgaaat    336660
     gagtcagaca cagaaaaata aataccgcat ttctcagcgt tacttctaga agtaaatagt    336720
     agagtagtgg tgatgagatg ccaggaatga gagaaggctg agagataaga agaggtttgt    336780
     taacaaacac acaattacag gtagacagga gggatgtgct ctagtgttct acagcacagt    336840
     agggtgacta cagttaacaa tatattgtac gttttctgtt tacaagaagc cagaagagag    336900
     aattttctat gctaccaaca caaataaatg ttagtgtctg aactgacgaa tttgctcatt    336960
     gttctgattt tggtcatacc aagtggcaca catgtattca aatatcacac tgtatcccat    337020
     aaacataagc agttattatg tgccaacttt gaaaaatcct ttaattaaaa agaattatat    337080
     tggcgtacat tacaaatgat tcaacacaga gacaggaata aataccattt ttctttgaaa    337140
     tagttaatta actaacaatg tagttacatt catttgcacc aaatcgtgta tttgataatg    337200
     gtatgcatag acagagttat gcataggata atatctttta attttagact actacttaat    337260
     actataaata taaataattt taaaacaact aagtaaaaag aataaagctg agaaaatgtg    337320
     tgtgtggtgt gtgatgtgtg agctttttct tgtgcaccac tgtgtccttg gtggatgtgt    337380
     ggttcatgtg tttgttttta tttactctgt ttggggttct ctttgcttct aggatctgta    337440
     gttcagtttc tttcacaaaa ttgggaacat tcttcgctat tatctttttc aaatagtttc    337500
     tgtgtattta taatttctcc ttctcagatt taaaatatac acatactata attttgatat    337560
     taatgtttag tttctttctt cactctcttt tcgtttgcaa tttactttgt gaaatttcta    337620
     gtgacatact aatcacatgg ttttattgaa aagctgagcc agctctactg aggtgtgtgc    337680
     caaaagattg ctcgatgttc atacagcatt gcttttgatt tcttacgcat ttccatttga    337740
     tttattctta gtattttcat atttcagttc cctatctatg tccacgattt ctttaagaga    337800
     ttcttgcata tgaatcatag ttactttaca tatcttgttt aattagatat ttataatatc    337860
     tgtttcatct acaaatctca tgctgatcat ttgtttatta cgactttggt acttctcatt    337920
     aatgtatgta ataattgttg atagccacag atactgggat ggacagtgga tactggctta    337980
     ttatttcatt ttatgcattt ctgcctgtat ttgaccacac tttacctttg ccaggccttt    338040
     actgtggaag tatctgtgaa tcttctcaga actatatttg acattcactt ttgcagtgga    338100
     catcaaagtt gaagtctgtt cttctgtgtc caccagagac ttcagatcct ccagtgatat    338160
     cttgtttttc tttcctgctt ggctttgtct cttcacctgt tccctcctcc agagaatcat    338220
     gttcagctcc ctcaggtgga tttaaatgtt atttaactga caattgtgaa attggtggaa    338280
     agcaatagaa taaagggaga ttttctgacc tttcctgggt tcatattgtg aacatgagtc    338340
     tgggtgtgac cttcccaatg tttctgaact tcctccagat gagatgttgg tctgtgtgtt    338400
     cttgctcttt tccctgctgt ggagtcctct tgttttcccc agttgttccc tcccacagct    338460
     ccaacgttct ctttcggtgt tatcacctta cagatttgct gactagaact gcagattaag    338520
     gctctgatta aataagaagg aggggagata cttctcaatg gaacttaggt gaagacctct    338580
     tttcccatct cagttcctaa gggattgccc cagtgcccct aagatactgg ttttggtggc    338640
     ttgcccctcc agaataattt ctttgttctc cagtggggat atggaaggtg ggtctgaacg    338700
     cttttcagaa gggtgggcac tttttctctc ctagacagac acaatgggac agaacaattt    338760
     tggtgactgt ccccattttg gggaaaaagg attcaatagg ataggaaaac tcttcagtct    338820
     gtggtcccta agaaattcac cctacaacac atttaccaca cttgacttca agcaatccaa    338880
     tatatatgtg tgttttcatc ttgtaatagc ctacatttta catgccatac tctgcctcag    338940
     ttcagctcat accccagctt tgttactctt tacaagaact tgcctctccc tagatttcac    339000
     atttgctgtt tatcttaaaa cttcaagtat ctaaagtatt atttttaaaa aatggccagt    339060
     tgtggtggct cacacctgta atcccaacgc tttgggaggc tgaggtatgt ggatcacctg    339120
     aggtcaggag tttgagacca ccctggccaa catggtaaaa cctgtctcta ctaaaaatac    339180
     aaaaaaaaaa tagcttggca tggtggcagg cacctgtaat cccagctact tgggaggctg    339240
     atactggaga atagcttgaa cccaggaggc agagtttgca agtcgtacca ttgcactcca    339300
     gcctgggcga cagagtgaga ctctgtctca aaaaaaaaaa ttccaaaatt ccagctcctc    339360
     tgtttatcta tttttgttga tactgttgtt gtaaaacata agtaaaatat attattcatc    339420
     tatgtacatt tccaagctgt gtagaagaat ttttaataag acccagagta aaaaaagaat    339480
     gcaaatatgt aggggccagc cctacagggt ctgtggatct ttctccccat gtgcagagat    339540
     gagagatcgt agaaataaag gcacaagaca aagagataga agaaaaaaca gccggaccca    339600
     ggggaccact accaccaaga cacagactag aagtggcccc gaatgcctgg ctccgctgtt    339660
     atttattgga tacaaggcaa aaggggaagg gtaaggagtg tgagtcatct gcaatgattg    339720
     ataaggtcat gtgggtcacg tgtccaccag acagagggca cttccctgtt tggcagccga    339780
     ggcggagaga gagagaggac agcttaggtc attatttatt ccattctctt ctcagaaaga    339840
     tcaaagactt taatactttc actaattctg ctactgctat ctagagggcg gagccaggtg    339900
     tacagagtgg aacatgaaag tgaaacagga gtgtgaccgc tgaagcacag catcacagag    339960
     agacgtttag gcctctggag ggctgcgggc aggtttgact gatgtcaggc cttccacaag    340020
     aggtggtgga gcagagtctt ctctaactcc cccggggaaa gggagactcc ctttccaggt    340080
     cttctaagta atgggtaatg ggtgccttcc caggcactgg cgctaccgct agaccgagga    340140
     gccctctagt ggccctgtcc gggcgtgaca gaggctcaca ctcctgtctt ctggtcactt    340200
     ctcactgtgt cccttcagct cctattgctg tatggcctgg tttttcctag gttataattg    340260
     tagagcaagg attattataa tgttggaata aagagtaatg ctacagactg atgattaatg    340320
     atattcatat ataatcatat ctataaccta tttctagtac aactattctt attttacata    340380
     ttctcttcat tacactggaa cagcttgtgc cctcagtctc ttgcctcagc acctgggtgg    340440
     cttgccgccc agacaaatat tgttaagctt cttaatagaa aaacaaatta tggtaaatgt    340500
     gttcactgga atactacccg tcatttataa taaattaatg cctgatacac agagcaacaa    340560
     ggtaaaatat ctaagtattt atgttgagta aaataagcta aacaaataag aatatatact    340620
     atgtaatttc atttttataa attctgataa ataaaaatgc atctgaagta aaataatgaa    340680
     gataagtagt tgcctggggt aatggtagaa gaagggaggg ggagaggagg aggaatacag    340740
     cagaacaagg ggaaatgttg agaagaattc acttgtccac tttcttggta atgatagcag    340800
     ttacatcatt tttattagtt gtacatttta aatatgtgaa gtttattatc tttcaattaa    340860
     gcctcataaa atgtcttaca agcaaacaaa tggaaactta gacaaggaaa gagtaataga    340920
     aagacagaaa aaataagttc aatgtcagaa gtacctgaaa attaatgtgc ctggatccta    340980
     gttctctcca tattttcaga agagtgctgg agggcagcaa aaccacacat gctcttatta    341040
     cggaatgtgg gttctgataa aaacactaga cacatccagc tttgtcctgg agttggttta    341100
     gggggatgtc agagacagtg atgaagagca cagggccaga taccggggtt cactcatccc    341160
     agacatgagc tccgagacgc atacagagcc cccccatgtg tgggtttact tccacttctg    341220
     taaatggaga aaatattgtc tcctacagaa catagtttac atgaatattt aaaatgaaat    341280
     agggtgatta gtgcaaagtg tttatcacag cacaatttca taataagaca gcatattttc    341340
     caaatgcaat cattgccagc aaacttctac agggcaccgt cgtcttatct gggtacagcc    341400
     tactcctcaa gggtcccacc ctagagcttg ctatatagta ggagatatgc aaatagggcc    341460
     ctccctctac tgatgaaaac caacccaacc ctgaccctgc agctctcaga gaggtgcctt    341520
     agccctggat tccaaggcat ttccacttgg tgatcagcac tgaacacaga ggactcacca    341580
     tggagttggg gctgtgctgg gttttccttg ttgctatttt agaaggtgat tcatggaaaa    341640
     ctagagagat ttagtgtgtg tggatatgag tgagagaaac agtggatatg tgtggcagtt    341700
     tctgaccttg gtgtctcttt gtttgcaggt gtccagtgtg aggtgcagct ggtggagtct    341760
     gggggaggct tggtacagcc tggagggtcc ctgagactct cctgtgcagc ctctggattc    341820
     accttcagta gttatgaaat gaactgggtc cgccaggctc cagggaaggg gctggagtgg    341880
     gtttcataca ttagtagtag tggtagtacc atatactacg cagactctgt gaagggccga    341940
     ttcaccatct ccagagacaa cgccaagaac tcactgtatc tgcaaatgaa cagcctgaga    342000
     gccgaggaca cggctgttta ttactgtgcg agagacacag tgaggggaag tcagtgtgag    342060
     cccagacaca aacctccctg caggggtccc ttgggaccac cagggggcga cagcacactg    342120
     agcacggggc tgtctccagg gcaggtgcag gtgctgctga gggctggctt cctgtgatgg    342180
     cctggggcgg cctcattgtc aaatttcccc ggggaacttc tccagattta caatcctgta    342240
     ctaatatttg atgtctctaa atgcaacctt ttttttcctt tttgtgtctg tttttttttt    342300
     ttaaaacagg aggacacatc ctcacctcca cagaagccac agtgtcactt tgggggcaga    342360
     aataatcctt tcgtggtcaa cagggtgaga gttttgagga atcccaggga aacctgggga    342420
     atgttttcca attagactca gggcagagac ctccatggga atctctgatt agaacaggct    342480
     ttgagttctg atgggagcca agagagaggc tcacccaggg tcagggttct taaaacctga    342540
     tggttttcac agcaatcccc cttcatcttg tgaaactggg cacatctgac tcagactgat    342600
     tcagttgacc ctctttctgc taatccattt tccttcccag tagacttgat tctcacagat    342660
     ccctttcttc ttctctttcc tgaaaacaga ggatgtgttt tctgtagtct aaattccaag    342720
     gctcaggtcc gcaggagctg ggtaggctga ggggtcttcc tcactcacta ttgcctggaa    342780
     aatcctgctg tcttctgtgc atggaggcat ttggaaaatg aagcaggcat tagtcatgaa    342840
     gggaataata ctagttttct ccaatgggat gttgatgtag agctgatctt atgcttctca    342900
     cactgtcaca aagtttggac tctcacctgt gactttgagg agagcttgtg ataccttatc    342960
     ttgttttaat atgaatagac tctcccttag ctcaggaagc tggaacagac tccatttggc    343020
     tccttcattt gtaagacatc aagggctcct cacccacccc cttcctcaag gacttaactt    343080
     gtttaagctg actcccagca tctcaaagag tgcgattaac tgataaggta ctgtggcaag    343140
     ctgtgtccgc agttcccagg aatttggcca ggtgatggta ccctaaagcc cctgcatttg    343200
     tgtctggcag ataacaccca gagcccccac acctatcatc ttgtgatgaa tttaaagccc    343260
     ctgcacctgg aactgtttgc ctgtaaccat ttgtcctttc aacttttttg cctgttttac    343320
     tcctgttaga atgctacagt taggctcccc ctcccctctc taaaccaaag tataaaagaa    343380
     aatctagcac cttcttcggg gctgagagaa tttccagcgt tagccatctc tcagttgcca    343440
     gctaataaag gactcctgaa ttcatctcaa agtgcggcgt ttctctctaa ctcgctcggt    343500
     tacaacaagc tgaggatgga cactccattg tgcagtgagc tctgggtgac agtaatcgta    343560
     gggtctcccc gggcagccta aggtcaatac tgctggccgt cgggaaagac aggctggaat    343620
     tcctgggaag acctgcatct gccatctacc acggagtcct atggtcttct gttacactct    343680
     ctttgaatca gcaccaccta gattatctaa aacactcttt gtgacttcat gactgggaaa    343740
     aaataatggc agtctctact aacacctgtg ttaagccatg ggagcaacac ctaggctagt    343800
     gtgtgattga gtagttgaga ctgtggtcta gtcaaggtga cacataaaat tgattgttgc    343860
     cattatgata ttttatttta tatttgacaa tataatcatg ctcatattat aaatatttct    343920
     gttacattat ttgtgtcaga ggctttggaa ccagaacaac ttcatcttga ataaggggta    343980
     ggaaaaataa gactgagacc tgctgggcta cattcccagt aagctaaggc gttcttagtc    344040
     acaggatgag ataggaggtc tgcacaagat ccaggtcata aagaccttgc taataaagtt    344100
     tacagtaaag aaactgggct gaagcccacc aaaaccaaga tggtgacaaa agtgacctct    344160
     gtttgtcctc attgctcatt atatgctaag tattatgctt taacattcta aaagacactc    344220
     cccacagagc catgacagtt tacaaatgcc atagcaacat caggaagttt ccctagaaac    344280
     taaagagtgg aggaaacctc agctttggga attgcccggg gaattcatga ataatccttc    344340
     ttttgtttaa cgtataatca agaaataacc ataaaaatgg acaaccagca gcccatacca    344400
     cttctctgcc tatatagtag ccattcttta ttctcttact ttcctaataa acttgctttc    344460
     cttttattct gtagatttgc cccaattggg atccctttcc agtaacattt gtgttattat    344520
     tggttttcta agcagaaaac tctgagagga ggtgtcagcc gggcttcctg ggttgagtag    344580
     aggctcagaa agcagtgaaa ctcactcatt tcctgcatca ggacttactt tggtcctgga    344640
     tgaataatat tgaagatata tgcttaaaat attcctaaca tcacaatttg tgcatgtgtt    344700
     ttcttcccta agaaagctat aaacagtgaa aattttgctg taagcttccc tgtgtcctct    344760
     ctccctctct ccattccccc tcccctgaaa ctaaaaggaa tgttttaaca ttatgggctt    344820
     ttaagtctgt atttctgtga ccagcagacc ttatctatgc tcccaattca aattccttgt    344880
     aaacacaatt tgtaaaatcc tgcgagatcc tgtctccttg gccacgccac tgcaaggtca    344940
     taaagtagat caaacttaag ttacaattct gattttcctc aagatctgag acatgttaat    345000
     tgtctttgtt tcttgctcag gtaacatctc tttttgctct tttttatctc tttttgcact    345060
     taaatctaag tttacattag tcatgtatct agaatgtaga aatatgtctt aaagttactt    345120
     aactttttaa aaaatttatg gggtttccag aagatgtgag aacctccttt atttataaaa    345180
     tagctgattg cacgttgctc aataatttcc tctttaattt atctctctcc tgtcacatta    345240
     tactatgata agcaaaaaga aatgaaggca taccatcaac agtttactta gatatctcct    345300
     cagctaaatt tctaaggtat aatttactaa ttctaatttt atccaattta acataattaa    345360
     gataaattat ataacaaggt acacatatgt tccagttcta acaccatgtt tgtgacagaa    345420
     ataaaacaga gcttctctct gtaatgtggt catctgagcc ctgaggtgat gggtcacaca    345480
     ggtattttta tgatatttaa gggtttggtc aagaacagtg ataacaagtc tgaacaagac    345540
     tggtcatact tgaaaggtta caggtaaagt tactgctgtt attttatatt aacgccttat    345600
     tagaataaag ctcagaggaa gaggccacat cctaggtcac agtaggagga ggaatggagc    345660
     tgtgctctgc tctccacact acttttcaca tcccaggcac aagcccaggt tccatgacgc    345720
     acggcatcta gagggcagtt ctcaggggat gatctcaggg cacctgcttc ctcgggcagg    345780
     gcgcttcctt ctctcaattg cagacttgct ccttctgcaa ggtctgaagt cagcacttgt    345840
     attcccatga tttttacagg ttctcttccc taatatggca aaaatctttt tattaaattt    345900
     tcaattttat tttctctgaa gcacactgca ttagcagaaa cgaatatatc acattcctta    345960
     tccgcccaca cagccaaaga ttcctgaaga cagagctgat gtgacgtact cataggtgga    346020
     tctctgcccc tcagaggtgg ccttggtctt caagtttcag caatttctag gaagccaaag    346080
     acacctccat ctactcctcc ctgctcgaca gctcacctga gaacagcttt ctcattggaa    346140
     tgtcttgtgt ttaagaaata aaagttgctg tttgaggtta gggagcccag gtgcacctac    346200
     cagatccagc ccaggattgg agatactttt cagaagacaa catcacctga gacacgacca    346260
     gtcccactgt ttcactttca caatttcagc ttcttcagaa gaaaattaaa attgttgaga    346320
     cttgttcata agcgttgtgc catgtccttt ctctgttttc ttgcctgttc attgatgtca    346380
     tgccaggtgc cacttctatg taataggatc agaattctgc ctctagtaac acatcagagg    346440
     tgaggtttga ttgtactttt ggtttatgct ccaaaacgta gattataaaa ttcatcaccc    346500
     tcatttttat gtcaaaagta atctgcataa tctggatgtc aatacttttt ggaatctatg    346560
     aaataacaga aattgcaaga aatattaccc actccaaacc tggagagaca ggcatgtgca    346620
     gaatggcaat tgacacctgc ttaactggag cagaagctgc agaagccaca agctgttgag    346680
     ggcacttaca tggtaagcac tacagacgtc tgaagacaga tgtggactca gtaaatgtaa    346740
     cagttccaga gggtcttata cttctaagtt ttctggaatt tctttccaga aacctcaaga    346800
     ttctaaaatg tacattccaa agacatttcc tatagatcaa aattgcagat attaattact    346860
     gagaaacata ccacgagcct tcttcaaaag cttctacagg aaaggacttt tccagaacct    346920
     tatcctatgt gaaggaagac aaatctccca ctgcagattt ctctcccatt cttccataat    346980
     tgtagaaatg agtaaagtta gccaatagag gtaagatata agtaaataat ccagggatgc    347040
     tgaaaacaaa aaagggagta atcgcccaaa atgagctttt cccctggaga cgcctggtca    347100
     agatcatagg ccagaagagg aagctgactg tgtctctagg tttccactgt cagaacaggc    347160
     agtgcttacc tgcactgcac aatccattct aaccaggatg atggctctgg attaaaggtg    347220
     aaagtgtggc aatgcacaga ctctatctga ggagaacaca ggaaaactaa aggacaacgg    347280
     cagagggtga gacaagaaca gtagagcaat ctgaagcctc tgacatcatg atttttaaga    347340
     ccaatgtctt aaacactcct tcattgacct cagcttcttt catcatgaag cctttgcagt    347400
     gactctatct gagaaaaaga aaataacctc ctgcatgcac ttctcaagtc tctgcttgta    347460
     tcctgtttgc tttaggtctc taggggaaaa agtcagatac ctggacctag tgtcagtgta    347520
     ggaggcacat cctaaaagct acaaactagg aagaagggaa gatgggtgtt attggaatag    347580
     tggatatgga gtgggctttc ttctgaaagt atatgcacct gctggtattc tcagatgcaa    347640
     cattcaactg caagagccca ttgaagaaac aaggcactcc caaatctcct gtaagattct    347700
     gtattcattt cagataagcc cacatgtccc cgcattaact ttttcttcag acaagcacat    347760
     acatctgaaa ctgaacagct atgtggcaaa ataagctcag gatagaagta attgtggact    347820
     ccagctctga caatttgtag atctgcattt ttaaaattct aactgaagac tttgctttat    347880
     tgtagaggac agtggtttac agctcgaatt gcacagccta caggcagatg tccatttcct    347940
     ctgggcaagg tttattttta tttgtttact gtacttattt gttgacaaac attgatacta    348000
     taaagatacc ctaacagcgt ccacatgaaa gaaaaagaaa gagcaatgga cacatcaacc    348060
     ctgaacatcc agtcccagga atcctttgac cctgccctcc ttgcaaccca gagatataga    348120
     ttggatgagc cctgctgagc agaacacaca tgtccccagg agaaagacat ggaaatgagg    348180
     cccctccctg ctaatgaaaa gcagctcttc ccctcttctc ctgcaggtcc tggtgaggag    348240
     ccacccaata tctgtgccct tcctcagtgt ccacaccatc gggtctatga tgatctgggc    348300
     ttcacttgtc atcactctca atattgaggt tccccgttaa acagactgag tgaactgtgg    348360
     ctgctccacg tgggggctgt tctcagtctg ttgcttctgt gcttgcagag gtcccctgtg    348420
     aagttaatta ctggagtctc tcagagaaat actacagacc aagaattctc agacttttct    348480
     ggaaaccctg tggattcact ttcactgaaa acagcataag cttggtccag caggcttcat    348540
     gacaggggtg ggtgtaggtg ataacatcag taattcaagt ggaagttctc agtgggactc    348600
     tccttgagta caaagaagat taacagtcct cagagacacg cttttcagat gattctctct    348660
     taagatgatt aacctgagag ctcaggaaaa ttccgtttat tactgtgagg gacacggtga    348720
     ggggacatct gtgtgagctc agacacaaac ctgcctgcag ggagacacaa acctccctgc    348780
     atggtagatg cttctcagaa ccaccagggg gtgcacagga aaccagaagg tgctcaggac    348840
     accagggggt gctcagaacc accatgactc actcaagacg ccaggggcgc tcagaaccac    348900
     cgtggggcac tcaggacacc agggggactc aggaaactag ggggtactca gaaccaccag    348960
     gcggtgctca ggacaccagg gagctctcag aaccatctgg gggcactgag gaaaccaggg    349020
     gactcaaaca ctagggtgtg ctctgaagca ccagcggacc ctcaggactg cagggggcgc    349080
     tcaggacact agggaacact cagaaccacc aggaagcgct cagaaccacc agggggccct    349140
     caggacatca gagcgtgctg aggacctccg ggggcgctca ggaccttcag agagtgatca    349200
     gaacaccaga gggcgctcag aacaccagga ggtgctcagg acagcaaggg gctctcagga    349260
     cactagggtg tgctcagtaa accaggggtc cctcacaacc accagggggc actcaggaca    349320
     ccaggggatg ctcaggacac caggggccac tgaggacacc accgctccct tagcaggcag    349380
     ctccacatca ggcccctggg ttggggcagg aagggtgttt tccttttgga tcttgccact    349440
     aaactcttgg gagtttttct ccttcctttg tggtttcaag aaacattggt agattcttct    349500
     caggtataaa gctctgcttt cttggattat gtaatgtttt tggtttcgga tgttaccaga    349560
     attacactgc actgtgagag gattcattcc tcgtgtgtgc aatagtgaat gaaagctcca    349620
     atgttagggg tggctttgaa agctacgtta ggggtggctg agggcagtta gcaggaaatg    349680
     atcatcacta tagaaggcta ctcatttctt tgcacatttc cataaataat tgtagtttat    349740
     gccctaaaaa ctgcatgttt tcttggccct ttttcttaaa tgcctccaat ccaagaccag    349800
     tcatctaatt aagctgtatg tcaaagacca ccaatcaagt taagtctgtt taatgaaaca    349860
     ctttgtaaac aaaaaagtac atctgtgttt gtatagtcag ctttaaattt tacattgctt    349920
     tacaaatatt aattttgtaa atttagtctc ataattatcg tcagtattta aaatcttaaa    349980
     gtcttgctat gttaaattaa gtaatcttag ctttctcact atgaattaga gttactaaga    350040
     attagaatag taagagcatg taataagctt ttggtgaagt ttataaagaa agatgaagat    350100
     acattttttg ctttaaaata ttttgttttc cagtttacag gacctttcta ctggttttaa    350160
     gataacaatc actgtttaca tctaaccctt tttttaaaca cctgctgttt aagattataa    350220
     aattataaaa ttaaaaacct agttaaaacc agattgatct ttgtaattta acaagatgtt    350280
     cagtattgtt gttttaataa aaaaaaatag gtaaatactt agctactaga aaaataatca    350340
     tctacttaat cataaggttt tacttaggta aacacctaaa tttcatgggt tataaacatg    350400
     gttaataggt gaaaaacttt aatggacaag tattacagtt ttcataaata ttctaggtaa    350460
     gctatttaaa aaataaatta ggtaaatgaa ataaaataaa ccatttaaat aaacttgttc    350520
     tacaatttaa aaatctaaag tttaattaaa taatatatat tatataaatg tttatgcatt    350580
     actaattgtt taaaatatat atactataag gaaaactttt taaaaataca tattataaga    350640
     aaatattttt tgaaaacatt tgtttttaga aaaataattt atttaattca aaggttaatt    350700
     ataaaatgtc ataaacatac ccagttaata agagaggttt aaagaaagtt ctagacatag    350760
     agaagtactt ttggtaagaa aggttaaaat aaaaaaaaat tatatgagag ggaattttgt    350820
     aggataactt tttatatata aaagtgacta tttatgaaag aataatgttt agaataaaac    350880
     aagatgttca agtatgccat aaatggttcg tgtaagtcaa aataaggttt atagaaagct    350940
     aacttattaa aaaaacttca tgttatcgag ttgactataa ttgaaaggga agaatttatt    351000
     atagtcttta tagggatctg gctttcatat gaaaatatac taacacactg aagattggtt    351060
     agaatgacaa aattgtctta aagtattgat ttattcaata aaattataag gtattataat    351120
     tttttaaccc aaaattttaa cttttgttgc atcttgccat ttttattttt ttccatttga    351180
     gaaggcttga gaggatctca actttttcat cagctccttt aacattgttt cttacttaca    351240
     gcagttagcc tctgagttaa cttctaactg ttgttagttt ctgactgcta ttatttcctg    351300
     atgttaaaat cctctatctt aaagttctaa ataaaatgtt ttctttcaat ataatattca    351360
     gtgtcgttgg cttttcttta actgtctaaa tttttctatg aaaccaaaat cttcacttgt    351420
     aaagacacat tcttcctagg tctgattaat tcaaatactt ttttcattag agttgacttg    351480
     caggttatgt acatggagtt ccccatagga aaaaacagtc acagtgcaga aggctttatt    351540
     tttgctattt ggtaactggc atgagacaaa ttttaaattt cattgaaata attcctatgt    351600
     aagtgttatt aagtttttca actacttagt aatactgaga gtttaagaca atagaaatta    351660
     atgttatgac attcatgtaa ctatctgtat aacttttaaa gtccttgtgc tgctacttta    351720
     ctgagctttg aatcctaggt ctaaaaagga cacacaacac tttgggaggc taaggtgggc    351780
     aaatcacctg aggtcagaag ttagagacca gcctggccac catgacaaaa ccccatcttt    351840
     actaaaaaat acaaaaatta gctgggcatg gtggcaggtg cctgtaatcc cagctacttg    351900
     ggagcctgag gcagagagaa ttgcttgaac tcggggaggt ggacgttgca gtgagcagat    351960
     attgcactac tgcactccag cctgggtgac agagtgagac tccatctcag aaaaaataaa    352020
     taaataaaat aaaaaataaa aaggacacca agtccagcta aatcttaaac accgacagca    352080
     attaaagccc catctacaga cctggaagaa aatgacaaga aaaattgatc acactctcaa    352140
     gacacaagcc cagaaattga aactacttaa ccaccccagc cccagggact attacagaag    352200
     aagtggcttt gtaagattgt aaaagctaat tttgagagat gaaatcggtt cagagtttct    352260
     ttataaatta aacattaatg caaggctagc atctgggccc ctgtgccaga ttgaccaggg    352320
     tttcttgaag aattaatcca catttaaatt taaaaaacag ataaaactgt ataaaagatc    352380
     tatgcaaatt atttttatgg taaaagtaat tataatttaa tagatttatt tttcagaatt    352440
     gatagtttta acttttctca tgctgttctt ctaaggggtt atattttaga aaatcaattc    352500
     tactctttca aaaatatttt ttttcttttt tttagaaatc actgagtttt catgtggcta    352560
     aataaataac ttattttaca ataatctgta atcctatttt gtaatatcaa gtgttgtaaa    352620
     cttttgatat ttgacaaagt tcacaaaata aaattctaaa ttcagtcatt tgaacaccct    352680
     gaaaagaaac acatttagct tatttggtac agttaaatta tacaggaagt aatgtcaaat    352740
     ttgcaatggt tattaacttt ggactatatt tatataaatg tggactatat aatttgaata    352800
     tacttatgta aagagtatat gttccaaaat tctattagat tcaagtgatt tttatatgtc    352860
     ttagtatcag tagtacttat gattataatt taaaattttt gtttatcaca gaaataacca    352920
     aattttctcc tcaattctgt ctttaaccag gggtattcta aaaggtcggt cattcacaat    352980
     tgttgtttta ctttgattct ttatcaggtg gcttataata atctatagaa ctttgagtag    353040
     tactcttaaa tatacaatat acaattttga caattttata aattgtgcct ttggtataga    353100
     gagaaaaact tccatgagtc tcatgagacc tgaagcattt atgatgattg ttaatctaat    353160
     atcaagcagg acaggatgta attgcatgaa ctgaatgaaa aggagactga aataattttt    353220
     ataacttatt ctttaaagca tttgctattt acttatgttt tattgttcag aatcaagaaa    353280
     actttgtctt ttaagctatt cacagttttt aacaatttta actatactct attgagaaaa    353340
     attgaaaata taatttcttc ttctctacat aatttctcca aaatttggaa actgtaggta    353400
     ttcttatatc aaaatagtta tttgcatagg ttcgataaaa atctgctttc ttccataaca    353460
     gagcacaatt agagacaatg gtcactttac caaggcttta acttgaatga catattttct    353520
     gatttacttt ataaaatgaa gagctgtaca gctgatataa gcccctttgg aaaactggca    353580
     ttttaccttt tttttttttt tttaacaggg ccctgagctg tagtaagtaa ataattttgc    353640
     tttctgacag gcccaggaaa cccaagtttt cttggatact tgaaaaaata aaaagtaaac    353700
     caatccatat agctatctga tggcacagat aaaatattgg ctgggcttga ggcttttaaa    353760
     gatcttaccc ttagattcct tataaaaaat agcaaaagca atgtatgaat aaaacagcct    353820
     atgtacaaca aacaacaaca aaaacaaaaa caataaaaag agagctaata tgttaagtga    353880
     ttatttttgc tgcatcttat acaaaaaaat caggccaagt ataataagcc taaaatttat    353940
     tttacaaata aattagtcct attatgattt tgtctccaat aaaattgggg aattataggg    354000
     agaaatattc tttcaaaata aactatagtg catctgttat taggttctaa ccttgtccac    354060
     ttgtttttca atttacaata ttttctacaa tttggactca attttaaaat atttctttcc    354120
     acaagtctcc aaaataacat tttcagtgat tttcttttta aatatttttt tcctatttga    354180
     aatctccaga aggtaaactg tgctttctta cagctagtca acttaaactc taaaataaaa    354240
     taaaatcaat gatatggttt gtctctgtgt tcccaccaaa atctcagttt gtatagtaat    354300
     aatttccaca tgtcaagggt ggaaggaggt gaagataatt gaatcacggg acgtgttttc    354360
     tccatgcttt tatcctatta gtgagtgagt tctcacaaga cctgatggtt atataaaggg    354420
     cctccctctt cacatgacac ttctctctcc tgcagccatg tgaagaggta cgtgtttgct    354480
     gccctttcca tcatgactgt aagtttcttg tggcctcccc agccatgtag atctgtgaga    354540
     caattaaacc ttttttttaa ataaattatc cagcatcagg tatgtcctta tagcagcatg    354600
     aaaattgtct aataccagca acttaattat atacaaaaat tccttttata ccctcctact    354660
     gtgaagggaa aataaatctt gagaccccaa aattactaag ctaaagagaa gagtcaatgt    354720
     ggtgttacag gagatagaaa gaaattattt aggtagatag ttgggatgag agagtctctg    354780
     gcaaaataac ttttcttcta acaaaaatca gctcagaaat aacttcttct ctaattacac    354840
     acagttcaaa gaaatcactt ctaacaaaaa gcagactaaa taatcaggct gtgaaatata    354900
     gataagcaac tctgccacag agagggtgtt tctgggtgta atcaccaaac ctcacatata    354960
     taggatgggt cccagtaaaa acagtgagcc ttaataagca cattcctttt cttttctggg    355020
     agtacactaa gatagaaaag ctggaagctt gcacggggtt tgcgacgccg gcacctgtga    355080
     ggaagtacct gggaccaggc aagaaaaccc ttctggcctt tcttagcaca tgcacggtgg    355140
     aagaagataa gcagtgtgga ggagatcaag caaagtgccc gcctgcccaa tgaaagcatg    355200
     aggtggggtt gccagagact ttgctctatg cagatggcac acattgtcct aactgttttt    355260
     gcaccctatg ctgataagac accgtctccc cacgagcaca tttataaaaa tccttacatt    355320
     ttactgcagc acgataaccc atttgggacc cctctctgtg acagacagct ttttttttat    355380
     tttacctatt aaacttgtgc tctaacctca cccttagcat gtctgcgacc ttgatattca    355440
     cggccgtgag acaaagaagt tcgggtggta ttccagacaa cgaggctgcc atactggaaa    355500
     gtgctttggg caaatctgcc tccccttctg tttaaagtga ttcctctgag gctaacctga    355560
     gaccaataca cagctgattg cttcctcttc actatcattt atgtaaaaac gaagatccac    355620
     tgagtcagac taaattgtgc attcagtggt aggctaataa agtactcaaa agaacgcaac    355680
     ctattgtctc ttatctactt ctaaactgca gtccgtgctt ttgattcgtc ctgccttaca    355740
     ggaaaaatcc aaagtacatt ttacatatat tgattgatgt ctcatgtctc tctaaaatgt    355800
     ataaaagcaa gctgtacttc aatcgccttg ggcacatgtc tcaggacttc ctgaggatgg    355860
     tcatgggtga gttatctttg gcaagataaa cgtattagtc tgttctcctg ctgctaataa    355920
     aacataacaa agaccgggta attgataaag gaaagaggtt taattgactc acagtttcac    355980
     atggctgggg acacttcaca atcttgtcag aaaagcaagc gacatcttac acggtggcag    356040
     actagagaga gcttgctcag ggcaactcct ctttacagaa ccatcagatc taatgagaag    356100
     tatttactat catgagaaca gcatgggaaa gacctgcccc tgtgattcaa ttatctctca    356160
     ttgggtcctt cccatgacac atgggaactg caggactaga attcaaggtt agatttggat    356220
     ggcgacacag acaaaccaca ttagtaaact tcctaaattg actgagacct gtctcagata    356280
     tttgtcgttt accttattca tgttcagcct tcagagttcc aaggcctata tcagttttcc    356340
     aggattgttt cccctttttg ttgtttgtta tttcctcctt tatttatatg ttttacttct    356400
     cttttttctc ccactatttt cttattaatg gatgtgaaac ttcacaacat ttgaaatata    356460
     ggtaacaatg aactataata acaacttggg acctatttat ctagaaataa tccgtcctac    356520
     ccatgaaaga aaaaaacaaa aacaaaaaca gaagcccaga aacttatttt gtggtaaaat    356580
     gcttcctctg aaatattttg gaaaggaaaa agttgggaag atatgaaatg aaaataaaaa    356640
     cttatgatgt caattcatta cgtcatgagg ggggaaaaaa ctaaaggatg atccatgcaa    356700
     gaaactgatt ttcctttcat tcctaagaaa atagccacag acagataaaa tgttagatat    356760
     cttcacagat agctacaatt tgttcatctt tgaaatactt agtgcaggag aaccacttga    356820
     tattctattt ccctatgtgc ttctttttca ttactacatg taagttttca tgcaattcct    356880
     ctttcccctc tagccagctt ttccccttta tatattgaaa gccctaaaaa atgtcttttg    356940
     ggaatggcac taaccacgca ttgtttctgt gattactttt cttccaagca tgccataact    357000
     ttggaaaaat taattttaat ttgattgaga gctctctcag aaacctttgg ttacactagg    357060
     caaaatccaa aatcaggagc caattattgt aaaaatcagc catagtacac tgtgcgtctg    357120
     tgtgtgtgtg tgtgtgtgtg tgtacatgtg tgttttaatt tttgcgagct ttgagccatg    357180
     tagttctctc tgtggagatg ctatttggcg tgactttgaa tagagaattc taaaagaaat    357240
     aagaagctcc tatgagttct ctgaaagttt ctggactcac catggatctt gactgtgtca    357300
     ctgcatctga cagtcccagg gaacagactc tctggtggtt tcatagaatc tatgcttggg    357360
     ctctccctgc agtttactgg gtatagtaat gacaaatcac tgtttcaaga gacaatttca    357420
     aaagcattag atgctgctga gagaggattg tgaaccaggg aactgcccct ttcttcttgg    357480
     agagcgacat tgggagaata tgctctgtga gcccaaacag catcctcccc tgcagggtga    357540
     gggcagagct gcaggacagg cccagaaccc actccacaca gatgtcagcc ctggagctgc    357600
     tgcagaggag tctgaggaga aaatttttcc agcacctgaa ttacacttat ttcaaaacaa    357660
     aaatgcaatt aaaaagttaa aacaagtaat taatgtccag gcacagtggc ttacacctat    357720
     aatcccagaa atttgagagg ctgaggtggg aagatttatt gaacccagga gtttgagatc    357780
     agtctgtgaa aaatagtgag gcttcatctt tatttttgaa aataaaaata aaataaaaca    357840
     taaaaaattt agcaaaaact acaatgcgtc tttacatacc ccatcatact tgaagcattt    357900
     acctaaccca atgaggtgat gaggacccaa tttgaaagga gaaaatttta gacttttcat    357960
     atatcttaat agttggaaga tgtagaagaa atatatttat atcaattaaa tgtgtgcaaa    358020
     tattgacaga cacaccatac caggagttca acttgcaaag ggtaaaacca aaaaagtttg    358080
     agattgttaa tgtgccattt gaaggtgaga tcgttttgag gaccatgtcc tgtgagagtt    358140
     tgtttctcta ttagaggagt tctgtactca taaagttctg gacatgccag gagacaagta    358200
     tcagtaaaca aatatcagaa cttgaacttc agcttcccac tcttgcattc tccatgtgtc    358260
     atctctctac tatttctcat tctagatcag gtctttagct atgaaatatt ccacctaatt    358320
     aacatgtaaa tagattgaag tccacagagt taaatatgta tattttctcc tgtttttccc    358380
     agttgttccc tcccacagct ccaatattct ccactgttac catcaccttc tagatctgct    358440
     gccatgccct gcagattaag gatttgattc cacgacagag aggaggtgca tttcaatgga    358500
     actttggtga gaacctcggt ttttatccca tttcctctgg ggctccacca gtgcatctgg    358560
     aataatgggt tcagtggctg gcccctgcag ggtgattcct tagttctgta gtgaagatga    358620
     gggaggtggg tctgaatgca tttcagaagt atgggctctc ctctctcaga cagacacttt    358680
     gggaaaataa gatttttctg actgcaccca ttctagaaca aaggaattca attggataag    358740
     aatgctgata aaaaaaaact caaaccaaat taaatttaaa ggagtttaat tgagcaatgg    358800
     atgattcatg aattgggcag cccccggaat cacagcggat tgaaagagaa ttcagtgcag    358860
     ccatgtggtt gaagaagatt tatacatttt tttaaatgat gtacagaaat cagaagtgag    358920
     gtacagaaac agctggattg gttacaggct gatgtttgtc ttatttaaac acagtttgca    358980
     cactcaagag tgtatgagtg gttgaggtat ggctgctgaa attggccaac gctcagctgt    359040
     tggtgaggtg ctcaggtaca tactcctgag ttaggttttc aatctcgtcc acctattcag    359100
     gtaggttaca gtttgtccac aaggactcaa acatagaagt acggagtcct cctcaggcca    359160
     tatttaattc actttatcag tgcccttcag catatggttc ctgagaattt cacacggcaa    359220
     catgtttacc accctagaat ttaagcaatc caatacatgt aataggctgt atttcacatg    359280
     gcaacttctg cctcagttta gctaacacta tggctttgtt tctctctaca agaactcatt    359340
     tctcccaaaa tttccatttt cctgaaagga aaataaatct ttgggaccct catatcacta    359400
     agccaaaggg aaaagccaag ctgaaaactg tttggggcaa acccaccttc cattctttcc    359460
     ctaaaatgat agctgttaag gtgtttacaa gctacatatc tccttcaaaa tttgaccgca    359520
     cagaaaatcc ttgttgacca aggacattgt agaccaagca gagagtcatt tctctgctca    359580
     cgtaagtcaa atgcatatct gattgctccc tttgctctat tgtttcacta agccagacta    359640
     aggcctatgt gactattcct gtaaactgtg cattcagtta aaggctaatc agaaactcaa    359700
     ataatgcaac catttctctc atacctacct atgatctggt agctctcccc ccaacttcaa    359760
     tttgtcctgc ctttttgaac taaataaata taccttacat atattaatta atgtctcatg    359820
     tctccctaaa ttgtgtaaaa ccaagctgtg tcccaccaca ctgggcacgt atcatcagga    359880
     ctccctgagg ctgtgtcaca ggcatgtcct tagtcctgga aaaatgaact tcctaaatct    359940
     attgagatta gtctcagata ctctttggtt tataagtatg ttttctatct cataacttca    360000
     attatctgaa atactaaagg aaatctgcca aataacacat tatcttgttt atctgttatt    360060
     gttgttgcaa aaataaatat atttatataa tttatgtata attgatcatc tttgtacatt    360120
     accaaactga gtagcagatt tgttagtaag acccaatgta ataaaaaatt caaatatcaa    360180
     ttagcaactt aatagaaaaa caaattacgc tacatttgtt tgccgaaatg ctaccaattt    360240
     ttataaaaaa ataaacacat ataacataaa tggtttaatt ctcagtattt ctattgagaa    360300
     aaataagcca aatacataag cgaatatact atattatttc attcttataa attctaccaa    360360
     ataaatacta gtctaaagaa atatgaaaac atcagtagtt tataaagaaa tggtagaaaa    360420
     agggaagggg aaaaaacaaa taatataaaa gatcaagagg aatgctgagg ggagttgact    360480
     tgttacctca ttgaaaatag agattatttt ttcaaagttt actattgcac atgttaaata    360540
     tgtgaatttt atcatctgtc agttaaaact cataaaattt attacaagta aacagccgac    360600
     attttataca aaaaagggat gataggaaga aacaaataaa tacattaaat gtcagataca    360660
     ccaaaaactt atctgcctga cccctagttg tctccgtaat ttttggatga aaaccagccc    360720
     acccctgacc ctgctgctct gggagaggag ccccagcctt gggattccca agtgtttgca    360780
     ttcagtgatc aggactgaac acacaggact caccagggag tttgtgctaa gctgggtttt    360840
     ccttgttgct atattaaaat gtgattcatg gagaactaga gagattgagt gtgagttaca    360900
     tgagtgagag aaacagtgga tatgtttggc aatttctgac cttggtgtct ctgtgtttgc    360960
     aggtgtccag tgtgaggatc agctggtgga gtctggggga ggcttggtac agcctggggg    361020
     gtccctgcga ccctcctgtg cagcctctgg attcgccttc agtagctatg ctctgcactg    361080
     ggttcgccgg gctccaggga agggtctgga gtgggtatca gctattggta ctggtggtga    361140
     tacatactat gcagactccg tgatgggccg attcaccatc tccagagaca acgccaagaa    361200
     gtccttgtat cttcatatga acagcctgat agctgaggac atggctgtgt attattgtgc    361260
     aagagacaca gtgaggggaa gtcaatgtga gcccagatac aaacttccct gcaggaacgc    361320
     tggaggaaac cagctgcagg gggcgctcag gagccactga tcagagtcag cccaagaggc    361380
     aggtgcacac agaggctgat ttcctgtcag ggtgtgggac ttcgtctttt taccatttct    361440
     ctagggaacc tctctaagtt cagaattctg tgcttaccag tgtcatctct acatgttttt    361500
     taatgattat tttaatatga gaacctattc ttacatgcac aaaatgcaga tggatgctta    361560
     cagagatgaa aagtcctcaa ccatggtcac caggatcagc cttgaggaaa ctcaggggtg    361620
     cctggtgaat cttctccagt cagactcagg acagaaacct ctgtgagatt ccctgactag    361680
     atcagtcttc aggaattttg ataccagcca atagagaggc tgggacaggg tcagtgtcat    361740
     gtagaacctc acaggtttca cgtctgaccc ttctccttct cctgacacta aagtatgcaa    361800
     atcagtatca gcactgatct gggtcccctt ttgctcttaa accattctat ttctttttat    361860
     ttgttgttgt tcttgctttt cctcgtactt ctcttgctcc ctgtaaaatg gggaggtgtt    361920
     tcttgctgca aaagccccaa gcctcaagcc cattccctgc agctcaggcg gggctcaggc    361980
     tgtggctcct gcagccacat gggagtggct gttggggctt tctcttctcc cattgctcag    362040
     caccctccag tgtgttgtgt ggagactacc tgggaacgaa tgtggacaac aggagtgaag    362100
     gggatgagct tgcgtggaca aaatgggatg tggatgtgaa atttatcctg tgctgtacaa    362160
     acaaccacag attcaacttc ctcaccagta gtattagaaa gagggtgtga aagttgtcag    362220
     aataaaaatg gaaacacttg tgttaacacc ctgacaaatg gaactaggaa tgaccatgaa    362280
     gaagtttctc atgcatatac tccagaaaac aagaactacc gtaaatggat tctgcttaac    362340
     cacagccttg gatagaagaa accacaacct tacaaaaatc acttctacaa ggatatcttc    362400
     ccagcaactc cctgtttaac cctacagtga tgccaccctt agtcctacaa aaatcacttc    362460
     tacaaggata tcttcccagc aactccctgt ttaaccctac agtgatgcca cccttagtcc    362520
     tgattctagg agcacaggat aatcctctca aaatgactta tgtaacccac ctcattttca    362580
     ctgcgtaaac ctatggattc acatcctgga gtcactgctg catttgttct taatggtcat    362640
     tagccccttt cacaatattt gaggctgttt ttctctgatg tctcttctaa aataaagaat    362700
     ttccaagtgg acagcagtaa agaagctatt taggaagatg taatgggaag gcatctttaa    362760
     cattctgttg aatgtttcca catggcactt caatccccta aaatactttg ctgtatcagc    362820
     atgtgatgaa tcggagtata atattgaagg taaaatggga aatacatggg cttttgatga    362880
     atcaagtcat aggatgagaa tgtctgtgtc cttgaggaag gagaccatgg gttgtaagtt    362940
     ctagtggagg tacctttggc aaagggagtt catgagtttc tgaaccttat gctactttta    363000
     atttaaggaa gcaatttatg atgtatgttt acttaaatct ttccagagac atttgtcagt    363060
     aaggacaggg tagcatttgt gtaatagtga tgacttagag agttattttg taattgctcc    363120
     tgtaaggtat gcacattgct cacttgatac agaagttcaa atgtcacagg tggaaaaaca    363180
     ggaataaagg tgggcagatc acaaggtaag gatcacaagg ttaaccatcc tggctaacac    363240
     ggtgaaaccc catcgctact aaaaaataca aaaaattagc caggcatggt ggcgggcacc    363300
     tgtagtccca gctactcagg aggctgaggc gggagaatag cgtgagcccg ggaggcagag    363360
     cttgcagtga gccgagatca caccactgaa cttcagcctg ggcaacagag ggagactcca    363420
     tctcaaaaaa aaaaaaaaag aaaacaggaa taaagcaaat tttatgaact gtcatgactg    363480
     tagtttttgg caaggacatt atcatgtcaa cctgtaaata aacagacaac aataaaacat    363540
     atcaaatcca tggggagtct gacctgtgtc cctctgtctt ataagcacaa ggctttgcca    363600
     catccaaaat attattcagg ctccagggta taaaatgctt ttggactgtg gaaggtaaca    363660
     gctctcccct caggcagggg taaggtatct ggggaatgca gagttgtgtt cataaggaag    363720
     atgtcattat cgtgtcttct cctgtgcctg gtagcaggtc ctgaaggtga gcgtctcaga    363780
     tgtcagacat gggtctatcg ggttaggata ggtacatgtg actgacaggg actgattctc    363840
     catgtaatca catgccctgt cccaggagca gctgcaggag tcagccctgg acctgaatag    363900
     cacacactta ccctctgcct cacctacact gttactggcc actccgtcac aaccagtcct    363960
     tactagtgga cctggatctg ccggctctca gggaggggct gcaatggata aaatccattg    364020
     ctagtggtgg tgggagtcca tttgtcttgt ggaaaatggc agcatttcct tattttataa    364080
     ggcataataa tgctatgttg tgtacacata ccacattgtc tttatccatt tgcccattga    364140
     cagacactta gtttccatat cttggctgtt gtgaatacag ctgcaataat cacaggagcg    364200
     caggtatctt cacaaggcgg taatttcatc tctttgggta tatttccata agctgcatca    364260
     ctggtcatat ggtatttctg ttttaattca tttaggagcc accacactgt tttgcctaat    364320
     ggtaatgatg ggaatacaga atgtcatagc cgctacgaag aacagtttta gatttgaggt    364380
     ataatccaaa agcaaataat gtttgatcag ggttctcata tgaggctcta ataaataagt    364440
     ccagaaagtt ttcccatctt gggcaaggat gagtttgccc ctaattgtct ttgaggaaga    364500
     agattggcag aacgtgagat ggagtctgtt ggcaggattc aggattcagt aataactagt    364560
     aatggcacag aaaaatagag agatagagac atacagagaa agagagaata tgaatctcat    364620
     aagaggaaaa tttgctaaat atcagcattg ggttttcagt catagacaca tttgtatgag    364680
     ccaggaacca tggagtcaag tgaggagtgg agaatatgtt cagtccaaaa atcagcatat    364740
     tctcagaggc acccattgcc ccatcacaca ggtggagaat tttggaaacc agtgaagtgc    364800
     gagttcacat taggtgatta agctaccatg tttcgataaa ctttcattaa catgcaaagt    364860
     acttccatag ataactgatg cacaaatata cgagatgcct ttttgcgttt atggatgtct    364920
     agagaaaaat aagtgagaaa atttttccag gttgcagaga tctgtttaag ttgcagattc    364980
     cataggagag tgtctttgaa tgaatactgt tctattaatt aaataggtca aaattccctc    365040
     tgttggagca gccttccaat tatatagatt tctttatagc ttcttgagtt gtgaaacata    365100
     aacccaagga ttgacttact ggaatatgac tgctgtgttg attaaatttc tgataaggtt    365160
     tcttccaatg atttgagaat agctttcctg tttttttact caaggaaatg aattttcaca    365220
     aggtctcagg acatcacgtt tcaggtgctt tagttaaaga gactcttctt gggggcagtc    365280
     agctttcttc caaccatgag ctcatttctt cagcaaacca ttcgctttgt gcctctatta    365340
     agaattatga agcttttgta cttcaatgca ctgaaaatca tgccccttga attaaatgtt    365400
     gattggcact gacatgaatt ggccactctt gtatatgtgt cctatgttat gggctcaaca    365460
     taacagtgga cagagaatcg tccattagct gcctctgatt gtggcactga ccagattgag    365520
     aatcctcaga gtcatccatg aaaaagagag tccttaggtt catgaggttc ttggagacat    365580
     tcaggtaagt ggaggagaga aacaggattg ggggctgcca gccatttcaa caagactggg    365640
     aacaattatc atctacgttt aaaggtctac atcattccaa acaccctcca ggtttctctg    365700
     agttcattgt gatgattaca ttcagctgct tctccagtga gtttaaatga gcatgtggca    365760
     attcagatga gcctggctgt ggggtttatt atatgtaaat ctgaactaca taaacaaaga    365820
     gggcatgtct gaccgtatga gggtgcaaaa tcctggagac cccacacccc acactcttgt    365880
     gctttctttt ctccagcaac ctccaggttt ttttgtatga gaattgtaat agattatctc    365940
     atggctaagt catcaagaga cgtaaactct gagaaataca agcagaaaat tctccagata    366000
     gaagatccag gggaagaaca tttctagcac cttatctatg taggggaagg cagtccatct    366060
     ccattacaga cccctcacag cagccttcct ttataaggaa aatgggtcaa attagccaat    366120
     agggtaagaa tcctataatg ttccagccag aaaagggaaa gcatcaattt gacttctgga    366180
     gactctgtat ataggtcaca ttccatagaa agaagagcac taaattatta agattcaatt    366240
     gtaactacat atgatacttc cgggatgcac aaattaaaga tgaaagtgtg acaatgcaca    366300
     ggctctgtca gagaaggaga acgtagggaa actgaaagag aatggcagag agtaagacaa    366360
     ggacagaaga gcaatatgaa gcctctgaca tcaactctgt aaggacaagg tcttggcaac    366420
     tcccgcattg accacagatt ctgttaccat gggccatttg cagggttcct agctaaggaa    366480
     aaagaaaaaa aaaaaaactg catgcacttc ccaagtctcc acttgtatcc tgtttttctt    366540
     agatctctag gacaaaaaaa atggcataaa cctagaccta gtgtcagtgt aggaggtact    366600
     ttctttatag gcaagacact aggaagaagg gaaaatgtgt gttattggac taggagatac    366660
     agagatggct ttaatctgaa tagatctaca cccgcaggta ttctcgaatg caacattcaa    366720
     ctacaagagc ctaatgaaga aacacgaccc tccccaaacc cctgcaagct cttgtattca    366780
     ctgtgtctcc actgatttag tgcacctgga gcttcagagc actggctccc tcctgtgtcc    366840
     tagaatcttt tctttgggtc tttctgcaga attcaatagg attagttagg ctaatcaatt    366900
     gtttcaagag atggtgttgg cagcatatat tgtcgctgga agagtattct aagccaggac    366960
     acaggcactt tatgccggac taaagactct ggagaaaatg ttttgtgagc cctgacagaa    367020
     acctccttga aaggtaaggg ctgggcagga ggggcactca ggagccacgc agcacacggt    367080
     ccagccctag aacaggaggc tggggaggag gtttcctctc agggcctcgg ttttccttcg    367140
     tcagaaaaaa aaaaatctaa aataaccgtt caacaagttg ctgatatgcc ttcaaatatc    367200
     ctgctacaat ggaacaattc atataacttc aggcaatgag aactattttt taaatcgggc    367260
     ttctaggatt ataagtatct taatagtgaa aatgtgaaga ataggtatga ttttactatt    367320
     tcaatggata cagaattgtg ggagtcacta tattcctatg aacaaaaaat tcagatttca    367380
     gtgttaagta atgttgccta cattgtgtga gtgacggggc agtggtggat ctgagagtgt    367440
     ggtgggtgca cggacataat gattcagaaa gcaatatgga aagatgagta tctatggata    367500
     cgaactgaaa gtatgtaaat acttcacaaa atactaataa acggagttga atataaaacc    367560
     cataattatc caaaacacaa atttcttgga agttattttg ggaacatgat ttcataaaga    367620
     actccaaact cttgtttcaa cttctgactc ctcgtttctg tgatataaga aaaccatttc    367680
     caattatgca tctcagggca attctgtaaa cccagagcat ttctgctgaa gatcctgggg    367740
     aatcaagaca ccgggcaggt gatggagaca ctgtctcagg tgcgcccaac gaatctcaga    367800
     ggaacctgct ggagagtcac gtggaacatc tacagtcagt ttctcagagt caacagtgag    367860
     ctgtgttggt gcctgagggg accatgatgg ggccaaggca cgtgctcagt gtcgtggaca    367920
     gtgatggtcc agaaatgatc tagatggtct tgacgctaat gaaatatggg ttcagagtga    367980
     ggagcataat ctgtggggac ttgttcttca gtgaaaggat cctgtccgca aacagaaatg    368040
     gagcaggaca tgcatttctt caagcaggat tagggcttgg accatcagca tcccactcct    368100
     gtgtggcaga tgggacatct atcttctttc tcaacctcga tcaggctttg aggtatgaaa    368160
     taatctgtct catgaatatg caaataacct tagatctact gaggtaaata tggatacatc    368220
     tgggccctga aagcatcatc caacaaccac atcccttctc tacagaagcc tctgagagga    368280
     aagttcttca ccatggactg gacctggagg gtcttctgct tgctggctgt agctccaggt    368340
     aaagggccaa ctggttccag ggctgaggaa gggatttttt ccagtttaga ggactgtcat    368400
     tctctactgt gtcctctccg caggtgctca ctcccaggtg cagctggtgc agtctggggc    368460
     tgaggtgaag aagcctgggg cctcagtgaa ggtttcctgc aaggcatctg gatacacctt    368520
     caccagctac tatatgcact gggtgcgaca ggcccctgga caagggcttg agtggatggg    368580
     aataatcaac cctagtggtg gtagcacaag ctacgcacag aagttccagg gcagagtcac    368640
     catgaccagg gacacgtcca cgagcacagt ctacatggag ctgagcagcc tgagatctga    368700
     ggacacggcc gtgtattact gtgcgagaga cacagtgtga gaaaccacat cctcagagtg    368760
     tcagaaaccc tgagggagga gtcagctgtg ctgagctgag aaaatgacag gggttattca    368820
     gtttaagact gtttagaaaa cgggttatat atttgagaac aaagaacaat agaaacacaa    368880
     tcgaattgta agagaaatat tccattcaag agccaccaca taagccaaac tgacagagtg    368940
     ggaaaggcca cactcagtaa agttgataca aacataccat aaaggtgcta ctatgaacaa    369000
     gtttttgaat tagatgaata aatcatttgg agcaaggtta tttggtcata tgttaagagt    369060
     aagcatgatt cttacaaagt gggaaaattg tctttcaaat gtttctgtca cttcttacca    369120
     taaagttcat tttagaggtt ttaggattac agtgaaattg cacagaaggt gtgagaattc    369180
     ccatgaatcc ctgccccgca cggacaccgc ctcctccact acagccatcc tgccccacag    369240
     tcacaaataa gtcacaatgg atgaatctac aagaactctt ggttctttct ttttctggtg    369300
     atcccctaat ataacaagcc taaattatct tggaacaccc aggtattttc aatggctttc    369360
     tagaagtgat attagtcaga gggaaagtga gtgaggctat tactatttga gcactttctt    369420
     ccaaaatcca caaaatatat gttaatttgg agtttttctt acttctggtt tacaatgtcc    369480
     cttcccagag agtaagattt ttaagctttt agtgaggctg gaaaaaaaaa tttttaaaaa    369540
     agagaaataa gctttcctgt attaggctga cttatcccag cggcagcaac aagcacagcc    369600
     cagacccagg aaaagtctta ataatattat ctaatgtgct ctggagactc tttcagcact    369660
     ccctcaacat agggagaaga aaaacaaatt ttcctttgtc ttatgatatg agtttataga    369720
     ttcttgttct ctgtaactag taacttcaag tattctgttt tatctaagaa gcacaacgaa    369780
     ggtcatgaga agcctgagca ggccagaact acagctgtct aggtaccgga gtgagtgtta    369840
     tgagatcaac cagtgcaagg ctctttagaa caaaacctag ataacagaca tctgggttgc    369900
     atagcaatgg tcatgtgtaa tcctgagtta tgaacctgtt acaatttgat taactgtctc    369960
     tgtcctgcct ccgtatccct gcttttgtgc actctaagct tgcttcaagc tagcccaccc    370020
     cattttggga agtgtgtata aaagtcaagc gctctctttg ttctgtgccc agtctttggt    370080
     cattgagtct gctgggtctg ggtgtactca gtaataaaaa tatcctcctg tatacacccc    370140
     aagatctctc tctggtcctc cagattctgc aacatttcag gcagattcac atctctaaaa    370200
     ggccagcaag ttctggtcaa tcccataatg aaaatccttt aatgagactt ggcaaacgtg    370260
     acaataagag actccttgta taatgcccta gagttggatt agacacactg tgagctcttg    370320
     ggtggtggtt ctgaataagg cagtttgtgc agcaaatgca aacacatgca tgggatccag    370380
     gcaggaacaa aagcttccct ttacaaagtg ggtgggcatc tggagggagc cctcagaggt    370440
     gggcagtggt cgtccttgct gactgcacat tagccagagg cgtgaccata attggtcttg    370500
     cagggaaaga gcaccactga ggtcataggt tatgaaaatg tttgtcatcc tccagtgagc    370560
     aagtccatct gcttgcttgt gggtgtcaac tccatggtgg atacactctg ggagatgaca    370620
     agatgcacac aaacctcctc tcactaatta tccactacca cacactcaag accaaccttt    370680
     gctccagaaa ggaatacgtg tctgtggaaa tagacagagc ttaagtattt tgtaacctgg    370740
     tgaacatact gtgcaaaacc aaacgtttca ggaagattag ctcagaaatg tttatcaagt    370800
     gactgaaggg cagtggcggg tgaggtgatg ggacagcctc agggctgcac atgaggaggg    370860
     ctccctctcc catgcaggct tttcctccag gagctgcacc aggaactcaa ggaagatcag    370920
     ggagaattct gagaacaccc tgctgtggag ctgcctagag aagaagaata aatgatgaaa    370980
     aatacaactc tgagtaatgc atgggttttt gttcatgaaa actctctctc tgaaagcttg    371040
     tgaaggtctt gaaatacccc tgattagctg aagacaaaca tttaaaccct ccttccacag    371100
     ggagttcaag caggctggat gtgtccttct atggatgatc ttcctcagcc ccttcctctt    371160
     cccagctcat cactggctct ctgtgtaaaa agttctcatc agcggaatgt ggttgatgaa    371220
     gtgaggtctt caattttctc atcttctatg tggtcatgtt attttcctca tctgaagttt    371280
     aaaaactcac ctgcatgcag cacatgacag gctaaaatct cttgtggaca aaacagtaac    371340
     aaaggcaccc accatggttg agcatcccgt gttgctgaca acgaccacca ggggtcaacg    371400
     tcctcttcac aatcctgtgt gagagcagca cttgagtgat ttcaataaca acttcccagg    371460
     agaatcagct gaaaactact tgtcccattt tccatacaga tataacccct ctattttcct    371520
     gaagaaatag aaagagctga atgctgaata cactgaatgt ctgctggttt tgcaagtttg    371580
     tgactatatc actttctaat ttctgacctg tgcagaccac tgtacagact tttctcactg    371640
     gtgggaccag ccttccagat gtcaaatata actgagcttc ttcatataaa agtcaacaca    371700
     agctcctcat ggtttcagtg ctcactgaat ggagttggaa ataaaaccca caattatcca    371760
     taacacaaat tccttggaag ttattttggg ataatgagtt cataacctgt agaccaagag    371820
     tccaagagtg tttctattga agagcctggg ggatcaagac accaggcagg tgatgcagac    371880
     actgtctaaa gagtgcccag cggctctcag agggacctac tggatactca cgtgggacat    371940
     cagcaatcac tttctcagag tcaccagtga gctgtgctgg ttcctgaagg gtccaggata    372000
     gggccaaggc acctgctctg tgtcgtggag agtgattgtt ccagaaatca tagaggtggt    372060
     ctctatgctt ataaaatcta tgttcacagt gagaagtctg ttctgagagg gcttattctt    372120
     cagtgaaagg acctctgccc acaaatgttc ataaatggag cagggcattc atttcctcaa    372180
     gcaggatcag ggcttgagtc atcagcatct cactcttgca aggctgatgt gtcgtttgtc    372240
     ttccctttct tatcatcgac caggctttga gctatgaaat gccctgtctc atcaatatgc    372300
     aaataacctg agatcgactg aggtaaatat ggatatgtct gtgccctgag agcatcaccc    372360
     aacaaccaca tccctcctct agagaatccc ctgaaagcac agctcctcac catggactgg    372420
     acctggagaa tcctcttctt ggtggcagca gtcacaggta aggggctccc aagtcccagt    372480
     gatgaggagg ggattgagtc cagtcaaggt ggcttttatc cactcctgtg tcccctccac    372540
     agatgcctac tcccagatgc agctggtgca gtctggggct gaggtgaaga agactgggtc    372600
     ctcagtgaag gtttcctgca aggcttccgg atacaccttc acctaccgct acctgcactg    372660
     ggtgcgacag gcccccggac aagcgcttga gtggatggga tggatcacac ctttcaatgg    372720
     taacaccaac tacgcacaga aattccagga cagagtcacc attaccaggg acaggtctat    372780
     gagcacagcc tacatggagc tgagcagcct gagatctgag gacacagcca tgtattactg    372840
     tgcaagatac acagtgtgaa aacccacatc ctgagaccgt cagaaacccc aaggaggagg    372900
     cagcttcact gaatgaggag gttacagggc ttacgatgtt taaagttgtt cagaaaatag    372960
     gctaagcaat tgaggaatat gagtaataga aatatgtatg cactctatac aggaaatatt    373020
     tctaataatt gtcaccctat atgcaaaatt cgcagagagg taaaagcaga aatcagtcaa    373080
     gctgatgcaa agttccccac gtaggctttg tgcagatgta agttctaaaa tcagatagat    373140
     aaataatttg gagcaagatt gcttgataac atggctaatg ctgaatatga ttcctaaaaa    373200
     ctggccaaaa tatattccaa tttgtctctg ccacctctct tacataaaat gtattaaaaa    373260
     gtagttttaa gaccacagca aaattgaaca gaaggtgcag agatttccta tgtgcccctg    373320
     cttcacacat gcacagcctt ccccactgtc accatcctgc cccagagtca tcaataagtt    373380
     acaatggatg aacttacatg gatggattgg ttctttcctc ttccggcggt cccttggcat    373440
     accaagtcta aactatcttg aagcacaaca ggttcttcga gtgggttcct gggaatgaag    373500
     ccagttagag gaaaagtggg tggggctatt cctattagga gtctttttta gaagactcat    373560
     aaaatgtata tgttcctata gattctgtga ctcctgactt agtatccctt cccagacggt    373620
     aagcttccta aatgtttaga ggcagatcca tatctatgga aagaaagcaa gttctagtga    373680
     atcccataag gaatgtcctt taatgagaag tggagacctt ggtcatgagg cacatcatgt    373740
     atgattttct ataattcctt tagattcact gtaagctttt gagggtgttt ctggatgagg    373800
     ccctttgtac agaaaatgaa aactcaggca tgagttccag gcacaaccaa ccaacttcct    373860
     tccaaagtgg gagggaatag aagaaaccct ctcctgtgtg ggcgctggtc cccctccatt    373920
     gctggctgca cattagccag aggcatgagc ccaattagtc ttggagggtg acagccccac    373980
     tggggttgct ggctatagaa atgcctgtcc tcttccagct gagtgagtca acctgctcgc    374040
     ttgttggagt caactgcatg gcaggtgcac tctggaagat gacaagatgc acacaaacct    374100
     tctgtaaagt atcaattact acacactcaa aaccaaactg tattccagag acaggtgtct    374160
     gcagggataa acagaattta agcatttttt gaatagggaa gacactgcca aatgccatat    374220
     gtttcaggaa gtttaactca gaaatgttga tgacataact cagaaacgtg aggtgacatg    374280
     acagcctcag gggctgcaca tgaggagggc tcacttcccc atgcaggctt ttcttccagg    374340
     aactctacca agaactcaca gaagatgagg gagattctga aaacatcatt ctgtggtgct    374400
     gcccagggag gaaaaataag atatgggaaa aaaaaactat ataaattatt agatttgtta    374460
     atacaaacta tttctgaagc cttgtggagg tcctgacata agccatcatt agctgtgaac    374520
     aaatatctac accctccttt cctggggagt tcagttaggt tgcgtctctt cttttatgga    374580
     cagtatcccc caaccccttt atttcctgca cacctgctgc tctccatggg acgagttctc    374640
     atcagtgaaa tgtggttgat gtagtgaggt cttcactttt ctcattgtat tagtcaggat    374700
     tatctagagg gagaactaac aggatagagg tctgtatttg acggggagtt tttaaggagg    374760
     actgactcac acgatcacaa gatgaagtct cgtgataggc cgtctgcaag ctgaggagca    374820
     aggaagccag tccaagtccc aaaacctcga aagtcaggaa gacgacggtg cagccttcgg    374880
     tctgtcgcca aaggccagag agcccctggc aaaccactgg cttaagtcca agagtgaaaa    374940
     agctgaagaa cttggagtct gatgttcgag ggcaggaagc atccagcatg ggagaaagat    375000
     ggaggctgga agactcagca agtctagtct ttccaatttc tcctacctgc ttcattctag    375060
     ccatgctggc agctcattag atggtgccca gggaggttga ggttgggtct gcctcaccca    375120
     gtctactgac tcaaatgtta atcttctttg gcaataccct cacagacaca ctcaggaaca    375180
     attctttgca tctttcagtg caatcaagct gacactcagt attaaacatc acactcatct    375240
     tctgtgttgt catgttcctc tcttcatctg aggttaagga actcaccagc atgtagcaca    375300
     tcgtagctta atgtctctca tggacaaaat agtggcaaag gcacccacta gggttaagca    375360
     tcctgtgtcg ccgacagcca ccaccacagg accaagtctc tctaccatcc tgtgtcaggg    375420
     catcgcctca gtgattgtat tggcaacttc cctcgagagt catcttaaaa acggcttgtc    375480
     tcatttccca cgaaggtata gcccatccat tttctcttcc tagcaaaatg gaaaaggatg    375540
     aatacacgga atgtctcctg gtcttgcaac tttgtgactt tattcctttc taatttctga    375600
     tcaatgcagc ccactgtaca attttttttt tcactgaaat gacccacatt ctggatgtta    375660
     aatataattg aacttcctca tttataattc agcacaagta cctcagagtt tctgtcctca    375720
     tgcaccactc taaacttaca caagttgttt atttatttgt tttattttct ggcatctcta    375780
     cttggcgtct tcatcatacc cattttatgt tatttctaca aatgattgtt aaaatttata    375840
     ggtacttaat taaatattca ggaaacaaga gaataaaatg aatgtaaaat aaaataaaag    375900
     taaataaatt aatatttaaa ataaagaagc cagcagctga aagcaaggaa aatctattat    375960
     gctgggtcag agagtatgga gaactcatag tacaatatct tctgcacgtt aatcattact    376020
     tctactcaag ttacagggtc ctctctcttt atatcagagc tttccctaac atgtgcaatg    376080
     aagactgaac acagattcct ccctcaagac tgacctagca tctctcactg agcctgagct    376140
     gacaagtcat agatatcaca tttgtctaag aaaataatga caccctgtaa agttacctac    376200
     cagcgatatg gagacaattt gaataatata aataaagcct aaacttgata ggtagtcatt    376260
     tatccttaga aggattgact gctgtctcta catgtgtttt ttatcacaga gaagctacta    376320
     gtgccacagt ccaagttgct gaatatttta tttcctagca tgagttacta cagaatgtgt    376380
     tcaacatgaa agaatgcagc tgttgttaag ggagaggaga tgtgattatg gaacacgagc    376440
     agggatccag cagacattcc tcttccacgt ttgccccaaa acagagacac cagtaaaacg    376500
     atgaaatgat tgactttagt ctccacagtc taatgtgaga tttataccag atgttttctg    376560
     ccactgtttt atagtagaaa atataatctc taaagaaatt ccatttttag agacaatacc    376620
     atcctccagg aagctgcaaa tgcccataac agagagtatt atagtgcctt gtggccagga    376680
     gctacaacac ctgggtttgg aaactaatgg acagaaatat caggttttct tgccaaatct    376740
     atgatgaaaa tttggagaat gtttctttcc atctccataa caatatgttg tgatggagtg    376800
     gaggtcctgg tacataagaa ggtatgtgtt cacagtgggt acagaattag acccataaaa    376860
     tgattgttct actggggttc ccttgttgtt ggaccagctg accaagaaag tgttgtaaga    376920
     ggtgtagtat ttagtgaata ctgtcatatt tttgaaggta ccatatttga agctaccagg    376980
     agaaacagag atacatcaat ctagcctagg gagactctat gaatttattt tatttccttc    377040
     aaaatgaggt aaaaaattgc atcagttgaa atgccgatga taatcaagtg atgaaagaca    377100
     acatccactc agtgttttag gtgtggttta acccaaaagt tatacaatat gtgagaacct    377160
     gaactctgga gccctggcca tctctcagaa gaatctagaa ggtagactga gcctctacca    377220
     cagtgtgatg tccatggaga ctggggatgg ctactgtctc aatcttggga tcacggaata    377280
     gtcttaatat gattctgtgt gtatgtggat cattgtgtgt aagtgggagg attcctataa    377340
     ctgtgtctgt gtgtttgtga gtgtctatgt ttctgtgtgt gagcttgtgt gtctttgttt    377400
     tattttaaaa tgagtaattt agtttctgat cattagactg tgtctttgag ggagtgtgtg    377460
     ggagaatgtg catgcctgtg tctgtgagtt agtttgtaaa agggtatgtt atattgaatg    377520
     tgtgtgtctg ggtccttcag tatttgagcc tattaattta catgaacatg aattttttga    377580
     aggtgtgtaa gtataaaact tttgtaaatg taattgtttg aatgctagtg tgtgcagagc    377640
     tatttgagtg tgaatgtgtt acttgtatta cttagtgagt gtgtgcccct atgtgagttt    377700
     gtttgggtga gtcattacct atgactctac atatgaaggt gttgagggtg tgtgtgagtc    377760
     tgtgagcatg ttttcattac ctatgactct acatatgaag gtgttgaggg tgtgtgtgag    377820
     tctgtgagca tgtttatgtg catgtgaaat gttgttatgt atgctttgaa agacttgtct    377880
     cttgacctca atatcatttg tctgaaaata ccaggtatgt ttaagccctt agaatctaag    377940
     gaatagtttg ttgtttagga taaatcctca ataagattaa caactgattt ctcaccaaaa    378000
     atcaggaagg ccaaagctca ctggagtggc atatcaaaag tgctaaaata cactatcaat    378060
     caaaaattcc atactagcaa gaacaccttc aaaagtgaag gagaaatcaa gataatagaa    378120
     ataaacaaaa gctatggagt ccatcaaaac cacaaattcc taatgagaaa tactaaatag    378180
     agtcctgcag gctgaaatga aaacagagtg gagaatattt aaaaatctta cacctaaaag    378240
     aacaacaaca acaagtatcc cagtaatcac atttgtgtaa tgaaaaccaa tattgtttta    378300
     tttttgcctt gagtctcatg ttctattcct tttttaaaaa aaaaatagat ataatagtaa    378360
     tgacaaattt gaataatgtg cctataattt atggttataa aattcattat aaatttgaat    378420
     aatgtgcata taatttataa tgtatgaata tgtaacaaca caataagtga aaatggatac    378480
     agctgtagag aagcagagtt gtatgcacta ttgaactaag gcaatcataa tttaaactac    378540
     tctgttataa atctgaatta ccaattgtaa tatccaggtt agctataaaa acttaagtaa    378600
     aagtaaaaaa aaaaaaaaat tagcaggaca gctgactaaa cgtgcctgac actcttcaac    378660
     cccacatgaa ataaccacag aaatgaataa acagctgaga ctagagaaat tcaacagagt    378720
     tagatatcat acaaaataat aaagcaaaaa gcttacagct gaagaattta atgaataaaa    378780
     taaaaatgca gtttacagat tcaataacag aaaataacga gcaaaaataa gaatttctga    378840
     gcttgaagac aagtctttca aaataattcc agcagaccaa aaagaaaaaa aaagagaatg    378900
     aaagagagaa ggaaggaagg aaggaaggaa ggaaggaagg aaggaaggaa ggaaggaagg    378960
     aaggaagata aaattttaaa aacatagaaa ccctatagtt ttcaaaacac aatttgaaaa    379020
     caagtttttg agttatggaa aatccagggg aaaattccag agggaaaaaa atgaaaatga    379080
     tgtaggaaac atatttaata aaacaagagc agaaaacgtc ccatatcatg ggagatagat    379140
     ggttattcag atccaggaaa ctcaaatatt ccaaacagac tgaaactaaa cagatcctct    379200
     ctgaagaatg ctttcctcaa attatcaaaa gacaaagata gagcatgata gagtaggata    379260
     gtatattcaa aatattgaaa taaaaaagta tccagccaat aatataatac ctagaaacat    379320
     tattattcac aaactaacaa atatataatg tttatcggac taacatgaac aggaaatcca    379380
     tcacctccag gctggcctta caagaaatgc tcaagattct tacatctgga agagaaaaca    379440
     taatagccac aattatgaaa aatttttaaa aaaccataaa acccactagt aaagccaata    379500
     cacaaaaaag aaaataaatg aatcaaatct tatcactaca aaaattacaa aactacaaaa    379560
     ataaccaata ggttaagaat aagaaacaag agatacacaa aacaatgaga aaagaatcaa    379620
     tgaaagtgaa agaagtaaga cctcttctat ctattattag cttgaaagta agtggattaa    379680
     attttacatt taaaatacat agactgagtt gaagagaaaa gaaaagaaaa aaagaaacaa    379740
     acaaacaaaa gtgccaacta taggctgcta tgttaagccc attttgcatt gttataaaga    379800
     agtacctgag tctgtacaat ttactttaaa aacaaaaaca gtctacaaat tcaatgcaat    379860
     tcccatcaaa atatcaccat cattcttcac agaactagaa ttcatatgga accaaaaaag    379920
     acttggatag ccaaagcaag actaagcaaa aagaacaaat ccggaggcat cacattacct    379980
     gaattcaaac tatactatag ggccacagtc accaaaacag catggtactg gtataaaaat    380040
     aagcacatag atcagtggaa aagaatagag aacccagaaa taatgccaaa tattatatca    380100
     atgaatcttt aacaatgcaa acaaaaacat aaggtggggt atggagacca tattcaacaa    380160
     atggtgctgg gataattggc aagccatatg tagaagaact atatcctcat ctctcacctt    380220
     atacaaaaat caactcgaga tggattaagg acttaaatct gagagatgaa aacataaaaa    380280
     ttctagaaga taacatctga aaaactcttc tcaatattgg cttaagcaaa gtcttcataa    380340
     ccaagaacca aaagcaaatg caacaaaaac aatgataaat aggtaggact tgattaaact    380400
     aaaaagtttc tgcacagcaa aataaacaat cagcagtgta aaaagacaat ctatagagtg    380460
     ggagaaaatc ttcacaatct atacatccaa caaaggacta atatctagaa tctacgagga    380520
     actcaaacaa attagccaca acaacaaaaa caacaacaaa aaacaattcc ataaaacaat    380580
     aagctaacga cataaacaga ccatcctcaa aataagatat acaaatggca aataaacatg    380640
     aaaaaaatgc tcaacatcac taatgattag ggaaatgcaa atcaaaacca caatgcagat    380700
     gcaagtcaat accacattac tcctgcaaga atagtcataa tcaaaaaata aaaaaataga    380760
     tgttggtgat gatgcagtga aaagggaaca ctggcgggaa tgtaaactag tacaaacacc    380820
     atggaaaaca gtgtggagat tccttaaaga aaaaaaggag aacaaatatt tcatccagca    380880
     gtcccattac tgggatctac ccagaggaag tcaccataca aaaaagatac ttgcacatgc    380940
     acatgcatgt tcacagcagc acaattcgca gttgcaataa tatggaaccg gcccaaatgc    381000
     ccatcagtca atgggtggat aaagaaattg tgatacacac acacacacac acacacgcac    381060
     accattttat atatatatat atatatataa tttttttttt tttttttttt ttttttgaga    381120
     cggagtctcg ctctgtcgcc caggccggac tgcggactgc agtggcgcaa tctcggctca    381180
     ctgcaagctc tgcttcccgg gttcacgcca ttctcctgcc tcagcctccc gagtagctgg    381240
     gactacaggc gcccgccacc gcgcccggct aattttttgt atttttagta gagacggggt    381300
     ttcaccttgt tagccaggat ggtctcgatc tcctgacctc gtgatccacc cgcctcggcc    381360
     tcccaaagtg ctgggattac aggcgtgagc caccgcgccc ggccatatat atataatttt    381420
     atattttcta tatatacata ttttttatat atgtacatat atacgtgtat atgtgtatat    381480
     atgtatagat gtatatatat acacgtgtat atatatgtgt gtgtgtgtgt gtgtatatat    381540
     ttaccatgga atattattaa gccataaaaa ggaacaaaat aatgacagtc acagcaacct    381600
     ggatggaatt ggagaccatt attctaagtg aagtagctca gaagtgggaa actaaacatt    381660
     atatgttctc actcatacgt gggaaataag ctatgaggat gcaaaggcat aagaatgata    381720
     caatagactt tggggacttg gaatagatga ggggggctga gggataaaag actacaaatt    381780
     gggtacagtg tatactgttg gggtgatggg tgcgccaaaa tctcataaat catcaccaaa    381840
     gaaattactc atgtaaccaa accctacctc ttcccccaaa acctatggaa ataaaaataa    381900
     aattatgtaa agtataataa aataaaggta tatttggctg acagttctgc aggctgtaca    381960
     agaagcatag tactggcatc tgcttctgtc gatggcctca ggaagttttc aattgtggca    382020
     gaaggtgaaa aaggagcacg tatgtgaatg gcgatagaag agaagaaaag gcagaaaatt    382080
     aaggttgttg ggtgggtgcc atgctcctta aacagccagg tatcatgtga acgaatagaa    382140
     tgagaacaca ctcattacca cgtgaagggc atcaaggctt ttatgagaga tccgtgttca    382200
     cgacccaaaa acctccctct aggtcctacc tccaacaccg gagataacat tgcaacatga    382260
     gatctgaagt gaacaagcat ccaaactata tcagttacct acaaaaaact cactccaatt    382320
     gtaatgatgc acttacgttg aatatgaagg tatggaaaaa gttatgtaat gcaaataaaa    382380
     actaacttca cctggagtag ctatacttgt agtggataaa ccagacttta agttaaaagc    382440
     tgtaaagaca gacaaagaag gacactatgt ataaataaag agttcaaccc aggaagaagg    382500
     cataagaatt ttaaagatat atacccaacg atacagtacc caggtatgca gagcaaatgt    382560
     tatcagatct aaaaggatag acaccaatac aataattgct ggcgacttca atccctcact    382620
     gaaagcattg cacagtttat ccaaagagaa aatgaacaga aaatttcaga ttaaaattgc    382680
     accagaggcc aggcacaata actcacatct gtaattcccg cactttggga ggctaaggca    382740
     ggtggattgg tagagagctc aggagtttga gactagcctg ggtagcatgg caaaaccctg    382800
     tctctacaaa aaaaataaat aaataaaaaa taaaaaacag ccagtgtggt agcatgtgcc    382860
     taaagatcca gctacccagg aggctgaggt gggaaaatca ccagaaccca gggagataga    382920
     ggctccatag agccctgatg gcacccatgc acgccctcct gggcaacaga gcgagactgt    382980
     atctcaaaaa caataattgc accacagact gaacggactc aacagacact tacagaacac    383040
     ttcgccaaac agcagcagat tgcacattct ttttaacagc acaatgaaca tcctccagaa    383100
     tttaatatat ttggacacaa aaaagtctcg aaattataaa aatatcaaag ccatatcaag    383160
     tatcatttcc aaacacaatg aaataaacta gaaaccctag aggatggaat atttgaaaca    383220
     atgcaaatat atgaaaatta aacaacatgc ttctgtgtga ccattggata aaggaataac    383280
     ttaaaatgaa actttaaaaa tttattgaaa taaataaaaa tagaaataca atatagcaaa    383340
     agctactggt tacagctaaa gcaatattaa gagaaaagtt tctagcaata aacacctaaa    383400
     tcaaaaaagt agaacgattt ctcaaaaacc acccacctat gtgtcttaag aactagagaa    383460
     gcaagaacaa acagaattca taattgtaga aaaaatagac agtaaatatc agagcagaat    383520
     tttaaaatac agaaaaaaaa atctataaag gatcagcaag gccgggcgcg gtgactcatg    383580
     actgtaatcc cagctttttg ggaggccgag gggggcggat catgaggtca ggtgattgag    383640
     accatcttgg ccaacatggt gaaaccccgt ctctaccaaa atacaaaaaa ttagccaggc    383700
     gtggtggcac acgcctatat tcccagctac ttgggaggct gaggcagggg aatccctcga    383760
     atctgggagg ccgaggttgc agtgagccga gatcgcgcca ctgtactcca gcctggcaac    383820
     agaacaagac tccatctcca aaaataaata agtaataaca aaagggatca gcaaaacagt    383880
     tgttttcttg aaaatgtaaa aaagcaagaa ggcattatct agattaacta caaatgaaaa    383940
     gagaaaatgc ccatatacat acaatcagaa atgaaaaaag agacatcaca atgataccac    384000
     aaaaatacaa aagatcatta gagggtatta tgaacaacta tatgctagag aaaattcaag    384060
     aacctagagg aaatggataa attcatgaac acatgcaaac taccaagact gaaccaagaa    384120
     gaagtagaaa gcctaaacag aacaatgaat aacaggagat aataataatg aaaagtctcc    384180
     catcacagaa atgttcagga cctgatggtt tcacagttgt gttctactta acttaaaaaa    384240
     aaatcatgaa aacagattat tgttaaacta ttcaaaaaat tgaagaaaaa ttatttaatt    384300
     ataattttaa tattattttt aaaatttcaa cttttatttc aggtacaggg ggtacatatg    384360
     caagtttttt tacatgggca tattgtgtga tgctgaggtt tggggtatgg atcccctcac    384420
     ccagggagtt agcataatac ccaatagtca gtctttcaac ccatgcacct ctctctccct    384480
     ccccactcca gcagttcaca gtgcctattg tttccatggt catgtccaca tgtgctcaac    384540
     ctttagcact cactttcaag tgagaacatg tgatattttg tttccttttt ctgtgttaac    384600
     ttgtttacga ctatgacctc cagctgcatc catgttgctg caaaggacat gattttgttt    384660
     cttttgtgac agagtctcac tctctcacca ggcaggagtg cagtgacgcg atctcagctt    384720
     actgcaacct ctaagtccct ggttcaagtg attctcctgc ctcagcctcc cgggtagctg    384780
     ggattacagg catgtgccac tatgcccagc taaattttgt atttttagca gagagggggt    384840
     ttcactatat tggccaggat gatcttgatc tcctgacctc atgatctgtc tgcctcagcc    384900
     tcccaaagtg ctgggattat aggtgtgagc caccacactt ttttttatga atgtgtggta    384960
     ttttatgatt ttatattgca tacataccat tttattcatc taatacatca ttgattggca    385020
     cctagattga ttccatatct ttgctgttgt gaatagtgtg gcaagaggca tgagtgcatg    385080
     tgcatttttt ggtagaatga tttattttac tttggctata tacctaatga tgggattgct    385140
     gggtcaaaca gtagctctgt ttcaagttct ttgagaattc tctaaactgc tttgcacagt    385200
     gctgaactaa tttacatttc caccacccat gtatgtgttc ccttttcttc acttcccacc    385260
     cagcagctgt tttttgttta acttttttca tattagctat tatgactggt ctgagatggt    385320
     atttcattgt tgtttttatt tgcatttctc tgataattaa tgatgttgga aatttttttc    385380
     atgtctgttg gccactgaga ttgacaggct tacaagtgtc ttagtccagc agttttcggt    385440
     aacaggctat ccactttcat tttctcttcc tcacatgccc catttctggg ttgtccatcc    385500
     caaccaacag ttactgctgg agctgaatcc actagcccgc ttccatgggc tgtcaggaac    385560
     acattgcaag ggtgacacac acgatgatcc atctctgcag agccaactct ccttctccag    385620
     agattcatcc aagaaacaat tttgactata cctgagctct gtgacatctg aggacatggt    385680
     ttgtattact gtgcaagaca aacagtgaga ggaagtcaat gtgagtccag acataaacct    385740
     tcctgctgag aacaatggaa agcttttctt ctaagataag gaataagaaa agaatgccca    385800
     gtcttaataa ttctaatcag catattgatg atagttttaa ccatagaaat ttgagaaaga    385860
     aaaaaatagt aaaaggcata caaattgagg ggaataagtt aaattgtttc tgtttacaga    385920
     caatataact ttataaattc caaaaaaata aaaaattctg aaaaaacagc cactgaaaac    385980
     taacaaacaa atttagtaac attgcagtat acaaaattta tatggaaaac tcagtagtgt    386040
     tactaaaaac taaaaattaa ctatttaaaa atcataagac aatgtcagtt gcatttacaa    386100
     tattaaataa cttacttagt ggccatgcac agtagtttac acctgtaatt cttgtacttt    386160
     gggaggtagg ggagaatcac ttgaggctag gagtttgaga ccagcctggg caacacagca    386220
     agaccaatat ctatttctaa aaatcactta gaaaaaatgt tggtcaaata agtgaaaatc    386280
     tgtgcattga agactatatg acattgatga aataaataaa tctatatgca aatatatggg    386340
     aaactattcc atattcacag attgaagata ataatactgt taacatgtct atactaccca    386400
     aagcaatcta cagatccaaa gcaatttcta tacaaatgac attttcaaca aactactttt    386460
     taaatgataa aattgaatca caaaagaccc cagatgtcca aagtaacctt gaatacaaac    386520
     atcaaagcgg ggggaggttt cctagtacct gaactgcaaa tatgctatga aactacaata    386580
     ataaaactgc atgctgctga ctttctaaat aaactttaat attctgaatt gaagttattt    386640
     gacaatttct tctatcattt tatctttact aaagtgttgc atgagtgttt atctagacag    386700
     ataatgtgca aagagtcttc ctgggtggta aaatcccata agcgtctcca ggagctccga    386760
     gctctctcta ggcttctcag tatgttcagg ttcaagagac agaaagaatt ctctcagccc    386820
     ttcactgctt tttgccctct tgacggtcag aagtccagaa catgctttgt ggggacataa    386880
     tagaggggac caaaatagac actttcagca aacccactat aacaagttct gctgcccatg    386940
     ggcatcttct ccaggtacat gttggtcgcc agaccctcag gctcacctac accatgtgga    387000
     ggaggacctc tacccacact catttcagca caactaccgt gtagctcagc atcagtaggt    387060
     cacactacaa tacattaaac atcactcagc taattactta ttgaggtttt catttgtcca    387120
     tatgctatca atattgggca gtttcaaatg acaacctaga ggacttaatg atagttaaat    387180
     ttatgtcttc tattatggaa aaacatccct tacctaatga caggttcagc tgtttttgcc    387240
     aagatggtgc tcttccctgg cctgctagtc tcctacatga gaagcatcaa tgaactgggc    387300
     agcaccacac acttgtcaag tgatcactgg gaatgatgtc agctacagag gatggggtag    387360
     ggttgctcat acctatgctc cttacttcta ggacacatca ggtgtctatg gatttagagc    387420
     atgtctgcac cctggtggac tgcacacttt acccagggag aaaggtctct gagtgtgggt    387480
     ggcaagccac ctaggtgcca aggtaagaga ccgagggcac aagctgttcc agtataataa    387540
     aatatataaa ataagaatag ttatactaga tatagatcat agatatgatt atatatgaat    387600
     atcattaatc attagtttat agcaattact ctttatccca atgttattaa taatcctcgc    387660
     tctataatca taacctagga aaaaccaagc catacagaga taggagtgga agggacatgg    387720
     tgagaagtga ccagaagaca agactgcgag ccttctgtta tgcccggaca tggccaccag    387780
     agggctcctt ggtctagcgg taacaccagc ttctgggaag atgcccgttg ccaagcagac    387840
     cgtggtctag cggtagcgtc agtgtcaagg aaaaacaccc gctacttagc agaccaggac    387900
     agggagtctc cctttcccca ggggagttta gagaagactc tcctcctcca cctcttgtgt    387960
     agggcatcag tcaggcccac ctgagttatc tggaggccta atcatctccc tctgatgctg    388020
     tgcttcagtg gtcactctcc tagtctgcta tcatgttcca tcctgtatac ctggctctgc    388080
     cttttagata acagtagcaa aattagtgac agtactaaaa gtctctgata tgcagaaata    388140
     atggcgtaag ctgtctgtct ctctccctct ctctctctct ctgcctcagc tgtcaggcag    388200
     gaaaggaccc ctgtccagtg gacacgtgac ccacttgacc ttacctatct cattggagat    388260
     gactcacact ctttaccctg ccccttttgc tttgtgtcca ataaatatca gtgcagccag    388320
     acattcgggg ccactaccgg tctccatgtc ttggtggtag tggtcccctg ggcccagctg    388380
     tcttttatct ctttgtcttg tgtctttatt tctacaatct ctcatctcca cacacaggga    388440
     gaaaaaccca ccgaccctgt ggggctcgtc cctacatctg agcttctcaa tggaggtcta    388500
     cttctctatg agactagcac cttctgtgga gtctcatgct gaatgtctcc ttcttgatta    388560
     tggtcattgg ttatgcagtg ctgtaattag aaacccttac agaataaact acaaaacaag    388620
     aatgatacta ccttcttcag acatgacaat tctacctaat gtgcacaact ggacctattg    388680
     aaatcttagc caggagagga tgcaggcata aagcagatga ttttccgatt gttccaccct    388740
     accgggttat attgaacagt ggacacagga ttcaacaggt accgattatt aatccagtga    388800
     ggaaattata gaatagtccc aagtctaggc agagaagagt agctgaagac agtactttcc    388860
     ctgcagctct gagattcact aaaatgtcca gctctgtccc tcactcgtga atatcacaga    388920
     ctaaaggaca cccttcccac agcattggct ctccatgaag atttgaaatt gatgcccagg    388980
     cctcagggtc tgctgacaga gctgtgacag tgggagtgga actcacacag agcaaaacag    389040
     agtgtgttgc ataataaggc tgtacaacat tttcataatg gagggaatct cttggtccca    389100
     ggaagatgac aatttaatat gtctcaggcc tgtggagctc tctttaggag ctttgtgtgt    389160
     ggcttctttg tatactctgg ctccaggaac tccagtgagg gaagagattc gtcacctcct    389220
     gtgcgaaggg ggctgaagcg aggaagacaa gtggcctggc ttccacagaa cccataagct    389280
     aaaacactcc gagttccctt gactgcaaga aatgcagctg tcatcacggt ttcaagtgcc    389340
     agagtcttta atgattatgt ttcaactcat gagagtgcca ggaaaattcc accagtatga    389400
     actactcaaa tatgtcattt tgtaacatta cttcattgct tgctctcata tcctccatgt    389460
     ttctgaaaaa gcaaagtttc catgctttat catctgcacc aatgaggcca tatttaagct    389520
     atattttcat atgaacgtct cttgaagatt ttaaaataga acaaatgttg aacaatatgc    389580
     ttaaaagaaa aaaaataaag aaagcagtct atgttgactc atgtgacatt gataatgtat    389640
     attagattat attggttgct tatttagttg gtcatgggag aagagccttc tttccagcca    389700
     aagaatacta agaactccct cttctaatga cgaatttcag ctgatcttgc tgagaatatt    389760
     tagcaagaag acagaaacag agactcctaa agggagctcc agagtcctga gatacattta    389820
     agttttcatt ttcctggaac ccagagattc cccactgttc tacaagacaa gaccccattc    389880
     tatgaccctg tctgaggaga gctcaggggc agtgagctcc ccctcacatt accccttcac    389940
     cctatgccct tggggctctg tccatgcaaa taagtcccgg gttcaagaac gaatagaggc    390000
     agggctgtga gtgttatgct gtgtgctggc tctgagctgg ggtctctagt agcactctcc    390060
     acatccatag atacatggcc cctccgctac tccttctctg cctgctggct gccctctgtg    390120
     gtgagttact catgatttca tacgtgggga gacacgggat gaggcaaatg tgtctgtgac    390180
     tcacagatgt tctctattct caggggttca cagtgaggac caccttgtgc aatgggagga    390240
     agaagtagtg gtccccttgg tcatgctcag cctcacctat gccgcctatg cacattcaat    390300
     ttctgaacat tctgtttcct ggatccacca tctcccatca aaaggtcttc agtgtgttgg    390360
     tgtgatatgg gttaagggaa acactaagcc ccaaccttca gggcagagct agcatctcca    390420
     gaaacacata gtaaaaaaca agaaaactta cagctgagaa gtgtgatggc tggggaggca    390480
     ggcgtgtatt actgtgctca aggcactgtg actcgaatcc agagtgaact cagacacaaa    390540
     cctgccctgc aggggttctt gggaccacaa ggggaaggat caggtcacca gggtgtactt    390600
     aggaaccact gaactgggtc aggcacagga ggtgggggtc agggctcctc cccagggaag    390660
     ggcttttaat ctccatgcct tgtaaacctc tgacaagcca ggcagacaca gacatatttt    390720
     taaactatgg aaacagatgt acctttcatt ttggaggaaa aagatcataa gtgtgaaagc    390780
     aaaacttacc aagggccagg tactggaata actgtaatct acataatcac tctaagaatt    390840
     ctaagaattt acaagtgcaa tcatcctcat ttcataaact caccttaaat aagagctttc    390900
     taactgatcc catatattct gattttgttt atttcttcaa cactttatta tcttcaaggc    390960
     atttcactga tgtcaatttt agaagaatca tatgcaaaca gcctctcgtg gtggttgaaa    391020
     gcccacagcc tcgccagtcc tgtggatatt tctggatgtt ctcagagtct ccattgtctg    391080
     tagagaagaa ttgtcccaat agttatttca cttttgtctt ccacaaagtt ggaaagatgg    391140
     ggatcactca ggggaatcga gatcaacaaa cagaaagcct gaacccacca tatacaagat    391200
     aggatgtatt tttttaggaa tatattaatt cccagaccct caggctcaca taccccatgt    391260
     ggaggggaac acacatccac tccttcactc aagccaagct ggccatgcag ctcagcttct    391320
     gcaggctcag gactgcaagc tctccagtgt gggagtggag ctaatgggag gtgaggccaa    391380
     gtctctgccc tcacagagtc tcatgatgaa gatctgatga aatatttcca cagtaacatt    391440
     tctttggata tataaatgta gaatatattt tgaaataatg aatgatgtat cttctgatgg    391500
     attcatgttg gggaggcaat agaggaaaaa taaaggtaat tttaaatttc tgctcccagc    391560
     tacagcactt gattttgatt tagtgcatga ctgtttgaaa ttttttgatg cagtcaactt    391620
     cagcagccca gacctggaag cccagccaat aaatgctcca gattgggaaa tttaaactga    391680
     tgggagctgt ttcatggagg aggaaaaatg ctgagccagt tgtgcgatca taactattga    391740
     ctggataata gaagcctgtg ccctttctgc cagtacctct ggtccgaaac ctgagctaat    391800
     caccctcacc aagttgagta aacattgaca cctatactaa gtacaccttc atggtggtgc    391860
     atgctcatgg ggccatctgg aaagaaagag gccttgtctc atcaggaaat aaggacatta    391920
     aacacccaat agaaatggtg ttaaactact agaggtgtgt tctcatcctt ctcatcctcc    391980
     attatgcatt gccctgagca cccaaaagat gactcattgg cacctaaagg aaatcagact    392040
     gcagaaaagg ctgagaagtg agcagtgcag gatgcacacc tgctatgagc tttgattcca    392100
     catctggagt ttccatagtt caaactccat tatactaaat agacaagaag catgcctgcg    392160
     actggggatt tggtaaaaca gatccagatt tattctgaaa aactaatgct tatggattat    392220
     tcttactctc cgaggccctg gtctacaatt ttaaaacatt tacatgaggg cacacactcc    392280
     aaatgagatt cccttgcaga ctttgtatga ccatgtgtca gaatggtcag aaaactgttc    392340
     agaaagtcac atacgagtgt gttttgtgtg ccaaaaatat ctttgagact gaacaaaatc    392400
     tttgcaacga agaggtacaa cacagagaac tgtgactctt ggaagattgg caagtggatt    392460
     cactcacatg cctgtggcca gtggcaacta taaattcctc ctgattgttc tagatattgt    392520
     ttcaagatgg gtagaagcat atctcaccaa gtcccaaaga gccacatgac tggctaaggc    392580
     ataattcaag gaaatcgttg ccaggtttgg actcccatgc actatacaca gtgacaacga    392640
     tctttctttt atttcagagt ttactcaaaa ggtaagtcaa gcactgcaaa tcgaatggaa    392700
     accacagtca tcatggagac cacagtcagc agtaaagaaa taaaagataa caccttgaag    392760
     aagacaatag ccaaactcag gcaggaaatt gacctgcgtt gggatagatt ttttcttatt    392820
     gccttgttct ggggcagaac ggtgccctga agtgggcttg ggaaaagtct ctttgaaatt    392880
     atatatagga ggcccttcca gacctcttgc cagtaacagc acctttagag ctggtaaaag    392940
     agcccagagt taaaccatat gtgcagattg gtgcagacct tgctaactgc acatcagctt    393000
     gctatttcca ggcctgttta ttccacagtg agcctcttca ccccttccat ccaagagaca    393060
     agatgccact acagtcttgt aaaagtcaaa aacaagatca gcagctgact gaaaggtgga    393120
     acagacccca tgatgtgcta ctgattaacc tttgttcagt taagttatcc aatgtaaagc    393180
     catgggtgca tcgctgttgg aaaaaacctg tcccacctca atttgacccc agataacaga    393240
     ctacctggtc acataagcct aaggaaaacc tgaagttgct gataaagagg agacaagaag    393300
     acagataggt aatacccttc tgttaatata aaattcttaa tgagttctat agttataagt    393360
     acattatttc tccttattaa aactaatctt ttttgctgaa tgggtggaag atggtggctt    393420
     ccctgcaaaa gaaaacatag ttggttctgt aaagagtttg cccatttctt ccactatagg    393480
     cctgccctgg cataatacaa gctcccaacg taagaattta tgattttcca tccattggag    393540
     aagccatctt ttccatctat tataaaaaca gaagacactt cattaatatc actcccacta    393600
     taggctatgc catatccagt gaggtaggat ggctgacagt ggtgcgaatc caagtaattg    393660
     gtcaagcact gctatgtgtg aagaaacacc acagcatact ctacactgtc actcatgata    393720
     tggaattgtt gcctccttag caatgtaact gagcccttaa tatgtggctg ggatgacaag    393780
     gcaatgccag cctgcagagt ggaatctgtt cttccctctg gggttagcta cagcctgtgg    393840
     cactcatggc tggccctact ttccttacaa ctggactaaa agaagcaact ggatatgacc    393900
     ttatatccca ggatgtaact gtccaaagtg cattctcttt atgtcaactg gaaaagtgtg    393960
     aaagacagac actactgaca taaacagaca tcctagttgt tctgcccact agccagctct    394020
     tccccccagg tggcagtcat agatttagaa ttgcaagtag aggccctggc taagcaaacc    394080
     accagtccct ttaacaacac ctaccatgcc atcatcattc ttactgaaga aacctcacag    394140
     atttgacaag tgacactatg aagctatatg accctaggta ttgcaactac agccaaaggt    394200
     aacacttgtg tattaatgaa cactgaatgt tgcatatata tacaagatta gtcccacaat    394260
     ataactcaag ctatgcaagc attagatacc catatttctg ctagagatgg atgccctctc    394320
     tcagaacccc atgacagcat ggtttagttg acttcctaac acatggaaga gtttcatata    394380
     tagtagagct ggtattctgt tcattgctat cttcaattgc tatggatttt attgctatct    394440
     tgcactttaa gtgagaatgt aatactgacc ttctcaaaaa ctcctaggtc cttgcaccat    394500
     aatgctccaa caaacaccta ctgtaattcc ggagactcca gaatatttcc aactccagtt    394560
     taatggattc ccttccaata catacaacct atgccctttc taaagaagta gccagaatga    394620
     ctatgacaca tgttttccat agaaacgaaa tggaatttga cagtggggaa tgatatacag    394680
     gcagcttaat ttcaaaatgc attttaaact tagtatttga ggttttaata ttatttttta    394740
     tactccctga aacctgaaat ttcacactta cattttaatt cagacttaat agcaacaaat    394800
     aaccagaaca catgaattgc ctgtaatctc tataccttgc attgtaagcc acatttcaag    394860
     tgtaacctta cttgtcagca cagtatgttt accacaggca taattgcttg tcctgactgt    394920
     ccagaaggca gggtggtatt aagtatgcgg cctctggagc tgggaagtct ggctttgtat    394980
     accgtctctg ccactgctaa ctggaggaca atgggccagt tacttcctgt accccagttt    395040
     tctcatatgt aaagtagggt tgttgtaaga atgaaattag ttaatattta gtacctaaca    395100
     actaataggc attacatggt agctattggt attgctatcg tcagagaaca cacaggagtc    395160
     cattgttatt tttaccctcc ccctccaaac aaaatgtgta agacactaga catggtagct    395220
     ctgtaacggg gttcgtcatg tattaaactg tgtacccaaa tgatatgtca aagtcctgac    395280
     aaccagcact tcagaacacg tccttatttg gaaatatggt agctggagat gttattagtg    395340
     aagatgaggt cttacttgag cagggcaggc cctcaatgtg ggaagatggg tgtctttcta    395400
     agtacaagga aatttggaca cagagacaga gacatacagt gtgccatatg acagcagagg    395460
     caaagatcag agtggtgcag cagtaaacca atgcatgtcc gggattacca gtcacctcca    395520
     caagtgagga aagggcaagg aagaattctc ctgagtctca gagggagtgc agcccagctc    395580
     acacttgatt ttggacttct agtctctaga actgtgagag aatacagtcc tgtcattttt    395640
     aagccaccca gtttgtagta tttcattttg gcagccctag gatattcata cagaattctt    395700
     cacccaaaag gaagcactct ttcacacaaa cagtcaaagt cctggatgac agacaacctg    395760
     gagactgcaa acctccaagg aatcaaagga agttctataa attctagggg ccccatcttt    395820
     caaaggctgc tcctcaagtc cacacccaca ctttggaagg agaatcctgc tcttggacat    395880
     gggatcctta gtgagccaac tgccaataat ccaacatcgt catgaattaa agatctctga    395940
     catttcctct tcaccaaggg gaattctgtc aagtagtaag taaaaccctt cttcagtaat    396000
     ctccacacca aacagttctg catggcttgg gacgcctcag gaaacttata atcatggcag    396060
     aagaaaaagg ggaagcaagg cagtcataca tggtggcaga agagaggtta aacttttctg    396120
     tctctcttgg gtgtaaagat ataatcttga ggcaaacagc agaatcaatt ttcttacgcc    396180
     ttgaaataga ctccaccttt ctccctttca ccataaatat tcccttcaca ttaatctaac    396240
     tttatgtttg catcgaacta tgtgcctttt tggaagttcc ggaagctaat ttgagacaga    396300
     tagacaaagt cgggagaccc tgatgcagaa ttccagaggt gacttcaatg ttgctagtta    396360
     acaacccagg cattgcccag atgatgccag cccaagatct aggtggactg ggatgccaga    396420
     gagccgccag aacaagacgc acagacctgg ttctcagccc aaatcttgca tgcctttctt    396480
     accaactttt ccttttttaa acccctgcct ttccttcaaa attcaagtgg ttgcttggga    396540
     tgggaatgcg gtcacttccc ctttaccatt atggttaata caataatctt tgtaccagat    396600
     ctctctcttg ttaattggat cctgcaggtg gcagattccg gaccagagtg aggttagaaa    396660
     ctgaatgcac atactgggtg gttcacagac acggtgttct gtgaagttta tgggtggatt    396720
     cctggggaag cctgcaagcc tgttagtgat tagcagaacc gcatccagct actgggatct    396780
     gcacgccagg tgccgtgagc ccctcaccat gttccctggt cccagctgcc cgcggatggt    396840
     tcagccttga caacgggctc ggtgttctgg gtggtgggag agcaaggggg ccccttgggc    396900
     agcgtgctac agggctgtga atccaggggc ccacccggtg ttccctgtgg agtcactgag    396960
     ggaatgaggg gcttctcagg gcacagagct gtgccgcctt tgtgcagtgg tggctgaata    397020
     cagtgacact gggcgcgtgg tgagaagcgg ggcaaggtca gttcactgga ccctccgccc    397080
     tgagcctcag atgagtcggg ggtccccaga caggcccggc ctctgccctg cggcggacac    397140
     tggagccttt gctgtggctg ccaccgagga gccttgacct caaagcagcg ggaacctctc    397200
     tacccacctc ggaaacctga aggcagcggc ggcctctccc agccaggacc tcgccggcat    397260
     ccgggtctcc aggcccaacc ctgtaccaca taaaggaagt ccccgctgag ctaccggcgg    397320
     gacacagcgc cgttgggtgg gcgggcgcgg cggggcggga agcacggagc agctgccaca    397380
     gagatgcgcg ggggctgtcg ggaagtgggc ggtccgggag gcgcggggct gatgggacgt    397440
     gggcggtccg gggacgcgcg ggggatggcg ggacgtcggc gatcggagat gccagggggc    397500
     agctgggacg tgggcggtct ggagaggcgc gggggcagct gagatgcagg tggtccggga    397560
     cgcgcggggg ctggcgggtt gtgggcggtc cagggatgcg agggggcagc tgggacgtgg    397620
     gagttccggg gacgcgtggg gtcgccagga agcggggtct aggacttaga tccgctttct    397680
     cttcaggcca tgcagccccg aagctccgaa tcctgggatg actcctgtcc atgttggaag    397740
     ggaccctgcc agtcctggca aggttcaaag gccttagggc aagaaatgtt agtagagtcc    397800
     tggaagacga tgtggggaac gggtagcggc caccggatgg tgatcgttct tctacaagac    397860
     cttgacatgg atggggagaa acagagaaga accttccaag tttgtcccac tggacatgcc    397920
     caccacactt taccagccct ttctagaagg cctgtgcgta acacatgaaa aagctccgct    397980
     cgacctttcc ctgacctttt aaaagaaaac atttgctgca tctaatccgc ctagatataa    398040
     ggaggttccc aaatgtatga cagagtcaga aaattacatg tcttgtaagt taccatgtgc    398100
     ttgtcttttg ttttccgttt tttttcgttt ttttgttttt tgtttttgag acggagtctc    398160
     actctgtcgc ccaggctgga gtgcagtggc aggatcttgg ctcactgcaa cctctgcttc    398220
     ctgagtttca acaattgtgc tgcctcagcc tcccgagtag cttggattac aagtgtgcac    398280
     caccacgcct ggctaatttt tgtagttttt agtagagatg gggttttgcc acgttggcca    398340
     ggctggtctt gaactcctga cctcaggtga gccacccacc ttggcctctc aaagtgctgg    398400
     gattacaggt gtgagccacc gtgcctggcc gtaagttacc atgtgctttt taaaaaaatc    398460
     atagcaaagg ggtgtcttct ggaaatgaca ttttgaaatg gtgttattag accacccctg    398520
     gaagggacac agtaaccaca cgtccacgtt cgttcagtgg gtgagaggac atggagggga    398580
     gacctgggca ggaagggaag agggttccat gccaggctgc tcatatttag aagacatttt    398640
     catatcattg tcattgtttt cttgtgtgcg ttttattcct cgctattgta tacatcattg    398700
     gaaattctaa gtattctttt gaaatatcta gtctttctag atgttctgaa gtgcctgatg    398760
     tatgttaaaa ttacaggtgg taaaataata aattttgtaa atatcttttt gttaaaattc    398820
     atatgcagtg ttttatttta ttttgatgtt ggtggggggt ggggaggatg gccaaatccc    398880
     tgcttgatca acacacattg cgtttgtgct ctggttcagg ggaggagaga ggaggagaaa    398940
     gtgcagactt ccaggcctct gtgcgcaccg ggaggagaga ttaatgatca tctcttctgt    399000
     ctgtgtgttt gttttattta tctatggata tcctgtgtat aaaggatgaa caagtcctat    399060
     ttataacatc taatcttttc aggtgttaag ttgccagtgt atgacggaag cagcgttatt    399120
     aaacgaacgc agcttgtaca tattgtgtta aaattcataa aaagccagtc ttctgaaaag    399180
     aacccttggg ccctccctcc gcagcctccg gcgccagctg caggaagacc tggccagggg    399240
     aaggcgggcg gcacaagctg ctgctggctg tggggaaggc ggtggagccc aggcctccac    399300
     aggctcccca caggctcccc acaggctcgc gcggctctgg tcgctgggct ccgcacgggc    399360
     cgcgtggggg cgcctgctgg gagcgaggag gcgccatcct ggctcgcctg ctccaggagg    399420
     acgctctggg cctgcgcaga tgcagttctc caggatgggc gcgggcgctg ggggccgcgc    399480
     tccttgggct gctgcgcctc cactgagccg cctgggtgtg aggacctcgc gccggcgcct    399540
     ccgggaagga cgcggagccg ccgcggccga gcccagggct gcagctgctg cccgacgccc    399600
     gcgagaaggc ccttccgcca ccgcccgcgg gttcgaactt ggagcagctg ctgccagctg    399660
     aggcgccagc ggcgtcggga gctcctcaga gtcagtgttt tgtgctggat tcggcatgtc    399720
     ctctgaaaac tcgggaattt gtcactgaaa tggtgacagg aggtgagaag tttttttaga    399780
     cgttgccttc gctgaccggg cgccgggctc aggcctggaa tcccagcact ttgggaggcc    399840
     gaggcgggcg gatcgcttga gctccggagg tggagaagct gccttcgtgg taactgtggt    399900
     cttaagttca gctgagaacg ataaatggct tttccttgaa ttggctgctt ttgtgatttc    399960
     tctcaggctc atctttcctc agttaaaaat ccagtggtag gtgtagctta gagacgggaa    400020
     atttttggtt ttgttttggc tacacgtaag tcttggaaat tattttcttt tacgttccaa    400080
     tgtgagcaaa tccagaagga tgtcagatta aacaccgaat ttaacaaatt cagccgggca    400140
     cggtggctca cgcctgtaat cccagcactt tgagaggccg aggcgggtgg atgatgaggt    400200
     caggagtttg agaacagcct gaccaacata gtgaaactcc gtctctacta aaaatacaaa    400260
     aattagccgg gcgtggttgt gcgcgcctgt aatcccagct actcaggagg ctgaggcagg    400320
     agaatcgccg agattgcgcc actgcactca gcctgggcga cagagcgaga cttcgtctca    400380
     aaaaaacaaa aaaaaaaatc aatcattgga atactgttgt tcattacaat taatgaacgt    400440
     ttgatacacg cataaacgca ctaaattcac gagtacatat aagtaaaata agccaaaaaa    400500
     aaaaaactac atcctgtatg atgacacttt tatacattct acagaataga aactgaagtg    400560
     acatgaaggt cgggagttga atggagaaaa ggtgagagaa aagagggagg aaggcgttgc    400620
     aagagaataa gagaaaatgt tgagggtaat tgatttgttt tctatcttaa taatggtgat    400680
     ggttacatca ataatttcca aattgtacat tttgtgtgtt aattagaatt ttatattatt    400740
     caagctatta caaataaata cctgtataaa tttcttaggg acggtgtaag ggagatggat    400800
     aaatcacata aaaagtcaga tatttctgaa tgtacaatga ataaccctta gctctatctc    400860
     tattttttgg taattctaaa atgcagcagt tttttttaat gtttttatta caagaagggt    400920
     ttcttcaaac cacaccaggt atgcgtaact ctgggataaa gttggctaag ggagctgtgg    400980
     gatccggtgg agaggagcac aggtcccgat cctggagccc attcttgtca catgtgagct    401040
     ccaggacaca tctcatagct ccctcatgct tctgggttta cattcacatc tgtaaatgga    401100
     ggaccaatgg gcatctgcct ctaacaatgt attttcgtgt gttaagataa tgtatgctaa    401160
     atgtttacca cagcacaatc ttttactaag aaattacatt ttttccaaat attatcattg    401220
     tcttcaagcc ccaccaccca ctagaaaact tcatctgctc tgtgccttgc cctgtcctca    401280
     gacatcctgc ccaagagact tctatatagt aggagacatg caaatagttc cctccctctg    401340
     ctgatgaaaa ccagcccagc cttgaccctg cagctctggg agaggagctc aagtgccagg    401400
     attcccaggt gttttcactt ggtgatcaga acttaacaca gaggactcac catgttgttt    401460
     gggctgagct gggctttcct tgttactatt ttaagaggtg attcatgaag aactacagat    401520
     attgtttgtg agtggatatt agagaaacag tggatatgtg tggcagttgc tgaccaggat    401580
     ttctctgtgt ttgcaggtgt gcagtatgag gtgcagctgg tagagtcttt tttttttttt    401640
     tttttcactt tttagcgaac atccatgggt tacaaaataa tgggttggct tttcttccaa    401700
     cactttacag acaccatcaa ttttcctctt gcttataagg ttttaaccag aagaatgctg    401760
     tcatcatctt tcctgttctt ttagaaggaa tgccccctca actcatctcc acttgtctgc    401820
     atgtatttct atttgtcttt ggttttcagc agttttaata agattcacct aaatgtgtgt    401880
     gtgtgtgtcg aggggtgtta tgctattgtt ctgtgttctc tgagatgcat ggattcaccg    401940
     tttactctgt ctccattttt gtgaaaacaa ttagaaaaaa agtcagtgtg agcctagaaa    402000
     caagcctccc tgaagtgggc actggaccac ctgggggcgc tcaggaccca ctgagcacaa    402060
     gagccagccc cagggcaggt gcagaggggt gttaaggtct ggtttcctgt cagccctgtg    402120
     gcttcctctc cataaaacag tttcctttgt ggcacatctc tggattcctt atcctttttt    402180
     tcctgtgaag tctgaagaag aaacatttgt cgtaacaaga gaaaaacttt ctcatatgca    402240
     ccaaaggcag agtcacctac agtcatttac tcctgtttct gaatgtcaat aaggtgtcaa    402300
     tgcttctgaa gttaatcagc taaatctata aaaggtgcgg tgtttaactc agcatggcag    402360
     cccagctcaa cagaactcca agggccagtg agcaagcagg caggataaag tgcatgctgg    402420
     gcattggggc agagggagtt agcatccagt gcaagagaag aaagcccccg tggtggtcat    402480
     tgtcaggact ccaagcccac agttccaatt gtaggtgata ctgggcaaag gaagagagac    402540
     cccaccaatg gttagtgtgg atttcgagtc tgatggttcc acactcacac tccaggtgaa    402600
     tatgaaaaca tttattaact ctatttttga ggtgtctgct gagagcagca caggcctctc    402660
     aagaaatttc aaactggaat ttcctcagta gacaggaaag gaggctggct cagggcttta    402720
     taatgatttg gtggtggggt tgggggtgcg tttctactca ggagaaggag attgtgtgat    402780
     ttaaacctca caggggcatc agataaggga gcttctgtga tttcttacta gatttaccac    402840
     atgcagggga taaggaggag gaagaataaa ccttaattcg tcagcaacga ggcaccaaaa    402900
     ataggacctg acgctttatt ctccctagca gcttaagaaa atgagtgaaa aagagagaga    402960
     agagtccact atgtgtgaaa agcaaacagg tctaaagaaa ataaaaattt tattactgta    403020
     agcacataat aaaaagaaag agaagaatga atgagacagg caggggtgct gaatcaatgt    403080
     cctgagtggg gccttttcat tatccacagt tatcagttaa ttctgaaggt ctcaggtcag    403140
     cttcctgctt caaaatatca caggccctta cagtatatgt gaaaatcctc ggtttgtcgt    403200
     tcttcactaa agtagcctta taattagatg tacttctgaa tttaatcttc agttgtgtta    403260
     aataaaaaaa aacacaatct acaatttagg aaagtgagag tttattttta tcaaggttta    403320
     cagccatccc atatgctgga aagcatagct tttggtaaag acgagagaaa ggcactccca    403380
     ggaagaaggg gttgggcaga agctttatgc tgaagggttt ggctaaacag acataatcaa    403440
     caggttacag gaggggctac tgatgttcat ggaggtggtc ctcacacatg catactgaac    403500
     aaacatgtct gtaacgtatg acccctgttc acttaccagt ggagacttag catttaaatt    403560
     cattacagtc aggccctatg tgccaatagc agaagcagaa acacaaaggc actcagggtg    403620
     cagcctctgt aaacggccag agccaggcca tggtgagtgg tctctgatca ggagaaaggt    403680
     cctgatatca ctctagtgtt caatcaaagc tggggttatg gcttgtggaa caggggtcag    403740
     ttcatcaggg gatgggctgc aattgtcttc atagtgcttg tctcagtgcc ggtgcttact    403800
     gagccgctag agaaaacgaa taacctattg gcagttagaa gacagtttac cttttaagtg    403860
     tgggagtgag tggcagaacc cttgcctgac atggctttag gtcttgttta taatttgaca    403920
     tcttattgcc acagagattc tgttctgtca ttcttttgat ctctatttta acattagtgt    403980
     tggtcattgt tgtgtctaaa ccgcaaaggg gagtgagtgt aagaggcgcg tctgatcttg    404040
     tcatggctgg gatctcagtt tttaggcttt tctggagtcc cttagatgaa cagatggact    404100
     atttagtcag ttggggcact taggatttta tttttgtttt acagttcata aatatgaacc    404160
     ctagtattga cattgtgcat attcactgct gtgttgtggt taaaattacc tggtaagatt    404220
     cccttcaaag ccgttcaaga gcaatatcct accctgatgt ttcttccagt aaataccatt    404280
     ccctgtttta atgtcattag aggctattga aaagatgtag gaaaatgata gcttaatctg    404340
     tcgatgactg gagtgggtaa gatacagtca tcactttcaa tgctttgtca tgcgtgcatg    404400
     taataggaag aggtgagctc tgggggaaaa tctctaaatg tatgggcctt ccagccacca    404460
     aatcataagg tgatgactga tgtctcactg agggacttga ctataatgct ctagtgctag    404520
     ggagagaaac tttggccaag gaagttcaag attttcttaa ggttttgaca agtttagttc    404580
     aagtatgaca attttcttca acattttcag aagattgtgg atgggaatga tgctgagtta    404640
     tataagtaat tctaatgcct tctgtgttta gataatatta taaagaagac tgtttggtat    404700
     aatgacccac tacaatatat atacttttag ttgttacacc atttcatttt cctttaggat    404760
     tttactttct aagttacatg aatcagttcc ctaaaatata ggcgtgcatc taaaatttat    404820
     tcttttgttg agaattttta cgtgttccag aagatatgag aaacttcttt gcgtgaagag    404880
     catcccaaag tcaggcactc ctgtaaaaca tgtacttaat ccttgtcttt ggcttattga    404940
     tgagctctgt tgaccacaat aaaacttctg tcatgcgggc tgacttgaca tcaggtagag    405000
     gaacagattc tgaatgtact actgaattga gtgcataacc tccagtttcg ttactgatgt    405060
     atgattcatc agagatgata ctagatcaag gatttcaatc ggaatctaat ctaaaagatg    405120
     tgaataaact ttcctaattt agatagtatt actgtgagca cactgctagt attcagccat    405180
     gtctcctgtc tgtcacactg aattcacgct aactttgatc taaaagttaa cacttgttct    405240
     aatgttcaaa tatgttattt ttattctaat gtctctagat tattatctgt ggctatttta    405300
     ggcaagagtg aaggaaagca aggttaactg actaaaaatg agaaaaagca caacctaatg    405360
     taatggaagt caggacatca gcatctccag gcttgtctat ttctagagtt catgcaacca    405420
     ggggatttct tttctcattt ttacacaaag aaacacccaa attgtccatt tcatctccca    405480
     ttgactctta tcatctgtct ctgtcagttt gcttagaatt ttggcctcca gcttaatcca    405540
     agttgctgca aaaacatgat ttcattcatt tttacagctg catggtattc cactgtgtat    405600
     aagtaccacg ttttcttttt tcagtctgta actgatcggc atttagtttt attgtatgtc    405660
     tttggtactg tgaatagcac agtgatacac acgtgagttg atgtgtcttt ttgttccaat    405720
     gattggtttt ctcttggaca tatacccagt aatgagattg ctgaggggaa tggtagctct    405780
     gttttaaatt ctttgagaaa tctccagatt gatttcatca gttcttgggc caaattatat    405840
     tttcaccaaa agtgtgtaag tggtccgtta tctctatagc cttgcctgca tctgttaatt    405900
     tctgatcttt cagtgatagc aattctgaca tatgtgagaa agtatatcat ttggttttga    405960
     ttcgcactta tctgatgact gaatatgctg agcattttta taggttagtt ggccacttct    406020
     atgtttgtat ttgagaagtt tctgtgcatg tcctttctcc atttttaatg gggttatttg    406080
     gtttatgctg gtgattaagt tccctatgaa ttaccacctt acacacacta agataatcag    406140
     cattcaaaaa agaaaaatga atagaaaaac ataaccactt gtaggtatag acatactgaa    406200
     tttggaatgg tcatggcttg catgttggta ttgaaatggg acagcaactt tggaaaatgg    406260
     tcttcacaga aaacctcata aatgtagcta gtgtcaccac tcacagaagt agatggagaa    406320
     tacaactcaa gtgtccatcg actgccagag ggatgaagac gccgcggtgc gtctgtaacc    406380
     cacagggatg aagacgccgc ggtgcgtctg taacccacag ggatgaagac gctgtggtgc    406440
     tttaaaaagg agtgaagcac tgacataggc tgcaactcgg atgggccttg taaacattgg    406500
     gtgagtgaac tgagggagac acggaagtct acatcctgaa ttgtcctatt gacatgaagt    406560
     atgcagagga ggaaaatccc tagagacaga tcacaggggg tgagtggagg ggaaatggag    406620
     agtgacgctt aatggagctg aggttttctt ttctgtgatg aaaatgttcc caaaccaaat    406680
     actggtgata tttgcacaac attgcaaatg tgtaaagtat cactgacccg tacacttaat    406740
     cgtggctaaa attgtaaatt ttatgtcatg tgtatctcaa cacaatagaa atgagcacca    406800
     cattttgttt agtgactcaa caacaataac atttaggttt aaattctgga ttccccaaca    406860
     gaaccagtgc ttcctgctgg agctggatcc atcagccccc agggaaggga ctggagtggg    406920
     tcaggtgcac aggtcatgaa gggagcacaa attctaaccc actcctcaag agtccagtca    406980
     ccacctccag atctatgtcc aaaaacagct cttcgtatgg ctgagtgaca ttagcaacaa    407040
     gcacacagcc atgtttgttt ttttgttttg ttttggtgtg tgtgtgtgtg tttttgatag    407100
     agtcttgtgt cacccaggct ggagtgcagt ggggcaatca atcttggctc actacgagct    407160
     ccacctcctg ggttcaagtg attctccagc ctcagcctcc aagtagcttg gactgcaggc    407220
     actcaccacc acacctggct aatttttgta tttttagtag agacagggtt tcactatgtt    407280
     ggccaggctg gtcttgaact cctgaccttc tgatccaccc gactcggcct ccaaaagtgc    407340
     tgggattaca ggtataagcc actgtgccca gccacaaaca agtattttta agcaaaagac    407400
     acagtgaaga gacttcagta tgagcccaca cacaaacctt cctgtgggag tttacagaac    407460
     agcagtgggt gctgaggaca gaagccagca cccaggaacc agcagggaaa cccagggggc    407520
     atttggcacc gcctgaaggc tcaggaccat tgtggggctc agtggtcagg caggctcaag    407580
     gttcagcctc agagcaggtg tagcaggcgg ggaaaggctc tgaagacgga gtttagtgtc    407640
     accttctcat ttccactact aaacaccctc cagcacatct attctaatgt gtatgggtgt    407700
     tcatgtgttt agagaatatt atttatgtta tgaatctata gccatcttgt gggtgcatca    407760
     agttaaccac ttcaacctat gtggaccctg ttcattagga ataagtccct gtatttgagg    407820
     acctcacaaa ttaataatta tgtagaatca ctttcttttt cagtctcctt tccttcctct    407880
     ttctttcttt ctcactcaca cactgacaaa cacacggggt gccataacat taattacctg    407940
     atgtattaaa gaatattgat tcatttgcaa cttgaccagt ttagctgttg ttcatgttgt    408000
     tgtaagatca ggaacatgtt tctcagctgt gtactcctct aagctgagca gcagctttat    408060
     ttgaaataca cagaaactga aaacaatcca aatattaatc agcaccttca taagtaaaca    408120
     aattgtggaa aagtcgttta ttgggatagt acccactact acaatcagta aatgttggat    408180
     acgatcaaca gcatggttca attcacaagt acatatgatg agtatagtga gccaaagcac    408240
     atacatatga ttccgtttta taaactgtac aaagtgaata ctcattggaa attacataac    408300
     aaagatcact aactgacttc tccatagtaa gagaagcgaa ggtataggag ggagaaattg    408360
     tgagagacaa agagaaaatt gagaggcgaa ttgatttgtt ttctctgtga atggtcatta    408420
     ggtcaatgtt tgtcaaatgg tgaacatatt gtgtgaagat tatatgtctg tattacttca    408480
     ttaaagctat tataaataaa agtctaatgt ggtagaaaaa gatgaagaga gaaataaaaa    408540
     taatacaaga aaagtcatga actcctgaat gaattaaccc ttagtttttc tctattactt    408600
     ataaaaacac caagatacag ccaaataata tcacgatatc attataagaa gagtgttttg    408660
     taaacctcac tgggaattta tagctctttc ctagagttaa ttttgggaac agttggatcc    408720
     aattgtgaga aatgcaggct ggacactgag actggctctt atgagatgtg agctcttgtc    408780
     tatgtcacat ggtccttcca tacttggggg tttacattca catctgtaaa tgaaggaaac    408840
     attgactctc aaagaacata tttcatgtgc atgtaaaagt atgaatgcta gtgagaatta    408900
     attacttatg aagtataatc acccacatcc actcttggac acagcccact ctgaggcatc    408960
     tgttacagaa ctcattatat agtaggagac atgcaaatag ggtcctccct ctgctgatga    409020
     aaaccagccc agccctgacc ctgcagctct gggagaggag ccccagccct gagattccca    409080
     ggtgtttcca ttcggtgatc agcactgaac acagagaacg caccatggag tttggactga    409140
     gctgggtttt ccttgttgct attttaaaag gtgattcatg gataaataga gatgttgagt    409200
     gtgagtgaac atgagtgaga gaaacagtgg atatgtgtgg cagtgtctga ccagggtgtc    409260
     tctgtgtttg caggtgtcca gtgtgaagtg cagctggtgg agtctggggg agtcgtggta    409320
     cagcctgggg ggtccctgag actctcctgt gcagcctctg gattcacctt tgatgattat    409380
     accatgcact gggtccgtca agctccgggg aagggtctgg agtgggtctc tcttattagt    409440
     tgggatggtg gtagcacata ctatgcagac tctgtgaagg gccgattcac catctccaga    409500
     gacaacagca aaaactccct gtatctgcaa atgaacagtc tgagaactga ggacaccgcc    409560
     ttgtattact gtgcaaaaga tacacagtga ggggaagtca gcgagagccc agacaaaaac    409620
     cttgctgcag gaagacagga ggggcctggg ctgcagaggc cactcaagac acactgagca    409680
     tagggttaac tctgggacaa gttgctcagg aaggttaaga gctggtttcc tttcagagtc    409740
     ttcacaattt ctccatctaa cagtttcccc aggaaccctg tctagatctg tgatctggat    409800
     ctgctgaaac tgcctgtgtc accttcctca cctgtgactt tgggggagct gattgtggac    409860
     actccagtgt gtgggatttc ttggtgacag caattgtgtc ttctgtctag gcatgtctag    409920
     ggctggccat caggaagggc aggctggaat ttttggaaag aggcgcacct gccatccacc    409980
     aggaaatttt gttgtctttt gttctgctag aattaaatca gacacaccaa ggttaactag    410040
     cactatcttc ctagcttgag aaacttgatg gcaggcttga ataacacctg tatgaagcca    410100
     tcagagcaac aactagatta atgtctgcta gaattaaatc aggcacacca aggttaacta    410160
     gcactatctt cctagctcga gaaacttgat agcaggcttg aataacactt gtatgaaacc    410220
     atcagagcaa cacctagaat agtgtctgat ttaataattg agactatggt ctagccaagg    410280
     agacacataa aacatgattt ccatggttgg ttcatatttt atatttatta atagaatctg    410340
     gctggtatta taaacagctt tgccaaaata tatgtgttag tgttggtctt cggagaagca    410400
     aactctgtga ctggattagc ttcgtgtaca gtttccttcg tgtacagttt ccctgtccac    410460
     tcttctgaga ggaaatgcag aggtggtgga aggggtctgg ggtagctgaa tgcatgtgag    410520
     ggaagtcggt ccctgagtga aggagaaagg gaggaggact gggtggaact ttcctaaact    410580
     tctgtgttgt tctatgaagg tccagcaagg tcactgaatc agagtcgtgt cacaggttcc    410640
     catcagggac ccagtgactc ccagcagtgg ctctgctcag atcagcgcag agcttgttcg    410700
     tctcctgaga gtggagcaca ggacgtggct ccaacaccag ccatggcatg gaagacagag    410760
     agcagccctg ggtgcctggg tcaggtgcat tgtgctccct gcaggtggag ggagggaagt    410820
     gctgactcag ggcccagaga ctgtgggttc tatacagaac atacactttt acttcatttc    410880
     tgtggatgac atagaaacaa acatgcagtc tgtaaacaat ggtgattcct acatttgccc    410940
     caattgcttt attccttaat tctgcagaat gtcctggcac agaattgcct ttctcatgtg    411000
     gaattgtgac gtgtgttgag gatgtgtgat ccctactttc attgttttcc tccatgtaca    411060
     gagctcctaa atagagtaca gctgaccctc cttctacact gttgctctcc tccccacaga    411120
     aagagcaaat aattacctga ggctgaatct gaggtgggat ctgtcctctg aacctcagag    411180
     cctgcagaga cccccagctg cagattcatg gagtcaggtg tttgtacatg tgggaacctt    411240
     gagctgttct tttgtcagtg aacactcctt aaaactaatt gtgggttcag aattaggaca    411300
     cccattgatc tatcacacct cagctcattc tgccactcag aatctccaga aattcaggaa    411360
     atggttgaat gtacattttt gtgacaaatt tttctcattt cacttagttg tgagtttggt    411420
     tagcagaaag tgctaccatt tatgggtccc aaactgatga agctcatttg cttcaacaga    411480
     agttaggagg ctcctaaaac ttctcatcag cccttctctc tgcacttcat gtgaaattca    411540
     gttttacctg gaattctggt gtgttggttt agccagagtt ccccatcctc catgtctgat    411600
     tttcctgggt atctgatcag tttccttgcc cttcaccata cctcggtgac gtctaatcac    411660
     cctggcccaa catcaagaat tctggcaggt tggtttaagc aagatttgct gtgcccctga    411720
     tgtttcctct cagtaatttt ccatcttccg gaccccaccc tgctgcttgg ctttaaatcc    411780
     ccatgtttcc attctgcatt tggagttcag ctgaatctct ctcctcactg caaaacacca    411840
     ttgccctgat cccgacacct accatgagga ccctggataa agtcttcctt actgtgctgg    411900
     aacaagtgtc tttacttgat attttttctt taataaatct acctttagtg cttcctaaat    411960
     tgctcaagga acctcgaaaa ataagataag tgcgattatc cattttactg tgtgtgtatg    412020
     tgtgaatttc aatttccatg tgtggtgtcc agaagtcctg agcacaaaca gttttgcttc    412080
     tctgtccaat tctttgcttc tgaaagccag gagtcatccc aacattggta aattggcgat    412140
     ttgtttttac tatttagtga ctgacatatt tcacttaacc tgaagcttca tgaacacatt    412200
     gatgaaaagt agatagaaga cttgtttgga aatgccagat ttagggtaga ctttaccacc    412260
     cacgtgtgtc tggagatctc cttaggattc caagaacagg gtggtttttt tccaccacag    412320
     gacaaggatg agaacctcca agctgtgatg ataaaggaca ggcagggcca tgtgtggaca    412380
     aggatgggct gctgccacgt ccagagtgtt ctctcctagc aacgccgcca tctccttcct    412440
     cacactgtgg tatttgtcag atgggaaaca tgcctagaat ttatctttga catatggatg    412500
     gaagcccagg ctagaggcat ttgacagctt atttctgaac ctacttcttt aaggaatgcc    412560
     cagatccctt gctctgcttc ctggttctta acccctttag ctcccttccg ctcctgcacc    412620
     catgcactgc ctctttacgg ccacttcccc acacccttca ctgtagcacc attttcttgg    412680
     gctccttgga aatatcacag ctttggttta actcacagat caggcacact catgggcccc    412740
     tccagaggtt ctcgatttcc ctccctcagg ctcctccctg gggtcctaac ttggccatct    412800
     ctatcctctc ttctcagcct ggccatccac aatccatccc atctggactt gtgtatagca    412860
     ctgggttaac tcagcaggta tgaattgttc aaaccctgca cattcctaaa aaataactaa    412920
     aaaaaaaaaa aaacttttgt tcatttattg ctgttaccta ctgtgatgag atctccgagt    412980
     ccataaaata ttgcacctga taacagtgtc tgtggatgtc tgtagccttg ggacatgcgg    413040
     tacaagttta atcagtgatt tatagtgaat gtctactttt gttttgctgg agagggctgt    413100
     cgtctgagta ttgtggtcag ttccccaggt gttccatagt ctatgattca ttccccaaac    413160
     acagccctgg acaagagagc tcagcggcac tccccttgtt gtcaacactt cacacaagcc    413220
     atcttacctc acagctgaac aattacacat gtgtagaagg ctccactgtg agaggacacc    413280
     tgagagcttg tgcctggatt ctccagggct tcactccttg tgccttttgt ctttacttat    413340
     tttaaattgt atcatttttc tgtaataaac tgttcctgtg aatagaacag ttatgagtcc    413400
     tatgttttta ctgatttttc agcctacagg tggcagatga gaactctcaa cacagttgtg    413460
     ttagaagaag gatttcctag agagaccctg actcaatgat gatacatggc tgaagcattg    413520
     catggaaaac gtaagtgttc aggtatggaa tggcaaaatt tgatacctgg ggagtgacag    413580
     aatactacag tctattacag cagctgagct gaaatcggtt tctggaggta aatgggattt    413640
     agaaattata aatccaactc ccaaggagct ggctcaatca atacataatg aaatgtgaaa    413700
     tgattagaaa tagcctacat atgcaatttc catctgtgag aactaagtga aaatccagga    413760
     gagagttggc agggagaggg atactgcacc aacctcagat tttgaccatt ttcatttagg    413820
     gctgatgggc tcatgaccac tagagttagc aattacactg tgataaaaga taaaagcttc    413880
     acaaactcta cccacccaga agggggctca ggtggtgaaa tagggagggt aaggcatgaa    413940
     acgactgaaa tatgggagga tagtgagatg ggttctctct ttttgagttt tatcttttgc    414000
     ttctttacta atttttgtta atctggattt ggagaatgac tagtgtaaaa gtggattctg    414060
     ttttgagtcc cttagaatgg aggagaatgt gtgggccaat gttgtgccca gagactacta    414120
     cagaacaata gtagatgaat gtacatgacc caaacacaaa gctttgtggt tgctgatggg    414180
     agagctgcac tgccctggcc tactggataa cagcttctcc aaagtgtatt taccctgaca    414240
     atggagattg cctttctctt ttaagtgaaa aaaatggaac actacaataa agtagtggta    414300
     atgctgtgtg tgtatgtata agacattctg gagtgggttt gtgataattg ggatatgtat    414360
     cacttatcct tttatttgta taaatttatg gggtgaaagt gccgttttgt tacatggata    414420
     tattgttgag ggataaaatc tgggctttga gtatagccat catctggatg gtgtatattg    414480
     tacccattaa gtaacttctc atcccacatg ccactcccac cctcccaacc ttccaagtct    414540
     ccaatatttc ttattccaca ctctgtatcc atgtgtacat attatttagc tctcacttat    414600
     atatgacatg tggtattcaa ctttattctt ctgagttctt taacttaaga tagtggccta    414660
     cagtttcatc catgctactg gaaaatacat aatttcattc cttgtctatt attgagtagt    414720
     aattaaatgt gtatatgtac atacacatgt atagtgtgtg tatgtgtggg gatgtgtgtg    414780
     tacatatatg tatatgtatg aacatatacg actgtttcct ttatctaatc atttactaat    414840
     agaaacttag tttggtacca tgtatttgct attgtgaata gagcagcaat aaacatataa    414900
     atgcaggcat ctatctgata taataattta tcttcccttg ggtagatacc cagtagtggg    414960
     actgctgaat gaaatattag ttccaatatt aattatttgc aaaatctcca tactgttttc    415020
     catagagctt atagtaattt acatttccac tagcagtgta taagcactcc tgcttctctg    415080
     catccttact agcatctgtt ttttgttttg ttccattttt ctttttgact gttttaatag    415140
     tagcttttct gactggtata agatggtatc tcactgtgtc tttaaattgc atttttctga    415200
     tgattaatgt cattgattat tttttcatat gctggttgat gatttttttg tcttttgaaa    415260
     aataaacatt gtagtaattt gctcattttt aatgtggtta tttgtgggtt tttgtgttgc    415320
     ttttgtttct tgtagtttgt aaatattagc tctttgtcag atacagagtt taaaaatagt    415380
     ttatcccatt ctgtacgctt tcggttaagt tttttgatta tttgttttgc tgttcagatg    415440
     ctttgcttct tatttgtatt aagtccaaat ggtctatttc ggtttttatt gtttactttt    415500
     aaggttgtag tcatgaattc tttgcctagg acaatctcca gaacaatatt tcctagaata    415560
     gcatctgcaa ctttcagact ctcaggtctc ccagttaagt cttttatcca tattggctta    415620
     attttggata tagtgagaga tacgggtcca gttttattct gctgcaaatg gctgttcagt    415680
     ttttcctgca caatttatat aacaaggtga cctgttccca gtgtatgctt cttgtctagt    415740
     ttttcacagt cagtttggct gcaggcattt gactttattt atgaaatgtc tattctgttc    415800
     cactcatcta tggctttctg ctagattgtt acttgattgt tacttgtgta taataatgct    415860
     actggttttt gtatgctttt ttcatttatt ctaaaacttt actgaattaa ctcatcaatt    415920
     ctaggagtat tttgaaagaa tattagtttt tttaagtaca aaatcatatt atcagcaaac    415980
     acaaagagtt tgacttcctc ttttccaatt tgagtgcctt tatttctttc tcttgcctaa    416040
     tttctctggc taggatttca agttccatat tgattaagag tggtgaaagt gggcatcctt    416100
     gacgtgttct gagtcttagg aggaatactt tcaacatatt cctattcagt ataatgttgt    416160
     ttcggagttt gaaggtgatt tatggagaat gagagatgtt gagtgcgagt ggacatgagt    416220
     gagagaaaca gtagatatgt gtggccgttt ctgaccaggg tgtctctgtg tttgcaggcg    416280
     tccagcgtga ggcgcagctg gtggagtctg ggggaggctt ggtacagcct gggtgggtcc    416340
     ccgagactct catttgcagc ttctagattc accttcagtg acttctgaat gcactggatc    416400
     cgccaggctt ctgggaaagg gctggagtgg gttggccgta ttagaaccaa acgtaacagt    416460
     tacacgacag aatgcgctgc atctgtgaaa ggcaggttca ccatctcaag agatgattca    416520
     aagaacacac tgtatctgca agtgaatacc ctgaaaaccg agtacacggc catctattac    416580
     tgtactagag acagtgaggg ggaggttaac gtaggcccat acacaaatct ccctgcaggg    416640
     gcgcgcaggg ccaactgggg gcgctcggga cccactgagg atgggacagg tcccaggggc    416700
     gggtgcaggg ggaggtttcc tttctcagct gcaggaggcg ggtttgtttt tgcaggaata    416760
     tggagtctta tgaggttttg atattttact atggttattt atcatgattt tttaaaattg    416820
     ggatttgtgt tttagtaatt tttaaattta tatgtagggg tatttttaaa aattaagttt    416880
     tagggtacat gtgcacaacg tgcaggtttg ttacatatgt atacatgtgc catgttggtg    416940
     tgctgcaccc attaactcgt cacttaacat taggtatatc tcctaatgct atccctcccc    417000
     cctcccccca cctcacaaca ggccccggtg tgtgttcccc ttcctgtgtc catgtgttct    417060
     cattgttcaa ttcccaccta tgagtgagag catgcggtgt ttagtttttt gtgattgcaa    417120
     tagtttgctg agaatgatgg tttccagctt catccatgtc cctacaaagg acatgaactt    417180
     atcatttttt atggctgcat agtattccat ggtgtatatg tgccacattt tcttagtcca    417240
     gtctatcatt gttggacatt tgggttggtt ccaagttttt gctattgtga atagtgccac    417300
     aataaacata tgtgtgcatg tgtctttata gcagcatgat ttataatcct ttgggtatat    417360
     acccagtaat gggattgctg ggtcaaatgg ttatttctag ttcaagatcc ctgtggaatc    417420
     gccacactga cttccacaat ggttgaacta gtttatagtc ccaccaacag tgtaaaagtg    417480
     ttccctattt ctccacatcc tctccagcac ctgttgtttc ctgacctttt aatgattgcc    417540
     attctaactg gtgtgagatg gtatctcatt gtggttttga tttgcatttc tctgtaccct    417600
     aaaacttaaa gaataaaaaa aatccttcaa aaataattct tcctaataat atgcacttat    417660
     tctcctaggt tgtattaaca tctgttgata tcttcaacta catagctatg gcgacattaa    417720
     tttacatctg tagacatatg tgtaaataca caaacttatg catacatgtc tagtctttta    417780
     tatttaatat aataaaatca ttataaaata tgtcctaatg aatgaaactt aatgattaac    417840
     taaatataaa ttatagtaat ccattattca ttgcaatgat tctctatagt ttacataaat    417900
     tggtatctat ttgtaagctt aaatatagtg tattggtcat tttaaaatgg ccaagaacaa    417960
     atttcaaatg tccctgtcac acaaacacac acaataagga tttgaggatt tgagttgata    418020
     tatatgtcaa ttagctagat tcagttattc catattgcat tcataaatca taacatagct    418080
     ttgcactcta taaatacata gtcaaaattt ctcaattttc aatgaaattt taattataca    418140
     ttttttaatc cgtcctaggt catgattttt ttctccctgt caggatatga ttagattgtc    418200
     ctgagaaact cattcagcct cctgcctcct gaaggcttca aaggcttcag gaagtaagct    418260
     cctggatggg cagaagcagg caaatctttc atgtgcacag gacctggagc atctctcttt    418320
     ggattaagcc ccctcctcag gattacaggg ctcttcattt ttctcaacag gctgttgtac    418380
     cagataagca caaaaactta atttcattat gctttgcttt ttttaaaaaa aaaaaatgaa    418440
     ggtaataatt ttaacaataa acatattaca acctgctaca catgagaccc ttcttgtgct    418500
     tcgacctttc ttctcaggag tttatatgta ttacatatat tcagtttttt tctgagttgg    418560
     aatgcttatt acagattatt ccccttatta cagattactc ttttaattta tctcttagaa    418620
     tgatttttcg agagcccctg cctgcccgcg cctggtggaa tggagcggcg cctgggctga    418680
     gccaggcgcg caggggcctc cgcacgtgcc gcgccggtag cagacgccaa gcgggcggac    418740
     agtgagcgtg agaggccggg tcggagtggc cgccggagca gtgccgggga tggaagaaca    418800
     gcccatcttc accaccggag cacaggtctt ccagattgac cccaacacca aaaagcactg    418860
     gatgcctgcg agcaacaggc ggtcaccgtt tcttacttct atgatgtcac aaggaacatc    418920
     tatgggatcg tcagtgtgga cggagccaag gtgaccataa acagcacaat cacaccgaat    418980
     atgaccttca ccaatgtgtc acagacgtct gggcagtggg ccgacagcag agccaacacg    419040
     gtgtttggtt tggggttttc ctctgagcag cagctgacaa agtttgcaga gaaattccag    419100
     gaggtggagg aagcggccaa gacagccaaa gacaagaccc aggagaacat ggagccctcg    419160
     agtaataatc ccgagaatcc gggcatggag ccccatcttc tactccggca tccagtgtca    419220
     acgggacgga cgatgaaagg cctctcaggc cgctgcagct gacacgcacc tgcagtctgg    419280
     gaaccacaag ctgaaggcgg cctcgacgca gagagctgcc cacgggaaga ggtgggagat    419340
     ggagctgcag atccggcggg agagcgacgc ccggctgccc acggcgctgc aggagtcggt    419400
     ggccagcgtg gagcagagga agaggccgtg agatccacag agaggagaag aacacgcagc    419460
     tgaagaggaa gatcgaggag ctggaggcgg agctccgaga aaacgagaaa gagtggaagt    419520
     ccccaaaagc aaagtaaaac tactcctcag ctcaggtgag tgcaactgtg tctctgagaa    419580
     gatggaggcg gcagagagag gatcaaaacc tggaagacaa agtgcgttcc ctgaagagag    419640
     acactgagga gagcaaatcc agacagcgcc acctgggggt ggaggtgaag agtttcccgg    419700
     aggtgctgga ccggaagatc cacaacctgc ctgacttccg cccaggcctt gcatactgtg    419760
     caccaataac tagggctggc cgaggcccag gcccctcctg tgagtcccaa gcgtgtgtgc    419820
     gagaccagat ggcgctagga cgttccctgt gtgcgttgct tctgtaaatg caggcgcagt    419880
     ttcttgtatt tccaaaccaa ctgtgccgtc cactcacccc ttcccagaat agaaatctct    419940
     tgtccaggca cagtgtctca cgcctgtaat cccagcactt tgggaggtcg aagcgcggga    420000
     ggatcacgag gtcaggagac cgagagcatc ttggccaaca tggtgaaacc ctccctgtct    420060
     gtactaaaat acaaaaaatt agccaggctt ggtggcaagc acctgtagtc ccacctactt    420120
     gggaggctgg tgcaggggaa tcgcttgaac caggagagag agtttgctgt gagccgagat    420180
     cgcaccactg tcctccagcc tgggcaacag agcgagactc catctcaaaa aaaaaaaaaa    420240
     aagaaaaaga aaaatttcct ctcgcttctc tggccttgtg aggttgtgga caactggaag    420300
     attttgactc aggaatccag aactaggtct accttcaaca tttacacagt caggccaggg    420360
     atgtttatat ttttcataag ggctgttgaa accatatgaa ctgaaaaaaa gcactttcta    420420
     atccaaatat tgatattctt tacaccaggt catcgggctc cttttatcga atagcattca    420480
     gggtatttga atgtccatca ggcgccaggc ccagggggca cagggagaac aacattcctc    420540
     tccgtcaata acgagaggct ttaaaacaac tgtttagtgg agacttatcg agatggcaaa    420600
     caggtttctg gtgggtacat tttctggcct ggggatcacc tgcatccacg atattgccct    420660
     ctgcccccca gtttgtatgg ttgcgacaat gttccttttc ttggttttaa tttctgagcg    420720
     gatgattgtg gtgcgggaac agcacacagt gagggtgcct agcacaatgc ctggtgcaaa    420780
     gtaggttttt tataaacgtt tgtgcggctc acacctgtaa tcccagcaat ttgggaggct    420840
     gaagcaggcg gatcacctgg ggtcgggagt tcgagaccag cctgaccaac atggagaaac    420900
     ctcttcttta ctaaaaatat gaaaaaaaaa atagccggga gtggtgcatg cctttaatct    420960
     cagctactcg ggaggctgag gcaggagaat cacttgatac cgggaggcag aggttgcagt    421020
     gagtggagat cgtgccattg cactccagcc tgggcaacaa aagtgaaatt ccgtctcaaa    421080
     aaaatttttt tctaaagccc cactcaaaac cagggtatta ttagcagtgt aatagttgga    421140
     ccacagttga aaatacattc aattcagttc atgtgctctc aaatatcctt tttcgtttta    421200
     aagttgttct cagttgtaat attactcaaa atattagtaa tttatactaa tgacacaggt    421260
     tacaatattg tatacataat ttagtgacat ttgattggaa aaaatacatg ttccccatgt    421320
     cttgagtatt ttttccttct ctataaaatg tatgcttata attatttaag tttcagatgc    421380
     tagcattatc tttttgatat ctgggattta attttagtag gtatactgga atgcctttta    421440
     ttaattcata taataatgtt catattttag ataggatttt aatattaatt ttactattta    421500
     ctgaatttca aacttttcta ctttttttgt ttttatgaga taaaattcac acataagaaa    421560
     aaatgcatag atctgaaatg tgtcactaga gagtttctgg caaatgtgaa tacctttgtc    421620
     cccagcacct aaggtagcct gaagagcaag tctctcccca aaacatgcgt ctttttctat    421680
     gcctgtggtc aaatcctgca tggggaaagg tttcgatttc tgacactata gatgtatttt    421740
     attctgcttt gaactttata taaatggaat caaacattat agactttttt tggtaagggg    421800
     ctacttttct atttttgagg ttaattcatg ctagctaatg tataaaatta gatcaacata    421860
     ttgtcgttta ttcaattcat agacagactg tgatatgaac caccaaaatt ttcatgtaca    421920
     tggaaagaga gagagagaaa gagaggaagc agagatattt tatatctgag tcagtccatt    421980
     aagtaaataa atgacaatat tttcatttat ttttatgtca gtggacttta aatttgtgtc    422040
     cagtttatga atattatatg cagagctgtt acaaatagct tagtgtaagt tttctgatgt    422100
     tctattttta ttgagaaaat atgaagtatg tatttcttta ttctaagagt aagtttaatt    422160
     tttttcagtg gtattgaggc ataattgaaa tatttttata atatatatgt ttaaggtgca    422220
     ccaattgatg ttttgatatt gtattagtcc attctaacac tgctataaag aaatgcctga    422280
     ggctgaggtg ggcggatcat gaggtcagga gatcgagacc atcctggcta acacggtgaa    422340
     accccgtctc tactaaaaat acaaaaagtt agctgggcgt ggtggcaggt gcctgtagtc    422400
     ccagtactcc ggaggctgag gcaggagaat ggcgtgaacc tgggaggtgg agcttgcagt    422460
     gagctgagat tgcaccactg cactccactc cagcctgggc gacagagcga aactctgtca    422520
     aaaaaaaaaa aaaaaaaaaa aaaagcttga gactgagtaa tttggaaaaa aaaaaagatg    422580
     taagatgttt aattgacttg tgagaactca actcatgcat tgtcatgaaa acagcaccag    422640
     gagtcagttc taaaccattc atgaaggacc cacgccatga cccagtcacc tccaaccagg    422700
     tcacacttcc acaattgagg attataatac gacgtgagat ttggggtagg acacagatcc    422760
     aaagcatatc agatatacat tgtaaaatgc tcatcatagt caaggtaatt tgcatatcca    422820
     tcttctcaca gagctaccgt ttaatttttt ttaaatgtag agcttgtgtg tgtatgtgtg    422880
     tctgataaca acacctaaga cctactctta gcaaaaatca cttttacaat atagtattaa    422940
     atacaggaac attgctgtgc attagatctc cagaaatgat tcagcttgca caactgaaac    423000
     tctgtgccct ttgaccgata tcacccaatt tccctctcct cccaggtcgt gggacccagt    423060
     actctactcg ctgctatcaa gaacttggat attttagatc ctacatgcag atgacatcgt    423120
     gaagcatttg tctttcggca tctggcttat tccacttagc accatgtcct ctagctccat    423180
     ccgtgttgtt gcaaatgtca gaatttcctt tttttttttg aaagccaaat gaaattcagt    423240
     ttatatatat acattttctt tatacagtca tcaatctatg gtcattaaat tctttcacaa    423300
     atctacacta ttataaataa tcttgcaatt aacatgtatt tgacatcata attttatttc    423360
     ctttgacgat ataacaagaa gtgggatcac caggtcatat gatagcttta tttttcaatt    423420
     tattgactaa ccaatcttac catacgatat aaggataccc tcttcaccac attcttgcca    423480
     acatttgtta tcttttgtct tgctgataat aaccatttta agtggtgtga ggtgatatct    423540
     cattgggctt tttatttgaa ttcccctgat aattaggaat gttgagtacc tttttaggat    423600
     ctgtttttca tctgtgggtc ttctgaaaaa aaatctaacc aggtttttgc cctctgtatt    423660
     aggtcagttg atatttgctg ttgagttgta tggttaattt atatatttgg gtggaacttc    423720
     ttgttagata tataattgca catagttttt tttactgtgc ttgcttttgg tattaaattc    423780
     aaataatttc tgaatcaatg acgaatattt ttccatgttg tctatgattt atggtttcag    423840
     gttatgttca tttttagttg atttttgtat atggtgttag agaaggtcta gtttcatttc    423900
     attttttttt tgcacatgca tgaccatttt ctacaccatt gattgaggag actgtccttt    423960
     cttcactgtg tgttcttggc atacaaaatt aggaagacac ataattaaaa aagaaaacat    424020
     cagatgaata ttcctggtga acatagacct aaaagttctg agcaaaatac tagcaaatag    424080
     aatccagaag cactttaaaa tgtgatacat catgatcaag taggctttac ccctggaagg    424140
     caagtttcat tcaacatcca aaaatcagta actgattcac tatgtaagca aaataaaagc    424200
     gaaaaacata gattatctca atagatgctg agaaagcttt taatagcatc caacatccac    424260
     tcataataaa aaccctcaac agactaggca tcagaaaaat atacctcgaa attataagag    424320
     ctatctatga caaaccacag tcaacatcat actgaataag caaaagttca aaccccttga    424380
     gaattgaaaa aagacaagga tgccgtctca ccactcctat tgaacatagt attagaaatc    424440
     ctagtccgag gaaccaggca ataacaaaaa taaaaggcag ctgatatggt ttggatttgt    424500
     gtccccaccc aaatagcatg tcgaattgta accaccaatg ttggaggtgg cgtctggtaa    424560
     aggatgatta gatcatgggg atgtgttttc ctctctcatg ctgttctcct gacagagctc    424620
     tcaggagatc tggtttcaaa gtgtgtggta cctctccctt atctcttcct cctgctgcag    424680
     ccatgtaaga catgatgttt cccattctgt catgattgtc aggttcccca ggcctcccca    424740
     gccatgcttc ctgtacagcc tgcagaacca tgaattaaat taaattaaag aattaaatat    424800
     ctttataaat tactcactcc tgtgtgtttc tttgtagtgt gagaatggac taatgcagca    424860
     tccaaatggg aaaagaatgc aatgtatctg tccaaactga tgataaaatt ctatacctat    424920
     aaaattctaa agactctgac aaaataattc aagagagata aacaactttg gtaaagtctc    424980
     aggatacaaa aatcaatgta caaaagtctc cagcatttct atatcccaac cacatccaag    425040
     ctgagagtga aatcaagaac acgatcctgt cccacttaca atgctcacaa aaaatgaagt    425100
     gcctgaaaat acaggtaaaa aacaaggtga aaactgtata aggagaacta ccaaacacag    425160
     cagaaagaaa tcacaaatga cagaaataaa tgtgaaaaca tttcatactc atggattgga    425220
     agaattgata ttgtaaaaat tgtcatactg cccaaagcaa tttacagatt caatgctatt    425280
     tacataaaac tctcaccagc attcttcaga taaatagaaa aaaaattcta aaatttatat    425340
     ggaataaaaa aaagaccctg aatagccaca gcaatcctaa gcaaaaagaa caatgccaga    425400
     ggcatcatgg tactgaattt aaactacacc ataaagtcat ggtaacaaaa acagcttggt    425460
     actggtaaaa gggcagacat gcagaaaaag tggaacaaaa gagaaagcag aaataaagct    425520
     gcatgcttgc aaccatctga tatttgataa ggctgacaaa acaagcaatg gggaaaaaac    425580
     tctattcaat aaatggattc tggcagatat gcagaatgca agagtgaagg aggttgggca    425640
     gcttctacct agatttcaga agatgtgcat ggagaaccct atatgccaag gaagaagcct    425700
     gctgcaggag tggagccacc acagacagtg tctattagga caataccaaa gatggggaaa    425760
     gatggggtcg gaacccccat tcagagtccc cactagggca cttcatagta aagctgtggg    425820
     aatgtggcca caaccctcaa gatcccagaa tggtagagtc aaaggcagct tgtcctctca    425880
     gcctggaaaa ccttctggca cttgactcta acctgtgaaa gcagcaccat gggctgtgcc    425940
     cagctacagg gatgatattc ctgaggtttt tgggaccccc tttgtcctag tgtgccctgg    426000
     tggaagtaca taaagtcaag agagactatt ttgtagcttt aagatttaat atctgcctta    426060
     ctaggcttta ggtgtgtcta gagcctgttg gcagttcctt tggccaattt attccttttg    426120
     tattgacaat atttacccaa ttcctgtggc actattgcac cttggaagta aacaactgtt    426180
     taaaactttt tttttttttt tttgagacgg agtcttgctc tgtcgcccag gctggtgtgc    426240
     agtggcgcga tctcggctca ccgcaacccc tgtctcctgg cttcactgcc tcagcctccc    426300
     gagtagctgg gattacaggc gctcatcacc acgcccgact aatttttgta tttttagtag    426360
     agacagggtt tcactgtatt agccaggatg gtcttgatct cctgacttcg tgatccaccc    426420
     gcctcggcct cccaaagtgc tgggattaca ggcgtgagct gccacgccca gcctgtttaa    426480
     aacattttgc aagctcatag ctgcagagaa attgccttaa gactcagagg agactttggt    426540
     attttcagtc ggaggttgac caagtgaaga gtttggtcac tattaaaaaa actattaaaa    426600
     aaaagatgac tttgcaatct aagaaggaca tgagatcagg acacaagagt aaaatggtat    426660
     agtttagatg atggtccctg ctaaatctca aggtgaaatg tgattcacag catttgcggt    426720
     ggggccgact gggaggtttt gagacatggg gaaagatcct tcaggaatgg cttggtatcc    426780
     accccatggt aattagtgaa ttatttctgt attatctact gtgagatctg attttcaaaa    426840
     taatctagca accatcctcc cctttcatgt cctccctctc accatgtaac acagcctgtt    426900
     tcccctttgc cttccaccat gactgtaagc tttctgaggc catcacatga tgcagatgct    426960
     gatgccatgc ttctcactta gcctgcagaa ctatcagcca aataagccaa atagactagt    427020
     acactgtcca aagcaatata cagattcaat gcaatttcta ttaagtaacc aatataattg    427080
     attacatatt ttaaaaaacc ataaaattta tatggaatca aaaaagagct tgaacatcaa    427140
     aagcaaccct aaggaaaagt aacaaagctg gaagcaccac attgccggac ttcaaattat    427200
     actacacaga tataataaga atgacatcat cgtagtgaca aaaaataaaa attgatgtaa    427260
     cacaacagac agcccagaaa taaagccaaa tatctacgac cacctgttat ttgacaaaac    427320
     tgacaaaaaa tatacactgg agaaacaacc ctttattcaa taagtggtgc ttggaaaatt    427380
     gggcagccac acaaagaaga ataaaaccag acttctatct cacaatagac aaaaattaag    427440
     tgaatatgga ttcaacactt aaaaatataa actgaatcta caaaaatact tgaagaaaat    427500
     gcaaaaaaaa agttctctgg acattggcct aggcaaataa aacatgacta agatttcaaa    427560
     agcaaatgca atgaaaacac agacaaacag gacttaagta aaaatcttct gcacagaaaa    427620
     agaaataatc aacatggtga gtaaacaacc tgcaggatgg tagaaggtat catctcacct    427680
     cagttaacat ggctcatatt aaacacacac acacacacac acacacacac acacacaaac    427740
     aaaacactaa ccaatgctcg taaggataca gagaaaagaa aactcttatg cattgttgag    427800
     gagaatgtaa actagtacag tcactatgga gagcagtgtg gaggtacctc aaaaaactac    427860
     aaatagaact accgtatgat ccagcaattc cactactgca aatttatcca aaggaatgaa    427920
     aatcattgta tcagagaatt ctgcacccca gtgtatgtgg caggaccttt cacaatagcc    427980
     aagatatgga atcaacacag gtgttgaaca caagagaata atggttaaag atgatgtggt    428040
     gcatatacat gacgaagtac tcatcagcca taagaaatga aatcatgttg tttgtggcaa    428100
     tgaggatgga actggaggac attatgttaa atgaactaag ccagggacag aaagtaatac    428160
     accttgtttt ctcactcata ggtggatact taaaaaaaaa tggtctccta gaagtaaaga    428220
     atagaacaga ggataccaga cgctgggaag ggtagggtaa aggacgagat aggcagagat    428280
     ttgttaaata atacaaaatt agagctaggg aggaggaata gattataatg atctttacca    428340
     ctgtaggatg gatacggtta acaataatat gtagttttaa atagttagaa gaaggatact    428400
     gagtgttcct aacaaaaaga aatggtttga gatgatggat gtgctaatta ccctaatctg    428460
     acccctatgc attatatgta tcaaaacatc actatgtacc tcatgaatag ttacaattat    428520
     tgtctattaa aattatattt taaaataaca caaaattcat tggcagtgac ataaatggag    428580
     gctctgggat aggtgtgggg ataaagggag gctctgggat acattacagg cctaacatgt    428640
     cagtgggcag aggagcttcc attagctttt tctatttgta gcactgagta gatgtgacat    428700
     ttctcaaagt ttgccctaca atagagacct gagttccagg gaggtcttgc aggcagctgt    428760
     gcagatggaa gaagcaggag tagagggttg ctggcccatt caacaatagt aggatgatta    428820
     ttgtgcatgt agagaagtca acattgtttt aagtagttgc atcactggat gacatgaaaa    428880
     acaaatccag tggaaatcct taaatattat tggtagtgaa actgttttgg gtgaggaaat    428940
     gttcactgaa gttaagtgag ccagtaaagc tgttaagaac tataaaaaca ttggattgga    429000
     ttcagctttg tctgaattta atgtgattat tactggctca ggcaggggtc acaaatcaag    429060
     atggataggt ggtaccaacc agaaaaagcc atggaccaga aagattcaca gctgaattta    429120
     ttcggatgta taaagaagag ttgctaccaa tcctagtgaa actattcccc aaaaattagg    429180
     ataagggact cctccctaaa ttattctgtg agaccagcat tattctgata acaaaacctg    429240
     gcagagacta aaaaaaaaga taactttggg ccaatattca tgatgcaaaa atgctcaatg    429300
     aaatactagc aaactgacca cagcagcaca tcaaaaagct aatccaccat gatcaagtag    429360
     actttgttcc taggatgcaa ggttggttca tatacgcaaa tcagtaagtg tgattcgtct    429420
     cataaacaga actaaaaaca aaaccatgct atcaatggac atagaaaagg tattcaatga    429480
     aatttaacat tgtttcatat ccaaaaccct caactaggca tgaagaaaca tacctcaaaa    429540
     taataaccat ctatcatgaa cccacagcca aaatcatact gaatgggcaa aagctggaag    429600
     cagttttccc tgaaaactgg aacaagaaaa gcattcccac tctaaccact cctgttcatc    429660
     atagtcatgg aggtcccagc cagagcgtgc aggcaagaga aagaaaagca tcagaaccgg    429720
     aagagaggat gtcaaaccat ctctgtagat gaaatgatca taatcccgag tctgtagttt    429780
     ggtgattgac attaatttgg aaaaattgtc agtcatcatt gctttaaata tttcttctta    429840
     acctttctct gttccttctc tttcccatgt tataccctta taattgtctc acaattcttg    429900
     gataagctgt tctggtttct tatgggtgtg tatttgtgta cttgtatgtt tgtgtgtgta    429960
     tttgtgtgtt tactttttaa aaaaaatatt tctccatttt cagtttggga cattgctact    430020
     gagatatttt taatttcaga tttttttcct cagccatgct gcgtctacat ataagtccat    430080
     tgaaagcatt gcttaattct gttacaatat tcttgatatc tacatttttt tgctttttaa    430140
     gagtttgcat atttcgactt gcataacaca actgttcctg gatgccctct gcttaactca    430200
     tcacagccct tagcatatga gttatagcgg ggtcttaaat tccttgtttg gtaattccaa    430260
     cttacatgac gtagctgagt ctcattctaa tgcttgttct gtctcattac actgtgatat    430320
     ttttctttta gtatgtctcg tgattttttt agccagaaat tatgtaccag gtaaaagaaa    430380
     ctgctgtaaa gaggccttta gcgatgtcat tgtaagatgt ggagagaaat gtgttccata    430440
     atcctacggc catggcttac tctagcaagc ttaggccttt gaaaataaac ccttacatga    430500
     aagtgaaaac ataagtctta gatttggaga atttgcttgc aaatcaaata tttggaaaat    430560
     gacttttatc acaaatgtac aaattacctt acaattgaac aacaacaaaa ccacacaaat    430620
     taattttaaa atgggcaaag atctgaatag aaatcgcatc taaaaataaa tataaagaga    430680
     tcaacttcat tttcattagg tatatgcatg tttaatattc aaaaaatact actatcttat    430740
     gaggatgggt aaaatacaca atatagataa taccaaatgg tgatgaggaa gttgaaaaac    430800
     aggaacattc actctttgct ggtggtaatg ctgaaatggc acatagacat gtacagttaa    430860
     taatttagtg tatattacaa aacagctaga agagaagatt tagaattgtt aacacaaaga    430920
     aataatcaat gatggagatg aaagatatac cagttatcca gatttaatca ctgcatataa    430980
     taatctttta tcaaaatata taccccataa agattttgaa ctcatgtatc tatataaatt    431040
     aggtattaaa aaacagcaat gcaggggaat ccagctaatt gcacattccc tgaatgtacc    431100
     ctttcttgtc aaaaaggagt tccagagact cttgaaaacg tagtgcagtt catgtgagaa    431160
     atagaggttc agaaatgcct cattataccc tcctcccttt ggaattcaag cacaactaac    431220
     cagtattttt cattaaaaca gatcttaaga ctcagaaaac agattctttg cagcagtaag    431280
     ataccaaatt ctaacctgac tcaagaatag gatcacatga cagagagcag accttgaaaa    431340
     gaatcaaaga cttttaccct aaaatatatt tatttgacat attttaaaat caccctgcac    431400
     agttatcttt tgagagagaa atttacgttc tgtagcgaat ctccttccct ttccaggtct    431460
     agttgttaga ttagctgaga gtctagcatc ttttaaatgt catagtagaa aacatttgct    431520
     atctactgcc tctaacggtg gccacataag agaattcatc tacataataa gaatcttgtt    431580
     ctccaaaacc cgttatccta acctatgtag ggaaaagaga gatcagactg ttactctatg    431640
     tagaaaagga agacgtaaga aactccattt tgatctgtac cctgaagaat tgttttgcct    431700
     tgagatgctg ttaatctgta acttttgccc caaccttgtg ctcacagaaa catatgttgt    431760
     atggaatcaa agtttaaggg atctagggct gtgcaggatg tgccttgtta acaatatgtt    431820
     tacaggcagt atgcttggta aaagtcatca ccattctcca ttcttgatta accaagggta    431880
     acaatgcagt gcagaaagcc acagggacct ctgcccaggg aagccgggta ttgtccaaca    431940
     tttctcccca ctgagacagc ctaagatatg gcctcgtggg atggaaaaga cctgaccgtc    432000
     ccccagcctg acaaccgtga agggtctatg ctgaggagga ttagtaaaag aggaaggcct    432060
     ctgtctcctg catgtccctg ggaatggaat gtctcggtat aaaacccaat tgtacatttg    432120
     ttctattctg agataggaga aaaccaccct gtggctggag gtgagacatg ttggcagcaa    432180
     tgctcctctg ttactcttta ctacactgag atgttagggt ggagagaagc ataaatctgg    432240
     cctatgtgca catccagcga tagtaccttc ccttgaactt atttgtgaca cagattcctt    432300
     tgctcacacg ttttcttgct gcccttctcc ccactatcac cctgttctgc cacattcccc    432360
     ttgctgagat agtgaaaata ataatcaata aatactgagg gaactcagag accggtgccg    432420
     gtgtgggtcc tccgtatgct gagcaccggt cccctgtgcc tactgttctt tctctatact    432480
     ttgtctctgt gtcttatttc ttttctcagt ctctcatccc acctgacgag aaatacccac    432540
     agttgtggag gggctggccc ccttcaaaca cagaaacgtt tttctactga tttcagctct    432600
     ttaaataaaa agttaagtct ttcaatcaga aaatctttga attcacctgt aagctgtaat    432660
     cccctcccca cactgcctca cttgtagctc ttttatcttt atgaaacaaa tcgacacatg    432720
     tctcatatgt attgactgat gtctttctct ctaagacatt aaatttaagc tgcaatcaag    432780
     ccactttggg cacacgttct caagatcttc tggggctgtg tcacagggca tggtcctcac    432840
     atttggctcg aataaatcta aatattttgc aaagttgact ttttcattaa tagttcttac    432900
     agaacctact ctgtgctctt tgttacaagc ataaaatcaa tatagtatta gttataatag    432960
     tattgggtat accacatatt ggcaatttct tatgaagtta ggcatagact aacaatggga    433020
     tcaagtgtaa atgttttaat gtatttatgc aagttatttt gatatttatg ttctgcagat    433080
     acttctatca gatttattat atcagctaat tatttgtttt atgatttttt gcattttttg    433140
     tagatgataa caccatatct tttctaggcc tttattgtag aaaattgtgt gaaactactc    433200
     aggactacat ttgaaagtcc ttattgcagt ggctgccaga attgaagtac atttcctctg    433260
     tagccaccag acacccagat tcctccagta acaccttaat tttgtttcct gcttggcttt    433320
     gggctttagt cttgtgctat ccagagagac tctgtctttc aaatccccca ggtcattcta    433380
     aagccacatg gaccggacaa ttgtcaatgt ggtgggaagg gtcgcactga acgggcactc    433440
     tctgtccttc tgagggagcc atactgcagg cagacatcac agtcctggct caggttgtgt    433500
     ccttccaagt atccctgccc tcctgcaggt gtgatgctga tccatgcatc ttcctcccat    433560
     tcctgggtag agggtctcct tgttttttcc cagatcttct tccagcagcc actatactat    433620
     gtcttcccac tgatgtcttc agcttcctct tttctgccct tcccagcagg atgaggcttt    433680
     tattcctgaa gaaagagaga ggaggtggga gatgaggctg ttattcctga gagaatgaac    433740
     agggctttgg agcttttctt tcttctatcc cagtttctat gagttcctcc agtaccagta    433800
     acacaaaggt gttagtggct ttgcccctgc atgttaattc tttagcaatg tagtggtgac    433860
     agtggaggtg ggtctggatg catttcagct gcagttgctg tttttctttc cagcacagaa    433920
     ccatcttggg agctacaggg aagagctttt tgtaatgtgt cctcaatcct gggagaaaag    433980
     ttttcaatag caataggaat ctctcagttg tatgttctct gagaatttaa acaataactt    434040
     atttattata ctcaatttta aacaatgtaa taaatatgtc taatttaatc ttgcattagc    434100
     ttatgtgaca tttgctggcc attgccccag ataaggccat tttctcttcc tgtttatccc    434160
     tgcaagtgac tgacttttgc aagatgtaag gtttcttgtt tgccctataa catcaataat    434220
     ctgaaatatt aaagaaaatg tgctaatttg cagatcagta aggttagttg tttctgtaaa    434280
     aatatatatg tttttttgag atggagtctt gctctgtcac catgctggag tgcagtcgtg    434340
     cgaacttggc tcactgcaac ctccgccccc ttggttcaag cgattctcct gcatcagcct    434400
     ccatagtagc tgggactaca ggagtcttgg cctccaaaag tgctgggatt tctggcataa    434460
     gccatcgcac ccagccaaaa ataatatatt ttaaggggtg catacatctc caggctgagt    434520
     aggagattta tttgtagtac tcagtaactg gaatttacac aactattaag gagcattata    434580
     aataaatagt aagggtgcac tcattaactg taatactgtt cctcattaga atcaacacat    434640
     tctttagttc atgtcctttg cagggacatg gatgaagctg gaaaccatca tagtcagaaa    434700
     actatcacag ggacaggaaa ccaaacaccg catgttctca ctcataagtg ggatctgaac    434760
     agtgagaaca catggacaca gggaggggaa tgtcacacac cagggcctgt cggggtgggg    434820
     agcaagggga gggacagcat taggagaaat acctaatgta gatgacaggt tgatgagtgc    434880
     agcaaaccag catggcacat gtatatctat gtaacaaacc tgcacgttct ccacatgcat    434940
     ctcagaactt aaagtataat tttaaaaaca cattatttat atagaaccaa attactggct    435000
     cacaagtaat tatactgtgc agaaaaccac acaaatgaaa ttgaaactat aagatttcac    435060
     ttgaacaaaa tcttgaaaaa tagatctaaa gtaacaaaga tgactgactc aatatggtga    435120
     gaggaaaatg actgggaggc aggaatgaga ggaacacagg gaagctttta agtgtaattc    435180
     attggtatct tgattgttat ttgggatgca caggtgagca catgtgtaat aacatttttt    435240
     tcctttggag acattttctt gctttgtcat ccaggctgga gctccgtggt gacatcatag    435300
     ctctctgcag cctcagaccc tgggtctcca gcaatcctcc tccctctgcc ctatctagct    435360
     gggactacag ctgtgcacca ccatgctcgg cttcagtgct tattcaacca gacactttaa    435420
     ttatgcagta tttattacgt ggcaataatg cctcaataag ggtattgcaa ataagtggat    435480
     ggataatttg ttcaattaag attgatggaa atatagacac ttacatgaca aatgtatgac    435540
     aatcaagaaa ataaaactgt aggaaacata ctttactttt tgttaggtaa tcgcaacagt    435600
     gcatacacat cacaccatgt tctcattaca gagaaaaggt tctgcaaacc tcacttggtg    435660
     tgacctcctg tatgctgggc ttggttcagg gagaagtcag gaccagtggt tagaagcacg    435720
     ggcccagata accagactcc ctctgaccaa atgtgagtgc tggggacatt gtacaatcca    435780
     tctgtgtttt gctgatattt tttcatctgt aacatggaaa taacattgat actacatacc    435840
     atggtttctg tgcatatgga aaaataaaag atgataggtg caaactttaa atacacacag    435900
     tttatgtaga tcaattgtac ctcaataaaa ctgttttaaa ataaaaatta caaaactata    435960
     aattttatag gttttaaggg tttatcatag aacaaactta caataagaaa ccagaatttc    436020
     caaatgctat caatatcaca aatctccccc aggacactct cacatgctct gagccccact    436080
     ctctcctcag gcgtcccatc ccagagcttg gcatgtatta ggagacatgc aaatagggcc    436140
     ctccctctgc ttatgaaaac cagcccagcc ctgaccctgc agctgtggga gaggagcccc    436200
     agccctggga ttttcaggtg ctttcatttt gtgatcagga ctgaacacag aggattcacc    436260
     atggagtcat ggctgagctg ggtttttctt gccgctattt taaaaggtaa ttcattgaga    436320
     actattgaaa ttgagtgtga gtggataaga gtgagataaa cagtggattt gtgtggaagt    436380
     ttctgaccag ggtttctttt tgtttgcagg tgtccagtgt gaggtgcagc tggtggagtc    436440
     tgggggaggc ttggtccagc ctggggggtc cctgagactc tcctgtgcag cctcaggatt    436500
     ctcctttagt agctatggca tgagctgggt ccgccaggct ccagggaagg ggctggactg    436560
     agtggcacat atctggaatg atggaagtca gaaatactat gcagactctg tgaagggccg    436620
     attcacaatc tccagagaca attctaagag catgctctat ctgcaaatgg acagtctgaa    436680
     agctaaggac acggccatgt attactgtac cagacacagt gagaggaagt ccgtgtgagc    436740
     ccagacacaa acctccctgc aggggcacgc ggggccacca gagggtgccc aggatcccct    436800
     gaagacaggg acagcccaaa ggcaggtgca gatggatgtc aagagggtct gtggcttcgt    436860
     ctacatctaa ctgtttcctg gtgagcctct gtatatttat ttttctgtgc ccactaatga    436920
     ggttgctaag tttaaacact tttttttttg agatgaagtc tcgtgcttgt gccccaggct    436980
     ggagtgcaat ggcatgatct cagctcactg caacatccgc ctcatgaatt caagcgattt    437040
     tcctgcctca gcctcccaag tagctgggat tccagacaac tgccaccacg cccggctaat    437100
     ttttgtattt ttagtagaga cagggtttca ccatgttggc caggctagtc tcgaactcct    437160
     gacctcagga gaccctccca ccttggcctc ccaaagtgct gggattacag gcatgagcca    437220
     ctgtgcctag ccaaaaaaga cttcattatt ataggaggaa aaattctcat ctgcaagtgt    437280
     cactgatgga agcagaaaaa ttgcccagga agtcaggtga ggctgtagac actgtcaacc    437340
     caggatgcaa atctcaccac aagtgcagga gaaagggcag ggggttgatg gatcatccta    437400
     actaacgtat ggtttaagct gagcccagca agaccctcag tgcctcccta accacagttg    437460
     cccttcaggg ataacccatg tgctcaacag cagccacacc ttagtatctc cactgtgcag    437520
     tcattgtcta ggacaagctc ccaggatgtg gggctttggc acaaaccagg tcatggatgt    437580
     cagagagggg cagctgggtg tctggtccat aggctccctg atgttggagg gttgagaggt    437640
     gcattctctg ggccaacata tactctaggg atatttactt cagaacacat acttttattt    437700
     gttggtttat ggttaatggg gtattcatcc cttcaatcat ttgtcctttg atttacaaaa    437760
     acattccaat ttcactttgg aagtaatttt attatgtgca attaagtcat tattgattat    437820
     agtcacacta ttttgctact aaatagtagg tgttattcat tctttctaac tctgtatttt    437880
     gtacccatta accatatcca cctgttctct ggcccccact agcctcccag actccagtta    437940
     ccatccttct accatccatg ttcctgagtt taattctgtt gatttttaga gcacacacat    438000
     aagtaagaac ctgcaatgct ttttttttca gtgccttgtc tttttcactt aacatagtga    438060
     cctccagttc catccatgtt gttgcaaatg actggatctc atggaatacc taattttatt    438120
     cggttctttc atatgacgta gagaagcaag catgcagtct acaaacaact gtaattttca    438180
     catttacccc aaatgaatta ttacttattt ctgtgcaggg tccaggcaca tagtagcctt    438240
     tctcttgagg gactgttaaa tttagagtga gatcacttgt ttctcatatt atttgattcc    438300
     tgtccatgtg cagagatctt aagtaatctg cttctttctg cacactaact ctgaccttct    438360
     cgacaaaaag agtagagatt ttcatgtttc ctgagtctgg ggtaggagtt agtcctacac    438420
     aactcagagc ctgcagagac ctcccagtgc agttttatag acacaggtgc tttctcatgg    438480
     ggggtgagac ctccacttcc tgtgattgct gctcagatct aactgtgatt tcagatttag    438540
     gacaccatta ggctctcaaa ccatttctat tctaaaaatc agcctcatca tagaaaatgg    438600
     aaataattca atatccattt ccctgaccgt atttcatcct tgtttttaat ttggtgcaag    438660
     tttattacat atttgcctga atctaacttc agaatcaact gaattggtct agaaatctca    438720
     aaaattgaac agaagtggat tttattattt ttaccaaaat gtgtgtaggt agggtttcca    438780
     atgtatcgtc cagaacttgt gcatgctaac aactgttttt ctcagtcaat tatcagcctg    438840
     gtgaagccct tgcaaacccc ctcactcacc tgtgctgcct ctggattctc tgtcacaatc    438900
     agtgcttcct gtaggcactg gatggaaagc acaggagtgg gtcaggtgca tacgtgatga    438960
     gtggaggatg aattccagcc cacttatcat gaattcagac aagcccacat gttcccacat    439020
     gcactatatc ttcagacaag cagatgtgtc tgcacatgaa ttcttctttc agacaaacac    439080
     gtctgtcccc tcatggactc ttgccacaca caagcacacg tggcttcttg tggactgtac    439140
     tttcagacaa gcacacattg tttcatgtgg acatcttcct cccacaaatg cattttcccc    439200
     tgagtagact cttctcccac aagcccatat gtccgcacat ggacatttct cacagacaaa    439260
     cacccatttc cccatgtagt cccttccctc agacaagcac acgtattccc aggtagactc    439320
     ttcccttaga caagcacaga tgttcccata tgaacccttc catatgacaa gcacacatgt    439380
     tcccatgtga actcccgtat aacaagcaca catatttcct cacgggctca tcactcagat    439440
     aggtctgcat gtccacatgt ggactctact ctcagacaag cccacaggtc ctcacatgga    439500
     ctcttctctc aaacacccgt tctccattgt agactcttcc cttagacaag cacgcatttt    439560
     cccatgtgaa ctcttctgca tgggaagcac acatattccc acagggactc ttctctgaaa    439620
     cacccattct ccattgtaga ctcttccctt agacaagcac gcattttccc atgtgaactc    439680
     ttccgcatgg aaagcacaca tattcccaca gggattcttc actaagacaa gcatgattgt    439740
     ctccactgtg acccttccct cagagaagta cacttgtcta catatggact cttctctcag    439800
     acaagcaaac atgtccccac gtggactctt tcttctgaca agcacacgtg tccccgtgtt    439860
     gttataatca ttttctcacc tctgtctcac aaagttccct gtcctccaag aataattttg    439920
     acctctcact tctcattcac actatggtga ttggtgcaca gcattttcac ccaagagaaa    439980
     tgcaggcagc tctcagaatt tattgtgatg gaggcaggaa gacaaacaca aagataaaac    440040
     ttgtcgctac cttgctgggc tgtgttctgt gcagagtctc tgaaggctgc cctggggctg    440100
     tgtcctaagc tgtatctctc tgattccact aggagaatgc acatagagaa attcaagctg    440160
     aatggtgtcc tgatccctgc ctcattctgc tattcttccc tattcctcag aaattcaccc    440220
     aacattgccc tctgtcttgt attgtctaca ttctcacaga ctctctcata gaacttcagc    440280
     ttcaatggtc agagccctgg tcacttcttc ccaactctcc gcacatgcca agtgcacatt    440340
     agagactcaa taatgaggct caccaggggc agtagtagtt tcatctatca cagtggagat    440400
     gaaaatccat gatttaaaat aggtttctaa aaaagaaaat atgtctcaca tcaaacacaa    440460
     atgagagaat gcaacggcat aattcatttt caggtttttc atatgtaaat ctcctaaagt    440520
     gcataattta gtttttagtg tggttcctga tccatgaatc cttttgcctt cttcattatc    440580
     tttcattcaa acaaacagag accataccca ctgttcacag tgtagcctca tggagaacat    440640
     cttcaccagt cagcccagtc agtgagtgag gcctttaaat ccttcatgct aattcaaatt    440700
     tctggcttta ttcttagtag accccaggtt ttcggggatg ttgggttgtg ccagcactcc    440760
     taaataaggg agatagaaat tatttcctca ggcatcctct tgcaacattt ggacaattgg    440820
     ccatagtttg attattgagg tgtgatgcat caaaataatg ttagttcaat cttctctcaa    440880
     ccagtcattg aacattgaaa ttttgtaatt atttatttgg atatctctga agtttacctc    440940
     ttgaatattt aagtcatatt ataaatagtt taagctgcct actaggtaaa atgatctgct    441000
     gcagaatttt gacttccttg ctgtgcattt ttgcatttaa attcaagttt ctattactcg    441060
     tgtgtctaga atgtaaaaat acgtcttaca ttttctcgaa tttttcattt cactggagtt    441120
     tccagaagat gagaaaacct cccttgtgta taaaatacct ggtaacacat tgctcaatag    441180
     tttcgttgct gagaccagct cagtcgggag accctaaccc agcggtgcta gaggaattaa    441240
     agacacacac agaaatatag aggtgtgaag tgggaaatca ggggtctcac agccttcaga    441300
     gctgagagcc ctgaagaaag atttacccac gtatttatta acagcaagcc agccattaat    441360
     attgtttcta tagatattaa attaactaaa agtatccctt atgggaaatg aagggatgga    441420
     ccgaattaaa gggggtgggt ctggctagtt atctgcagca ggaacatgcc ctaaggcaca    441480
     gatcactcat gctattgttt gtggtttaag aacgccttta agcggttttc tgccctgggg    441540
     gggccaggtg ttccttgccc tcattccggt aaacccacaa ccttccagcg tgggcattat    441600
     ggccatcatg aacatgtcac agtgctgcag atattttgtt tgtggccagt tttggggcca    441660
     gtttatggcc agattttggg gggcctgttc ccaacatttc ctctttaatt tttttctcct    441720
     ctcatattgt actgttataa gcaatgagat atcaggacac atcatcaaca ctttgctaag    441780
     aaatatcttc agctaaattc ccagagtgtc atatactaat cctaatttta actaaggtaa    441840
     cataattaag atacattcta taacaagtca cacattttat ccagtatcca gtaacatgtt    441900
     tgtgacagaa acaaagcagg tgcttcctct gcaaagggtc atctgaggtc tgagttgagg    441960
     gtcacacagg tatttttctg gtttacaagg gtgggaacaa caacagtaat aacgactctg    442020
     aacagcactg gcccttcctt gaaaggttgc aggtaaagct actgctgcta tatcaaatta    442080
     acaccttact agaataaagc ccagagggag ggtccacatg ctaggtcaca gagtgaggag    442140
     aaatggagct gtgctctgct ctccacacta cctacaagac cccagacaca cgcccaggtt    442200
     ctactgatgc aaggcatcta cagtgcagtt ctaaggcacc tgcttcctgg ggcagggagc    442260
     atcctcctgt caattgtgca cctgctcctt ctgcaagcta ttaagtgagc acttctattg    442320
     ccatgaatta tacatgttct ctctcttaac atgtatattg aacatgctta cttactttgc    442380
     attttatttt tctctgaaat acactggatg agcagaagaa aataaatcac acccctgatg    442440
     ctttccacac atacaaagat tcctgaagac agagctgact gatatcctca ccagtagacc    442500
     actgcctttc agagatgatc ttggtattca agttccagca attcttgaag ttaaagggca    442560
     cctctatctc ctctccactg ctttgcacct cacctgatat cagctttctc attagaatgc    442620
     cttgtgttta aggaatgaga gttcctgtca caggtgcaaa ggcccaggtt ctcccgctgg    442680
     ctccagtcca gggctaggga cacctgtaag accatgatat catctgatac ataaccttta    442740
     catcataagc tttacataac ttttacatac ataacatcac acatagcatt caggatacac    442800
     acatcagatc acctttacat gctctaacat gtaaagatta ggaaagtcat caagctcatt    442860
     cttatgtcaa aaaacatctg cacaatttga acatcaacaa tttttgaaat ctatcaagga    442920
     attgaaattg caagggaaat tacccattcc aaatactgaa gatacagaca tgtccaggat    442980
     cagaggtgac actggctgaa ctggaaaaga acttgaagaa agcacgagtt ggtgagagca    443040
     cacacatggt aaatgctatg aaagcctaaa cggcagatgt ggactagggt gagattcctg    443100
     gggacctaca ctccgtagtc ttctgaactt tccttcaaaa tcttccagat tctcaggtat    443160
     acaatccaaa taattttttt atgggtctga attggggagg attaattacc gagaaatgta    443220
     ccaagagcct tctaaaaaaa aaaaatcctg taggaaatga gattttccag aacctggagg    443280
     aacctacagt tccctaaaga cagacactga ggagaataaa tctcgacagc gccacctgga    443340
     ggtggaggtg aagagcttcc tggaggagct ggacgggaag atccacgacc tgaatgactc    443400
     tcgcccaggc ctcccaagct gggcaccgac aactagggct ggtggaggcc cgggatcccc    443460
     accgtgagtc ccaagcatgt gtgcgagacc agatggcgct aggacgttcc ctgtgtgcgt    443520
     tgcttctgta aatgcagacg cagtttgtcg tgtttccaaa ccagttgtgc cgtccactca    443580
     ctcctttcca gagtagaaat ctcctctccg ccgggcgcgg tggctcacgc ctgtaatccc    443640
     agcattttgg gaggtcgagg cgggcggatc acgagatcag gagatcgaga ccatcctggc    443700
     taacacggtg aaaccctgtc tctactaaaa atacaaaaaa ttagccaggc gtggtggcgg    443760
     gtgcctacag ttccagctac tccgaggcta tggcagtaga atcgcttgaa cccgggaggc    443820
     ggaggttgca gtgagccgag atcgttccac tgcactccag cctgggcgac agaacaagac    443880
     tctgtctcat taaaaaaaaa aaaaataggc tttaggaagt agctcctaga tgggcagaat    443940
     cggtttaacc ctgtgtgtcc acaggatctg gagcctctct ctccttagat taggccacct    444000
     cctcaggatt acagggctct tcagttttct caacatgctg ttgttgcccc aaataaacac    444060
     aaaagcactt tattttctta tgcttacctt taatttttca aaacaacata aaggtgataa    444120
     ttttaacaat aaacatatta caacctacta tacatgatac ccttcctgta gttcgaggtt    444180
     tattctcagg aatttatatg tattacttat tttcaattgc tttctgatat gggatgtcaa    444240
     tgccacttta ttacagtctt tattctttta agttatctct aaaatctcta aaaattaatc    444300
     tttccaaagc accactcaaa accagtgtat tattagaact tgtgggtgta atcgttggac    444360
     caatattaag aataaattct taatggacta actcagatat aaaatttctg tccttcctct    444420
     ctctctacat atatacataa cgactttgag gtttcagttg aatcagtcca ttcattgaat    444480
     gtatgacaat aggttgatct aattgtatac gttaaatagc atgaagaaac ctcaacaata    444540
     gcaaaagtag ccccttacca aaaacagctc tataatgtta gattccactt atgagaagtt    444600
     caaaagagaa aaacatggat ctgtattgtc agaaatctaa acatttccgc acgcaggagc    444660
     tgaccacagg cacaggaaga cctgtgttgg gggatggact tgctcttcgg tctaccttag    444720
     gtgccgggga caagagtatt cacgtttgcc agaaactctc tagtgacaaa ttccagatct    444780
     atgcatttcc tcttatatgt aaattttctc ataaaaacaa aaaataatgt ataaaataat    444840
     ttaaaattca gaacataata aaaatgacat tacatgccta tccaaaacat tcagtaatga    444900
     acattattat atgaattaat aaaatgcatt caaatatacc tactgaaatt aacttctgga    444960
     aaataaaaag ataagggtaa catccgtaac ttaaaaatta ataagcatat atttcataga    445020
     gaagaaaaag gtagtcaaga catatgtgga atgtgtattc ttttttccaa tcaaatgtca    445080
     ttaaactatt taaacaattt tttaacctgt gtcataagta tagattacta atatttgtct    445140
     gatattacaa tgtgaacaag ttttaaagaa aaaaggatat ttgacagcat gtgaactaaa    445200
     ttgaatttat tcttaatatt ggtccaacga ttacacccac aagttctaat aataccctgg    445260
     ttttgagtgg tgctttggaa aaattaattt ttagagattt tagagataac ttaaaagaat    445320
     aaagaatgta ataaagtgac attgacatcc catacataac aacatattga tctatttttt    445380
     gttgatggac tttaaatttg tttctagttt atgaatatta taagcaaagc tgttacaaat    445440
     accttagtgt aagttttcct atgttgtctc atttttattg tgaaaacatg acgtatgcat    445500
     ttctttcttg taagagtaat tttaactttt ttcagtttca ttgaggtata tttgaaaaaa    445560
     ttttttaaaa gtatatggtt gaggaacaat gcaccacttg atgctttgtc ttaggccatt    445620
     ctcacattgc tataaagaaa tgcctgagac taatttgaaa aaaagaaaaa gatgtttaat    445680
     tggctcatga ttctgcaggc tctataggaa atgtagttgt ttattcttct ggggagactc    445740
     aggaaagtta gaatcatggc agaagacaaa gaggagcagg gcaatcacat ggccagagca    445800
     ggagccacag gaggcgtgca gcggggggcg gggcgggtct acacaccgtt agacaccaga    445860
     tcttgtgaga actcatgcac tgtcatgaga acagcagcag gagttgttgc taaaccattc    445920
     atgaaagacc taccccatga cccagtcatc tcccaacagg tcctactttc aggaatgagg    445980
     attataatat aacatgagat ttgggtcaag atacggatcc aaaccatatc agatacacat    446040
     tgtaaaatga tcatcgtagt caaggtaatt tgtgtgtata tcatctcaca gagctacaat    446100
     tttatttttt taaatttact gcatatgtgt gtgtgtctga tgagaacatc taaaatctac    446160
     ccttttagca aaaatcgttt ttacagtaaa gtattaacta taggaacatt gctgtacatt    446220
     agatttccag aactcattca ccctgcacaa ctgaaactct gtaccctttg atcaacatca    446280
     cccaaattcc ctctcctccc aggtcctgac accctctagt ctactctctt cttttaagac    446340
     cttggatatt ttagatccca cacgtaaatg agatcatgaa gcatttgtct ttctgcatct    446400
     ggcttattcc actcagcatc atgtcctcta ggcccatcca tgttattgca aatgtcagaa    446460
     tttccttctt ttcaaagcgt cataaattta gttttatgta tacacatttt atttatacat    446520
     ttatcaatgt atgaccctta aattatttta caaatctaca ctattattaa taatcttgca    446580
     atgaacatgt cttttgcata ataattttat ttccttttaa tatataacaa gaagtggaat    446640
     cactagatca taagatagtt ttattttcaa tttgttaagt aaccaatcct accacatcat    446700
     ataaggattc ctttttctcc acattgttgc caacattttt aaagttttgt cttgctgata    446760
     atagccattt taactgcttt gaagtgatat atcattgtgc tttttatttg aattcccctg    446820
     ataattagga atattgataa acttttcatt ttccgtttgc catgtgtgtg tcttctgaaa    446880
     aaaaaatcta tccagaattt tgcctttttt aatcgggtca cttgttattt gttattgagt    446940
     tgtatggctt atttatatat tttatttgaa cttcttgtca gatatataat tacacatagt    447000
     ttttcctgtg ttttggtatt aaattcaaat aaatcagttc tgaatcaatg acaggaagtt    447060
     ttttcttttt ccatgttgtc tatgagttta tggtttcagg tattatgttc atttttagtt    447120
     gatttttata tcatgttaga ggaggcctaa ttttatttct ttttatatgg atgcccagtt    447180
     tttacaccat tattgaaaag actgtccttt ctctactgtg tgttcttggc acactccatc    447240
     aggaagagac ataatgaaaa aagtatataa gatgaatatt cctgatgaac atagatctaa    447300
     aagtcctcaa caaaatacta gcaaatagaa tccagaagca ctttaaaatg taatacatca    447360
     tgatcaaatg ggatttaccc tgggatgcaa ggttcattca acatccacaa atcagtaact    447420
     gtgattcgcc atgtgagcag aataaaagca aaaaacatag gattatctca atagatcctg    447480
     caaaagcatt tgataggatc caacatccac tcgtcataaa aaccttcaac agactaggca    447540
     tcaaaacata cctcaaaata ataagagcca tctgtgacaa actcagtaaa catcatgcta    447600
     aatgagcaaa agctcaaaca ccttgagaac tggaacaaga gaaggatact gtctctcact    447660
     actcctattc aatataacac tataaatcct agttagaaca atcaggcagg agcaaaaatg    447720
     aaaggcagct gatgtggttt tgatttgtgt ccccacccaa atctcatgtc aaactgtaat    447780
     ccccagtgtt ggaggtgggg cctggtgaaa ggtgattcga tcatggggtt gtgttttcct    447840
     ctctcacgct gttctggtga cagagctctc aggagatctg gtttctaagt gtgtggcacc    447900
     tcccccttgt ctcttcctcc tgctcctgcc atgtaagaca tgatgcttcc ctttctgtca    447960
     tgattgtcag gatcctgatg cctccccagc catgcttcct gtacaactcg cagaaccatg    448020
     agcaattaaa actcttttct ttataaatta cctagtctca ggtgtttata gtctgagaat    448080
     ggactaacac agcatccaaa caggaaaata agtcaatcca tctgtcttca ctgatgatac    448140
     aattctatgc ctagaaaatc ccccaaagta attctagaat agataaataa ctttagtaaa    448200
     gtgtaaggat gcaaaatcaa tgtacaaaaa tcaccagcat ttctatatcc caaccacatc    448260
     catgctgaga ataaaatcaa gaacacagtc ctatcccatg tacaatatct tcaaagaaaa    448320
     tgacatgctt aggaatgcag gtaacaaaca agatgaaaga tctctgcaag gagaacaaca    448380
     aaacactgca gaaataaatc ataaatgaca caaataaatg gaaaaaaaca tttcatgctc    448440
     ctggattgga ggaatcaata ttgccaaaat ggtcatactg cccaaagtaa tttgcagatt    448500
     cgatgttatt tccatcaaat tattaccatc attcttcaca gaattaaaaa acccatacat    448560
     attctaaaat atatatggaa ccaagaaaga ccctgaatag ccaaagcaat cctaaacaaa    448620
     aagatcaatg ccagaggcat catgctacca gatttaacat tacaaagtaa gaaaaacagc    448680
     ttggtactgg tgcaagagca gacacacaga aaaaaaaaaa gaacaaaaca gaaaacagaa    448740
     atagagctac acacctaaac catttgatat ttgataaggt tgacaagaat caagccatag    448800
     attaaaaaaa aaaactccgt attcagtaaa tagtgctggg agacctggat tcgggcagat    448860
     atgcagaatg caacagtgaa ggaggctggg cagtttctac cttgatttca gatgtgcaga    448920
     gagccctatg tgcccaggaa aaagcttgct gcaggagtgg agccaccaca gagagcctct    448980
     actagggcag tgccaaagat gtggaaagat ggggttggag cccccattca aagtccacac    449040
     tggggcacta catagtggaa ctgtgggaat gtggccacag atctcaagac tgcagaatgg    449100
     tagatccatg ggcaacttgc accctcagcc tagaaaagct tacagcactt gactctgact    449160
     catgaatgga gccccaagga ctgcgcccag caaaactaca gtgatgggct gccggaggtt    449220
     ttggggagcc aacacttgtc ccactgtgcc ctggttgaat tacataaatt tgagagaggt    449280
     tattctgtaa ctttaagatt taatatctgc tctactgggt tatatgtgtg tgtggggggg    449340
     gggagcgctg ttggccattc ctttggccaa tttatctatt ttggattgga ataatttacc    449400
     caatgcctgt accataattg taccttggaa tacacaacat ttgcaatgtt tttttttttt    449460
     aattttgcaa gctcacagat ggagggaaat tgccttaaga ctcagatgag actttagaca    449520
     tttgagttgg aggctgacca agttaagact ttttgttatt ttagtattat tatactttaa    449580
     gttttagggt acatgtgcac aatgtgcagg tttgttacat atttatacat gtgccatgtt    449640
     ggtgtgctgc acccattaac tcatcattta gcattaggta tatctcttaa tgctatccct    449700
     cccccatccc cccaccccac aacagtccct ggtgtgtgat gttcccattc ctgtgtccat    449760
     gtgttctcat tgttcaattc ccacctatga gtgagaacac gcggtgtttg gtttgttgtc    449820
     cttgcgatag tttgctgaga atgatggttt ccagcttcat ccatgtccct agaaatgaca    449880
     tgaactcatc attttttatg gctgcatagt attccatggt gtatatgtgc cacattttct    449940
     caatccagtc tatcattgtt tgacatttgg gttggttcca agtctttgct attgtgaata    450000
     gtgccacaat aaacatacgt gtgcatgtgt ctttatagca gcatgattta taatcctttg    450060
     ggtatatacc cagtaatggg atggctgggt caaatggtat ttctagttct agatccctga    450120
     ggaatcgcca cactcccttc cacaatggtt gaactagttt acagtcccac caacagtgta    450180
     aaagtgttcc tatttctcca catcctctcc agcacctgtt gcttcctgac tttttaatga    450240
     tcgccattct aactggtgtg agatggtatc tcattgtcgt tttgatttgc atttctctga    450300
     tggccagtga tgatgagcat tttttcatgc gtcttttggc tgcataaatg tcttcttttg    450360
     agaagtgtct gttcatgtcc tttgcccact ttttgatggg gttatttgtt tttttcttgt    450420
     aaatttgttt gagttcattg tagattctgg atattagcca tttgtcagat gagtaggttg    450480
     caaaaatttt gtcccattct gtaggttgcc tgttcactct gatggtaatt tctttcactc    450540
     tgcagaagct ctttagttta attagatccc atttgtcaat tttcgctttt gttgccattg    450600
     cttttggtgt tttagacatg aagtccttgc ccatgcctat gtcctgaatg gtattgccta    450660
     ggttttcttc tagggttttt atggttttag gtctaacatt taagtcttta atccatcttg    450720
     aattaatttt tgtataaggt gtaaggaagg gatccagttt cacctttcta catatggcta    450780
     gccagttttc ccagcaccat ttattaaata gggaatcctt tccccattgc ttgtttttgt    450840
     caggtttgtc aaagatcaga tagttataga tatgcggcat tatttctgag ggttctgttc    450900
     tgttccattg gtctatatct ctgttttggt accagtacca tgctgttttg gttactgtag    450960
     ccttgtagta tagtttgaag tcaggtagca tgatgcctcc agctttgttc ttttggctta    451020
     ggattgactt ggcaatgcag gctctttttt ggttccatac gaactttaaa gtagtttttt    451080
     ccaattctgt gaagaaagcc attggtagct tgatggggat ggcattgaat ctgtaaatta    451140
     ccttgggcag tatggccatt ttcacgatat tgattcttcc tacccacgag catgcaatgt    451200
     tcttccattt gtttgtatcc tctttaattt cattgagcag tcgtttgtag ttctccttga    451260
     agagatcctt cacatccctt gtaaattgga ttcctaggta ttttattctc tttgaagcaa    451320
     ttgtgaatgg gagttcactc atgatttggc tctctgtttg tctgttattg gtgtataaga    451380
     acgcttgtga tttttgcata ttgattttgt atcccgagac tttgctgaag ttgcttatca    451440
     gcttaaggag attttgggct gagataatgg ggttttctag atatacaatc atgtcatctc    451500
     caaacaggaa caatttgact tcctcttttc ctaattaaat atcctttatt tccttctcct    451560
     gcctgattgc cctggccaga acttccaaca ctatgttgaa taggagtggt gagagagggc    451620
     atccctgtct tgtgccagtt ttcaaaggga atacttccag tttttgtcca ttcagtatga    451680
     tattggctgt ggctttgtca tagacagctc ttattatgtt gagatacatc ccatcaatac    451740
     ctaatttatt gagagttttt agcatgaagt gttgttgaat tttgtcaaag gcgttttctg    451800
     catgtattga gataatcatg tggtttttgt ctttggttct gtttatatgc tggattacat    451860
     ttattgattt tcgtatgttg aaccagcctt gcatcccagg gatgaagccc acttgatcgt    451920
     ggtggataag ctttttgatg tgctgctgga ttcggtttgc cagtatttta ttgaggattt    451980
     ttgcatcgat gttcatcaag gatattggtc taaaattctc tttttttgtt ttgtctctgc    452040
     caggctttgg tatcaggatg atgctggcct cataaaatga gttagggagg attccctctt    452100
     tttctattga ttggaatagt ttcagaagga atggtaccaa ctcctccttg tacctctggt    452160
     agaattcagc tgtgaatccg tctggtcctg gacttttttg gtttggtaag ctattaatta    452220
     ttgccccaat ttcagagcct gttattggtc tattcagaga ttcagcttct tcctggttta    452280
     gtcttgggag agtatatgtg tccaggaatt tatccatatc ttctagattt tctagtttat    452340
     ttgcgtagag gtgtttatag tattctctga tggtagtttg tatttctctg ggatcggtgg    452400
     tgataccccc tttatcattt tttattgcat ctatttgatt cttctctctt ttcttcttta    452460
     ttagtcttgc tagtggtcta tcaattttgt tggtcttttc aaaaaaccag ctcctggatt    452520
     cactgatttt ttgaagggtt ttttgtgtct ctatttcctt cagttctgct ctgatcttag    452580
     ttatttcttg ccttctgcta gcttttgaat gtttgctctt gcttccctac tgaaaaaaga    452640
     tgatcatatt ttgcaatgca aggacatgag aattttgggc tcgagagtgc aatgatatag    452700
     tttagatgtt ttcccttcca aatgtcatgg tgaaatgtga ttctcaatgt tggaggtggg    452760
     accgactggg aggttttggg tcatggggaa agatccttca ggaatggctt gggaaccacc    452820
     ccatggcact tagtgaattc ttgctgtgtt agctactatg agatctgatt gttaaaaaga    452880
     gtctggcaac ccttcttgcc actcatgtcc cagctctcac catgtgacat agcctgttcc    452940
     ccctttgcct tccatcatga ttgtaaagca gatcctggtg ccatgcttct cacacagcct    453000
     tcagaactgt aagccaaatg tgcctctttt ctttgtaaat cacttggcct caggtattaa    453060
     tttataggaa tgcaaaagag actaacacac cgtccaaagc attacacaga ttcaactcta    453120
     tttttatcaa atgaccaata taattgatta catatttaga aaaaaatact aaaattccta    453180
     cagaatcaaa aaagagtctg aatagcaaaa gcaatcctaa gcaaaaagac cgaagctgga    453240
     agcaccacat tctctgacct caaattatac tacatgaata taataagaaa gacagcatgc    453300
     tactagtaga aaaaaatagc ccagaaagaa agccaaatat ctaaaaccaa ctgttgtttg    453360
     acaaaactga caaaaatata cactggagaa acaaccctct attcaataag tggtgccggg    453420
     aaaattaggt ggcttatgtg gaagaataga acgagacttc catatcaccg tagacacaaa    453480
     ttaactgaat atggattcag tgtttaaatt tataaactaa aactataaaa atacttgaat    453540
     aaaatctaaa aagagtcctc tggacattgg tctaagcaaa caatatacga ctaagacttc    453600
     aaaagcaaat gcaataaaaa cagaagtaga caaacaggat ttagttgaac taaaggtctt    453660
     ctgcacagca aaagaaataa ccaacatggt gaccagacaa tctgcaaatg gaaaaaatat    453720
     ctggaatcta ttcatcttgc aaagggctaa tatatagaat ctacaagtaa ctcaaataag    453780
     tcaactaaaa attacaaata acttcattaa agaatagaca taaacagaca tttatcaaaa    453840
     gaatacacag aagtggccaa cacaaataag aaaatatact cagcatcacc aatcatctga    453900
     taaatgtaaa ttagaaacaa catgatacgg catcttccac cagtcagaat ggctgttatt    453960
     acaaataaaa acagcaggtt ttgcagacaa acataggaaa aataatgatt tatatatgct    454020
     tggtgagaat gtaaattagt acaacctcca tgaaaaacaa catagaaatt tctcaaagaa    454080
     ctaaaattag aattaccatt tcttccagta agctgtccca gtaggcatgt tcctcccaaa    454140
     cttttatatc agagaatgtt gcctgcactc atatgtttat ttcaacacca ttttcaatag    454200
     aaaagtcaaa taatctaagt gtcaatcagt ggatgattag ataaaatatg atatatgtaa    454260
     atcatggaat actatgcagc cagtatggta tgaattcagt gtgaccagcc cctggacaag    454320
     ggcttgagtg gatgggatgg atcatcacct acactgggaa cccaacatat accaacggct    454380
     tcacaggacg gtttctattc tccatggaca cctctgtcag catggcgtat ctgcagatca    454440
     gcagcctaaa ggctgaggac acggccgtgt atgactgtat gagagacaca gtgtggaaac    454500
     ccacatcccg agggagtcag aaaccccgag ggaggaggca gctgtgctga gctgaggcag    454560
     tggtgcagca gtttctttaa cttccatatg atctcatttt gcatcatctt ctacttttat    454620
     attagctaag aacttggggt agacaggtgc tcctaagaga tccttaactt gcccattttg    454680
     atgggttttc cagaagacgt gagaagccac tttgttagca aagcatccca aatccatgcc    454740
     ctgttctaga tacatgtgag cccatttcct ggtctttgct taactgacaa gctctcatca    454800
     gtgcacctgg gctaatttca catcaggtag aggaacgcgt tataaaggaa agctaatgtt    454860
     gtaatagcaa ttcctgctta aaaaccttca gcttcattgt ttttgtgtaa tccatcagca    454920
     aattatgtta gttcaaggtt ctcaatggga gtttctaata aatagaaagg atgtatagag    454980
     cttcccctaa ttaaaatgaa acaattgtga acacaacctc ggtattcagc catgtctcca    455040
     cccttcacac ccttcgccac aaaggaattt tcacctctcc tggaagctgg gttcattttc    455100
     aaattagtta tttttttcaa tgtaatatct caagattatc gtatatgact attttagcag    455160
     aaagtgaatt atgggaactt gaactaaaca actgaaaaca aattcacaac taattaaaca    455220
     agatgccaga atgtgattgg ctccaggctt tgtaattcag cagttcatgt acccagactg    455280
     gaaatttaca tgtcttcttg ttaccttcac agcacagtca actcccatta tgtaagaaat    455340
     ggtgactgca ttcccaaggg ttatgcatag atatgaaaat agactgggta aggtgaggag    455400
     ttgattgttt aaattcccct ctgaagaagc agcatcaact caacaaacca cctctcttca    455460
     ctctgtgact agagctatgt cacaggccac atggacctaa atccttgatg gatataacat    455520
     gactacataa attgggctga tcatttttat gctataaaat taatagatga cactgcactc    455580
     cagcctgcac aaccaagcaa gtcttcatct gtaaaatcta aaaaagaaaa attagtaggt    455640
     actgacttcg aaatttttga taataatatt ttcaccaccc aaatttaatc acacccacat    455700
     gttacctgca tcttcactga aaagttccca gtcacgatga gttccttcaa tgctccatgt    455760
     gttcaaatct ggacatcaag agagtccaga gaataaaaca caatgacggc agtgaaactg    455820
     atatatattc agcacctctt aactcaggag gactccatac accctggcac acagctgctt    455880
     ttctaaatgg ctcacaatga ctccagctca ctcacagagc tcagacagaa acctcccttc    455940
     agggtgggag ctgggtggca gggggcactc agtacccgca gaggtgaaaa tgagctttca    456000
     gatggaactt ccctgtcacc tcaacatgga atttattgtt tcatttcatt acctctcttt    456060
     ccataatggt tcatttcttt tggcctgttc attactgata tttttcagag caatctcact    456120
     tgaatcttta ctcttttgca ttttgtctcc ttgacaatgt tgggaagttt tacctccagc    456180
     atcataacat gatctagtga tctgacacat tgtgcaaaca atacctacaa attcagaacc    456240
     tctttgtttt tctttccaca aaatataatt ctttctgttc tgtgtatgag catgtcttag    456300
     caaccctgta cacacccaca tagatgtcta caagcctatg aattgttctc tgtaaataaa    456360
     aatttatctc aaattccttc aatgttcata attctgagag tgaggaaggt ccttctcaat    456420
     ctgttcaaac aaaatgccca gagaccatcc agtaggtaag gagttcacct ggctctggtg    456480
     tggggtctgt ctctttccct ctgttgtccc acaggtcagc ccagttgttc acgtcctaac    456540
     aagaaagccc aggtttgtcc tgattttaaa acacatcaaa cttctgatga ctctcctgtt    456600
     acccacatcc atggagatag attatttatt atataattca gcaaactaat gtcaaatgcc    456660
     caagttgcaa taccgcacat cctagggtat gttcatgcaa ttcaatggag gagaaaatct    456720
     ttcagagaca gatggatctg aaatgataaa tatgtgggta aggactctgg gcttgagtat    456780
     cattgtccag ccatgtttca caagtgtgtc ctgtcaggga aggatcagag ttccttgtgc    456840
     tgtcagaggg aaggggtcac agagttcctc tctggttccc aggaaaggta atcgcactaa    456900
     tcttcatgat cttcatgaga ctatcctcca gtgctgacct gttacggagt ttttgtctga    456960
     agttctcact gcaatcccca atctacatat tttcaatcag aagtgtttag aggccaggac    457020
     acatcttcaa ggtcacacat tgagaaggat ggagatatgt cccactacct tctcctacga    457080
     tctcagacag aatcccaaat ttcaaaagga cacagaagga cagctctcag gtgcttttaa    457140
     aaaatgaccc acttccaggg acagtgagct tccctgtaac catggtggat gttctgaact    457200
     acaataaaca ttggatggat ccagtattgt ttgaagtcac tgtcattatt acattcagct    457260
     gttgtttcaa tgtgtctgaa agggtaaatg actatttaga tggcctgggt gtgtggttgg    457320
     ttttatatga atctttaagg gttgaacagt actgacccta ttccaaaatc tgtccttgat    457380
     ccaggatcac actcatctct cagaccagct ccttcagcac atctctttac ctggaagaag    457440
     aggactctgg gcttggagag gggaggcccc aagaagagaa ctgagttctc aaagggcaca    457500
     gccagcattc tcctcccagg gtgagctcaa aagactggcg cctctctcat cccttttcac    457560
     tgctccgtac aaacgcacca cccccatgca aatcctcact taggcgccca caggaagcca    457620
     ccacacattt ccttaaattc aggtccaact cataagggaa atgctttctg agagtcatgg    457680
     atctcatgtg caagaaaatg aagcacctgt ggttcttcct cctgctggtg gcggctccca    457740
     gatgtgagtg tttctaggat gcagacatgg agatatggga ggctgcctct gatcccaggg    457800
     ctcactgtgg gtttttctgt tcacaggggt cctgtcccag ctgcagctgc aggagtcggg    457860
     cccaggactg gtgaagcctt cggagaccct gtccctcacc tgcactgtct ctggtggctc    457920
     catcagcagt agtagttact actggggctg gatccgccag cccccaggga aggggctgga    457980
     gtggattggg agtatctatt atagtgggag cacctactac aacccgtccc tcaagagtcg    458040
     agtcaccata tccgtagaca cgtccaagaa ccagttctcc ctgaagctga gctctgtgac    458100
     cgccgcagac acggctgtgt attactgtgc gagacacaca gtgaggggag gtgagtgtga    458160
     gcccagacaa aaacctccct gcagggaggc tgagggggcg ggcgcaggtg cagctcaggg    458220
     ccagcagggg gcgtgcggag ctcacggaat acaaggccgg gtcaggagca ggtgcagggt    458280
     gagcggggct tgctcatctt ctcagagatc atcatctccc tcctcgccag cacctcagct    458340
     ttccgtagag gtcctctttc tttattgtgt gtggttctac ttcctcacat ccttgtgcca    458400
     ggaaagaaag gagtaaggca aattttcctg ttacaattga agtttcacca attactaaga    458460
     actttcctgc aagtacctgc acagcccatt ataccttatt tatatatgta tatattctaa    458520
     tgcttctcac catctcttga tttgtgtcat caatttaatt gtgccctttt tgaaattcat    458580
     atgctgaaac tttaaatcca atggatctat atcggaattt taatggtata attaatgtta    458640
     aatgtggtca taaatgagac cctaatgcaa tagagctgtt gtctttataa gaagaggaag    458700
     agacaccaga tacctctcac ttctcacatg cactcagaga agaggccacg tggagacata    458760
     gtgcactaga aggtgggcct ctgcaagcca ggaagaagcc gcaccaagaa ccaaccctgc    458820
     cagcaccttg atcttctaca ttcagactgc agaattgtaa gaaaatcaat atttgttgtt    458880
     taagccaccc actccttttg tcttcttacg aagacccaga caggctaata ccacacaact    458940
     ctgttagctc catctcctgg agggagaagc agccccctga agctgggcac atcgctcaga    459000
     ttttcacatg aattaggcaa aaacagtagc tctcatataa aaactgtcac gtccctgttg    459060
     ggacaaggtc ttttaaacaa gccctggggc tttgtcacaa atgttgcatt ttatccttta    459120
     ttaggactta attaattgac aatgagtacc agctggatgg aaactgacca ctgaccatct    459180
     tctgctgtct ccttattata tcacagaaaa ccacagcaac attactctat gtcttcaact    459240
     ttctaaattt gtactgaatc tattgctaaa tgaggagcta catggggtct gagttttgtt    459300
     accttcttcc cagtcttccc caattaccaa gcatagaaga tactttcagt gaaatttagc    459360
     tgtcaatgcc cccaacacca catcatgttt taaggtccaa ggactttctt tggggggcta    459420
     ttgaaaaaca cttttgaatg gaaaatccta aagcatacaa cagctgaaag aatggcccct    459480
     gtgcacatga aggctgaagg ggtggatgat agggtacgtt cctccaaggt gttcctgggc    459540
     atgtgatggt tggatacctc atgcatacga aaacaaggac tgaactgaga tagaaacaag    459600
     ggccatatcc tataggaaaa caagaaaaaa aggggcatcc ctgaagagag ttaattagac    459660
     ttggtgggtt taagatgaaa ttttttccaa agttttttaa tgttctaagc taaatatgaa    459720
     tattttcaat aaactgcaaa ttaaaaggag aatgaattta gaagtttaga cagaaaaaag    459780
     ccatgagggt gtagaaagca aatccacaaa aaactgtcac atggcagagg ccagaatgga    459840
     gctgatgcag ctacatcatt attctgcaga cctagttgag accctttggt gtttaaggca    459900
     ggtccagcag tgaagaactc ctttagtttt tgtctgggaa agttctaatc taatcttcat    459960
     ttctgaaaag gatttacatg gtaaagattc gtcttggcag ggttttattt atttttagtc    460020
     ccccccaacc cacgtcaatt tgagtaaagt catgtgttac gtaacaatgc tgcagtcaac    460080
     agtagaccac atatatgttt gtggccacat aatgttgtaa taccatattt tcactaaact    460140
     ttttccatgt tttgatgtgt ttagatagag agatacttac catggtgttt ccatttcctg    460200
     cagtgttcag tgcagtaaca cgctgtacag gttagttgtc tgggaaccct gcgctaatcc    460260
     acataccctg tgtgtagtag ggtattcctt gtaggtttgt gaaaattcac ccttgatgct    460320
     tgcacaatga tgaaattgcc cagggacaca tttctcaaaa agaacctctg tcaataaacc    460380
     acacaccatt gaatacaatc cctgtgcctt ctgaactgaa aaccttctgc tgagaaatct    460440
     gttccttttc tttctgtttt ctaaagcctc ttttatttcc agcaatgtgg ttttaaactg    460500
     tgtcacattt tttgtcattg ggtttatctt acgtaaaatt tatagagctt cttcattgtg    460560
     gatgaatatt tgcttccctt ctccaaattt ggaaatattt tttgctatta ttttgaataa    460620
     tttatctctt ctctctctct ctctctctct ctctctctct ctctctctct ctccctctct    460680
     ctctctctct ctctctctct ctctctctct ctctctcccc ctctctccct tcactacccc    460740
     ttttgtcttg tctttgtata actcctcaaa tgcgtagttg agtgtttagt gtctttcctg    460800
     gcccataagt cttgaactct tttttatttc tttcacttaa atttttcttc tctgtctcta    460860
     ctatttcaaa tgatctttct ttgagtttac tgtttcttct attacttgat ctagtttggt    460920
     ctaaaatctt gttaaaattt ttggctgtat tcttcaggat caatattttt gtttagtcat    460980
     cttttaaatg ttttatcttt attaaaattc tcacattgtt cttgcatgct gccactagct    461040
     cattgaacat gtttctaaca tttactttaa agtttctgtc aggtaatcca tgttcttcct    461100
     tttcattgat gtcagtttat ggaccttttg gttgttcttc tgtttggacc atgttcccct    461160
     gcttcttcat tttccttgac tgtctttgtt gttatctgtt ctttataaca aaagccatca    461220
     tatacttctt caaagactag gctcatagag atcaccctca ccaatcagcc cagacagtaa    461280
     ttgtgggtct ctcaaaactt gataggattc aaacttctgt ctgttcttag tggcccctag    461340
     gttttagagg atgatagatt gtgccaggac tcacaggtaa gggaggtaga aactatgtac    461400
     tcatgaccat ggggtctgtg ctgatctgag cttctcttgt catcactctc aatattcaac    461460
     ttccccgtgg atcaggctca gctgtggctg ctccacgtgg ggctgttctc agtctgttgc    461520
     ctctgtgtgt gcagaagtcc tctgtgaagt taactagtgg agtcagacag gaaaatacta    461580
     cagaccaaga attctcagac tgttctgcaa agcccctgga ttcactgaaa aaagaacaag    461640
     tttggtccag caggattcat ggcaagtgtt ggtgagggag ataacagtaa ttcaagtgga    461700
     agttctcaat gggactcgcc ttcagtacaa agaagattaa cagtcctcag agacactgtt    461760
     cagaagattc tcttttaaga taataaaact gagagcccaa gacaagtctg tgtattactg    461820
     tgagggacac agtgtgggga catctatgtg agcacagaca caaaagtccc tgcagggaga    461880
     caggagggga ctgcatgtta gttgctgctc agaaccacca gggggcattc aggacaccag    461940
     taggcactca gaaccaccaa ggagagctca gaatcaccag ggggcactca ggaaactggc    462000
     aggtgctcag aaccaccaag gggcgctcaa gatcccagcg ggagctcata accaccaggg    462060
     ggcgctcagg acactagggg gctcagaacc actagggggc gcccaggacc accaggtctc    462120
     tcaagacagg agggcgcact caggacacta gggggcgctc agaaccatca gggggcaatc    462180
     aggacaccag agggacttca gaaccaccag ggggcgctga gaaccaccag ggggtgctca    462240
     ggacatgacc ggccctcagg accaccaggg gatgttcaga acaccaggaa gcactcagga    462300
     caccagagag cgatcagaac accagggggc actcagatcc tccagggggc actcaggaca    462360
     acagggagct ctcaggacac cagggtgagc tcagtaaacc agtgttcgct cagaaccacc    462420
     agggagcact gaggacacca ccgctccctt aggaggcagc tccacatcag gtccctggat    462480
     ccgggcaggg agggcggttc cttttggatc ttgccactaa ccttttggga gttttcctgc    462540
     ttcctttgtg gtttcaagaa tcatttgcag attcttctca tgtacaaagc tctgctttct    462600
     tggattatgt aatgtttttg gctttggatg ctaccagaat tacgttgtac tgtgagagga    462660
     ttcattcgtg gtgtgtgcaa tagtgaatga aagctccaat gttaggggtg gctttgaaag    462720
     ctacgttagg ggtggctgag ggcagttagc agaaaatgat catcactata gaaggctact    462780
     catttctttg cacatttgca taaacaattg tagtttatgc cctaaaaact gcatgttttc    462840
     ttggcccttt ttcttaaatg gctccactct aagtccagta atctaattaa gctgtgtttc    462900
     aaagaccacc aatcaagtta attctgttta atgcaacact ttgtaaagaa aatgtacatc    462960
     tgttttttag agtcagcttt aaattttaca ttactttaca aatattgatt tgtcaaattt    463020
     agtctcataa ctatcttcag taatttaaaa tcttagtcat gtcatgttaa attaagtaat    463080
     cctaggtttc tcacttgaat tagggttact aagaattaga atagtaaaag agtataatta    463140
     gcttttggtg aggtttataa agaaagatga ggattttttt ttttttttgc ttaaaaatat    463200
     tctgttttcc agtttacagg gcttttcaac tggttttaag ataaccactg tttacatcta    463260
     aacttttttt tgtaaatgac tgctgagttt ctatacatat tccatagcta gagtttcaaa    463320
     gtaaaagctc tagtatcttt gtattagtgt gtgtatgcgt agatgtgtta atatgtgcat    463380
     gtatatatat gtgtgtgtgt gtgtgtgtgt gtgtgtgtgt gtgttgtggc tactaggtac    463440
     aaaattggct ttgaaataaa tattttcaaa ttaagtaaat gagcccaatg catttgaagt    463500
     acatgtgact taaataaata tttaataaat aagttggctt taaaattatt ggtaaaaaca    463560
     aattagagat attttaacaa ttatcagcat accttatagt ttatattaat tcatcaagtg    463620
     attttatatt taaaatcaca gctagatatt atatggtgtc aaaatttttt atgatgatta    463680
     taaaattatt aacctcgttg gccatatgcg gtggctcaca cctgtaatcc cagctctttg    463740
     gaaggctgag gtgggtggat cacgaggtga ggagattgag accatcctgg ctaacatggt    463800
     gaaaccccat ctctactaaa aatacaaaaa gaaaaaaaaa attagctggg catgctggca    463860
     ggcgcctgta atcccagcta ctctggaggc tgaggcagga gaatgcttga accccagagg    463920
     tggagcttgc agtgagctga gatcgcacca ctgtactgca gcctggatga cagagcgaga    463980
     ctccgtctca aaaaaaaaaa ttataaacct cattaaaacc agaatgatct ttgtaatttt    464040
     ttgacaaata tgatgtttaa tattgttgtt ttaataaaaa acaggtaaat agttattaga    464100
     aaaacaatta tttgcttaat tataaggttt ttactcaggc aagcacctga aattcatggg    464160
     ttataacatg gttaacagag aaaaaacttt aaaggatgaa ttacaatttt cataagtaat    464220
     ctagataagc tacttaaata aaatagggaa atgtaatcaa ataaaccttt taaataaact    464280
     tgttctacaa tttaaaaatc taaagtttaa ttaaataata gatattgact aaatgtttag    464340
     gtcattacta attgttttaa aaaatatata agaaaatatt tttcttaaaa aattattctt    464400
     agaaaaataa tgtaattcaa agttatttat ataacatcat aaaaagtaag agagttttaa    464460
     agaaatttat tgacatagag aagtactttt ggtaagaaag gttaaaataa aaaaatttta    464520
     tatgagaaat aatcttgcat ggtaattttt tatcctaaaa taaaatgact aagaaagaat    464580
     gacgtttaga ataaaacaag atgttcaact atgccataaa tggtttgtgt aagtgaaaat    464640
     aagatttata aaaagttaat ttattaaaaa accttcatat tatcaagttg actataattg    464700
     aaagggaagt atttataata gtctttatag agatctggct tttatataaa atatagtaat    464760
     acactgaaga ctggttagaa tgacaaaatt gtcttaaagt gttgatttac taaataaaat    464820
     tataagatat tttaattttt taacccccaa atttaacttt tattgcatct tgccattttg    464880
     attttttccc tttgagaaga cttgagaggc tctccatttt ttcatcacgt cctttaacat    464940
     tttttttctt acagcgatta gcttcttact gacttaactt ctaactgttg ttagctttta    465000
     actgctatta ttgtctgatg ctaaaaacat tttatcttta agttctaaat aaaatgtttt    465060
     ctttcaatat aacattctac actcttggct tatctttaaa tgtctacatt tttctatgaa    465120
     accaaagtct tcacgtgtaa tctgagtcac attcttggta tgtctaatta attcaaatgc    465180
     ttttttcatt agagttgacc tgcaggttat ctacatgcat tttccaatag ggaaaagcaa    465240
     tcacactgta aaaggttttg ctttgctatt tggtaactgg cataaaacaa attttatatt    465300
     ttattgaaat aattcctatg ttactgttat taaggtttcc aacttactga gattttaaga    465360
     aaatataaat taatgttatt acattcatgt agctatctac attactttta aaggccttgt    465420
     gctgccacat tactgacctt tgactcctag gtctaaaaag gacacacagc attttgggag    465480
     actaaggcag gcagatcacc tgaggtcagg agtttgagac cagcctggcc aacatggcga    465540
     aaccctgtct ttataagaaa tacaaaaatt acccaggcat ggtggtggat gcctgtaatc    465600
     ccagctactt ggaagcctga ggcagggaga attgcttgaa cctgggagac agagattgca    465660
     gtgagccaag attgtgccac tgaactccag cctgggtgat agagcaatat tccatctcag    465720
     aaaaaatata taaataagag aaaaaggaca ctaagtcctg ctaaatctta aacactgaca    465780
     gtaattaaag ccccatctac agacctggaa gaaaatgaca ataaaaattc atcatactct    465840
     taagacataa ggccagaaat tgaaactact caacaactcc aggcccaggg actattacag    465900
     aagtggattt gtaagattgt aaaagctaat tttgagatat aatattactt gagagtttct    465960
     ttataagtta agcattaata ttaaatgcac actaatggaa gtctagcatc tgggcccctg    466020
     tgacagattg acaagggttt cttgaagaag taatccactc tttagcttaa aaaattatta    466080
     aaatttataa aagacctatg aaaattatat tttatggtaa aagtagttat aatttaatag    466140
     acttatttct cagaattgag agatagtttt aatttttctc atgatgttct tataaggggt    466200
     tattgtttag aaaattaatt ctttcaaaaa taaaagtttt tgctttttta agaaatcact    466260
     gagttttcac ttggctaaat aaataactta ctttacaata atctgtaatt ccattttgta    466320
     atatcaagtg ttctaaataa acctttgata tttgacctcc ttcacaaaat aaaagtctaa    466380
     attcagtcat ttgacctcat tattcttttt tgatatttga tctcctgaag ccaaaaagaa    466440
     acatatttag cttctttggt acaattaaat tatactggaa gcaatgtcaa atttgaaatg    466500
     gtgtttaact ttggactata tttatataaa tgtggactat ataattggag tatattcata    466560
     taaattaaag agtacatgtt ctaaaatggt atgaggttca attgactttg atatgtcagt    466620
     atattttgtc agtagtactt atgattatta tataaaattt ttgttcatca cagaaataac    466680
     caaactttct cctcaattct gtctttaacc gtggctattc taaaacttca gccatccaca    466740
     attgttttcc tttgattctt tatcaggtgg cttataataa tctatagaat tttgaggggt    466800
     actcttaaat acacaattgt gacaatttta taaattgtgc catttatata cagagaaaaa    466860
     cttccaagac tctcatggag acctgatgca tttatgatgg ttgttaatat aatgtcaaac    466920
     aggacaggac ttaattgcat gaactgaact aacaggagcc tgaaataaat tttatggctt    466980
     attctttaaa gcatttgcta attacttaca ttttgttttt cagaatcaaa aaaaaaattc    467040
     tcttaagcta ttcattgttt ttaacaattg agtatagtat attctagtga gaaaattcaa    467100
     aaacacaatt tcttcttctc tacataattt ctccaaaatt tggaaactgt gagtattgtt    467160
     atatcaaaat attaattcac ataggttcaa tataaatctg ctttcttccg taacagggca    467220
     caattggaga caatgatcat tttaccaaga ctttaacttg aatgacatat tttcatattt    467280
     actttataaa atgaatttaa gctggatagc tgataaaagc cccttgggaa aactggcatg    467340
     taacctgttt tgttcagggc cctgagctgt agtaagtcaa taattttact ttctgacaga    467400
     cccaggaatc ccaagttgtc ttggaaaatt gaaaaaagaa gaagtaaccc aattcatata    467460
     gctgtctgaa ggcacagatg aaacaatgtc tgggcttgat gcttttaaag attctaccct    467520
     tagatttctt ataaaaaatt tccagcaagg tcaatttatg aattaaatag cctatgtaca    467580
     aaaaatagaa aaaaagagag ctaatatgtt aaattactat ttttgcctca tcttatacaa    467640
     aaaaattaag cctaaaactt atattacaaa taaatttgcc ctactatgat tgtgtctcta    467700
     ataactttgg ggaattatag agagaaatac tgtttcaaaa taaacaacag tgcatctgtt    467760
     attagattct aaccttgtcc acttgttttt caatttatat tattttctac aatttatatt    467820
     agtttctaca atttatatta gtttctataa aaatttatat ttgcagaagc ctccaaaata    467880
     tttttcctga tttaaaatta ccagaagtta aactgtgctt tcttatagct agacatctta    467940
     tactctaaag aaaataaaat caatgacatg atttggctct gtgtccccac cccaatctca    468000
     tcttggattg taataattcc cacatgtcaa gtggaaaaag gtgggaataa ttgaatcatg    468060
     gaggctgttt tctccatgct gttctcctgt tattctcaca agatctgatg gttgtataag    468120
     gggcctccct cttcccttgg cacttctctc ttgcagccat gtgaagaagc acgtgtttgc    468180
     taccccttcc atcatgattg taaatttctt gtggctttct cagccacgca gaactgtaag    468240
     acaattaaac cttttttctt tataatttac ccagcctcag gtatgtcctt atagcagcat    468300
     gaaaacggac aaatgtaagc aacttaatta tatacaaaaa ctcttttata cctgcctact    468360
     gtgaaaggaa aataagtctt gagaccgcaa aatcaccaag ctaaatggaa gagtcaaact    468420
     ggtgttacag gagatagaaa gaaattattt tggtagatag ttaggatgac agaatttctg    468480
     gcaaaaacat ttcttctaac aaaaatcagc ttggcaatta cttattttct attcacacag    468540
     agttcaaaga aatcacttct aacaaaaagc agcctgaaag atcaggctgt aaaacatata    468600
     agcaactctg ccacagagag ggtgtttctg ggtgtaatca ccaaacttca catacataca    468660
     ctgggcccta gtaaaaacag tgggccttaa tgagcacatt cctttccttt tctgggggca    468720
     ctataacatc agaaagctgg aaacttgcac aagtttagga tgcctgcact tgcaaggagg    468780
     tacctgggac ctagcatgga aaatcctctt ccccttttta gcactctggg agtggagccc    468840
     cagccttggg attcccaggt gtttcccttc agtgatcagg actgaacaca cacaactcat    468900
     catgcagttt gtgctgagct gggttttcct tgttggtatt ttaaaaggtg attcatggag    468960
     aactacagat gttgagtgtg agtggacatg agtgagcaaa acagtgggtt tgtgtggcag    469020
     tttctgacct tggtgtctct gtgtttgcag gtgtccagtg tgaggtgcag ctggtggagt    469080
     ctgggggagg cttggtacag cctagggggt ccctgagact ctcctgtgca gcctctggat    469140
     tcaccgtcag tagcaatgag atgagctgga tccgccaggc tccagggaag gggctggagt    469200
     gggtctcatc cattagtggt ggtagcacat actacgcaga ctccaggaag ggcagattca    469260
     ccatctccag agacaattcc aagaacacgc tgtatcttca aatgaacaac ctgagagctg    469320
     agggcacggc cgtgtattac tgtgccagat atacacagag gggaagtcat tgtgcgccca    469380
     gacacaaacc tccctgcagg aacgctgggg ggaaatcagc ggcagggggc gctcaggagc    469440
     cactgatcag agtcagtccc ggaggcaggt gcagatggag gctgatttcc tgtcaggatg    469500
     tgggacttca tcttcttcca acagtttctc taatgaacct ccctaatttt agaattctgt    469560
     ggttcctaat gtcatctcta catattttca aaagatcatt ttaatatgag gacataacct    469620
     ctcatgtacc aaatgcacat tgatgcttac aaagatgaaa agttctcaac cattgtcacc    469680
     aggatcgcag tcctgaggaa gctcacgggt gtctgatgag tctcctccat tcaggcccag    469740
     gacagaaacc tcagggagac tcctggacta gaacggcagg gattctgatc acagccaata    469800
     gagagcctgg gccagggtca gtgtcctgta gaagctcaca gggttcacat ctgacccttc    469860
     tcctgaccct aaagccaatc cgcatcagca ctgatctggt gctccttttg ctcccaatac    469920
     atgttctttc tttggagtgt ttgttctccc ttttttattt gcttttcttt cttcctgaaa    469980
     aagaaacaca tggtctctat gctctacact ccagggctca aggcattttc ttagaactca    470040
     ggcaaggctc aggcttggct actccagccc acgtgggaga ggctgatggg atttccttct    470100
     ctccccatat tctcagggcc ctcctctgtg ttgtgtgtag actcatctgg gaatgcaatt    470160
     ggccgttaat agtgaagggg ataaactcat ttgatcaaaa tgggatgtgg atgtggaatt    470220
     aaccctgttc tatgcacact gtcagagtca tcttcttcag aagtagtgtt agaaagagct    470280
     tgtgaacttt atcagaatca aaatggatcc acttgtgtta aaaccctaat gaatgaagct    470340
     ggggaaggcc atgaaggagg gttctaacac acatattcct gataagaaga actatcataa    470400
     atagaccctg cacagccaca accttttaca cagaggccac cacagcctta aaagctttat    470460
     ttctccaagt aaatctgccc cgcaactgcc tattcaacct tacaccgcta tctcaaaata    470520
     gctgctgtca ccctcctcat ttttccttaa ttctcttttt tattattttt taaattatac    470580
     tttaagttct agggtacatg tgcacaaagt gcaggtttgt tacatatgta tacatgtgcc    470640
     atgttggttt gctgccccca ttaactcatc atttacatta ggtatttctc ctaatgctgt    470700
     ccatccccct tcccccaacc ccactacagg ccccggtgtg tgatgtttcc caccctgtgt    470760
     ccacatgttc tccttaattc tttatcttcc ttttcctacc tgaatgtacc catacatatt    470820
     ttaattgaaa cgcacatcct agaaaaaata ttattatact ttagagtctc tgtctgttat    470880
     tcaggttgac atgacaagct acagacggac atgccacttt tctgtgagac atagaggatg    470940
     acagtttttg gagatgtcta ggaatatcca atgtccataa gatcagccat caataagtgc    471000
     agactggagg tcccagagag aggcgaagct gctgaatcat cgtggaattt cattttctcc    471060
     agttctgctc tgatggaatc aggcccaccc attttatcaa tgacaatcta cctaacctag    471120
     agtcagctga tgacagtatt aatatcatct attaagtaaa gtcataacta tatatatagt    471180
     gagagaggga gagagagaga gacggagtct cgctctgtca cccagggtgg agtgcaatgt    471240
     cgtgatcttg gctcactgca acctctgtct ccggagttcg agcagttctc ctgtctcagc    471300
     ctcctcccag tatctgggaa tacaggcaca caccaccatg tccggccaat ttttgtattt    471360
     ttagtagaga tggtgtttca ccacattggt caggctggtc tcaaactact gacctcaggt    471420
     gacccacctg ccttagcctc ccaaagtgct gggattacag gggcaggcca ccaagtccag    471480
     ctaatttttg tatttttagt agagaccagg tttcacgata ttggtcaggc tggtctcaaa    471540
     ctcctgatct cagcctccca aagtgctggg attaaagatg tgagctactg tgcccggcca    471600
     acaccaccta tattaatgtt ggattgaata actacaaagt ctaccctaac caagtgtacc    471660
     atcaaactta ccattaccca gaggaaagaa tctttaacat gagatctata ttcttaaatg    471720
     ttttaaggtg taaaactgac cacagtacaa tcacccaatt atgattttgc taatgagttg    471780
     tagaaactgc atgtatattt tggatattaa cactttttca gatgcacacc ttataattat    471840
     attctctcat tctgtaggtt gcaatttttt ccacactttg cggcatcttt ttgttgtgat    471900
     gtagttctgc ttgttcaatt ctgcttttat tatctgtgac tttaatttga aatatagaaa    471960
     atcatccttt tttatatact ttaaatttta gagtacatgt gcacaacatg caggtttgtt    472020
     acatatgtat acatgtgcca tgttggtgtg ctgcacccag taacttgtca tttaacacta    472080
     ggtatatctc ctaatgctat ccctctcccc tccccccaac ccacaacagg ccccggtgtg    472140
     tgatgttcca cttactgtgt ccatgtgttc tcattgttca attcccacct atgagtgaga    472200
     acatgcggtg tttggttttt tgtccttgtg atagacttct gagaatgatg atttccagct    472260
     tcatccatgt ccctacaaag ggcatgatct catcattttt tatggctgca tagtattcca    472320
     tggtgtatat gtgccacatt ttcctaatcc agtctatcat tgttggacat ttgggttggt    472380
     tccaagtctt tgctattgtg aatagtgccg caataaacat acgtgtgcat gtgtctttac    472440
     agcagcatga tttataatcc tttgggtata tacccagtaa tgggatggct gggtcaaatg    472500
     gtatttctag ttctagatcc ctgaggaatc gccacactgc cttccacaat agttgaacta    472560
     gtttacagtc ccaccaacag tgtaaaactg ttcctatttc tccacatcct ctccagcacc    472620
     tgttgtttcc tgacttttta atgattgcca ttctaactgg tgtgagatgg tatctcattg    472680
     ttgttttgat ttgcatttct ctgatgacca gtgatgatga gcattttttc acgtgtcttt    472740
     tggctgcata aatgtcttct tttgagaagt gcctgttcat atccttcgcc cactttttga    472800
     tggggttgtt tgcttttttc ttgtaaattt gtttgagttc attgtagatt ctggacatta    472860
     gccctttgtc agatgagtag attgcaaaaa ttttgtccta ttctgtaggt tgcctgttca    472920
     ctctgatggt agtttctttt gctgtgcaga agctctttgg ttcaacatac tcaaatcaat    472980
     aaacaaaatc cagcatataa acagaaccaa tgacaaaaat catatgatta tctcaataga    473040
     tgcagaaaag gcctttgaca aaattcaaca accttcatgc taaaaactct caaaaaatta    473100
     ggtattgatg ggacgtatct taaaataata agagctatct atgacaaacc cacagccaat    473160
     atcatactga atggacaaaa actggaagca ttccctttga aaactggcac aagaaagggt    473220
     tgccctctct caccactcct attcaacata gtgttggaag ttctggccag ggcaatgaga    473280
     cagtagaagg aaataaaggg tattcaatta ggaaaagaag aagtcatatt gtccctgttt    473340
     gcagatgaca tgattttata tctagaaaac cccattgtct cagcccaaaa tctccttaag    473400
     ctgataggca acttcagcat agtctcagga tacaaaatca atgtgcaaaa atcacaagca    473460
     ttcttacaca ccaatcacag acaaacagcc aaatcatgag tgaactccca ttcacaagtg    473520
     cttcaaagag aataaaatac ctaggaatcc aacttacaag ggatgtgaag gacctcttca    473580
     aggagaacta caaaccactg ctcaatgaaa taaaagagga taaaaacaaa tggaagaata    473640
     ttccatgctc atgggtagga agaatcaata tcgtgaaaat ggccatactg cccaaggtaa    473700
     tttatagatt caatgccatc cccatcaagc taccaatgac tttcttcaca gaattggaaa    473760
     aacctacttt aaagttcata tggaatcaaa aagagcccgt attgccaagt caatcctaag    473820
     ccaaaagaac aaagctggag gcatcacgct acctgatttc aaactatacc acaacgctac    473880
     agtaaccaaa acagcatggt accggtacca aaacagagat atagaccaat gaaacagaac    473940
     agagccctca gaaataatgc cgcctatcta caaccatatg atctttgaca aacctgacaa    474000
     aaacaataaa tggggaaagg attccctatt taataaatgg tgctgggaaa actggctagc    474060
     catatgtaga aagctgaaac tggatccctt ccttacacct tatacaaaaa ttaattcaag    474120
     atggattaaa gacttacgtg ttagacctaa aaccataaaa accctagaag aaaacctagg    474180
     caataccatt caggacatag gcatgggcaa ggacttcatg tctaaaacac gaaaatcaag    474240
     ggcaacaaaa gcccaaattg acaaatgcga tctaatatga aattcttaat gagttctata    474300
     gttataagta cattatttct ccttattaaa actaatcttt tttgctgaat gggtggaaga    474360
     tggtggcttc cctccaaaag aaaacatagt tggttctgta aagagttcac ccatttcttc    474420
     cactataggc ctgccctggc ataatacaag ctcccaactt aagaacttat gattttccat    474480
     ccatgggaga agccatcttt tccatctatt ataaaaacag aagacacttc attaatatca    474540
     ctcccaaaat aggctatgtc atatccagtg aggtaggatg gctgacagtg gtgagaatcc    474600
     aagtaattgg tcaagcactg ctatgtgtga agaaacacca cagcatactc tacactgtca    474660
     ctcatgatat ggaattgttg cctcctcagc aatgtaactg agcccttaat atgtggctgg    474720
     gatgacaaag caatgccagc ctgcagagtg gaacctgttc ttccttctgg gattagccaa    474780
     aggccggtgg cactcatggc tgaccctact ttccttacaa ctggacgaag cgaagcaact    474840
     ggatatgaac ttctatccca ggatgtaact gtccaaagtg ccttctcttt atgtcaactg    474900
     gaaaagtgtg aaagacagac actattctca taaacggata tcctagttat tccacccact    474960
     agccagctct tccccccagg tggcagtcat agatttagaa ttgcaagtag aggccctggc    475020
     taagcatacc accagtccct ttaacaacac ctaccatacc atcatcattc ttactgaaga    475080
     aacctcacag atttgacaag tgacactatg aagctatatg accctaggta ttgcaactac    475140
     agccaaaggt aacacttgtg tattaatgaa ccctgaatgt tgcatatata tacaagatta    475200
     gtcccacaat ataactcaag ctatgcaagc attagatacc catatttctg ctagagatgg    475260
     atgccctctc tcaggaccct gcgacagcat ggtttagttg acttcctaac acatggaaga    475320
     ctttcatata tagtagagct ggtatactct tcattgctat cttcagttgc tgtggatttt    475380
     attggtacct tgcactttaa gtgagaatgt aatactgacc ttctcaaaaa ctcctaggtc    475440
     cttgcaccat aatgctccaa caaacaccta ctgtaattcc ggagactcca gaatatttcc    475500
     aactccagtt taatggattc ccttccaata tatacaagct atgccctttc taagaagtag    475560
     ccagattgac tatgacacac gttttccata gaaacaaaat ggaatttgac agtggggaat    475620
     gatatacagg cagcttaatt tcaaaatgca ttttaaactt attttttgag gttttaatat    475680
     tattttcata ctccttgaaa cctgaaattt cacacttaca ttctaattga gacttaataa    475740
     caaataacca gaacacatga attgcctgta atctctatac cttgtattgt aagccacatt    475800
     tcaagtgtaa ccttacttgt cagcacagta tgtttaccat aggcataatt gcttgtcctg    475860
     actgtccaga aggcagggtg gtattaagta tgcggcctct ggagctggga agtctggctt    475920
     tgtatactgt ctctgccact gctaactgaa ggacaatggg ccagttactt cctgtacccc    475980
     agttttgtca tatgtaaagt agggttgttg taagaatgaa attagttaat atttagtacc    476040
     taacaactag taggcattac gtggtagcta ttcgtattgc tatcgtcaga gaacacacag    476100
     gagtccattg ttatttttac cctccccctc caaacaaaat gtgtaagaca ctagacatgg    476160
     tagctctgta acggggttcg tcatgtatta aactgtgtac ccaaatgata tgtcaaagtc    476220
     ctgacaacca gcacttcaga acacgtcctt atttggaaat atggtagctg gagatgttat    476280
     tagtgaagat gaggtcttac ttgagcaggg caggccctca atgtgggaag atgggtgtct    476340
     ttctaagtac aaggaaattt ggacacagag acagagacat acagtgtgcc atatgacagc    476400
     agaggcaaag atcagagtgg tgcagcagta aaccaatgca tgtccaggat taccagtcac    476460
     ctccacaagt gaggaaaggg caaggaagaa gtctcctgag tctcagaggg agtgcagccc    476520
     agctcacact tgattttgga cttctagtct ctagaactgt gagacaatac agtcctgtca    476580
     tttttaagcc acccagtttg tagtatttca ttttggcagc cctacgatat tcatacagag    476640
     ttcttcaccc aaaaggaagc actctttctc acaaacagtc aaagtcctgg atgacggaca    476700
     acctggagat tgcaaacctc caaggaatca aaggaggttc tataaagtct aggagcccca    476760
     tctttcaaag gctgctcctc aagtccacac acacactttg gaaggagaat cctgctcttg    476820
     gacatgggat ccttagtgag ccaactgcca ataatccaac atcatcatga attaaagatc    476880
     tctgacattt cctcgtcacc aacgggaatt ctgtcaagta gtaagtaaaa cccttcttca    476940
     ggaatctcca caccaaacag ttctgcatgg cttgggacgc ctcaggaaac ttataatcat    477000
     ggcagaagaa aaaggggaag caaggcagtc atacatggtg gcagaagaga gaggttaaac    477060
     ttttcagtct ctcttgggtg taaagataca atcttgaggc aaacagcaga atcaattttc    477120
     ttatgccttg aaattctccc tttcaccata aatattccct tcacattaat ctaactttat    477180
     gtttgcatcg aactatgtgc ctttttggaa gttccggaag ctaatttgag acagatagac    477240
     aaagtcggga gaccctgatg cagaattcca gaggtgactt caatgttgct agttaacaac    477300
     ccaggcattg cccagatgat gccagcccaa gatccaggtg gactgggatg ccagagagct    477360
     gccagaacaa gacgcacaga cctggttctc agcccaaatc ttgcatgcct ttcttaccaa    477420
     ctttcccttt tttaaacccc tgcctttcct tcaaaattga agtggttgct tgggatggga    477480
     atgcgttcac ttccccttta ccattatggt taatacaata atttttgtac cagatctctc    477540
     tcttgttaat tggatcctgc aggtggcaga ttccggacca gagtgacgtt agaaactgaa    477600
     tgcacatact gggtggttca cagacacagt gttctgtgaa gtttatgggt ggattcctgg    477660
     ggaagcctgc aagcctgtta gtgattagca gaaccgcatc cagctactgg gatctgcacg    477720
     ccaggtgccg tgagcccctc accatgttcc ctggtcccag ctgcccgcgg atggttcagc    477780
     cttgacaacg ggctcggtgt tctgggtggt gggagagcaa gggggcccct tgggcagcgt    477840
     gctacagggc tgtgaatcca ggggcccacc cggtgttccc tgtggagtca ctgagggaat    477900
     gaggggcttc tcagggcaca gagctgtaac gcctttgtgc agtggtggct gaatacagtg    477960
     acactgggcg cgtggtgaga agcggggcaa ggtcagttca ctggaccctc cgccctgagc    478020
     ctcagatgag tcgggggtcc ccagacaggc ccggcctctg ccctgcggcg gacactggag    478080
     cctttgctgt ggccgccacc gaggagcctt gacctcaaag cagcgggaac ctctctaccc    478140
     acctcggaaa cctgaaggca gcggcggcct ctcccagcca ggacctcgcc ggcctccgcg    478200
     tctccaggcc caaccctgta ccacataaag gaagtccgcg ctgagctacc gggacacagc    478260
     gccgttgggt gggcgggcgc ggcgcggcgg gaagcaccgg ggcagctgcc acagagatgc    478320
     gcggggactg tcgggaagtg ggcggtccgg gaggcgcggg gctgatggga cgtgggcggt    478380
     ccggggacgc gcgggggatg gcgggacgtc ggcgatccgg agatgccagg gggcagctgg    478440
     gacgtgggcg gtctggagag gcgcgggggc agctgagatg caggtggtcc gggacgcgcg    478500
     ggggctggcg ggttgtggga ggtccaggga tgcgaggggg cagctgggac gtgggagttc    478560
     cggggacgcg tggggtcgcc aggaagcggg gtctaggact tagatccgcc ttctcttcag    478620
     gccatgcagc cccgaagctc cgaatcctgg gatgactcct gtccatgttg gaagggaccc    478680
     tgccagtcct ggcaaggttc aacggcctta gggcaagaaa tgttagtaga gtcctggaag    478740
     acgatgtggg gaacgggtag cggccaccgg atggtgatcg ttcttctaca agaccttgac    478800
     atggatgggg agaaacagag aagaaccttc caagtttgtc ccactggaca tgcccaccac    478860
     actttaccag ccctttctag aaggcctgtg cgtaacacat gaaaaagctg ctctcgacct    478920
     ttccctgacc ttttaaaaga aaacatttgc tgcatctaat ccgcctagat gtaaggaggt    478980
     tcccaaatgt atgacagagt cagacaatta catgtcttgt aagttaccat gtgcttgtct    479040
     tttgttttcc gttttttttc gtttttttgt tttttgtttt tgagacggag tctcactctg    479100
     tcgcccaggc tggagtgcag tggcaggatc ttggctcact gcaacctctg cttcctgagt    479160
     ttcaacaatt gtgctgcctc agcctcccga gtagcttgga ttacaagtgt gcaccaccac    479220
     gcctggctaa tttttgtagt ttttagtaga gatggggttt tgccacgttg gccaggctgg    479280
     tcttgaactc ctgacctcag gtgagccacc caccttggcc tctcaaagtg ctgggattac    479340
     aggtgtgagc caccgtgcct ggccgtaagt taccgtgtgc tttttaaaca aatcatagca    479400
     aaggggtgtc ttctggaaat gacattttga aatggagtta ctagaccacc cctggaaggg    479460
     acacagtaac cacacgtcca cgttcgttca gtgggtgaga ggacatggag gggagacctg    479520
     ggcaggaagg gaagagggtt ccatgccagg ctgctcatat ttaaaagaca ttttcattgt    479580
     agggacatgg atgaaattgg aaaccatcat tctcagtaaa ctatcgcaag aacaaaaaac    479640
     caaacaccgc atattctcac tcataggtgg gaattgaaca atgagatgac atggacacag    479700
     gaaggggaat atcacactct ggggactgtg gtggggtggg gggagggggg agggatagca    479760
     ttgggagata tacctaatgc tagacgatgc gttagtgggt gcagcacacc agcatggcac    479820
     atgtatacat atgtaactaa cctgcacaat gtgcacatgt accctaaaac ttaaagtata    479880
     attaaaaaaa aaaaagaaaa ataaaagaca ttttcatatc gttgtcattg ttttcttgtg    479940
     tgcattttat tcctcgctat tgtatacatc attggaaatt ctaagtattc ttttgaaata    480000
     tctagtcttt ctagatgttc tgaagtgcct gatgtatgtt aaaattacag gtggtaaaat    480060
     aataaatttt gtaaatgtct ttttgttaaa attcatatgc agtgttttat tttattttga    480120
     tgttggtggg gggtggggag gatggccaaa tccctgcttg atcaacacac attgcgtttg    480180
     tgctctggtt caggggagga gagaggagga gaaagtgcag acttccaggc ctctgtgcgc    480240
     accgggagga gagattaatg atcatctctt ctgtctgtgt gtttgtttta tttatctatg    480300
     gatatcctgt gtataaagga tgaacaagtc ctatttataa catctaatct tttcaggtgt    480360
     taagttgcca gcgtatgacg gaagtagcat tattaaacga atgcagcttg tacatattgt    480420
     gttaaaattc ataaaaagcc agtcttctga aaagaaccct tgggccctcc ctccgcagcc    480480
     tccggcgcca gctgcaggaa gacctggcca ggggaaggcg ggcggcagaa gctgctgctg    480540
     gctgtgggga aggcggtgga gcccaggcct ccacaggctc cccacaggct ccccacaggc    480600
     tcgcgcggct ctggttgctg ggctccgcac gggccgcgtg ggggcgcctg ctgggagcga    480660
     ggaggcgcca tcctggctcg cctgctccag gaggacgctc tgggcctgcg cagatgcagt    480720
     tctccaggat gggcgcgggc gctgggggcg gcgctccttg ggctgctgcg cctccactga    480780
     gccgcctggg tgtgaggacc tcgcgccggc gcctccggga aggacgcgga gccgccgcgg    480840
     ccgatcccag ggctgcagct gctgcccgac gcccgcggga aggcccttcc gccaccgccc    480900
     gggggttcga acttggagca gctgctgcca gctgaggcgc cagcggcgtc gggagctcct    480960
     cagagtcagt gttttgtgct ggattcggca tgtcctctga aaactcggga atttgtcact    481020
     gaaatggtga caggaggtga gaagtttttt tagacgttgc cttcgctggc ctggcgcggg    481080
     gctcaggcct ggaatcccag cactttggga ggctgaggcg ggcggatcgc ttgagctccg    481140
     gaggtggaga agctgccttc gtggtaactg tggtcttaag ttcagctgag aacgataaat    481200
     ggcttttcct tgaattggct gcttttgtga tttctctcag gctcatcttt tgtcagttaa    481260
     aaatccagtg gtaggtgtag cttagagacg ggaaattttt ggttttgttt tggctacacg    481320
     taagtgttgg aaattatttt cttttacgtt ccagtgtgag caaatccaga aggatgtcag    481380
     attaaacacc gaatttaaca aattcagccg ggcacggtgg ctcacgcctg taatcccagc    481440
     actttgagag gccgaggcgg gtggatgatg aggtcaggag tttgagaaca gcctgaccaa    481500
     catagtgaaa ctccgtctct actaaaaata caaaaattag ccgggcgtgg ttgtgcgcgc    481560
     ctgtaatccc agctactcag gaggctgagg caggagaatc gccgagattg cgccactgca    481620
     ctcagcctgg gcgacagagc gagacttcgt ctcaaaaaaa caaaaaaaaa tcaatcattg    481680
     gaatactgtt gttcattaca attaatgaac gtttgataga cgcataaacg aactaaattc    481740
     acaagtacat ataagtaaaa taagccaaaa aaaaaaaact acatcccgta tgatgacact    481800
     tttatacatt ctacagaata gaaactaaag tgatatgaag gttgggagtt gaatggagaa    481860
     aaggtgagcg aaaagaggga ggacggagga gttgcaagag aacaagagaa aatgttgagg    481920
     gtaattgatc tgttttctat cttaataatg gttatggtta catcaataat ttctaaattg    481980
     tacattttgt gtgttaatta taattttata ttattcaagc tattacaaat aaatacctgt    482040
     ataaatttct tagggacggt ttaagggaga tggataaatc acataaaaag tcagatattt    482100
     ctgaatgtac aatgaataac ccttagctct atctttattt tttggtaatt ctaaaatgca    482160
     gcagattttt ttaatgtttt tattacaaga agggtttctt caaaccacac caggtgtgtg    482220
     taactctggg ctaaagttgg ttaagggagc agtgggatcc ggtggagagg agcacaggcc    482280
     tcgatcctgg agcccattct tgtcacatgt gagctcctgg acacatctca tagctccctc    482340
     atgcttctgc gtttacattc tcatctgtaa atggaggacc aatgggcatc tgcctctaac    482400
     aatgtatttt catgtgttaa gatgatgtat gttaagtgtt tactatagca caatctttta    482460
     ataagaaatt acattttttt ccaaatacta tcattgtctt caagccccac cacccaccag    482520
     aaaacttaat atgctctgtg ccctgccctg tcctcagaca tccagcccaa gagactttta    482580
     tatagtagga gatatgcaaa tagttccctc cctctgctga tgatgaaaac cagcccagcc    482640
     gtgaccgtgc agctctggga gacgagctca agtgccagga ttcccaagtg ttttcacttg    482700
     gtgatcagaa cttaacacag aggaatcacc atgttgtttg ggctgagctg ggctttcctt    482760
     tttactattt taagaggtga tacatgaaga actacagata ttgttcgtga gtggatatta    482820
     gagaaacagt ggatatgtgt ggcagtttct gaccaggatg tctctgtgtt tgcaggtgtg    482880
     cagtatgagg tgcagctggt agagtctggg ggagacttgg tacagctgtg gtggggttgg    482940
     gggtgtcctg agactctcat gtgcagcctg cggattcatc ttgagaagca atgacatgaa    483000
     ctgggcccac cgggcttcaa gaaaggggct ggaatgggtc tcatacatta gtgctagtgg    483060
     tggtagccta tactatgcag acactgaagg gtagattcac catctctaga gacaatggca    483120
     agaacatgct gtacttgcaa atgaacagtc tgagagatga ggactcggct gtgtgagaga    483180
     catggtgagg ggaaatcagt atgagcccag ccagaaacct ccctgcagga accctggggt    483240
     ggggggaaat cagctgcagg gggcactcag gacccactga tcagaatcaa ccccagaagg    483300
     aggtgcacat ggagattagg ggctggattc cttccaggat gtgggacttc aacttcttct    483360
     gacagtttcc ccagggaacc gctctaagtt tagaattctg tgtcatctct atgtatttaa    483420
     aaaagatcat tttaatgtga ggaactaact attctcacat gcacaaaatg cagattcaca    483480
     ctcacagaga tgaaaagtcc tcaactgtgg tcactaggat cagagtcctg aggaatctca    483540
     ggcgttcctg gtgagtcttc tccaatcaga cccaggacag acacctcagt gagattccat    483600
     gactagaaca ggctttatgg attttaaaaa aaatagactg agccagggtc agtgtcatgt    483660
     agaacctcac aggtttcaca tctgaccctt ctcctgacac taaagtgtgc aaatcagcat    483720
     caatactgat ctggtgctgc ttttgctccc aatctgtgtt ctttcttgag ttgtcccttt    483780
     ttcatttgct tttcctgctc cctgaaaaaa gaaagatgtt gtctctgtgg tctaaattcc    483840
     agggctcaag cccttttctt ggagctcagg tgggtctcag gttgtggctc ctgcagccac    483900
     gctggggagg ctgatgggac tttcttctct cccattgctc agggccctcc actatattgt    483960
     gtggagactc atctcagaat agatgtggcc agtgggagct gaaggggaca agcttgtttg    484020
     gtcaaaatga gatacaaata tggaattcac cctgtgctgt acaaactgtc acagagtcac    484080
     tttctgaaac aatagtgtta gaaagaactt gagaaagttg tcagaatcaa aattgggtca    484140
     cttgtgttaa aactctgctg aatgaagctg ggaaaggccg tgacggaatg ttctcatgct    484200
     catatccctg ataccaagaa ctatcgtaaa tagactctta aaaccacaat cttgcacaaa    484260
     ggtgcccaca accttaaaag aattacttct ctgagtaatc ttcccagcag ctctctgtgt    484320
     aagattggac tgatgccacc cttgttattg attcttctat cccggagaag ttgtctcaaa    484380
     acagcttatg taaccctcct catttttctt taaaccttta tctttcttta cctgcctgag    484440
     tgaacccatg catgttccca ttgcaatgtc catcttcaaa taattatcat tttactttag    484500
     agtctctttt tgtctgttat tcagatttga caagctgcag atggacacat cacgtttctg    484560
     taagacttag gggatgacag ttttgaggca tggctgggga agtacaatgt ccataagatt    484620
     gaccctcaat aagtgcaggc tgtaagtctc agagctgaag ctgctgaatc accatagaat    484680
     ttcatcttta gttctgctct gatggaatca ggcccacctg tgtaatgaat gacactctac    484740
     ctaacctaga gtcaactgat gacaggttaa atatctagac agcaaatttg caacaccact    484800
     tagattagtg tttgaataac tacaaagtat agccgagcca agtatatcgt aaagcagact    484860
     attatacatg gagaatcttt aacatgacat ctacaattct taattttttt tacgtataaa    484920
     actgatggtt ttacaatcag gctgtcatga ttttgctaat gagttgtaga agttacatgt    484980
     atgttttaaa tattaacagt tttttcagat gcctggtttg caactatatt ctcccattct    485040
     gtagatttgg aattttttcc atgctttgca aaatctttat attttgatgt agttccagtc    485100
     cttcaattct gcttttgttg tctgtgcttt taatcttaaa tttaggaaat cattgctaac    485160
     cttttggtgg gagagaattt tacaattgca ggctatacat ttaactgttt aacccatcta    485220
     gagttgattt tttgtgttta ttctaaccta atattcttaa ctatttgcaa gtgaaaaccc    485280
     agtttgttta acaccctctt taggggagag tatgatttag ccgttgtgta gaaaatgtta    485340
     taaatcactt tgccttcata ttggttctca acttgaaaat gagtttgcca tcaatacgtg    485400
     ggtttatttc tgagctctct atatgtattt atgccagtac catcctgttt tgattatgtt    485460
     tgtaattaat tttgaagtct gagagtaaaa tgcctccaag tttattcttg cccttttgca    485520
     gatattagac aaaatatttt aacatttcac catgaagtat gataacagct atggcttttc    485580
     ataatgaatt ttattgtgtt taggttattt tcttgtattt ctattttggg tagagttttt    485640
     atggtgaaac cttgaatttt tttaatgtta ttttctgtct gagaagttaa tgggaccttc    485700
     ttcgtattta atttggcata ccaaatgtat tgatttgagg attttgaatc atctgtgcat    485760
     ctcaaaaata aatttgagtt ggtcatggtg tatgatcctt ctaaagtcct gtggaactta    485820
     gttgactatt atttggtgtg tggagggggc tcatttatgt ctactaggaa tattggtctg    485880
     tagtttactt ttttgtgacc ttgtctatta ctgaaaatac ggtaatttgg gccttataga    485940
     aagactggaa ggttggcatc tcaccaattt ttgaaaaagt tagagaatgg ttggcattac    486000
     ttctttaaat gttgagagtc ctttttttac ttaacatgtg acctatcatg gagaatattt    486060
     agtctttgct tcggaagcat acatattaca ctgctcttgg ttggaaggtt ctgtagatgt    486120
     ctattaggtc aatttgttca atagtgttgt tcaagtccac tgtcttctta agaattctct    486180
     taatgatgtt ctatccatca ttgaaagtga ggtgctaaag tcttctccta atttttatta    486240
     ttttctattt atctctccat atctcttaag gtttgcttaa tgcagttata tttatttttg    486300
     tacaaatatg taaaaaataa atgaaataat aattgctaag taattttaat ggcaaaatca    486360
     cataataaga aattatattt tcccaaatgc tgtcattgac actaagctcc tccaggaatc    486420
     tcacatctgc tctggacacg gctctcctgg tgtcctaccc cagagcttgc tatagaggag    486480
     gagacatgca aatagggccc tcactgtcct gatgaaaacc agccttgcct gcgtctacgg    486540
     gagaagagcc ccagtccaga agtaccaggg gtttccattt ggtggtcagg tctctgaaca    486600
     cagaggactc actatggagt ttgggctgag ctgggttttc cttgttgcta atgtaaaagg    486660
     tgactcatgg agaactagag atattgagtg tgagtggaca caagtgagag aaacagtgga    486720
     tatgtgtggc aggttctgac cagggtgtct gtgtgtgttt gcaggtgtcc agtgtgaggt    486780
     gcagctggtg gagtctttgg gaggcttgtt atagcctggg ggtccctgag actttctttt    486840
     gcagcctctg gattcacctt tagtaccttt attaggtact ggatgagctg ggtccatcag    486900
     gctcctggga aagggctgga gtaggtctca tttatgagtt gttgtgtagg tagcacaagc    486960
     tatgcagact ctgtgaaggg tcgattcacc ctctccagag atgatgccaa gaaatcactg    487020
     tatctgcaaa tgaacagcgt cagagccgag gataggtctg tgtattactg tggtggcatt    487080
     gtgtgcatcc cttgtttagg tacatgcaga gatgctgctt tggtgtgttc aggggctcct    487140
     gttttgggga caccaatttt ggagtttgca gtatccttga gtccagtacg ttcatggtgg    487200
     cattgggtct ggggtgtgca gtggcaatga gtccaggata catgcagctg atgccgtatc    487260
     ttgtgaggac aagaacaact acaaaacgat cctattaata aataggcaaa tgacttgaat    487320
     acacattttt caaaaaatac atacaaatga catatagata tataaaatgt taaatatgag    487380
     taataatttt taaaattcaa atgtaatcta caataaatta tcaccccata cctgttagaa    487440
     tagctatttt taaagaagat gaaagataac cagtattggg gcagaagtgg agtaaaggga    487500
     gcctatatat attgttactg aaaatgtcaa ttagtacaga cattatggag aatactatgg    487560
     aggttcctca aaaaataaaa atagaactac catagtttcc aacaagccca ctactggcta    487620
     tgtatccaca ggaagtgcaa tcagtatgtc aaagagacat ctgtgccccc acgttcattg    487680
     caggaatatt tatgatagta atgatgtggg aacatcctaa attaccataa atggatgaat    487740
     tactaaagaa aatgtagtat gtacccaatg gagtaatgtt catcctaaaa taagaaggga    487800
     atcctgcatt tgcaacagca ggcatgactc tagaggacca tatgtgaagt tccacaggca    487860
     ggcgcagaag acaagtaatt cgcgatttca ctgtagtttg tttaaatggt agactcacag    487920
     aagtagagtg gagtgttggt taccaggggc tggaggggtg gactggaaaa ggcgagattt    487980
     tggtcaaagg gtgaaatgtt ccagttagac agaaggagaa agttacagtg ttctaatgca    488040
     caagatggaa aatatggcta ataatgcatt atatatttca aaatggctaa aaatgcaaat    488100
     gttaaaaatt ttctcactaa gaaatgatac agcctgggcg cggtggctca cacctgtaat    488160
     cccggcactt tgggaggctg aggcgggcga atcacaaggt ttggagttca agaccagcct    488220
     ggccagatgg caaaaccccg tctccactaa aaacacaaaa aatagccagg tgtggtggca    488280
     cgcggttgta atgccagcta ctcgggaggc tgatgcagga gaattgcttg aacccgggag    488340
     acagaggttg cagtgagctg agatcatgcc actgcactcc agcctgggtg acagtgagac    488400
     tccaactcaa aaaaaaaaaa aagaatgaaa tgatagctgt gtgaggtgat ggatatgtca    488460
     aatagcttga ttaacaattc cataatatat acatgtatca tagcatgtgt gaggtgacct    488520
     gtatgaaggt gaagtgacct gggggctgtg taagattacc tgggggtatg tgtgaggtga    488580
     acacggcaca tatgaggtga ccagagacac atgtgagggg acatgggaga tatgtcgggt    488640
     aagccagggg gatttgtgag gtgacacaga gcaagtgtga ggtgacgtgg atgcatgtaa    488700
     ggtgatttgg ggcacatgtg aggtgacgtg ggggatatgt gtgagctgac ctcgggtgag    488760
     tgtgaggtga tccaggatgg atgtgaggtg acctgggggt gtgtgtgagg tgacttgggc    488820
     acatgtcagg tgacatcggg gattggtggt gatgtgggtg tgtgtgaggt gacctaagga    488880
     ggtatgaggt cacctggggc atctgtgggg acctggaaat gtgtgaggtg ggtacaggtg    488940
     aagtcaccta gggcacatgt gaggtgacct gtggaatgtg tatggtgacc tgggaccgtt    489000
     tgaggtgaca tggggaatgc gtgaggtgac ctggggcatg tgtgaggtga caaagggcat    489060
     gtgtgcagtg acctgggtgc atgtgaggtg acctggtgca tgtgttaggt tacctggggg    489120
     atgtgtgagg tgaacagggg caggtgtgaa gtgcctgagg aatgtgtgag gtgaactggt    489180
     gcatgtatgt ggtgacctgg gggatttacg aggtgacctg cgggatgtgt gaggtgacct    489240
     tgggtgagcg tgaggtgacc tgacgtatgt atgaggtgac cagggtgtgt ctgaggtgat    489300
     gtggggtata tgtgcggtga cctgggaaat gtttgaggtg acctggtgga tgtgtgaggt    489360
     gacctgcagg atgtgtaagg tgacactggg gatgtgtgag gtgactcagg gcacatgtga    489420
     ggtgaaattg gggatgcgtg aggtgattca gggcatatgt gaggtgacct tggggatgtg    489480
     tgaggtcacc ttgggtgagt gtgtggtcat ctgggatatt tcccaacttc gtaaggaaag    489540
     cctgcaacaa cacatggcat tagaagcttc acactctagc accattaatg cgtacatcct    489600
     caaggaactc taaatgtttt gaggttagct taaatgccat ggagcggcac cttccccagg    489660
     cacactcata tatgcatcct gggtctccct gccaagctat atctcctcaa atttacagtt    489720
     tatgtttgct ctgtgacatc aactctgata tgttcaagtg tgtttttttt tctttatttg    489780
     tagttgttca ggcttgttgt ttcactctca ttactctggg ctcagtcctc tcctcaggtg    489840
     tcccacttca gagctcgctg tgaaatagga gacatgcaaa tagggccccc cctttcctga    489900
     taaaaagcag cccagtcctg accctgcagc cctgggagag aagcaccagc cctgggattc    489960
     tcaggtgttt ccactttgtc atcagcaaca aacaaattac catggaattt gggctgagct    490020
     gggtttttct tgctgctatt ttaaaaggtg attcatgaag aactaaggat attgagtgag    490080
     tggacatgag tgagagaaac agtggatttg tgtggcagtt tctgaccagg gtgtctctgt    490140
     gtttgcaggt gtccagtgtg aggtgcagct ggtggagtct gggggaggct tggtacagcc    490200
     tgggggatcc ctgagactct cctgtgcagc ctctggattc accttcagta acagtgacat    490260
     gaactgggtc catcaggctc caggaaaggg gctggagtgg gtatcgggtg ttagttggaa    490320
     tggcagtagg acgcactatg cagactctgt gaagggccga ttcatcatct ccagagacaa    490380
     ttccaggaac accctgtatc tgcaaacgaa tagcctgagg gccgaggaca cggctgtgta    490440
     ttactgtgtg agaaacactg tgagaggtcg gaagtgtgag cccagacaca aacctcctgc    490500
     aggaacgttg ggggaaatca gctgcagggg gcgctcagga cccactcatc agagtcaacc    490560
     ccagagcagg tgcacatgga ggctggggtt tgtttcctgt caggatttgg gacttcctct    490620
     gcttctgaca gtttctctag ggaaactctt taattttaga tttctgtgcc caccaatgtc    490680
     atctctacat ttttttaatc attgtatatg aggactcgtt ctcacatgca caatatgtat    490740
     attgccacct atgggaatga aaggtcctca accatggtca ccagcatcag agtcgtgagg    490800
     aagctcaggg gtgcctggtg agtcttctcc agtcagactc aggacagtaa cctcaagggg    490860
     attcccttgt gagaactcac acattttcat gagaacagca ccaggagtca gttctaaacc    490920
     attcatgaaa gacccactcc atgacccagt cacctcccac caggtcaccc cttcacaact    490980
     ggggattata atacaacatg agatttgggg caggacacaa atccaaacca tatcagatac    491040
     acattgtgaa atacgcatgg tggtcaggta atttgtattt ctatcacctc acagcgctac    491100
     cattttatta ttttttttaa tttactgcat gagtgagtgt ctgatgagaa cacctaagat    491160
     ctaccctttc agcaacaatc atttttacaa tacagtatta actataggac cattgctgta    491220
     cgttagatct ccagaactca tccaacctgc acaactgaaa ctctgtacaa tttaacacac    491280
     atcacccaat ttccctcacc tcccaggtcc tgggacccac tattctactc tctgctttca    491340
     agagcttgaa tattttagat cccacatgta aatgagatca tgcagcattt gtctttctgc    491400
     atctggctta ttcaactcag catcatgtcc tccaggccca tccgtgttgt tgcaaatgtc    491460
     agaatttccc tcttttcaaa gccaaacaaa atacagatgt atgtatacac attttcttta    491520
     tacattcatc catttacagt cattaaatta ttttacaaat ctacactatt attaataatc    491580
     ttgcaatgaa catgtctttg gcaaagtaat tttatttcct ttgcatatat aacaagaagt    491640
     gggatcacca gattatatga tagctttatt tttaacttat caagtaacca atcctaccac    491700
     aggatatttc cctttccccc acattcttgc caacatttgt catctgttac atttctgata    491760
     atagccatat taactagtgt gagttgatat tgcattgtgc ttctcatttg aattcctctg    491820
     ataattagga atgttgagaa ctttttcgtt ttctgtttgc catgcatgta tcttctgaaa    491880
     aaaattatcc aggtttttgc ccttttttat cagttcattt gttatttgct gttgaggtgt    491940
     atgggttatt tatacatttg gacagaactt cttgtcagat ccataattgc acatagtttt    492000
     tcctgtgctt tgttattaaa ttcaaagaaa tcagttccga attaatggca ggaatttttt    492060
     ttgctccatg tcatttatga gtttatggct tcaggtatta tgtccatttt tagttgattt    492120
     tttttttttt tgggagacgg agtcttgctc tgtagcccag gctggagtgc agtggcgcga    492180
     tctcggctca ctgcaagctc cgcctcccga gttcacgcca ttctcctgcc tcagcctccg    492240
     agtagctggg actacaggcg ctggccaccg cgcctagcta attttttttt gtatttttag    492300
     tagagacggg gtttcactgt ggtctccatc tcctgacctc gtgatccgcc cgcctcggcc    492360
     tcccaaagtg ctgggattac aggcgtgagc caccgcgcgc ggcctattag ttgatttttt    492420
     tatatggagt tagagaaggc ctaattttat ttcttttgca tatgaatgcc agttttacac    492480
     cattattgaa aagactgtcc tttctctact gtgtgctctt ggcacacaaa atcaggagag    492540
     acataatgac aaaaaaaata tgagatcaat agtcctgatg aacatagacc tgaaagtcct    492600
     caacaaaata ccatcaaatt gaatccagaa gcactttaaa atgtgataca tcatggtcag    492660
     gtgggcttta cccctgggat gcaaggctcg ttcaatatcc acagtaactc tgattcacaa    492720
     tgtaaacaga ataaaagcaa aaagcatatg attatgtcaa tagatgctga gaaagcttcc    492780
     gataggatct aacatctact catgataaaa accctcaaca gactagacat caaagaaaca    492840
     tacttcagaa taagagccat ctacaacaaa cccacagtta acatcatact aaatgagcca    492900
     attaaaacac ttatctttat aaatgaccca gtctcgggtg tttctttaca gtgcgagaat    492960
     ggactaagac agcatccaaa taggaaagga agtcaatccg tccatcttca ctgatgatat    493020
     aattctatat ctagaaaatc ctaaaaactc tgccaaaata attctagaat aaacaacttt    493080
     agtaaagtgt caggatacaa aatcaatgga caaaaattac cagcatttct ataagccaac    493140
     cacatccgag ctgagagtat aatcaagaac acaatcctat tcgacttaca gtatccacaa    493200
     agaaaatgaa atgcccggga atacagataa caaagtgaaa gatccctaca aggagaacta    493260
     tgaaacacag catacataaa tcataaatga tacaaataaa tgggaaaaca tttcatgctc    493320
     atggattgga agaatcaata ttgtaaacat tgtcataatg cccaaaggaa ttcagattca    493380
     atgctattta cataaaacta tcatcatcat tcttcacaga atcagaaaaa aatctattct    493440
     aaaatttata tggaaccaaa aaagaccctg aatatccaaa gcaatcctaa ggaaaaagaa    493500
     taatgccaga ggcatcatga tactgacctt aaactacacc ataacgtctt gctaacaaaa    493560
     acagcctgta ctggtacgaa agcagacctg caggaaaaaa aatagatcaa aatagaagac    493620
     agaaataaag ttgcacacct gcaacccttt gatctttgac aaagctgaca aaaacaagca    493680
     atggaaaaaa aaaactctgt gttcagtaaa tggttctggg ataactggat tctggcaggt    493740
     atgcaaaatg caagagtgaa ggagcctgtg cagcttctac ctagatttca gatgtgtaga    493800
     gagccctgca tgcccaggaa gaagcctgct gcaggagtgg atccaccaca gaaagcctct    493860
     actagagcag tgccaaagat ggggaaagat ggagttggag cccccattca gagtccccac    493920
     tagggcactt catagtaaag ttgtgggaat gtggccacaa ccctcaagac cccagaatgg    493980
     tagagctacg ggcagcttgc accctcagcc tagaaaagct tccagcactt gactctcact    494040
     tgtgaaggca gccccgtggt ctgtgcctag caaacctata gggattggct gcctgaggtt    494100
     ttggggaccc aatccttgtt ccagtgtgcc ctggttgaag tacataaagt caagagagat    494160
     tattttgtag cgttaggatt taatatctgc agtgctgggt tttatatgtg tgtggggcct    494220
     gttggttgtt cctttggcca atttatccct ttgggattgg aattatttac ccaatgcctg    494280
     catcatcatt gcaccttgga agtaaacaac ttttattttt taaatttgca agctcacagc    494340
     tggagggaca ttgccttaag actcagatga gactttgaaa atttgagttg gacgttgatg    494400
     aagttaagat tttgggcact attgaaaaaa ggtgattata ttttgcaatg taagaagaat    494460
     atgagaactt tgggtccgag agtgcaatga tataatttag gggttttccc ctccaaatat    494520
     catggtgaaa tgtgacccac aatgttggag gtggggccaa ctgggtggtt tgggtcatgg    494580
     ggaaagattt ttcaggactg gcttggcatc caccccatgg taattagtga attcttgctg    494640
     tattagctac tgtgagatct gattgttcaa aagagtcttg caatcctgct accctctcat    494700
     gtaacccctc ctcacaatat gacacagcct tctccccctt tgccttccac catgactgta    494760
     agcttcctga ggccctcaca agaagcagat gctggtgcca tgcttctcac acaacctgca    494820
     gatctgtaag ccaaataagc ctcttgtctt tgtaaatcac ttggcctcag gtattaattt    494880
     atagcagtgt aaaatagact aatacactgc ccaaagcaat atacagagtc catgcaattt    494940
     ctatcaagta actaattaat tatgtatttt taaaaatcct aaaatttata tagaaccgaa    495000
     aaacactctg aatagcaaaa gcagtcctaa ccaaaacgaa caaagctgga agtaccacat    495060
     tccctgactt caaattacac agcacaaaga taataagaaa ggcagcatgg tagtggtaga    495120
     aaaaaaatca agagcccaga tatatagcca aatatctaca accaacagtt ctttgacaaa    495180
     actgacaaaa atatacactg gagaaacaac cctctattca ataagtagtg ctgggagaat    495240
     tagatagcct tatgtagaag aataaaatga gacttctgta tcatcataga cacaaattaa    495300
     ctgtgaatat ggattcaatg tttataaact cataaaaata cttgaaaaaa tctaaaaata    495360
     atcctctgga cattggccta agcaaataaa atatgactaa gacttcaaaa gcaaatgcaa    495420
     tgaaaacaaa attagacaaa caggattaat tgaaccaaag atcatctaca catcaaaaga    495480
     aagaaccaat atggtgaaca gacaacctgt gaatggtaaa aaaaaaaaaa tgtgcagtct    495540
     attcatcaaa tatatagaat ctacaagtaa ctcaaataag tcaactagaa ataacaactt    495600
     cataaaagag tgagcaaaaa cgagatattt ctcaaaagaa gacatacaag tgttcaacag    495660
     aaataaaaca tgctcagcat cacaaatcat ctgataaatg taaattacag acaacatgat    495720
     atagcatctt ccaccagtca gaatggctat taaaaataaa acagatgttt gcagagaagc    495780
     ataggaaaaa taatgctttt tatatgcttg atgagaatac aaattggtac aacctctatg    495840
     aaaaacaata cagaaatttc tcaaagaact aaaattagaa ttaccatttg acccagtaac    495900
     ccctcctggt gggtatgttt ctcccaaaag gaaacttcta tataagaaaa agtcacctgc    495960
     attcctattt ttattgcaac actattttca atagaaaagt caagtaatct ctctaagtgt    496020
     caatcagtgg atgattaaat aaaatatgat agatgtaaat catggaatac tatgcagcca    496080
     taaaaaatga aattgtgtct ttcacagcaa catgaataga gttgaaggac atttcctaaa    496140
     taaaataact caaattctct tttataattg gaaagcaaac aatgggtaca catgtacata    496200
     gagaaaaaca atagacactg ggtactgcaa aagaaggcaa ggtgagagga aagtgagggt    496260
     taaaaaatca gctattggct ccaatgttca ctatggggta acagttacat cagaagcaca    496320
     aacctcacct ttatgaaata tattcatgtt acaactctgc acatgtgcac cctgattcta    496380
     tgaaaataaa aacagaaaac atgaacagaa ttgttagact gctagctaga ttgaccaaca    496440
     aacaagaaaa atgcaaaagt agcaattctt tgttttgtaa ccctttctca ttttctggta    496500
     ccataagaaa ttgtagacta tcgtcctata ttcttttctg aagtgctacc attagccatt    496560
     tctcaaagaa attctggttc cttttttctt ttattttctt tcattttttt tagacagagt    496620
     ctgcaacgtc cacctcccaa gttcaagcga ttctcctgtc tcagcctccc aagtagctgg    496680
     gattacagac gtgcaacacc acacctggct aatttttgta tttttagtag agatgaggtt    496740
     ttgctatatt ggccaggctg gtcttgaact cctgacctca aatgatctgc ccacctcagt    496800
     ctcctaaagt gttggaatta tgggtgtgag ccactgcacc cagccgctgg ttccttttac    496860
     agaagaatag tattaaaaac tcatatcttg attcttgatg tactttttgt taatttcttg    496920
     tagaacatct caagtaatca atacaggaag tatttgttgt gtattaaccc atatatatat    496980
     atacccacat ctaaacggtt tttatttatg ttcctatatt atgctaaact tgcaaatcca    497040
     ctgagatacc ctaagttaat accacatgaa tgattttcac cttccttcta tgcctttccc    497100
     taacctagca ctccaaccgt ggggaacccc ctcccaccac acaccatcca ttccctttgt    497160
     agtccatttc caggattcct gtagagtgga acccgattgt gtaagttgtg ctcttttgtg    497220
     gaacatcatc aagtggagtc cagtgctgac gtgcagtttc ttttttcttt aatcttatgg    497280
     actacactat ttctgaacct acttagtacc tcttttgatt tcatacatta gtataatggc    497340
     ataagatatt ttctatatag tctgcattcc atcctggaat tcctaatcac ctattttttt    497400
     aaaatttctt gtggattaag attgtattag tcagtgtccc ctagagggac agaacaagta    497460
     agatacacat acacacacac acacacacgg agtttattaa ggagtattaa ctcacactat    497520
     cacaaggtcc acaataagcc atttgcaagc tgaggagcaa acaagccagt ccaagtccca    497580
     aagctgaaga acttggaatc cgatgtttga gatgaggaag catccagcag gggagaaaga    497640
     tgtaggctgg aaggcgaagc cagtctaatc ttttcatgtt cttctgttta ctttttattc    497700
     tggccacact tgcagctgat tagattgtgc cacccagatt aagggtgtat ctgccttttt    497760
     cagcccactg gctcaaaggt taatctcctt cggcgacacc gacacagaca cacccgggat    497820
     caatactctg catccttcag tgagatcaag ttggcactca gtatttagca ttacaagtcc    497880
     accctttgtc aacttgaacc catacacatc tcctgagatt atacataatc ttcaaataaa    497940
     gacaagaata aagtcataat tatgcctaat aatacaatta ttcttcttac aactggaaac    498000
     acaccaatcc ccaatccaaa tggtactaca tatgctccac tccctcaagg acacagccag    498060
     gatcatctga tgacttggtg tctagatggc ttcagcactt ttaattttac ctccaatgtt    498120
     agactccact ttatcaatgc agatgtagca gaatatttta agacattgat atgctgaaaa    498180
     attcaacagt tcataaaagt tgccttccca ccacagccag tggttactac tgaataagga    498240
     cctgccatcc tctagggaga agctataatg gatggggtgc actgttggtg gttatggaaa    498300
     tcgatccatc gtggcaaatg gcagcatctt tctttgataa cattatattc tattgtgcat    498360
     atataccaca ttgtctttat tcatttgtct atctactgac agattttttc catatgtttg    498420
     ctgttattaa tagtgtttca ataaacatag gatgcagata tcttcacaag gtggtaattt    498480
     catctccttt ggatatactc ccagaaacag gattcctggt catacaatat ttctggcttt    498540
     aattcattta ggagccttta tactgctttc cataattgtg aaaatgtaga atggtatagc    498600
     cattatgaaa aacagtttca gttttgaggt atgatctgta aacaaataat gtttgttatg    498660
     gctctcaatg agagtttcta ctaaatatga tggaaggtca ggaaggtctc tcaccttaaa    498720
     gcagggatgg attatgcctc ttatttccta aaagcaaaag attggaagca tgcatgatgg    498780
     aggtggttgg caggtttcag gatgatctca taagaggaga atctgttgga taaaactgtt    498840
     gggttttcag tcatagacac acctacactc aatatgaaat gatgaaatca agtgaggatc    498900
     tagaatgtgt tcgcttgaag caacagcata ttctcaaagg cacctattgc tccatcacaa    498960
     gggtgatgaa cttttgaaac cagtaaagtg tgagagcaca gtaagtgata aagttatcac    499020
     atttacatga agtttgttta atgtgccaag ggctcgcaca gattaatcat aaataaacac    499080
     aatgtatttt agcatacata gatgtctaga gaagaatctt cttagagaaa cttcttcagg    499140
     ttacagaaat ctgtgtaagt tgtagatcta atggagaagt gtctatgaat actggtctgt    499200
     ttttgaagta tggaaagatg atctcttttg gagtaggctt gcgattgtgt tgatttctta    499260
     attgtttctt gagttgtaac aaaagaacca aaaaaatgac taaccggagt ttcatggctg    499320
     tgttgataaa aatttttcat aagctttctg tcaattattt gagaccagat ttcctgattt    499380
     ttttctccaa tagatacatg ttcacaagat ctcaggaaac cacctctcag gtgctttagt    499440
     ttaaaagact agcttcctag aaaaagaaga ttctgtctaa ccatgacaag ttggtttcat    499500
     ctgcaaacca tcaagtttga gacctcattg tcaagaatca taaaaatttt aactccaatg    499560
     tactgaaaat cataaatctc gaatttaatg ttgataggca ttgacaaaat ggttcattct    499620
     tggatgcgtc ctatgttgct ggctcaacat atcagttaat tgagaatcct cacttagctc    499680
     cctctgtttg tagcactgag ttgattgtca atcttcagag tcatccgttg aaggagagtc    499740
     ctgaggttca tgggattctt gtagacactc aggggaacaa aagaaagaaa cagaattgag    499800
     ggctgccagc catttccaca tcactaggaa taattaccat ctaagtataa aggtctgcat    499860
     cattcagaac accctgcatg acaggctgat gcaaaaaatc caaccctaca gaggctccac    499920
     agcaaccttt acagttcttt ccgggaagaa ataatctcca agttaagtga gtcagtaaag    499980
     ctgctctgag ctacagtaaa aattggattg ggcccgattt gtctgagttc agtgtaatta    500040
     ttatactcag ctgctgcccc aatatagact gaggatggat aatttaaatg agcctggcta    500100
     tgtggtttgt tatatatctg aactaaataa acataaaggg cctgtctgga ctagcgtgag    500160
     ggtgagagat cctgggagcc ccagcccccc atactcttat ctcccttcct ccaggaacct    500220
     ctaggttctc agggtgagaa tccacaagat ccattcatgg ctctattacc aggagaccaa    500280
     aatctctgac cttgttcata cagacaaaca ggcgaaggac atttttagat ccttatctat    500340
     gtggcgaaag gcaatctatc cccattacaa gccttaccag caaccttcct ttgtcatgaa    500400
     aatgggtaaa attagccaat aggattacat ttaagaaaat tttcctgtga tgttccagcc    500460
     agaaaacagc aaaaatcagc ttcacacctg gagacttctt gcatcggtca cagcccagag    500520
     aaagaagatc accatagcat taaaatgcaa ctgtaagaac atgtaatgct tccatgttcc    500580
     acgcattacg tctcaccagt ttagtcaata tggattaaat atgagagtgt ggcaattcgc    500640
     aaactctatc tgaggaggaa aatcggataa aaaatgttat gaaaaataaa gcaatttgaa    500700
     gcctctgact tcagcaactt caccactaat gaaatgatgt aaccctcatt ggcctcaaat    500760
     ttagttttca cggggcatct gcagggttcc aaagtgagac caggtgaatt caatgtgcat    500820
     gcacttccca agtgtccact tgtattctgt ttctttactt ctgtttacag aaagtagaca    500880
     catattcagt cttagtacca gtgtagggag cgctttccat gagatggata ccagaaaaaa    500940
     atggcaaaca tgggatccgt taatataaaa attagccacg atgtatatat atatatgtgt    501000
     gtgtgtgtgt gtgtgtgtgt acacacgcgc gcgcatgtgt gagttgaata gcagagttgg    501060
     agtgggtttc tatccacatg tacctgcacc tgcaggtatt ctcaggtgcc ataatcaact    501120
     gtaggaccct aaaggaaata agagtctccc ctcaacccct gaagagtgtt tgggttcacc    501180
     gtgtgtccaa tgattctgtg cctcttgagc tccaggaaag ggctccctgg tgatgcatga    501240
     gatcttttct tggagtctct ctgcagagtt cactgggttt cctaaaggca attcactatt    501300
     tcaaaagatg gtgtgaggag catgtggtgt ccctaaagga gaattctgag ccagggcaca    501360
     accactttat actgagctgg atacactggt aggaatatac tctgtcagct cagatagaaa    501420
     cctccctgca tggttggggc tgggctgcag ggggcgctcc ggatacaccc agcacaggct    501480
     cccgccccag agcaggtgca caggaggctg gggagaggtt cctcccaggg cctgggactt    501540
     cctttaaaaa tatctaaaat aagtatttca caaagactgc tgaagtttgt ataaatatct    501600
     attcaattgt gagcatttat caaactggat gttgtaatga gaaccacttt tacaatgggg    501660
     atttcaaact ctgctggagg tcaggaagag atcctttctt ataaataaat gcaatttttg    501720
     gataaacaca gtcattccct aaataacgca ttcacatatt atggtctaga aatgatgcaa    501780
     gttgaccctg agacagtcaa atgtggtttc aaagtgaggt gctgtccttg aggagcttgt    501840
     tctccagtgg gggaagctct gtcaacacag agttcaggga tgtgtagggg acacatggcc    501900
     tctaacagga ttacggcttg aaccctcagc ttctacagtt gtgtcaccca tgtgtctgtt    501960
     tctcatactg ggtcaggaat tgggctatta aatagcatcc ttcatgaata tgcaattaac    502020
     tgaggtgact atagtatctc cgttccctga gagcctcacc caacaaccac acccctcctc    502080
     tggagaagcc cctagatcac agctcctcac catggactgg acctgaagga tcctcttctt    502140
     gatggcagca gcaacaggta aggggctccc cagtctcagg gctgaggaag aaaccaggcc    502200
     agtcatgtga gacttcaccc actcttgtgt ccactccaca ggtgcccact ccctgcagct    502260
     ggtgcagtct gggcctgagg tgaagaagcc tggggcctca gtgaaggtct cctataagtc    502320
     ttctggttac accttcacca tctatggtat gaattgggta tgatagaccc ctggacaggg    502380
     ctttgagtgg atgtgatgga tcatcaccta cactgggaac ccaacgtata cccacggctt    502440
     cacaggatgg tttgtcttct ccatggacac gtctgtcagc acggcgtgtc ttcagatcag    502500
     cagcctaaag gctgaggaca cggccgagta ttactgtgcg aagtacacag tgtggaaacc    502560
     cacatcccga gagtttcaga aagcctgagg aaggaggcag ctgtgctgag ctgaggcagt    502620
     ggtacagcag ttttctgaac ttccatagta tctcattttg cattgagttc cgctttaata    502680
     ttagccaaga atatgggata gacgggtgct cctaagagat ccttaacttg cccattttga    502740
     tgggttttcc caaagacgtg agaagccact tttttcgcaa agcatcccaa agccatgccc    502800
     tgctccagaa acacgtgtat ccatttcctg gtctttgatt aactgacaaa ctctcatcag    502860
     cgcacctggg ctaatttcac atcaggtaga aatatgtgct ttaaagcaag gctaacgttg    502920
     taatagcaat tcctgcttaa taaccttcag cattgttgtt gtgtgctcca tcaactaatt    502980
     acgttagttc aaggttctca atgggagttt ctaataaata taagggatgt atagaagttc    503040
     ccctaattaa aacaattgtg aagacaacct cagtgttcaa ccatatttca acccttcacc    503100
     acaaaggaac tttcatctct cctggaagtt gggttcattt tcaaattagt ttttttattt    503160
     taatatctca agattattgt atgtgactat tttagcagaa agtgaattat gggaacttga    503220
     actaaccaac tgaaaataca ttcagaacta attaaacaag atgccagaat gtgattggct    503280
     ccaggcattt taaattcaac aggttatgta accaggcttt aaatttgcac atcttcgtgt    503340
     taccttcatg acacagtcaa ctcccattat gtaagaaatg gtgagtgcat tcccaagggt    503400
     cttgcacagt tataaaaata gacttgatga ggtgaggagt tgtttaaatt cccctctgaa    503460
     gaagcagcat caacccaaca aaccactctc ttccctctgt gactagagct ctgtcacagg    503520
     ccacatggac ctaaatcctt gatggagatt acaggactac gtaaattgga ctgatcgttt    503580
     ttatgctgtt aaattaatag gtgagtctgc actccagcct gggcaacaga ataatcttgt    503640
     ctgtaaaata caaaagaaag ataaattaat agatactgac tttgacattt cggataataa    503700
     tattttcata aaccgaattt aattataccc acattgttac ctacaccttc actgaaaagt    503760
     tcctagttat gttgagttcc atcaacactc cacatgttca aatctggaca tccaagagag    503820
     tctagagaat aaaacgcaat gagggcagtg aaacttgcgt atattcagca cctcttaact    503880
     caggaggact caatacaccc tggaacactc tgcttttctg aatggctcac aatgactcca    503940
     gctcactctc caacctcctc aaacatctgg cctctgtttg ccctaagttc acgctctgct    504000
     cttagtctat gttctgaagt ctttgtagag gtgaaaatga gctgtcagat ggatcttcct    504060
     tctcactgca acatggaatt tgctatttca cttaatgacc actctttcca caatggttga    504120
     tttcttttgg cctgttcatt actggtgatt ttcaagggaa tctcagttga atctttactg    504180
     ttttgcattt tgtctccatg acaatgttgg gaagtttttc ttctagcagc ataacatgat    504240
     ctagtgacct gacacatttg cagcaaacaa tacctacaaa ttcagaagct ctttggtttt    504300
     ctttccacga aatataattc ttgctcttct gtgtatgagc acatcctagc atccctgtac    504360
     acacccacgt agatgtctac acgccgatga aatattccct gtaaataaaa aaagtatctc    504420
     agtttctctc aatgttcata attctcctga gggtgaggaa ggtacttctg ggtctgctca    504480
     aacaaatggc ccagagacca cctggtaggt aggtaaggag ctcacctcgc tctggatatt    504540
     gagtctgtct ctttccctct gtcgtctcat agaaggccag cccacttgtt cagctcctaa    504600
     gaagagagcc caggtttatc cagattatac aacacaacca gcttctgatg actctcctgt    504660
     tacaacatcc atggagatat tttgtgtatt atataattca ccaaactaat gtgaaatgcc    504720
     caagttgcaa tactgcacac cctagggtat gttcttgcaa ttcagcggag gagaaattct    504780
     ttcagagaca gatggatctg aattggtaaa tatgtgggta cgaattctgg gcttgagtgt    504840
     cattgtccag ccatgtttca caggtgtgac ctgtcaggga agaaccagag ttccttgttc    504900
     tctcagaggg tagagctcac agaggtcctc tctggttccc aggaaaggta atttcactaa    504960
     tcttggtgat gagactatcc tccagtgctg atgtactata gagttttcat ctgaagctgt    505020
     cactgctatc cccaatgtac atcttttcac acagaaatgt ttagaggtca ggccatattc    505080
     tcagggttac acattgagaa ggatggagat atattctact accttctcct gagatctcac    505140
     acacaatctc aaatttcaaa aggtctcaga agggcagctc tcaggtacta tttaaaaata    505200
     acccacttcc tgggacaggt agcatccttc taaccatgat ggatgttctg aactacagta    505260
     cacattgcat ggatccaggt ttgtctcaat tcactgtgat tattacactc agcagctgtt    505320
     tcaatatgtc tgaaggggta aatgacaatt taggtgacct gggtgtatgg ttggtgttat    505380
     atgaatcttt aaatgtagaa cagtattaac tgtattccaa aatctgtctt tgatccatga    505440
     tcacacttgt ctcccagacc agctccttca gcacatttcc tacctggaag aagaggactc    505500
     tgggtttggt gaggggaggc cacaggaaga gaactgagtt ctcagagggc acagccagca    505560
     tacacctccc agggtgagcc caaaagactg gggcctccct catccctttt tacctatcca    505620
     tacaaaggca ccacccacat gcaaatcctc acttaggcac ccacaggaaa tgactacaca    505680
     tttccttaaa ttcagggtcc agctcacatg ggaagtgctt tctgagagtc atggacctcc    505740
     tgcacaagaa catgaaacac ctgtggttct tcctcctcct ggtggcagct cccagatgtg    505800
     agtgtctcag gaatgcggat atgaagatat gagatgctgc ctctgatccc agggctcact    505860
     gtgggtttct ctgttcacag gggtcctgtc ccaggtgcag ctacagcagt ggggcgcagg    505920
     actgttgaag ccttcggaga ccctgtccct cacctgcgct gtctatggtg ggtccttcag    505980
     tggttactac tggagctgga tccgccagcc cccagggaag gggctggagt ggattgggga    506040
     aatcaatcat agtggaagca ccaactacaa cccgtccctc aagagtcgag tcaccatatc    506100
     agtagacacg tccaagaacc agttctccct gaagctgagc tctgtgaccg ccgcggacac    506160
     ggctgtgtat tactgtgcga gaggcacagt gaggggaggt gagtgtgagc ccagacaaaa    506220
     acctccctgc aggtaggcag agggggcggg cgcaggtact gctcaagacc agcaggtggc    506280
     gcgcggcgcc cacagatccc gaggccgggt ccggagcagg tgcaaggagg gcggggcttc    506340
     ctcaacagct cagtggtctg tctcctcgcc agcacctcag atgtccccag gactctcttt    506400
     ctttattatc tgtggttctg cttcctcaca tccttgtggc agggaagaaa ggaggaagac    506460
     aatttttctg tttactgttg aggtttcacc aattactaga aactttccta caagttcctg    506520
     catgactcat tttgccatat atggattctc accatctctt gatttgtttc atcaatcgaa    506580
     ttgtgcccta tttgaaatta acttactgaa accttaaatc caatggatct atactggaat    506640
     tttaatgatg taattgaggt taaatgtggt caaagtgtga gaccctaatg caataaaccg    506700
     ttgtctttat aagaagagga agagacacca gagacctctc acttttcacg tgcacacaga    506760
     gaagaggcca tgtggagacg tagtgcacta gaaggtggcc ctgtgcaagc caggaagaag    506820
     ccatactaag aaccaatttt gccagctcct tgatcttcga cattcagact gtagaattgt    506880
     aagaaaatca atatttgttg tttaagccac ccaatcctgt tgtcttctta cgaagaccca    506940
     aactgactaa taccacctaa ctctgttagc tctgtctcct ggagggagaa gcagccccct    507000
     gaggctgggc actgcatctc tcagatttcc acgtgaagta ggcaaaaata gtagttctca    507060
     tataaaaatg tgtcatggct ctgttggcca tttttgagca tggtctctga aaccagccct    507120
     ggtgtgtgtg tgtaacaaat gtcccttatc ttttatttgg acataacaaa tagacgatag    507180
     gtaccagctg gatggagatt ggccactgat catcttctgt tctccttagt atgtcacaga    507240
     aaaccacacc aacatcacca gcatcactgt ttttcttcta ccacctcaaa cggactacag    507300
     aaatgatccc tgcagtatgg tctatttctt cggctttcga aatttgcact gaatctcttc    507360
     ctaaatgggg agctacatgg ggtctgagtt ttgttccttt cttcccagtc ttccccaagt    507420
     accaaggaca gaatagactt aaaatttggc catcaatgcc cccaaacacc acatcacttt    507480
     ctaaaatcca catcctgcat ccatccttct ctggacaccc ctcatcaggc tacccaggaa    507540
     tggccagaat ctggtatcag cttatgggct gaggccacga gttatacaca tgtgtgattt    507600
     cagtcacaca cactctactt caggaccaca cctgtgttct gaggagctca ggcacctgct    507660
     gatctcagta attctctaat aaatcacaca cctcttatta ataaaggtcc agatggcccc    507720
     atcagctgca gagcagtgga gtaaagctca tgggtgggtc catcaggcag aagtcagaca    507780
     atggaaggga tggctggtca cttccatttt cactgatgtt cgcaatgaat tttaatggaa    507840
     taaaacaata ttaactacaa agagacagaa aagaagactg gctcgtcaag atatttaagg    507900
     acaaggaact ttatttgggg ggaaagtgaa agacacttct aaatggaaat ccctaaagca    507960
     catacagcag ctgacagagt ggccactgtg cacatgaggg ctgaggagat ggatggtaga    508020
     gtccattcct gcaacatgtc tctgggtgtg tgatggttag actcctcatg catatgaata    508080
     taagagctgg actcatggag aaaaaaaggg ccatatccca taggaaagga agaaaaaaaa    508140
     acagatgggc acccctggag agagctcatt agatttggtg tgtttaagat gaaaatttct    508200
     tccaaaaatt gtaatgttct aagctaaata tgaatctctt caataaactg agaataggag    508260
     aatggagcta ggacttgaga gaggaaacaa atcttgagag agcagaaagc aaatccacaa    508320
     aaactgtcac atgacagagg tcagaatgga gctgatgtag ctacttcact attctgcaga    508380
     cttattgacc atgtggagaa ggggcttgaa caaatggaga cgttctccaa ccttctgaat    508440
     cagcctcctt cttatgcatg agtagaaatc atagttctgg gggtgacctt cccaattttc    508500
     ctgtccatac ctcttccccc aggggtagag tgtcttccca accacaatgg tttctcacca    508560
     gtgttctcag cttctcctcc attgtactta ccctgcagat taagaatttc ttctagatgg    508620
     agttctttgg gaattttctg ttttctttag tttctgttga ctccaccaca cccccatagg    508680
     atacaggttt tattggattt cccctggaaa tagtgggggt ggatccaggc gttcaacagt    508740
     cctcgctgtt cttcccttcc tgtcagcacc acaggacagc agataaggga gttgactgtg    508800
     gatttttcaa attcctggga aaagcctgca aggactagta gattcacact ctaataccat    508860
     tagcacatgt atccaaaaag aaacctcact aaatatttcc aggttagcct gttcctctct    508920
     cagtgccatc cagtggcacc tgccctgggt tcacgaacat gtgggcccca gtcctctctg    508980
     ctggcatctc tctcctcaca tttcagtctt ctcgttagct ctgtgaaagc agctcagata    509040
     tgttaaaagg ttttcttctt catttattca gtttttcagg tttactgtta aggaggtcag    509100
     aataagatca tagtttcctc atttttcaca tttgcacgct gagtagccac tttctatata    509160
     aaagccagaa actaagggaa caaatcaaat atccatatcc actacaggtg aacgttaaaa    509220
     aatttgacat atgattatgg actaaagtac aatgcagaat tagaatcaag gcatcctcat    509280
     tctcataaaa gcatgtctac attctcaaat aactctgctg agtgaaagta gctgaagaat    509340
     taagagtgca cttcataaac ttctaattgt ataaactgca gaaggtacaa ctattctaaa    509400
     gtaacagagc agatttgaaa tttgggagaa atgggtgttg aaagtaattg ggtggtgaga    509460
     tgaaattaca gagaagtgac agaaagattt aggggttaac ttaattgtac acaacctgat    509520
     taaagtttgc acacatacgt taccattttc caaatggtgc agtgtagatt tgaattaatt    509580
     attaattgta cttaaagcag taacaaagaa acaaatgaat atgttggttg aggaggagca    509640
     aaaaatagat gggtatgaac actcaaaaca tctcagactc ttgaaagtac acatgcttga    509700
     acactggttt tctctgtata ctttaggtaa agagcagaat acattaaaag aaaacagaga    509760
     tgtcctggcg ggggtggaat cctgcaaacc tcactaggcg tgtcccacac tgccctggag    509820
     ttgtctcagg ggagcagtct cctctagtgg tcagagacac aggcatggat aatggggcta    509880
     atactgtcca gctgtgtgac cttgaacaca ttgtataaac actgttccat atgtaattta    509940
     tcttccttaa aatgtaacat tgacacttgc attaaatgta ttctacaaat atgtcaaaaa    510000
     gaagatgacg actgctaaat gattatcaag gcacaatcat ataatataat gatattttcc    510060
     tgagtgataa gatgactacc aatctccccc agggcacttt gtctgctctg agccctgcgc    510120
     ctcctcagga ttcccatccc agagcttgct atacagtagg agacatgcaa ataggtttct    510180
     ccctctgctg atgaaaacca gcccagtcct gaccccacag ctctgggaga gaagcgccag    510240
     ccctgggatt cccaggggtt tctatttggt gatcaggact aaagacagag gacccaccat    510300
     ggagcttggg ctgagctggg ttttcactgt tgctgtttta aaaggtgaac tagagagatt    510360
     gagcgtgagt ggataccctt gagagaaatg gtggattatg tctgggagtt tctgaccagg    510420
     atgtctacga gtttgcaggt gtctagtgag aggtacagct cgtggagtcc ggagaggacc    510480
     caagacaacc tgggggatcc ttgagactct cctgtgcaga ctctggatta accttcagta    510540
     gctactgaat gagctcggtt tcccaggctc cagggaaggg gctggagtga gtagtagata    510600
     tacagtgtga tggaagtcag atatgttatg cccaatctgt gaagagcaaa ttcaccatct    510660
     ccaaagaaaa tgccaagaac tcactgtatt tgcaaatgaa cagtctgaga gcagagggca    510720
     cagctgtgtg ttactgtatg tgaggcacca ggtaagagga catcagtgtg aacacagaca    510780
     cagtttcctg caatgacaag ggaggaggct gggctaaaag gggcactcag gacccactga    510840
     aaacgggcag ctctagggca ggtacagatg gttatcatgg gctgctttcc ttcagggtct    510900
     gtggcctcct ctgcatctac cagttcccct cggagcctct ggacatttat gtttctgtgg    510960
     ccacccctga catctctgga ttagaaaagt ttattatagg aagaggaaac attctcattt    511020
     gtcccaaagc agatgtaagt aatggaagca aaaaatgcac aggaggccag gcggggctgt    511080
     agatactgtc accccagaat gtcaatctca ccactagtac tggagaggga agggagtttg    511140
     atggaccttc cctaacaacc ctgtggtcca agataagtcc aggaagacca ttggtgcctc    511200
     ccagagcaca gttgtccatc agggatgtcc aatgcgtccc agcagcagcc atgcctcagt    511260
     gtctccactg tgcacagcca ctgtctggga ggagctccca ggatgggtgt ctttggcaca    511320
     caccaggtgg cgggtgttag agtgcagtgc agcagctggc tgcctgttct attgggctcc    511380
     ctgatgctgg agagatggga ggtgcattcg cagggccagc accctgtttg tgaattttta    511440
     tataaaaacc ctgattttac ttcatttttg cagatgacat agataactaa aaacagaatc    511500
     tgcaaagaaa ttgtaatttt caactttacc ccaaattcat tgtttcttaa ttctgtgcaa    511560
     gatccagaca tattattgcc ttcctcatga taatttattc tatgtaaaac cgaaatcatt    511620
     ttttctccta ctctttgttt ctgttcaagt acagagatct tgagcaaagt aagttgggtt    511680
     ctttccacac actaaccctc accttcccca gagaatgagc agagattgtc ctcagtctga    511740
     gtctaaggga ggagctgttt ctgcacaact cagagcctgc agagaccccc aggtgcagct    511800
     tcagtgagtc agacatttct ccatgtgggc gacctccagt gcctgtgact gctgctcagg    511860
     cctaattgtt ggttaagtat tacgacaccc tttaggtgat cacatctcaa gcctattctg    511920
     aaaatcacca tgaacagaga tagttcaatg gccattctcc tgacataagt ttctctttat    511980
     tacttggttc caagtatgga gaaaaatgtg accctatatt tgtctgaatc caacatcagc    512040
     atccacggca ttacgctagg agactcacta attgagcaca agtgagctcg ttattctcat    512100
     agaaatgtaa gtatttggaa atttcagcgt gttctccaga acctgtggct gccaacaact    512160
     gtatttctcg gtgcactctt ggcctggtga agccttcaca gaacctctcc ctcacctgtg    512220
     ccatctctgc attctccatc acaaccagtg tttccttctg gagctgcatc catcagcgcc    512280
     cccatgggag gtactggagt ggatcgggtg cataggtcat ggagggagca cagattactc    512340
     ccctcttctc aagagtccag tcaccatctc cagatccatg tccaaaaagt acttcttctt    512400
     acaggtgaac tatgtgagca acaaacacat agccatgtat tttagagcaa aagacacagt    512460
     gaggaaacca cagtgtgaac tcatacccaa gcctccctgt gggggtgcat aggacagcca    512520
     gggttactca ggacaccagg ctccctcagg acaccaaggg gcactcaaga ccattgtaga    512580
     ggcatgcagg tagctggggg ctctcaggaa ccatggggga aaatcaggac accaaagggt    512640
     gcttggtaca gcagggggct caggattatt gtggggattc agaaagagca ggttcaaggc    512700
     tcaacctcag ggcatgtgca gctggtgtga aaaggggctg gatgaggcgt tttgtgtcac    512760
     catcatgttt caccaccaga caccctccac tacctctatt ctaatgcatg tgtttgtatg    512820
     attagaaaat gatattgata taaatatata accatagcta ggtgtgtcaa gttgtcctct    512880
     ccatcttata tcagccttgt ctgtaaggac taattcccca taattacttg agaacctcat    512940
     aaattgtggt caattatgta ggattcctct ctttttctgc cttccttcct ccctccttct    513000
     ctctctcaca cagaaactta tatacagcca ccccacaaca cacataaatc tataactttt    513060
     attacctgat gtattgataa gtaatctaaa gttacataaa aatcagtagt tcactgtcaa    513120
     tattgtagga gaagggaagg tctaggaagg aggaattaca gaacaagagg atattttgag    513180
     ggtaattgac ttgttatcta tgtggataat gataatgtct atgaccatat ttgtaaaatt    513240
     gaacacttcg tgtggagact attatttttt tatttaaccc catgccatta ttttgccgta    513300
     ttctagcatt tctctaaaaa tacagaaatg ttcagcactc attcatgtgt atattcagga    513360
     gtttctgact tttcatgtat tttatttatc tctgtctaat tgctttgata ccaaatttta    513420
     cctagtataa ttagtactac ctttagcact gattataagt ctcactcctc catcatctcc    513480
     ttttttgcca cacaagctga atctagtttg gactcatagg agctgcttca ttcaatgcca    513540
     gtgggagttt caaaccctat caacccccct ataaactgtc attaagaaag tttatcaaac    513600
     tccaccagtc atgtgatatt agttcgtttt cacagtggta taaagaacta cctgagacga    513660
     ggagtttaca aagaaaagag gtttagttga ctcacagttc tgcatggctg gggaggccac    513720
     aggaaactta taatcatggt ggaaggtgaa ggcgaagcac ggcacatcac acagcgcagc    513780
     aggagagaga gggggaagtg acacatagtt ttaaactatc agctcttgtg agaactcact    513840
     cactatgatg agaacaacat gggaaaactg ccctcatgat ccaataacct ctcacctggt    513900
     ccctcccttg acatgtgggg attagaattt gagatgatat ttaggtgggg acacaaaaca    513960
     aatctatagc acatgtccag ctgtgtcctg gagttgtttc agggatccag tgtgtactgt    514020
     tgatagaaac agtgatcatc attctagccg gtgtgagatg gtatctcctt gtggtttaga    514080
     tttgcatttc tctgatggcc agtgatgatg agaatttttt catgtgtctt ttggctgcat    514140
     aaatgtcttc ttttgagaag cgtctgtgaa aaaaaaaaag aaacagtgac accaagctca    514200
     caccatccgt tgtagttgac aacatgcaaa gccaagagat ctcaactgag atttagtgtg    514260
     tgtgtcatgt ctgatgaagt cataagctca gagcaagtga atatggaaaa gtgtattatc    514320
     tgcacagtgt aggtgtctgc tgagtgcagg gcaggtctcg caggaaaatc taaaatggct    514380
     tgaaagaaga ggaaaggaga ctggctcagg gtttttataa tggtttagtg gtggtggcac    514440
     agtgaggctt cccactcaca gatcggggtt tataaggttt gaaactccca ctgccattga    514500
     atgaagaagc tcctgtgagt ccaaactaga ttcaccttgt gtggcaaaaa aggagatgat    514560
     ggaggagtga gccttaagta atcaacaggc atgcaccaaa agatagagcg acaacttatt    514620
     ctaggtagca agaataaaaa taatgaaaaa gaaataaggg ttcagtatgg gtggacaaga    514680
     cccagatcta cagaaatgag atgactttag aaatataagg aaataataat gagaaaaaga    514740
     aggaggggat ggggaattag ggtcctggcc taatgtcttg ggtagaagct tctcacaatc    514800
     aaggactatc agcttattct gcaggtcttg ggtcacacgt ctgcttaaaa acatcagaaa    514860
     tgccaggcaa tcaatgagga tgctcagttt agcatctcct atttgagtag atttacagtt    514920
     gtgtggaatt cttaattgat tctttttggt ttgtttttag aggcaggctc tcacactgtt    514980
     ccacagctta aagtacagtt gtgtgatcat agctcactgt aattttgagc tccggactca    515040
     cgtaatcttc ctgtattagc ctcctgaata tctagaatta gaggggaacg ccacccctca    515100
     cctgcttatt ctttaaaata ttttttcata gaaatagcat ctctttatgt tgcccaggtt    515160
     ggcttcgatt tcctggtctc atgtgatttc cctcactttg cttctgaaag tggtgtgatt    515220
     atagggaaga tccactgcat ctgtcctgaa ttgattcttc agttgtaaaa catgaagcca    515280
     ataattaact gcctgactgt tttgtgcagt gagttagtta aaagtttctg ataagattcc    515340
     ttccaatatg attcaagagc agtattttct gctgatgttc ctttcagttt ccttgttgaa    515400
     gatcagaaga cactgcggaa aagatgtagt aaaaaggcag ttttaactct tcatgattgg    515460
     agtggatatc acataggaat ccctttccgt attttgtaac acatgcatgc aatagaacaa    515520
     agatgatccc ttggtgtaaa atctataaac gtatgggcct tttagctacc aagtcatagg    515580
     gtaataactg atggaccctg aggagtgaac catgattcca tagtgctagt gggagaaccc    515640
     ttggccaagg aagtttacat tttattaaaa ttgataattt ttatgtagcg atgccatttt    515700
     ttaaacattc ccagaaggtt gtgagtggga ttgactctgt cttctgtgaa caatgacagt    515760
     gccctccatg gttagataat attttaaact agattgagct agagtggttt ctgtattgaa    515820
     tcactatatt tttttagctt catgttagct ttttgtgtgt tagcatttgc tttaaaatga    515880
     tattaatcag ccctctagta ggtagaaatt catctgaggg tttcttccct tgttgtccat    515940
     ctcaatagga tttccagaag acgtaagaac cctctctgtt tctaaaaata ttccaggctg    516000
     ggcgctgtgg ctcatgcctg taaacctagc actttgggag gccgaggtgg gtggacccct    516060
     cgaggttggg agtttgagac cagcctggtc agtatggtga aacctcatct ctactaaaaa    516120
     tacaaaaatt cgccgggcat ggtggtgtac actcgtaatc ccagctactt gagaggctga    516180
     ggtgagagga ttgcttgaac cctggaggcg ggggttgcag tgagccgaga ttgtgccact    516240
     gcacttcagc ctgggcgaca cagcgagact ccgtctcaaa caaaacgaca caaaaaattc    516300
     caaagttgtg caccctctaa aagcatatgt acttaattct catttttaat ttattaaaca    516360
     tctctaataa gttcaatgtt tcctgccttc tgagttgatt tcctaacaca tagaagaata    516420
     tatcctaaat gaaagtttgt gttcttaata caaattacta gttaataacc tttactttta    516480
     ttattgaggt attatacatc aatgttaatc aatcctctca atgggactct tacctaaaga    516540
     atatataaaa tattttcctg atcatgacat ataatagatg tgaacacatt cttagcattc    516600
     agccatgtct cttgtctatt aatattataa accacatgct aactttgatt ttattggtaa    516660
     ttgttctaat ttcttttttt tcccacttct ctggttgttt tttttttaac tttaagtttt    516720
     agggtacatg tgcacaatgt gcaggttagt tacatatgta tacatgtgcc atgttggtgt    516780
     gctgcaccca ttaactcgtc atttaacatt aggtatatct cctaaagcta tccctccccc    516840
     ctccccccac cccacaacag gccccagtgt gtgatgttcc ccttcctgtg tccatgtgtt    516900
     ctcattgttc aattcccacc taggagtgag aacatgtgtt gtttggtttt ttgtccttgc    516960
     gatagtttgc tgagaatgat ggtttccagc ttcatccatg tccctacaaa ggacatgaac    517020
     tcatcatttt ttatggctgc ataggattcc atgatgcata tgtgccacat tttcttaatc    517080
     cagtgtatca ttgttggaca tttgggttgg ttccaagtct ttgctattgt gaatagggaa    517140
     ttgttctaat ttcaaactag ttaattttta tcttcatgca gctagattat tatgtgtggc    517200
     tatttattcc gagagggata aagacaacat taacaatttt cactgcaggc atgtctaggc    517260
     aactccctgt gcactatgac cctggggcgt tggagattct atggggactc ttccctacct    517320
     gcctaggaga gttctctgcc ttctacctct agcattttcc tctttgaaga agtacatcta    517380
     actgtcatta gaatagagac aaagacaagt cttaactgct tccagctgag gagggatgct    517440
     gtttggggaa gatctctctt ggaggtctaa gggaccccag gaaaagggag ccattatccc    517500
     aggcttcagt tgcatgacca tttggagttt gatggtctga aaatgagaag aggcaaatct    517560
     ggttattaga agacatgtat gaaaaccaaa caaggtggca aggacagctt gaaagaaaat    517620
     tccaaggctg ctgacattcc tagataactg cagctgtagt tatgcctgct aaggtttggg    517680
     cgcatggggc ttggcttttg tcagctccct gggatttatt ttcccaaaca aagaaacctc    517740
     caggttaggg gcaccctatt cattcccatc acctggcatg atttaaagga taattgctta    517800
     gaattaaaat attgatccag attttttata ttccccatcg ctttttgttt cttctgggct    517860
     gtagccagag atcattgatt ggcgctcagg aataagcaga gttagtctaa aatgcaggca    517920
     aatacttaaa caactgaaga gattagaatt taaagacaag tgtatgatat gttttgaaat    517980
     acaatgtttc tctttccagt tttggttttt gtcggcagca aataatgata agactgagtt    518040
     gtttgcaaaa taaactttag tcttaaactt ggcctgatta tttgcataaa gtgcagcaag    518100
     aatattaata ataattctgt aggaaaagcc tgcaagcacc aggagcttca cagtctaaca    518160
     ctatgagcac gtgcatcctc acgcaactca ctgaatattt ccaagccagc ctgttccgat    518220
     cttaaatgcc atccagtggc atctgcccca ggtacactaa tacatgggtc ctgcttctct    518280
     ctgcagcctc ctctctcctc agatttcagg ttttgtttat tgtttgtttt ctctctgaca    518340
     tcaacacaga tatgttgaag gttttctttt ttttatttgt agttgttcag ctttgttgtt    518400
     aatgaggtca gaataagctc atagtttaca catttttaca ttcccatgcc gagtagctgc    518460
     ttttctctat caaatccatt aactgagaga acaatcacat ttcgttacag gtgaacagtt    518520
     aaatagtttg gcatatattt ctgtgctgga atctaatgca gcttgaaatc aagtcatgcc    518580
     tcactcattg aaaaaaacat ggctaaattc tcaaagaatt gtgctgagtg aaagaaacta    518640
     aggaattaag agtaaatttt acatgataca tttgtagaaa ttttagaaga tgccactatt    518700
     ataaattaac atggagaaga tttaaatgtt tctgagaata tgctattggg agtaatgggg    518760
     atgtgagtta aatttcagag gaataagaga aagatttagg gattaatttt ttcaaacctt    518820
     gattgaagtg ctgagtaaat ggttgcaaac ataggtctac atttttcaaa tcattcacca    518880
     taaatttgaa ttatttatta attacactcg aataaagcaa taaagaaact gatgagataa    518940
     tatttgactg aattgcatca ataaatagat cgatattaac acaaggaata taactgattt    519000
     ccaaaaacat acacatgaac catggttcac tctgcgtatt tagataaatt acagaaagtt    519060
     gtcataacag atggggaatc ctgcagactt cactaggcat gggccatgct gccctggagt    519120
     tgtctcaggg gagctgcctc ctccagaggt tagagcacag gcccaggtaa taggactaaa    519180
     tttttagatg tgttatctta gacacactgc acaactgctg tgttctctat gtaaattatc    519240
     tcctgtaaaa tataacattg aagcctgcat taaatatatt gtgtaaatat gtaagaataa    519300
     aagaaagtta tgagagctaa gtgttaatca aggcacaagc atataagata taactatatt    519360
     ttcctgaatg atggaattac taccagtctc ccccaggaca cttcatctgc cctgagccca    519420
     gcctctcctc agatgtccca cccagagctt gctatatagt gggggacatg caaatagggc    519480
     cctccctcta ctgatgaaaa ccagcccagc cctgaccctg cagctctggg agaggagccc    519540
     agcactagaa gtcggcggtg tttccattcg gtgatcagca ctgaacacag aggactcacc    519600
     atggagtttg ggctgagctg ggttttcctc gttgctcttt taagaggtga ttcatggaga    519660
     aatagagaga ctgagtgtga gtgaacatga gtgagaaaaa ctggatttgt gtggcatttt    519720
     ctgataacgg tgtccttctg tttgcaggtg tccagtgtca ggtgcagctg gtggagtctg    519780
     ggggaggcgt ggtccagcct gggaggtccc tgagactctc ctgtgcagcg tctggattca    519840
     ccttcagtag ctatggcatg cactgggtcc gccaggctcc aggcaagggg ctggagtggg    519900
     tggcagttat atggtatgat ggaagtaata aatactatgc agactccgtg aagggccgat    519960
     tcaccatctc cagagacaat tccaagaaca cgctgtatct gcaaatgaac agcctgagag    520020
     ccgaggacac ggctgtgtat tactgtgcga gagacacagt gaggggaggt cattgtgcgc    520080
     ccagacacaa acctccctgc aggaacgctg gggggaaatc agctgcaggg ggcgctcagg    520140
     agccactgat cagagtcagc cctggaggca ggtgcagatg gaggctgttt cctgtcagga    520200
     tgtgggactt tgtcttcttc tgacagttcc ccagggaacc tcttaaattt agaaaactgt    520260
     gcctaacaat gtcttctcta tgcatatgag gaccttttct ccctggcaca aaatgcagat    520320
     tgacgctgac acggatgaaa attcctcaac catggtcaca aggatcagag tcctgagtaa    520380
     cctcagggct tcctggtgat tcttctccaa tcagacccag gacagggacc tccgtgagat    520440
     tccctgactg gaacagtctt tatggatcct ggtcacagac aatagagagg ctgaaccagg    520500
     gtcagcgtca tgtagaacgt cacagatttc acgtctgatc cttctcctga cacgaaagta    520560
     tgcaaatcag tatcagcacc gatctggtgc ttcttttgtt cctaatccat ttactttctt    520620
     ttttcgtcgt ttttctcctt tttccatttg tttttcctgc tttttgcaaa aggaagatgt    520680
     tttccctgtg agatgcaggg gatgacaatt ttgggagatg gctggaacat ccaatatcct    520740
     cagggccgac catcagtaag tgcaggctag aagtctcaga aagagctgaa gctgcttaat    520800
     caccgtggag ttttaccttc tccagttctg ctctgatgga atcagggcca agcaggttat    520860
     caatgataat ctacctaaca tagagtcaac tgattccagt ttcaataacg tctgttaaaa    520920
     attcacacca ccacctggat tactgttttg tcaaatcaat acacagtatt gtccagctaa    520980
     gtagacccaa agacggaccg ttgcccatgg agaaaaacat taacctgagt tctaggttct    521040
     tacagtgtta aaggtgtaaa actgattatt aaaaatgagg ctatttttct ttttgctgtt    521100
     gagttgtaga agtttctttt ccattttgac atgaaaactt tttcagatat atggcatatt    521160
     atccaattct gtaagttgta gttattttgt tgctttgcag aatctttttc ataatctatt    521220
     cccacttgtt caattctgct tttttttgta ggtgatttga atgtaaaatc ctgaaaaaga    521280
     ttgctaattt tttgagtgtt gagagtttta caattagagg tattacagtt agatatttga    521340
     ggcatttgga gtgaattttt gtgtttattc taacctaaaa ttcttaattc ttttcatggg    521400
     aaaatccagt tttcatacca ccctctttgg aagacactac aatttagcca tgttatattg    521460
     atggttctca tgctaaaaat cagcttgtca tcaatatgtg ggtttatatc taagctctat    521520
     ataggtattt aagccaaaac tttctatgtt tttaataaat gttgaggcct ggaagtgaaa    521580
     tgcctaaagc tttcttcttg ccttgttaca gatattgtac caaaatattc taacctttta    521640
     ctattgagtg taataatagc tgtggccttt cttaacggct tttattatgt tcaagttgtt    521700
     ttcttgtctt cctactttgt tcatagtttt aataatgaaa ctgatttttt tcaaagtctt    521760
     tttctgtgtc tgatgaaatg ttactgagat attttttctt tagtttttta atgtgtacca    521820
     aactgattga tttgagaatg ttgaatcaag tatgcatctc aggaagaaat ttgagttggt    521880
     catggtgtat gtcttctaaa acactttgga gcttagttta ctattgttgg ggattaattc    521940
     atgtctacta atgatattgg tctgtagttt cctttattgt ggtgcctttg tctattactg    522000
     gtaatactat catggtagtc tcatagaaag agtttagaag gtgtatggca gactaccttt    522060
     aaaatagatt ttatcagtgg agaaatggtg atagtttttt cttcactttt ctgttgggaa    522120
     gaattttatg ttgtttaaaa gatattcaga atgacttaac ctggtttatg agctttcatt    522180
     ctattccttt cttccattct ttttgaagaa ctgcttacct ttcctattta tttttaagtt    522240
     tgtttttaga catatgcaat acattttgag gtgaaacctg gtggaatttt ttccaataaa    522300
     ttagtaaaaa taaatcattt aattgactat tttattcagg ttgatttgtt taatatttgc    522360
     taaaggccag ttctttaagc tatgacacat aataaatccc aaatggcagt acctcattgt    522420
     ttacttagct tttgtactta tatttttcag aggaagaacc actactgtaa attgtaaata    522480
     gccaatacat aattgtattg tatgcaaatc tgtgactgtt tacagtgtca tctctgagaa    522540
     acataaagtt tatttactat atatatataa agagtttgga aggtggactc caccaatttt    522600
     tgaaagagtt acagaagggc tgctattact tctttaaatg ttaaggttca ttttatgatg    522660
     taacagataa cctatcatgg agaatgttca atgtgttctt gagaaggatg tgtattacgt    522720
     gactcttggt tggaaggttc tgtaaatatc attcagataa atttgttcaa tagtgttgtt    522780
     caagttcaga ggcacattga gaattttctt tctggatttg ctaaacatta tggtcatgag    522840
     gtattaaggt cttgtgttat ttttgtattg ttttctattt ctttatatct cttacagttt    522900
     gcttaatgca gttatatttg tatttgtaca catgtgtaaa aacaaaaatc ataattgcta    522960
     aatgagtttt atggcacagt cacattataa gtaatatttt tccaaatgct accattgcca    523020
     ctaaagtcct cctggagtct gacgtctgct ctgggcactg ccttcttctc aggcgtccca    523080
     cactggagct tgctatagag gaggaggcat gaaaacaggg ccctccctct cctggtgaaa    523140
     tcaagcccag acctgaccct gcagctctgg gagaacagcc acagccctgg gattcccagg    523200
     ggtttccatt tggtaatcag gactgaacac agagaactca ctatggggtg tgaattaagc    523260
     tgaatttttc ttgttggtat tttaaaaggt gactcctagg gaactagagt gagtgagagt    523320
     gagtggatat gagtgagaga aacagtggat atgtttgcca gtttctgacc aggatgtgtg    523380
     tgtattttca ggtgttcagt gtgaggtgga gctgatagag tccatagagg acctgagaca    523440
     acctgggaag ttcctgagac tctcctgtgt agcctctaga ttcgccttca gtagcttctg    523500
     aatgagccga gttcaccagt ctccaggcaa ggggctggag tgagtaatag atataaaaga    523560
     tgatggaagt cagatacacc atgcagactc tgtgaagggc agattctcca tctccaaaga    523620
     caatgctaag aactctctgt atctgcaaat gaacactcag agagctgagg acgtggccgt    523680
     gtatggctat acataaggtc ccaagtgagg aaatatcggt gtgagtccag acacaacatt    523740
     tcctgcaaaa agaagaaagg agtctgggcc gaaggggaca ctcagcactc acaaaacagg    523800
     tgcagcccca aggcaggtgc agatggaggg agggtaaggg ctgctttcct tcagggtctg    523860
     tggtttcctc tgcttctaat atttcccctc tgaacctctg tatatttatg ttttgtgccc    523920
     accatgaggt ccctggatta gaaaactaat ttaaaagagg aaatattctc atatgtccca    523980
     aaaacagatg taagttttgg aggcataaaa atgcatagga gccgcgtgag tctgtagaca    524040
     ctgccacccc acaatgccag acccaaaact agtgctggag aagggtggga gtttgatgga    524100
     gcttccctga tgaccccgtg gtccaagcta agtccagcaa ggccattggt gcctcgctga    524160
     gcacagttgt ccatcaggga tctcccatgt gtcccagcag cagccatgcc tcagtatctc    524220
     cactgtgcac agccatcgtc tgggaggagc tcccaggatg ggtgtctttg gcacacacag    524280
     gtgatgggtg ttagagtgca gtgcagcagc tggctgcctg gtctattggg ctcctggata    524340
     ttggagggat tggaggtgca ttctcaaggc cagcacgctg tttgttaatt tttgtataaa    524400
     aaccatgtga tttaattcat tttctcagat gacatagata attaataaca caatctgcaa    524460
     acaattgtaa ttttcaactt taccccaagt tcattgtttc ttaattctgt gcaggatcca    524520
     gacatggtat tgcccttctc atgagaaatt gttcgaccta aactgaaacc agttgtttct    524580
     cgtatacttt ggttctcccc acatgcagag atcttgatta gagcaagttt ggtactttcc    524640
     acgcactcac cctcacctcc ccagataaag agcagaagtt ctccttagac tgagtctgag    524700
     ggaggagctg ttcctgtacc actcagggcc tgcggagacc cccaggtgca gcttcactga    524760
     gtcaggtgtt tcactccctg tgattgctgc tcaggtctaa ttgtgggctc agaattagga    524820
     cagtcttcag gttatcacag gtcaatcata ttctaaaaat catcattatc acacaccgtg    524880
     gtaataattc aatgttcatt tttctaaaca gcagtttctc ttttttattt ggttgcaagt    524940
     ctgaggaaag gaacatgtga taatactttt taatctaacc tctaaatcta ctgaattgtt    525000
     ctaggagact cacaaatcgg acaaagtgag ctctttatgc tcataaaaaa atgcatgtat    525060
     ttgggaattt caccgtgttg tccaggaccc gttaacatgg acaactgtgt ttcgcagtgc    525120
     acttctggct tgagacatcc tcacagaccc tctccctcac ctgcactgtc tctggattcc    525180
     ccatcataac cagtgtttcc ttctagaatt gtatctgctt gcccctagaa gatggacagg    525240
     agtggatcag gtgcatgggt tgtgaaggga gcacaaatta caacccactt ctcaagagtc    525300
     catatccgga tccaagaaac agttcttaca gctgagctct gtgcccagtg aacacacaac    525360
     tacgcatttt gaagcaaaag atgcaatgaa gggccttcat tgtgagccta gacacaaacc    525420
     tccctgcagg ggtgaatagg agcagcaggg ggcattcggg gcagtatggg ggcttaggat    525480
     gattgttagg ggtcaggatg agcaggacca aggcttcgca tcagggcaag tgcaacaggg    525540
     cagaaaaggg gctgtagatg tgggttgttc tcaccatcat atttcaccac cagacaccct    525600
     ccactacatc tcttctaatg tgtctgagtg ttgatatgat tagaaaatgg catttatgta    525660
     aatactacta tgtacccata tggaggtgca tccagttgtc ctctccatct tatgtggacc    525720
     ttgtccatca agcactaagt ccctgtaatt acttgagtac ctcacacatt atggtcaaaa    525780
     gatgtagggt ctccttttga catggtctct cctcctgcct tctctctctg tcacacaaaa    525840
     acacgtgaac tgacacacac acagagcttc ccaactttaa ttatgtgatg tattgaagca    525900
     aattgattag tgtgcagctt ttctgctttg cctgctattc atgttatgta aaaataagaa    525960
     ccatgttttt ctcagcttgt cacttctcta agctaagtag catctttgtt tattataccc    526020
     agagaccaaa agcgatccaa ctgttattca gcagcttaac tggtaaacaa attgtggaaa    526080
     attcatttac tggaataata cccactgtta caatcaagta ctgctggata cactcaacac    526140
     catgcttaaa ataacaagca cctgaataag taaaataagt caaacaaatg aaagtgcata    526200
     catacgattc cacttctata atttctataa agtaaaaatg aacttaaagt tacatgaaaa    526260
     gatctgtagt tgactgggga catggtacaa gaagagaagg tatagaaagg agaaactaca    526320
     ggagagcaaa aggaaatttc taggataatt gattttttct ctgttagtaa aagtgaggat    526380
     tatgtcacta tttgtaaaat tgtacacttt atgtaaagat tcttatttgc taatttcatc    526440
     tcattaaaat attgcaattt tttaaatgtc tagtttggta gaaaaggtag tagagagaga    526500
     tgaataaaat acataaaatt cagagagtcc tgaatacaca catgaatgga ccctgggtct    526560
     cactgtactt tgagggagac actagaatac aaaaacataa tgacaggatt tcagtacatg    526620
     aaaggagctt ctcaaacccc aggaggcatg tccaactgca tcttggagtt aactcaggga    526680
     gcaggcatgt cctttgaaag gagccgtgac accaagctcc cagcatccat tgtagccgac    526740
     accatgccaa tgccaagaga tctcaactaa aattttctgt ggatgttgag tctgattatg    526800
     ccacacactc acaccaagtg agtatgcaaa ggatagttac ctgcatcctt aaggtgtctg    526860
     ctgagagcag ggcaggtctc tcattaaggt ccaaagtggc ttgatagagc agggaaggag    526920
     actggctcag ggttgttatc atggtttggt ggggagtggg gcagagcatc gtacttgcag    526980
     gaagggtttt gtggggtttc aaggtcaaat tggcatgaaa agagggagct cctgtgattt    527040
     ctaactagat ttaccttgta tgcttaaaaa aagagatgat ggaggaaaga gcctgaagtt    527100
     atcagcagtc aggcctaaac atagagtctg atgacttact ctaaatagca agtatacaaa    527160
     tgatgagtaa gaaaagagac aagactccaa tatgggtgga caacagccag gtctgcagaa    527220
     aatgagaaga cagtttataa gcaaagaata atgagtagga ggaggatatg gaggaggatt    527280
     ctggtccaat gtcttctgtg gaagcttttc atgatttcag atcatcagct tattctgaag    527340
     gtcttaggtc acctgttttc ttcaaaatat cagaagtgcc agatgatatg tgaggatgcg    527400
     cagtttaatt tcctctgagt agctttacaa ttgtgtgaaa ttcttaattt ttttttgaga    527460
     tggagtctca ttctgttgtg caggttggac tacagtgctg tgatcataac atagctcatg    527520
     gtgatttcaa attcctggct catagaatcc tcccacctca acctcctcac tagcaagtag    527580
     ctaggtttcc agggcgcttc atctacactt ggccattctt gtcattcttt tttttttttt    527640
     tttttttttt aggtggattt tccctcttct tgcccagact ggagtgcaat ggtgccatct    527700
     cagctcactg caatctcccc ctcccaggtt caggtgattc tcctacctca gcctccagag    527760
     tagctgggat cacaggcatg tgacaccatg ccccgctaat cttgtatttt tatcagagat    527820
     gacatttctc catattggtc aggctggtct caaactccct acctcaggtg atccttccac    527880
     ctcagcctcc caaagtgctg gaattatagg cgtgaggcac cgcaactggc ctcttcattt    527940
     ttattcatat gttccttcag cagccactat gtcttcccac tgatttcttc agtttctgcc    528000
     ttttcctttt gaataaggct gttactcctg agggaagatg ggaggtgggc ctggacaggg    528060
     acttggtgca ttcctctctc ctgtcccagt tcttattggt ttctccagtg tctgtagaac    528120
     agtggttttg gtggctttac ctctgcagat aatttctctt gcaatgtagt ggtgatgggg    528180
     aggtgtgtct ggatgcattt cagctatagt tgctgttttg ctttcccaga cagcaccatc    528240
     ccaaagggta gaggctggag cattttgtga tgtatcccca gtactgaaga aaaaggcttc    528300
     aatagcagga ggaattcctc aactgtatac actctgagaa tttaaacaat aacttctcta    528360
     tcacactcaa attgaaacca tccaatgaat atgtctactt taatcgtgtg ctaacttaaa    528420
     tggcatttgg cagcctctgt cccagaaaag attatcatct gctcctgttt atttccctgc    528480
     acgtccttat ctctcttcag atttcagata tattgtttgt cctataacat caaaaatttg    528540
     atgtatatgt gctaatttgc agatcagtaa gtttagtagc tgttgtaaga ataataacat    528600
     atttttatcg ggtgcttaca tctccaagct gagaagcacc tctatgtgta atactaagaa    528660
     actagaaatg atacaaatat caagaagata catagataaa aagtaatggc atgctaattt    528720
     actgtaataa catccatcat gagaatcaat acattgttga tgctcaacat gtttgcatca    528780
     taagtagtta cgtgcgagaa gccacacaaa taaaacacat actatataat tcctgtataa    528840
     taaattcttg aaactcaaaa ctaaggtatt aaatgtgaag gactgactca gaatatggtg    528900
     ggggaagaaa ataattggga aggaggaatt gtagagcaac acaaggaaac ttttaagtgt    528960
     aatttgttca ttatttggat ggcttttggg gatgcacagg tgagcacgag tggaattaca    529020
     ttgtgttttg tttgtttctt ttttttttct gaagagatgt ggtccttctc tgcgacccag    529080
     gctggagtgt agtggtggga tcatagttca atgtagcctc taacttctgg tctccaacaa    529140
     tactcctgca tctgccatct aagtagctgg aactaccgtt gtgtgccagg aggtttggct    529200
     tggcttgagt acttattaaa ccacacactt tttgcaatat ttaatgtata gtaataatgt    529260
     ctcaataaga ctactacaaa tcaatgaatg aataatttgt tcagtacaga ttcatggaaa    529320
     aatagacact aacatgatga atgtctgaca tttatgaaaa tacaactgca tgaaatgtgc    529380
     ttctctttac attcattagg taaacacaat agtgcataca catcacaccg tgctttcatt    529440
     acaggaagaa agatctgaaa atgtcactgg ggtgaaccac attgtgctgg gcttggttca    529500
     gggagcagtc aggcccagtg ttgtgacctt caaccacaga atccttaaaa aataataaaa    529560
     gaggctcccc caaagtcccc atcagttccc ggactcgcta tgtttctgga tcgtatcagt    529620
     gcatccggag ctccctggtg gctttagtga ttccttgctt gccatgctga ggtctccctg    529680
     tagattatgt tgggttttct gaggccgttt tgctatttaa gacccactcc ctggcacaga    529740
     gcgattccct ctaaacctga tagaggttct gaactaaaaa taacattaag tgaatcctgg    529800
     tgtgtctgaa ctcaagtgat tgttacatta agctgctgtt gcaatctgtt tcctcacctg    529860
     ggaaaagagg agccaggaca tagtgagttg aggccccagg aagataactg aattctcaga    529920
     gggcacagcc agcatcctcc tcccagggag agtctaaaag actggggcct ccctcatccc    529980
     ttttcacttc tccatacaga ggcaccaccc ccatgcaaat ctcacttagg cacccacagg    530040
     aaaccaccac acatttcctt aaattcaggg tccagctcac atgggaaata ctttctgaga    530100
     gtcctggacc tcctgtgcaa gaacatgaaa cacctgtggt tcttcctcct gctggtggca    530160
     gctcccagat gtgagtgtct caaggctgca gacatggaga tatgggaggt gcctctgagc    530220
     ccagggctca ctgtgggtct ctctgttcac aggggtcctg tcccagctgc agctgcagga    530280
     gtccggctca ggactggtga agccttcaca gaccctgtcc ctcacctgcg ctgtctctgg    530340
     tggctccatc agcagtggtg gttactcctg gagctggatc cggcagccac cagggaaggg    530400
     cctggagtgg attgggtaca tctatcatag tgggagcacc tactacaacc cgtccctcaa    530460
     gagtcgagtc accatatcag tagacaggtc caagaaccag ttctccctga agctgagctc    530520
     tgtgaccgcc gcggacacgg ccgtgtatta ctgtgccaga gacacaatga ggggaggtga    530580
     gtgtgagccc agacacaaac ctccctgcag ggaggcggag ggggcgggcg caggtgctgc    530640
     tcaggaccag cagggggcgc gcggggccca cagagcatga ggccgggtca ggagcaggtg    530700
     cagggagggc ggggcttcct catcagctca gtgctctccc tcctcgccag cacctcagct    530760
     gtccccagga ctcctctttc tttattatct gtggttctgc ttcctcacat ccttgtggta    530820
     ggaaaggaag gaggaaggca aattttcctc ttagagtcaa agtgtcacta attactagga    530880
     actttcctac aagttcctga atgtcccatt tttccttctt aattaaaaaa aatatatatt    530940
     ctaatacttc tcaccatctc ttgatttgtg tcatcagttg aattgtgctg tctttgaaat    531000
     tcaaatgctg aaaccttaaa tccaattgat ctatattgga attttaagga tggaattaag    531060
     gttaaatgtg atcataagtc tgagattcta atgcaataga tctgttgtct ttataagaag    531120
     tggaagagtc accagagacc tctcactttt cccgtgcacg cagagaagag gccatgtgga    531180
     gacatagtgg actagaaggt gcaagccaag aagaagccgc accaagaacc aaccctgcca    531240
     gcacctggac cttggacatt cagacttcag aatggtgaga aaatcaatgt ttgttgttta    531300
     agccacccac tcctgttgtc ttcttatgaa gacccagaca gactaatacc acataactct    531360
     gttagctcct ggagggagaa gcagctccct gaggctgggc acatctctca gatatccata    531420
     tgaagtaggc agaaatagta gttctcatat aaaaatgtgt catggccctg ttggccattt    531480
     ttttttgacg gagtctcgct ctgtggccag gctggagtgc agtggtgcaa tctcagctga    531540
     ctacaaaccc cgtctcctgg gttcaggcaa ttctccttcc tcaacctcct gagcagctgg    531600
     gactacaggt gtccaccacc atgccagcct attttttttt tgtattttta gcagagacag    531660
     ggtttcactg tgttagggtg gtctcaaatc tcctcactgc atgatctgcc tgcctcggtt    531720
     tcccaaagtg ttgggattac aagtgtgagc caccgcacct ggcccctgtt ggccattctt    531780
     ggggcaatgt ctctgaaacc agccctggtg cctgtaccac aaaattttct tttatctttc    531840
     atgtggatat aacaaatgga caatgaggac cagctgcatg gagactgacc actgaacatc    531900
     ttctgctgtc tcctaagtaa gtcacaggaa aacacaccaa catcaccaac ataactgttt    531960
     tccttcaact tcctcgaacg aactatagaa atgatccctt aaagtatagt ctattccttc    532020
     aactttctca atttgcactg aatcccttcc taaattagga gctacatagg gtctgagttt    532080
     tgttcccttt ctcccagtct tccccaagta tcaaggacag aatagattta aattaaattt    532140
     ggccgtccat gcccccaaca ccacatcggt ttctaacatc cttgtcatgt acccatcctt    532200
     ctgtgggcac cccacatcag gttgcccagg aatggccaga aggtgtcatc accttatggg    532260
     ctgaggctac aagttataca cacgtgtgat ttcagtcaca cacactctac tgcaggacac    532320
     acctgtgttc tgaggaactc aggcgcctgc tgatctcagg tcttctctaa taaattacac    532380
     acctcttatg aatgaaggtc cagatggccc catcagctgc agagcagtgg attaaagctc    532440
     atgggtgggt cagtcaggca gaagtcagac aatgggataa tagaagtcag acaatagtca    532500
     ctgaggttcc caatgtattt taatggaata aaacaatatt agcaacaaag gggcagtaaa    532560
     gaagactggc tcttgaaggt atttaaggac caggaacttt atttgggggg aaagtgagag    532620
     acacttttac atggaaagcc ctaaagcaca tacagcagct gacagagtgg ccactgtgca    532680
     catgagggct gaggagacgg atgataggtt acgttgctcc aagatgtccc tgggtgtgtg    532740
     atggttggac tcctcatgca tatgaaaata agagctggac tcagggagaa acaagggcca    532800
     taccccatag aaaaggaaga aaaaaagcag atgggcaccc ctggagagag ctcattagat    532860
     ttggtaggtt tgagatgaaa attacttcca aaggttttaa tgttctaagc taaatatgaa    532920
     tctcttcaat aaagtgagaa ttaaaagaag aatggagcta ggagttgaga gaggaaacaa    532980
     attatgagag agcagaaagc aaatccaccc aaactgtcac atgacagagg ccagaatgga    533040
     gctgatgcag ctacttcact attctgcaga cctagttgac catgtggaga aggggttcga    533100
     acaaacgggg atgttctcca accttccaaa acaacctcct tcttatgcat atgcagtaat    533160
     catgattctc agggtggcct tcccaagttt cctgttctta cctcttcccc caggggtaga    533220
     gtgtcttttc caacccacag tggtttctct gccatgtcct cagcttctcc tccattgtac    533280
     ttaacctgaa gattaaggat ttcatctgga tggagtttcc tgggaatcct ctgttttctt    533340
     tggtttctga tgaccacatc acaggccata ggacacaggt tttagtggat ttcccctgga    533400
     gacagtggag gtgaatccag gcgttccaca gtccttaccg ttcttctctt cctgtcagca    533460
     ccgcaggaca gcagataagg gagctgactg tggatttttc agatccccgg gaaaaagcct    533520
     gcaaggacta atagtttcac atctaacacc attagcacat gcatccaaaa ttaaaaaaaa    533580
     aatcctcact aaatatttcc aggttagcct gttcctctct caataccata ccgtggcacc    533640
     tgccctgggt tcggtaacat gtgggcccca gtcctctctg taggcgtctg cctcctcaga    533700
     tttcagtcct cttttttgct ccgtgaaagc aactcagata tgttaaaagg ttttcttctt    533760
     catttattca taatttttca ggtttgttgt taatgaggaa agagtaagat catggtttcc    533820
     tcatttttca cattcccata ctgagtaact gctctctctc tatgaaagcc agaaattaag    533880
     agaacaactc aagtatccat atccattaca agtgaatgtt agacaatttg atgtatgatt    533940
     atgaactaaa gtacaacaca ggattagaat ccaggcatcc tcattctcat aaaatcatgg    534000
     ctgcattcac aaataactgt gctgagtgaa agtagctaac aaattaacag tgcacttcat    534060
     aaacttctat ttgtataaac tgcagaaggt acagctattc taaagcaaca gagaagatta    534120
     catattggtg ggaactgggt tttgaaagta atggggtggt gagatgaaat tacagagaag    534180
     tgacacaaag atttaggtgt gaattgtaca caaattaatt gaagctctga ttaaagggtt    534240
     gcacacatgt taccattttc caaattatgc agtatatatt tgaattaatt attaattgta    534300
     tttaaataaa gcagtaacaa agaaacaaat gaatacattt attgaggtgg agccaaaact    534360
     aaatgggaat gaacactcga aacatctctg actcttgaaa atacacaagc gtgaacactg    534420
     gttctctcta tgtatttcag gtcaatagca gaatacacta aaagaaaaca gagatgtcct    534480
     ggcaggggtg gaatcctgca gacctcacta ggcatgtctc acactgccct ggagttgtct    534540
     caggggagca gtctcctcta gtggtcagag gcacaggccg agataatggg gctaactctg    534600
     tccagctgtg tgaccttgaa tgcattgtat aaacactctg ttctctgtgt gatttatctt    534660
     ccttaaaatg tgacattgac acttgcatta aatgtattct acaaatatgt caaaaagatg    534720
     atgactgcta aatgattatc aaggcacaat catataatat aatgatattt tcctgagtga    534780
     taagatgact accaatctcc cccagggcac tttgtctgct ctgagccctg cacctcctca    534840
     ggattcccat cccagagctt gctatacagt aggagacatg caaataggtt tctccctctg    534900
     ctgatgaaaa ccagcccagt cctgaccgca cagctctggg agagaagcgc cagccctggg    534960
     attcccaggg gtttctattt ggtgatcagg actaaagaca gaggacccac catggagctt    535020
     gggctgagct gggttttcac tgttgctgtt ttaaaaggtg aactagagag attgagcgtg    535080
     agtggatacc cttgagagaa atggtggatt atgtctggga gtttctgacc aggatgtcta    535140
     cgagtttgca ggtgtctagt gagaggtaca gctcgtggag tccggagagg acccaagaca    535200
     acctggggga tccctgagac tctcctgtgc agactctgga ttaaccttca gtagctactg    535260
     aaggaactcg gtttcccagg ctccagggaa ggggctggag tgagtagtag atatacagtg    535320
     tgatggaagt cagatatgtt atgcataatc tttgaagagc aaattcacca tctccaaaga    535380
     aaatgccaag aactcactgt atttgctaat gaacagtctg agagcagcgg gcacagctgt    535440
     gtgttactgt atgtgaggca ccaggtaaga agacatcagt gtgaacacag acacagtttc    535500
     ctgcaatgat aagggaggag gctgggctaa aaggggcact caggacccac tgaaaacggg    535560
     cagctctagg gcaggtacag atggttatca tgggctgctt tccttcgggg tctgtggctt    535620
     cctctgcatc taccagttcc cctcggagcc tctggacatt tatgtttctg tggccacccc    535680
     tgacatctct ggattagaaa agtttattat aggaagagga aacattttca tttgtcccaa    535740
     agcagatgta agtaatggaa gcaaaaaatg cacaggaggc caggtggggc tgtagatact    535800
     gtcaccccag aatgtcaatc tcaccactag tactggagag gcatgggagt ttgatggagc    535860
     ttccctaaca actctgtctt ccaagataag tccagcaaga ccattggtgc ctcccagagc    535920
     acagttgtcc atcagggatg tccaatgctt cccagcagca gccatgcctc agtgtctcca    535980
     ctgtgcacag ccactgtctg ggaggagctc ccaggatggg tgtctttggc acacaccagg    536040
     tggcgggtgt taagagtgca gtgcagcagc tggctgcctg gtctgttggg cctgatgctg    536100
     gagagatggg aggtgcattc ttggggccag catgctgttt gtgaattttt atataaaaac    536160
     tctgatttta cttcattttc gcagatgaca tagataacta aaaacagaat ctgcaaagaa    536220
     attgtaattt tcaactttac cccaaattca ttgtttctta attctgtgca agatccagac    536280
     atattattgc cttcctcatg ataatttatt ctatgtaaaa ccgaaatcat tttttctcct    536340
     actctttgtt tctgttcaag tacagagatc ttgagcaaag taagttgggt tctttccaca    536400
     cactaaccct caccttcccc agagaatgag cagagattgt cctcagtctg agtctaaggg    536460
     aggagctgtt tctgcacaac tcagagcctg cagagacccc caggtgcagc ttcagtgagt    536520
     cagacatttc tccatgtggg cgacctccag tgcctgtgac tgctgctcag gcctaattgt    536580
     tggttaagca ttaggacact ctttaggtga tcacatctca agcctattct gaaaatcacc    536640
     atgatcagag atagttcaat ggccattctc ctaacataag tttctcttta ttacttggtt    536700
     ccaagtatgg agaaaaatgt gaccctatat ttgtctgaat ccaacatcag catcaactgc    536760
     attatgctag gagactcact aattgagcac aagtgagctc gttattctca tagaaatgta    536820
     agtatttgga aatttcagtg tgttctccag aacctgtggc tgccaacaac tgtatttctc    536880
     ggtgcactct tggcctggtg aagccttcac agaacctctc cctcacctgt gccatctctg    536940
     cattctccat cacaaccagt gtttccttct ggagctgcat ccatcagcgc ccccatggga    537000
     ggtactggag tggatcgggt gcataggtca tggagggagc acaaattact cccctcttct    537060
     caagagtcca gtcaccatct ccagatccat gtccaaaaag tagttcttct tacagctgaa    537120
     ctatgtgagg aacaaacaca tagccatgta ttttagagca aaagacacag tgaggaaacc    537180
     acagtgtgaa ctcataccca agcctccctg tgggggtgca taggacagcc agggttactc    537240
     aggacaccag gctccctcag gacaccaagg ggcactcaag accattgtag aggcatgcag    537300
     gtagctgggg gctctcagga accatggggg aaaatcagga caccaaaggg tgcttggtac    537360
     agcggggggc tcaggattat tgtggcgatt cagaaagagc aggttcaagg ctcaacctca    537420
     gggcatgtgc agctggtgtg aaaaggagct ggatgaggcg ttttgtgtca ccatcatgtt    537480
     tcaccaccag acaccctcca ctacctctat tctaatgcat gtgtttgtat gattagaaaa    537540
     tgatattgat ataaatatat aaccatagct aggtgtgtca agttgtcctc tccatcttat    537600
     atcagccttg tctgtaagga ctaattcccc ataattactt gagaacctca taaattgtgg    537660
     tcaattatgt aggattcctc tctttttctg ccttccttcc tccctcattc tctctctctc    537720
     acacagaaac ttatatacag ccaccccaca acacacataa atctataact tttattacct    537780
     gatgtattga taagtaatct aaagttacat aaaaatcagt agttcactgt caatattgta    537840
     ggagaaggga aggtctagga aggaggaatt acagaacaag aggatatttt gagggtaatt    537900
     gacttgttat ctatgtggat aatgataatg tctatgacca tatttgtaaa attgaacact    537960
     tcgtgtggag actattattt ttttatttaa ccccatgcca ttattttgcc gtattctagc    538020
     atttctctaa aaatacagaa atgttcagca ctcattcatg tgtatattca ggagtttctg    538080
     acttttcatg tattttattt atctctgtct aattgctttg ataccaaatt ttacctagta    538140
     taattagtac tacctttagc actgattata agtctcactc ctccatcatc tccttttttg    538200
     ccacacaagc tgaatctagt ttggactcat aggagctgct tcattcaatg ccagtggttg    538260
     tttcaaaccc tatcaacccc cctacaaact gtcattaaga aagtttatca aactccacca    538320
     gtcatgtgat attagttcgt tttcacagtg gtataaagaa ctacctgaga cgaggagttt    538380
     acaaagaaaa gaggtttagt tgactcacag tgctgcatgg ctagggaggc cacaggaaac    538440
     ttataatcat ggtggaaggt gaaggcgaag cacggcacat cacacagcgc agcaggagag    538500
     agagggggaa gtgacacata cttttaaact atcagctctt gtgagaagtc actcactatg    538560
     atgagaacaa catgggaaaa ctgccctcat gatccaataa cctctcacct ggtccctccc    538620
     ttgacatgtg gggattagaa tttgagatga tatttaggtg gggacacaaa acaaatctat    538680
     agcacatgtc cagctgtgtc ctggagttgt ttcagggatc cagtgtgtac tgttgataga    538740
     aacagtgatc atcattctag ctggtgtgag atggtatctc cttgtggttt agatttgcat    538800
     ttctctgatg gccagtgatg atgagaattt tttcatgtgt cttttggctg cataaatgtc    538860
     ttcttttgag aagcgtctgt gaaaaaaaaa aagaaacagt gacaccaagc tcacaccatc    538920
     cgttgtagtt gacaacatgc aaagccaaga gatctcaact gagatttagt gtgtgtgtca    538980
     tgtctgatga agtcataagc tcagagcaag tgaatatgga aaagtgtatt atctgcacag    539040
     tgtaggtgtc tgctgagtgc agggcaggtc tcacaggaaa atctaaaatg gcttgaaaga    539100
     agaggaaagg agactggctc agggttttta taatggttta gtggtggcgg cagagtgagg    539160
     cttcccactc acagatcggg gtttataagg tttgaaactc ccactgccat tgaatgaaga    539220
     agctcctgtg agtccaaact agattcacct tgtgtggcaa aaaaggagat gatggaggag    539280
     tgagccttaa gtaatcaaca ggcatgcacc aaaagataga gtgacaactt attctaggta    539340
     gcaagaataa aaataatgaa aaagaaataa gggttcagta tcgatggaca agacccagat    539400
     ctacagaaat gagatgactt tagaaatata aggaaataat aatgagagaa agaaggaggg    539460
     gatggggaat tagggtcctg gccgaatgtc ttgggtagaa gcttctcaca atcaaggact    539520
     atcagcttat tctgcaggtc ttaggtcaca cgtctgctta aaaacatcag aaatgccagg    539580
     caatcaatga ggatgctcag tttagcatct cctatttgag tagatttaca gttgtgtgga    539640
     attcttaatt gattcttttt ggtttgtttt tagaggcagg ctctcacact gttccacagc    539700
     ttaaagtaca gttgtgtgat catagctcac tgtaattttg acctccagac tcacgtaatc    539760
     ttcctgtatc agcctcctga gtatctagaa ttagagggga atgccacccc tcacctgctt    539820
     attctttaaa atattttttc atagaaatag catctcttta tgttgcccag gttggcttcg    539880
     atttcctggt ctcatgtgat ttccctcact ttgcttctga aagtggtgtg attataggga    539940
     agatccactg catctgtcct gaattgattc tttacttgta aaacatgaag ccaataatta    540000
     actgcctgac tgttttgtgc agtgagttag ttaaaagttt ctgataagat tccttccaat    540060
     atgattcaag agcagtattt tctgccgata ttcctttcag tttccttgtt gaagatcaga    540120
     agacactgcg gaaaagatgt agtaaaaagg cagttttaac tcttcatgat tggagtggat    540180
     atcacatagg aatccctttc cgtattttgt aacacatgca tgcaatagaa caaagatgat    540240
     cccttggtgt aaaatctata aacgtatggg ccttttagct accaagtcat agggtaataa    540300
     ctgatggacc ctgaggagtg aaccatgatt ccatagtgct agtgggagaa cccttggcca    540360
     aggaagttca cattttatta aaattgataa tttttatgta gggatgccat tttttaaaca    540420
     ttcccagaag gttgtgagtg ggattgactc tgtcttctgt gaacaatgac agtgccctcc    540480
     atggttagat aatattttaa actagattga gctagaatgg ttgctgtatt gaatcactat    540540
     atttttttag cttcatgtta gctttttgtg tgttagcatt tgctttaaaa tgatattaat    540600
     cagccctcta gtaggtagaa attcatctga gggtttcttc ccttgttgtc catctcaata    540660
     ggatttccag aagacgtaag aaccctctct gtttctaaaa atattccagg ctgggcgctg    540720
     tggctcatgc ctgtaaaccc agcactttgg gaggccgagg tgggtggacc cctcgaggtt    540780
     gggagtttga gaccagcctg gtcagtatgg tgaaacctca tttctactaa aaatacaaaa    540840
     attcgccggg catggtggtg tacactcgta atcccagcta cttgagaggc tgaagtgaga    540900
     ggattgcttg aaccctggag gcgggggtcg cagtgagccg agattgtgcc actgcacttc    540960
     agcctgggcg acacagcgag actccgtctc aaacaaaacg acacaaaaaa ttccaaagtt    541020
     gtgcaccctc taaaagcata cgtacttaat tctcattttt aatttattaa acatctctaa    541080
     taagttcaat gtttcctgcc ttctgagttg atttcctaac acatagaaga atatatccta    541140
     aatgaaagtt tgtgttctta atagaaatta ctagttaata acctttactt ttattattga    541200
     ggtattatac atcaatgtta atcctctcaa tgggactctt acctaaagaa tatataaaat    541260
     attttcctga tcatgacata aagtagatgt gaacacattc ttagcattca gccatgtctc    541320
     ttgtctatta atattataaa ccacatgcta actttgattt tattggtaat tgttctaatt    541380
     tctttttttt tcccacttct ctggttgttt ttttttttaa ctttaagttt tagggtacat    541440
     gtgcacaatg tgcaggttag ttagatatgt atacatgtgc catgttggtg tgctgcaccc    541500
     attaactcgt catttaatat taggtatatc tcctaatgct atccctcccc cctcccctca    541560
     ccccacaaca ggccccggtg tgtgatgttc cccttcctgt gtccatgtgt tctcattgtt    541620
     caattcccac ctaggactga gaacatgtgg tgtttggttt tttgtccttg cgatagtttg    541680
     ctgagaatga tggtttccag cttcatccat gtccctacaa aggacatgaa ctcatcattt    541740
     tttatggctg cataggattc catggtgcat atgtgccaca ttttcttaat ccagtgtatc    541800
     attgttggac atttgggttg gttccaagtc tttgctattg tgaataggga attgttctaa    541860
     tttcaaacta gttaattttt atcttcatgc agctagatta ttatgtgtgg ctatttattc    541920
     cgagagtgat aaagacaaca ttaacaattt tcactgcagg catgtctagg caactccctg    541980
     tgcactatga ccctggggcg ttggagattc tatggggact cttccctacc tgcctaggag    542040
     agttctctgc cttctacctc tagcattttc ctctttgaag aagtacatct aactgtcatt    542100
     agaatagaga caaagacaag tcttaactgc ttccagctga ggagggatgc tgtttgggga    542160
     agatctctct tggaggtcta agggacccca ggaaaaggga gccattatcc caggcttcag    542220
     ttgcatgacc atttggagtt tgatggtctg aaaatgagaa gaggcaaatc tggttattag    542280
     aagacatgta tgaaaaccaa acaaggtggc aaggacagct tgaaagaaaa ttccaaggct    542340
     gctgacattc ctagataact gcagctgtag ttatgcctgc taaggtttgg gcgcatgggg    542400
     cttggctttt gtcagctccc tgggatttat tttcccaaac aaagaaacct ccaggttagg    542460
     ggcaccctat tcattcccat cacctggcat gatttaaagg ataattgctt agaattaaaa    542520
     tattgatcca gattttttat attccccatc gctttttgtt tcttctgggc tgtagccaga    542580
     gatcattgat tggcgctcag gaataagcag agttagtcta aaatgcaggc aaatacttaa    542640
     acaactgaag agattagaat ttaaagacaa gtgtatgata tgttttgaaa tacaatgttt    542700
     ctctttccag ttttggtttt tgtcagcagc aaataatgat aagactgagt tgtttgcaaa    542760
     ataaacttta gtcttaaact tggcctgatt atttgcataa agtgcagcaa gaatattaat    542820
     aataattctg taggaaaagc ctgcaagcac caggagcttc acagtctaac actatgagca    542880
     cgtgcatcct cacgcaactc actgaatatg tccaagtcag cctgttccga tcttaaatgc    542940
     catccagtgg catctgcccc aggtacacta atacatgggt cctgcttctc tctgcagccg    543000
     cctctctcct cagatttcag gttttgtgta ttgtttgttt tctctctgac atcaacacag    543060
     atatgttgaa ggttttcttt tttttatttg tagttgttca gctttgttgt taatgaggtc    543120
     agaataagct catagtttac acatttttac attcccatgc cgagtagctg cttttctcta    543180
     tcaaatccat taactgagag aacaatcaca tttcgttaca ggtgaacagt taaatagttt    543240
     ggcatatatt tctgtgctgg aatctaatgc agcttgaaat caagtcatgc ctcactcatt    543300
     gaaaaaaaca tggctaaatt ctcaaagaat tgtgctgagt gaaagaaact aaggaatgaa    543360
     gagtaaattt tatatgatac atttgtagaa attttagaag atgccactat tataaattaa    543420
     catggagaag atttaaatgt ttctgagaat atgctattgg gagtaatggg gatgtgagtt    543480
     aaatttcaga ggaataagag aaagatttag ggattaattt attcaaacct tgattgaagt    543540
     gctgagtaaa tggttgcaaa cataggtcta catttttcaa atcattcacc ataaatttga    543600
     attatttatt aattacactc gaataaagca ataaagaaac tgatgagata atatttgact    543660
     gaattgcagc aataaataga tcgatattaa cacaaggaat ataactgact tccaaaaaca    543720
     tacacatgaa ccgtggttca ctctgcgtat ttaggtaaat aacagaaagt tgtcataaca    543780
     gatggggaat cctgcagact tcactaggca tgggccatgc tgccctggag ttgtctcagg    543840
     ggagctgcct cctccagagg ttagagcaca ggcccaggta ataggactaa atttttagat    543900
     gtgttatctt agacacactg cacaactgct gtgttctcta tgtaaattat ctcctgtaaa    543960
     atataacatt gaagcctgca ttaaatatat tgtgtaaata tgtaagaata aaagaaagtt    544020
     atgagagcta agtgttaatc aaggcacaag catataagat ataactatat tttcctgaat    544080
     gatggaatta ctaccagtct cccccaggac acttcatctg ccctgagccc agcctctcct    544140
     cagatgtccc acccagagct tgctatatag tgggggacat gcaaataggg ccctccctct    544200
     actgatgaaa accagcccag ccctgaccct gcagctctgg gagaggagcc cagcactaga    544260
     agtcggcggt gtttccattc ggtgatcagc actgaacaca gaggactcac catggagttt    544320
     gggctgagct gggttttcct cgttgctctt ttaagaggtg attcatggag aaatagagag    544380
     actgagtgtg agtgaacatg agtgagaaaa actggatttg tgtggcattt tctgataacg    544440
     gtgtccttct gtttgcaggt gtccagtgtc aggtgcagct ggtggagtct gggggaggcg    544500
     tggtccagcc tgggaggtcc ctgagactct cctgtgcagc ctctggattc accttcagta    544560
     gctatggcat gcactgggtc cgccaggctc caggcaaggg gctggagtgg gtggcagtta    544620
     tatcatatga tggaagtaat aaatactatg cagactccgt gaagggccga ttcaccatct    544680
     ccagagacaa ttccaagaac acgctgtatc tgcaaatgaa cagcctgaga gctgaggaca    544740
     cggctgtgta ttactgtgcg aaagacacag tgaggggaag tcattgtgcg cccagacaca    544800
     aacctccctg caggaacgct ggcgggaaat cagcggcagg gggcgctcag gagccactga    544860
     tcagagtcag ccctggaggc aggtgcagat ggaggctgtt tcctgtcagg atgtgggact    544920
     ttgtcttctt ctgacagttc cccagggaac ctcttaaatt tagaaaactg tgcctaacaa    544980
     tgtcttctct atgcatatga ggaccttttc tccctggcac aaaatgcaga ttgacgctga    545040
     cacggatgaa aattcctcaa ccatggtcac aaggatcaga gtcctgagta acctcagggc    545100
     ttcctggtga gtcttctcca atcagaccca ggacagggac ctccgtgaga ttccctgact    545160
     ggaacagtct ttatggatcc tggtcacaga caatagagag gctgaaccag ggtcagcgtc    545220
     atgtagaacc tcacagattt cacgtctgat ccttctcctg acacgaaagt atgcaaatca    545280
     gtatcagcac cgatctggtg cttcttttgt tcctaatcca tttactttat tttttcgtcg    545340
     tttttctcct ttttccattt gtttttcctg ctttttgcaa aaggaagatg ttttccctgt    545400
     gagatgcagg ggatgacaat tttgggagat ggctggaaca tccaatatcc tcagggccgg    545460
     ccatcagtaa gtgcaggctg gaagtctcag aaagagctga agctgcttaa tcaccgtgga    545520
     gttttacctt ctccagttct gctctgatgg aatcagggcc aagcaggtta tcaatgataa    545580
     tctacctaac atagagtcaa ccgattccag tttcaataac gtctgttaaa aattcacacc    545640
     accacctgga ttagtgtttt gtcaaatcaa tacacagtat tgtccagcta agtagaccca    545700
     aagacggacc gttgcccatg gagaaaaaca ttaacctgag ttctaggttc ttacagtgtt    545760
     aaaggtgtaa aactgattat taaaaatgag gctatttttc tttttgctgt tgagttgtag    545820
     aagtttcttt tccatttgga cattaaaact ttttgagata tatggcatat tatccaattc    545880
     tgtaagttgt agttacttgg ttgctttgca gaatcttttt cataatctat tcccacttgt    545940
     tcaattctgc tttttttttg taggtgattt gaatgtaaaa tccagaaaaa gattgctaat    546000
     tttttgaggg ttgagagttt tacaattaca ggtattaaag ttagatattt gaggcatttg    546060
     gagtgaattt ttgtgtttat tctaacctaa aattcttaat tcttttcatg ggaaaatcca    546120
     gttttcatac caccctcttt ggaagacact acaatttagc catgttatat tgatggttct    546180
     catgctaaaa atcagctcgt catcaatatg tgggtttata tctaagctct gtataggtat    546240
     ttatgccaaa actttctatg tttttaacaa atgttaaggc ctggaagtga aatgcctaaa    546300
     gctttcttct tgccttgtta cagatattgg accaaaatat tctaaccttt tactattgag    546360
     tgtaataata gctgtggcct ttcttaacgg cttttattat gttcaagttg ttttcttgtc    546420
     ttcctacttt gttcatagtt tttataatga aactgatttt tttcaaagtc tttttctgtg    546480
     tctgatgaaa tgttactgag gtattttttc tttagttttt taatgtggtg taccaaattg    546540
     gttgatttga gaatgttgaa tcaagtatgc atctcaggaa gaaatttgag ttggtcatgg    546600
     tgtatgtctt ctaaaacact ttggagctta gtttactatt gttggggatt aattcatgtc    546660
     tactaatgat attggtctgt agttttcttt tattgtggtg cctttgtcta ttactggtaa    546720
     tactatcatg gtagcctcat agaaagagtt tagaagatat atggcagact acctttaaaa    546780
     tagattttat cagtggagaa atggtgatag ttttttcttc acttttctgt tgggaagaat    546840
     tttatgttgt ttaaaagata ttcagaatga cttaacctgg tttatgagct ttcattctat    546900
     tcctttcttc cattcttttt tttttttttt tttttttttt tttttttttt tttttgagac    546960
     ggagtctcgc tctgtcgccc aggctggagt gcagtggcgg gatctcggct cactgcaagc    547020
     tccgcctccc gggttcacgc cattctcctg cctcagcctc ccaagtagct gggactacag    547080
     gcgcccgcca ctacgcccgg ctaatttttt gtatttttag tagagacggg gtttcaccgt    547140
     tttagccggg atggtctcga tctcctgacc tcgtgatccg cccgcctcgg cctcccaaag    547200
     tgctgggatt acaggcgtga gccaccgcgc ccggcctctt ccattctttt tgaagaactg    547260
     cttacctttc ctatttattt ttaagtttgt ttttagacgt atgcaataca ttttgaggtg    547320
     aaacctggtg gaattttttc taataaatta gaaaaaaata aatcatttaa ttgactattt    547380
     tattcaggtt gatttgttta atatttgcta aaggccagtt ctttaagcta tgacacataa    547440
     taaatcccaa atggcagtac ctcattgttt acttagcttt tgtacttata tttctcagag    547500
     gaagaaccac tactgtaaat tgtaaatagc caatacataa ttgtattgta tgcaaatctg    547560
     tgactgttta cagtgtcatc tctgagaaac ataaagttta tttactatat atatataaag    547620
     agtttggaag gtggactcct caccaatttt tgaaagagtt acagaagggc tggcattact    547680
     tctttaaatg ttaaggttca ttttatgatg tgacatataa cccatcatgg agaatgttca    547740
     atgtgttctt gagaaggatg tgtattacgt gactcttggt tggaaggttc tgtaaatatc    547800
     attcagataa atttgttcaa tagtgttgtt caagttcaga ggcacattag gaattttctt    547860
     tccggatttg ctatacatta tggtcatgag gtattaaggt cttgtgttat ttttgtattg    547920
     ttttctattt ctttatatct cttacagttt gcttaatgca gttatatttg tatttgtaca    547980
     catgtgtaaa aacaaaaatc ataattgcta aatgagtttt atggcacagt cacattataa    548040
     gtaatatttt tccaaatgct accattgcca ctaaactcct cctggagtct gacgtctgct    548100
     ttgggcactg ccttcttctc aggcatccca cactggagct tgctatagag gaggagggag    548160
     ggcccctctc ctggtgaaaa caagcccaga cctgaccctg cagctctggg agaagagcca    548220
     cagccctggg attcccaggg gtttccattt ggtaatcagg actgaacaca gagaactcac    548280
     tatggggtgt gaattaagct gaatttttct tgttggtatt ttaaaaggta actcataggg    548340
     aactagagtg agtgagagtg agtggatatg agtgagagaa acagtggata tgtttgccag    548400
     tttctgacca ggatttgttt gtattttcag gtgttcagtg tgaggtggag ctgatagagc    548460
     ccacagagga cctgagacaa cctgggaagt tcctgagact ctcctgtgta gcctctagat    548520
     tcgccttcag tagcttctga atgagcccag ttcaccagtc tgcaggcaag gggctggagt    548580
     gagtaataga tataaaagat gatggaagtc agatacacca tgcagactct gtgaagggca    548640
     gattctccat ctccaaagac aatgctaaga actctctgta tctgcaaatg aacagtcaga    548700
     gaactgagga catggctgtg tatggctgta cataaggttc caagtgagga aacatcggtg    548760
     tgagtccaga cacaaaattt cctgcaaaaa gaagaaagga gtctgggcca aaggggacac    548820
     tcagcactca caaaacaggt gcagccccaa ggcaggtgca gatggaggga gggtaagggc    548880
     tgctttcctt caggatctgt ggtttcctct gcttctaata tttcccctct gagcctctgt    548940
     acatttatat tttgtgccca ccatgaggtc gctggattag aaaactaatt tgaaagagga    549000
     aatattctca tatgtcctaa aaacagatgt aagtattgga ggcataaaaa tgcataggag    549060
     ccgggtgtgt ctgtagacac tgccaccccc aatgccagac ccacaactag tgctggagaa    549120
     gggtgggagt ttgatggagc ttccctgatg accccgtggt ccaagctaag tccagcaagg    549180
     ccattggtgc ctcgctgagc acagttgtcc atcagggatc tcccatgtgt cccagcagca    549240
     gccatgcctc aatctctcca ctgtgcacag ccattgtctg ggaggagctc ccaggatggg    549300
     tgtctttggc acacacagtt gatgggtgtt agagtgcagt gcagcagctg gctgcctggt    549360
     ctattgggct cctggatatt ggagggattt gaggtgcatt ctcagggcca gcacgctttg    549420
     tcaattttta tatgaaaacc atgtgattta attcattttc tcagatgaca tagataatta    549480
     ataacacaat ctgcaaacaa ttataatttt caactttacc ccaagttcat tgtttctgaa    549540
     ttctgtgcag gatccagaca tggtactgcc cttctcatga gaaactgttc gatctaaact    549600
     gaaaccagtt gtttctcgta tactttggtt ctccccacat gcagagatct tgattagagc    549660
     aagtttggta ctttccacgc actcaccctc acctccccag ataaagagca gaagttctcc    549720
     ttagactgag tctgagggag gagctgttcc tgtaccactc agggcctgcg gagaccccca    549780
     ggtgcagctt cactgagtca ggtgtttcac tccctgtgat tgctgctcag gtctaattgt    549840
     ggctcggaat taggacagtc ttcaggttat cacaggtcaa tcatattcta aaaatcatcg    549900
     ttatcacaca ccatggtaac aattcaaggt tcatttttct aacggcagtt tctctttttt    549960
     atttggttgc aagtttgagg aaaggaacat gtgataatac tttttaacct aacctcgaaa    550020
     tctactgaat tgttctagga gactcacaaa ttggacaaag tgagctcttt attctcataa    550080
     aaatgtgtgg ttttgggaat ttcactgtgt tgcccagaac ctgttaacat caacaactat    550140
     gtttctcagc acacttctgg cttgagacgt ccttgcagac cctctccctc acctgcactg    550200
     tctctggatt ccccatcata accagtgtgt cctgctagaa ttgtatctgc ttgcccctag    550260
     aagatggaca ggagtggatc aggtgcatgg gttgtgaagg gagcacaaat tacaacccac    550320
     tgctcaagag tccatatcca gatccaagaa acagttctta cagctgagct ctgtgcccag    550380
     tgaacacaca actacgcatt tttaagcaaa agacgcaatg aagggccttc attgtgagcc    550440
     tagacacaac cctccctgca ggggtgaata ggagcagcag ggggcattcg gggcagtatg    550500
     ggggcttagg atgattgtta ggggtcagga tgagcaggat caaggcttcg catcagggca    550560
     agtgcaacag ggcagaaaag gggctgtaga tgtgggttgt tctcaccatc atatttcacc    550620
     accagacacc ctccactaca tctcttctaa tgtgtctgag tgttgatatg attagaaaat    550680
     ggcatttatg taaatactaa tatgtaccca tatggaggtg catccagttg tcttctccat    550740
     cttatgtgga ccttgtccat caagcactaa gtccctgtat ttacttgagt acctcacaca    550800
     ttatggtcaa aacaggtagg gtctcctttt ggcatggtct ctcctcctgc cttctctctt    550860
     ctgtcacaca aaaacacgtg aactgacaca cacacagagc ttcccaactt taattatgtg    550920
     atgtattgaa gcaaattgat tagtgtgcag cttttctgct ttgcctgcta ttcatgttat    550980
     gtaaaaataa gaaccatgtt tttctcagct tgtcacttct ctaagctaag tagcatcttt    551040
     gtttattata cccagagacc aaaagcgatc caactgttat tcagcagctt aactggtaaa    551100
     caaattgtgg aaaattcact tactggaatc atacccactg ctacaatcaa gtaatgctgg    551160
     atacacccaa caccatgctt aaaatagcaa gtacttgaat aagtaaaata agtcagacaa    551220
     ataaaagtgc atacatacga ttccacttct ataatttcta taaagtaaaa atgaacttaa    551280
     agttacataa aaagatctgt agttgactgg ggacatggta caagaagaga aggtatagaa    551340
     aggagaaact acaggagaac aaaaggaaat ttctaggata attgattttt ttctctgtta    551400
     gtaaaagtga ggattatgtc aatatttgta aaattgtaca ctttatgtaa agattcttat    551460
     ttgctaattt cacctcatta aaatattgca attttttaaa tgtctagttt ggtagaaaag    551520
     gtagtagaga gagatgaata aaatacataa aaatcagaga gtcctgaata cacacatgaa    551580
     tggaccctgg gtctcactgt acttttaggg agacactaga atacaaaaac ataatgacag    551640
     gatttcagta catgaaagga gcttctcaga ccccaggagg catgtccaac tgcgtctggg    551700
     agttaactca gggagcaggc atgtcctttg gaaggagccg tgacaccaag ctcccagcat    551760
     ccattgtagc cgacaccatg ccaatgccaa gagatctcaa ctaaaatttt ctgtggatgt    551820
     tgagtctgat tatgccacac actcacacca agtgagtatg gaaaggatag ttacctgcat    551880
     ccttaaggtg tctgctgaga gcagggcagg tctctcatta aggtccaaag tggcttgata    551940
     gggcagggaa ggagactggc tcagggttgt tatcatggtt tggtggggag cggggcagag    552000
     catcctactt gcaggaaggg ttttgtgggg tttcaaggcc aaattggcat caaatgaggg    552060
     agctcctgtg atttctaact agatttacct tgtatgctta aaaaaagaga tgatggagga    552120
     atgagcctga agttatcagc agtcaggcct aaacacagag tctgatgact tactctaaat    552180
     agcaagtata caaatgatga gtaagaaaag agacaagact ccaatatggg tggacaacag    552240
     ccaggtctgc agaaaatgag aagactgttt ataagcaaag aataatgagt aggatatgga    552300
     ggaggattct ggtccaatgt cttgtgtgga agcttctcat gatttgagat catcagctta    552360
     ttctgaaggt cttaggtcac ctgtcttctt caaaatatca gaagtgcctg atgatatgtg    552420
     aggatgcgca gtttaacttc ctctatttga gtagctttac aattgtgtaa aattcttaat    552480
     taattaattt ttttttgaga cggagtctca ctctcttgtg caggttggac tgcagtggtg    552540
     tgatcataac atagctcacg gtgattttaa attcctggct catagaatcc tcccacctca    552600
     acctcctgac tagcaagtag ctaggtttcc agggcccttc atctacactt ggccggttct    552660
     ttacattctt tttttttttt tttttaggtg gagtttccct cttcttgccc agaccggagt    552720
     gcaatggcgc catctcagct cactgcaatc tccccctccc aggttcaggt gattctccta    552780
     cctcagcctc cagagtagct gggatcacag gcatgtgaca ccatgccccg ctaattttgt    552840
     atttttagta gagatgacgt ttctccatgt tggtcaggct ggtctcaaac tccctacctc    552900
     aggtgatcct tccacctcag cctcccaaag tgctggaatt acaggcgtga ggcaccacac    552960
     ctggcctctt cgtttttatt catatattcc ttcagcagcc actatgtctt cccactgatt    553020
     tcttcagttt ctgccttttc cttttgaata aggctgttac tcctgaggga agatgggagg    553080
     tgggcctgga cagggacttg gtgcattcct ctctcccctc ccagttctta ttggtttctc    553140
     cagtgtctgt agaacagtgg ttttgatggc tttacctctg cagataattt ctcttgcaat    553200
     gtagtgctga tggggaggtg tgtctggatg cacttcacct atagttgctg ttttgctttc    553260
     ccagacagca ccatcccaag gggtagaggg tggagcattt tgtgatgtat ccccagtact    553320
     gaagaaaaag ccttcaatag caggaggaat tcctcaactg catacactct gagaatttaa    553380
     acaataactt ctctatcaca ctcaaattga aaccacccaa tgaatatgtc tactttaatc    553440
     gtgtgctaac ttaaatgaca tttggcagcc tctgtcccag aaaagattat catctgctcc    553500
     tgtttatttc cctgcaagtc tttatgtctg ttcagatttc agatatattg tttgtcctat    553560
     aacatcaaaa atttgatgta tatatgctaa tttgcagatc agtaagttta gtatttgtta    553620
     taagaatagt aacatatttt tatggggtgc ttacatctcc aagctgagaa gcacctctgt    553680
     gtaatactaa gcaactagaa atggtacaaa tatcaagaag atacatagat aaacagtaat    553740
     ggcatgctaa tttatggtaa taacatccat cattagaatc aatacattgt tgatgctcaa    553800
     catgtgtgca tcataagtag ttatgtgcga gaagccacac aaataaaaca catactatgt    553860
     aattcctgta taataaattc ttgaaactca aaactaaggt attaaatgtg aaggactgac    553920
     tcagaatatg gtggaggaag aaaataattg ggaaggagga attgtagagg aacacaagga    553980
     aacttttaag tgtaatttgt ttattcatta tttggatggc ttttggggat gcacaggtga    554040
     gcacgagtgg aataacattt tgttttgttt gtttgtttgt ttttttctga agagatgtgg    554100
     tcctactctg cgacccaggc tggagtgtag tggtgggatc atagttcaat gtagcctcca    554160
     acttctggtc tccaacaata ctcctgcatc tgccatctaa gtagctggaa ctaccgttgt    554220
     ttgccagcag gcttggcttg agtacttatt aaaccgcaca ctttttgcaa tatttaatgt    554280
     atagtaataa tgtctcaata agactattac aaatcaatga atgaataatt tgttctgtac    554340
     agatttatgg aaaaatagac actaacatga tgaatgactg acatttatga aaatacagct    554400
     gcatgaaatg tgcttctctt tacattcatt aggtaaacac aatagtgcat acacatcaca    554460
     ccgtgctttc attacaggaa gaaagatctg aaaatgtcac tggggtgaac catattgtgc    554520
     tgggcttggt tcaggaagca gtcaggccca gtgttgtgac cttcaaccac agaatcctaa    554580
     aaaataataa aagaggctcc cccaaagtcc ccatcagttc ctggactcgc catgtgtctg    554640
     ggccgtatca gtgcatccgg agctccctgg tggctttagg gattccttgc ttgccatgct    554700
     gaggtctcgc tatagattat gttgggtttt ctgaggccgt tttgctattt aagacccact    554760
     ccctggcaca gagcgattcc ctctaaacct gatagaggtt ctgaactaca aataacgtta    554820
     agtgaatcct ggtgtgtctg aactcacatg attgttacat taagctgctg ttgcaatctg    554880
     tttcctcacc tgggaaaaga ggagccagga catagtgagt tgaggcccca ggaagataac    554940
     tgaattctca gagggcacaa ccagcatcct ccttccaggg agagcctaaa agactggggc    555000
     ctccctcatc ccttttcacc tctccataca gaggcaccac ccacatgcaa atctcactta    555060
     ggcacccaag ggaaaccatc acacatttcc ttaaattcag ggtcctgctc acatgggaaa    555120
     tactttctga gagtcctgga cctcctgtgc aagaacatga aacacctgtg gttcttcctc    555180
     ctgctggtgg cagctcccag atgtgagtgt ctcaaggctg cagacatgga gatatgggag    555240
     gtgcctctga gcccagggct cactgtgggt ctctctgttc acaggggtcc tgtcccaggt    555300
     acagctgcag gagtcgggcc caggactggt gaagccttcg gacaccctgt ccctcacctg    555360
     cgctgtctct ggttactcca tcagcagtag taactggtgg ggctggatcc ggcagccccc    555420
     agggaaggga ctggagtgga ttgggtacat ctattatagt gggagcacct actacaaccc    555480
     gtccctcaag agtcgagtca ccatgtcagt agacacgtcc aagaaccagt tctccctgaa    555540
     gctgagctct gtgaccgccg tggacacggc cgtgtattac tgtgcgagaa acacagtgag    555600
     gggaggtgag tgtgagccca gacacaaacc tccctgcagg gaggcggagg agccggcgca    555660
     ggtgctgctc aggacgagca gggggcgcgc ggggcccaca gagcaggagg ccgggtcagg    555720
     agcaggtgca gggagggcgg ggcttcctca tcagctcagt gctctccctc ctccccagca    555780
     cctcagctgt ccccaggact cctctttctt tattatctgt ggttctactt cctcacatcc    555840
     ttgctgcagg ttttaacagt cactggcacc cttttttcaa caaattccca caaggcccat    555900
     tttaccttct tgacaagcaa aatattctaa ggcttctcac tatcccttga ttggcgtcat    555960
     ctactgcatc gtgtctttct cttaaattca tgtgatgaaa ccccaatccc agtggctctg    556020
     tattggacag agggatttta agagggcaaa taagttaact gtggccataa atgtgggacc    556080
     ctaatccagt aggactcttg tccttatgag aagtggaaga catcagaggc ctctctctcc    556140
     acatgcacac agaggagagt ccatgtgggg atgcgctgca tgagaaggtg gccctttgca    556200
     agcctggaag aggcctctca aaaaacttac cctttctgca cccagatctt ggacgtcaag    556260
     actccagaac aataagaaaa ttaatatttg ttatttgaaa tagcctagtc tgtggtatct    556320
     tctcatggaa agccaagtag attcagagat tcaattgtgt tagctccgtc tcctgcatgg    556380
     agtagcagcc caccgaggct ggacacatct ctcaaattat tttcataaag aaaaatagat    556440
     ccaagatgaa aaccccacca catttctgct gagccattca cttagcagca gtctctgaga    556500
     tcagccctgg gggtatgttc taggtcgtgt atctcacttt ccactatgag aatccatgta    556560
     gataatgaga accagccaga tggagtcctg atccctgccc accttctgct tccccccaag    556620
     tgacacacgg tcacagaaaa ccactccagc atcaccctgt gtcttgtact ctctaaaatc    556680
     ccactaaagc tgaccctaaa ctaggagcta tttggggctg gactcattct gttcttagtc    556740
     ttccataagg tccaggtgcc gagtagagcc tcagtaaaga gtgagcttca attcccctaa    556800
     cccacatcat tcccttacat ccttgtcatg cacctgtcct ctgcaggcac ccagggcagg    556860
     ttacacagga attgcctgaa gctggtctca ggtgccctgc tgagaccact ggttatgcac    556920
     acgtatgatt tcaggtcatg caggctcttc tgcaggacag acctgtgtcc tgtagagcac    556980
     agacacttgg tgacccaggg cctcctctaa taactcatac atctcatgtc agtgtatatc    557040
     caggtggccc ctcagtgcag cacactaggg tgattaccct cacaggtggc tttttcaggc    557100
     ggtgaacaga aaatggggtg ggcacgctgg tcattcccat catcggtgat atccaagtga    557160
     attttaaata aatgaaacaa aactaacaac aaaggggaag gatatgtacc tgtatctggg    557220
     aggtagactg ggtcccagaa ctgtactggg agtgggtgtg aaaggccctt ttgtgtggga    557280
     agccctccag catgagtgaa agctgaagga atgatcactg taggtggagg ctgatggaga    557340
     agctggcagg gtacatttcc tgaagctgtt cccgggtgcg tgatggttca atggcccatg    557400
     tacatgaaga ggagggctgg actcatgtag gatcaaggaa actaccccat ggcaatggaa    557460
     gaaaagaaaa tggagatgga cacccctgga ggcagcttat tagctttggt ggaggaacga    557520
     tgggaattta ttccaatgac cccagcattt tcagctaaat atgagttgca ttaacagaca    557580
     gaattatgaa gggaagggag aaggagttta tagagggaaa aagcagaaca gaaaaaaaga    557640
     actatgcaag tctatcaaat ggcagatgac agagtggagc tgatgcagct acttcattct    557700
     tcccggaacc taccagagac cttggcattc ctgtaaggca ggtctagtgg tgatgcactt    557760
     gtccactttt tgctttttgg agaaacgttt gaaacatcct acattactga agaaaagctt    557820
     taattagtgt aatattttta cttgacagtt ttattggttt tgttttattt tcagcaactt    557880
     gaatagaatc atgcacatca tgatatttgg gtgaacaaca ggccacatac acgataatga    557940
     tctcattaaa ttataatact gtattttcac tgcatttctt ccatacttag atatgttcat    558000
     atatatacat acctaccatg gtgattcaac tgcccacagt attcactaca cttacatgat    558060
     gtacagattt gttatctagg agcactatgc tatcccacac agcctgggtg tagcagggta    558120
     tttcacgtag gttcacgtaa acacactcta tgatcctcgc atgatgacaa attgcctaag    558180
     gacacattgc tcagatgtat ccctactgtt aagctacaca tcattgtatc tcatctctct    558240
     ccatttttgg tctctgataa aattctcgtg ttgtttttgc attcttctct gagctcattg    558300
     aacacattta taaggtttcc tttgaatttt ctgccaggta atcaatatac ctccttttaa    558360
     tttgtgtcag tttctagtgc tttgctttgt tacagaattt ggactgcttt ttttctcttt    558420
     cattattatt atcactttgt acattctctg ttgctttctg ctcattagaa ggaaaagcca    558480
     tggtaaccac tcttcacaga atatgctcag ggcaaatgcc atcacctgtt agcccagtcc    558540
     atgatagtgg tgtctcttaa aatttcatac tggtccaaac tttttgttgt tgttgttagt    558600
     ggcactcaga cctatagagg attttaagtt ttcccctact ctaaattagg tggcatcaaa    558660
     attcatcctg caggcagtct ctgataaagt ggagaaattg gacaaagttt tagcatgtat    558720
     atggttcata aaaataatat gagtttaagg ttttctcagc cagttgctac atattgatat    558780
     tttataatca tttctttgaa tgtctctgga gtttatcttt tgaaaattta gatcagaata    558840
     tataccaggt ttgaggcttc ttgtctggct taacgatctc ctataggtca gcgtgtgaaa    558900
     aggcacagag tctatctgaa gaggagaact cagggaagcc ccaaaacaac agaggagaca    558960
     aaactgagga ctttgaggaa tctaaagctt ctgggacctg cagcctccag acatgctcag    559020
     caccctcttg ttatccacgg actaatttct ttctcttttc tgagtggtat tttctagggt    559080
     tcccgtcctc cactttgaca ctttggtatg tccatgtgtg tccattgtgt ttcagccagg    559140
     gacacagaac caggaaaaca tcatggcttt tctagaggga gctgacctgg gtaatcaaca    559200
     gttggagtag aaagtgcctt tgtccatgtg tcacaggcct aagggccatg ggcaggaggt    559260
     caggaagaga ggatgaccag acggctgggg aaaccaggat gaacaaagcc actctccttc    559320
     ctggtgagaa ggggcatgtg gaagcctggt gtccctgtag ggctgaacca cactcacctg    559380
     cccactgcta cctgccaacg aggtgaccca cagagcagca gcagctcctg agtcactcac    559440
     agaggctgcc tcctcctcca aaccctccag tgtatgtgct tccataagtg tgagccccag    559500
     cagatcccaa agttttcaat gggttcatct agctagtgtc tgtcctcagc cccatcccca    559560
     ggctcctgcc ctggttggtg tccacagtgt cccagcagcg gtgacagtga cagccagggc    559620
     ctgcactttc cagagcagca agtttggaag aatcaagtcc cagaaccagc ctggatttgg    559680
     ggcaaacaca caaatttcac tctgagggga agtgctggaa agtagccttg agtcttcctc    559740
     agctcacctg tggctgatcc tctgcatccc atcagcgata catgagccct ccccaacccc    559800
     agcgtggagc tgtttcaatc gcagcaggat gggaatgtgt cttctttctg gggacttttt    559860
     aagcacagct cttatgttcc tgagatttga ggtttataaa ggtgtgtcag tcacccacag    559920
     agtggacctg cacaagtaag acaaggacag gatccccagg agaggcacct catatgagac    559980
     gggacaggca ggtcccatca cagacactgg tcctcagaaa ccctgaccag ctactcacct    560040
     tcccaggtca cctgtgtctg atcagttcct gtgtttgctc ctgaaaagat ccccatcaag    560100
     ggtccccagg tcctgccccc ttccttgatg tgcctctggg agtggggctg gctgggctgt    560160
     gggtctgctg tgactttccc cacaggtcag cggctcagtg caggctgcgc caactcggtg    560220
     gggctgggct gtggccgacc ctcccagggg gagcctggat gcttctcggg ggcatctgca    560280
     ggatcccagg ttctgggaga acagggaagc cacagtgtgt gcagggcccc atctccaggt    560340
     gtaaagtgtt tgctgtcact ctgttgacaa gggcatatcc ccaggcctag gggcactgtg    560400
     ggtgcacaac caatgtggta gacacagaga gtaaggcaca tttccctccc attactgtgg    560460
     ggaatctagg cactgtgtgt gtgtgtgtgt gtgtgtgtgt gtgtgtgtgt gtgtattagg    560520
     agttttgcag tgggctctga gccccgtgat tttgtaggtt tagaaattat ctgatgagat    560580
     gttcatccac aggaaccgac aagagattag gtgttctccc aaagcccctg ggagctcctg    560640
     gactcattgt gagtgtggac tgatccattg cttccggagc tccacagaag gggctccctg    560700
     gtggtttcat agaatccttg cttggggtct ttctgcagag ttcactggct ttcctacggc    560760
     cagtttagta ttgtaagaga tggtgtcagc agcacatggt atcacatgtc actaaatgag    560820
     cattctgagc caggacacag catcttcata ctggggggag ggaaatgctt tgggagccca    560880
     gacaggagcc tctctgcagt gcaagggctg ggctgcaggg ggcgctcaag gcccacccat    560940
     cacaggctcc acccccagag caggtgcacc ggaggctggg gagggattcc tctcaagatc    561000
     tgtgtcattc ttctaaaaaa aatctaaaat gagtatttga caaagactgc tgaacattct    561060
     ataaatatcc tattcaattg tgagcattta tcaaatttga tgttgtaatg agaaccactt    561120
     ttatactaga tatttctaac cctgctagat atcttaatag tatgcagctg gaggttaagc    561180
     aagcttttct tttatataaa taagtgcaac tttggagaaa cacactcatc ccccgaataa    561240
     gacattcatg tattaaagcc tagaaatact ttaaattacc tctgagccat tcaagtgtgt    561300
     gttcacagtg aagtcctgtt ctagggaaaa ttgttctcca gggggagaag ctttgtcaac    561360
     atagagctta gggatgtatc aggaggcaca tgacctctaa ggggattata gcttgaaccc    561420
     ttagcatcct cctgttggta atacatgtgt cactctccaa tctttctcat gcagtatcag    561480
     gtatgaaata ggatcgctca tgaatatgca aataactgag gtaaatatag atatctttgt    561540
     gccttgagag catcatccaa caaccacaac tctcctcaga agaagcccct agaccacagc    561600
     acctcaacat gtactggacc tggaggatcc tcttcttggt ggcagcagca acaggtaagg    561660
     gacctcccag tcaccgggct gagagagaaa ccaggccagt caagtgagac ttcacgcact    561720
     cctgtctcct ctccacaggt gtccactcac aggtgcagct ggtgcagtct gggcctgagg    561780
     tgaagaagcc tggagcctca ttgaaggttt cctgcaaggc ttctggatac accttcacaa    561840
     gctatgctat cagctgggta tgacaggccc atggacaagg gcttgaggaa atgggatgga    561900
     tcaacaccaa cactgggaac ctaacgtatg cccagggctt cacaggacgg tttgtcttct    561960
     ccatggacac ctccgtcagc atggcatatc ttcatatcag cagcctaaag gctgaggaca    562020
     cgtgcaagag gcacagtgtg gaaacccaca tcctgagaga accagaaatc ctgagggagg    562080
     aggcagctgt gctgagctga ggcagtgaca gggacaacgt ggctgcaccc tttgttgaca    562140
     tagggttata cacatacata tatatgcacc caacactgga gcacccaggt atatcaagca    562200
     aatacaatta gagataaaca gagagataga cccaatacca taatagctgg agactttaac    562260
     accccacttt cagcattgga cagatcttcc agacagaaaa ttaacaatga aatcttagac    562320
     taaatctgca tgacagacca agtggacata atggatattg atggaacact tcatccaatg    562380
     gctgcagagt acacaatctt ttcctaagca tatggatcat tctcagggat agaccacata    562440
     gtagaccaga aaatttgtca tttttaatta aaaagatatc atgtaccttc tctgatagta    562500
     atgaaagaaa actaaaataa taagaggaat tttgaaaaca catggaaatt aaacagtatc    562560
     ctccttaatt aaacaatgtg cttccaaact gaactatatg ctctaaaatg ggttaataaa    562620
     gaaacaaaca agaaaatttt acaatttatt gaaagaaatg ataatgaaaa cacagcatat    562680
     gaacacctat gaaatacagt gaacatagta ctaagaggaa agttcacagc tataagcacc    562740
     tatgttaaaa aagtagacaa ttataacata aacatcttag gaatatctct caaaaaaaac    562800
     tagaaaagca agagcaaatc aaatccaaac atagtaaaat aaaaaagtta agatcagaac    562860
     gttaataaat aaaattgaaa taaataaaac aataccaaag atcgatcaaa tgaaaagttg    562920
     ttttttaaaa agataaaaga catctataaa cctttaacca gaccaagaaa aaaaacaaag    562980
     aagaccaaag taaataaaat cagaaagaaa actggaaata ttacaaccaa tagtgcagaa    563040
     aatcaaagga tcattagagg ctactaggag caactaaatg ccaataaatc gaaatatctg    563100
     gaagaaaagg ataaatttag agacacatac aacctaccaa gattgagcca ggaagaaatt    563160
     gaacagatca gtaacaagta gtgagagtga agccataata aaaggtctcc cagcaaagaa    563220
     aaaacccggg acttgatgac ttcatcactc aattatgaca aacatttaaa gaagagataa    563280
     tacctaatct cactcaaaat attccaaaaa tagataaaaa gtagataagg aagggaagaa    563340
     ttcttccaaa cttactgtac aaggccaatg ttacccttat atgaaaacca gataaagcca    563400
     taccaaaaaa aaaagaaaac tacagttcaa tatcactaat gaatatggat gcaaaactcc    563460
     tcaaggaatt cttagcaaac caaattcaat aacatatcaa aagagcattc atcatgacca    563520
     agtgtggctt atcccagaga tgcaaggatg gttcaaccta tgcaaatcaa tcaatgtgac    563580
     acatcgtatc aacagaatga agggcagaag acgtaagatc aggccttacc ttgttctcct    563640
     cactcaaagt ctaccattag ttctttggtc ctcttgacca gtcctgctgt acttggtctc    563700
     ccgtgattct gttcccaggt cctgaaacac tgggcttctg cacacctgga gtctctctct    563760
     ttgaacatgg agaccctgta tcctcagaga gggatgatgc ctcattgtgg ctcccatgcc    563820
     ccatgcaaac aattcttcca acacagtcat gatttttcct gttggaaatt gaaaggggat    563880
     gtgggaattg atgtgtgtgt gtgtgtgtgt gtgtcctcag gcctgttggg cctgttgaga    563940
     acacagcagg gctctatgta caacaggaaa gcaatgtttt ctctatacca tctattttct    564000
     tataaagtgt tcataggtaa tcaaaaggaa atgaatcaca gtttataaga tgttgtggac    564060
     tggactgatg ttgtcactca aggctactat gggggctcca ggaaaagaaa cctagaaaag    564120
     gataagactg tgagcaaaga agcaaaacct gggaagcagc agggagaggg tgagcagggg    564180
     tgactcaact ggggagggat aacaccactt accacagagg gtgaaaactc cgtgctcagc    564240
     aaagaggaga acttactcag catttcccct aatcctccct ccagaagcgc gactgccagg    564300
     aaagttcaca atgtatcatc tccagctggg aacccaaaag aaacaggaga ttttagggcg    564360
     taggtcctgg gcacaaggtg tttccctggt tcctacccct tccctctctg tccagcaaaa    564420
     ctgcttgttt ctatcacttg ccttcctgtc cttggggtaa gaatggatgc tttgacactc    564480
     agcagaggga tgctctttca gaggaggaat gagtttgtgt tatctgatgt ctgagcagga    564540
     cactcacctc tgggaagagg ctccaagaca gaaggtcgcc gtggttcctg ccccttatat    564600
     gtgaggggct cttgacacat gcacactttt cctcaaactc tgtttatacc ccagcaacaa    564660
     tgctgggtgt tgtcagcctc tctcctcccc agatactgct ctatcatcag cttgagcaca    564720
     gtttccccat gaaaggcatt attctctctc tcaagaagat caacttttta catgtcttcg    564780
     atggatgaca gtttttctgt gtgagaatct agaaataaga aatggggtaa tggggacaag    564840
     tgagctctct gtgtgactca ctaggaagtc cactgttatt ccaggtgtcc tgtcccaggt    564900
     gcagctgcag gagtcaggtc caggacccat gaggccctca gacactgccc tctgcctgtg    564960
     ctgtgtctgg attctttttt ttaccatgga tattatttga gatgaatcca ccatcccgtg    565020
     ggcaacaggc tggaatggct ggggcacagt tactagactg gatcatcaag gaatacacat    565080
     catgcaccct ccacccaggt ccatgtcccc atcaacagtg actcaaccaa gagccagttc    565140
     tctgtgaagc tcagctccat gaccacctag gacacggctg agtattactg tgaaagactc    565200
     agtgaggagg tgtccttatg agccctgaca caaacctgtc agggcactta ggacctccag    565260
     gaagactcaa gaccaccaag gggactcacg accactgggg aagggcaggt tgcagtaagc    565320
     acagggccag ccccagagca ggtgcaggtg gagatgagag gctggtttac tgccagggtc    565380
     tggggctgct tctccattta gatgtttcct tcagagaccc tcccttattt catgatgctc    565440
     tgcttagctg taagtctctg aaatatcaca gttttgttgt accaggagga aaccttctct    565500
     caggtgctaa atgtagaaca atctctctgc cagtggttgc cagggtcaca gccctgtgga    565560
     agctgaaggg tacccagtga gtcttctcca gtcacattca ggacaggaac cttagtgggg    565620
     ttccctgagg agaacagtct ttaggaattc taacctcagc caagagaggc tgggccaggg    565680
     tcagtgtcag gtagaacctc acaggattta cgtctgaccc ttctcctgac actaaagcat    565740
     gcaaatcagt atcagcactg atctggggcc ccttttgctc ctagcccatt ctatttcttg    565800
     ttagttgtat ttgttgttga ttttcctttt gctgttcctg atccctgtaa agtggagatg    565860
     tggttcctgc tgtaaaaatt ccagggctca agccctttcc ctgcagctca ggtggggctc    565920
     agactgtggc tcctgcaacc acgtgggaga ggctgatggg actatcttct ctcccgttgc    565980
     tcagaaccct ccagtgtgtt gtgtggagac tcacctggga atgcaagtgg ccaatagttg    566040
     tgaaaggtat gagcttgttt ggtcaaagtg gaatgtggat gtggaattta tcctgtgttg    566100
     tgcaaactaa cacaggttca ccttcctcac ctgtagtgtt agaaagagtg tgaaagttgt    566160
     aagaatcaaa acagagccac ttgtgttaac accctgacaa atggacctag gaatgaacat    566220
     gaaggagggt tcttaggccc atattcctga taacaaaaac tatcataaat agactctgct    566280
     tagccaccat cttggataga agcccaccac aaccttatta aaaattacac ttgcaagaac    566340
     atcagctcag aaactggctg ttcaacctta cactgatgcc accattgcta ttgattctac    566400
     aactatatga taattatctc aaaacaactt ttgtaaccca cctcattttc acttcataaa    566460
     cctgtggatt gacatgttgg agttactgct gcatttgtag ataatgataa ataataaggc    566520
     cttttaacaa tgtttgaagc tgctattctc tgatgtctct tccaaaataa agaatttcca    566580
     agtggatgac aatgaaaaaa ttgttagaaa tatttggtgg taaggcatct ttaacatcct    566640
     gttgaatgtt tctgcatagc acatcaatcc cttaaaatac tttgctatat ctgcatttaa    566700
     tgaatgagtc taatattgag gtgcaattga aaatacctgg gcctttgatg aatcagctca    566760
     tagggtgata atgtctgggt ccttgagaaa gcagaccctg tgttgtaagt tttagtagaa    566820
     gtaccttttg caaagaaagt tcatgtgttt ctgaacatta tgctactttt aacttaagaa    566880
     tgcaatatat gatttatttt tacttcaaac tgggtatata cccagtaatg ggatcgctgg    566940
     gtcaaatggt atttctagtt gtagctcctt gaggaatcac cacaatgtct tccacaatgg    567000
     ttgaactaat tttcactccc accaacagta taaaaatgtt cctatttctc cacatcctct    567060
     ctagcagctg ttgtttcctg acttgttaat gatcaccatt ctaactggaa tgagatggta    567120
     tctcatcgtg gttttgattt gcatttctct aatgactagt gatgctgaac attttttcat    567180
     ttgtctgttg gctgcataaa tatcttgttt tgagaagtgt ctgttcatat cctttgccca    567240
     ctttctgatg gggctgtttg ttttttcctt gtaaatttgt ttaagttctt tgtagattct    567300
     ggatattagc cctttgtcag atgggtagaa tgcaaaaatt ttctccgatt ctgtaggttg    567360
     actgttcact ctgctgatag tttcttttgc tgtgcagaag ctccttagtt tacttagatc    567420
     ccatttgtct attttagatt ttgttgtgat tcatacttgg ttaattttta attgcattgt    567480
     tttcttctga ttagttgtaa gtatacctgc atattgatca aaaaagccgt ttaacaaatc    567540
     aaaaggaaac atttaacaaa tacgtgactt ggaagtattt tctccaagtc tgttgttgtc    567600
     ttttactccc ttgtcagtgt gtattgcaga aaaatatgta tgtgtgtgtg tgtgtgttta    567660
     caaattaaag aaacaggtat aatttcattt atccatagat cacgtctttg gcattacatc    567720
     tgaaatctca tcataaaata tactaatagt gatttttttt tccatgtctc taatctcagg    567780
     ccacaatcta ctcatgagtg tttaaacatc acctacttga ttagaggact atcaccctaa    567840
     gatatttgga atatttctgg aaggagatgt gttctttttc ccattatttc tctatttagt    567900
     catttattaa tatccgcatt ggtttatgaa tgtctatttc atactctgaa gtaaatccat    567960
     gctatattat tcattttatt gttcaaatca ccacagcttt attatgtgct tggaactcat    568020
     ttagtttgga tgctgcatcc ttacagcaca cctcatcatt ttgtttttga acactttcct    568080
     gtttcctggt attacaatac attctaagcc cattttctat attaactttt tcatacatag    568140
     aatcagacat ttttctaaag attgcttctt tctgatgtta aaatatagta ttaaaataaa    568200
     atttgtgata ctgggtatat gtgttgttaa tgtggtataa gtacttttag gacctctcaa    568260
     ccaactggcc tagtaaatgt gcatgtttat atgaactcat gtttaaggac acattgaaac    568320
     tatttatgta tcttatattc tgtaacttta ttacattaaa aatgagaata cactggtctc    568380
     ctcacccaac tatgttacca ccatatcgac ctttctaggc ttccttgact gtccataatc    568440
     acccactgca aagtgaggaa ccccatccca tcatatgcca ttgtattact tagctgcaca    568500
     atttcaggac acatgcattg cagcatcaga aatgtaaagc tgtacccttc ttggaaccat    568560
     ttttatctac tagaatagag tgcttatgtg cagtttcttt acactttaaa cttatagaat    568620
     ttcctcattt tcaaagttcc ttagatcagc aacttcgttt tccactttct tcatgaagtc    568680
     atttcaatta cactgtataa tttctttatt tgaattctgt aaaagccaaa actacagtca    568740
     agtaaacata gagaggatat tccaggaatt tacagagtgg gtatcaaata agttaaaaag    568800
     gcaatgttta agaagagtaa aactattttt agtgatatgc aatggttgag acatgacaca    568860
     attaatttgt ctaagctcat aattttgtga tgaaaaatat aaacctaaat atatacaatt    568920
     taaaatgatg tttagcagta cattatccct aggatcaaat gcagactgta taaaattatc    568980
     taataactta tttggtgagg atgccatgag acacatgcaa caaagaatga actaattttc    569040
     cttatttgca tataagatgt tgacattcac taaagatctt taatttaaaa aaaaatcaat    569100
     tttctatgtg acccaggttt tttcttccag acaagcaaat aacccacagg attattttct    569160
     ttccttgatt gagaaagatt ttccccaaac ttcagctcag ttcaggcata tactgtccct    569220
     aaatgggcat ttaccctcag atgagtccac acacctgtca acacgtggag tcttctgtca    569280
     gacaaacaca cctttactga tgtggattct tccctcagac aaaaacacat gtccccacat    569340
     ggacactttc ctcagactac catgtatgtc cttacattta cgctttcctc agaaaacaga    569400
     cttttcctca tgtggactct tgtctcagac aagcaaatat gtctccatgt gaactcttca    569460
     ctcagataag tacacatatg tccacattta ctgtttcctt acacaggcac atatattcaa    569520
     tgttgaattg ttttgtggca aaatgatctc aagataatgg taattataaa tcccctccct    569580
     aataaagtgt agatctgtat tttttttgtt gtaacctaac tttgccttat tgtcaaaaac    569640
     agtaatttgc agctctaaat gcaccagtta gagacaggtg tccattttct ctggaaatgt    569700
     atttttattt tcttactgga catatttgtt gataatgttg ctactatgaa gatacctgaa    569760
     cagtatccac attaaagaat aaaaaagagt aatgggcaga ataaccctgt gcatccagac    569820
     ccagaaatcc tttgaccctg ccctcccgaa atgtagacac agaggatgga tgagcagtgc    569880
     tgagcagtgt acccatgacc acaaacatga agacatggaa atgtgtcccc actcctcctc    569940
     atgaaaggca gctcatgccc tcttccttca ggccctggta tggagccacc ccatgtctct    570000
     gcccttcctt agtgtccaca ccatggggtc tgcactgatc tggattccct tctcatcccc    570060
     ctcaatatta gtgtgccttg tgaatcaggt ccagctgtgg ctgttctcag tctgtttcct    570120
     ctgtattcac agaagtcctg tgtgatgttt actgatggag tcagaaggag aaaaatttaa    570180
     cagcccagca gttcactgag actgtcctgc aaagcctctg gtttcgggtt tactgggtgc    570240
     agcatgagct tggtccagca cacttcacaa caggaattgg tgtgggtagc aacagtgaga    570300
     gatcatatgg gagttctaag ggttactctc catgagtaca gataaatcaa cattcccaag    570360
     tgacaccctt tcaagtgcag tctaccttac aaggaccaac ctgaaagcca agggcaaggc    570420
     cgtatattac agtgagggac acaggggagg gaagatctgt ctgagcccag acacaaaaat    570480
     ctctgcagag agacaggagg gaactgcgtg gtagatgctg ctcagaacca ccatggggtg    570540
     gccaggaaac aaggggtgct taggacctct agggagcgct caggacgcca ggggggtgct    570600
     cagaaccact aggggacgct caggacacta ggtggcgctc aagacaacac tactgcactc    570660
     atgtcaccac ggggtactca gaaccaccag ggggcgctca gaaccaccag ggggcgctca    570720
     gaaccaccag gtggcgctca gcaccaccag ggggcgctca ggacaccagg gggcgctcag    570780
     aaacactcgg ggacgctcag gacaagaggg ggtactcaga accaacaggg ggcgctcagg    570840
     accaccaatg ggtgctcaga accacaaggg ggcgctcagg acataggtgg tgctaaagac    570900
     aacaattctg ggctcaggtc accagggggc gctcagaacc accaggggac gctcaggaca    570960
     tgcaggttct cttaggaggc agctgcacat caggagcttc agaggctgtg atttcctttc    571020
     agaccctgga aattcctgac ctggtcaagc aaaagtcttc cccaggatgt ctgatttttc    571080
     cttttaattc catgatttct tttacctaca aaacactaac ttagaacagg gatgtaatgc    571140
     aacttttatt gctgcgtatt ttctgagtaa tactagcaat gatctctcgg gacaattatt    571200
     aaaataggtt atttatatat tttaattatt aaaaataata ctattattaa aatagtaaaa    571260
     ttttttaaat cacacttgta atcccagcac ttggggagac tgaggcagac agattacttg    571320
     agctcaggag ttttagaaca gcctggcaac atggtgagat gctttctata cataaaagaa    571380
     gaaatagctg agattggtgc aaccagctac ttaaaaggct gaggagggag gattccttaa    571440
     gcctgggagg ttgaggctgc agtgagctat gattgtgcct ccacacacca gccttggtga    571500
     cagagtgaga ccctgtctta gaaaaaaaaa tgcatatcct caatacagac tatttccatg    571560
     aatagagttt tgtgtttttg tgctgtaata gtcaaacaat tgtatatgtt ttctaacttt    571620
     aactcagcat atgcatggtg ttttgtttct ttttctttca tctgctgttt gtggaaaata    571680
     aacagcactt taaaggctct gtttctccac tttgtttggc ttccagtgtc ctgtttttca    571740
     gactgtttct ccaccttccc ttcttctttg aaagccattt atcttcctca gtctccatgc    571800
     agaaaaagaa agtcccttta ctttctgtct tccaagtctg gtgaatcagt tcccttcttt    571860
     tcataatcac tgaagccaac caagtttaga agtataacag ttctccttag aatacgctca    571920
     tctacctgca ctctctgccc tcctcatcct tttctaggtc cctccagaca tcaccctcat    571980
     cccataccct cccttcctta agtaccacag agtgggctct tcacctcctg ctgccctcta    572040
     tgtgctcagc cctggggctc actagtcctt tgattatgaa gtccaaatcc ctatgtgctt    572100
     gcacactctc acgccaccct ctaggaagct gccattgtga gagagtcctg aaaagcatgg    572160
     gctgggttta gtttcataat gcatactatg gtcaattagt tattctattt gaacattcta    572220
     tttaattaat agatagagtt aattaaatag agttattcta tttaattcta ttaaactatt    572280
     aataaattag ttattcaatt tattaataac aggccaatat ttaaaataaa taccattgca    572340
     cgtttaagtg acatatttga caataattgc atttacatac agttttacca aaacatgtat    572400
     cgaagtgtat ttgttttttt aaatattgga ataggcagac atacacatag gaaaacatta    572460
     ttttgtacta caatctcaaa ctgcaaacac aatttaaatg ccattaaata attagaataa    572520
     tatgaaacaa atgggcatgg tgttttggtg tttaaatata cattcatttc acctgatcat    572580
     acttatgtgt ctttgctggg ctgctgtgca tgtatgtgta tttgtatgac catgaagctt    572640
     tcagatacat cattaaatta cataaagtgt taccatgcac ttccctcttt taggggacaa    572700
     agtaaatctt tcagggcaat cgcagggaaa atgtgtggtt ggatcttcca cacagagtcc    572760
     ctaccatgac attgccaagt agagctatgg aagggggcta ccaacctcca gaccccagaa    572820
     tggtagatcc acaggcagct tgcagctcga gtctggaaaa gccacaggca cccagctcca    572880
     acccatgaga gcagccttat gggctgcatt agggaagtca caaaggtagg gctgcccaag    572940
     gccttgggca cccacccttg caccaggtca caagacatgg agtcaatgat tattttagag    573000
     ccttaagatt gaaggactgt ccgactaggt tttggccttc cataaaaatc tttacccaat    573060
     ttttgggcca ttttctcttt ttagcatagg actcctcacc caatttctga cccatcattg    573120
     tactttggat gtaaataact tatttttgat ttttcagtct cataggtgaa aggaactttt    573180
     ccttgagtct gaactttgga ttttgaatta atgccaaaac aagttcagat tgtcagggac    573240
     cactggggag ggattattgt atattgcaat gtaagaaaga cataaggggc agaatgatgt    573300
     ggtctggatg tgtgttttct ccaaatctta tgttgaactg tgaaccccag tgttggaggt    573360
     gaagtctgct gggaggcatg gcattagggg catatccctc atgagttact tcgtgtcatc    573420
     cccttggtga tgaatgagtt atcagttcac atgacagctg attgaaagag cctggtacca    573480
     ctcccttctc ttatcttccc ccctattgtc atgtgacact cttgctcccc ttcatcttct    573540
     gccttgactg taagctttct gaggcactca gcagaaacag atgtcagcac cacacttgct    573600
     gtacagcctg cacaactgtg agccatataa aacctatgtc ctttataaat tatccagcct    573660
     caggtatttc tttacaattc aataatggac gaacagaaga agtatttaat tgtgcagcaa    573720
     attaggggtg tggaatcatt aaaatggact taacgcctgt agaggtaaag acacaagagt    573780
     ctctctctct ctctctctct ctctctctct ccttgaaccc ttctctgtct ttacatacac    573840
     acacacacac acacacacac acacacacgc tccacgtgaa gatgtcatgt gaccaaatag    573900
     caatatggtg gctgctatga gccaacagta gaagcctcaa aataaaacct atcttgccag    573960
     catgtttatc tttgaatttc cagaattgag gaactgagat aaaattaagg ttgtgtaagt    574020
     taccaagtat gattgttttt aatgtttgaa ctgactaaag agaatttggg atgaagaagt    574080
     ggggttctga tgttacaatt atttcaacac tgtggaaagt gccttggaat tgggttatgt    574140
     gtaaaaggct ggatagtttt gacatgcata aagaaattta tatattaagt gtcattctga    574200
     tagattaaaa tgagtagagt gctattggaa actggataaa tggtgatctt tgttataaag    574260
     tgacaaaaaa aacttgtcta gactatgttg tgatatttag taaaagagaa ctttgagcag    574320
     caaaattgaa tatttagctg agaacacttc taagtaaaat gttgaaagtg tagtttggtt    574380
     tctcctgggc atttataata agaaatagat gaattgaaaa tgaaattata agggagaaaa    574440
     tatccaaaac ttaaaggctt ggaaaattct caatctatct gtgttgcaaa aatcagaaag    574500
     tgtgttctga agagaacagt gagtatgagg ctgtaatatc acttgataaa gaatttatgg    574560
     tattaaaaga gaataaatgc aggttttaaa gaacaaggat ggagatgaga caaaatggac    574620
     gaatactgtc agattcttca gatttaagag gatggagcta cttggttgca aatgtgcacc    574680
     attcttccag aacaaagaaa aataaacctt gaatttattt agaagtaatc atgaccacta    574740
     ccttggtttc aacagaccag accatccatg cttaaaacct tgaggaaaag acctcctacc    574800
     actgcctgga acttaggaac tcccagtgag ccttgtgtgt agggctgcat ggggtctttg    574860
     ggatgggacc ttaacccagc agttacccat tggtgcctct acttaaagca ttggaattaa    574920
     ttctgctgct gcaatgagct gggaaagaag aagcttggcc cagagaagat tccacttgag    574980
     ccttaaggtc tatggtaatt tgttttgtta tgtgttgtac ttgctgggga cacatcaccc    575040
     ctcctttctt ttctattttt ctctttgggg gagaaaatgt ctattttatg cctaactcac    575100
     cactgtattt tgaaatcaaa taacttgtcc agcgctatgg actgaatgtg tatgcaactc    575160
     caaaattaat attgtggaac cctaatcccc agtgtggtgt aattggggtg tgggccattt    575220
     aaggggtgat caggtgaaaa ggttgaagcc gtcataaatg agattagtgc tctaataaaa    575280
     agtcacggga gagcttttct ctcttgcttc cctgtcatga gatgatacga taaaattacg    575340
     gccatctgca attcaggaag gaaaccttta catacacatg attggctgtc ttgggcttcc    575400
     cagcattcgg cactgcacag aataaatatg tcttgttaaa gtcatgcact ttatgacact    575460
     ttgttaaagc agccccaata aaataagata tctggtttta ctggctcaga gttggagaga    575520
     aatttgtttc agaaataatt ctacctcaaa tcttactcat gtatggttta gttaatattt    575580
     aaattatgtt ttaaacttgt ttagagttga tgatgaaatg agttaagagt ttggggactg    575640
     ttataatcaa atgaatgaac tgcgaatgtg taaatgccat gcatttgggg gatggtcttt    575700
     tctattctgg gtctttcatg cctacgtgtg aattgaagga ttgcttttct tatttctgtg    575760
     aagaaggcca ttattatttt gatagataat ttatctaatc tgtaaataac tttgggtagt    575820
     atgaaaattt caatactatt aattcttcca atccataaat gtggagtatc tgggatatat    575880
     gtcctattta tgtgtgtcca tttcattttc tattaccaat aatttatagt tatcctctag    575940
     aaatctttct cttatttggt aacatttatt cctaggtata ttttgcagct attgtaaaag    576000
     gaaaggcttc atggatttca ttctcagatg ttttgctgtc aacatataga aatgccacca    576060
     attattgtat gtagatgatg aatcttacaa ctttacttaa tttgcttatg aattctaata    576120
     gtttgtgaga aggctttagt tttctttttt taatataaga tctttttgtc tgctcacagg    576180
     aaatctgact tccacaattc caaatttgag gtcatttcct tttactgtta actaaatact    576240
     ctggctagca tttcagtact attctgaata aaagtgataa aaatggttat ttttgtcttc    576300
     ttccacttct tagatgaaaa tttcccggtt tttccatgct tcgtgtgatg aaagctgtgt    576360
     ttttctcata tatgcgcttt attttttgag atatccttct tgtataccta gtttttgata    576420
     atttctatca gaaatgaatg ttgaatttta ttctatgatt ttctaaaata tcttgactac    576480
     acaataacac agtaatatgc aggtcaatac atatcaactg aatttcactt aatatcatcc    576540
     caacttctaa tatttatttt ttagtattag aacatgcctg tttccatatt atatttgctc    576600
     acaggtattg gtacatattt tagctaattt gtcattattt tactctaaga aataactcaa    576660
     aaaatcattt ttattatgaa ttgaaaggta acatcggcaa aattagccca atcttaatct    576720
     ttcccagact ttgaaacatt attgaagaca atgagagggg aaagtcagct ttagttgagc    576780
     ttctaagagg taagtgaggt aaaaacccag ggcttattgg ctacgacttt gaatgttact    576840
     gtcttctttt ccataagcca catccagacc attttctaaa tctaagaaaa ctaattttat    576900
     ctcactgctt cttagtcttg tttcctgcag ctgacaacca aaactgtcag cgatagagcc    576960
     cagtgtaaat caccagcctt taattattgc agaactatga ttttacaaca acattgtgag    577020
     ttacataatg atcctacttt atggccgcgg tgcaaatgct taacttccac taaatcacag    577080
     agctcagaat ggctagcatg gaggtgtata tgtatatgtg cattgttcta gaaatttcca    577140
     attactgtat attattgttt gcagtaatta ggtcaatctg aactgacaaa agtctccatg    577200
     gccttataag cacagagacc acctccacgg caggtggaaa tcaaggctgg gaacagtcta    577260
     ccagctgctc ccctgggatt cctccaatta ccctgtttaa agcctctgct cactcatgcg    577320
     ctggaagagg gatgtcagag atgcagtcta ctcttcctaa agggctgaca gggcataaag    577380
     acaagactga ttatccatgg aacattccta taggaatgca cctatgtgca gacacacaaa    577440
     taagctgtgt gtcttgcagt gtgtaaatgg ctgagatgcc atttacactt ctttacacaa    577500
     tacattttta aatgttttgt tgatgtattc tctatcattc aaaaaaatca tcactttccc    577560
     cctaaacaaa gaggattttt atcagaacac ttatgggaag ccccttgctc tcccagattc    577620
     ccaccctcat ttctcctgaa ggaagaaaga aaaccatctc tattgatttc cactctcatc    577680
     ctctaggact aaaaccagaa agctgcatgc tccctgggtg agccttagag aagaccctgt    577740
     ctgtaggagc aggaatctca gagccttggc tgagaggcat ggtcctgaaa tgagatggag    577800
     tcccccatgg agatcccacg tggacgccca cacctgaggg ctcactgctc ctcaccacag    577860
     atgcactccc ctactgagtc ctgagacctg agtgcacccc atagagtagg actcagatga    577920
     gggaatgcaa atctccacca gctccaccct cctctgggtt caaaagctga gcacggggcc    577980
     tcgctcagtg actcctgtgc cccaccatgg acacactttg ctacacactc ctgctgctga    578040
     ccaccccttc ctgtgagtgc tgtggtcagg gacttcctca gaagtgaaac atcagttgtc    578100
     tcctttgtgg gcttcatctt cttatgtctt ctccacaggg gtcttgtccc aggtcacctt    578160
     gaaggagtct ggtcctgtgc tggtgaaacc cacagagacc ctcacgctga cctgcaccgt    578220
     ctctgggttc tcactcagca atgctagaat gggtgtgagc tggatccgtc agcccccagg    578280
     gaaggccctg gagtggcttg cacacatttt ttcgaatgac gaaaaatcct acagcacatc    578340
     tctgaagagc aggctcacca tctccaagga cacctccaaa agccaggtgg tccttaccat    578400
     gaccaacatg gaccctgtgg acacagccac atattactgt gcacggatac cacagagaca    578460
     cagcccagga tgcctcctgt acaagaacct agctgcatct cagtggtgct ccctccctac    578520
     ctctgcagaa caggaaagtg tgactgagat gccatttcct gccagggctt gtgtttccta    578580
     taccaaccag actcagagcc ctgtctgttt tcctattctg tactattaaa tggcatgttc    578640
     cctgttagtg attcacgcaa gcagaggctg tatcctgttt gacaaagatt gagcatgaaa    578700
     gattcccatt acctgggcca catgcatcac tgatatgtgg ccacttattt cttgagctca    578760
     tatccttcca aggggctgaa tacaaatatc tataacatag gccatcttaa actgcaggaa    578820
     gtaatgttgg agaagaatgt ggagataaaa gagcaggatg attaattaaa atccagatac    578880
     catctttttt tgcagagaaa aatttacact aataaagtaa ttttatgaaa caacaagaaa    578940
     gatggaatgt gtcctcatca tggagtgtct cacttgcagt gcacaaataa atttctaaaa    579000
     attttatagg gaaggtgtga tagatgaggc tgatttccac acagggagtg attaaatacc    579060
     caggtaagta taaaatccaa cacttggaaa ggaaaatgcc tcagcttgac atcagaaacc    579120
     cagctcagta agtctgagga gcaatgcgga aaagaaatga aaaattcggg ccgggcgcgg    579180
     tggctcacgc ctgtaatccc agcactttgg gaggccgagg cgggcggatc acgaggtcag    579240
     gagatcgaga ccatcctggc taacacggtg aaaccccgtc tctactaaaa atacaaaaaa    579300
     ttagccgggc gtggtagcgg gcgcctgtag tcccagctac tcgggaggct gaggcaggag    579360
     aatggcgtga acccgggagg cggagcttgc agtgagccga gatcgcgcca ctgcactcca    579420
     gcctgggcga cagagcgaga ctccgtctca aaaaaaaaaa aaaaaaaaaa aaaaaaaaaa    579480
     aaaaaaaaaa agaaatgaaa aattcgacaa cacttttctt atgaaatccc tagctcatgt    579540
     ttgaacacga aaacagaatt gtccacaatt ttaggacagc catcgcatga caacaaacca    579600
     ctacaatgat aggagcatca ctgagtcaac ggagttcaag cagtgagcag atcagtactg    579660
     aggccccagg agaaccaagg ctctttggta cattttacag aacccagaac cgagccacat    579720
     aatctatggc caagtgatct ttgacaaagt taacaaaaat taaccaacgt atacaccagg    579780
     gaaaggacac cccattcaat aaagggtgct ggggatattg gatagctata tgcacgcgaa    579840
     tgaaactgga cccccaccct caacatgtaa ataattaaca caaaatagat taaaggttta    579900
     aatgtaagtc ttcaaactgt aaatgtattc aaataaaaca ccaggacaac tcttctggac    579960
     aaaaagcaaa caatagacaa atgggactta atgaatctaa caagtttctt cacagcaaca    580020
     gagtgaacag acaaagtgca gaatgataga aaatatttgc acactgtgca tctgacagag    580080
     tactaatata cagaattaac aagaaactca aaccacaaca acaaaagaaa acaagaacca    580140
     aataacccca ttaaaatgag caaagaacat gagtagacat ttttgcaaat aacacataaa    580200
     aatggacaat agatatataa aaatgctcag cattactaat aatcagggaa atgcaaatta    580260
     aaactagtga gatataccat tcacaatggc tgttattaaa aagtcaaaaa taacagatgt    580320
     tggagaggat gaaatgtaaa gggaactctt agacactgtt gatgagaatg tggacgagta    580380
     cgacctctat agagaacagt atggattcag tatgatattg actgtgcatt tgtcataaac    580440
     agctcttatt attttgagat acattccatc aatacatagt ttaatgagag tttttagtat    580500
     taaggggtgt tgaattttat tgaaggcctt tgctgcatct attgagataa tcatgtgttt    580560
     tttttccatt ggttctgttt atgtgatgga ttatgtttat tgatttgcat atgttgaacc    580620
     agccttgcat cccaggtatg aagccaactt gatcatggtg gatttgaaaa ccggcacaag    580680
     acaaggatgt cctgtctcat cactcccatt caacatagta ttggaagttc tggccaagga    580740
     aatcaggcaa gagaaataaa taaagggtat tcaaatagaa aaagaggaag tcaaactgtc    580800
     tctgtttgca gatgacatga ttatatattt agaaaacccc atcatctcag ccccaaatct    580860
     ccttaagctg ataagcaact tcagcaaagt ctaaggatac aaaatcaatg tggaaaaatc    580920
     acaaacattc ctatatacca ataatagaaa aacagagagc caaatcatga gtgaactctc    580980
     attcacaatt gctacaaaga gaataaaata cctaggaata caacttacaa aggatgtgaa    581040
     ggacctcttc aagagaactg caaaccactg cccaaggaaa taagagaggg cacaaacaaa    581100
     tggaaaaaca ttccatgttc atggatagga agaatcaata tcgtgaaaat ggccatactg    581160
     cccaaagtaa tttatagatt caatgctatc cacatcaagc taccattgac tttcttcaca    581220
     gaattagaaa aaaactactt taaatttcat atggaaccaa aaaagagccc atatagccta    581280
     gacaatccta aggaaaaaga acaaagctgg aggcatcacg ctacctgact tcaaactata    581340
     ctacaaggct acagtaacca aaacagcatg gtactggtac caaaacagag atacagacca    581400
     atggaacaca acagaggtcc cagaaataat gccacacatc tacacccatc tgacctttga    581460
     aaaacctgac agaaacaagc aatgtggaaa atgatcccct atttaataaa tggtgttgag    581520
     aaaactagct agccatattc agaaaactga aactggatcc cttccttaca ccttatacaa    581580
     aaattaactc atgatggatt aaagacttaa acatgagacc taaaaccata aaaacccaag    581640
     aagaaaacct aggcaatacc attcagtaca taggcatggg caaggcctta atgactaaaa    581700
     caccgaaagc aatggcaaca aaagtcaaaa ttgacaaatg ggatctaagt aaactaaaga    581760
     gcttctgcac agcaaaagaa actatcatca gagtgaatgg gcagcctaca gaatgggaga    581820
     aattgtttgc aatctatcca tctgacaaag ggccaatatc tagaatctgc aaggaactta    581880
     aacaagctta caataaaaaa aaaacaaccc catcaaaaat tggcggaagg ttatgaacat    581940
     acacttctca aaagaagata tttattcagc caacaaacat aaaataaagt tcaacagcat    582000
     tggtcgttcg agaaatgcaa atcaaaacca caaagagata ccacctcaca ccagttagaa    582060
     tggcgatgat taaaaagtca ggaaacaaaa gctgctggag aggatgtaaa gaaataggaa    582120
     cgtttttaca ctgtggtcgg tagtgtaaat tacttcaacc attgtggaag acagtgtggt    582180
     gattcttcaa ggatatagaa ctagaaatgc catttgtccc agcaatccca ttatttggta    582240
     tatacccaaa ggattataaa tcattctgct ataaagacac atgcacacat atttttattg    582300
     cagcactgtt gacaataaca aagacttgga accaacccaa atgcccatca aagatagact    582360
     ggataaagaa aatgtggcac atatacacca tggaatacta tgcagccata aaaaggatga    582420
     gttcatgtgc tttgcatgga catgggtgaa gctggaaacc atcattctca gcaaactatc    582480
     acaagaacat aaaaccaaac accgcatgtt ctcactcata agtgggagtt gaacaatgag    582540
     aacacaagga cacagggagg ggaacaaaca tcacacaaca gagcctgtcg gggagtgggg    582600
     ggctggaggg gatagcatta ggagaaatac ctattgtaga tgatgggttg gtgggtgcag    582660
     caaaccacta tggcctatgt atacctatgt gacaaacctg cacattctgc acatgtgtcc    582720
     cagaacttaa agtatagtta aaaaaaaaga gagagagaga gaaaggaaac agtatggaga    582780
     ctttccaaaa aatgagaaac agtactggcc tttattctac caatcccact actgggtagc    582840
     tacccaaaga gaaagaaatc atatttcaaa aagataccca cacttctatg tgtaccacaa    582900
     atctattccc aatagcacat atgacaacct gagtgtccac caacagatga ttttataaaa    582960
     gaatatagca tatatgcaca atttaatact agtcagccac aataaggaat gaaactgtgt    583020
     cttttgcagc aagatgccta gaactggggg acattataat tagtgaacta actcacaaac    583080
     agaaagccac atatcacaca ttattactta taagtgggag gaaaacagtg tgtacacaag    583140
     gatatggaga gagggattat ggacattgga gacttagcag aatgggaggg taggagttgg    583200
     gagcatgatg aaaaatcacc taatgggtac aatgtacgtt acttgggtgt tgggtacact    583260
     aaagccaata ccactgtgta atatttccat gtaacacagt tgcacccata gcccttaaat    583320
     ttatacaaat aaagataaaa acaattacaa tttaaaatat aaaattatta atagttgacc    583380
     aaagatcttg aaaattaaaa tttaaatcat gatcaataaa tgaaattaat atcagttgaa    583440
     cttcatttaa cttaaaatcc ttttgtactc aagtgatttt aagaaaatga atgtcaagtt    583500
     ttagatgagg agatttgcaa atcatatcac cacctatgta attatagtaa gaaccctcaa    583560
     aactcaacag tgaataaaag aagaggcaac ccatggataa aataggcaaa ggtttgtgca    583620
     ggcatttcat taaagaagat gtacagatta cacataggca tataaacagg atctcaacag    583680
     gatttttcat cacagaaatt caaatcaaga ccacaataag atacccaaac acccttttta    583740
     gaatggctga aattaagaag aaagatggat cataccgatg ctggtgagca taccaggttt    583800
     ctagagtcta aaacattgct aatgggaatg caaaatgaaa cagctacaca ggaaaataat    583860
     tcttagtttc ttgtaaaatt atatatgccc ttaacacatt acctaaaaat ccccctcctg    583920
     taatacagta atacatggct gtgtattact gtgaaagagg catagtgtga aaacccacat    583980
     cttgaaaccc tgagggagaa ggcagctatg ctgtggctga ggagattaca gggattattt    584040
     gattaaagac ttttttagaa agtgaggtta aatcagccgg gcgtggtggt tcacacctgc    584100
     aatcccatca ctttgggagg ccgaggtggg tggatcacga agtcaggaat ttgagaccag    584160
     tctggccaac atggttaaac tccatctcta ctaaaagtgc aaaaaattag ccgggtgtgg    584220
     cggtgtacac ctgtaatccc agctactctg ggcgctgaga caggagaatc tcatgaatcg    584280
     gggaggctga ggttgcagtg agccgaaatc gacccactgc accccagctt gggtgacagt    584340
     gcaagactcc gtctcaaaaa aacaacaaca acaacaaaaa gaaagcgagg ttaagtcatt    584400
     gaaaaatagg aaaatataaa tgtgtatgca ctctaattat ttgggaaatt ttccatacaa    584460
     cttgtattct gtaaacaaaa ttcagggagt ggaaaacaaa tcaaattaat aaaactgata    584520
     taagaattcc tctgaagata ttagtgtgag cactaacaca cacttttgaa tgggtgttgt    584580
     aaatattttg gaacacacct cttaggtcac attttaattc tacatttatg taaattatat    584640
     aaaatatcga aattttttaa tgtctccatt ttgtgcagtt ataattttgg tttcatgcca    584700
     gcaatgcatg atagattccg ttcttccaca tcctcgttgc catttggcac tataagcatt    584760
     gtgtatttta accattctaa tctattggtg gtgatagctc attgttgttt aaatgcacat    584820
     gtctctaaaa ttttatattt aattatttta tataattgtg atgaagtgtt tgttatggta    584880
     tttggctcat ttttgatagc attgtttatt tttgatcagt tgtaagtttc tttacatacc    584940
     cattatataa gtcacttaaa aaagtactta gctggtcatg aaccccagga tgagatgcag    585000
     actgtataga aatctctagc aatgttactc agtgtgggca gggatggcat gagatacggg    585060
     gaataaagaa taaagtaact ttctccattt ggatataaga tgtgtccatt cactagagag    585120
     ttttctttca gaaggttccc atgtgagcca gtttccttcc tgacaaggaa atcacccaga    585180
     ggattctcat aatctcttaa gtgagaaaag ttttcctcaa actccagctc agtctagctc    585240
     acactgtctc taaatgggca attactttca gacatgtaca cacatctgtc ccaacgtgga    585300
     ctcctccctc agacaagcac acacattgca atgtggactc tcccctcaga aaaccacaca    585360
     tgccccacaa tgactccttc ttcacataag catatatgcc tgatgctgaa ctgctgtgta    585420
     gcaaaattag ctcagaatac aagcacttat ggactctagc cataacaatg tgtagatctg    585480
     cattttattt taaattctaa atgaacattt tgccttcttg ttaagaacag tggtttacag    585540
     ctctacatgt agagactaca ggcagatata tgtttcctct gggaaaagtt tattttcatg    585600
     tatttactgg gttattttgt tgataaacat tcatctataa agacacctga aaagtgccct    585660
     cattagagaa aaagaaagtg tagtttgcag attaactctg agcatccaat cccaggaatc    585720
     ctttgatctg ccctccctga aatccacagg cagagggggg atgatcgctg ataagtagca    585780
     aacacatgtc cacaggaagg gagacatgga aatgtggtca ctcccacact caagaggagc    585840
     agctcatccc ctgtccgtag tggtcctggt gaggaaccac cccacacctg tgccttcctc    585900
     agtatccaca ccatggggtc tgtgctgatc tgggcttccc ttctcatcac tctcaatatt    585960
     agtgtccttg cggatcaggc tcagctgtgg ctgctccaga tgaggctgtt ctcagtctgt    586020
     tccctctgtg tttgcagaag tcctgtgtga agttcaccgg gggagacaga ggaaataacg    586080
     gtgcagccgg gggctatctg agtctctcct gcaaagactc tggattcacc ttcactgatt    586140
     gcagcataag cttggtccag caagctccag gaccagggtt gatgtgggca gcaacaggga    586200
     gaaattgaag aggaagctct cagtggtgcc ctccatgaat acaaagaatc ttcacagtcc    586260
     ccaggacacc cttacgtgca tggtctcact gatatcttta cttcctttat cacttttgtt    586320
     atgtaaatca caatgaatag tgtattcctc atctattata catttgttaa gtcttttttg    586380
     gtgtctttaa aaaaactgat aactttatag tatgtaatat ccttaagtcc tgaaagtgtt    586440
     ttttgatgtc taccttgtct taaatttata cagctactat agcttttctt ggttcatgtt    586500
     ttctcatatt tatcttttct atttgtgaat atgtagagaa actttcttgt acatagctaa    586560
     tagttgtgta ttgctttttt attttggcag tccttaaata ttctaatttt ttatgatact    586620
     ttaagtttta gggtacatgt gcacaacatg caggtttaaa caaacaacct catcaaaaag    586680
     tgggcaaagg atatgaacag acacttctca aaagaagaca tttatgcagc caaaaaacac    586740
     atgaagaaat gctcatcatc attggccatc agagaaatgc aaatcaaaac cacaatgaga    586800
     tatcatctca caacagttag aatgacgatc attaaaaagt caggaaacaa caggtgctgg    586860
     agaggatgtg gagaaataag aacactttta cactgttggt gggactgtaa actagttcaa    586920
     ccattgcgga agtcagtgtg gcgattcctc aggtatctag aactagaaat accatttgac    586980
     ccagccatcc cattactggg tatataccca aaggattata aatcatgctg ctataaagac    587040
     acatgcacac gtatgtttat agcagcacta ttcacaatag caaagacttg gaaccaacct    587100
     aaatgtccag caacgataga ctggattaag aaaatttggc acatatacac catggaatac    587160
     tatgcagcta taaaaaatga tgagttcctg tcctttgtag ggacatggat gaaactatct    587220
     attgcttttt taaatcaact ataataaatt ctattttttt ttttgagaag gagtctcgct    587280
     ctgtcgccca ggctggagtg cagtggtgcg atcagggctc actgcaagct ctgcctcctg    587340
     ggtttatgcc attctgctgc ctcagcctcc cgagtagctg ggactgcagg cacccaccac    587400
     cacgcctggc tagttttttg tatttttagt agagatgggg tttcaccgtg ttagcgagga    587460
     tgctctcaat ctcctgacct tgtgatccac ctgtctcagc ctcccaaagt gctgggatta    587520
     caggcatgag caataaattc tatttttaaa ccaacaatat ttttttctgt taatttttgt    587580
     ttaaattaac attttataat gatgcttatt tctctattaa catggtattt aattaccttt    587640
     ttataaatat tgtatagtta ccataagatt tacaataaaa attgtatgta attagattct    587700
     aatgtaaatc ctgtgatggc cttgatatga agaacacaga tagtgtaact gtgtgctttg    587760
     aattcctcct tctcgccact ctttcttgtt tattctgttt gcacttacat atgctgtaaa    587820
     atagaatatg taatttttta ttactttata cagttatatc ttatagcaat tacaaatgtt    587880
     aaagagtaca attcatctag atttttccat ttttacaatc tttatttctt tgtagagatt    587940
     tctattttga gtgcatatca catggctact cgcagagaat ctttgtaaaa tgtgccctga    588000
     aacatgaatg tgctgacaac acatattctc cagatctttt tttctgacag aattttattt    588060
     gccattcacc tttaatgaaa ttttaatgca tgtagaattt cagtttgcat ttcacctgta    588120
     atttattttc ttgtaattat tattagtttt tcctggagat gatcacacat tacattctgc    588180
     tgggccttta ttaaccatgt ttctgcgaac ctagtcaggc ttgggtttca agtgtatggt    588240
     tgccgtggct atcagagttg aaatcagctt ctcctgttca caaaaacttc aggttcctct    588300
     ggtgatacct gcgtttgtgt ccctgtttgg ctttggatct tcacatttca ttctccccaa    588360
     agaaaggctg tctctttcag ctgtggcagg tgcatcctgc tgacacattt acttggtgac    588420
     tgtttgtggg atgaaggggc ttggacacag gggcacgttt tccaaccttc tggctgagcc    588480
     tccttcttag ctatgggtgg tgagactggc tctgatgcat gatcttccaa gtgttcctgt    588540
     tccttccctt cctcagtgtc acagtgtctc ttcccagtca tgctgttttt tcatcagtgt    588600
     cctcagcttc tgacccactg tccttacccc acagattctg gatttcattc ctcaggaaac    588660
     agacgggagg tgattctggg tagagtttcc ctgatgtcct ctgtttcctt gtgttctagt    588720
     tgattctacc agtgcccaca ggacacaaga tttaataaat gtctcctgca tataatgaag    588780
     ggggattcag cattgaacgg agctactgtt cttcctcccc agtcaacacc acaggacagc    588840
     aggtgggtga gttgtctggg gatttcccca attctgtagg aaaagcctgc aagtgccagg    588900
     agtttcacac tctcacacca ttagcacaca catcttcaag caactcatta aacatttcca    588960
     ggttagcttt ttcctatctt caataccatg ccatgagtgg cacctgcccc aggtactcta    589020
     ataaacgcac cctagttctc tctgcgggcc cctattttct catatttcag ggattttttt    589080
     ctctgtgaca tcaactcaga tatggtgagg ggtttatttt ttgtaattct tcatgttttt    589140
     tgttaacgag gtcagaataa tatcattttc tcaattttta cattctaacg cttagtatct    589200
     gatttctaaa taaaattcag aaactaagac aacaaatgaa gtatccatta tttggtgcat    589260
     tgttaaacaa tttgagaaat attcatgtac tgaaatagaa tgaaccactg aaatcaaggc    589320
     atgcctcatt cacataagaa catgagggca ttatcaaata attttgctga gtggaggaag    589380
     ctaaagaatt caccatcaat ttcatgtgat ttcatttgta taaatcacag aagatgcaat    589440
     attctaaatt aacatagaga agatctgaat ttctctgaaa atagggtggg cagagtaaca    589500
     acgtggtgaa ataaaattat agacaagtta gataaaaaaa attagaggtt aacttaattg    589560
     ctaaaacatg attgaagtgc tgattcaatg actgcacaca tatatcaaca ttttccaaat    589620
     gctgcactat caatttgatt tcattattga ttgttttttt aataaagcag taacaaaaat    589680
     gaatatattg gctgagaaag agcaagaaat ggataaatac cgacacttga ataatcaagg    589740
     actcctgaaa atacacacat gtgaacactg attgtagatt tcaggtaaac actagaaaag    589800
     gcaaaattac acaaagttgt tatgacagga gggacatcct gaaaacctct ctaggcatgt    589860
     cccacaccac cctggagctg tctcagggga gcagtctcct ccagtgttta gaggcacagg    589920
     cacgcataat agggctaact ctggccagat gtgtgatatt ggacacattg cacaactgct    589980
     ctgttctgta tgtaatttat cttctctaca aatgtaacat tgacagttgc attaaatata    590040
     ttctgcaaat atgtaaaagt taaataagct gatgtctgct aaatgattac caaggcacaa    590100
     tcacataatc tgaagttata ttttccagag tgataggatt acctccagag ttttccagga    590160
     cactctcatc tgctccgggc actgccttct cctcagccgt cccacccctg agcttgctat    590220
     atagtaggag acatggaaat agggcccact ctctgctgat aaaaaccagc ccagccccga    590280
     ccctgcagct ctgggagagg agcccagccc ccgaattccc aggtgttttc atctggtgat    590340
     cagcaccgaa cacagaggac tcaccatgga gtttgtgctg agctgggttt tccttgttgc    590400
     tattttaaaa cgtgatctat agagaactag agatattgag tatgaatgga tatgagtgag    590460
     aaacagtgga tacgtgtggc agtttctgac cggggtgtct ctgtgtttgc aggtatccag    590520
     tgtgagatgc agctggtgga gtctggggga ggcttggcaa agcctgcgtg gtccccgaga    590580
     ctctcctgtg cagcctctca attcaccttc agtagctact acatgaactg tgtccgccag    590640
     gctccaggga atgggctgga gttggttgga caagttaatc ctaatggggg tagcacatac    590700
     ctcatagact ccggtaagga ccgattcaat acctccagag ataacgccaa gaacacactt    590760
     catctgcaaa tgaacagcct gaaaaccgag gacacggccc tgtattagtg taccagagac    590820
     acagtgaggg gaggtcagtg tgagcccaga cacaaacctc cctgcagggc catgcgggtg    590880
     gtttcctttc tcagctgcag gaggcgggct tattgttgca ggactctgga gacttattag    590940
     gttgtgatat tttactatgg ttatttatca tgaacttttt tattgggaat tgtgttttat    591000
     taatttttat tttatatgta aggttatttt tttaaaatac ataccttcaa gaaataattc    591060
     ttcctaataa tttgcacttg ttctttttag agttttgtta acatctgttg atatcagcta    591120
     ctacatagct atagggacat taatttacat ctatagacat atgtgtaaat acacaaactt    591180
     atacatatat gtggtctttt atatttaatg ttataataaa attattataa caattataaa    591240
     atacatccta aagaatgaaa catatatagt aatccatatt cctttcaatc attgcctata    591300
     gtttacataa attgatgtct atttgtaacc ttaaacatag tgtattggtc attttcaaac    591360
     atccaagaac aaatttcaaa tgtccttgtc acacacacaa aaataaaaat ttgaggattt    591420
     gaggtgatat atatgtcaat cagctcgatt caattattcc atattgcatt cataaatcat    591480
     aacatagctt tattcactat aaatacatac aaccaaaatt tctcaatttt caatgaaatt    591540
     ttaattatac atattttatc tgtcctaggt catgaatgtt tttctccctg tccggatgtg    591600
     attaagattg tcctgagaaa caaattcagc ctcctgcctc ctgaaggctt caggaagcag    591660
     ctcctagatg gggtgaagca ggcaacccgt gtgtgtccac aggacccgga gcctctctct    591720
     ccttggatta ggccccctcc tcaggattac agggctcttc agttttctca acatgctgtt    591780
     ataccagaga agcacaaaac acttaatttc attatatttt tcttcaattt ttcaaaacaa    591840
     cacaaaggta ataattttaa caataaacat attacaacct actatacatg agacccttcc    591900
     tgtgcttcga ggtttcttct caggactttg tatgtattac atgttttcag ttttttcctg    591960
     agatggaatg ttgacgtctc cttattacag attattcttt taatttgtct cttagaataa    592020
     ttttttccag agctcctgcc cgcccgcgcc tggcggaatg gagcggcgcc agggctgagc    592080
     caggcgcgca ggggccgccg cacgtgccgc gcggggagcg gatgcccagc gggcggagag    592140
     ccagcgccag aggccgggtc ggagcggccg ctggagcagc gtcagaatgg gagaacagcc    592200
     catcttcacc ccccgagcgc gggtcttcca gattgacccc aacaccaaga agaactggat    592260
     gcctgcgagc aacaggcggt caccatttcc tgtttctatg atgtcacaag gaacagctat    592320
     gggatcatca gtgtggacag cgccaaggtg accataaaca acacaattac acagaatatg    592380
     accttcacca acacgtcaca gacgtctggg cagtgggccg acagcagagc caacacggtg    592440
     tttggtttgg ggttttcctc tgagcagcag ctgacaaagt ttgcagagaa attccaggag    592500
     gtggaagaag cggccaagac agccaaagac aagacccagg agaacatgga gccctcgagt    592560
     aatcatcctg agaatcaggg cgtggagccc catcttctac tccggcatcc agtgtcaacg    592620
     ggacggacga tgaaaggcct ctcaggccgc tgcagccgac acgcacctgc agtctgggaa    592680
     ccacaagctg aaggcggcct cgacgcagag ctgcccacgg gaagaggtgg gagatggagc    592740
     tgcagacccg gcgggagacc gacttccggc tgcccacggc gctgcaggag tcgacggcca    592800
     gtgcggagca gaggaagagg ccgtgagtca gagtgcgact gtgtctctga gaagatggag    592860
     gcggcagaga gaggatcaaa acctggaaga taaagtgcgt tccctaaaga taggccctga    592920
     ggagagcaaa tcccgacagc gccacctgga ggcgcttcct ggtggcgctg gaccggaaga    592980
     tccaggcctg cataacttcc gcccaggcct cccaggctgg gcaccaacaa ctagggctgg    593040
     cagaggcccg ggatccccac cgtgagtcct aagtatgtgt gcgagaccag atggcgctag    593100
     gacgttccct gtgtgcgttg cttctgtaaa tgcaggcgcc gtgtgtcgtg tttccaaacc    593160
     agttgtgcca tccactcact cctttccaga atagaaatct ctcgcttctc tggccttgtg    593220
     aggttgtgga caactggaag attctgactg aggaaaccag aaccaggtct accttcaaca    593280
     tttacgcagt caggccaggg atgtttatat cgttcataag ggttgttgga accatatgaa    593340
     ctgaaaaaaa gcattttctc atccaaatat tgatattctt tacaccaggc catcgggctc    593400
     cttttatccg atagcattca gagtatttga aggtcctcca ggtgccagcc ttgggggcac    593460
     agggagaaca acattcctct ccgaaaacat ggggaagctt taaaataact gtttagtgga    593520
     gacttaccca gagatggaaa acaggtttct ggtgagtaca ttttctggtt tgggaatcac    593580
     ctgcatcccc gatcctgccc cgtgcccccc agtttgtatg gttgtgacaa tgttccattt    593640
     ctttgtttta atttctgagc agttgactgt ggtgcgggaa cagcacacag tgagggtgcc    593700
     tagcacaatg cctggcgcaa agtaggtgct ttataaatat ttgttcaatt aaaaaaagag    593760
     ggaggctgag gcaggagaat cacttgaagc tgggaggtgg aggttgtggt gagccgagat    593820
     ggcgccactt cactccagcc tggcaacaga gcaagactcc atttcaaaaa aataaaataa    593880
     aataaataaa ataaataaat aaaataaaaa atattttttc caagccccac tcaaaaccat    593940
     ggtattgtta gaggtgtaat agttggacca acattgaaaa tacattccat ttagttcatg    594000
     ggcgtgcaaa tatccttttt tctttaaaaa ttgttctcag ttgtaatatt acaaaaaata    594060
     ttggtaattt ataccaatga cacaggttaa aatgttgtat aaataattta atgacgtttg    594120
     attggaaaaa atacatgtgc cacatgtcga caattttttc cttctctatg aaatatatac    594180
     tcattaataa gtttcagatg ttacagttat ctttttgatt tctgggattt aattttagta    594240
     ggtatacttg aatgcatttt attaattcct ataatgatgt tgatatttca tgtaggattt    594300
     taatactaat tttactattt actgaatttt aaactattct acatgtttta tttatataag    594360
     aaaaaaatta catatgagaa aaaatgcgta gacctgaaat gtatcactag agagtttctg    594420
     tcaaatgtga atacctttgt tcccaacacc taaggtagcc tgaagagcaa gtccttcccc    594480
     acaacatgcg tctttttctg tgcttgcggt cagctcctgc atggggaaat gttttgattt    594540
     ctgacactat agatccattt tttttctgtt ttgaacttta tataaatgca atcaaacatt    594600
     ttagacctta atttggtaat gggctacatt gctatttttt gaggttattt cctgctattt    594660
     aaggtattaa attagatcaa catattatca tttattcaag taatggactg actctgatat    594720
     gaaacctcaa agttttcaca tatatatgga gagagagaga gagagagaga gatgagggag    594780
     agaggtttta tatctgagtc agtctgttaa ataagtaaat gacagtattt tgatttattt    594840
     tcctgtaaat cgactttaaa tttgtttcca gtttatgaat attataaaca aagctgttac    594900
     aaatgtctta gtgtaagttt tcctatgttg tcttcttttt attgagaaaa tatgaagtat    594960
     gtatttatta taagcttaaa ttttttcagt tttattgagg tataattgaa atattttaat    595020
     aatgtatagg tttaaggtgc cccacttgac gttttgatgt tgtattagtc cattctcacg    595080
     gtgctataaa aaatgcctga gactgaggga tttgagaaaa aaagtgtgtt taattggctc    595140
     acggttctgc aggctgtaca ggaagtgtag tggcttctcc ttctggggag agtcaggaaa    595200
     gttaggatca tggcagaaga caaaggggag caggtgcgtc acatggccag accaggagcc    595260
     acaggaagca gggaaggtct acacactgtt aggcaactag atcttatgag aactcacaca    595320
     ctgtcatgag aacagcacca ggagtttgtt ctaaaccatt catgaaagac ccaccccatg    595380
     tcccagtcat ctcccaccag gtcccacctt cacaattgag gattataata caacatgaga    595440
     tttggggcag gacacagatc caaaccacat cagatacaca ttgtgaaatg ctcatcatgg    595500
     ccaaagtaat ttgtatatcc attttctcat ggagctacca ttttattttt ttaaaatgta    595560
     ctacatgtgt gtgtatgtgt gtgtgtctca tgagaacacc tacgatctac ccgtttagca    595620
     aaaatcacat ttacaataca gcattaaata caagaacatt gcagtacatt agatctgcag    595680
     aactcattca ccctgcacaa ctggacctct acccttcgac cgacatcatc tgatttccct    595740
     ctcctcacag gcctgggacc cactattcta ctctctgctt tcaagagctt gaatatttta    595800
     gatcccacat gtaaatgaga tcattaagtc tttctgcatc tggcttattc cactaagcat    595860
     catgtcctct agccccatcc ctgttgttgc aaatgtcaga atttccttct tttcaaagcc    595920
     taataaaatt cagttttctg tgtacacatt ttctttacac attcatcaat ctgtggtcat    595980
     taaattattt tacaaatcta cactattata aataatcttg cagtgaacat gtgtttgaca    596040
     taataatttt atttcttttg aatatatgac aagaagtggg atcaccagag catatgatag    596100
     ctttattttt caatataatg aataaccaaa cctaccacac catgcttttg aagtcttagt    596160
     catattttat ttgcctaggc caatgtcccg aaaaatttct cctacatttt cttcagtttg    596220
     tacatttaaa tattgaatcc atattcaatt aatttttgtc tatggtgaaa aactagaagt    596280
     ctagttttat tcttctatgt gtggctgtct aagtttttca gcaccactta ctgaatagag    596340
     ggttgtttct ccattgcata tttttgtcag ttttgtcaaa gaacaggtgg ttgtagaaac    596400
     ttgtctttat ttcttggctg tctattcttt tacattatta ttattgttat tgtttttagt    596460
     agcagtacca tgctgtcttt cttactgtat ctgtgtagta taatttgaaa tcagggaatg    596520
     tggtgctttc agctttggtc tttttccttg ggatttcttt tgctattcag gctgtttttt    596580
     gattccatat atatttaagg atttttttta aaacatgtaa tcaattatat tggttacttg    596640
     ataaaaattg cattgaatat gtatcttgct ttggacagtg tattagtcta ctgtgcattg    596700
     ctataaatta atgcctgagg ccaagtgatt tacaaagaaa agaggcttat ttggcttaca    596760
     gttttggagg ctgtgtgaga agcatggcac cagcatctgc ttcttgtgag ggcctcagga    596820
     agcttacggt catggtggaa ggcaaagggg aagcaggcag tgtcctgtgg tgagaggggg    596880
     cacagaagag gggaggaggg tcgctagact gtttttaaca atcagatttc aaattagcta    596940
     ataaagaaat aattcactaa ttaccatggg gttgatacca agccatacct gaaggatctt    597000
     ttccccacaa tccaaaaact cctactagga cccacctcaa atattttgga tcacatttca    597060
     ccgtgagatt tggaagggac aatcacctaa actgtagcat ttcactcttg tgtcccctgg    597120
     tctcaaatcc ttgtaagatt gcaaaatcat cttttttaaa aatagttccc aaaagtgtta    597180
     acttgatcaa cctccaactg aaaattacaa agtctcgcca gggtcttaag gcaatttccc    597240
     tccagctgcg tgctggcaaa atgttttaaa gagttatgta cttccaaggt gcaatattgc    597300
     cattgggtaa atattgtcaa tacaaaagga ataaattggc caaaggcccc acacacacct    597360
     aaagcctagc aggacagata ttaaatctta aagctacaaa ataatctatc ttgactttat    597420
     gttcttagtc agggtacacc aggacaaagt gggtcccaaa atcctcaggc atctcatcct    597480
     tatggctggg cacattccat ggggctaata acacaggcca gagtcaagtg ccagaagtag    597540
     ttccaggttg ggagtacaag ctgcctttgg atctaccatt ctgggatctt gagggttgtg    597600
     gctgcatttc tgtagctcca ctgtgaagtg ccctagtggg gactctgaat ggggttccaa    597660
     ccccatcttt ccccatcttt ggtactgcct tcgtagaggt tgtttgtggt ggatccagtc    597720
     ctgtagcaag cttcctcctg ggcacatagg gttctctaca catcttctga aatctaggta    597780
     gaaactgccc agcctccttc actcttgcat tctgcatatc tgccagaatc cagttatccc    597840
     agcaccattt actgaacaca gagtattttt tccacattgt ttgtttttgt cagccttatg    597900
     aagtatcaga cggtttaggt gtgcagcttt atttctgttt cctattttgt tccgtttctt    597960
     ctgcatatct gctcttgtac cagtaccaag ctgtttttgt taccatgtca ttatggtgta    598020
     gcttaaagtt gggtatcatg atgcctctgg cgttcttctt tttgcttagc attgctttgg    598080
     ctattcaggg tcttttttgg ttccatacaa attttagaat agattatttt ttctaattct    598140
     gtgatcaatg atgttgatag ttttatgtaa atagtactga atctctaaat tgctttagac    598200
     agtgtgataa tttttacaat attccttctt ctaatccata agcatgaaat gttttcccat    598260
     ttatttgtgt catgtatgat ttatttattt atttatgctg tgttttgtag ttctccttgt    598320
     agagatcttt caccttgttt gttacctgta tttccaggca tttcatttgt gtgtgtgtga    598380
     atattgtaag tgggatagga ctgtgttctt gatttcactc tcagcttgga catagttggg    598440
     tatagaaatg ttagtgattc ttgtacatta agtttgtatc ctgagactct accaaagttg    598500
     tttatctatt ttagaattat tttggcagag tctttagaat tttctatgta tagaattata    598560
     tcatcagttt ggacagatat atctacttct tttcccattt agatgttgta ttagtctatt    598620
     tgcacactat aaagaaacac ctgagactgc ataatttata aagatttttg attgtctcat    598680
     ggttctgcag gctgtacagg aagcatggct ggggaggcct caggaaccca acagtcatga    598740
     cagaaaggga agcatcatgt cttacatggc tggagcagga agagagagga ggtaggtgcc    598800
     aagtacttgg aaaccacatc tcctgagggc tctgtcatgg gaacagcaca aaagagggtg    598860
     aagcatcccc atgatctaat catctctcac cagtccccac ctccaacact gggaataaca    598920
     atttgacatg aaatttgggt ggggatgcaa atacaaacca tatcagctcc gttttatttt    598980
     ttctattgcc tggttgctct gactaggatt tccagtagta tgctcaatag gagtggtgag    599040
     agagggcatc cttgtcttat ttcagttctc aaggggtttg agcttttgct cactcagtat    599100
     aatgttgagt gtggttttgt catagatggc tgttattatt ttgagttttt tttttttttt    599160
     ttttgagacg gagtctcgct ctgtcgccta ggctggagtc caatggcatg atctcggctc    599220
     actgcgacct ctgcctttcg gggtcaagcg attctcctgc ctcagcctcc catgtagcta    599280
     ggattatggt tacgggcatg cgccatcacg cccagctaat tttttgtatt tttagtagag    599340
     acagggtttc actgcgttag ccaggatggt ctccatctcc tgacctcgtg atccgcccgc    599400
     ctcagcctcc caaagtgctg ggattagagg cgtgagcccc cgcaccaggc tattttgagg    599460
     tattttttga tgcctagtct tttgagagtt tttatgagtg aatgttggat ctcatcaaaa    599520
     gctttctcag catgtattga gaaaatcata tgttttttgc ttttattttg cttacatgga    599580
     gaatcacagt tactgaattg tggatgttga atgaaacttg ccttccaggg gtaaagccta    599640
     cttgatcatg acgtatcaca ttttaaagtg cttctggatt ctatttgtta gtattttgtt    599700
     gagaaatttt aggtctatgt tcaccaagaa tattcatctg atgttttctt ttttaattat    599760
     gtatcttcct gattttgtat cccaaggaca cacaatgaag gaaggacagt ctcccaacaa    599820
     atgatgtaaa aactggacat ccatatgcaa aataaataaa attagacctt ctctaacacc    599880
     atatacaaaa atcaacaaaa ataaacataa catgaaacca taaactcata gacaacatgg    599940
     aaaaataatt catgtcattg attcagaaat gatttatttg aagttaatcc caaaagcaag    600000
     cccagcaaaa aactacatgc aattatatat ctgacaagaa gttctatcaa aatgtataaa    600060
     taatccatac aactcaatag caaatatcaa atgacctcat aaaaagagca aaaacctgaa    600120
     tagatttttt tcagaagaca catgcatgaa aaacagagca taaaaatgtc ctcaagatga    600180
     ctaattgtca gaggaattcg aatcaaaagc acaatgacat atcacctcac accacttaaa    600240
     atggctatta tcagcaagac aaaagataag aaatgttggc aagaatgatc cacagccata    600300
     aagagagctt tgattggcca agagactgat ttcagtaact ttctcctgtg aagagaccac    600360
     tgaccatgga cttgtcctgg ccaatttcca gaggctgtgc actgtgtgtc tttctgcccc    600420
     tgcacaaagc ccttttgata tgcaggaccc agctgtaatt catttagttc ttaagtcctc    600480
     accccaaagc aaagctgaaa tgcatgtatc atgtgtgttt gcttattaga catgagagcc    600540
     tccacgccat gtgaatatcc atagctcctt ctatagcctg ctgagtgtgt acactcggcc    600600
     aatccatttg cacaaatttg tttctcatct ttccctctct taaagtgcct gctcacagtc    600660
     tctgtgggag gctttccttc ccagcctgtt aagatggccg tcctgcagct tcaacccttt    600720
     ctgagaaata aagtctcctt tctaaattta taaactgggt gattcttccg ttgaaagcac    600780
     aagctgttgg gaagagttat atggcaattc cataaatgcc tggaagacaa gtgtggatag    600840
     ggtagggtaa gaagtcctgg gggctgcaca ccccactctg atggaatttc cctccagaaa    600900
     cttctgggtt ctcaagatga gaatccaaac agattccctc atggctctgt catcaggaga    600960
     actatactct gataagtatg cccagaactt cctccagaca gaccctatag ggaaaaaata    601020
     cttttcaaga tctttattct gtgaggggaa gggattcttt tccatcccag acagcttcat    601080
     cttagccttc ctgagtcatg aaaaggagta taattaataa aacatctggg gtcagattca    601140
     agaaaataat ctgtggatgc tgcagccagg agggggagtg gaggacggag gaaaatcagc    601200
     tgtaccactg aatactcctt gtaaatggta cagcctagag aaagaatagc aagaaatcat    601260
     tggaattcaa tttccagaac atatactgct cccctaccca ccacatcacc tcaccaccaa    601320
     tatgattaat ctggattaaa gagaaaagtg tggaaaggca cagactccat ctaaggacta    601380
     gaatttaggg aaaccaaagg cagtgggaga gaaaaagtca aggacagtaa agtgacttaa    601440
     aacctcttag accaacagct tcaggaacaa ggtcacatcc tctccctcac tgaaattaga    601500
     tttacctctc atggggcatc tgcagggtgc gcaggtgaga actggcaaag agaacatgaa    601560
     ggcactttcc aaatctccat tagtacctag ctcgctttag ctgtttggaa aaagcaaaac    601620
     cacaacaaac ataaccagga ctaggatcag tgttggaagc acttttcact ggcaagacac    601680
     taagaaggag ggcaaattcg aggccagtta ctatgcaaat atgccactgt gagtgtgtgt    601740
     ggtggtgagc tttgagacat agggtctgtg ggtaaagtta taatcgataa aatcttcaac    601800
     cacagaatcc taaaaaaaag agccttcccc aaagtcccat cagttcctgg actcgccatg    601860
     tgtctggacc gtatcagtgg atccggagct ccagggaagg ggctccctga tggcttttat    601920
     gattccttgc ttgccgtgct gaggtctccc tgtagattat gttgggtttt ctaaggccta    601980
     ttttctattg taagaggtgg tgggggaaac aatttctgtc actgaaagag cattctgagc    602040
     cagggcacag ccccttccta ctgagcttga gattctggga ggaaaaagtt ctctgacagg    602100
     aggaaaaagt tcccagacag gaacttccct gcagggcagg agctgagcgg cagggggcgc    602160
     tcagggagca cccagcacag gacccagccc tggggcaggt gcacaggagg ctggggcagg    602220
     gttttctctc aggaattgaa tgttccttat ttcaaagcaa taatgaccta acatttaaat    602280
     aagaatttag caagtactga tgtgcctgta agtattttat tgtatacgca gcctatacct    602340
     aacccaataa tttaatgcaa accggattta aaaggagaaa tgtctaggtc tttcaaatgt    602400
     atttctagtt aggaattgag gggtggtttt attaattcaa tgggtgttac tgtccggaga    602460
     cacactcatc ccagaagtta gatgtgcaga gggcaaggct caggaaaagt tcaggtagac    602520
     agtgagccat atgaacaggg aatcacattg aggaccatgt cctgtgagat tctgggtttc    602580
     tgtgagagga gttctgtctt catggacttc tgggcatgtc agaggacaaa tattaaacaa    602640
     agttcagggc agggagctca gcatcccact gtggcgtggt ccacatgtca cctatcttct    602700
     tcctcagggt ggcgtggcct gagctatgaa aaacctgcct catgaatatg caaatgcact    602760
     gctgtctacc gaggtaaata cagatctttc cttgcccaga gaccatcaca caacagccac    602820
     atccctcccc tacagaagcc cccagagcgc agcacctcac catggactgc acctggagga    602880
     tcctcttctt ggtggcagca gctacaggca agagaatcct tagttccagg gctgatgagg    602940
     ggactgggtc cagttaagtg gtgtctcatc cactcctctg tcctctccac aggcacccac    603000
     gcccaggtcc agctggtaca gtctggggct gaggtgaaga agcctggggc ctcagtgaag    603060
     gtctcctgca aggtttccgg atacaccctc actgaattat ccatgcactg ggtgcgacag    603120
     gctcctggaa aagggcttga gtggatggga ggttttgatc ctgaagatgg tgaaacaatc    603180
     tacgcacaga agttccaggg cagagtcacc atgaccgagg acacatctac agacacagcc    603240
     tacatggagc tgagcagcct gagatctgag gacacggccg tgtattactg tgcaacagac    603300
     acagtgtgaa aacccacatc ctgagagcgt cagaaaccct gaggaatgag gcagctgtgc    603360
     tgaggctgag gagatgacag gatttatgaa gtttaaatct gtttagaaaa tgggttgagt    603420
     aattgagtaa aacagcaatg gaaagatgtg tacactgtca ttatgtagga aatagtattt    603480
     tcaactgtca ccctataagt aacactcaca gagtgggaaa ggcagccatc aatcaagctg    603540
     atgcaaacat tcccatgggg gctttgtggg gacatacatt ttataattgg atctataaat    603600
     cattagaaac aggattgctt gatctcatgg taagactaaa tataatttcc aagaagtggc    603660
     cgaaatttct tccaaaatgt ctttgccctt ccttttacac tgaatttatt ttaaaggagt    603720
     tttaggatca caacaagttt gagtagaaga aacagagttc ccatgcactc ctgccccccg    603780
     acgctcagcc ttctctgcta tcaacaccct gcaccagtgt cataaatcgg ttacagagga    603840
     tgaatctcca cagacacatt ggttgtttcc ttttctggtg gtccctggta taacaagcct    603900
     caactatctt gaagcacccc aggttcaagt ggattcttca agtgggttac tgggaatgat    603960
     gccaggtaga aggaaagtgg gtgggaccat tcctctttgc actctttcct caaggatcca    604020
     taaaatgtac atttacatgg agctcctctt tctcctggtt ttatatgcct cttccccaat    604080
     ggtaaggttt cccagtgttt acagcggggg tccccagtcc ccgtgctgtg gatccgttaa    604140
     ggtgtgcagt gcgttaggaa ccgggccaca cagctggaga tgagtggggg cgagccagca    604200
     tcgctgcctg agctctgtct cctgtcggat cagctgtgca ttagattctc ctacaagccg    604260
     aattcacttt ttttatatat atactttaat tctagggtac atgtgcataa cgtacaggtt    604320
     tgttacatag gtatacatgt gctatgttgg tttgctgcat tcatcgaatt ctattgtgac    604380
     ctgcacatac aaggggccta gactgagcgc tccctatgag aatctaatgc ctgacgattg    604440
     cagggggaac aagttcatcc caaaaccacc cgccgccatc atccatggaa aaattctctt    604500
     ctaccaaact gttccctggt gccaaaatgg ttggggacac tggtgtagag gcaggtctgt    604560
     accttggaaa gccagcagct tctggtgaat cccataatta atgtccttta atgagaagtg    604620
     gagactttgg tcatgagggc cttcgtgaat aatgccctac agttggattc aaaacattgt    604680
     aagctcttgg ggggtgtttc tggataaagc cctttgtgca gaaaatgcag acacatccat    604740
     gggatccatg caggaaaaaa gcttccttta caaagtggtt tggcacctgg aaggagctct    604800
     cagggttggg cactgtaccc tttgctggct gcactttagc cagaggctta agcctgattg    604860
     gtcttgcagt gggagagcct tcctggggtc aaaggttaca aaaatgcctg tcatcttcca    604920
     gctgagcaag cccatctgcg tgcttgtcag tgtcaacccc atgaggggtg cactctggaa    604980
     gatgacaaga tgcacacaaa cctcctccca ctgattatcc actaccacac actcagctgc    605040
     aacccgtgct ccagaaacgg acaggggcct gcaaggataa acagaacgga agtattttat    605100
     tacctgggaa agcactgcca aatgccacgt ttcaggaagg ttaactcata agtgttcagg    605160
     aagtgactga agggcagtgg tgggtgaggt gacaggactg cctcagggct tcacatgagg    605220
     agggctccct cccacacagc cttttcctcc aggagctgca ccaggaactc acagaggatc    605280
     aggaaggatt ctgagaacat tcttctgtgg cgctgactag aggggcaaaa taaattataa    605340
     aaaataaatc aattctaaat atagtatgat gtttgttatt agaaactatt tctggatcct    605400
     gtgggaaccc tgacataagc caccattctc tgtgaacaaa catttaacct ctccttccat    605460
     ggggaggtcg acgagcttgg attggttctt tcatggatag tatcccccaa actcctctcc    605520
     actccctgcg tatcccccgg tctctcagtg agaagttctc ctgagtggaa tgtggttgat    605580
     gtagtgagat cttcaaattt cccgtcttct gtgttctcat gttctcccct tatctgagat    605640
     taagaaatgc acctgcatgt agtaaattat agcctactgt ctctcatgga caaaataatg    605700
     gccacaccaa ccaaccctgt ggctgacagc aaccactggc ggccaccatc ccctccacag    605760
     cctgtgttgg aacatcacct gagagattgt gttagcaact ttccttcaga atcatcttaa    605820
     aaactttgtg tcccatttca catggagacg tcatttttcc atttcatctt catagagaaa    605880
     tagaaggaga agaataccaa atacacttcc tatctccaga ttattcaaat ataattgtcc    605940
     ctttatcacc ttctgatttc tggtcttcac agaatactct acaaattttt ctcactggcg    606000
     ttataccaaa agttgtgaag caccagatac tgggattaat ttaaccaaca ttattattct    606060
     aataattctt agaatttaga attacaatta tcatcttttc gtatacggat aaactaacct    606120
     atgcctttga aaataagccc ttacatgaga gtgaaaagct atagatttgg aggatttcct    606180
     tgcaaatcca atatttggaa aaggtttttt atcacaaata tacaaattaa cttacaattg    606240
     aacaacaaaa ccacagaatt taattttaaa atgggcagag acccgaatag aaaccatatc    606300
     caaaaaacaa acataaaaag tgatcaactt gattttcatt aggtgtactt atgtttaata    606360
     tttaaaaagt tctactaaac tatgagaatg ggtaaaaatt acaaggtaga caatatcaga    606420
     tggtgatgag gaagttgaaa aacaggaact ttcacccctt gctggtggga atgccgaaat    606480
     ggcacataga aaactacagt taataattta ttgtatattt cagaatagct acaagataag    606540
     atttggaatg ttcataacac taagaaataa tcagtgatag agatgaagga tatcctagtt    606600
     atccagattt gatcactgca cataaaatgc ttttatcaaa atatcagttg taccacctaa    606660
     atattttgaa atattgtgta tctatataaa tcaaacatta aaaaacacca atgcagtgga    606720
     atccagctaa ttgcagattc ccattagatg ggatgtgctg catgatctgt aaactaatca    606780
     gaatctgtgg gtttgtaaag gactttagaa atcaactagg ctgcttgttt ctaaagataa    606840
     gtctccctag tttaaatatt ttcttaatgg gtgacgattt tgaacataga agaaatagta    606900
     atatttgtgt cttgtaagtt ttcttcttct catgtcacga gaaaaggttt gcagggagga    606960
     atctgggacc atctttcaag tctcaacttc actgtgatta cctcagtaag tattttctga    607020
     tacccctgac taagataagt ctcgtatctg tcctagaaac taaaagtaga attctaagca    607080
     tctacctgac cagatggacc cttcctcttg accaaggagt tccagagaca cttttaaacc    607140
     tagctccggc cgtgttagaa atagaggatc agaaatgcct cattataccc tcctcccttt    607200
     ggaattcaag cacaactaac cagcattttt atgaaaacag agcttaaggc tcagaaaaca    607260
     gattatttgt agcagtaaga taccaaattc taaccttact cgagaatatc atcacaggac    607320
     agagagcagg cattgaaagg aatcaaaggc ttttacccta aaatatattc ctttgacata    607380
     ttttaaaata gccaggcaca gttatctttt gtgagagaga tttacattct gtagagaatc    607440
     tccttccctt tccaggtctt tttcttatac cgaggagatt agctgagggt ctagcttctt    607500
     ttaaaggtct taggagaaaa catttcccag ctattgcctc taaaggtggt cacataagag    607560
     acttcatcta catagtaaga atcttgttct ccaaaacctg ttatcttaac ctagaaacat    607620
     ttttctaccg gtttcaggtc tttagaaaaa agcttaaatc tttcaatcaa ttgtcaatca    607680
     gaaaatattc gaactcactg taagttgtaa tccccgcccc acactgcctc acttctagct    607740
     gttttgtctt tatggaccaa atcaacatat atctcacatg tattgtttgg tgactgtctc    607800
     tctagtacac taaatttaag ctaccatcaa gccactttgg gcacatgttc tcaagatctt    607860
     ctggggctgt tcacagttca tggtcctcac attcgcctca gaataaatct ctctaaatat    607920
     tttgcaaggt tgctttttca tcagtagttc taacagaacc tactctgtgg tttttgttaa    607980
     aagcattata ttaatatagt attagtaata ttagtattag ttataacaca tgttggaaaa    608040
     ttcttatgaa gttatgcata gactaacaat gggatcaagt gtaaatgttt caaagtattt    608100
     aagcaagtta tttcaaaatt tatcttctgc agatacttct atcagattca ttatatcagc    608160
     taattacttg ctttatgatt tttgcatttt attgtagatg atcacaccct atattttctt    608220
     ggcctttatc gtagaaaatt gtgtgaaact agtcagggct acatttgcaa gtaattgtcg    608280
     cagtggctgc ctgaattgca gtgggtttcc tctgcagcca ccagtcactc agattactcc    608340
     aatgacacct taattttgtt tcctgcttgg ctttgggcct tagtcttgtg ctctccagac    608400
     agactctgtc tttcatctcc cctaggtcag gccagagcca catggacctg atgattgtca    608460
     atatggtggg aagggtcgca ctgaacgggc actctctgtc cttctgaggg agccatactg    608520
     caggcagaca tggcagtcct ggctcagggt gtgtccttcc aagtatccct gcccttcctg    608580
     caggtgtgat gctgatccat gcatcttcct cccattcctg ggtagagggt ctccttgttc    608640
     tttccccaat cttctcccag cagccactat gtcttcccac tgatgtcttc agcttcctct    608700
     tttctgccct tcccagcaga tgagtctgtt attcctgagg gaagagagag gaggtgggag    608760
     atgaggctgt tattcctgaa agaatggaca gagctttgga gcttttcttt cttctacccc    608820
     agtttctacg agttcctcca gtgcctgtaa cacagagctg tcagtggctt tgcccctgca    608880
     tgttaattct ttggtcatgt agtggtggtg agggagctgg gtctggatgc atttcagcca    608940
     cagctgctgt tccacttccc cacacagaac cgtctcggga ggaagaggtt ggagattttt    609000
     ctgatgtgtc ctcaatcctg ggagaaaagt tttcaatagc aataggaatc tctcagttgt    609060
     atgtccgctg agaatttaaa caataacttc tttataatac taaattttaa acaatccaat    609120
     gaatatatct aatttaattt tgtattagct tatgtgacat ttgctggcct ctcccccaga    609180
     taaggtcata ttctcttcct gtttgttcct gcaagtcact gaatttcaca agatgtaagg    609240
     tctcttgatt atactataac atcaataatc tggtgtacta aagaaaatgt gctaatttgc    609300
     agcttactaa aattagttgt tttagtaaaa ataatatatt tgttatagca tgcctacatc    609360
     tccaggctga gtagaagctt tatttgtaat actcagaaac tgcaaataac acacatatta    609420
     aggagctttc taaataaata ctatgggtga attctttaac tggaatactg ttcctcatta    609480
     gaatcaacac attctttata cacaaccaaa ttactgactc ccaagtaatt atactgagca    609540
     agaagccaca caaatgaaat tgaaaatata agatttcact ttaacaaaat cttgaaaaat    609600
     agatctgaat taacaaagat gagaggttga ctcagtatgg tgagagggaa ataatgggga    609660
     ggcaggaatg agaggaacac agggaggctt ttaactgtaa tcatcgttat ctcgattgtt    609720
     attttggata cacaggtgag cacacgtgaa attatgtttt ttttccttcg gagacatggt    609780
     tttgctttgt cgtccaggct ggagctccgt ggtgagatca tagctctctg cagcctcaga    609840
     ccctgggtct ccagcaatcc tcctccctct gccctatgta gctgggacta caggggtgca    609900
     ccaccatgct cggcttcagt gcgtattaaa ccgcacactt caattatgca gtatttatta    609960
     tatagcaata atgcctcaat aagggtatta caaataagtg gataatttgt taaagattga    610020
     tggaaagata gataccaaca tgagaaatgt atgacactca agaaaataaa actgtaggaa    610080
     acttgctttt ctttatattt gttaggtaat caccacagtg tgtacacatc acaccatgtt    610140
     cccattacag agaaaaggtt ctgcgaacct cacgagctgt gacccctgtg tgctgggctt    610200
     ggttcaggga gaagtcaggt ccagtggtga gaagcacagg cccagatgcc caggctcact    610260
     ctgaccaaat gtgagcactg gggacattgt aaaacccacc tgtgcttttg ctgataattt    610320
     ttcatcttta acatggaaat aatattgata ctatatacca tggtttctct gcgtatgtaa    610380
     aaataaaaga tgattggtgc taactttaaa aatatgcagt ttatgtagat ctatggtacc    610440
     tcaataaaac tgttttaaaa taaaaattac aaaattataa gatttttagg ttttaaggtt    610500
     taagtttatc acaaaacaaa ctgacaatag gaaagcacaa tttcccaatg ctttcaatat    610560
     cacagatctc cccgaggaca ttctgacatg ctctgagccc cactatctcc aaaggcctct    610620
     caccccagag cttactatat agtaggagat atgcaaatag agccctccgt ctgctgatga    610680
     aaaccagccc agccctgacc ctgcagctct gagagaggag cccagccctg ggattttcag    610740
     gtgttttcat ttggtgatca ggactgaaca gagagaactc accatggagt ttgggctgag    610800
     ctggcttttt cttgtggcta ttttaaaagg taattcatgg agaaatagaa aaattgagtg    610860
     tgaatggata agagtgagag aaacagtgga tacgtgtggc agtttctgac cagggtttct    610920
     ttttgtttgc aggtgtccag tgtgaggtgc agctggtgga gtctggggga ggcttggtac    610980
     agcctggggg gtccctgaga ctctcctgtg cagcctctgg attcaccttt agcagctatg    611040
     ccatgagctg ggtccgccag gctccaggga aggggctgga gtgggtctca gctattagtg    611100
     gtagtggtgg tagcacatac tacgcagact ccgtgaaggg ccggttcacc atctccagag    611160
     acaattccaa gaacacgctg tatctgcaaa tgaacagcct gagagccgag gacacggccg    611220
     tatattactg tgcgaaagac acagtgaggg gaagtcattg tgagcccaga cacaaacctc    611280
     cctgcaggaa cgatgggggg gaaatcagcg gcagggggcg ctcaggaccc gctgatcaga    611340
     gtcatcccca gaggcaggtg cagatggagg ctgtttcctg tcagggtgtg ggacttcatc    611400
     ttcttctgac agtttctcta gtgaacctct ctaacctcag aattctgtgc ttactaatgt    611460
     catctctacg tattttttaa aagatcattt taatatgagc acctattctc acacgcacca    611520
     aatgcagatt gacgcttaca gagatgaaaa gttctcaacc atggtcacca ggatgagagt    611580
     cctgaggaaa ctcaggggtg cctggtgact cttccgcaat cagtcctggg acagaaacct    611640
     cagggacagc ctttatgagt tttgatcaca tctaacagag aggatgggcc agggccagag    611700
     tcatgtagaa cctcacaggt tccacgtctg acccttctcc tgacactaaa gccaatcagc    611760
     atcagcactg acctggtgct ccttttgctc ccaatccatg ttctttcttt ggagagtttc    611820
     ttctcccttt tttatttgct tttcctgttt cctgaaaaag aaacagatgg tccccttggt    611880
     ccacattcca gggctcaagg cattttcttg gagctcaggt ggggctcagg ctgtggctcc    611940
     tgcagccacg tgggagaggc tgatgggact tccttctctc cccattttct caggaccctc    612000
     cactgtgttg tgtgtaggct catctgggaa tgcagttgac cattaatact gaaggggaca    612060
     aactcatttg atcaaaatgg gatgtggatg tggagttaac cctgttccgt gcacagtgtc    612120
     agagtcgtct gcttcagaag tagtgttaga aagagctggt gaaatttatc agaatcaaga    612180
     tggagccact tgtgttaaag ccctaatgaa tgaagctggg gaaggccatg aaggagggtt    612240
     ctaacacaca tattcctgat aataagaact accataaata gactctgcac aaccataacc    612300
     ttgcacagaa gccaccacaa cattaaaaga attacttctc ccagtgtatc tgcccagcaa    612360
     ctccctgttc aactttacac cgatgtgccc ctgttattga tccttctagc ccaggacaat    612420
     tgtcttaaac agctgatgtg accctcctca tttttccttt attctttatc ttcctatacc    612480
     aacctgaatg cacccataca tttcaattgc aatacacttc ctagaataca tattattata    612540
     ttttagagtc tctctctctc tgttattcag ggttgacaag ctacagatgg acatgccact    612600
     ttcttttttt atgacttttt ttaaaaattt tattattctt gtactttaag ttttagggta    612660
     cctgtgaaca atgcaggttt gttacatatg tatacatgtg ccatgttgtt gtgctccacc    612720
     cattaactcg tcatttagca ttaggtatac ctcctaatgc tctccctccc cactaccccc    612780
     accccacaac agtccctgga gtgtgatgtt cctcttcctg tgtccatgtg ttctcattgt    612840
     tcaattccca cctatgagtg aggaaaaaaa tgatgagttc atgtcctttg tagggacacg    612900
     gaagaaactg gaaaccatcc ttctatcatt ctcagcaaac tatcgcaagg acaaaagaca    612960
     tgccactttt ctgtgagatg tggaggatga cagtttttgg agatgtctgg gaatacccag    613020
     tgtccagaag atcatccatc aataagtgca ggctggaggt cccagagaga ggcaaagctg    613080
     ctgaaacacc atggaatttc attttctcca gttctgctct gatggaatca ggcccacctg    613140
     ttttgcttgt cttaagtaaa ttcacagcac cacatatatt agtgttgcag tgaataacta    613200
     caaagtctat cctaaccaag tgtaccatca aatttaccat tacccagagg gaagaatctt    613260
     taacatgaga tctatgttca taatttttta aggtgaaaaa gtgatcacta tccaatcagg    613320
     caaatatgat tttgctaatg agtggtagat gttgcatgta tttttggatg tcatcccttt    613380
     ttcagatgca tggcttgcaa ttatattttc ccattctgta ggttggaatt atttccacac    613440
     tttgtagaat ctttttgttg tgatgtagtt ccacctgttt aattctgctt ttgttgtctg    613500
     tgcctttaat gtgaaatata ggaaatcatt gttaatatat tattttctgg gtcggggaaa    613560
     ttttacaatt gcaggccata tgtttaaata tctggcccat tcagagtgat ttttgtgttt    613620
     attctaaccc aaaattctta actctttgca tgtgtacatc cagtttttct aacacattca    613680
     tagggagaga cttggtttat ctgttttata ttgtgggttc ttatgctgaa aatcagtttg    613740
     ccattgatac atcagtttat ttctgagctc tttctacata tttatgccaa ctccatcctg    613800
     tttttattat tctacttttg ttattacttt tgaaatctgg aagtaagatg taagtttatt    613860
     tttgcctttt tacagatgct atagcaaaat attttaacct ttcactcttc catataaaag    613920
     tatggcttta aataatgact tttattatat acaggttata ttcttgtatt cctaactttt    613980
     agaggtttta ttatgagatc ttgaattttt taaatgttat tttctatgtc tggtgagaag    614040
     ttaactgatg cttttccttt attctcttaa tttaccacat cagatttatt gatttgaaga    614100
     tattgattca tcagcgcatc tcaaaaataa tttggagctt gtcatggtgt acaatccttt    614160
     taaagtctta tagaacttag ttgactatga attgggagta gcttgtttac atatactaag    614220
     aacatttgtc agaagtttgg ttttcttctt ttgccttgtc tattactggt aatacagtga    614280
     ttggagcctc ttagaaagag attagaaggt ggggacctca cggatttttt aaaaagttag    614340
     agaacggttg tcatcacttt tttaaacgct gagagtcatt tgttatctaa tatataaccc    614400
     atcatgagga atatctctag tgtgcttggg aagcatgcat attacgctgc tgttggttgg    614460
     aatgttccgt agatgtctgt tagggcagtt tgttcgagag tgcttaagtc cacgggcttc    614520
     ctaagaattt ttctgtggat gctctgtaca ttattgaagg cgaggttttg aagtctccta    614580
     atttttttat agttttctat ttctcctttt atacatctta aagtttcata aatgtagtta    614640
     tatttctagt ttttcaaata tgtaaaagag aaataaaaat atttgcttag taattttaat    614700
     gagaaaatca cataacaaga aattatattt tccaaatgct gccatcaaca ctaaactcct    614760
     ccaggagtct cacatctgcc ctgggctctg ctctctcctc aggcatccca cctcagcatt    614820
     tgctgcagag gaggggacgt gaaaataggg ccctcccttt cctgatggaa accatcgcag    614880
     ccctgagcct acagctctgg gagaagagcc ccagtccagc attaccagga gttttgattt    614940
     gacaatcagc tctgaacaca catggctcac catagagtta gggctgagct gggttttctt    615000
     gttgctattt taaaaggcaa ataatggaga actagagata ttaagcatga gtggatgtga    615060
     gtgaaaatac agtgattatg tgtggcaggt tcggaccaaa atgtccctat gtttgtaggt    615120
     gtctagtgtg aggtgcaggt ggtggagtgt aggggagaga caagaggcag aaaagtgtgc    615180
     aggaggccgg gtgaggctgt agacactgtc agcccactat gccaatccca ccacgagtgc    615240
     tggagaaggt gggagtctga tgaagcttac taacaaccct gtggtccaag ctaattccat    615300
     caaggccatt ggttcttccc agagcatagc tgtccatcgg gaatctccca tgtgcccagc    615360
     agcagccacg cgttagcaac ttcactgtgc acagtcattg tttgggagga gctccaggat    615420
     gtgtctcttt gtcaaaaact gggtgatggt gtcagagagc tgtgccgggt gcctggtcca    615480
     cgtgctccct gacattggag gtataggagg tgcgttctca gggtggaaac accttttatg    615540
     gaaatgtaca taataaatca cgattttcct tggttctctt agacgacatg gagaagcaag    615600
     catgcaccct gcaaacaatt gtaatttcct gctttacccc aagttcatga tttcttgtaa    615660
     actttgattt ctgtccaagt acagagatac tgattagagt gagtttggtt ctttccacac    615720
     actaaccctc acctccccca gatagagcac attgtcctca ctctgagtct gagggaggag    615780
     ctgttcctgt acaactcagg gactgcagag acccccgtgt gcagctttgc agagtcagat    615840
     ctttcctcat gtgaggcgac ctccactgcc cgtgattgct gctcaggtct aattgtggat    615900
     tcgggattat gacacccttt gggctatcac aggtcaatct tattctgaaa atcaccgtaa    615960
     ttatagacag aggtaataat ccagtaccca ttcttctgtc cgcagtttgt ctttcttatt    616020
     tggttgaaag tttgaagaaa aaatgtgacc atatattcat ctaattctaa cctctgaatc    616080
     tgctatttgc tctcggggac ctacaaattg caaaacagtg agttcttcat tttcatcaaa    616140
     atgtgcaaat ttgtgaattt caacatgttg ttcagaacct gtgcatgccg acaactgtga    616200
     ttctcggtgc actcctggcc tggtgaagcc ctcacagatc ctccccctgg cctgtggcat    616260
     ctctgcttct ccattacaac cagtgcttcc tgctggagct ggatccgtca ccccccgggg    616320
     aagggactgg agcgaatcag gtgcacaggt catgagggag tgcacaatcc aacccactcc    616380
     tcaagagtcc agtcaccatc tccagatcca catccaaaaa acagtttctc ctacagctga    616440
     gctaccttaa caaggagtac acaaccatga tttttataca aaagacacag cgaggggaag    616500
     ccattgtgcg ctcagaacac tctacaaatt ttcctcccta gtgttttacc aaaactggta    616560
     tatatttcag atactgaaat atttacaacc tacgttatta tgctaagaat tctaagttag    616620
     aagttagaat gataattgtc tctttttttt cctttttttt tttttttttt ttgatactga    616680
     gtctcactgt tgttgcctgg gctggagttc aatggtgcaa tctcggctca ctgcaacctc    616740
     tgcatcccag gttgcagcaa ttctcctgcc tcaacccccc aagtagttga gattacaggc    616800
     gcctgccacg atgcccggct aatttctgta tttttaatag agacggggtt tcaccatgtt    616860
     ggcctggctg gtctcaaact cctgacctca ggtgatccaa ccgcctcagc ctcccaaagt    616920
     gctggaatta caggcaggag ccactgcacc tggccaatta tcctcttttc atagatggat    616980
     aaactaacct aggcctttga aaataaaccc ttatctgaga gtgaaaagat aagccataga    617040
     tttggagagt ttgcttgcaa atcaaatatt tggaaaagga cttttattac aaatatacaa    617100
     attaccttac aattgaacaa caaaaccaca caatttaatt ttaaaatggg caaagacctg    617160
     aatagaaagt gcatctaaca ataagtataa aaagtgacca actggatttt cattaggtaa    617220
     atgtatgttt aatattgaaa aaatcctagt aacctaatag aatgggtaaa atacacaata    617280
     tagacaatac caaatgttga cgaggaagtt gaaaaacagg aattttcacc cattgctcct    617340
     gagaatgctg aattggcaca tagacaacta cagttaataa tttattgtgt atttcaaatt    617400
     agctagaaga taacatttag aatgttctta ccacaaagaa gtagtcaatg attgagatga    617460
     aggttgaagg ttgtcccagt tatccagata tgcacataat tttctttttt tcgaaatatc    617520
     acatgtacca cataaaggtt ttgaactatt atgcatctat ttaaattaca cattaaaaaa    617580
     caccagtgga agggaatcca gctaattgca gattgccatt ggatgggacg ttctgtataa    617640
     tctgtaaact aatcagaatc tgtgtttttc taaaggacat taggaatcgt tatactagtt    617700
     tctaaagata agccttgcaa gtttaattat tttcctaatg ggtggcgatt ttgaacattg    617760
     aagaaatgtt agtatttgtt tctggttaag ttttcttctt ctagtctcag gagaaaaggt    617820
     ttgcagggag gaatctgaga ctatctttca agtctcaact tcaatgtgat tacctcagta    617880
     aatattttct gatacccctg agtagggtaa gtctcatata tgtccaataa actaaaagta    617940
     gaattttaag cgcccaagct gactgaatgg aaccttcctc ttcaccaagg agttccagag    618000
     acttgaaaac ctagttcagg ccacattaga aacagatcag aaatgcctca ttataccctc    618060
     ctcactttgg aattcaagca caactgacca gcattttcat gaaaacagag gactcaaggc    618120
     tcagaaaacg gattccgtgt agcaataaga taccaaattc taacctgact cgagaatagc    618180
     gtcatgtgac acagagcagg ccttgaaagg aatcgaaggc ttttacccta aaatatattt    618240
     ctttgacata tttttaaatc gccctgcaca gttatccttt gtgagagaga tttacattct    618300
     gtagagaatc tccttccctt tccaggtctt tcgttatact gaggagatta gctggaagtc    618360
     tagcatctct taaaggtctt agtagaaaaa catttgccat ctatttcctc taagcgtggc    618420
     cacataagag acttcatctg cataatatga attttgttct ccaaaacctg ttatcttaac    618480
     ccagacactt tttactactg atttctggtc tttagataaa aacttaagtc tttcaatgaa    618540
     ttgccaattg aaaaatcatt taattcacct gtaagcggta attatctccc cacactgcct    618600
     cacttctagc tgttttgtct ttatgaaaca aatcaacaga tatctcacat gtatcaattg    618660
     atgttattaa atttaacctg caatcaaacc actttgggca catgttatca ggatcctctg    618720
     gggctgcgtc acagggcatg gaccccacat ttggctcaga ataaatctct ctaagtattt    618780
     tccaaagttg gctttttatc gatagaatct actctgtggt ctctgttaca ttcaaaagca    618840
     tgatagtaat aaagtattag ttattaaaaa taagcacatg ttggcagttt cttacgaagt    618900
     tacgcataga ctaactatgg gatcaagtgt aaattttcaa agtatttatg cagttatttc    618960
     aaaatgtatg ttccacagat acttctatca gatcctttat attagataat tatttgtttt    619020
     atgacttttt ttgtagatga tcatgccgta tcttttctag gcctttattg tagaaaattg    619080
     tgtgaaacca gtcagggcta catttgaaat tcattgtcac agtggctgcc tgaattgcag    619140
     tgggtttcct ctgcagccac cagacaccca gattcctcca gtgacacctt aattttgttt    619200
     cctgcttggc attgggcctt aaattgtgtg ttccagagag actctgtctt tcatctcccc    619260
     caggtcatcc cagagccaca tggacctgat gattgtcaat gtggtgggaa gggttgcact    619320
     gaacgggcac tctctgtcct tctgagggag ccacactcca ggcagacatg gcagtcctgg    619380
     ctcaggacgt gtccttccaa gtatccctgc ccctcctgca ggtgtgatgc tgatccatgc    619440
     atcttcctcc cattcctggg tagagggtct ccttgttctt tccccaatct tctcccagca    619500
     gccactatgt cttcccactg atgtcttcag cttcctcttt tctgcccttc ccagcagatg    619560
     agtctgttat tcctgaggga agagagagga ggtgggagat gaggctgtta ttcctgaaag    619620
     aatggacaga gctttggagc ttttctttct tctaccccag tttctacgag ttcctccagt    619680
     gcctgtaaca cagagctgtc agtggctttg cccctgcatg ttaattcttt ggtcatgtag    619740
     tggtggtgag ggagctgggt ctggatgcat ttcagccaca gctgctgttc cgtttcccac    619800
     acagaaccat ctcgggagga agagggtgga gatttttgtg atgtgtcctc aatcttggga    619860
     ggaaaggttt caatagcaat aggagtctct cagttttaag tcctgtggaa atttaaataa    619920
     taacttcttc attatactta attttaagca atcgggtaaa tatatctaat ttaatcttgc    619980
     attaacttat atgacatttg ctggcctctg cctcagataa aattatattc ttttcctgtt    620040
     tatccttaca agtcactgat ttgattttca caagaggtaa gttctctcat ttgccctata    620100
     catcaataaa ctcatgtatt aaataaaatg tgctaatttg tagctcagta aggctagttg    620160
     ttgtaaaaat aatacttttt tattggatga ctacatctcc aggcggagta gaagatacat    620220
     ttgtaatact cagaaactgg aaataacaca aatgttaagg agatttataa ataaatgata    620280
     ggggtgtact cattaactgg aatactgttc ctcgttagaa tcaacacctt tatacacaat    620340
     caaattactg actcacaagt aattattctg agcaagaagc cacacaaatg aaatggaaac    620400
     taaaagattt cactttaaca aaatattgaa aaatagatct gaattaacaa agatgagagg    620460
     ttgactgagt atagtgagag agaaatgacg ggaggcagga atgagaggaa cacagggaag    620520
     cagggaagct tttaactgta atcatcatta tcttgattgt tattttggat acacaggtga    620580
     gcacatgtga aattacattt tttttctttg gagacatggt cttgctttgt catccaggct    620640
     ggagctcagt ggtgagatca cagctctctg cagcctcaga ccctgggtct ccaatcctcc    620700
     tccctctgcc ctatgtagct gggactacag ttgtgcacca ccatgctcgg cttcagtgtg    620760
     tattcaactg cacactttaa ttatgcaata tttattatat agcaataatg cctcaataag    620820
     ggtattgcaa ataagtgaat atataatttg ttagataaag actgatggaa agatagacac    620880
     gaacatgaga gatgtatgac acttaagaaa ataaaactgt gggaaacatg ctgttcttta    620940
     caattgttag gtaatcacaa aagagcatac acatcacacc atgttcccat tacagagaaa    621000
     tggttctgta aacctcacta ggtgcaaccc ctgtgtgctg ggcttggttc agggagaagt    621060
     caggtccagt ggtgagaagc acaggcccag atgcccaggt cactctgacc aaatgtgagc    621120
     tctggggaca ctgtacaacc catctgtgct tctgctggta atttctcatt tgtaacatgg    621180
     aaataacatt gatactacat accatggttt ctctgcatgt gtaaaaataa aagattattg    621240
     gtgtgaactt caaaaatatg cagtttatgt agatctattg tacctcaata aaacttttaa    621300
     aataaaaatt acaaaattat aagatttata ggttttaagg gtttaccaca gaacaaactt    621360
     acaataagta accacaattc ccaaatgcta tcaatatcac aaatctcccc caggacgctc    621420
     tcacatgctg tgagccccac tctctcctca ggcctctcac cccagagctt actatatagt    621480
     aggagacatg caaatagagc cctccctctg ctgatgaaaa ccagcccagc cctgaccctg    621540
     cagctctggg agaggagccc cagccctggg attttcagat gttttcattt ggtgatcagg    621600
     actgaacaca gaggactcac catggagtca tggctgagct gggtttttct tgccgctatt    621660
     ttaaaaggta attcattgag aactattgaa attgagtgtg agcggataag agtgagagaa    621720
     acagtggata cgtgtggcag tttctgacca gggtttcttt ttgtttgcag gtgtccagtg    621780
     tgaggtgcag ctggtggagt ctgggggagg cttggtacag cctggggggt ccctgagact    621840
     ctcctgtgca gcctctggat tcaccttcag ttactactac atgagcgggg tccgccaggc    621900
     tcccgggaag gggctggaat gggtaggttt cattagaaac aaagctaatg gtgggacaac    621960
     agaatagacc acgtctgtga aaggcagatt cacaatctca agagatgatt ccaaaagcat    622020
     cacctatctg caaatgaaga gcctgaaaac cgaggacacg gccgtgtatt actgttccag    622080
     agacacagtg aggggaggtc agtgtgagcc cggacacaaa cctccctgca gggccgcgcg    622140
     gggccaccag ggggcgctag ggacccactg agggcgggac aggagcaggt gccgggagag    622200
     gtttcctttc tcctcagctg gaaaagtcag gtttgtgttt gccggactct ggagctttct    622260
     aggctgtgac gttttattac ttgtgtttat tatgaattta ttatcgttag tatttaaatt    622320
     ttagtaattt aaaaattata tatatatgtt tacactttaa tgaaataagc attcctattt    622380
     gcaccgattc ttccagagtt ttattaacat ttgttgacgt cagcagctac gaagctatag    622440
     ggacataaat ttataaccat agaaagatgt ataggccagg cgcggtggct cacgcatgta    622500
     atcccagcac gttggaagcc ataggtgggc tgatcacttg aggccaggag ttcgagacca    622560
     gcctgggcaa aatagtgaaa ctccatctct actaaaaatg caaaaatcag ctgtgcgtat    622620
     tggcatgtgc ctttaatccc agctactcgg taggctgaag caggagaatc acttgaacac    622680
     ggaaggcaga gattgcagtg agctaaatgg cgccactgca ctccagcctg ggtgacagag    622740
     caagattcga tctcaaaaaa acaacaaaaa acaaaaacaa aacaaagaaa gaaaaaatat    622800
     ataaatacaa agacatatgc atatatgtgt aggaattcat aataaacatt acaataaagt    622860
     aattctaaaa atatgtccta aagaatcaaa cttaatgatg agctaaatgt aaattatttg    622920
     agtatttcac aattgatttt ggttattttt aattgtttac atcaattgat ttatatttgt    622980
     tcatttaact actgttaaat aatggttatt tcaaatgtgg ttgtcacaat aaataataag    623040
     aatttgaatt gataaatgat aattatctta atcatttctt aattatctta gttatcccat    623100
     atggtattca caaatcataa cattgcttct taccttgtaa atataaacaa ccataattcg    623160
     tgaatttaaa ataaaatttt ttattttaat tttttaaaat ttatcccaga tcattatctt    623220
     tttcttcagt tccagctctc atttgatggt ctcaagggcc catctgcacc cctgtctcct    623280
     gaaggcttct gggagtggct gtgggacagg cagaagcagg agcccatgtg agtccacacg    623340
     acctggagcc tccctctcct tggattaggc catctcctcg ggatcacagg gctcttcatt    623400
     atcctcaccc cgctgttgta ccaaacaagc aacatcacac ttcaattcat cacgctttgc    623460
     tttaattttc taaatcatcg tgaaggtgat aatgttaaca gtaaatgtat cactaacaaa    623520
     taggataagc ccttttccat gggacagggt ttcttacaag gacttgacat gtattatgta    623580
     ttttccattt atttatagat gagatactaa tatctccata ttggagacaa ttcctttaag    623640
     tcatttctta aaattaattt ttcctaggac tccaaacaaa tccatgatat agttgaaaat    623700
     ttcaggtgta agagttgggc caacattgag actatattaa catttataac atgtgcttgc    623760
     aaatattgtt atttttctgt cggctgtccc cagttatggt ttgatacaaa atgttagtaa    623820
     tttatcctaa taaaacagtt tacaatatta tatcaatagt ttattaactt tgtataacta    623880
     ataatataaa atgttcaaca tattttagcc atattttact tatttgtgaa atgtgtgctt    623940
     attagttttt cgttttaaga tgtcaccttt atctttttta tttctgggat tttattctag    624000
     tagatataac taaatgtctt taaataatta ttgtaataat cacatttata ttttctattt    624060
     gaattttcat atgcatgtta ttaagatttt aatttcaata aaaatatttt cctccaattg    624120
     tctattttct gtttttatga gataacatat acaaaaatgc atagctcttc aatgtacagt    624180
     ttgagggttt ctggcaaatg tgcacgtttg tctccagcac ctaagttagg gtgaggagca    624240
     ggtccatctc cacaagtgtc ctctcagtgt ttccagtcag ctctcacata aggatttttt    624300
     tttaatttca acttttaatt tagatacaga ggggacgtgt gtggacttgt cacacgggat    624360
     tattgagtga tgctgaggtt tggaatccag attccatcac cccctccctc cactctccag    624420
     cagtccacgg tgtctattgt tcttctattt atgtccatgt gtgctcaatg ctgagctccc    624480
     acataggaga atatgtggta ttcggttttc tgttcctgca tttatttgtt tatgattaag    624540
     accaacagct ccattaattt tgcggcaagg gacatgattt cattcttttt catggctgtg    624600
     tagtattata tagtgtagat gtaccatatt ttgtatattc agtctaccag tgatgtgcat    624660
     ctgggttaaa ctacgtcttt gccactgtga ctagcacagc catgaatacg catgtgtagg    624720
     tgtctctttg gtagaattat ttatttactt tttagtgtat accctgtagt ggaattgctg    624780
     ggtaaaatga tatctctgtt ttaagttctt tgagaaatct ccagtctgct ttccaaagtg    624840
     gcaagactaa tttatattct catcaacagt gtataagtgt tctcttttct ccaaagtccc    624900
     acaagcatcc tttgtttttc gacttttttg tgataccatt ctgagtggtg tgttgctgct    624960
     cacctacaat catctcatat ttgataaggc tgatgaaaag aagcaatgag gaaaggactc    625020
     cctgttcaat aaatgatact gggacaacgg gctagccata tgatgaagat tgaagctgac    625080
     cctgtacttt caacatatat aaaattaact caaaattgat taaagcctta aatgtaggac    625140
     ctcaaaccat aaaactcctt gaagacaccc ttgaaaacac tcttatcgac actagttttg    625200
     acaaaaaatt tttggctaag tatccaaggc cagttgcaac aaaaagaaaa atagacaagt    625260
     gggacctaat tagttaagga gtttctgcac agtaagacaa acaaacaaac aaacaacaaa    625320
     caaaactatc agcagtgtaa gcagacaatc tacagaatgt gagaagatat tcacaagcat    625380
     tgcatcccac aaagccctag tatccagagt ctgtagataa acaaatcaaa aagaaccaaa    625440
     aaaaaaaaaa ccctttaaaa attggctgat atggtttgct gtgtccccac ccaaatttca    625500
     acttgaattg tatctcccag aattcccatg tgttgtgaga gggacccagg gggaggtaat    625560
     tgaatcatgg gggctggtat ttcccgtgct attctcttga tagtgaataa gtctcatgag    625620
     atctcatggg cttatcaggg atttccgctt ttgcttcttc ctcattttct cttgccaccc    625680
     accaggtaag gagtgccttt ttcctcccgc cgtgtttctg aggcctccca agccatgtgg    625740
     aatgtaagtc caatcaaact tctttttcct cccagttttg gatatgtctc tatcagcagt    625800
     ttcaaaatgg actagtacat aggcaaacga cacagacact tctcaaaaga atacatgcaa    625860
     gtgcccagca aatatattgt aaaaatgttt aacatcacta atcatcagag aaatgcaagt    625920
     agaaaaatgt tctgatttct gtcactatag gaggtccctt tttcttgttt agaacttcat    625980
     atacatggaa taaaatatta tagatcattt ttgtaagggg caacttttac tatttgtgag    626040
     gttcattcat gtaatagcat ttatcaaggt tttgtcatca tatgtataaa tatgtatgta    626100
     ctcatacaca tatagatatt tcatatctaa atcaattaca ttaataaatg acagattatt    626160
     aaataaataa atctgtttat taagtaaata agtgacaata tgtatatcta ttttcctgtt    626220
     gatgggattt aaatttgttt ccaaaataaa catcataaac aaaactatta taagtatctt    626280
     tgtacaagtt cttctgttta tattcacaca ttttattgat aaaatatgta gatatatagg    626340
     tatgcattat aacttttcag ctttatggag ctatcactga caaataaaat tgtatatatt    626400
     tagggtacac cacttgatgt attgatgtac ctggggagat gctcatcatg atcaaggtaa    626460
     tcggcatgcc tgtcctctca gagagttatc attttatgtc tttaatttac tgtgtgtgtg    626520
     tgataaaaac acctaatatc tactcttctg gcaaaagata cctttataat acaatattca    626580
     ttagtatagt cacattgctt tacgtttcat ctccagaact atttcaacct gtgttttagt    626640
     ccattctcac cctaatgtaa ggaactacct gagactggga aatttacgaa gaaaagtggt    626700
     ttagttgact cgcggttctg caggcttaac aggaagcatt attgggaggc atcaggaaac    626760
     ttacagacat catggaaagt gaaggggaag caaggacctt cttcacatgc tggcagggga    626820
     gagagagaga aagagcaagg cgagatgagc cacactttta aaccatgaga ccttgtgaga    626880
     tcttgcaaga atcacaaagg ggaaggtcgc ccccattatt caatctctcc ccatcagacc    626940
     cctcctacaa cacttgggga ttaaaattta acatgagatt tgggtgaaaa tgcagagaca    627000
     aaccatatca ttccacccct ggtccatgac agatctcatg tccttctcac attgcaaaat    627060
     atatttatcc cttctcaacg gtctcccaat cttaactcat ttcagcataa tcataaaaat    627120
     ctatagtcta aagtctcctc tgacaaggtc agtttcttcc ccctataaat ctgtaaaatt    627180
     aaaaacaagt tagttatttc caagatacaa ctggggtgca tgtactaggt aaatgctccc    627240
     aatccaaata ggagaaattg gccacagaaa atggattaca ggtcccatgc aagtccaaac    627300
     accagcaggg cagtcattaa atgttaaagc tccaaagtaa tttttctttt accccatgtc    627360
     tcacatgcag gacacactga tacaagagat ggtctcccaa ggccttggga agctccaccc    627420
     ttgtggctct gtagggtcca gcgcccatgg ctgctttcat aagctggcat tgagttcctg    627480
     tggcttttct aggcacaccg tgcaagctgt tggtggttct accattctgg ggtctagagg    627540
     atggtggccc tcttctcaca gatccactag acagtgcctc aatggggact ctgtgtgggg    627600
     gatccaacat cacattttcc ttctacactg gcctagtaga ggtactccat tagggctcag    627660
     ccccttcatc agacttctgc ctgaatatcc aggcattttc atatatcatc tgaaatctag    627720
     gaggaggttc ccaaacctca gttcttgcct tatgtgcacc aacagactca acaccatgtg    627780
     gaagcaacca aagcttaggg cttgcaccct ctgaagcaat ggtctgagct ggaacttggt    627840
     cccttctagc tatggctggc acgagacaga caggggtgta aggtgccatg tctcaaggct    627900
     gtacagagca gtgaggtact gggcctggac catgaaatca tctttctctc ctgggcttct    627960
     gggcctgtga tgggaggggc tgccacaaag atctctgaaa tgccctggga acattttccc    628020
     cattgggctc ctcattactt attcaaattt ctgcagccag cttgaatttc tccccaggac    628080
     atgggttctt cttgtctacc agatggtcag gctgcaattt ttttccaacc ttttatgctc    628140
     tgcttccctt ttaaatatga gtttcaattt cagaccatct tttcatgaat gcataggact    628200
     tatgttttca gaaacagcca gggaaaatct tgaatgcttt gctggttaga aatttattct    628260
     gccagatacc ctaaatcatc tctccctagt tcaacatagc acagatctct agggcagggg    628320
     caaaatgccg ctagtctttt tgctaaagca tagcaagtgt gagcttcact ccaattcaca    628380
     agaagttctt catcttcatc tgagaccacc tcagcctgga ctttattgtc catatcacta    628440
     tcagcacttt ggtcaaaacc agtcagtaag cctctaggaa atttcacttt tccacgactt    628500
     cctgtcttct tgtgagcctt ctaggctttc ccaacatctg tccattacaa agtcacttcc    628560
     ccattttcag gtatcgtaat agcagtaccg cactcctggt gcaaattttc tgtattaatc    628620
     cattttccca ctgctataaa gaattacttg agactgggta atttatgaag aaaagagggt    628680
     tagttggctt acagttctgc aggctaaaca ggaagtgtta ctgggaggca tcaggacaca    628740
     caatgatggc agaaggtgaa ggggaagcaa gaacattctt cacatggtgg caggagaaaa    628800
     acagcaagtg agcggggagg tgccacactt ttaaaccatc agatctcttg agaattttat    628860
     catgagaaca gcaaagagga agatcacctc atgattcaat cactttatat taggcccttt    628920
     cttcaacatg tagggattac aatttgacat gagatttgag ggggaacgca gggccaaaac    628980
     acatcaacct gcataaccga aatttttacc ctttgaccta catcgcccaa tattttcctc    629040
     ctctcagggc ctgggagcta ctattctact ctgcttccaa gagctgaaat attttacatt    629100
     ctacatataa atgagatcat gcagcatttg tctttctgtg tctggcttat tctaattagc    629160
     ataatgttct ctatgatgct gaaaatgtaa gaatttcctt cttttaaagg ctgaataata    629220
     ttcaatttta tgtatgtata tgccacattt tatctgttca ttcataaatg gacattgact    629280
     tattttccat atctaggcta ttatgaataa cctcacaatg agcatatttg atacactcac    629340
     ttcattttct ctagatgtac acttagaagt gggtataccc tatgttcaat tcattcagta    629400
     atcttaatgt tgtttttata atggctgtac taatttacat tttgttccaa accatacatg    629460
     gatacctttg taccaaattt tcaggtctac gtttaagtgt taaatccatt tcttgttgat    629520
     ttctgtgtat tttgtgaggg aaggtccttt tttttttttc cttagagtta gaatctcgct    629580
     ctgtcaccca ggctggagtg caatggcatg atctcggggc actgcaacct ctgcctcccg    629640
     ggtttaagcg attcttctgc ctcaggctcc cgagtagctg ggattacagg tgtgcatcag    629700
     cacgcctggc taatttttgt atttttagta gagacagggt ttcaccatat tagccagctg    629760
     gtctcaaact cttgacctcg tgatccacct gcctcaggct cccaaagtgt tgggattaca    629820
     ggtgtgagcc atcacgccca accagtccat ttaatttctt ctacatatgg atacccagtt    629880
     ttcacaccac ttgctgaaga gactgtcctt tgtctattgt gtgttcttgg caacttgtca    629940
     aagatcagtt tattgagaag aagtgggttg attatcaggt gtctataatg ttccattaga    630000
     gtatatgtct gtttaaatgt cagcattata ctgttttgat ttagatagat ttgattttga    630060
     aattatagaa tatgatatat tcagctttgt tatactttcc ccaaaattac tttggctatt    630120
     ggaaggcttt tgtatttgta tataaattgg agcattttaa aaatattttt gtaaaaacat    630180
     gccatggaga ttatttactt tattgacaaa taatagatat acctaattta tgggagcatg    630240
     caatatattt atacatttac aaatatgtga agatcagatc aggattggta tatctatcac    630300
     gttaaacagg tatattttct ttatgctagg cacattggaa tgactctctt ctagttattt    630360
     taacgtgtac attagattat ttttaactat agtcacctta ctgatctatc agacatttag    630420
     gttttatttc tcctatataa ctatgtatct gaattcagta atcaacctct ctttatcccc    630480
     ttctctcttg tatccaccca ggcttctggt aaccaacaat ctactcttcg tcttcaggaa    630540
     atccaatttt ttagttttga cataaaagta agaagatgcc atatttgtct ttctttgctt    630600
     ggcttattta acttaacata acggcttcca gttccattca tattgttgca aatgacaaga    630660
     tatcgtattt tatggctgaa taatattcta ttgcgtacat agattacatt ttctctatcc    630720
     atttgcccac ctgcagacac tgaggtgctt tcatatctgg gctattgtga gtagagctgc    630780
     aaagaacatg gagtgcacat gcctttatga ggcggtgatt ttatcttctt cagaacatac    630840
     ccagaagagg atttgccacg tcatcaggta tttctatttt taatttattt tggagtcttc    630900
     atattacttt gtacaatagt ggaaatgcaa taggaataac cattataaaa agcagtttca    630960
     gttttgaggt atgatccaaa aacaaataat gtttgaccat gattctcact gggagtctct    631020
     aatgagcctg gtggaaatgc aggaagtttt ctaaccttgt taagaaaatt atgactttgc    631080
     acatttttct ttttaaggct ggtaattggc gtgattccag atgcatactg ttggcgggtt    631140
     tcagaatgat acagaaaatc attatgagaa ggcaagaaaa agaatgatag agagagagag    631200
     agggagagag agagagagtc aggccaacaa tgagaaaaaa taaatttcac aagaggagaa    631260
     agtgctggat atcagtgttg ggttttggtc atagacacat ctgtactgag taagaaacta    631320
     tgaagtcaag ggaggagtct agaatttgtt caagtgaagc atcagcatat tctctgaggc    631380
     acatattgtc accccatcat aagggtgatg aatttttgaa tgccatgaaa tgtgagttca    631440
     cagtaagtaa tcatgtttcc atgaaattta gttaaaatgc caagggtgtg ttcagatcac    631500
     tcatgcacaa atacacaaaa tttgcctttg catttatagc tatctagaga aaaagtgcat    631560
     tgagatgtca gcaggttaca gagatctgtt taagtttggg atcccatggg agagtgcctc    631620
     ttagtgaagg ttggtctatt aattcagtaa gtaaaaattc tctctatggg agtagcttct    631680
     aattgtgtgg aaatttcaat tccttattga gtttttaaaa aatatctcag ggactgactc    631740
     catggagttt tactgctgta ttgataaaaa ttatcatcaa agaccaaagg tagataaaac    631800
     cacaaagatg gggagaaacc agagcagaaa agctgaaaat tctaaaaatc agagcacctc    631860
     ttctcctcca aaggaacaca gctcctcgcc agcaacagaa caaagctgga cagagaatga    631920
     ctttgacaaa ttgagagaag aaggcttcag acaattggca ataacaaact actctgagct    631980
     aaaggaggat gttcaaaccc attgcaaaga agctaacaac cttgaaaaaa gattagacaa    632040
     atagctaact aaaagaaaca gcatagagaa gaccttaaat aacctgatgg agcttaaaac    632100
     catggcacga gaaatacatg atacatgaac aagcttcagt agatgattag atcaactgga    632160
     agaaaggtta ccagtgattg aagatcaaat gaatgaaatg aagtgagaag agaagtttag    632220
     agaaaaaaga gtaaaaacaa atgaacaaag cctccaagaa atatgggagt atgtgaaaag    632280
     accaaatcta tgtctgattt gtgtacttga aagtgacggg gagaatagaa ccaagttgga    632340
     aaacactctt caggatatta tccaggataa cttccccaac ctagcaaggg aggccaacat    632400
     tcaaattcaa gaaatacaga gaacaccaca aagatactcc tcgagaagag caactacaag    632460
     acacataatt gtcagattca ccaaagttga aatgaaggaa aaaatgttaa gtgcaggcag    632520
     agagaaatgt cgggttaccc acaaagggaa gcccatcaga ctaaaagtgg atctctcagc    632580
     agaaactcta caaaccagaa gagagtgggg gtcaatattc aacattctta aagaaaagag    632640
     ttttcaaacc agaatttcat atccagccaa actaagcttc ataagtgaag gagaaataaa    632700
     atcctttaca gacaaacaaa tgctgataga ttttgtcacc accaggcctg ccttacaaga    632760
     gctcctgaag gaagcactaa acatggaaag gaacagccag taccagccac tgcaaaaaca    632820
     tgccaaattg taaagaccat cgatgctagg aagaaactgc atcaactaac gagcaaaata    632880
     accaactaac atcataatga caggatcaaa ttcacagata acaatattaa ccttaaatgt    632940
     aaatgggcta aatgctccaa ttaaaagaca cagactggca aattggataa agagtcaaga    633000
     cccatcagtg tgctgtattc aggagaccca tctgacatgc agagacacac ataggctcaa    633060
     aataaaggga tggaggaaga tctaccaagc aaatggaaag caaacaaaca aaaaaagcag    633120
     gggttgcaat tccagtctct gataaaacag actttaaacc aacaaatatc agaagagaca    633180
     aagaaggcca ttacataatg gtaaagggat caattcaaca agaagagcta ttttaaatac    633240
     atatgcaccc aatacaggag cacccagatt cataaagcaa gtccttagag acctacaaag    633300
     agacttagac tcccacacaa taataatggg agactttaac accgcactgt caacattaga    633360
     cagatcaacg agacagaaag ttaaaaagga tatccaggaa ttgaacgcag ctctgcacca    633420
     agaggaccta atagacatct acagaactct ccaccccaaa tcaacagaat atacattctt    633480
     ccaaacaccg cattgcactt attccaaaat tgaccacata gttggaagta aaacactcct    633540
     cagcaaatgt aaaagaacac aaattataac aaactgtctc tcagaccaca gtgcaatcaa    633600
     actagaactc aggattaaga aactcactca aaactgctca actacatgga aactgaacaa    633660
     tctgctcctg aatgactact gggtacataa tgaaatgaag gcagaaataa agatgttctt    633720
     tgaaaccaat gagaacaaag acacaacata ccagaatgtc tgggacatat ttaaagcaat    633780
     gtttaaaggg aaatgtatag cactaaatgc ccacaagaga aagcaggaaa tatctaaaat    633840
     tgaaactaac atcacaatta aaagagctag agaagcaagt gcaaacacat tcaaaagcta    633900
     gcagaacaca aggaattact aagatcagag cagaactgaa ggagatagag acacaaaaaa    633960
     cccttcaaaa aaatcaatga atccaggagc tggtctttgg aaaagatcaa caaaattgat    634020
     agaccactag caagactaat aaagaagaaa gagagaagaa tcaaatagat gcaataaaaa    634080
     atgataaagg ggatatcacc accgatccca cagaaataca aactaccatc acagaatact    634140
     ataaacacct ctacgcaaat aaactagaaa atttagatga aatggataaa ttcctggaca    634200
     catacaccct cctaagacca aatcaggaag aagttgaatc cctgaataga ccaataacag    634260
     gctctgaaat tgagacaata attaatagcc taccaaacaa aaaaagtcca ggaccagacg    634320
     gattcacagc caaactctac caaaggtaca aagaggagct ggtaccattc cttttgaaag    634380
     cattccaatc aacagaaaaa gagggaatcc tccctaactc atttcatgag gccagcctca    634440
     tcctgatacc aaagcctggc agagacacaa caacaaaaaa agagaatttt agaccaatat    634500
     ccctgatgaa catcgatgca acaatcctca ataaaatact ggcaaaccaa atccagcagc    634560
     acatcaaaaa gcttatccac cacaatcaag tttgtttcat ccctaggatg caaggctgat    634620
     tcaacatatg caaatcaata atcgtaatcc atcatataaa cagaaccaaa gatcacaatc    634680
     acatgattat ctcaatagat gcagaaaagg ccttcgacaa aattcaacag cccttcatgc    634740
     taaacactct caataaactg ggtattgatg ggacttatct caaaataatt agagcttttt    634800
     atgacaaacc cacagccaat atcatactga atgggcaaaa accggaagca ttccctttga    634860
     aaactgtcac aagacaggga tgccctctct caccactcct attcaacata gtgttggaag    634920
     tatggccagg gaaatcaggc aggagaaaga aataaagggt attcaattag gaaaagagga    634980
     agtcaaactg tccgtgtttg cagatgacaa ggttgtatat ttagaaaacc ccatagtgtc    635040
     agcccaaaat ctccttaagc tgataagcaa gtttagcgaa gtctcaggat acaaaatcag    635100
     tatgcaaaaa tcacagacat tcctatacac caataacaga cagagagtca aatcatgagt    635160
     gaactcccat tcacaattgc ttcaaagggg ataaaatacc taggaatcca acttacaagg    635220
     gacgtgaagg acctcttcaa ggagaactac aaaccactgc tcaatgaaat aaaagaggat    635280
     acaaacaaat ggaagaacat tccatgctca tggataggaa aaatcaatat catgaaaatg    635340
     gccatactgc ccaaggtaat ttatagattc aatgccatcc ccatcaagct accaatgact    635400
     tttcttcaca gaattgtaaa aaactacttt aaagttcata tggaaccaaa aaagagacca    635460
     cattgccaag acaatgctaa gccaaaagaa caaagctgga ggcatcacgc tacctcactt    635520
     caaactatac tacaaggcta cagtaatcaa aacagcatgg tactggtacc actacagaga    635580
     tatagatcaa tggagcagaa cagagccctc agaaataata ccacacatct acaaccatct    635640
     gatctttgac aaacctgaca aaacaagaaa tggggaaagg attccctatt taataaatgg    635700
     tactgggaaa actggctagc catatgtaga aagcttaaac tggatccctt ccttacacct    635760
     tatacaaaaa ttaattcaag atggattaaa gacttaaatg ttagatctaa aaccataaaa    635820
     accctagaag aaaacctagg caataccatt caggacatag gcttgggcaa ggacttcatg    635880
     actaaaacac caaaagcaat ggcaacaaaa gccaaaatgg agaaatggga tctaattaaa    635940
     ctaaagagct tctgcacagc aaaagaaact accatcagag tgaacaggca gcctacagaa    636000
     tgggagaaaa tttttacaat ctacccatct gacaaagggt taatatccag aatctacaaa    636060
     aaacttaaac aaatttacaa gaaaaaatca aacaacccca tcaaaaagtg ggtgaaagat    636120
     atgaacagac acttctcaaa agaagacatt tatgcagcca acagacacat gtaaaaatgc    636180
     tcatcatcac tgaccatcag agaaatgcaa atcaaaacca caatgagatg ccatctcaca    636240
     ccagttagaa tggtgatcat taaaaagtca ggaaacaaca ggtgctggag aggatgtgga    636300
     gaaataggaa cacttttaca ctgttggtgg gactgtaaac tagttcaacc attgtggaag    636360
     acagtatggc gattcctcaa ggatctagaa caagaaataa catttgaccc agccatctca    636420
     ctattgggta tatacccata ggattataaa tcatgctgct gtaaagacac atgcacacat    636480
     atgtttattg tagcactatt cacaatagca aagacttgga accaacccaa atgtccatca    636540
     atgatagact ggatttagaa aatgtggcac atatacacca tggaatacta tgcagccata    636600
     aaaaatgatg agttcatgtc ctttgtaggg acatggatga agctggaaac catcattctg    636660
     agcaaactat cacaaggaca gaaaaccaaa cactgcatgt tctcactcat aagtgggaat    636720
     tgaacaatga aaacacttgg acacaggatg gggaacatca cacactgggt cctgtcatgg    636780
     ggtgggggta gtggggaggg ataccattag gatatatgcc taatgtaaat gaggagttaa    636840
     tgggtgcagc acaccaacat ggcacatgta tacatatgta aaaatcctgc acgttgtgca    636900
     catgtaccct agaacttaaa agtataataa aaagattaat atgattcctt ccagtgtttc    636960
     aagaaaactt tttctccaaa attttttcca gcgtataaat ttttctggct gaatattata    637020
     agaggctgtt gctaaaaaga catactagaa tggcagcttt aatctgttca tgataggagt    637080
     gagcataata catgatctct ttgagtgttc tgcctcatcc aaatgcagtg tggcaaaggc    637140
     tagtattgga ggtaaaacct ctaaatgaat tttttccagc taccaagtca taggacaata    637200
     gcagatacgt ccttgaggta gaggaccata atgccacagt gttagtagca gtacatttgt    637260
     ccaagggagt tccagggttt attaaaatta tgataatttt aatgtaagaa taccattctt    637320
     ttaaacttat gtggaagatt gtaggaggtt ttcacaatgt tgtcctttac tatcagccat    637380
     gcctcacata atcactttat aatactttca tgggtgttct tcactcagtt catgcaaacc    637440
     ctggcagttt acaggctaaa aacacaaaat acagaagctt ttaatcaact gaaagagctg    637500
     tagccttttc tcaaggaatt atttcaagac acggagaaca cagatggatg ataaataaaa    637560
     tacattcaca tccccttaag ggtagaaatt gaggaaaatt tacccaaagg tgtccaaagg    637620
     gccctgtgac ttggtttctg tctaagtccc agtgttaggg ttcttccagg attttgtggc    637680
     tagctcgtca ctcctcatca agaattaacc tctgctgttt cctcaaagtg tttatttaat    637740
     ctgataacca gtttgtctat aaattataga gttgaaatgc aacaaatttc atttgaaata    637800
     atctggcaca aattatctaa gcaaagtcga taatgatgtt ttttcattca tttgtattta    637860
     agatcatcag ctaagcgttg atatttttga taacacccat gtctctctca gcagttttgt    637920
     cttctgagga tatgatctgg cgtggcagtt ctcttagctt cagtgttacc tctttttgcc    637980
     ttgacttcca cctttatatt tgcccggaat ctgggatata ggagtgcctc taagagtccc    638040
     ctaacttgcc cattttggtg agtgttctgg aacctatgag atgctctttt gtttacaaag    638100
     caccccaaag ccatgtactg tgccaaaagg tgtttatttc ctagtttgac aaattagaaa    638160
     gttctaataa atacaatcac ttctgccatc tgggctgatt ttatatcaga tagaggactg    638220
     tgttctaaag gaaatcttac attagtaaga gcaattttag tcattaacct agagttgtac    638280
     tattgaggtg caagtcataa gaaataatat taggtcgagg ttctcagtgg gagtgtctaa    638340
     atgtcttgtg tgtacagggt ctcctctatt aacatgaagc atttttgagc acagtcagtt    638400
     tccagctatg cttctccctg tctcactatc accacaaact aactatgacc ccacctggaa    638460
     cctaggttaa tttccaaata agtcattctt taatctttgt gcctctagat tattatgtga    638520
     gtcaatttca gtagaaactt aaaaagaaca tttgaacgga ctaaacaaca cagaaaatca    638580
     ctaataaaat gcaaagccta gatgtgagag gctccaggcc tggataatgg aacacttcat    638640
     gtatgcaggt aatttctttt cccaattcaa cactgataca cccagcatat cagcttcatg    638700
     ccccatttta ctcctattat gtagagatat ggcaatgaca ttctcaaggt ccacacacat    638760
     atatgaaaat tggtaggact aggggaggag atgagtctgc acttctcctc tgaaggacca    638820
     ggaaagcctg gacagaccat ctccccggcc tccatgactg cacctcctgc ccacatggac    638880
     actcacccct gataggataa gaggattcca ttgatgaggc tgagtatttt atgatataag    638940
     ttactagaga gtgacattga ggtttcaaaa actaatattt ataacctaaa tttaatttcc    639000
     cccaaattgt tacctctttc ttcagtgaaa aattgcttgc cgtgattgag atttaggtag    639060
     attccaaatg ttcacaacta agcttccaag agcgtattgg gagcaaaaac tgaatgaggg    639120
     tagagaaacc catcatcatg tattcaacac taaattcaag tggactcagc actgcctttg    639180
     atcactgctg cttctctgca gagttcaggt ttctacttct cacgattcca acatagactc    639240
     tctctctccc aaatgtttgg cctccacttg ctgtaaggtc gcactctgtt cttagtttgt    639300
     tctctgaacc attttatgag ggtggtgatg agccatttaa tggaaatttt atatcatttc    639360
     agctgaatcc catgggggat catgaattat tcttctggtt atagtaatgg atttttcttg    639420
     gtgtgttcat taccagtaat attcaaagtc atttcattta aatctttgat gcttcatttt    639480
     ttgttgctat gacattcttt cttctattga atcttcccac tagcattata acatgatcta    639540
     gtatccaggc tcagttgtca tcaataataa ccacgtatgt caaaacctat gtcttctttc    639600
     cacaggagac atatttttct cttttcaggg gatgaacaca cactgctgag tgacccccac    639660
     tcacaagtac atatacacaa ttatgagatt tttatttatt tgactaataa gatgtatcat    639720
     tctcccttaa aattcttcaa cccccagaag cccgtgacaa actcttctgc atctgttcat    639780
     accataaact cagaaaacaa accacatggt gagtaaaagc tcacacggtt ctggatatgg    639840
     ggtccatctc ttcccctgca atgtcctgca gcacctcaga ccacctgtcc tggttctatc    639900
     aaggaagttt acctccagca cagtaaagaa aacaattgaa ggaggagaga acctgcagat    639960
     gatagacaat attcaaaact attcatatga caagggatta ctatcaagca cataaactca    640020
     actgaactgc aaataacaat atgattaata atgggcgaat aacatggata aacgtctctc    640080
     agaggaagac atgcaaagga tagatagata gatcgatagg gatatataca tacatatatt    640140
     gctagaaata actaatatca ggaaaatgca aattcacaat gagctatctt tccttaatct    640200
     ttgctggaaa gtctgttagc agaaagacaa cataaggaaa acaaaagctg gccaggcttc    640260
     atggaaatga acacttttat agaccacttg tggaaatgta aattagtaca gccatcatgg    640320
     gaaaaaaaag acaactacca tataaatcag caatgcaact gctgagtgtg tgtgtgtata    640380
     tatatatata tatatatata tatatatata tatatatata taaaattaaa agaaaaaact    640440
     aatattgaag agatacctgt cgacccatat ttattgcagc cctaaacaca attacgatat    640500
     gaaattaatg tatgtgtcca tcaaaacgtg aatgggtaaa taaaacgggg tatatttaca    640560
     caatggaata ttattcagcc ttaaaaatga aattccgctg tttgaaacaa catggatgta    640620
     actgggaacc gttgtgttga gtgaaacaag ccagacacag aaaaaaaaat acctcatttt    640680
     ctcaatgtta tgtagaagtt taagaaagtt catcttctag aaggaaatag tagagtagtg    640740
     gttatgaaat atcagaaatg ggtggagacc gagagataag aagtggtttg ttaacaaatg    640800
     cacaattaca ggtagacagg tgggatacgc tctagtgccc tacagtgcag aagggtgact    640860
     acagttaaca atatattgta cactttatgt ttacaagtag ccagaagaga gaattttgta    640920
     tgctaccaag caaagaaatg ttaatgtctg agctgatgaa ttagctcgtt gttctgattt    640980
     ggtcaaacca cattgcacag atgtattgaa atatcacact gtaccccata aatataaaca    641040
     atcattatct gccaacttaa aaaatccttt aattaaaaaa gattttattt gcatacatta    641100
     caaatgattc aacacagagc caggaaaata taacattttt atttgaaata gttaaattcc    641160
     taacaaaata gttacattct tttgcaccaa actgtgtatt tgatcatggt aagtatagac    641220
     agacttatgc aaagaataat atattttaaa cttagacttc tacttaatac tataaataaa    641280
     aataatttta gaacagctaa gtaaaaggaa taaagttgag gaaatgtgtg tgtggtgtgt    641340
     gatgtgtgag ctttttcttg taaccacact gtccatggtg gatgtgtggt gtgtgtgtct    641400
     gtgtctgttt gtgtttcctc tgctaggggt tctctgtgat tctaggatct gtagttcagt    641460
     gtgtttcaaa aaattgggaa cattcttagc cattactttt tcaaatactt tctctttatc    641520
     tgcaatttct ccttttcaga attaaaatat acatatacta cactttttga tattaatgtt    641580
     tagtttcttc agtctctgtt cctttgcaat ttacattgtg aattttctag agacaattta    641640
     agtgcatggt ttcatttaaa agctgagcca gctctactga ggggtatgcc caaagtttgc    641700
     tcaatgttta tactgcattg cttttggttt cttatgcact tccatttgat tctttcttaa    641760
     tatttatatc tctcagttcc ctatctagtc ctgcattatg tccacagttt cttgaagaga    641820
     ttctcacata tgaattacag ttactttaca tatcttgttt aatcagatat ttctaatatc    641880
     tgtatcattt acaaatctca ttctgaacat ttgtttattg tgactttagt atttctcatt    641940
     aatataggta ataattgttg ttgataacca gacatgttgg gtcagacagt tgatattatc    642000
     ttattatttc attttatcca ttttctgcct gtatttgacc acactttatc tttgccaggc    642060
     ctttaatgtg gaaatgtctg aatcttctca gagctacatt tgacatttac ttttgcagtg    642120
     gacgtcagag ttgaagtctg ttcttctgtg cccgacagag acttcagatc ctccagtgat    642180
     accttgtttt tctttcctgc ttggctttgt ctcttcacct gttccctcct ccagagaatc    642240
     tccttcagtt ccttcagggg ggttaagatg ttatattcaa ctgacaattg taaaattgtc    642300
     atagaataaa gggatattta gaataaaggg atattttctg acctttcatg ggtccatatt    642360
     gtgatcctga gtctgggtgt gacctttcca ttgtttctga actccctcca gatgagatgt    642420
     tggtctgtgt gttcttgctg ttttccctgg gtgagagtcc tcttgttttc cccagttgtt    642480
     ccctcccaca gctccaatgt tctcttttgg tgttatcatc ttccagattt gctgacctga    642540
     cccatagatt aaggctctga ttcagtatga aagggggagg tacttctcaa tggaacttaa    642600
     gtaaagacct ctttttccat ctcattttct aagggattgt cccagtgccc ctaagatcct    642660
     ggttttggtg gcttgcccct gcagagtaat ttcttagttc tgcaatgggg ataaaggagg    642720
     tgagtctgaa tgcatttcaa agtgtgggtt ctttttctct cacagacaga cacattggca    642780
     cggaaagatt tttgtaactc tccacattat tgggaaaact gattcaagag gataggaaag    642840
     ctcttcagta tgtggtccct gagaaattca tacaacacct tttaccacac ttgacttcaa    642900
     gcaacccaat atatatatgt gtgtttttct cttgtagtag cctacatttt acatgccaga    642960
     ctctgcccag ttcagctcat atcctggcct tgttactgcc tacaagaact tgcctctccc    643020
     tgcatttcag atttgatgtt tttcttaaaa cttcaagtat ttaaagtatt tttaaaactt    643080
     tgcaaaatcc cattttctct gtttacctga tattgttgat gttgttattg taaaaagtaa    643140
     gtagaatata ttcctcatct atgtacgttt caaaactgag ttgcagcttt ttttggtaag    643200
     acccagagtc ataaaagact gcaaatactg ttaagctgct taacagaaaa acaaatgctg    643260
     gtaaatgtgt tcactggaat actacccgtc atttataata aataaatgcc tgacacacag    643320
     aacaacataa aatatctaag tatttatacc gagtaaaata tgccaaacaa ataagaatat    643380
     atactatgtc atttcatttt tataaatgct gatgaataaa aaatgcatct gaagtaaaat    643440
     aatgaggatc agtagttgct gggggaaatg gtagaaggga aggggagagg aggaggaata    643500
     cagcagaata agaggaagtg ttgagaagaa ttcacttgtc cactttcttg ataatgacga    643560
     cagttacatc acgtttatta attgtacatt ttaaatatgt gaagtttatt atctttcaat    643620
     taagccacat aaaatgtatt acaagcaaat ggaaatttag acaagcacgg agtaatagaa    643680
     agatacaaac atatgttaaa tgtcagaagt atctgaaaat tagtgtgtct ggaccctagt    643740
     tctctccata ttttcaggtg agtgctggtg gcaacatcac acatgctctt actacagaaa    643800
     gtgggttctg aaaaccacac caggcacgtc cagctttgtc ctggagttgg tttaggggga    643860
     tgtcacagcc agtgacgagg agcacagggc cagataccag cgttcactta tcccagacat    643920
     gaactcctgg atgcatacag agccccctcc acttgtgggt ttacttccac ttctgtaaaa    643980
     ggagaaaata ttgactccta cagaacataa tttacacaaa tatttacaga tgaaataggg    644040
     tgatcagggc aaagtgttta tcacagcaca atttcataag acagcatatt ttccaaatac    644100
     catcgtcagc aaacatctgc agggcaccgt cttattatct gggtacagcc tattcctcca    644160
     gcgtcccacc ctagagcttg ttatatagta ggagatatgc aaatagggac ctccctctac    644220
     tgatgaaaac caaccgaacc ctgaccctgc agctctgaga gaggagcctt agccctggat    644280
     tccaaggcct atccacttgg tgatcagcac tgagcaccga ggattcacca tggaactggg    644340
     gctccgctgg gttttccttg ttgctatttt agaaggtgaa tcatggaaaa gtagagagat    644400
     ttagtgtgtg tggatatgag tgagagaaac ggtggatgtg tgtgacagtt tctgaccaat    644460
     gtctctctgt ttgcaggtgt ccagtgtgag gtgcagctgg tggagtctgg gggaggcctg    644520
     gtcaagcctg gggggtccct gagactctcc tgtgcagcct ctggattcac cttcagtagc    644580
     tatagcatga actgggtccg ccaggctcca gggaaggggc tggagtgggt ctcatccatt    644640
     agtagtagta gtagttacat atactacgca gactcagtga agggccgatt caccatctcc    644700
     agagacaacg ccaagaactc actgtatctg caaatgaaca gcctgagagc cgaggacacg    644760
     gctgtgtatt actgtgcgag agacacagtg aggggaagtc agtgtgagcc cagacacaaa    644820
     cctccctgca ggggtcccca ggaccaccag ggggcgcccg ggacactgtg cacggggctg    644880
     tctccagggc aggtgcaggt gctgctgagg gctggcttcc tgtcatggcc tgcggcggcc    644940
     tcattgtcaa atttccccag ggaacttctc cagatttaca attctgtact gacatttcat    645000
     gtctctaaat gtaaaacttt tttgtccttt ttgtttcttt gtttttgtaa caggaggaca    645060
     caccctcacc tccacagaaa ccacagtgtc actttggggg cagatgatcc ttctgtggtc    645120
     agcaggatga aagttccgag gaatctcagg ggaacccgaa gagtgtttgc cagttagact    645180
     cagggcagcg acctccacgg gaatctctga ttagaacagg ctttgagttc tgataggagc    645240
     caagagagac gctcacacag ggtcagggtc cttaaagcct gatggttttc acagctatcc    645300
     cccctggtct tgtaaaactg tgtacatatg actcagactg attcacttga ccccctttct    645360
     gctaatccat tttccttctc tgcatacttg attctcacaa ttccctttct tctcttcccc    645420
     gaaaactgag gatgtgtttt ctgtagtcta aattccaggg ctcaggtctg caggacctgg    645480
     gtaggctacg gggactttct cactcaccat tgtctggact ctcctgttgt cgtctgtgaa    645540
     cagagatgtt tggaagatga agtggacatt agtcatgaag ggaataatac tagttttctc    645600
     caatgggata ctgatgtaga gctgatcttg tgcttctcac actgtcacag agtttggact    645660
     ctcacccgtg actttgagga gagctgagga tggacactcc attgtgctgt gagctctggg    645720
     taacaatagt agggcctggc taggcagcct aaggtcaata ctgctggcct tcaggaaaga    645780
     caggctggaa ttcctgggaa gacctgcatc tgccgtccac catggagtcc catcgtcttc    645840
     tattatgccc tgattgaatc agccccacct aggttatcta gaacactctt cgtgacttac    645900
     gaaaaaataa tgggaggctc tactaacacc tgtgttatgc catgggagca acacctaagc    645960
     tagtgtgtga ttgagtagat gagactgtgg tctagtcaag gtgacacata aaattaattg    646020
     ttgccattat aatattttat tatatatttg gcaatatagt cgtgctcata ttataaatac    646080
     ttttgctacg ttgttgttat gtcagaagct tttgaataag aacagcttca tcttgaatag    646140
     gggctaggaa aaataagact gagacctgct gggctacgtt cccagtaagc taaggcgttc    646200
     ttagtcacag gatgagatag gaggtctgca caggatccag gtcataaaga ccttgctgat    646260
     gaaggttaca gtaaagaagc tggccacagc ccaccaaaac caagacagtc acgaaagtga    646320
     tctctattca tcctcactac tcattatatg cttattgtat tgccttaaca tgctaaaaga    646380
     cactccccct agtgcatgac cgtttacaca tgtcatggta acatcaggaa gtttccctgc    646440
     atggtctaca aaggagggga ggaaacctca gcttcaggaa ttgcccaggg gactcatgaa    646500
     taatccatcc gttgtgagaa atacattcat ccctttgaga gaaaatgcac agggggtgga    646560
     atgaggctgg gaagctgatg gcacatggtg gaagcctgtc cctgagtgaa ggagaggggg    646620
     aagctggatt gggtggaagg tccctatatt tgtgtgctgt gcaaggaagt gcaaaatata    646680
     attgagtttt gtgcaagtca gtgctgcctc tcaggtgatc cccatgactc ccagaaatgg    646740
     ctctgcttag gaatccctga ggagtcagtc atttcattag agtagaccac aggacatggg    646800
     cttcagcaca cgccatgcca cagatgtcag aaagcagctg ctgggaacct gacccacctg    646860
     cattttgctg cctgtaggaa aaggcaggga ggtgcatcct cagggacaac acactgtttt    646920
     ggatttctat aaagaacacc tgcttttact gtgttttatt ggaaaataca tgaacaaatg    646980
     tgagcctgca aacaattgta attttcagtt attttaattg cattcatttg taattctgca    647040
     tgatatgctg acacagagtt gcatttttca tagatattgt tcaatgaatc aagaacatgc    647100
     attttccact ttcactggtt tttctccgtg tgcagagacc ttgagtagag cctgtctggc    647160
     ccttatccac actatctctt gtgtccccgg gaaaaagcag agatttgcct gactgcagaa    647220
     tctggggtag gagtctgctc agctcacagc ctgcagagaa gcccagtgaa gttttatgga    647280
     gtcagagagc tttcccatgt ggggaacctc tagcttaccc atggccagag attactggtc    647340
     agctgtaatt gagagtttaa caattagagc actcagaagc ctatctcatc gcagcttcat    647400
     tatgtaactt accaccatta gactgcggta ataactcaat cttgattttt ctgaccccat    647460
     tttctcatct tattcatttg taaattgggt tatagaaaat gttatgtttt agactctgat    647520
     tcaaatctca gaagtttatg aactcatcag gaataataca aaatgtgtct agttttgggg    647580
     aaatgtaaat gaatgtccat gcagtgtgtg tgtttgttgt gtggggatat gtcagtcaca    647640
     ccccaatgaa catgatttct gaattatatc atcaaactac aattagattt gctgaaaatt    647700
     gcctgcagta atgtctgtga tgatgaacct gtcaccttgt tgtgaggatg ctcagtgcac    647760
     aggcgaggtt gcacaggaga catccaagga gcagccccca ggcttggcag aattcctggc    647820
     caggagccag acataggact gggtgtctca aaagcagatt ccccaacccc atccctttat    647880
     ttggactgca tgggcatcat gaggaccagc aggagatgct cagaactcac tcaaggcaga    647940
     gcaccattta ggagcaggtg cagcgaggaa acagccagag aacctctgac tagaagcttg    648000
     ggttgttttc ttttgcttaa gagcatccaa gcctaagtat atcaaccact atatgtatat    648060
     atataagtag actatccact atatatagag agagatagat ataatctgta tatatataca    648120
     gattatatct atctatatac attatatata cagattatat atatataatc tgtatatata    648180
     agaaagttat gagttacaga ttatatatat acatttatat atatacattt atatatattt    648240
     atatacattt atatatatac attttataca tattatatat atacagagca aaacgaggta    648300
     aatatgactc tgccattaac tccttgaacc tgggtcattt tttctgagaa tgccgtcatc    648360
     tacagcatga tggttctcat gtcaaaatca acacgcatag ttagtcacca tgaagactgt    648420
     tttatctcat ggtagaaatg tttatttaat taaaaaatta catttcatac gaaagggtct    648480
     gtattatttc tttctcacac tgctgtaaag acatcacctg agaccgggta attcgtaaag    648540
     gaaagaggtt taatggactc acagttctgc atggctgggg agcctcagga aacttacaat    648600
     tatggtggaa ggtgaagggg aagcaagtcc cttcttcaca aggcagcagg atggagagag    648660
     agagagagag agagagagag agagagagag agagagagag agagagagag aaggagaagc    648720
     aggataaaga gccggaaact tatcagacaa accaatctcc tgagaactct atcaggagaa    648780
     cagcgtggag gaaaccactc ccacaatcta atcacctctc accagacccc tcgcctgaca    648840
     cttggggttt acaattcagt attagctttg ggtgaaaaca cagagccaaa ccatgtcaga    648900
     atttctaggt gagaattggt caattcataa gttattagtg aagtatcaga aaaataaata    648960
     gcaccagtgt tgttgttatc cagttaattt ataaaccatg tcaatatcca ttttatgaga    649020
     ggtgtaggaa aagtgtgaga tggatagata gatagataga tagatagata gatagatcta    649080
     ccaaatacaa tatatataac tatattatat atattactat gtatataata tatagtaagt    649140
     gatatatggt atatatatat gacattaatt ctaccaaata cttcacatat gtatgtgcat    649200
     atatatataa tattaggtat gatatatata tgaaatatca ggacatatat atatatatat    649260
     tttataatgt ctcctgggat ttagtgttga attttttaat atctacatgt attccaggat    649320
     aaatgttggt ggacttcata aaatatattc taatttttat gtgcaagatg atcactttca    649380
     aataaggttt agctacagaa accagatgta tctttctacc agaaattact ggaaagcaca    649440
     gtgaaatttc ttaatcagga ttttaagatg cttgacacca ctcaacaaag acagtgatta    649500
     ctaagagaga gaggaaataa gcataaggag ccctccggtg aatgcaggct cagatacttc    649560
     ctcaaaggtg ttgccaggct acagtgctgg agtggaaacc atgtggattt agcattaata    649620
     aaatggagct ggaaacccac gtaggccaag aagtgtggat accacaggat agcaagtgga    649680
     ttgcagaaaa ctgtcagaga gtgaacccca tagatggcag gaggaacacc cagacttgac    649740
     agagtgcaga tcagtgtgta gaaagcaggg aaagttgtac gtgagaacac accctgctgc    649800
     aggaagctct ccctaaaatc cagagccatg gtaaatatat gaatatacac ttacaaactt    649860
     taaaacatac ggaaaggcac cataataaag tcaaataaag gaaaggtggg ttggctacat    649920
     taaaatataa aaagagtatt tctgagcaaa taataatact tatgagggga acaattttat    649980
     tctaccatgg caaatgattt cagagagcaa cattcgaaat gcttatggcc ataatgagga    650040
     agcttcaaac aagtatacga atagaattat taccagagtg gtagtaccag agccctcagc    650100
     tttctgatga aaactgattc ttattatgaa agctaattat tattataagc ttattattat    650160
     taacactaat tattattaat tataatatta tataattatt ataatattat tatgaaagct    650220
     tattattatt atgaaactga tattactaga gccctaagct aagtgttatt accagagtcc    650280
     tcagctttcc gatgaaacaa caacgtctga taaaaattgt aataacccaa acatataaaa    650340
     tcaattcaaa ttattctaag gatgcacagt aaataaaaat cattgattga taaagtgtgc    650400
     tgctatagac tgaatgttta tttccgccct cagacatttg ttggaattct aaccccaatg    650460
     tgatggtgtt acgaggttgg gccgttggta agtgaatggg tcacctttgg aagatgacta    650520
     ggccccatcc accctcatga atggaatcag tgcccttgtt agagactcca gaaagctccc    650580
     tctcctcttc caccaggtga aggaaaagta gaaagatggt caactatgaa ggaggaagca    650640
     ggttctcacc agataccagt ttcaaagcat gttggccttg cacttgctga ccttcagaat    650700
     tgtgacaaat acatttatgc ttgatctgaa ccacccagac tattctctta cagcagctaa    650760
     aacatcaagt ctcagctagg gcagtgaggg cccacaatac ttgagagacg agcatcaccc    650820
     tctcctttcc agttcaggat gacagaagct tgatctcggg ctgaactgac aagcagagag    650880
     tccttcctct cccccagatc gcagttgagg ccgtggtgtc agatctctgt ggttgagagt    650940
     gaattccagt ctgtgatttc atggatgcct ttcttcatct agttccactt atgagacaga    651000
     gattccatgc tatgagttat aggccaagaa tagaggaacc ccaattattc ccacccagct    651060
     cacctgtagg gcagaggatc tggagagctt tctgggtgag gcacaaagag actgtatgag    651120
     tccacccctc ccatgcagtt tcctgtactt aaagcagagg tgtccctctg acacagatga    651180
     accctttctt ccgcaccaag gcagaggcgg ttatattttt acagtggcag acagagggca    651240
     taaaaacaga aatgcagagc tctctctaat ggatgtgact ttatttgcag taaaaggaga    651300
     ataatttcaa ccctaggttt tttctaaaat attataaata tttatggtaa gttattaata    651360
     gaaagctggt ggcttcatga gagcaacaca ttaaacagga atccactcat ttcagcagac    651420
     agaaggagaa aaaccataat ttaggaataa tgagctgggc actgtggcac tgcctgtggt    651480
     tccatacact tgggagtctg atgtgagaat aacacttgag cccaggattt caaggccaac    651540
     ctgaaaacgt agctagaccc catttagtaa aataaataac attttggggt caggacaaag    651600
     ttcaaaaact gcccctgaaa tgaagcccaa atttaattgt gtaacactgt ggattaatgg    651660
     atgacacaac agtgtcacca tgcctgctgt gagtgcagtg taacacttgg atgacatatg    651720
     aagagataca gacacccaaa cagagacatc agacagtgaa gctgtcaaag gctgcactgc    651780
     cgagcgtcag gctttttatg gaaaccgtgt gtacatctgg aactgcacct gggaatgacg    651840
     acatcaaccc tttaatagtg tcggggaaag agacgtcact gaaatagaca aactaaggga    651900
     ctcaagaaat aaatagagcg attggtttta ccaatacacc agagcgtttt gggtgatcac    651960
     tattcagagt ttctgaattg tgttacctaa ctgtcattca gaaaataatt atgagataca    652020
     caaagaaaaa aataacgtca cgaatgtaca ggaaaatttc cagaaaacac aaactgtctt    652080
     tgagttggta cagattttgg acttaacaaa tggagacttc aaaacaatta ttataaaaat    652140
     gcacaaatca caaaggaata ctatcctaga aaagttaagg aaggtatgat gacagtatct    652200
     caccaaaaag ttaatatcaa taatgagaga gggtttttga aagaaagtta tagacacgct    652260
     aaaattaaaa acacagtaat tcaaaactat atgtcagagg tgataagcag tatacttgaa    652320
     cctaaaaaga aaaaaaagca cacttagata tatattagta gatatgctat attcaaagaa    652380
     tagtgaaaat tgcaataaaa tgaaataagc ttaacataaa aggtggaccc tgctagacaa    652440
     atcaacatat gtttagtgtg aacattagca tgagaagaga gaaagaaata tgtcacaaaa    652500
     atatgtcata atataatgac agataattcc atgattttta tgataaatac taatatacac    652560
     attgaagtat gaatagctca acaaactcaa ggtaaaataa gttataagag atcctcaaca    652620
     aaacacatta taataaaata ttttacagaa aaactactga gaaaatcttg aaggcagtga    652680
     gagaaaaaca acttctcata tacaagataa ttctatcaac agctgatttc tcatcaataa    652740
     cagctaatgc taaaaggcag caggatgaca tattttccct ttgtatccca ttttatcctc    652800
     aaaggccacc tgcatacaaa gtagaccagt cttacaagaa aatcacagcc ctagtgttct    652860
     tctctcaagg agcccggcta tggacctaat ggtgccctcc ccaaagtcat aagtataatt    652920
     cccaacctgc aatgttacag tatttggaaa tgagactttt gaaagtgata gagtttgaat    652980
     atttgtcctc tcccaaggtc atgttgaaat gtaatcacca atgctggagg gggggcctgg    653040
     tgggaggtat tggataatag aggcagatcc ctcaagaatg gcctatgcaa tccatgtggt    653100
     gatgagtgaa ttttctgtta gttcgcatga aatctggttg tttagaaaag tctggagcct    653160
     cctccatctc ttctcttgcc ctttcttggc cccatctctt ctcttgcccc ttctcttgtc    653220
     atgtaatgca cctgtgtgcc tgctgcctct ttgccttcca caatggttat aaactttctg    653280
     aggccctcac aaagcagatg ctggcaccac gcttattgta aagcctagca aatggaaagc    653340
     caaaatgaaa cctgttttat ttataaattt tccagcctca ggtactcttt cctagcaatg    653400
     caaggacaca gcaacacaga aaattggtat caagaagtgg agcattgcta ttaaaaatat    653460
     gtgaaaatat ggacgcagct ttggaacagg ataaccgtca gggatttgga gggtttggag    653520
     ggctcagaaa aagtcaggaa gataaagaac agttgggaaa gttttagaga ctggttaaat    653580
     ggtttgacca aaatgatgaa ataaatatgg acagtgaagt caaggcagag aatgtctcaa    653640
     atgtaaatga gaaagttata gggaagtgaa gtaaagatcg cctgtcaagc aaatcacttg    653700
     actgcattat gttcatgtcc tggggatctg tgcaagtttg aacttacgag tgacgactta    653760
     gggtatctga cagaaaaaaa attgtcagta gcaaagcatt caagatggag tctgcctgct    653820
     tctaacagtc taaaatcagg tgcagaagtg aataaatgac ttaaagttgg aacttctatt    653880
     taaaagagaa gtggaacatg gaagtttaga aaatgtacag cctggttctc tggtaaagaa    653940
     ataatccagt tacactgtga agcaaccgct tgctagaaag attagcttga ctaaaaagga    654000
     gctgactgtg gtaatacaaa acaataggaa aatagtcttg aagtcaattc agaaatcttt    654060
     gaggccaatt ctctcattac aggcttaggt ataggagcag agaatgtttt tgggggtgag    654120
     accagctagg gctcaaaggt acagctcggt tcaccacgtc tgatggcgca agctgtgatc    654180
     cttggtggct tccatgtggc ataaagcctg ggtctgcaca gtatgcaaga gtcaacatgg    654240
     cttggtgact ggcctctaga tttcagagga tgtgtgagaa aggctaggtg ccagaaatga    654300
     gcctacctca gggtcagagt acacacagag taatttttcc agggcaatgc tgaggtgaaa    654360
     tgaagagtta aatcccccaa aagagatccc caggagaaca ctgtgtagtg gagctgtgga    654420
     gagaaggtca ctggccctct agaacccaga atgggagatc caatagcagc atgtgcccca    654480
     agcctagaaa cgctgaaggc actcagctct aaactgtgac agcagccgtg ttggctgcac    654540
     tgagaaaagt cacataagcg gttttgctct cagacttgcg agcccataat tgcaccggct    654600
     gcaagatgtg gagtcactgg tcactttgga ggcttaagat cgagcgcctg tcctgctagg    654660
     ttttaggctt gcatagagcc tgttactcat tcatttggca agttcctctc ttttaaaatg    654720
     gagatgttta cccaatgtcc gcaccaacat tgtacctttg aagtaaataa gttctttcta    654780
     attttacagg ctcacagatg gaagaaatgt gggcatgaat ctcagacaag actttggact    654840
     ttaatggtga aacaagttca gatttaaggg gcagatttcg aggggtgggt attgggaaga    654900
     aaatgatccc attttgcagt tgagaaagat ataagatttt gggtgataag agttatgtgg    654960
     cttccatata tgtccattca aaaatctcat gtttgactgt tatccccaat gttggttggg    655020
     gttttgcgtt acgggggcgg attcctcctg agtggcttag cgccatccac ttggtgacga    655080
     gtgagttctc acatagttag atcactcagg atctgattgt ttaagggaga cgtggatctc    655140
     cccctttcct tctttcccct gctcaccatg tgacactcct gcttcccctg tgccttttac    655200
     cagaaatata aggtttttga agccctcacc agaagcagat agcagaacca cacttcctgt    655260
     acagccttca aaacagtcag ccaaattaga atttattttt tataaatttc tcatccccag    655320
     gaatactttt atagcagtta aggaatttct ataagaaaga aagtactgaa tttaaaggag    655380
     acgatgaggg tgaaacatca gaatgaaatt actgacctca tgagtaaaga caccagacag    655440
     tctctctctc tctacttctc tctctctatt tctctctctc tctctctctc tcacacacac    655500
     acacacacac acacacacac atgccctaaa tgaaaagatc atatgatcaa gtaacaatat    655560
     gatggctgct ctgagccaaa agtagaagcc tcaaactaaa cctatcttgt cagcatcttg    655620
     atcttggagt tcacagcact gataaatgga ataaaattaa tgttgcacga gtcaccaagt    655680
     atgacagttt gtaatggcag cttaaactga cgaaagaaaa tttggggtta agaagaggaa    655740
     atctgatgtt acaattattt gaacattgtg aaagtagctt ggaattgggt tatgtgtaac    655800
     aggatggata cctttgacat atatgaagaa atttgcatat tgagggtcat gctgatagac    655860
     agaaatgagt ggttttggaa aatggagcaa tagtcatcct tatcataaag ttttttaaaa    655920
     aaaacttggc tgggcagtga tataatattt agtgaaagag aagttgtgag tagtgagaaa    655980
     ttgtgagtag ctgagaatag ttctaagtgt tgaagatatt ttgcttgatt cctcctgagt    656040
     gcttaaaata agtgaggtag ataaattaaa atcagaaaaa aataaaagaa aaagaataca    656100
     aaagctgagg gcttagaaaa ttcacaacct atccatattg caaaaatcag aaactgtatt    656160
     ctacaaagaa cactgagtat atggctcaaa tatcactcag taagaagctt aagatactct    656220
     atgaaaagaa atatttcagg ttttaaagaa tgagaattga cctgagacaa atttgatgca    656280
     tactgttaga tttttcagat ttaatggcct ggaataatag agctacttgg ctgcaaatgt    656340
     acacaattct tccagaagag agaaaaataa gctttaaatt tatttagaaa ccatcagacc    656400
     actgcctcta tttcaacaga ccagaaagcc tgtttctaaa atcttgagga agaaaccacc    656460
     caacagaggc ttgggttggg agcacccagc aagccctgtg tgtagggctg catgaggccc    656520
     ttgacatggg agctgggcct agcagtgcct caggagcggt gcctctgcct gcagctttgg    656580
     agctgattct gctgctcgaa taagctgaga aggaaaaaga atgtttcaga gaagattaca    656640
     gtggaggctt aaagcatatg gcaatttgtt ttcctatgca ttgtatttgc tggagacaaa    656700
     ccacccattt taccccctat ttttttcttt ggcaatgaag ctttctattc tatgcctaat    656760
     tcaacaggac acagcagcgg ctgtatccag tttgacaaga ttcagcatga aaggttccta    656820
     ttacctgtgc cggatgcatc actgatatgc ggccaccagt ttcttaggtt catatcactt    656880
     taagggacca aatacaaatg tcaatagcat atgtcattct taaactgcag aaatggtgtc    656940
     aaagaagaat gtggagataa gagagcagga ggaattatga aaatccagat agcatcttct    657000
     cttctagagt ggaaggtaca ctagtaattt catgaaacaa taaaatagat gaaatgtgtc    657060
     atcatcatgg agtgtctcac agtggcagtg cacaaataaa tttctaaaaa ttccataagg    657120
     taggtgtgat ggatgaggct tatttccaca cagggagccc ataaataccc agataagtaa    657180
     aaaatccaac acctggaacg aaaaaatgcc tcatcttgac atcacaaacc catctcagta    657240
     agtccgagga gtaatgtgga aatgaaacgg aaaattcaca aacacattta ttgtgaaatt    657300
     tttagcacat gtttgaacat taaaacagaa ttgtccacaa ttttaggaaa accactccat    657360
     gacaatgagt cactacactc atagaaacac cactgagtca acagagttct aactgtcagc    657420
     aggtcagtac tgaggcctca ggagaatcaa ggctctaggg tacattttac agaacccaga    657480
     attgagccac acaatctata gccaaatgat ctttgacaaa cttaacaaaa attaacaaaa    657540
     atatatacca gaaaaaggac accccattca ataaatggtg ctggggaaat ggaaatgaac    657600
     atgcagaaga acaaaactag acccctaccc tcaacacata catactcaac acaaaatgaa    657660
     ttaaagactt agatataaga cctcaaacta taaatgtact caaataaaat accaggacaa    657720
     ctcttctggg cattggctga ggtaaagaat ttatgactaa caccccaaaa gcacacccaa    657780
     gagaaaaata gacaaatgag acttaattaa cctaaagaga tatttcgcag caacagagtg    657840
     aacaggcaag ttgcaaaatg acagaaaatt ttcgcacacc ttgcctttga caaggaacta    657900
     acatgaagaa attacaagga attaaactat acaacgacaa caacaggaag aagaaccaaa    657960
     taaccctatt aaaatgagca aaggacatga gtagacattt ccaaagaaca catacaaatg    658020
     gataataaat acataaacaa tgctcaacat cactaaccat tagggaaatg caaattaaaa    658080
     ctacaataag atattatctt acaaaagcca cagtgactat tattaaaaac tcaaaagtat    658140
     cagatgttgg tgaggatgga aggtaacagg aactcatcta cactgtagat gaggatgtgg    658200
     acgaggacaa cctctatgga aaatagtatg gagtctgccc aaaaaactgg aaatagaact    658260
     gccatttgat gcagcaatcc caccactggg taactactca aaggaaaata aatcattact    658320
     tcaaaaagat atccatgctt ctatgtttac cacaaaacta ttcttaataa cacacatgtc    658380
     aacctgagtg tccaccaaca gatgatttta taaaagaaca tagcacgtat acacaattca    658440
     atactagtca accacaataa taaatgaaac tgtcttttgc agcaagatgc ctagaactgg    658500
     gaacaacata attagtgaac taactcacaa acagagagtc acatgtcaca cattattatt    658560
     tataagtgga aggtaacaac gtgtccacaa ggatatggag agagaaatga tggacaccgg    658620
     agacttagaa ggatgggagg tggaaggtgg gagcatgagg agacattacc tagtgggtac    658680
     aatgtacatt atttgggtga gagtacacta aaagccaaga ccactgtgga atatatccat    658740
     gtgcaaaagt tgcactcata gcctttaaat ttatataaat aaatgtacaa acaaaaaaat    658800
     taaaaatata aaataattaa cagttgacca atgatcttaa agttaaaatt tagaccaggc    658860
     gtggtggctc acgcttgtaa tcccagcact ttgggaggtc gaagcaggtg gatcatgagg    658920
     tcgggagttt cagaccagcc tggccaacat ggtgaaaccc catctactaa aaacacaaaa    658980
     attgaccaga tgcggtggca ggcacctgta atcccagcta ctcgggaggc tgaggcagga    659040
     gaattgcttg aacccaggag gcggaggttg caatgagccg agatcatgcc actgcactct    659100
     agcctgggcg acagagcaag actccatctc aaataaaata aaataaaatg taaaaaatga    659160
     tcaataaatg aaattactat cagttgaaac tcattaaatt taaagacatt ttctactcaa    659220
     gtaactataa gaacatgaat gtcaagtttc agatgggaaa ctattttcaa atcagataac    659280
     caccaattta attataataa gaactctcag gactcaactg tgaaaaaaaa aaaaagaaag    659340
     aaagaaagaa acaacccatg gataaaatag gcaaaggttt gtgcagacat ttcatcaaat    659400
     aagatgtgca gatgacataa gcatataaac aggctcttaa caggattttc cattaaagga    659460
     attcaaatca agcccacaat gagaaaccac tatacacttt ttagaatggc tgaaattaag    659520
     aagaaataca ggtaatacca atgctgatga gcataccaag ttcctagtgt ctacgacatt    659580
     gttaatggga ctgcaaaatg aaacagctac tctggaagat aatttttagt tttttctgca    659640
     atcaaacatg cccttaacac atgacctaaa tatcccactc ttgaattttg cttcagagaa    659700
     atataatctt atattcacac aaaacctcta ttcaaatatt caagatattg cgtgtgtgtg    659760
     tgttagaaac taaaaataac ataaatatct caaaatctga ataggtgaaa aaactaggaa    659820
     gcaactataa attgaatacc atcagcaata aaaaatatca aatgatcgat tcacaaacca    659880
     ttacaggtga actccaggca ttacgctaag tgagagaagc cagtctcgaa tatcaaaggg    659940
     acacagctgt aagcagcacg gtcatcctca ggtgtcagtg gtttgggctg ggctttcttt    660000
     gtctctttcc tgaccagacc cagatgttga gctctgccac ttgcagatgg aaaattctac    660060
     tattttcaac catgcactga ggtttgaatt acttcacaga ctgaccaaac aaacatgggc    660120
     tccactgaag agtgtctggc atttgtttca accacaagag aacttttccc agctcttcca    660180
     tgtccttggt tctctcctgc aagccagcag ccctgcagtg tagcctgcat ctcccatgca    660240
     tccacccagc tccttccaag ggccttccac cacaccctcc actgtttttg agagcactgg    660300
     caggctttca atttttccac attctgttgt tactgaagtt aggatgttta ggacttatta    660360
     agatcatatt ttatgactga atttcattgc cccctctctc ctgggacaga gctcctaaac    660420
     aaggttctgc aggtgtagac aaagttgagc tgttttattc ctcagcctag gagctgagcg    660480
     ctcagtggag ggtcgggcag gagcttccca ccttctcagc acttcggtta tggtggggca    660540
     gaacctctgc cataggacag agctaggaac cagagaccca atgttcccag tggcgctgga    660600
     cccagggcag agcctccatc catgagtggg gctctatgga aggagtgagt ctctggctct    660660
     cagtagctct tgtccagcac tgaacctcag catcatgtgc tgtgggcagg gtcagagggc    660720
     caacgtactg gcccctgggg aagagtttcc tctggtggga gttggtagaa ggtgccctgt    660780
     cttcttggct gcatctgtcc gcagtggagt ttacatcatg ctgagctggg atgtggaagg    660840
     aaggaagagc accttagatc aaatacgatg actggcctta ctgagttttc tatattttct    660900
     taaataaata tttcttcact tgctttatgc tgttagatcc tttccaaacc ctgtaatttt    660960
     ttcaaaataa ttttcactgg tctcatgagg gcatggattc attgagcccc tcatgctgtt    661020
     aaagagaaat agaactgttt ttttttttca ctttttagcg aacatccatg ggttataaaa    661080
     taattagttg acatttcttc caacacttta cagataccat caactttcct cttgcttgtg    661140
     aggttttaac cagaagaatg ctatcatcat cttttctgtt cttttggaag gaatgccccc    661200
     tctactcacc tccacttgcc tgcatatatt tctatttgtc tttgcttttc agcagtttta    661260
     ataagattta cctaaatgtg tgtgggggaa gcagggggtg ttattctgtt gttctgtgtt    661320
     ctctgagatg catggattca ccatttactc tgcctccatt ttggggaaca cagttagaaa    661380
     aaatgtcagt gtgagcccag aaacaagcct ccctgaaggg ggaacaggac cacccggggg    661440
     cgctcaggac ccactgagca caagagccag cctcagggca ggtgcagacg ggggttaagg    661500
     tctggtttcc cgtcagccct gtggcttcct ctccataaaa cagtttcctc tggggcactt    661560
     ctctggattc cttatcctgt tcttcctatt cttcctgaag aagaaacatt tgtcgtaaca    661620
     agagaaaaat tttctcacat gcaccaaagg cagagtcacc tacagtcact tattccggtt    661680
     tctcaatgtg aataaattat caatgcttct gaatttaatc agctaaacct attaaaggtg    661740
     cggtgtttaa ctcaacactg ccgccagctc aacagaactc caagggtcag tgagcagcag    661800
     gcaggataaa gggcatgctg ggcactgggg cagagggagt tagcatccag tgcaagagaa    661860
     gaaaggcccc gtggtggtca ctgtcaggac ttcaagccca tagttccaat tgtaggtgac    661920
     cacgttcaaa agaagagaga cctcatccat gattagtgtg atgtgtcaag tctgatagtg    661980
     ccacactcac acctcaggtg attatgaaaa gatttaccaa ctctactatt gtctgctgag    662040
     agcagcacag gcctctcaag aaattccaaa ctggaatttc ctcaatggaa caggaaagga    662100
     ggctggctta gggctttata atgatttggt ggtggggtcg gggggtcatt tatactcagg    662160
     agaaggagct tgtgtgattt aaacctcaca ttggcatcag atgagggagc ttctacgatt    662220
     tctgactaga tttcccatat gtgggggaca agggagaaga agaataaacc ttaattcatc    662280
     agcagcaagc accaaaatag gacctgacac tttattctcc ctagcagctt aagaaaatga    662340
     gtgaaaaaga gagatgaggg tccactgtgt gtgaaaagca aacagaaata aagaaaataa    662400
     aaatttttat tatggtaagc acataataaa aagaaagaga agaaggaatg agacagacag    662460
     tggtgctgaa tcaatgtcct gggtggggcc ttttcattat ccactgtcat cagtttattc    662520
     tgaaggtctc aggtcagctt cctgcttcaa actatcacag gcccttatag ggtatataaa    662580
     aatccttata ggatatccac agtttgtcat actttactaa agtagtctaa taattagatg    662640
     aagttctgaa tttaatattc agttataata aatattaaaa aataccacaa tctacaaatt    662700
     aggaaaccga gactatattt ttatcaaggg ttacagccat cccatatgct ggaaagcata    662760
     gttttggtaa agatgagaga cagacactcc caagaagaag gagttgggca gaagatttat    662820
     gctgaactgt ttggctaaac agacttagtc aacaggttac agagaggcta ctgatgttca    662880
     tggaggtggt gctgacacat gcatactgaa cacacatgca tttaacatgt gagtcctgtt    662940
     caattgccag tggtgactta gcatttaaat tcattacagt caggccctat gtgcaaacag    663000
     cagaagcaga gacacaaagg cactcagggt gcagcctctg taaacagcca gagccaggcc    663060
     atggtcagcg gtctctgatc aggagaaagg tcctgatatc aaatgttcaa tcaaagctgg    663120
     ggttatggct tgtggaacag ggggtcagtt cagcagggat gggctgcaat tgtcttcata    663180
     gtgcttgtct cagtgccagt gcttactgag ccaccagaga aaacgaataa cctattggca    663240
     gttagaacat agtttatctt ttaagtgtag aagtgagtgg caaaatcctc gtctagcaag    663300
     gccttaggtc ttgtttataa tttgacatct tactgccact gttctgtcag tcttatgatt    663360
     tctattttaa cataagtgtt ggccattgct gcgtctaaac cacaaaggga agaaggtata    663420
     acgaggcgtg tctgatctct tgtttagaca tggttggaaa cagtttttgg atttttacgg    663480
     ggttccctag gccaagagat ggactattta atcagtcgtg ggttttaggg atttattttt    663540
     aggttacagt ttataaatat gaacccaact attgactttc tgcaatttca ctgctgtgtt    663600
     gtggttaaaa gtacctgata aaattccttc caaagtggtt caagagcaat attctaccct    663660
     gatgtttctt acaatagata taattccctg ctttaatgtt attagaggct attgaaaaga    663720
     tgtagggaaa tgatggttta atctgttgat gaatagtgtg ggtaaaatgc agtcatcact    663780
     ttcaacactt tgtcatgcat gcatgtaata ggacagaggt gagcattgtg ggtaaatctc    663840
     taaatgtatg ggccttccag ctaccaaata ataggctaat aactgatgtc tccctgaggg    663900
     gattaactat aaggctctct tgctagggag agaatctttg gccaaggaag ttcaagattt    663960
     tcttaaggtt tggacaattt tagtttaagg atgacaattt tcttcaacat tttcagaaga    664020
     ttgtggatgg gatggatgct atgttgtata cataatgcta atgccttcca tatttagata    664080
     atattataaa cccctgactg ataagactgc ttgcttggtg tgatgaccca ctacaatatg    664140
     tatactttca gttggtatgc tatgtcattt tccttcaggt ttttactttc caagttacat    664200
     tagtcaggtc tctaaaatgc aggactgcat ctaaaatgtg ttattttgtt gtgcattttc    664260
     acatgttcca gaaggtggga gaaatttctt tgtgtgaaga gcatcccaaa gtcaggcact    664320
     cctctagaac acgtgtttaa tccttgtctt tggcttattg atgagctctg ttgactgcaa    664380
     taacttctgc catccgggct gacttgacat cacatagagg aacaaattct gaatgaaagt    664440
     ttgctctact caattgagtg cataacctca tttcattact gatgtataat gcatcagaga    664500
     taatattaga tcaaggattt caatgggaat ctaatctaaa agatgtaaac aaattttcat    664560
     aatttagata aaattactgt gagcacactg ctgctattca gccatgtctc ctatcacact    664620
     gaatccacac taactttgac ctaaaagtta gcatttgttc taatgttcag aattgttatt    664680
     tttatcataa cgtctctaga tgattacaga gtgtggctat gttggccatc agtaaaggaa    664740
     agctatttta actgaatgaa aatgagacaa agcacacaac ctaatgtaat ggaagtcagg    664800
     acatgagtgt ctccaggctt ttgtgtttca agagttcatg caccagggga tttcttttct    664860
     tacttgtaca cagagcaaca cccaaaatgt tcatttcctc ttccatcgac tcttatcatc    664920
     tgtttctatg tcagttagct ttgaattttt gaccccagct gcattcaagt agctgcaaat    664980
     aagcgtgatt tcattctttt ttatagctgc atagtattcc actgtgtata agaaccacgt    665040
     tttctttatc cagtctataa cggatggaca tttaggttga ttccatgtct atggtactgt    665100
     gaattgcaca gtgatacaca cgtgagtgga tgtgtttttt tgttacaata atttgttttc    665160
     ttttgggcat atacccagta atgggattgc tgaggggaat tgtaggtctg ttctaagtta    665220
     tttgagaaat ctctagatcg atttccacag tgctttggcc aagttacatt ttcaccaaaa    665280
     gtatgtaagt ggcgcttttc tctatagcct tgcctgcatc tgttaatttc tgatctttta    665340
     gtgatagcaa ttctgacatg tgttagatag tatgtgattg ggttttgatt tgcacttatc    665400
     tgatgattga ggatgctgag tatttttgta tgttagttgg ccacttctat gtctgtattt    665460
     gaaaagtttc tgtgcacgtc ccttccccat ttttaatggg gttatctgat ttatgctgct    665520
     gatttaagtt ccatatagat tactgacttg cacacacgga gaaagccagt attcaaaact    665580
     aaatagaaaa acataatcac ttgtaggtat agacatactg aatttggaat ggtcatggct    665640
     tgcatgttgg tattgaaatg ggacagcaac tttggaaaat ggtcttcact gaaaatctca    665700
     tacacaagtg tacctggtgt caccacttac agtagttgaa tggagaatac aactcaagtg    665760
     cccatcgact gccacaggga tgaagacgcc gcggtgtgtc tgtgtgtctc taacccacag    665820
     ggatgaagac gctgcggcgt gtctatgtgt ctataaccca cagggatgaa gacgccacgg    665880
     tgtgtctgtg tgtctgtaac ccacagagat gaagacgccg cggtgtgtct gtgtgtctgt    665940
     aacccacaga gatgaagacg ccgcggtgtg tctgtgtgtc tctaacccac agggatgaag    666000
     gcgccgcggt gtgtctgtgt gtctctaacc cacagggatg aagatgccgc ggtgtgtctg    666060
     tgtgtctgta atgcagcttt aaagaggagt gaagcactga cataggctgc aacttggatg    666120
     agccttgaaa acattgggtg actgaactga gggagacacg gaagtctaca tccctaattg    666180
     tcccattgac acgaagcacg cagagcagga aaatcccaga tcacaggagg tcaggggaag    666240
     ggaaacacag agtgaccgct taatggaggt gaggtttcct tttctgtgat gaaaatgttc    666300
     ccaaaccaaa tagtggaaat ggttgcacag cattgtgaat gtgtaaagta tcactgaccc    666360
     atacacttac caagggctga aatggtaagt tttatgttat gtgtatctca acacaataaa    666420
     aatgagtgcc acattttgtt tagcatatca atgacaataa cagttcaggt aaatgctgga    666480
     ttcctcatca caaccagtgc ttcctgatgg agctggattc aggggaggga ctggagtggg    666540
     ttgggtgcac aggtcatgaa ggcagcaaaa attccaaccc actcctcaag agtccagtca    666600
     ccacctccag atctatgtcc aaaaaacagc tcttcctatg gctgagctac atgagcaaca    666660
     agcaaataac catgtttttt gttgttgttg tttagttttg ttttgctttg tttttagaca    666720
     gagtctcgct gtgtcaccca ggctggagtg cagtggtgcg atctcagctc actgcaatct    666780
     ctgcctcctg agttcaagtg attctcctgc ctcagcctcc ctagtagctg agactacagg    666840
     cactcactac cacgcccagc taatatttgt atttttagta gagacagggt ttcactatgt    666900
     tggccaggct gttcttgaac tcttgacttc gtgatccacc cgcctcagcc tcccaaagtg    666960
     ccgggattac aggcgtgagc caccgcgctc agccacaacc atgtattttt aagcaaaaga    667020
     cacagtgagg ggacatcagt gtgagcccag acacaaacct ccctatgcgg gttcacagga    667080
     cagcatgggg tgctgaggac agaggtgggc actcaggaac cagcagggaa acccaggggg    667140
     cgcttggcac ttcatggggg ctcaggacca ttgtgggctc agtggtcagg caggctcaag    667200
     gctcagcctc agggcaggtg cagcaggcgg ggaaaggccc tggagactgg gtttagtgtc    667260
     accttctcat tgcgccactg gacaccctcc actacatcta ttctaatgtg tatgagtact    667320
     tatgagttta gaaaataaca ttgatattat aaatctatag ccatatgtgg gtgcatcaag    667380
     ttaccctctt caacctatgt ggaccctgtt catcaggaat aagtccctgc atttgaggac    667440
     cttataaatc aataattatg tagaatcact ttctttttca gtctcatttc ctccctcttt    667500
     ctctctctca cacacactga caaacacgct ggttgctata actttaatta cctgatgtgt    667560
     taaaaaaaat tgattcattt ggaacttaac cagtttagct gttgttcacg ctgttatgta    667620
     aaaaagatcc ctaaatgact tctacctggt aagaggaggg aaggcgttgg aaggtgaaat    667680
     tgcaggagaa aaagaaaatc gtgaaacgga ttgatttgtt ttctctatga atggtgatta    667740
     ggtcaatatt tttcaaacgg tgaacatatt gtgtgaagat tatttgtcaa tattatctca    667800
     ttaaaactat tacaaataaa agtgtataat ttggtaaaaa aaatgaagag agaaataaaa    667860
     ataatacaag aaaagtcatg aactcctgac tccacacgtg agttaaccct cagtttttct    667920
     atgttactta gaaaaacact aagatacagc caaagaatat catgatatct ttataagaag    667980
     agggttttgt aaccctcact cgaaatttct agctctgtcc tagagttaat ttagggagca    668040
     gtcagatcca gctgtgagaa acacgggcca gacagtgaga ctggctttta ccagatgtga    668100
     gctcttagac atgtcacatg gcccttccgt gcttgggggt ttaccttcac atctgtaaat    668160
     gaaggaaact ttgactccca cagaacataa ttcatgtgca tctataagta gaaatgctaa    668220
     tgagaattaa ttatttatga agtataatca ctcgcatcca ctctgggaca cagcctactc    668280
     tgaggcatcc cttccagaag tcactatata gtaggagaca tgcaaatggg gtcctccctc    668340
     tgccgatgaa aaccagccca gccctgaccc tgcagctctg ggagaggagc cccagccctg    668400
     agattcccac gtgtttccat tcagtgatca gcactgaaca cagaggactc gccatggagt    668460
     ttgggctgag ctgggttttc cttgttgcta ttttaaaagg tgattcatgg atcaatagag    668520
     atgttgagtg tgagtgaaca cgagtgagag aaacagtgga tttgtgtggc agtttctgac    668580
     cagggtgtct ctgtgtttgc aggtgtccag tgtgaggtgc agctggtgga gtctggggga    668640
     ggtgtggtac ggcctggggg gtccctgaga ctctcctttg cagcctctgg attcaccttt    668700
     gatgattatg gcatgagctg ggtccgccaa gctccaggga aggggctgga gtgggtctct    668760
     ggtattaatt ggaatggtgg tagcacaggt tatgcagact ctgtgaaggg ccgattcacc    668820
     atctccagag acaacgccaa gaactccctg tatctgcaaa tgaacagtct gagagccgag    668880
     gacacggcct tgtatcactg tgcgagagac acagtgaggg gaagccagtg agagcccaga    668940
     cacaaacgtc cctgcaggaa gacaggaggg gcctgggctg cagagggcgc tcaggacaca    669000
     ttaagaacac agtaaactca gggaccaggt gcccagggag gttaagggct ggtttccttt    669060
     cagagactta gctgtttctc catctaacag tttccccaga accctgccta gatttgtgat    669120
     ctgtatctgc tcaaactatc tgtgtcacct tcctcacctg tgactttgag gaagcttagt    669180
     gtggacattc cagtgggtgg agagatcttg gtgacagcaa ttgtgtgttc tgtctaggca    669240
     tgtctatggc tggccatcag gaacgacagg ctggaattct tggaaagagg cacacctgcc    669300
     atccatcagg aaattttgtt gtcttctgtt ctgctagaat taaatcaggc acgcccaggt    669360
     taactactac tatcttccta gcttgagaaa attaatggtg ggcttgaata acacctatat    669420
     gaaatcatca gagcaacacc tagattcgag tctcatttaa taattgagac tatgttctag    669480
     ccaaggagac acataaaact ggtgattgcc gtggtcagtt catatcatat atttgtcagt    669540
     agaatctggc tggtattatg aacagctttg ccaacacatg tgttagtgtt ggtcttcgga    669600
     gaagcacatt ctatgaatgg attaggttta tgtacagttt cctaaggaac cggtccacgc    669660
     ttttgagagg aaacgcagag cgggtggaag gaggctgggg aagctgaagg catatgaggg    669720
     aagttggtcc ctgagtgaag gagagaggga ggataactca gtggaacttc cttaaactgt    669780
     gctgttctag gaaggtccag caagtcactg tatctgagtc atgtcaaagt ttcccatcgg    669840
     ggacccccgt gactcccagc agtggctcta ctcagatcag cgcggagctc attgttctcc    669900
     tgagagtgga gcacaggacg tggccccagc accagccgtg gcatgcacat cagagtgcag    669960
     ccccaggcgc atggctcagg tgcactgtgc tccctgcagg tggagggagg gaagacctga    670020
     ctcagaaccc atatgatgtg ggttccatac agaacacaac ttttacttaa tttctgtgga    670080
     tgacatagaa ataaacatac agtctgtaaa caatggtgat tcctacattt accttgagag    670140
     ctttattctt tgattctgca gaatgtcctg gcatggtgtt gcctttctca tgtggaagtg    670200
     taacataagt tgaggacatg tgttccccac tttcattgtt ttcctccatg tacagagatc    670260
     ctgagtggag tacagctgac cctccttcca cactgttcct ctcctcccca cgggaacagc    670320
     agataattac ctgaggctga atctgaggtg ggatatgtcc tgtgcacctc agagcctgca    670380
     gagacccccg gctgcagatt catggagtca ggtgtttgtg catgtgggaa tctcgagctg    670440
     tgctgttgtc agtgaatgct gcttagaact aattgcagga ttcagaatta ggagaccccc    670500
     tgatctatca cacctcagct cattctgcca ctcatcatct tcagaaattc tgaatatggt    670560
     tcagtttaca tttttgtgat gtactgttct catttcattt agtcatgagt ttggtcagca    670620
     gaaagtgcta ccatttatgt gtcccaacct gatgaagcta atttgcttca cgagaagtta    670680
     ggaggctcct gaatcttatc ctaagcctgt ctctctgcac tttcctttaa aattcagttt    670740
     tacctggaat tctgttgtgt tgatttagcc agttttcccc atactgagtg tctgattttc    670800
     ctggatatct gatcagtttc cttgtccttc accataactt gggtgacatc tgatcaccct    670860
     ggtctaactt caacaaaaat cctggcaggc tgccctaagc aggatttacc tcactcctga    670920
     tgtttcttct tggtgatttt ccatcctgtg ccccaaccct gctgtatgcc tataaatcct    670980
     cacgttctca ttctgtattt ggagttctgc tgaatctctc ccccactgca aaatcccact    671040
     gccctggtcc ctacacctat catgagggcc ctggatgaag tcttccttac tgtgctggaa    671100
     caagtgtctt catatataca ttttttcttt aacaaatcaa cctttagtat ttcctaattg    671160
     atcaaaggac ctcaaacatt gcatgaaagc gaggatctcc attttactat aaggggtgtt    671220
     ttgtgaattt cagtttccat gtgtggtgtc cagaagtcct gggcaccaac ggttgtgctt    671280
     ctctgtccaa ttctttgatt ctgaaacccg ggagtcatct gaacatgagt aaataggtga    671340
     cttttttcac tatttagtga ctgccttatt tcatttaccc tgaagcttca cgaacacatt    671400
     gatgaaaagt agatagagaa cttgtttgga aatgcaagat ttatagtaga cgtaatcacc    671460
     tacatgattc tggaggtctt cttagtgttc caagaaccag atgtttttcc caccacagga    671520
     caaagatgag aacctccacg ctgtgaggat agaggacagg cagggcaaag atgagaacct    671580
     ccacgctgtg aggatagagg acaggcaggg cagtgtgagg acaaaagggg ctgtggtcac    671640
     gtccagggtg ttctctccta gcaacgcctc cagctctttc ctcactctgt ggtatttgtc    671700
     agatgggaaa catacctcaa atttatattt gacatattga tggaagtcca ggcaagaggc    671760
     atctgaaagc ttatttctga acctgcttct gtaaggaatg cccatatccc ttggtctctt    671820
     tcctctttct taactccatt ggctcccttc ccctcctgca cacacccact gacgtttatg    671880
     gccacttccc cacacccttc actgtagtac cattttcttg ggctcctggg aaacccaaca    671940
     gctttagtta aactcacagc tatgtacacc cataggcccc tcaggaggtt ctcagtttca    672000
     ctcctacagt ctcccaggct gctccttggg gtcctaacct gaccatctct accctctctc    672060
     ctgagtctgg ccatccattg aacgtccctg ctgccctcta atctggattt gtgtacagta    672120
     gaaggttaac tcagcagacc tggattgttt aaaccctgca cattcctgaa aaacattgtt    672180
     catttatggc tctcagttac ctcctgatga gaactctgag cccttaaaat gctatgccag    672240
     ataacagtgt ctgtggttgt ctgcagcctt gggacctgta gtacacattt aatcatagtg    672300
     ctttgtagtg aatgccagct attttttttt cctgggtagc gggggagctg ttgtctgagt    672360
     attgtggtca gttgaaatgc ctaaccttgt tttcactcta actcactact ttgaatcttc    672420
     cctgtttgtc tctttaatca cctagccttg cttctcatgt aagtaagact ctctctagct    672480
     gggaaagctg gacaaactcc aattgacccc ttaatttaca agacactaag ggctcctcac    672540
     ccaaccccct tccgtaagga gttaacctgt gtaaacagat tctcagcatt tcaaaggagc    672600
     ccagttaact gataaggtac tagcaccaac aatgtatgaa gttcccagga tttttctcaa    672660
     ggagataaca acataaagcc ttgagttcat gcccggcata gcccctatat ctaattataa    672720
     tgaaagattt agagccctgc acctggtact gttgctcttt ttgtaaccat ttgtctttta    672780
     aattgtttat ctctctgtaa ccattttgct tcttttgatt cttgcatgtt tttacttccg    672840
     tagaattatt gcacttgagt tcccctcccc ttcctaaacc taggtatgaa agttaatcaa    672900
     gccccttcct cggggcccag agaattttga gcattagccg tctctttggc cgctggctta    672960
     ataaaggact cttaattcgt ctcaaagtgt ggcgttttct ctaactcgcc tgggtacaac    673020
     atttttggag gccgcagcga gatattaaca ccactgggcg agagccggtc tcgctccggg    673080
     ctcccccgga aggatggcca gctcggagga gggacaccac ctgaggaaag aattttcagg    673140
     tctgcaaaga gtgaccgcct tccagaggag agcggatcga ccactgtgtc agcgccctaa    673200
     aagtcaacat ctgagtcctc agcttctgac cccggggtca ggtaggtcgg atgtgacttc    673260
     gtttccggtg agaggggagc ggccctgacg agggcgctga gtcctcagct tctgaccccg    673320
     gggtcaggta ggtcggatgt gacttcgttt ccggtgagag gggagcggcc ctgacgaggg    673380
     cactgagtcc tcagcttctg accccggggt caggtaggtc ggatgtgact tcgtttccgg    673440
     tgagagggga gcggccctga cgagggcgct gagtcctcag cttctgaccc cggggtcagg    673500
     taggtcggat gtgacttcgt ttccggtgag aggggagcgg ccctgacgag ggcgtccctc    673560
     ttttgactca gcccattact ctaggatgct agtgggttga gccttcgttt tccggtaggc    673620
     gccttcgtat cttggtttgg gtgggaagtg gtcctgacca ggaccctccc ttgacttagc    673680
     tcaagaccca ggacgctgga gagctaagcc ttggtttctg gcagacctct ctctctctct    673740
     tctatcttcc atccttcttt tggaaatctc cgggaaaggg aaaaaaaaaa aacctgttat    673800
     aaactctgtg taaatggtgt gtgaatgtgg gaggacaaag gcttgcgttt gtcttccagt    673860
     ttgtagctcc atggcgaaag ctacggagtt tgagtgggcc ctcacctgcc gttctgtggt    673920
     gacctcataa ggcttaaggc agcatcgggc atagctcgat ccgagctggg ggtttatact    673980
     ggcctgccaa tgctaagagg aacccaagtc ccctcacggg gagtggccag gcaggcatct    674040
     aaatgatccc atcacgggac ccccttccct tgtctgtcta atagagaagg taaaacagga    674100
     aaactgtcat cattgtttac atgccctagg gtcaattgtt tgttttatgt ttattgtttt    674160
     gttcggtgtc tattgtcttg tttaatagtt gtcaaggtgt tacatgtcag gacatcgata    674220
     tagtccacga ggtctgggta aaaatttctt caaggtcctt agtgctgatt ttttgtcaca    674280
     agaggttaaa tttctaatca atcatttaga ctggccacca cagtcttgtc ttttctgtca    674340
     taaacaagta agctgttgtt acggaaaaga gtgtggagaa cattcacctg attggaattt    674400
     ctggcaccat gaaggttgcg ggtatttaga ttgtcatacc ccacgtccta gtgattggtc    674460
     cccttctaaa ctgaactggt ggtgggttca aaacagccac cctgcagacc ttcttgctca    674520
     cctcttctgt cattctgtaa cttttcctgc gcccttaaat aggaccttgt gtaaagaaac    674580
     ctacgcccgt catgctttac ttcgtttaga ctcctattct gttccactgt ggctactttc    674640
     tcatcttaag gacgatccga gtggtccttt tccccctcgt ccctgcccct taccccgcac    674700
     atctcgtttt ccagtgcaac agcaagttca gcgtctccaa gacttcgctc tgctctcact    674760
     ccttgaaccc ttaaaggaaa aagctgaatt tgaactgttt gcctttgaat cgtggagaca    674820
     taaaaaacac ttaggatata ggtctagaag aagaagaaga gggagaacgc ctagatcgaa    674880
     ctggcccagg agacctcagg ctggcctcca atccccctcc ctcaatctta aagctacagc    674940
     aatgtggcaa gtggtattag ctgttgtggt ttttctgttc tttctgatca tgttaattct    675000
     gtttttccga cactccagcc ccccagggaa agagtttctc tgccggtgtt ggatctgata    675060
     tctctgctca agactttgct aaattgtctt taaataataa taataataat aaacgggaaa    675120
     cacctcctcc tggccccgta agggttggag ccctctccag tgtatgctgg aaaatttttc    675180
     tcttggtttc tcagaggact atggagtccg ccttagaaaa ggcaaggtct ggacactctg    675240
     tgaactagaa tgaccaaagt ttggagtcgg atggccctgt gaagggtcat taaatcctac    675300
     cattgttcaa gccgtgtgat gggttgttac cggaactccc ggccaccctg atcagtttcc    675360
     ctatatcgat caatggctaa gtttgattag gaatcctcct ccatggctct gttcatgtgc    675420
     cattcgcaat tccacctcca aagtcctcct gagccaggcc gcgtttttgc ctcaaccctc    675480
     cgccagttcg gctccccctg ttttgcctcc ctctgaagaa gaggagagtc ttcctcaccc    675540
     agttccaccg ccttaccacc agcctgctcc cttggcgcca tcccgtgtct cttcgactgc    675600
     gtcccctgtg ggctgtgacc gcccattgcc ttccgctacg accgcatggg aggaagcagc    675660
     ccctctactc ccactgagag aggcacgagt cccgcggctg atgagcgctc agcccccttc    675720
     ctggtttatg tccctttttc tacttctgac ttgtataatt ggaaaaccca taatcctccc    675780
     ttctctgaaa agcctcaggc tttgacctca ctgatggagt ccgtgctccg gacccatcga    675840
     cccacctggg atgactgcca acagctcctt ttaactctgt ttacctctga ggagagggag    675900
     catatccgaa gagaggccag aaagcacttc cttgcatcag ccggtgggcc ggaggaggaa    675960
     gctagagacc tcctggagga ggaggaagct agagacctcc tggaggagga ggaagctaga    676020
     gacctcctgg aggaggagga ggtctttccc tccacctggc ctaactgggg cccagattcc    676080
     tcaggtggaa ggagggcttt ggacgatttt caccagtatc tcctcgcggg tattaaagga    676140
     gccgcttgga agcccataaa cttgtctgag acaactgaag ttgtccgggg gcctgatgag    676200
     tcaccagggg tgtttttaga acctctccag gaggcttatc gaattcacac accttttgac    676260
     ctggcgactc ccgagaatag ccgtgctctt aatttggcat ttgtggctca ggcagcccca    676320
     gatattagga gaaaactcca aaaactggaa ggatttactg ggatgaatat cagtcagctt    676380
     ttagaaatag cccagaaggt ttttgaaaac cgagaatttg aaaaacaaaa acaagcgaca    676440
     caggcagctg aaaaagctgc cgataaagca tataaaagac aagcaaaaat cttggtaaca    676500
     gctatccaag agggcagaaa ggaaaggcct gcattccaga aaaatggcca aggaacctcg    676560
     ggttcccacc agaaaagtga aagaggtgaa caggcccctc taggaaaaca ccaatgtgcc    676620
     tattgcaaac agactgggca ttggaaaaag gagtgcccgt tactgccaaa agaaaaatct    676680
     gaaaacaaaa aggttctcac cctggccgca acagaggagc ctgatgattg atggggccag    676740
     ggctcccttg ctctcggccc ccaggagccc atggtaactg ctacagtggg gggccagcct    676800
     gtatgtttcc tagtagacac cggggcagag cactccgtac tggagactcc cctgggcagt    676860
     gtctcaaata aaaaaattgc tgtacaaggg gtaactggag ctattcaaga atatcctgtc    676920
     acacacacct gagaagtaaa cttgggacag aaaagagtga cacactcttt cctagtagtt    676980
     ccagagtgtc ctattcctct ccttggacgg gacctactcc ataagttaca ggcctcaatc    677040
     tccttttcag ctcagcaggc tcatctcaca ctaggaaatg caacttcccc cactgcccaa    677100
     ctcttgctaa ctacccctct gtcagaagaa taccttctgg tttcaccgtc gtaatcaccg    677160
     gaggagaata ctaatactct tttgttagat ctacagacac ttttccccca agtttcggcc    677220
     gagtcaaacc ctcccggact ggctaaacac catccgccag tagtcataga actcttggcc    677280
     actgccatac cggtccaggc aaagcaatac cccacaagtc agcaggctag agaggggatt    677340
     aatccccaca ttcaacgact gttacaagct ggcatactta ccccatgcca gtcggcctgg    677400
     aacacgccat ttttgccggt ccagaaacct ggaacaaatg attactggcc agtacaagac    677460
     ttaagggacg ttaataaatg gactgttgct gtccacccaa ccattcctaa tccctatact    677520
     ctactcagcc tgctcccacc agaacataca gtatacactg tccttgacct gaaagatgct    677580
     ttctttgcta tccctctggc ccccaaaatc cagccgattt ttgctttcaa atggacagat    677640
     ccaagatcag gagacactac ccaactgact tggactcagt tacctcaggg ttttaaaaat    677700
     tcccctaccc tttttggaga ggctcttcag caagatctta tacccttccg agccagtcac    677760
     cttaactgta ctcttctcca atatgtagat gacattttaa taacaactga aactatggat    677820
     ggttgtctac aacacacaag agacctgctc tacctccttc aggagctcgg gtatggagtc    677880
     tcagccaaaa aggcccagct ttgtcttccc agagtgtcct acctggggta caagataaac    677940
     aaaggaaaaa aggcactcac tagtgcccgg aaagaagcca tcctgcgaat ccccactccc    678000
     gccaccaaga gacaggtaca tgaattccta ggagccctgg gatattgtcg tctttggata    678060
     tcggggtttg cagagattac aaagcctttg tatactgcta caaaaggtaa tggcccactg    678120
     atttggacag acaccgagga acaggctttt cagaatctga aaaaggcttt aactgcagcc    678180
     caggctttag ccctcccaaa tatctcaaag ccttttcatc tgtttgtcca tgagagccag    678240
     ggagttgcta agagagtgct tactcagact ttaggaactt ggagaggccc agtggcttat    678300
     ttatctaaga aggtggatcc tgtggcctca ggatggccaa gttgtttgcc agccatagtg    678360
     gctacagcaa gcctagtcca agaaactgat aaattaactc taggccagaa tttaaccctt    678420
     acagttcctc atgctgtaga gacttgacta cgaagtgctt caggtaaatg gatgtcaaat    678480
     gctcgtattt tacaatatca gagtttactg ttggatcagc cttgtttgac tttctctccc    678540
     acaaggtgtt taaatcctgc tactttactc ccagatccag attctaatac tcctgtccat    678600
     gactgtcagg agctgttaga agctaccgaa actggcaggc cagatcttca agatgtgctc    678660
     ctgagaaagg cggacgccac cgtgttcacc gacggcagca gttttctcga acaggggata    678720
     cgcaaagctg gtgcagctgt taccacagag acagatgtgt tgtgggttca ggctttaccg    678780
     gcaagcacct cagcacaaaa agctgaattg attgccctta ctcaagctct ccgatgggtt    678840
     aaagataaac gtattaacat ttatactgac agcaggtatg ctttcgctac tgtgcatgta    678900
     catggagcca tctactaaga acgcgggttg ctcaggtcag aaggaaaaat aattaagaac    678960
     aaagaggaaa ttttagccct gcttgaagct gtgtggctcc ctcaacaagt ggctgtaatc    679020
     cattgtaaag gacatcaaaa agaaaacacg gctgttgccc gcggtaaccc aaaagcggtt    679080
     tcagcagctc gggaagtggc gctgtcttca gctccgtcca taaacctgct accggcagtt    679140
     tcttttccac agccagatct gcctgacaac cccgcgtact caacagaaga agaaaaactg    679200
     gctgcaaacc ttagagcaaa taaaaatcaa aaaggttggt ggattcttcc tgactctaga    679260
     atcttcgtac ccccagctct tggagaaact ttagtcagtc acctacattc taccacccat    679320
     ttggtgggac aaaattagct cagctcctcc ggagctgttt taagatccct catctacaaa    679380
     gcctaacaga tcaagcagtt ctctggtgca cagcctgcgc ccaggtaaat gccaagcaag    679440
     gtcctaaacc cagcccaggt caccgtctcc gaagaaactc gccaggagaa aagtgggaaa    679500
     ttgactttac agaggtaaag ccacaccggg ctgtgtacaa gtacctttta gtatcagtag    679560
     acaccttctc cggatggact gaggcatttg ctaccaagaa cgagacagct aacacagtag    679620
     ttaagttctt actcaatgaa atcatcccct ggtacgggtt gcctgctgcc atagggtctg    679680
     ataatggacc tgccttcacc tcgtccatag ctcaatcagt cagtaaggca ttaaacattc    679740
     agtggaagct ccattgtgcc tatcaacccc agagctctgg acaggtggaa tgcatgaacc    679800
     ataccctaaa aaatactctt acaaaattaa ttctagacac cagtgaaaat tgggtaaaac    679860
     tccttccttt agccctactt agagtaaggt gcacccctta tcaggctggg ttctcacctt    679920
     ttgaaattat atatgggcgg gcaccaccta tcttgcctaa gctaaaagat gcccattttg    679980
     cagaaatatc acaagctaat ttattacagt acctgcagtc tctccaacag gtacaagaga    680040
     tcattctgcc acttgtttga gaagcccatc ccagtccagt tcctgaccag atggggccct    680100
     gccattcatt ccagctcagt gacctggtgt ttgttaaaaa gttccagaga gagggcctaa    680160
     ctcctgcttg gaagggacct cacaccgtca tcctcacgac gccaacagct ctgaaggtgg    680220
     acggaattcc tgcttggatt catcactccc acatcaaaaa ggccaacaaa gcccaaacag    680280
     aaacatgggt ccccaagcct ggatcaggac ccttaaaact acacctaagt cgggtgaaac    680340
     tgttaaatta actcttttta tttgcttctt ttgtttatcc ttgcctgtaa tgtcttctgt    680400
     gccttcctac tccttccccc tcacctctct cacaacagga cgtgttttcg ccaatactac    680460
     ttggaaggct ggtacctcca aggaagtcac ctttgcagtc gacttgtgta tactgttccc    680520
     ggagccggct ggtacccacg aagatcatcg cgacctgcca gtcatgggag caggaggtgt    680580
     cgaccttgca gcaggatttg gacactctgg gagccaagcc agatgtggaa gctccaaagg    680640
     tgctgaaaaa ggactccaaa aggttgactt ttacctctgt cctgaaaatc accctgatgc    680700
     tagctgttga gatacctacc agttcgtccg cccggattgg gcatgtgtaa ctttagccac    680760
     ttactctggg ggatcaacta gatctccaac tctgtcaata agtcgtgctt ctcgtcccaa    680820
     atcgtgttct aaaaataatt gtaaccctct taacatcatt gtccatgaac ctaattcagc    680880
     tcaatggtac tatggtatgt catggggatt aagactttat gtcccagggt tcgatgttgg    680940
     aactatgttc accatccaaa agaaaatttt ggtcccctgg agcccaccca agccaatcgg    681000
     acctttaact gatctaggtg accctatgtt ccaaaatcac cctgacaaag ttaatttaac    681060
     tgtgccccca ccattctcag ttcctaagac ccagctacaa agacatcaac tccaacccag    681120
     tctgatgtct atacttggtg gagtacatca ttttcttaac ctcagccagc ctacactagc    681180
     ccaagattgt tggctatgtt taaaagcaaa acccccatat tatataggat taggagtaga    681240
     agtcgcactt aaaggtagtc ctttatcctg tcatgcacaa cctcatgctt tcacattagg    681300
     agatgtgtct ggaagtgctt cttgtctaat tagtactgga tatgacttat ctatttctcc    681360
     ttttcaggct atctgtaatc agtctctgct tactcccatg agcatctcag tctcttacca    681420
     agcacctaac aatacctggt tggcctgcac ctcaggtctc actcgctgcc ttaatggaac    681480
     taaatcagaa cccctcttgt gtgttctagt tcatgtcctt ccccaggtat acgagtacag    681540
     tggatcagaa ggacaactcc tcattgctcc cccggaatta catcccaggc tacgcagagc    681600
     tgccccacta ctggtttctc ttttagccgg tcttagcata gctgggtcag cagctattag    681660
     tacagctgcc ctagttcgag gagaaactgg actaatgtct ttgtcccaac aggtagatgc    681720
     tgatttaaat aatcttcagt ctgccataga tatactacat tcccaggtag agtctctagc    681780
     tgaagtagta cttcaaaact gccgaggctt agatctgctg ttcctctctc aaggaggatt    681840
     atgcacagct ctaggagaaa gctgttgctt ttacgccaat caatctggag tcataaaaga    681900
     tacactccaa aaagttcaaa aaaatctaga taggagccaa caagaacgag aaaataacac    681960
     agcctggtat caaagcatgt tcaattggaa cccctggtta actactttaa tcactggttt    682020
     agctggaccc atcatcatca tattattgag tttaattttt ggcccttgta tattaaattg    682080
     gttccttgat tttgtaaaac aacgtatagc gtctgtcaaa cttatgtatc taagaactca    682140
     atataacccc cttgttgtaa ctgaagaatc aacgacttga ttcccctaaa acacaagtgg    682200
     ggaaatgaaa tgcctaacgg tgtttttact ttaactcgtt actttgaatt ttgtcctgct    682260
     tgtctcttta atcacctaac cttgcttctc atgtaaataa gactctctct agctaggaaa    682320
     gccagacaaa ctccaattga ccccttagtt tacaagacac taagggctcc tcacccaacc    682380
     ccctttcgtg aggagttggc ctgggtaaac agatcctcag catttcaaag gagcccaatt    682440
     aactgataag gtacccacac caacaatgta tgaagttccc aggaattttc tccaagagat    682500
     aacaacatga aaccttgagt ttgtgtccgg catagaccct atatctaatt ataataaaag    682560
     atttagaacc ttgcacctgg taccgttgct cttcttgtaa ccatttgtct tttaagttgt    682620
     ttatcactct gtaactattt tgattatttt gattcttgca tgtttttact tctgtaaaat    682680
     tattacattt gagtccctct ccccttccta aacctaggta taaaatttac tcgagcccct    682740
     tcctcgtggc cgagagaatt ttgagcatga gctgtctctt tggcagccgg cttaataaag    682800
     gactcttaat tcgtctcaaa gtgtggcgtt ttcttaactc acctgggtac aacagtgcag    682860
     ctggtggagt ctgggggagg cttggtagag cctggggggt ccctgagact ctcctgtgca    682920
     gcctctggat tcaccttcag taacagtgac atgaactggg tccgccaggc tccaggaaag    682980
     gggctggagt gggtatcggg tgttagttgg aatggcagta ggacgcacta tgcagactct    683040
     gtgaagggcc gattcatcat ctccagagac aattccagga acttcctgta tcagcaaatg    683100
     aacagcctga ggcccgagga catggctgtg tattactgtg tgagaaacac tgtgagagga    683160
     cggaagtgtg agcccagaca caaacctcct gcaggaacgt tgggggaaat cagctgcagg    683220
     gggcgctcaa gacccactca tcagagtcaa ccccagagca ggtgcacatg gaggctgggg    683280
     tttgtttcct gtcaggattt gggacttcct ctgcttctga cagtttctct agggaaactc    683340
     tttaatttta gatttctgtg cccaccaatg tcatctctac atttttttaa tcattgtaat    683400
     atgataggag gacttattct cacatgcaca atatgtatat tgccacctac gggaatgaaa    683460
     actcctcaac catggtcacc agcatcagag tcgtgaggaa gctcaggggt gcctggtgag    683520
     tcttctccag tcagactcag gacagtaacc tcaaggggat tcccttgtga gaactcacac    683580
     actttcatga gaacagcacc aggagttggt tctaaaccat tcatgaaaga cccactccat    683640
     gacccagtca cctcccacca ggtcacccct tcacaattga ggattataat acaacatgag    683700
     atttggggca ggacacaaat ccaaaccata tcagatacac attgtgaaat gctcatgatg    683760
     gtcagggtaa tttgtatttc tatcacctca cagcgctacc attttattat ttttttaaat    683820
     taactgcatg agtgagtgtc tgatgagaac acctcagata taccctttca gcaacaatca    683880
     tttttataat acagtattaa ctataggacc attgctgtac gttagatctc cagaactcat    683940
     ccaacctgca caactgaaac tgtacaattt aacaaacatc acccgatttc cctctcctcc    684000
     caggtcctga gaccctctat tctgctctct actttcaaga gcttgaatat tttagatccc    684060
     acatgtaaat gagatcatgc agcatttgtc tttctgcatc tggcttattc cactcagcat    684120
     catgtcctcc aggcccatcc gtgttgttgc aaatgtcaga atttccctct tttcaaagcc    684180
     aaataaaata cagatttatg tatacacatt ttctttatac attcatccat ttacagtcat    684240
     taaattattt tgcaaatcta cactattatt aataatcttc aatgaacacg tctttggcaa    684300
     agtaatttta tttcctttgc atatataata agaaatggga tcaccagatt atatgatagc    684360
     tttattttca acttatcgag taaccaatcc taccacagtg tattcccttt ccctcacatt    684420
     cttgccaata tttgtcatct attacatttc tgataataac catattaact agtgtgaggt    684480
     gatatcgcat tgtgcttttc atttgaattc ctctgataat taggaatgtt gagaaccttt    684540
     tcgttttctg tttgccatgc atgtatcttc tgaaaaaagt tatccaggtt tttgcacttt    684600
     tttatcagtt catttgttat ttgctattga ggtatatggg ttatttatac atttagatag    684660
     accttcttgt cggatacata attacacata gtttttcctg tgctttgttg ttaaattcaa    684720
     agaaatcagt tccgaattaa tggcaggaat tttttttgct ccatgtgatt tatgagttta    684780
     tggcttcagg tattatgtcc aatattagct gattttttta tatggagtta gagaaggcct    684840
     aattttattt cttttgaata tgaatgccag ttttacacca ttattgaaaa gactgtcctt    684900
     tctctactgt gtgctcttgg cacacaaaat caggagagac ataatgacaa aagaaaatat    684960
     cagatgaata ttcctgatga acatagacct gaaagtcctc aacaaaatac tatcaaatag    685020
     aatccagaag cactttaaaa tgtaatacat catggtcaag tgggctttac ccctgggatg    685080
     caaggctcgt tcaatatcca cagtaactgt gattcacaat gtaaacagaa taaaagcaaa    685140
     aaccatatga ttatgtcaat agatactgag aaagcctctg gtaggttcta acatccactc    685200
     atgataaaaa ccctcaacag actagacatc aaagaaacgt acctcagaat aatagccatc    685260
     tacagcaaac ccacagtcaa caccatacta aatgagccaa ttaaaacact tgtctttata    685320
     aattacccag tctgaggagt ttctttatag tgtgagaatt gactaataca gcatccaaat    685380
     aggaaaggaa gtcaatccgt ccaccttcag cgatgatata attctatacc tagaaaatcc    685440
     taaaaagtct gccaaaataa ttctagaata aacaacttta gtaaagtgtc aggatacaaa    685500
     atcaatggac aaaaattacc agcatttcta taagccaacc acatccaagc tgagagtata    685560
     atcaagaaca aaatcctatc caacttacag tatccacaga gaaaatgaaa tgcctgggaa    685620
     cacagataac aaacaaggtg aaagatcact acgaggagaa ctataaagca cagcagaaat    685680
     aaataataaa tgatacaaat aaatgggaaa catttcatgc tcatggattg gaagaatcaa    685740
     tattgtaaac attgtcataa tgcccaaagg aattcacaga ttcaatgcta tttacataaa    685800
     actatcatca tcattcttca cagaatcaga aaaaaatcta ttctaaaatt tatatggaac    685860
     caaaaaagac cctgaatagc agaagcaatc ctaagcaaaa agaacaatgc cagaggcatc    685920
     atgatactga ccttaaacta caccataatg tcttgctaac aaaagcagct tggactggta    685980
     cgatagcaga cctgcagaaa aaaaaaaata gatcaaaatt gaaaacagaa ataaagttgc    686040
     acacctgcaa ccttttgatc tttgacaagg ctgacaaaag caattggaaa aaaaaaatct    686100
     ctgtattcag taaatggttc tgggataact ggattctggc aggtatgcag aatgcaagag    686160
     tgaaggagcc tgtgcagctt ctacctagat ttcagatgtg tagagagccc tgcatgccca    686220
     ggaagaagcc tgctgcagga gtggatccac cacagaaagc ctctactaga gcagtgccaa    686280
     agatggggaa agatggagtt ggagccccca ttcagagtcc ccactagggc gcttcatagt    686340
     aaagttgtgg gaatgtggcc acaaccctca agaccctaga atggtagagc tacaggcaac    686400
     ttgcaccctc agcctagaaa agcttccagc acttgactct cacttgtgaa ggcagccccg    686460
     tggtctgtgc ctagcaaacc tatagggatt ggctgcctga ggttttgggg acccacccct    686520
     tgttccagtg tgccctggtt gaagtacata aagtcaagag agattatttt gtagtgttag    686580
     gatttaatat ctgcagtgct gggttttata tgtgtgtgag gcctgttggt tgttccactg    686640
     gccaatttat ccctttagga ttggaattat ttacccaatg cctgcaccat cattgcacct    686700
     tggaagtaaa caactttcat ttttaaactt gcaagctcac agctggaggg acattgcctt    686760
     aagactcaga tgagactttg gacattttag ttggaggttg atcaacttaa cattttggga    686820
     actattgaaa aaaggtgatt atattttgca gtgtaagaag aatatgagaa ctttgggtct    686880
     gagagtgcaa tgatatcatt taggggtttt cccctccaaa tgtcatggtg aaatgtgacc    686940
     cacaatgttg gaggtggggc caactgggtg gttttgggtc atgaggagat ttttcaggac    687000
     tggcttggca tccaccccat ggtaattagt gaattcttgc tgtattagct actgtgagat    687060
     ttgattgttc aaaaaagtct ggcaaccctc ctaccctctc atgtcccccc tcctcaccat    687120
     atgacacagc ctcctccccc tttgccttcc accatgactg taagcttcct gaggccctcg    687180
     caagaagcag atgcttgtgc catgcttctc acacagcctg cagatctgta agccaaataa    687240
     gcctcttgtc tttgtaaatc acttggcctc aggtattaat ctataccaat gtaaaataga    687300
     ctaatacact gcccaaagca atatacagag tccatgcaat ttatatcaag taactaatat    687360
     aattgattac atatttttaa aaatcctaaa attcatatag aaccaaaaaa gggtctgaaa    687420
     agcaaaagca atcttgagca aaaagaacaa agctggaagt accgcattcc ctgacttcaa    687480
     attacacaac acaaagataa caagaaaggc agcgtggtaa tggtagaaca aaaatcaaga    687540
     gcccagacat acagccaaat atctacaacc aacggttctt tgacaaaact gacaaaaata    687600
     tacactggag aaacaaccct ctattcaata agtagtgctg ggagaattag atagccttat    687660
     gcagaagaat aaaatgagac ttctgtatca tcatagacac aaattaactg tgaatatgga    687720
     ttcaatgttt aaatttataa actcataaaa attcctgaag aaaatctaaa aaaaatcctc    687780
     tagacatttg cctaagcaaa taaaatatga ctaagactga cttcaaaagc aaatgcaatg    687840
     aaaacaaaat tagacaaaca ggattaattg aaacacagat tgtctgcata gcaaaagaaa    687900
     taaccaacat ggtgaacaga caacctgtga acagtaaaaa aaaatttgca atctattcat    687960
     caaagggcta atacatagaa tctacaagga actcaaataa gtcaacaaga aataacaaat    688020
     aacttcatta aagagtgagc aaaaaccaga catttctcaa aagaagacac agacgtggcc    688080
     aaaacaaata aaacatactc agcatcacca atcatctgat aaatgtaaat tacaaacaac    688140
     atgatatagc atcttccacc agtcacaatg gctgttatta caaataaaaa acagcaggtg    688200
     tttggagaga agcataggaa aaataatgct ttttatatgc ttggtgaaaa tataaattag    688260
     tacaacctct atgaaaaaca atacagaagt ttctcaaaga actaatatta gaattaccgt    688320
     ttgtcccagt aatccctccc agtgggtata ttcctcccaa aaggaaattt ttatatcaga    688380
     aaaagtcacc tgcattccta gtgtctttca cagcaacgtg aatagagttg taggacatta    688440
     gcctaaataa aataactcaa aaagatcttc tcttttataa ttggaaagta aacaatgggt    688500
     acacatgtac atacataaaa aacaaaagac actgggaact gcaaaagagg gcaaggtgag    688560
     aggaatgtga gggttaaaaa atcacctact ggctccaatg ttcactagtg ggcgatagtt    688620
     acaccagaag cccaaacctc acctttatga aatatattca tgttacaaaa ttgcacatgt    688680
     acaccctgat tctactgaaa aaaacagaaa taaaacatga acagaattgt tagactgcta    688740
     gctagatttg tcaacaaaga aaaatgcaaa aggagcaatt ctctgttttg gaaccctttc    688800
     tcattttctg gtacaattag aaattgtaga ctaatcccta tattctcttc tgaagcccta    688860
     gcattagcca tttctcaaag acatgctggt tccttttttt tttttttttt tttgagacag    688920
     ggtccagact cactgcaact tccacctcct gggttcaagc gattctcctg tctcagcctc    688980
     ccaagtagct gggattacag gcatgcgcca ctgtgcctgg ctaatttttg tatttttagt    689040
     agagacaggg tttcgctatg tgggccaggc tggtccaaac tcctgacctc aaaagatctg    689100
     cccacctcag cctcctaaag tgctggaatt acaggtgtta gccactgcac ctggtcactg    689160
     gtttctttta tagaagaata gtattaaaaa ctcaaatctt gattctgggt gtatttttta    689220
     ttaatgtgca ctcatttctt gtagaaattc tcaggtaaaa aatctaggaa gtatgtgttg    689280
     tgtattaacc catatataca cagacatcta aactattttt atctaatata tgtacctata    689340
     ttatgctaaa cttgcaaata aaatgacatg tccaccctaa gttaatacca catggatgtt    689400
     tatcgcattc cttctatgcc tgacactaac ctcccactcc aaccacaagg aaccccattt    689460
     ccccatacgc cgtccattct ctttgtagtc caattccaag attcctgtgg agtgtaacaa    689520
     gatgtgtaag ttgtgctctt ttgtggaaca tcgtcaattg gggtacaatg ctgatgtgaa    689580
     gtttcttttt tctttaatct tattgcctac accatttctg aatctactta gtacctcttt    689640
     tgaattcata catttgtaat ggaattagac attttctatg ttatctgcat tccatcctgg    689700
     aattcctaat cacctatttt taaaaaaaat tcttttgaag taagattgta ttagtcaggg    689760
     ttccctagag gggcagaaca aacaggatat atatacgcac acaggataca cacacacaca    689820
     cacacacaca cacacacaca gagtttatta aggagtatta actcgtatga tcacaaggtc    689880
     ccacaatagg ccatttgcaa gccgaggagc atggaagcca gtccgagtcc caaagctgaa    689940
     gaacttggag tcccatgttc gagacaagga agcatccagc acgggagaaa gatgtaggct    690000
     ggaaggctaa gccagtctaa tcgtttcatg ttcttctgtt gttgttgttt ttttttccct    690060
     ctggccacac tggcagctaa tgagattgta ccacccagat taagagtgtg tctccctttt    690120
     tgagcccact ggctcaaaag ttaatctcct ttggcaacac cctcacagac ccacccggga    690180
     tcaatacttt gcacccttca atctaatcaa gttgacactc agtatttagc attacaagtc    690240
     caccccgtca acttgaaacc atacacatct cctgagatta tacataatct tcaaacaaag    690300
     acaataataa agtcataatt acacctaata taatacaact ttattcgttc aactggaaat    690360
     gcaccaatcc ccaatccaaa tgctattaca tatggtccac tccctcaagg acacagccag    690420
     gatcatccta tgacttggtg tctagaagac tcatgcactt ttaattttac ctccaatatt    690480
     agactccact ttatcacatg cagacactgc agaatatttt aagacatcga tgtcttatgc    690540
     tgaaaaattc aacagtacat aaaattgcct tcccaccaca gccagtggtt actactgaat    690600
     aaggatctgc catcccctag ggaggtgcta tagtggatgg tgtgcactgt tagtagctat    690660
     ggaaattgat ccattgtggc aaatggcagc atctttcttt tataacatta aaccatatta    690720
     tattgtatac acataccaca ttgtctttat tcatttgtct gtatgctgac catcagatag    690780
     tttccatatc ttcgctgtta ttaacagtgt ttcaataaac ataggatgca gatatgttta    690840
     caaggtggta atttcatcta cttgggtata ctcccagaaa caggatttct ggtcatataa    690900
     tatctccagt tttaattaat ttaggacgct ttatgttgct ttccataatt gtggaaatgt    690960
     agaatggtat agccattatg aaaaacaatt ttggttttga gttatgatcc ataaacagca    691020
     tttgcttatg gctctcaatg agagtttcta ctaaatatga tggaaggcca ggaaggattc    691080
     tcaccttaaa gcttggatgg attatgaatt tacctcttat ttcctaaaag caaaagtttg    691140
     gaagcataca tgatggaggc tgttggcagg tttcaggatg atctcataaa aggagaatct    691200
     gctggataaa aatgttgggt tttcagttat agacacacct acactcaata tgaaatgatg    691260
     aagtcaactg aggatctaga acatgttcac ttgaagcaac agcacattct caaaggaact    691320
     tattgctcca tcacaagggc gatgaacttc tgaaaccagt aaagtgtgag tgcacagtaa    691380
     gtgataacgt tatcacgttt acatgaagtt tgtttaatgt gccaaaggct ggtacagatt    691440
     aatcttacac aaatatatga aacatatttt tccatacata gatatctaga ggataatcct    691500
     cttagcaaaa cttcttcagg ttacagaaat ctgtgtaagt tgtagatctc atggggaact    691560
     gtctctttat aaatactggt ctgtttttca agtcaggaaa aattatctct gttggagtag    691620
     gcttccaatt gtgtagactt ctgaattgct tcttgagttg taacaaatga accaaaaaat    691680
     ttgactaatt ggagttttgt ggctgtctta ataaaaattt cttataagct ttctttcaat    691740
     tatttgagac cagttttcct gattttattt ttctccaata gataaatttt cacaagatct    691800
     caggaaacca cctctctggc tctttagttt aaaagactag cttcttagga aaagaagctt    691860
     ctttctaaca ttgaagagct catttattct gcaaaccatg aagtttgagt ctcattgtca    691920
     agaatcatga aaatttttac tccaatatac tgaaagtcat gactcttgaa ttcaatattt    691980
     attggcattc acaaaagtga ttcattctca gatgtgtcct atgttgctgg cccaacatat    692040
     cagtgaactg agtatcctcc cttagctctc tctgtctgta gcactgagtc tacaatcttc    692100
     agagtcatcc atgaagggga gtcctgaggt tcatgggatt cttgtaaaca ttcaggtcaa    692160
     cagaagaaat aaacaggatt gagggctgcc agccatttcc acgccattag gaataattac    692220
     catctaagta taaaggtcta catcatacag aataccccac aggataggct gatgcaaaaa    692280
     attccaaccc cacagaggct ccacagcaac ccttacagtt ctttcaggga agaaataatc    692340
     tccaagttaa atgagtcagt aaagctgctc tgagctacag gaaaaattgg atttggccca    692400
     ggtttgtctg agttcaacgt gattattata ctcagctcct gctccaatct ggactgagta    692460
     cggggaattt aaatgagcct ggctgtgtgg tttgttatat gaaaatctga actatataaa    692520
     cataaatggc atgtctggac tagcatgagg gtgagagatc ctgggaactc caccccccat    692580
     actcttatcg ccctttcctc caggaacctc caggttctca gggtgagaat ccacatagat    692640
     cccttcatgg ctctatttct aggaaaccaa atctctaata aatacaccca gactttcttc    692700
     agacacagaa accatgggaa aggcatcttt agatcctcat ctatgtgggg aaaggtaatc    692760
     catccccatt acaagcccat actagcagcc ttcctttatc atgaaaatgg gtaaaattag    692820
     ccaatagatg taaatttaaa gaaaatttcc tgtgatgctc cagccagaaa agagcaaaaa    692880
     tcagcttcac ttctggagac ttcccgtata ggtcacagcc cagagaaaga agatcaccac    692940
     agcattaaaa ttcagctgta agaacatgta atgcttccgt gttccacaca ttatgtctca    693000
     ccagtttagt caatatggat taaatacgag agtgtggcaa tgcacaaact ctatctgagg    693060
     aggaaatagg agaaaaatgt taaggaaaat agaagaattc gaagcctcta ataccaggaa    693120
     cttcagcaca aagaaaatga tttcaccctt attggcctca aatttatttt tcctgtggca    693180
     tctgcagggt tccaaagtga gaaaaattaa ttcactgtgc atgcacttcc gaagtgtcca    693240
     cttgcattct gatatcttta cttctatttg cagaaagtag acacatattc agccttagtg    693300
     ccagtgtagg gagtgctttc cgtgacatgg ataccagaaa aatagggtaa acatagggcc    693360
     cattaatgtg aaaatcagac attgtgtgtg tgtgtttgtg tgtgtgtgcg tgtgagttga    693420
     atagtagagt tggagtgggc ttctatccac ctacacctgc aggtattctc aggtgccata    693480
     atcaactgca ggaccctaaa ggaaataaga gtccccccaa acccctgaag agtttttggg    693540
     ttcaccgtgt gtccaatgat tcagtgcctc ttgagctcca ggaaagggct ccccagtgat    693600
     gcatgagatc tttcttgggg tctccctgca gagttcactg ggattcctaa ggccaattca    693660
     gtatttcaaa agatggtgtg agaagcacag gctgtcacta aaggagaatt ctgagccagg    693720
     gcacagccac tttatacttg gctggggaca ctggtaggaa tatactctgt gagatcagac    693780
     aggaacctcc ttgcaggggc agggcagggc tgcagggggc gctcaggaca cacagagcac    693840
     aggcttccgc cccagagcag gtgaaggagg ctggggaggg gttcctctca gggcctggga    693900
     cttcctttaa aaaatctaaa ataagtattt cacaaggact gccgatgttt atataaatat    693960
     cctattcaat tgtgagcatt tatgaaactc gatgttgtaa tgagaaccac ttttacaatg    694020
     ggaatttcaa acttccctag acatcttaat agtaagcagc tggaggtcag gaggagatcc    694080
     tttcttataa ataagtgcaa tttttggaga aacacactca ttcccaaaat agcacattca    694140
     catattaagg tctagaaatg attcgagttg cccctgagac agtcaaatgt gggttctaag    694200
     tgaggtgcgt gtcctggggg agcttgttct ccagtggggg aagctctgtc aacacagagt    694260
     tcagggatgg gtaggggatg cgtggcctct aacaggatta cggcttgaac cctcagcttc    694320
     tacaattgtg tcgtccatgt gtcatgtatt tgctctttct catcctgggt caggaattgg    694380
     gctattaaat agcatccttc atgaatatgc aaataactga ggtgaatata gatatctgtg    694440
     tgccctgaga gcatcaccca aaaaccacac ccctccttgg gagaatcccc tagatcacag    694500
     ctcctcacca tggactggac ctggagcatc cttttcttgg tggcagcagc aacaggtaac    694560
     ggactcccca gtcccagggc tgagagagaa accaggccag tcatgtgaga cttcacccac    694620
     tcctgtgtcc tctccacagg tgcccactcc caggttcagc tggtgcagtc tggagctgag    694680
     gtgaagaagc ctggggcctc agtgaaggtc tcctgcaagg cttctggtta cacctttacc    694740
     agctacggta tcagctgggt gcgacaggcc cctggacaag ggcttgagtg gatgggatgg    694800
     atcagcgctt acaatggtaa cacaaactat gcacagaagc tccagggcag agtcaccatg    694860
     accacagaca catccacgag cacagcctac atggagctga ggagcctgag atctgacgac    694920
     acggccgtgt attactgtgc gagagacaca gtgtgaaaac ccacatcctg agggtttcag    694980
     aaaccccagg gaggaggcag ctgcactgaa tttgaggaga ttacagggct tacaatgttt    695040
     aaagttgttt agaaaatgag ctgagcaatt gaggaatgtg agtaatggaa acatggatgc    695100
     actctatata ggaaatgttt ctttcaacag tcaccctata tgcaaaattc agaatggtaa    695160
     aggcagcaat cagtgaggct gacgcaaata ttcccatgga ggccttgtgc agacatacgt    695220
     tttaaaatca gatagataaa taatttggaa caagattgct ggtaacgtgg ctaagactaa    695280
     atatgattcc taaaaactgg ccaaaatcta ttccaaattg tctctgccac tccttttaca    695340
     taaatgtatt aaaaagtagt tttaagacca cagcaaaatt ggacagaagg tgcagagagt    695400
     tctcatgtgc ccctgcttca ccatgcacag cctttcccac tgtcaccatc ctgccccaga    695460
     gtcatcaata agttacaatg gatgaactta catgggcgga ttggttcttt cctcttctgg    695520
     tggtctcttg gcataccaag cccaaattat cttgaagcac cataggttct actgtaatgc    695580
     ctaaccttgt tttttactct agtttgctac tttaaatttt cccttttttt gtctccttaa    695640
     ttgccagcca tgtttcccat atgaatagac tctccctggc tgggaaagct gggcaaactc    695700
     catttgacct tttgatttat aagacattaa gggctcctta cccaaccccc ttcttcaagg    695760
     aattaacctg tgtaagcaga tcctcagcat ttcaaaggag cccaattaac tgataaggta    695820
     ctggaacaaa caatgtatga agttcccagg atttttctca aagagataac aacataaagc    695880
     cttgagttca tgcccagcat agcatcatat ctaactataa tgaaggattt agagccctgc    695940
     acctggtaac gttgcttttt gtaaccattt gtcttttaaa ttgtttatct ctctgtaacc    696000
     atttgcttct tttgattctt gcatgttttt acttctgtag aattattgta tttgagttcc    696060
     cctccccttc ctaaaccaag atataaaagt taatcaagcc ccttcctcgg ggccgagaga    696120
     attttgagcg ttagccatct cttcggcagc cggcttaaat aaaggactct taatttgtct    696180
     caaagtgtgg cgttttctct aactcgtttg ggtataacac tacaagtggg ttcctgggaa    696240
     tgatgccagt tcagaggaaa agtgggtggg gctattcata tttgggctct tttttagaag    696300
     attcataaaa cagatatttt cctatacgtt ctgtgactcc tgatttacta tcccttccca    696360
     gagggtaagg tccctaagtg ttttgcggta tatccatgtc tatggaaaga aagcaagttc    696420
     tggtgaatcc cataaggaat gtcctttgat gagaagtgga gaccttggtc atgaggcaca    696480
     tcatgtatga ttttctataa ttccgttaga ttcactgtaa ttttgggggg tgtcctgtgg    696540
     aatgggtctt ctgtgtccct gcatgttcag ctatatctgt gtggtgccac ttacacttaa    696600
     tgagatgaga ttcctgctgc ttgtatcagg tcactggtga tctctgaagc tgcttctggt    696660
     ttctgcttta atatgtaaag agcatgtcaa acatcaccca cattcttaca ataataaaag    696720
     cctggaaaat tgattatcaa taatttcttg aatctgttga agaaatgaaa ttgcagggaa    696780
     aaccaggacc cccaaaacta gaaagacatt aaaatagaga taatcagact gatggatcaa    696840
     agttctgtgg caataagata ccaaattata aacaaggcct aaagtcatgg attaagtcac    696900
     tcacccctac acttaaaaaa atgactgtac tgtaacttcc acagggcttt tttgttttct    696960
     gtagcaccta aacaagcact ggttctgagg taagcatatt aaaacatttg cagctcatgg    697020
     aactccaaac gtggtaactg accctctccc acaagccata aatagagctt tgtttggaca    697080
     agagacagat ttcagtaact gtcttctgag aagagactac tgaccatgaa cttgtcctgg    697140
     caatttacag agactgtgca gtgtgtgtct ttctgcctct gcacaaagcc cttttgatga    697200
     acagggcccg attgtcattc atttagttct taagtcctca tccccaaagc aaacactaaa    697260
     tgcatgtaac atgtgtgttt gcttattaga catgacagcc tgcccactat gtgaacatca    697320
     acagatcctt ctatagcctg ctgagtgtgt acacttggcc aatccatttg catgaattca    697380
     tttctcctct ttccctctct tgaagtgcct gctcactgtc tctgtgggag gctttgcttc    697440
     ccagcctgtt aaggtggctg tcctgcagct ttaaccattt ctcagaagta aagtctcctt    697500
     tctaaattta taaattatgt gactgttccg ttgaaagtgc aagctggtgg gaacagttac    697560
     atggtaattc ggtaaattgc tggtggacag gtgtggacag ggatagggta agaactcctg    697620
     ggggctgcac accccacact tcgatggaat ttccctccag aaacttctgg gttctcagga    697680
     tgataatcca aacagattcc ttcatggctc tgtcatcagg agagctactc tctgataaat    697740
     atgctcagaa cttcctccag acagatccta cagagaaaaa atgcttttca agatctctat    697800
     tctatgtgag gggaaggtat tcttttccat cccaggcagt ttcatcttag ccttcctgtg    697860
     tcataaaaag gtcaggttca agacaataat atgtggatgc tgcagccagg aggggggagt    697920
     agaagacgga ggaaaatcag ctgcaccact ggagactcct tgtaaagggc acagtctaga    697980
     gaaaaaacat caagaaaaca ttgaaattca atttccagta catatactgc tcccctgccc    698040
     accacatcac ctcttcacca gtatgatcaa tctggattaa agagaaaagt gtggcaatgc    698100
     acagactctg tccaaggact agaccttagg gaaaccaaag gcagagggag aggaaaagtt    698160
     aaggacagtg aagcggttta aagcctctga gaccaacagc ttcaggacca aggccacggc    698220
     ccctctgtca atggccttag atttacctct cacggggcat ccacagggtt cccaggtgag    698280
     aacaggcaaa aacaaggtga agacactttc caaatctcca gcagtactga gcttgcttta    698340
     gctctgtttg aagaaacaaa acaacaacaa caaacaacaa caaccgcaaa tataaccagg    698400
     attaaggtca gcgttggaag cacttttatt aattttttta aattttttat ttctcagatg    698460
     gggtcttgct ctgtctccca ggctggagtg cagtggtgcg gtctcggctc tgcaacctcc    698520
     acctcctggg ttcaagcaat cctccaccct agcctcccaa gtagctggga ttacaggtgc    698580
     atgccacggc gcccagttaa tttttgtatt ttttagtaga gacagtgttt tgccatgttg    698640
     gtcaggctgg tctcgaactc ctgacctcag gtgatccgcc caaattggcc tcccagagtc    698700
     ctgggattac aagttaattg agttaataaa gctgttctga actatgaaaa acattagttt    698760
     gctcccagat tttcttgatt tcagtttgat tattatattc agctgctgcc ctaatatgtt    698820
     tgagcatgtt actatttaaa tgaccctggc tgtttgattt gttatatata aatctaaact    698880
     aaaacaactt aaaggacagg tttgtactat tattaggatg agatattctg ggtgcccctc    698940
     atttgctcca tgaacctcca catttttaat ttagaattca cataaattcc ttgtgagttg    699000
     tgtcagtcat cgagagacct aatctctaac acctttaccc agaaccttct ccagatagac    699060
     cacccacagg aagaacattt atagtaattt atctacatgg tggaaggtac ttcactgcca    699120
     ttgtagaacc ctcttcccag ccttccttta tgaggcaaat gattgccagt atggtaagat    699180
     tgaacaaaag gatgtgccag tagggtaaga ttgagcaaaa atagtcctga gatgctgcag    699240
     acagggaagg acgtaataga caaatatcag ctttattaga ggagactcct tgtaaaggtc    699300
     atagccaaga aaaagaagac aaaatcactg agattaaatt ctaagaatct tggtccagtt    699360
     caatcaagcc atcaacagcc aggtggcttc agtggccact atgcatatta ccccatgcct    699420
     aaatgttctg gcttgtacaa agagcttctc tgtaaattgc acattgccat agtggaagat    699480
     ggcatgggag attttgtgac aaacacttaa ggtgcaataa tagctatttt tcctttatca    699540
     tgacaatatg tggcatgctt ctagattcat ttcatgccaa agaagttcct cagaagggta    699600
     aggaaatgga gaatcttaca tggtccaatg cagtccccaa gaagggactt tgaatggaca    699660
     gttacactgg ttactacaat gttgtctctg accttctcag gatcgaagat agtcatgagt    699720
     atcaaggtgg agatgggtct aggtgagaga aaaggaagta gtggttcttg agaagaatgg    699780
     tatggccaag ataagcctga actatctctc ctcttccagt tcctgcaact tcatattgcc    699840
     tgattgaggt cacttgccta tggtggtaat gacatcagca atgcaataaa ttctctggtg    699900
     gctttatatc tggtttataa catgggagat gtttcctcaa gagtggtgat gccagtgtgg    699960
     cttctgctct ctggtggtgt tggaatctgc attagtctgt gggtttggag gaaggagatt    700020
     caggccatgg ggaagaatct gacactctca caccctctgg tgccttcggt actggactgg    700080
     ctcctgccct cactatggtg attgcatcag atattggcct ccccaccagt acaacacgtt    700140
     gtaacatggg ctcctttgta cctggcttta atccaagaaa gttgtcgact tgactctttc    700200
     gtgacattgt atagcctggt ttgtcacaat ctttatttct ggaattatca gtgctgccat    700260
     cttggctgtc ttcaagtatg tcatcctcac agtgtgaagc tgtttgacat taacattttt    700320
     gtcaatgttt gggactacct tacatattcc ggctcagttc aaaatgagct atgtaatgta    700380
     gccaggtttc cactgagttc cactttgctt cttggctgtc tattcctgcg caagtttcta    700440
     tgtattttgt atcaggcttc aattccatta tgtttaaaat gttgtctcta aagataaaca    700500
     aagatttttt taaaacaacc aaacatgcag ccatttgacc gagtgtggtc tgcattgtta    700560
     gtctcaccat cttctgccct aacaagcacc gaatctaaca acacaaatat catggaagct    700620
     ttccttggag tagctcctgt aattagaatc attgtattct gccctcctgt cagtggtgga    700680
     acgttctatt ggcacatgtg gaaacctctt agagggacga gtttctttga acacaataaa    700740
     attttaagtt aggaataact tatttgaaag caattcattg aaggttattg ctaagaagaa    700800
     gttagaaaca gtcttttggc agtcatttct tcaagatgta tggcagtgtg ggagggatat    700860
     ataaagtgga ataggaactt gggctagtta aatggaatag cctccaatgt taatctgttt    700920
     accttttaac tgaatgaaaa agcctatagt tgtaagaaaa gaaaaaaaac acaatcctca    700980
     cacattccct tcccatcagc attgaagatg agtgtggcaa tgcagagact ctatataggg    701040
     aggagaacat agggaaatgc aaagacaatg ggagaaaaaa gtcaaggaca gtagagcaac    701100
     tggaagcttc tgacaccaac agcttgatga acagggtcac tcaacccttg gtggacacag    701160
     agtcacttct cttagggaaa ctgcagtatt ctcaggtgac aatatgcaaa catgatgaat    701220
     gtacacttgc tacgtctcca gttgtgtact gttttcctta cttctgtttg gagaaggaag    701280
     tcatatactc aggtctagta tccgtgtaga gggtgctcac catttgcaag atactaggaa    701340
     aaatggcaag ttagaggccc actgttgtaa aaatcaacca gttctgtgtg tgtgtgtgtg    701400
     tgtgtgtgtg tgtgtgtgtg tgtgtgtgtt attgaaaaag aagacatgga gtgggcattg    701460
     atccacaagt atatatacct acaggtattc ttagatgtaa tattcaattg caggagacca    701520
     aacaagataa agcatccccc aaaacccttg aaaatttttg tgctcactat gtgtccactg    701580
     attaaatgta tcctgagctc cagggaaggg ctccccgatg gtttcttaga gttcactggg    701640
     tctcctaagg tcaatgtttt caaaagatgg tgtcagtaac atatggtgtc actgaaggag    701700
     cattttaaac tagggcatga cctcttccta tagccctaga gacactgaga gagaaatcct    701760
     ctgtgagccc agatggaaac ctccatgcag ggcagaggca gtgctgcagg gggcgcccag    701820
     gacccaccca gaacaggctc cagccccaga gctggtgcac aggaggctgc agaggggatt    701880
     tctcccagaa ttgagtgttg ttttattgaa aaccaaaaaa ttataacatg ttaaaattag    701940
     aattttgcaa agactatttc tattatctct atacatttag aaataaacaa atcaacaatt    702000
     cagtgagaac cacatttaaa aggcaaaatt gttacttgtt tgagatattt tcctagtaaa    702060
     aagatgaaga acagggatgg ttttattaaa tcaaaaggta cacatttcag aagacacaga    702120
     gatcccaaga gtaagactgc agacagcaaa gcctagaaag ggcggaggct gtccatgtgc    702180
     catgtcctgt gtgagtcacc ttcttagtgg agaagttctc ccttcacaaa gttttgggca    702240
     tataaaggga aacacgacat taaataagaa cgaggacttg tacctcagca tcccacagtt    702300
     gtgtggttca tgtgagatct attttctctt tctcatgctg gatcaagggt aaggctatga    702360
     agtagtagtt ctcgtgaata tgcaaatcac ctgaggtgaa cactacagat atgtctgtgc    702420
     cctgagagca tcacccaata accacacacc acaaagacac tcgctgagaa gagcaaccct    702480
     aagacacata attgtcagat tcaccaaggt tgaaatgaag gaaaaagttt taagggcaac    702540
     cagagttgtg ccatggcaca gtgttgtgcc atgtcctttc tctgttttct tgcctgttta    702600
     tttatgtcag atgtgccacc tacatggaat aaggtcagaa ttctgcctcc agtaacacat    702660
     caaaggtgac ctttgattgt acttttggtt tatgctccaa aatattgatt ataaaattca    702720
     tcaccgtcat attttatgtc aaaataaatc tgcataatct gaatgtcagt acattttgga    702780
     atctattaaa taactgaaat tgcaagaaat attacccact ccaaaaactg gagagatggg    702840
     catgtccaga atggcagtta gcacttgctt acctggtgca gaaactgcag aagccacaag    702900
     ctgttgaggg cacttacatg gtaaccacta cggacgtctg aaagacaaat gtggactcgg    702960
     taaatgtgac cgttccagag ggtcttatac ttctaaggtt tctggacttt ctctccagaa    703020
     acctccagat tctaaaatat acaatccaaa taaatttccg atgggtcaga attgaagatg    703080
     aaatgaagat gattaattac agagaaatat accaggagcc cacttcaaaa gcttctaaag    703140
     ggaatgactt ttccagaacc ttatcctatg tgaaggaaga caaatctccc attccagatt    703200
     ctcccccatt cttccattat tatatgaatg agtaaagtta gccaaaaggg gtaagacgta    703260
     cataaatagt ccagggaggc cgaaacctca aaagggagta acagccaaaa atgagctttt    703320
     cccctggaga tgctttgtca aggtcacagc ccagaagagg aagcctattg aatctctagg    703380
     tttccattgg aagaacaggc agtgcttacc tgcactgcac aatccattct aactaggatg    703440
     atggctatgg attaaagatg aaagtgtggc aatgcacaga ctccatctga ggagaacaca    703500
     gaaatactaa gacaatggca gagggtgaga caaagacagt agagcaatgt gaagcctctg    703560
     acatcgtgat ttttaagacc aacatcttgt aaatgccatc atagttctca gcttctttta    703620
     ttatgggacc tttgctggtt tcctagctga gaaagtgaaa ataacaacct gcatggactt    703680
     cccaagtctc cacctgtatc ctgtttgctt tagatctcta ggagaagaaa gtcagataac    703740
     tgggcctggt gtcagtgtag gaggcacttc ctaaaagcca catattagga agaagggaaa    703800
     atgtgtgtta ttggaatagt ggatatggag tgggctttca tctgaatgta tctgcacctg    703860
     ctagtattct cagatgcaac attcaactgc aagagcccag tgaagaaaca tggcactcgc    703920
     aaatctcttg taagtttttg tcttcatttt ggttccacta atgaagtgaa tctggagctt    703980
     cagggaaggg gctccctcct gtgtcattga atccttgctc tgggtctccc tgcagagttc    704040
     aataggttta gtaaggctaa tcaattgttt caagagatgg tgtcagcagc atatggtgtc    704100
     actgaaggag tattctacac tagcacacag ccatttcatg ctgggctaga gaagcctggg    704160
     ggaaatgatt tgaagtctca gcaaggaacc tccttgcaag gcaggggctg ggctgtcggg    704220
     ggcaccgagc aggtgagtca gaaatcaata tgtaaagatg aggacctatg gatatgaatt    704280
     gaaaatatat aaatacttca aaaaattcca gtaaattgag tccaaaatta accccaaatt    704340
     attcaaaaca caaattcctt gacaattatt ttgggagcag tgagttcata aagaattcaa    704400
     aactactgtt tcagcttctg attcttattg ttcctgagat gagaaaatca tctctaatca    704460
     cacatcacac agcaaatctg taaacaagag tgtttctatg gaagatcctg ggggatctga    704520
     acaccaggca ggtgctggag accctgtttc aggagcgccc agcagatctc agagggacct    704580
     gctggtcact cacgtgggac atcagcagta actttctcag tcaccagtca gctgtgctgg    704640
     tgactgatgg acccaggaca gaaccaaggc acctcctcag tgtcatggag agtgatggtt    704700
     ccagaaatca tccaggtgct ctctgtgctt ataaaatgta ggttcactgt gaggagcgtg    704760
     ttctgagggg gattgttctg tgaaaggacc tctgttcacg agtgttcata aatggagcag    704820
     ggcatgcatt tcctcaaaca ggaatagcgc ttggaccatc agcatctcac tcttgtaaat    704880
     ctgatgtgtc atttatcttc cctttcttat tattgaccag gctttgcgct atgaaatgct    704940
     ctgtctcatg aatatgcaaa taacctgaga tccactgagg tgaatttgga tgtctgtgcc    705000
     ctgagagcat cacccaacaa ccacatccct cctctataga agcccctgag agcacagctc    705060
     ctcaccatgg actgtacctg ggggatcctc ttcttggtgg cagctgccac aggtaagggg    705120
     ctcccaagtc ctagtgatga ggaggggatt gagtccagtc aagggggctt ttatcatctc    705180
     ctcccttctc ctcacagatg tccattccca ggttcagctg ttgcagcctg gggctgaggt    705240
     gaagaagcct gcgtcctcag tgaaggtctc ctgccaggct tccagataca ccttcaccaa    705300
     atactttaca cagtgggtgc gacagggccc tggacaaggg catagtggtt gggatgcatc    705360
     aacccttaca atgataacac acactacgca cagaagttcc ggggcagagt caccattacc    705420
     agtgacaggt ccgtgagcac agcctacatg gagctgagca gcctgagatc tgaagacatg    705480
     gtcgtgtatt cctgtgtgag agacacagtg cgaaaaccca catcctgaga gtgtcagaaa    705540
     ccccaggaag gaggcacctg tgctgacaca gaggagatga caaagattat tagattaaag    705600
     attttcttag aaaatgacac taagtcatta aagaaaagga acaatattaa tgtgtatttg    705660
     agaaatttta attatttgag agatttttca tacagcattt attctgtaaa caaatttcaa    705720
     tgattacaga atgaatcaaa ttaatgaaac taatacagaa cttcctctga aggtatcttt    705780
     gtaaacatta atttcttaat cagtgctgta agtattttgg aacacagaca caaaatcaca    705840
     ttttaagtct gcatttatgt ctattaaaaa tgccaaaaaa atctcctttt gtgcatgtag    705900
     cattttgaat tcccaccatc aatgcatgat agttcttggt tttccacatt catattgtca    705960
     tgtatcctta cgagaattgt gtgttttaac caatctagta ggtgagtaat gggatctaat    706020
     ttttatttaa atgcacatgt ccctccaaaa gttcatattt aacaattttc gtataacttc    706080
     tgttgagacg cctctcctga cattcggttc attttaactg cattgcttca tttcgattca    706140
     ttgcaagttg acttgcatat tgtttataaa agtcatttca caaattaaaa gaattcattt    706200
     cacaaatatg tgacttggaa gtattttctc caagtctgtg gctgtcttta ttcccttatc    706260
     agtgtgtatt gcagaaacat atgtgtgtgt atgtgtctgt gtgtgtatgt gtctgtttgt    706320
     acaaatttag actcaaaaac atgtaaaatt gcattcattc atagatgatg tatttggcaa    706380
     tatatatgaa gtctcattat aaaatacact aatagtgatt attttttcca tgtctctcaa    706440
     ctcaggccac aatcaactca tgagtgttta aacttcatct acttgattgg aggactatcc    706500
     atttcagata tttggaatac ttctgtaagg aaatgtgttc gtcttcccaa tatttattta    706560
     tataatcatc tattaatatg tgtattggtt tatgaatata tatttcatac tctgaagaag    706620
     atccatgcta tattattcat tttatcgttc aaatcaccac agctttatta ggtcctggga    706680
     gctcatttaa tttggatcct gtatccttac agcacacctc atccttttgt ttttgaatac    706740
     ttccctgttt ccttgtatta caataaattc taagctcatt ttctatatta ccttttttgt    706800
     acatagaatt agcctttttt ctaaagattg cttgtttcta atgttaacga atagcagtaa    706860
     aataaaaaaa attgtgatac cgtgtacaat tacttctagg acctctcaac ctactggcct    706920
     agtagacgtg catgtttata tgaacccatg tttgtggacg catcaaaact atttatgtaa    706980
     ctaatcttct gtaactttat tatattaaaa atgagaacac actggtctcc tgacccaact    707040
     atgctaccac atggaccttt ctagccttcc ttccttgact gtccataacc acccactgca    707100
     aagtgaggaa tcctatccta ccaactgcct tattattacc tagttgcaca attttaggac    707160
     acatgcatag cggtatcaga aatgtaaagc tcattggaaa tatgtttatc tactagaata    707220
     gagtgttatg tgtagtttct ttacatttta aacttataga atttcctcat ttccaaagtt    707280
     aattagatta gcaacttcat tttctacttt ctttagtgaa gtcatttcaa ttacattata    707340
     taatatcatt tatttgaaat tcggtataaa ccaaaactat agtcaagtaa acagatagag    707400
     gatattcaag gaacttagag agtgagtatt aaataagtac aaatggcact gtttaagaat    707460
     agtaaaatta tttttagtga tataaaatgg ttgagacacg atgcagttaa tttgtctaag    707520
     ctcataaatg tgtgatggaa aatataaacc taaatatata caatttttgt aaaaagtatt    707580
     tagcagttca ttaaccccag gattaaatgc agactgtatc aagttatcta ataacttatt    707640
     tggtgagggt ggggatgtca tgagatgcat gcaaaaaaga atgaagtaat tttcctcatt    707700
     tgcatttaag atgttaccat tcactaaaga cctttaattt taaaaaaatc aattttctac    707760
     gtgacccagg ttttttcttc ctgacgagca aataacccag ataattcttt tctttccttg    707820
     gttgagaaag attttcccca aacttcagct cagttcaggc acacactgtc cctgaatggg    707880
     catttaccct cagatgggta acacacctgt caacatgggg actcttctgt cagacaaaca    707940
     cacctttact cacgtggatt cttccctcag accaacacac ctgtccccac atggactctt    708000
     tcctcagacc accacatatg tccttacatt tactctttcc tctagtaatg acatttcctc    708060
     aagtggactc ttgtctcaga caaacaaaca tgtctgcatg tgaactcttc actctgataa    708120
     gtacaaatat ttccacagtg actgtttcct gacacaagca catatatcca atgttgaact    708180
     gttttatggc aaaatgatct caagataata attataaaat ccctcctgac aaggtgtaga    708240
     tctgcatttt tttattgtac cttaactttg ccttattgtc aagaacaata gtttgcagct    708300
     ctatatgcac cgattagaga ttggtgtcca ttttctctgg aaatgtattt ttatattctt    708360
     actggatgta tttttttgat aatgtttgct actgtgaaga tacctgaaca gggtccacac    708420
     tagaaaataa aaaagactaa tcggcagatt aaccctgtgc atccagaccc acgagtcctt    708480
     tgatcctgcc cccctgaaat ggagacacag aggacagatg agcaatgctg agcggtgcac    708540
     ccaagaccac aaaaagaaag acaaggaaat gtgtcccctc ccctcctcat gaaaggcagc    708600
     tcatcccctg ttccttcagg ccctggtgag gagccaaccc atgtctgttc ccttcctcgg    708660
     tgtccacacc atggggtctg ccctgatttg ggcttccctt ctcatcaccc tcaatattag    708720
     tgtcccttgt gaatcaggtc cagctgcggc tgctccacat ggggccgttc ttccatttcc    708780
     tctgtgtttg cagaagtcct gtgtgaaatt tactgataga gtcagggggg aaaaattgta    708840
     cagcccagtg gttcactgag actctcatgc aaagcctctg atttcacctt tactggctac    708900
     agcatgagct tggtccagca ggcttcatga cagggattgg tgtgggtgga aacagtgagt    708960
     gatcaagtgg gagttctcag agttactctc catgagtaca aataaattaa cagtcccaag    709020
     tgacaccttt tcatgtgcag tctaccttaa agggaccaaa ctgaaagtca aggacaaggc    709080
     cttgtaatac tgtgagagac acaggagagg gaatatctgc gtgagcccag acagaaaaat    709140
     ctctgcagga agacaggagg gagctgcatg gtagatgctc ctcagaacca ccagggcacc    709200
     ttggggacaa cctgggggca ctcagaacca ccagggtgtg cttaggacca cgggacactc    709260
     aggatattag ggggtgctca ggatcacgag ggggcgctca tgacaccagg ggacactcag    709320
     aatcacaagg gggcgctcag gacacgaggg tttgcctggg accaccagca ggcactcagg    709380
     tcaccagggg gtgctcagga ccaccagggg gcgctcagga caccaggggg cgctcagaac    709440
     caccaggggg cgctcaggac acctgggggc gctcagaagc atcaggggtg ctcaggatat    709500
     cagagtgccc tcagtaccac gagggggcgc tcatgacacc aggggcactc agaaccacca    709560
     gggggcgctc agtacagggg ggttctctta ggaggcagct ccaaatcaga agcctgagag    709620
     gctgtggttt tcttttaaac cttggtgatt cccgacctgg tcaagcaaaa gtcttcccca    709680
     ggatctctca ccatttcttc cttgtaagtc catgattact tttacctaca aaacattaac    709740
     ttagaacagg aatttaattc aacttttaat gctgcatatt ttccaagtaa tactagcaat    709800
     gatctctcag gacaattttt acaataggtt atttatatat tttcttgatt aaaaataata    709860
     ctattattaa aatagtaaag ttataaaaat cacacctgta atcccagcac tttgggaggc    709920
     taaggcaggc agatcacttg agctcaggac ttttagaaca gcctggcagc acagtgaggt    709980
     cttttctata tatatatata aaataataat aataataata aatacctggg tatggttcca    710040
     cctgtggtag cagctacttg aaaggctgag gcaggaggat cccttaagcc tgggaggtta    710100
     atgctgcagt gagctgtgat tgtgccactg cactccagcc tcgttgacag agtgagaccc    710160
     tatttaaaga aaatgtatat cctcaataca aactgtttca atgattagag ttttgtattt    710220
     ttgtgctgta atagtcaaac aattgtacat gttttttaac attaactcag catatacatg    710280
     gtattttgtt tatttttctt tcatctgctg tttttggaaa ttaaacacga ctttaaatgc    710340
     tcttgttctc cattttggtt ggcttcaggt gtcctgtttt tcagactatt tctccatctt    710400
     ccctttctct ttgaaagtat tttactttcc tcagtctcca tgaaggaaaa agaaagtccc    710460
     tttactttct gacctccaag tctggtgaat cagttctctt ctcttcataa tcactgaagc    710520
     caaccaagtt tagaggataa tggttctcct tagaatatgc tcgtctacct gcagactctc    710580
     tgccctaact cacccttttc cagggtcctg cagaccatct cctcgtttcc ctaagtacca    710640
     cagagtgggc tctgcagctc ctgctgccct ctgtgtgctc agccctgggg ctcactagtg    710700
     ttttgatgat caagtccaaa tccccatgtg cttggaccct ctgagaccac cctctaggaa    710760
     gatgccattg tgagtgagcc ctgaaaatca tggactgtgt tcagtttcat attcctggat    710820
     gttctctgtt gtaaaggaat attgacaaat aaatactaga gtttgtattg aatattcatg    710880
     ccaaaaaagt tttttaaaaa tttttcaaat gaaacaattt tatcttgcct agtttgaaaa    710940
     ctacaatcta aatttaacaa ataatgtaat acaacgtttg tcctattagt tattcaattt    711000
     attaaaaaca gactgatatt taaagttaat accattgcac atttgagtga catatttggc    711060
     aagaacagca tttacattca gctttaacaa aacatgtatt taaatatatt tgtcttttta    711120
     gtattggaat aggcagacat acacgtagaa gagcattatt ttctactaca acctcaaact    711180
     gcaaacacaa tttaaattca attaagtaat taaaaaaata tgaaacaaat gggtgtgctg    711240
     ttttggtgtt tagatataca ttcacttttg catgggcaca tgtatgtgtc tttgctgggc    711300
     tgttgtgtat gtatgtgtgt ttgtagaacc atgaagtttt caaatacatc attaaatgac    711360
     atagttatat taatcttggc caggcatggt ggctcaagcc tgtaatccca gcactttggg    711420
     aatcttaggc aggcagatca cctggggtca agggtttgag accagcctgg tcaacagggt    711480
     aaaaccccgt ctctactaaa aatacaaaaa ttagccaggt attgtggcac aagcctttag    711540
     tcccagtgtg gcagggaaat gtggggtcgg aaatcccaca cagagtttct actggtgtac    711600
     cacctagtgg agctgtaaga agaaagccac tgccctccag aacccagaat ggtagatcca    711660
     ctgacagctt gcaccatgtt tctggaaaaa ccacataccc tcaatgccag ctcgtgaaag    711720
     cagccaggag ggagactgtg ccctgcaaaa gttttttaaa acttttcatt tgaaaagttt    711780
     taaaaaagtt ctgtcacagg ggcagaactt cccaagacca tgtgaaccca cttgttacat    711840
     caggtaacct ggatttgaga catggagtca aagaagatta ttttggaaca ttaagatttg    711900
     acttcccagc tggatttcag acttgcatgg gacctgtagt ttggaccaat ttctccattt    711960
     ggaatggctg tatttacaca atgcctgaac tcccattctt tctaggaagt aactgacttg    712020
     gtttttattt tacaggctca tagatggaag agacttgcct catctcaaat gagaccttgg    712080
     actgtggact tttgagttaa ggctcaaatg agttaagact ttggcggact cttgaacagg    712140
     catgattggt tttgaaatgc gatagcaatc atgactctag aggaccacac gtgaagtgca    712200
     acaagcaggc gcagaagaca agtgattcgt gatttcactg tagtttgttt aaatggtaga    712260
     ctcacagaag tagaatagaa tgttggttac caggggctgg aggggtggac tggaaaagga    712320
     gagattgtgg tcaaagtgta aaatgttcca gttagacaga aggagtaagt tacagtgttc    712380
     taatgcacaa gatggaaaat atggctgtga atgcattata tatttcaaaa tggctaaaca    712440
     tgcaaatgtt aaaatttttt cccactaaga aattatacag gtcgggagcg ggggctcaca    712500
     cttgtaaacc cagcactttc ggaggccgag gtgggtggat cacaaggtca ggagttcaag    712560
     accagacttg ccaagatggt gaaaccctgt ctccactaaa aacacaaaaa ttagccaggc    712620
     gtggtgttca ccacaggaga attgcttgaa gcaaaggctg cagtgagcca agatcgtacc    712680
     actgcactcc aaccggggtg acagagtgag actcagtctc aaaaaaaaaa agaaatacat    712740
     ttatgaggtg ctggatatgt taaatagctt gattaacaat tccataatgt atgcatgtat    712800
     gatagcatgt gtgaatggac ctgtatgaag gtgaggtgcc ctggtcacgt gacagtgagg    712860
     tgacttgggg gaggtgtaag gttacctggg ggtatgtgtg cagtgaactt gggcacatgt    712920
     gaggtgaccc agggtgcatg tgtggtggcc tgggtgcgtg tgaggttacc tggacatgta    712980
     tgaggtgaca tggggaattc gtgaggtgac ttggatatgt atgatgtgac ctcaggcatg    713040
     tgtgaggtga cctggcggac gtgtgaggtg acctggggga cgtgtgaggt gacatgggga    713100
     atgtgtgagg tgacctgggg gaatgtgtga ggtgacctgg gagcattcga ggtgacgtgg    713160
     ggggtgtgtg agcttagcag gagtgtgtgt gaggtgacat ggggtatatg tgaggtgacc    713220
     cccctgacgt gtgaggtgac ctgagcacat gtgaggtcac ctaaggcaca tctgagttga    713280
     cctgggggat gtgtgaggtg acctagggaa atgtatgagc tgacaaaggg catgtgtgca    713340
     gtgacctggg tgcatgtgag gtgacctggt gcatgtgtta gtttacctgg gggatgtgtg    713400
     aggtgaacag gggcaggtgt gaagtgcctg aggaatgcat gaggtgacct ggtgcatgtg    713460
     tgtggtgacc tagcagattt acgaggtgac ctgcgggatg tgtgaggtga atagggactt    713520
     gtgtgaggtg gccacaggaa tgtgtgaggt gacctggggg atgtgtaagg tgacattggg    713580
     gatgtgtgag gtgactcagg gcacatgtga ggtgacctgg gggatgtgca aggtcacctt    713640
     gggtgagtgt gaggtgacct tggggatgtg caaggtcaca ctgtgtgagt gtgaggtcat    713700
     ctgggatatt tcaaacttcg taaggaaagc ctgcaacagt atatggcact aaaagcttca    713760
     cactccaaca ccgttaatgc gcacatcctc aaggaactct aagtgtttcc aggttagctt    713820
     aaatgccatg gagcgacacc tgccccaggc acactcatat atgcatcctg ggtctcccta    713880
     tcaaccccta tcctcaaatt ttcagtttat gtttgctcca tgatatcaac tctgatatgt    713940
     tgaaggtttt tttttttctt ttatttgtag ttgttcaggt ttgctatttc actctcatta    714000
     ctctgggctc agtcctctcc tcaggtgtcc cacttcagag cttgctatat aataggagac    714060
     atgcaaatag gaccctccct tttctgatga aaagcagcgc agccctgacc ctgcagccct    714120
     gggagagaag ccccagccct gggattctca ggtgtttcta ttgggtcaac agcaataaac    714180
     aaattaccat ggaatttggg ctgagctggg tttttcttgc tggtatttta aaaggtgatt    714240
     catggagaac taaggatatt gagtgagtgg acatgagtga gagaaacagt ggatatgtgt    714300
     ggcagtttct gaccagggtg tctctgtgtt tgcaggtgtc cagtgtgagg tgcagctggt    714360
     ggagtctggg ggaggcttgg tacagcctgg ggggtccctg agactctcct gtgcagcctc    714420
     tggattcacc ttcagtaaca gtgacatgaa ctgggcccgc aaggctccag gaaaggggct    714480
     ggagtgggta tcgggtgtta gttggaatgg cagtaggacg cactatgtgg actccgtgaa    714540
     gcgccgattc atcatctcca gagacaattc caggaactcc ctgtatctgc aaaagaacag    714600
     acggagagcc gaggacatgg ctgtgtatta ctgtgtgaga aatcctgtga ggggacacaa    714660
     gtgcgagccc agacacaaac ctcctgcagg aacactgggc gaaatcagct gcagggggtg    714720
     ctcagggccc actcatcaga gtcaacccca gagcaggtgc acatggaggc tggggtttgt    714780
     ttcctgtcag gatttgggac ttcctctgct tctgacagtt tccctaggga aactctttaa    714840
     ttttagattt ctgtgcccac caatgtcatc tctacatata tttttttaaa tcattataat    714900
     acgaggactc attctcacat gcacaatatg tatattgcca cctacgggaa tgaaaactcc    714960
     tcaaccatgg tcaccagcat cagagttgtg aggaagctca ggggtgcctg gtgagtcttc    715020
     tccagtcaga ctcaggacag taacctcaat gggattccct gactagaatg gcttttaggg    715080
     atttcgatta cagccaagag agaggctgga ccaggttcag tgtcatgtag gacctcacag    715140
     attttatgta tgacatttct cctgacaata aagtatgcaa atgagtatca gcactgatct    715200
     ggtgcttcct gactttcatt tttagtggtt ctcgcttttc catttgattt tcctgctttc    715260
     tggataaaag gatgttgtcc ctgtggtcta aatcctgggg ctcaagccct ttccctggag    715320
     cttaggtggg gctcaggctg tgactcctgc agccattggg agaggctgct gagactttct    715380
     tctctctcat tgttcaggat actccactct gttttctgga gacgcgtctg agaacgcgtg    715440
     tggccattag taatgagggc acaagcttct ttggtcaaaa tggggtgagg atgtggaatt    715500
     gatcctgtgc tgtgtaaact gtcacagagt caccttcttc accagtagtg ttagaagagc    715560
     ttgtaaaatt tgtcggaatc caactggagt cccctgtgtt caaaccctga caaatggagc    715620
     tggggaaggc catggatgga agcttctcat gcacataccc ctgagaacaa gacctagctt    715680
     tataacactc caaaaaacca caaattgaac aaaggcaacc acaacaataa aagaattact    715740
     tatatccctg agaacaagag ctagctttat aacactcaga ataaccacaa caacaaaata    715800
     attacttata tccctaacaa caagagctag ctttttaaca ctcggaaagg ccacaatctt    715860
     gcacaaggca accacaacaa tgaaagaatt acttctgtga aaatatctgc acagcaactg    715920
     cctatccaac cttacactga tgtcaacctt gttattgatg cttctagccc aggatcgtcc    715980
     tcatcaaaat gtcattcaga tcctgcgtat gaaaaaaatt gtgtttttca gtgcactcat    716040
     gttctagtaa agccctcaga gaccctctcc ttcacctgtg ctgtctctgg atttccaatc    716100
     ataaccagta cttcctcctg gagctgtatc tgccagcccc cagggaagaa gctgaagtgg    716160
     gtcaggtgtg taggtcacga ggaaacacac agtgcaaccc gcttctcaag agtccagtca    716220
     ccacctccag atccacattc agaaaacagt ttttcctaca gctgagctac ccgcacaatg    716280
     agtaaaccac catggatttt tatacaaaag acacagaagg ggaggtcatt gtgaggccag    716340
     acacaaacct ccctgcaggg aagctcagga caccaggggg tgctcagaca ccaagggctc    716400
     tcaggacaca tcaaggcagg tgcaagaggg gaaaaaaggt gctggaggtg gggtttttca    716460
     tcactgtcat attttactct ccactatatt tattctaatg tatattattt tattattaga    716520
     caatgatatt tatataaata tatagccata cgtaggtgca ccaagttgtc ctttccatct    716580
     tatgtggacc ttgtccatta ggacgaagtc cctgtatgta tttgagcacc tcataagtta    716640
     tggttaatta tgtaggatct ctcacttttt ctgtgtccct tcctccctcc tctctcacac    716700
     acaaactgac acccacaaag agttctacaa ctttaattac ctgatgcgtt gaagaaaatt    716760
     tactgaagtg cagctttttc agtttaactg ctgttcatgt tgatgtagga ataagaacat    716820
     tgtctttctc aactgtgtag ttctctaagc tgagtagcac ctttatttat aatacccaga    716880
     tactgaaagc aatccaaata ttgatcagca ggttaagcag taaacacttt gttctaaatt    716940
     cgttcacttg aataataccc actgttcaaa caagtacggc tggaaatgct caacaagggt    717000
     aaattcacaa gtacttatga cgagtaacat aagccaaaga aatacaagta catacataca    717060
     gttccacttt tataaatttt ataaaatgaa aactaatcta aagttacata atgaaaacca    717120
     gtagttggcc gtggacatgg tatgagaagg gaaggtgtag caagtggaaa ttacaggaca    717180
     acgagaggaa attttgagga taattttttc tgtattgaga aaggttatgg ttatgtcatt    717240
     atttgtcaaa ttgtacacat taagtgaaga ttattacttt ctaatttcga cttcttaaaa    717300
     tactacaaat ttaaacatat ataatttggt agaaaaggag ttagagatgg ataaaatata    717360
     tgagaaataa aaaagaaaat ctcagaacga tggcataagg attcaaccat caaactaaat    717420
     aaattttaaa tttctcaaca cagaattata gattcataaa accagctcct ggattcattg    717480
     attttttgga attgattttc atgtctttat ctccttcagt tctgctctga tgttagttat    717540
     ttcttctctt ctgctagctt ttgaatttgc tcttgcttct ttagttcttc aaattgtgat    717600
     gttagggtgt cagttttaga tctttcctgc tttctcttgt agacatctag tactataaat    717660
     ttccctctac acactgcttt aaatgtgtcc cgaagattct cagatgttgt gtctttgttc    717720
     tcattggttt caaagatcat ctttatttct gccttcattt ctttatttac ccagtagaca    717780
     ttcaggaaca ggttttccag tttccatgta gttgtgcagt tttgagtgaa tttcttcatc    717840
     ctgagttcta atttgattat tgcacttgtg gtctgacaga ctgtttgtta tgatttctgt    717900
     tcttttgcat ttggcgagga gtgtattact tccaattatg tggtcagttt tagaatatgt    717960
     gcgatgaggt gctgagaaga atgtatattc tgttgacttg gggtggagag ttctgtagat    718020
     atctattagg tctgcttcaa gtcctgaata tccttgttaa ttttctgtct cgttgatctg    718080
     tctaatattg acagcggggt gttaaagtct cccactcttg ttgtgtggga gtctaagtct    718140
     ctttgtaggt ctctaaggac ttcctttatg aatcggggtg ctcctgtatt tggtgcatat    718200
     atatttagga tatttagctc ttcttgttgc attgatccct ttaccattat gcaatgcctt    718260
     tctttgtctc ttttgatctt ttttggttta aaatgtttta tcagagatta ggattgcaac    718320
     tcctgctatt tttttttttt gctttccatt tgcttagcaa atattcctcc atccctttat    718380
     tttgagccta tgtgtgtctt tgcacatgag atgggtctcc tgaatacagc acatcaacaa    718440
     aataaataga cagctatcca gattaataaa gaagaaaaga gagaagaatc aaataaatgc    718500
     aataaaaagt aatgtagggg atatcactac tgatcccacc gaaatacaaa ctaccatcag    718560
     agaatactat aaatgcctct atgcaaatat actagaaaat ctagaagaaa tgcataactg    718620
     cctggacaca tacaccgtcc caagtctaaa tcaggaagaa gtcaaatccc tgaatagaac    718680
     aataacaaat tctgaaattg aggcagtaat taatagcgta ccaaccaaaa aaaaagtccg    718740
     tgaccagatg gattcacagc tgaattctac cagaggtaca aagaggaact ggtaccattc    718800
     cttctgaaac tatttcaaac aatagaaaaa gagggactcc tccctaactc tttttatgaa    718860
     gccagcatca tcctgatacc aaaacctggc agagacacaa caaaaaaaag aaaagctcag    718920
     gccaatatcc ctgatgaaca tcaatgcacc aattctcaat aaaatactgg caaaccaaat    718980
     ccagcagcac atcaaaaagc ttatccacca cgatcaagtg ggcttcatcc ctgggatgca    719040
     aggctggttc aacatatgca aatcaataaa tgtaatccat cacctaaaca gaagcaatga    719100
     cagaaaccac ataatcatct caaaagatgc agaaaaggac ttcaacaaaa ttcaacaccc    719160
     cttcatgcta aaaactcaat gctctaggta ttgatggaat gtatctcaaa ataataaaag    719220
     ctatttatgg caaacccaca gccaatatca tacagaatgg gcaaaaactg gaagcattta    719280
     ctttgaaaac cagcacaaga caacgatgcc tctctcacca ctccaattca acatagtatt    719340
     gagagatctg gccagggtaa tcaggcaaga gaaagaaata aattgtataa aaataggaaa    719400
     agaggaagtc agattgtctc tgtgtgcaga tgacatgatt gtatatttag aaaaccccat    719460
     catctcagcc caaaatctcc ttaagctgat aagcaacttc aacaaagtct caggatacaa    719520
     aatcaatgtg caaaaatcac aagcatttct ataacccaat aacagaaaaa cagagagcca    719580
     aatcatgagt gaactcccac tcaaaattgc tacaaagaaa ataaaatacc taggaataga    719640
     acttacaagg gatgtgaagg atctcttcaa ggagaactgc aaaccaccgc tcaaagaaat    719700
     aagagaggac ccaaacaaat ggaaaaacat tccatgctca tgaataggaa gaataaacat    719760
     catgaaaatg gccatactgc ccaaagcaac ttatagattg aatgctatcc ccatcaaact    719820
     accaatgact ttcttcacag aattggaaaa aactatttta aagttcatat ggaaccaaaa    719880
     atacatcctg catagccatg acaatcctga gcaaaaaaaa aaaaaacaaa gctggaggca    719940
     tcatgctacc tgacttcaaa ctatactaca aggctacagt aaccgaaaca gcatggtatt    720000
     ggtaccaaaa cagacatata gaccaatgga acagagcaga ggcctcacaa ataacaccac    720060
     acatctacaa ccatctgaac tttgacaatc ttgacactaa caaacaatgg ggaaagaatt    720120
     ccggaactat ttaataagtg gtgttgggaa aactggcgag ccatatgcag aaaactgaaa    720180
     ctggacccct tccttacacc ttatacaaaa actaactcaa gatggatgaa agacttatcc    720240
     agacctgaaa ccataaaaat cctagaagaa aatctaggca ataccatcca ggacataggc    720300
     atgggcaaag acttcaagtc taaaacacca aaagtaatgg caacaaaagc taatactgac    720360
     aaatgagata taattaaagg aaagtgcttc tgcacagcaa aagaaactat catcagaatg    720420
     aacaggcaac ctacagaatg ggagattttt gcaatctatc catctgacaa agggctaata    720480
     tccagaatct acaacaaatt tacaagaaaa aaaaacccat caaaaagtag gtgaaggata    720540
     tgaacagaca tttctcaaaa gaagacattt atgcagccag caaacatgaa aaaaccctcc    720600
     tcatcactgg tcattagaga aatgcagatc aaaaccacaa tgagatacca tctcacacca    720660
     gttagaatgg tgaccattaa aaagtcagga aacaacagat gctggagagg atgtggagaa    720720
     atagtaacat ttttacactg ttggtgggag tgtaattaag ttcatccatt gtggaggaca    720780
     gtgtggcgat tcctcaagga tctagaacta gaaataccat ttgacccagc aatcccatta    720840
     cttggtatgt accccaagga ttataaatca ttctactaca aagacacatt ggggcaccat    720900
     tcccaatagc aaagacttga aaccaaccca aatgtccatc aataatagac tggataaaga    720960
     aaatgtggca catatacacc atggaatact atgcagccat aaaaaaggat gagttcatgt    721020
     cctttgcagg gacatggatg aagctggaaa ccatcattct cagctaacta acagaagaac    721080
     agaaaaccag acaccactgt tctcactcgt aagtgggagt tgaaccatga gaccaggtgg    721140
     acacagggag gggaatatca cacactgagg cctgttggga ggtaaagggc taggggaggg    721200
     atagcattag gagaaatacc taaggtagac ggcaggttga tgggtgcagc aaaccaccat    721260
     ggcgtgtgta tacctatgta acaaaactgc acattctgca cacgtacccc agaacgtaaa    721320
     gtatcattta aagaaaggat tacatattcc attaaatata tatgagaaat cagagactcc    721380
     tgaatataca catgaataca cactgggtct acctatattt ttagggaaac actagaatac    721440
     agcaaaataa tgtcatgatt ttattaaaaa tgggggttta tcaaaccaca ccaggcatgt    721500
     ccagctctgt cctggagctg gttcagggaa caggtgggtc ctgtgtttgg gagccatgac    721560
     aacaagctca cagcgtcagt tctagttgaa acctcaaaaa ggcaaaggga tctcaattaa    721620
     aatatcatgt agatgtcaca tctgtgggtg ctgcatactc ccccgatgtg aatatggaaa    721680
     ggttaattac ctcttgaact ctctgccgag attagtgctg gtctctcagg aacacccaaa    721740
     atggctcgat agagccagaa agcagactgg ctcagggttt ttgtgatggt ttcgtcgtgg    721800
     gtcagagtga ggcttcccac gcacaggaag gggattgtac agtgcgaaac acccactggt    721860
     gtcaaatgag gaagctcccg caattcctaa ctagattcac attgtgtgat aaaagacaca    721920
     caatagacat aacttcatgg tcggatatca caaaatgccg ttaaaagatg tggtaaaaag    721980
     gcaactttaa atttttagtc attggagtgc gtacaacaca aaaatcattt tcaatatttg    722040
     taacacatgc atgcaatagg acaaaggtat gtgatgaggg taaaatctcg aaagtgtgaa    722100
     tcttccagct accaagtcgt agggtaataa ctgatgtgtg ctgagggagg aaaccatcac    722160
     gccatagtgc taatggtata acccttggtg aagagagttc aatattttat tgaagttttc    722220
     ataattttta tttaaagatg ccatttttca acatactcag atgattgtga gtgccattga    722280
     ttctgtatcg tatgaacaat ggcaatacct tccatgttta cataatatta aaaaccacgt    722340
     taagactgaa tatttggtgt catggtgcat cacgttattc tagcttccat actagttgtt    722400
     ttatttgttt gttttctcct ttgttggcat ttggtttcga attcatatgt caggtctcta    722460
     taatgtagga atttatttaa aagtgtcttt ttttgtccat tttgatgggg cttccaggag    722520
     acgtaagaac ctcctctgct tgcaacacat tttaaaatta cgcactaatg tagaacatct    722580
     gtatttaatc ctggttttta gttaattgac aagctctaat aagtgaagta acttctccat    722640
     tctgggctga ttttacatca ggtataggaa tatatcctag gtgaaagttt gtaccagtaa    722700
     tatgaattat taggtaataa cctccacctt tatgatttac atgttattca tcaagaaata    722760
     gtgttaaatc aggagtctca atgaaagtat tacctaaggg atatacaaaa tatattgcta    722820
     atctacataa aacagatgtg aacacactct tagtatccag ccatgtttcc tgtcaaccac    722880
     acagtaactt tgacttcact tgggacttgt tctaattttc aaattagtta cttattaatc    722940
     ttaatgcttc tagatattat gtgtgactat tttagcagag agtgaagaaa gacagcctag    723000
     gccgactaaa caatgaggaa aatcacaacc tgatgaaaca ggaagcctct ggatgtgagt    723060
     ggctccagga ttgaataatt tgacaactca tgtacccagg aagtttcttt tctcaatttt    723120
     ccacactgat gcatccagaa agtcagcttc accccccagg tgactcccat aatgcagtga    723180
     catggtgaca atattcccat ggtcacatac gtattttata tattggctgg aaaaggggca    723240
     gagacagttc tgcaattctc ctctgaagga ccaggaacac ctcaacagac cacctcccct    723300
     gcccccatga ctagaactgc accacgtgcc cacatggaca ctcatccctg atggggataa    723360
     gaagactcca ttgatgaggc cgactatttt agcatataaa ttagtaaaga ctgatataaa    723420
     ggtttcaaca actaattgaa gtctgttctt ctatgtccac cagagatata gatgctccag    723480
     cgatatcttg tttttctttg ctgcgtgact gtgtctcttc tccttgttcc atcctccaga    723540
     gaatctcttt cagctcccac aggtgcattc ctctgttaca tgtaactgac aattgataaa    723600
     ttagtggaag cccttacact aaaggaagag tctctgacct catcttggtc catattccta    723660
     gaaaggcatt gtgcccgtaa gtctgggtgt gaccttctga gtgttcctgt ccctcctcca    723720
     gaggagatgc tcatctgtgt gttcttgtcc ctttcactgg ggtagaacca ccctgtttcc    723780
     cccaggtgtt ccctcccaca gctccagtgt tccgcatcag tgtcatcacc tcccagatct    723840
     gctgccctgc cctgcagact aaagctctga ttccataaga aggagagtta tgtctcaaca    723900
     gaacttcgtg gcagtgacct ctgttcccat ctcaattcct gaggggttgc accagtgccc    723960
     ctagggtact ggttttggtg gttcccctgc aaagtaattt ttggttctgt agtggatata    724020
     agggagtcga gtctgaatgc ctttcaaaaa tgggggctct tgttctctcc cagacagaca    724080
     ctttgggaaa ggaagatttt gtgactgccc cttttttggg gaaagggatt caagaggata    724140
     gaaaagctct tcagtatgtg gtcccttaga atttcaaact acaacaagct taccatattc    724200
     agtttcaagc aatcccatat atatttgtat ttttatcttt aacagactat atttcatatg    724260
     ccagactctg ccttaggtaa tctcatatgc tggctttgtt actctctaca agaacttgct    724320
     tcttgttaaa tttaagattt tttttaactt cagttatcct aagcattcag aaatttgcaa    724380
     aatttcatct tgactgttta tctgttattg ttgatgtagt tgtaagaaat aaagaatata    724440
     tatccctcat ttatgtacat tttcaagtcg agtagtagat ttttagtact accagagtaa    724500
     taaaataatt tgaacattgt taagctgctt aacagaaaat caaattatgg taaatttgtt    724560
     caatggaata ctacacaaca tttataataa ataattgtct gatacatgca acaagaaggt    724620
     aaaatatcta agtatttttg ctgagtaaaa taaaccagac aaatgagaag atttaccata    724680
     taatttcatt tatataaatt ctggaaaata aaaactgaac ttacttaagt aatataacaa    724740
     caaggtaaaa tatctaagta ttgatgttca ggaaaataaa gcaaatgaat aagaatatgt    724800
     actatgttat tccattttaa taaactctga taaattaaat tgaatctaca gcaatataaa    724860
     gaagaacaga atttaccatt tggggaaatg gtagaagaag ggaagaggaa aggaggagga    724920
     atatagaaga atgagaggga aattttgtga attttctggt tcaccttggt aactaggatg    724980
     gttacatcag gtttatcaat tgtacacttt aaatatgtga agtttattat cagtaaactg    725040
     aaatttataa aatttattac cagcaaacaa atgaaaactt gcacaagaaa taagtgacat    725100
     aaagatagaa aaaatattaa atttcagaaa cacctaataa tttatcttcg tgaaccctag    725160
     ttctcaccat atttttaggt gaatgctaga atgcagcaaa attacacatg ctctcaatac    725220
     agaaagtggg tttcacaaac cacactaggc atgctcagct ctgtcctgga gttgggttag    725280
     ggagtaatat agggccagtg gatgaggagc acaggcctag atactggggc tcactaacct    725340
     caggtatgag ctcttagata catacaaagc ccctccacgc atgggtttac ttccccatct    725400
     gtaaattgag aaaccattga cccctaaaaa tatgatttac acaaatatgt aaaaatgtaa    725460
     gagagtgatt agtgcaaagt gtttatcaca gcacaatttc ataacaagac agcaagtttt    725520
     ccaaacagca tcattgtcat tagattcctg cagggcatca ttaccttatc tgggccctgc    725580
     cctctgctca ggcatcccac cccagagctt gctatatagt aggtgacatg caaatagggc    725640
     cctccctctc ctgatgaaaa ccagcccagt cctgaccctg cagctctggg agaggagccc    725700
     cagccttggg attcccaagt gttttcattc agtgatcagg actgaacaca gaggactcac    725760
     catggagttt gggctgagct ggattttcct tgctgctatt ttaaaaggtg atttatggag    725820
     aactagagag attaagtgtg agtggacgtg agtgagagaa acagtggata tgtgtggcag    725880
     tttctgatct tagtgtctct gtgtttgcag gtgtccagtg tgaggtgcag ctggtggagt    725940
     ctgggggagg cttggtaaag cctggggggt cccttagact ctcctgtgca gcctctggat    726000
     tcactttcag taacgcctgg atgagctggg tccgccaggc tccagggaag gggctggagt    726060
     gggttggccg tattaaaagc aaaactgatg gtgggacaac agactacgct gcacccgtga    726120
     aaggcagatt caccatctca agagatgatt caaaaaacac gctgtatctg caaatgaaca    726180
     gcctgaaaac cgaggacaca gccgtgtatt actgtaccac agacacagtg aggggaggtc    726240
     agtgtgagcc cggacacaaa cctccctgca ggggcgcgcg gggccaccag ggggcgctcg    726300
     tgacccactg agggcgggac aggtcccagg agcaggtgcc gggagaggtt tcctttctcc    726360
     tcagctggaa aagtcaggtt tatcttcgca ggactctgga gtcttctagg ctgtgatatt    726420
     ttgttactta tatttattat gaattttatc attaatactt aaattttagt aattattaac    726480
     attctacata ttattatatt tttaagtata tactttcaag aaataaacat tcctaattgc    726540
     ataataatta ttgtaacata attattgtaa catataatta tatcctaaat gcataataat    726600
     tattgtaaca atcacattct cattttgtat ttgaattttc atatttattt tactaagatt    726660
     ttaattttaa tataaaatat ttttctacaa ttgtctgttt tccatttttg tgagataaaa    726720
     ttagcatata caaaaatgca cagattctcc aggtacagtt agagggtttc aggcaaatgt    726780
     gcacatactt gtctacagca gctaagtgag ggtgaggaac aggtgaggtc catctcccca    726840
     caaagtgtcc tcttagtgct tccagttagt tctcacataa ggattttttt ttatttcaac    726900
     tttataattt agatacagag ggttcatgtg tggatttgtt acatgggatt actgagtgat    726960
     gctgaggttt ggaatacaga tttcttcacc ccctccctcc atggtctagc agtccacagt    727020
     gtctgttgct cccatattta tgtctatgtg tgctcaatgc tgaggtccca cttacaagaa    727080
     tatgtggtgt tcagttttat tttcctgaat taatttgttt aggattaaga actccagctc    727140
     catttgtttt gctgcaaagg acatgatttc attctttttt atggctgtgt agtattatat    727200
     attttacatg taacacattt tatatatcca ctctaccatt gatgtgcatc tgggctgatc    727260
     tttgtctttg ccactgtgaa taacacagca ataaacatac acatgcatgt gtctctttgg    727320
     tagaattatt tgtttacctt gtagtgtata ccctgtaata ggatagctgg ttcatataat    727380
     atctccattt taagtttttt gagaaatctc cagtctgctt tccaaagtgg gtggactaat    727440
     ttatattccc attaacagtg tgtaagtgct ttcttttcac cacagcccca tcagtatcca    727500
     ttggtttttg actttttagt gataaaaatt tgagtggtgt gaggctgcac acctacaacc    727560
     atatgatctt tgataaggct gacaaaaaaa caagaaatga gaaaggaatt ccctgttcca    727620
     taaatggtgc tgggacaact agctggccac acgacaaaga ttgaaactgg atgtctgctt    727680
     tcaacatata taaaaattaa ctcaaaattg ataaaagatt taaatgtaag acatcaaact    727740
     ataacaatcc ttgaagacaa cgtgaaaata ctcttctcga caccagcttt gacaaatact    727800
     ttttggctaa gtatgcaaaa gcaattgcaa caaaaacaaa aatagataag gagagactaa    727860
     tttgctaaag agttactaca cagcaaacca accagctaat caaccaaaca aacaaataaa    727920
     caaacaaaac tatcagcata gtaagcagac aacctacaga atgtgggaag ttattcacaa    727980
     atgttgcatc caacaatgcc ctaatatcca gaattgtatt agctggtttt catgctgcta    728040
     ataaagacat acccgatatt aggaaattta tcaaaaaaga agaagaagag gtttgatcca    728100
     cagttccaca aggctggaga ggcctcacag tcatggtgga atgtgaaagg cacgtctcat    728160
     atggcagcag acaagaaaag agaacttgtg caggaaaatc cccctttata aaactatcag    728220
     attttatgag acattcactc tcaagagaat agcatgggaa agacccaccc ccatgattta    728280
     attatctcac agggggttta ttccacaaca ttaggaaatt actgaagctc caattaagat    728340
     gagaattggg tggggacaca gtcaaatcat atcattctgc ccctggctcc tcctaaatct    728400
     catgtactca catttcaaaa tggatcctgc cttcccaaca gtcccaaatt cttatttcag    728460
     cattaactga aatgtccgca ttccaaggcc tcatctgaga caaagcaagt cccttctgcc    728520
     tatgagcctg taaaatcaaa agtaagttaa ttacttccta gacacaatgg gagtacaggt    728580
     attgggtaaa tagagcaatt ccaaatggga gaagttggcc aaaacacaga ggctaaaggc    728640
     cccgtacaag tccaaaatcc agtgggacag ttaaatctta aagctcaaaa aagatctatt    728700
     ttgactccat gtctcacatc caggtcatgc tgatgtaagt gttgggttcc catggtcttg    728760
     ggcagctatg tgcctctggc tttgcagggt acagtctttc acccagctgt tttcaccggc    728820
     ttgctctgag tgtccatggc ttttctaggt gcatggtgca agctgttggt ggatctacta    728880
     ttctggggtc tgaataactg cggccttcta atcatagctt cactaggcaa tgcaccagtg    728940
     gggactctgt gtgaaagcac ccaccccaca ttttctttcc tcgctgcccc taacagaggt    729000
     tcccatgtgg gcccctccct gcagccacct tctgcctgga catccaggca tttacataca    729060
     tcctccgaaa tctgggcaga ggttattaaa cctcaattct tgacttctgt gcacttgcag    729120
     gctcaacaca aaatggaaga tgctaaggct cggggctttc accccctgaa gccacagcct    729180
     gagttatatg taccttggcc ccttttaatc atggctggag cagctctgat gcaaggaagc    729240
     aagtccctag actgcacaca gcagagggac cctgagccca gcccatgaaa tcattttttc    729300
     ctcctaggtc tctggcttat gatgggagaa ccttccacaa aggtctctga catgccctgg    729360
     aacattttct gcattgtctt ggtgaccaac atttgggtcc tcgttactta tgtaaattta    729420
     cgcagctggt ttgaatttat cctaaccaaa tgggatttta ttttctattg cattgtcggg    729480
     ctgcaaattt tctgaacttt tatgttctac ctccttttta aaactgagtg tgtttaacag    729540
     cacccaagtc acaccttgaa tactttgttg cttagaattt tttactgcca gatacctggt    729600
     atcatctctc tcaagctcaa agttccacaa atctctaagg cagaggcaaa atgccaccag    729660
     tctctttgct aaagcctaac tagagtcacc tttgctccag ttcccaacaa gttccttgtc    729720
     tccatctgag gccacctcag cctggatttt attgttgaca tcattatcgg cattttggcc    729780
     aaagccattc aacaaatctc tagagagttt caaactttca cacatttttt gtgtcttcct    729840
     cttagccctc caaactgttt catcctcagc gtgttttcca attccaaatt tgcttttatg    729900
     cttttgggta tcttatcaga agcacttcac tcaactgata ccaatttacc gtattagttg    729960
     cttttcatgc tgctgataaa gacatacctg agactgggca tttacaagag aaagaggttg    730020
     aatagactca cacttccacg ttgctgggga ggcctcgaaa tcatggcaga acatgaaagg    730080
     cacatatcac atggtggcat acaagagaag agaggacatg cagggaaact cccctttata    730140
     aaatcatcag caagacagga gaactcatgc agggaaactc ccctttatca aatcatcaga    730200
     tctcatgaga ctaattcact atcatgagaa tagcatggga aagacccccc cccaccatga    730260
     ttcaattatc tcccacgggg tccctcccac aacacatggt aattatggga gcagcaattc    730320
     atgataagat ttgtgtgggg acacaatcaa accatatcaa gaatgtatag gaaacttaaa    730380
     caaatcaaga atcaaaagac aaataacccc attaataaat gggcaaataa caagaacaga    730440
     cacttctgaa aagaagactt acaggtggcc agcaatattt taaaagattc tcatcatcac    730500
     taaccatcag aaaaatgcaa atagaaaaat gttctaattt ttgtcattat agattgattt    730560
     tttctgtttt gaacttattt ttgctatttt ttaggtttat ttatgtaatt tcatgtctca    730620
     aggttttgtc atcgtatata tacatatgta tgtactaata cacatatgaa tatttcatat    730680
     ctgaatcaat ccataacatc agtaaatgac agtttattaa gtaaataaat cagtttatta    730740
     tgtgaaataa tgacgatatg tatatttgtt ttcctgttga tgaaatttaa atttgtttcc    730800
     aacataaata ttataagcaa actgttataa ctatttttgt gcaagttttt ctgtttatat    730860
     tctcacatat tgataaaata tgtagaaata taagtatgct tttttattat aagacttaca    730920
     ttttcagctt tatggagcta aagttgacaa ataaaattgt atgtatttaa ggtacaccac    730980
     ttgacgtatt gatatacatg ggaaaatgct ggatggtaga taagtaaaca atgctaaaca    731040
     gcactaatta tcagggagat gcaaattaaa actgcagtga tatttcttaa accagtcaga    731100
     acagctacta ttaaagagcc aaaaataaca ggtattggtg aggattgaag gcaaaaggaa    731160
     cgcttggaca cttgtggtga ggatgtagat tagcacagcc tcgatggaaa acagtatgga    731220
     gatttttcaa agaagtaaaa gtagaactac tttgattcga taaccacaaa taactaccta    731280
     aaggaaaaat aaatcattat atcagaatga taagcagact tttgttttcc tgcagaacta    731340
     ttcataatag cacagtcatc aaacttagga attaacctat gcctaacaac agatagtttt    731400
     ataaagaaaa tgttacatat atatacattt aaatactatc cagccatatt aagggatgca    731460
     atcatatctt ttgcagctac atggatggaa ttcatcatta ttttaagtat aataattgag    731520
     aaacagaaca tcatacacca catgttctca cttataaatg agagtgaact catatgtgca    731580
     caaggacata gagaaaggaa tgatggacat tggagactca gaaagatggg agagcaaaag    731640
     gtgggagaat ggtgagaaat tacttaatgg gtatgatgta cattatttgg atcatggata    731700
     tgttaaagcc aagactacta tgcaatatat atatgtaaca aaattgcagt cactccccat    731760
     aaatttatac atataaaata aaaacaaata caattaaaat gattaacagt tgataaacaa    731820
     tactgaaaat taaaatttgt gatcaataaa tgaaaataat attagttgaa cttcaaattt    731880
     taaaacattt tctactcaag tgactatcaa gaaaattaag gacaagctgc aaaggaaaaa    731940
     atatttgcaa gtcatatatc taccaatgta attataacaa gaaccggcaa acctcaaaga    732000
     tgtacagatg acacatcagc ataggaatgt gattttccac cagagaaatg caaatcaaga    732060
     ccaaaaggag acactactat agactttcta gaaagacaaa aaataaaaaa gaaatactga    732120
     caatatcaga gttggtgagg aagtcagtca ccctagagac taatatattg ctagtgggaa    732180
     tgcaaaatga aaccgtttct gggaaaatca tttacagttt cccataaaat taaacatgtc    732240
     cttaatccat gacctagaac tctcactcct aagtatgtcc tacaaaggat taaaatcata    732300
     tgttcacaca cgtattcaga tgtttaacat tgtgtgtgtg tgtgtgtgtg tgtgtgtggt    732360
     gtgtgtgtgt gtgttagaaa ctaaaaacaa catggacgtc tttgaaaatt tgacacaaac    732420
     cattacaggt gaactccaga ctttcttctg agtgacagaa ggcctgcctg aaagatcccc    732480
     agagacacag tcgtggattt cactgtcacc ctcgcatgtc actggcttgg gctgggctct    732540
     ctctggctct tccctgacca ggaccagatg ttgagctcca ctacctgcag ttggaagttt    732600
     atattttcaa caatgcactg aggtctaagt tgctctgcag atggaaccaa acaaacatgg    732660
     gcgcctttga acaaacagtg cctgacattt gtactgaccc caggagaact ctttccagct    732720
     ctcattcttc ttggttctct cctgcaggcc agcagccctg aagtttagcc tggatctccc    732780
     atgcatccac ccatctcctt ccaagtgcat tttaccacag cctccactgt ttttgaagca    732840
     ctcttgggct ttgtaattct ccacactctg ttgtaaagga agtcaggtcc ttcaagacca    732900
     gattcgggac tctattttat gaccaaattt cagcctcacc cctgctcctg agacagagct    732960
     cctagataag attctgcagg tggagattag gagtgttttt cttcttcaat gtagttgctg    733020
     agcgctcagt gcagggttgg ggagaaactt tccactttgt cagcatgcag ctcctgctgg    733080
     ggtagacctt cttccataga agcagggttg ggaaccaggg ggccaatgtc ctcagtgctg    733140
     ctgcacccag ggcagagcct tcatccatca gtggggctgt ggaagaagtg agtctctggt    733200
     tctcagtagc tcttgtccag aactgagcct ctgcagcatg ttctgtcggc cccagtgtcc    733260
     tggcccctaa ggagcagcat cctaaaatgg gagctagcgt atttgagaat aacaacacct    733320
     acacattcag aagctctttg gctttctttc cagctaatat aatttccttt tttttgtgta    733380
     gcaacctgta cacacgcata ttgatgcata cagacctatg acactttttt ctcgataagt    733440
     aaaaaattat tgatcactgt gatcttttct ccaagttcac catttccctg aaggtgagca    733500
     caggtccttc tgcatgtgtt caaacaaaag gcccagagac tacctggtaa gtgaggtgct    733560
     cacctggttc tggatgtttg gtctgtctcc tcccctctgt tgccccacac aaggtcagcc    733620
     cactctttcc aggtccgaag aagagagcac aggtttgtcc tgattatatg actcacccag    733680
     cttctgatga ctctcctgtt gccagcgtcc atggcctcag tgaaggtgtc ctgcaaagct    733740
     ctggatacac cttcgccagc tacgacattc actgtgtgtg acaggcccct ggataagggt    733800
     ttgaatggat ggtagggagc tactctggca atggtaacac aggctatgca cagaagtttc    733860
     agggcagagt caccatgacc agggacacgt ccacgagcac agcctacatg gagctgagca    733920
     gtcagagatc tgaggacata gatgtgtact actgtgcgag acacacagtg tgaaaaccca    733980
     catcctgaga gagtcagaaa tcctgaggga ggtggcagca gtgctaggct tgagagatga    734040
     cagggatttt atttgcttta aagacttttt ttagaaagcg aggttaattc attgcagaaa    734100
     aaaggaaaat agaaatgtgt atggactcta attatgtggg aaattttcca tacaactttt    734160
     gttctctaag caaaattcag ggagtggaaa acaaatcaaa ttaataaacc tgataaaaga    734220
     attcctctga aaatttagtg tgagcataag tttttgaatg ggtgttgtaa atattttgga    734280
     acacagctgc tagatcacat tttaactcta cacttatctc cattatataa aatatcaaaa    734340
     tgttttaatg ttttccattt tgtgcaatta taattttgtg ttataatcac attttaacac    734400
     tttaactcta cacttatctc cattatataa aatatcaaaa tgttttaatg ttttccattt    734460
     cgtgcaatta taattttgtg ttcatgccag caatgcatga tagatcttgt tcttccgcat    734520
     cctcattgcc atttggcact aagagtattg tgtattttaa tattctaata gattagtagt    734580
     gatagctcat tgctgtttaa atgcacatat ttctaattaa aattttgtat ttaattattt    734640
     tatataattg tgatgaagtg tctcgtatgg tatttggatt attttttatt gcattgtttc    734700
     tttttgatca gttgtaagtt tccttatata cccattatat aagtcactca caaagttaac    734760
     aaaaaattga ttaacaaata tgtgttttac aagtgtattc tccaaattgt tgttgtcgtt    734820
     ttactcccat atcagtgtct gtgggagaaa aatatttata tacatatata tgtgtgtgtg    734880
     catatatata tatatatata tatatatata tatatatagt gtgtgtgtgt gtgtgtgtaa    734940
     acttagataa aataattatt tcataaatca tacttttggc atcatatcta aaaactaatt    735000
     atgaattcca ctaacaggat ttttctcttg tctctaatct caggccacaa tcacagcata    735060
     agcatttaaa tttctcctat ttgatgagaa gattattaac ttaagatgtt tagaattctt    735120
     ctgaatggaa ggtgcctttt ttctaatttt ctgtattcaa taatctgtta atatcagtat    735180
     tggctcatga atgtttattt tttactatgg agaagatcta gtgctacatt atttatttta    735240
     tcgctcaaat caccacagct tttttttagg ttctgtgagc tcatttagtt tggattctgt    735300
     atttttacag catgccccat ccttttgttt ttgatcactt ccctatttcc tggtattaca    735360
     agaaatacta agctcattat ctctattatc ttttccacac atagaatcag ttatttctcc    735420
     aaggattact ggtccctgat attaaagaat tatattaaaa cacaaaatta tgatgttgga    735480
     tgtgtgtgtt gttaatgtac tgtcagtgtt tctagaatct ctaagctaac aggcctagaa    735540
     aatgtgtatg tgtatattaa cccatgttaa ctgacccatc taatctattt atgtatccaa    735600
     tcttctgtat gtttattgca tcaaacttta gaacactggt atctacaatc tactatgatg    735660
     atacatgaat gtttcaagcc ttccttcctt gcctgtccat accacctact gcaaagtgag    735720
     gaacccctcc catcatttgc cgttcattca atttgttgta caattttaga atatatgcgt    735780
     cgtggtatta gaattgttaa cttgtacccc tgttggaagt atgtttattg actagaataa    735840
     agtgtttaag tgcagtttct ttatacttta gacttacaga acaccctgag ttctaagtta    735900
     tataggtgag aaactttatg tgccaccttc ttcagtgagg ttatttgaaa tatgttgtat    735960
     acattttatt tgacattctg taaaagacaa aactgtagat gtcataaata tatgaggatt    736020
     ttctagaaat ttagagagag ggtatgcatt aggagaaaca ggtactgttt gcaaacagtg    736080
     aaatttttta tgatctgcag tagtgaacac atgacacaat ttgttaattc tcacaatttt    736140
     atgatgtaaa ctgtgaatct aaatatatac aacttacaaa attatgtagc acatcatgaa    736200
     catgcaaagt gtacaaaaat aaaatatcaa atacatttac accgcgtggg cagggaattg    736260
     catgagatgc aggcaacaaa gaatgaagta accttcccca tttgcacata agatgtttcc    736320
     attcacaaga gacctttctt ttatcaggtt catgtgcaac caagtttccc tgctgacaag    736380
     catttaagcc agatgattcg caacttcctt tgactgagaa agatttccct caaacttcag    736440
     ctcagtgcag gcacacacca tctctgaatg ggcatttacc atcagacaat gcccacacct    736500
     gtccccacat ggacctttcc ctcagacaaa caaacacatc ctcaggttga ctcttccctc    736560
     agacaagcac ccctgtcttc atgtgaactc ttccctcaga taagcacaca tgtccccaca    736620
     ttgacttttt cctcagacaa gcacatatag ctgacaacga acagttatgt ggcaaaatga    736680
     gctcaggata gtggtaatta tggactccag ctctgatagt ttgtagaaat tgtcattttt    736740
     aaaattctga ctgaagactt tcctttattg tagaagacag tcctttacag ctctaattgc    736800
     acagcctaca ggcaggagtc catttcctct gggcaaggtt tatttttatt tgtttactgt    736860
     acttatttgt tgataaatat tgatactaca aagatagcct atagggtcca catacgagaa    736920
     aaaaagagta atgggcagat caaccctgca aatccagtcc caggagtctt tgaccctgcc    736980
     ctccctggaa tccagagaca gagatggaag aggcctgctg agcagtgcac tcatgtcccc    737040
     agggagaaag acatggaaat gaggcccctc ctctgcaaat gaaaagtagc tcatcccctg    737100
     ttcctgtaga tcctggtgag gagccatccc acatctgtgc ccttcctcag tgtccacacc    737160
     atggggtctg tgcggatctg ggctgctctt gtcatcactc tcaatgttta ggttccccgt    737220
     ggatcaggcc ctgctgtggc tgttgcctct gtgtttgcag aagtcccctg tgaagttaac    737280
     taatggagtc agacagagaa atactacaga ccaggaattc tgccttttct gcaaagcctc    737340
     tggattcact ttcactgaaa acagcataag cttgatccag caggcttcat gacaggggtg    737400
     ggtgtgggta ataacaataa ttcaaataga agttctcagt gggactctcc ttgagtaaaa    737460
     agatgattaa caatcctcaa atacactcag ttcaggagat tctcttttaa gatgattaac    737520
     ctgagagctc aggaaaagtc cgtgtattac tttgagggac acagtgaggg gacatctgtg    737580
     tgaactcaga caccaacctc cctgcaggga gacaggaggg gactgcctgg tagatgcttc    737640
     tcagaaccac cagggggtgc tcaggacatc agggggcact cagaaccatc aggggatgct    737700
     caggacacca gagggcactc aggacactgg ggggcggggg gtcactcaga accaccaggg    737760
     ggcgctcagg acactggggg ggggggggtc actcagaacc accagggggc gctcaggaca    737820
     ctatgagggg gggtggtcac tcagaaccac cagggggcac tccggacacg gtggtgaggg    737880
     gtagctcagg atagcagggg tgctcagaac caccaggggg cgctcaggac actgaggggg    737940
     ggtcactcag aaccaccagg ggacactcga gacaccaggg agccctcagg acactagggg    738000
     gagctcagaa acaccaaagg gcaatcaaga caccaggggg atctcagaac caccaggagg    738060
     tgctcaggac accaggggtc tctgaaccac tagggggaga ttaggacccc agggggctca    738120
     gaaccactag ggggttttca ggagaccagg gggcgctcag gacaccaggg ggcgataggg    738180
     acaccaggtg gttcagaacc actagagtgt gctgagaacc accaggggtg ctcagaaccc    738240
     cagggggctg ctaaggacaa tagggagtgc tgagaaccac cagtgggcgc tcaggacacc    738300
     agggggatct cagaatcacc aggctgtgct caggacaccg gggggtgctc aggacctcca    738360
     ggagcacaca cgacacaaag gatagctcag gacctacagg gggcgatcag aacgccaggg    738420
     tgcgttgagg acaacaaggg gctcacagga cacaacgatg tgcttagtaa accgggggac    738480
     actcacaacc accagggggt actcaggaca ccagggggtg cccaggaaac cagggagcac    738540
     tgaggacacc accgctccct taggaggcag atccacatca ggtccctgag tgggagcagg    738600
     gaggagggtt cctcttgtat cttgtcacta acatgttggg agtttttctg cttcctttgt    738660
     ggtttcaatt attggctgat tcttcggtat aaagcagagc aagtataaag ctctgctttc    738720
     ttggattatg tcatgttttt ggctttggat gctaccagaa ttacattgta ctttgagagg    738780
     attcattcat ggtgtgtgca atagtgaatg aaacctgtaa ttttaggggt ggctttgaaa    738840
     gctatgttag gtgtggctga gggaagttta caggaaatgg tcatcactac agaaggctac    738900
     tcatttcttt gcacatttgc gtaagcaatt gtagtttatg aattaaaaac tgcctgtttt    738960
     cttggtcctt tttcttaaat ggtcccactc taagggcagt aatgtaatca agctgtgttt    739020
     caaagatctg caatcaagtt aagtctgttt aatgaaatgc tttgtaaaga aaatgtacat    739080
     ctatttttta gagtcacctt tacattttac attgctttac aaatattaat ttggtaaatt    739140
     tagtctcata attatcttca gtaattaaaa atcttaaagt catgtcatgt taaattaagt    739200
     aatcctaggc ttctcactgt gaattagggt tactaaaaat tagaatagta agagagtata    739260
     atcaatttat ggtgaagttt attaagaaag atgaagatac gttttttgct taaaaatatt    739320
     ttgttttcca gtttacaggg cctttctact ggttttaaga tgacaaccac tgtttacatc    739380
     taaccctttt ttgttgaaca tctgttgagt ttctattcat attccacagc tagagtttta    739440
     aagtaaaagc tctagtatct ttgtattagt gtgaatgtgt gcttgtatgt attatgtaca    739500
     tatatatata ttttttgtta tgtgttatgg ctacaaggta caaaattgac tttaaaataa    739560
     ataactattt taaattaagt caatgagccc taatgcatct gaagtacatg taacttaaat    739620
     aaatatgtaa taaataagct ggcttcaaaa ttattggtaa aataaaattg gaaatatttt    739680
     aacaattatt agaatacctc atagtttata tcaattgatc aagtgatttt atatttaaaa    739740
     tcacagctag atattatatg gtgtgaaaca tttctatgaa gattataaaa ttattaacct    739800
     agttaaaacc agaatgatct ttgtaatttg acaaataaga tgtttaatac tgttgtttta    739860
     ataaaaaaca ggtaaatagt tattggaaat acaatcattt atttaataag aattttactt    739920
     aggtaaacac ctgaaattca tgggttataa tatgggtaac agggaaaaaa ctttaaagga    739980
     tgagtattac tgttttcata aatgatctag gtaagctatt taaaaaataa attaggttaa    740040
     tgtaacaaaa taaacctttt aaataaactt gttctacaat ttaaaaatct aaagtttaat    740100
     taaataatag atattaacta aatgtttagg tcattactaa ttgttttaaa aaatgtatac    740160
     tataagaaaa tatttttgta aaaaatttgt tcttacagaa agattttatt taattcagag    740220
     gttacttata aaacatccta aacataacca gtaaataaga gagatgccac tgcactccag    740280
     cctgggtgac agagcaagat tctgtctcaa aaaaaaaaaa aaagaaaaga aatttttaga    740340
     catagaggag tacttttggt atgaaaggtt aaaataaaaa aaaataattt tatatgagaa    740400
     agaatcttgt aactttttat cctaaaataa aaatgacttt atttaagaaa cagtgatgtt    740460
     tagaataaaa ctatatgctc aagtatgcca taagcgtttt gtgtaagtca aactaaggtt    740520
     tgtaaaaagt taattaatta aaataacttc atattatgaa gttgactata attaaaaagg    740580
     aagtatttat aatagtcttt atagatttgt agagatctgg ctttcatata aaaatatata    740640
     aatatactaa agattggtta gaatgaaaaa ttgtcttaaa gtattgattt actcaataaa    740700
     attataagat attttaattt tttaacccaa aagtttaact cttactgcat cttgccagtt    740760
     ttattttatt ctcttttgag aaggcttgag aggatctcaa atttttcatg agctcctcta    740820
     acattttttt tcttacagca gttagcctct aacgatgtta acttctaact gttgttagct    740880
     tctaactgct attattgcct gatgctaaaa atcttttata ttaaagttct taataaaatg    740940
     ttttatttca atatagtatt ctgcactctt gggttttttt aaatgtctat atttgtctat    741000
     ggaaccaaaa tcttcacttg taatccaaga cacattcttc ctatgtctaa ttaatcaaat    741060
     actttgtttc attagagttg acttgcaggt tatctgcatg gatttcccca cagggaaaca    741120
     cagtcacact gctgaaggtg ttttttccca tttggtaact ggcataaaac aaattttata    741180
     ttttcttgaa atacttcctc tgtagctgtt tttaagtttt tcaactactt aggaatactg    741240
     agattttgag aaaatataaa ttattgttat tacgttaatg taactatctg cataactttt    741300
     aaaggacttg tcctgctaca ttactgatct ttgattccta ggtctaaaaa ggatacacaa    741360
     cactttggga ggcttcagag ggtggatctc ctgagggcag gagttagaga cgaacctggc    741420
     caaaacggca caatctcatt ttaataaaaa atacaaaaat tagctgggcg tggtggcggg    741480
     cgcaggtaat cccaactact tgggagcctg aggcagggag aattgcttga acctgggagg    741540
     cagaggttac agtaagccga gattacacca ctgcactcca gcctgggtga gactctgtct    741600
     caggaaaaac aaataaatac aataaaaata aaaaagacac cgagtcttgc taaattttaa    741660
     actctgacag caattgaagc cccatctaga gatgtggaag aaaatgacaa taaaaattaa    741720
     tcacacactt aagacacaag gccggaaaat tgaatctact caaccactcc aggcccaggg    741780
     actgttacag aagaagcggt ttgtaagatt gtaaaagcta attttgaaag atgaaattac    741840
     ttgagagttt tttatacagt aaacattaat attaaaggca cactaatgcc aggctagtag    741900
     ctgggccatg tgtcagattg acaagggttt cttgaagaat taatccactt tttaatttga    741960
     aaaacttata aaagtttata agagatcatt tgaaattaaa ttttatggta aaagtaatta    742020
     taatgtaata gatttatttt tcagaattga gagacagttt taacttctct catgctgttc    742080
     ttataagggg ctattgttta gaaaattaat tcttctcttt gaaaaataaa agtttttgct    742140
     ttctttcaaa atcactgagt tctcactggg ctaaataaat aacttacatt acagcaatct    742200
     gtaatcctat tttgtaatat caagcattgt aaacttttga tatttgacaa acttcacaaa    742260
     ataaaattct aaattcagtc atttgacctc attattcttt tatgatatta ggtccccaaa    742320
     gccaaaatta aacatattca gcttatttgg tataattaaa atatgcagga agcaatgtca    742380
     aatttgcaaa agtgtttaac tttggactat atttaaataa atgtggacta tataattgga    742440
     ctatatttat ataaattaaa gagtatattt tccaaaattg tatgagattc aagtgatttg    742500
     atatgtctta gtatatttta tcagtagtat ttatgattat tatgtaaaat ttctgtttat    742560
     tgcagaaata accaaatctt cccctcaatt ctgtctttaa ccatggctat tctaaaactt    742620
     cagtcatcca cagttggtgt tttactttga ttctttatca ggtggcttat agtaatctat    742680
     agaattttga ggagtactca aatatacgat tgtgacaatt ttataaattg tgccattggt    742740
     atagagatta aaacttccat gactctcatt gatacctgat tcatttatga tgattgttaa    742800
     tctaatatta agcaggacag gacttaattg catgaactga attgacagaa gactgaaatt    742860
     gtttttatgg cttattcttt aatgcatttg ctaattactt atgttctgtt ttttcagaat    742920
     caggaaaagt ttgtctttta agctcttcac agttgttaac aattgagtat ggtatacttt    742980
     actgataaaa aaataaaaac ataatatctt cttatttaca taatttctcc aaaatttgga    743040
     aactgtgagt attcttatat caaaatagtt atttgcatag gttcaataaa aacctgcttt    743100
     cttccataac agggcacaat tggagacaat ggtcatttta ctaaggcttt aacttgaatt    743160
     atatattttc agatttactt tataaaatga atctaacctg gagagctgat aaagcccctt    743220
     gggaaaactg gcatgcacat tttttttttt ttacagggcc ctgaactgta gtaagtaaac    743280
     aatttaattt ctgacagacc caggactccc aggttttctt ggaaacttga aaaaagagaa    743340
     agtaacccaa ttcacatagc tatctggtgg cacagataaa atattggctg ggcttgaaga    743400
     ttttaaagat tctacccttt gattccttat aaaaaatttc cagcaaagtc aatttatgaa    743460
     taaaattgcc tatgtacaaa caaaaaatag aaaacaaaaa gagagctaat atgttaaagg    743520
     attactttgc tgcatcttat acaaaaaaac caggccaagt ctcataagcc taaaactgat    743580
     tttacaaata gattagtcct actatgattt tgtgtctaat aaaattgggg aattatagag    743640
     agaaatatta tttcaaaata aactatagtg catcagttaa tagattttaa ccttgtccac    743700
     ttgcttttca atttatatta ttttctacaa tttggactga attttaaagc gtatctttgc    743760
     acgagtctcc aaaataatgt tttcagttat ttcccttttt aaatattttt cctggcttga    743820
     aatcagcaga agttaaactg tgctttctta cagctagaca atgtaaattc taaaagaaaa    743880
     caaaatcaat gatatggttt ggctccgtgt ccctacccaa atctcacctt gtattgtaat    743940
     aattcacaca ttgcaagggt ggaatgaggt ggagataatt gaatcatagg ggctgttttc    744000
     ttcatgcttt tctcctgtta gtgagtgagt tctcacaaga tttgataatt ttatgagggg    744060
     cttccctctt cacttaacac ttctctctcc tgcagtcatg tgaaatagca tgtgtttgct    744120
     accccttgca tcatgattgt aagttttttg tggcctcccc agccatgcag aactgtgagt    744180
     caattaaacc tatttttctt taaaaattac ccagcctcgt gtatgtcctt atagcaacct    744240
     gaaaatggac tgatacaagc aacttaacta catacgaagt ctccttttgt acctgcctat    744300
     tgtgaagaga aaataaatct tgagacccca aaatcactaa gctaaagaga agagtccagc    744360
     tggtgtaata ggagatagaa agaaattatt taggtagata gttaggatga aagagtctct    744420
     ggcaaaactt ttcttctcac aagaattagc tcagaaataa cttcttttct aatcagacac    744480
     agttcaaaga gatcacttct aacaaagagc agcctgaaag atcgggctgt aaaacataga    744540
     taaacaactc tggcagagag ggtatttctg tgtgtaatca ccaaagttca catacatagg    744600
     atgggtccca ataaaaacat tgggtcttaa tgagcacatt cctttccttt tctggggtca    744660
     cactgagata ggaaagctgt tagcttgcac ggggtttggg atgcctccag ctgcaaggag    744720
     gtaccaggga cctggcatgg aaactcctcc cccctttttc agcacatgca tggtggaagg    744780
     agataaggaa cgtggagcag accaagctaa gtccccacct gcataataaa agcatgagat    744840
     ggggctgcca gagacttcgc tctctgcaga tggcacacct ggtcctgctt ttgcacctta    744900
     tgttgataag aagccatctc cccacgagca catttataaa aatccttacg ttgataagat    744960
     actgtctccc cacgagcaca tttataaaaa tccttacatt ttactgcggc acagcaaccc    745020
     atctgggacc ctttctgtga cagagagatt ttttttcctt ttacacatta aatttctgct    745080
     ctaacctcac tctttgtgtg tctgtgtcct tgattttcat agccacgaca caaagaacct    745140
     ttggtgatat tccagataac aaaggtgctt tactggagag ggctttgggc aacctgcctc    745200
     ccattctatt caaagtgatt cctctgaggc ccacacgaga cagatacata tctgattgct    745260
     tcctcttcag tatcatttat gaaaaaatga agattcacta agtctgacta aattgtggat    745320
     tcagtggtag gctgataaag gacttaaaat aatgcaacct actgtgtctt atctacttct    745380
     aaactgcaaa accccctttc aatttgtcct gtcttgaagg aaaaaaaatg tacattttac    745440
     atatattgat tgatgtctca tgtctctcta aaatgtataa aatcaagctg tacttcaatc    745500
     gccttgggca catgtctcag gacttcctga ggctggtcat gggtgggttc ttaactttgg    745560
     caaaataaat gtattagtct gttctcctgc tactaataaa aacataacca agcctgggta    745620
     atttataaag caatgaggtt taatggactt atagttccac atggctggga atgcttcaaa    745680
     atcatgtcag gaaagcaagg gacatcttac atggtggcag acaagagaga gcttgcacag    745740
     gggaactccc cttgataaaa ccatcagatc taatgggact tattcactat catgagaaca    745800
     gcatgggaaa gtgctgactt actgcaggag aactacataa ttttatattt tcctatatgc    745860
     ttctttttca ttacacatgt aaattttcat accatccaaa tttcccctta cccagctttt    745920
     cctctttata tattgaaagc cctaaaaatt gtctttgggg aatggcacta accacacaca    745980
     gtttctgtga ttacttttat ttttcttcca ggcatgtcct ggaaagacat aatttccttt    746040
     gggaaaatta attttaattt gattaagatc tgtctcagaa accttcggtt tacactagga    746100
     aagatcccaa attaggagcc aattactgta aaaatcagcc ataccactct gtgagtgtgt    746160
     gtgtgtgtgg cggggggtgt atatgtgtgt gtacatgcat gttttcattt ctgtgggctt    746220
     taagccatgt agttctctct gcgaagatac tatttggcat gacctttgaa tagagaattg    746280
     taagagaaat aagaggctcc tatgaattat ccgaaagttt ctggactcac catgtatctt    746340
     gactgtgtca ttgcatctga cagtcccagg gaagtgactc tctggtggtt tcatgaatct    746400
     gtgcttgggc tctctctgca gtttactggg tatagtaatg acaaatcact gtttcaagag    746460
     acaatttcga aagcattaga tgctgctgag agaggattat gaaccagggg acagcccctt    746520
     cattctgggg gagcgacatt gggagaatat gctctgtgag cccaaacagc ttcctcccct    746580
     gcagggtgag ggcagagctg caggacaggc ccagaaccca ctcaacacag atgtcagccc    746640
     tggagctgct gcagaggagt ctgaggagaa aattttacca gcacctgaat tacacttatt    746700
     tcaaacaaaa atgcatgtcc tgtgagtgtt tgtttcccta ttggaggagt tctgtactca    746760
     tgaagttctg gacatgccag cggacaaata tcagtaaaca aacatcagaa cttgaacctc    746820
     agcttcccac tgttgcattc tccatgtgtc atctctatta tttctcatgc tagatcaggt    746880
     atttagctat gaaatattcc agctaattaa cgtgtaaata gcttgaagtc tactgagtta    746940
     aatacatata ttttctcttg tttttgccag gtgttccctc ccacacctcc aatagtctcc    747000
     actattatca tcatcttcta gatcttctgc gatgccctgg agattaagga tttgattcca    747060
     tgacagagag gaggtgcatt tcgatggaac tttggtgaaa accttggtct ttatcccatt    747120
     tcctctgggg ctccaccagt gcctctggaa tcatggtttc agtggcttgc ccctgcatgg    747180
     taggtcattc ctttattctg tagtgctgat gagggaggtg ggtctgaacg catttcagta    747240
     gtatgggctc tccttctgtc tcagacagac actttgggaa aggaaaattt ttctgagcgt    747300
     cctcattcta gaacaaaggg attcaattgt ataggaatgc tgaaaataga aaaccttcag    747360
     ccaaattaag gttaataaga ttaattgagc aatggatgat tcatgaattg ggcagccccc    747420
     agaatcacag cagattcaaa gagacttcag tgcagtcacg gggtggaaga agatttatag    747480
     attagaaaaa tgatgtacag aaatcagaag tgaggtacag aaacagctgg attggttaca    747540
     ggttgctttt gtcttattta aacaaggtgt gcaccctcaa gagtgtacga gtggttgagg    747600
     tatggctgct ggaattggcc aagactccgc tgttgttaca ggcgcatgct ccaaagttgg    747660
     cttttcaatc tcgtccacct attcaggtag gttacagttt gtccacaagg actcaaacac    747720
     agaagtacgg agtccttttc aggccatatt taattcactt tatcagtgcc cttcagtatg    747780
     tggttcctga gaatttcaca tgacaacacg tttaccacac tggaatttaa gcaatccaac    747840
     acgtttgtag ctttgtcttg taatgggcta tatttcatgt ggcagcctcg gcctcagttt    747900
     agctaacact atggcttcat ttctctctac aagaactcat ttcttccaag atttccatgt    747960
     tcctgaaagg aaaataaacc tttgggaccc ccatatcact aagccaaagg gaagtcaagc    748020
     tgaaaattgt ttggggcaaa cccacctcca ttctttccct aaaatgagag ctactaaggt    748080
     ttttaaaagc tacagacctc cttcaaaatt tgaccacaag taaaatcctt gtggacccag    748140
     gacagagaga gtcatttctc tgctcatgta agtcaaacgc atacctgatt gctccctgtg    748200
     ctctactgtt tcactaagtc agactaaggc ctacgtgact attcctgtaa attgtgcatt    748260
     cagttaaagg ctaatcagaa actcaaataa tgcaaccatt tctctcaaac ctacctatga    748320
     tctagaagcc ctctccccac ttcaagttgt cctgcctttc tgaactaaat caatgtacat    748380
     ctgatatata tatattgatt aatgtctcat gtctccctaa attgtataaa accaagctgt    748440
     gcccacaagc ttgggcacat gtcgtcagca ctccctgagg ctgtgtcaca ggcatgtcct    748500
     taatcttgga aattgaactt cctaaatcta ttgagattag tctcagatac tctttggttt    748560
     acaggtttgt ttttatttca taacttcaat tatttgacat gctaaagaaa atttgccaaa    748620
     tagcgcattc tcttgtttat gtgttattgt tgttgcaaaa ataatatatt ttatatataa    748680
     tttatcatct atgtacatta ccaaattgag tagcagattt attagtaaga cccaaagtaa    748740
     tgaaaagttc agataccaat tagcaactta gaaaaacaaa ttatgctaca tttgtttgct    748800
     gaaatgctac ctattattta taaaaaataa acaatacaac ataaaaggtt taattctcag    748860
     tatttctatt gagaaaaata agccaaatag atgagtacat actatattat ttcattctta    748920
     taaattctag agaataaaaa ctagtctaaa gaaatatgaa aacatcagta cttttataaa    748980
     gaaatggtag aagaaaagga gaaaaacaaa aatatatcta taaaagagca agaggaattc    749040
     tgaggtgagt tgacttgtca ccttcttgaa aatagagact ttctttatca aagtttacta    749100
     ttgtacaggt taaatatgtg aattttatca tctgtcaatt aaaactcata aaatttatta    749160
     caagtaaaca agtgaaattt tagacaaaaa agggatgata agaaggaaca aataaataca    749220
     ttaaatgtca gatacaccaa aaatgtatct gcctgacgcc tagttgtctc tgtattttta    749280
     ggtaaatgca gcaaaatcac acaggttgtc gtggcaggaa gtggattctg caaaccacac    749340
     taggccgttt atctctgtcc tgtagttggt tcagagcaac tgaggccagc tgtgaggcgc    749400
     ataggcccag gtactaggac tcactcatgc cagatataag ccctgagaca cgtacatagc    749460
     ccctccatgt gtgggttcac ttttacatct gtacatgaag aaaccactga ctcctaaata    749520
     acatcatcta tacacatagg taaaaaaatt aaaaatatga tagttgttaa atgtttatcg    749580
     cagaacaatt tcaaaataag gcagcatttt cccaaataca atcattgtca tcaaaatccc    749640
     ccaggacgct ctcatctact ctgcgccctg ccttcacctc agatgtccca ccccagagct    749700
     tgctatatag taacagacat gcaaatagtt gactccctct cctgatgaaa accagcccag    749760
     ccctgaccct gcagctctgg gagtggagcc ccagccttgg gattcccaag tgtttgtatt    749820
     cagtgatcag gactgaacac acaggactca ccatggagtt ggggctgagc tgggttttcc    749880
     ttgttgctat attagaaggt gattcatgga gaactagaga tattgagtgt gaatgggcat    749940
     gaatgagaga aacagtgggt atgtgtggca atttctgact tttgtgtctc tgtgtttgca    750000
     ggtgtccagt gtgaggtgca gctggtggag tctgggggag gcttggtaca gcctgggggg    750060
     tccctgagac tctcctgtgc agcctctgga ttcaccttca gtagctacga catgcactgg    750120
     gtccgccaag ctacaggaaa aggtctggag tgggtctcag ctattggtac tgctggtgac    750180
     ccatactatc caggctccgt gaagggccga ttcaccatct ccagagaaaa tgccaagaac    750240
     tccttgtatc ttcaaatgaa cagcctgaga gccggggaca cggctgtgta ttactgtgca    750300
     agagacacag tgaggggaag tcagtatgag cccagacaca aacctccctg cagaatgcct    750360
     gggggaaatc agctgcaggg ggcgcacagg acccactgat cagagtcatc cccagaggca    750420
     ggtgcagatg gaggctggtt tcctgtcagg atgtgggact tcatcttttt agagtttctc    750480
     tagggaacct ctctaagttc agaattctgt gcttaccaat gccatcccta catattttta    750540
     aaatgattat tttaatatga aaacctattc tcttatgcac gaaacacaga ctgatgctta    750600
     cagagatgaa aagccctcaa ccattgtcac caggatcaga gtattgagga aactcagggg    750660
     tacctggtgg gtcttctcca ctcagactca ggacagaaac ctcagtgagt ttccctgact    750720
     aggacggtct ttaggaattg tgatcgcagc caatagagag tctgggcaag gatcagtgtc    750780
     atgtagaact tcacaggttt cacttctgac ccttctcctg acactaaagt atacaactta    750840
     gtgtcagcac tgatctgggg ccccttttgc tcttagccca ttctatttct ttttatttgt    750900
     tgttgttgtt cttgcttttc cttgtagtgt tcctgctccc tgtaaagtgg ggatgtggct    750960
     cttgctgcca aagctccagg tctcaagccc attccctgca gctcaggtgg ggctcaggct    751020
     gtggctcctg cagccacgtg gaagaggctg atgggacttt cctctctccc attgctcagc    751080
     accctccagt gtgtcacgtg gagactcacc tgcgaatgga agtggccaac agtagtgaag    751140
     gggatgagct tgtgtggaca aaatgggatg tggatgtgaa atttatcctg tgctgtgcaa    751200
     agtagcacag agtgagtcac cttcctcacc agtagtgtta gaaagagggt gtgaaagttg    751260
     tcagaatcaa aatagatcca cttgtgttaa aaccctgaca aatggaacta ggaatgacca    751320
     tgaaggaggt ttcccatgca catactcctg ataacaagat cgaccataaa tggattctgc    751380
     ttaaccacaa cctttgatag aagccaccat gaccttataa aaatcacttc tacaaggaca    751440
     tcttcccagc aaatcacggt ttaaccctat actgatgcca accttggtat tgactctaca    751500
     agcaaggaaa atcctctcaa aacaatttat gcaacccacc tcattttcac tcataaacct    751560
     atggattgac atcctggagc cactgctgca tttgttgtta atggtaatta gcccctttta    751620
     caatatttgt gactgttttt ctccgatgtc tcttggaaaa taaataattt acaagctgat    751680
     ggcagtaaag aaactattta ggacattttt aacatcctgt tgaatatttc tgcatagcac    751740
     atcggtcccc taaaatactt cgctgtattg gcatgtgatg aatcagagta taatgctgaa    751800
     ggtaaaatgg gaaatacatg ggcttttgat gaatcaagtc atagggtgag aatgtctgtg    751860
     tccttgagga aggagacctt gggctgtaag ttctagtggg agtacctttg gcaaagggat    751920
     ttcatgagtt tctgaacgtt atgctacttt taatttaaga atgcaactta tcatttattt    751980
     ttacttaaat ctttccagaa gatatttggc agtaaggaca gggtagcatt cgtgtgatac    752040
     tgatgactta gagagttatt ttgtaatttc tcctgtaagg tatgcacatt gctcacttga    752100
     tacagaagtt caaatgtcac aggcgggaaa ataggaataa agcaaatttt attaaatgtc    752160
     acgactgtag tttttggcaa ggaagtgctt catgtcaacc tgaaaataga cagacaacaa    752220
     taaaacatat tcaaatccaa agggagtcag acctatgtcc ctctctctta taagtacaag    752280
     gctttgccac atccaaaata tcctttaggc tccagggtat aaaatgcttt tggactgtga    752340
     agctaacagc tctcccctca gacagggata aggcatctgg ggaatgcaga gttgtgttca    752400
     tgaagaagat ggcattactg tgtcttctcc tgtgcctggt gatggctccc ccagggtgag    752460
     tgtctcagat gtcagatgtg ggtctatggg gtgagtgtag gtacatgtga ctgacgggga    752520
     ctgattcccc atgtattcac atgccctgtc ccaggagcag ctgcaggagt cagccctgga    752580
     cctgaagagc acacacttac cctctgcttc acctacacca tttctggcca ctccatcaca    752640
     accagtcctt actactggac ctggatctgc cagctctcag ggaggggctg caatggatac    752700
     aatgcattgc tagtggtggt gggaatccgt ttgtcttgtg gaaaatggca gcatctcttt    752760
     attttataag gcagaataat gttatattgt gtacacatac cacattgtct ttatccattt    752820
     gtccatcgac agacacttag tttccatatc ttggctgttg tgaatagcgc tgcagtaagc    752880
     acaggagagc aggtatcttc acagggtggt aatttcatcc catttaggtg tatttccata    752940
     agctggatcg ctggtcatat ggtatttcag ttttaattta tttagaagcc accacactgt    753000
     tttgcataat ggtaatgatg ggaatgtaga atgtcatagc cgctacgaag aacagtttta    753060
     gatttgaggt ataatccaaa agcaaataat gtttgatcat ggttctcata tgaggctcta    753120
     ataaatctag tggaagtcca gaaagttttc ccaccttggg caaggatgag tttgccccta    753180
     attgtctttg aggcagaata tttgcagaat gtgagatgga gtatgtcggc aggattcagg    753240
     atgattcagc cataaatagt aatggcacag aaaaacaggg agttagagac atgcagagaa    753300
     agaaagagat agagagaata tgaatctcat aagaggaaaa tctgctggat atcagtgttg    753360
     ggttttcatt catagagaca tcagtgtcag caagaaacca tgaagtcaag tgaggagtgg    753420
     agaacatgtt cagtctgaaa atcagcatat tctcagaggc acccattgtc ccatgacaca    753480
     ggcagagaat ttcggaaacc agtgaagtgg gagttcacaa taagtgatga agttatcatt    753540
     tttccaaact ttcattaata tgcaaagtat ttctatagat cactcatgca catacacaca    753600
     aaatgtgttt ttgcatttat gggtgtctag agaaaaataa gtgagaaaat ttttccaggt    753660
     tgcagagatc tgtttaagtt atagattcca caggagagtg tctttgaatg aatactattc    753720
     tattaattaa ataggacaaa attccctctg ttggagcagc cttccaatta tgtagatttc    753780
     tttattgctt cttgagttgt gaaacataaa cccaaggatt gacttactgg aattcgagtg    753840
     ctgtgttgat aaaatttctg ataagttttc ttccaatgat ttgagaatag ctttcctgtt    753900
     tttttactca aggaaatgaa ttttcacaag gtttcaggac atcacgtttc aggtgtttta    753960
     cttaaagaga ctcttcctgg gtgcagtcag ctttcttcta accatgagct cacttcttca    754020
     gcaaaccatt cagtttgtgc ctctattaag aattatgaag cttttaaact tcaatgcact    754080
     gaaaatcatg ccccttgaat taaatgttga ttggcactga catgaattgt ccactcttgg    754140
     atatgtgtcc tatgttatgg gctcaacata tcagtggact gagaatcctc cattagctgc    754200
     ctctgactgt ggcactgacc agattgagaa tcctcagagt catccatgaa aaagagagtc    754260
     tttaggttca tggggttttt ggagacattc aggtgagtgg aggggagaaa caggactggg    754320
     ggtggccagc catttcaaca agactgggaa agattagcat ctaagtttaa aggtctacat    754380
     cattccaaac accctgcatg acaggctgac aggaagaaat ccaaccccac agcggctcca    754440
     tagcatctct ttagtatact tggtagtgaa gctgtttcag ggaagaaacg tccactcaag    754500
     ggagtcagtg atgcttctct gagctacaat aaaccgtgta ttggacccag gtttctctga    754560
     gttcaatgtg atgattacac tcagctgctg ctccagtgag tttaaatgag catgtggcaa    754620
     ttcagatgag cctggctgtg tggtatgtta tatgtaaatc tgaactatgt aaacataaag    754680
     ggcatgtcgg aactagtgtg agggtgagag atcttagagg ccccacacct cacactcttg    754740
     tgcttatttg ctccaggaac ctccaggttc tttgagtgaa aatcaacata gatcctttcc    754800
     tggataaatc atccaaatat ctaatctctg agaaatacac atgcacattt ctccggatag    754860
     accatccagg ggaaagacat atctagaacc ttatctgtgt tgggtaaggt agtccatctc    754920
     cattacagac cctcccagca gccttccttt atcatgaaag tggataaaat tagccaatag    754980
     agaaataatc ctataatgtt atagccagaa aagggaaagc aacagtttca tttctggaga    755040
     ctctgcgtat aggtcacaac ccagagaaag aaaggatgac tgaattatta agaatcaatt    755100
     gtaagaacat ataatacttc caggatgcac actttgtttt ctcaccagta taatctgggt    755160
     taaagatgaa agtgtggaaa tgcacagact ctatgtgagg aggagaacat agggaaactg    755220
     aaagacaatg gcagagaata agacaaggac agtaggaaaa tctgaagcct ctgacataaa    755280
     ttttttgaag acaaggtctt ggcaaatcca ttgacctcag attcttttat catggggcat    755340
     ttgcagggtt cctagctgag aaaaaaaatg catgcacttc ccaagtctcc acttgtattc    755400
     tgtttgcctc agatcgctaa gtgaaaaaaa gccataaacc taggcctagt gtgtgtgtag    755460
     gaggcacttt ttataggcta gaaaatagta agaaaggaga atatgtgtta ttggaatagc    755520
     atatacaaag gtgtctttat tctgaattta tctgtacctg cagatattct caggtgcaac    755580
     attcaactgc aagagcccag cgaagaaact aggcactccc caaatccttc aagtttttgt    755640
     gttcattgtg tgtccactga ctcaggaaat gtgaagcttc agaaaagggg gtcccttctg    755700
     tgtcacagaa tcttgtctgt gggtctccct ctgtaggttt agtaaggcta atcaattgtt    755760
     aaaagacatg gtgtcagcag catgtggtgt cactgatgga gaattctaaa ccaggacaca    755820
     gccacttcat gctgggctag agactctgaa ggaaaatatt tgtgagcccc gacagaaacc    755880
     tcattgcaag gcaagagctt ctgtggaagg gggcacttgg gagccaccaa gcgcaggttc    755940
     cagccctgga gcaggtgcac agctggggag gaggtttcct ctcagtgcct gggttttcgt    756000
     ttgtcaggaa aaaacaatct aaaataactg ttcaaaaagt cgctgatgtg ctttaaatat    756060
     tctatcacat caaaaccatt catataactt aaggcactgg gaactatttt ttaaagtggg    756120
     tttctagaac tataatatct taatagtgag aatatgaagg ataggcatgt ttttactaat    756180
     tctatgggta cagattagtt gaagaaacta cattcctatg aataagaaat tcagatttca    756240
     gtgttaagta atgttgctta cattgtgtga gtgacagggc agtggtggat ctgagagtgt    756300
     ggcaggtgca cagaccaagt gagtcagaaa tcaatatgga aaggtgaggg tctgtggata    756360
     tgaactgaaa gtatgtaaat acttgacaaa atactaataa atggagttca aaataaccca    756420
     aaattgttct aaacacaaat tccttgacaa ttactttggg agtagagagt tcataatgga    756480
     ctccaaactc ctgctttatc ttctgattcc cattgtctgt gagatgagaa aatcagctct    756540
     aattatgcat cacagggcaa atctgtaaat caagagtgtt caatagaatt gaagaccctg    756600
     ggggatcagg acatgaggca ggtgctggag acagtgtctc aggagcaccc agtagatctc    756660
     agaggtccct cctggacact catgtgggac ataagcatca ctttctcaga gtcaccaatg    756720
     agctgtgctg gtggctgatg ggtccaggaa aagaccaagg cacctgctca gtgtgatgga    756780
     gagtgatggt tccaaaaatg atccaggtgg tctctatgct aatcaaatgt aggctcacag    756840
     tgaggagcct gttctatatg ggcttattct tcagtgaaag gacgtctgtc cacaaatgtt    756900
     tgtaaatgga gcagggcatg catttcctca agcaggatta gggcttcgac cgtctgcatc    756960
     tcactcttgt taggctgatg tgtcatttat cttccctttc ttatcatgga ttgggctttg    757020
     agctaagaaa ggctttgtct tatgaatatg caaatatact gatatccact gaggtaaata    757080
     tgttctgtgc cctgagagaa tcacctgaga gaatcccctg agagcacatc tcctcatggg    757140
     ctggacctgc aagatcctct tcttggtggc agcagccaca ggtaagcagt tcccaggtcc    757200
     aagtaatgag gaggggattg agtccagtca agggggcttt catccactcc tgtgtcctcc    757260
     ccacaggtgc ccactcccag gtgcagctgg tgcaatctgg ggctgaggtg aagaagcctg    757320
     gggcctcagt gaaggtctcc tgcaaggctt ctggatacac cttcacctac tgctacttgc    757380
     actgggtatg acaggcccct ggacaagggc ttgaatggac aggattttag ttatttgaga    757440
     gatttttcat acaacattta ttctgtaagc aaatttcagg gattgtagaa tgaatcatat    757500
     taacaaatct gacacagaac ttcctctgaa tcaatctttg taaacatcaa tttctgaatc    757560
     aatgttgtaa atatttcaga acacaagcac aaattcacat tttaactcta cttttatctc    757620
     tatttaaaaa atatcaaaaa atctcatttt gtgcatgtaa cgttttgaat gcccaccatc    757680
     aatgcatgac atttcttgtt tttccacatt catgttacca tttatcatca tgagaattgt    757740
     gagttttagc catgctgata ggtgagtaat ggcatctaat ttttatttaa atgcacatgt    757800
     cccaaataaa aaattcgtat taaacaattt ttatataatt tttgctggga tgcctttcct    757860
     gatatgtggt tcatttttat ctgcattgtt ttcttttcat tagttgtaag tttacttgca    757920
     tattgattat aaaagtcact taacaaatcc aaagaattga tttaacaaat agatgacttg    757980
     gaagtatttt ctcccagtct gtggttgtct ttttctctct tatcagtggg tatttcaaaa    758040
     aatatatgtg tgtctgtgtg tctgtgtgtg tgtgtgtgca cagatttaga caaaaaacat    758100
     aaaaaattat tcattcatag atcatgtatt tggcattata tctgaagtct cattataaaa    758160
     tacactaata gtgattattt attccatgtt gctaatctca ggccacaatc aactcatgag    758220
     tgcttagcct tcacctattg attggaggac tatccacctg agatatttgg aatacttcca    758280
     tgaggagatg tgttcttctt cccatcattt ctttatttaa tcatctatta acatccgtat    758340
     tggtttatgg acatctattt catactctga agaagatcca tgctacatta ttcattttct    758400
     tgttcaaatc tccacagctt tattaggtgc tgggacctca tttagtttgg atcctgcatc    758460
     cttacagcaa agctcatcct tttgtttttg aacacttccc tgtttcctga tattacgata    758520
     gattctaagc ttgtttcttt atcacctttt tcatacatag aattagccat ttatataaag    758580
     attgcttgtt tctgatttta aagagtagtg ttaaaataaa atattgtgat aatgggtatg    758640
     tgtgttgtta atgtggtata agtacttctg ggacctctca accttctgtt ctagcaaatg    758700
     tgcatgttta tatgaatcat gtcatgtcca tatatccaat gttgaactat gttgcgaaaa    758760
     gtgatctcaa gataatggta attatcaacc ccctccctaa caaggtgtag atctgcattt    758820
     ttttttcatt gtaactcaac tttgcctcat ggtcaggaac agtggtttcc agctctaaat    758880
     gtactgatta cagcgagatg tccgttttcc ctggaaatgt atttttaagt tcttactgga    758940
     tgtatttgtt gataatgttt tctactatga agatacctga acagtgtccg cactagagag    759000
     taagaaagag taatgggcag attaaccctg tgcatccaga cccaggaatc ctctgatcct    759060
     gccctccctg aaacggagac acagaggatg gatgagcaat gctgagcggt gcacccaaga    759120
     ccacaaaaag aaagacatgg aaatatgtcc cctcccctcc tcaagaaagg cagctcatcc    759180
     cctgttccct caggcccggg cgaggagcca ccccatgtct cttcccttcc tcagtgtcca    759240
     cactgtgggg tctgcactga tctgggcttc ccttctcatc accctcgtat tagtggccct    759300
     tgtgaatcag gtccagctgt ggctgctgct catggggctg ctctcagtct gctctctctg    759360
     tgtttgcaga agtcctgtgt gaagtttact gatggagtca gagggagaaa gattgtacag    759420
     cccagcggtt cactcagcca ctcctgcaaa gactctggat caccttcact gattgcagca    759480
     taagcttggt ccagcaggct ccaggaccag agttggcgtg ggcagcaaca gggagaaatt    759540
     caagaggaag ttcttacatg cacccttacg tgcacggtct cactgagatc tttacttcct    759600
     ttatcacgtt tgttctgtaa atcacaacga atggtgcatt cttcatctat tatacacttg    759660
     ttaagtcttt ttttggcatc ttttaaaaaa ctggtaactt tatcctgtgt aatatccctg    759720
     ttaagtccta aaagtctttt ttgatgtcta ttttttctta actttacaca gctactatag    759780
     atttattttg gttcatattt ttgtaatcca tgttttctca tctttagttt ttacatttgt    759840
     gaatacgtac agtaattttc taatacatag cttctagttg gggcttgctt tttaaaatca    759900
     actataataa gctctatttt taaactaata ttatttttct gttatattgt ttaaattagc    759960
     atttcacaat gatgtttatt tctctactaa catataatct aattcacttt tataaatatt    760020
     atattggtta ccataagttt tacaataaga attgtatata attagattct ataagaattg    760080
     tatataattg gattctaatg cagatactgt gatggccttg atatgaagaa cagagacagt    760140
     gtaactgtgt gctttgaatt cctccttctc actgctcttt cttgtttatt cccagtttgc    760200
     acttacatat actataaaac ataatatata aattttttat tcctttcata gttatataat    760260
     atagcaatta caaagataaa aactgcactt catcttgatt ttcccatttt gtacagtctt    760320
     aatttcttcg tatagattta tgttttgaat gtatatcaca cggctactag cagagaaagt    760380
     ttgttaaaat gtgcactgaa gcatgaatgt gctgacaata aattgtctca agatcttttt    760440
     gtaatgacag aattttattt gcctttcact tttaatgaaa atgtaatgca cgtagaattc    760500
     cagtttgcat ttcacttgta atttattttc ttgtgtttat tattttgttc atggagatga    760560
     tcacacatta cattctgctg ggcctttatt aaccacattt ctatgaacct agtcagggtt    760620
     gggtttcaag tgtatggttg ccacggctat cagagttgaa gtcagcttct cctgttcaca    760680
     aaaagttcag gttcctccag tgatacccac ttttgtgtcc cggtttggct cttcccattt    760740
     ctttctcccc agagagagcc tgtctctttc atctgtggca ggtgcatcct gctgacactt    760800
     ttacttggtg attgtttgtg gggtgaaggg gcttggacac agggggatgt tctccaacct    760860
     tctgaccaag catccttctt agctatgggt ggtgagagtg gctctgaagc atggtcttcc    760920
     aagcgttcct gttccttccc ttccccaagt cagagtgtct ctttctagcc acagtggtct    760980
     tccatcagtg tcctcagctt ctgacccact gtccttactc cacagactca gggtttcatt    761040
     cctcaggaaa gagacgggag gtgtttctgg gtagagtctc cttggtgtcc tctgtttcct    761100
     tctgttctag ttgattctac cagtgcctga aggacacaag atttaataaa tgtctcccac    761160
     atatcgtgaa gagggattca gcattgaacg gagctactgt tcttcctccc cagtcaacac    761220
     cacaggacag caggtggctg agttgtctgg ggatttcccc aattctgtag gaaaagcccg    761280
     caagtgccag tagtttcaca ctctcacagc gttagcacac acatcctcaa caactcatga    761340
     aacatttcca ggttagcttt ttcctatctt caataccata caatgagtgg cacctgcccc    761400
     aggtactcta ataaatggac ctagttttct ctgcaggctc cccattttct cagatttcag    761460
     ggtttttttc tctgcaacat caactcagat atgttgaagg gttcattttt tgtagttctt    761520
     caaatttctt gttaatgagg tcagaagaag atcattttct catttttttt tttacattcc    761580
     cgtgcttagt atctgctttc taaataaaat tcggaaacta agacaaaata tcaaatatcc    761640
     actatttggt gcattgttaa actatttgag aaatattcat gtactgaaat acaatgaaca    761700
     attgaaatca aggcatgcct cagtcacata agaatgtgag gacattatca aataattgtg    761760
     ctgagtgaag gaagctaaag aattcacagt aaatctcctg tgatttcttt tgtataaatt    761820
     gtagaaaatg caactattct aaattaacat ggagaagatc tgaatttttc tgagaaaagt    761880
     gtggtgaggt aacaagatgg tgaaataaaa ttacagagaa gtgagagaaa aaaatttgga    761940
     ggttaattta attgctaata gcacgattga agtgctgatt caaaggctgc acacatatac    762000
     caacattttc catatcgtac actataaatt tgaattcact attgattgaa tttttgaata    762060
     aagcagtaac aaaaatgagt atattggctg aggaagagca agaaagagat gaatattgac    762120
     acttgaataa tcacggactc ctgaaaatac acacatgtga acactgattg catatttcag    762180
     gtaaatacta gaaaaagcaa agtcacataa agtcgttatg acaggtggga taccctgcaa    762240
     atcacactag gcatgtccca cactgccctg gagctgtctc aggggagcag tctcctccag    762300
     tgtttagagg cacaggcacg gataataggg ctgacgctgt ccagatgtgt gatattggac    762360
     acattgcaca actgctctgt tatgtatgta attcatcttc tctaaaaatg taacattgac    762420
     acttgcactg aatatattct gcaaatatgt aaacattaaa taagatgatg actgctaatt    762480
     gatcatcaag tcacaatcac ataatctgaa gttatatttt cctgagagat aggattacct    762540
     ccagtgtttt ccgggaccct ctcatctgct ctgggcactg ccctctcctc cagcgtccca    762600
     ctagagcttg ctatatagta ggagacatgc aaatagggcc ctccctctgc tgataaaaac    762660
     cagccgagcc cagaccctgc agctctggga gaagagcccc agccccagaa ttcccaggag    762720
     tttccattcg gtgatcagca ctgaacacag aggactcacc atggagtttg ggctgagctg    762780
     ggttttcctt gttgctatta taaaaggtga tttatggaga actagagaca ttgagtggac    762840
     gtgagtgaga taagcagtga atatatgtgg cagtttctga ccaggttgtc tctgtgtttg    762900
     caggtgtcca gtgtcaggtg cagctggtgg agtctggggg aggcttggtc aagcctggag    762960
     ggtccctgag actctcctgt gcagcctctg gattcacctt cagtgactac tacatgagct    763020
     ggatccgcca ggctccaggg aaggggctgg agtgggtttc atacattagt agtagtagta    763080
     gttacacaaa ctacgcagac tctgtgaagg gccgattcac catctccaga gacaacgcca    763140
     agaactcact gtatctgcaa atgaacagcc tgagagccga ggacacggct gtgtattact    763200
     gtgcgagaga cacagtgagg ggaggtcagt gtgagcccag acacaaacct ccctgcaggg    763260
     gtccccagga ccaccagggg gcgcccggga cactgtgcac ggggctgtct ccagggcagg    763320
     tgcaggtgct gctgagggct ggcttcctgt catggcctgg ggctgcctca ttgtcaaatt    763380
     tccccaggaa cttctccaga tttacaattc tgtactaaca tttgatgtct ctaaatgcaa    763440
     tacttttttt gtcctttttg tttctttgtt tttttgcaac aggagtacat atcctcagct    763500
     ccacagaagc cagggtgtca ctttgggggc agaaataatc ctttcatggt taccaggata    763560
     agagtcctga ggaatcccag ggaaacctgg agagtgtttt ccagttagac tcagggcaga    763620
     gacctccatg ggaatctctg attagaacag gccttgagct ctgacgggag ccaagagaga    763680
     ggctcgccca gggtcagagt ccttaaaacc tgatggtttt cacagctatc ccccctcgtc    763740
     ttgtaaactc agactgattc agttgaccct ctttctgcta atccatttcc ttctctgtag    763800
     gtttgattct cacagttcgc tttcttcttc tcttccctga aaacagacga tgtgttttct    763860
     gtagtcaaaa tcccagggct caggtctgca ggacctgggt aggctacggg gactttctca    763920
     ctcaccattg tccggacact cttgtcttct gtgcatggag gcatttggaa aatgaagtgg    763980
     acattagcca tgaagggaat aatactagtt ttctccaata ggatattgat gtagagctga    764040
     tcttgtgctt ctcacactgt ctcagagttt ggactctcac ctgtgacttt gagaagagct    764100
     ggggatgggc actccattgt gctgtgagct ctgggtaacg ataattgtag aatctgccta    764160
     ggcagtctaa ggtcaatact actcgtcatc aggaaagaca gctggaattc ctgggaagat    764220
     ctgcatctgc cgtccaccat ggagtcccat cgtcttctgt tatgctctct ttgaatcagt    764280
     cccacctaga ttatctagaa cactcttcct gacttaggaa aaataatggc aggctccact    764340
     aacacctgtg ttatgccatg ggagcaacac ctaagctagt gtgtgaatga gtagatgaga    764400
     ctgtggtcta gtcaaggtga caggtaaaat tgattgttgc cattatgata ttttatttta    764460
     tatttggcaa tatagtcatg ctcctattat aaatattctt tgagacggag tcttgctctg    764520
     ttgccagcct ggagagtgca gcagcacgat ctcggctcac tgcaacctct tccacctccc    764580
     aggttcaagc agttctcctg cctcagcctc ccgagaaact gggattacag gtgcacgcca    764640
     ccatgcctgg ctaatttttg tatttttagt atcgacaggg tttcaccatg tgggccagtg    764700
     tggtctcgat ttcctgacct cgtgatctgc acacctcggc ctcccaaagt gctgggatta    764760
     caggcatgag ccaccacacc tggcctagtt ttattaatgt ctgcccatac cagcaactac    764820
     atacctatgg ggacattaat ttacatctgc agacatatgt gtaaatacac aagcctatac    764880
     atacatgtgt agccatttat atttaacatt ataataaaat aattctaaaa tattttctaa    764940
     agaattaaac ttaatgatga gctaaatata aattagagta atctataatt gatttcgatc    765000
     attctctata gtttgcataa atggatgtct atttctaagc cttaacatag tgtattcgtc    765060
     attttaaaat agtcaagaaa aaattacaaa tgttcttgtc acaaaaaaag ataaggattt    765120
     gatgatttga ggtaatatat atgtcaatta actcgattca attattccgt attgcattca    765180
     caaaccataa catacctttg tgccccataa atatatacaa ccaaaatttc tcaattttca    765240
     atgaaatttt aattatatat ttttaaatct gatgcctctc cttggattaa gccacctcct    765300
     caggcttaca gggctcttcc attttctcaa catgttgtta taccagatga gcacaaacac    765360
     attaatttca ttatgcttag ctttaatttt tcaaaacaac ataaaggtga taattttacc    765420
     aatagacgta ttacaaccta ctgtgcatga gaccctttct gtgcttcaag gtttcttctc    765480
     aggactttat atgtattgca aattttcatt tttttctgat atggaatgct gatttctctt    765540
     tattatagat tatcagttta tttttcaaat ttatctctta aaattaattt tttcaaagtc    765600
     ccactcaaac cagggtatta ttagaacttt gacgtgtaat agttggacca actttgagaa    765660
     tacattcaat ttagttcacg tgctctcaag catcttttct tctttaaaag ttgttgtcag    765720
     ttgtaatatc acacaaaata ttagtaattt atgctaatga caggttaaaa tgctgtataa    765780
     ataattaaat tacatttgat tggaaaaaag gaatagatgt tcctcatatc ttgacttttt    765840
     ttcttctcta tgagatgcat gcttattaat tattaagttt aagacgttac ccttgtcttt    765900
     ttgatttctg ggatttaatt ttagtacata tacttgaatg cattgtatta attcatataa    765960
     taatgttcat attttggata gggttttaat attaatttta ttattactga attttaaact    766020
     attctacact ttttttgttt ttatgagata aaatttacat ataagaaaat atgcatagat    766080
     ctggaatgta tcactagaga gtttctggca aatgtgaata cccttgttgt ccccagcacc    766140
     taaggtagct gaagagcaag tccattccca catcgcgggt ctttccctgt gcctgaggtc    766200
     agctcctgca tggggaaatg ttttgatttc tcacactaca gatccatttt tttctgtttt    766260
     gaactttata taaatggaat caaacattat agaccttttt ttggaaagga gctttgctat    766320
     ttttgagatt aattcatgct atttaatgtg taaaattaga tcaacatatt gtcatttatt    766380
     taattcatgg actgactctg atatgaaacc tcaaagtttt catgtatata tggagacagg    766440
     gagaggaagg agaaagattt tatatctgag tcagtccatt aagtaaataa atgagaatat    766500
     tttgatttat tttcttgtcg atgtacttta aatttgtttc cattttatga atattatagg    766560
     caaagctgtt acaaatatat tagtgtaggt tttcctatgt tgtctcattt ttattgagaa    766620
     aatatgaagt atgtatttct ttattttttt atttttgatt attatttatt tatattttaa    766680
     attttacttt aagttctggg atacatgtgc tgaacgtgca ggtttgttac ataagtatac    766740
     atgtgccgtg gcggtttgct gcacatatca accagtcatc tatgtttgaa gcgcctcagg    766800
     cattaggtat ttgtcctaat gctctccctc ccccttcctc ccacccctga tgcatcctag    766860
     tgtgtgacgt tcccctccct gtgtccatgt gttctcattg ttcaactccc acttttgagt    766920
     gaaaacatgc ggtgtttggt tttctgttcc tgtgttagtt tgttgaggat gatgatttcc    766980
     agcttcatcc atgtccctgc aaaggacatg acatcattct tttttgtggc tgcatagaat    767040
     tccatggtgt atatatgcca cattttcttt atccagtcta tcattgatgg ttatcacatg    767100
     gttatcacat tctttggttt tcatttagga gcatatgtct tgagtagaaa tttggtcctt    767160
     tccacacact aagcctcacc ttcctcagag gaagagcaga gattcatcca actttgtctg    767220
     aggtgggagc tgcttctcca caccttggag cttgcagaga cccctaagtg cagcttcatt    767280
     gagtcaggtg tgtctccatg tgggggactt ctgctgccag tgattgctgc tcagttctaa    767340
     ttgtgggttc ggaattagga cacttaggtt atcacgcctc aaaaattacc atcattatag    767400
     agaaaaataa taatacaatg ctaatcattg tcacttatta cttattatta ttggagtata    767460
     agtttggaga gacaaaattg ttaccatata tttgcataaa tgtaatgtca gaatctcctg    767520
     aatgggtctt gaacacttac aaactggata gaagcagatt cttatttttt ttctatactg    767580
     gtgtattttg ggatgtcaac attttgtcca gaatctgtga gtaccaataa ctgtgtgttt    767640
     caagactctt cttgggttct gagacaagaa atgccattac tacctgaagg attgagacaa    767700
     ctttgaacaa tgtcagagca atcatctcta attgcttcta aacttcagtt ttatcaattc    767760
     ttatgctctc tgaattattt ctgattttag ctattaaagg attctctctt tttctcttag    767820
     ttaattgagc taagaatttg tctatttttt tagaaaaaat ctctttgatt tgttgatttt    767880
     tctattgctt gtaaagtcca tttttcactt acttatttca tttactctaa agcctcataa    767940
     acacattggt gacatgtaga tagagaattt gatcgtaaat gccatgtttt tgttagactc    768000
     agctgatcat gtcaggaagc cccctgaggg ttctaggaac tgactgtggc tcccaccact    768060
     ggacaaggat gagaacctcc atgctttggg gatgggagga catgcagggc agtgtgaggg    768120
     gaggagggtt gctggcaggt ccagcattgc ttcccctggc tgcaccactc atgtccttcc    768180
     tcactcagag attcagtcat ttcttcccag gtggaaaaat cagttaactt tatctttgat    768240
     atatggatgg gagcccaaga tagaggcttg tcagtcatta tttctgtgcc tgctgccttg    768300
     ggaatgtctg aatttcttat gctctgtcct gggtctatac cccagtggct cccctactcc    768360
     atggcaccca cctgtgcctg tgcatggccc ctgatcacag cctccgtgaa acatctggtt    768420
     tggctcagga agaacccccc ttgccttgtt ttcctgctgt ccctggtcca catccttctc    768480
     catccacagg ctctcagtgg tgcctcccat ccacactgac cctacatggg gtcatacctt    768540
     gtccaccact tcctctctct tttcttctga gccttgcctt tgattccaac agcctgtttc    768600
     catctcacct ggacttgtgt acaggatggg gtttaccgag caggcttggg ttgtggaaac    768660
     cctgaacatt actgaaaaag tgctttctca agccaggtcc atattcagct cctgggagat    768720
     gatatctgtt ttttgggcat agacctcccg gtagtatttt taaaccactt tattaaagta    768780
     aggtttatat atgtaaaagt atagattatt taacatactt aattcattga gtttgaagta    768840
     tacatctgca aagctgtcac cacaatcaag gtcataccca tagcactcac ttccaaaaca    768900
     ttcctcttat ctctattact attattatta gctaaaatgt atttttgttt gtgtttgtct    768960
     gtgtctctct gtgtgtagcc tgggtgcaca ctatactagg tgttttcata atgacttatg    769020
     gtgaatgcct attattgctc aagaggccgg cgtctgagca ctggaggtca gttgtacagg    769080
     tgccacacac atctgtgatg ggccccaata aacagcactg ggcaccagca ctcaggtgag    769140
     cttccctggt ggacaatgct tcacacatgt tgtcatgcat cactgctggg agaactgggg    769200
     acaagagact ccccagggaa agaacatctg agagcttgtt cctggattct cagggccttc    769260
     cccttgctgc ctttgttcat tttaattcat attcatttgc aataataaag ctgcacccat    769320
     gagaataaca gctcgtcttg agtcctttac ttctattatg tggcctgagg atggtcttgg    769380
     gatgctcaac acaattacat cagaggtgga aattgctaga aagaccctga ctactgacac    769440
     atggctagag cattgtttat agtatcaaag gatgagaaag tgtgggataa aagacattca    769500
     atacctggat gtctgcagaa tcacatggca ggagatggcg tgtgtgctcc agtaaagagg    769560
     aggaagtgaa agtaatcaat ggaatttgga aatggcagac acagctccct aggagttgtt    769620
     ccctgaataa caaaaagtaa aaataatgag gaacaaccca aatattcaag tcatatctgt    769680
     gatagctaaa ataatagtaa aaagatgcac atccagcctt gcactgtgcc ctatgagctg    769740
     aggctactag actcccattc accaccaaag gggacagttg tccagaaaca acacacaata    769800
     cacaacagcc acacagaagg tagcccaggg gctgggctgg ggcagagaat ccactagaca    769860
     actagaacaa ggaggacagt gtggaaagtg gcatcattct gtgatatgat tgatatcatt    769920
     aactgatatc atcggattgt gaacaatatt agccaatgga ctatgagagt gactgatgta    769980
     gcagcttgtc tttggcttca tgtgctgcag aaaggaggag tatgtttggg aggatgctgg    770040
     acccacagct cacaacagac acatcacaga tggctctagc tgattgcaac ctcaaataag    770100
     atgttcctga tgggtcagct ttcctggtgc actggattcc agattgtgta aaatttctct    770160
     accctgagaa tgagactgcc cttcctctct ttgaaatgca aagtgggctc tccagatagg    770220
     gaggctaatg ctgcacacgg aagccctgct gctgtgtgtt tatgttgatg ctgacagtca    770280
     ctgcttagca tgcaccttac ccaggtcata gcctgtgctg tgtgtttatg ttgatgctga    770340
     cagtcattgc atagcatgca aattacctag gtcatagcct gtgttgtgtg tttatgttga    770400
     tggtaatggt cattgcttag catgcacctt acccaggtca tagcctgtgc tgtgataaag    770460
     agaagtcatt ttgcttctgc accccaggta tacaattcct gtgggtaatc tggcaaatat    770520
     tttagaaact gtgtagtttc tgttgccttg gcttttcatt tggatctttt agatgttaag    770580
     gaaatgagag tggttcacag gaaaatgggg caaatacagg ggacagtaaa aagccccctc    770640
     cgagcaatgt ggatatcata aaataaaaaa aataagaata aaagaactaa acagattgtg    770700
     ctggggtgga attaaaacag cactaccagg agttgggtgc agcaatgggg atcccttcag    770760
     gtccatcagc aatacagaga cctaaacaaa tatttcctat ttaggttgaa ttggcagagt    770820
     gtaaaatcta gaagatgaaa tggccaggaa acatttgtcc ttgattttgc atgggtgaag    770880
     gtctggcagg ttaatcaaat tgagggttgt taaaggcctg gggtggtcct gccctttctg    770940
     ggtgcccagg ccatagtcac attcatggga aattgccatg gaataaagag tagttttttg    771000
     tggtagttct tgttatcagg cataagtgca tttgaatttt ctgttcacgg cctttcctgg    771060
     ctctatttct cattttttta acagacatga ttttatctag atttgtcaac tttcacaggg    771120
     tcacagagaa gggtggaagg agggatgccc tggtttgggt cttgcaggac cacagacaag    771180
     agtggggagg ggcaagaaga ggttgtaagg aattattgag acctattctg cccctcccag    771240
     gaggctcagt tcagcctttt ctctgcaact gaggttctgg gttataaacc ctgtagactc    771300
     ttcccttcag gtcagggcgg caactatgca aatgcaagtg ggggcctctc cacttaaacc    771360
     cagggctccc ctccacagtg agtctccttc accacccagc tgggatctca gggcttcctt    771420
     ttctgtcctc ctccaggatg gggtcaaccg ccatcctcgc cctcctcctg gctgttctcc    771480
     aaggtcagtc ctgccgaggg cttgaggtca cagaggagaa cgggtggaaa gcaactcctg    771540
     attcaaattt tgtgtcttcc atacaggagt ctgtgccgaa gtgcagctgg tgcagtccgg    771600
     agcagaggtg aaaaagcccg gggagtctct gaggatctcc tgtaagggtt ctggatacag    771660
     ctttaccagc tactggatca gctgggtgcg ccagatgccc gggaaaggcc tggagtggat    771720
     ggggaggatt gatcctagtg actcttatac caactacagc ccgtccttcc aaggccacgt    771780
     caccatctca gctgacaagt ccatcagcac tgcctacctg cagtggagca gcctgaaggc    771840
     ctcggacacc gccatgtatt actgtgcgag acacacagtg agagaaacca gcactgagcc    771900
     cgtctaaaac cctccacagc acaggtgcag aggaagctgc cagagacaca ctccccaggg    771960
     gcctctctat tcatccaggg aggaaacact ggctgtgtcc tgaggagtga gaaccagaga    772020
     acaacgtggg agggttccca gtccctaagg caactagatg gggcacctgc tcctgggagg    772080
     tggattctct gagggggctc ttgttctaca aggttgttca tggtgtatat tacatggtta    772140
     aaataaaaaa gctgtctaat atgcacctct taagcatgac agtcttttaa ttagtgaaaa    772200
     gtctacacag ttaatcattg ctggcatgct ttcctccctc ctatcttctc ttgctcccta    772260
     catctattat tttctactta atttttcaaa atcatttgat ccctttgtga actattacta    772320
     ggttctgttg tttttgttgt tgttttcctt tcaataatat gtactgaata attcatcttt    772380
     gtgccaattc ataagttctt gggtataata aatacttctc tcataaaaat tagataaatt    772440
     aaaataaata aattttaaaa aacatacaac ctatcaaaac tgaaccatta agaaataaaa    772500
     aatctgggct ggttgtggtg ggtcatgcct gtaatcccag cagttttgga ggctgaagca    772560
     ggtagatcag gagttctatg tgaatctgtg gtcaggagtt caagatcagc ctggccgaca    772620
     tggtgacacc aagtctctac taaaaatata aaaattagct gggtgtggtt gtgttcacct    772680
     gtagtcccag ccactcggga tgctgaggca ggagaatggc ttgaacctga gaggcagagg    772740
     ttgcagtaag ctgaaattga gccactgcac tccagactgg gaaacagagt gagactccat    772800
     ctcaacaaca acaacaaaaa aatctgaaca gaccaatgag taaagagatt gagtcagtga    772860
     tttttcaaac atctcaaatc aaagaaaagt caaaaatttc atggctccac tactgaattt    772920
     tatcaaaaat taaaacaaaa taactagaat cactactcaa atttccaaca aaataaagag    772980
     gaagaaatac attcaaactt attttggaag gccctatttc caaagcaagg gaaagacact    773040
     acaagtaaat gaaactacag gctaatatcc ctgattatca tagtttcaaa aactctcaag    773100
     agtgatgaca aacaaaattc aacagcacat tgacagtata attcactatg gtcaggtgtg    773160
     gtttatctct aggatgcaat gaagtttcaa cctgcagaaa taaatgtgat atatcaaatg    773220
     aaaatattga aggactaaaa ccatatgcac catatgtcca tgtcaataga tgcagaaaga    773280
     gcctctgaca aaatccacca cactctcatt ataaaaaatc tgaacatatt atgcataaaa    773340
     gatatacagc tcaacataat aaacaccaca tatcagaagt gcacatctaa cattatcctc    773400
     aatgatgaaa attttatttt cctctaagac tagaaactag acaagatgct cactatcacc    773460
     aatattatta aacacagcac tgggtggtct agacaaaata ggccagaaga aaaaaataga    773520
     agtcatccat atagtaatga ataaatttta aatatatgtt ttacatatta tatgctcttc    773580
     tttatgcaaa gccctaaaga ctccaccaaa aaaggctgga tgtaatgaag agattcaata    773640
     aagttgcaga atacaaaatc aaacttacat ttcaagatgg caggttagag gcattgctag    773700
     tatacctctt cctcttggaa ggacaaactg atatggagag atgaacattg tggatttatt    773760
     ttcaagaagc aatgcaggaa ctgaacaaaa acactgaaat aatctacaaa ctttccgaaa    773820
     aaagcaggaa gctgcagcct acactgtgag tcaggtgaag ggctgcgagt ccccagagtt    773880
     tgagggaaga gtttgcctct gggatacaca tccacacctg ggatcctgaa agtccaggcc    773940
     aaaggagaag gccccaacct cacccagtgc aaggacccac tgtgcagggt tgtggaatat    774000
     aaaagtagga acagcaggaa gacccttgca taaactctca gatctcagtg cggtctgaga    774060
     gctgccattt cttaaggttc ttcacagagg accctgggaa gaactgccaa aaatttcagg    774120
     caggggtcac aggttgaaag aagctctcaa caggggtgtg tgatataacc tagaggggac    774180
     gaactccctt ggccagagtc tgacaagtga agagtgaaaa gtgggctgca agttcaggag    774240
     ccatgggtgc aggagctggt gcctggcttt gcagcagaca ggaaggagca tggcatgaaa    774300
     cccatggctg cagtctctat ggggacagct tataactcct ggcatttgca gatattgatc    774360
     acaggctgac tggaactcat ctcactgctg ccaatggaac accacaggag tggctctgcc    774420
     tcaccaagtg tgtgggaact gtatggggct taccgtcacc tggctctcac cactccctgc    774480
     acaaacttct atgcagcaga agcagatgtg ctgacctctg gaacatcaac ccagtggcct    774540
     gagaaccacc cctgtccccc acacccacag gggctgctgc ttgccctgca cacagagact    774600
     cagagcagaa acccacctga cctagccctc accttgcttt gtgcagccac ctgccctggc    774660
     aatttaacac aaaggacagc atcttttggg agctacatag ccacacccac tgcctgagaa    774720
     tccatagtag cccccattcc tgggcaacat aaggcttgca aaaatcccac tgctaataat    774780
     gcagctggtg ctctttcaca aacaccacct cttagctgga ggccaaccaa ccatgcatta    774840
     cagtatctcc tggtagagta acactgcatc ccagaaggag aaaatgactg tgcgatctca    774900
     gctgtcacca tggtctgcac cactctggat gaacgggggt cccgagtctg tccatgtgac    774960
     cagttcatca ctactgtaac cagcattcaa gaaagccagc ataagaaggc catcaatatc    775020
     caaggaattt cacagagtct ccttcactcc cctgccactc ccatcagacc cggttctgct    775080
     gctcactgtt gaaaaatgtg aggacagttc acatcaccgg atcacttaca gatatttgcc    775140
     aacaccagct tatagacagc ctttctggaa tacatcatgg tgactgcatt cctcagaagg    775200
     cgcactctcc acgttctggc ttgcaagaga cacagagtca caattcccct ctattcggca    775260
     catcaacatt tgtacagatg aaaagacatg cctgtcttat ctgaatagct ggaatactgt    775320
     ctgaaaagtg gatagatttc ctactgatct ggcaagagag ctgaggttgc tccaaccctt    775380
     ccccgtgata agacctcagt gtgactcact aaaagctcct ccagccaccc ctgtcaaggc    775440
     tgggacttaa tttacccacc tgttttagcc acaactgctt tctacccagg gacaagtccc    775500
     ccattggtct gaagcctgaa ccaacaaaca agtaaacaaa tcactgggga aaagcaaata    775560
     aacacgggca tgccacagat gaatgagata agatttaaga gacctcaacc attataaccc    775620
     catagaagac aatgaacttg ctcacacact gagttgattt ctactgcaac caccacatag    775680
     gaaagcgatc acgcaaagac tctgtataac cagggagctc ctacagagtc ttcattcccc    775740
     taaggaccaa aaatcaaatc aggctgtaat taattataag cataaaaaat ctcattctta    775800
     agggaaaaat acatttaaaa acaaacaaac agtcaaatca aactagaaaa tctaccaaaa    775860
     caagaagaaa tgagaaaaat aacttgggaa atatgagaaa acagggtggt ataacagcgc    775920
     aaaaaggtca cactacctct ccagcaataa accttaacca aaatgaagtc tttaaaatac    775980
     cagaaaggaa aatcagaatg ttgattaata agctactcaa gaagatatca aaaaaggtga    776040
     aaatcatcat aaataaattt atataagcag ttcagaatat aaatataaat ttttatagag    776100
     agatagatat cctaaagaga aaccaatcag aacttctgaa aataaaaaaa aaaatgcaga    776160
     gaattagtaa gggaactaca aaatgccatg aaaagtttta aaaatagact agaaaaaaag    776220
     taaaatatta ggagaaaaag aaaaaggaaa cagtagaaga aaaaaagaag aaaaaagtag    776280
     aaagactaga ctatttttga aataacccaa tcagataaaa ataaatataa aaagagccaa    776340
     aagaagtgag caaagtctcc aggaaatatg ggattatgta aaacaaataa acctaagaat    776400
     aattgatgtt gctgaagaag aaaaaaataa taataagttt ggaaaacttc tttgatggaa    776460
     taattgagaa ctactcccct gggctgacta aagacctaga tgtccaaatc caagaagttc    776520
     aaggaactcc tggaaaactc attgcaaaaa taccttcacc aaccatacag tcattaggct    776580
     acctaaagtc tacatgaagg aaataatcta agagcagtaa aacaagacaa aacaaaacaa    776640
     agaaatccat cagactaatg gcagacttct cagcagaaac ttcacaagcc agacagatta    776700
     aggttctatc ttcaaactcc ttaaacagaa caactttcaa ccaagaattt ttttttatcc    776760
     tgtcaaacag ttttataaat aaaaaaagaa atagtcattt tcagaaaaaa aaatgctgag    776820
     caaattcgtc actagcaaac cagcactaca agaaatgcta aaaggagtcc taaaccttga    776880
     aacaaaagcc caatacgcac aaaaaatgga acctcttaaa aattaaaaac tcacagggcc    776940
     cattaaacaa tgacacaata taaaagaaaa caaaaactag gtaacaatta acatgacaaa    777000
     gaaaatagta actcatatct aaatattcac attgaatgta aatggcctaa atgctccaca    777060
     aaaaaaatat gcaactgcgt actagattta aaaagtcaca atggaaatac ctgatgtctt    777120
     taagagactt acctaacacg caaagattta tgtaaactca aggtaaaatg gtggcaaaag    777180
     gatttcaaca aaaatagaaa ccaagactga gcaggaataa ctattctgat atcagacaaa    777240
     atggacttca aagcaacaac aataaaaaaa aagacataga tgatcactat acaatgataa    777300
     aaggatcaat tcaacaaaaa attacaatta tacatttata tgcaccaaac accggcggaa    777360
     ctagattcat gaaataagta ctactagacc tcaaaaactg agttagatag caaatcaatc    777420
     atagtgggag acttctatac aataatgaca gcaatagaga gatcttcggg acagaaagtc    777480
     aagagataaa caatgtcctt aaatgactca ctggaagaaa tggatgcagc agatatttac    777540
     agaacactct atccaagatc tgcagaatat acattcttct aatcagcaca cgcaacattc    777600
     tccaaggtag agcatgtaat aggccacaaa acaagtctta ataaaattta aaacaatgaa    777660
     atcatatcaa gtatcttctt agaccatagc agaataaaac taaaaatcaa ctttctaaag    777720
     aactttcaaa actgaacaaa tacatagaaa tgaagaaatc tgcttctgaa taatatctag    777780
     gttaacaatg acatcaagaa gaaaatttaa aaattatctt aattaaatga taataatgag    777840
     acaagttatt gaaacttcaa aaataaagca aaaacagtga taagaggaaa gtttatagtc    777900
     ccaactgcct acatcaaaaa gtctgaaaca gcatgtcaca actcaagaaa gtggagaaac    777960
     aagagcaaac caaacctgga ggcagaagaa gaaaagaaag aacaaagatg agagcagaac    778020
     taaatgaaat tcaaacaaaa aaatacaaaa aaattcaatg aaataaaagc tggttatttg    778080
     aaaaaataaa caaattcatg gatcatcagc tagattaacc aagaaaagaa gagcaaagat    778140
     acaaataagc tcaattagaa gtgaaacgga cattacaatc tacataacta atataaaaaa    778200
     taatttagaa ccaccaagta catgttcatg tacacaatgt agaagactta gaggaaatgg    778260
     tcaaatttct agaaatctac aagtctcata gattaaatca ataagaaata gttactttga    778320
     atatataaat aacaaagagt gagattgtat cagtaattcg agaattgcca acaataaaaa    778380
     caacaacaaa tagggccagg tgaattcaca gttgaatgtt atcaaaaatt tacagaagaa    778440
     ttgctaccaa ttttgctgaa acaatttttt taatttagaa aaaaagaatc ctccctaaat    778500
     tattccatga agctagtata accaagatac caaaaccagg aaaacacaca tacacacaca    778560
     cacacacact ctctctctct ctacagatga atttccctga taaatataga tgcaaaaata    778620
     cacaaaaaat aatagctatc tgagttcaac agcacatcaa aaatacaatt cctcatgatc    778680
     aagcgggttt catctcagaa atgcacaatt atttgaacat acacaagtca ataaatgtag    778740
     tacatcacat aaacagaatt gcaaacaaaa accttatgat tgtctcaata gatgtagaaa    778800
     aagcattgaa caaaattcag catttttcat gataaaaacc tctaaataaa cgaggcatag    778860
     aagaaacctg actcaaacta ataaaagtta catatgaaaa acccacagcc aacgtcatac    778920
     tgaatgcaaa aaagttaaaa gcatttcccc tgagaacaca aacaatacaa ggatgcccac    778980
     gttcaccaat tttattcaac atagttctgg aagttctagc cagagcaatt agtcaggaga    779040
     aaaaaaagta tccaaattaa aaaagagaaa gtcaaactat cactgtttac agatcaagtg    779100
     attatatcct tagaaaatcc tagactcctc ctaaatagtg ttagttttag taaatgaatt    779160
     cagctaattc tcaggttaca aaataaatgt acacaaatta gtagcaccac tgtacattaa    779220
     caacaacaaa gctgagaatt caaccaagaa ctccatccag tttacagaag ctgtaaaaac    779280
     atagaatatt tagcaatata cttaactaaa agggtaaaag atctctacaa ggagaactac    779340
     aaaacactgc tgaaagaaat catagatgac acaaacaaat ggaaatgcat cccctgatca    779400
     tggattggaa gaattagtat tgtgaaaatg accatactgc ccaaagcaat ctacagattc    779460
     aaagcaattc ttaggaaaat acccacatta ctttctacag aattaaaaaa caataatgct    779520
     aaaatttata taaaaccaga aaaaaagccc aaatagccaa agaaatccta ataagataaa    779580
     aaattggagc catcacatta ctgaacttca aattatacca caaggctgca gttaccaaaa    779640
     caacatggta ctgatataaa tgtaggctta tagaccactg gaacaaaata gagaacccag    779700
     aaataaagcc acatatgtaa agccaactgt tgttttgcaa aacataccaa aatatgaatt    779760
     ggaaaatata cactgcattt aataaatggt acagggaaaa ctagcaagcc acatggaaaa    779820
     gaataaaact ggatttctat ctgtcaccat ataaaagacc aactcaagat gaatcaaatt    779880
     cttaaatata agacatgaaa ctctaaaagt tctagaaaac aatattagaa atcactttta    779940
     cacatcaggg taggtaaaga acttataaat aagaccccaa aagcaaacgc aacaaaaaca    780000
     aaaataaatt tatggaacct atttaaacga aagtgtttct gcacagcaca aataatagtc    780060
     aacagagtaa aaagacaact cacagaacgg gagaaattat gtgcaaactt cacatctgac    780120
     agaagattag tatccagaat ctacaaaata ttaaaacaag tcgccaagaa aaaaacaaat    780180
     aatcccattc aaaagaggac aagggacata aatagatagt tctcagaaga agatatacaa    780240
     atggccaaca aacatataaa aaactgtgta gcatcactaa tgatcaggaa aatacaaatt    780300
     aaaaccacaa tctaataaca cctaatcctg caagaatggc cactataaaa agtcaaaaaa    780360
     caaaagggaa tgcttataca ccgctggtga aaatgtaatt tagtataacc actgtggaaa    780420
     acactgttga gatttcctga aaagcaaaaa agtagatcta ctgttttgtt ttgttttttc    780480
     ttttttaaga tggagtctcg ctcagtcaca cgggctggag tgcagtggcg caatctcggc    780540
     tcactgccac ctccgcctcc caggttcaag agattcaccc gcctcagcct cccaagtaat    780600
     tgggattaca ggcacccacc atcatgcctg gctatttttt tttttttttt tttgtatttt    780660
     tgtagcgatg gggtttcacc atgttggcca agctggtcta taactcctga cctcaggtga    780720
     tccttccccc tcggccccct aaagtgcgga aatcacaggt gtgaaccact gtgcccggcc    780780
     agatctactg tttgatccag caatctcagt actctttatt tcaaaggaaa taaagtcatt    780840
     atatgaaaaa gatgtgtaca tgtatgttta tagcagcaga atttaacatt gaaaagatgt    780900
     tgaaccaatt taagtgccta ttgactaatg agttcataaa gaaaatgtga catcgcctgt    780960
     aatcccagca ctttgggagg ctgaggaggg cggatcacaa ggtcaggaga tcgagacaat    781020
     cctggccaac atggtgaaac tccatctctg ctgaaaatac aaaaagttag cagggcatgg    781080
     tggcgcacgc ccgtagtccc ggctactaag gaggctgagc caggataatt gcttgaacct    781140
     gggaggcaga ggctgcagtg agccgagatc aggccactgc actccagcct gggtgacaga    781200
     ggaagacact gtctcaaaaa aaagaaaaag aaaagaaaat gtgacatact tacaccatgg    781260
     aatactaata aaccattaaa gggaatgaca taatgtcttt tgcaacaact ttgatgaagc    781320
     ttgaggcaac tattataagt gaagttacag cagagtggaa aagtaaaaac tctatgttct    781380
     cacttataag tgggagctgg gctatgaact tgcacagaca tacagcgtga tataagggac    781440
     tttagagact tagaagggaa ataataggag ggacactaga gagaaaataa atgcacttta    781500
     ggtacagtgt acactattca ggtgacgagt gtactaaaat ctcagaattt attgctatgt    781560
     aattcatcca tctaacaaaa aaaatcttta cccctaaagc tattgaaatt aaacaaaagt    781620
     caacctacaa aaataagtat cttccgtata cactaaaaaa tgattattca aaattaaaat    781680
     caagaaaata aatctaaata caacagctta aaattcataa aataaatttt accaaaataa    781740
     atttaacaag gatacaaaac atctgcacat taaaaattat aatatattga ttaaaatgga    781800
     agaaaacaca aataaatgga aggctatctt gtgttcttgt gttactggaa catacattgt    781860
     taatatgtcc accctaacct aagcatttac agattcaatg caatcccatt caaaatttca    781920
     aagacattca ttcacataaa ttgaaaaaaa aattctctaa tttgtatttc tggggtgact    781980
     gaccctgact atcaagttga aatcagacta ctactccaca atggaggtaa gaaacagtat    782040
     gtctggaata caggagattt cttagggcat ctcttagtat tactttacca tgggattaag    782100
     gtcaatggga aactacaaca acctaattca ggcaggaaca caaatggccc aggtacttca    782160
     gaaataaaag tttgggtcac tcaaccaggt aaaaaaaacc tgactagcca aggtacttat    782220
     tgaaggcaaa aaaaaaaaaa aaaatacaga atgcgtgcta gaagaaggta gttatcaata    782280
     ccaggaacaa gcatgctacc agaagtgagg actctaattg ttatgagtat ttcctcctta    782340
     tcttgttaag aatatatttc tgcatgtata taactgtgct aagaaaatat cttcaccgtg    782400
     gcggatgtgg tggctcatac ctgtaatccc agcactttgg aaggccaagg tgggtggatc    782460
     acctgaagtc tggagttcga gaccagcttg accagtatga tgaaaccctg tctctaccaa    782520
     aaatacaaaa attagctggg cgtgctggca ggtgccttta gtcccagctt cttgggaggc    782580
     tgaaacagga gaatagcttg aacccttaac ccaagaggca gaggttgcag tgaaccgaga    782640
     tcatgccact gcactccagc ctggatgaca gactgagact ccgtctcaaa aacaaacaaa    782700
     cgaacaaaaa tatattcatt gttttcattg aatttgtttt ctttttatca tgtatcataa    782760
     gatttattga tttcatatca gcatttaaat gttgttaaac ggatgtagtg gcatttaggt    782820
     taaggattag tgcactttca gttgtatgaa ggatagctgt attatgttag gcataattat    782880
     gaccttatta ttgtctttat ttggagatca agcatgattg cagctagatg tgtaggggtg    782940
     ctaagttgac aagaggtggg cttttgatgg ttaatactag gtgtcaactt gattggatta    783000
     agggatgcct agatggctgg gaaaatattg tttcttggtg tatctcttgg ggttttgcca    783060
     gaggaggttg atatttgagt cagtggactg gaagagaaaa cccaccctca atgtggatgg    783120
     gcaccatcca atcagctgcc agcacagcta gaataaagca ggtggaagaa gatgagatag    783180
     cattggttgc tgtgctttat gtttttgttt tgttttcctt gctggctgcc tttttgtttc    783240
     ctcctgccct tggacatgag acttcaggct ctttggtggt tggatgccag ggcttgaagg    783300
     aggggcttcc tgagggctcc tgggcccttt gtcagagact caagggtgca ctgttggctt    783360
     ttctagtttt gagacttttg gatttagact gagccactac cagtttgtct ctttccgtgc    783420
     ttttagaaag cctatcatgg aactttcatc ttgcaatttt gtgagaaaat tctttctaat    783480
     aaaatattta tatatataca tatgtcatat tagttctatt tttctggaga accctgagta    783540
     atacaacttt gatacacaaa acaacattgt tgtactcaca aaaatgtatg ctaactaaaa    783600
     taagccaaca agtaaaagtg cataaagtat aaatctattt ctacaaacca caaaaaatga    783660
     acatgaatct aaagtaacaa aaaagatcag tagttgctta gggacagagt ggaaaattat    783720
     attttttcaa atgctaaaaa taacactctc atctactatg aacattgctc tctcctcagg    783780
     tatttcaccc cagaactgac tctacaacag gagacatgca aacaggttcc tgttcatgta    783840
     ctaacacttc ctagcacttc tttttcatgt acttctttga cttatttgtt gatgaaagtt    783900
     gataccataa aaatatctaa acagggttca cattagagaa aaagaagaga gtgacaggaa    783960
     gattaagcca gagcatccag ttccagtgat tctttgatcc tgccctccct gaaatctaga    784020
     agcagaggga tgagccatgc tgagcagagc acacttgtcc agggagaaag aggagaaaga    784080
     ggtgtaaatg tgtccttccc atgctccctg tccataaagg gaacgtcaac tgatgtctgt    784140
     gcccttcctt agtgtctaca acatagagtg tgtgctaatc tgtgcttcac tttttatcac    784200
     tctcagaggt agggcttctg tggatcaaat acagctgtgg ctgctcctca gcctgttccc    784260
     tctgtattcg taatagtcct acatgaagtt cattggtgga atctcagata cttttttaca    784320
     aagcctctga atttaccttc attgactcca tcattagctc agcccagtag tcttcaggcc    784380
     aggggctggt gttggggggc aacagtgagt gatttgagtg gaatttgtta gtggtaccct    784440
     ccatcaatac aaagaagaat cataatcctc agggacaccc ttgtcagcac agtctccctc    784500
     aaaatgacca acctgagagc cgagaacaag gtcacgtgtt actgtgagag acacagtcag    784560
     cagacaccag tgtggtgagt ccagacgcaa acttccctgc agggaaacag gaggggactg    784620
     caaagtaggc atgctcagac ccaccagtgg gcgatgggga cacacaggag tgctcagaat    784680
     ccagcagggg gctctcagaa caaccaggag gcacttagaa tgctggggga agctcaggac    784740
     accaggggcc acacagaacc accggggggc actcacgaca caagggggtt ctcaggaacc    784800
     accaggagat gctcggaacc accagggggc gctcattaac cactatggga cactcaggac    784860
     taaccaggag acgttcagga accaccagag ggcgctcaga accgtcagag gacagtcaga    784920
     accaccaggg ggcgctcagg acaccagggg ttgctcagga ccccaggggt tgctcaggac    784980
     cccagagggt gctcagcaac aaccaagtgg tgctcagatc caccaggggg cgctcaggaa    785040
     gtgagggggt gcccaggaac caccaagggt cgcttaggaa ccacatgggg cactcagaac    785100
     caccaggggg cgctcaggac accctgggga gctcagaacc accagggggc aatgagaaca    785160
     ccagagggcc ttcaggaaac cagggttctc ttagcaggca gctcctcatc aggtccctgg    785220
     ggagggtgag tttctttatt taggtcttgt gattcctgac ctggtgaagc aaacgtcttc    785280
     ccagggtctt ttactatttc ttctttagaa tccatggttt ctctcaccta caaaacctta    785340
     acttagaaca ggagttcaat taaactatta attcttaata ttgttgtaat aatactagta    785400
     ataatatctc agtacaattt taaaatgaag aaattgtgta tgcttacttc aaattatttc    785460
     cacaaacagc ttttcgtttc tgtgctgtgt cagtcacact atcataaagg gtttctaaca    785520
     ataactcagc atatgcatgg tgctgagttt ctttctcttt catcctctga ttgtgacccc    785580
     tcaggctgtt tccccacctt ccctttttct gtccaaacct ttcatattcc tcaatttcca    785640
     ttcaaggaac cagcaagtcc gtttacattg cctcatccgt gtctggtgaa tcagttcact    785700
     ttccttcatg atcaccgaag ccaatcaagt atgggcaggt aaatgttctt agaatatgtt    785760
     cattcagact cactgcccac ctcacccctt tctagggtcc tgcagacatc acccacaccc    785820
     catctcctcc cttccctaat taacagagtg ggctctgcag ctcctgctgc cctctgtgtg    785880
     ctcagccctg gggctcacta gggctttgat gatgaagtcc acatctctaa agtatttgcg    785940
     cactctcaga ccaccctcta gcaagctgct gttgtgaatg aatcctgaga atcctgggca    786000
     gatttcagtt ccacattgct ggatgttctc tagcattcag gagtattagc aaataactac    786060
     cagagtgaaa ttggatgcaa aaaaatttaa aggaatactt ttaaatagaa atattttaat    786120
     ctgactttta tgaaaataca atgataatac aatcaataat gtaataaaat tttaatagta    786180
     atgtttatta cttattcaat ttgtgaatga ctgatattca aaaaggatgc tgctaacaca    786240
     aatgagaaat aaaccaacac aaagttcata aataaatacc attgtacatt aaagtaatat    786300
     aattttcaag aatggcattt atgttaactt ttaccagaac atgcattgaa atacatttgt    786360
     ttattgacat tggaatagac agaaatatat atatagaaga acattatttt agactacaat    786420
     ctcaattact ataaacacaa taatttcaat tcaatccaat aattaaaata ttatgcaaaa    786480
     agtgtgcctt ctttttgtgt ttgaatatgc atttattttt ggttttgcac atatgtgtat    786540
     cattgttgag catgtgtggt ttttttggtg tgtgtgtgtg tgactatgaa gtaggaaata    786600
     cattattaac ttatagggtt ataacaatct aagttttgcc atgttaaaag ataataaaga    786660
     acacacaagt aaaaaatcat agtgtatttc aaatgcctca gacttccaca gaagtcatat    786720
     caataatgaa catactagtt caaggttttg aaaaagacac atgtaataag atatgtcaaa    786780
     ttgaattaat agaatattgc tatgcagttg agaaaaacat catacaatca gtctcactac    786840
     tggccaaata aatgcttatg ataataatac ctgtagtcca tcctattgta gagaataaaa    786900
     taactatata aatgccactt ccattactag aatttagtat tagatacttc taatattttc    786960
     ctgatacaaa taaacatttg tcaatgtctt agacatttca aaattatttt aaaaaataat    787020
     ttgcataatg tttgtgatag tatcattttc aacaacctat tccataagtt cttatggttt    787080
     ttatttttat catagtgctt ttgcagttta cacacttttg atttgtaata agtatagatg    787140
     accagaacaa ttttagaaga aatacatagt gtgttccctt ccatctccaa gtttcttcta    787200
     aggttaactt ttacataaac atggcacatt ctactaacac atttatacct atggatttat    787260
     atttattaga ctccagacat tatttgagct tcaacaattt cccaatattt ttcttgtttg    787320
     ttttcctttt ttttttaaat atgaggtctg actccactct tgcttttgtt tgtttctttg    787380
     ttccaaggct caacctgggt caccatattg aattcaatca ctctttacct ctaatctttt    787440
     tgggttaatt ggagtctgca ttcctactgt tttccagttg aacattttat acatgaaaat    787500
     tagccaagta ggtttttact gccatttagg taacttaaat cttttcatat tttttcactg    787560
     tctgttcttt caatcacaat tggtactgaa ttttacattt actataaatg ttagtttatt    787620
     ttctgaaaca tttgttaaat cttttttgat gtcttctaga aaagtaaacc ctttattctg    787680
     tgtaatattt ttcttcagtc ttaacagtat attttctaat gaccacttat tctgaaatta    787740
     aaatagcttc tcaataattc ttttcattca tactttcatg gaacatgtca tcacatcttc    787800
     aactttctat ttgtaaaaat acagagtaac tttcttgtaa aaacctaaaa gttgaatctt    787860
     ttttttaaga agctatgaca aattatcctt ttaaactttt atctcttttt ccggttaatt    787920
     tttttttttt tgagatggaa tctcgctatc attgcccagg ctggagtgca atggtgagat    787980
     ctcagcttac tgcaacctct gcctcctggg ctcaagcgat tctcctgcct cagcctccca    788040
     agtagttggg attacaggca tgcaccacca tgcacagcaa attttgtatt tttaatagaa    788100
     acagggtttc tccatgttgg tcagtctggt ctcgaactcc tgacctcagg tgatccgcct    788160
     gcctcggcct cccaaagtgc tgggattaca ggcgtgagcc actgtgccca gcctgttaat    788220
     tcttgcttta ttttggcatt ttattgtaaa gtttatccta ttaacttact tatgacttaa    788280
     tttattttta ttaatgtttt gttggctacc acaaggtttg caataataat tttatatcat    788340
     cagattctaa ctcagataat gatatttttg taccagttgt tcccttctac agcttcaata    788400
     ttctccactg ttacaatcaa tcaccttcta gatctgctgc catgccctgc agattaagga    788460
     ttttattcca tgacagagtg gaggtgcaac ttgatggaac tttgatgaga acctcggttt    788520
     ttatcccatg tcctttaggg ctccatcagt acctctggaa taatggtttc agtggcttgc    788580
     ccctgcaggg tagataattc cttatttcta tagtgctaag gagggattgt gtctgaatgc    788640
     attttagaag tatgtggcct ccctctgtct tagaaagaca ctttgggaaa taaagatttt    788700
     tctcactgtc cccattctag ggtaaaggga ttcactagga taggaatgct gatcaaagaa    788760
     aaccttcagg caaattaaat ttaaaagagt ttaattgagt aatggatgat tcatgaatca    788820
     ggcagccccc agaatagatt cgaagaggct tgaatgtagc cacatggtgg aagaagattt    788880
     atagatgaaa aaaaaatgat gtacagaaat tagaagtgag gtacaggaat gactggattg    788940
     gttacaggtt ggtgtttgtc ttatttaaac acagtctgaa cactcaaggg tgtatgagtg    789000
     gttaaggtat ggctactgga attgaccaag gctcagctat tgttacaggt gcatactcct    789060
     aaattagggt ttcaatcttg tccacacatt caggtaggtt acggtttgtc cacaaggact    789120
     caatcataga agtacggagt ccttctcagg ccatatttaa ttcacttttt cagtgccctt    789180
     cagtatgtgg ttcctgagaa tttcacacga caacacgttt tccacactgg aatttaagca    789240
     atccaacacg tatttgtctt tatcttgtaa taggctatat ttcatatggc agcctctgcc    789300
     tcagtttagt taacaccatg gctttgtttc tctcttggag aaacaagaac ttgtttctcc    789360
     caagatttcc gtgttcgtga aaggaaaata tacctttggg acccccaaat cactaagcca    789420
     aatgtgaaag tgaagctgaa aactggggca aacccacctc ccattctttc cctaaaatga    789480
     tagctactaa ggtttttaaa agctgcatac ctccttcccg atttgaccac aaggaaaatc    789540
     cttatgaacc aaggacagac agaataattt ctctgctcac gtaagtcaaa tgcatatctg    789600
     attgctacct ttgctctatt gtttcgctgc gtgaaaaata ggcctacgtg actattcctg    789660
     taaattgtgc attcagttaa tggctaatca gaaactcaaa agaatgcaac catttgtctc    789720
     atatctacct atgatcttga agctctctcc ccacttcgag ttgtcctgcc tttctgaacc    789780
     aaaccaatgt acatcttaca tatattaatt aatgtcccat gtctctctaa attgtataaa    789840
     accaagctgt gccccacaac cttgggcaca tgtcgtcagg actccctgag gtggtgtcac    789900
     aggcatgtcc ttaatcttgg aaaatgaact tcctaaatct attgagatta gtctcagata    789960
     ctctttggtt tacaggttta ttttttatct tataacttgt attatctgaa atattaaaga    790020
     aaatttgcca aactacacat tctcttgttt aatctgttat tgtttttata aagggaaata    790080
     tatttatata atttatgtat aatttataat ttatgtacat tatgaagctg agtagcagaa    790140
     ttattaataa gatctagagt aataaaaaat tccaggatca attatcagct taatagaaaa    790200
     agaaattatg ctacatttgt ttgctgaaat gctacccatt tataaaatat aaacacacac    790260
     aacaaaaagg tttaactctc agcatttcta ttgagaaaaa taagccaaat agttaagata    790320
     tatactatat tacttcattc ttataaattc tagaaaataa aagctagttg aaagaaatat    790380
     gaaaacatca gtagttttat aaagaaatgg tagaagaaag gaaggagaaa aaacaaaaaa    790440
     aataagagag caagaggaat cctgagggaa gttgacttgt caccttcttg aaaatagaga    790500
     ttttttttca atgtttacta ttgtacaggt taaatatgtg aattttgtta tctgtcaatt    790560
     aaaacgtatg aaatttatta caagtaaaca attgacattt tagacaaaat aggaaataca    790620
     gcaaggaaca aataaatatg ttaaatgtca gatataccta aatatttatc tacctgaatc    790680
     ttgtctccac atttgtaagt aaacacagca aaatcagaca ggttctcgtg acaggaagtg    790740
     gattctgcaa accacacttg gcccgtttat ctctgtcctg tagttggttc agagcaactg    790800
     aggccagttg tgaggagcat aggcccaggt actaggactc actcatgcca gatataagcc    790860
     cttagacaca tacatagccc ctccatgtgt gggttcactt ttacgtctgt acatgaagaa    790920
     accactgatt cctaaataac ataatttata cacataggta aacataatta aaaatgtgat    790980
     agttattaaa tgtttatcat ggaacaattt cacaataagt cagcattttc ccaaatacaa    791040
     tcattgtcat cgaaatcccc aaggacactc tcatctgccc tgggccctgc cctctcctca    791100
     ggcgtcccac cccagagctt gctatatagt aggagacatg caaataggtc cctccttctc    791160
     ctgatgaaaa ccagcccagc cctgactccg cagctctggg agaggagccc caggcccggg    791220
     attcccaggt gtttccattc agtgatcagc actgaagaca gaagactcat catggagttc    791280
     tggctgagct gggttctcct tgttgccatt ttaaaaggta attcatggag aactagagat    791340
     agtgtgagtg gacgtgagtg agagaaacag tggatatgtg tggcagtttc tgaccttggt    791400
     gtctctttgt ttgcagatgt ccagtgtgag gtgcagctgg tggagtctgg gggaggcttg    791460
     gtccagcctg gggggtccct gagactctcc tgttcagcct ctggattcac cttcagtagc    791520
     tatgctatgc actgggtccg ccaggctcca gggaagggac tggaatatgt ttcagctatt    791580
     agtagtaatg ggggtagcac atactacgca gactccgtga agggcagatt caccatctcc    791640
     agagacaatt ccaagaacac gctgtatctt caaatgagca gtctgagagc tgaggacacg    791700
     gctgtgtatt actgtgtgaa agacacagtg aggggaagtc agtgtgagcc cagacacaaa    791760
     cctccctgca ggaacgctgg agaaaaacag ctgcagtggg cgctcaggac ccactgatca    791820
     gcgtcaacct cagaggcagg tgcacaagga ggctgatttc ctgtcacgat gtgggacttc    791880
     atctctttac agtttctcta gtgaacctct ctaagttcag aactctgtgc ttactaatgt    791940
     catctgtaca tattttttaa aatgattatt ttaatatgaa aacctattct cctgtgtaca    792000
     aaatgcggat tgatccttac agaggagatg aaaactcctc aaccatggtc accatgatca    792060
     gagttctgag gaaactaggg gcttcctggt gagtcttctc caatcaaatt caggacagga    792120
     acctcagaaa gattcctgac tagaactgag gtattgctcc tcacaagaga gctctagctc    792180
     cggggggtct gttcctgcag acccctgatt cggtggtgta tgaataaaat gtacactgac    792240
     acacagatac tctgtttttc cagtgcagct gagggtccga ggccacttac agactccaag    792300
     aagaatcctg taaagaatgg cagcagtggc cctgagcagc tcgcactcca ggcgtgtatt    792360
     tagtatagaa ttaacaacag aagctctgag tcaacacact tgtggataat taacatggtt    792420
     aagagagtag ttctacgaat gagtaaagct cagggactgt ggtctgaagt aaataccatt    792480
     agggggcaat attcttggtc gacctcccgc tgagagggtc ttctggctta aaggttagtt    792540
     aatggggtag ggtaaccaga cttaactggg gaagcctctg ttgtccctag tatttaccct    792600
     acaacctaat gctctaaggt aagaaccggc catcttcagc ctgttcagtt attgcaagct    792660
     atgtaaactt ccggccttcc aaaaaagttt gtgactattt cctataactt tcgttaatat    792720
     ttccctttaa tatttctgcc accatcctga atgaatccca gcaagaacag tctttgggga    792780
     ttctgattac agaaaaaaga gaggctgggc cagggtcagt gtcatgtaga acctcacagc    792840
     tttcttgtgg gacccttctc ctgacaccga agtatgcaaa tcagcatcag cactgatctg    792900
     gtgcttcttt tgttcctgat tcatttactt tcttttttag ttgttgttct catttttcca    792960
     tttgcttttc ctgctttctg agaaacaaag atgtttttgc tgtggtcaaa attccaggcc    793020
     tcaagccctt ttcctggagc tcaggtgggt ctcaggctgt ggctgctgca gtcacgtggg    793080
     agaggctggt gggactttct tcactcctcg tcactcagga ccctccactg tgttgcatgg    793140
     agactcatgt cgaaatacaa gttgccagtg agaacacagg ggacaagctt gtttggttaa    793200
     cgtgggatgt ggatgtgttt ctaatcctgt tctgaaaaac cttcacacag taacgttctt    793260
     cactagtgga ggaggaagtg ggtgtgaacg ttgtcagaat aaaaatggag ccacgtgtgt    793320
     tataatcttt acaggtgaag ctggaggagg tcatgaatag agggttctca tgcacacatc    793380
     cctgataaca agaactaccc taaaaatact ctgcacaacc acaaccttga acaaaggcta    793440
     ccacaacaat aagagaatta atactgtggg gatatctgcc ctgcaactcc ctgttcagcc    793500
     ttaaactgat tctacccttg ttattgattc ttctacccca ggataattgt ctcaaaatag    793560
     ctcatgtaat cttctcattt atcctttaga atacttgtct tcctttacca acctaaatgt    793620
     gaccatggat aatcccattg caatgctcat tttcaaataa atacgatttg attttggaga    793680
     atctctttct gtctgatatt taggtttgac aagatgcaga ccactttcct gtgagatgta    793740
     gggaatgaca atttttgggg gtggctggaa acatccaata ctctcagggc cggccatcag    793800
     taaatgcagg ctggatgtct tagaaagagc tcaaggtcct taatcaccat ggagttttac    793860
     cttctccaga tctgttctga tggaatcaag gccaagctgg ttatcaaaga taatctacct    793920
     atcatagagt caactgatta cagttttaat aaagtctatt aaaaatccac actaccacct    793980
     ggattagtgt ttggtcaaat aactacaaag tattggccag ccaagtacac cataagacag    794040
     accattaccc atgtagaaaa acatttaaca tgagttctag gttcttatat tgttaaaggt    794100
     gtaaaaccga ttatttttaa attacgctat ttttatttta tcgagttgta gaagtttcat    794160
     ttacgttttg gatgttaaca cttctttaga tacatggtat attatccaat tctgtgagtt    794220
     ggaattattt cgttgctttg cagaatcctt ttttaaatct agtcccactt gtccaattct    794280
     gttttttttt ttatgtcctt tgaatgtaaa agccagaaaa agattgctaa ttttttgagg    794340
     gttgggagtt ttacaattgc aggtatttca tttaaatatt gaattcattt agagttaatt    794400
     ttttttgttt attctaacct aaaattctta attctttgca tgtgaaaatc caattttcat    794460
     aacctgctct ttggaagtca ctataattta gcaattgtat atcgattgtt ctcatgctga    794520
     aaatcagctg gccatcagta tgtgggttta tatctcagcc ctgtatgcat ttatgccaat    794580
     accattctga tttattactc tgtgtttcta ataaatgttg aggcctggaa gtgaaatacc    794640
     tcaagctttc ttcttgcctt gtttcagata ttagaccaaa atattctaac cttttactat    794700
     tgagtataac aatagctgag acttttctta atggctttta ttatgtacaa gttgtttgct    794760
     tgtcttcata ctttgttcag agtttttata atgaaactct gaattttttt tcaaatgttt    794820
     ttgtgtctga tgaaatgtta ctgagatatt ttttctttag ttttttaatg tggtgtacca    794880
     aattgattga tttgagaatg ttgaatcatc tgtgcatctc aggaagaaat ttgagttggt    794940
     catggtgtat ggtcttctat aaaatcttta gaatttattt tactgttgtt ggacggttaa    795000
     tttatgtcta ttaatgatat tgatctatgg ttttatttaa ttgtggttcc tttgtctatt    795060
     actggtaata ctgtaacggt agcctcatag aaagagtttg gagggtttgt tgcagactgc    795120
     ctttaaaata gattttatca gtggagaaac ggtgatagtt tttttcttca gttttctgtt    795180
     gggaatagtt ttatgtattt taaaagatat tttgaatgac ttaacctgct taaagggctt    795240
     acataatatt cctttcttcc atgcttttta aagaactgct tacctttcct aattattttt    795300
     agggtgattt ttttcataaa gattgtgcaa tacattttga ggtgaaactt agtggatttt    795360
     ttctaatgaa ttagaaataa taaatcactt aattgactat tgtattaagg ttgatttgtt    795420
     gaatatttgc taaaggccag ttctttaagc tgtgccatgt actaaatccc taaccgacta    795480
     ttttattcag gttgatttgt tgaatatttg ctgcagacca gttatttacg ctatggcatg    795540
     taatacatcc caaatggcag taagtcattg tttatttagc ttttgtgctt atatttttca    795600
     gaggaaaaaa cactactgta aattgtaaat agccaataca taacagtatt gtatgcaaat    795660
     ctgtgactgt tggcagtgtc atctctgaga aacagataaa gtttatttac tatatataaa    795720
     aaaaagagtt tggaaggtgg actcctcacc aatttttgaa agagttacgg aagggttgcc    795780
     attacttctt taaatgttaa gactcatttt atgatttaac atataaccta tcacggagaa    795840
     tgttcaatgg gtgcttgaga aggatgtgta ttacatggct cttggttgga aggttctgta    795900
     aatgtctttc aggaaaattt gttcaacagt gttgttcaag ttcagaggct tgttaacaat    795960
     tctctttctg aatttgctat acattattgt taagtgaggt attaaggttt tcccatattt    796020
     ttatattgtt ttctatttct ctatatatgc atatatatat taaagtttgc ttaatgcatt    796080
     tatatttgta tttgtacaca tgtgtaaaaa ataaaatagt aattgcctag tgagtttcat    796140
     ggcacagtca cattatgaat aatcatattt tcccaaacgc tgccattcca ctaactcctc    796200
     cagatacatg gtatattatc caattctgtg agctggaatt atttcgttgc tttgcagaat    796260
     ctttttttgt taatctagtt ccacttgttc aattctgctc ctccaggagt ctcatatctc    796320
     ctctgggcac tgccttcttc tcaggcatcc cacactggag cttactatag acgaggaggc    796380
     atgaaaacag gacactccct ctcctggtga aaacaatccc agacccaacc ctgcagctct    796440
     gggagaagag ccacagccct gggattccca ggggtttcca ttttggtgat caggattgaa    796500
     cacagaggac tcactatggg gtgtgagtta agctgatttt ttcttgtttg tatttaaaaa    796560
     ggtgactcat agacaactag agtgagtgga tatgagtcag agaaacagtg gatatgttcg    796620
     gcagtttctg accaggatgt ccatgcattt tcaggtgttc agtgtgaggt gcagctgatt    796680
     gagtccatag aggacctgag acaatctggg aagttcctga gactctcctg tttagcctct    796740
     ggattcacct tcagtagcta ctgaatgagc tgggtcaaca agactccagg gaaggggctg    796800
     gagtgagtaa tagatataaa atatgatgga agtcagatat aacatgcaga ctctgtgaag    796860
     cgcagattca ccatctccaa agacaatgct aagaactcgc tgtatctgca aatgaactgt    796920
     ctgagaacct gaggacatgg ccatgcatgg ctgtacataa ggttccaagt gaggaaacat    796980
     aggtgtgagt ccagacacaa aatttcctgt gtctggaaat tttgaaagaa gaaagaagaa    797040
     aggagtctgg gccgaagggg acactcagca ctcacaaaac gggtggaggc ctagggcagg    797100
     tacagaaaag cagtcaaggg ctgctgtcct tcaggatctg tgccttcctc tgcatatagc    797160
     aggtgccctc gaatcctctg cacttttatg tttctgtgcc taccatgagg tccctggatt    797220
     acaaaacttt aatttgaaag aggaaacatt cttatatgtc ccaaaaaccg atgtaagtat    797280
     tggaggtgta aaaatgcaca ggcagttgga tgaggctgta gacactgcca acccacaatg    797340
     ccagtctcac aactagcact ggagaatagt gggagttcaa tggagcttcc tacctatctt    797400
     gtggtccaag ctaactccag cgaggccatt ggtgccatca gggacctccc atatgtccca    797460
     gcagcagcca tgcctcagta tctctattgt gcacagccat agtctgggag gagctcccag    797520
     gatgggtgtc tttggcacac acaggtgatg ggtgttagag tgtagtgcag cagctggctg    797580
     cctggtctat tgggctccct gatgttggag ggattagagg tggattctca gggccagcac    797640
     actggatttt tgtatgaaaa ccatgatttt acttcatttt ctcagatgac atagataatt    797700
     aataacacaa tctgcaaaca attgtaattt tcagctttag cccaaattca ttgtttctga    797760
     attctgtgca ggatccagac atggtactgc ccttctcatg agaaattgtt cgacctaaac    797820
     tgaaaccagt tgtttctcgt atactttggt tctccccatg tgcagagatc ttgattagag    797880
     caagtttggt actttccacg cactcaccct cacctcccca gataaagagc agaagttctc    797940
     cttagactga gtctgaggga ggagctgttc ctgtaccact cagggcctgc ggagaccccc    798000
     aggtgcagct tcactgagtc aggtgtttca ctccctgtga ttgctgctca ggtctaattg    798060
     tggctcggaa ttaggacagt cttcaggtta tcacaggtca atcatattct aaaaatcatc    798120
     gttatcacac accatggtaa caattcaagg ttcatttttc taacggcagt ttctcttttt    798180
     tatttggtta caagtttgag gaaaggaaca tctgataata ctttttaacc taacctcgaa    798240
     atctactgaa ttgttctagg agactcacaa attggacaaa gtgagctctt tattctcata    798300
     aaaatgtgtg gttttgggaa tttcactgtg ttgcccagaa cctgttaaca tcaacaacta    798360
     tgtttctcag cacacttctg gcttgagacg tccttgcaga ccctctccct cacttgcact    798420
     gtctctggat tccccatcat aaccagtgct tcctgctaaa ttatatctgc ttgcccctag    798480
     atggacagga gtggatcagg tgcatgggtt gtgaagggag gacaaattac aaccacttct    798540
     caagagtcca tatccagatc caagaaacag ttcttacagc tgagctctgt gcccagtgaa    798600
     cacacaacta cgcattttta agcaaaagac acaatgaggg gacttcatta tgagcctaga    798660
     cacaaatctc cctgcagggg tgcataggac cagcaggggg cactcggggc agcatgggga    798720
     cttaggataa ttgtcgggga tcaggatgag caggatcaag gctcagcatt gggacaggtg    798780
     caagagggca gaaaaggggc tgtagatgtg ggttgttttc gtcatcatat ttcaccaaca    798840
     gacaccctcc actacatctc ttctaatgta tctgagtgtt tatatgatta taaaatggca    798900
     tttatgtaaa tactaatata tacccatatg aaggtgcatt gagtggtcct cgccatctca    798960
     tgtggccctt gtccatcaag caataagtcc ctgtattcac ttgagtacct caaacattac    799020
     agtccaaaca tgtaaggttt ccctttttct tggtctctcc tcctcccttc tctctctgtc    799080
     acacaaacac acatgaactg acacacacac agagcttccc aactttaatt atctgatgta    799140
     ttgaagcaaa ctgattaata tgcagctttt ctgctttgcc tcccatttat gttgtggtga    799200
     aaataagaac catgtgtttc tcagctgggc acttctctaa gttaagtagc agctttgttt    799260
     attacaacca gagagcaaaa gtaatccagg tgttagtcag cagcttaaga ggtcaacaag    799320
     ttgtggaaaa ttcatttact ggaataatac ccactgttac aatcaagtac tgttggatac    799380
     actcaacacc atgcttaaaa taacaagtac ctgaataagt aaaataagtc aaacaaataa    799440
     aagtgcatac atacaattcc acttctataa tttctctgaa gtaaaaatga acttaaataa    799500
     aaaggtctgt agttgactgt ggatgtggta caagaagaga aggtgtggaa aagagaaact    799560
     acagaagagc aaaaggaaat ttctagggta actgattttt ttctgtgtta gtaaaagtga    799620
     ggattatgtc aatatttgta aaattgtcca ctttacgtaa agattattat ttgctaattt    799680
     cacctcatta aaacattgca aattttaaaa tgtataattt gttagaaaag gtgctagaga    799740
     gagattaata aaatacgtaa aagtcagaga ctcctgaata cacacatgaa tgaaccctgg    799800
     gtctcgctct acttttaggg agacactaga atacaaaaac ataatgacag gattacagtt    799860
     catgaaagga gcttctcaaa ccccaggagg catgtccaac tgcgtcctgg agttaactca    799920
     gggagcaggc atgtcctttg gaaggagcca tgacaccaag ctcccagcat ccattgtagc    799980
     tgacaccatg caaatgccaa gagatctcaa ctaaaatttt ctgtggatgt tgagtctgat    800040
     tatgccacac actcacacca agtgagtatg gtaaggatag ttacctgcat ccttaaggtg    800100
     tctgctgaga gcagggcagg tctctcatta aggtccaaag tggcttgata aagcagggaa    800160
     ggagactggc tcagggttgt tatcatgatt ttgtggtgtg ggcatcctac tggcaggaag    800220
     ggttttgtgg ggtttcaagg tcaatttggc atcaaataag ggagctcctg tgatttctaa    800280
     ctagatttat cctgtgtgct ttaaaagaaa gagagatgat ggaggaaaga gccttatgct    800340
     attagcaatc aggcataaaa atagaacctg atgacttact ctaaatagca agtataaaaa    800400
     taatgaggaa gaaaagagac aagattccaa tatgggtgga caacaaccag gtctgcagaa    800460
     aatgagaaga ctgtttataa gcaaagaata atgagtagaa ggaggatatg agaaggattc    800520
     tggtccaata tcttgtgtgg aagcttttca tgattcgaga tcatcagctt attctgaagg    800580
     tcttaggtca cctgtcttct tcaaaatatc agaagttcca gaggatatgt gaggatgcac    800640
     agtttaactt cctctatttg agtagcttta caattgtgta aaattcttaa ttcttttttt    800700
     taagatgagt tctcactctg tagcacaggt tggactgcag tggtgtgatc atagctctct    800760
     gattttaaat tcctggctca tagaatcctt ccacctcagc ctcctgagta gcaagtagct    800820
     aggtctagag ggcccttcat ctacacctgg ctgattcctt tttattcatt ttttcataga    800880
     aacagggtct ctctatgttg cccaacctgc ttttgaacac ctggtctcat gcaattctcc    800940
     aggcttggcc catcaaagtg ttgggattac aagtgtaagc cacctcatcc agtagtctgc    801000
     actgattttt ttgttataaa gtatgaatcc aataattgat tccctggatt ttttctgcag    801060
     tgctgtggtt aaaagtacct tataagattc cttccaatgt ggctgaagag cagtgttttt    801120
     ccctaatgtt tcttccaata gaaatgattt tctggttgaa gttcacaaga aactgttaaa    801180
     aagatacagt aaaaaggcag ccgtaaactg ttggtgattc gagtgtatat aatacaggaa    801240
     tcactttcta tattttgtaa cacatgcatg cagcaggaaa aaggtgagtg atgagagtca    801300
     aatctctgaa tgtatgggcc tcccagatac aattcatagg ctaataactg acgtgcccca    801360
     aggagtgaaa tgtgatgcca cacatgctag caggagaaca cgtggccaag gaagtttttt    801420
     ataatttcta tttaaaaatg tctatttcca ttttttattt tttttatatt ttcctacttt    801480
     attcaaagga aagatctaaa attgacaccc taacatcaca attaaaagaa ctaaaaaagc    801540
     aagtgcaaac acattcaaaa gctagcagaa ggcaagaaat aactaagatc agagcagaac    801600
     tgaaggagat agagacacaa aaaatccttc aaaaaatcaa tgagtccagg agctgttttt    801660
     ttgaaaagat caacaaactt gatagaccac tagcaagact aacgaagaaa agagagaaga    801720
     atcaaataga cataataaaa aatgataaag gggatatcac caccgatccc acagaaatac    801780
     aaactaccat cagagaatac tataaacacc tctatgcaaa taaactacaa aatctagaag    801840
     aaatggataa attcctggac acatacaccc tcccaagact aaaccaggaa gaatttgaat    801900
     ctctgcatag accaataata ggctttgaaa ttgaggcaat aattagtaac taaccaacca    801960
     aaaaaagtcc aggacaagac ggattcacag ccgaattcta ccagaggtac aaggaggagc    802020
     tggtaccatt ccttctgaaa ctattccaat caatagaaaa agagggaatc ctccctaact    802080
     cattttatga ggccagcatc atcgtgatac cgaagcctgg cagagacaaa acaaaaaaag    802140
     agaattttag accaatatcc ctgatgaaca tcaatgcaaa aatcctcaat aaaatactgg    802200
     caaactgaat ccagcagcac atcaaaaagc ttatccacca tgatcaagtg ggcttcatcc    802260
     ctggaatgca aggctggttc aacatacgca aaccaataaa cgtaatccag catataaaca    802320
     gaaccaacga caaaaaccac atgattatct caatagatgt agaaaaggcc tttgacaaaa    802380
     ttcaacaaca cttcatgcta aaaactctca ataaattagg tattgatggg atgtacctaa    802440
     agataataag agctatttat gacaaaccca cagccaatat aatactgaat gggcaaaaac    802500
     tggaagaatt ctctttgaaa actggcacaa gacagggatg ccctctctca ccactcctat    802560
     tcaacatagc attggaagtt ctgtccaggg caatcaggca agagaaagaa ataaagggta    802620
     ttcaattagg aaaagaggaa gtcaaattgt ccctgtttgc agatgacatg attgtatata    802680
     tagaaaaccc catagtctca gcccaaaatc tccttaagct gataagcaac ttcagctgaa    802740
     taactttgta tgactaataa tataaaatgt tccacatatt tttagccatg ttttacttat    802800
     ttgtgaaatg tgtgtttatt aatttttcat tttaagatgt cacttttatc tttcttgttt    802860
     ctgggatttt attctagtag atatagctga aggtatttta ataattattg taataatctc    802920
     acattttata tttgaatttt catatccatt tcattaagat tttgattttg atataaaata    802980
     ttttcttaca attgtctttt ctatttttat gagataaaat taatgtatac aaaattgcat    803040
     acatcttcaa tgtacagttt caggtgtctg acatatgtgc acacatttgt ctccagcacc    803100
     taagttagga tgaggagcag gtcaatctcc acaacaagtg tcctctttgg tgcttccagt    803160
     cagctctcac ataaggattt ttatttaatt tcaagttttc attcagatac agagggcata    803220
     tgtgtggact tgtcacatgg gattattgag tgatgctgag gtttggaatc cagattccat    803280
     caccccctcc ctccactctc cagcagtcca cagtgtctat cattcgcaca tttatgtcca    803340
     tgaatgctca atgctgaggt cccacttaga ttatgtggta ttcggttttc tactcctgca    803400
     ttgatttatt taggtttcag gcccccagct ccaatcattt tgctggaaag gacatgacta    803460
     tattattttt catggctgtg tagtgttata tattgtagat gtaacacatt ttgtatattc    803520
     agtctaccat tggtgtgtat ctgggttgtc tttgccacta tggatagcac agcaatgaac    803580
     atagatgtgc atgtgtcttt ttggtagaat tgtttgctta ctttttagtg tacacccagt    803640
     agtgggattg ctgggtaaaa tgatatctct gttttaagtt ctttgagaaa tctccagtct    803700
     ggttctcaaa gtggtaagac taattcatat tccaatcaac agtgtataag tgttctgttt    803760
     tctccacagc cccaccagca tccatcgttt ttcgactttt tagtgatagc cattctgagt    803820
     ggtgtgtggc ttctcaccta cagtcatctc atctttgata agactgacaa aaacaagcaa    803880
     tgaggaaggg actccctgtt caataaatgg tgctggggca actggctatc cgtatgatga    803940
     agattgaagc tggacgtcta ctttcaacac acataaaatt aactcaaaat tgaccacaat    804000
     tttaaatgta agaccacaaa ccataaaaat cttttaagac aacctaggaa atactcttct    804060
     tgacaccagc tttgacaaag aatttttggc taagtatcca aaagcaattt caagaaaaac    804120
     aaaaataggc aagtgggacc taattaatta aggagttact gcacagcaaa acaaacacac    804180
     aagcaaccaa aacttcagca gataagcaga caacctgcaa aatgtgagaa gatattcaca    804240
     aacattgcat ccaacatagc actaatatcc agaatctata gggaacttaa acaaatcaga    804300
     ggcaaaacac aaataacccc tttaaaaatg ggctgatatg gtttggctgt gtccccacca    804360
     aaatctcaat ttgcatttta tatcccagaa ttcctacatg ttgtaggaag gatctagggg    804420
     gaagtaattg aatcatgggg gccagtcttt ctcatgcaat tctcgtgata gtgaataact    804480
     ctcatgagat ctgatgggtt tatcagatgt tcccgctttt gcttcttcct catttcctct    804540
     tgccaccacc atgtaaagag tgcctttcac ctcccaccat gattctgagg tcctcccagc    804600
     catgcagaac tgtaattcca atgaaacctg tttttgttcc cagatttcag tatgtcctta    804660
     ctagcagttt gaaaacagac tgatacctgg gaaaataaca caaacagaca cttctcaaaa    804720
     gaatatatac aagtggccag caaatgtatt ttaaaaatat ttagcatcac taatcatcag    804780
     aggaatgcaa atagaaaaat gttctggttt ctgtcactat aggtccattt tttcctgttt    804840
     tgagcatcac ataaatggaa tcaatattat agttcatttt ttgtaagggg ctacttttgg    804900
     tatttgtgag gttcattcat gtatttgcat ctatctaggt tttgtcatca tatatataca    804960
     tatgtatgta ctcatgcaca tctagctatt tcatatctca atcagtccat tacgttaata    805020
     aacgacagtt tattaagtaa ggaaatcagt tcattaagtg aataagtgtc agtacatata    805080
     tctattttcc tgttgatgga atttaaattt gtttccaaaa taaatattgt aaacaaacct    805140
     gttataaata tcgtcgtaca agttcttttg cttatattct cccattttta ttgataaaat    805200
     atgtagaaat ataagtatgc attataactt ttcagcttta aggagctata actgacaaac    805260
     aaaattgtat gtatttaagg tacaccactt aaggtattga tatacttggg cacatgttca    805320
     tcgtgatcaa ggtaattggc atgcctatca tctcagagaa tatcatttta tgcctttaat    805380
     ttattgtgag tctgtgataa aaacacctaa gatctactct tctggaaaca gataagttta    805440
     taatataata ttcattagta tagttgcatt gctgtatgtt tcatctccag aactccttca    805500
     acctgtgtat cagtccattc tcaaactact ttagagaact acctgagact gggaaataca    805560
     tgaagagaag tgaattagat gactcacagt tctgcaggct tagcaggaag ccttataggg    805620
     aggcatcagg aaaattacga taattgtgga aagtgaaggg gaagcaagga ccgtcttcac    805680
     atgctggcag gggaaagaga gaaagagcaa ggggagatgc gccgcacttt taaacctcga    805740
     gatcttgtga gaactctaac acaagaagag caaaggggaa agccgcctcc atgattcagt    805800
     cactcttcat cagacccctc ctacaacact tgaggatgaa aatttgacat gagatttgga    805860
     tggaaacgct gagccaaaca gtatcattat acccggccac tcacaaatct catatccctc    805920
     tcacattgca aaatataatt atcccttctc aacagtctcc cagttttcac tcaattcagc    805980
     attaacacaa acttctacag tccaaagtct cctctgagac aaggtaagtt tcttggatgt    806040
     ataaccctgt aaaataaaaa acaagttagt tacttccaag atactatgag atgaatgcac    806100
     tgggtaaatg ttcccattct aaatgggaga aattggccac tacaaatggg ctacaggccc    806160
     catgcaagtc caaacactag cagggcagtc attaaatctt aaagctccaa aaaaatttcc    806220
     ttttactcca tgtctcacat ccaggacacg ctgatgtaag aggtgatctc ccaaggcctt    806280
     gggaagctcc acccttgtgg ctctgcatgt tagactcccc atagctgctt tcataggctg    806340
     gcattgagtt gctatgtctt ttccaggcac accatacaag ctgctggtgg ttctaccatt    806400
     ctggggtctg aaggacagtg gtcctcttct cacagatcca ctagacagtg cccaagtggg    806460
     gactctgtgt gggggatcca acaccatatt ttccttctac actggcctag tagaggtact    806520
     ccattagagc tcagcccctg catgagactt ctgcctggat atccaggcat tatcatacat    806580
     catctgaaat ctaggaggag gttcccaaac ctcaactctt gcctcatgtg caccagcagg    806640
     ctcaacacca cgtggaagca accaaagctt agggcttgca ccctctggag caatggcctg    806700
     agctggacct tgaccccttt tagccatggc tggcacagga cagacaggga tgcaaggcgc    806760
     catgtcccaa ggctgcacag agcagtggca tcctgggctt ggacaatgaa accatttttc    806820
     tcccctgggc ttctggacct gtgatgggag gggctgccat aaagatctct gaaatgccct    806880
     ggggacattt tttcccactg gggtccttgt tacttatgca aatttctgca gccagcttga    806940
     atttctcccc agaaaatgag tttttcttgt ctaccacttg gacaggctgc aatattttat    807000
     caaactttta tgctctgctt cccttttaaa cataagttcc aatttcagac catctcttta    807060
     tgaatgcaca tggcttctgt tttcagaaac agccaggtca actcttcaat gctttggtgc    807120
     ttagaaattt cttctgccag atatacctta aattatgtct ctgtagctca acatttcacc    807180
     gatctctagg gcagggggaa aatgccacta gtctttttgc taaatcatag caagtgtgac    807240
     cgtcactcca gttcacaata agttcttcac cttcatctga caccacctca gactggactt    807300
     cattgcccat atcactatca gcattttgat aaaacccatt caacgagcct ctaggcagtt    807360
     tcaaactttt ccacatcttc ctgtcttctt gtgagcactc caagcttttc caacctctgc    807420
     ccattaccca gttacaaagt cacttccaca ttttcaagta tcttaatagc agtatcccac    807480
     tcctggtgca agttttctgt attagtccat tttcacattg ctataaagac tacctcagac    807540
     tgggtaattt atgaagagaa gagttttagt taactctcag ttctgcaggc taaatgggaa    807600
     gcattattgg gaggcatcag gacgaacaat gatggtggaa ggtgaacggg aagcaagaac    807660
     cttcttcaca tggtggcagg agagagagag caagtgagcg gggaggtgcc acacttttaa    807720
     accatcggat ctcttgagaa ctttatcacg agaacagcga agaggaagac aatcccatga    807780
     cctgatcact ttatcttagg cccttccttc aacatgcagg gtctacaatc tggcatgaga    807840
     tttgagtcgg aacacggagc caaaccacat taaccttcat aacgggaagt ttttaccctt    807900
     tgaccaacaa tacccaattt tttcctcctc ccagcccctg cgacctacta ttctactctg    807960
     cttccaagag cttgaatata aaatattcaa tatataattc tatatataaa tgagatcatg    808020
     cagcttttgt ctttctgtgt ctggcttatt ccacttagca taatgttctc tatttgttgc    808080
     aaatataaga atttctttgt ttttaaaggc tgaataatac tcagttttat gtaggtataa    808140
     gccacatttt atctgttcat tcgtagatgg acattgactc gttttcctta tctagactat    808200
     tatgaataat ctcacaatgg acatagattt gtcacactca gtttattttc tctagatgta    808260
     tgctcagaag tggggatgct ctatgtccag ttcactgagt aatcttcatg ttgtttttca    808320
     tactggctgt aataattcac attttgttcc aaaccataca tggatacctt tgtaccacat    808380
     attcaggtct ttgtgtaact cttgaatcca tttttagctg atttctgtgt attgtgtgag    808440
     gtgagttcta tctatttctt ctgcatatgg atacccgttt ttcacaccac tcgttgaaga    808500
     gactgtcctt tctctactgt gtgtcttggg aacttgacaa agatcagttt attgggaaga    808560
     aatgggttgg ttgccaggtt gtgtttaatg tttcattgga ttatatgtct gtttaaatgc    808620
     cagcattata acattttgat ttatatagat tcgattttga aattatagat tatgatatat    808680
     tcagctttgt tatttatgcc caaaattaat ttggctattt gaagtctttt atatttttat    808740
     ataaattgga gctttttaaa aacattttgt aaaatcatgc catggagatt atttatttat    808800
     tttattggca tatagtagat atacctattt tatgggaaca tgtaatatat tgatacattt    808860
     ataaatgtgt aaagattagt gtaagattta catatctgtc acattaaaaa tgtacccttt    808920
     ccttgtgctg agcacatttg aatgactcct ttcactattt taatgttgtg cattagatta    808980
     ttattaacta tagtgacctt actgatctat caaacattta ggtcttattt ctcctctata    809040
     aatgtatatt tgtatacagt aatcaacctc tccttatctc cttctctctt ctatccatcc    809100
     aggattctgg taagcaacag tctattctat cttcaggcga tccagtattt tagttttgac    809160
     gtaaaataag aagatgcaat atttgtctct cttttcttgg cttatttatc ttaacataat    809220
     gacttccagt tccattcata ttatagcaaa tgacagaata ttgggtttca tggctgaata    809280
     atatcctatc gcatacatag attacattta ctctatccat ttgtccacct acagacactg    809340
     aggtgctttc acatctgggc tattgtgaaa gagctgcaaa gaacatggga gtgcacatgc    809400
     cttcatgagg tggtggtggt tttatcttct ttagaacata cccagaagag gatttgccaa    809460
     gtcataaggt atttctgttt ttaatttatt ttggaatctt catactactt tgcacatagt    809520
     ggaaacacaa atggagtaaa cattaaaaaa gcagtttcgg ttttgatgta taatccagaa    809580
     acaaataatg tttgaccatg attctcattg ggagtctcta atggatctgg tggaaatgca    809640
     ggaagttttc taaccttgtt caggaaatta tgagtttgca cctttttctt tctgaggctt    809700
     gaaattggca tgattcatga tgcatactgt tgggaggttt cagaatgata tggcaaaaaa    809760
     cataatgaaa aggcaagaca gagagagagg gagagagaga gagtcaggcc tagaaagaga    809820
     aaaaataatt ttctcaacag gagaaagtgc tggatatctg tatcagtgtt gggttttgct    809880
     catagacaca tctgtaccga gtgaggaacc atgaagtcaa gggaggggtc tagaacttgt    809940
     tcaagtgaag catcagcata tttctcacag gcaccaattt ccaccacatc acaagggtga    810000
     tgcatttttg aatgccatta aatatgagtt cacagtcagt aatcatgttt ccatgaaatt    810060
     cagttaaaat accaagggtg tgtccagatc actcatgcac aaatacacaa aatttgcttt    810120
     ttcacgtgtg gctatctaga gacaaagtgc actgaggaga cttcagcagg ttacagagat    810180
     ttgcttaagt ttggaaaacc ataggagagt gcctttaagt gaaggttggt atataaatta    810240
     aataggtcaa aatcccctct atggagtagc gttccaattg tgtggaattt ttaactcctt    810300
     attcacgtta taaaacataa tccctgggac tgactccgtg gagttttact gctgtgttga    810360
     caaaaatatc taacaagctt ccttccagtg tttcaaggga agtttttctc taaatttttt    810420
     ccaatgtata aattttcctg gctaaagata gtaaaaggct gttaaaaaca caactggaat    810480
     ggcagcttta atcttttgct gataggagtg agcataatac atgaatctct ttgagtgctc    810540
     tgcttcatcc aaatacaata ggtcaaaggc tagtactgga ggtaaaattt ccaagtgtgt    810600
     atttttccag ctaccaagtc ataggaaaat aacagataca tccttgagac agaggaccat    810660
     aatgccacaa tgctggtagg agtacatgta gacaagggag ttcaagggtt tcttaaaact    810720
     gataatttta atgtaagaat accattcttt taaacttatg tggaagactg tgggaggtta    810780
     taacaatgcc atattttagt atcagccctg cctcacagaa tcattttata atagttcctt    810840
     tcatgagtgt gcttcactcg gttcatgcaa aaacctggca ggtcacaggc taaaaacaga    810900
     aaatatagaa gcttttaatc aactgaaaga gctgtagcct ttgtcaagga attattggaa    810960
     gacacagaga acacagatag atgataaata aaatacattc atatcccatt aagtgtggaa    811020
     attgaggaaa gtttacataa atgtgtccaa gtggcaaact gtttatttaa tctggtaacc    811080
     aatttttcta taaattgtag agttgaaatg caacaaatac atatttgaaa tgatctggca    811140
     caaattaagc aaatatggta actatgtatt ttcactcatt tttatttaag atcagcggct    811200
     cagcattgat atttttgata atatctgtga ctcagcagct ttaccttttg aggatatgat    811260
     ctggtatggc agttttctta gcttcaatgt tacctctttt tgcattgact tctaccttta    811320
     tatctgccag gaatctgggg aaaaggagtg cctgtaaacg ttccctaact tgcccatttt    811380
     ggtgggtttt ccagaatgtg tgagatgctt tttttttttt ttcaaagcat cccgaaccca    811440
     tgcactgccc tgaaacatgt ttatttccta gtttaacaaa ttggcacttc taataaatgc    811500
     aatctcttct gccatctgtg ctgattttac atcagataga ggactgtgtt ctaaaggaaa    811560
     tcttatatta gtaagagcaa ttttgtcatt aacctagagt tttattattg aggtgtgatt    811620
     cataacatat aatattaggt agaggttctc agtggcagtg tctaaatctc ttgggtgtac    811680
     agtgtcttcc ccgttaacat gaagcattta cgagcacagt catagtttcc agccatgctt    811740
     ctccctgtct cacaatcacc acaaactaac tatgaccaca actggaactt ggtgagttat    811800
     tctttaataa gttttaagtt tgtctttaat ctttaagcct ctagattatt atgtgagaca    811860
     taatctttat gcctctagat tattgtgtaa agctatttca gaagaaagtt aaaaagaaca    811920
     ttttaactga ctaaacaaca caggaaatca ttaataaaat gcaaagtgta gatgtgagag    811980
     gctccaggcc tggataatgc aagagttcat gtatgcaggc agtttctttc cccaattata    812040
     caatgataca cccagcatgt cagcttcatg ccccatttga ctcctattat gcaagcgcat    812100
     ggaaatgaca tgctcaaggg tcacacacag atatgaaaac aggtgggagc aggagaggag    812160
     acgactctgc acttctcctc tgaaggacca ggaaagcctg gacagaccat ctccccggcc    812220
     tccatgactg cgcgacgtgc ccacatggac actcatctct gacaagataa gaggactcca    812280
     ttgatgaggc tgaacatttt atgatttaaa ttactagaga cttacattga ggtttcaata    812340
     actaattttt ataacccaaa tttacttacc cccatgttgt tacctctttc ttcagtgaaa    812400
     aattgctttt gctgtaattg agttttagga agattcccaa tgttcacaac tgagcttcca    812460
     agagagtgtc gagaacaaaa actgaatgag ggcagagaaa cccatcttta ctgtgttcac    812520
     cactaaactc aagtggactc ggcactgcct ttgatcactg ttgcttctcc gcagagttca    812580
     ggtttctatt tctcacaatt ctaacacaga ctctccctct cctcaaaggt ttggcctcca    812640
     ctggctgtaa ggtcacactg ttctgaattt cttgtctgaa ccattttatg agggtggtga    812700
     tgagccattt aatggaaatt ttatatcatc tcagcatgaa tcctatgggc ggtcacgaat    812760
     tattattctg attatggtaa ttgatttttc tcagcatatt cattactagt gatattcaaa    812820
     aacccttttt atttaaatct ttgatgcttc attttttgtt gctatgacac tttttcttct    812880
     actgagtctt cccactagca ttataacatg acctaatatc caggctcagt tgttatataa    812940
     caaccacata tgtcaaaagc tatgcattct tttcacagca gacataattt tctcttttct    813000
     ggagatgaac acacactgct gagctacccc cactcacaag aacatatgga aaatcatgac    813060
     atttttattt acttgactaa taaattatgt cattctctct tcaaattctt tatcccccag    813120
     aatgccatga caaactcttc tgcatctgtt caaaccataa actcagaaac cacatggtga    813180
     gtaaaagctc acttggttct ggatattggg tcaagctctt cccctccaat gtcccacagc    813240
     attgggacct cagcccacct gtccaggttc tatcaagaga gtttagctcc ttcacagtga    813300
     aggaaacaat tgagttaaga gggaacctgc agaagataga caatattgaa aaccattcat    813360
     atgacaaggg attactatca agcatataaa ataaactcaa cccaactgca aataataatg    813420
     tggctaataa tggaggaata acattaatac atatttctca aaagaagaaa agaaaaaggg    813480
     cagatagatg atagatagat aaatagatat attggtatgt atacatacat atatcgctag    813540
     aaaacactaa tattcaggaa aatgcacatt cacaatgaga tgtcttttca tccttcatct    813600
     ttgcttgaga gtcagaaaat ctaaataaat aaataaataa ataaaacagg agctggccag    813660
     gattcataga aagggaaact cttatagacc attggtggaa atggaaatta gtgaagccat    813720
     catgggaaaa aatagaacta ccatataatt cacaatccaa ctgctgagta tgtatctatt    813780
     taaatcatta aagtaaaaaa ctaatattga agagatacat gtacacccat gtttattgca    813840
     gcactatcca ccatagctaa catatgaaat caacatatgt gtccatcaac agatgaatgg    813900
     ataaataaaa tgtgttatat ttacacaatg gaatattgtt cagtcttaaa aatgaaattc    813960
     tgctgttaga agcaacatgg atggaactgg acaccatcat gttgagtgaa acaagacaga    814020
     cacagaaaaa taaatacagc attttctcag tgttatgtgg aaattttaaa aagttgatct    814080
     tctagaagta aataatagag tagtggttat gagacgccgg gaatgggagg agactgagag    814140
     acaataagag gtttgttaac aaacgtataa tttacaggca gataggaggg atgtgctcca    814200
     gtgatctaca gtgcagtagg gtgactgctg ttaacaatat agtgcacatt ttatgtttac    814260
     aagtagccag aagacagaaa tttgtatgtt accaacaaaa agaaatgtta gtgtctaagc    814320
     tgatgaattt gttcattgtt ctgatttaat cataccacat ggcacacatg catagaaata    814380
     tcacactgta ccccatacag ttattatgtg ctaactttaa aaaatccttt aattaaaaca    814440
     gattttattt gcatacatta caaatgcttc aacacagagc caggaataaa taccattttt    814500
     ctttgaaatg tgaatttcct aacaacgtag ttacattcat ttgaaccaaa ccgtgtattt    814560
     gatcatggta agaatagaca ggcttatgca tagaatgata tattttaatt ttagacttct    814620
     acttaatacc ataaattcaa ataattttaa aacaactaag taaaaactat aaagttgagg    814680
     aaatgtgtac gtggtgtgtg atgtgtgact ttttccttgc gcaccactct gtccatggtg    814740
     gatgtgtggt gtgggtgtct gtgtctattt ctgtttactc tgcttgaggt tctctgtgat    814800
     tctaggatct gtagttcagt gtctttcaca aaactgggaa cgttcttagc cattatttct    814860
     ttcaaatact gtctctgtat ctacaatttc tcctttcaga tttaaaatgt acatatacaa    814920
     tactttttaa tattaatgtt tactttcttc actctctttt ccttgcactt tactctgtaa    814980
     aatttctagt ggtattttaa gtgagtggtt tcatttaaaa ggtgagccag ctctactgaa    815040
     gggtgtgccc aaagcttact caatgtttat actgcattgc ttttgatttc ttatgcattt    815100
     ccatttgatt ttttcttagt attttcaact ctcagttccc tatctagtcc ttcattatgt    815160
     atacagtttc cttaatagat ttttacatat gaattatagt tactttatat atcttgttta    815220
     attagatagt tctaagatcc atatcatata gaagtctcat tctgatcatt tgtttattat    815280
     gactttggta tttctcatta atatatgtaa tctttgttga tagccagata ttttaggttg    815340
     gacagttgat attggcttat tacttcattt tatgcatttt ctgcctgtat ttgaccatac    815400
     tttatttttg ccaggccttt aatgtggaaa tgtttgaatc ttctcagagc tacatttgac    815460
     gtttactttt gcagtagacg tcatagttga agtctgttct tctgtgtcca ccagagactt    815520
     cagatcctcc agtgatacct tgtttttctt tcctgcttgg ctttgtctct tcaccctgtt    815580
     ccttcctcca gagaatctct ttcagctcct tcaggtgggt taagatatta tattgaactg    815640
     acaattgtga aattggtgga aggcaataga ataaagggag attttctgac ctttcatggg    815700
     tctatatttc tacgaaggca ttgtgaccct gagtctgggt gtgacattgc cggtgtttct    815760
     gaacttctgc cagatgagat gttggtctgt gtgttcttgc tgttttccct gctgtggagt    815820
     cctcttgttt tccccagttg ttccctccca cagctccaat gttctctttt ggtgttatca    815880
     gcttccagag ttgatgacct gacctagaga ttaaggctct gattaaataa gaaggagggg    815940
     agatacttct caatggaact taggtgaaga cctctttttc catctcagtt cctaagggat    816000
     ggccccagtg cccctaagat cctggctttg gtggcttgct cctgcagagt aatttcttag    816060
     ttctgcaatg gggattaagg aggtgggtct gaatgcattt cagagtgtgg gctctttttc    816120
     tctcccagac agacacattg ggacaaaaga tttttgtgac tgtccccatt tttgggaaaa    816180
     aggtttcaag gtataggaaa gctcttcagt atgtggtccc tgagaaattc acgctacaac    816240
     acatttacca cacttgactt caccaattca gtatctatgt atttttttct cttataatag    816300
     cctacatttt tatatgccag actctgcctc agttcaactc gtacccatgc ctttttactc    816360
     tctgcaagaa cttgtctctc cctaaatttc ggatttgatg attatcttaa aacctccagt    816420
     atttaaagta tattttaaaa ttggcaaaat tccatctcct gtgtttatct gttatgttga    816480
     tgctgtaaaa cagtaagtaa aatatactcc tcatctatgt acattttgaa gctgagttgc    816540
     aggttttttg gtaagaccca gagtcacaga gaattcaaat attgttaagc tgcttaatag    816600
     aaaaacaaat tatggtaaat gtgttcactg gaatactacc catgatttat aataaataaa    816660
     tgcctgacac acagaacagc agcaaaacca cacatgctct tattacagaa agtggcttct    816720
     gaaaaccaca ccgggcatgt acagctttgt cctggagttg gtttaggggg atgtcagagc    816780
     cagtgacgag aagcacaggg ccagatggca gcgttcactc atcccagaca tgagctcctg    816840
     ggtgcataca gagccccccc atgtgtgggt ttacttccac ttctgtaaaa ggagaaaata    816900
     ctgactccta cagagcataa tttacacatt ttttaaaaaa tgtaataggg tgatcagggc    816960
     aaagtgttta tcacagcaca atttcataag acagcatatt ttccaaatac catcattgtc    817020
     agcaaacttc tgcagagcac cgtcttctta tatgggtaca gcctattcct ccagcatccc    817080
     actagagctt cttatatagt aggagacatg caaatagggc cctccctcta ctgatgaaaa    817140
     ccaacccaac cctgaccctg caggtctcag agaggagcct tagccctgga ctccaaggcc    817200
     tttccacttg gtgatcagca ctgagcacag aggactcacc atggagttgg ggctgagctg    817260
     ggttttcctt gttgctattt tagaaggtga ttcatggaaa actaggaaga ttgagtgtgt    817320
     gtggatatga gtgtgagaaa cagtggattt gtgtggcagt ttctgacctt ggtgtctctt    817380
     tgtttgcagg tgtccagtgt gaggtgcagc tggtggagtc tgggggaggc ttggtccagc    817440
     ctggggggtc cctgagactc tcctgtgcag cctctggatt cacctttagt agctattgga    817500
     tgagctgggt ccgccaggct ccagggaagg ggctggagtg ggtggccaac ataaagcaag    817560
     atggaagtga gaaatactat gtggactctg tgaagggccg attcaccatc tccagagaca    817620
     acgccaagaa ctcactgtat ctgcaaatga acagcctgag agccgaggac acggccgtgt    817680
     attactgtgc gagagacaca gtgaggggaa gtcagtgtga gcccagacac aaacctccct    817740
     gcaggggtcc cttgggacca ccagggggcg acagggcatt gagcacgggg ctgtctccag    817800
     ggcaggtgca ggtgctgctg agggctggct tcctgtcgcg gtctggggct gcctcgttgt    817860
     caaatttccc caggaacttc tccagattta caattctgta ctgacatttc atgtctctaa    817920
     atgcaatact ttttttgtcc tttttgtttc tttgtttttt tgcaacagga gtacatatcc    817980
     tcagctccac agaagccagg gtgtcacttt gggggcagaa ataatccttt cgtggtcaac    818040
     aggatgagag tcctgaggaa tcccagggaa acctggagag tgttttccaa ttagactcag    818100
     ggcagagacc tccatgggaa tttctgatta gaacgggctt tgagctctga tgggagccaa    818160
     gagacaggct cacccagggt cggggtcctt aaagcctgat ggttttcaca gctatccccc    818220
     ctcgtcttgt aaactcagac tgattcagtt gacccttttt ctgctaatcc attttgcttc    818280
     tctgtaggtt tgattctcac agttcgcttt cttcttctct tccctgagaa caaacgatgt    818340
     gttttctgtt ttcagggaaa atcccagggc tcaggtctgc aggacctggg taggctgagg    818400
     agactttctc actcaccatt gtctggacac tcctgttgtc ttctgtgcat ggaggcattt    818460
     gggaaatgaa gtggacatta gtcattaagg gaataatact agttttctcc aattggatat    818520
     tgatgtagag ctgaacttgt gcttctcaca ctgtcacaga gtttggaccc tcacctataa    818580
     ctttgaggag agttgatgat ggatagtcca ttgtgggtga gctctgggta acagtaattg    818640
     tagggtctgg ctaggcagcc taaggtcaat acttctggac ctcaggaaag acaggctgga    818700
     attcctggga agacctgcat ctgccatcca ccgtggagtc ccatcatctt ctgttatgct    818760
     gtgattgaat cagccccacc tagtttatct agaacactct tcatgactta ggaaaacata    818820
     atggcaagct ctcctaacac ctgtatcatg ccatgggagc aacacctaag ctagtgtgtg    818880
     attgagtaga tgagactgtg gtctagtcaa gacgacaggt aaaattgatt gttgtcatta    818940
     tgatatttta tatttggcaa tataatcatg ctcatattat aaatactttt gctacatttt    819000
     ttgagtgtgt cagaggcttt ggaactggaa caactccatc ttgaataggg gctaggaaaa    819060
     ataagaccga gacctgctgg gctggattcc tagtaagttg atgcagtaag ttagtcacag    819120
     gatgagatag gaggtctgca caagatccag gtcataaagg ccttgcagat aaatcttaca    819180
     gtaaagaagc tgggcaaagc cctccaaaac cgagacagtg gctaaaatga cctcagtttg    819240
     tcctcactgt tcattatact ctaattataa tgcattaaca tgctaaaaaa cactccctcc    819300
     agccccaaga cagtttacag ttgtcaaggc aacatcatga agtttccttg tgtggtttaa    819360
     aaaggagggg aggaacactc aacttcagga attgcctgga ggactcatga ataatccatc    819420
     cattgtttaa catataatca agaaataacc ataaaatgga caaccagcag cccatactgc    819480
     tgctccacct ctggtgtagc cagtctcatt attttacttg cataagaaac ttgttttcat    819540
     tttaatctgt ggattttccc caaattggga cccctttctc ctaatatttg tgctattatt    819600
     cgtgttctga gcagaaaacc ccaagatgga atgaagtttc cctacatttt tataagaagt    819660
     acattcaccc ctttgagcga aaatgcacag ggagtggaat gaggctggga agctaatggc    819720
     gtatgatgga agcttgtccc tgactggagg agagagggag ggaggattgg gaggaaggtt    819780
     cctatatttt tgtgctatgc aaggaagctg caaaagataa ctgagtctcg tgtaagtcag    819840
     tgctacctct caggggatcc ctgtgactcc aagaaagggc tcttcttagg aatcactgtg    819900
     gagtcagtcc tttcattaga gtagaccaca ggacatgggc ctcagcacac gccatgccac    819960
     agatgtcaga aagcagctgc tgggaacctg acccacctgc attttgctgc ctgtatgtgg    820020
     agggagggag gtgcattctc agggtcagca cactgtttgt gaatttttat agagaacccc    820080
     tgcttttgct gtatttttat tggaaaatat atgaacaagt gtgcacctgc aaacaactgt    820140
     aattttcaca tttccttcag ttgcattatt tcttaatcct gaatgacgtc ctgacacaga    820200
     gttgagtttt tcacaggcat tgttcgaagg atcgagatga tgcattttcc acattcactg    820260
     gtttttctcc atgtgcagag accttgagta gagcacatct ggcccttacc cacactatct    820320
     cttgtgtccc ctgggaaaga gcagagattt gcctgactgc agaatctggg gtaggagtct    820380
     gcacacctct gagcctgcag agaagctcag cgaagttttg tggagtcaga gagctttccc    820440
     atgtggggaa cctctagctt cctcactctc ggagattact ggtcagctgt aattgagtgg    820500
     ttaagaatta gagcacctgg gagcctgtct catcacagct ccattatgta acttaccacc    820560
     attagactgc gggtaataac tcaatcttga tttttctgac ccgattttct catcttattc    820620
     attactaatt ttgtttatgg aaaatgttat gtttagttta tgattaaaac ctcagaactt    820680
     tctgaactta tcaggaataa tacaaaatgt gtccagtttg ggggaaatgt aaatgaatgt    820740
     ccatgcagtt tgggtgtgtg tgtgtgcgtg tgtgtgtgca tgtgtgtttg tttgtgtgtg    820800
     tgtggatatg tcagtcactt acaccacaat gaacatgatt gctgaattac atagtcaaac    820860
     taaaattaga tttgctgaaa attgcctgca gcgatgtctg tgaagctgac cctgtcactt    820920
     tgctgtgaag aaactcagtg tacaggcgag gttgcacggg agagctaaaa gcttcagcac    820980
     acaggacagg ctgagctcct gaactggagc caggcacagg actgagcacc tgagaaacag    821040
     aacccccagc cctcctttat ctgggctgga tgggcaccag aggacccgca ggagatgctc    821100
     agaactcaca caagacagag cctcagtgag gagcagaggc agcgaggaac cagccaggga    821160
     gcctgtgacc gccacctcgg gttgtcttat tttgcttaac agcatccaaa cctcaggaga    821220
     ccatttatta cgttatttag acctcacgcc ctttctgtcc cgaagtaaaa caaacataaa    821280
     tatgactctg cagttagctc tttgaacctg ggttattttt ctgagaatgt catcttcgtc    821340
     atcaacatca tggtcatcat gtcaaaatca acaggcaaat ccagtcaccg tgaagacagt    821400
     ttcatggcat gattcataca caatcattcc attctacaga gaatctcttt cagattctta    821460
     aggtggatta aagtgttata tttaattgcc aattgtgaaa ttggtggaag acattatact    821520
     aaaggaattg tctctgacct ctcatggggt catatttcca gaaaatcatt gtgatcctga    821580
     gtgtgagctg actttccaag tgttcccaac cctcctccag atgagatgct ggtctgtgtg    821640
     ttcttgctcc ttcccctggg gcagattcct cttgttttcc ccagttgttc cctcccacag    821700
     ctctcataat ctctgttggt gtccccaact tccagatctg ctgccatgac ctgcagatta    821760
     aagctctgat tccgtaagaa agtgagggga gctgcttctc aatagagctt tgatgggaac    821820
     ctctgttcat acctcaattc ttaaggggct gcaccagtgc cctaggatgc tgattttcat    821880
     ggcttgctcc tgcagggtaa tatcttagtt ttgtagtggg gatcagagaa ctgggtctgg    821940
     atgcatttca gaagtgtgag ctcattctct cccagacaga tactatggga aaggaagatg    822000
     ttttgcaggg gtaaatgatt caagagatta gggcaactct ccagtatgag gtcccttaga    822060
     atttctcact ataacatgcc taacacactc gacttcaagc aatccagtgt atatttgttc    822120
     ttcatcttgt aatagcctac attttatatg acagcctctg cctcagggaa tttcatactc    822180
     tggcttcatt actctctaca ggaacttgcc tctccctaga tttcaaatat tttgtttgtt    822240
     ttcaaccctc aattatctga agcattaagt aaaatttgca aacttccatc ttttctgttt    822300
     atctgttatt gttgatacta ttgttgtaag aatgaatata tgggcccggt gcggtggctc    822360
     atgcctgtaa tcccagcact ttgtgaggcc gaggtgggtg gatcacgagg tcaggagatc    822420
     gagaccatct tggctaacac ggtgaaaccc catctctact aaaaatacaa aaaattagcc    822480
     aggtgtggtg gtgcgcacca gtagtcccag ctacttggga ggctgaggca ggcgaatggc    822540
     gtgaacccgg gaggtggagc ttgcagtgag ctgagatcgc gccactgcac tccagcctgg    822600
     gtgacagagt gagactctgt ctctaaaaat aaataaataa ataaataaat aaatatgtgt    822660
     gtatatatat atatttctca tctatgtaca tttttaagct tagcagcata ttttttagtg    822720
     agatccagaa taataaaaga atccaaacat ttttcagctg cctaatagag aaacaaattc    822780
     tggtaaattt tttcactgga atactactca acatttataa taaatatatt tctggtacac    822840
     agaacaacaa ggaaaagtat ctaagtattt atgctgagta aaataagcca gacaaataag    822900
     aatatgtact atattactcc atttatgtaa tttatttcaa atgaaaaaaa tctaaagtaa    822960
     tatacaaaag atgagtagtt acctgggaaa agggtagaat aaaggaaggg gaaatgtgga    823020
     aacatacaga agaacaagag aaaatgttga ggggaactca cttgtccacc tcagtaatga    823080
     tgacgcttac atcatgtttt caattctaca ctttaaatac gtgaagtgta ttatctgcca    823140
     attaacactc acaaaattta ttgcaagcaa acaaatgaaa aattagacca aaagcggtgg    823200
     tataaagata caaaatatcc attaaaagtc agaaatatct gagaatttac ctcctgaacc    823260
     ctagttctct ccttattttt aggtgaatgc tagagtgcca caaaaatcac tcaagttctc    823320
     aatacaggaa gtgggttcca caaagcacac taggcatgtc caattcttac ctggagttgg    823380
     ttcagggagt aactggggcc actgatgtgg agcacaggcc aagctaccag ggctcattca    823440
     tctcagacat gagacacaca cagcccctcc tccatgtgtg ggtttatttc acatctgtaa    823500
     atggagaaac cattgacttc tacagaacat aatttgcaca aatatgtaaa aatataatag    823560
     tgcaaaatgt ttatcacaac acattttcat aataagacag tgtatttccc aaataccata    823620
     attgtcaccc aactcctgcg gggccctgtc attttatctg gggtctgccg tctcctcagg    823680
     gttcccaccc cagagctctc tgtgtagcag gagacacgca aatagggccc tccccatgct    823740
     gatgaaaacc agctaaaccc tgaccctgca gctctggcag aggagcctca gccccgggat    823800
     tcccaggtgt ttctgcttgg tcaacactga acatacagga ttcaccatgg agttggggct    823860
     gaggtgggtt ttccttgctg ctattttaaa aggtgattta tggagaacta gagctattga    823920
     gtgtgaatgg acataagtga gcgaaacagt ggatatgtgt ggcagtttct taccaggatg    823980
     tctctgtgtt tgcaggtgtc cagtgtgaga tgcagctggt agagtctgga gcaaacttga    824040
     caaagcctgg gtgtcccaga gactctcctg tgcagcctct ggattcacct tcagtagcca    824100
     tagcacgcac tgggtccccc aggctccagg gaagggtctg cagtgggtcc cagttattag    824160
     tggtagtggt agtaccatgt actacgcaga ctctgtgaag ggccgattca ccatttccag    824220
     agacaatacg aaaaactcac tgtatctgca aatgaacaga ctgagggcag aggatgcagc    824280
     tgcatatgac tctgtgagag atacggtaag gagaagtcag tgtgagccca gacacaaacc    824340
     tcccttcagg gtacctggga caaccaggga aagcctggga cactgtgcac tgtgctgacc    824400
     ccaggggcaa gtgcaggtgc tacaagggct gggtttcctg tcttgatctg gggctgcgtt    824460
     gttaccaaat tacccgaggg aacttctcta gatttgcaat tctgtactaa catttgatgt    824520
     ctgtgactgc taaacttccc tcacatattt gttttttgtt tgtttgtttt gttttgtttt    824580
     ttgtaacagg aagacccatc ctcaccccta cagaagcccg agtgtcacct tgggggcaga    824640
     aatgaccctg ccttggtcac attgatcagt gtcctgagga agcccagtgg aacctgggga    824700
     gtgttttcca gtcaggctca gggcagagac ctccgtggaa atctctgatt agaataggtt    824760
     tgggattcag acttgggcca agagggagtc tcacccatgg gagggtcctt aaatcctgac    824820
     agtttttaca gctgttctcc ctcctctggc aaaactgtgc acatctgact cagaactgat    824880
     ccagctggcc cttacttgct aatcaatttt tcttctctct acacttcatt ctcatagttc    824940
     ccttttcttc ccttcccaga aaacagagag tgtgttttct gtggtctaaa tctcagggct    825000
     caggcctgtt acctggaact caggtgtggc ccaggctgtg gctcctgtga gacctgggga    825060
     ggctgagggg actttcccac tcaccattgc ctggacccgc ctgctgtctt ctgtgcatgg    825120
     aggcatttgg gaaatgaagt ggacattagt cactgagggg ataatggtag ctttctcaaa    825180
     tgagatactg atgtagagct gatcttctgt ttctcacact gtcacagaat ttcctccttc    825240
     atctgtgact ttggggaaag ctaaggatgg acactctatt gtgctgtgag ctctgggtga    825300
     cagcaattgg agggtctggc taggcaaatc ctacagggtt tcaggtcaat ggagttggaa    825360
     tttctgggaa gacctacact gtcatcctcc atggagtccc atcgtcatct cttatgctct    825420
     gatggaatca gccccaccta ggttatctag gacaatcttc ctgacttagg aaaaaaatga    825480
     tggcaggctt tactaacact tatattatag gatcagtaca acacctaggt tagtgtgtaa    825540
     ctgaataaat gactctatga gtcaagggga cacataaaat tgattgttgc catgacattt    825600
     atttatttat ttatttattt atttattgga aaccagagtt ttattgttac tgaaatgagt    825660
     ctcgccaagc attcaagaat gggatttttt aaggatagct tggtgggttg ggggcagcca    825720
     gtgagtcatg agtactgatt gatcaggtca gagataaaat catagggagc tgaaggtgtc    825780
     ttgtgctgag tcagttccta ggagggggcc acaagatcgg atgagccagt ttattgatct    825840
     gggtgatgcc agctgatcca tcaagtgcag agtctgtaaa atatctaaag cattgatctt    825900
     aggctttata atagtcattt tatccccagg agcaaactag gaagggttag aatttgtagc    825960
     caccagctgc atgactctta aaacataatt tctaatattt tggctaattc gttagtcccc    826020
     caaaggcagt ctagtcccca ggacgaagga gatttgtttt gggaaagggc agttattgtc    826080
     tctgttttaa actataaact aaactaagtt cctcccaatg ttaggtaagc ctacacccag    826140
     gaatgcacaa ggacaacctg gaggtttgag gcaagatgga gcaggttagg tcacagctct    826200
     ttcactgttt cagttgcagc tttgcagtgg tggcttcaat tcctcccttt gagttttgac    826260
     cttatcgact tgttttgttt ctctttaagt gatggtttta taaccatcta tgaaactcta    826320
     acaggtgctc ttacatgcag atttctgatt aaaaactctg aagatggtta caatagtata    826380
     gagataaaac tatcaaatag aagactatct tttttgactt ttcacttaaa atgttgccca    826440
     agaaaacttt tcttttgagc tatttatagc tttcaacaat tgagtatagt cctgtacata    826500
     aaatttggag aatatttgtt tctctctgtc cactttctcc agaatttgga aactctttgt    826560
     gaatatcctt aacttatggc aatatgattt ttcataagtg cagtaagatt tttttttttt    826620
     tgcaacagga cacaattgga gaaactggtt attttaccaa ggctttgact gtaatggcgt    826680
     gcttcacctt aagcaatcga acttgactta tacagccaat aagagcccct tgggaaaact    826740
     ggcctgtacc ttgtccacac agtctttgta caaggttcct gaccagtggt aagtaaagaa    826800
     tgtcactttc taacaggccc aagaacccca tgttttcctg ggaccccaga ggaaaaataa    826860
     tttaccagac ttacaggtat ttgatggtaa aaactcatgg ctgggcttgg ctttaaaaaa    826920
     gtcttatctg agattcctta tggaacagag ttccaccaaa gccaatttta aaagcctgtg    826980
     taaacaataa ttatttgtat tgtgctttat gcaaataata aggccatgaa taagactaaa    827040
     gtttattttg caaataaatc agtcctatct tgatttgttt taaataaaaa tggggacaag    827100
     agagaaaaaa attatgcttt aaaaatatag tacacctgtt gtttgctgtt cttgtttttt    827160
     tttcttcagc agtttggacg aaattctaaa ttatttgtag gctacaaagc cataaactaa    827220
     tgcgttcaaa tctttacttt taaaactgcg aattgcactc cttaccctag ttctcattat    827280
     gtactttata gtatgttgtt ccctcaaatg tggtgctaga attatagatg acagcactga    827340
     cagctttgcc atgcaagcct tggaacccca ggcaggcctg cgtgagtaca ctcagacagt    827400
     tgcaatgagg ttccagtcct cttatcttga ggtcagcacc tacccctact acgccccagg    827460
     tcatcaataa aaagttgtaa cagtcttggc ccttcttaca tcttcattat ccaacaccat    827520
     gatttttgtt ttttgccaaa acaattgcca aatatagtca taatcatagg ataaataatt    827580
     ttgctgaaat atatgtgtta ttattggtct tttgagatgg aaagtccaag actggattat    827640
     gctttcatgc atttttcata agaagtactt ccactctttt gacaggaaat gcacaggggg    827700
     tgacatgtgg ctgcagaagc tgatggcata tggtagaaat ctgtccctga gtaaaggaga    827760
     gatggacgga ggattgggtg aaacattctt aggtttctgt gctgtgcaag gaaggtgtaa    827820
     aacatatttg agtctactac aagtcaacat tacccaaggg ggaacccctg tgactccagc    827880
     aatgactctg cctcagaatc actaaagagt ccttcttttc attagagtgg accacaggat    827940
     ctgggccata gcatagacgt cagaaagcag cagcttgaca cctgatccat gtgcattttc    828000
     ctgcctttaa agggagggag agaggtgtat tctcagggtc aacacactct ttgttgattt    828060
     ttacacagaa cactggcatt tactttattt cattgaaaga tatatgaaca gatgtgtgtc    828120
     tgcaaatgat tgtaattttc acatttattc caattgcata atttttaaat tctgcttgat    828180
     gtcctggcac acagttgtgt tttctatagg tatttttcaa taaatcaaga aaatacattt    828240
     tccactttca ctggcttttc tccatgtgca gaggccttga gtagatcatg tctgactatt    828300
     agtctgtttt catgctgctg ataaagacat accagagact tggaagttta caatagaaag    828360
     agttttattg gacttacact tccacatgac tggaaaggcc tcacaatcat ggtggaaggt    828420
     gaaaggcaca tctgacatgg tggcagacaa gagaagagag cttgtgcagg gatctccctt    828480
     tttacaatca tcagatctcg tgaaactcat gcagtattac atgaacagtg ctggagaaac    828540
     ccgcagccat aattcaatga cctaccactg ggtccctacc atgacactgg gaattatggg    828600
     agttaaattc aagatgtgat ttgagtgggg acacagccaa accatattat tttgccccat    828660
     ggcccctccc aaatctcatg tcctcacatt gcaaaaccag tcatgccttt cctacagtcc    828720
     cccagagtca acttgtttca gcattaactc aaaaatccac agtctaacat ctcatctgag    828780
     acaaggcaag tcccttccac ctataaacct gtaaagtcaa aagcaagtta cttcctagaa    828840
     acaatggggg tactggcatt ggataaacac acccatccaa atgacagaaa ttgcccaaaa    828900
     caaaggggcc ctctgcaagt ccaaaatcca gcagagcagt caaatattaa agctccaaaa    828960
     tgatccccta tgactccatt tcccaaatcc aggtcatgct gatgcaacag atggtttccc    829020
     ttggtcttgg gcagctccac ccctgtggct ttgcagggta cagcctccct cctggctgct    829080
     ttcacaggct ggtgttgagt gaatgtgcct tttccaggca catggtgcaa gctgtcagtg    829140
     gatctaccac actgggatct ggaggatggc ggccctcttc tcacagctgc actatgcaat    829200
     accccaatag ggactctgtg ttggagctct gaccccatat ttccctttct cactggccta    829260
     gcagtggttc tccatgaggg tcccactgct gcagcaaact tctgcctgga cacccaggtg    829320
     ttttcataca tcttctgata cccaggcaga ggttctcaag gaccagttct tgacttctgt    829380
     gtactcactg gctctacacc atgtggaagc tgccaaggct tggggtttgc accatctgaa    829440
     gccatggcct gagccctaca ttggcgtctt tcagccatag ctggagtggc tggacacagg    829500
     gcaccaactc cttaggctgc acacagcacg ggcaccctgg gcctggacca caaaaccact    829560
     tttgcctctt aggcctccag gcctgtgatg ggaggggctg ccgtgaagac ttctgacatt    829620
     cacaggagac attttcccca ttgtcttgag gattaacatt tggctcctca ttacttatgc    829680
     aaatttctgc agctggcttg aatttcacct cagaaaataa gattttcttt tctactgcct    829740
     tgccaggttg taaattttct gaacttttat gctgtttccc ctttaaaact gaatgccttt    829800
     aatagcactc aagtggcttt ttgaattctt tgctgcttag aaatttcttc tgccagacac    829860
     cctaaatcat ctctctcaag ttcaaaattc cacaatttgc tccagttccc aatgagttcc    829920
     tcatctccat ctgagaccac ctcaagctag accttattgt tcatataact atcagcattt    829980
     ttgtcaaagc aattcaacaa gtctctagga agttacaaac tatcccatat tttccggtct    830040
     ttttctgagc cctgcaaact cttccaatct ctgcccgtta cccagttaaa aagtcacttc    830100
     catatttttg gttatctttt cagcaatgtc tcactctact ggtaccaatt tactgtatta    830160
     gtctgttatc acgctggtcc caagactgga caattcataa aagaaaggtt tattggactt    830220
     acagttccac atggcttggg aggcctcaca attatggtgg aaggtggaaa tcacgtctca    830280
     catggtggca gactagagaa gagtgcacat gaaggaaaac tccccttttt aatccatcag    830340
     atctcatgaa actcattcac tatcacacga atagctcagg agagacccat gcccataatt    830400
     cagtcacctc ccatcaggtt ccttccatga catgtgggaa ttgtggaagt tacaattcaa    830460
     gatgaaattt gggtggggac acaaacaaac catatcactg accttctccg catcatctct    830520
     catgacccca gagaaagagc agagatttgt ctggggtagg agctgctcct gtggaactta    830580
     gaggctgcag acgaccccag tgcagtttta tggagtaaga gagttttccc atgtctggga    830640
     acttctagca tacccactgc cagagattgc tggttggctg ttattgaggg gttcagaatt    830700
     agatcacctg gtagcctatc tcactgcagc cccattgtgt aacttaccac caatggcctt    830760
     cacgtgagga ctcaatgtta actgttctga cctcattttc ttacctcctt tatttgtaag    830820
     tttggttaca gatttttttt ttactttaat ttcagggata tgtgtgctga atgcgcaggt    830880
     tttagtttct tattcaagcc ttataattgc tcaactgatc aggaataata caacatgtat    830940
     gcagttttgg ggaaatataa atgaacatcc atgcaaaatt ttttgtttgg atatgtccgt    831000
     cagttacacc cctatgaaca taatagccgg tcctcaccag aaattgctgg tcctcaccga    831060
     tctcaaaagc atctagatct tcaacttcct agccttgaga attcagaaaa caatgtttat    831120
     gatatttctg agccacccat ttaatggagt tctgttatag cagctgaaac agactaaacc    831180
     atttaccaag tctcagtaag ggcagtgagg gtccaccatg cttgagagac aagcattact    831240
     ctctcatttg cagctcagga tgacagaagc atgatcacga gctgatctga aaagaagatg    831300
     tggctttctc tccctcagat ctcagttgaa gccatggtgt cacatctcta tggtggagac    831360
     taaattccag tctgtgatgt aatggatgcc tgcctcctcc caccctcact tacgagacag    831420
     agtttccatg ccatgcgtta ttgaccaaga accaagaagg actccaatta ttctcacccc    831480
     tgctcacttg tatggcagag gttctgggga tattcctggg agaggcataa tgagactatt    831540
     taagtccacg attcccaaac agttttatgc tcttaaagca ggagtatcac tttgagatat    831600
     atgaataatt tcatgtgcac taaggtggag gctgttatat ttttataggt aaagatgcat    831660
     ggcataaaga cagagataca gcactctgga aattgtgtga ctatatttgc agttgactgt    831720
     gaataatttt taccatgaag ttatcctaaa acacataata tatttttact gtaagtaatt    831780
     aatgaaaagc tgacagctcc acaagagcaa tatccacttg gtttagcaga gagaatgaga    831840
     aatagcataa tttaagaata atcagttaga cactgtgcca tgtgcctgta ttttcaacta    831900
     gtgaggatgc taatgtggga agatcaccca agcccaggat ttcaaggcca gcctgcaaga    831960
     tattgctaga cccattttct taaaaaaaaa aaaataacat tctgggtcat gacaaacctc    832020
     aaaaactgac cctctaaata ggaacaaatt taattgcatt aaaatgtgga tgaatttttg    832080
     acacaaaaca gtgacagcat gccagcaatg agtacagaat aacatccagt tgttatgtta    832140
     agagatacag actccgaaaa agagacatca ggtagaggct gtcaaaggcc tcactgccta    832200
     atgactgctt ttccattgat actgtgtgta tatcccggaa tgcacctgtg aatgaggaca    832260
     tcatgtcttt catagtgtga gagaaagaga cattactacc catctgacaa aggattgatc    832320
     agaatatacc aggatctcag acaactctat aggaaaaata tctagtaatc cgaataaaaa    832380
     gcaggcgata tttatttctt aaaataagac tttcaaattg caaacaggca tataaaatgt    832440
     gctgtgtaac actgatcatc agaacaatgc aaaacaaaac tacagtgaaa tatcatctca    832500
     tctgatttta aaatgatttc atccaaaagc cagacagaaa caaatgctgg gaaggatatg    832560
     gagaaatgag aacactcata acctgttggt gggcacttaa gttaataaaa ccactatgga    832620
     gaacagttta gagtttcctc aaaacctaat agtagtgttg tcatataatc cagcaatcct    832680
     gctgctggca tgtactccct cccacaaaaa aaaaatgttg tttgcactac catgtttgtt    832740
     atagcactgt tcgtaatagc caaaatttga aagcatccta actgcccatt aacagatgat    832800
     ggataaataa aatgtggtac atatagcata tggattaata ttatgccata aagagaatga    832860
     gattttttca tttgcaacaa cgtggatgaa actggagttc attatattaa ttgaaataat    832920
     cccagaaccg aaagacaaac ttcacatatt ctcatatatt tgtggaagtt aaaaattaag    832980
     acaactgaac tcgtggagat agagagtgaa aggatggtca ccagaggctg ggaaggtcta    833040
     tggtagatgg ggattattgg ggatgctcaa tggatgaaaa aagtggttag aaataacaaa    833100
     taatacatag tatttgataa cacaacagac taagtataat ccttaataac ataattatag    833160
     attgtaaaat aactaaaagt gtataaatgg aatgttggta actcaaagaa ttaatacttg    833220
     aaaggatgta ggaaccattc atcataatgt gagtattatg cagttcatgc tatatcaaag    833280
     tcaggtaccc cataaatata cacatatacc atttacccac aaaaattcaa aattaaagta    833340
     tttatgtatc tcatcttctg tatctatatt acattaaacc taagaagata ctggtttctt    833400
     cacactacca agatccacat agacatccgt agccttcttt ctttgcctgt ccataatctg    833460
     acattgaaaa atgaggaaac ctttcctatc atatgacatt aatttactta gtgcacaatt    833520
     tcaaaataca caaattgtgt tagaattatt aaactgaccc ttgttgaaaa tatgtttatc    833580
     gactagaata ccgtgcttat atgcagctta tttatatttt acaatcacaa catcaaatta    833640
     tttccaaaga tacttgtttc tgatattcat gccccccttt ctgcagtgaa attagtacaa    833700
     acctgtttat cattgttaga tatttttata cagctttcat tttattctgt gtttcatgac    833760
     cagctaaata cttttgaagt tgtatgcatt taggttaaca ctttgtctca aaaaattatg    833820
     agttttcaca aattatttat gccatgtgtt tatcattgaa caccatgaaa atagtttcat    833880
     tgtgattaaa aagtgtgtgt ttcacctaat ccacacacct tttccccaga ttcctggaaa    833940
     accacaaagg tttactggat ctatattttt gtctttggca aaatagcctg gctattctct    834000
     tagcttcttc tgcaaagcat caagattcac cttcactgac tgcagcattt attgggccct    834060
     gatggctgga ggagaggggc tggagtgggt ggtagccatg agtgacccaa gtggaagttc    834120
     tcagtggtgc tctgctccag cacaaagaag attcacagtt cctggggaca acacttaaca    834180
     tcacaatctc ccttaaaatt atctactgga aagctgagga gtaggcagtg tattactgtg    834240
     agagacacag tgagggacat atgggtgagt ctagacgcaa acttccctgc aggaagacaa    834300
     gaggggactt tgtggtaaat ggtgctcaga accactaggg gacactcaga accactagga    834360
     gacactcagg atagcagaag gtgctcagga tgccgcaggg tgctcaaaac actaggggtg    834420
     ctcaagacca gcagggggca ctcaggacac caggagacac ttaggacatc agggggcgct    834480
     caggacacca gggggcgctc agcacaagca ggggccacag aatgacaagg gtgcattcca    834540
     gaccaccagg aaacactcag gaccaacaag agattctcag aaaccaccag gtgtaacagg    834600
     accacctaaa ggcccacaag acaaccagga gacactcatg gccactgaag aacattgagg    834660
     acaccagggg ttcagggcca acagggagtg ctcggggcca ccatggggct ttcagtaacc    834720
     acgagggaat caatcactca ggacaccacg gttttcttag gaggcagctc cacatcaggt    834780
     gcctggggaa ggtgacgttt gcttttagac ctagctgatt cctgagctgg tcaagcaaaa    834840
     gtcttcccac gatctctcac tatttcttcc ttgtaaccca tggtttcttt cacatacaaa    834900
     tcattaactt aggataggaa ttcaattcaa cttttaacct tgcatatttt tagaataata    834960
     atagcaatag taattcaatg tgttttgata ataagaaatt gtgtatgcct aattcaaact    835020
     ctggttccac gcatgggttt ttgtttcttg tgctgtgcca gtcacactac agtcaatgct    835080
     tttctaataa taactcaaca atgtcacggt gttaagtttc ctttcatttt gtctgccatt    835140
     tgtggagatg aaacaccact ttcacgggtc agttcctcca ctttggttgg gttctggtct    835200
     tctgctcttc acactgtttc tccaccttcc cttctgtcaa atccttttgt cttcctcagt    835260
     ctcctggcaa agaagcaaca aatctattta ttttgtctcc tccacgtctg gtgaatctgt    835320
     tcacttctct tcatgatcat tgaacccaac catgtttaga aggataacag ttcaccttac    835380
     aatatgctca tgtaggtcag gtgcagtggc tcactcctgt aatcccagca ctttgggagg    835440
     ccaagacggg tggatcatct gaggtcgagt ttgagatcag cgtggccaac atggcaaaac    835500
     cctgtctcta ctaaaaatag aaaaattagc caggcatggt gtcgggcacc tgtaatccca    835560
     gctactcggg aggctgaggc aggagaatca cttgaaccca gggtgagagg ctgcagtgag    835620
     ctgagataat gccacttaac tccagcctgg gcgacagaga gaaactccat ctcaaaaaaa    835680
     aaagcatatg ttcatctatc cgcagactct ctgacatcgt cacccttttc tagggtcctg    835740
     cagacatcac cccacaacat ccagtccttt tcctaagtac cacagagtgg gctctgcagc    835800
     tcctgctgct ctctgtgtgc tcagcgctga ggctcaccag tgctttgatg atgaagttca    835860
     aatccctaat gtgtttgcaa ttctcagaca accctctagc aagctgccat tgtgagtgca    835920
     ttctggggat cctgggctga tttcaggtcc atattgctgg atgttctcta gcatccaggc    835980
     gtgttggcaa ataaacatct agaatttgta ttgaaaattt gtggaaaatc aatggtagaa    836040
     aacaatccta atttctaaat aagaacattt tatcctgact ttataaaaac acaatgttaa    836100
     tttaaccaat aatgtaataa aatgtgttta aagtgatgtt tatcttgtta ttagatgtat    836160
     taataattgg tattttaaag tatactaaca gcataaatga caaacaaacc aacacagcga    836220
     ttatacataa ataccattgt acattaatta atttggcaag aatggcattt attttcattt    836280
     ctaccaaacc ttgcctttaa atatgtttgt taattggcat taggatagtg aaacaatcac    836340
     acagagcaac aacattttgg aatattacat tatactataa aatagtacat tcgattcaat    836400
     tatataatta aaataacatt aaataaattc atgttttggt ttgatatttg gatatgtgtt    836460
     catttttgta ttttcttttt ttgtatcttt tgtagagatg gggtttcacc atgttggaca    836520
     ggctggtctc gacttcctga cctcaggtga tttatcgggg gaacccaccc ccaatatttc    836580
     aatgtagggt ctttctattt tctgtaagtg tcggctggtc tgagaaataa agagaaagag    836640
     tacaaagaaa ggaatgttac agctgggcca ccgggggtga catcacatat tgataggtcc    836700
     gtgatgcccc ctgagccgca acaccagcaa gtttttatta gggattttaa aaggagaggg    836760
     ggtgtacgaa cagggagtag gtcacaaaga tcacatgctt taaggggcaa aaagcagggc    836820
     aaagatcaca tattccttcc ccagggtatt aattactaat attccttgct gggaaagaat    836880
     ttagtgatat cttccctact tgcacatcag tttataagct ctctgcaaga agaaaaatat    836940
     ggctctattc tgcccgaccc cgcaggcagt cagaccttat ggttatcttc ccttgtttcc    837000
     taaaatcgct gttgttctgt tctttttcaa ggtgcactga tttcatattg ttcaaacaca    837060
     catgttttac aatcagattt catattgttc aaacacacat gttttacaat caatttgtac    837120
     agttaatgca atcatcacag ggtcctgagg tgacttacat cctcagctta caaagataac    837180
     aggattatga gattaaagaa attatgaaag tattgatttt gggaactgat aaatgtccat    837240
     attaaaatga aatcttcaca atttatgttt agagattcca gtaaagacag gtgtaagaaa    837300
     ttataaaagt attaattttg ggaactgata aatgtccata ttaaaatgca atgcctaggg    837360
     ccaacaaagg agtcatatta atatgaaaaa taatagctga atattttgga aatgacacat    837420
     gcagtaacaa atgtctatat gaaattaaga agatattgac acaaaattga gaaaaaatta    837480
     acataattat gctcactact ggtttataaa atgcctataa taacactttt taaatcatat    837540
     tatagaaaat aaaataacta tataaatagc actctctttg cttgatttta gtatttggtg    837600
     cttcaaatgt tttttataca aataaacatt ggtcattttt tgatggtaat ttcaatattt    837660
     tcttccagaa tacatgttca taaatgtaat tgaaattgta taattttcag aaacttattt    837720
     tatcagttgt gacttgtatt tttatgatag tctttttaca tttatatact tttgattcag    837780
     aataattata gataaaaaaa ttataaagtt aatacagtgt gttcccatct ccaagtatct    837840
     cctaagatga atatcctcta tcatcataag acatggtgtt aacacattta cattgataag    837900
     tgtaaatata ttcaatacca gaccttattt tatttatcat ttttccaata atttctattg    837960
     taaatgaaaa ataaaattct aagcttcctc aagcaactaa atgaactcaa ccctcagcca    838020
     agggaatttc aaagtaaatt ctaaaacttg ttccagcaat gatgggaaag tgagaggtca    838080
     gacatgcctc attacatgct cctctctttg gaattaaggc accagtggcc agcattagta    838140
     ctacaacaaa gatcttaaga cgggcaaaac tatatttttg ctgtaaacac tttctatagc    838200
     aaaaagacat caaattacaa cttgattcta gtatagcatc acatgacaaa tagcaaaacc    838260
     taatgattta ttctgtatga aatctgctac ctggaggctt catctacata ataagaacta    838320
     tgatctccag gaccccttat cttaatccag acactcagtt gtaatgattg tattacctta    838380
     catttcaacc aattgccaat cagaaaatct ttgaattctc ctatgtcctg gaaccctgtg    838440
     cataaacttt cctgcctttc tagactgcat aaatggtata gctcacatgt gttgattaat    838500
     atctgcctgt aacttctgac ccactaaagt atataaaatc aagctgtaat caagccattt    838560
     tggacatgtg ttctcaggaa ttcctggggt cgtgtcatgg gccttggtaa ctcatatata    838620
     tctcagaatg agccatttta aatatttcat agtttgtccc ttttagtgga tatcttcttt    838680
     tctttttttt gggacagaat ctctctttgc tgttgctttc gtatatttgt ttgtttccag    838740
     attccatcaa ggccatcagt ttgaattcag ttgtcttgtt cctttattct cttctggtaa    838800
     atgacagttt gtatttctga agttttctag ttgaatattt tatagataaa aatttatcaa    838860
     gtaggttgat agtgccattt gggactctta tatctttacc tatttaatca ttgcttgttt    838920
     tctcaatcac aatgagtgtt gatttctcca tgttctatac atgttagtct attttctgtt    838980
     ccaaatccat ttattaggtc ttttaaaatg gattttggaa aagttgatcc tattatttta    839040
     tgcaatattt cacttgagtt gcaaaggtat tctttttatg attattttgt ctgaaattaa    839100
     catagctatt caagttttct ttggtttata ttttcatggt ctatattttt ccatcttatt    839160
     tcttctcttt gtgaatatgt taagaagttt tcttggagaa agataattgc tgggtctttt    839220
     ttttttttag tcaactatga ggaattattt tttaatgata ttaccctttt gtccaataat    839280
     ggttgttttg tctttttaca ataatatgta ttgtttctat taatttatta tttaattatt    839340
     attgatgaat atcttattgg ttaccataag gataacaata agacatttct ataattgaat    839400
     tctaattcaa aaatgtagta cttctttttc ttttttattt ttttgagacg gagtctcact    839460
     ctgtcgccca ggctggagtg cagtggcaca atctcagatc actgtaagct ctgcctcctg    839520
     ggttctcgcc attctcctgc ctcagcctcc caagtagctg ggactacagg cacctgccac    839580
     catgcccgga taattttttg tatctttagt agagacaggc tttcaccgtg ttagccagga    839640
     tggtctctat cttctgacct tgtgatctgc ctgacctggc ctcccaaagt actgggatta    839700
     caggcatgag ccaccgtgcc cagcctaccc ctttatatat agaacagaga tattataact    839760
     atatatgtct aattcctctt ttctatccct tttttatttt atcagtttgt aattacataa    839820
     gctataaaaa tgtaatattg acatttttat aattgcttta cacatttatg taataaagaa    839880
     aacacaaatg caaaaactaa atttaaacct catttttttc attgttgaca atctttatct    839940
     tttggataca ttcaggtatt ggatgtatat catatggctg ctcatctaga aactctgcta    840000
     aaacgtctat ggcaagatta atatgctggg aatagatttt ctcaggattt gtttgtccga    840060
     cagagtattt atttgtcttt gatttatcat ctcaagagac aatgtcaaga agatgctgtt    840120
     tctgcaaatg ggcaatctgc aaaccaagga cacgtcacta cattactgtg caagagaagc    840180
     acatgaggaa aggccggtgt gagacacaaa cctccaggaa cacctgggct aatgagctgc    840240
     agggggtgct caggacccac tgatcagtca accacagagg ggagtgcaaa ggttaggact    840300
     gctttcctgc aagcattgaa aaaagaagaa aaaaactccg tctgacagtt tctccaagga    840360
     acttctgtaa ttttagaatt ctgtgcctac caatgacatc tccaaatatt tttttaaaat    840420
     attgtgtttt gaggacatac tctcacatgg acaaaacaca aattgacaca gagatgaaaa    840480
     gccctcaacc atagtcacca aaattggaga catgtagaag ctcactggga cctgttgagt    840540
     cttctccaat cacactcaga acagagacct tagtgggtct cccttagagt agtcattaag    840600
     gatggtgata acagcctaaa gatggtagac catggtcagt gtcatataga atatggggac    840660
     cctcacaagt tttttgtctg acccttctcc tgacactaaa ttatgcaaat taataacact    840720
     gatctggtgc ttcttttgat tctaatttat tttattttta gttgtcgttc tcacttttcc    840780
     tttggatttt cctgctccct ggaaaaggta aatgtggtct ccgtgacctc aattcaaggg    840840
     ctgaagccct ttccctgtag ctcagctggg gctcaggctg tggctactgc agccatgtgg    840900
     aagaggctga agggactttc ttcactctcc ttgctcagga ccatccactg tattgtgtat    840960
     aggcttctct ggaaatgcaa gtggccattt gtagtgaaag aaatatgttt gtctggttaa    841020
     aatgggaggt ggatgtagag ttaattggct gctacataaa ctgtccttct ccaccagtgc    841080
     ttttaggatg agattgtgaa atttgtaaga atcaaaatgg agtcacatat gttaaaaccc    841140
     tgacaaatgg attcaggaag tgtagggaga attcttacac acatatccct gacaacaaga    841200
     actatcataa aatagttctt gcaaaaagac caacatgacc tcataatcat gacttctgca    841260
     aagacttcta ctcagaatct acttgcccag ccttagatta atgccatctg aattacactg    841320
     atcatgttac tatcactgct cctcaccaca gatgcaacac cctcctgagt cctgaaacct    841380
     gactccatcc catagagtag ggcacagatg aggggaatgc aaatctccac cagctccacc    841440
     ctcctctggg ttgaaaaagc cgagcacagg tcccagctca gtgactcctg tgccccacca    841500
     tggacacact ttgctccacg ctcctgctgc tgaccatccc ttcatgtgag tgctgtggtc    841560
     agggactcct tcacgggtga aacatcagtt ttcttgtttg tgggcttcat cttcttatgc    841620
     tttctccaca ggggtcttgt cccagatcac cttgaaggag tctggtccta cgctggtgaa    841680
     acccacacag accctcacgc tgacctgcac cttctctggg ttctcactca gcactagtgg    841740
     agtgggtgtg ggctggatcc gtcagccccc aggaaaggcc ctggagtggc ttgcactcat    841800
     ttattgggat gatgataagc gctacagccc atctctgaag agcaggctca ccatcaccaa    841860
     ggacacctcc aaaaaccagg tggtccttac aatgaccaac atggaccctg tggacacagc    841920
     cacatattac tgtgcacaca gaccacaaag acacagccca gggcacctcc tgtacaaaaa    841980
     cccaggctgc ttctcattgg tgctccctcc ccacctctgc agaacaggaa agtgcagctg    842040
     agatacgttt tcctgccagg gcctgcattt cccatcccca ttagactcag agccctgtct    842100
     tcctccttct tctttaataa taaatggcat gactcctgtt aatagttcat agaagcagaa    842160
     gctgagtcct gtttgtcaaa cattcagcat gaaatgttca tgttacctgg gccagatgca    842220
     tcactggtat gtggccgcca gtttaatggg ctcatactac tccaagcggc cgaatacaaa    842280
     acagcagaac atatgccttt attaaactgc aggaaatgac actggagaaa aatgtgggga    842340
     taagggagca ggaggaattg tgaaaattca gataatagct tttctcgtag agagaaaggt    842400
     gcgttaataa aataatttta taaaacaata aaacagatgg aatgtgtccc catcacggag    842460
     tgtctcacag taacagtgca gaaataaatt tctaaaaatg tcgtagggta ggtgtgatag    842520
     acaaggctta tttccacaca gggagcccat aaatacccat gtaattaaaa atccagctct    842580
     tagaaaggga gtgcctcaac ttgacatcag aaacccacgt cagtgagtct gaggagcaat    842640
     gtggaaaaga aatggaaaat tcaacaatac ctttcttatg aaaatcctag ctcacgtttc    842700
     aacatgaaac ataactgtct acaattttag gaaaaccatc ccatggcaac aagtcactac    842760
     actcaaaagg tactgagcta acagagttcc agcagtgagc aggtcagtgc tgaaatcccc    842820
     ggagaatcaa ggctctcagg tacgttgtac aaaacccaga actgagccac acaatctatg    842880
     gtcaagtgat ctttgacaaa gtcaacaaaa atatgcacgg ggaagaggcc acgctcttca    842940
     tgccatgttg ctgggacaat tggactgcca tatgcagaag aatgaaactg gacaccaacc    843000
     tgtcgacata tacataacca taagaaaatg gactgaaggc ttgattatag gacttcaaaa    843060
     tataaatgta ctcgaagaaa acaccaggag aactcctctg gacattgggc ttagtaaata    843120
     atttaccaca aaaacctcaa aagcacaagc aataacatca aaaatagatg aatggaattt    843180
     agttaaacta aagagcttcc acacagcaac agagtgaaca gacaaattgc agaatgctaa    843240
     aaaatatttg cacacattgc atctggcagg ggactgatag gcagaattta cacggaactg    843300
     aaacaacccc accacaacaa cagctatgaa aaaacaaata acctcattaa aaattggcaa    843360
     aggacacgag taggaatttt tttaaagaac acatataact ggacaagtgc taaacatcac    843420
     tgattatcag ggatatgcaa attaaaactg caaagaggta tcttacacca gtcagaatgg    843480
     ctactataaa agaagcaaaa atagcagaca tcggtggcga tcaaaggcaa agggaagtct    843540
     cagtcattgt tggtgaggat gtagatgagc acagcctcta tggaaaacaa tgtggagatt    843600
     tttcaaagaa ataaaagcag aactaccttt gattcaatga ccccaccatt gggtaactac    843660
     ccaaaggaaa aataaatcat catatcagaa tgataaccaa acttttgttt tcctgcagaa    843720
     ctattcacag tagcatagat atcaacctaa ggaattaacc tgtgtcttac aacagataat    843780
     tttatttaaa aagtcacata tatatacaat taaatactat ccagccatat taaggaatgg    843840
     aatcatgtat tttgcagcta catggatgga actctgggtc attattttaa gtctaataaa    843900
     cgagaagtag aacagcacac accacacttt ttcacttata aatgggagct aagtaatatg    843960
     tgcacagggc catagagaaa ggaatgatgg acattgcaga ctcagaaaca tgggagagca    844020
     gaagttggga gaatggtgag aaattactta aagggtatga tgtacattat ttggatcatg    844080
     gatacattaa agccaagacg actatgcaat atatatatgt aacaaaattg cagtcactcc    844140
     ccgtaaatgt atacgtataa aataaaaaca aatacaattg aaatgtgaaa ttattaacag    844200
     ttgatcaaca atcctgaaaa ttaaaacgtg tgatcaataa atgaaaataa ttttagttga    844260
     acttcatcaa attttaaaac attttctatt caaccgacta tcaagaaact gaaggacaag    844320
     cttcagggga gaaagtattt gcaaatcata catctaacaa tgtaattata ataagaaccc    844380
     tcaaaactca acagtaaaaa gaagcaatct gtttataaaa taggcaaagg tttgtgcaaa    844440
     ctttctatca aagaagatgt acagatgaca catcagcata tgaatgtaat tttccagtag    844500
     agaaattcaa atcaagacca aaaggagaca cgactttagg ctttagaatg aggaaaaata    844560
     aagaagaaat actgacagta ccagtgtcag tgagcatgcc aggccccgag agactaaaat    844620
     attgctagtg caaaatgaaa gagtttctgg gagaactgtt tacagtttct tataaaatta    844680
     aacatgtcct taatccatga cctagaactc tcatccctga gtatgtccta caaaggatta    844740
     aaatcatgtg ttcacacaca cacgtattca gatgtttaac attgtgtgtg tgtgtgtgtg    844800
     gtgtgtgtgt gtgtgtgtta gaaactaaaa acaacatgaa catctttgaa aatttgacac    844860
     aaaccattac aggtgaactc cagactttct tctgagtgac agaaggcctg cctgaaagat    844920
     ccccagagac acagtcatgg atttcactgt caccctcaca tgtcactggc ttgggctggg    844980
     ctctctctgg ctcttccctg accaggacca gatgttgagc tccactacct gcagttggaa    845040
     gtttatattt tcaacaatgc actgagggct aagttgctct acagactgaa ccaaacaaac    845100
     atgggcgcct ttgaacaaac agtgcctgac atttgtaccg accccaggag aactcttccc    845160
     gctctctttc ttcttggttc tctcctgcag gccagcagcc ctgcagttta gcctggacct    845220
     ctcatgcatc tacccatctc ctcccaagtg cattttacca cagcctccac tgtttttgta    845280
     attctccaca ctctgttgta aatgaagtca ggtccttcga gaccagattc gggactctat    845340
     tttatgacca aatttcagcc tcacccccac tcctgagaca gagctcctaa ataagattct    845400
     gcaggtggag attaagagcg tttttcttcc tcaatgtagg agatgagtgc tcagtggagg    845460
     gatggggagg agctttccac tttaccagca tgcagatcct gctggggtgg accctctgcc    845520
     ataggagcag ggctgggaac caggcagcca atgtcctcct ccttggtgct gcactcaggg    845580
     cagagcctgc atccatgagt ggggctgtgt ggaagaagtg agtctctggt tctcagtaag    845640
     tcttgtctag aactgagcct cagcagcatg acctgtgggc aaggtcggag ggccaatgtc    845700
     ctggcccctg gggaagagtg tgctctgctg ggagctgggc agaaggaatc ctgtctactt    845760
     ggctgcagcc atctgtggtg gaatttacat catgctgagc tcgggcatag aaggaaggaa    845820
     gagaatcttt gatcaaatat caatatcatg tctgacctta ctgaggtttt gtagattatc    845880
     tcgaataaat gttccttcac tttctgtatg ttgacagggc ctttccagac ccgttaattt    845940
     ttcaaaatta atctcaccag tttcatggag gcatggatat atcgagctcc ttgtaccgtc    846000
     atgtgagatg tggaactgtt tccttcactg gttataaaat aatgggttga cttttttccc    846060
     aacactttac agataccatc aattgtccta ttgcttatat ggttataacc aaagcaatgc    846120
     tgatattatt catttctatt ctattgcaag gtatgccccc tctactcgtc tccaattgct    846180
     ggcatgtatt tccatttgtc tgtatgtttt agtaatttga atatgattca cctaagagtg    846240
     tttgtgtgtg tgtgggtatg tgtgtgtcgt gtgtgtgtgt gttaagggct tatcctgcta    846300
     tttggtgttc tctgacctgc actgatccat gacttacaat ttctctaatt tggaggaaaa    846360
     aatagaggga aagtgagttt gagtccagac acaaacctca ctgaagtgag cacaggattc    846420
     taaccccagg ggtgctcagg acacgctgat cacaagagcc agcctcaggg gcaggtgcag    846480
     atggaggctg agctcttctt tcccgtcagc actgtggctt cttctccata aaacatcttc    846540
     ccttgaggaa tttatctgaa ttcatgattt tttgcttatc tccaaagtct ctgaaatgaa    846600
     aatgtttgtt ttaataaaag aaaaatcttc tcacatgtgt aaaagacagt ttcctacagt    846660
     cgcttaatct tgtctttaaa tgtcaattat caatgcttct gaagtaaatc agctaaatct    846720
     ataaaagaca ctttgtttaa ctcaacactg cagcccagca tgacagcact gaaagggcca    846780
     gcaagcagca ggcaggatac ggtgcatgcc agacactggg gcggaaggag ttagtatcca    846840
     gtgcaagaga agaaagtgcc cgcggtggtc actgtcagga cccaagctca cagctccaat    846900
     tgcaggtgac cccatgcaaa ggaaaagata ccctcaccaa tgtttagtgt ggatgtcgag    846960
     tctgatggtg ccacattcac acaccacgtg aatatggaaa gatttaccaa ctccattatt    847020
     gaggcgtctg ctgagggctg cacaggcctc tcaataaagt ccaaactggc tcaatagagc    847080
     aggaaagaag ctgcctcagg gtttttatga tggtttggtg ttgaaggaat ggtggggctt    847140
     cctactaagg ggaaggagct tgtataattt gaacctaata ctggcatcca gtgaggacac    847200
     ttccatgatt ttctactaaa tttcccatgt gtgggggaca aaagtgaagg aaaaggatta    847260
     atcctgtcat cagtaatgag gcatcaaaat agagtctgat gacttattct acttagcaac    847320
     ttaagcaaat gagtgaaaaa tgaggtaagt gttcactctg ggtggacagc aaataggtct    847380
     gcagaaaata gagttttttt aagtgtaagc acataacaaa aagaaagaga agaacaaatt    847440
     gaacagggat cttggaccaa tgtcctgggt ggaagctctt cactctgtga gatcatcagc    847500
     ttgctctgag ggtctcgggt caagtccttc ttcaatatat ctcaggcctt acaggataca    847560
     tgggaatgct cagcttggtg tccttcacta gggaaaagaa agagaaatca gactgtcact    847620
     gtgtctatat agaaagggaa gacataagag actccatttt gaaaaagacc tgtactttaa    847680
     acaattgctt tgctgagatg ttgttaattt gtagctttgc cccagccatt ttgacccagc    847740
     cactttgagc caacctggag ctcacaaaaa catgtgttgt ataaaatcaa ggtttaaggg    847800
     atctagggct gtgcaggacg tgccttgtta acaaaatgtt tacaagcagt atacttgata    847860
     aaggtcatcc gcattgtcta ctctcaataa accaggggca cagtgcactg tggaaagccg    847920
     cagggacctc tgcccttgaa ggcggggtat tgtccaaggt ttcttcccat gtgatagtct    847980
     gagatatggc ctcgtgggat gagaaagacc tgaccgtccc ccagcctgac tcccgtaaag    848040
     ggtctgtgct gaggagggtt agtaaaagag gaaagcctct tgcagttgag ttagaggaag    848100
     gccactgtct tctgcctgcc cctgggaact gaatgtcttg gtataaaacc cgattgcact    848160
     tttgttcaat tttgagatag gagaaaaacc gccttatggt gggaggcgag acatatttgc    848220
     agtaatgctg ccttgttatt ctttatgccg ctgagatgtt tgggcggaga gaaacataaa    848280
     tctggcctac atgcatgtcc aggcatagta ccttcccttg aacttaatta cgacatagat    848340
     tcttttgctc acatgttttt tgctgacctt ctccccacta tcaccctact ctcctactac    848400
     attccttttt gctgaaataa tgaaaataat aatcaataaa aactgaggga cctcagaggc    848460
     cggtgccagt gcaggtcctt ggtatgctga gtgccggttc cctgggccca ctgttgtttc    848520
     tctatacttt gtttctgtgt cttatttctt ttctcagtct ctcatcccac ctgacgagga    848580
     atacccacag gtgtggaggg gcaggtctcc ccttcgtact tgagcagcct tacaactggg    848640
     tgggtttatg aattaattcc tcggttgcat tacatgaaaa atcacacaat ctacaaattt    848700
     taaaaaataa gcctttactt tttataaaag attacagcca tttcacaggc tgggaagcac    848760
     agccattggt gaagacgaga gacaggcact cccaaggagg agcggtgggg cagaaacttt    848820
     atgctgaacg tgttggctaa acagacataa tcaacggatt acaggagggg ctatgcgtgt    848880
     tcataaaggt ggtcatgaca catgaatatg ggagcaaaca tacacgtcac atgtgaccca    848940
     cttagcagtg gggacttagc atttaaatgt gttacagtta ggccctaagt gccaaaagct    849000
     ggagcagggg cacaaagacc ctcagagcgc agcctccata aatggccaga accaggcctt    849060
     ggtcagtggt ctctgatcag gagaaaattc ctgaaatcaa tctggtgtcc catccaagct    849120
     ggggttatgg ctgtggaatg gggggtcagt tagtcagagg atgagctgca gttggttaaa    849180
     tcgtgcttat ctcaggatca gtgcttattt tgctgctaca gaagaaaata acagtttata    849240
     ttctttaaga gttggggtga gggatgtaac ccctgcctgg catggcctta ggtcttgttt    849300
     ataacggggc atcttattgc cccagagaat ctgttctgtc agtcttatga tctccatttt    849360
     aatgttagtg ttggtccatg ttgtctctga accacaaaag ggagtgggta taatgaggtg    849420
     tgtctgatct cttgttctgc ccagctggga actcagtttt taggtgtttt tggtgcctcc    849480
     ttggccaaga gggggaacca ctcaatctca tggggtgact gcaattttac ttttagttta    849540
     aagtttataa atatggacct aagtattgac attctaaaag tcactccttt gttttggcta    849600
     aaagtacttg gcaagagtcc ttgaaatgag gttcaagagc agtattttct catgacttta    849660
     tttgcaatag acacatgaga gactgttgaa aagatgaagt accaaagtag ccttaatctg    849720
     ttggtgaatg gagttggtat aatacagaaa tcactttcaa tattttgtaa cacatgcatg    849780
     cgtgcaataa gacagcggtg agcactgagt gtaaagtctc taaaggtacg atctttcagc    849840
     caccaaataa tagtgtaata acggacatac tctggaggag ctgactataa taccgtaaca    849900
     ccacggggag aaactttggc caaggaagtc caagattttc ttttcctttt cctttttttt    849960
     tttttttttg ttgttgttgt ttgagacaga gtctcactct gttgcccagg ctagagtgcg    850020
     gtggtgcgat ctcgactcac tgtgacctct gtctccctga ttcaagcgat tcctgtctcg    850080
     gcctcccaaa agctgatatt acaggcatgt gccaccacac ccagctaagt ttttcatatt    850140
     tttagtagag acggggtttt gccatgctgg ccaggatgat ctcgcactcc tgacctcaag    850200
     tggtcctcct gccttggcct cccgaagtgc tggtattaca gcgtgagcca ccacacccag    850260
     ccgagatttt cttaaagttt tgaagatttt aatttaaggg ttacaatttt ttcaacattt    850320
     ccagaaagct gtagacagta ttattctgtg ttatatgagt gattgtagtt ccttccattt    850380
     ttagatagta ttacaaacaa ttttagggct gcttgcttta aatagataga tgatagatgg    850440
     ctacatacat acatacatac atacgtacct cgatagagat atagttgcaa acataaatac    850500
     atagatagat agagttacag atgtatttta gttattatgc tacttttttc tttgcctttt    850560
     cttcctatgt tatgttagtc aggtctccag gacttaggag tgcatttaca atcccttccc    850620
     ttgtccattt tgacgtgttc tagacaatgc gagaatcctt ttgtgtgtgg atcacttcca    850680
     agtcatgtac tacccttgca ataaatgtgt ttgtgtatgt ctttgcctta ctgacaagct    850740
     ctgctgagtg cagttatttc tgccgtctga gctaacttta cacttggtag agaaatatat    850800
     tctagatgga ggcttccact attaatttaa attactaatt cataacctcc agcttaatta    850860
     ttgaggtata atctatcaga gatggtatta gaacaaaggt ttccaatgaa atctaacata    850920
     agggatgtga acaaattttc ctaatttagg taaaataacc ctgagcccat tcttagtatc    850980
     cagccatgtc tcctatctat cacactgaat ctcacactaa ctttgacctg acttgggact    851040
     tgtaagcttc aaatatgacc ttttgattgc aatgcctcta attcaggacg tgtggttatt    851100
     ttagccaaga gtaaagaaaa ccaaatttaa ctcactaaaa atgacgaaga ttaaaaccta    851160
     atgaaatgga aactgggata tgagtggctc cagacttggc tattttaaga gtctatgtgc    851220
     ccaggtgatt tcttttctcc gttttacaca caggcaaacc caaaatgtca gcttcatctc    851280
     ccgttgactc tcatcatctc gttcttcatc agcttgctta gaatgttgac ttccagctgc    851340
     atccatgttg ccacaaaaga catgatttta ttctttttct gtgaatgcat agtgttccat    851400
     ggtatacatg taccatattt tcttgatcaa attcaccatg aaaggacaca taggttggat    851460
     ttatgtctta ggtattaaga gtagcatagc aatgaacatg taagtgggtg tattttttgg    851520
     tagactgatt tattttcttt tgtgcatata cccacatatg agatggccgg gccaagtggc    851580
     agctctgctt caaatacttt tcaaatctcc agactgcttt ccacagtgct tggactaatt    851640
     cacattccac ccaacactgt acaagtgttt cctgttctct gcagcctcgc tccagactgc    851700
     tttccacagt gcttggacta attcacattc cacccaacac tgtacaagtg tttcctgttc    851760
     ttcgcaccct ctctccagac tgctttccaa gattcctctc ctccactttg acactttggt    851820
     atatccatgt gtgtcctctc tggttcagcc agggacagag aaccaggaag acatcatgca    851880
     ttttctagag ggagctggct tggggaatca acgaccggag tagaaagtgc ctctgtccat    851940
     gcatcacagg cctgagggcc ctgggcagga ggtcaggaag agaggatgac cagacagctg    852000
     gggaaactgg gatgaacaaa gccagtttcc ttcctggtga ggagggacag gtcgaagcct    852060
     cgtgtccctg tagggatgaa gcacactcac ctgcccactg ctacctgcaa atcaggtgac    852120
     cttcagagca gcagctcctg agtcactgat ggtgacttcc tcctcctcca aaccctctag    852180
     gatctgtgct tctcagagtg ggaacccccg caggtcccca agttttcaat ggattcacct    852240
     agctagtgtc tctcctcagc cccatcccca ggctgctgcc ctggtgctgt gtacactgtc    852300
     acaacagggg tgacagtaac agctggtgcc tgcactttcc aggccaagaa gtttgaaaga    852360
     agcaagtcac agggccagcc gggatatggg gtaatcacac aaatttcctt cttgggggaa    852420
     gtgctgggaa acatccttga gtcctcctca gctcacatgc ggctgctgct gttcatccca    852480
     gtgtggggac acatgagccc tccccatgcc caggatggag ctgtttcaat cacagcagga    852540
     tgggaatgtg tcttctctat gggggctttc ggagcacagc tctcctgttc ctgagatttg    852600
     agatttataa agacaagtcc cacagagtgg acttgcacaa ataagacagt acaggatccc    852660
     caggagagac atcccatatg agaagggaca ggcaggtccc atcactgacg ctggtcctca    852720
     gaaaccctga ccagctactg accttcccag gtcccctgtt cctgatcagt gcctgtgtct    852780
     gctcctgaga agatccccat caacagtccc cagggttctg cccccttcct tgatgtgcct    852840
     ctgggagtgg ggctggctgg gctgtgggtc tgctgtgact ttccccacag gtcagcggct    852900
     cagtgcaggc tgcaccgact cggtggggct gggctgtgtc tgactctccc gggggagcct    852960
     gaatgcttct cagaggcatt tgcaggatcc caggtcctgg gagaacaggg aagcaacagt    853020
     gtgtgcaggg ccccgtctcc aggtgtaagg tgtttgctgt tgctctactt gacaagggca    853080
     tatccccagg cctaggggca ctgtgggtgc acaaccaatg tgctagacac agggagtaag    853140
     gcacagttcc ctcccattac tgtggggaat ctaggcactg tgtgtgtgtg tgtgtatgtg    853200
     tgtatgtaga ttaggagttg tggagtgggc aggtttagaa attatctgat gagatgttca    853260
     tccaaaggaa ctgacaagag attaggtgtt ttcccaaagt ccctgggagt tcctggactc    853320
     actatgagtg tggactgatc cagtgcttcc ggagctccag ggaaggggct ccctggtggt    853380
     ttcatagaat ccttgcttgg ggtgtttctg cagagttcac tggctttcct acggccaatt    853440
     tactattgta agagatggtg tcagcagtac atggtgtcac gtgtcactaa aggagcattc    853500
     cgagccagga cacagccact tcatacttgg gggaaaatgc tctgggagcc cagacaggag    853560
     cctctctgca gtgcaagggc tgggctgcag gggacgctca aagcccaccc agtacaagtt    853620
     tcagccccag agcaggtgca caggaggctg gggagggatt cctctcgaga tctgtgtcat    853680
     tctttaaaaa atctaaaata catatttgac aactgaacat tctataaata tcctattcaa    853740
     ttgcgagcat ttatcaaact tgatgttgta atgagaacca cttttacact ggagatttct    853800
     aacccttcta gatatcttaa tagtatgcag ctggaggtta aggaaactac ttttcttttc    853860
     tataaataag tgaaactttt ggagaaacac actcatcccc caaataatac attcatgtat    853920
     taaagcctag aaatgcttat attacctctg agccatttga gtgtgggttc ccagtgaagt    853980
     cctgttctag ggaaaattgt tagccaatgg gagaagctct gtcaacatag agcttaggga    854040
     tatggcaggg ctcacatggc ctctaagggg attacagctt gaaccttgag catcctccgt    854100
     gtgtcatctc tctgctcttt ctcatgcaat atcaggtatg aaataggatc actcatgaat    854160
     atgcaaataa ctgaggtaaa tatagctatc tttgggccct gagagcatca cccaacaacc    854220
     acacccctcc taagaagaag cccctagacc acagctccac accatggact ggacctggag    854280
     gatcctcttc ttggtggcag cagcaacagg taaggggctc cccagtcact gggctgaggg    854340
     agaaaccagc acagtcaagt gagacttcat gcactcccat ctcctctcca caggtgccca    854400
     ctcccaggtg cagctggtgc aatctgggtc tgagttgaag aagcctgggg cctcagtgaa    854460
     ggtttcctgc aaggcttctg gatacacctt cactagctat gctatgaatt gggtgcgaca    854520
     ggcccctgga caagggcttg agtggatggg atggatcaac accaacactg ggaacccaac    854580
     gtatgcccag ggcttcacag gacggtttgt cttctccttg gacacctctg tcagcacggc    854640
     atatctgcag atctgcagcc taaaggctga ggacactgcc gtgtattact gtgcgagaga    854700
     cacagtgtgg aaacccacat cctgagagtg tcaaaaaccc tgtgggagga ggcagctgtg    854760
     ctgagctgag gcagtgacag ggacaacgtg gctgcaccct ttgtagggtt ataaacatac    854820
     atatatatgc acccaacagt ggagtaccca gatatatcaa gcaaacatta ttagagctaa    854880
     acagagagat agacccaata cagtaaaagc tggagacttt aacaccccac cctcagcatt    854940
     ggacagatct tccagacaga aaattaacaa tgaaatctta gactaaatgt acatgacaga    855000
     ccaaatggac ataatggata ttgacagaac acttcatcca atggctgtag agtgcacagt    855060
     cttttcctaa acatatggat cagtctcaag gatagaccat atagtagacc agaaaattag    855120
     tcatttttta attgagaaga tatcaagtac cttctctgat cataatggaa gaaaactaaa    855180
     ttaataagag gaatcttgga aaactatgca aacacatgga aattaaacaa tatgctccct    855240
     aattaaacaa tgtgctccca aattgaacca catgctccaa aatgggttaa taaagacata    855300
     aaaaatttta aaatttattg aaagaaatga taaagaagac aaagcatatg aaaacctatg    855360
     aaatacagtg aaagcagtat taagaggaaa gtttatagct ataagcactt actttaaaaa    855420
     agtagacaac cataaaataa acatcctagg aatgtctttt aaaaaataga aaagcaagag    855480
     caaatcaaac ccaaacacag taaaaaaaaa aaaaggaaag atcaaaacgt taatacataa    855540
     aattgaaata aagaaaacaa tacaaaagat ggatcaaatg aaaagctgtt ttttaaaaag    855600
     ataaaagaca tctacaaacc tttaaccaga ctaagaaaaa aagaaagaag accaaagtaa    855660
     atgaaatcag agagaaaaat ggaaatatta caaccaatgg tgcagaaaat caaagaatca    855720
     ttagaggcta gcaggaacaa ctaaatgcca ataaattgaa atatctggaa gaaatggata    855780
     gatttagaga cacatacaac tttgattgaa cgaggaagaa attgaaaacc tgaacagatc    855840
     agtaacaagt agtgagaatg aagccgtaat aaaaggtctc ccagcaaata aaatctcggg    855900
     acttgatgac ttcattgctc aattatgaca aatatttaaa gaagagataa tacctaaccc    855960
     cactcaaaat attccaaaaa cagataaaga ggggaggaat tcttccaaat ctcctgtacg    856020
     aggccagcat tacccttata caaaaaccag acaaagacac acctaaaaaa aaagaaaact    856080
     ataggtcaat atcactgatg aatatggacg caaaactcct caaggaaatc ttagcaaacc    856140
     aaattcaata acataacaaa agaaccattc atcatgacca agtgcagttt atcccaggga    856200
     tgcaaggatg gttcaaccta tgcaaatcaa tcaatgtgac acatcatgtc aacagaatga    856260
     agggcagaag acataagatc aggccttacc ttgttctcct cactcaaagt ctaccatcag    856320
     tgctttagtc ctcttggtca gtcctgctgt acttggtctc ccatgattct gttcccaggt    856380
     cctgaaacac tgggctcctg cacacctgga gtctctctcc tctttgaaca tggagatcct    856440
     gtatgctcag agaggggtga tgactcatgt ggctcccatg ccccatgcaa acaattcttc    856500
     caaaacagtt gtgacttttc ctgttggaaa ttgaaagggg atgtgggaat ctatgtgtgt    856560
     gtgtgtgtgt gtgtgtgtgt gtgtgtgtgt gtgtgtttcc tcaggcctgt tgagaatgca    856620
     gcagggctct atgtataaca ggaaagcaat gtttctccat accatctatt ttcttataaa    856680
     gtgttcatag gtaatcaaaa ggaaattaat cacagtttat aagatgttgt ggactggagt    856740
     gatgttgtca ctcaaggcta ctatgggggc tccaggaaaa gaaatctaga aaaggataag    856800
     actgtgagca aagaagcaaa acctggggag caacagggag ggatgagcag gggtgactca    856860
     accgggaagg gattaaacca cataccacag agagtgaaaa cgctgtgctc agcaaagagg    856920
     agaacttact cagcatttcc cccaatcctc cctccagaag cgtgactgcc aggaaagttc    856980
     acaatgtatc atctccagct gggaacccga aagaagcagg aggactgtag accacaggtc    857040
     ctgggcacca tgtgtttccc cagttcctac tcctattctt ccctctctgt ccagcaaacc    857100
     tgcttgtttc tgtcacctgc cttcctgccc ttggggtgag aatggatgcc ttgaacactc    857160
     agcagaggga tgctctttca gaggaggaat gacttcgtgt catctgacgc ctgagcagga    857220
     aaatcacccc tgagaagagg ctccgagaca gagggtcgcc atggttcctg ccccttacat    857280
     gtgcggggct cctggcacat gcacacttct cctcaaactc tgtttaggag aacaatgctg    857340
     ggtgttgtca gcctctctcc tccctagata ctgctctgtc atcagcctga gcacaatttc    857400
     cccatgaaag gcgttaatct ttctctcaat tagatcaact ttttacatgt ctttgatgga    857460
     tgacagcttt tcagtgtgaa attctagaaa taagaaatgg ggtgatgggg acagtgagct    857520
     ctgtgtgtga ctcaccagga tgttccctct tattccaggt gtactgtccc aggtgcagct    857580
     gcaggagtca ggtccaggac ccatgaggcc ctcagacact gccctccacc tgtgctgtgt    857640
     ctggattctt tttttttacc ggtggatatt atttgagctg aatccaccat cccctgggca    857700
     acagattaga atggctgggg cacagctacc aggaatacac atcatgatca ccaagatcac    857760
     caagatcatc atcaagatca ccaaggaata cacatcatgc accctctacc caggtctgca    857820
     tctccatcaa caatgactca accaagagcc agttctctgt gaggctcagc tccatggctg    857880
     cctaggacat ggctgagtat tactgtgaaa gactcagtga ggaggtgtcc ttgtgagccc    857940
     tgacacaaaa cttgctctca gggcactgag gaccgccagc aagactcagg accgtgaggg    858000
     ggactcaaga ccaccagggg aagagcaggt tgcaggaagc acagggccag ccccagagca    858060
     ggtgtgggtg gagatgagag gctggtttac tgccagggtg tggggctgcc tctacatcta    858120
     catgtttcct ccagggaccc tcccttattt catgatgctg tgcctagctc tacgtctctg    858180
     aaataccaca gttttgttgt accaggagga aaccttctca caggcactaa atgcggaaca    858240
     atccctctgc cagtggtcac cagggtcaca gccctgtgga agctgagggg aacccagtga    858300
     gtcttctcca gtcacactca ggacagggac cttagtgggg ttccctgaag aaagcagtat    858360
     ttaggaattc taacctcagc caagagagag gctgggccag ggtcagtgtc aggtagaacc    858420
     tcacaggatt tacgtctgac ccttctcctg acactaaagt atgcaaatct gtatcagcac    858480
     tgatctgggg ccccttttgc tcgtagccca ttctatttct tgttagttgt gtttgttgtt    858540
     gattttcctt tcgctgttcc tgatccctgt aaagtggaga tgtggttctt gctgtaaaaa    858600
     ttccagggct caagcccttt ccctgcagct caggtggggc tcagactgtg gctcctgcag    858660
     ccacgtggga gaggctgacg ggactatctt ctctcccgtt gctcagaacc ctccagtgtg    858720
     ttgtgtggag actcacctgg gaatgcaagt gaccaatagt tgtgaagggg atgagcttgt    858780
     gtggtcaaaa tgggatgtgg atgtggaatt tatcctgtgt tgtgcaaact aacacaggtt    858840
     caccttcctc acctgtagtg ttagaaagag agtgggaaag ttgtcagaat caaaatggag    858900
     ccacttgtgt taacaccctg acaaatgaaa ctaggaatga ccatgaagga gggttcttaa    858960
     gcccatattc ttgataacaa gaactatcat aaatagactc tgcttaacca caatcttggg    859020
     aagaagacac cacaatctta taaagaatta cttttgcaag gacatcatcc cagacactgc    859080
     cagttcaacc ttacactgat gccacccttg atattgattc tacaactaca ggatcgttct    859140
     ctcaaaacaa cttttgtaac ccatcccatt ttcacttcat aaacctatga actgatatcc    859200
     tggattactg ctgcgtttgt tgataacgtt aaataataag gccttttgac aatgtttgaa    859260
     gctgcttttc tctgatgtct cttctaaaat aaagaatttc caagttaatg acaataaaaa    859320
     gataattagg aagatttggt gggaaggcat ctttaacatc ctgttgaatg cttctgcata    859380
     gcacatgagt cccctaaaat actttgctgt atctgcattt aatgaatcag agtctaatat    859440
     tgaagatgta attgaaaata catgggcttt tgatgaatca agtcatacgg tgataatgtt    859500
     tgtgtccttg agaaagcaga ccctgtgttg taagttttag cagatttacc ttcggcaaaa    859560
     aagctcatgt gtttctgaat gttatgcgac ttttaactta acgatgcaac atatgattca    859620
     gttttattta gaactttcca gaagattttt ggcagtaagg acagtgcagc atttgagtaa    859680
     cactaaggag ctaaaaagag ttattttgta attgctcctg tgaggtatgc acatcgctca    859740
     ctcagtattg aaattcaaat gccacagatg ggaaaatcag aataaggaaa attttatgaa    859800
     ctggcatggc tgtagttttt gtcaagcaag taattcatgt ctgcaagaaa atacatacaa    859860
     caataaaaca cattcaagtc cagggggagg ctgacctctg ccctctatat tacaagtaca    859920
     aggtggtatc acatccaaat tatttttcag actccagggt ataaagcgct tttggacggt    859980
     gaagctaaca gctctcccct caggtgtggc tgaggtatgt ggagaatgca gagttgtgtt    860040
     catgaagaag attacattat tgtgtctgga gacagctccc cagggtgtgt ctcagatgtc    860100
     agacatgcgt ctataagtca agcaaagaga acacattgag tccaaaaatc agcatattct    860160
     tacaggcacc cattgctcca tcacatgggt gagaaatttt tgagacaagt gaagtgtgag    860220
     ttcacagtaa gtgatgcagt tatcatattt ccatgaactt tcattaacaa ggcaaggact    860280
     tctctagatc actcatacac aaatatacaa aatgtatttt tgcatttgcg agtgtctaga    860340
     gaaaaaagaa tctgttgaga aaacttcttc aggttacaga gatctgttta agttggagat    860400
     ctcacaggag tgtgtctttg agtgaatact ggcctattaa ttaaataggt caaaattccc    860460
     tctgttggag tagccttccg attatgtaga tttctttatt gcttcctgag ttgtgaaaca    860520
     taaacccaag cattgactta ctggaatttg actgctgtgt tgataaaatt tctgatatgg    860580
     tttcttccaa tgatttaaga atagctttcc cctttcttta ctccaggaaa tgaattttca    860640
     caaggtctca ggacaccata tttcaggtgc tttagttaaa gagactgact tcttgggggc    860700
     aggcctcttt cttctaacaa tgagctcact tcttctgcaa agcattcagt ctgtggctct    860760
     attgccatga atcatgaaga ttttacactc caatgcacta aaaataatgc ctcttcaatt    860820
     aaatttccat tggcattgac atgaattggc cactcttgga tgtgtgtcct atgttatgag    860880
     cccaacatgt cagcggactg agaatcctct gttagctact tctgtgtgta gcactgagca    860940
     gactaacaat cctcagagtc atccatgaag ggagtcctga ggttcctggg gttcttggag    861000
     acattcaggt gagttgagag gagaaacagg attgtgggct gccagccatt tcaacaacaa    861060
     tgggagtcat taccatctaa gtgtaaagtc tacatcattc aaaatatcct ccatgacagg    861120
     ctgataagaa gaaatccaac cacacaatgg ctccatggca gctctttagt atatttggga    861180
     gtgaggcttt tttagggaag aaatgtccac tccagtgtgt ccctgatgct gctctcagct    861240
     aatgtaaaca gtgaactgga accaagattc tctgagatca atgtgaggat taatgctgct    861300
     ccaatgtgtt taaacaagca tgtggcaatt tagacgagcc tggctgtgtg gtttgttata    861360
     tgtaaatctg aactaaataa acagaaaggg catgtctgaa ctagtgtgag ggtgagagag    861420
     ctcatagacc ccaaactcgt ttttatttcc tcctggaacc tccaggtcct ttggtacctc    861480
     caggtacttt gtccatgcat cacaggcctg agggccctgg gcaggaggtc aggaagagag    861540
     gatgaccaga cagctgggga aaccgggatg aacaaagcca gtttccttcc tggtgaggag    861600
     gggcaggtcg aagcctcgtg tccctgtagg gatgaagcac actcacctgc ccactgctac    861660
     ctgccaatca ggtgacctgc agagcagcag ctcctgagtc actgatggtg acttcctcct    861720
     cctccaaacc ctctaggatc tgtgcttctc agagtaggaa ccccagcagg tccccaagtt    861780
     ttcaatggat tcacctagct agtgtctctc ctcagcccca tccccaggct cctgccctgg    861840
     tgctgtgtac agtgtcacaa caggagtgac agtaacagct ggtgcctgca ctttccaggc    861900
     caagaagttt gaaagaagca agtcacaggg ccagccggga tatggggtaa tcacacaaat    861960
     ttccttcttg ggggaagtgc tgggaaacat ccttgagtcc tcctcagctc acatgcggct    862020
     gctgctgttc atcccagtgt gggggacaca tgagccctcc ccatgcccag gatggagctg    862080
     tttcaatcac agcaggatgg gaatgtgtct tctctatggg ggcttttgga gcacagctct    862140
     cctgttcctg agatttgaga tttataaaga caagtcccac agagtggacc tgcacaaata    862200
     agacagtaca ggatcctcag gagagacatc ccataagaga agggacaggc aggtcccatc    862260
     acagacgctc atcctcagaa accctgacca gctactcacc ttcacaggtc cccagttcct    862320
     gatcagtgcc tgtgtctgct cctgagaaga gatgaaaatt caagacaaat acttctcatg    862380
     atcataggct taaaaattct caacgtagga aatcaaatcc agtagtttat caaaaagtta    862440
     acacactgca atcaagtagg cttcattctg caaatgcaag gctgctttaa ctgctttcca    862500
     cagtgcttgg actaattcac attccatcca gcactgtaca agtgctttaa cctgtgcaaa    862560
     gcaataaatg tgatgctcta ctaaacagaa tcaaaagcaa aaaccatgtg agtattccag    862620
     cagatgcagg aaaaaagctt tcaatagaat ctaacaacgc ttcatgataa aaatcctcaa    862680
     aattatagac attgaaggag cacacctcaa tctgatgagc cgtctatgac aaacccatgg    862740
     ccaacatcat actgaatggg caaacactgg aagcattttc cttaagaaca gaaacaagac    862800
     aaggatgccc gctctcacca cttctattca gcctagtact ggaagtattc accagatcag    862860
     gcaagagaaa gaaataaaag gcatccaaat aaaagaaaaa gaagaatgca aaccctctgt    862920
     tttcactgat aatatgattc tatatgtaga aaatattgaa ggctctgcca aaagtctcct    862980
     agtattgata cattcgttta ggaatgtttc aggatataaa atcaatgtac gaaaatcaca    863040
     gtaacctttc tagtcaccaa caacgttctg gctgagagtg gaatcaagaa catgctctcc    863100
     caatagccat caagacagtg gaatacctag gaatagagtt aagccaatga gatgaaatat    863160
     ctctactaga aaaacagcaa aacacatgtg aaaaaaatca gagataaaac aaataaatgg    863220
     aaaaataaac attccatgct tatgaattta aacaataaat aatatatgaa aaagttttaa    863280
     aatttgtgat taattctacg tagcaatgcc atttttttca acatttacag aatattgttg    863340
     ctggtaatag cagtttagca tttgagtaat ggaaacacct tacagaatca ctttataaca    863400
     gttgcttcct gtagtgggtg tgcttcagtt acctgatgaa aacatcggca tgtcacaggt    863460
     cgaaaacata aaatatgtct atcaactgag aaggttgtag gcttttgtca agtaattact    863520
     tcaagtcatt cagaaaaaca gcagacagta aacgaaactt gttcaaattc catggagagt    863580
     caaagctgaa gaaaatccaa ctaaaagtgt ccaaaggccc ccaaggctca gttcctcggc    863640
     aagtcccacc ttcacaggtt ttccagaatt ttgtttttaa ctgatttaca ctgagtgaag    863700
     tagattacca ggagttttcc attattttaa cataaaatcg ggggctaagt gttgttattt    863760
     tataaagaca tctttgaatc tctcaggaaa tttacctcct gagggttcag ataagatcct    863820
     actctaggtt aagattacat gtttggcgca atgaactggt acaaatctta ctttagcttt    863880
     catgttatct atttttccgc actgacttcc acctttttat tagtcaagta tatgggatgg    863940
     aagagtgctt ctaagaggtc cttaacttgc ccatttcaat ggattttcaa gaagacatga    864000
     gaaaccactt cgtttgcaaa gcatcccaaa gccatgtcct gctccagaaa cgtgatctca    864060
     tttcctggtc gtttcttaac tgacacgctc taatcagtgc atctgggcca atttgaaatg    864120
     aggtgaagaa atgtgtccta aagtaaagct aacattgtaa taggaattcg tgtttaaaaa    864180
     catttagttt tattattggg caggatccat caacatataa cagttgaagt ttctcaacag    864240
     gagtttaata aatataagga atgtacagaa gtgtttccta atttagataa aatgtagtga    864300
     ttacctaggc tgtgagtgaa atcttggtaa tctgctatgt ccatgcccat cactctgtgc    864360
     acctcaaact aattttgacc tcagcagaaa cgaggttaat tttcaaatta gtaatttttt    864420
     tccaatttaa tttatttaga tgattatgaa tgactatttt agcagaaaga gaatcaagga    864480
     aacttgaact aaataaccag aatttaaaat gacaaacaat tcaacaagat gccaggatgt    864540
     gagtggctcc aggcccaatt agttcaaaac tttgtgtgcc caggttctgt attttcctaa    864600
     aattgcacat ctccagtcca gcttcatgcc acagtcgact cccaccgtgt aagacatggc    864660
     gacagcattc ccaaaggtcg tgtgcaattc tgaaaatgga ataagcaagg tgaggagatg    864720
     atagtttata ttctcctctg aggaggaaga attgcctcaa cagaccactt ctcctgcatc    864780
     tgtgactaga actatgtcac aggcacacat ggagcaaaat ccctcaaggg gataatacgg    864840
     taacattaat cagggtgatc attttttttt tttttttttt ttttttgaga cggagtctcg    864900
     ctctgtcgtc caggctggag tgcagtggcg ggatctcggc tcactgcaag ctccgcctcc    864960
     cgggttcacg ccattctcct gcctcagcct cccaagtagc tgggactaca ggctcccgcc    865020
     actacgcccg gctaattttt tgtattttta gtagagacgg ggtttcgccg ttttagccgg    865080
     gatggtctcg atctcctgac ctcgtgatcc gcccgcctcg gcctcccaaa gtgctgggat    865140
     tacaggcgtg agccaccgcg cccggccgat catttttaat actataaaat tcatagatga    865200
     cactgcattc cagcctgaga gacagagcaa gaccctgtct ctaagaaaaa tagaacaaat    865260
     caatagatac tgataccaac attttagata atgaaatttt cataacctaa ttttaaatac    865320
     gctcacatca ttacataaat cttcccggaa atattcctag tcatgttgag tttcatcaac    865380
     ttttccagtg ttcaaatcta gaaatccaat agactcttga ggataaatca aaatgagggc    865440
     agtgaaactg gtatctgttc agcacctgtt aactcaggag gactcaatac accctggcac    865500
     actgctgctt ctccgaatgg ctcacaagga ttccagctct ctctccatcc tcctcaaaca    865560
     tctgaccgca cttgccctaa gttcactctc tgctcttagt ctgtgctctg aagtcttccc    865620
     agaggtgaaa gtgagctgtc agatggaact tccctctcac ctcagcgtgg aatttactac    865680
     tacatttaac tatcactctt tccataatgg ttgatttctt ggtctgttca ttacagacca    865740
     aaggcatctg atttgaatct ttatttcttt gcatttgtct ccatgacaat tttgggaggt    865800
     tttaccacca gcactatgac acgatgtagt aacatgacac atttgtggtt aacaacacct    865860
     acaaattcag aagctcttcg gttctcttcc cagcaaatat agctgcttcc tttctgtgta    865920
     tgaacacatc ctggaaaccc gtacacaccc acatagatat atacatggct atgacatttt    865980
     cttctctgta agtgaaaatt aacctcaatt tcatacgaag ttcatcattt ccctgaaggt    866040
     gaaggtaggt cgtttttcat ctattctaac aaaagtctca gacaccagct ggtaagtgag    866100
     gagctaccct gcttctggat gttggatctg tctcttcccc tttgctttac cacagaagat    866160
     tggccacttg tccaggtccc aagaagagag tccaggtttg tcctgataat atgactcatc    866220
     caacttctga taactctact gttacacaca ttcatggagg tagcctatta attacataat    866280
     tcactaaaca aataccaaat acccacattg caatacccca tacccaaggg tatgttcatg    866340
     cattcaatga aggaaaaggc ttttcagaga cagatagatc aggctagatt ggtcaatata    866400
     tgggtgaaaa cactggactt gaatgtatct gttttccccc atgtcacatg tgagacctgt    866460
     cagacaggac cagggttcct tgtgcactca gaggtgaggg ctcacagaat tcctctctgg    866520
     tttccaggaa aggtaactgc agtaatcttg gtaatgagaa tatcctccag tgctgaccta    866580
     ttatagagtt tacatatgaa attttcactg caattcacaa tctactcttt cacacagaag    866640
     tgtacagagg tcaggacaca tcctcagggt cacacattga gaatgatgaa gatatgtccc    866700
     acgagttttt cctaaggtct cagaaagaat tccaggactc aaaaggtctc agagggcagc    866760
     tcccagtgcc ttagttaaaa tggtggctca ggcctgtaat cccagcattt tgggaggcta    866820
     aggcaagtgg atcacctgag gtcgggacat tgagactagc ttggccaaca tggtgaaacc    866880
     ttatctctac taaaaatata aaaattagac gggggtggtt gtgcgtgcct gtactcccag    866940
     ctactcggga ggctgaggca ggagaatcac ttgaacccag gaggcggagg ttgcggtgag    867000
     ccgagatcgg gacactgcac tctagcctgg gcaaaggagc aaaagttcat ctaaaaaatt    867060
     tattttaatt taaaaatttt aaaaaaatgg cccactccct agaacagaga gattccctct    867120
     aaacatgatg gaggtcccga actatacatt aagtgaatcc tggtgtgtct gaactcacat    867180
     gattattacg ttaagctgct gttccaatct acttcctcac ctgggaaaag aggagccagg    867240
     gcatggctag ttgaggcccc aggaagagaa ctgagttctc aaagggcaaa gcaagcatcc    867300
     tcatcccagg gcgagcctaa aagactgggg cctccctcat cccttttcac ctctttatac    867360
     aaaggcacca cctacatgca aatcctcact taggcaccca caggaaacca ccacacattt    867420
     ccttaaattc agggtccagc tcacatggga aatactttct gagagtcatg gacctcctgc    867480
     acaagaacat gaaacacctg tggttcttcc tcctcctggt ggcagctccc agatgtgagt    867540
     gtctcaaggc tgcagacatg ggggtatggg aggtgcctct gatcccaggg ctcactgtgg    867600
     gtctctctgt tcacaggggt cctgtctcag gtgcagctgc aggagtcggg cccaggactg    867660
     gtgaagcctt cggggaccct gtccctcacc tgcgctgtct ctggtggctc catcagcagt    867720
     agtaactggt ggagttgggt ccgccagccc ccagggaagg ggctggagtg gattggggaa    867780
     atctatcata gtgggagcac caactacaac ccgtccctca agagtcgagt caccatatca    867840
     gtagacaagt ccaagaacca gttctccctg aagctgagct ctgtgaccgc cgcggacacg    867900
     gccgtgtatt actgtgcgag agacacagtg aggggaggtg agtgtgagcc cagacacaaa    867960
     cctccctgca gggaggcgga ggggaccggc gcaggtgctg ctcagagcca gcagggggcg    868020
     cgcggggccc acagagcaag aggccgggtc tggagcaggt gcagggaggg cggggcttcc    868080
     tcatcagctc agtgctctcc ctcctcgcca ggacctcagc tgtccccagg cctcctcttt    868140
     ctttattatc tgtggttctg cttcctcaca tcctcgctgc aggcaagaaa gaagggagaa    868200
     atttctctgt ttaccattaa ggttttaaca gtcactggca cctttttttc aacaaattcc    868260
     cacaaggccc attttacctt cttgaaaaaa tatttcaagg cttcccacca tctcttgatt    868320
     ggcgtcatcg gctgcatcgt gtcctgctct taaattcatg tgatgaaacc ccaatcccag    868380
     tggctctgta tttggagaga gggattttaa gaaggcaaat aagttaactg tggtcataaa    868440
     tgtgggaccc taatccagta ggactcttgt ccttatggga agtggaagac atcagaggcc    868500
     tctctctcca catgcacaca gaggagaggc catgtgggga cgcgctgcat gagaaggtgg    868560
     ccctttgcaa gcctggaaga gggctctcaa aaaactaacc ctttctgcac ccagatcttg    868620
     gacgtcaaga ctccagaaca ataagaaaat taatatttgt tatttgaaac tacctagtct    868680
     gtggtatctt ctgatggaaa gccaagcaga ttcacagata caattgtgtt agctccgtct    868740
     cctggaggga gaagcagccc accgaggctg acacatctct cagattattt ttataaagaa    868800
     aaatagatcc aagatgaaaa ccccaccaca tttctgctga gtcattcact tagcaggctt    868860
     ctctgagatc agccctgggg ggctgtgttc taggtcatgt ctctcacttt ccactatgag    868920
     aatccatgta gatcatgaga accagccaga tggagtcctg atcccggccc accttctgct    868980
     gctccccaag tatcccaaag aaaaccactc cagcgtcacc cctgtgtctt ctactctcta    869040
     aaatcccact aaagctgacc ttaaaccagg agatatttgg ggccgggctc ttgttctctt    869100
     cttcttagtc ttccataagg tccaggtgcc gagtaaagcc tcagtaaaga ttgagcttca    869160
     gttcccctaa cccacatcat tcccttacat ccttgtcatg catctgtcct ctgtgggcac    869220
     tcagggcagg ttacacagga attgcctgaa gctggcctca actgatgtgc tgagaccacg    869280
     ggtcatgcac atgtatgatt ccaggtcatg caggctctac tgcaggacag acctgtgtcc    869340
     tgtggggcac agacacctgc tgaccccggg tctcctctaa taactcacac acctggtgtt    869400
     tgtgtgtgtc cagatggccc catgagcaca gcacactagg gtggccaacc tcacaggtgg    869460
     ggccgtcaga tggaatggac aggctggtca cccccatcat cagtgatttc ccagtgaatt    869520
     ttaaataaat taaacaaaat taacaacaaa ggggaaggat gtgtacctgt ctctgggagg    869580
     tatacagggt cccagaactg tgctgggagt gggtgtgaaa tgcccttttg tgtagaaagc    869640
     cctccagcat gagtgaaagc tgaaggaatg atcactgtgc aggtggagga tgataggcag    869700
     ctggcagggt acatttcctg aagctgttcc tgggtgcatg atggttggat ggcccatggt    869760
     gatgagggct ggactcacat aggaacaagg aaattatccc atgggaatgg cagaaaagaa    869820
     aacggagatg gacacccctg gagacagctt attagatttg gtggaggaaa aatagaaatt    869880
     tattccaatg accccagcat tttcagctaa atatgagttg cattaataga cagaattatg    869940
     aaaggaaggg agaaggagtt tatggaggga aaaaaacaga agataaatta aaattaaaaa    870000
     ctatgcaaat ctatcaattg gcagataaca gagtggagct gatgcagcta cttccttgtt    870060
     ctggaaacct gccagagaca cttagcattc ctgtaaggcc ggtctagtgg tgatgaactt    870120
     gtccactttt tacttttttg ggaaacttct caaacctcct gcattattga agaaaagctt    870180
     taatccatgt aacatattta tttggaagtt tcttggcttt gtttttattt tcagcaactt    870240
     gaatagaatc gtgcacatca tgatatttcg gtgaacaaca ggccacctac aggacaaagg    870300
     tctcattaga ttataatact gtattttcac cgcatttttc catgcttaga tatgttcatc    870360
     tgtataaaca tctaccatgg tgattcagct gcccacagta ttcactacac ttacatgaca    870420
     tacagatttg ttacctagga gcactatgct atcccacgca gcctgggtgt agcaaggtat    870480
     ttcatgtagg tttgtgtaaa cacactctat gatccttgca caatgacaaa ttgcctaagg    870540
     acacattgct cagatgtatt cctactgttt ttcatttttt tttagatgga gtctcactct    870600
     gtcacccagg ctggagtgca gtggcacaat cttggctcac tgcaacctcc gcctcccagg    870660
     ctcaagcgat tctcctgcct cagccttccg agtagctggg attacaggca tgaaccacca    870720
     cgcccagcta attctttttt tctatttttt agtagagatg ggatttcacc atgttgccct    870780
     ggctggtctt gaactcgtga cctacatgat ccacccactt tgccctccca aagtgctggg    870840
     attacaagcg tgagccactg cgcctggcca gtgtatccct actgttaagc cacaaatcat    870900
     cgtatctcat ctctctccct tttttgtccc tgttaatatt ttcatgttgt gcgtgcattc    870960
     ttctcctagc tcagtgaaca tatttataag gtttactttg aattttctgc taggtaatca    871020
     atgtatctcc ttttaatttg tgtcagtttc tagtgctttt tttgttaaat aatttggacc    871080
     acttttttct gttttgtgat tattgttatt attattattt tttactttct ctgttgcttt    871140
     ctcctcatta gaagaaaaag ctaccgtaat cactcttcac agaatatgcc cagggaaaat    871200
     gccatcacct atcaacccag gctgtcattg tggcatctct taaaacttca taatggttga    871260
     aattttttgt tgttgttgtt attagtggca ctcagaccta cagaggattt taagttttgc    871320
     tcctactcta aattaggtgg cattgaaatt catcctgcag gcagtctctg ataaagtgga    871380
     ggaattgggc agttttgtta tgcatatggt tcataaaaat aatatgagtt taaggttttc    871440
     tcagccaagt gctatatcct gatattttat aatcacttct ttgaatctct cttgggttta    871500
     tcttttgaaa atttagatca gaatataaac caggtttgag gctttttgtt tggtgtaaac    871560
     atctcctata ggtcattgcg tgaaaaggca cagagtctat ctgaggagga gaactcaggg    871620
     aagcctcaca acaacagggg agacaaaacc gaggtcacta aaaaaatatg aagcttctgg    871680
     ctcctgcagc ctccagacat gccccacacc ctctcattgc acacaaatga attcctttct    871740
     ctttcctgag tgatctttgc caagattcct ctcctccact ttgacacttt ggtatatcca    871800
     tgtgtgtcca ttctggttca gccagggaca cagaaccagg aagacatcat ggcttttcta    871860
     gagggagctg gcttggggaa tcaatggttg gagtagaaag tgtctttgtc catgtgtcac    871920
     agccctgagg gacacgggaa gaaggtcagg aagacaggat gaccagacgg ctggagcaac    871980
     cagtatgaac aaagccactt tctttcctgg tgaagagggg catttggaag cctggtgtcc    872040
     ctgtagggga tgaaccacac tcacctgccc actgttactt gccaacgagg tgacccgcag    872100
     agcagcagat cctgagtcac cgacagtggc tgcttcctcc tccaaaccct ccaagatctg    872160
     tccttccagg agtgtgaacc tcagcagatc cgcacgtttt caatgggtcc acttagctag    872220
     tgtctgtcct cagccccatc cctgggatcc tgtcctggtg ctgtgtacac tgtcacaata    872280
     gtggtgacag tgacagctgg tgcctgcact ttccaggcca agaagtttga aagaagcaag    872340
     tcacagggcc agcctggatt tggggcaaac acacaaattt cactctgtgg ggaagtgctg    872400
     ggaagcagcc ttgagtcctc ctcagctcac gtgtggctgc tgcttctcat cccaggtggg    872460
     acacatgagc cctccccatg cccaggatgg agctgtttca gtcacagcag gatgggaatg    872520
     tgtcttctct atgggggctt ttgaagcaca gctctcctgt tcctgagatt tgagatttat    872580
     aaagacaagt cccacagagt gaacctgcac aaataagaca gtacaggatc cccaggagag    872640
     acatcccata tgagaaggga caggcaggtc ccatcactga cgctggtcct cagaaaccct    872700
     gaccagctac tgaccttccc aggtcccctg ttcctggaac agttcgtgtg tctgctcctg    872760
     aaaagatgcc catcaagagt ccccagggtt ctgccccctt ccttgatgtg cctctgggag    872820
     tggggctggc tgggctgtgg gtctggcgtg accctctcca caggtcagcg gctcagtgca    872880
     ggctgcaccg actcggtggg gctgggctgt ggctgaccct cccaggggga gcctggactc    872940
     ttcttagggg tatctgcagg atcccaggtt ctgggagaac agggaagcca cagtgtgtgc    873000
     agggccccat ctccagtaaa gtgtttgctg tcgctctgtt gacaagggca tatccccagg    873060
     cctaggggca ctgtgggcgc acgaccaatg cgcgagacac agggagtaag gcacagttcc    873120
     ctcccattac tgtgaggaca ctagacacag tgtgtgtgtg tgtgtgtgtg tgtgtgtgtg    873180
     tccattactg tggggactct aaccaccgtg tgtgtgtgtg tgtgtgtgtg tgtgtgtgtg    873240
     tgtgtattag gagttttgca gtggcttctg agacccatga ttttgtaggt ttagaaatta    873300
     tctgatgaga tgttcatcca caggaaccga caagagatta ggtgttctcc caaagcccct    873360
     ggaagctcct ggactcactg tgagtgtgga ctgatccagt gcttccggag cgccacagaa    873420
     ggggctccct ggtggtttca tagaatcctt gcttggggtg tttctgcaga gttcactggc    873480
     tttttcctac gaccaattta ctattgcaag cgatggcgtc agcagcacat ggtgtcacgt    873540
     gtcactaaag gagcattctg agccaggaca cagccacttc atactggggg gaaaatgctc    873600
     tgggagccca gacaggagcc tctctgcagt gcaagggctg ggctgcaggg ggcgctcaag    873660
     gcccacccag cacaggctcc agccccagag caggtgcaca ggaggctgcg gatggagtcc    873720
     ccttaagatc tgtgtcattc ttctaaaaaa atctaaaata agtatttgac aaaaactgct    873780
     gaagagtcca taaatatcct attcaattgc aagaatttat caatttacac tggagatttc    873840
     taaccctgct acatatctta atagtaagca tctggaggtc aattaagctt ttattttata    873900
     taaataagtg caacttttgg agaaacacac tcatccccca aataacacat tcatgtatta    873960
     aagcctagaa atactttaaa ttacctctga gctattcaag tgtgggttcc cagtgaagtc    874020
     ctgttctagg gaaaattgtt ctccagtggg agaagctctg tcaacataca gcttagggat    874080
     atggcagggc acacatggcc tctaagggga ttatagcttg aacccttagc atcctcctgt    874140
     tgggtaatcc atgtgtcatc tctccactct ttctcatgct gtgttaggta tgaaatagca    874200
     tcgctcatga atatgcaaat aactgagatg actatagata tctttgtgcc ctgagagcat    874260
     cacccaacaa ccacatccct cctcagaagc ccccagagca caacgcctca ccatggactg    874320
     gacctggagg atcctctttt tggtggcagc agccacaggt aaggggctgc caaatcccag    874380
     tgaggaggaa gggatcgagg ccagtcaagg ggaattccat ccactcctgt gtcttctcta    874440
     caggtgccca ctcccaggtc cagcttgtgc agtctggggc tgaggtgaag aagcctgggg    874500
     cctcagtgaa ggtttcctgc aaggcttctg gatacacctt cactagctat gctatgcatt    874560
     gggtgcgcca ggcccccgga caaaggcttg agtggatggg atggatcaac gctggcaatg    874620
     gtaacacaaa atattcacag aagttccagg gcagagtcac cattaccagg gacacatccg    874680
     cgagcacagc ctacatggag ctgagcagcc tgagatctga agacacggct gtgtattact    874740
     gtgcgagaga cacagtgtga aaacccacat cctgagagtg tcagaaaccc caggggggaa    874800
     gcagctgtgc tggcatggag gaaatgacaa agattattag attgaagact ttctcagaaa    874860
     atgatattaa gtcattaagg aaaaggaaca atataaacat gtatttgaga aattttaatt    874920
     atttgagaga tttttcatac aatatttatt ctgcaagcaa atttcaggga ttgaattaat    874980
     aaaactgata cagaacttcc tctgtaggta tctgtgtaaa catcaatttc tgaatcagtg    875040
     ttgtaaatat tttggaacac acacacaaat cacattttat ctctactttt atctctatta    875100
     ttaaaaatgc caaaaaaact cattttgtgc atgtagcatt ttgaattccc accatcaatg    875160
     catgatagtt cttggttttc cacattcata ttgccattta tcattatgag aattgtgtgt    875220
     tttaaccatt ctaataggtg agtaatggta tctaattttt agttaaatgc acatttccgt    875280
     aataaaaatt cacatttaac aattttcata taatttttgc cgagatgcct cttctcatat    875340
     ttggttcatt tttaactgca ttgttttctt ttgattagtt gtaagtttac ttgcatattg    875400
     attataaaat catttaacaa attaaaagaa ttcatttaac aaatatgtga cttggaagta    875460
     ttttctccaa gtctgcggct gtcttttact cccttatcag tatgtattgc agaaaagtgt    875520
     gtgtgtgtgt gtgtgtgtgt ttatacaaat ttagatttta aaaatgtaaa atgttattca    875580
     tccacagatc atgtctttgg tattatatct gaaatctcat tataaaatac agtaatatca    875640
     attacttttt ccatgtctct aatctcaggc tacaatcatc tcatgagtgt ttaagcttca    875700
     cctacttgat tggaggacta tcaacctaac atatttggaa tacttctgta aaaagatgtg    875760
     ttcctcttcc tattatttct ttatttgatc acttattaat atgtgtattg gtttatggat    875820
     gtctatttca tactctgaag aagatccatg ctacattatt cattttattt ttcaaaccac    875880
     catggcttta ttatgtgctg ggagctcatt gagtttggat cctgcatcct tacagctcac    875940
     ctcatgcttt tgtttttgaa cacttccctg tttcctgcta ttataataaa ttctaaactc    876000
     attttctata ttatcttttt catacataga atcagccatt tttctaaaga ttgcttgctt    876060
     ctgatgttaa agaatagtat ttaaaaaatt gtaatactgg gtatgtgcat tgttaatgtg    876120
     gtataagtac ttgcaggacc tctcaaccaa ctggcctagt aaactatgta tctaaccttc    876180
     tgtaatgtga ttacattaaa aatgagaaca cactggtctc tctacccaat tatgctacca    876240
     catggatctt tctagccttc cttccttgac tgtctataac ctctcactgc aaaatgagga    876300
     accccatcca accatatgcc attttattac ttagctgcac aatttcagga cacatgcata    876360
     gcagtatcag aaatgtaaag ctgtaccctt gtaggaaaca tgtttatcta ctagaataga    876420
     gtgcttatgt tcagtttctt tacactttaa acttacagag tttcctcatt ttcaaagttc    876480
     cttaggtcag caacttcatt ttccactttc ttcagtgaag tcatttcaat gacactgtat    876540
     aatttgattt atttgaaatt ctataaaagc caaaactgta gtcaagtaaa caacaacata    876600
     tagaggatat tcgaggagtt tagagactgg gtataaaata agttaaaaag acactgttta    876660
     agaagattaa aattattttt agtgatatgc aatggttcag atatgacaca attaatttgt    876720
     ctaagcacat agttttgtga tggaaaatat aaacctaaat atatacaatt aaaaaaaatt    876780
     tagcagttca ttaacccaag gatcaaatgc agattgtata aaattatctc attacttatt    876840
     ttgtgagggt ggagatttca tgagatgtat gcaacaaaga atgaggtaat tttcctgatt    876900
     tgcatataag atgttgccat tcactaaaga cctttaattt tttaaatttt tttttaaatc    876960
     aattttctat gtgacccagg ttttttcctc ttgacaagca aataaccgac aggattattt    877020
     tctttccttg gttgagaaat atttccccaa acttcagctc agttcaggca tacactgtcc    877080
     ctgaatgggc atttaccctc agatgggtac acacatctgt caacatgtgg actcttctgt    877140
     cagacaaacg cacctttact cacgtggatt cttctctcag acaaacacac atgtccccac    877200
     gtggactctt tcctcagact accacatatg ttcttacatt tactctttcc tcagaaaaca    877260
     gacatttcct catgtggact cttgtctcag acaagcaaac atgtctccat gtgaactctt    877320
     cactcacata agtacacata tgtccacatt tactgtttcc ttacacaagt acatatatcc    877380
     aatgtcgaat tgttctgtgg caaaatgatc tcaagataat gataatcata aaccccctcc    877440
     ctgacaaggc gtagatctgt atttttttca ttgtaaccta actttgcctt attgtcaaga    877500
     acagtagttt gcagctctaa atataccaat tagagacagg tgtccatttt ctctggaaac    877560
     gtatttttat gttcttactg gacatatttg ttgataatgt ttgctattat gaagataccc    877620
     caacagtgtc cacattagag aataaaaaag agtaatgggc agattaactc tgtgcatcca    877680
     gacccagaaa tcctttgacc ttgacttccc tgaaatgtag acacagagga tggatgagca    877740
     atgctgagca gtgcacccat gaccacaaaa agaaagacgt ggaaatgtgt cccctccact    877800
     tctcatgaaa ggcagctcat cccctgttcc ctcaggccct ggcgaggagc caccccatgt    877860
     ctgtgccctt cctcagtgtc cacaccgtgg ggtctgcatt gatctggatt cccttctcat    877920
     ccccgtcaat attagtgtcc ttcgtaaatc aggtccagct gtggcttctc ctcacggggc    877980
     tgttctcagt ctgttttctg tgttcacgga agtcctgtgt gaagtttact gatggagtca    878040
     gagggggaaa aattttacag cccagtggtg agactctcct gcaaagcctc tggtttcacc    878100
     tttactggtt acagcatgag cttggtccag catgcttcac aacagggata ggtgtgggtg    878160
     ccaacagtga gtgatcaagt atgaattctc agggttactc tccatgagta caaataaatt    878220
     aacaatctca agcaacaccc ttttaagtgc agtctgcctt acaatgacca atctgaaagc    878280
     caaggacaag gtcatgtatt actgtgagtg acacagtgag ggaaaccctg tgtgagccca    878340
     gacacaaagc tcaccgcagg gagacaggag gggactatgt ggtagatgct gctcagaacc    878400
     accagggggc actcagaacc atcagggagg gtgcacagaa ccaccaggag gggctcagga    878460
     caccaggggg cgctcagaac caccaggggg cactcaggac catcagggag ggtgcacaga    878520
     accaccagga ggggctcagg acaccagggg gcgctcagaa ccaccagggg gcactcagga    878580
     ccatcaggga gggtgcacag aaccaccagg aggggctcag gacaccaggg ggcgctcaga    878640
     accaccaggg ggcactcagg accatcaggg agggtgcaca gaaccaccag gaggggctca    878700
     ggacaccagg gggcgctcag aaccaccagg gggcactcag gaccatcagg gagggtgcac    878760
     agaaccacca ggaggggctc aggacaccag ggggcgctca gaaccaccag ggggcactca    878820
     ggaccatcag ggagggtgca cagaaccacc aggaggggct caggacacca gggggtgctc    878880
     agaacactag gaggtgctat gaatcactag ggggcgctca ggactcaagg gagcactcag    878940
     aaccaccagg gatagctcag gacaccaggg ggcactcaga accgccaggg ggcactcagg    879000
     accatcaggg agggtgcaca gaaccaccag gaggggctca ggacaccagg gggcgctcag    879060
     aaccaccagg gggcactcag gaccatcagg gagggtgcac agaaccacca ggaggggctc    879120
     aggacaccag ggggtgctca gaacactagg aggtgctatg aatcactagg gggcgctcag    879180
     gactcaaggg agcactcaga accaccaggg atagctcagg acaccagggg gcactcagga    879240
     ccatcaggga gggtgcacag aaccaccagg aggggctcag gacaccaggg ggcgctcaga    879300
     accaccaggg ggcactcagg accatcaggg agggtgcaca gaaccaccag gaggcactca    879360
     ggacaccagg gggtgctcag aaccaccagg aggtgctcag gacaccaggg ggcgctcaga    879420
     acactaggag gtgctatgaa tcactagggg gcgctcagga cacaagggag cactcagaac    879480
     caccagggat agctcaggat accagggggc actcggaacc gccagggggc gctcaggaca    879540
     ctagggggcg ctcagaacca ccagggggtg ctcaggacac caggaggcac tcagaaccgc    879600
     cagggggcgc tcaggacact agggggcgct cagaaccgcc agggggcgct cagaagaagc    879660
     agggggtgct cagaacacca gagggtgctc agaagcacca gggggcgctc aggacaccaa    879720
     ggggcactca tgagactgtg gcaagggggt gctgagaacc acaggatgtg accaagacac    879780
     cagggggcac tcagaactgc cagggggtgc tcaggacacc agaggattct cagaaccacc    879840
     aggggatgct caggaaacta gcgggtgctc agaaccaccg gaggacactc agaaaaccag    879900
     gggatgctca ggaaccacca gggggcgctc acgacaccag cgggcagtca gaaccaccag    879960
     ggcatgctca gaaccaccag ggggcgctca ggacaccagg ggatgctcag gacactaggg    880020
     gcgctcagga accaccaggg ggcgctcacg acaccagtgg gcagtcagaa ccaccagggc    880080
     atgctcagga ccaccagggg gcgctcagga caccagggga tgctcaggac accaggggtc    880140
     gctcaggaac caccagggga tgctcaggac actagggggc gctcaggaac caccaggggt    880200
     cacccaggac accaggggtc gctcaggaaa ccagagggtg cccaggaaac caggggaggt    880260
     tcaggaacca ccagggggca ctgaggacac caaggggtgc tcagaaccac cagggggcgc    880320
     tcaggacact agtaggcact gaggaaccac cagggggggc tcaggacacc agaggtcgct    880380
     cagaaaacca gggggtgctc agaaccacca gggggcactc aggaaccacc agtgggtgtt    880440
     caggacagca agaatggctc aggacaccag ggagcactca ggacctccaa ggggctcttt    880500
     ggaggcagct ccatatcagg tacctgggga ggatgaggtt tccttttcca ccttggtgat    880560
     tcctgacctg gtcaagcaaa agtcttcccc aggatctctt acgatgtctt ccttgtaact    880620
     catggtttct ttcacctata aaacattaac ttagaacagg ggttcaattc aacttttaac    880680
     tctgcctatt ttcagagtta tactagcaat gatatatctc agtatatttt ttttaattgt    880740
     gtatattcaa tccaaagtct ggctctatgc acaatttttt tgttttctgt gctgtcagac    880800
     acactattgt aaatgctttt ctaacaactc agcatatgca tggggtccag tttcttttcc    880860
     tttcatcggc tgtttgtgca gatgaaacac cactttaagg gctcctgtcc tccactttgg    880920
     cccctggtgt tctgcttctc aaactttctc catcttctct ttttctgtca aaatatttta    880980
     tcttcctcag tctccatgca ggaaacagga agtcctttta cttcctgtcc tccatgtctg    881040
     gtaaatcagt tcacttcttt tcatgatcac tgaagccaac caagtttagg agagtaacag    881100
     ttctccttag aatacactct acctgcagac cctctgccct catcacactt ttctagggtc    881160
     ctgcagacat aacccccacc cattcctctt tttccctaag taccacagac taggctctgc    881220
     aacttatgct accctctgtg tgctcagccc aggggctcag tagtgctttc atgaagtcca    881280
     aatccctaat gtgtttgccc agtctcagac caccctccag caagctgcca ttgtgattga    881340
     atcctgcaaa gcatgggctg ctttcagttt cctattgctg gatgttcttt attataaagg    881400
     catattggca aataacgact agagtttgta ttgaaaatta acgccaaaaa gttttttaaa    881460
     aaatttttca aatagaaaag ttctatcctg cctagtttaa aaaaatacaa tgttacttta    881520
     atcaatgatt taataaaaat ttaagtgatg tttgtcttat tagttattca atttattaat    881580
     aactgactga tatttaaaaa gtaaatactg gctgggcgca gtggctcacg cctgtaatct    881640
     cagcactttg ggagggtgag gtgggtggat cacctgaggt cgggaattcg agaccagcct    881700
     gaccaacatg gagaaacccc ctctctacta aaaatacaaa attagctggg cgtggcgggg    881760
     aagctgaggc aggagaatcg cttgaacctg ggaggcggag gttgcggtga gccgagaaca    881820
     cgccattgca ctccagcctg ggcgacaaga ccaaaactct gtctcaaaaa aaaaaaaaag    881880
     taaataccat tgtacactta agtaatatat ttggcaagaa tggcatttac attcattcaa    881940
     aaatgaaact gcaaatacga gttacattca attaaataat taaaataata tagaaaaaaa    882000
     tgggtgtgtt gttttggtgt ttaatataca ttcatttttg catggacggg tatatgtgtc    882060
     attgctgggc tgttgtgtat gtgtgcgtgt gtgtgtgtgt ctgtgtgtac aactatgaag    882120
     tttaaaatat attattaaat tacgtagtta tattaatcca aatttatcat gttaaaatat    882180
     taggaaaaaa acaccagtag agaaattaca gagaacatca gcaatgccta cagcatttac    882240
     aagagtcaca ttaataacaa acaaactagt tcaaatgttt agatatgaca catgcagtag    882300
     aaaacgttca catggtatta acacaaaaat ggtgcacaac tgaggaaatt ataatacgtt    882360
     catgatattg gctacataaa tgcttatgat agtaatgctt ttcatccatc aaatgcttat    882420
     gataatgctt ttcatccatc atattataga tgataaaaca actctataaa cacttccatc    882480
     actagcgttt aatatgagat gcctcacatc tttttctgaa ataaataaac atctgtccac    882540
     cacttcgatg atcatttcag gattatcctc tgaaataatt atccataata attttagtaa    882600
     caattttatt attttcagaa gcctatttta taaggtcttt gaactattat ttttatgatt    882660
     gttactttat attttacaca ctttttattt ggaataatta taggttatca gaacaattgt    882720
     aaggaaaata cagtgtgttc acatccatct ccaagttttc actaaagtta atatgtcaaa    882780
     aaaaacatgg gacatgggac taatatattt acattgataa gtttctgttt attcagctct    882840
     gggatttatt tgaattttgc caatttttaa cagtttcctt ttttcctttt tcttttcttt    882900
     ttgagataag gtctcacttt cctattgctt tttgtttgtt tctttgttca acccaggtaa    882960
     ccacatcaaa ttcagtcacc atgttcctct catatcttct ggttaatcac agtttgggtt    883020
     cctgctgtct tcccattgaa tattctataa atgaaactag tcaaataagt tgattctggt    883080
     cacttatata tttacctatt ttatcacgtt tgttttgtca atcacagtaa gtgtcgaatt    883140
     cgctatctgt tatagatgtt agcctatttt ctatcccaga tccattggtt aaatctttgg    883200
     tgatgccttt tagaaaactg atccctttac cctatgtaat atgccccttg attcctgaaa    883260
     gtcttatgtc taccttgtct gaatttaaca tagctaagca cgctttcttt tcattcatat    883320
     tttcatagtc catgttttcc tgtatttaac ttttctatgt agagcaaatt tctgtacaga    883380
     gctagtagtt gggtcttgct ttttaaatca actataataa attctatttt aaaactggta    883440
     ttactatttt tctgttaatt tctattttaa tttggcattt tatgatcatg tttatttctc    883500
     tattaactta ttgtttagct catcttttat gaatattgta ttggccctaa gatatacaat    883560
     aagaattgtg tataatcaga ttctaattca aataacgtaa aacctcttca taggttgtag    883620
     agctattata acttattctt ctaaaccctc tttctcatcc gttgtcttag tttattctca    883680
     gtttgcactt atatgtgcta taaaatataa tatgtgcatt tttatcatta catagacata    883740
     tattagaaca attaaaaata taaaaactac atttcaactt cattttttca ttcttgacca    883800
     cattttttat ttggatagat tcatgtttcg gatgtatatc atatggctac tcaccctggc    883860
     ggaaaatttg ccaaagcacc tactgaagga tgaatgcact agcaataaat tttctcagaa    883920
     tcgatttgtc ttacagggta ttcatttgac tttcgcttta aatgaaattt ttaatatata    883980
     tagaattcca gtttgacttt aatttgtaat ttattttctt gtactcatgt attcattatt    884040
     ttcttcctga agatggtaac acattccatt ctgctgggcc ttcattatag atatttgtgt    884100
     gtatctattc agggctatat ttgcaattta tggatgccac aattatcaga gttgaagtca    884160
     gcttctgctg tccacagaga tttcaagttc ctcccatgat acttgctttt gtgtccctgt    884220
     ttgatcctgg gtctttatat ttagttttcc ccagggaggc tgtctgtttc agctgtggaa    884280
     agtgcaccct actgacagtt taaattgatg actgtgtggt gaaggaggtt ggacaaagcg    884340
     ggacttcctc caaccttctg actgagtctc cttcttatgc aggagtagta agcatagttc    884400
     tggggagtgg ccttccacat tgtcctgtcc ttaactcttt ccccagggct ggaacgtctt    884460
     ttcccagaca caactgtttt tcaccagtgt ccccagcttt ttacccacta tccttaccct    884520
     aaagagtaag gatttctttc ctgaggaaag agataggagg tgtttctgga tcaagtttcc    884580
     ttggtgtcgt ctgtttcctt ttgtttctgt tgacttcacc acagctcata tgacacatgc    884640
     tttggtggat ttcccctgga ggtagtggag gtgcattcag gcattccaca ggagctgctg    884700
     ttcttttccc cagtcaacac cacaagacac cagatgagga agttgtccgt ggatttttca    884760
     agttctctag gaaaagcttg caagcactag gccaatctaa caccattagt acatgcatac    884820
     taaaaaaaaa aaagtcatta agtatttcta ggttagtctg tttctatctc aaatgccatc    884880
     cagtggcacc tgccctatgt acactagcag gtaggtcctg gttctctctg caggctccta    884940
     tcttctcaga tttcagtttt cttgtttgct tggtgaaatc aactcagata tgttgaatgt    885000
     tttttctctc ttttatttgt agctgttcag cttcgttgtt aatgaggtca gaataaaatc    885060
     acagttttct catttttttc acattcccac actgaatagc tgctttccgt ataaaagcca    885120
     gaaactgaga gaacacattg aatatccatt acaggtgaat gttaaacaat ttgagatatg    885180
     tttgtgtact ggaataaaat gctgcattac aatcaagtca tcactcattc acataaaaca    885240
     tggccacatt ctcaaataat gtagggacct gagtgcccct ccatctactg gcctctcctg    885300
     gggccctagc ctggccacac cttcttgcag ggcagtcttg gacgccctgg gatcccgcac    885360
     caaaatttct gccctggcag aacatgcctg actggtggag agctccaatc gggcagccct    885420
     catgtgcaca caccagctta cacacttcct ccgaatactg taggttcacc caggcccacg    885480
     taacttccca catcactttg cagtcacatg tctgtatagg tgggttttgc ttttcttgtc    885540
     ccaccattgc gtggagtgca gtcccctccc cccaccccaa ccaccatggc agaggaagct    885600
     ttggtgggga aaaagccagg gccgctcctg tcagcgtccc gcacttgcgc taattctgca    885660
     cagagaatag cagatcatct cacacattca gaaatcactc ctgcttgtgg ggcatgaata    885720
     cggcacccgg gcctgtgccc acaagtgtcc catccctgag ccaacacctc ctccagtgtg    885780
     accttgaaca cagtcaccaa cagggcccca cacacccaca gacgcaatgc ctctgccact    885840
     gtggcgaaca cctgcaggga ggcaggcacc cagacaccca ctagcactct gccacagctg    885900
     ccacacctcc aacagcccag gacagtggat tcctaacctt aaggagccgg agaaccaagt    885960
     cagggactag tataacttcc cccagagtca gagcacacag tctaggtgtt gggagctgag    886020
     cactggccac ctaaattttt ccagaaatga agccagttgg ctgaatccac cttataccac    886080
     aatcaaaccc tcaaggtcat ccaatagggt aaaagaaaat aaaaatgtat ccaaaggtca    886140
     gcaacttcaa agattgaagg tggataagcc cacaaagatg agaaagaacc agtgcaaaag    886200
     tcctgaaaac aaaaagggcg ccctctttcc tccaaacaac cacagcacct cttcaacagc    886260
     agttctgaat ggggctgaga tggctgaaat gacagaaaca gaactcagaa tatggagagt    886320
     gaaaatgtag atgaatacag ctatttatgg agaatactat aaatgttcct caaaaaataa    886380
     agaaacaaaa tctactgtag aatccagcag tctcactgct ggctatgtat ccaaaggaaa    886440
     tgaaatcaac atgtcaaaga gatatctgca ctccatgttc acgttcattg cagcattatt    886500
     taaaatagta aagatatgga aacatcctaa attcccatga atggatgaat gaataaagaa    886560
     aatgcataca gacacaacag agtaatgttc atccttaaat aagaaggaaa ccctgcctct    886620
     gtgacagcat gcatgaatct agaggacctt atgccaagtg aaacaagcca ggaacagagg    886680
     aagagtcatt catgatttca ctgtatatat taaagcagta gacttgcaga ggtagagtag    886740
     aatgttggtt accagggcct agaggggtgg actgggaaag ggagatgtgg gttaaagtgc    886800
     acaacgttcc agttagacag gaggtataag ttatgccttt ctaatgcaca gcatgtcaac    886860
     tatagctgat aaggtagtat atatttcaaa attactaaaa aaataaacat tagaatttcc    886920
     ccactaagaa atgataaatt tgtgaggtga tgaatataag cggcttgagt tacccagttc    886980
     ataatgtata catgtatcat aactaaacaa catatgtcat aaatatatgc aaaaattatt    887040
     tgtaatttat aataaaataa gtttcatatt taaataatta cattaagaaa atgaacagaa    887100
     actttcagat ttcaagaata ttttatatat atatatatat atcttaaaac aaacttgcaa    887160
     cagaatatag aaataagttt tacgactcaa tggaaaagaa cagaattcaa taaaaactgg    887220
     ctaaaagaaa caacagctgc atcattatag aaaattctgg aataatcagc catataaaga    887280
     ttctcactct cttagaacta gaattccgta ggacttgtaa ttcctcctga cctgggtggg    887340
     aggcaaaagg aagaacagct aatggtgatt cagtgagttt tatacctgtg tgtacttctg    887400
     ggctcactca gcagaaagaa aagaagaaaa gaaagaaaga gagaaagaaa gaaacagaaa    887460
     gaaagaaaga aagaaagaaa gaaagaaaga aagaaagaaa gaaagaaaga gaaagaaaga    887520
     agaaggaaga aggaaggaaa gaaaagaaag aaagaaagag agagagggag agagggaagg    887580
     aagggacagc agaagtcatt gtggtgtgtg tgaaaacaca atccttgggc tcccccacat    887640
     ccatctctac tccagtccca tcaatgtcca gcaaatacat tttctaagat gaagtatttt    887700
     aaactttcta aatcctgcta gaaaacccct cagctctttc agttttgctc tatcacttga    887760
     attattgaat taaatctagt ttttgtgggc ctatcaatac cataagccaa aataacacat    887820
     gaagaaattg cactgagaca catgaaaacc ttctgaaagc tccataattt cagatctgca    887880
     ttcttatttc cccgaaccta aatcactgaa tagagactca gaacgagttg atcttgttcc    887940
     tgaacgtgca cagagccaag gacatcctgt ctgtctggaa cagctcaggt ttgttcctgt    888000
     ttctcctaga ggatataaaa tcttgagtta gggaaaaaca gccagggaca ccctgggctt    888060
     tgttcttctc tcccctggag gcaggatgtc cttcagagct ttgtcccagt gggtaacaca    888120
     gctgctgagg tgtacaaccc acgtggcctc gttttggtca cttttgcatg gtgagcctgc    888180
     tttgcaccat ggcctacaat atgcgtgtgt aactaatctg tctccatctt caaaatgaca    888240
     ttattccaca tcaaatctag tgcaggtgcc tcacacagaa cattctcaat tacctccatc    888300
     attcataaaa ttgatgccat taatttcaag tatacataca tcagactcat ttaacgtatt    888360
     gttattctca ttgtttgaaa cataactttt agatcaaata attaacaata ataaaaatat    888420
     aaattttgaa gtcaggtaat gtgatttctc tagttgtgtt ctctttgctc agaatggctt    888480
     gggctgttct gcatcttttg tttttccaca tatattttag gattttttaa aaatttctgt    888540
     gaagaatatc attgttgttt tcatagggat tgtactgagt ctgtagattg ctttaagtat    888600
     tatggacatt ttaacaatat tgaatctttg aattcataaa catggaatat tgttccatct    888660
     tgtgtcctct ttatttcctc aatgttttat agatttattg tagttttttt ttactttgtt    888720
     cattacacat tgtatgcctg taccaaaaca tcacatatac ccaacaaata gaaatatata    888780
     tactattatg tgcttataac aattaaaaat tatgtatgta tattgaatct attcaaaatc    888840
     agaaactatt tctttttact gtttgtaagg tcttgtctct aggctataaa aagaatttgt    888900
     aaaactcaac agaaagcata atataaacag aattctaaaa tgagtgaaaa tctgaacaaa    888960
     cacctcacca agaaaaaatg ttatctgaaa ataagaatat ataaaattgt tcagtatcaa    889020
     ttgtcataaa ctgatactca tatttaccaa atacaaaagt aaacatgatg tatttcaaca    889080
     gaattgattc tcaaatattt gtatactcat acagtggaat actatcagtc ataaaaacta    889140
     tgggttatta attcagaaga caacatttta acattttttc taagtgaagg aagatggaca    889200
     aaagagacta agtattgtac aattccattc atgagacctg ctaaatacag taaaattaaa    889260
     aggattttaa aaacaggttt gtgataggca gggcttttgg ggaaagacaa gagactgact    889320
     tggcaaagct caggggatat ttttagggta aaacaaactg tgtgccattg tgatatgcct    889380
     aatttcttat tatatttgtt cagagttaat agtgtacgtt tcaaccaact cggtgatttt    889440
     atattttcta tttgctgata gagacatgtt catttttgtc aatcactttg ctaaatgtgg    889500
     ctgagaggct gttgaaatga acgccgagca aatgtattca ccaaatctac aagagcaaat    889560
     tatttgccaa ttgctgattg agtggggtct ataatgtatt tgagattggg tgtggggatg    889620
     ttattgtgtg agatcatgat gtttagacca tgacactctc tggtgaggga tcactcattc    889680
     attgcacatt taatgaaagg caggtaggag gagcagaagg ggatgagtca cactcctgac    889740
     cacagccaca ggttattgaa ggcagaactg atgtaatccc ctaaggtaga ccactgcccc    889800
     tccaaggtga ccttatccta gagttgacac acatcctggg acaccagaga caactccttc    889860
     tctccccttt ctctgcactt cagctggaag caactgtctc accgagcacc ttgtgttaag    889920
     gaatgagagt tcctgttcca ggtgtgaggg cccaggtgca tccacttgat ccagcacaag    889980
     agcaagaaca gccttccaga aaatgacatc gcctgaggta taaccagctc tcacctgctg    890040
     cagcttcctc tgaataaaaa ggaaactgtt gaaacttcct cataagtgtc ctgctgtgcc    890100
     attccctttg tccccacatg ttcagttgtg tctgtccaga tgtcactttt gtgtagggag    890160
     attagggttc tgcttccagt accagaacac acatgacctc ttaggggact tcagggtttt    890220
     gctgacatat gtgatgatct taaaagtcat tagctccatt tctacatcaa aaaacatctg    890280
     aaccagagga gcacataggc tcaggcctgt aatcccagca ctttgggaag ccaaggcaga    890340
     ggaatcactt gaggtcagga atttgagacc agcctggtga acatggtgaa accccgtctc    890400
     tactaaaaaa tatatacaaa aattagccag gcgtggtggc actagcctgt aatcccagct    890460
     acttgaaagg ctgaggcagg agaattgctt gaacccagga ggtggagatt gaagtgagct    890520
     gagatcgcac cacttcactc cagcctgggc gacagagtga gcctccatct aaaaaaaaaa    890580
     aaaaatttat atatatatat atatatatat ataataaata tatattatat attatatata    890640
     tattttatat aatatatatg tatattttta tatattatac atacatattt atatattata    890700
     tataatatgt atatataata tataatatgt atatttatat attatataat atgtatattt    890760
     atatatatta tacatatata tttttatata tatatatctc caaaccatct aaatatcaag    890820
     tattttttaa tccatctaag agctgaaatt gctgaaaaaa ctactccctc caaaagctgt    890880
     agagacaggc acatccacag tcacagcaga gacttgctga cttggaaggg aagctcctgg    890940
     agacacattg gtgagaacat ttacctggtg attatgctga atgtctggag gacaaatgtg    891000
     gactaggggg agggtgagca ctcctagagg ctgtacaccc cacacttgtg tggacttgcc    891060
     ctccagggcc ttcaggttct cgtggaagca attaaaatag attccctata gccatgaact    891120
     ggggaggagt aatcacggag aaaagatgca caaaaagact tttctagaaa gctcatccaa    891180
     gggaaggtat tctccaaaat cttagtttat gtgggggaag gaaatacttc caaattacag    891240
     acccctcctc ctcagccttc ctctatcacg caaatgataa aattagccaa gaggagtcag    891300
     attcaaggcg gtagccctgg gtgcagcatc tgcagaaggg aggaaagaga gaaaatcagc    891360
     tgtatcactg gagattcctt gtgaaggtca ctgctcagga gaagagggca accaaaccag    891420
     ggagagtcaa ctgtaagaac ataccatgct cccctgcccc acacattacc tcctcaacag    891480
     catcattaat atggattaaa gagggcagtg tgattgcttt agatctgttt gagaaagaaa    891540
     gtcacatact gaggcctagg gtcagggtcg gcggcacttc ccgtgagtaa gatactacga    891600
     agaaggaaaa attaggggtc cataactgtg aaaatcagcc acagtgtgtg tgagaatgtt    891660
     tgtgtttgtg tttctgtgtg tttgagtagg agttattgga acagcggacg tggagtgagc    891720
     tttaatccac atccatctgc agcttcaggt attctcagat gcagtattca tctgcaagag    891780
     ccgaaatgag aaaagagcca cctccaaccc ccccagagtt ttagcctccc tttgtttcca    891840
     gtgatccagt gcatctagac ctccaggaag tggactccct ggtgatttta gcgattcttc    891900
     tcttggagcc accctgaaga ggacattggg tttccaaagg cccattcact atttcaagag    891960
     gtggtgtcat cagctcatgt tgtcactgaa ggagcattct gagccaggtc acagtcactt    892020
     cctagtgagc tacagaggct gagagaaaaa tgctctgtga gacccaatgg gaagctccct    892080
     gcagtgcaag gtctgggtgg cagggagcgc tagggcctcg cccagcacag gctgcagccc    892140
     tggagcaggt gcaagggagg ctggggaggg gttcctccca gggtctgatg tcttcctttt    892200
     ctcggacaaa catgctttaa taagttaaac aagactttag taaagactat tgatgtgtct    892260
     ttgtgtcttt cagtatacag ttctatttgt aggatttatc taacctaaca agtcaatgag    892320
     aatcacatgt aaaaggagaa atttctagga ttttcagata tcttaatagg taggagatgg    892380
     agaaaaggga tggttttatt aattcagtgc ttgccaatct taacagagac agtagtaaga    892440
     catgcagaaa gcaaagccca gaaaagtatg aaggtgtcaa agtgccattt aagtatgggt    892500
     tcacttggag gaccatgttc tgcgggaact tgttttcagc agacaatcta ttttagcaga    892560
     gttctgggca tacaagggga cacacatcat taaacaagga ttgggacagg gacttcagcg    892620
     tcccactgtt gcatggccca taaattatgt gtgttctctt tctcatcttg gatcaagtct    892680
     agagctatga aatagtatcc ctcatgaata tgcaaataac ctgagattta ctgaagtaaa    892740
     tacagatctg tcctgtgccc tgagagcatc acccagcaac cacatctgtc ctctagagaa    892800
     tcccctgaga gctccgttcc tcaccatgga ctggacctgg aggatcctct tcttggtggc    892860
     agcagccaca ggtaagaggc tccctagtcc cagtgatgag aaagagattg agtccagtcc    892920
     agggagatct catccacttc tgtgttctct ccacaggagc ccactcccag gtgcagctgg    892980
     tgcagtctgg ggctgaggtg aagaagcctg gggcctcagt gaaggtctcc tgcaaggctt    893040
     ctggatacac cttcaccggc tactatatgc actgggtgcg acaggcccct ggacaagggc    893100
     ttgagtggat gggatggatc aaccctaaca gtggtggcac aaactatgca cagaagtttc    893160
     agggctgggt caccatgacc agggacacgt ccatcagcac agcctacatg gagctgagca    893220
     ggctgagatc tgacgacacg gccgtgtatt actgtgcgag agacacagtg tgaaaaccca    893280
     catcctgagg gtgtcagaaa ccccagggag gaggcagctg tgctggggct gagaaatgaa    893340
     agggattact atttttaatg ttgtttacag tatgtcatta ataaattgaa aaaaagtaac    893400
     aatagaagta tatactctaa ttatatggga actttgtttt ttcagttttt tcattttttt    893460
     tttttttttt ggtttgtttg tgacagagtc tcactctgcc acccaggctg gagtgtaacg    893520
     gcacaatctc agctcactac aacctccacc tcccaggttc aagcaattct cctgcctcgg    893580
     cctccagagt agttgggatt acaggcaccc gccaccatgc ccggtgaatt tttgtatttt    893640
     tagtagagac ggggtttcac catgttagct aggctggtct caaactgctg atctcaggtg    893700
     atctaccctc ctcagcctcc caaagtcctg ggattacagg cgtgagccac tgcgcctggc    893760
     ccaattatat gggaattgtt tatataatta tcaccctata agcaaaattc atggaggagg    893820
     aaaagctcta ctgaagaaag ctgataccgg cattcccatg aaagtatctg tgtagaagta    893880
     agtattaaaa tcagttgaat aggcaaggca tggtggctca cgcctataat cccagcactt    893940
     tgggagaccg aggcaggtgg atcacaaggt aaggagttca agatcagcct gcccaagatg    894000
     gtgaaacccc ttctctacta aaaatacaaa gaattagctg ggcgtggtgg tgggtgcctg    894060
     taatcgcagc tattcgggag gctgaggcag agaattgctt gaacctggga ggtgaaggtt    894120
     gcagtgagcc gagatcacgc cactgcactc cagcctgggc gacagagtga aactccatct    894180
     caaaacaaaa caaaacaaaa caaaacaaaa aaacagttga ataaagtacc ttagagtcat    894240
     ctgttcaatt aacatgttta actccaaaga aatactgaaa atattttcca aaaaggaagt    894300
     gccattttac gttcctacca acagtgaata agattttctt ttctggagcc ttgtcagtat    894360
     tcactaatgc tttgctgtgc agccgttgta atattatagt aaatgagtag cagtatttaa    894420
     tggttgtttt aaatatacat attcttaata caaagtcttg atgaacactt ttttatacat    894480
     tgttttatga ggtgtgtgtt cagatctatg tatgccagaa atgcctggca gcgttaattt    894540
     aagcacactg taagaatgac cctatagttt atgaagaatg tatgttcaga gctctgagct    894600
     aagaaatcca ggagctgtca acccagaagt ttattccttg tctgtgaagg acatctgaat    894660
     ccctggccta tcccttggaa cacaggatgt ccaggtgatt gatgctcttt gttaaatctg    894720
     gaggttgcta ggtagagggt gctaagtgaa aatcataata taaactacat gtgttttaca    894780
     aatggtagtg gttttcctgt ccaacacact tttcctgggc cacattgtat gcaagtcctc    894840
     aatacaccct aggtcttgtt catgggctcc aggtctcctc ttcagccttt tggacatggt    894900
     gccacgccta ttacagtcaa taggggtcta gcatgacaac tggtaggccc agaacaaggt    894960
     caaagaaaat cctgcaagct cttagacaac agcgtcaagg aaggggagac ctgtgaggaa    895020
     atcccaggca ggccatgcac atctctgtgg gcccaacagc tgcaatcctt gatggatggg    895080
     gcccgctgca tgtgtacggg gatgcctcca aaatgccaaa agttctggag gacctgttgc    895140
     ctgaggtgga tgtgacaatg tgacaaagtg acagtcagat tcctgagctg tggcagctgt    895200
     tggccactcc tgactgcact ctgagcaacc actgaggcag agctcattgc acaggctagg    895260
     gtgtgtcagc cacgagaaca gttgtaacta taatgagatg ccgcctgtag ggataggata    895320
     gcaaattgga gactattgtt tatttggtag gccatttaaa gtgttgctga ctgccacacc    895380
     aatgcattag gactactatg actacgtcat cctgggagcc taagtcctgg cgtccgatgt    895440
     agagctccag tgggaaggag atgaaggtta ggatgagtcc ataaaggttc ttgctctgca    895500
     gcccctgctt tgctgtctca cttggtgaac agaggatggg aggtcaatgc ggacaaagtc    895560
     cagggtccag gcttatcagt caaatacttg gtgtcatctg gttacataag actatagtta    895620
     ttccatattt catcatagat aagataagat gcaggtctac tcatgtccca ccacaccaaa    895680
     gcagttggaa acctcccaag gcctcctggg acattggcga tcctttattc cccatttcgg    895740
     caaacccctt gggcccccat ggcacttaga caagaaggtg ccccactgcg actgttccaa    895800
     aagggaggat gagggctctg aagaagctga agtcacagtg aaatgaatac aaaccttggg    895860
     agttctagtg cagggacagc cctgtgaatt ggatgtagtc agttaccctg aggggtttag    895920
     gtggggactg tgttaaaggc aaggacataa gtgtgtgtcc ctaagaccct ggtctcaaag    895980
     acagaaggaa gctgaagtga gatatactgt ttaggagtag caactgcact acatgtcatg    896040
     ccttacgagt gtaggatgtg acaaagaggg ccactccaca tccggaacaa cctttagcag    896100
     gctggctaaa ggatgccttc cagacacaaa agccttggaa tgccaggaca cagtctgtag    896160
     ccaaatggta cttgtggtgg tcaccaccaa cataaaagtg ggccaactgt gccagcagaa    896220
     gttagcccca cagaactttc ccccacctaa gagaaggcag tgcaccacaa tgcggaattc    896280
     caccactgtg gaattggggg agcttagaat tggattcaga cacaagggga gagagtggat    896340
     cacagggtgg cttctctatg ggataggggg tggagagtat tatactctct ggactcaaga    896400
     tgagtaaaat gacacccatc acaaaccatc cagccctatg atggcacctt tatggtaagt    896460
     ggttgcaggc tccaaggggg ccgggtccaa tgaggaagat gcccccacag ctcgttctca    896520
     atggcagact atagaagagt tgcaggatat cttctgggag tcgagatgag gcatgctaat    896580
     tatgctgaga attattgaag tcccaacaat gaattgttta ctgcaaaata aaagctacag    896640
     ttatgtattc agtgcctacc caatggcatg gtgcactgat ttccatgtta agccccctgg    896700
     gagggcagcc aacatttcat gtgccccagg tagttgctga cttaggagaa aagaagaaac    896760
     tgagtaagca agggatgcac cctactgtga tgaagaacaa tggcaccaaa ggaagagaga    896820
     cagccaagga gccagtcagg gtggccagac aacgaatgtg ctctaactgg caacacctac    896880
     cagttctctg ggcccatagc ggcaataggt ggttatggaa gggccacgga agtcagaccg    896940
     gttgaactag tgatacgacc tgggggactg ccacccagac cctgtgtagt atacacagct    897000
     tccatcctag aacacatgag aatggatatc ttcttaggcg tgaccctcca aacaacggcc    897060
     agggaattcc aacggagagt tagagtggtg atgtgtgtga ccaagcagaa ggcaaactgg    897120
     atgccagtag agctgccaac ccatggggag tcccacagct ggagcaacac cacctgccct    897180
     gggaggggaa gatgatccaa tcatgaagat tgttaaggag ctagcccagg taggcattag    897240
     gaggccactg catggttcct acaacagacc tgcatggccc atgcagaggc cagttgagac    897300
     atggagaatg acagtagatt actgggagtt aaataaggtg gtctcccgag tgaatgcagc    897360
     tgttcctaat atctcctcca gtctgacgag aataggagag gtgttagcca cgtagcattt    897420
     ccttatcagt ttagtcaata ccttcttcag catttctgtt gccccagagt caagatcaat    897480
     ttgcattaac ctaaaaagaa caatggactt ttactgtctt gttccaggga tatttacaca    897540
     gcccaaatct cacagcctag tgacctccaa cctcagtcga tgggctgacc caaaggggat    897600
     acatgttttc cactacattg gtgttatcat gataacctct gagtcttttt tcagcttata    897660
     aattacagcc cctgtcttgc tgtctcactt gctgaataga ggatgggagg ttaatacaga    897720
     caaaatccag ggtccaggct tatcagtcaa atagttggtg tcatctggtt gggtaaaact    897780
     aaagtcattc catctgccat catagataag gtgcaggcct acccacgtcc caccacaaca    897840
     aagcagctgc aaactctcaa ggccttctgg agcatcagtg tccttttatt ccttttattt    897900
     gacatccctg aaggaggctg ctaggggaga ctgtgtccct cctaaattca tgtgctgaag    897960
     tcccaaccct tggtccttca gaatgaaatc atacttggat tagtgtcctt taaagaggtg    898020
     aataagttaa agtgagattc ctggagtggg gccctaatgc aatctgactg ttgttataag    898080
     aagaggaagc aggagggagg gtgcacaggc cccgagggac ggccatgtta ccacagaaca    898140
     gtgagaaggc gccatctgca tgccagggag cgagacctca gaggaaaccc acccagctgg    898200
     cagcttgatc ttaggctttc atcctccata agtgtgagga aattggtttt gtattgtaag    898260
     ccatccgatc tgtggtattt cgttataaaa gccctataaa atgaatacag taggtaatag    898320
     gagagcttct atacattgaa aaagtcggat ggccagaaaa acctagacac tcctgttcag    898380
     acctgagcag ggtgatggac ctgctttggg acaggagagg ggaagagatg aacccagcac    898440
     ccagacccag ctgagcccat tcctcagcag gccgtccctg ggccggagct tgcacaggtg    898500
     tgaaagagcc tgtcttggtc ttcaggggct catggagttg gacggagaat ggtgtagact    898560
     caagaacacg tcatcggtgt gcccgtgttt atgtgaatgg gatgtgtttc tagggtgtgc    898620
     tcatccccaa agaagaatta atcaggtctc ttgggctaga aagaggttgt ggcatttgtg    898680
     tgtattaata actgtggtcg gacagtaaat tatgttaaac tgcttatggg aaggcacaat    898740
     ggaaagaaac actttgttac agaaggaaaa aaaaggtgat tatttaaatg aggtgccttt    898800
     gaaggtcacc atgccaagag gagcccatca catgatagtg ctggctttca tgttcaggag    898860
     atcaggaggg tccgtccgct ggcttttatg acaccctaga cagagctgag agtgtaatgt    898920
     atgaatggag gggaagtgga gagaggggag gccaaatgtt tggtgggaat ggagggtcac    898980
     tattggagcc attaggaaat acacaagcat gaattatgct ggaggacaga acagtgttcc    899040
     tggggaatat tgtgttgctt tgggagctgc tgaacataca ggagtttcac tgttcctagt    899100
     tctcaaattc tctagactct cttgacaacc cagttttaaa tattgggaat ataggtaaga    899160
     cacattcgtt attaaaaatt attaagagaa gatgtaggaa gaaatttaaa gtaatccatt    899220
     tggttatgaa aatttagtta cagcgaactg tgatgtccgt ttcttacttg gaataatgga    899280
     atgtaagtca ttagtcatct caacggttca tttttccata accatcaatt acaaaactgc    899340
     tgagtaattt cctgaattgc ccaccataga aactgacctc acatttcctc aatgagaaac    899400
     tgccagtccc gttgatccag cctcgttctt cccatcaggg attttgtatc tctgtggacg    899460
     tgtggcacag tgctgcatat ccatcggcat atggcctcag gaaaggcgcc agcctatcca    899520
     tgcatgatga agcttactta ggggatgaag cccgcatgct gggtgagcca gtgccgacag    899580
     ctgaaagaat caactgcctg gtgtatgatg cttttatgaa aacaagccca gggcctcttg    899640
     cattcttctg tattagattc tctggtgaag atttttattc atttctgcct gaaattgcca    899700
     catataatta cctagaagca ttacaataaa ctgatttgga agttaactga cttcctggtg    899760
     aggttaaaat gagtgtcagg tgcatagtga gacagaccgg agacatggat gcatagcaaa    899820
     cttgtgctca ccatggtttc tatcttagtt agggaaactt ctgtaccttc cttagatgtt    899880
     caggcactcc attgaggacc ctggcataac attatttatt gacagaccat agctcaaagt    899940
     atagaactgg atactaccaa ggaggatata ctattactat tttatcttta tcttaaaata    900000
     tactcttcca tctgaggtga aaattaatcc agatggtaga acttattgca gttactacag    900060
     cattttagca aatcaaaagc cgcagaacaa acatatggac agatggcagg tatgttttcg    900120
     gaatcgtaaa caagttcgtg atgactgtaa aaccaagggg tgtctcacga gggctggaaa    900180
     cctctcacaa tgaaacaaca caatgaggat ctttgaaaag tactctgacc tcctggtgag    900240
     ctggctgata cggaggctga gctccatgta gaaagccaaa ggaatttctg caggacgtca    900300
     tcatgccaag cacagccgta acctgggtcc cagccctttt cacacgctca atggttagat    900360
     cttgggaggg aatcaaagaa gccatagtaa aatatcaaaa tttaaacccc gattttgaat    900420
     ttaaaaagtg ttaaaatatg gttgtggcct acactcagaa aatctgtgtc cttcagatgg    900480
     tttctcagtg gcaccagatg gtttcaagtg gctattcatt aagtttctca gtgaaattac    900540
     cagacataga ataaataaat tgtcactgtc ttaaatcaac ccatgggaaa ggaaaactgt    900600
     gtaaatacag cagagaggaa acattgctca agggaaaaac aatctccaga aagtattgtt    900660
     aaagaaacag aggccctctt tccagccagc gccgagcgat gggcacctct cgggacaact    900720
     ggcacaaggg ccgcaaagct gggggcaaga gatggccctg ccacaagaag cggaagtatg    900780
     agttggggcg cccagctgcc aacagcaaga ctgacccgtg ccgcatccac acagtccgtg    900840
     tgcgaggagg taacaagaaa tactgtgccc cgaggctgga cgtggggaat ttctcctggg    900900
     gctcagaatg ctgcgctggt gaaacaaggg tcatcgatgt tgtctacaat gcatccaata    900960
     acgagctggt tcgtaccaag accgtggtga agaattgcat cgtgctcatc gacagcacgc    901020
     cctaccgaca gtggtacgag tcccactgtg cactgcccct gggccgcaag aagggagcca    901080
     agctgactcc tgaggaagaa gagattttaa acaaaaaacg atctaaaaaa attcagaaga    901140
     aatatgatga aaggaagaag aatgccaaaa tgagcagtct cctgggggag cagttccagc    901200
     agggcaagct tcttgcgtgc atggcttcaa ggccgggaca gtgtggccga gcagatggct    901260
     atgtgctaga gggcaaagag ttggagttct atcttaggaa aatcaaggcc cagaaaggca    901320
     aataaatcct tgttttgtct tcacccatgt aataaaggtg tttattgttt tgttcccaaa    901380
     aaaaaaaaga aagaaaaaga aacagaggca tcacacttac tagaaaaaca tattctattt    901440
     catatattat ggggatatga cgtgatgttt tgacgtatgc gggcattgtg aaattattaa    901500
     atcaagtaaa taaacatgtc catcacctca catacttatt ttttatggtg taaacgtgta    901560
     aaatctactc tcttatcagt tttcaagtat atagtacatt agtatcatgg aagtcaccat    901620
     gctgtgcaat agatcttcaa acgaattcct tctatctaac caaaactctg taccctttca    901680
     ccaacgtctc agctttcaca tgcccctgac gccagcccct ggtaggcacc attctactct    901740
     ctacttctct gagttcaaca tttttagatt gcatgtgtaa gcaagatcat ggagtaattt    901800
     tttatacctg gcttatttca ctcaacataa agagtcaaat gctcaacatc actaatcatc    901860
     agggaaatgc aaattaaaac catgataaga tatcacctca cacatgttac aatggcttag    901920
     tctcagtctg tctttttgtt actataaccg aataccagag actgggcaat ttctaaagaa    901980
     aaggaattta tgctttatgg tgcttgagtc agagaagtct aatatcaaga cactggcatc    902040
     tcacaaggga cttctcactg cctcatctca cagcagaggt gggtgagcaa gagaccattt    902100
     gtccacgaga gaaaggagac catcttttat tagaaattca ctcctataat aactaaccca    902160
     ctccattgat agtgacagta atccattcat gaggacagag ccttcatgac ttgatcacat    902220
     aataaaggtc ccacctctca acactgttgc attaaagatt atttccagat cctaaacttt    902280
     gggagacaca tttaaaccat agcattccat tcctaatatc aaaatttatg tccttatcac    902340
     aatgcaaatt acattcattc catcccaatt gtctccaaag tcttatccag catcagtgca    902400
     aaagtctgaa gtccaaagtc tcatctaaat cagatatgag tgtgactcga ggcacaattt    902460
     agcctgatat aaattgtttc catctgcgag cctataaagt caaaacaagt tatctacttt    902520
     caaatacagt gaacaatggg gcaggtatgg gatagaaatt cccattccaa agctcagaga    902580
     gaggcaagga gaaagcggtg cctagttcaa aacccaacag ggaaaaaaac attaagtctt    902640
     atagctggaa aatcatcctc tttaacggca tcttgtgcac actggggagg gggatgggcc    902700
     cccaaggcct ccggcagtct tgcctctata tattttctgg gttcagtcca ctcagccgct    902760
     ctcacaggtg ggactctcag gcctctagct ctcctaggct gactggaaac tctttgtggt    902820
     acctccaaac ccacatttct gcttggcatt gtgctaaggg cccagtgtgg tgactctgtc    902880
     tctgcaacaa gtcactgccc gagaccttag gctgtcctta ggctgcccga gaccttaggc    902940
     cgtccacagc attctttgaa atctaggtgg agaaagccat gccctcgtgg ttcttgtatt    903000
     ctgcacacct gcagaattaa caacacatgg atgccatgga agttgatgac ttgtaccatt    903060
     aaagtgatgg cttgagccac acctaggtcc tcctgagcca cagcatgggc agccaaggag    903120
     tgctgtgcct ggacaccggg aacagagtcc taaagtgcct gctagaagtg aggccataga    903180
     tttccttcaa atttctccca ccatataacc tcgttcatgg ctctgaactt ccaccttaca    903240
     gaaggaccta gggatgaaca caattcagcc acgttctttg ccactttatg gcaaggatgg    903300
     cctttgctcc attttccgat gagctattct tctttttctc ctgagacctc atcagaacgg    903360
     cctttattgt ccacatttct actgacatct aatggtcatc acctaaataa tctctaagaa    903420
     gtttcagaat ttcctcacag ctctcttctt ctgagtcctc aaaagaatca cctctagtgt    903480
     tctattcagg gcaatccaga ctttttatag tctgatcctc caaattattc cagtctttgt    903540
     gcattactac atccacttct acattttggg gtatttgtta tcgcaacagc cccacctctt    903600
     gatactgatt tttcgtctta gtccactttg tggtgcaatg agtgaatacc acacactggc    903660
     taaagtataa ggaaaaggaa tttattttct cgcagctcta gaggctggga agtcaatatc    903720
     aaggtgttag catctggcaa gagccttctt gctgtgatgt ccatgtggaa ggcaggagag    903780
     caggtgcgaa ggatggaaag gggtttaaac tcatttttta atgaggaacc caggcctgta    903840
     gtaactaatc tgctaccaca atgagtaacc tactctgacg ataatggcat tcattgcttc    903900
     atgagggcag agccctcatg acctaatcat ttcttaacat tcccacctct ggacactatg    903960
     gaatttggga ttaagtttcc aatacacatc ctttctaaac agcaggggct ttttaatagg    904020
     ttgaccaccc aaggctgcag gaggctctga agcagtggcc tgaggttggc tgtcctttgt    904080
     gagaatggag agaagtgaac tgactcatgg agacacaagt agatgaggta aaggcattca    904140
     ttgcttcatt acatggatgg tgaggtcgat tgaaggcatt aacggattaa agatggtggc    904200
     aaaaccgtct gaggtggaga ccacggggag tccatcagaa atggaggaca cgtcccaata    904260
     aatggtgctt catttccctg caaagcagat gaaagcaaag aacaaaacac aacatcatag    904320
     tgtacactga gcagtggatt gagagaagag tttcctaagg cataactgac agagtggaga    904380
     agacacacaa atctttgcat gatgctaaca tttggactgt ggcttcatta tttcttatta    904440
     atattttact gaaatatcgc tagaaggaga ctgaaaatga agtgtgaaaa gttaaatggg    904500
     atttctgctc tatgtccttt tcagatgaga ggaactaggg aattccaggg aagaaacaat    904560
     aatagctgct gagcaaggct tttgcagggc aggacaagga atccccaaag agaaaacgga    904620
     aacctcagct tcactttgca tctgctcctg agccaggtcc tgagcgaccc ctgtaggtcc    904680
     tgagtgcccc tccgtaggtt ctgagcatcc cttggttgct gggcgccctc tggtggtgtc    904740
     tgagcccctc tggtggtttc tgagcccccc cgccttatgt ctgatcctcc ctggtggtgt    904800
     ccgagtgccc ctgctagtgt ctgagccccc tggtggtgtc tgagtcccct tcttagtgtc    904860
     tgagccaccc tattagtgtc tgaggacccc tgatggtgtc tgagccccca gttagtgtct    904920
     gagccaccct attagtgtct gagcccccct ggtggtgtct gagcacagga gagctcctct    904980
     gaaggaaggg tctacatggg gacaggcgtg cttgtctcag ggaagggtcc atgtggggac    905040
     aggtgtgctt gtctgaagga aggttccaca tggagacagg tgtgcttgtc tcagggaagg    905100
     gtccacatgg ggacaggtga gcttgtctga ggggacagaa gtgcttgtct caaggaaggg    905160
     tcctcatgtg gacaggtgag ctcttttgag ggaagggttg acctggggac aggcatgctt    905220
     gtctgaggta agggtcctcc tggggacagg tgtgcttctc tcagggaagg gtccacgtgg    905280
     ggacagaggt gcttgtctaa ggcaagaatc caagtaggga caggtgagct cgtctcaggg    905340
     aagggtccag gtggggacag ttgtgctcat ctgagagaag cgtcgaagtg gggacaggtg    905400
     tgcttgtctc aaggaagggt ccatgtgggg acaggtgtgc tagtatcaag aaagggtcca    905460
     catagggaca ggtgtgcttc tctcagggaa gggtgcatgt ggggacaggt gtgcacatcg    905520
     gagagaatgg tccacctggg gacaggtgtt cttgcctcag ggaagagtcc accttctcag    905580
     ggaagaagtg tgctcctctg agggaagggt gcacatgggg acaggtgtgc ttgtctcagg    905640
     gaagggtcca tgtgggaaca ggtgagctca tctgagggaa gagtccacgt ggggacaggt    905700
     gagctcatct gagggaaggg tccacatggg gacaagtgac ctcgtctgag ggaagggtcc    905760
     acgtggggac aggtgagctc gtctgaagga agggtccact tggggaccgg tgtgctcctc    905820
     tgagggaagg gtccacgtgg ggacaggtgt gctcctctgg agggaagggt ccacgtgggg    905880
     acaggtgagt tcatctgaag gaagggtcca catggggaca gttatgctcc tctgagggaa    905940
     gggtccatgt ggggacaggt gtgcttgtct cagggaaagg tccacgtggg gacaggtgtg    906000
     ctcaccttgg ggaagaggac agatgagctc atctcaggga aggggccatg tggggacagg    906060
     accaagggtt gggacttcag cacaagaatt taggaggaac acagtcttcc ctagcagcct    906120
     ccttcaggga tgtcaaatat tttccttctg ttccctgtga aagccttaaa ggggtaggga    906180
     aagggcgttc aacctgcaca ctcgtagagg ggaaaccagc ttcattagta atcgttcatc    906240
     tgtggtaaaa aggcaggatt tgaagcgatg gaagatggga gtacggggcg ttggaagaca    906300
     aagtgccaca cagcgcagcc ttcgaaacac accacggtca cgttaagttt aaatggagtg    906360
     accacattcg ccaggaaagg gaaatattta cacttttgaa gaaacagtaa tttgtgtttc    906420
     tgattatgat ctggccttgg attttccctc ccctcataag caatgacaga attggcagaa    906480
     atatgtgaaa cgttagttct cagacatgag acacccacag agggccccct gtgcccttcc    906540
     ctgagagctg atcagctcct gcatctgaag aaatgaccaa agaccaggag agaaccacac    906600
     agaagcatcg gagggacagc acctggggct ctgatggggt caggaatagc atctgttccc    906660
     aatagatgga ctaagtaaaa agtatcataa ttcacaagag ttttacatag cacagaagaa    906720
     aaagttaccc tatatcaact gttgatcttg tgaatccagg aactctggat tcaaggtggt    906780
     cgggcacatc ttgatttacg catttcaggg acacatgaga catcagtcaa tataagtaag    906840
     aaggacatta gttccatcca gaaaggctga gacaactcaa agcaagtcct ccccacttag    906900
     ggcttccagg tcacaggtag gtgagagaca gatggttgca ttcttttgag tttctgataa    906960
     gtgtttgcaa aggaggccat gaggatatgc acctgtctct gtgagcagag ggacaacttt    907020
     aaatagactg ggaggcagat ttgtcctgag tggtttccag cttgacgggg cccaagatat    907080
     tttcctttca caatctggta acttcaaaca aaacttcaaa gccacaacaa aacaacacaa    907140
     caacaaaaag aataagacat gggtacttat taagagtaga aaaacattca gtccccaagg    907200
     aaaatattgg cagtgtctac ctccacatga caaaggagta agcagtgtga gccacagaaa    907260
     ggagcactat taacccacag agcaaccgag aataacacgg gtgatgcaag ggcattggac    907320
     gcacatcatt gcattttgta gattcagaaa gaaacggaaa agattgacgg tggtaaaaga    907380
     gacagccctg cttccctctc ccttttccct ccccgatgag gcctcacagc catgaccctc    907440
     agcctcatcc cgcagtgcag cagctgccgt cctgtccagg cccaccccct gccccgccct    907500
     gggactgtta cctcattccc tcccggagtc caggtgcccc ccggggtgtg gtgcgggagc    907560
     ctggggaggc cctttgttct ctgtcagggt ctccctggga gggacgcagc caccgcagct    907620
     ggttggggcc tggcttcgcc caggacagtc ctttcctttc ccattgtctt tggatgacta    907680
     tcgctgggct gggacatgag gcgggcagag gcgcgggtca cccttaggac ccccctcttg    907740
     ctgctggggc tctgggcgct cctggctccg gtccggtgtt ctcaaggccg tcccttgtgg    907800
     cactacgcct cctccgaggt ggtgattccc aggaaggaga cacaccatag caaaggcctt    907860
     cagtttcccg gctggctgtc ctacagcctg tgttttgggg tcaaagacac gtcattcaca    907920
     tgcggaggaa acaccttctt tggcctagac atctgctggt gacaactcag gatgaccaag    907980
     gagtcttgca gatgggtgac ccctacatcc ctccagactg ctagtacctc ggctacctgg    908040
     aggaggtgcc tctgtccatg gtcaccgtcg acacgtgcta tggggacctc agaggcatca    908100
     tgaggctgga cgaccttgcg tacgaaatca aacccctcca ggattcccgc aggtttgaac    908160
     atgttgtttt tcagatagtg gccgagccca acgcaacagg gcccacattt agagatgatg    908220
     acaatgagac agaccccctg ttctctgaag caaatgacag catgaatccc aggatatcta    908280
     attcgctgta tagttctcat agaggcaata taaaaggcca cgttcaatgt tccaattcat    908340
     attatcgcat atatggcaat attacaactt gttccaaaga ggtggtccag atgttcagtc    908400
     tcattgacag cattgctcaa aatattgatc tgcggtacta tatttatctt ttgaccatat    908460
     ataataatcg tgacccagcc cctgtgaatg aatatcgaat tcagagtgca atgtttacct    908520
     attttaaaac aacttttttt gatacttttc atgttcattc atccacacta cttattaaat    908580
     acgtgccaca tgaatctaac tatgaacctg aaaggtataa cttctgttcc cgtatagccc    908640
     tgttacacat tggtactcca ggcagacatt atttattggt agccgtcata ataacccaga    908700
     cacagatgag aagtattggt ctggagtatg atgataacta ctgcacatgt cagagaaggg    908760
     cctcctgcat tatgcagcga tttcctggga tgacagatgc gttcagtaac tgttcttatg    908820
     gacatgcaca aaattgtttt atacattcag gccagtgtgt ttttgaaaca cttgctcctg    908880
     tgtataacga aaccatgaca acggttcgct gtggaaacct catagtggag gggagggagg    908940
     aatgtgactg tggctccttc aagcagtgtt atgccagtta ttgctgccaa agtgactgtc    909000
     acttaacacc ggggagcatc tgccatatag gagagtgctg tacaaactgc agcttctccc    909060
     caccagggac tctctgcaga cctatccaaa atatatgtga ccttccagag tactgtcacg    909120
     ggaccaccgt gacatgtccc gcaaacgttt atacgcaaga tggaaccccg tgcactgaag    909180
     aaggctactg ctatcgtggg aactgcactg atcgcaatgt gctctgcaag gcgatctttg    909240
     gtgtcagtgc tgaggatgct cccgaggtct gctatgacat aaatcttgaa agctaccgat    909300
     ttggacattg tattagacaa caaacatatc tcagctacca ggcttgtgca ggaatagata    909360
     agttttgtgg aagactgcag tgtaccaatg tgacccatct tccccggctg caggaacgtg    909420
     tttcattcca tcactcagtg agaggagggt ttcagtgttt tggactggat gaacaccatg    909480
     caacagacac gactgatgtt gggcgtgtga tagatggcac tccttgtgtt catggaaact    909540
     tctgtaataa cacccagtgc aatgtgacta tcacttcact gggctacaac tgccaccctc    909600
     agaagtgcgg tcatagagga gtctgcaaca acagaaggaa ctgccattgc catataggct    909660
     gggatcctcc actgtgccta agaagaggtg ctggtgggag tgtcaacagc gggccacctc    909720
     caaaaagaac acgttccgtc aaacaaagcc agcaatcagt gatgtatctg agagtggtct    909780
     ttggtcgtat ttacgccttc ataattgcac tgctctttgg gacagccaaa aatgtgcgaa    909840
     ctatcaggac caccaccgtt aaggaaggga cagttactaa ccctgaataa cactaattca    909900
     gcctcccgat ccctgtaaag atacagagaa tataacagca aaatctatga aacaggatca    909960
     ggggaaggga tggcaaagct caagtccaca tttcttgaag tccacaggaa gcacagggtc    910020
     ctgtttcaca tcacagggaa acgggaggca ttggcttctg tcccaggttc ttgtaggtcg    910080
     ctgatgctca ctctgaaata aatcttcaaa aacacattgg tgccttccac attttcttag    910140
     actcctctgg gagcccaaac ttggccagaa cctcttgcct ggagagacat gaatgagcat    910200
     ctggctcttg acctgaggtc tctggtccca gaattaacgg aagttgccac cagctcctta    910260
     cagggaacat tcatgacatt tctccagaag agagctccag agcaatgagc ttcctcattc    910320
     cccaggtaat ctgtccttct ctaaacccga agtcagttta gggtgatcca gggctactcc    910380
     ctgttccctg tctgttcctc acgggggtgc tgtgggcttt tcagtgagag ggacttgggt    910440
     tcaaatcccc caccaagcaa atccccctac ctggggccga gcttcccgta tgtgggaaaa    910500
     tgaatccctg aggtcgattg ctgcatgcaa tgaaattcaa ctagaaaaat aggtagacgt    910560
     gaggggcaag ctgtctgtca tttagtgtga gctctgtgag tggcagctgc cccctttctt    910620
     cctgccccca catttccttg aactgaaaca ggaagggaag ctgagtaagt cgtgatgagg    910680
     aagagaaacc aggcttgtag cagcacaggc tggtccgggt ggaaaacagg gctaggtgta    910740
     tcactgagtt attgtaaagg aaaatggaag ttaaatgtat aaataactga atgagataac    910800
     attttatttt aacttaaaat tcacactaat attgactttt aaaatgcagt gtagatatgt    910860
     cagagagaat ttcaaaggca aagcccaccg acggaagaaa tcacccttcc cataccatcc    910920
     acagaaaact gttggtattc tagggtagta ctgagatcta gcatttttct gaatacatcc    910980
     gtggttctag atgtcctgct tccatagata ttgtttagaa ttcccacccc tttctccaaa    911040
     cacagcttga tatcctttct ctgaacctgt tagaaatttc ctccattcag ctgtcataaa    911100
     gatgcgagca atccattcct gtgcctctgt cagtgtgttc tattattttg tgggtgaacg    911160
     ctaatggaca gttaagtgtg aggtcagtga atacagtgcc ctccctctat gtgtccttcg    911220
     ggtgtgaggg gttttgctga tagagcagca ggccccatcc caccctttat gcatctccgc    911280
     cccccacctc acgctccagc tgacctctcc cctgtggcct ggggcgttcc ccagggggaa    911340
     tgacctctcc tctctccagg gcccacccac tcagtgcccg tgcaagacca ccacgcttgg    911400
     cacggcccca cctcgcgtca gggcctgtgt cccctgcccc accccctaaa cagatgggaa    911460
     ccactgggac tctgctcagg gcagggggcg gaggtatgtg tgaaaggaag gcaaatgtgc    911520
     actctgttgg agaaatatta taggtagttt gagcaaaaaa tctaatgcca tgtgaacttt    911580
     tagaatgata cgtattttaa caaagaacat gaccaataga gtttgtattg aagccaggaa    911640
     aacactattt agagcaacag caatatcaaa aacacaagcc aacagttcac caagaaaaac    911700
     caccattaac cccatggaaa tggtcttcca agagcatcgg cacttaaatc ctcgaaatct    911760
     gcctgcctca gcacctgttg tcctgacctg ccctcctgtg tgtcctaatc actcccaaac    911820
     acggggcctg cactgtggga gattcacgct gtgccaggtg gagggagcag gacaactgct    911880
     aacaggttgt tggtgtggat gccgaggcca cccaagcagg tgtaaactcc cacctgtggg    911940
     ccagggaaga gtgcacggga gacatgtccc gggcataggg tgagggagag ctgtgggggc    912000
     tctgggttct gaagtgggtt ctgaagaggg ttctggcctg gcagggataa gaccaaccag    912060
     catgtgaggc caggctggag tctggacctc tgaagctgca agggtcatgg gctgcttggc    912120
     cccaggggct gtcctggttc tctatggagt actttcaaac attctttctt cttccaatcc    912180
     ccctccttct ctcccaaagc ctgcatctcc caaatcctct ttgtcggatc ctcggcttca    912240
     ctctgcatcc gtcctgagca tcgatcttcc aattccatcc tcttctcttc tgctatgtct    912300
     aagctgctgt gaagccacct gctgtaattt actgctttat atttaatatt gtaccgtaca    912360
     tctgttctgt ttccctcatc ataaatgctt catttcatgc tcagcatctg agaacacaag    912420
     gccttgtcag ctgtcacctc cttccgttct ctgtttcctt cctcctatcc ccatattgct    912480
     catcatgtcc agtctcctgc catcctgaat gcttctgatg gaaggtctga gatgtctcat    912540
     gagcactgtg aagattcttt gtaatgtgac cttgttccag gcaggaattc tccttcaccc    912600
     agccctggaa gccaagtata ggcagatggc catgctcaat caaagactga gctaacttaa    912660
     cagtggcttt ggttttaagg tttctccaat ccccagggca caggatttca gggaattcag    912720
     gtgagagtct gggtgttacc cttcaggagg ctgtaaactc catttcacct agtctacacc    912780
     acagactatg gaaactatat atatatatag ttctgtccct ctagagaaac ctaatatgta    912840
     tatatacaat atataatacg tattatatat tatatataat atatatatat attagagttt    912900
     attgaggagt attaaactca caatcacaag gtcccacagt aggccatctg caagctgagg    912960
     agcaaggaag ccagtccgag tcccaaagct gaagaatttg aagtctgatg ttcgacggca    913020
     ggaagcatcc agcacaggag aaagatgtag gctgggaggc taagccagtc tagtcttttc    913080
     acgtttttct gcctgcttta tatcctggcc acactggcag ctgattagat ggtgcccacc    913140
     tagattaagg gtgggtctgc ctttcccagt ttactggctc aaatgttaat ctcctttggc    913200
     aacaccctca cagacacacc caggatcaat actttgcatg cttcaatcca atcaagttga    913260
     cactcagtat caaccatcac aagtccaccc cttgtcaact tgaacccata caaatctcct    913320
     gagatcatac ataatcttca aataaagaca ataataaggt cataattaca cctaatgtaa    913380
     tacaactatc ttttgtacaa ccagaaatgc accaatcccc aacccaaatg ctattatgta    913440
     aagttaagaa cacttaaatg ctgatatgaa gtcaataaat tttatgtcac atgataaagg    913500
     aaaaaagaaa tgaaggaatt ttcttagtac aagtgtgtac atgcacaaac atgtttttaa    913560
     caaaagaaga aggaaatact gatgacaatt acggtcctca tttctgcaac tgatcacgtg    913620
     gttgtagctg gtattgatga ctaccttctt ctactaccca ttctgtattc cctttgcctt    913680
     cagcaagcat cacagcaggt agagtttttt ctcctagtgg agtgatgcaa accttcattc    913740
     ctgaagggtc tgggccattt gtagtcctgc ctggattggg ctgttgtagt ttcccgttga    913800
     ccttaatgac agggcatggt aatgttaaga gacgccctaa tggatctcct gtattccata    913860
     catattcttc cttgcctcca ttgtggagta atagactgat tgcatcttga tagtccaggt    913920
     caatcagccc agccaacact gtaactcccc tcttagcctg tggacttaaa ggtaggaggg    913980
     gcccaaagtg gccaggtgga aatctttact tccagtttaa tggaattgtt gttgtttctc    914040
     ctgatggcag cattattccc actggaacta agacctctag gccaacagaa tgtaatgtca    914100
     tgggaccagg aagcaaaaat tttgctagtg gatcactagg ggtgatggtg aatggtgcca    914160
     tttccacttc caccccttga ttcctggatc catgaattat ggctatggga gaaagagtac    914220
     catatattgg atgctgattt ggagcataca tggccttttg gagagctttg ccccagccct    914280
     gcaaagtatt ggagcctagt tgacattgta attgtgactt tgaaaggcca ttccattctt    914340
     ctatcaatcc agctgcttca ggatgatggg gaaaatggta agacaagtga atcccatgag    914400
     catgagccca ctgccacact tctttagccg taaagggagt gccttggtca gaggcaatgc    914460
     tatgtggaat actgtgacag tggataaggc attccatgac tccacagatg gtagtcttgg    914520
     cagaagcatt gcatgcatat ctgcagtaag tgtctatttc agtgaggaca aacctctgcc    914580
     ctttccagga tggaagaggt ccaatataat ccaacctgcc atcaggtagc tcactgatca    914640
     ccctgaggaa tggtgtcatt tgggtagaga cccagagcca aaccagatca cgccacccaa    914700
     cccctcccaa atctcatgtc ctctttgcat ttcaaaacca atcatgcctt cccaacagtc    914760
     ccccaacatc ttaactcatt tcagtattaa ctcaaaagtc caaatccaaa gtcacattgg    914820
     agacaaggca agtccctttc atctatgaac ctgtaaaatg aaaaacaagt cagttacttc    914880
     caagacaaaa tgggggtaca ggcattagat acatgctccc atttcagttg ggagaaatga    914940
     gccagaataa aggggcttca ggtcacatgc aagcccaaac tccagtgggg cagtcattaa    915000
     atcttaaagc ttcaaaataa tctcctttga ctccattcct cacattcagg gcatgcttat    915060
     gcaaagtggg ggctcctaca accttgggaa gctctcaccc tgtggctttg cagctctgac    915120
     cccatggctg ctctcatggg ctttgcagag ttcagccctc ctggctgctc tcattgagtg    915180
     catgcagctt ttccaggtgc acagtgcaag ccgttaatgg atctaccatt ctggggtctg    915240
     aaagatggtg gccctcttct cacagcccca ttagtcactg tctccagtgg ggactctgtg    915300
     tgggggctcc aaccccacat ttcccttctg cactgcccta gcagaggctc tccatgaagg    915360
     ctttgcccct ggcgcagact tctggctgga catccagtca tttctataaa tcctctgaga    915420
     tctgggtgga ggatcacaaa gctgaactct tctcttccgc acatccctag gcccaacatc    915480
     atgtagaagc caccaatgat tggggctttc tgaagcaatg gcctgagctg tacattggac    915540
     ttttttagcc acagctagac ctggagcagc tgggacacag ggcaccaagt cccaaggctc    915600
     caaagagaag ctgggccctg gacccagccc atgaaaacat ttttccctga taggcctcca    915660
     ggcctgtgat tggaagggct gctgcaaaga tctccgacat gccctggaaa cattttcccc    915720
     attgtcttga ttattaatat tcatctcttc attacttatg caaatttctg cagccaactt    915780
     gaatttctcc ctagcaaatg tgtttttctt tactaccaca tggccaggct gcaaattttc    915840
     caaactttta tgctctgttt cccttttaaa cataagttcc tatttcagat catctctctc    915900
     aagggcaaag ttccacagat ttctagggca gggacaaaat tccatcaagc ttggttttat    915960
     acattttaga gaggcatgag acatcaatca aatacattta agagacacat tggtttggtc    916020
     cagaaaggtg gaacaactca aagctagggc ttccaggcta taggtgaatt taaatatttt    916080
     ctggttgaca attggttgag tttgtctaaa gacctgggat agatagaaag gtaatgttca    916140
     ggttaagata aagattgtag agtccaaagt tcttttgaag tcttatagtg gctgccctta    916200
     gagataatag gtgacaaatg tttcctattc aaatcttagt tgaactcttt aggattggga    916260
     ggttctagaa gaaaaagatc tagctatgtt aatagagatt ctttacagat gcaaattttc    916320
     ccccacaaag aacagctttg cagggccctt tctttctttc tttctttctt tctttctttc    916380
     tttctttctt tctttctttc tttctttctt ttttagatgg agttttgctc ttgttgccca    916440
     ggctagagta taatggcacg atcttgtctc accacaacct ccacctcctg ggttcaagta    916500
     attctcctgc ctcagtctcc cgagtagcta tgattacagg catgcaccac cacacccggc    916560
     taattctgta tttttagtag acacagggtt tctccatgtt ggtgaggctg gtctcgaact    916620
     cccaacctca ggtgatccgc ccacctcagc ctcccaaagt gctgggatta caggcatgag    916680
     ccaccatgcc cggcctgcag ggccatctca gagtatggca aagaaacatg ttttggggta    916740
     aaatattttg attttcttat ttgtcttata atgttatgcc agagtcagtt tggaaagtaa    916800
     atcatgatat ataggtttaa ataaaaccca tctgatgaga atttatgatt tgtagagcat    916860
     gcctccccag actctttaga taggaatttg ggcaagatga aaaaaaaatc agagtttagt    916920
     cctcactacc taagaccagc tcagcttgga cttcactgtt catgtcacta tcagcatttt    916980
     agtcaaaacc actcaataag tctctaggaa gttccaaact ttcccacatc ttcccttctc    917040
     ctttcaagtt ctccaaactg ttccaaccgc tgccaggagg tacccagttc caaagttgct    917100
     tccagatttt gagttatctt tatagcagtt ccccactcct ggtaccaatt tactatatta    917160
     gtctgtttcc acagtgctat aaagaactgc ccgaaagtgg ttaatttgta aagaaaagag    917220
     gtttaattga ctcacagtgc tgtgtggtta gggtcggagg ctcaggaaac ttgcaatcat    917280
     ggtggaagtg gaagcaggca tgtgacacat ggcagcaggt gagagagaga aagagagaga    917340
     gagggaatga aggaggaacc accatacatg gataaaacca tcagatctca tgagaactca    917400
     ctcactatca ggagaacatg aggacagcat gggggaaacc acccccatga cccagtcacc    917460
     tcccaccagg tccacccctt gacacataag gattactatt tgagatgaga tttgtttaat    917520
     gacacagagc caaaccacat cagcatgtga caaaggtcta atatcaagaa tctatgaggg    917580
     ggcagttcca aaatggctga ataggaacag ctccagtcta cagctcccag catgagctac    917640
     acagaagaca ggtgatttct gcatttccaa ctgaggtact gggttcatct cacgggggct    917700
     tgttggacag tgggggcagg acagtgggtg cagcccacca agagtgagct gaagcagggt    917760
     gaggcattgc ctaacccagg aagtgcaagg ggtcagggaa ttcccgttcc tagccaaggg    917820
     aagcggtgat ggacggcacc tggaaaatcc ggtcactccc accctaatac tgcacttttc    917880
     caacggtctt agcaaatggc acaccaggag attatatcct atgcctggct tggaggttcc    917940
     catgcccacg gagcctcgct tattgctagc acagcagtct gagatcaaac tgcaaggtgg    918000
     cagtgaggct gggggagggg tgcccacaat tgctgaggct tgagtaagta aacaaagtgg    918060
     ctgggaagct caaactgggt ggagtccact gcagctcaag gagacctgtc tgcctctgta    918120
     gactccacct ctgggggcag ggcatagctg aacaaaaggc agcagaaacc tctgcagact    918180
     taaatgtccc tgtctgacag ctttgaagag agtagtgttt ctcccacatg gactttgaga    918240
     tctgagaatg gacagactgc ctcctcaagt gggtccctga cccccgagta gcctaactgg    918300
     gaggcaccct ccagtagggg cagactgaca ccttacacgg ctgggtgccc ctctgagatg    918360
     aagcttccag aggaattatc aggcagcaac atttgctgtt cagcaatatt cgctgttctg    918420
     cagcctctgc tgctgatacc caggaaaata gggtctgcag tagacctcca gcaaactcca    918480
     acagacctgc agctgagggt cctgactgtc agaaggaaaa ctaacaaaca gaaaggacat    918540
     ccacatgaaa accccatctg tacatcacca ttatcaaaga caaaaggtag ataaaaccac    918600
     aaagatgggg aaaaaacagg gcagaaaagc tgaaaattct aaaaatcaaa gtgcctctcc    918660
     ccctccaaag gaatgcagct cctcgccagc aatggaacaa agctggatgg agaatgactt    918720
     tgatgagttg agagaaggtt tcagatgatc aaacttctcc gagctaaagg aggaagttgg    918780
     aacccattgc aaagaagcta aaaaccttga aaaaagatca gatgagtagc taactagaat    918840
     aaccagtgta gagaagtcct taaatgacct gatggagctg aaaaccatgg tatgagaact    918900
     acgtgatgaa tgcacaagct tcagtagccg attcgatcaa ctggaagaaa gggtatcagt    918960
     gattgaagat caaatgaaag aaatgaaggg agaagagaag tttagaggaa aaaaaagtaa    919020
     aaagaaagaa acaaaccctc caagaaatat cagactatgt gaaaagacca aatctatgtc    919080
     tgattggtgt acctgaaagt gacagggaga atggaaccaa gttggaaaac actctgcagt    919140
     atattatcca gcagaacttc cccaacctag caagacaggc caacattcaa attcaggaaa    919200
     tacagagaac cccacaaaga tactcctcga gaagagcaac tccaagacac ataattgtta    919260
     gattcaccaa agttgaaatg aaggaaaaaa tattaagggc agccagagag aaaggtcggg    919320
     ttaccctcaa agggaagccc atcagactaa cagctgatct ctcagcagaa actctacaag    919380
     ccagaagaga gtgggggcca atattcaaca ttcttaaaga aaagaaattt caacccagaa    919440
     tttcatatcc atccaaacta agcttcataa gtgaaggaga aataaaatcc tttacagaca    919500
     aacaaatgct gatagatttt gtcatcacca ggcctgccct acaggagctc ctgaaggaag    919560
     cactaaacat ggaaaggaac aactggtacc agccactgca aaaacatgcc aaatcataaa    919620
     gaccaccaaa gcgaggaaga aactgcatca actaacgagc caaataacca gctaacatca    919680
     taatgacagg atcaaattca cacataacaa tattaacctt taatgtaaat gggctaaatg    919740
     ctccaattaa aagacacaga ctggcaaatt ggataaagag tcaagaccca tcagtgtgct    919800
     gtattcagga gacccatctc acatgcagag acacacatag gctcaaaata aaggcatgga    919860
     ggaagatcta ccaagcaact ggaaaacaaa aaaaggcagg agttgcaatc ctagtctctg    919920
     ataaaagaga ctttaaacca acaaagatca aaagagacga agaagaccat tacataatgg    919980
     taaagggatc aattcaacaa gaagagctaa ctatcctaaa tatatatgca tccaatacag    920040
     gagcacccag attcataaag caagtccttg gagacctaca aagagactta gattcccaca    920100
     caataataat gggagacttt aacaccccac tgtcaacatt agacagatca acgagacaga    920160
     aagttaataa ggatatccag caactgaact cggctctgca ccaagcagac ctaatagaca    920220
     tctacagaac tctccacccc aaatcaacag aatatacatt cttttcagca ccacaccaca    920280
     cctattccaa aattgaccac atagatggaa gtaaagcact cctcagcaaa tgtaaaagaa    920340
     cagaaattat aacaaactgt ctctcagagc acagtgcaat caaactagaa ctcaggatta    920400
     agaaactcac tcaaaaccac tcaactacat ggaaactgaa caacctgctc ctgaatgact    920460
     actgggtaca taatgaaacg aaggcagaaa taaagatgtt ctttgaaacc agtgagaaca    920520
     aagacacaac ataccagaat ctctgggaca cattcaaagc agagtgtaga gggaaattta    920580
     tagcactaaa tgcccacaag agaaagcagg aaaaatctaa aattgacacc ctaacatcac    920640
     aattaaaaga gctagagaag caagtgcaaa cacattcaaa agccagcaga aggcaagaca    920700
     taactaagat cagagcagaa ctgaaggaaa cagagacaca aaaaaaccct tcaaaaaatc    920760
     aatgaatcca ggagctggtt ttttgaaaag atcaacaaaa ttgatagacc actagcaaga    920820
     ctaataaaga agaaaagaga gaagaatcaa atagatgcaa taaaaaatga taaaggggat    920880
     atcaccaccg atcccacaga aatacaaact accatcagag aatactataa acacctctac    920940
     ggaaataaac tagaaaatct agaagaaatg gataaatttc tcgacacata caccatccca    921000
     agactaaacc aggaagaagt tgaatctctg aatagaccaa taacaggctc tgaaattgag    921060
     gcaataatta atagcttaac aaccaaaaaa agtccaggaa cagatggatt cacagccgaa    921120
     ttctaccaga gctacaagga ggagctggta ccattccttc tgaaactatt ccaatctata    921180
     gaaaaagagg gaatcctccc taactcattt tatgaggcca gcatcatcct aataccaaag    921240
     cctggcagag acacaacaaa aaaaaagaga attttaggcc aataaccctg atgaacatca    921300
     atgcaaaaat cctcaataaa atactggcaa accgaatcca gcagcacatc aaaaagctta    921360
     tccaccatga tcaagtgggc ttcatccctg ggatgcaagt ctggttcaac atacgcaaat    921420
     caataaacgt aatccagcat ataaacagaa ccaacgacaa aaaacacatg attatctcaa    921480
     tagatgcaga aaaggccttt gacaaaattc aacaacactt catgctaaaa actctcaata    921540
     aattagatat tgatgggacg tatctcaaaa taataagagc tatctatgac aaacccacag    921600
     ccaatatcat actgaatggg aaaaactaca agcattccct ttgaaagctg gcacaagaca    921660
     gagacaccct ctctcaccac tcctattcaa catagtgttg gaagttctgg ccagggcaat    921720
     caggcaggag aaggaaataa agggtattca attaggaaaa gaggaagtca aattgtcgct    921780
     gtttgcagat gacatgattg tatatctaga aaaccccatc gtctcagccc aaaatctcct    921840
     taagctgata agcaacttca gcaaagtctc aagatacaaa atcaatgtgc aaaaatcaca    921900
     cgcatttcta taacccaata acagacaaac agagagccaa atcatgagtg aactcccatt    921960
     cacaattgct tcaaagagaa taaaatacct tggaatccaa cttacaaggg acgtgaagga    922020
     cctcttcaag gagaactaca aaccactgct caatgaaata aaagaggata caaacaaatg    922080
     gaaaaacatt ccatgctcat gggtaggaag gatcaatatc ctgaaaatgg ccatactgcc    922140
     caaggtaatt tatagattca atgacatccc catcaagcta ccaatgactt tcttcacaga    922200
     attgggaaaa actgctttaa agttcatatg gaaccaaaaa agagcctgca atgtcaagtc    922260
     aatcctaagc caaaagaaca aagctggagg catcacgcta cctgacttca aactatacta    922320
     cgaggttaca gtaaccaaaa cagcatggta ctggtaccaa aacagagata cagaccaatg    922380
     gaacagaaca gagccctcag aaataatgcc gcatatctac aactatctga tttttggcaa    922440
     acctgacaaa aacaagaaat gggaaaacga ttccctattt aataaatggt gctgggaaaa    922500
     ctggctagcc atatgtagaa agctgaaact ggatcccttc cttacacatt atacaaaaat    922560
     taattcaaga ggattaaaga cttaaatgtt agacctaaaa ccataaaaac cctagaagaa    922620
     aacctaggca ataccattca ggacataggc atgggcaagg acttcatgtc taaaacacca    922680
     aaagcaatga caacaaaagc caaaattgac aaatgggatc taattaaact aaagagcttc    922740
     tgcacagcaa aagaaactac catcagagta aacaggcaac ctacagaatg ggagaaaatt    922800
     tttgcaatct acttatctga caaagggcta atatccagaa tctacaatga actcaaacaa    922860
     atttacaaga aaaacaaaca accccatcaa aaagtgggca aaggatatga atagacactt    922920
     ctcaaaagaa gacatttatg gagccaaaag acacatgaaa aaatgctcat catcactagc    922980
     catcagagaa atgcaaatca aaaccacaat gagataccat ctcacaccag ttagaacagc    923040
     gatcattaaa aagtcaggaa acaacaggtg ctggagagga tgtggagaaa taggaacact    923100
     tttacactgt tggtgggact gtaaactagt tcaaccattg tggaagtcag tgtggcgatt    923160
     cctcagggat ctagaactag aaataccatt tgacccagcc atcccattac tgggtatata    923220
     cccaaaggat tataaaacat gctgctataa agacacatgc acacgtatgt ttattgcggc    923280
     actattcaca atagcaaaga cttggaacca acccatatgt ccaacaatga tagactggat    923340
     taagaaaatg tggcacatat acaccatgga atactctgca gccataaaaa aggatgagtt    923400
     catgtccttt gtagggacat ggatgaagct ggaaaccatc attctcagca aactatcaca    923460
     aggacaaaaa gacaaacact gcatgttctc attcataggt gggaattgaa caatgagaac    923520
     acttggacac aggaagggga acatcacaca ccagggcctg ttgtggggtg gggggagtgg    923580
     ggagggatag cattaggaga tatacctgat gttaattgat gagtttatgg gtgcagcaca    923640
     ccaacatggc acatatatac atatgtaaca aacctgcacg ttgtgcacat gtaccctaaa    923700
     acttaaagta taataaaaaa atttttaaaa aaagaaacac ctgctttttt ctgttttcca    923760
     tttgcttagt agatttttct ccatcctttt actttgagcc tggggatgtc attgcatgtg    923820
     agatgggtct cttgaagaca gcatacattt gggtcttgct tctttctcca acttggcaat    923880
     tctctgccct ttaattgggg catttagccc atttacattc aaggttaata ttgatatgtg    923940
     catatttcat cctgttatca tgttgttagc tgctcaatat gcagatttga ttgtatagtt    924000
     gatttatagt ggcaatcgtt atgtacttaa gtgtgttttt gtggtggcca gtaacgttct    924060
     tccattatca tatttagcaa tcccttaagg gcctcttgta aggcaatcta gtggtgatga    924120
     atacccttag catttgcttg tctgaaaagg atcttatttc tccttcactt gtgaagcttc    924180
     gtttggctag atatgaaatt cttgcttgga atttcttttc tttaagaatg ctgaatatag    924240
     gcccccaatc tcttctggat tgtacagttt ctgctgaaac ctccattgtt agcctcattg    924300
     ggttcccttt gtatgtgacc tgaaccttct ttctagctgc ctctaatatt ttttttcctt    924360
     tcaaccttta agagtctgat gtctgatggc tatatgtctt agggatggtt gtcatgtata    924420
     atatcatgca gaggttattt gcatttcttg aatttgaatg ttggcctctc tggtgaggtt    924480
     ggagaaattt tcatggagga tagcctgaaa tgtttttcaa gtttcttttt ttctctttct    924540
     cttcttaagg gataccaatg tgtcatagat ctggtctttt tacataattg cacatttctc    924600
     tgaggtttta tgccttcttt tttattcttt gttctttatt tttgtctgac tgagttaatt    924660
     cagagaatca gtttttaagc tctgtgattc tttcctcagc ttggtctatt ctgctgttaa    924720
     tacttgtaat tgtattctga aattcttgaa gtgagttttt tagctctatc aaatcagttt    924780
     ggttctttct taaaatggcc atttcatctt tcagcttctg tatcatttta ctttatttct    924840
     tagctccctt ggattgggtt tcaacattct cctgaatctc agtgatcttc tttcctgtgc    924900
     atattctgaa ttctatgtct gtcatttcag ccatttcagt caggttaaga accattgctg    924960
     ggaaaccagt gtgattattt ggaggtaaga agacactctg gattttagag ttgcagagtt    925020
     tcttgcatta attctttctc atctttgtgg gctgtttctt taatctttga agtggctgtc    925080
     ctttggatgt ttttgtcttt tttgttgttt tttggtgtgt gtttttgttt gtttgttcat    925140
     ttgtttgttt tttgctctta tcttctttga tactcttgca ggtttgattg tggtataaag    925200
     tggattcagt tagcagtgtt tcttgaaaat cttagagagt ccaggctcac ctcagcactc    925260
     ttgtggtgtg ttctctgctc tgggactggg cccctggctt tattctctgg ccccttgagt    925320
     ttagaaactt gctgcattgg aggggctgag gtgttcccag tccattggcc acaacactat    925380
     agtagggggt gccggccaaa gcacttcatt agagtggtgg cagtgggatc cattcttact    925440
     catgggtgcc agcagttgtg gagtcatggc agggtgcaca tgcatctgct ggggtggggg    925500
     tactggcagg agcagagtgg cagcatccct acataggttc ctgctggcag tcacagcgca    925560
     gtgaggtgtc cgtgtgttgg cagggacagg gtggtggggc acacatgcac atgcttgctg    925620
     gtggtagagg gagttgtgat ctgctgtgca ctcatgccag caaagcagtt gggaggtact    925680
     atgggtggac tggtgcacat cagcagaggc tggcctgctg gaggtctcca atggttaggc    925740
     atggtctgct ggcaaaggag ctatgatgag ggcccccagg aaacaccctg gttgggcttc    925800
     caaggctgta ctgcaagcag gcacagccag cctggggccc caggagaggc cagaaggcaa    925860
     ggaaattctc atttcagatg ggccctgtcc catggacaag accaccctgc tttattcagg    925920
     tcccatagtc actctaaggt taaaatctcc tagaggaggt tggtgagcct tgggggatgg    925980
     gtgtcccctg gctgtgctcc actacagcca ttctcatgtc aaacactctg ggctttacac    926040
     agactggagt cctgcccctg gcatctctct aagcagctgt cccttccagc acaagtgtcc    926100
     atgggggtca tggggtctcc tgctgctagg attctggagg cccatggcaa cagcaggcca    926160
     ctcctcacct gttcaactca acctttcccc aggagttgct gggagccagg aatgagtcct    926220
     ggtgcttggc atccccatgc agggttccca tcttcctcca ccttcagctc agcatctgtg    926280
     tcctccccca tctactctca atccactctc aatgcctccc cttcaaagat ctgcttggaa    926340
     agcacccgtc ttcctgatgt ctcactccct ccatgacaga tattcctcct ggctgcatct    926400
     agtcagccat cttgactcac ctctgaagtc tttttaatta ccacttcggt taaattagta    926460
     actatcattt tacaatggcc tgtgattctg ttttgatcaa atattttgag ccttttagca    926520
     tctataacaa atgttctcaa aaatcaaaat cctaaatcaa gtctctgact tagtcttatt    926580
     tctggggctt attaaggcta taaaaactaa tcaccataag gttgtacaag ctttttacag    926640
     cttccagtca ggctatgaac tccagtatca ccacctccag cctcataatt acatatattg    926700
     gaagaaaatc agttaaagga ctccctctag acccttgaaa gggtgtgaga gacaacatgg    926760
     tttcgcctgc cttcatgtgt cccagtccat ccctgtggct gcctctgtcc acctcagctt    926820
     gcccactgtc tttccttccc aactgtctgc cctgctgact tctggcttca gtgacagatg    926880
     caaagacaag gtgacagccc cacatagacc atttaaccag tcccacattt gcataagcta    926940
     aatggtcatg taacagtctg ttgcccaggc tggtctcaaa ctcctgggct caagtgatcc    927000
     acccaccttg acccccaagg tgctgggatt acaggcttga gccacagtgc ccagccaaga    927060
     acccgttttt gagtgggcac cttggcacac acctgtaaac gcaacacttt gggaggccaa    927120
     ggtgggagtg tggcttgagg ccaggaggtt gaggttgcag tgagctatga tggcaccacc    927180
     tcactccagc gtgggtggca gagtgagatc ctttcgaaaa aaaaaaaaaa aaaacttgtt    927240
     ttctctgcag ccgggctccg tgaccaaaca caaacacaaa cttcccctcc agagggtcca    927300
     ggaggggctg ggctgcagga ggtgcttagg gcctcttagg gaatggtaag tgaccaccca    927360
     acgcaggcac tcagccccag gggcatatgc agagagaggg tccaggagga gctgggctgc    927420
     aggaggtgct tagggcctct tagggaatgg taagtgacca tccaacgcag gcactcagcc    927480
     ccaggggcat atgcagagag aggctgggag gacactttca gtgactgggg ttacaaaccc    927540
     caaccataag acattgctgg ctctgtgagc cgccacctcc agaaatctcc cacttagttc    927600
     ttagcactta tccactcttc ccttttccta ctctcaattc ctggaggatg ccctcctttc    927660
     tcaggctcag accaacctac cagctccact ctagacctga acacataact cctccctctg    927720
     tctccacctg gaaatctcat cagtgcctca catttacact cctgaaaatc aggtcctgcc    927780
     tacccaccct cttgctccac ctgattcctg ccctgtttca gccagagacc ttgcagtctc    927840
     ctttaactct caaacccacc catgtcgtgt gagcatactg actgtgttct atgcaagaaa    927900
     gagcagtttc ttggtggtcc tgcggtttta ttagtccaga ggcaaagcgt tggcagagct    927960
     ggtttcttct gaaccctggg agggagattc tgttttcatg ccttttccag attctagaac    928020
     ccatattcct tgctctgtgt ccccttcttc catcttcaaa ggccatcctc tcatctctgt    928080
     gtccatcatc acatcaccct tcccctgact ctggttctcc tgcttccact tataagcacc    928140
     cctgtgatta catcataccc acccagacaa tgcagggcca tattctcccc tctcgagatt    928200
     aatttaatca catctacaaa gttcctcgtg ccatatgagg tcactaaacc acatgttctg    928260
     ggggtttgaa tgtaaacatt tgggggatgc attattcagc cacccacaag cactgctccc    928320
     cactggccac acactatgca cagccgagat catgcaagtg aggcacgttc atcaacagca    928380
     gcttcagcag gaaactatat gctccacttt cctgccattt gtatctggat ttttttttcg    928440
     ctatcattgt agaaagagtg gtattgtaaa attaaagatg gattattttc tttctagaac    928500
     actttggcaa tctatccaac attatttatc cccttctgag tgtcaagtgt gaggtcattc    928560
     tttcattgag agctcaatgc ctacaattat gataatgcat attgggtact ttcacacatc    928620
     agaaagttct tctttcttaa aatctgttct tgaattattc attcttctct agctttttgt    928680
     tgatctattt tataatttta gaaaaatcag aaagtaactt gaagtatctg tcatctctac    928740
     aggtttacct ccctctttgt ggccttcaga atgtcatgac acgcttttcc cttgctcatc    928800
     acatggtttc tatgtatgag acctcatcac aggagctgtg gtcccccggg agcaggcatc    928860
     tgtggatggt gccttgctcc tggctgctgg gacctgtgtg ctgccagtgg cactccacga    928920
     cggtgatttc ccagctcagt tttgcagctc cagatggtgg gtgagacact aggaccactt    928980
     tgtgaacagc gagggcttgg ggtttgcttt tctaccatgt ccagggctgc tgttcatgag    929040
     ggaatgtttc taacctgaca tcatggctga agccaactta gaacctctct agccgtatgg    929100
     ggagtaggtg agtgatacag atgttaatta gctcagtgga gccactcccc tatgtagaca    929160
     tgttacaaaa cattatgctg tacagaataa atataggtca tttttatgtg tcaatcaaaa    929220
     gagaaactaa ttatttaaaa aaaaaaaaac ctctctactc aagccgaaac ctcagctcca    929280
     gtcccacaag tcacacaagg ctgctcccgt cctgtgtatg ttaaacctac ctcagaaatg    929340
     caaggggcat tcaggtttca ttctcaattc aaatgccctt tttaattttg tctattccta    929400
     gcacctggca acttccagct ctttttttcg ggctcattca ttatttaaag cacgtataat    929460
     ttttcaccca cattctaaca catgtagtac tgtagagaat ccttccctag gaggatctac    929520
     agcattagaa aagaattaag aactccaata tttacaagaa ggaaaaagca aaaagagatc    929580
     aaaaaatggg caacttctag aaatagaaaa ccctcatgag tatgatgata aatcgctggc    929640
     acacatgtga atagttactt gatgcttata gtgatgtctg ggaaaataac atgaaatact    929700
     tataatctgt ttctcacaca tgtaattcaa aagaatagag agaagatgat ttgaaatatt    929760
     cttaagtttg taggaaaaaa gctacttcca tatgcataat tgcatgtatt ttgatactgc    929820
     cattattaag aactatctaa gagggtccat taaaaataaa atttcttggc tgggcacagt    929880
     ggctcatgcc tgtatcccag cactttggaa ggccgaggca ggtggctcac ctgaggtcag    929940
     aagttcaaaa ccagcctggc caacatggtg aaacgctgta tctaccaaaa atacaaaaat    930000
     tagctgggca tggtggcatg cacctgtagt ctgagctact tggggggctg aggcaggata    930060
     atcgtttgaa cccaagaggt gtaggttgca gtgagctaag atcataccac cgcactccag    930120
     cctgacagac aaagcaagac tctgtctcaa aaatattaaa ataaaatttc tcattcctat    930180
     tacagagtaa tttaattcat taatgccctg ccctgttaca aaactcattt gtaaaatact    930240
     aattgtaatt gtgaaaaaat ggcaattgat actaatttta aattctaaaa acagggcacc    930300
     catattaaag attattctgc agtaagagaa ttagctataa cattttgtaa taaggtggag    930360
     aaaacattct ccaacttaca atggttggtg agaagaaagt ttccagcacg gtagatggac    930420
     cctaagagcc cttgttgaaa taataagaca aaaagatata cagagagata agccagatga    930480
     agggagacag agagagagag agagagagag agagagaggc acagaaatga gagatacaaa    930540
     gtgaaagagg gcaacctgtg gggtcatcag acagacacag gggaagagga gaaacacagg    930600
     gcggtggctt gcccttggac tgttcttagt tcctcaaaac gtaacagctt tgcccaacct    930660
     aagggaactt tcagcagctg ctcttctgcc ataggcctct ttcctgcctt gttttcatgt    930720
     ggctgactgt ttctgttcct gcaggtctta gctcatcaga caggcattta ttacctctgt    930780
     gtcaacagtg ggagcttcca ttactctcta gcatgacact ccccttcctc ttttaggaaa    930840
     tttaacatgg aagtgagttt gccatcggcc ttctccccac agtgttaaca gtggtgagga    930900
     agccagcctg ttccaccttg cccctcccat gattccaaca ctgagttcag acttgtcaca    930960
     tggaacttat ctttgcatgt ttgtggcaca gacagatgga cccaaccatg gattagtgga    931020
     tggatggatg gatggatgga tggatggatg gatagatgga tggatggatg gctgagtagg    931080
     tgtgtggatg gaagagtgaa aagatagatg gatgcatgta tgggtggatg ggtaggttga    931140
     tggatgcatg gatgggtgga tggatgggtg agtggatgaa tgggtgggta ggtgggtggc    931200
     tacatgcatg gatgagtact tggatagata agtgagtgga tggatggatg gatggatgaa    931260
     tgggtatgtg aagggatgga tgtattagag tgggtaatta ggcaggcatg agctgatagt    931320
     caagggattg ttaaactgcc tctctaaaat aattattggt ctcggctgga cgcggtggct    931380
     catgtctgta atcccaacac tttgggaggc tgaggtgggc ggatcacaag gtcaagagat    931440
     tgagaccatc ctgaccaaca tggtgaaacc ctgtctttac tgaaaataca aaaattagct    931500
     gggcgtggtg gcgtgcatct atagttccag ctactcggga ggctgaggca ggagaattgc    931560
     ttgaacctgg gaggaagagg ctgcagtgag ctgagattgt gccactgcac tccagcctgg    931620
     tgacagagca aagctctgtc tcaaataata ataataataa taaataataa ttgatctcag    931680
     ccagcgccaa gaaaaggcag tctcccaata gatagaaaac acccgaaact ggtcatcagc    931740
     agcttcctga taagatctca ggcattgggt gagtgggctc aagcatatgc actaagaggc    931800
     aaagtggcag agtttaactg gcacataatc ttcctctagg aacactctaa tagtaagaga    931860
     aggacacctc aaatgagcat gtgcacattt cattaaaccc actgtgtatg cagcccctcc    931920
     caagtgctgg caggccactg tacatgtggg cagcccactc caagggaaga atcaagggag    931980
     aagaaataca aatcccagaa ccatgtcaat gtataaaacc ccaagtcaag ggccggacag    932040
     agcacttaga tctctcaagt cgcccactta gccctcttcc aagtgtactt tacttccttt    932100
     agttcccact ttaaaacttt aataaacatt tactcctgct ctaaaacttg cttgggtctc    932160
     tcactcttct gtatgcccct tggccaaatt ctttcctcca aggaggcgag aatcaagttg    932220
     ctgcagacct gtatggattc gctcctgcta acagatagct ggatgggtgg acagatgcat    932280
     gaattagtgg atggacgttt ggatgtgtgg gtgggtgggt ggattgtggg atggctggat    932340
     gaatgcatgg ctggatgggt ggacacatgc atgaattagt ggatggatgt ttggatgtgt    932400
     gggtgggtgg gtggattgtg ggatggctgg atgaatgcat ggctggatgg gtggacagat    932460
     gcatgaattc gtggatggac gtttggatgt gtgggtgggt gggtggattg tgggatggct    932520
     ggatgaatgc atggctggat gggtggacag atgcatgaat tcgtggatgg acgtttggat    932580
     gtgtgggtgg gtgggtggat tgtgggatgg ctggatgaat gcatggctgg atgggtggac    932640
     agatgcatga attcgtggat ggacgtttgg atgtgtgggt gggtgggtgg attgtgggat    932700
     ggctggatga atgcatggct gggtgggtgg atggatgcat ggataagtgg tggacggatg    932760
     gacgggtgag tggatgggtg gatgtgtgtg tggatgggtg gataggaaag ccctctaatt    932820
     gattacaggg ctcagtgtgt gcttcaacat catgatggca tcatcacatt ggtccctgta    932880
     tgaagcagtg ggggaggaga gtgtaccagg ggagcaggaa tgacttttct ccagaatcga    932940
     cctctcccac cctgcagcct gggctgtgca ggccacattg gagaaggtgc ggtcgactac    933000
     tcctaaatgt tgttgtgtcc aatggcttgt tgacgttgat gtaggaatga gcctacatct    933060
     ccaccataga tggaactgtt tgggtcccca aagcagaaag cctcttctgt tgcaggtgct    933120
     gaagtttcca tcttcttctg cttatacgga agctcacgca tcccttgggt ggcaggcgtc    933180
     aggttcctgt gcgcactgag ttcccccctt acatgctttg gacagaagtg tgagacacac    933240
     aagattgctg caggaagtcc acctgtgggg atgctgcgac ttctccagca agaacacgag    933300
     tctgctcatt gaccatcacc acacataaca aattaagtgt cccttttttg ataacacgtc    933360
     attgtttcac agagtattct tttaaagtgt ataagttgac tgcagttatt attttttact    933420
     tctgttacta atttactcat aattaggcac aatttacact taagaaattt cttaatagtt    933480
     ttttcctcct taaggtgaac tacagtcaga taacatactt atcaattgtc tctagctctt    933540
     gtcagaaaaa catatagatg tgtgtgtgcg tgtgtcttgg cctttccaat gatgaattaa    933600
     gatgtgcatt gagaaggcat tcactttatt tgacgttaag gaagtaccaa gaagacgctc    933660
     tccacagacc ctgggaaagc cagcagctgc accccgaggc tgtgccaggc agggaacaag    933720
     gaggcagcac cacctgctgg gcagggaaaa tgtcctccca gtccctgccg cttctctgca    933780
     gaggcacaaa gagctgcccc ttctcctggg ccttctcctg ggctgatgag attgctcccc    933840
     gatatgccaa atcagggttg tgcatctgag gctctgtcta gactctcagc tccttcctac    933900
     tcctgcaaag tgaagaaaac aatgccaagg ggtcctggag gcgtctctac ccctggagag    933960
     ttttgactct cttcaatagt ctccactacc ctgccctcac tccatgtcct ccgtttctcc    934020
     ctaaagcggt gcccagtctg attgcactgt ggcagggata acgaggggcc aggacatcag    934080
     gggagagaag tttctacctg agtcacagca gcggctgccc tgcagactcc tgaagacaca    934140
     agacacattt ccatcccaga gacccagcga aatgcaacct caaactagag acagccagtt    934200
     attttttctt gttctgtcct ggagaggcca ctgagaaagt cgagcccctt gttgaggaaa    934260
     acatgagatc tctgtgtgtc gtcctctgcc tgatggctgt acctccatgt gagtgtctca    934320
     gagatttcag aacgggggct gtgggctgtg gtgtccgctt gtgactcatc tctttgcttc    934380
     ttgtccctga gtgtcctgca tcagatgcag ctactggagt catgcccagg gctggtgagg    934440
     tcctcacaga cctctgggcc tggacccagc agccctctgg gaaggcgctg gggcacctca    934500
     gctccagggg cagcacacac ttcagcccag cctttctggg ccaactctcc atctgtagag    934560
     acacatccaa ggcccagtta tccctgcagc tgagctccgt gatggccaag ggcagggccg    934620
     cacattcccg tgggagacag aatggggacc tcagcgtgag cccagacaca aacctccctg    934680
     cagggaagca caagaccacc aggcggcgct ccagaccaca cagcggcccc agaagcaggt    934740
     tttagggggc ggggcagacg tgtccgcgtt gagtcaggtc attggtttta ctttccctga    934800
     gcaaacggcc tctgccaagg actcactgca cctctcacct tcacagttgt tttttttttt    934860
     tttttttaat caccctgtag agttttgcta gctaatttag atattgagga gtgcttcata    934920
     cttccttggg cctctgcttg cagaaacata gcaattgtaa ggaggcacgt gggaaagccc    934980
     cggctcggtg acccggggga tgctgctgtg gccctggcaa gagggcgtcg ggccgcagta    935040
     acaaaggtgc agacggctct cagcctgcgc ccgcggagta caacacataa gggctgtaac    935100
     ctaacgaaaa aagaatcgca gtgcaactgt cctgcatttg agtttgtgat cagttttgcc    935160
     ctttgtcttt aacaggttct aacataaaat tttgaatgtt ggttcaagcc ctgtgggtaa    935220
     aatgcactta cccacattcc ttaaacaaat agaacactga ggtggaaatg ttttgaaaaa    935280
     gtagttttca gacatttgga aacaagcatc acaggatcat aacccctgag aaaagaaaaa    935340
     caaatgaacg aatcctgcta ttgcctgaaa gcagctgcca ggacacacgg aaaggcttag    935400
     tgagctgagc ggacagagag cagagttcaa ggcagcagca gcccgagggg aggagcaccg    935460
     gggagcaggc tgctgtgcag ccaggatggg ccggggtggg gcggggggag aacagctgga    935520
     gacttgccgc agggaggggg atccctcagg tttggggctg agaactgact tatgcctgac    935580
     ttatgcctgc atgaaaagaa actactcgat atcaggggga aatcaccaga aacctgtgga    935640
     cccaaaacta cacagagcct acacaaggaa agcattgttt gtgttctccc agccagggtg    935700
     gaaagacctt gagatatgta aagcttcaag caatcttccg aagtaatctc gtgagtagtg    935760
     gtgccacatt aattcaggac taaaggctgc tctgaactga acctaagaaa tgcttcaagt    935820
     gtagcctgga gcccgggtgc agtggctcac acctgtaatc ccagcactgt gggaggccga    935880
     ggcaggcgga tcacttgagg tcaggacttt gagaccagcc tggccaacat ggcaaaacct    935940
     gtctctacta aaaacacaaa aattagctgg gcgtggtggc agatgcctgt aatcacctcc    936000
     cacctggacc cttccttgat acatcagaat tacaactaga gatgagattg gggtggggac    936060
     acagagccaa accgtatcac ataggaacct aaaaggataa taaagtagga aaacttccca    936120
     catcagtaac cctttatccg atagtaatcc caatctgcaa agtaaaactg tgtgatttta    936180
     ctaagataac ggaatcttct ctacaggact ttccagtgca aaagctcccc accctcacca    936240
     tgaaatgcac gtgaccattt ccaatttgtg taaagtcctc agttagtact gagacttcgg    936300
     aaggttagaa atccctttgc tcatgctgca tggtccggat gagatgtaag aatcattagc    936360
     taatagacat gcaacagctt ttgtgtgaaa gatgttatga gacatttaag gtatttgctt    936420
     gtgattacta agcattcatt gtatcattgg agcacatgtg cttttatacc ctggagaaat    936480
     tccagtaatt gaattgctgg gttgaatggg attttgattt ggattaaatt taaactatag    936540
     attttattta gggaaaactg gcatcttaat tatgttattg gggggccctt gctcccagag    936600
     ctcccaagat ggtggcaggc cgcttccaaa atgaccgcag gccacttcca agatggtggc    936660
     aagcctcatg ttctctgacc tggggttctt ggcctcacgg attccaagga atggaagctt    936720
     gggccatgca gtgagtgtta tagctctatt agaagccgtg ggtcacggaa gagaaccgtg    936780
     gaacccagtg actagtgttc agctcgatta ggacgaaccc aggcacttag ccgtgcagga    936840
     acaatggcga gcatttggtc cgatcgagag tggcaatggg cgcctcgctg gatcaggagc    936900
     acagcggata ccctgatgga tccggaggga tggaagccag cggtgggtct cccacggggg    936960
     caaacagcag tggtggacgg tgagcgaaag cgaagctcga gccgtaacaa acatggacca    937020
     gaagagtgca gttgcaagat ttagtagagt gaagacagag ctcccataca aagggagggg    937080
     acccaaagag ggtagctgtt accggctcga atgcctgggt ttatatcccg atcattgtcc    937140
     ctcccgctgt gctctcaggt gatagatgat tggctatttc tttacctcct gcttttgcct    937200
     aattagcatt ttagtgagct ctctttacta tctgattggt cgggtgtgag ctgagttgca    937260
     agccccgtgt ttaaaggtgg aagtggtcac cttcccagct gggcttaggg attcttagtc    937320
     ggcctaggaa atccagctag tcctgtctct caattacact gagttttcca atccatgcat    937380
     ccaatatgtg gtgtatctct tcatatgttc atagcctctg agcaatgttt tacaattttc    937440
     tgtgtaaaga actccacatc gttttatgtt tcttctaagg tatatcctga ttgcttttta    937500
     tgtcttcaca agttttttcc tttcaaaatt aattttccaa ttgtttggtg ctaatatgct    937560
     caaatgtcct tgattttctt agtttgaaca gtccgttttt gttttgggga tttatttttt    937620
     tttcagattc tttaagattt tctatgtcta taaccatata atctctgaac agagacagtt    937680
     ttgctttttc ctttcaactt gaggtaggtt ttctgggtag ttcaggacgc gcaggcactg    937740
     ggtgggtggt gttagcagct gcacggtgcc ttggagagga cactctcggg ggactgtggc    937800
     cgctgctcag ctgtgaccgt tcttatagca ccagcagctg cggccaccat tcttatccaa    937860
     tttccaaagc cacaccacag gccctctcaa gaacgaggcg tggaggctat gccctctcct    937920
     ggacacatca tcattcccaa gccccacgat gtgggcccca tgggacgcac acctttgtct    937980
     gtccagacct cagccccacc tcctcatcct gcaccagaac tcttcagagc ccagtgcatg    938040
     aaatgggcta ccaaggaaat gagggtaggt tcctgagagg aaactggccc tgcatttggg    938100
     agctaggagt ctgctaattc gcctggcagc actgtgcagc cctccgtggc tacagtccac    938160
     cccgtgccca tcagtgcctc cttcctgtgc aagcctggac ctcgccctgg gctcaggatg    938220
     ggctgtagac cgagaatgca ggcgggaaag tctttgtcta tcggggccat agtcaggttc    938280
     tacagtgagt cagggaaagg cctgtggagg tgtggatgag gacaatgggt ccaccatcaa    938340
     caggaggaca caggttcgac cccttgcaga ggcacagtcc cacatcactg ggaggcagcc    938400
     acactcactg cctcgccctc tcctcacaca gtgcagtttc catgttcaca gccccagcca    938460
     gtcaccagga atgccctggg ggcggccttt ccccagtgca ctccgagccc tcccttggct    938520
     gtgcggtgag ctccatgccc aggagatatc cacccatagt cctccggaaa gcagctgacc    938580
     tgccatgccc tggaaccaca aatccccaca gatcagccag cctgcagtgg gccttggatg    938640
     tggtgaggag tggtggcacc cccgttccca ccccacagat gcaacgcctg tgggtgacgc    938700
     atgtgagtac tgaggagtag agggtagaac tgtaggcccc gagaaccaca gaaactcggg    938760
     tgttacactc tggggccatg taaggagaaa gtgtcactgg acagaaacag gcccctccta    938820
     gacactgtgt gcgccatagt cacctgtcat tagctctcac tcttgcagat tcatgattga    938880
     ggtggttaaa aaaaaaaaag ctcctactca cccatccaac cccatcctgg ggtgtttcca    938940
     ccacccttgg ggtttgggat gagctgccct tgcccactgt gctctgtgga cctcccttta    939000
     gaagctcaca gctccctgca ctcggctcca tcctgcccca ccacacagaa gcaaaacccc    939060
     tctcctttcc actgcaggct tttcctggac cagaatgctg acctgctgcc cttcactccc    939120
     gaagtggtgg gactgcctgg ggtggtgtgg gtgttgagcc ttcttactct agggacctgg    939180
     cacctggccc caggggcaca gggatggtgc atctgcctag ggatgcctcc tcatgccagg    939240
     gggtgggggt tagtaccatc ggccctcagg atttgttgca tgaatgagtg aatgggtgaa    939300
     taaatgaagg ggatctgatc tatgaataag ggtatataga ctttggttga tgtaggacgc    939360
     caaatgctgg aatttcagag tcatcacacc caggggccct gcctctgagc tcctctttgc    939420
     atccaatctg ctgaagaaca tggctctagg gaaacccagt tgtagacctg agggccccgg    939480
     ctcttcaatg agccatctcc gtcccggggc cttatatcag caagtgacgc acacaggcaa    939540
     atgccagggt gtggtttcct gtttaaatgt agcctccccc gctgcagaac tgcagagcct    939600
     gctgaattct ggctgaccag ggcagtcacc agagctccag acaatgtctg tctccttcct    939660
     catcttcctg cccgtgctgg gcctcccatg gggtcagtgt cagggagatg ccgtattcac    939720
     agcagcattc acagactgag gggtgtttca ctttgctgtt tccttttgtc tccaggtgtc    939780
     ctgtcacagg tacagctgca gcagtcaggt ccaggactgg tgaagccctc gcagaccctc    939840
     tcactcacct gtgccatctc cggggacagt gtctctagca acagtgctgc ttggaactgg    939900
     atcaggcagt ccccatcgag aggccttgag tggctgggaa ggacatacta caggtccaag    939960
     tggtataatg attatgcagt atctgtgaaa agtcgaataa ccatcaaccc agacacatcc    940020
     aagaaccagt tctccctgca gctgaactct gtgactcccg aggacacggc tgtgtattac    940080
     tgtgcaagag acacagtgag gggaagtcag tgtgagccca gacacaaacc tccctgcagg    940140
     gatgctcagg accccagaag gcacccagca ctaccagcgc agggcccaga ccaggagcag    940200
     gtgtggagtt aagcaaaaat ggaacttctt gctgtgtctt aaactgttgt tgtttttttt    940260
     ttttttttgg ctcagcaaca gagatcatag aaaacccttt ttcatatttt tgaaatctgt    940320
     tcttagtcta atggagattc tctaatatgt gacaatgttt ttctcttgct gtttttggaa    940380
     ttctttgtct ttgacttttg acaacttgac ttttgacagt gtgcctcaaa gaagttctat    940440
     tttgggttct gtgaacctcc tggatctggg aagttttcag ctatgatttc attaaacgtg    940500
     ttttctacac catttcccta ctcttttgga atacccataa tgcaaatatt tgttcactta    940560
     attgtgtccc ataaatgctg gggattttct tcattccttt ttactctttt tttcttttta    940620
     ttcatctgcc tgaattattt caaaagatct gtcttcaact tcagaaactc ttttgcttgg    940680
     cctagtctaa tcttgaaggt ctcaattgta cttttaattt cattcattga attcttcaac    940740
     tctggaattt ctgttggttc ttttttatga tacttatctc tttgttgaat tcctcattca    940800
     aatgataaat tgttttcctg atttcactga attttctatc tgtacactat tgtatctccc    940860
     tgagtttctt agagattatc cttttgaatt atttttctga cattctgtat atttccttat    940920
     gattggggtc tgctactgga gaatgactgt tgtctttttc aggtgtcgtg tttcctggcc    940980
     ttttcatgtt ttatgtgttc ctacgttgat ttctacacat ctggcggacc agtcatccct    941040
     tgcaatttaa tggagtaggt tttgcaggaa aagacttcct agtacagacg ggtctcaggg    941100
     tgtcagtgtg gcggggcgtg ctggctttag ttctaggttg acgcagtagc gtagtctcca    941160
     tgtcgtttct tcagctgccg tccacattgg tgacgtttgc gagtgtctca gtggcctggg    941220
     ctgagaggtt tgtggcagtg gaagtgcaac gttgctagag gtggactcac caggctgttt    941280
     ctgaggtcgg ggcacatgca tgcacatggt ggattgacca acttggtgcc aggctcacta    941340
     gggttgggga catggggctg tttctcaggc ccaggatgca aacacaagtc tctttggctg    941400
     gcctgggggt gtggcttctg agggcaatcc acagggctgt ttctcaggtt caggacacaa    941460
     gtgcatggcc gctcaactgg cctgggcatg tgtctcccag ggccacccca tgggctcttt    941520
     ctcagaccca ggacatggcc acatggcttc ctcagctggc ctgggtgtgt gtctgctggg    941580
     gggctgcagg ggcacagggt tatttctcag gccggggtca tgggcgcaca gctgcttgct    941640
     ggcttatagg agtgcctgcc aggggtggcc catgatgctg tttctcaggc ctaatttcag    941700
     gtgcagagcc tttgggcagg tcaacggcat acctgtggaa attggagtgg atgccacagg    941760
     gctatttctc aggtgcctga gtgtgggcac atatccactc tgccagcctg gagttagtat    941820
     caggtgctcg gtggctcagg ggcctctcct gctcagggga gggccctcag cagcttggcc    941880
     aaatcaatgg tggattcacc ctgggcaggc ctggcaggct cttcctccag ctggatgtgc    941940
     agcagcaggg gttgggtttt ttgctgtgca gggccagagt cacggccaat cctcagccta    942000
     ggctctgcac agccagggtt gtggcattca gccacccaga tatgggcatc ctgaagatgg    942060
     agccccaatg ctagaaaggg gcagtggcta ccagcctcag ggcaggatgc actccagagg    942120
     cggctccggt ctcaaggtgg cgctgggctg cagcagctag gctcacagtg gatgaatggg    942180
     ggcagggagt acacaccttg tgctcctaat ctgggatcat tcctggcagc tcccaaactt    942240
     ggctgagggc ttgcaaaacc tgtggaattc tcctgttgca agggctgtag atgtttgcag    942300
     tggcagtggg tgctggcggg aaatctgctt accttttccc tacatgggaa gtccctcctg    942360
     tgtccagacc aatccgatct gggtggggaa gacaaggctg caaaggccag gtgcctccat    942420
     gctgccctcc gatcaccacg ggtgcgtttc cacacctcca ctgcactccg tcagtctccc    942480
     ttcaacactc cagtcaaacc ttagctgttt cttctttgcc ttattccttc ctcatgggga    942540
     gggtgtgggt gaacaccagg cttctctaag ttcttcatcc atcttgctga tgtcattctc    942600
     catccaggca tgggttttta agaagtagtg aatactgaaa tttcagcaga ggacacctct    942660
     ataaaaattc tgcaactgga aaacctcctt aaattggctg attgtcatta caattggagg    942720
     aaaactgcca ataatttcaa atttagaagg ctgagactct ataaacaaag actaacaata    942780
     tgttttctga tatttttccc caaaataata cttttccaag acgaaaattt ttccagggta    942840
     tataagcaca tgtgctccaa tgatacaaca aatacttact aatcataagc aaatacctta    942900
     aatgtcttat aacatctttc acacaaaagc tgttgcatgt ctattagcta aaaattctta    942960
     tgtctcgtcc agatcatgca gcatgagcaa agggattttt gaccttcaaa agtctcagta    943020
     ctaactgagg actttacaca aattggaaat ggtcacctgc atttcatggt ggtggtgggg    943080
     agcttttgca ctggaaaatc cttctgtaga gaagattcca ttatcttggt aaaattacat    943140
     agttttattt tgcagattgg gattaccaac tgataaaggg ttactgatgt ggaagttttc    943200
     ctactttatt ctcctattag gttcctatgt gatatggttt ggctctgtgt ccccacccca    943260
     atctcatctc caattgtaat tcccatgtgt ccagggaggg tccaggtggg agtgattaga    943320
     tcaagggtgg tttttcccag gctgttttca tgatagggtg ttatcatgag atatgatggt    943380
     ttaaaagtgg caggttcccc tgctctctct ctcgcctgct gccacataag acgtgccttg    943440
     ctttcccttc accttctgcc atgattgtaa gtttcctgag gcctccccag ccatgcggaa    943500
     ctatgagtca attaaacctc ctctctttat aaattaccca gtctcaggta gtatctttat    943560
     agcggtgtga aaatggacta atacactatg gctttgaatt aataatttaa aatttgtcag    943620
     cttggcaata aaacatcctg ttgacattta ttttttaggt aatattttaa attggcagtt    943680
     tcattcatgt ttttacaaat tcttattttc agggtgttta aggcctttgc tttgaacttg    943740
     gtggttcctt acactccatg ctgttagtga agagggacca ggttgggagg cattggtttg    943800
     ggtggtggtc aggaagggca gagtgatttg agtagggtct gagtggataa tagctcatca    943860
     gtttggaatt tataaatgac cagggatgat ttaaggagat tcctgccaga cacctatgcc    943920
     atggccatgc cctatctgga tctccagccg tgagatgaga acccagccat gcgggggagt    943980
     ctgttcgttc tgctcaatgt tgtaagtggc acatgctatt ggataatgta gaattgaatg    944040
     gatatcattt tattattata atttacaaac ttcctacaat aaacttatca cctttataca    944100
     tagaaacaaa tataagtaca ttttccctcc cctatgtcat tttgagcccc tctctccaaa    944160
     ccatcctccc actctgcgac ctcactgtcc ctgcatttgg ctatgctctg gcaagtccct    944220
     gcttagacaa gcactcacca gaccacccta ctcagcctcc ccttcagcgc ccacctggcc    944280
     cacctgctca aatacatgtt gagtggtcac acacatggac tgaacaccat ctattccatg    944340
     cactgcccca gtgaccgcac tgagcagcaa gagagaaatg atgcattagc tatcaaggat    944400
     gccaattcaa atgctggagt ctttctcaga tacttttcaa tgttcaagaa ttgttgattg    944460
     tgaattctat acccaatgaa actatccttc agaaatgagc agaaaatgga taccttctca    944520
     aataaacaaa aactaaaaga attcttgcta caagatgtac tcttaaagac tggctaaagg    944580
     aagttcttca aacagcaagg aaattgaatt gatcttatgt cctgcacact tgctaaattt    944640
     cctctcaatt ttagcagcac tgtttagatt ccataggatt ttccatacaa acagtcatgt    944700
     ggtctatata tagagacaga ttttcctctt ttccagtggg gataaattta tgtctttttc    944760
     tttctgtgtt acagcaggta ggacctccag tacaatgtta aacagaagtg gtgaaaacag    944820
     acattcttgc ctgtttccta acgttggagt ttggtctttt actatggtgt cagatgttag    944880
     ctgtagggtt tttataaatg cccttcatca cattgaggaa gtttgctcct atgcctaatt    944940
     ttctgagagt cttttaatgt gacactcatg ctagaattta ttaaatgctt tctgtctact    945000
     aagatgatta tgcagttctt atattaacat gaataattac atttatttat tctttaatat    945060
     caaggcaatt ttgcattcct gagacaaacc ccatttagtc atcatgtgtt gttattgtta    945120
     catattgttg gattcaattt cctcaaaatt tgttaagaat tgttacatct atgtttacaa    945180
     ggaagattag tctgtagggt attttttctt ataataactt tgcctagttt tgcaatcagg    945240
     gtaatgctgg actcacagaa tgagttggga agctatttcc tcctcttcat ttttctgaaa    945300
     gaatttgtat aaaattggaa ttatatcttc cttaaaggtt tgcaagattt cataatgaag    945360
     tcattggcct agagttttct ttgtgggaaa gtctttgttt gtttgttttg tggtttgggt    945420
     ttttttttaa gagacacagt ctcactctgt tgcccaggct ggaatgcagt ggtgtaatca    945480
     tagctcacag cagcctcaac ctcctgggct caagcaatcc tcctacctca gccttcagag    945540
     tagctgggac tacgggcatg taccaccaca cccagctgtt tgtttgtttg tttatcgctt    945600
     tgtcttgttt ttgaggtctt attatgttgc ccaggctggt cttgaactcc tggcctcaag    945660
     taatcctcat gcctcagcct cccacagtgc tggaattaca ggcatgagcc actgcacaca    945720
     gactgtggga aagtttttaa ctaaaaattc aattttctct tccttttcca gtgagctttc    945780
     cagtgtcttt caattaatgt atctatttta tctaagttgt tgaatttatt gtcaaaattt    945840
     ttttaaacaa tattcctctc ttagaggttg aacatctgta gaatctgtag tgatggcacc    945900
     tcttaaatcc ctgatcttgc tcatctgtgt cgtctctctt tctctaatca gtatgcctaa    945960
     agtttaattt cattgatttt cttaaaaaac tggttttggt tttattgatt tttttcccta    946020
     gttttttgtg ttacatttca ttgacttctg ctctgatatt tactatttcc tttctactgc    946080
     ctacagtaag tttaatttgc tattttctta gtttcctaaa gtggaagcta agtttattga    946140
     cttgaggcct ttcctctgtc tggatgcgga tatttgctgc taaacatttc cctccaacac    946200
     catgctgtga gttttagtta cagcgggctt ggagttggcc tgagaaattc tacttaaaca    946260
     gctgcaccta tcatgtaagt gataaatgat gtacctgcct ggccctcacc cctggtcaaa    946320
     gaatgggatg tactaatgag caatgttgct gcgtagctgt ggatttcaag gtattttctg    946380
     tgtggtttta tcatcagcat tgtttgttga tgactgcaag actgatgatt tgcacctggc    946440
     ctcggtgaga tccccgaaag accctgcaga tgggctggtt acttagcaga aaatatgaca    946500
     acgtggccag caggaaacag gaaggtacaa tcggctgcag gtgagctgtt ggaagtaagt    946560
     tccaattttc ctattttgta tttgcatttt aatagtgaga ctgcgcttat gttatttgtg    946620
     tgaaacagct ttattcatag cactgtaatt taaagagaaa acccattcat gggaacaaca    946680
     aacgacctag acaccaaggt agctcatgcc atccaaggct atactgtgca gtgattggga    946740
     aaatgggcac tggtcccaga agtctgatcg acactctgcc actggctagt cccgtgctgg    946800
     ggggcgagga tccacactct gccactggtt agtcccatgc tggggacaag tatccacact    946860
     ctgccactgg ctagtcccgt gctggggggc gaggatccac actctgccac tggttagtcc    946920
     catgctgggg acaagtatcc acactctgcc actggctagt cccgtgctgg ggggcaagga    946980
     tccacactct gccactgatt agtgttgtga agatttaaat aaagaaccca caccatattc    947040
     tttgacttgt gctttccgta tactgagaga tagtaagagt acattattat tatttataaa    947100
     gtaaactaga aagcacatgg gaagacaaga agaaaacctg aataaacatg aattacccca    947160
     ttttcctcag gagaaaactt tcacactctg aaggtacaca aattagccta caaatttaat    947220
     gtaaagcaaa tagactgttg taggtaccaa ttctcaatgt cacagtgtta catggaaagt    947280
     aaaatacaca agaacagccc aaaagatgga aacaatggac gtggtcaaat gacatcagta    947340
     caacatccat atggtcctaa gtagccatct ttaaaatggg ttagaaatgc cttcaatcat    947400
     tcacacagac acatgcattg aacaaactct aagaagtgtt cttacacggg aaaagcaagt    947460
     tacagatgca tgggcatgat atggatgtag atgtgtgtat gtgcatccca ctcatacaca    947520
     aaatacccag catcgcccac atgcctgctg tgtgcgtaag tgtgagcgag tgcacagaca    947580
     acagcgtgca gaaattcaaa ccaagctgtg ggtacttgtt accactggga agggagtcgg    947640
     tcacagaggg aaagagaaac aggacatcag cctttgactt cagaactgtt cctgcctttt    947700
     cacatcctgt gctgttttca gcatcgtcgg agcccttaac acacatcacg ggagtaagag    947760
     tgtgttagag ggagcattcg gtgggacaga tattgccatg gcttgtggat agagttcaca    947820
     gtccttaata atccccgaga tggcagccaa gagctacgtt ctcaatcacg cagcttcacc    947880
     ccagaaactg acagaaaccc aacaaccaaa aggtgtccat tctgacagcc tcagcctgtg    947940
     ctggctcaga tgagcaaaaa tgtacagata ttaataatga tgttgatttg aagagcacag    948000
     aggggggtat gcatgataag ggtccaaatt tttaccttaa aaaagaatac atttacttct    948060
     caatcaccta cataacgatc attttttaaa aaactgatca aatttggtgt tacaagggca    948120
     cgttgcaaat tcttctggct acttttctct gactattcta attacgttac cgtgttttct    948180
     cctgtatgtg cccgttcatg tgaatgtcat ttctggctac ttttctctga ctattctaat    948240
     tacgttaccg tgttttctcc tgtatgtgcc cgttcatgtg aatgtcattt ctggctactt    948300
     ttctctgact attctaatta cattaccgtg ttttcctgta tgtgcccgtt catgtgaatg    948360
     tcatttctgg ctacttttct ctgactattc taattacgtt accatggttt ctcctatatg    948420
     tgcccgttca tgtgaatgtc atttctggct acttttctct gactattcta attacgttac    948480
     cgtgttttct cctgtatgtg cccgttcatg tgaatgtcat ttctggctac ttttctctga    948540
     ctattctaat tacattaccg tgttttcctg tatgtgcccg ttcatgtgaa tgtcatccag    948600
     gcagatttcc caaatccggc ttcctgtaac caagggctga aagagggaac ggtttcctgg    948660
     gaatcctttt tgcagtttat tttacccgga ggcagaagcc cacggttccg tgaagagtct    948720
     attgctctcc cctctctcct tttgtgtctc tatttttaat tgacaaaaaa gcaaatgtga    948780
     agattcctgg ggtacaatgc aaagtgacaa tgcctgtcta tattgtggga tgattaaaac    948840
     aaggtaagtg gcatatccat cacctcacac acttatcatt ttgtggtgag aacatttaaa    948900
     atctcatctt ttagcaattt tgaaatagtc attattgtta actatagtca ccatgctgtg    948960
     caacagatca aaagaactga ctcctcccat cagcagaaac ttcatgccct ttgaccagca    949020
     tctctccttt ccccgtccac gactaacccc cagcccaaga gaacagccaa cacccacctc    949080
     gctgctgcca cacgacatgt cgggctttga tgggatggag gtgagggtgg ggaagacaat    949140
     tccaaagctg gagcactggc ctcacagctc agacactctt ctacttatcc tgagagaatg    949200
     atgtgctgag accaactaaa cctcccctgc tcttcccaca tggcagaaaa gaggcaaccc    949260
     agggaagcca ttgccaggac atcatggtca cccaaccctt gtgcagaaag gaagcacctg    949320
     cccaggatgc catagcaccc aaccctcatc cccaaggaaa cacagcccag ggcaccatgg    949380
     acacccaacc gtcatcccca ggggaggaca cagcccaggg caccatggac acccaaccct    949440
     catccccagg ggaggacaca gcccagggta tcttggacac ccagccctca ttcctagggg    949500
     agtacacagc ccagggcatc ttggacatcc aaccctcatc cccagggaag gacacagccc    949560
     agggcatctt ggacacccaa ccctcattcc cataagagca cacagcccag ggcatcgtgg    949620
     aggcccgacc ctcatcccta ggggaggaca cagcccaggg caccatggac acccaaccct    949680
     catccccagg ggaagacaca acccaggcca ccattaacac ccaatcatgt gcagggaggg    949740
     tgtccttgga gcctgggact cttgccagtg aagcggtgga caagaaactg aggatgcgat    949800
     cagcacacag aaatctcagg cagcctagga tacatgaggc ctctcacccc tgggaacact    949860
     gagcagccac caggagccca caccttgagg tacagcagga gccatgcgct cttgctcttg    949920
     ctcactcaca ctcctgcaca cagccactga cacacgccct cgtgcacgtt gcagattaac    949980
     tccactggcc ttgcacttgc aacgctggag gctgagaggt atccccaggt tcttttctcg    950040
     tgagaggggc aggctgactt tcactctcct ccatgtgcta gaggcagctc caccaacact    950100
     ggctgccctg agtggatgca cctggctctg gaattcctgt catttgcttt ggatccagga    950160
     gcccctgcct catgtagcta cttaacagaa aggaggaatc cacccaggac atgcccagac    950220
     gggagcctca caggatggac agtggtgtct ggggtcacgg gcagccctga cccagcagcg    950280
     ccagcaccag cacacccagt ggggaaggcg gggaggccca aacgccaccc acagtttgtt    950340
     actccactgg gtgggacccg gcacccctgc cttcctgaca ccctggagtc cctgcctctt    950400
     cctagagccc ccaagcccat ctgcctcaga gcatccagag acagacctgg ggagccattt    950460
     cctcaggccc tggacaagga aacagggaat tccaggttat gggtgcctgg ggcaggtctc    950520
     aggcaggtgc tgggaaccag agagaggggt caccgcgagg cctcaggcct ggcaccagca    950580
     ctttgagcct cagtttacca gcccacgagg tgctgagtct ggactggatg accttcccac    950640
     ccccagtgac ctctgccctt tcccgagcat gtcagctctg ctccagcatc ctggtgtgag    950700
     cgcaatgcca ctttttttct caacaaatac gaaaggagga aggtgccccc agggccctgt    950760
     gccctgagga tgcctgtgtg gaggggtcca tttcatcact ggtgtcactc acaggaaggg    950820
     acgaagccac ctgccttgac ggagcttact ccacctccgc cgaaggccgg ggaggtccct    950880
     cacagagaac ctgaggccca gcaggctgca gaggtgctgg catggaatga ctgctcagac    950940
     gcccggggcc ggcagagagg acggatgtgg gggaggtgca cactgaggag cctctccttg    951000
     gaggtggaga cacgtgcacc acatggacca ggacacagtc cacgaagcct cgcatccccc    951060
     tgagctgcag ctcaagggcc tctctctgag cccagagtcc cacccctggg aggcagctgc    951120
     cccagctctg agggaggagg gcatccacca ggccctccat ctcctggggg caccagccca    951180
     gcccagaggc tctgcaggac tctgcacctc caattcatgg ccaggacttt ctggatgtat    951240
     cttaaggact gaggactcca catcagggac cacacaagac cggggtcccg gacacggggg    951300
     ttgggggtga gcatgtcacc gggatgggct gtggcgtcac tctggtactt catccggaca    951360
     gccagggacc aaagccacgc cctcagcccc accccacccc tgcctcacat ggcaacgcag    951420
     ggtctgcaga tgcaggagag tgagaagcat ggtagccagg cagactagag gacccgagct    951480
     ggggttgagc acctccctgt ctacccaggg catggcctgt gagactgcag gtggcctagt    951540
     gtgtgctgca ggctcaaggt cctgccccag ggagcatgac attcaggccc agaaattgca    951600
     tcgtgctgca cacagtccaa ggggataacc ctgtgaagtt caggtcacca gcaggcttgg    951660
     ggtcaagacc gagctgcaga ggacaggttt ctggaaggca cagcatcatg ggtggaggga    951720
     cttggagcaa ggtccttagc cccgggacca gtgaatgtgt gcccttatag ggaaaggggg    951780
     tctttgcaga agcaagttag ctgaaaatca tgaagtggag aggctcccct ggattaaagg    951840
     ggtgagccct aatgtaatca caagtgtcct tctaggaggt tggcagaggg agactgacat    951900
     agacagaagc caggtgaggt gggaagcgga ggcagaggcc gagagagcag acgctacgcc    951960
     ctggccctga agacggagga ggagccgaga gctcagggat ggagagactg gaggaggcag    952020
     ggaagttctc cccgcaagcc tggagggagc atggcctcca gcacccccag accttggccc    952080
     tgcaggattc atctggacct gtggtataaa tggtgtttaa gccactgggc tgtgcaaatt    952140
     gtcatagcag ccatggcgca ttcctagagg gagccctggt ggggacccag caggcagcga    952200
     cggggccctc acaagcctgt gagccactca gagccgcgag agtggctagg cttggtgagg    952260
     tgcaggccac gcgcacctcc actaaggcag ccttagggcc cacacttcct ctctctctct    952320
     ctctctctct ctctctatct ctctccctcc ctccttccct ccctcccgct ctcttggttg    952380
     gacagctctc catcatcccc ctggacacga ccacctccca aggccgagct ggggcgcttt    952440
     gctcgaggtg agcactgaca tcctgggggt gtgaggggca cctgcccagc ggccccgtgt    952500
     gcaggatggg cggtgggccc tagctggcac tgggcatatg gcccggctgg tgcctgcagg    952560
     ctgcagcttt tctggggtgg ctgggatcag tgaaggcctc cagagtctgg gcctgggatc    952620
     cctgcagtgc tggctgagga caggcggggc tgggcagtga gggcactggg tcactatcac    952680
     cacccacggt ttattacttc actaggtggg acctggcacc cctgccttcc tgacaccctg    952740
     gaatccctgc ctcctcctac agcccccaag cccatctccc tcagagcctc cagagacaga    952800
     cctggggagg catttcttct gcccccagca gaagcccggg aggccgggaa ggcacagtgg    952860
     gtctaaagga gaggatccca ggactgcctg aggggtgact ccgacgaggc aagcatagag    952920
     cccactgaga agcggggtgg gagccccacc agggatgggc tagttcctca tgaaggacca    952980
     ggacccagga aggacaaggg ggcctgctgg ggcagggtct gctatgccgg agtccctgtg    953040
     agcctggccc agacctgcct ctctctttcc tcattggtcc ccacaggtcc gtggtggttg    953100
     ccgtatcggg aggccccatg gtggcagggg tgggacacct ggtatacgtc gccaggtgtg    953160
     tccaataggc tcatgctcac accttctcct ggcacctggg caaagcctga gcacccaggc    953220
     actgaagtga gggcaaggcc tcggggcccc acaggatggc cgaggagaca gctgcagggc    953280
     gcctgggacc cctgggctca ggaggtagaa ggatacagcc tgaaaaccca caccacaagc    953340
     tcaccggcca gtgcaggccc acagagctcg aggaggcagc cctgagcctc ccagggagag    953400
     atgctctgtg cacgccggca caggccctgg gttacaaacc ctaggcacag cccaggagag    953460
     gcccaggccc cagtccagca aggggttgca ggaagcaaga ggtccccggc cacagcatga    953520
     gataagccca tcaagccagg gccaggtggg caatgggagg caggcagggc ttgggggtga    953580
     gtccctgctg cagcgccgtc cactgtcgac cggaggagtt tcttccctgt gcggagtcca    953640
     cgggcctcct gtgagtgtgt gcatgggcac aagtgtgtgt gtggctctgc tgtgtgtctg    953700
     tacacacgta tgttttgggt ttttttgtgt ctcagaccac agagtctgcc cctcccacca    953760
     aagcccaggc agaaggatga acccacgccc ctggggccca ggcctcagca gcctctgcag    953820
     gatcattgtt cccagttgtc acttgccttt gccacagccc tatttctcca caattcctta    953880
     aagtcctcaa catgcattta aggcacaaag gtgaaactgc ccagaaacat ctgactccgc    953940
     cgtggaaccc aggagcaagc tgggttagct aaggagcggg gccgttggca gaggctgggg    954000
     atccaggctg aactttggag gaggcatgtg ccagcatggg ctcctgacta tgtcctcctg    954060
     ggacaaaccc aaacccactc tttgaatatg ggagggactt tgctggcccc ggccctgacc    954120
     gcagcacttg gaaactgagg agtggtcgcc tcctccgtgt cacagctgcc cgttcaccat    954180
     catagaagca actctgtcac ctccatgggc ccctctgtgg ctgctgcctg ggtccaagct    954240
     gagcccagct gcccaggccc agaaggaaag cccaggccag gtgcccagca cagaggcagt    954300
     cacatacccc ggggagagcc acagcaagca gccaatgttg cccaggagag gagtagctga    954360
     caaggtagaa cgtgagctgc catcggctcg agaggctttg ctggtcctcc tggggctctg    954420
     gacatgacca ggaggagcga gggaagaagt cgcatggtgg tcccatcctg ggtggggcct    954480
     gatggcagct ggccacccgt cccagagtgg cagccagatg ccagcgccat tcccacagtc    954540
     acatcattgg tcacagaatg caggacatag agtgtcttct ttccatcaca gtgctgtcca    954600
     gacccatagc ctagggtaga cctggaagat tcaatgtcca cacccggggc tggagcgtag    954660
     ccatgagcca cgccccctgc ccgtgcatgg aaagccagcc caagctctgc tccatcccta    954720
     gccaaagtca gtgtcctttc cccttctccc aagtgagctc tagccacctg cctaccctgc    954780
     catctgagga tgacagcctt cattccattg gaacctggct ctgccaccag caggcttgca    954840
     gtcctgggca gactccgtca cctctctatg cctcagcctt tccatctgca caggaggaag    954900
     atgatgatgg tggtgatgat gatggcgatg gtttcctttt gcatctgagg caaggactaa    954960
     ttgagatgat acacatcagg cactgggtat ggtgctggtc cttcctgagc actcaatcta    955020
     tgtgagctgt ccttgtgaaa tgggtgtcac cacatttccc cacgcagaac atcctttgtc    955080
     tgccatactt gaaacgtctg ccccaatact aacagctcct catggaagat gtgcacaccc    955140
     acccaccctc atactcccaa aggtgcccgt gctttatcaa gccaaagtcc agccaggaac    955200
     tttacagcag catccctttc cctctccaag caccaaggag caaggcaaag cactacatct    955260
     tccatctgga ggcaatgcca ccctcttctc ccattttcac tgccatccct aagaggcagt    955320
     gcttccccaa aaggttccat agcagcctgc ctacagcaac tctgttcaca cgagtttcag    955380
     catccttgca gtggctcccc tgccatgctg tggctcttca ttcaccctct tctcctgctc    955440
     cccgtgacag gcatagattc tgagtgatct ggatacattg ctttgtttaa taacattaca    955500
     gcttctgtgc tgaaaaagat acagcagata gagaaggcaa ttgttgaaca caaaatagtg    955560
     acagcagaga tgacggcaag ttggcatttt tcttttctag caataaaact taaagctgac    955620
     tcaaggagaa atggaaatca taattggaac agtaatcctc aagaaagcat taagattatt    955680
     aaataattgc cctcacagat gacttcaggc caagatggct ttatgggtga agtttagact    955740
     ttcacaaaac taatcagttc ccataagaac tgctccagga tttggaggaa catgggaaag    955800
     tctattaaag ggatcacaat tcacagtccc cagagtaaaa catgggctaa cttgcatttt    955860
     ggcaaagagc caaatgttat aaatgacatc ctagaaggcc aaattctgtc catctcgttg    955920
     aacaaggact tacaccagga atttagaact atttatagct catcccacca ctcaggccaa    955980
     tgatgaccca tgatcatctc accagaaatg gaaagactca gatgattaat agagtctcaa    956040
     tttctctgag acatctaaga gcccagccca agcccagacc caggagggca cccaggcctg    956100
     gacagagaac actgatatca caccagccct ccagagggaa gcagagactc cttcaagctc    956160
     tggaaacaca ggcccagaca gctgcctaaa gttgggcagg cttcactgca aacccaaatc    956220
     atgaagctag gtaacacctt tacagattct ttacatttaa aaatcatcaa aacaagagta    956280
     aataataaac tcaaataata ttaatctaat atgtaaaggt cttgtaccat tattatgcaa    956340
     acaacataca taagctaata agaaaaagaa caaatccctt aagaaatcgg caaaaaggat    956400
     ataacacaat ttctaaaaga aaacaaatgg ctagcacaca taaggaaaac actttgtgaa    956460
     cagacattct tcagaacatt atttataatt ataaaatagt tgaaagcaag atagtgcctg    956520
     aagaaattat ggtgcataca ttagtgggac tattctgcaa acattcccaa ttatacttgt    956580
     cacatatctg tgataacgtg acagccagca ttcatggggt gacctcattt ggtaaaaggg    956640
     tgcaaagctc aacacgcatt gtgagatgac tgtggtgtaa aattagtggg attattccgc    956700
     aaacattccc aattatactt accgcatatc tgtgataaca tgacagcatt catggggtga    956760
     cctcatttgg taaaagggtg caaagctcaa cacgcattgt gagatgactg gtgtaaatac    956820
     aaagaccaaa ctgtgaaaag gagtccatca attaatcgat gcttaccttc agttttgggc    956880
     taatttttaa agtatgctat aagcatatgc tcctgttata acagaatgga gggattatga    956940
     gagatgatgc aggtgtgtcc tgggcctccc ctggcccact gggccctaga gatgccttcc    957000
     caggcatcgc tgtcagggct tccctcagag ggagtcctgt attgacctca ccaccaaggt    957060
     ctggagcagg ggatccttag atattggttg gggttatctc accttaggtc tgaatttggg    957120
     gttgtcttag actgttttgt gctgttagaa tagaataccc aagactggga aatttatact    957180
     gaacggaaat ttatttctca cagttctaga ggctgtgaag tccaagagca caggtgccag    957240
     agcaagtcca agagcaaggg aaagtccaaa gcaagtccag gagcatctgg cgaggacctt    957300
     cttgctgtgt catcacatgg cggaaggcaa gaaagagagc aagagggggc cgaactcacc    957360
     cttttataac agcaccaatc ccacccatga ggtggggacc ttatgaccta atcactcttc    957420
     atactgttac aatggcaatg aaatttcaac atgagttttg gaggagagaa gcattcaaac    957480
     cacagcaagg gtgctcctac ctcctctctc agggcatctg cagaaagagc tgcaactgca    957540
     cgtccttcct ccgtccatcc tccatccctt cccaatgtcc gtgcatatcc tgtgacccag    957600
     gaggtctggc atagggggtg ctcctgcctt aggtctgagg ccctgtctga agaggggtag    957660
     gtgaggaggc catctgatgg tctgggccaa gacagtcaca ggacgcatca tttatcatca    957720
     aggaggctga gggttgagtc tccaggtcca gggaactccc cacaaagtgg gaaccctgcc    957780
     cagctccaca cagcctctgc tgggggaccc tgctctggtg cagagcctgg ggacaggtct    957840
     tgagctcagc cagagtctgc ctccctgtca tttaggaact aaaccaagcg gcaggatgct    957900
     ggagcccagc ccccatctga ccttacaggg ccaaggctgg ggccctgggt tcccctcaag    957960
     gcgcagcagg actggagccc caggcagtgc aggagtggcc aaagctgggg cttcctccag    958020
     agcccccaag catcacggca ccaagaaggg taggaccctg gcctgaggaa ttggcaccaa    958080
     agccccagaa actaccctgg acaccatgga gagaggcctg gaggggaagc accaggcact    958140
     gcctcccctt ctgatcccac ctgaggtggc tgccaagccc agagagccgc tctgatgtcc    958200
     cccagccctg cagcccaggg atacctgtac tgtgcccctg ggggacccct ggccagtctg    958260
     tgcaaagaag tcaccaccct acactcagag acagtggggg tcctcgtccc acatcctcag    958320
     agcatggccc ggctgctgca gggatggtct cctggtcctc agagcatggc ccggctgctg    958380
     cagggatggt ctcctggtcc tcagagcatg gcccagctgc tgcagggatg gtctcctgga    958440
     ggccccccag tgctctattg tcagggctcc ctccaccccc cgcaccaaga gagagccaga    958500
     ccccagcaag gcttccagtg gcttcaggtc acacccctag gctgacccca gccccattaa    958560
     cacctgcctg agaaagctcc acgcaccaga actgaccgtc tgctccaact cttgacctcc    958620
     cgttctcagg gcgtctgctg aaaaggctgc aactgcacat ccttcctccg tccgttcccg    958680
     atgtccgtgt gtctcctgtg gccaggaagg tctttctcgg gacctgagag ccgctccctg    958740
     aagtgtcccc attgggaagg atggggcctg tgtctccagg ctctgggagg acagaatcct    958800
     gacctcaaca gtggccggca cggacacaac tggccccatc ccggggacgc tgaccagcgc    958860
     tgggcaactt ttcccttccc cgacgactga gccccgagca ccctccctgc tcccctacca    958920
     cctcccttta caaggctgtg gcctctgcac agatgataat ggagcttggc tcattcccct    958980
     agagtcggta gggagttaag gacaaaactc agtttcctcc acctgaactc aagtctgcct    959040
     atgtttacct aatcacacct ggtggacagt ttggacaaac ttgcacactc agagacacag    959100
     acacttctag aaatcattat ctccctgccc cggggacccc actccagcag aagtctgcta    959160
     ggcactggcc tgggccctcc tgctgtccta ggaggctgct gacctcctgc ctggctcctg    959220
     tccccaggtc cagagtcaga gcagactcca gggacgctgc aggctaggaa gccgccccct    959280
     ccaggcgagg gtctagtgca ggtgcccagg acaagaaaga ttgtgaatgc aggaatgact    959340
     gggccacacc cctcccgtgc acgccccctc ctgccctgca ccccacagcc cagccccccg    959400
     tgctggatgc ccccccacag cagaggtgct gttctgtgat cccctgggaa agacgccctc    959460
     aacctccacc ctgtcccacg gcccaaggaa gacaagacac aggccctctc ctcacagtct    959520
     ccccacctgg ctcctgctgg gaccctcaag gtgtgaacag ggaggatggt tgtctgggtg    959580
     gcccctagga gcccagatct tcactccaca gaccccaacc caagcacccc cttctgcagg    959640
     gcccagctca tccccctcct cctccctctg ctctcctctc gtcgcctcta cgggaaatcc    959700
     gggactcagc agtaaccctc aggaagcagg gcccaggcgc cgtttaatag gaggcttcct    959760
     cacaatgaaa cttttagaaa gccttgacta caatgatgac cttggtgtgg ctgtgaacac    959820
     tgtcagctcc cacagctgct gcagcaaaaa atgtccatag acagggtggg ggcccggggt    959880
     cgtctgctgt cctgctcagc ccacagcacg catggaggat ctgaggtgcc acacctgacg    959940
     cccaggccag aacatgcctc cctccagggt gacctgccat gtcctgcatt gctggaggga    960000
     caggggcagc ctatgaggat ctggggccag gagatgaatc ctattaaccc agaggaaaac    960060
     taacaggacc caagcaccct ccccgttgaa gctgacctgc ccagaggggc ctgggcccac    960120
     cccacacacc ggggcggaat gtgtacaggc cccggtctct gtgggtgttc cgctaactgg    960180
     ggctcccagt gctcacccca caactaaagc gagccccagc ctccagagcc cccgaaggag    960240
     atgccgccca caagcccagc ccccatccag gaggccccag agctcagggc gccggggcag    960300
     attctgaaca gccccgagtc acggtgggta caactggaac gaccaccgtg agaaaaactg    960360
     tgtccaaaac tctctcctgg cccctgctgg aggccgcgcc agagagggga gcagccgccc    960420
     cgaacctagg tcctgctcag ctcacacgac ccccagcagc cagagcacag tggagtcccc    960480
     actgaacccc actgaatggt gaggacgggg accagggctc cagggggtca tggaaggggc    960540
     tggaccccat cctactgcta tggtcccagt gctcctggcc agaactgacc ctaccaccga    960600
     caagagtccc tcagggaaac gggggtcact ggcacctccc agcatcaacc ccaggcagca    960660
     caggcataaa ccccacatcc agagccgact ccaggagcag agacacccca gtaccctggg    960720
     ggacaccgac cctgatgact ccccactgga atccacccca gagtccacca ggaccaaaga    960780
     ccccgccccg gtctctgtcc ctcactcagg acctgctgcg gggcgggcca tgagaccaga    960840
     ctcgggctta gggaacacca ctgtggcccc aacctcgacc aggccacagg cccttccttc    960900
     ctgccctgcg gcagcacaga ctttggggtc tgtgcagaga ggaatcacag aggccccagg    960960
     ctgaggtggt gggggtggaa gacccccagg aggtggccca cttcccttcc tcccagctgg    961020
     aacccaccat gaccttctta agataggggt gtcatccgag gcaggtcctc catggagctc    961080
     ccttcaggct cctccctggt cctcactagg cctcagtccc ggctgtggga atgcagccac    961140
     cacaggcaca ccaggcagcc cagacccagc cagcctgcag tgcccaagcc cacattctgg    961200
     agcagagcag gctgtgtctg ggagagtctg ggctccccac cgccccccgc acaccccacc    961260
     cacccctgtc caggccctat gcaggagggt cagagccccc catggggtat ggacttaggg    961320
     tctcactcac gcggctcccc tcctgggtga aggggtctca tgcccagatc cccacagcag    961380
     agctggtcaa aggtggaggc agtggcccca gggccaccct gacctggacc ctcaggctcc    961440
     tctagccctg gctgccctgc tgtccctggg aggcctggac tccaccagac cacaggtcca    961500
     gggcaccgcc cataggtgct gcccacactc agttcacagg aagaagataa gctccagacc    961560
     cccaagactg ggacctgcct tcctgccacc gcttgtagct ccagacctcc gtgcctcccc    961620
     cgaccactta cacacgggcc agggagctgt tccacaaaga tcaaccccaa accgggaccg    961680
     cctggcactc gggccgctgc cacttccctc tccatttgct cccagcacct ctgtgctccc    961740
     tccctcctcc ctccttcagg ggaacagcct gtgcagcccc tccctgcacc ccacaccctg    961800
     gggaggccca accctgcctc cagccctttc tcccccgctg ctcttcctgc ccatccagac    961860
     aaccctgggg tcccatccct gcagcctaca ccctggtctc cacccagacc cctgtctctc    961920
     cctccagaca cccctcccag gccaaccctg cacatgcagg ccctcccctt ttctgctgcc    961980
     agagcctcag tttctaccct ctgtgcctac cccctgcctc ctcctgccca caactcgagc    962040
     tcttcctctc ctggggcccc tgagccatgg cactgaccgt gcactcccac ccccacactg    962100
     cccatgccct caccttcctc ctggacactc tgaccctgct cccctcttgg acccagccct    962160
     ggtatttcca ggacaaaggc tcacccaagt cttccccatg caggcccttg ccctcactgc    962220
     ccggttacac ggcagcctcc tgtgcacaga agcagggagc tcagcccttc cacaggcaga    962280
     aggcactgaa agaaatcggc ctccagcacc ctgatgcacg tccgcctgtg tctctcactg    962340
     cccgcacctg cagggaggct cggcactccc tgtaaagacg agggatccag gcagcaacat    962400
     catgggagaa tgcagggctc ccagacagcc cagccctctc gcaggcctct cctgggaaga    962460
     gacctgcagc caccactgaa cagccacgga gcccgctgga tagtaactga gtcagtgacc    962520
     gacctggagg gcaggggagc agtgaaccgg agcccagacc atagggacag agaccagccg    962580
     ctgacatccc gagcccctca ctggcggccc cagaacaccg cgtggaaaca gaacagaccc    962640
     acattcccac ctggaacagg gcagacactg ctgagccccc agcaccagcc ctgagaaaca    962700
     ccaggcaacg gcatcagagg gggctcctga gaaagaaagg aggggaggtc tccttcacca    962760
     gcaagtactt cccttgacca aaaacagggt ccacgcaact cccccaggac aaaggaggag    962820
     ccccctgtac agcactgggc tcagagtcct ctccaacaca ccctgagttt cagacaaaaa    962880
     ccccctggaa atcatagtat cagcaggaga actagccaga gacagcaaga ggggactcag    962940
     tgactcccgc ggggacagga ggattttgtg ggggctcgtg tcactgtgag gatattgtag    963000
     tagtaccagc tgctatgccc acagtgacac agccccattc ccaaagccct gctgtaaacg    963060
     cttccacttc tggagctgag gggctggggg gagcgtctgg gaagtagggc ctaggggtgg    963120
     ccatcaatgc ccaaaacgca ccagactccc ccccagacat caccccactg gccagtgagc    963180
     agagtaaaca gaaaatgaga agcagctggg aagcttgcac aggccccaag gaaagagctt    963240
     tggcgggtgt gcaagagggg atgcgggcag agcctgagca gggccttttg ctgtttctgc    963300
     tttcctgtgc agatagttcc ataaactggt gttcaagatc gatggctggg agtgagccca    963360
     ggaggacagt gtgggaaggg cacagggaag gagaagcagc cgctatccta cactgtcatc    963420
     tttcaagagt ttgccctgtg cccacaatgc tgcatcatgg gatgcttaac agctgatgta    963480
     gacacagcta aagagagaat cagtgaaatg gatttgcagc acagatctga ataaattctc    963540
     cagaatgtgg agccacacag aagcaagcac aaggaaagtg cctgatgcaa gggcaaagta    963600
     cagtgtgtac cttcaggctg ggcacagaca ctctgaaaag ccttggcagg aactccctgc    963660
     aacaaagcag agccctgcag gcaatgccag ctccagagcc ctccctgaga gcctcatggg    963720
     caaagatgtg cagaacatat gtttgtcata gccccaaact gagaatgaag caaacagcca    963780
     tctgaaggaa aacaggcaaa taaacgatgg caggttcatg aaatgcaaac ccagacagcc    963840
     agaaggacaa cagtgagggt tacaggtgac tctgtggttg agttcatgac aatgctgagt    963900
     aattggagta acaaaggaaa gtccaaaaaa tactttcaat gtgatttctt ctaaataaaa    963960
     tttacagccg gcaaaatgaa ctatcttctt aagggataaa ctttccacta ggaaaactat    964020
     aaggaaaatc aagaaaagga tgatcacata aacacagtgg tcgttacttc tactggggaa    964080
     ggaagagggt atgaactgag acacacaggg ttggcaagtc tcctaacaag aagagaacaa    964140
     atacattaca gtaccttgaa aacagcagtt aaaattctaa attgcaagaa gaggaaaatg    964200
     cacacagctg tgtttagaaa attctcagtc cagcactgtt cataatagca aagacattaa    964260
     cccaggttgg ataaataaac gatgacacag gcaattgcac aatgatacag acatacattc    964320
     agtatatgag acattgatga tgtatcccca aagaaatgac tttaaagaga aaaggcctga    964380
     tatgtggtgg cactcacctc cctgggcatc cccggacagg ctgcaggcac actgtgtggc    964440
     agggcaggct ggtacctgct ggcagctcct ggggcctgat gtggagcagg cacagagccg    964500
     tatccccccg aggacatata cccccaagga cggcacagtt ggtacattcc ggagacaagc    964560
     aactcagcca cactcccagg ccagagcccg agagggacgc ccatgcacag ggaggcagag    964620
     cccagctcct ccacagccag cagcacctgt gcaggggccg ccatctggca ggcacagagc    964680
     atgggctggg aggaggggca gggacaccag gcagggttgg caccaactga aaattacaga    964740
     agtctcatac atctacctca gccttgcctg acctgggcct cacctgacct ggacctcacc    964800
     tggcctggac ctcacctggc ctagacctca cctctgggct tcacctgagc tcggcctcac    964860
     ctgacttgga ccttgcctgt cctgagctca catgatctgg gcctcacctg acctgggttt    964920
     cacctgacct gggcttcacc tgacctgggc ctcatctgac ctgggcctca ctggcctgga    964980
     cctcacctgg cctgggcttc acctggcctc aggcctcatc tgcacctgct ccaggtcttg    965040
     ctggaacctc agtagcactg aggctgcagg ggctcatcca gggttgcaga atgactctag    965100
     aacctcccac atctcagctt tctgggtgga ggcacctggt ggcccaggga atataaaaag    965160
     cctgaatgat gcctgcgtga tttgggggca atttataaac ccaaaaggac atggccatgc    965220
     agcgggtagg gacaatacag acagatatca gcctgaaatg gagcctcagg gcacaggtgg    965280
     gcacggacac tgtccaccta agccaggggc agacccgagt gtccccgcag tagacctgag    965340
     agcgctgggc ccacagcctc ccctcggtgc cctgctacct cctcaggtca gccctggaca    965400
     tcccgggttt ccccaggcct ggcggtaggt ttggggtgag gtctgtgtca ctgtggtatt    965460
     acgatttttg gagtggttat tatacccaca gtgtcacaga gtccatcaaa aacccatccc    965520
     tgggaacctt ctgccacagc cctccctgtg gggcaccgct gcgtgccatg ttaggatttt    965580
     gactgaggac acagcaccat gggtatggtg gctaccgcag cagtgcagcc tgtgacccaa    965640
     acacacaggg cagcaggcac aacagacaag cccacaagtg accaccctga gctcctgcct    965700
     gccagccctg gagaccatga aacagatggc caggattatc ccataggtca gccagacctc    965760
     agtccaacag gtctgcatcg ctgctgccct ccaataccag tccggatggg gacagggccg    965820
     gcccacatta ccatttgctg ccatccggcc aacagtccca gaagcccctc cctcaaggct    965880
     gggccacatg tgtggaccct gagagccccc catgtctgag taggggcacc aggaaggtgg    965940
     ggctggccct gtgcactgtc actgcccctg tggtccctgg cctgcctggc cctgacacct    966000
     gggcctctcc tgggtcattt ccaagacaga agacattccc aggacagctg gagctgggag    966060
     tccatcatcc tgcctggcca tcctgagtcc tgcgcctttc caaacctcac ccgggaagcc    966120
     aacagaggaa tcacctccca caggcagaga caaagacctt ccagaaatct ctgtctctct    966180
     ccccagtggg caccctcttc cagggcagtc ctcagtgata tcacagtggg aacccacatc    966240
     tggatcggga ctgcccccag aacacaagat ggcccacagg gacagcccca cagcccagcc    966300
     cttcccagac ccctaaaagg cgtcccaccc cctgcatctg ccccagggct caaactccag    966360
     gaggactgac tcctgcacac cctcctgcca gacatcacct cagcccctcc tggaagggac    966420
     aggagcgcgc aagggtgagt cagaccctcc tgccctcgat ggcaggcgga gaagattcag    966480
     aaaggtctga gatccccagg acgcagcacc actgtcaatg ggggccccag acgcctggac    966540
     cagggcctgc gtgggaaagg cctctgggca cactcagggg ctttttgtga agggtcctcc    966600
     tactgtgtga ctacagtaac taccacagtg atgaacccag cagcaaaaac tgaccggact    966660
     cccaaggttt atgcacactt ctccgctcag agctctccag gatcagaaga gccgggccca    966720
     agggtttctg cccagaccct cggcctctag ggacatcttg gccatgacag cccatgggct    966780
     ggtgccccac acatcgtctg ccttcaaaca agggcttcag agggctctga ggtgacctca    966840
     ctgatgacca caggtgccct ggccccttcc ccgccagctg caccagaccc cgtcctgaca    966900
     gatgccccga ttccaacagc caattcctgg ggccaggaat cgctgtagac accagcctcc    966960
     ttccaacacc tcttgccaat tgcctggatt cccatcccgg ttggaatcaa gaggacagca    967020
     tcccccaggc tcccaacagg caggactccc acaccctcct ctgagaggcc gctgtgttcc    967080
     gtagggccag gctgcagaca gtccccctca cctgccacta gacaaatgcc tgctgtagat    967140
     gtccccacct ggaaaagacc actcatggag cccccagccc caggtacagc catagagaga    967200
     gtctctgagg cccctaagaa gtagccatgc ccagttctgc cgggaccctc ggccaggctg    967260
     acaggagtgg acgctggagc tgggcccaca ctgggccaca taggagctca ccagtgaggg    967320
     caggagagca catgccgggg agcacccagc ctcctgctga ccagaggccc gtcccagagc    967380
     ccaggaggct gcagaggcct ctccaggggg acactgtgca tgtctggtcc ctgagcagcc    967440
     ccccatgtcc ccagtcctgg gggcccctgg cacagctgtc tggaccctct ctattccctg    967500
     ggaagctcct cctgacagcc ccgcctccag ttccaggtgt ggttattgtc agggggtgtc    967560
     agactgtggt ggatacagct atggttacca cagtggtgct gcccatagca gcaaccaggc    967620
     caagtagaca ggcccctgct gtgcagcccc aggcctccag ctcacctgct tctcctgggg    967680
     ctctcaaggc tgctgttttc tgcactctcc cctctgtggg gagggttccc tcagtgggag    967740
     atctgttctc aacatcccag ggcctcattc ctgcaaggaa ggccaatgga tgggcaacct    967800
     cacatgccgc ggctaagata gggtgggcag cctggcgggg acaggacatc ctgctggggt    967860
     atctgtcact gtgcctagtg gggcactggc tcccaaacaa cgcagtcctc gccaaaatcc    967920
     ccacggcctc ccccgctagg ggctggcctg atctcctgca gtcctaggag gctgctgacc    967980
     tccagaatgg ctccgtcccc agttccaggg cgagagcaga tcccaggccg gctgcagact    968040
     gggaggccac cccctccttc ccagggttca ctgcaggtga ccagggcagg aaatggcctg    968100
     aacacaggga taaccgggcc atcccccaac agagtccacc ccctcctgct ctgtaccccg    968160
     cacccccaag gccagcccat gacatccgac aaccccacac cagagtcact gcccggtgct    968220
     gccctaggga ggacccctca gcccccaccc tgtctagagg actggggagg acaggacacg    968280
     ccctctcctt atggttcccc cacctggctc tggctgggac ccttggggtg tggacagaaa    968340
     ggacgcttgc ctgattggcc cccaggagcc cagaacttct ctccagggac cccagcccga    968400
     gcaccccctt acccaggacc cagccctgcc cctcctccca tctgctctcc tctcatcacc    968460
     ccatgggaat ccagaatccc caggaagcca tcaggaaggg ctgagggagg aagtggggcc    968520
     actgcaccac caggcaggag gctccgtctt tgtgaaccca gggaggtgcc agcctcctag    968580
     agggtatggt ccaccctgcc tatggctccc acagtggcag gctgcaggga aggaccaggg    968640
     acggtgtggg ggagggctca gggccccgcg ggtgctccat cttggatgag cccatctctc    968700
     tcacccacgg actcacccac ctcctctcca ccctggccac acgtcgtcca caccatccta    968760
     agtcccacct acaccagagc cggcacagcc agtgcagaca gaggctgggg tgcagggggg    968820
     ccgccagggc agctttgggg agggaaggat ggaggaaggg gagttcagtg aagaggcccc    968880
     cctcccctgg gtccaggatc ctcctctggg acccccggat cccatcccct ccaggctctg    968940
     ggaggagaag caggatggga gaatctgtgc gggaccctct cacagtggaa tacctccaca    969000
     gcggctcagg caagacccaa aagcccctca gtgagccctc cactgcagtc ctgggcctgg    969060
     gtagcagccc ctcccacaga ggatgaaccc agcaccccga ggatgtcctg ccagggggag    969120
     ctcagagcca tgaaggagca ggatatggga cccccgatac aggcacagac ctcagctcca    969180
     ttcaggactg ccacgtcctg ccctgggagg aacccctttc tctagtccct gcaggccagg    969240
     aggcagctga ctcctgactt ggacgcctat tccagacacc agacagaggg gcaggccccc    969300
     cagaaccagg gatgaggacg ccccgtcaag gccagaaaag accaagttgt gctgagccca    969360
     gcaagggaag gtccccaaac aaaccaggaa gtttctgaag gtgtctgtgt cacagtggag    969420
     tatagcagct cgtcccacag tgacactcgc caggccagaa accccatccc aagtcagcgg    969480
     aatgcagaga gagcagggag gacatgttta ggatctgagg ccgcacctga cacccaggcc    969540
     agcagacgtc tcctgtccat ggcaccctgc catgtcctgc atttctggaa gaacaagggc    969600
     aggctgaagg gggtccagga ccaggagatg ggtcccctct acccagagaa ggagccaggc    969660
     aggacacaag ccccctcccc attgaggctg acctgcccag agggtcctgg gcccacccca    969720
     cacaccgggg cggaatgtgt gcaggcctcg gtctctgtgg gtgttccgct agctggggct    969780
     cacagtgctc accccacacc taaaacgagc cacagcctca gagcccctga aggagacccc    969840
     gcccacaagc ccagccccca cccaggaggc cccagagcac agggcgcccc gtcggattct    969900
     gaacagcccc gagtcacagt gggtataact ggaactacca ctgtgagaaa agcttcgtcc    969960
     aaaacggtct cctggccaca gtcggaggcc ccgccagaga ggggagcagc caccccaaac    970020
     ccatgttctg ccggctccca tgaccccgtg cacctggagc cccacagtgt ccccactgga    970080
     tgggaggaca agggccgggg gctccggcgg gtcggggcag gggcttgatg gcttccttct    970140
     gccgtggctc cagtgcccct ggctggagtt gacccttctg acaagtgtcc tcagagagtc    970200
     agggatcagt ggcacctccc aacatcaacc ccacgcagcc caggcacaaa ccccacatcc    970260
     agggccaact ccaggaacag agacacccca ataccctggg ggaccccaac cctgatgact    970320
     cccgtcccat ctctgtccct cacttggggc ctgctgcggg gcgagcactt gggagcaaac    970380
     tcaggcttag gggacaccac tgtgggcctg acctcgagca ggccacagac ccttccctcc    970440
     tgccctggtg cagcacagac tttggggtct gggcagggag gaacttctgg caggtcacca    970500
     agcacagagc ccccaggctg aggtggcccc agggggaacc ccagcaggtg gcccactacc    970560
     cttcctccca gctggacccc atgtcttccc caagataggg gtgccatcca aggcaggtcc    970620
     tccatggagc ccccttcagg ctcctctcca gaccccactg ggcctcagtc cccactctag    970680
     gaatgcagcc accacgggca caccaggcag cccaggccca gccaccctgc agtgcccaag    970740
     cccacaccct ggaggagagc agggtgcgtc tgggaggggc tgggctcccc acccccaccc    970800
     ccacctgcac accccaccca cccttgcccg ggccccctgc aggagggtca gagcccccat    970860
     gggatatgga cttagggtct cactcacgca cctcccctcc tgggagaagg ggtctcatgc    970920
     ccagatcccc ccagcagcgc tggtcacagg tagaggcagt ggccccaggg ccaccctgac    970980
     ctggcccctc aggctcctct agccctggct gccctgctgt ccctgggagg cctgggctcc    971040
     accagaccac aggtctaggg caccgcccac actggggccg cccacacaca gctcacagga    971100
     agaagataag ctccagaccc ccaggcccgg gacctgcctt gctgctacga cttcctgccc    971160
     cagacctcgt tgccctcccc cgtccactta cacacaggcc aggaagctgt tcccacacag    971220
     accaacccca gacggggacc acctggcact caggtcactg ccatttcctt ctccattcac    971280
     ttccaatgcc tctgtgcttc ctccctcctc cttccttcgg gggagcaccc tgtgcagctc    971340
     ctccctgcag tccacaccct ggggagaccc gaccctgcag cccacaccct ggggagacct    971400
     gaccctcctc cagccctttc tcccccgctg ctcttgccac ccaccaagac agccctgggg    971460
     tcctgtccct acagccccca cccagttctc tacctagacc cgtcttcctc cctctaaaca    971520
     cctctcccag gccaacccta cacctgcagg ccctcccctc cactgccaaa gaccctcagt    971580
     ttctcctgcc tgtgcccacc cccgtgctcc tcctgcccac agctcgagct cttcctctcc    971640
     tagggcccct gagggatggc attgaccgtg ccctcgcacc cacacactgc ccatgccctc    971700
     acattcctcc tggccactcc agccccactc ccctctcagg cctggctctg gtatttctgg    971760
     gacaaagcct tacccaagtc tttcccatgc aggcctgggc ccttaccctc actgcccggt    971820
     tacagggcag cctcctgtgc acagaagcag ggagctcagc ccttccacag gcagaaggca    971880
     ctgaaagaaa tcggcctcca gcgccttgac acacgtctgc ctgtgtctct cactgcccgc    971940
     acctgcaggg aggctcggca ctccctctaa agacgaggga tccaggcagc agcatcacag    972000
     gagaatgcag ggctaccaga catcccagtc ctctcacagg cctctcctgg gaagagacct    972060
     gaagacgccc agtcaacgga gtctaacacc aaacctccct ggaggccgat gggtagtaac    972120
     ggagtcattg ccagacctgg aggcagggga gcagtgagcc cgagcccaca ccatagggcc    972180
     agaggacagc cactgacatc ccaagccact cactggtggt cccacaacac cccatggaaa    972240
     gaggacagac ccacagtccc acctggacca gggcagagac tgctgagacc cagcaccaga    972300
     accaaccaag aaacaccagg caacagcatc agagggggct ctggcagaac agaggagggg    972360
     aggtctcctt caccagcagg cgcttccctt gaccgaagac aggatccatg caactccccc    972420
     aggacaaagg aggagcccct tgttcagcac tgggctcaga gtcctctcca agacacccag    972480
     agtttcagac aaaaaccccc tggaatgcac agtctcagca ggagagccag ccagagccag    972540
     caagatgggg ctcagtgaca cccgcaggga caggaggatt ttgtgggggc tcgtgtcact    972600
     gtgaggatat tgtactaatg gtgtatgcta tacccacagt gacacagccc cattcccaaa    972660
     gccctactgc aaacgcattc cacttctggg gctgaggggc tgggggagcg tctgggaaat    972720
     agggctcagg ggtgtccatc aatgcccaaa acgcaccaga ctcccctcca tacatcacac    972780
     ccaccagcca gcgagcagag taaacagaaa atgagaagca agctggggaa gcttgcacag    972840
     gccccaagga aagagctttg gcgggtgtgt aagaggggat gcgggcagag cctgagcagg    972900
     gccttttgct gtttctgctt tcctgtgcag agagttccat aaactggtgt tcgagatcaa    972960
     tggctgggag tgagcccagg aggacagcgt gggaagagca cagggaagga ggagcagccg    973020
     ctatcctaca ctgtcatctt tcgaaagttt gccttgtgcc cacactgctg catcatggga    973080
     tgcttaacag ctgatgtaga cacagctaaa gagagaatca gtgagatgga tttgcagcac    973140
     agatctgaat aaattctcca gaatgtggag cagcacagaa gcaagcacac agaaagtgcc    973200
     tgatgcaagg acaaagttca gtgggcacct tcaggcattg ctgctgggca cagacactct    973260
     gaaaagccct ggcaggaact ccctgtgaca aagcagaacc ctcaggcaat gccagcccca    973320
     gagccctccc tgagagcctc atgggcaaag atgtgcacaa caggtgtttc tcatagcccc    973380
     aaactgagag caaagcaaac gtccatctga aggagaacag gcaaataaac gatggcaggt    973440
     tcatgaaatg caaacccaga cagccacaag cacaaaagta cagggttata agcgactctg    973500
     gttgagttca tgacaatgct gagtaattgg agtaacaaag taaactccaa aaaatacttt    973560
     caatgtgatt tcttctaaat aaaatttaca ccctgcaaaa tgaactgtct tcttaaggga    973620
     tacatttccc agttagaaaa ccataaagaa aaccaagaaa aggatgatca cataaacaca    973680
     gtggtggtta cttctgctgg ggaaggaaga gggtatgaac tgagatacac agggtgggca    973740
     agtctcctaa caagaacaga acgaatacat tacagtacct tgaaaacagc agttaaactt    973800
     ctaaattgca agaagaggaa aatggacaca gttgtgttta gaaaattctc agtccagcac    973860
     tgttcataat agcaaagaca ttaacccagg tcggataaat aagcgatgac acaggcaatt    973920
     gcacaatgat acagacatat atttagtata tgagacatcg atgatgtatc cccaaataaa    973980
     cgactttaaa gagataaagg gctgatgtgt ggtggcattc acctccctgg gatccccgga    974040
     caggttgcag gctcactgtg cagcagggca ggcgggtacc tgctggcagt tcctggggcc    974100
     tgatgtggag caagcgcagg gccatatatc ccggaggacg gcacagtcag tgaattccag    974160
     agagaagcaa ctcagccaca ctccccaggc agagcccgag agggacgccc acgcacaggg    974220
     aggcagagcc cagcacctcc gcagccagca ccacctgtgc acgggccacc accttgcagg    974280
     cacagagtgg gtgctgagag gaggggcagg gacaccaggc agggtgagca cccagagaaa    974340
     actgcagacg cctcacacat ccacctcagc ctcccctgac ctggacctca ctggcctggg    974400
     cctcacttaa cctgggcttc acctgacctt ggcctcacct gacttggacc tcgcctgtcc    974460
     caagctttac ctgacctggg cctcaactca cctgaacgtc tcctgacctg ggtttaacct    974520
     gtcctggaac tcacctggcc ttggcttccc ctgacctgga cctcatctgg cctgggcttc    974580
     acctggcctg ggcctcacct gacctggacc tcatctggcc tggacctcac ctggcctgga    974640
     cttcacctgg cctgggcttc acctgacctg gacctcacct ggcctcgggc ctcacctgca    974700
     cctgctccag gtcttgctgg agcctgagta gcactgaggg tgcagaagct catccagggt    974760
     tggggaatga ctctagaagt ctcccacatc tgacctttct gggtggaggc agctggtggc    974820
     cctgggaata taaaaatctc cagaatgatg actctgtgat ttgtgggcaa cttatgaacc    974880
     cgaaaggaca tggccatggg gtgggtaggg acatagggac agatgccagc ctgaggtgga    974940
     gcctcaggac acaggtgggc acggacacta tccacataag cgagggatag acccgagtgt    975000
     ccccacagca gacctgagag cgctgggccc acagcctccc ctcagagccc tgctgcctcc    975060
     tccggtcagc cctggacatc ccaggtttcc ccaggcctgc cggtaggttt agaatgaggt    975120
     ctgtgtcact gtggtattac gatattttga ctggttatta taaccacagt gtcacagagt    975180
     ccatcaaaaa cccatgcctg gaagcttccc gccacagccc tccccatggg gccctgctgc    975240
     ctcctcaggt cagccccgga catcccgggt ttccccaggc tgggcggtag gtttggggtg    975300
     aggtctgtgt cactgtggta ttactatggt tcggggagtt attataacca cagtgtcaca    975360
     gagtccatca aaaacccatc cctgggagcc tcccgccaca gccctccctg caggggaccg    975420
     gtacgtgcca tgttaggatt ttgatcgagg agacagcacc atgggtatgg tggctaccac    975480
     agcagtgcag cctgtgaccc aaacccgcag ggcagcaggc acgatggaca ggcccgtgac    975540
     tgaccacgct gggctccagc ctgccagccc tggagatcat gaaacagatg gccaaggtca    975600
     ccctacaggt catccagatc tggctccgag gggtctgcat cgctgctgcc ctcccaacgc    975660
     cagtccaaat gggacaggga cggcctcaca gcaccatctg ctgccatcag gccagcgatc    975720
     ccagaagccc ctccctcaag gctgggccac atgtgtggac actgagagcc ctcatgtctg    975780
     agtaggggca ccaggaggga ggggctggcc ctgtgcactg tccctgcccc tgtggtccct    975840
     ggcctgcctg gccctgacac ctgagcctct cctgggtcat ttccaagaca gaagacattc    975900
     ctggggacag ccggagctgg gcgtcgctca tcctgcccgg ccgtcctgag tcctgctcat    975960
     ttccagacct caccggggaa gccaacagag gactcgcctc ccacattcag agacaaagaa    976020
     ccttccagaa atccctgcct ctctccccag tggacaccct cttccaggac agtcctcagt    976080
     ggcatcacag cggcctgaga tccccaggac gcagcaccgc tgtcaatagg ggccccaaat    976140
     gcctggacca gggcctgcgt gggaaaggtc tctggccaca ctcgggcttt ttgtgaaggg    976200
     ccctcctgct gtgtgactac agtaactacc atagtgatga acccagtggc aaaaactggc    976260
     tggaaaccca ggggctgtgt gcacgcctca gcttggagct ctccaggagc acaagagccg    976320
     ggcccaagga tttgtgccca gaccctcagc ctctagggac acctgggcca tctcagcctg    976380
     ggctggtgcc ctgcacacca tcttcctcca aataggggct tcagagggct ctgaggtgac    976440
     ctcactcatg accacaggtg acctggccct tccctgccag ctataccaga ccctgtcttg    976500
     acagatgccc cgattccaac agccaattcc tgggaccctg aatagctgta gacaccagcc    976560
     tcattccagt acctcctgcc aattgcctgg attcccatcc tggctggaat caagaaggca    976620
     gcatccgcca ggctcccaac aggcaggact cccgcacacc ctcctctgag aggccgctgt    976680
     gttccgcagg gccaggccct ggacagttcc cctcacctgc cactagagaa acacctgcca    976740
     ttgtcgtccc cacctggaaa agaccactcg tggagccccc agccccaggt acagctgtag    976800
     agagagtcct cgaggcccct aagaaggagc catgcccagt tctgccggga ccctcggcca    976860
     ggccgacagg agtggacgct ggagctgggc ccacactggg ccacatagga gctcaccagt    976920
     gagggcagga gagcacatgc cggggagcac ccagcctcct gctgaccaga ggcctgcccc    976980
     agagcccagg aggctgcaga ggcctctcca gggagacact gtgcatgtct ggtacctaag    977040
     cagcccccca cgtccccagt cctgggggcc cctggctcag ctgtctggac cctccctgtt    977100
     ccctgggaag ctcctcctga cagccccgcc tccagttcca ggtgtggtta ttgtcaggcg    977160
     atgtcagact gtggtggata tagtggctac gattaccaca gtggtgccgc ccatagcagc    977220
     aaccaggcca agtagacagg cccctgctgc gcagccccag gcatccactt cacctgcttc    977280
     tcctggggct ctcaaggctg ctgtctgtcc tctggccctc tgtggggagg gttccctcag    977340
     tgggaggtct gtgctccagg gcagggatga ttgagataga aatcaaaggc tggcagggaa    977400
     aggcagcttc ccgccctgag aggtgcaggc agcaccacgg agccacggag tcacagagcc    977460
     acggagcccc cattgtgggc atttgagagt gctgtgcccc cggcaggccc agccctgatg    977520
     gggaagcctg tcccatccca cagcccgggt cccacgggca gcgggcacag aagctgccag    977580
     gttgtcctct atgatcctca tccctccagc agcatcccct ccacagtggg gaaactgagg    977640
     cttggagcac cacccggccc cctggaaatg aggctgtgag cccagacagt gggcccagag    977700
     cactgtgagt accccggcag tacctggctg cagggatcag ccagagatgc caaaccctga    977760
     gtgaccagcc tacaggagga tccggcccca cccaggccac tcgattaatg ctcaaccccc    977820
     tgccctggag acctcttcca gtaccaccag cagctcagct tctcagggcc tcatccctgc    977880
     aaggaaggtc aagggctggg cctgccagaa acacagcacc ctccctagcc ctggctaaga    977940
     cagggtgggc agacggctgt ggacgggaca tattgctggg gcatttctca ctgtcacttc    978000
     tgggtggtag ctctgacaaa aacgcagacc ctgccaaaat ccccactgcc tcccgctagg    978060
     ggctggcctg gaatcctgct gtcctaggag gctgctgacc tccaggatgg ctccgtcccc    978120
     agttccaggg cgagagcaga tcccaggcag gctgtaggct gggaggccac ccctgccctt    978180
     gccggggttg aatgcaggtg cccaaggcag gaaatggcat gagcacaggg atgaccggga    978240
     catgccccac cagagtgcgc cccttcctgc tctgcaccct gcacccccca ggccagccca    978300
     cgacgtccaa caactgggcc tgggtggcag ccccacccag acaggacaga cccagcaccc    978360
     tgaggaggtc ctgccagggg gagctaagag ccatgaagga gcaagatatg gggcccccga    978420
     tacaggcaca gatgtcagct ccatccagga ccacccagcc cacaccctga gaggaacgtc    978480
     tgtctccagc ctctgcaggt cgggaggcag ctgacccctg acttggaccc ctattccaga    978540
     caccagacag aggcgcaggc cccccagaac cagggttgag ggacgccccg tcaaagccag    978600
     acaaaaccaa ggggtgttga gcccagcaag ggaaggcccc caaacagacc aggaggtttc    978660
     tgaaggtgtc tgtgtcacag tggggtatag cagcagctgg taccacagtg acactcaccc    978720
     agccagaaac cccattccaa gtcagcggaa gcagagagag cagggaggac acgtttagga    978780
     tctgagactg cacctgacac ccaggccagc agacgtctcc cctccagggc accccaccct    978840
     gtcctgcatt tctgcaagat caggggcggc ctgagggggg gtctagggtg aggagatggg    978900
     tcccctgtac accaaggagg agttaggcag gtcccgagca ctctccccat tgaggctgac    978960
     ctgcccagag agtcctgggc ccaccccaca caccggggcg gaatgtgtgc aggcctcggt    979020
     ctctgtgggt gttccgctag ctggggctca cagtgctcac cccacaccta aaatgagcca    979080
     cagcctccgg agcccccgca ggagaccccg cccacaagcc cagcccccac ccaggaggcc    979140
     ccagagctca gggcgccccg tcggattccg aacagccccg agtcacagcg ggtataaccg    979200
     gaaccaccac tgtcagaata gctacgtcaa aaactgtcca gtggccactg ccggaggccc    979260
     cgccagagag ggcagcagcc actctgatcc catgtcctgc cggctcccat gacccccagc    979320
     acgcggagcc ccacagtgtc cccactggat gggaggacaa gagctgggga ttccggcggg    979380
     tcggggcagg ggcttgatcg catccttctg ccgtggctcc agtgcccctg gctggagttg    979440
     acccttctga caagtgtcct cagagagaca ggcatcaccg gcgcctccca acatcaaccc    979500
     caggcagcac aggcacaaac cccacatcca gagccaactc caggagcaga gacaccccaa    979560
     taccctgggg gaccccgacc ctgatgactt cccactggaa ttcgccgtag agtccaccag    979620
     gaccaaagac cctgcctctg cctctgtccc tcactcagga cctgctgccg ggcgaggcct    979680
     tgggagcaga cttgggctta ggggacacca gtgtgacccc gaccttgacc aggacgcaga    979740
     cctttccttc ctttcctggg gcagcacaga ctttggggtc tgggccagga ggaacttctg    979800
     gcaggtcgcc aagcacagag gccacaggct gaggtggccc tggaaagacc tccaggaggt    979860
     ggccactccc cttcctccca gctggacccc atgtcctccc caagataagg gtgccatcca    979920
     aggcaggtgc tccttggagc cccattcaga ctcctccctg gaccccactg ggcctcagtc    979980
     ccagctctgg ggatgaagcc accacaagca caccaggcag cccaggccca gccaccctgc    980040
     agtgcccaag cacacactct ggagcagagc agggtgcctc tgggaggggc tgagctcccc    980100
     accccacccc cacctgcaca ccccacccac ccctgcccag cggctctgca ggagggtcag    980160
     agccccacat ggggtatgga cttagggtct cactcacgtg gctcccatca tgagtgaagg    980220
     ggcctcaagc ccaggttccc acagcagcgc ctgtcgcaag tggaggcaga ggcccgaggg    980280
     ccaccctgac ctggtccctg aggttcctgc agcccaggct gccctgctgt ccctgggagg    980340
     cctgggctcc accagaccac aggtccaggg caccgggtgc aggagccacc cacacacagc    980400
     tcacaggaag aagataagct ccagaccccc agggccagaa cctgccttcc tgctactgct    980460
     tcctgcccca gacctgggcg ccctcccccg tccacttaca cacaggccag gaagctgttc    980520
     ccacacagaa caaccccaaa ccaggaccgc ctggcactca ggtggctgcc atttccttct    980580
     ccatttgctc ccagcgcctc tgtcctccct ggttcctcct tcgggggaac agcctgtgca    980640
     gccagtccct gcagcccaca ccctggggag acccaaccct gcctggggcc cttccaaccc    980700
     tgctgctctt actgcccacc cagaaaactc tggggtcctg tccctgcagt ccctaccctg    980760
     gtctccaccc agacccctgt gtatcactcc agacacccct cccaggccaa ccctgcacct    980820
     gcaggccctg tcctcttctg tcgctagagc ctcagtttct cccccctgtg cccacaccct    980880
     acctcctcct gcccacaact ctaactcttc ttctcctgga gcccctgagc catggcattg    980940
     accctgccct cccaccaccc acagcccatg ccctcacctt cctcctggcc actccgaccc    981000
     cgccccctct caggccaagc cctggtattt ccaggacaaa ggctcaccca agtctttccc    981060
     aggcaggcct gggctcttgc cctcacttcc cggttacacg ggagcctcct gtgcacagaa    981120
     gcagggagct cagcccttcc acaggcagaa ggcactgaaa gaaatcggcc tccagcacct    981180
     tgacacacgt ccccccgtgt ctctcactgc ccgcacctgc agggaggctc cgcactccct    981240
     ctaaagacaa gggatccagg cagcagcatc acgggagaat gcagggctcc cagacatccc    981300
     agtcctctca caggcctctc ctgggaagag acctgcagcc accaccaaac agccacagag    981360
     gctgctggat agtaactgag tcaatgaccg acctggaggg caggggagca gtgagccgga    981420
     gcccatacca tagggacaga gaccagccgc tgacatcccg agctcctcaa tggtggcccc    981480
     ataacacacc taggaaacat aacacaccca cagccccacc tggaacaggg cagagactgc    981540
     tgagccccca gcaccagccc caagaaacac caggcaacag tatcagaggg ggctcccgag    981600
     aaagagagga ggggagatct ccttcaccat caaatgcttc ccttgaccaa aaacagggtc    981660
     cacgcaactc ccccaggaca aaggaggagc cccctataca gcactgggct cagagtcctc    981720
     tctgagacac cctgagtttc agacaacaac ccgctggaat gcacagtctc agcaggagaa    981780
     cagaccaaag ccagcaaaag ggacctcggt gacaccagta gggacaggag gattttgtgg    981840
     gggctcgtgt cactgtgagg atattgtagt ggtggtagct gctactccca cagtgacaca    981900
     gacccattcc caaagcccta ctgcaaacac acccactcct ggggctgagg ggctggggga    981960
     gcgtctggga agtagggtcc aggggtgtct atcaatgtcc aaaatgcacc agactgcccg    982020
     ccaaacacca ccccaccagc cagcgagcag ggtaaacaga aaatgagagg ctctgggaag    982080
     cttgcacagg ccccaaggaa agagctttgg cgggtgtgca agaggggatg caggcagagc    982140
     ctgagcaggg ccttttgctg tttctgcttt cctgtgcaga gagttccata aactggtgtt    982200
     caagatcagt ggctgggaat gagcccagga gggcagtctg tgggaagagc acagggaagg    982260
     aggagcagcc gctatcctac actgtcatct ttcaaaagtt tgccttgtga ccacactatt    982320
     gcatcatggg atgcttaaga gctgatgtag acacagctaa agagagaatc agtgagatga    982380
     atttgcagca tagatctgaa taaactctcc agaatgtgga gcagtacaga agcaaacaca    982440
     cagaaagtgc ctgatgcaag gacaaagttc agtgggcacc ttcaggcatt gctgctgggc    982500
     acagacactc tgaaaagccc tggcaggatc tccctgcgac aaagcagaac cctcaggcaa    982560
     tgccagcccc agagccctcc ctgagagcgt catggggaaa gatgtgcaga acagctgatt    982620
     atcatagact caaactgaga acagagcaaa cgtccatctg aagaacagtc aaataagcaa    982680
     tggtaggttc atgcaatgca aacccagaca gccaggggac aacagtagag ggctacaggc    982740
     ggctttgcgg ttgagttcat gacaatgctg agtaattgga gtaacagagg aaagcccaaa    982800
     aaatactttt aatgtgattt cttctaaata aaatttacac caggcaaaat gaactgtctt    982860
     cttaagggat aaactttccc ctggaaaaac tacaaggaaa attaagaaaa cgatgatcac    982920
     ataaacacag ttgtggttac ttctactggg gaaggaagag ggtatgagct gagacacaca    982980
     gagtcggcaa gtctccaagc aagcacagaa cgaatacatt acagtacctt gaatacagca    983040
     gttaaacttc taaatcgcaa gaagaggaaa atgcacacag ctgtgtttag aaaattctca    983100
     gtccagcact attcataata gcaaagacat taacccaggt tggataaata aatgatgaca    983160
     caggcaattg cacaatgata cagacataca tttagtacat gagacatcga tgatgtatcc    983220
     ccaaagaaat gactttaaag agaaaaggcc tgatgtgtgg tggcactcac ctccctggga    983280
     tccccggaca ggttgcaggc acactgtgtg gcagggcagg ctggtacatg ctggcagctc    983340
     ctggggcctg atgtggagca agcgcagggc tgtatacccc caaggatggc acagtcagtg    983400
     aattccagag agaagcagct cagccacact gcccaggcag agcccgagag ggacgcccac    983460
     gcacagggag gcagagccca gctcctccac agccaccacc acctgtgcac gggccaccac    983520
     cttgcaggca cagagtgggt gctgagagga ggggcaggga caccaggcag ggtgagcacc    983580
     cagagaaaac tgcagaagcc tcacacatcc acctcagcct cccctgacct ggacctcacc    983640
     tggtctggac ctcacctggc ctgggcctca cctgacctgg acctcacctg gcctgggctt    983700
     cacctgacct ggacctcacc tggcctccgg cctcacctgc acctgctcca ggtcttgctg    983760
     gaacctgagt agcactgagg ctgcagaagc tcatccaggg ttggggaatg actctggaac    983820
     tctcccacat ctgacctttc tgggtggagg catctggtgg ccctgggaat ataaaaagcc    983880
     ccagaatggt gcctgcgtga tttgggggca atttatgaac ccgaaaggac atggccatgg    983940
     ggtgggtagg gacataggga cagatgccag cctgaggtgg agcctcagga cacagttgga    984000
     cgcggacact atccacataa gcgagggaca gacccgagtg ttcctgcagt agacctgaga    984060
     gcgctgggcc cacagcctcc cctcggtgcc ctgctgcctc ctcaggtcag ccctggacat    984120
     cccgggtttc cccaggccag atggtaggtt tgaagtgagg tctgtgtcac tgtggtatta    984180
     tgattacgtt tgggggagtt atcgttatac ccacagcatc acacggtcca tcagaaaccc    984240
     atgccacagc cctccccgca ggggaccgcc gcgtgccatg ttacgatttt gatcgaggac    984300
     acagcgccat gggtatggtg gctaccacag cagtgcagcc catgacccaa acacacaggg    984360
     cagcaggcac aatggacagg cctgtgagtg accatgctgg gctccagccc gccagccccg    984420
     gagaccatga aacagatggc caaggtcacc ccacagttca gccagacatg gctccgtggg    984480
     gtctgcatcg ctgctgccct ctaacaccag cccagatggg gacaaggcca accccacatt    984540
     accatctcct gctgtccacc cagtggtccc agaagcccct ccctcatggc tgagccacat    984600
     gtgtgaaccc tgagagcacc ccatgtcaga gtaggggcag cagaagggcg gggctggccc    984660
     tgtgcactgt ccctgcaccc atggtccctc gcctgcctgg ccctgacacc tgagcctctt    984720
     ctgagtcatt tctaagatag aagacattcc cggggacagc cggagctggg cgtcgctcat    984780
     cccgcccggc cgtcctgagt cctgcttgtt tccagacctc accagggaag ccaacagagg    984840
     actcacctca cacagtcaga gacaaagaac cttccagaaa tccctgtctc actccccagt    984900
     gggcaccttc ttccaggaca ttcctcggtc gcatcacagc aggcacccac atctggatca    984960
     ggacggcccc cagaacacaa gatggcccat ggggacagcc ccacaaccca ggccttccca    985020
     gacccctaaa aggcgtccca ccccctgcac ctgccccagg gctaaaaatc caggaggctt    985080
     gactcccgca taccctccag ccagacatca cctcagcccc ctcctggagg ggacaggagc    985140
     ccgggagggt gagtcagacc cacctgccct cgatggcagg cggggaagat tcagaaaggc    985200
     ctgagatccc caggacgcag caccactgtc aatgggggcc ccagacgcct ggaccagggc    985260
     ctgcgtggga aaggccgctg ggcacactca ggggcttttt gtgaaggccc ctcctactgt    985320
     gtgactacgg tgactaccac agtgatgaaa ctagcagcaa aaactggccg gacacccagg    985380
     gaccatgcac acttctcagc ttggagctct ccaggaccag aagagtcagg tctgagggtt    985440
     tgtagccaga ccctcggcct ctagggacac cctggccatc acagcggatg ggctggtgcc    985500
     ccacatgcca tctgctccaa acaggggctt cagagggctc tgaggtgact tcactcatga    985560
     ccacaggtgc cctggcccct tccccgccag ctacaccgaa ccctgtccca acagctgccc    985620
     cagttccaac agccaattcc tggggcccag aattgctgta gacaccagcc tcgttccagc    985680
     acctcctgcc aattgcctgg attcacatcc tggctggaat caagagggca gcatccgcca    985740
     ggctcccaac aggcaggact cccgcacacc ctcctctgag aggccgctgt gttccgcagg    985800
     gccaggccct ggacagttcc cctcacctgc cactagagaa acacctgcca ttgtcgtccc    985860
     cacctggaaa agaccactcg tggagccccc agccccaggt acagctgtag agagactccc    985920
     cgagggatct aagaaggagc catgcgcagt tctgccggga ccctcggcca ggccgacagg    985980
     agtggacact ggagctgggc ccacactggg ccacatagga gctcaccagt gagggcagga    986040
     gagcacatgc cggggagcac ccagcctcct gctgaccaga ggcccgtccc agagcccagg    986100
     aggctgcaga ggcctctcca gggggacact gtgcatgtct ggtccctgag cagcccccca    986160
     cgtccccagt cctgggggcc cctggcacag ctgtctggac cctccctctt ccctgggaag    986220
     ctcctcctga cagccccgcc tccagttcca ggtgtggtta ttgtcagggg gtgtcagact    986280
     gtggtggata cagctatggt taccacagtg gtgctgccca tagcagcaac caggccaagt    986340
     agacaggccc ctgctgtgca gccccaggcc tccagctcac ctgcttctcc tggggctctc    986400
     aaggtcactg ttgtctgtac tctgccctct gtggggaggg ttccctcagt gggaggtctg    986460
     ttctcaacat cccagggcct catgtctgca cggaaggcca atggatgggc aacctcacat    986520
     gccgcggcta agatagggtg ggcagcctgg cgggggacag tacatactgc tggggtgtct    986580
     gtcactgtgc ctagtggggc actggctccc aaacaacgca gtcctcgcca aaatccccac    986640
     agcctcccct gctaggggct ggcctgatct cctgcagtcc taggaggctg ctgacctcca    986700
     gaatgtctcc gtccccagtt ccagggcgag agcagatccc aggccggctg cagactggga    986760
     ggccaccccc tccttcccag ggttcactgg aggtgaccaa ggtaggaaat ggccttaaca    986820
     cagggatgac tgcgccatcc cccaacagag tcagccccct cctgctctgt accccgcacc    986880
     ccccaggcca gtccacgaaa accagggccc cacatcagag tcactgcctg gcccggccct    986940
     ggggcggacc cctcagcccc caccctgtct agaggacttg gggggacagg acacaggccc    987000
     tctccttatg gttcccccac ctgcctccgg ccgggaccct tggggtgtgg acagaaagga    987060
     cacctgccta attggccccc aggaacccag aacttctctc cagggacccc agcccgagca    987120
     cccccttacc caggacccag ccctgcccct cctcccctct gctctcctct catcacccca    987180
     tgggaatccg gtatccccag gaagccatca ggaagggctg aaggaggaag cggggccgtg    987240
     caccaccggg caggaggctc cgtcttcgtg aacccaggga agtgccagcc tcctagaggg    987300
     tatggtccac cctgcctggg gctcccaccg tggcaggctg cggggaagga ccagggacgg    987360
     tgtgggggag ggctcagggc cctgcgggtg ctcctccatc ttcggtgagc ctcccccttc    987420
     acccaccgtc ccgcccacct cctctccacc ctggctgcac gtcttccaca ccatcctgag    987480
     tcctacctac accagagcca gcaaagccag tgcagacaaa ggctggggtg caggggggct    987540
     gccagggcag cttcggggag ggaaggatgg agggagggga ggtcagtgaa gaggccccct    987600
     tcccctgggt ccaggatcct cctctgggac ccccggatcc catcccctcc tggctctggg    987660
     aggagaagca ggatgggaga atctgtgcgg gaccctctca cagtggaata tccccacagc    987720
     ggctcaggcc agacccaaaa gcccctcagt gagccctcca ctgcagtcct gggcctgggt    987780
     agcagcccct cccacagagg acagacccag caccccgaag aagtcctgcc agggggagct    987840
     cagagccatg aaagagcagg atatggggtc cccgatacag gcacagacct cagctccatc    987900
     caggcccacc gggacccacc atgggaggaa cacctgtctc cgggttgtga ggtagctggc    987960
     ctctgtctcg gaccccactc cagacaccag acagaggggc aggcccccca aaaccagggt    988020
     tgagggatga tccgtcaagg cagacaagac caaggggcac tgaccccagc aagggaaggc    988080
     tcccaaacag acgaggaggt ttctgaagct gtctgtatca cagtggggta tagcagtggc    988140
     tggtaccaca gtgacactcg ccaggccaga aaccccgtcc caagtcagcg gaagcagaga    988200
     gagcagggag gacacgttta ggatctgagg ccgcacctga cacccagggc agcagacgtc    988260
     tcccctccag ggcaccctcc accgtcctgc gtttcttcaa gaataggggc ggcctgaggg    988320
     ggtccagggc caggcgatag gtcccctcta ccccaaggag gagccaggca ggacccgagc    988380
     accgtcccca ttgaggctga cctgcccaga cgggcctggg cccaccccac acaccggggc    988440
     ggaatgtgtg caggccccag tctctgtggg tgttccgcta gctggggccc ccagtgctca    988500
     ccccacacct aaagcgagcc ccagcctcca gagcccccta agcattcccc gcccagcagc    988560
     ccagcccctg cccccaccca ggaggcccca gagctcaggg cgcctggtcg gattctgaac    988620
     agccccgagt cacagtgggt ataactggaa cgaccaccgt gagaaaaact gtgtccaaaa    988680
     ctgactcctg gcagcagtcg gaggccccgc cagagagggg agcagccggc ctgaacccat    988740
     gtcctgccgg ttcccatgac ccccagcacc cagagcccca cggtgtcccc gttggataat    988800
     gaggacaagg gctgggggct ccggtggttt gcggcaggga cttgatcaca tccttctgct    988860
     gtggccccat tgcctctggc tggagttgac ccttctgaca agtgtcctca gaaagacagg    988920
     gatcaccggc acctcccaat atcaacccca ggcagcacag acacaaaccc cacatccaga    988980
     gccaactcca ggagcagaga caccccaaca ctctggggga ccccaaccgt gataactccc    989040
     cactggaatc cgccccagag tctaccagga ccaaaggccc tgccctgtct ctgtccctca    989100
     ctcagggcct cctgcagggc gagcgcttgg gagcagactc ggtcttaggg gacaccactg    989160
     tgggccccaa ctttgatgag gccactgacc cttccttcct ttcctggggc agcacagact    989220
     ttggggtctg ggcagggaag aactactggc tggtggccaa tcacagagcc cccaggccga    989280
     ggtggcccca agaaggccct caggaggtgg ccactccact tcctcccagc tggaccccag    989340
     gtcctcccca agataggggt gccatccaag gcaggtcctc catggagccc ccttcagact    989400
     cctcccggga ccccactgga cctcagtccc tgctctggga atgcagccac cacaagcaca    989460
     ccaggaagcc caggcccagc caccctgcag tgggcaagcc cacactctgg agcagagcag    989520
     ggtgcgtctg ggaggggcta acctccccac cccccacccc ccatctgcac acagccacct    989580
     accactgccc agaccctctg caggagggcc aagccaccat ggggtatgga cttagggtct    989640
     cactcacgtg cctcccctcc tgggagaagg ggcctcatgc ccagatccct gcagcactag    989700
     acacagctgg aggcagtggc cccagggcca ccctgacctg gcatctaagg ctgctccagc    989760
     ccagacagca ctgccgttcc tgggaagcct gggctccacc agaccacagg tccagggcac    989820
     agcccacagg agccacccac acacagctca caggaagaag ataagctcca gaccccaggg    989880
     cgggacctgc cttcctgcca ccacttacac acaggccagg gagctgttcc cacacagatc    989940
     aaccccaaac cgggactgcc tggcactagg gtcactgcca tttccctctc cattccctcc    990000
     cagtgcctct gtgctccctc cttctgggga acaccctgtg cagcccctcc ctgcagccca    990060
     cacgctgggg agaccccacc ctgcctcggg ccttttctac ctgctgcact tgccgcccac    990120
     ccaaacaacc ctgggtacgt gaccctgcag tcctcaccct gatctgcaac cagacccctg    990180
     tccctccctc taaacacccc tcccaggcca actctgcacc tgcaggccct ccgctcttct    990240
     gccacaagag cctcaggttt tcctacctgt gcccaccccc taacccctcc tgcccacaac    990300
     ttgagttctt cctctcctgg agcccttgag ccatggcact gaccctacac tcccacccac    990360
     acactgccca tgccatcacc ttcctcctgg acactctgac cccgctcccc tccctctcag    990420
     acccggccct ggtatttcca ggacaaaggc tcacccaagt cttccccatg caggcccttg    990480
     ccctcactgc ctggttacac gggagcctcc tgtgcgcaga agcagggagc tcagctcttc    990540
     cacaggcaga aggcactgaa agaaatcggc ctccagtgcc ttgacacacg tccgcctgtg    990600
     tctctcactg cctgcacctg cagggaggct ccgcactccc tctaaagatg agggatccag    990660
     gcagcaacat cacgggagaa tgcagggctc ccagacagcc cagccctctc gcaggcctct    990720
     cctgggaaga gacctgcagc caccactgaa cagccacgga ggtcgctgga tagtaaccga    990780
     gtcagtgacc gacctggagg gcaggggagc agtgaaccgg agcccatacc atagggacag    990840
     agaccagccg ctaacatccc gagcccctca ctggcggccc cagaacaccc cgtggaaaga    990900
     gaacagaccc acagtcccac ctggaacagg gcagacactg ctgagccccc agcaccagcc    990960
     ccaagaaaca ctaggcaaca gcatcagagg gggctcctga gaaagagagg aggggaggtc    991020
     tccttcacca tcaaatgctt cccttgacca aaaacagggt ccacgcaact cccccaggac    991080
     aaaggaggag ccccctgtac agcactgggc tcagagtcct ctctgagaca ggctcagttt    991140
     cagacaacaa cccgctggaa tgcacagtct cagcaggaga gccaggccag agccagcaag    991200
     aggagactcg gtgacaccag tctcctgtag ggacaggagg attttgtggg ggttcgtgtc    991260
     actgtgagca tattgtggtg gtgactgcta ttcccacagt gacacaaccc cattcctaaa    991320
     gccctactgc aaacgcaccc actcctggga ctgaggggct gggggagcat ctgggaagta    991380
     tggcctaggg gtgtccatca atgcccaaaa tgcaccagac tctccccaag acatcacccc    991440
     accagccagt gagcagagta aacagaaaat gagaagcagc tgggaagctt gcacaggccc    991500
     caaggaaaga gctttggcag gtgtgcaaga ggggatgtgg gcagagcctc agcagggcct    991560
     tttgctgttt ctgctttcct gtgcagagag ttccataaac tggtattcaa gatcaatggc    991620
     tgggagtgag cccaggagga cagtgtggga agagcacagg gaaggaggag cagccgctat    991680
     cctacactgt catcttttga aagtttgccc tgtgcccaca atgctgcatc atgggatgct    991740
     taacagctga tgtagacaca gctaaagaga gaatcagtga aatgcatttg cagcacagat    991800
     ctgaataaat cctccagaat gtggagcagc acagaagcaa gcacacagaa agtgcctgat    991860
     gccaaggcaa agttcagtgg gcaccttcag gcattgctgc tgggcacaga cactctgaaa    991920
     agcactggca ggaactgcct gtgacaaagc agaaccctca ggcaatgcca gccctagagc    991980
     ccttcctgag aacctcatgg gcaaagatgt gcagaacagc tgtttgtcat agccccaaac    992040
     tatggggctg gacaaagcaa acgtccatct gaaggagaac agacaaataa acgatggcag    992100
     gttcatgaaa tacaaactag gacagccaga ggacaacagt agagagctac aggcggcttt    992160
     gcggttgagt tcatgacaat gctgagtaat tggagtaaca gaggaaagcc caaaaaatac    992220
     ttttaatgtg atttcttcta aataaaattt acacccggca aaatgaacta tcttcttaag    992280
     ggataaactt tcccctggaa aaactataag gaaaatcaag aaaacgatga tcacataaac    992340
     acagtggtgg ttacttctac tggggaagga agagggtatg agctgagaca cacagagtcg    992400
     gcaagtctcc taacaagaac agaacaaata cattacagta ccttgaaaac agcagttaaa    992460
     cttctaaatc gcaagaagag gaaaatgcac acacctgtgt ttagaaaatt ctcagtccag    992520
     cactgttcat aatagcaaag acattaaccc aggttggata aataagcgat gacacaggca    992580
     attgcacaat gatacagaca tacattcagt atatgagaca tcgatgatgt atccccaaag    992640
     aaatgacttt aaagagaaaa ggcctgatgt gtggtggcaa tcacctccct gggcatcccc    992700
     ggacaggctg caggctcact gtgtggcagg gcaggcaggc acctgctggc agctcctggg    992760
     gcctgatgtg gagcaggcac agagctgtat atccccaagg aaggtacagt cagtgcattc    992820
     cagagagaag caactcagcc acactccctg gccagaaccc aagatgcaca cccatgcaca    992880
     gggaggcaga gcccagcacc tccgcagcca ccaccacctg cgcacgggcc accaccttgc    992940
     aggcacagag tgggtgctga gaggaggggc agggacacca ggcagggtga gcacccagag    993000
     aaaactgcag aagcctcaca catccacctc agcctcccct gacctggacc tcacctggcc    993060
     tgggcctcac ctgacctgga cctcacctgg cctgggcttc acctggcctg ggcttcacct    993120
     gacctggacc tcacctggcc tcgggcctca cctggcctgg gcttcacctg gcctgggctt    993180
     cacctgacct ggacctcacc tggcctgggc ctcacctgac ctggacctca cctggcctgg    993240
     gcttcacctg gcctgggctt cacctggcct gggcttcacc tgacctggac ctcacctggc    993300
     ctgggcttca cctgacctgg acctcacctg gcctcgggcc tcacctgcac ctgctccagg    993360
     tcttgctgga gcctgagtag cactgaggct gtagggactc atccagggtt ggggaatgac    993420
     tctgcaactc tcccacatct gacctttctg ggtggaggca cctggtggcc cagggaatat    993480
     aaaaagcccc agaatgatgc ctgtgtgatt tgggggcaat ttatgaaccc gaaaggacat    993540
     ggccatgggg tgggtaggga cagtagggac agatgtcagc ctgaggtgaa gcctcaggac    993600
     acaggtgggc atggacagtg tccacctaag cgagggacag acccgagtgt ccctgcagta    993660
     gacctgagag cgctgggccc acagcctccc ctcggggccc tgctgcctcc tcaggtcagc    993720
     cctggacatc ccgggtttcc ccaggcctgg cggtaggttt gaagtgaggt ctgtgtcact    993780
     gtggtattac tatgatagta gtggttatta ctaccacagt gtcacagagt ccatcaaaaa    993840
     ctcatgcctg ggagcctccc accacagccc tccctgcggg ggaccgctgc atgccgtgtt    993900
     aggattttga tcgaggacac ggcgccatgg gtatggtggc taccacagca gtgcagccca    993960
     tgacccaaac acacggggca gcagaaacaa tggacaggcc cacaagtgac catgatgggc    994020
     tccagcccac cagccccaga gaccatgaaa cagatggcca aggtcaccct acaggtcatc    994080
     cagatctggc tccaaggggt ctgcatcgct gctgccctcc caacgccaaa ccagatggag    994140
     acagggccgg ccccatagca ccatctgctg ccgtccaccc agcagtcccg gaagcccctc    994200
     cctgaacgct gggccacgtg tgtgaaccct gcgagccccc catgtcagag taggggcagc    994260
     aggagggcgg ggctggccct gtgcactgtc actgcccctg tggtccctgg cctgcctggc    994320
     cctgacacct gagcctctcc tgggtcattt ccaagacatt cccagggaca gccggagctg    994380
     ggagtcgctc atcctgcctg gctgtcctga gtcctgctca tttccagacc tcaccaggga    994440
     agccaacaga ggactcacct cacacagtca gagacaatga accttccaga aatccctgtt    994500
     tctctcccca gtgagagaaa ccctcttcca gggtttctct tctctcccac cctcttccag    994560
     gacagtcctc agcagcatca cagcgggaac gcacatctgg atcaggacgg cccccagaac    994620
     acgcgatggc ccatggggac agcccagccc ttcccagacc cctaaaaggt atccccacct    994680
     tgcacctgcc ccagggctca aactccagga ggcctgactc ctgcacaccc tcctgccaga    994740
     tatcacctca gccccctcct ggaggggaca ggagcccggg agggtgagtc agacccacct    994800
     gccctcaatg gcaggcgggg aagattcaga aaggcctgag atccccagga cgcagcacca    994860
     ctgtcaatgg gggccccaga cgcctggacc agggcctgtg tgggaaaggc ctctggccac    994920
     actcaggggc tttttgtgaa gggccctcct gctgtgtgac tacggtggta actcccacag    994980
     tgatgaaacc agcagcaaaa actgaccgga ctcgcagggt ttatgcacac ttctcggctc    995040
     ggagctctcc aggagcacaa gagccaggcc cgagggtttg tgcccagacc ctcggcctct    995100
     agggacaccc gggccatctt agccgatggg ctgatgccct gcacaccgtg tgctgccaaa    995160
     caggggcttc agagggctct gaggtgactt cactcatgac cacaggtgcc ctggtccctt    995220
     cactgccagc tgcaccagac cctgttccga gagatgcccc agttccaaaa gccaattcct    995280
     ggggccggga attactgtag acaccagcct cattccagta cctcctgcca attgcctgga    995340
     ttcccatcct ggctggaatc aagagggcag catccgccag gctcccaaca ggcaggactc    995400
     ccacacaccc tcttctgaga ggccgctgtg ttccgcaggg ccaggccgca gacagttccc    995460
     ctcacctgcc catgtagaaa cacctgccat tgtcgtcccc acctggcaaa gaccacttgt    995520
     ggagccccca gccccaggta cagctgtaga gagagtcctc gaggccccta agaaggagcc    995580
     atgcccagtt ctgctgggac cctcggccag gccgacagga gtggacgctg gagctgggcc    995640
     cacactgggc cacataggag ctcaccagtg agggcaggag agcacatgcc ggggagcacc    995700
     cagcctcctg ctgaccagag acccgtccca gagcccagga ggctgcagag gcctctccag    995760
     ggggacacag ggcatgtctg gtccctgagc agcccccagg ctctctagca ctgggggccc    995820
     ctggcacagc tgtctggacc ctccctgttc cctgggaagc tcctcctgac agccccgcct    995880
     ccagttccag gtgtggttat tgtcaggggg tgccaggccg tggtagagat ggctacaatt    995940
     accacagtgg tgccgcccat agcagcaacc aggccaagta gacagacccc tgccacgcag    996000
     ccccaggcct ccagctcacc tgcttctcct ggggctctca aggctgctgt ctgccctctg    996060
     gccctctgtg gggagggttc cctcagtggg aggtctgtgc tccagggcag ggatgactga    996120
     gatagaaatc aaaggctggc agggaaaggc agcttcccgc cctgagaggt gcaggcagca    996180
     ccacagagcc atggagtcac agagccacgg agcccccagt gtgggcgtgt gagggtgctg    996240
     ggctcccggc aggcccagcc ctgatgggga agcctgcccc gtcccacagc ccaggtcccc    996300
     aggggcagca ggcacagaag ctgccaagct gtgctctacg atcctcatcc ctccagcagc    996360
     atccactcca cagtggggaa actgagcctt ggagaaccac ccagccccct ggaaacaagg    996420
     cggggagccc agacagtggg cccagagcac tgtgtgtatc ctggcactag gtgcagggac    996480
     cacccggaga tccccatcac tgagtggcca gcctgcagaa ggacccaacc ccaaccaggc    996540
     cgcttgatta agctccatcc ccctgtcctg ggaacctctt cccagcgcca ccaacagctc    996600
     ggcttcccag gccctcatcc ctccaaggaa ggccaaaggc tgggcctgcc aggggcacag    996660
     taccctccct tgccctggct aagacagggt gggcagacgg ctgcagatag gacatattgc    996720
     tggggcatct tgctctgtga ctactgggta ctggctctca acgcagaccc taccaaaatc    996780
     cccactgcct cccctgctag gggctggcct ggtctcctcc tgctgtccta ggaggctgct    996840
     gacctccagg atggcttctg tccccagttc tagggccaga gcagatccca ggcaggctgt    996900
     aggctgggag gccacccctg tccttgccga ggttcagtgc aggcacccag gacaggaaat    996960
     ggcctgaaca cagggatgac tgtgccatgc cctacctaag tccgcccctt tctactctgc    997020
     aacccccact ccccaggtca gcccatgacg accaacaacc caacaccaga gtcactgcct    997080
     ggccctgccc tggggaggac ccctcagccc ccaccctgtc tagaggactt ggggggacag    997140
     gacacaggcc ctctccttat ggttccccca cctggctcct gccgggaccc ttggggtgtg    997200
     gacagaaagg acgcctgcct aattggcccc caggaaccca gaacttctct ccagggaccc    997260
     cagcccgagc acccccttac ccaggaccca gccctgcccc tcctcccctc tgctctcctc    997320
     tcatcactcc atgggaatcc agaatcccca ggaagccatc aggaagggct gaaggaggaa    997380
     gcggggccgc tgcaccaccg ggcaggaggc tccgtcttcg tgaacccagg gaagtgccag    997440
     cctcctagag ggtatggtcc accctgcctg gggctcccac cgtggcaggc tgcggggaag    997500
     gaccagggac ggtgtggggg agggctcagg tccctgcagg tgctccatct tggatgagcc    997560
     catccctctc acccaccgac ccgcccacct cctctccacc ctggccacac gtcgtccaca    997620
     ccatcctgag tcccacctac accagagccg gcagagccag tgcagacaga ggctggggtg    997680
     caggggggcc gccagggcag ctttggggag ggaggaatgg aggaagggga ggtcagtgaa    997740
     gaggcccccc tcccctgggt ctaggatcca cctttgggac ccccggatcc catcccctcc    997800
     aggctctggg aggagaagca ggatgggaga atctgtgcgg gaccctctca cagtggaata    997860
     cctccacagc ggctcaggcc agatacaaaa gcccctcagt gagccctcca ctgcagtgct    997920
     gggcctgggg gcagcccctc ccacagagga cagacccagc accccgaaga agtcctgcca    997980
     gggggagctc agagccatga aggagcaaga tatggggacc ccaatactgg cacagacctc    998040
     agctccatcc aggcccacca ggacccacca tgggtggaac acctgtctcc ggcccctgct    998100
     ggctgtgagg cagctggcct ctgtctcgga cccccattcc agacaccaga cagagggaca    998160
     ggccccccag aaccagtgtt gagggacacc cctgtccagg gcagccaagt ccaagaggcg    998220
     cgctgagccc agcaagggaa ggcccccaaa caaaccagga ggtttctgaa gctgtctgtg    998280
     tcacagtcgg gtatagcagc ggctaccaca atgacactgg gcaggacaga aaccccatcc    998340
     caagtcagcc gaaggcagag agagcaggca ggacacattt aggatctgag gccacacctg    998400
     acactcaagc caacagatgt ctcccctcca gggcgccctg ccctgttcag tgttcctgag    998460
     aaaacagggg cagcctgagg ggatccaggg ccaggagatg ggtcccctct accccgagga    998520
     ggagccaggc gggaatccca gccccctccc cattgaggcc atcctgccca gaggggcccg    998580
     gacccacccc acacacccag gcagaatgtg tgcaggcctc aggctctgtg ggtgccgcta    998640
     gctggggctg ccagtcctca ccccacacct aaggtgagcc acagccgcca gagcctccac    998700
     aggagacccc acgcagcagc ccagccccta cccaggaggc cccagagctc agggcgcctg    998760
     ggtggattct gaacagcccc gagtcacggt gggtatagtg ggagctacta ccactgtgag    998820
     aaaagctatg tccaaaactg tctcccggcc actgctggag gcccagccag agaagggacc    998880
     agccgcccga acatacgacc ttcccagccc tcatgacccc cagcacttgg agctccacag    998940
     tgtccccatt ggatggtgag gacgggggcc ggggccatct gcacctccca acatcacccc    999000
     caggcagcac aggcacaaac cccaaatcca gagccgacac caggaacaca gacaccccaa    999060
     taccctgggg gaccctggcc ctggtgactt cccactggga tccacccccg tgtccacctg    999120
     gatcaaagac cccaccgctg tctctgtccc tcactcaggg cctgctgagg ggcgggtgct    999180
     ttggagcaga ctcaggttta ggggccacca ttgtggggcc caacctcgac caggacacag    999240
     atttttcttt cctgccctgg ggcaacacag actttggggt ctgtgcaggg aggaccttct    999300
     ggaaagtcac caagcacaga gccctgactg aggtggtctc aggaagaccc ccaggagggg    999360
     gtttgtgccc cttcctctca tgtggacccc atgcccccca agataggggc atcatgcagg    999420
     gcaggtcctc catgcagcca ccactaggca actccctggc gccggtcccc actgcgcctc    999480
     catcccggct ctggggatgc agccaccatg gccacaccag gcagcccggg tccagcaacc    999540
     ctgcagtgcc caagcccttg gcaggattcc cagaggctgg agcccacccc tcctcatccc    999600
     cccacacctg cacacacaca cctaccccct gcccagtccc cctccaggag ggttggagcc    999660
     gcccataggg tgggcgctcc aggtctcact cactcgcttc ccttcctggg caaaggagcc    999720
     tcgtgccccg gtcccccctg acggcgctgg gcacaggtgt gggtactggg ccccagggct    999780
     cctccagccc cagctgccct gctctccctg ggaggcctgg gcaccaccag accaccagtc    999840
     cagggcacag ccccagggag ccgcccactg ccagctcaca ggaagaagat aagcttcaga    999900
     ccctcagggc cgggagctgc cttcctgcca ccccttcctg ccccagacct ccatgccctc    999960
     ccccaaccac ttacacacaa gccagggagc tgtttccaca cagttcaacc ccaaaccagg   1000020
     acggcctggc actcgggtca ctgccatttc tgtctgcatt cgctcccagc gcccctgtgt   1000080
     tccctccctc ctccctcctt cctttcttcc tgcattgggt tcatgccgca gagtgccagg   1000140
     tgcaggtcag ccctgagctt ggggtcacct cctcactgaa ggcagcctca gggtgcccag   1000200
     gggcaggcag ggtgggggtg aggcttccag ctccaaccgc tccactagcc gagactaagg   1000260
     aagtgagagg cagccagaaa tccagaccat tccatagcaa atggatttca ttaaagttac   1000320
     cagacttcag tgtaagtaac atgagcccca tgcacaacaa tcccttatga aggggaagtc   1000380
     agtgtcgcct cggatttctt gaaaaacaca aaaacttatc aatgcctgta aaagtctgtt   1000440
     ggaaagaaaa tatgattcaa gaatgttatg cccaacaaag ctggcatatt ttctacccgg   1000500
     acacactcag ggaatgtggt cccttgagtg cttctctcac tgcgtaaatc ctacgtggtg   1000560
     tttaagcata ttcataaatg tgtatgtcta tttttatgtg taagatggtt catttttatt   1000620
     ttatttattc aatatgtaca ataaagaata ttgacaaata ggctggacat ggtggctccc   1000680
     acctgtaatc ccagcccttt gggaggccga ggcgggcaga tcacctgagg tctggagttc   1000740
     gagaccagcc tggccaacat gatgaaaacc catctctact aaaaatacaa agattagcca   1000800
     ggcatggtgg tgcatgcctg taatcccagc cactcaggag gctgagacag gagaaatgcg   1000860
     tgaacccgga aggcggaggt tgcagtgagc cgagatcaca ccactgcact ccagcctggc   1000920
     gacagagcaa gattccatct caaaaaaaaa aagacaaaga aatttgtttt tttgaataaa   1000980
     gacaaatttc atcacacgaa gataaagatg caaagctcca gacaggaagg cacggacagc   1001040
     acagtgaagc ccggagcggg cgctgggggg ccaggggcat ggcgggggtg ccagcgtctc   1001100
     tcggtgccta ccatggccac tccagcctgt gttctcacga ggatggctgt gcaatgctag   1001160
     gagcgtgttc gaagctctag ggcaaccact ggaagtgagg ctgaggagca gagcccagag   1001220
     gcccgtggag ctgatgaaaa gaaagctgga gaaagtgttt gctgcctccc aacatggtaa   1001280
     gaaaagatag aaagagagag cacacggcaa agggagcttg ctgagggact ctttacaatg   1001340
     gcttgcacag agctcagggg gtctgggagg ctagggccct gcgcagggca gtcaccccag   1001400
     cctgctgacc aaggtttgct gcaggcagct ctgggggtgg ttgaggcgcg gtccctggag   1001460
     ccacccctca agggaacgag gcagcagagt gggccaaggc ccaggtcggc tgcaaggctg   1001520
     cccaggactt ggggtcctta catcagcagc cactgatgca gctggcccag agagaggcgc   1001580
     cgagcaggtt gcctccaggg gacaaaccag gtcggagagg gtgaggcagt ggatggagcc   1001640
     acaacaaccc cgggcacggg tgacacgcac gttcatgcac atctgaccct tcctccctca   1001700
     ccaaacaggt ccccctgcct tccccatggt tgcgaaaaag caaaatgtag acgttttttc   1001760
     ttttttaatt catgttttaa ttgacaaatg aagccgtata tatttattgt gtacaacatg   1001820
     atgctttaaa atatgtatac atcgtggaac agcaacgttg agctaattta acacgcatta   1001880
     cttcacatac ttgtcatctt ttgtggcgag aatgcttaaa atccactctc ttagtatttt   1001940
     ttaagaatgc aatacattgt tgtcaactgt ggtcaccgtc atgcatagcc aagctcccga   1002000
     cctcaccctc ctgccagctc aggctgtgca tcctttcacc agcatccccc accccggccc   1002060
     ctggccctgg taactaccac tctatactct acgtatgagt tcagcttttt aagattccac   1002120
     agatgaatga gatcatacag tatttgcttt ctatgcctgg cttattttag ttaacacact   1002180
     gtcctccaga tccatccgtt gttgcaaatg acagggtttc attcttttta aagtctaaag   1002240
     agtattccat tgtgtcaatg gacctcattt gctttatcca tgcatcaact atggacattt   1002300
     aggttgattc catttcttag ctgttgtgga tggtgctgca gtaaacatgg ggctgcagat   1002360
     gtctcttcaa catactgaca tcatgtcctt tggataaata cccagtagtg ggatcgctgg   1002420
     atcacaatgt acagtttttt ttttaatgga aactttcatt ttttggtgaa attaggaaaa   1002480
     cagataaaac ccacagaatc caaaatatat gtgaagatgc caaaaacagt tgacattggg   1002540
     cagaggtcac atggaaggaa gtgaatacat gacggggtgt gagggcccag aggcagctga   1002600
     aatacgcttt ctaaacacaa ggacctcttc tgagagggca gaagttttat cctgcacatg   1002660
     caatgaccag cacagctaaa atacactttc taaacatgag gacctcttct gagagggcag   1002720
     ctttatcctg caaatgcaat gaccagcaca ggacccagaa taaagagagt tgccagcgga   1002780
     cgcctggtgt ccatgtgtcc aggtgagttc gagatgcgga cggcgctggc cagccagtca   1002840
     caccctaagt caatctgctg catgcatttg tccttgccac agcagaaaac gagaaagcct   1002900
     ttgggctgca aagcttcaca ggctcctctt ctcccgactc catggaaaca gctacaaaga   1002960
     gcaggcccag tagagcttaa ttcatgaaaa tgagtaataa acttgaactg gaacagtatc   1003020
     gactttttag aaacggcagc aaagtgtata aaaaatattc accagaacaa tatttccaaa   1003080
     cgatgagatg agaatttcag ccaagtaatc ctccatggat agaaaataat gaagggattg   1003140
     gatttatgaa ggaaaatcat ggagctcaaa tacaagagaa gagaatcaaa aatgaacagg   1003200
     aggagataaa atatggtttg gccaaagtta caaaataaat tttttaaaaa cccttcatca   1003260
     tggcaagtag aaagagcgag aggaaaaaca gatcccgtgg aagacacaaa taggacatgg   1003320
     ggagaaaaat gaatgagatg aaacagagca gaaataaaat tttacggaac taaagacaag   1003380
     tgatctgaac ctgcctgggg cctgggggac ctcgccaccc tgaagggaaa gaacatgcct   1003440
     ggctggcttt gccacctgct cattgcagag ccccacagct tgcaacaaac ataggcggta   1003500
     gccagggagt ggttacagca ggccttgagc aagacccagt gttgtgctga cttcaggtct   1003560
     gacccagcac tgtcatagtg gtggtgtcca tagtggtagt gggggtgctt gtgtcactcc   1003620
     acccccatct ccaggaggct cagaacagac agagagagac tccatttgtt tgggagaaag   1003680
     taagggatga gaacaagagt ctctgcctgg taatccagag aattattcta gatcttggcc   1003740
     aagattatca aagcagtacc tctatgagtc ttttgggctt ggagtccccc taaagcagat   1003800
     atagctaaga tcacaacacc caagtccttt tgaatatgtg ggaagacttc ccaaggacag   1003860
     gagcaaacaa acaagcccag actgcaaaaa aacaagccca gactgcaata aacacctcac   1003920
     tcttcaatgc ccaggcactg aagaacatct cctagcagca acaccatcca ggaaaacatg   1003980
     gcctcaacca gtgaactaaa taaggcacca gggaccagtc tcggagaaat agaggtatgt   1004040
     tatctttcag agaattcaaa gtagctttgt tgaggaaact caaagaaatt caagataaca   1004100
     cagtgaagga attcagaatc ctatccgata aatttaacag agattgaagc aattaaaaag   1004160
     aattaagcag aaattatgga gctgaaaaat gcaattggca tactgaaaaa tgcatcagag   1004220
     tattttcata gcctcttata tcaagtagaa gaaagaatta gtgagcttga aaacaggcta   1004280
     tttggaaaag cacgataaaa ggagacaaaa gagaaaagaa taaataacaa tgaagcatat   1004340
     ctacaggatc tagaaaatag cctcaaaagg ccaaatctaa gaattattag ccttaaagag   1004400
     gaggtagaga aagagggatg gagagtttat tcaaagggat aataacagaa aacttcccaa   1004460
     acctagagaa agatatcaat atccaaatgc aagaaggatg tagtacacca aggagattta   1004520
     atgcaaagaa gactacctca aggcattcaa tactcaaact cccatatgac aaggacttta   1004580
     aaaagatcct aaaagcagca aaagaaaaga aatgaataaa atactatgga gctccaatat   1004640
     gtctggcagc agacttttca gtgaagactt tatatgccag gagagagtgt cataatggat   1004700
     ttaaagtgct gaaggaaaaa acttttaccc tcgaacagta tagctggtga aattatcctt   1004760
     caaacatgaa ggagaaataa tttgtttcca gacaaatgtt gagggatttc atgaacacca   1004820
     gacctgtctt ttaagaaatg ctaaagggag tacttcaatc agaaagaaac acgttagtga   1004880
     acaataagaa atcatctgaa ggcacaaaac tcaccggtaa tagtaagtac acagaaaaac   1004940
     acagaatatt ataacactgt aactgtggtg tgtaaactcc ttttgtttgt ttgtttgttt   1005000
     gtttgtttgt ttgtttgttt ttgtttttag acggagtttt gctccagccc aggctggagt   1005060
     gcaatggcac aatctcagct cactgcaact tccacctccc gggttcaagc aattctcctg   1005120
     cctcagcctc ccaagtagct gggattacag gcatgtgcta ccatgtccag ctaattttgt   1005180
     attttagtag agacggtgtt tcaccatgtt ggtcaggcta gccttatctt gagtagaaaa   1005240
     actaaatgat gaagcaatga aaaataataa ctacaacttt tcaagacata gtacaataag   1005300
     atataaatca taacaaaaag ttaaaaggtg gagggatgaa gttaaggcaa agagtcttta   1005360
     ttagttttct ttttacttgt ctgtttatgc aaacagtgtt aagttgtcat cagtttaaaa   1005420
     taatgggtca taagatacta tttgcaagcc tcatggtaac gtcaaaccaa aagcaataca   1005480
     acagatacac aaaaaacaaa aagcaagaag ctaaattacg tcatcagaga aaatcacctt   1005540
     cactaaaagg aagacggaga aaagaatgaa gagagagaag accaaaagca aatagcaata   1005600
     tggcaggagt aagtccttac ttatcaataa taccattgaa tgtaaatgga ctaaactctc   1005660
     caatcaaaag acatagagtg gctgaatcaa ttaaagaaaa aacaagaccc attgatctgt   1005720
     tgtccacaag aaacacactt tatctataaa gacacacata gactgaaaac aaagggatgg   1005780
     aaaaagatac tccacgccaa tggaaaccaa agaaagagca ggagtagcta cacttatatc   1005840
     aggcaaaata gatttcaaga caaaaactat aagaagagac aaggtcacta atgataaaca   1005900
     ggtcaattca gcaagaggat ataacaattg taaatatata tgcacccaat gctggagcac   1005960
     ccagatatat aaagcaagta tttactagag ctaaagagag aaatagactc caatgcaata   1006020
     atagctggag atttcaacat cccactttca acattgaaca gatcctccag atagaaaatc   1006080
     aacaaagaaa tattggactt aatctgcact atcgaccaaa tggatctaac agatatttac   1006140
     agaacatttc atccaacagc tgcagaacac acattctttt cctcagcaca tagatcattc   1006200
     tcaaggatag accatatgtt gggtcacaaa acaagtttta aaatattcaa atacattgaa   1006260
     ataatatcaa gcatcttctg tgaccacaat ggactaaaac tagaaatcaa taacaagagg   1006320
     aattttggaa actatataaa tatatggaaa ttaatgaatg ctgagtgggt caatgaagca   1006380
     attaagaagg aaactgaaat ttttcttgga acgaatgatc atggaaacag aaaataccaa   1006440
     aacctatggg atacagcaaa agcagtacta agagggaagt ttacagctac aaatgcttac   1006500
     attaaaaaag aagaaaaact tcaataaaaa aacctaacaa tgcatcttaa agaactagaa   1006560
     aagcaagagg aaatcaaatc caaaattagt agaagaaaac agtaaaggtc agagcagaaa   1006620
     taagtaaaat tgaaatgaag aaaacaatac aaaagatcaa taaaacaaca ggttgttttc   1006680
     ttgaaaagtt aaacaaaatt gacaaacctt tagccagact aagaaaaaaa gacagaagat   1006740
     ccaaataaat aaaatcagag atgaaaaagg tgacattaca acttacacca cagaaattca   1006800
     aaggatcatt agtggctact ataagcaact atatgccaat aaattggaaa atctagaaga   1006860
     aatgcagaaa ttcctagaca catacaacct cccaagatta aaccaagaag aaattcaaaa   1006920
     cctgaacaga ctgataacaa gtaatgagat caaagccgta ataaaaagcc tcccagtaaa   1006980
     gagaagccca ggacccgacg gcttcactgc tgaattctac caaacattta aagtagaact   1007040
     aataccaatc ctactcaaac tattccaaaa aatagaggtg gaaggaatac ttcaaaactc   1007100
     attatacgag gccagtatta acctgacacc aaaactagac aaagacacat gaaaaaaaga   1007160
     aaactacagg ccaatatgtc tgatgaatat tgacacaaaa atcctcaaca aaatactagc   1007220
     aaaccaaatt caactacaca ttagaaagtt cactcatcat gaccaagtgg aatttatcta   1007280
     acttgggatg caaagatggt tcaacatatg caaatcaatc aatgtgatac atcatatcaa   1007340
     cagaatgaac aacaaaaacc atttgatcat ttaattgata ctgaaaaagc atttgataaa   1007400
     attcaacatt ccttcataat aaaaattctc ttctatacta ggtacaaaag aaacttacct   1007460
     caacataata aagccatata tgacagtccc acagtatgat actaaatgag gaaaaactga   1007520
     gagcctttcc tctacgatct ggaacatgac aaagatgccc actttcatca ctgttattca   1007580
     acatagtact ggaagtccta gctggagcga tcagacaaga gaaagatata aaagacatcc   1007640
     aaattggaaa ggaataagtc aaattatcct catttgcata tggtatgatc ttctatttag   1007700
     agctaactaa agactccacc aaaaaaagtt attagaactg acgaacaaat tcagtaaagc   1007760
     tgcaggatac aaaatcaaca tacaaaaatc agtagcattt ctatatgcca acaatgacca   1007820
     atgtgaaaaa gaaattaaaa agtaacccta tttacaataa ccacaaataa acacctagga   1007880
     attaaccaaa gaggtaaaag atttctgtaa tgaaaactat aaaaactgat gaaagaaatt   1007940
     gaagagtaca ccaaaaaatg gaaagcaatt gcatgttcat ggattagaag aatcagtgtt   1008000
     gttataatgt ccatactatc caaagcaatc tacagattca atgcaatcct tatcaaaata   1008060
     ccaatgacat cattcacaga aatagaaaaa aaaaatccta aaatttacgt ggaaccacaa   1008120
     agacccagaa tagccaaagc tctcctaagc aaaaagaacg aaactgtagg aatgacattg   1008180
     cctgtcttca aattctacta cagagctata gatagtaacc aaaacagcgt ggtactagca   1008240
     taaaaacaga cacagagaca aacagaacaa aatttaaaaa cccagaaata aatccacaca   1008300
     cctacagcaa attcattttt gacaaagttg ccaagaacat actctgggga atagataatg   1008360
     atatctcttc aataaataat gtggggaaaa ctggatatcc atatacataa cagtgaaact   1008420
     agacccctct ctctctcact atatacaaaa atcaaatcaa aattgtttaa ggacttaaat   1008480
     ctaagacctc atactatgaa accactgcaa gacaaccttg gcggaaactc tccaagacat   1008540
     cagtccaggc aaagatttct tgagtaatat cccacaagca cagacaacca aagcaaaaat   1008600
     ggacaaatgg gatcacatca agttaaaaag cttctgcaca gtaagggaaa caaccaacaa   1008660
     aatgaagaga caacccacag aatgggagaa aatatttgaa aaatacccat ctggcaaggg   1008720
     attaaaaacc agaatatatg cagaatatat aaggagctca aacagtgcta tagaaaaaaa   1008780
     aatctaataa tctgatttaa aaatgggaaa aatgttagaa tagacatttc ttaaaataag   1008840
     acatacagat ggcaaaccga catggaacgg tgctcaacat catggattat cacagaaaca   1008900
     caatcaatca aaactaaaac taaaatgtgc tatcatctca ccccagttaa aatggctgat   1008960
     atccagaaga caggcaataa caaatgctgg caaggatgtg gggaaaaggg agcccccata   1009020
     cactgttgct gggattgtaa attagtacaa ccactgtgga gagcagcatg aaagttcctc   1009080
     aaaaaactga aagaaagcta ccataggatc cagcaatccc actgctgtgt atatactaca   1009140
     aaagaaagga agtcagtata tgaagaggta tctgcactcc catgtttgtt gcagccctgt   1009200
     tcacaacagc caagatttgg aagcaaccta agtgtccatc agcagttgaa tgtataaaga   1009260
     aaatgtggtg catatacaca atggagtatt attcaataat aaaaaggaat gagattgagt   1009320
     catttgcaac aacatggatg gaactggaga tcattatgtg aagtgaaata agccaggcac   1009380
     agaaagacaa acattacaat gttcttactt attaatgaga tctaaaaatc aaaacaattg   1009440
     cacccatgtt cataaagagt aaaaggatgg ttaccagatg ctgagaacgg tggtgggggg   1009500
     atagggaaag gtggcagtgg ttaacgggta caaaaaaata gaaagaatga ataagactta   1009560
     ctacttgata gcacagcaag gtggctatag tcagtaattt agttgtatat ttttaataat   1009620
     gaaaggtgta taattggatt gtttctaaca caaaggataa tgcttaagag gatggatacc   1009680
     ccattttcca tgatgtgatt atttcacatt gcacgcctag atcaaaacat ccaatgtacc   1009740
     ccataaatat atacatcttc tatgtaccca taaaaattct gtaaaataaa atatataaaa   1009800
     agaggtgaca gatatggaag acaggcaaag aagagacgac atccacataa tccgagtacc   1009860
     taagaaagaa tggagtccag tgcatctcag gagccaccat tctaagccaa ttttctctgg   1009920
     ttctctcagt caccctacta atacgtgggc aatcttgttt tatttcagga tagagttttt   1009980
     gaaattatag atttaagtat gctttctgtt ctattacttt tggtaattaa ttttagaaag   1010040
     aactaatttg ggcacaaatt tgaaaaaatt ctaaatccaa aaaaaaaaag aaaaaaacac   1010100
     acacacaatc atctataagg gggatgatga ccagtcctag atttctcacc agccacattc   1010160
     aagatcagta aatggtagga caaaacctgt agggtcctta agggggaaag aagtagtgga   1010220
     tagtccagag tctatataca gccaactgtt cttgaagaaa aaaggctgct gaaaaggagt   1010280
     tccaaacatt ctataatcca taatctcatg atgaaactac tagaggaaga ccaccagcca   1010340
     tcaaaaggtg cttggagaac ccagggccaa gaaccaaaag taaatattaa gtgtccttaa   1010400
     ctgcgagact aagatagaaa tgactgtggg ggaccatgtg gcctcaacag aggtgaaatg   1010460
     gtgtctgcct gacaaagtgg acattttaca atgatcaaaa cacagaatat gagatagaga   1010520
     gcacttctga attactgcct cactccaaat aactctcagc caaaggactt cagtaaaacc   1010580
     aaattgggca tattagacag tacaaacaaa ttctaagaaa ataatattac tgattacaat   1010640
     cacatgatgc tagagatgga ggggaaaagg aagaggaaac caggtaattt catactcgta   1010700
     tatagtaaag aactaaagta cattgtccaa agaagaacaa agaatatttt ggaaagttat   1010760
     aaaggtagcc actacacata gaagatagca aagaacaaga aaacttaaga tggaaaactt   1010820
     tttggaagca taaaaataga aaatataaac tactaagata agattgaagc caaacagatc   1010880
     tatgaaaaca acaaacatca atggccttaa cttgcctatt aaaaggaaga gactttcaaa   1010940
     ttggaccaca agataaaacc caactctata tagcatatga gtattacaca caaaatggga   1011000
     aaagctgaaa aaacttgggc aaaattcacc ccaagcaaat tccactgttt cctttgggac   1011060
     aaaatgccaa gctccatgcc agggaagatg attctcctca gaccttctcc tcactctccc   1011120
     agtcctctta gggaaggaat tgggtgttag aggagggaga ctctgtcgat tatcagctga   1011180
     agcagtggtg tgctcctgcg ttgcttctga cctgggaaat gaagcagcaa gactctttct   1011240
     gctgtgtctt tgcccagaag ggccatcccc ccagagcaga gtacccaggc cggcaggagc   1011300
     agtggtggaa gcgtggaaac cacgtctcct acagcagaga ccatcagaag cggagcctcg   1011360
     ggtataaggg aaacaacgcg ttctccctaa cctgggagtg acagacagcg tcattcctca   1011420
     cagtgatacc ctgtgttcta gccatctggc ccatgacaga gccagcccag agccagccca   1011480
     gagccagccc ctcaccaacc tggagcctgg ccagctcgcc aagctgcacc ataggcctgg   1011540
     aaggcgtgga gacctgcggc agtgccctgt cctcccgtga ggcctgccat ccctgccagg   1011600
     ggtcgcctct ggcttctcct ccaggaccgc acggtccaga ggctcagtgc ctggagtagg   1011660
     tgttgccccc ctgcttctag gcccagaccc tcccttgttc ctgaccccgg gcctttccct   1011720
     ctggcttgga catccagggc cctgtctcag ctggggagct gctcctgctc aaggactgtc   1011780
     ttccgcggga tcgaaaggcc gcgtcctgaa caatgcgtgg gccacgtgag cggagcaggc   1011840
     tctaaaggcc gcgtcctaaa cagtgcgtgg gccacgtgag cggagcaggc tctaaaggcc   1011900
     gcgtcctaaa cagtgcgtgg gccacgtgag cggagcaggc tctaaaggcc gcgtcctaaa   1011960
     cagtgcgtgg gccacgtgag cggagcaggc tctaaaggcc gcgtcctaaa cagtgcgtgg   1012020
     gccacgtgag cggagcaggc tctaaaggcc gcgtcctaaa cagtgcgtgg gccacgtgag   1012080
     cggagcaggc tctaaaggcc gcgtcctaaa cagtgcgtgg gccacgggag cggagcagac   1012140
     tctaaaggcc gcgtcctaaa cagtgcgtgg gccacgtgag cggagcaggc tctaaaggcc   1012200
     gcgtcctaaa cagtgcgtgg gccacgtgag cggagcaggc tctaaaggcc gcgtcctaaa   1012260
     cagtgcgtgg gccacgtgag cggagcaggc tctaaaggcc gcgtcctaaa cagtgcgtgg   1012320
     gccacgtgag cggagcaggc tctaaaggcc gcgtcctaaa cagtgcgtgg gccacgggag   1012380
     cggagcagac tctaaaggcc gcgtcctaaa cagtgtgtgg gccacgtgag cggagcaggc   1012440
     tctaaaggcc gcgtcctaaa cagtgcgtgg gccacgggag cggagcagac tctaaaggcc   1012500
     gcgtcctaaa cagtgtgtgg gccacgtgag cggagcgccc tctccactgc cctcggggcc   1012560
     gcagctccca gctcagctcc cagccctgct cagggcagcc aggccaggag gtaccatcca   1012620
     ggctaagtga ccctcagggg ggacaggtgc cccaggagat gccagctgtt gggagaggct   1012680
     gggggaccaa ctcgacctgg cctgtgggcc ctgccctggc cacccattgt aggatccagc   1012740
     cgccacgcct gtgacactcg tgtgctttcc ctggtgtgtg cttgtggcag gtgggggcag   1012800
     agggtcctca ggccagagag ccactccccc agcgccagac caccctcttc ctcactcccc   1012860
     cacctcaccc cctcacaggt gcctcccagg ccatcagggc ccaaccaccc ctaaacaaat   1012920
     gggttctcgg cccctcgtgg ctggaggtgg gttctctcac cattcccagc ctaaggctcc   1012980
     atccccatgc tggcagctgt tcaaccatgt ctagagagat ccactgtccc agacagcacc   1013040
     tcagggtccc ccgtcctgcc tggaaccctg taggaaactc cacaaaccgc cgccattctg   1013100
     tccacacccc tacaggagcc ccaaccctct ccccacatcc aggcttccct cccagacccc   1013160
     tcatccctgc ccgcacggtg cctgaggggg ccttcttggg cagcgcctaa gcaagccccc   1013220
     agcacccttc ggccccttca aggcacacag gccccctttc cacccagcct caggaaacca   1013280
     cctgtgtcct ccaacgacag gtcccagcct cccagccttt gccttgcctg ttcctctccc   1013340
     tggaactctg ccccgacaca gaccctcccc agcaagcccg caggggcacc tcccctgccc   1013400
     ccagacaccc tgtgcccgtc agttcatccc cagcagaggc cctcaccagg cacaccccca   1013460
     tgctcacacc tggccgcagg cctcagcctc cctgagggcc ccacccagcc cgcgtctggc   1013520
     cagtggtgcg tgcaaagccc ctcacccaga ctcggcggaa ggcagccagt gcaggcctgg   1013580
     ggaggggctc tccttagacc accttgcacc ttccctggca cccaccatgg gaagagctga   1013640
     gactcactga ggaccagctg aggctcagag aagggaccca gcactggtgg acacgcaggg   1013700
     agcccacgcc agggcgccgt ggtgagtgag gcccagtgcc acccactgag gcctcccgtt   1013760
     cagtgggacg acggtgaaca ggtggaacca accaggcaac ccccgccggg ccccacagac   1013820
     gggatcagag caggaaaggc ttcctgcccc tgcaggccag cgaggagccc tggcgggggc   1013880
     catggccctc caggcgagga ggctcccctg gccaccgcca cccgggcctc tctgctgctg   1013940
     ggaaaacaag tcagaaagca agtggatgag aggtggcgtg acagacccag cttcagatct   1014000
     gctctaattt acaaaagaaa aggaaaaaca cacttggcag ccttcagcac tctaatgatt   1014060
     cttaacagca gcaaattatt ggcacaagac tccagagtga ctggcagggt tgagggctgg   1014120
     ggtctcccgc gtgttttggg gctaacagcg gaagggagag cactggcaaa ggtgctgggg   1014180
     gcccctggac ccgacccgcc ctggagaccg cagccacatc agcccccagc cccacaggcc   1014240
     ccctaccagc cgcagggttt tggctgagct gagaaccact gtgctaactg gggacacagt   1014300
     gattggcagc tctacaaaaa ccatgctccc ccgggacccc gggctgtggg tttctgtagc   1014360
     ccctggctca gggctgactc accgtggctg aatacttcca gcactggggc cagggcaccc   1014420
     tggtcaccgt ctcctcaggt gagtctgctg tctggggata gcggggagcc aggtgtactg   1014480
     ggccaggcaa gggctttggc ttcagacttg gggacaggtg ctcagcaaag gaggtcggca   1014540
     ggagggcgga gggtgtgttt ttgtatggga gaagcaggag ggcagaggct gtgctactgg   1014600
     tacttcgatc tctggggccg tggcaccctg gtcactgtct cctcaggtga gtcccactgc   1014660
     agccccctcc cagtcttctc tgtccaggca ccaggccagg tatctggggt ctgcagccgg   1014720
     cctgggtctg gcctgaggcc acaccagctg ccatccctgg ggtctccgcc atgggctgca   1014780
     tgccagagcc ctgctgtcac ttagccctgg ggccagctgg agcccccaag gacaggcagg   1014840
     gaccccgctg ggcttcagcc ccgtcaggga ccctccacag gtagcaagca ggccgagggc   1014900
     agggacggga aggagaagtt gtgggcagag cctgggctgg ggctgggcgc tggctgttca   1014960
     tgtgccgggg accaggcctg cgctttagtg tggctacaag tgcttggagc actggggcca   1015020
     gggcagcccg gccaccgtct ccctgggaac gtcacccctc cctgcctggg tctcagcccg   1015080
     ggggtctgtg tggctgggga cagggacgcc ggctgcctct gctctgtgct tgggccatgt   1015140
     gacccattcg agtgtcctgc acgggcacag gtttatgtct gggcaggaac agggactgtg   1015200
     tccctgtgtg atgcttttga tatctggggc caagggacaa tggtcaccgt ctcttcaggt   1015260
     aagatggctt tccttctgcc tcctttctct gggcccagcg tcctctgtcc tggagctggg   1015320
     agataatgtc cgggggctcc ttggtctgcg ctgggccatg tggggccctc cggggctcct   1015380
     tctccggctg tttgggacca cgttcagcag aaggcctttc tttgggaact gggactctgc   1015440
     tgctggggca aagggtgggc agagtcatgc ttgtgctggg gacaaaatga ccttgggaca   1015500
     cggggctggc tgccacggcc ggcccgggac agtcggagag tcaggttttt gtgcacccct   1015560
     taatggggcc tcccacaatg tgactacttt gactactggg gccagggaac cctggtcacc   1015620
     gtctcctcag gtgagtcctc acaacctctc tcctgcttta actctgaagg gttttgctgc   1015680
     atttttgggg ggaaataagc gtgctgggtc tcctgccaag agagccccgg agcagcctgg   1015740
     ggggctcagg aggatgccct gaggcaacag cggccacaca gacgaggggc aagggctcca   1015800
     gatgctcctt cctcctgagc ccagcagcac gggtctctct gtggccaggg ccaccctggg   1015860
     cctctggggt ccaatgtcca acaacccccg ggccctcccc gggctcagtc tgagagggtc   1015920
     ccagggactt agcggggtgc cagttcttgc ctggggtcct ggcattgttg tcacaatgtg   1015980
     acaactggtt cgacccctgg ggccagggaa ccctggtcac cgtctcctca ggtgagtcct   1016040
     caccaccccc tctctgagtc cacttaggga gactcagctt gccagggtct cagggtcaga   1016100
     gtcttggagg cattttggag gtcaggaaag aaagctgggg agagggaccc ttcgaatggg   1016160
     aacccagcct gtcctcccca agtccggcca cagatgtcgg cagctggggg gctccttcgg   1016220
     ctggtctggg gtgacctctc tccgcttcac ctggagcatt ctcaggggct gtcgtgatga   1016280
     ttgcgtggtg ggactctgtc ccgctccaag gcacccgctc tctgggacgg gtgccccccg   1016340
     gggtttttgg actcctgggg gtgacttagc agccgtctgc ttgcagttgg acttcccagg   1016400
     ccgacagtgg tctggcttct gaggggtcag gccagaatgt ggggtacgtg ggaggccagc   1016460
     agagggttcc atgagaaggg caggacaggg ccacggacag tcagcttcca tgtgacgccc   1016520
     ggagacagaa ggtctctggg tggctgggtt tttgtggggt gaggatggac attctgccat   1016580
     tgtgattact actactacta ctacatggac gtctggggca aagggaccac ggtcaccgtc   1016640
     tcctcaggta agaatggcca ctctagggcc tttgttttct gctactgcct gtggggtttc   1016700
     ctgagcattg caggttggtc ctcggggcat gttccgaggg gacctgggcg gactggccag   1016760
     gaggggatgg gcactggggt gccttgagga tctgggagcc tctgtggatt ttccgatgcc   1016820
     tttggaaaat gggactcagg ttgggtgcgt ctgatggagt aactgagcct gggggcttgg   1016880
     ggagccacat ttggacgaga tgcctgaaca aaccaggggt cttagtgatg gctgaggaat   1016940
     gtgtctcagg agcggtgtct gtaggactgc aagatcgctg cacagcagcg aatcgtgaaa   1017000
     tattttcttt agaattatga ggtgcgctgt gtgtcaacct gcatcttaaa ttctttattg   1017060
     gctggaaaga gaactgtcgg agtgggtgaa tccagccagg agggacgcgt agccccggtc   1017120
     ttgatgagag cagggttggg ggcaggggta gcccagaaac ggtggctgcc gtcctgacag   1017180
     gggcttaggg aggctccagg acctcagtgc cttgaagctg gtttccatga gaaaaggatt   1017240
     gtttatctta ggaggcatgc ttactgttaa aagacaggat atgtttgaag tggcttctga   1017300
     gaaaaatggt taagaaaatt atgacttaaa aatgtgagag attttcaagt atattaattt   1017360
     ttttaactgt ccaagtattt gaaattctta tcatttgatt aacacccatg agtgatatgt   1017420
     gtctggaatt gaggccaaag caagctcagc taagaaatac tagcacagtg ctgtcggccc   1017480
     cgatgcggga ctgcgttttg accatcataa atcaagttta tttttttaat taattgagcg   1017540
     aagctggaag cagatgatga attagagtca agatggctgc atgggggtct ccggcaccca   1017600
     cagcaggtgg caggaagcag gtcaccgcga gagtctattt taggaagcaa aaaaacacaa   1017660
     ttggtaaatt tatcacttct ggttgtgaag aggtggtttt gcccaggccc agatctgaaa   1017720
     gtgctctact gagcaaaaca acacctggac aatttgcgtt tctaaaataa ggcgaggctg   1017780
     accgaaactg aaaaggcttt ttttaactat ctgaatttca tttccaatct tagcttatca   1017840
     actgctagtt tgtgcaaaca gcatatcaac ttctaaactg cattcatttt taaagtaaga   1017900
     tgtttaagaa attaaacagt cttagggaga gtttatgact gtattcaaaa agttttttaa   1017960
     attagcttgt tatcccttca tgtgataatt aatctcaaat actttttcga tacctcagag   1018020
     cattattttc ataatgactg tgttcacaat ctttttaggt taactcgttt tctctttgtg   1018080
     attaaggaga aacactttga tattctgata gagtggcctt cattttagta tttttcaaga   1018140
     ccacttttca actactcact ttaggataag ttttaggtaa aatgtgcatc attatcctga   1018200
     attatttcag ttaagcatgt tagttggtgg cataagagaa aactcaatca gatagtgctg   1018260
     aagacaggac tgtggagaca ccttagaagg acagattctg ttccgaatca ccgatgcggc   1018320
     gtcagcagga ctggcctagc ggaggctctg ggagggtggc tgccaggccc ggcctgggct   1018380
     ttgggtctcc ccggactacc cagagctggg atgcgtggct tctgctgccg ggccgactgg   1018440
     ctgctcaggc cccagccctt gttaatggac ttggaggaat gattccatgc caaagctttg   1018500
     caaggctcgc agtgaccagg cgcccgacat ggtaagagac aggcagccgc cgctgctgca   1018560
     tttgcttctc ttaaaacttt gtatttgacg tcttatttcc actagaaggg gaactggtct   1018620
     taattgcttg atgaagagca ggagactcat ttatgtgagt cttttgagtg accattgtct   1018680
     gggtcactcc catttaactt tccctaaagc ccatttgaag gagaggtcgc acgagctgct   1018740
     ccacaacctc tgaatgggga tggcatgggt aatgatgctt gagaacatac caagccccac   1018800
     tggcatcgcc cttgtctaag tcattgactg taggtcatca tcgcaccctt gaaagtagcc   1018860
     catgccttcc aaagcgattt atggtaaatg gcagaatttt aagtggcaaa ttcagataaa   1018920
     atgcatttct tggttgtttc caatgatgac tgttatctag agggaattta aaggcagggg   1018980
     tttactgcag actcagaagg gaggggatgc tccgggaagg tggaggctct gagcatctca   1019040
     ataccctcct cttggtgcag aagatatgct gccacttcta gagcaagggg acctgctcat   1019100
     ttttatcaca gcacaggctc ctaaattctt ggtctcattc tcaagatgtt ttaatgactt   1019160
     taaagcagca aagaaatatt ccacccaggt agtggagggt ggtaatgatt ggtaatgctt   1019220
     tggaaccaaa acccaggtgg cgctggggca ggactgcagg gaactggggt atcaagtaga   1019280
     gggagacaaa agatggaagc cagcctggct gtgcaggaac ccggcaatga gatggcttta   1019340
     gctgagacaa gcaggtctgg tgggctgacc atttctggcc atgacaactc catccagctt   1019400
     tcagaaatgg actcagatgg gcaaaactga cctaagctga cctagactaa acaaggctga   1019460
     actgggctga gctgagctga actgggctga gttgaactgg gttgagctga gctgagctga   1019520
     gctgggctaa gttgcaccag gtgagctgag ctgagctggg cttggctgca ctaagctggg   1019580
     ctgagctggg ctgagctggg cagggctggg ctgagctgag ctgggctggg ctgagctggg   1019640
     ctgggctggg ctgggctgag cgggtctgag cggggctgag ctgagctagg ctgggctggg   1019700
     ctgagctggg ctgagctggg ctgagctggg ctgagcaagg ctaggctgag ctgggctgag   1019760
     ctgagctggg ctgagcaagg ctaggctgag ctgagctgag ctgggctgcg ctgagctggg   1019820
     ctgggctgcg ctgagctggg ctgggctgag ctgggctagg ctgggctgag ctgggctgag   1019880
     ctaggctggg ctgggctggg ctgagcgggg ctgagcgggg ctgagctgag ctaggctggg   1019940
     ctgagcgggg ctgagctgag ctaggctggg ctgggctggg ctgagccaag ctgaaccggg   1020000
     ttgagcgtgc tgtgctgggc tgagccaagc taggctgagc tgagccaagt tgagcttagc   1020060
     tgggctgagc taacctgggc agggctgagc tgggctgagc taacctggac tggggctgag   1020120
     ctaacctggg cagagctgag ctgggctgag ctaacctggg ctggggctga gctaacctgg   1020180
     gctgggctca gctgagctga gctacgctgg gctgggctgg gctgagccga gctgaactgg   1020240
     gctgagcagg ctgtgtcggg ctgagccaag ctgggccgag ctcagcagag ctgagccgag   1020300
     ctgagcttag ctgggctgag ctaaccaggg ctgggctgag ctgggctgag ctgagctgag   1020360
     ctgaactggg ctgaacgggc tgaggcgagc agggccgagc tgagcagagc taagccgagg   1020420
     ctgggctggg ctaacctggg ctgggccgag ctgagcagag ctaagccgag gctgggctgg   1020480
     gctaacctgg gctgggctga gctgagctgg gttgggcagg gctgggctgg gctgagctaa   1020540
     gctgaactag ggtgagctgg gccgagccag gctggtttgg gctgagttga gctgacctgg   1020600
     actggggctg agctaacctg ggctgggctc agctgagctg agctacgctg ggctgggctg   1020660
     ggctgagccg agctgaactg ggctgagcag gttgtatcag gctaagccaa gctgggccaa   1020720
     gctcagcaga gccgagccga gctgagctta gctgggctga gctaaccagg gctgggctga   1020780
     gctgggctga gctgagctga gctgaactgg gctgaacggg ctgaggcgag cagggccgag   1020840
     ctgagcagag ctaagccgag gctgggctgg gctaacctgg gctgggctga gctgagctgg   1020900
     gttgggcaag gctgggctgg gttgagttgg gctgagctaa gctgagctag ggtgagctgg   1020960
     gctgagccag gctggattgg gctgagttga gctgacctca actgagctga cctaggctga   1021020
     gctgagctga gctgaagtag gctgtgctgg gctgagctgg gatgacctgg gctgggctga   1021080
     gctgatttgg gctgggttga gcagacctgg gctgagccgg gttgagctga gctgaaccag   1021140
     gatgagctgg gctgagctga gctgggctgg gtggtccagg ctgggctgac ctggaccagg   1021200
     ctgggccagg ttgagctggg ctaagccgag ctgagctgag ctgagctggg atgatctggg   1021260
     ctgggctggg ctgggctgag ctgacctggg ctgggctggg ctgagctgag ctgagctgag   1021320
     ttaagctgag ctgagctgaa ctgggctgtg ctgagctagg ctgggctggg ctgagctggg   1021380
     ctgggctggg ctgagccaga ttgtgcctgg ctgaactgag ctgggctaag ctgagctggg   1021440
     ctgagctggg ctgagctgag ctgggctgag tggggcgggg ctgagctgag ccggactggg   1021500
     ctgggctggg ctcagctgag ctgagctgaa ctgggctggg ctgaactggg ctgggctgag   1021560
     ctgagctgaa ctgggctggg ctgaactggg ctgggctgag ctgagcttgg atgagctggg   1021620
     ctgaactggg ctgggttgag ctgggctggg ctgagttgag ccagactgat ctgggctgag   1021680
     ccgagctggg ttaagccgag ctgggttggg ctgggctggg ttgggctggg ctgagccgga   1021740
     ctgggttggg ctgagctgag ctggactggg ctgagctgag ctggtctggg ctggctgagc   1021800
     tgagctgtgt tgagccaagc taggctgggc tgggctgagc tgggctgaac tgggctgagc   1021860
     ggagctgagc tgagctgggc tgggttgagc agagctgggg gctgagctgg gctgggctgg   1021920
     gctgagttaa gctgggctga cctgggctga gttaagctgg gctgacctgg gctgagctaa   1021980
     gctgggttga gccgatccgg gctgggctgg gctgggctga gccaggctag gttgagctgg   1022040
     gctgggctgg gctctgctgt gctgtgctga acagggctga gctgaactga gctgagctgg   1022100
     gctgagctgg gctctgctgt gctgtgctga gcagggctga gctgaactgt gctgagctgg   1022160
     gttgagctaa gctaggctgg gctgggctga gctgggatga gctgggctgg gctgggctgg   1022220
     gctctgctgt gctgtgctga acagggctga gctgaactga gctgagctgg gctgagctga   1022280
     gctctgctgt gctgtgctga gcagggctga gctgaactgg gctgagctgg gctgagccgg   1022340
     gctgagttga gcagagctgg gttgagcaga gctgggctgg gctgggctga gttgagccag   1022400
     gctgacctgg gctgagccaa gctgggttga gccaaactgg gctgggctgg gctgagccag   1022460
     gctggattga gctgggctgg gctgggctaa gctgggctgt gctgcactga gctgggctga   1022520
     gctgggttga actgtgctga gctgagctgg gctgtgctga actatgctta gctgggctgg   1022580
     gctatactgg gcttagctgg gctgggctaa actgggctta gctgggctgg gctatactgg   1022640
     gcttagctgg gctgggctat actgggctta gctgggctgg gctatactgg gcttagctgg   1022700
     gctgggctga gctgagatgg tcttaggtgg tctgagctca gctaggctgg gctgagctgg   1022760
     tctgagctca tctgagttgg gctgagctga gcttggcttt gctgagctgg ggtggggtgg   1022820
     gctgggctgg attgagctgg cctgggctgg gatgaactgg attgagctgg cctgggctgg   1022880
     gatgaactgg aggacatggc actgggccaa tcttcatgat cttgttggac atagatggat   1022940
     agcctcagct gagtctacac tgcgttcccc atcacactca ccctccctat actcactccc   1023000
     aggcctgggt tgtctgcctg gggagacttc agggtagctg gagtgtgact gagctggggg   1023060
     cagcagaagc tgggctggag ggactctatt ggctgcctgc ggggtgtgtg gctccaggct   1023120
     tcacattcag gtatgcaacc tgggccctcc agctgcatgt gctgggagct gagtgtgtgc   1023180
     agcacctacg tgctgatgcc tcgggggaaa gcaggcctgg tccacccaaa cctgagccct   1023240
     cagccattct gagcagggag ccaggggcag tcaggcctca gagtgcagca gggcagccag   1023300
     ctgaatggtg gcagggatgg ctcagcctgc tccaggagac cccaggtctg tccaggtgtt   1023360
     cagtgctggg ccctgcagca ggatgggctg aggcctgcag ccccagcagc cttggacaaa   1023420
     gacctgaggc ctcaccacgg ccccgccacc cctgatagcc atgacagtct gggctttgga   1023480
     ggcctgcagg tgggctcggc cttggtgggg cagccacagc gggacgcaag tagtgagggc   1023540
     actcagaacg ccactcagcc ccgacaggca gggcacgagg aggcagctcc tcaccctccc   1023600
     tttctctttt gtcctgcggg tcctcaggga gtgcatccgc cccaaccctt ttccccctcg   1023660
     tctcctgtga gaattccccg tcggatacga gcagcgtggc cgttggctgc ctcgcacagg   1023720
     acttccttcc cgactccatc actttctcct ggaaatacaa gaacaactct gacatcagca   1023780
     gcacccgggg cttcccatca gtcctgagag ggggcaagta cgcagccacc tcacaggtgc   1023840
     tgctgccttc caaggacgtc atgcagggca cagacgaaca cgtggtgtgc aaagtccagc   1023900
     accccaacgg caacaaagaa aagaacgtgc ctcttccagg tgagggccgg gcccagccac   1023960
     cgggacagag agggagccga agggggcggg agtggcgggc accgggctga cacgtgtccc   1024020
     tcactgcagt gattgctgag ctgcctccca aagtgagcgt cttcgtccca ccccgcgacg   1024080
     gcttcttcgg caacccccgc aagtccaagc tcatctgcca ggccacgggt ttcagtcccc   1024140
     ggcagattca ggtgtcctgg ctgcgcgagg ggaagcaggt ggggtctggc gtcaccacgg   1024200
     accaggtgca ggctgaggcc aaagagtctg ggcccacgac ctacaaggtg accagcacac   1024260
     tgaccatcaa agagagcgac tggctcggcc agagcatgtt cacctgccgc gtggatcaca   1024320
     ggggcctgac cttccagcag aatgcgtcct ccatgtgtgt ccccggtgag tgacctgtcc   1024380
     ccaggggcag cacccaccga cacacagggg tccactcggg tctggcattc gccaccccgg   1024440
     atgcagccat ctactccctg agccttggct tcccagagcg gccaagggca ggggctcggg   1024500
     cggcaggacc cctgggctcg gcagaggcag ttgctactct ttgggtggga accatgcctc   1024560
     cgcccacatc cacacctgcc ccacctctga ctcccttctc ttgactccag atcaagacac   1024620
     agccatccgg gtcttcgcca tccccccatc ctttgccagc atcttcctca ccaagtccac   1024680
     caagttgacc tgcctggtca cagacctgac cacctatgac agcgtgacca tctcctggac   1024740
     ccgccagaat ggcgaagctg tgaaaaccca caccaacatc tccgagagcc accccaatgc   1024800
     cactttcagc gccgtgggtg aggccagcat ctgcgaggat gactggaatt ccggggagag   1024860
     gttcacgtgc accgtgaccc acacagacct gccctcgcca ctgaagcaga ccatctcccg   1024920
     gcccaagggt aggccccact cttgcccctc ttcctgcact ccctgggacc tcccttggcc   1024980
     tctggggcat ggtggaaagc acccctcact cccccgttgt ctgggcaact ggggaaaagg   1025040
     ggactcaacc ccagcccaca ggctggtccc cccactgccc cgccctcacc accatctctg   1025100
     ttcacagggg tggccctgca caggcccgat gtctacttgc tgccaccagc ccgggagcag   1025160
     ctgaacctgc gggagtcggc caccatcacg tgcctggtga cgggcttctc tcccgcggac   1025220
     gtcttcgtgc agtggatgca gagggggcag cccttgtccc cggagaagta tgtgaccagc   1025280
     gccccaatgc ctgagcccca ggccccaggc cggtacttcg cccacagcat cctgaccgtg   1025340
     tccgaagagg aatggaacac gggggagacc tacacctgcg tggtggccca tgaggccctg   1025400
     cccaacaggg tcaccgagag gaccgtggac aagtccaccg gtaaacccac cctgtacaac   1025460
     gtgtccctgg tcatgtccga cacagctggc acctgctact gaccctgctg gcctgcccac   1025520
     aggctcgggg cggctggccg ctctgtgtgt gcatgcaaac taaccgtgtc aacggggtga   1025580
     gatgttgcat cttataaaat tagaaataaa aagatccatt caaaagatac tggtcctgag   1025640
     tgcacgatgc tctggcctac tggggcggcg gctgtgctgc acccaccctg cgcctcccct   1025700
     gcagaacacc ttcctccaca gcccccaccc ctgcctcacc cacctgcgtg cctcagtggc   1025760
     ttctagaaac ccctgaattc cctgcagctg ctcacagcag gctgacctca gacttgccat   1025820
     tcctcctact gcttccagaa agaaagctga aagcaaggcc acacgtatac aggcagcaca   1025880
     caggcatgtg tggatacaca tggacagaca cggacacaca caaacacatg gacacacaga   1025940
     gacgtgctaa cccatgggca cacacataca cagacatgga cccacacaca aacatatgtg   1026000
     gacacacatg tacaaacatg cacaggcaca caaagagaac actgactaca ggcacacaca   1026060
     cacacgggca cacacatgga tatgtgcaca catggacaca tacatgtgca ggacatgcac   1026120
     acacacagac acactagcac agaggcatac acacacagac acacacattc acaaacacac   1026180
     atgtgcatgc aaacacacac acatgtacag acacaagtac atggacacat gcacacccag   1026240
     agacacactg acacagacac acaggagcat gtgatacact aacacgtgga cacacacgtc   1026300
     tacccacagg cacacaacag atggacacgc gtacacagac atgcacacac ccacaggcac   1026360
     aacacgtgcg catgccggcc ggcccccgcc aacattctcc cagggccctg ccggatactc   1026420
     tgtccctgca gcagtttgct ccctgcgctg tgctggcacc ggggctttgg gcccaggctc   1026480
     tgcttgtcct tctgtctctg cttggaggtg ctgccatggc acccagcttg tgctctgcct   1026540
     ggggagcgga ggccccaggg atagcatgtg acccctgctg aggccaggct cctgatgaag   1026600
     gcagcagata gcccccacac ccaccggtga gcagaaccag agcctgtgcc atgtgctgag   1026660
     agcaggcagt gactaagcat atgggcccag agggcagagt ggctgccctg ggcagctgct   1026720
     cctcttagca ggaggcctca ggagatgagc tagagcaagt ctgcccctgc aaataccacc   1026780
     tgctccccaa cccacagcag ggagcaggcg aggtcagaca gcagcagccc gggaaggacc   1026840
     gagccccagc agggaaggca gggcccgagt gaggtctcca cacccaacgc acagtgctgt   1026900
     ctctaactgg ggccacctcc gagtccccgc cacactcttg gccctttgga gtcctgggct   1026960
     ccaggtgtct cccaagggcc catctgtgca ggggatgcaa ccccccgaat gtcctcatcc   1027020
     cactgtggag ctcaggtctc tgtctgctcc ctgggtcctg gcagggtagg acaagtccgc   1027080
     caggatgtcc ccatgcagac tctgctccaa gagggagctg gagagtcagg gccttggtga   1027140
     gggagtcagg atcgggttcc ccccagctca gtcctcccac ctgccagccc ccacagcaca   1027200
     gggcagggcc acaccccctg cttccccctc caggagagtc aggacatgct ggccgctgct   1027260
     ccgctggggc cccgccctcc agcccccacc ttggtctgtg tgctgcatcc cccacgctct   1027320
     ctctgccacc ccaggactct gaggaaaaga cctcagagtc ccagccctgc ccagtctcgg   1027380
     cctgtgcccc cgctgcatca ggctttcagg ggcccagccc atgccctggg cagtgcccga   1027440
     gcccccctgc acttgctctc cccacccctg ggtgcagcac agcctagggg ccaagggtgg   1027500
     gcctagagga tgggccccgg gggggctttg ctgggtgcca ccccagcctg accctattcc   1027560
     cccgtgctgt gtctcctgca gagggggagg tgagcgccga cgaggagggc tttgagaacc   1027620
     tgtgggccac cgcctccacc ttcatcgtcc tcttcctcct gagcctcttc tacagtacca   1027680
     ccgtcacctt gttcaaggta gcacggctgt ggcacaggga ggagggtgca gggcgagtgt   1027740
     ggggcccagg gagcagcctg ggctggacgt ctagcccgga ggcccccaca ccaccccact   1027800
     gggtcatctc tgccccggct cccttcccga ccacggggaa agcatttcac actgtctctg   1027860
     ttgcctgtag gtgaaatgat cccaacagaa gaacatcgga gaccagagag aggaactcaa   1027920
     aggggcgctg cctccgggtc tggggtcctg gcctgcgtgg cctgttggca cgtgtttctc   1027980
     ttccccgccc ggcctccagt tgtgtgctct cacacaggct tccttctcga ccggcagggg   1028040
     ctggctggct tgcaggccac gaggtgggct ctaccccaca ctgctttgct gtgtatacgc   1028100
     ttgttgccct gaaataaata tgcacatttt atccatgaaa ctgctttctg gtgagggttt   1028160
     ttgtttcttt ttcaaaactt tcctgctaca tgggcatctc aagggggacc ccagttccaa   1028220
     agggagctgt ggaaaagagc gctgggtcag ccagcgcagg gggtttcgag gaaagccacg   1028280
     tgcccaggaa aggggccgca gaagcaggtg ggccagactc agactcgggg catgcccagc   1028340
     ctgatggaag gaaggggact gagcaggaga gggttccagg cctggtcctc caagcacagc   1028400
     ctgaattgag agactggggc tcaggcctcg ggggcctctg tgtgtgctcc acatgcctac   1028460
     aactgcccgg gtcaccttgc cacccttccc agcaagccca gacagttctt ggccttgccc   1028520
     caaaccttca tgatgtgtgg tgcacgccac cgggatccag gaggtgcagg ctgagccctc   1028580
     gagagcatgt gggcctcacc gggatccagg aggtgcaggc tgagccctcg agagcatgtg   1028640
     ggcctgtctg cacagtgtgg gggccttgca ctccacagga gcacaggggt ggggtagcag   1028700
     tcgcgcccgt ggcaggggag tggaagttgg agcaaaagtt tcaggtgaac gagtgtctta   1028760
     gtctaattgg gcagctatca caaaacacta tcggctgcaa agcttgagca acagacattt   1028820
     gcctcccaca gcgctggagg ctgaaagtct aagatcaagg cgccggcaac ttcaacgtct   1028880
     ggcgagggcc aattcccagc tcagagacgc gcctcctcac tgcatcctcg cgcagccgaa   1028940
     gatggtgaag gagctctcag ggtctctgtc atacaggcac taagcccgtc acgagggtct   1029000
     ctctcataca ggcactaagc ccgtcacgag ggtctctctc atacaggcac taagcccgtc   1029060
     acgagggtct ctctcataca ggcactaagc ccgtcacgag ggtctctctc atacaggcac   1029120
     taagcccgtc acgagggtct ctctcataca ggcactaagc ccgtcacgag ggtctctctc   1029180
     atacaggcac taagcccgtc acgagggtct ctctcataca ggcactaagc ccgtcacgag   1029240
     ggtctctctc atacaggcac taagcccgtc acgagggtct ctctcataca ggcactaagc   1029300
     ccgtcacgag gtcctcatca ttgcccagat gctagggctc ccagtgccat ccccttgtgg   1029360
     atgaggatgg ggggcgtgac catgtcgtcc ttagaagccg ttagggaatg catagtgggg   1029420
     aggacaagtc tctgccatca ccctgaagat gggcaggtgg tggccaggct tggggcagtg   1029480
     ccgaaaaatg actccccagg taggtgggag caggatcaaa gagcagggga ttttttcaga   1029540
     gatggcagcc aagccaaagt tgggggctca tgccaggagg ctcacttggc catggtgcag   1029600
     tcacccaggg gtgacccaca gtccatggca aagtcaagga cagcagtggc tgtgagacca   1029660
     gggtggaggc aggtggctgg ggggagagga ccaggctgga aggactgggc agaccccaag   1029720
     gccatagaag tgtcctagag gaagcatcca ggatggcgtc ttgggagggc tcataagctg   1029780
     ggggcccaga aggaggacat ttcaggttct gaggagcagc ctggcacttg ctctgagcca   1029840
     gctgtcatgg gtatctgggg gctgctgggt acaagttgtg tccctcccac taaatgtgct   1029900
     ctccacatgg acccggcccg cccttctgtc cctgctggat ccctgagctg gcaccagccc   1029960
     tgccctcaga gacaatgtcc aggagacagg tggaggtgca cgtgtgggtc cctggggaaa   1030020
     tccatcctcc aaccgtgggc tccgagtcgc ctcccagcct ctcgctccag cttcacccca   1030080
     tgtagctcat cacaggctca acctcccagc ctgggggagg atggagtgag gagccccaca   1030140
     ctgcccaggg cacacccagg gggctgggga gtctgcactg ggctggggca gggaggcctc   1030200
     gtgcagcctg tggggctggc agctcaggac aacactcgta tccgttaact gtggccctgg   1030260
     caacactgca ccccagactg cgtggcttaa acaacagacg tttattccgt cctggttctg   1030320
     gaggccgggc atctgggatg gaggccttgg cggggctggc tcctctgtgt cacgggagac   1030380
     tctgttccag gctctctccc tgctgctggg ctttgccggc cgtctctggt gctcttggct   1030440
     tatggaagca gcaccatctt cacagggcgt tctccacacg tgctgtctgt gcccagattc   1030500
     ccccttttca tgaggacagc agtcatattg gatcagaggc ttgccctact ccagggtgac   1030560
     ctcatctgaa cttgattgca gctgcaaaga ctgtttccag acaaggtcac attctgtggt   1030620
     cctgggggtt aggacttcga cacattaatt tacaggggac acattttaac ccatgacagt   1030680
     ttgccctccg ttccccccat aatcatgtcc ttctcacagg caaaatccct gcatcccata   1030740
     gcaacatctc cagaacggct gaccccttcc agcaccaact cttagtccac aatctcagcg   1030800
     aaatatcacc tgcatcaagt gtgggagaaa cccagggtga gattcattct gggtgaaatt   1030860
     cctctccatc tgtggacctt tgtaacttga caagaagtta tctgcttcca aagtacaatg   1030920
     atggggcagg cataggatgg acattcctat tctaaaaggg atacatagaa agggagagag   1030980
     gagccatggg tcccaagcaa gcccaaaccc agcagggaaa gctgcattag atttaaggct   1031040
     caaggctcat cctcctgggt ccgtgctcca tcttccaggc ccactggggt gaccccatgc   1031100
     tcttcgccca tggtggccag agcccccaca gcctcctcat ctttggctct gatctcaaag   1031160
     tcacccttcc ttcatttttt ctggtcttct ttcccttcca tccaattggc attgtttctt   1031220
     ctgttttaca attcacaaaa tccttgtcag cttcccatga aattcctggg ggtttgagtc   1031280
     atcagacaag agagccctgc acagttatat cctcaataac cttatctcta ctcctggttt   1031340
     ctgctgagat ggttgattgg atccataagt catgattcca atctctttaa caaatggttg   1031400
     cccagacaca tccttggcct tgtttccaga gcatgctatt ggacaggctg agaattttcc   1031460
     agctgttcaa gttgtgggtc ctgtttactg aacactttct ctcacttttt actataagca   1031520
     gtcaggagaa atcaggttgt gccttcaaca cttttcttag aaatatcctc agctaaaatc   1031580
     caggttcatc acttgtaagt cctacttacc ataaatagaa tgcaattcag ccaagtgctt   1031640
     tgccactttc taacaaaggt cacctttcct ccaattttca ataacatctt cctcatttct   1031700
     gtctggggcc tcaccagagg catctttaac attcacattt ctagcaacat tctgtttgtg   1031760
     acaatttatg tattctctaa gatgacagga gttttctcta tagctctcct ctttctttct   1031820
     gagcccccac caggatcacc ttcaatgtcc aaatttctac catagtcttc aaggaaatct   1031880
     aggctttttc taacatgcac ctgaaaactc ttctcacctc tacacattac cctatcccaa   1031940
     attcatttcc acagttttag gtatcaatta aatcagataa ataaatcatt accctgaatt   1032000
     atttcagtta agcatgttag ttggtggcat aagagaaaac tcagtcagat agtgctgaag   1032060
     acaggactgt ggagacacct tagaaggaca gattctgttc agaatcactg atgcggcgtc   1032120
     agcaggactg gcctagtgga ggctctggga gggtggctgc caggcccggc ctgggctttg   1032180
     ggtctccccg gactacccgg agctgggatg cgtggcttct gccgccgggc cgactggctg   1032240
     ctcaggcccc agcccttgtt aatggacttg gaggaatgat tccatgccaa agcttttgca   1032300
     aggttcgcag tgaccaggca cccgacatgg taagagacag gcagccgccg ctgctgcatt   1032360
     cacttctctt aaaactttgt atttgatgtc ttatttccac tagaaggcga attggtctta   1032420
     attgcttctc ttaagccacc acttctaaag ccaaaatctg ttttagtttc ttatgagcta   1032480
     ttgtaacaaa gtaccacaac tgcatggctg aaacaacaga agtgtattct gtctcatttc   1032540
     tggaggccag gtgtctgaga tcaagctgtg ggcagggttg gctcccctgg gttggcaggg   1032600
     agaatctgtt ccaggtgtct gtcctggctt ttgttggttt gctggaaagc tttggtgttc   1032660
     cttggctttt ggaaacagct cctccgcctc catctttacc ttcacatggc attctgcctg   1032720
     cgtgcatgtc tgtgtctaaa tttccacttt gcataaggac accagccata ttgggtcaga   1032780
     ggctcacctg cctccagcgt gacctcatct taacttgatt gcatctgcaa aactctgctt   1032840
     ccaaagaagg gcacattctg aggccctacg ggtcagagct tcaacacgtc catttatttg   1032900
     gagacacaac tcaacccata acaagaccca tccctagtgt ctcaccagtg gtgctgtaga   1032960
     gggaggcctg gcagggctga catcccagaa cctcagggaa aaatgaaaac agcccagagg   1033020
     ggtccaagag gtggctcgag gaagggaatt aggaacacac tgttcaggtg gaagattctg   1033080
     agatgggccg tgaatactgc ctaggatgtg gagggttgac aatgggttga caatcctgct   1033140
     cagaaaatcc caggataaag gatgtaaatg gggagaagag gagggtcatc cagaatttgg   1033200
     gaaagcaggg cgacagtttc tgccccaagg gagaagggaa ggaggatggg gccaccgcca   1033260
     caccagatga ccttgcgtac caggccaaag aacgggaaca cctggcccca cctgagcagc   1033320
     aatagtcagt gtggtggtgg gcagacatgg gtggaggcag ggggtgagta gaaggttaga   1033380
     ctaagacgga gcacctgggg cctccaggga cccaggcaag aaccctgcac ttgctcagct   1033440
     gccctgggta cccaggtctc caggaaagtg aggctgagag ccaagcccag caggcagcca   1033500
     cacattctgg gacctgccac cctacagcct gctgtccatg agtaacaccc cttacaaggg   1033560
     gccaggtcag ctcatgggtt tatcccaggc agcagagccc ctggggccag gaatcaggga   1033620
     gaggagcatc caatcccacc agctcctccg gagccactca gagggccaga cgcatggccc   1033680
     tccacaggga ccccatggcc cctgcagggc agctgaggac ccgtggctgg gagcctgggc   1033740
     aggagggtca tacagcccta ggcccgttgc tcccaggctt gagtgcccct cctcccctca   1033800
     ggggcccaag ggaagtgggt tccagagagg ttgggggcag cagggaaggt ggaggtccca   1033860
     ggaatgccca gaggggcacc aaagcctctg gagggaagac ccctcccttc caggagctct   1033920
     cggcaacaag agcccagggt ccacaaagcc acaggtccca ctcggttatt ctgactcaca   1033980
     acacaggagc ggcagcaggg gcattcgtgt tcacgggcca cttggtcagc cccgctcacc   1034040
     ctgggcactc ctcctgggcc ccttttccct gccttccctg tcaccctgct gccagggtcc   1034100
     tctgccctgc cctgcccctt gtcctcagag cctccagcct cagactccca ctgtgtctgt   1034160
     cttccagcac ccaccaaggc tccggatgtg ttccccatca tatcagggtg cagacaccca   1034220
     aaggataaca gccctgtggt cctggcatgc ttgataactg ggtaccaccc aacgtccgtg   1034280
     actgtcacct ggtacatggg gacacagagc cagccccaga gaaccttccc tgagatacaa   1034340
     agacgggaca gctactacat gacaagcagc cagctctcca cccccctcca gcagtggcgc   1034400
     caaggcgagt acaaatgcgt ggtccagcac accgccagca agagtaagaa ggagatcttc   1034460
     cgctggccag gtaggtcgca ccggagatca cccagaaggg ccccccagga cccccagcac   1034520
     cttccactca gggcctgacc acaaagacag aagcaagggc tgggctgtga ggcaaccccc   1034580
     acctccccct cagagcacgt tcctccccct tcaccctgta tccacccctc cggaccctcc   1034640
     ccatctcagt ccctccgctc cctctctctg aggcccatct cccaataccc agatcacttt   1034700
     ccttccagac ccttccctca gtgtgcacgg aggcagcttg cccagcaaag gtgactgtct   1034760
     agtgggcttc ccacagccaa gctcccaccc catgctgcgg cccctccctt cttcctgctt   1034820
     ggctgcctgt gccccccacc tgcctgtcca caacccagcc tctggtacat ccatgccctc   1034880
     tgccctcagc ctcacctgca cttttccttg gatttcagag tctccaaagg cacaggcctc   1034940
     ctcagtgccc actgcacaac cccaagcaga gggcagcctc gccaaggcaa ccacagcccc   1035000
     agccaccacc cgtaacacag gtgagaagcc ccttccctgc acactccacc cccacccacc   1035060
     tgctcattcc tcagccgcct cctccaggca gcccttcata actccttgtc tgagtctcca   1035120
     agtcacactt tggtaaggag agggacactg aacggacctc taacaaacac ctactgccag   1035180
     ccagccccag tctgggggcc agcagatgcc aaacaaccag cagactccca gagcagacct   1035240
     gggccggctc cctggcccat ggacccagct ctgcctcgct gagctgaggc atgggctctc   1035300
     agcgcagcct cacatagagc caccctgccg aggcagtccg gcttgcagac tcacaggtca   1035360
     cttgggccgc agcagcccct ccccgtgacc ctcgcctccc gcccgcccca gcctggctct   1035420
     ctccaagtgt tggatcttgg tggccagcct gcttctcacc ctcaccctgc ctgccacctc   1035480
     agaatggcag gggaaagagg gccctcacca agaactttat ctgagaagtc tgaggcttgt   1035540
     gactctgacc tgcctgagat gtccatgtgg ccggggggac gggttcagtg ttcgggagaa   1035600
     ctcgggtacg tgcctgactt tctctgagta gggcaggaag ctgttaggag aagcagcagt   1035660
     gaggtgggct ggaccaacag gcagaatgac tgtccctcag ccaccctctg ggatgtgggt   1035720
     caagctctga caaaggcatg gcacagccat ggtggcccct gcttggatga gtggccacgg   1035780
     tgccctcacc ctgggccaga atctgcctcc actctgcagg tgcagaaaca cgacattccc   1035840
     gtctctaaac acacctagct cctaggcttg gggtgggcct atcaaatgca gggagatgga   1035900
     cacagcacaa gggccagagc ttcccatgag aaaggtgagg gcagctgctc cctgacccgg   1035960
     gcatctgcac ttgtccctct ccaccctcct catgggcagt ggagactcag caacaaaaca   1036020
     agttgagtgc attagcagcc agctctggag ccaagtcact caccccacgg ccttggctgc   1036080
     tggtggaggg gccttcccct gggcagcctc caagaagaca gccaagtgct cttactcaga   1036140
     ccacggcgct gcttcctggc acctcgattt cccacaacaa catggggtgc agacaggcta   1036200
     gggccccctg ccctggggcc tggacggcat ccagttaaag atgacccttc acgggcggtg   1036260
     cctgaggtgt gctgacctca gcagctaagc cctcaggtct ggtctgcact gccccacctg   1036320
     gaggacccaa ctgacccaga cacagccagg gttatggcat gaccccgtgg acggtgaccc   1036380
     acaggccaga tgcagccggg ggctgttttg tgtggcctag aaatgtcttt acagttgtag   1036440
     tgggatggag gaggaagagg aagagaggag gggagaggaa agcagggaag gggaaaaaga   1036500
     ggagttcaat gcaaccccaa aagccagaac agttttgagc tgaaagaaca aggcaggaaa   1036560
     catcccagta cctgacttca aaacatacta taaagcagtt gtaatcaaaa caggatcata   1036620
     aaaacagaca cacagaccca tggaacagaa aagcgagccc agaaataaat ctacatgctt   1036680
     gcagtccatt gattttcaac aaaggcacca ggaaaacaca atggggagag gacagtttcc   1036740
     tcaataaata gtgctgggga aactggatat ccatgtgcag actaatgaaa ctacacaaaa   1036800
     atcaattgaa aacagtctag gccaggcgcg gtggctcatg ccggtaatcc cagcactttg   1036860
     ggaggccgag acaggcggat cacctgaggt caggagttcg agaccagctt ggccaacatg   1036920
     gcgaaacccg gtctccacta aaaatacaaa aattagcaca tggtggccta cgtctgttat   1036980
     cccagctttt caggaggctg aggcaggaga atcgcttgaa tccgggaggt gaaggttgca   1037040
     gggagccaag attgcgccac tgcattccag cctgggcaat ggagcgagac tgtctcaaaa   1037100
     aaaaaaaaaa aaaaaagaaa agaaaacagt ctaaaggttt aactgaacag ataaagctac   1037160
     tagaagaaaa cataggggga aaactccatg acattagtct gagcaacgat ttttggatat   1037220
     gatcccaaaa gctcaggcag cactagtcac aaaagccaag atacagaacc aacctaagca   1037280
     cccctcagca gatgcacagg taaagaaaat gtggtacgta tggggcacaa tggaatacga   1037340
     ttcagccttt aaaaacagtg aaattctgtc attggcaaca atgtagatga acctgaagga   1037400
     cacttatgct aagtgaaata agccaggcac agaaggagca atactgcatg attgcactta   1037460
     catctggcag gttaaaaagg caaactctta gaggcagaca gtagagaggt ggtgccaggg   1037520
     agcgggcact ggtggctggg gagatgttgg tcaaagggca caaaactgca gttgggagga   1037580
     attagttcag gacatccctt gtacatgggg acagtggtta gtaacaacgg attgtatcct   1037640
     tgaaaaccgc taagaaaata gtttttaagt gttcttgaca caaaaagtga cacgtatgtg   1037700
     agatactgca tggtcattag ctggatttag ccattccaca atgtacacat atttcaaaca   1037760
     ttgtgttgta tatgataaac atgtataatt tttgtcaatt aaaaattttt aggaagagga   1037820
     ggagaagaga agaagaagga gaaggagaaa gaggaacaag aagagagaga gacaaagaca   1037880
     ccaggttttt tctgacccct gggctatcaa aacacctatt gcccaataac tagttggccg   1037940
     ttggtgccct aaactattga agcgattgct gttatgtgga tgggccccgg acacttagaa   1038000
     actcgtgacc cctgaggacc cccacgagga cagtcagggt ccccccgaac tcagggagca   1038060
     ctgaggaagg agctcttaga ggcgtggggc ccctcaggcc cctcagaggg ctctgccaca   1038120
     tgggtcaggg gcaggctgag ggggagtccc aggctccatg cccagcctct gtgcctctga   1038180
     ccagggtgtc ccccacaccg cctcctcccc agtgccctcc actggccaca cctggccaga   1038240
     agctggggag aggagagcac agtggttaag tcagtccctg cagggagacg gcaccagaaa   1038300
     aacctggcct gtggatgagt cccggcctgg cagccacaga gcagagagct ctggaagcaa   1038360
     cgaaggcccg agtctgctca gggaagagcg ggcagcagcc ccagggccgg acagtgacca   1038420
     agagtggcac cgcccatggc tcaacgggtc tttgcccaca gatcccccag cccctggaga   1038480
     cagggtctgt gtgcctggcc gtgcaggcag gcaccacact cagggggagg ccactgtgga   1038540
     gctctgtgca gagccccggg cgggagccta ctgctcccga aggtccggcc acagctgctc   1038600
     tcgtttgctc tcccctgcag agtgtccgag ccacacccag cctcttggcg tctacctgct   1038660
     aacccctgca gtgcaggacc tgtggctccg ggacaaagcc accttcacct gcttcgtggt   1038720
     gggcagtgac ctgaaggatg ctcacctgac ctgggaggtg gctgggaagg tccccacagg   1038780
     gggcgtggag gaagggctgc tggagcggca cagcaacggc tcccagagcc agcacagccg   1038840
     tctgaccctg cccaggtcct tgtggaacgc ggggacctcc gtcacctgca cactgaacca   1038900
     tcccagcctc ccaccccaga ggttgatggc gctgagagaa cccggtgagc ctggctccca   1038960
     ggtggggaga cgagggtgcc cacagcctgc tgacccctac gcctgcccca gggccatgac   1039020
     cccagctggg ccccagcagc accggtcatc ctccacagga aaggagaagg gaggcaccag   1039080
     caccctggcc ggccccactt ctctcccagt gcccccgtgg ccagagcctg acagcctccc   1039140
     ccacctcccc gcagctgcgc aggcacccgt caagctttcc ctgaacctgc tggcctcgtc   1039200
     tgaccctccc gaggcggcct cgtggctcct gtgtgaggtg tctggcttct cgccccccaa   1039260
     catcctcctg atgtggctgg aggaccagcg tgaggtgaac acttctgggt ttgcccccgc   1039320
     acgcccccct ccacagccca ggagcaccac gttctgggcc tggagtgtgc tgcgtgtccc   1039380
     agccccgccc agccctcagc cagccaccta cacgtgtgtg gtcagccacg aggactcccg   1039440
     gactctgctc aacgccagcc ggagcctaga agtcagctgt gagtcacccc caggcccagg   1039500
     gttgggacgg ggactctgag gggggccata aggagctgga atccatacta ggcaggggtg   1039560
     ggcactgggc aggggcgggg ctaggctgtc ctgggcacac aggccccttc tcggtgtccg   1039620
     gcaggagcac agacttccca gtactcctgg gccatggatg tcccagcgtc catccttgct   1039680
     gtccacacca cgtgctggcc caggctggct ggcacagtgt aagaggtgga tacaacccct   1039740
     cgccgtgccc tgaggagtgg cggtttcctc ccaagacatt ccccacggct gggtgctggg   1039800
     cacaggcctt ccctggtgtg accgtgaatg tggtcaccct gaacagctgc cctctctggg   1039860
     gacatctgac tgtccaagac cacagtcagc acctctggga gccagagggg tctccagaga   1039920
     cccccagatg tcaggcttgg gctcagtgcc cagcgaaagg tcagccccac acatgcccat   1039980
     aatgggcgcc cacccagagt gacagccccc agcctcctgc caggcccacc cttttccgcc   1040040
     cccttgaggc atggcacaca gaccagtgcg cccactgccc gagcatggcc ccagtgggat   1040100
     gtggtggcca cgaggggctg tacacacagc aggaggctgt ccgccctgct cagggcctgc   1040160
     tgcctatgcc ccagctgtcc agccaaggga ggcatggaag ggcccctggt gtaagctgga   1040220
     gccaggcacc caggcccccg gccaccctgc agagccaagg aaaggaagac acccaagtca   1040280
     acaaggggca gggctgaggg ctgtcccagg ctcttttggc ccgaggggct gccagcagcc   1040340
     ctgacccggc atgggccttc cccagaagcg accctgtgag gtggcctcac agagaacccc   1040400
     ctctgaggac agtgtctgac cctgcctgcc tcacacagat gggccccaca gcagtgggca   1040460
     acctgggggg cagcagccca acctgaccct gcagggactg ccccctgcag cagcagctgc   1040520
     ttctcagtcc cccaacctcc ctgtccccgc cagagggtct tccccgaagc tgcagcccca   1040580
     acccatggct gcccacctgg aaccgggact ccctgtccac tgccccctcc ccttcggggc   1040640
     cccatctgtg ctggggccca ggttcggcct acagattccc atcattgcca tggcctcctg   1040700
     accttgccta tccaccccca accaccggct ccatgctgac cctcccccag gctcccacgc   1040760
     ccagctggcc ggccatcccc aggcacagac agtctgggat ctcacaggtt agcctggacc   1040820
     atccacctgg ccagacctgg gagaggctgg aagctgccct gccaccatgc tccagggccc   1040880
     caggttgcag tactatgggg tgagggtgtg tgtgcacacc cgtgtgtacc taggatatcc   1040940
     gagtgtaccc ttgtgccccc aagcacaagt ctccctccca ggcagtgagg cccagatggt   1041000
     gcagtggtta gagctgaggc ttatcccaca gagaaccctg gcgccttggt caaggaagcc   1041060
     cctatgcctt tcttgcctcg atttcccctc ttgtctgctg agccagcagg ggccacgtcc   1041120
     tgggctgctg tgaggaggaa gtgagttggt gctaggaggg gctcctgtgt gtgcatgggc   1041180
     gggaggggtg caggtatctg agcaccccgg tctccacttg agagagcagg gcaggagctc   1041240
     cctgacccac ccagactaca cacgctgtgt ccacgtgtct cacattatct gtggcagagg   1041300
     atccggcttc tttctcaatt tccagttctt cacaaagcaa tgcctttgta aaatgcaata   1041360
     agaaatacta gaaaaatgat atgaacagaa agacacgccg attttttgtt attagatgta   1041420
     acagaccatg gccccatgaa atgatcccgg accagatccg tccacacccg ccactcagca   1041480
     gctctggccg agctcacagt acaaccacaa taaactcttg ttgaatgaac tctaggaagt   1041540
     ctgtgacgtg gctggttctt gtcaatgctt cctgcctgcc cacaggctct tcctcgtgga   1041600
     tggggctgtg cttgccatgg aagcgttttt cccggcctag gcttgccttg ggccccactg   1041660
     ccgtctccag ctggagatga ccttctatac acacatttgc tcatgacaga cccttgctta   1041720
     gcccccttcc atggctccct cctgctgctg ggataaaatc accttgcctg gatatcccct   1041780
     cctgggcccc tttccaccct ccttagtcag cacccccagt tcagggcacc tgctttcccc   1041840
     gctgcggaga agccactctc tccttgctgc ccggctgtgt cttgccttcc acaccttgtc   1041900
     acagtggcca cttcctaagg aaggcctccc tgtgtgcagg tgtgcagaag tgccccagcc   1041960
     tcccgtcacc tttgtcacgg gagcccaatc catgagagtc tatggttctg tctgtctgcc   1042020
     ccactcaggg cagcgacaag tccaggcggg gaggacacag taggcagaga tttgtcgagg   1042080
     ggacatatga gcaagagggt gaggctggga gctccctgga gataaccacg cctcctggga   1042140
     agactcgccg tcatttcagc tccacgctgt gcgggggtgg gtggaggggt agcctggccc   1042200
     tcatgaccag ggagcttctc actcagcccc cgttcctccc cagacctggc catgaccccc   1042260
     ctgatccctc agagcaagga tgagaacagc gatgactaca cgacctttga tgatgtgggc   1042320
     agcctgtgga ccaccctgtc cacgtttgtg gccctcttca tcctcaccct cctctacagc   1042380
     ggcattgtca ctttcatcaa ggtcagggga gcggccaggc tctcagtgac cctcggggtg   1042440
     ggtgtggggc aaggtgccct tccaggggac atgccagagt tggtccaggg atcctggacc   1042500
     aggcagaggc agggctgagg gagcctggag gacatgcagg ccctctgtgg cctgtggaca   1042560
     ctgtcgaagg ccctcttgac cctgtggata aaggacaaca ccccctcccc tgctcctctg   1042620
     tctcccctgc ccctccaccc ctcaggcttc tagccccctg tctgacccca ggggctgtct   1042680
     ttcaggtgaa gtagccccag aagagcagga cgccctgtac ctgcagagaa gggaagcagc   1042740
     ctctgtacct catctgtggc taccagagag cagaaaggac ccaccctgga ctcttctgtg   1042800
     tgcaggaaga tgcgccagcc cctgcccccg gctcccctct gtccgccaca gaacccagtc   1042860
     ttctagacca gggggacggg cacccatcac tccgcaggcg aatcagagcc cccctgcccc   1042920
     ggccctaacc cctgtgcctc cttcccatgc ttccccgaga gccagctaca cccctgcccc   1042980
     ggccctaacc cccatgcctc cttcctgtgc ttcccccaga gccagctagt cccacctgca   1043040
     gcccgctggc ctccccataa acacactttg gttcatttca cctgcctcct gttctttgtc   1043100
     ccagtggtct ggattcacct caagttaaag gacacaggga gctaacggca atgggaaggg   1043160
     agtttggggt gtcagcagca acagggaacg caatagccaa agctgtcaca gcaagaactg   1043220
     agcggagacc ttgctctgaa gttaatgggt taagaaacgc ccctccccac accaaacaca   1043280
     aacatttcac gctcactcgt acatgcacac acatgcacac aagcatctat gcacacatgc   1043340
     acatgtgcac agctcacgaa taaacatata tgcacacaca tgcagtacac tcacatgcaa   1043400
     acaggcagat gcacaggcat gggcacacat gcacaaggca tgcatgtgca ggtctgcaga   1043460
     cacacacatc tgcatgcaca tacacggacg tgcacctgca cacacacaag tgcacatagg   1043520
     catcaaagac acacatgctc gtgcacgcgc atacacacag acatgcacac ttatgcatgc   1043580
     acacgccatg acaggatggc acgggaacaa ccttgcacat ggagcctgtt ctgtgtgtat   1043640
     cgagagcctg cacctcttac tgtctgcatg actgtgggca agagcctgaa ctccgtacct   1043700
     ccatttcctc atcagaatgg ggtgaaaata cacctgtcaa gtagagtgag gtgagaagtg   1043760
     agtaaaatcc tatctgaaag gggctcagca aggacccagg tattggtcgg acctcctgtt   1043820
     atcatctgct gtgggctgaa catttgtgtc ctcctgaaat tcatacattc gtatattcat   1043880
     acctttcccc ctgaaatcct aacctccaat gtaatggtat tagaagatgg ggcttttggg   1043940
     ggtgctgagg tcataaaagg ggggccctca tgaagaggat tagcaccctt atgagagggg   1044000
     ccccagagag ctccctcacc ctttctcccc tgtgaggaga tgccgtctat gagctgggat   1044060
     gcaggcctca ccagacaccc atcggctgga gccttggtct gggacattca gcctccagaa   1044120
     tggtgggaaa taagtttctg ttgtttctca gccaccacgt ctgtagtatg tggaagtcat   1044180
     cagaatcaaa attgagtcac ctgtggtttt ttttttttct aaatccctga caaatagagc   1044240
     ctaggaaggc caagaagaga agagggttct catccataaa cacttgataa caaaaactat   1044300
     caccaaggac tctacaaaaa ctgcaactgg cacaaagacc atcacaacct tacacagaaa   1044360
     gtacttctgt gaggacatct tcccagcaac gggctgtcca acctcagact ggcattgcct   1044420
     ttgttattgg tccttgtaga gagggtaatt atctcaaagc aatcatgtaa tcctcctcat   1044480
     ttttcctttg aaagccttgg tctccctttg cctccctgaa tacgcacata gctgatcatg   1044540
     gcaggtgtat cccactgcag tgctctacct ccaaatagat atctttttct tttagacggc   1044600
     atttctcttg ttatttagat tgagatacat ggagtcagaa gtgggatgtg gagaaggatc   1044660
     actgctggaa ggagtcagta attcttgggc cggtgtgcag tattcacttg agccctttga   1044720
     gctctcagct ttttctgagc tactccgtct tctctcaggc caaccctccc tctttttgga   1044780
     agatgctttt ttaatattat gtggggtttg tttattagac tgccttaata aaggacctta   1044840
     tgtccctcct gggatgataa aaggcttttc gtgctttctg gcaagtcctg tttagcataa   1044900
     agacattcca gcctgagtac tctggtttcc acagagtttg cattctgtct ctgagggatg   1044960
     tcttctctgg tgaggtgcct cctttaatac tttgttggat atgcatgcct gggtgaacat   1045020
     atttgtgagc acccttatct tgccttcttt tgctgtggtt atgtgtatct gtaaatgacc   1045080
     tggctctttt cctctgcttg cttctaaaaa tctttggaga gcaaaataaa cattctaaat   1045140
     ggtgggcaca ggctgtctca ttagaagctg ctacagcagt caccaccgtc taaagcacca   1045200
     gtccaaactg ccggcgtggc ctcatagtat ctgtagaatg ttctttactg taaaaagatt   1045260
     aataagaagg ggaatggttt ttaaatatta aggcacagta agttttctgg aactcccgct   1045320
     ggctacgtct ttgtgcacat ttttaaacta atgggcaaat tacgtcaagg aaaatttaga   1045380
     gttccaatgg ttatttttcg aactctaaca aaaaccctgc aacagtagtc atcatgcagt   1045440
     gcctcctaag tcctctatct ctctattttg tttttctgcc tactttaaat ctgctgactt   1045500
     ttttactcat gttgcaataa aactcactgg ttacagcatt ccagccaaga tatttttggt   1045560
     tttttgtttg tttgtttttt gttttttgtt tttttagatg gagtctcact ctgtcaccca   1045620
     ggctggaatg cagtggcgct atcttgggtc gctgcaacct ctgcctctcg ggttcaagtg   1045680
     attctcctgc ctcagcctcc tgagtagctg attacaggtg cccgccacca tgcccagcta   1045740
     atttttgtat tttagtagag acaggctttc actgtgttgg ccaagctcgt cttgaactcc   1045800
     tgacctcaag tgatccacct gccttggcct cccaaagtgc taaaactata ggcgtgtgcc   1045860
     accgtgcctg gcccaagatt ttttaaagtt tcctaaaggt tttaaattag tggctttaca   1045920
     aattacaaca gctctatggt aaccaatgac ctagatgtaa gagtcatttc ttttgaaaat   1045980
     gtagattagc tttgcttggc taacaactgc ttaggctgat ggaatagcta actgaaggac   1046040
     tgatggtctc aaagaacaga actaggtaaa tatttacaga aattgggctt tcagatcgaa   1046100
     caggccaaaa tcttgagctt agagcagtaa tataaggtat ctctgtctgc cataaaaatt   1046160
     ttgctttgcc acaggtgcca gaaaaaggaa aaacactgct aaatgcttcc ctgcatgcat   1046220
     tgtctagtcc agaaaatcag accagcaacc aaaaaataga tttgttacta gtatagacga   1046280
     gttgaagatt ttgtttttca tatacaattc agccagtcct agctaaaacg caatcactgg   1046340
     aaatttaacc ttaaattcat ttaaaactga aaaaggtctt ttgaaatcaa actaatgcag   1046400
     aaactgcttt acccaaaatt ctgatccatg gccctcatta gacgacccat caggacaaat   1046460
     aaagtttagc ttgtgaacaa gtcccagttt tgtcaaaaat ataatttgga ttgaagtgtc   1046520
     ttttagaaac tgacacattt gtgttattat ctcatgacca aaattctaaa atgaaagcta   1046580
     cagtgtcttt atttgtgtgc gtatgtggtt aattgtgttt atgcatatgt atgtgtatta   1046640
     tgttgtatgt tgttatctac atggtaaaat ctggcatcat caacaaaaaa tctattaagg   1046700
     gattctattt agattggctt agataaataa gcattcataa aaaatatgta ctaattaacc   1046760
     caaatgcctt ttagttcatg tgacttaagt atatctttaa taaacaaatc agttttaaaa   1046820
     ttgttggtaa aataaaaata caaatgttct cagaattgtc agcatatatt tttccctgag   1046880
     tttactcgtc agacagcttt atatttgtct ctgatagatg ttttaagatg tcagggtttg   1046940
     acacaaagat gataaaacta taaacccatc ctaaaacaga ataatctttg tatgatcttt   1047000
     gataaataag actaatttgg ctgagtgcgg tggttcatgc ctgtaattcc agcactttgg   1047060
     gaggccaagg tgggcagatg actagaggtc aggactttga gaccagcctg gccaacacag   1047120
     caaaacccca tccctactaa aagtacaaaa ttagccaggt gtggtggtgg gcataatccc   1047180
     agctacttgg gagcctgagg caggaaagtc actggaaccc gggaggcaga ggttgcaatg   1047240
     agccaagaga gcgccactgc actccagcct aggcaacagc gagactccat ctcaaaaaaa   1047300
     aaaaaaaagg ctaatttaat attgcaggct taataaaaac agctgtatct tatgagctat   1047360
     cgacaaaata cccatacact tcactaagtt tcttactaag tgaacatcta ataatcacag   1047420
     gctataaaaa tggttaaaag ggaaataact ttaagtaatg gctagctttg tctaatatct   1047480
     caattttcat aagtaatcta ggtaaattat taaaaataaa ttaattaagc ctggtgtcat   1047540
     ggcacacgcc tatagtccta gctactcagg aggctgaggt gggaggattg cttgagcccg   1047600
     gaagttcaga ccaccctgag cgatgcaaca agacccccat cactaaaata aataaataaa   1047660
     taagataaat gtcaagaaaa taaatgttta taaataagct tcttatgtaa tttaagatct   1047720
     taaaattatg ttatcttcag ttaaataata gatactcatt aaatgtctgg gtcatttcca   1047780
     aataagattt ttaaaactaa cacaaattac tgaacataaa tgtttgttct tggcttctta   1047840
     aattttatag aaattttgta gaaagactaa atttatttgg gtctattaat atatgtaaaa   1047900
     attatgtcat agaaatatgt tttcaaaaat tataaaattt ttctcatcta taaaatagtg   1047960
     atatgtgaca aatggttaaa atttgcttgc tagaaaataa ggttactaag agttaaaatt   1048020
     ctaattaata tatataattc tgtctacaaa gtataccaca aaaaataaga tgtgtttttc   1048080
     atttttaaaa actataagac aggcatttat aatttatttt actgcaaaaa aaaatttatc   1048140
     taatttggag tttgtttaaa ggttgtttca aagcacagat ttaggaaaaa agtaaaaaca   1048200
     agatggaaaa gaaccactct catctgcccc cacgtaagac gtacctgctc cccttccacc   1048260
     acgactgtgt ttcccaaggc ctctccagcc atgtggaact gaatttaatc tacaaataga   1048320
     gttgatcaag taactttaag gtatatggac aataatatgc tttgcagcat aaagacctga   1048380
     aaacaaacta aatgcccatc agtgggaaat tgtttcaata aaccacagaa cattccgtgg   1048440
     aaaactccac agccattcaa aagaatgaag ggggagagat catcatggtg gacaggagtc   1048500
     aggactagat tgcagtccga ctgggatgga cagagcacat gtggaggctg gcaccgtgaa   1048560
     ttttagctcc agaacaactg caggaataaa tcaggaatcc caagaggatc cacagaccat   1048620
     gtgaaggaag cagcctgctc ctgcaggacc cagagacacc ccaaatactg tgagtgccca   1048680
     aactgtggaa gtgggaatgg gagatcgtcc atcctgcaca cacaccctca ctagagaaac   1048740
     caaaggtcta gttggtggga aaaggttcca accttacctg gagctgagtc aatttagaaa   1048800
     gctgagcgaa atacaggggt acagggagca gcgggaaagg ccctgggagc ttcctgggtc   1048860
     cccaggcagg ccattcctgc atggcaccat atgaatactt tgggagggtg gccagaggca   1048920
     cagggaaaat gtcacaggga gaaggaagtc tccagctgaa cgcaggggag ggcacgaatc   1048980
     ctgcagaccc cactggtaga ggaacaacca aagcccttct tcatagctgg gaagtaggta   1049040
     gcctggggca agttctctgc ttgcccaatg cctggaaaca gactcagtgc tgttggcgag   1049100
     gggcatggtg tgagtgagac ccaccttcag tttgcatggg agctgggtga cacctgtgac   1049160
     tgccggcttt ccccacttcc ctgacaacct gcatgactca gcagaggcag tcataatcct   1049220
     cctaggttca caactccatt gacctaggaa cctcacagcc atccccccca gcagctgcag   1049280
     caagaactgt ccaaggagac tctgtgagct cagacacacc tagccctgcc cgcatctgat   1049340
     ggtccttccc tacccaccct tgtagttgaa gacagagggc atatactctt gggagttcta   1049400
     gggccctacc cactgctggt tcctctccat actaccacca ctgatgctct ctggaaaacg   1049460
     ccatctcctg gcaggaggcc aaccagcaca aaaacagagc attaaaccac caaagctaag   1049520
     aaccttcatg gagtccattt caccgccccg ccacctccac tgggacagtt gctggtatgc   1049580
     acggcggaga aacccacaga cagttcacat cacaggactc tgtgcagaca acccccagta   1049640
     caagcctgga gcctggtaga cttgctgggt ggctagatcc agaagagaaa taacaatcac   1049700
     cacagctcgg ctctcaggaa gccacatcca caggaaaagg gggagagtgc tacatcaagg   1049760
     gaacaccccg tgggacaaag gaatctgagc aacagccttc agccccagac cttccctcag   1049820
     acagagccta ctcaaatgag aaggaaccag aaaaccagct ctggtaatat gacaaaacaa   1049880
     agctctttaa cacccccaaa aaatcgcact agctcaccag caatggatcc aaaccaagaa   1049940
     gaaatccctg atttacctga aaaagaattc aggaggttgg ttattaagct aatcatggag   1050000
     gcaccagaga aaggcaaagc ccgatgcaag gaaatccaaa aaaagataca agaagtgaag   1050060
     ggagaaatgt tcaaggaaat agatagcata aagaaaaaca ataaaaactt caggaaacat   1050120
     tggacacact tatagaaatg caaaatgctc tggaaagtct cagcaacaga actgaataag   1050180
     tagaagaaag aaattcagag ctcgaagaca aggtctttga attaacccaa tccaacaaag   1050240
     acaaagaaaa aagaataaga aaatatgagc aaagcctcca agaagtctag gattatgtta   1050300
     aatgaacaaa cctaagataa ttggtgtccc tgaggacgaa gagaaatcta aaagtttgga   1050360
     aaacatgttt gggggaacaa ttgagggaag cttccccagc cttgccagag acatagacat   1050420
     ctaaatacaa gagcacaaag aacacctggg aaattcatca caaaaagatc tttaaaacta   1050480
     aaactaaaac aagcaatcta caaaacctag gcacattgtc atctggttat ctaaagttaa   1050540
     gatgaaggaa agaatcttaa gagctgtgag acaaaagcac cacatgctta gtttcactgg   1050600
     atacaaaatt cttggctgat aattgttctg ttggaggagg atgaagatag ggctccaatc   1050660
     tcttctagct tgtagtgttt ctgctgagaa atctgctgtt aatctgatag gttttcgttt   1050720
     ataggttacc tggtgctttc ggttcaaaaa tttttttaat ttccatcttg atttcgtttt   1050780
     tgacccaata atcattcaag agcagtttat ttaatttcca tgtatttgca tggttttgaa   1050840
     ggttcctttt ggagttgatt tccagtttta ttccactgtg gtctgagaga gtgcttgata   1050900
     taatttcaat tttcttaaat ttattgaggc ttgctttgtg gcctatcata cggtctgtct   1050960
     tggagaatgt tccatacgcc gttgaataga atgtgaccat atgataggaa ataaactcca   1051020
     aaagaagccg aacaacaaca gtgacacaac ctgttgaaac ctctgggata cagcaaaggc   1051080
     ggtgccaaga ggaaagttca cacccctagg cgcctacgtc gaaaagactg aaaaagcaca   1051140
     aattgacatt ctaaggtcac ccctcaggga accagagaaa caagaacaaa ccaaatccaa   1051200
     acccagcaga agaaaggaat taatcaagat cagagcaaaa ctaaaggaaa ttgagacaaa   1051260
     aaaatacaaa agataaatga aacaaaaagc tggttctctg aaaagataaa taaaattgat   1051320
     agaccattag caagattaac caagaaaaga agaaagtccc aataacctca ataggaaacg   1051380
     aaatgggaaa tattacaact gacaccacag aaatacaaaa gatcattcaa ggctactatg   1051440
     aacgccttta cacacattaa ctagaaaacc tagaagagat ggataaattc tcaggaaaat   1051500
     acaaccctcc tagcttaaat caggaagaat tatataccct gaagagacca ataacaagca   1051560
     gcgagattga aatggtaatt ttaaaattac caagaacaaa aaaaagtcca ggaccagatt   1051620
     cacagtagaa ttctaccaga cattcaaaga agaattggtc ctattggtac tattccacaa   1051680
     gacagagaaa gcgggaagcc tccctaattc attctatgaa gccagcatca ccctaatacc   1051740
     aaaaccggga aaggacgtaa ccaaaaaaga aaactacaga ccgatatccc tgatgaagat   1051800
     agatgccaga aatccttaac aaaatgctag ctaaccaagt ccaacaacat atcaaaaaga   1051860
     taatccaccc caatcaagtg ggtttcatac cagagatgca gggatggttt aacatacgca   1051920
     agtcagttaa tgtgatacac cacataaaca gaattaaaaa caaaaatcac atgatcatct   1051980
     caatagatgc agaaacagca tttgacaaaa tccagcatcg ctttatgatt aaaactctca   1052040
     gcaaatcagc ataccaaggg acatacctca gtgtagaaaa agccatccat gacaacataa   1052100
     ttctgaatgg ggaaaagttg aaagcattcc ctctgagaat tgaaacaaga caaggatgcc   1052160
     cactgtcacc actcctcttc aacacagtat tggaagtcct agccagagca atcagacaag   1052220
     ggaaagaaat aaagggcatc catatcgata aagagaaagt caaactgtca ctactgatga   1052280
     tatgattgtt tacctggaaa accctaaaga ctcctccaga aagctcctag gactgaaaaa   1052340
     aaaattcagc aaagtttcca gatagaagat taatgtacac aaatcagtag ctactctata   1052400
     caccaacagt aattaagcag agaatcaaat caagaactca acccctttta caatagctgc   1052460
     aaaaaataaa ataaaatact tagaaatata ccttaccaag gaagggaaag acctctacaa   1052520
     ggaaaactac aaaacgctgc tgaaagaaat catagatgac acaaacaaat ggaaacacat   1052580
     accacgctca tggatgggta gaatcaatat tgtgaaaatt accacactgc caaaagcaat   1052640
     ctacaaattc aatgcaatcc ccatcaaaat accatcatca ttcttcacag aattagaaaa   1052700
     aacaattcta aaattcatat ggaggccagg cacggtggct catgcctgta atcctagcac   1052760
     tttgggaggc caaggcagta ggatcacttg aggtcaggag ttcaagacca gcctggccaa   1052820
     catggtgaaa ccccatctct actaaaaata caaaaactag ccgggtgtgg tggcatgtgc   1052880
     ctgtaattcc agctactact caggaggctg aggcaggaga atcacttgaa cctggaaggt   1052940
     ttgcagtgag cccagattgt gccattgtac tccagcctgg gcaaaagaat gagaatctgt   1053000
     cacaaaaaaa aattcatatg aaaccaaaaa agagcctaca tagccaaggc aagactaagc   1053060
     aaaaagaaca aatctggagg caacacacgg cctgattcca aactctacta taaggccata   1053120
     gtcaccaaaa cagcatggta ctggtataaa aataggcaca tagaccaatg taacagaata   1053180
     gagaacccag aaataaaccc aagtacttac agccaactga tctttgacaa agcaaacaaa   1053240
     aacgtaaagt ggggaaagga caccctattc aacaaatggt gctgggataa ctggcaagcc   1053300
     acgtgtagga gaatgaaact ggatcttcat ctctcatctt atacaaaaat caactcaaga   1053360
     tggatcaaag acttaaatct aagacctgaa actgtaaaaa ttctagaaac tataaaaatt   1053420
     tccaataaca ttggaaaaac ccttccaggc attggcttag gcgaggactt catgaccaga   1053480
     acccaaaagc aaatgcaata aaaacaaaga taaatagctg ggacataatt aagctaaaga   1053540
     gcttctgcac agcaaaagga acagtcagca gagtaaacag acaacccaca gagtgagaga   1053600
     aaatcttcac aatgtgtaca tctgacaaag gactaatatc cagaatctac aacaaactca   1053660
     aacaaatcag caagaaaaaa acagacaatc ccatcaaaaa gtggggtaag gacatgaata   1053720
     gacaattctc aaaagaagat atgcaaatgg ccaacaaata tatgaaaaaa tgctcaacat   1053780
     cactaatgat tagggaaatg caaatcaaaa ccacaatgcg ataccacctt acccctgcaa   1053840
     gaatggccat aatcaaaaaa tcaaaaaaca ttagatgttg gtgtggatgc agtgatcagg   1053900
     ggacacttct acactgctgg tgggaatgta aactagtaca gccgctatgg aaaacagtgc   1053960
     agagattcct taaaggacta aaagtagaac tactatttga tcctgcgatc ccactactgg   1054020
     gtatctaccc agaggaaaag acgtcattct acgaaaaaga tacttgcaca ctcatgtttg   1054080
     tagcagtaca attttcaact gcaaacttgt ggaaccaacc caaatgccca tcaatcaaca   1054140
     agtggataaa ggaactgtag tatgtatatg tgatggaata ctactcagcc acaaaaagga   1054200
     atgaattaat ggcattcaca gcgacctgga tgaggctgga ggctattatt ctaagtgaag   1054260
     taactcagga atggaaaacc aaacatcgta tgttctcact gacatgtgag agctaagata   1054320
     tgaggatgca aaggcataag aatgacacaa tagactttgg gaacttgggg gtaagggtga   1054380
     gagaggaaag agggataaaa gactacaaat atggtgcagc atatgctgct cgggtgatgg   1054440
     gtgcaccaaa atctcacaaa tcaccactaa agaacttatt catgtaacca aacaccacct   1054500
     gtattccaat aacctatgga aaaataaaca ttaaaaaaat taagaagact gaaagaatta   1054560
     aaaaatttaa aaaacagtaa gtagaagaga ggtatgtgaa gaaagttatg ggtatgaaga   1054620
     tgtatttttg gtacggaagg ttaaaaagaa aagagaattt ttttaaaaaa ggaagaatct   1054680
     tgcgtgataa atttttgtca taaagtaaaa tgactgctta ctttaaaaaa agaggtatca   1054740
     cacacaccag ggcctgttgt ggggtgggga gaggggggag ggataggatt aggagatata   1054800
     cgtaatgtaa atgacgagtt aatgggtgca gcacaccaac atggcacatg tatacatatg   1054860
     taacaaacct gcacgttgtg cacatgtacc ctagaactta gagtataata aatatatata   1054920
     tatataaaga aaattccaca cctgaagcca tgtgacaggc tacagacaaa acacagttaa   1054980
     aactttgttt catgtacaaa gttatttaaa atattgtaca aaatcacctt caggctgtct   1055040
     gtataaggtg tacatgaaat acaaatgaat tttgtgttta gacttgggtc tatccaagat   1055100
     atctcattat atatatgcaa atatcccaaa atccaaaaaa tataaaatct gaaacacttc   1055160
     tggtcccaag catttcagac aggggatcgt tagcctgtaa tgagagggag tatgggtcaa   1055220
     ttatgacgtt gaagattcaa aaaaaatttt tttttaattt tcagtcaagc tctaccaact   1055280
     acactaacta gaaaacactc aggcagattg ctttttgcta ggttaatatg gcatgattac   1055340
     aactgtgcat atgaataaca tgtatcagat tccttttgtt tgggaattat ttcattacaa   1055400
     cactaaacac tgcttatgta cgctgggtct ctttttcaaa gactacaaat aatgcttggt   1055460
     tctttgcttt caaaatatac caaacatgag gtacaagaat gtacccatcc atatttgacc   1055520
     aggggtagaa ttaaaaggtt tgggttgaag aagatgacat aacatcccaa gagggttgca   1055580
     tccctagggg tcatgggaac aaatgtggcc aagtcccttt ttaactggaa aggatttcag   1055640
     atccctgaac aagacaattc atcagtctta agcatagtaa gtcatcagca aataagagaa   1055700
     tgataaacac tagaaaaata atcaaatcat tgtctaaatt cattataaaa ctatatgaga   1055760
     aaactataaa actatatgag agagataaac agagacaaga agacaaggat aaaataaaaa   1055820
     aagaggtatc aaacaaagca gaacgcctca acatgtcata aaacatctga gtaagtcata   1055880
     ataaggtttg caaataatga atttacgaaa ggaattttgc gtgtgatcaa gatggctata   1055940
     attagaaggg aattatttat aagtctttct aaagactgag gtttgctatt aaaaatatgc   1056000
     taatataaaa ctaaagattt agtttcctgt gttagaacaa caaagttatc ttgaagtatt   1056060
     gatctgttct taaaactaca agaagttttt atttttaatt ctaaaatctg tttctttaac   1056120
     agaccatttc tattgcttcc tgggatccat ttactttccc tagtttcagg ttggaagtcc   1056180
     tcttcatgta aaacgagaat ttcatttctt gacatagtct tttcccccta aagcttctca   1056240
     gttttagatt tcagaacttc aacttctgtt gtatttcaca gcacatgatt tatagatcat   1056300
     gtatataaat atggatttat agatcatgta tataaataca gacttatcca tcagtgcctt   1056360
     cagctcttcc tccccatgag atggcctggg gtgatagctc tctctttcaa cttttttttt   1056420
     atcaactcct ataacatttt ttctcccatt ataactctgt tgttatggct caatgctgaa   1056480
     atgtttatcc tgaaagtcta gaaaacaaat gttctctcca gtataattcc agtataattg   1056540
     aattttccct tgtaaccagg aagtttctca tgctgttgct ttttctatgt gttcccctgc   1056600
     tcaggtacta gttatcttgt ttacatttct ctactaatgg tttacactta tagccttgga   1056660
     catactcttt ctctgcctaa ttaaactcag tgtctttttc atcagatttg acttccagtt   1056720
     tatctacatg ggcttcccat gaggagacac aatcacactg caggaggcat ttctttaact   1056780
     tttgggtaag tagcctaaaa aaaacaaaga ttttgtattt tattaggata attttttgtg   1056840
     ttgtctttat gaggtttttg gttacttagg taacttgagc tttgaagaag ttaggtcctt   1056900
     ttaatccatg taactttccg tattactctt caagtctttt gatgatcact ggttgaatac   1056960
     atggctatat ttaatagtga cctgagattc tgctttgatt agccatgttg aacctttgac   1057020
     atctttggca ggcttcctca ggatcaaaat tctaaaataa gtcttttttt atctagaatt   1057080
     gacttaggga ttttacagtt agacccctgg aaagcctcaa agaatttatc tctcatccta   1057140
     tagagaaaat aaatgattag gcttatttgg taaattatat gggaaacatt gtcaaataat   1057200
     aagtgatatt agatcttctt tcagttacat ttgtgggtat gctattgata tgatgtttca   1057260
     aagattatat aaattcatat cagtctataa cgttatcagc cataattttg gttatgctac   1057320
     atcttcttta aagctatatt tgtatggaga cgttactgat gtgagtatat tctaaagatt   1057380
     atgtgaaatt tataaaaccc tgatggttct gatgtgatgc tatcagtcac agtcatgatt   1057440
     ctgtttgcta ccttaaaaca ctatagtaat ttttaaaaag tcaatttcct tatcaattgc   1057500
     tgattataat gaatttttat cagatgttta accatggcca ttttgttttt gtgacccaga   1057560
     gttattgttt tgatttttct ccaaaagcat ttgtaatcag ctattgtcca aaattgcttt   1057620
     tcatggaaga gactcaaaca ggaactctta aatacggttt cccgataaaa gatcaatgga   1057680
     ctcaataaaa aattttcaga actctaataa agaaactgag aaattcaaaa acctccaatc   1057740
     aagctcaagc agaaaagctg acttcatgat attgaagaag tgatgagggg aatattttta   1057800
     tgaattttat ttgaagcatc gttcgttctt aaatgttttg tttgccagat ttaaggaaat   1057860
     tttctctcgt aagtcatcta tagtttacag taatttaata cagtatactt tttgtgaaca   1057920
     aagatgaaag caattatttt cccccctacg tgactcctcc aaaatttaga aactattcac   1057980
     aagtgttctt atgacgatgt ggccatttgt ataagtccag ataaaaacta gttgtctcct   1058040
     cactgcagga tgtaattgga aacatcaggt atattactaa ggctttggct gaaataccat   1058100
     atttgaaaaa tatacataga atgcctagtt tcagccgggc ggggtggctc acccctgtaa   1058160
     tcgcagcact ttggaaggcc aagccaagca gatcacccga ggtcaggagt tcgagaccag   1058220
     cctggccaac atggcaaaac cccatctcaa tcccgtctct actaaaaata caaaaattag   1058280
     ccaggtgtgg tggcgggcac ctataatccc agctgctcgg gagactcagg caggagactc   1058340
     ttttgaacat ggaaggcaga ggttgcagtg tgccaagatc acgccactgc actccagcct   1058400
     cggcaacaaa gtgagactcc gtctcaaaaa aagaaaaaag aatgcctggt ttccagggtt   1058460
     ccttataatg agtaaaaatc atcatttcct ggcaagccca gaaaccttaa aactgtaagt   1058520
     aaaagctaaa gcctgtcttg gtttggctta ccagcataaa gaggtattga agtacgagat   1058580
     tcctatgtga tcaatgtaca gaggaaaact tatgcttcca aagaaaagct gtaacacacc   1058640
     tgcttttaga ttgtagctct gtgcattatt ttcaagttct tgttttctac ctatagacta   1058700
     gatcctgaat tcttctagat tcttccaatc caactttctt ccatggagaa aaatgagaac   1058760
     tgctctgttc ctgaagccct ataagctgaa gctagataaa tgctaagaaa caagtctcat   1058820
     gcttgatgtc cgagccagac agaaagttca ccagactgcc caatgccacg actagagaca   1058880
     ttcaaactgc aaagcaagat gaggaatttc acattttcac tctgtagaca gcttctccca   1058940
     agtcatggga acaagactcc atatcataat gggactctta cctgtcctag ggcctacagt   1059000
     ttttacttgg cttcctattt tatttattca attaattgcc ttgaatcctg gattcattat   1059060
     ttattcaatt aattgccttt aatcctagac attgcataat cctattatgc aaactaggat   1059120
     tgtcatgtta ttactaattt tactttttat tttccctttt taaactttgt atctgttact   1059180
     tgctgaattt tttcagaagt acaacttcta acagaatatt gctagcccag cactttgaga   1059240
     taatagcaaa agaccacacc acagacaaaa ttgaacttaa tcatttactc caggtagact   1059300
     tagcctgaaa gccactgtct tcaaacctcc tttgttactc aaatgtggct aaaaggattt   1059360
     tgacactgac tcctagacat caatcactcc ttcaaacatg tgaccagacc agacacctgg   1059420
     gacaggccca tccaaatact gagggacatc agaacctacc acagcatggt ccatcagtga   1059480
     ggcttccaga gaaagacctt gaccaaaggg agaaaatatg atgaaagtcg tcagaatcaa   1059540
     aatggagtcc cttgtgttaa aaaacaaaca aacaaacgaa actctgacat ataaagccag   1059600
     agaaggctga gaagcattct catgcataaa cacctactag caaaaactat cacaaaagac   1059660
     tataaaaaac acagcctcgc acaaaggcca ttgcaacctt acacaaaaaa atacttctgc   1059720
     gaggacagct gcccagcaac tgcctgtgcg cctcatactg gcatcaccct tgttattgat   1059780
     ccttgtagcc aaagattatt tcaaaacgat catgtaatcg tcctcatttt tcctttaaaa   1059840
     acctttgtcg tcttttacct ccctgaatat gcacatagtt tatgatggta tgtgtgctcc   1059900
     cactgcagtg cttggggacc aagctccctc cacacctcac tgttcccaaa taaatatttt   1059960
     ctgttagagc ctctctctgt tcattattta gctcgacagg tatttggtac agcagccaaa   1060020
     acaggccaag acattgcccg gaatcttcca gcagtagact gggggcccgg atttgaccct   1060080
     ggtcagttgg acacgggctt ctctcactcc accagatgag gaacaaatag tgaggtgtgg   1060140
     cgggagcttg gtccccaaac agctcctccg gccatttgcc ctccccaccc acggaccaga   1060200
     gacaggacga gggtgagccc cggtgtcagc catgtgcccc aaccccggtg cctcatttgc   1060260
     tcctaggatg cgtcctgtag gtcatcttct tgcagctgtc ccgctcagtc tcccctcaac   1060320
     ccctgctcat gctcttggtg agcttatcaa tattatggtt tccaatagct tctgttcata   1060380
     gtttcaactc atatctgtcc ctaagtgtgg acttttattc cacgcgttgg cactggcagt   1060440
     cagctatgtg cctcctactc aaggcatctg aaactgcagg tgggtagtcg gctaagtcag   1060500
     gccccaaaag tgtcttcatc cagattccca gaactgtgac tctgagatct tacatggcaa   1060560
     aagggacttt gcagatgaga ttaagccaat gatcttgaga tgaggagagg ggcctggatt   1060620
     acttgggtgg ctccgaatgt gggcacaggt gtcacaggag ggaggcagag ggagacgtga   1060680
     gaaggagaag gtgccatgac tccgaagcca gaggctcctg ctgatcctcc ggccccaccc   1060740
     ccaccccacg gaccatgtgg tctttttaaa acatgaatct atcaggacgg cccattcaaa   1060800
     cccacagaga ggttcccact gcaccaagaa caaagtcaga actctcaggc ctgacccact   1060860
     gccctcggcc accctggagc cccagtgccc ccagacccgg tcctggcctc agagtgtaac   1060920
     aggctcactc ccaccaccca tctgcatggc aggcgccttg ccatcgcctc ccctctgaga   1060980
     gcacagacag ccacagctgg cctccgggtg aaaggatgga tgaacagtca ggccgggcga   1061040
     gtgctctcag agcctgctgg ccaacggagg cagccgctca ggcctctgcc tgagccatgt   1061100
     gcgatcacag ctgccgccca gctgtcccaa ggaggcagct cttggctttt cccctgggag   1061160
     tcgaattcgc agggagtccc atccacaggc ccctgggagg cgggaagaca tctcggtctc   1061220
     caaagccagt aacctgtgat tcccctgagg atggtgggaa cctggtgtgg ggcagatgat   1061280
     ctgtatttga gctttgtttc tctctagcaa ttatacctag gaaataccct aaaatgcacg   1061340
     acgcaatgca cattgacccc agcaaactcc ccaccctgcg cctgtttcta gtgccggggc   1061400
     tgctccagca cagcctgtgc acttgaccga atgtctacag ccctggccct cagccacact   1061460
     gtgttgtttt catgcacctt cacccaacag aattgctcca acagagtgat tcagggggct   1061520
     gccctccagc accacttccc ctttccatgt tacgtaaatg tccgttgagt cctgactgac   1061580
     tacagaaatc tgtcttctgg gggcatgtga tgccccaggt ggcccagggc cccgcacagg   1061640
     aatgtgttta cagctttccc ggcactgaag ccacagtcgt aactcagcct cctgacatgg   1061700
     gccagggccc tcgcctccgc acgctctgca gctcacccgt gtcgccacag cctgctgggc   1061760
     ctggacgcca ccctctaagg tggcactcgg ggcagaaaca tctgtttcca gctggtaaca   1061820
     aacaggacat gtctgcctcc cacacctaaa tcaatgcttg acaagcctgg cctgatctcg   1061880
     gggcgctcac caccagccac ctgcctgggc ccagccgacc tcctctccct tggtattttg   1061940
     agggaagggt ctgagccctt tctgcccccc tagacttgtg gacagaggcc ccccaagacc   1062000
     tggacagaaa tcaggccata gtctccagcc ccaaagagac ttccagaggc ctgcacctct   1062060
     cctctccagc cgaggccctg atgtctgcgg ctgtggccat agccggctct atacctgcca   1062120
     agtttattct agaaaacatt ggtttggaca agacctcaaa acggaattgg gaaaatgaaa   1062180
     gagaaaagag aaaggatttc tgctctcatt gtaaggacaa aaagcaaatt gaagaccagc   1062240
     actgatacag atcaataatc acaccagaaa aaaatatggc aaaatatcta tattctctta   1062300
     aggtgaataa gtgttctctc agaatgaggc taaggccagg aacagtgaaa agtaagactg   1062360
     ataaatttgc ccacaaggaa aaaaataaag tctctataag gacaaagcca ccaaaaacaa   1062420
     agtaagcacc ccaggaaaga aagtgaccaa agtatctaac atgaaagaga gcctgtattc   1062480
     gcatgagctc ccgagaaaca gaacccatag atgtggatgt ggatatcgac agaaataggt   1062540
     atcaacatag acacaggcta tggccacagg tatggataga gggagagatg cgagagagag   1062600
     attttaagga attggctcac acagttgtgg agacgaatgt ccaaaatctg cagggcaaac   1062660
     cagcaggctg gtgacccaga gaagagctga ggctgctgct cccgtccaaa gttcagcctg   1062720
     ctggcagaat tctgtcctcc ttggggactg ccgtctgttt tctcttaagg cctccaactg   1062780
     cctggatgag gcccacccac attatggatg atgatctgcc ttactcaaag tccacaggtt   1062840
     gaaatgttaa taacatctaa aaatcacctg cactatctag actggtgttt ggccaaatat   1062900
     ctgggtagta tggcctgccc aagttaacac ctaaaattaa ctatcagagt ccaagtaagt   1062960
     gaatgtaaaa aacaaacaaa caaacaaaca aaaacatgat gaatgagaca agctcgacct   1063020
     tgaagaagtt ttcagtggaa cgtggggaag aggagttctc tttagccagc tgcagacaca   1063080
     aatagccaat agaacacatg gagagtgtta gaagtcacaa atcgtcaatg gaaagcaact   1063140
     tacaatgtga tgttcattga acatctactg tgtgccaggc caagggctaa ggtggacacg   1063200
     tgcagtgtgt ctgcactgcg gcactaaggt cctggtgggg aatggataat acaattaaaa   1063260
     acaaatcaca aagaaaccag aagaggaaaa aaagcaatta aaaacaaata aatgctccat   1063320
     aaaggacaac agcgccagct gagggtgtgg ccatcaatgc caggcatcca gaatatgtcc   1063380
     ttggggacat ggaccactgg gaggtgcatc ccaggcaaaa ggagcaggag gggaagggtc   1063440
     caggccagaa acatgcttgc caagttcaag agcggtggga aggccagtgg gcttgggaag   1063500
     gagtgggctg gaaggacagg ccagaggcca gactgcatgg ggccagactg gccctgattc   1063560
     cagccttgga cttcactctg tggtgatagg agaccctcag agggacttgg ggaggggttg   1063620
     tgtgttttcc tgggagccac agaaaagtga gtgacactgg gctgaggaag gaagtcacag   1063680
     gacccaccag tgtctgcgca ggagtcccag tggaagagca gaaaaaatcc aggagaagat   1063740
     acaaaagtat tcaaaagtaa ttcaaaatag caaaaatcca aaatattcaa taatttaaat   1063800
     ggtattactt aaaatagtat tataaataat aaaaatatat tattaaaaat aatagattac   1063860
     tggctttaat accaatgtac agaaatccgt tttatttcta tagaccagca acaaacggaa   1063920
     aatgtgattc ttataaagat gccagatgtc tgcttctatt aagggcatac agggaactcg   1063980
     gaccagttag caaatctgaa agaagtattt taaaaatctg tttgaaggta atggagagat   1064040
     accagagtgg tgaataatag ccaccaaagt agctgccatt tgcagtcact tccccctgag   1064100
     cccctcccca aaagcatttg ctgattctgg gatagtcagt gagaagctga gcagaaatcc   1064160
     tgacaatctt agagctaggg agaaaaactc agaggccagg actcaccagg agaaaggcgc   1064220
     ttgggaagta catgcacttt ggattagaaa cccatggact ttccttatag gagcaaaggt   1064280
     gaaggaaatc atccctcaca ggaactgcaa ccagatgcac tttattcagg tccttagaaa   1064340
     agctcaacct ctaccactgg gttaagttga tctcagattt tgaattccac caggaaccag   1064400
     ccagaagcat atgaaaaccg tctctggaaa attataacgt cattttaggt tcagttgctc   1064460
     aacccaatac ttctgaatac agtatccagc acacaatcac aaagaaccac acatacaagt   1064520
     agacgaagca acatgagtaa gaacagcaaa aacggaagat caacagggac tccagacctc   1064580
     agctatggga attagcacat gaagactttc ttaatacttt ttatttcaat agcttttggg   1064640
     atacaagtag tttttggtta cacggatgaa ttctatagtg gttaattctg agattttagg   1064700
     gcacccatca tccaagtagt gtacactgta cccaatatgt agttttttat ccctcacctc   1064760
     ccccacccac ttgtaatccc agtgctttga gatgccagga ggtttgggac cagcctgggc   1064820
     aacatggcaa gaccctatct ctacaaaaaa tgtttaataa ttacctgggc atagtggtac   1064880
     ttgtctgtag cccccactat ctgagaggtt aggtgggata atcacttgag ccagaagttt   1064940
     ggggctacag ggagctgtga ttgaatcact gcactccagc ttgggtgaca gagtgaggcc   1065000
     ccatctctga aaaaaaataa aaataagtaa aattttaaaa ctgataaaat aacagtgcta   1065060
     ctaggtttga agagtttaaa cacaatctga aaatttcatc ataaattgaa aactataaaa   1065120
     cacacatagc agatcttttt taattcagaa ttttagaaca gaagtataca atgaagagct   1065180
     caaatgatgg gtttaagagc agacttaaca cagctgaaga aagaattagt gaatcagaag   1065240
     ttggatcaga agaaaatatc tagaatgaag gatagacaaa aacacagcat ggaactggag   1065300
     ggttaaaaga agagagtatc caccgataag gtctaacata taggtagtta gagtcccaga   1065360
     aggagaagtg agaaggaaga gaagaaacat ttttaaagag gcaattgcag aaatcttccc   1065420
     aaacttaagg aaatatccta ccccatcatt catatggaat aaaatacgta gaaaatctca   1065480
     tctatgccag gcgtggtggc tcatgcctgc aatctcagca actttgggag gctgaggcag   1065540
     gcagatcact tgaggccagg agcttcagac caacctggcc aacatggtga aaccctgtct   1065600
     ctagtaaaaa tacaaaaatt agctgggtgt ggtagtactc atctgtaatt ccagctgctt   1065660
     gggaagctga ggcaggagaa tctcttgaac ccgggaggtg gaggttgcag tgagccaaga   1065720
     ttgcaccact gcactccagc ctgggtgaca gagcaagact ctgaagaaaa taaaataaaa   1065780
     taaaaccaca tctatccata acattataaa actcctgaag actaaaaaaa aaaagaggct   1065840
     atcttaagag tagccaaggt cggggaagct cacaatacct ttaaaggagg accagtgagg   1065900
     ctggcagcta atgtcgtaac agaaacaaga acagaatgag atgctgtctt aggctggcag   1065960
     ctaatgtcgt aacagaaaca agaacagaat gagatgctgt ctttacagtg ctaaaagaaa   1066020
     tacctgctaa gccagggaaa ctatctttgg aaaataaaga tgacttttct ttttctcttc   1066080
     ttttcaattt tcattcccta gggctatcct aacaaggacc gtaaagaaca gaagtgtatt   1066140
     cttgcacagc tctggaagct ggaagtctaa actcaaggtg tcaggagggc tgggctctct   1066200
     ctaaagcctc tagggtagga tgcttcttgc cccttctagc ttcttttttt ttttttattt   1066260
     ctaaaacgac tttattgcta agaaccatga ttattactta aaatagattt ttcagttact   1066320
     gatataaaat atgttccttt tgagaacaaa tggacacata gaggggaatg acacacactg   1066380
     gggcctattg gagggtggag gccgggagga gggagagggt caggaaaaat aactaagggg   1066440
     cactaggctt aatacctggg tgatgaaatc atctgtacga cagatcccca tggcacacgt   1066500
     ttacctgcgt aacaaacctg cacgtgtacc cctgaactca aaataaaagg ttttttaaaa   1066560
     aatgttcttt gtgcttctaa aaatgtctaa ataatcctat atgcattttt cttatttgcc   1066620
     tcttatgcgt agcttacaga tttaaaagta caatgttgct tgttttgtat tcatcttggt   1066680
     ggatctaata atttacaaat aggacatagg catgctgata cagtcagcag ttaattcctc   1066740
     cttctgacag tggggtttgg acatacaacc agcaatgcgt gcaggtggag agacatagag   1066800
     gatctttttt tttttttttt tttgagatgg aattttgctc ttgttgccta ggctggagtg   1066860
     caatggttcg atctcggttc actgcaacct ccgtctcctg ggttcaagca attctcctgc   1066920
     ctcagcctcc tgagtagctg ggattacagg catgcgccac cacgcccagc taattttgta   1066980
     tttttagtag agatggggtt tcaccatgat ggtcaggctg gtctccaact cccaatctca   1067040
     ggtgatccac ccgcctcggc ctcccaaagt gctgggatta caggcgtgag ccaccacgcc   1067100
     ctgtacgaag ttttaggtca tctgtgattc acaaacgacc ggcttcgaga agctctggaa   1067160
     cgtgccaaac aaggcactgt ggagtagttt ggctccatgt gacctaagag aggaaaatct   1067220
     gttgtctttg ggaacattac acaattcagg taagatatgt gcatattctc aagggaaatt   1067280
     taaggtgctc atactcaata aataaacaca agagcagcgt tacagataat attcaaagag   1067340
     acagaggctt cttcatacct gatgtggtgg gaagaccact ggatgactgg acactgagat   1067400
     acgtggttga cagtctcgtc tctgaaacac atgatttgag ttttgagctt agacaaactt   1067460
     gtttttctcc agcatcagtt ttttcatcag taaacaataa taataatact gtgtttatgc   1067520
     caacatcatt aggaagatca ctttaaatgg tgtatataaa aggatccatc aactttaaag   1067580
     ccagtaccca atatacactc atagatatca gcgtattaca gaaaaatagt atttattttg   1067640
     tggtcaagca tatctggatt ctaatcccaa ttctactaat tgctccctta tcttttgtgt   1067700
     cccgtgatca tcaatgtttt tatcattaga atcttgataa tatctagctc accatgtttt   1067760
     tctggaaatt atatgagata acatatgcaa aggacttagt gcagcaccag atgtgactct   1067820
     tccagctcct gtggttgccg actgccctgg gtgttcccgg gcctgcagac gcatcgctcc   1067880
     agcctctgcc tctgtcttca tgcagtgttc tccctgtatc tgtgtctaaa tgtttctctt   1067940
     cttataatgg cactggccat agtggatcta ggacccactc tactgtagag tggcctcatg   1068000
     ttatcgtgat tatatctgcc aagtccctct ttccaaataa ggccacattc tcaagttctt   1068060
     ggtggacata aatttggggg tgactctatt caacccagta tactttctct tcctgagaag   1068120
     agcagtttgg actcaaagct gttcggtggg cagatcaagg gttggggggt gtctgcatgg   1068180
     tgggggtagg aggagagatg caggggccca gagcgggaag ccagtgtgga gtccaggctc   1068240
     ggagcaccca catgagcaga ggtgggcagc ctgatataga aagtcagatc ttaagtgggg   1068300
     tgagaagggt tccagatggg gaggagtgca cactagaatg gagggggagc cagaatggca   1068360
     gaggggaagg gagtactggc agagagggca ggttggtcac acaaggggat tggtcaagta   1068420
     agtaaataca ccgaaaagaa tagaaaagag agaaggcagt tccaaatacc aaaagggagt   1068480
     gatatggttt ggatctgtgt ccccacccaa atctcatgtc gaattgggtt tcaatctgtg   1068540
     tccccaccca aatctcatgt cgaattgggt ttggatctgt gtccccaccc aaatctcatg   1068600
     tcgaattgta atccccggtg ttggaggtgg ggcctggtgg gaggtggttg gatcacgggg   1068660
     gtgggttctc atgaatggtt tagcaccatc cccctagtgc tgttttcctt atagagtcct   1068720
     cccgagatct ggctgtttaa aagcacgtgg cacctccctg gtctcctttt ctcctgctct   1068780
     gggaatgtaa gacatgcctg cttcccttca ccttcacctt ccaccatgat tttaagttcc   1068840
     tgaggcctcc ccagaagcca agcagaagcc actatgcttc ccggacagcc tgcagaaccg   1068900
     tgagtcaatt aaacctcttt tccttataaa ttacccagcc tcaggtattt ctttatagca   1068960
     gttcaagaac agactacagg gagaaactgg aatgaatcct atggtactgg attggaattt   1069020
     gatccgtaag tttgaattca tggtttctaa cataaatagc cattatatag gagttgtcct   1069080
     gatttgtaga aaacacacta aaatattcat gaaggaggag gtgtcaggtc aaaaaaatac   1069140
     tctcttgaat gattcagata aaaattgttt gtacttttct tgcaacttcc aatttttggc   1069200
     tgaagctaaa gaaatatttt cagacaaaac agaatatttg ttgccagaag acacacatta   1069260
     aaggaactgc ttaaagatag gtttaagaaa gaaggaaaat gatcccagag gaagcctcag   1069320
     agatgaaaaa aagaatttaa gagtgtaaag tacacaggtg agtacatttt aagagttatt   1069380
     aactaataac aatgatgatg tgatggatag tgaggataga aaattaaaac aattcagatg   1069440
     catagcaata acatataagt aatgaagggc taataacact gcatcatcat atctgaaaag   1069500
     aagatcaaag aattagtgac aaatttcttg ataaatttaa ctaatagaaa aaaatgcaac   1069560
     tgtaagaaag tccatccaaa gggaagcaag gaaaaaggga taaaagaaga ccaacaagga   1069620
     cagcattatc tcacttattt gtggaatctt aaaagagttg agctcataaa agtagatatt   1069680
     agaatggtgg ttaccagagg ctagaggcag agagggagag aatggagcgt ggttggtcaa   1069740
     agggtacaaa gtctcaggta gacaggagga ataagttctg agatctattg catgacagag   1069800
     tgactatatt cagtaataat gtatattttg agataactaa gagagtaaat tgaaagggtc   1069860
     tctccacaaa aaaataagta aatgaggtaa cagatatatt aattagcttg attcaatcat   1069920
     tccacgttgt ttacatgtat gaaaatattg tggctgggca cagtggctca cacctgtaat   1069980
     cccagcactt tgggaggcca aggcgggtgg atcacttgat gtcaggaatt tgagaccagc   1070040
     ctggccaaca tggtgaaacc tagtctccac taaaaataca aaaatcagct gggcgtggtg   1070100
     gcacatgcct gtaatcctag ctacttggga ggctgaggca tgagaatcac ttgaacccag   1070160
     gagacagagg ttgtagtgag ctgagatcat gccactgcac tccagcttgg gtgacagagc   1070220
     cagactctgt ctcgaagaaa caaacaaaca aacaaaaaac attgcattga actccataaa   1070280
     tgtatctaat tatgatttgt caattagaaa ttatattaat tttaaaacca cattgctaga   1070340
     taggagattt aggctcaaat atatcagtat ttacattaaa tgtaaatggg ctgggagtaa   1070400
     tgtcactgaa aatggcagct ccaaaaatgt gtcccttcac gaaagcaaca attaagctgg   1070460
     caaaaaactg acagaattca ttttttcata actctagaat ataaccaaaa acttaaaaca   1070520
     aagggtgctt aatgaagaaa gaaactgcta aatttgggta agagagaatt gtggaatttt   1070580
     cttacctgcc tataaagccc tcattttcca gctcagaagt agccagggca acagcagccc   1070640
     atcttcccgg tgtggtttgc tggtgccaga gggagtaaaa aagccgttgt catcaaagaa   1070700
     ttgtgcttgc gaatctcaac ctatctgaca gctctctgag ggatcagctc aggatcttgc   1070760
     ctttgttttg ctcaccctgc tcctgccctt cctcccctgt gtcctacccc caacaaacac   1070820
     acacagaatt ctctacagga tggaacagcc tcctgggcag catacatgga aagtatttaa   1070880
     aggtatatac ttgtcacaac catctaggtc aacagataat agaaagggaa aacagtagac   1070940
     aggctaaaac gcctaggaag aaaaaggcta aggaaggaaa cgtggggaaa ttagaacttt   1071000
     aaaaaactcc cacatatact aaggaattca aacagccaca tgtatttcta gatgtatgct   1071060
     cagataagac ctgagaagac cctaagcttt tacctctggc tggtcattag gcttcacatg   1071120
     agcaggaagt aaaggctaag gcagagttgt aaatgacctg gttaagtgtt gcagcagtgc   1071180
     tctaacccag agccagcctg caaagaacag gagggtcttc ttcctctttc tttctttctt   1071240
     tcttcttttg gcttaagaaa tttaagaaga tctgtgaaaa cactagctga ccactaaact   1071300
     aacataacaa agatttcagt ggccacatat gacaaaagtc ttcacaaaag tagtttagac   1071360
     aagtcactaa acaactataa cacaaaacaa gcagcaataa caaaccctag ggagaagaaa   1071420
     gaatctagtt tcccatatta taatatttat tcaaattttc cagtttttaa caaagaagta   1071480
     tgagacatgc agaaaaacaa gaaatatgac cctttcacag gaaaaggtaa attaacggaa   1071540
     acttcccctg aggaagccca ggctgtgaac ttactagaaa aaggccttaa atcaactgtc   1071600
     ttaaatattc tcaaagagct aaaggaaacc aggagaacaa tgcctcagaa ctagtgatta   1071660
     tcagtaaaga gatagaaatt ataaaatagc gccaaataga attcttgaaa tgcaaagtac   1071720
     tgtaactaaa gtgaaaaact cactacaggt tcaagaggaa acttgagcag gtagaagaat   1071780
     cagtgaactt gaaggtaggc caattaaaat tctcccaact aaagagcaga aaaaagataa   1071840
     ggaaaatgag cttaatttga acaatatgat caataaattt gatctactag gcatatacag   1071900
     accttagaaa gagcagctac agaatgcacc ttcattttga gccaggtgga atattcacac   1071960
     attataccat ttatgaccac aaaggaagat ttaacacatt ttgaaaggct aaaagtattt   1072020
     agttatgatg tgatcacagt gctaagtcag aaataaataa caaagaggta agttaaaata   1072080
     cacatatgct tggaaaataa gataaaaact gctaaataac cctgaagcta aagaagaaat   1072140
     cataatggaa acattaaaat tttaaaaagt ttaaaatatt tttaactgaa taataaggga   1072200
     aatactacat atcaaaacat ggtgagatgc agcctaaaca gtgcttagag gaaaacttat   1072260
     agccttagaa gcatgtatta tgaaagataa aatcctgaaa gttgatgaac taaaaattca   1072320
     cctgaagaag ttaaaaaaga atgtaaaacc aactgaaaat agaaggaaag agatcattaa   1072380
     aataatataa gaaaatgatt gaactgagga taataaagca aagaaatgat caaccaagtg   1072440
     aattaacaaa attgataaat ctctgataac acttgttggg gaagaaaaga gaaaatgcaa   1072500
     ataactaatg tcagaaataa aaagggacga cacacacatc ctgcggatgt ttaaaatgta   1072560
     aaaaagaata tttgggaaag ctttaagcta ctaagtgtga acaattggat gaaatctaac   1072620
     tggataaatg taagtattaa aaaagtttaa tctacaattt ataaccttac cccagagaaa   1072680
     actgtgaacc agacatgatt tcatagggga tttctgatca atatttaggg aaatatcaac   1072740
     actcttagac aaactcttcc agggatctga agaagagtcc atgccctgta agctactgta   1072800
     tgaagccagt gtaatctgag ttccgttggg actgcaaccg ataaagtaat tcatcacaga   1072860
     aatgacctca ggaaactggc cttcagaacg ggcttctggg aagttgtctc acatatttga   1072920
     ggaccacagg cataattcat tgcccgcagg aaatggggca gccatacatg acatgtggtg   1072980
     gcaccatgat gtctcagact gtcactgaca ggagtgcatc caaagtacaa ggagagaaaa   1073040
     aggcatgggc aggtcaagat gcagtagcac tttgttgtca tggcaacaag agggctgcta   1073100
     aagattatgg agtcaccaac tttttctcat gaccatagag agtgtacaca ggggaaaaga   1073160
     aacagtgaaa gctatgaaca tagcaaaaag aacatataca gagagccatg gacgcttgaa   1073220
     ggagtccctt acctcctggt catgggtcag atagagctga tacccaacca attcactaca   1073280
     gatgaaagtc ttagaaattg tggggctaca acatgcaaag ttttatcatc atcccacata   1073340
     acactaaaca ggaggctcta actgcattct tgttggacat gtatccaatt tcagaatagt   1073400
     gttccaagaa tctgcctcct aggaccactt ttgttggtgt cagggatccc cagaagctga   1073460
     tcttgagaga atattttaag tggacagttt gtttgagagg tgatgccagg aaacactggt   1073520
     ggaagagttg gggatgaaag gaagccaata aaaagtgcac taataagcat gttaccatgg   1073580
     aaataactgt aactgaaatt caccagggcc ctcaggaagc cagtgtcaaa caaacacctt   1073640
     ctcagtacgt ccccccaaca cacaccagca gcaagggagt ttggggtatt tatatgctaa   1073700
     cttattagtc attggttgat ggcagtttgt gcagagcaaa atcctgtgca acttcaggcc   1073760
     tgttgtgcac agaaaccctg actaccagag aaaaccctca ggcaagaaga tgcagatgct   1073820
     agcagtttga ggttacacca gtatacacta aagtgtaaag gcccaaggca cctggagtgc   1073880
     accaaaaact attgccacag gtgtacaaat gttcaaatta acaagaaaat gccatgtcat   1073940
     cctccaaagt catggcacca attcacattc ccatcagcaa atctgagacc ttatccaata   1074000
     ccaatatcct gacctgaaac tagatcatca tttatttgca tctgtgtttc cttcttttat   1074060
     gtatcctttt gtctcttgat cagagttagc cacactcttt attttgtatg catcattccc   1074120
     tccctttcca taaaaaaaat ttatcaaata tggccgggcg cagtggctcc catctgtaat   1074180
     cccagcacgt tgggaggccg aggcaggcag atcaccagag gtcaggagtt caagaccagc   1074240
     ctggccaaca tggagaaacc ccgtctctac taaaaataca aaaattagct gggtgtcatg   1074300
     gcacacacct gtagtcccag ctactcagga ggctgatgca ggacaatctc ttgaaccttg   1074360
     gatgtggagg ttgcagtaag ccaagatggc accactgcac tccagcctgg atgacagagg   1074420
     cagactgtct caaaaaaaaa aaattatcaa atatgtatgt atatctgaac ttgtctgatt   1074480
     ctgtttgccc ttgagctatg taaaattata tcatactata tgcagttgtc tgcattggca   1074540
     ttttttacca atttttttac atcttgtttc caagattcat ctatgttgtt gtggggagtt   1074600
     gtaatttatt cacttttact gctgtatagg tttccagaat ttattttcct tggataattg   1074660
     tttcaaaatt tttctattat tagctgcctt actattgcat tcttgttcct gtctgttgag   1074720
     atcctgttat aggctgaata tgtcctcacc tctccccaaa ttcatatatg gaagccctaa   1074780
     tcctcaatgt gatggatttg gagatggggc ctttgggaag tgattagttt ttgatgaagt   1074840
     caggagggtg ggcccccacg gtggtggtgg tcatgagggt ggtcctcata ggaagaggaa   1074900
     gaaacggaag agcctcactc tctctgccat gtgaatgcac catgagaagg tggccatctt   1074960
     caagccagga agacagccct catcagaacc cagccacgct ggcacccaga ttctggactt   1075020
     ccaacctcca gaacaaagaa aactaaattt ttgttgttta agccacccag tctatggtat   1075080
     ttttgtcagg acagcccaca ctaagatgga tactagtggg agagtttctc tatgaaatat   1075140
     gcacaggagt ggaagtactg aggtatagaa tattatccct atttagtttt ctggaaagtg   1075200
     ttttatcaag catgttacca tgacaattaa ctagagtcta acacctctgg ggaattttga   1075260
     ttatcgttgt agaatatatg ccttagaatt atcaccaggg acaagtgacc tcaggcattt   1075320
     acacacacaa gctcccatca gccatgggtt aagagctgct ccaatgagta ctaattcctg   1075380
     gcacttctgt tctgtattca tttaggctct ggaagccaca ggaagcaatc agacaaaaag   1075440
     tttcaggtgt cgtggctggg catagtggct tatgcttgta ataccagcac tttgggaggc   1075500
     tgaagcagga ggatcacttc agcccaggag ttcagaacca gcctgggcaa tatagtgaga   1075560
     ccacatttct acaaaaaatt taaaaattag ctgagtgtgg tgatgcatgc ctatagtccc   1075620
     tgttactcag taggctaagg cagaaggatc gcctgagccc aggaagttga ggttgcagtg   1075680
     agccgtggtc tcaccactgt gtgcattcca gcctaggtga cagagcaaga ctctctctct   1075740
     ctctctctct ctctctctct ctctctctct ctctctctct ctctatatat atatatatat   1075800
     atatatatat atatatatat atatatatat atggctgggt gcagtggctc atgcctaata   1075860
     atcccagcac tttgggaggc caaggcgggc agatccctga ggtcaggagt tcaagaccag   1075920
     cctggccaac gtggtgaaac cccacctcta ctaaaaatac aaaaattagc caagcatggt   1075980
     ggtgcatgcc tgtagtccca gctatttggg aggctggagt aggggaatca ttgaacccag   1076040
     aaggcggagg ttgcagtgag ccaaggtcat gccaccacac tccagcttgg gcaacagagc   1076100
     gagactctgt aaaaaaaaaa aaaaaaaaga aaagaaaaga aaagaaaaaa gaaaaaaaaa   1076160
     ggaacacaga aggcacaaac agcatccact acagtcaccc ctcgcatgtc taaggtgcac   1076220
     ttgcacccca cagtgatgtc accacttcct attatttcct ctgaaaatga acccattttg   1076280
     agatgggctt gggccagaat tacccttacc cactgatccc tgtaaacact cactctaaca   1076340
     ggtgaggagt gaagagaata gggaggcttc tatggtattc tggtccctca attcattatc   1076400
     tgggttcatg gaacaactac agattctgct gttgcaactg atcccgaggc cttcattggc   1076460
     ctttgtcatc tcccttttgg agataggttt ctaagggttt cccttgacca tggcaagaac   1076520
     ttctgctggg ctatgctcta tgcctggtgg gatgatccaa acctccattt tctgcagaat   1076580
     tacagacccc aacaaaaatg tccttattcc aggtcttgag gtcccactcc ttcattattg   1076640
     gggtgctttc cttagtggaa gagaactttg agctcctgga ttcagccttc tttgtaatcc   1076700
     cactactctt acaatcaggt tctgggccca attttcagta gtttcttctc cagctgcagg   1076760
     agatggagtt ttttctaatg ctgtgatgaa ggttttctga aatttgcacc atttcctgag   1076820
     ttgaaagggg ttcaactgag cctgttgtta tatttcttca gtgattcaat ttcactaaac   1076880
     aacaaccaaa ctattccacc attcttatga tcctgtcttt ctcaaaggcc agagaaatta   1076940
     cataaggagc acatccccca acactgtaac actttctatc tatgtcccat tatcataatg   1077000
     ccaggagaag ttatttacaa agggttattt acaaaagtgc aagtgagatg taagagaacc   1077060
     actagggata gtgcagtaag ccaggttagg taacaggagg gctgttgctg cccacaaaac   1077120
     caaatctcaa ggtgttttag acaagacaca aactgtaaaa gacttattgg taaatttaac   1077180
     tatacaaaaa ttaaaaagtc atgtccatca aaagataaag aaagtgaaag acaagtcaca   1077240
     acccgggaaa atacatatgc aacacctaaa cttaacaaag gaatattatc aaaaatgtgt   1077300
     aaactcctac aaataaagaa gaaaaggata aatatcccaa tagaaaataa aggccaacag   1077360
     cttaggcaac atggcaaaac cccatctcta caaaaaatac aaaaattagc tgggcatggt   1077420
     ggcacacacc tatagtccta gctacttggg aagctgaggt gggaggatcg cttgacccca   1077480
     ggaggtcgag gctgcagtca gccatgattg tgccactgca ctccagcctg ggagacagag   1077540
     tcagaccctg tctcaatcaa tcaaccaatc aatcaagtaa aataaagacc aaaagacacg   1077600
     aacaggtatt tcagagaata aaaaaaatac ataaggccaa taaacagttt agaaggtgtc   1077660
     agcttcacta gcaatcagaa aaaaagtcaa atcaagagaa gaatattcaa gaaatcatgt   1077720
     tagcaaaatg atgttggcaa aaattaagaa gttgaaccat aatcattaag acatagattc   1077780
     acagcctcca agtacgaatg atcctccgac ctcagccccc caagtaactg ggactacagg   1077840
     tgcatgccaa catgcccagc taattttttt tcttttttgt ttttttcatt tatttgtaga   1077900
     gactgggttt tgccatgttg cccaggctag tctcgaactc ctgtgctcaa gcaatccgcc   1077960
     catcttggcc tcccaaagtg ctgggattac aggcgtaagc caccccaccg ggcccagaat   1078020
     gctttcttaa tctaaaaact cccctaagta gccaggcgca gtggctcacg cctgtaatcc   1078080
     cagcactttg ggaggccaag gcgggtggat cacgaggtca agagatggag accatcctgg   1078140
     caaacatggc gaaaccccgt ctctactaaa aatacaaaaa ttagctgggc atggtggcgc   1078200
     gtgcctgtat tcccagctac tcaggagact gaggcaggag aactgcttga acccaggaag   1078260
     cagaggttgc agtgagccga gatcacacca ctgcactcca gcctgggcga cagagcgaga   1078320
     ctccgtctca aaacaaaaac aaaaaccaaa ctcccctaag tagggctatt ttcctgtccc   1078380
     tgatgccacc ttgacaacat aagtcaacca ctgttccaca tttgtctttt aggaacgccc   1078440
     caggactgtc ggctgctgtc catggtgctg ccttggtcag acacgtggtg tccaagctgc   1078500
     tgtccgtggt tctgctttca tttttctgtg tagcataatg atttttttgt ttatttcttt   1078560
     gtttaattaa ttgattttct ctttttagaa cagcgctagg tttgcaaaca aattgaacaa   1078620
     aaagtccata gactttttac atacccagcg cctctctata catatacagt gtcacctact   1078680
     gtcattgtgc atctgtatgg cacatttgtt acaaatgatg agtcgatatt gatacattat   1078740
     cattaactga aatccgtagt ttaccttagg gttcactact tgtgttgtat agttcatgag   1078800
     ttttgacaaa tgtgcaatgt cacgtatcca ccatggcagt gttatgcaga atagtatccc   1078860
     ctatatttca ctattcattc ctcacccctc accccaaacc ctggcaactg ctgatctttc   1078920
     tactctctct gtagttttgc ctttccaaat atatagtttg gaaacgtaca ttatttagcc   1078980
     ttctccaact ggctgctttc acttagcaat atgtatttaa gattcctcca tgcgttctgt   1079040
     ggtttaatag ttttttgttt ttgttttttg tttttgatag ggtcttcctc tgtcacccag   1079100
     gctggagtgc agtggtgcaa tcatatttca ctatagcttc agcctcccag gctcaagcga   1079160
     tcctcttgcc ttagcctcca acacagctgg gattacaggc accaccatca tgctcatttt   1079220
     ttaatttttc atagagatgg tatctggcta tattgcccag gctgatctag aactcttggg   1079280
     ctcaagtgat cctcctgtct caacctccca aagtgctggg atcacagtgt gagccaccat   1079340
     gcctgccctt gatagctcac tttttattgc tgaataatat tccactgtcc ggatgtacca   1079400
     cggttaactg aaccattcac ctgtgaatgg tatcttagtt acttccaagt ttggcaattg   1079460
     cgaggaaagc tgctataaaa gctcacgtgc agatttttgt gtggacataa gttttcaact   1079520
     catttgaata aatacctagg agtgtgattg tatggtaaga taagatttag cttttaaaac   1079580
     tgtcaaactg tcttccaaag tggctgcacc atttgtattc ccaccagcaa tgaaagagag   1079640
     ttctttatgc ctcacatcct ccacagcatt tggcgttgag agctttttgt ttcatttgtt   1079700
     ttctttggga ttttctccat tctagtagct ggtcgtggta gctcattgtt gttttcattt   1079760
     gccatcccaa tggcacacca cgtggagcat attttcatgt gcttatttgc catccgcaag   1079820
     ccttctttgg tgaggtgtct gttcagatct tttgcccatg ttttaattgg gttgcttgtt   1079880
     ttcttattgt ttaaatttaa taatggttct ttaagtgttt tagatacaag tcttttatca   1079940
     gatatatatt ttctcctccc agtctgtggc ctgccttttt cattctcttg acaatgtctt   1080000
     tcacatgtag acattttttc attttaataa agctcagtta actaattttt ctttttatac   1080060
     aaattgtgat ttttgtgtta tatctaaaaa ctcattgcca agcccaagat catctggatt   1080120
     ttctcctgtt atcttctaaa atttcatagt tttaggcttt acatttgggc ctatgatcca   1080180
     ctttgagttg atttttgtga aaagcataag gcctgtgtct agattctttt tttttttttt   1080240
     tttttttttt tttttgcttt ggtgccatgt agtgaaaaga tgacgctttc tccattgaac   1080300
     tgcctttgct cctttgttga agatcagttg actatgttta tgggcgtcta ttatcagaaa   1080360
     gttttgttct atggatttat ttgtctatcc tttcaccaat tccacactcc tgattattgt   1080420
     ggcttcacag taagtcttgc ataaaagttg ggaagagtca gtcctctaac tttgttcttc   1080480
     ttcaatattg tgttgtctct tctgagtact ttcccttccc atataaactg tagaatcagt   1080540
     ttgccaacat ccataaaata atgtggtggg aatttgatta ggattgtgtt aaatctatat   1080600
     ataaagttgg gaagaactga catcttaaga atattgtttt tcttttcatg aacgtggaat   1080660
     atctctcgac tgatttagat cttctttcat ttcttttatc acagttttat agttttccta   1080720
     gtatatatct tgtatatgtt ttattagatt tatacccaag tatttcagtt tctggtgcta   1080780
     atttaaatgg tattcggttt tccactttaa atcccaattg ttcattgctg gtatataggg   1080840
     aaaaaatggc ttttgcccat taaacttaac gtccttcaaa tttgctttta ggccattctt   1080900
     gcgttgctat aaagaaatac ttgaggttgg gtaatttttt ttttttttaa aagaggttta   1080960
     attggctcac ggttctacag gctgtacaga aagcatggca gcctctgctt ctggggaggc   1081020
     ctcaggaagc ttccaatcat ggcaaacagc aaagggagag aagccatatc acatggccga   1081080
     agcagaagca agaaagagac tctgtggagg gggaggtggc acacacttct aacgactaga   1081140
     tctcgtctga actctgagcg agagctcact catcaccaaa gggctggcac aaaccattca   1081200
     tgaggatctg cccccatgat ttaaacacct cccagcaggc cccacctcca gcattgggga   1081260
     ttacatttca acatgagatt tgggtaggaa caaataccca aactatatca ttgctataat   1081320
     cacttattag ttccaggagg ttttttgtcg attatttggg attttctgca gagatatcat   1081380
     gtcatctgat gacaaacgca gcttcatttc ttctcctcca atctgtatat cttatacttg   1081440
     cttttcatgt tttaatgcat tagctaagac ttccagcatg gtgttaattg ggactggtaa   1081500
     ggggacctac tcacctgttg cttctcacca cgaagtacaa tgttagcagt aggttttttg   1081560
     tagatgtcct ttataaattt gagagtattc ccctttattc aaagtttgct gagagttttt   1081620
     atgataaatt gagttgtatt ttgtcagaca cttttttgtt atctattgat atgaccacat   1081680
     gagttttctt aatcttttga agagatggat tatattaatt gacttttagg tggaaagctt   1081740
     atattaattg attttgaatg tccaatcagc cttgtttcta ttactgagag ttctccagat   1081800
     taacagaacc aatagaatag ggtgtgtgtg tgtgtgtgtg tgtgtgtgag agagagagag   1081860
     agagagagac agagacagag agagagagag aattttaagg aattggctca catgggtgtg   1081920
     gaggcctgca ggtccaaaat catcaaatcg gtagtgtgga acctgaccta gggaagagat   1081980
     gatgttccag cttgagtcca aagatcttct ggaggcaaaa ttccatcttc ctcaggggac   1082040
     ctcaatctgt cttttttaat agtcttcaac tgattggatg aggcccaccc acattttgga   1082100
     ggatgatctg ctttacttaa agtctaacaa cttaaatgtt aatcctgtct aaaaaaatac   1082160
     cttcacaaca tccagactga tgtttgacca aatattgggt tccaagacct agccaagttg   1082220
     acaagtaaaa tcagccatca tatttgcata cctgggataa atctcaatgg gtatattatt   1082280
     ttttatacat ggttggattt catttgctag tatttcatta acattagatt ttacactgtc   1082340
     gatattgaag gtaagcaatc tttgaaaaag actactagaa ctcacccata gctgtttttc   1082400
     tccttccttt gactcctgct agactgtgtt tcccagtgcc atcacatgtg gatggagacc   1082460
     tgttaccagt tctgttcaat gagtaatgtg aataagtcag acagtacttt gaagagttgt   1082520
     gatgctgtaa caaaaaataa tattatgtgg ctcatgcctg taatcccagc acttcgggaa   1082580
     gctgaggcaa gaggatgatt tgaactgagt aattcaagat cagcctgggc aacatagcaa   1082640
     tactccatct ctagaaaaaa aattaaaaat tagccaagca tggaggcaca tgcctgtagt   1082700
     cccagccacc agggaggctg aggtggaagg atcacctgag cctgcagaag cagagggtgc   1082760
     agtgagctga gatcatacca ctgcacccca gcataggtga caaagtgaga ctctatctca   1082820
     aaaaaataaa ataaaaaact tgccttagaa tatgagaagc agttagcaag gaagctggtg   1082880
     tcaggctgga ggactgcaga tccttgtcat gccttaataa atcagttgtt aacatttgga   1082940
     aggcagagtc cctgcctatt cagcctgcag ctctagggta agagattgca aaatcaagta   1083000
     ttaagagttt gttggctact attagctccc ttcattaatt aactagagaa aagaggttag   1083060
     gtcagagaat tggtggcttt gtaagcaaaa attttaaaag aatagagaaa atccaaaact   1083120
     tacaaggagg gagtggaaaa gctggcttct tctagaaccc aaacaagaca ggacaccaag   1083180
     aaaggtttta gatgatgagg atggccaagg tgtgatttgc agtgtggcct tcccatccat   1083240
     ggcctctttc acagataatc taatttttaa tttattattt ttgtggatac ataataatta   1083300
     tatattaatg tatttatggg gtacacatga tattttgata catgcctaga atgtgtaatg   1083360
     atcaaatcgg ggtagttaaa atatctatca cctcaaaatt tatcatttct ttgtgttgga   1083420
     aacatttcaa atcttctctt ctagctattt tgaaatatac aataaattat tgttaactat   1083480
     agtcaccaca ctgtgctgtt aaacactaga acttattcct tctaaccaac tgttttgtgt   1083540
     gtgtgtgtgt gtgttttttt tttttttgag acagagtctt gctctatcat ccaggctgaa   1083600
     gtgcaatggt gcaatctcga ctcactgcag cctccgtctc ccgggttcaa gcgattctcc   1083660
     tgcctcagcc tcccaagtag gtgggattac aggcacatgc catatgccct gctatttttt   1083720
     gtacttttac tagaaacagg gtttcaccat gttgtccaga ttggtctcga actcctgacc   1083780
     tcaggtgatc cacccacttc agcctcaaag tgctgggatt acaggcgtga gccacaacac   1083840
     ccaacctacc caattgtatt tttgtatcca ttataccaac ctctcttatc tcctccccac   1083900
     agcagtgatc cttcccagcc tctggtaacc accattctcc tctctacctc tatgagatcc   1083960
     acatttttag ctcccacata tgaaagagga cacatgatat ttgactttct gtgcctggct   1084020
     tatttctctt taacataatg acctcccttt ccatccatgt tgctggaaat tataggattt   1084080
     cattcttttt tatggctgaa tagtattcca ttatgtatat ataccacatt ttctttatcc   1084140
     atttatccat tgatggacac ttaggttgat accctatctt agctattgtg aacagtgctg   1084200
     caataaatat gggagtgtag atatatctcc aaaatattga tttcctttct tttggatata   1084260
     tatgcatcag tgaaattgct gaattatatg gtagttctat ttttaatttt ttgaagaact   1084320
     tctacactgt tttcaataat ggctgtacta atttacattc caactagcag tgaatgagca   1084380
     ttctcctttc tccacatcct catcagcatc tgttcctttt tgtctttggt aataactatt   1084440
     ctaaatgggg taagataata tctcattgtg atttcgattt gcatttccta gatcattagt   1084500
     aatgttgagc attttttcat gtacatgttg gtcacacttg catgtcttct tttgagatat   1084560
     gcatattcat gtcttttgtc cattttaatg ggatttttct tgttgtggag ttgaattcct   1084620
     tgtatattct gaatattagt cccttgttgg atgaatttta tcccatcaaa aagttgactc   1084680
     ttcactctgt tgactgtttc cttgctgtgc agaagctttt tagtttaata tagtcccatt   1084740
     tgtctatttc tgtttctgtt gccagtcatt ttgaggtgtt agccataaaa tatttgctta   1084800
     tgacaatgtc ctgtagtgtt ttccctatgt tttcttctag tagttttata atttttagtc   1084860
     ttatgtttaa gtctttaata cattttagtt gtttttttaa ttcagtgaga gattgggacc   1084920
     tagttttatt cttctgcata tgaatatcca gttttcctag catcatttat tgaagaggct   1084980
     gtcctttccc agcactttca ttgaaaatca gtaggctata aatatgtggt tgttttgttt   1085040
     ctaggttctc aattctgttc cattggtctg tgtctatttt tataccaatg tcatgctgtt   1085100
     ttgtttccta tatcctcatg atatattttg aagtcaggta gtatgatgct tccagcttta   1085160
     ttctttttcc taagggttgc tttggctata caagctcttt tttggtttca taataatttt   1085220
     gggattgttt ttccatttct ttgaaaaaat gacattgata ttttaatagc gattacattg   1085280
     aatctgtaga ttgctttgga tagtatggtc attttaacaa tattaattct cccaattcat   1085340
     gagcatggaa ggtctttcca tttgtttgtg tcctcttcaa tttctttcat cagtgccttg   1085400
     tagttttcct tgcagaagtc tttcacctcc ttggtgaaat ttatttatat ggatttttgt   1085460
     agctattgta catgggattg ccttcttggt ttctttttca gctagttcat tatcagggta   1085520
     tagaaatact actgactttc atatattgat ttgtatcttg caactttact aaatttattt   1085580
     atcagaccta agagtttttg gtggagtctt tgggtttttt cctttcagcc agtatatatc   1085640
     ttttaagtgg aaaattctat ctgtttacat tcaaggttat tattgatacg tggggactta   1085700
     tttctgccat tttgttggtt gttttcttgt tgttttgtct atcctgtgtt cctttctttc   1085760
     tctcttattg tttatcattg tggcttggtg gttcctgtag tggtaacatt tgtgtctttt   1085820
     ctcttcctct ttttttgtat ttgccgtgcg gctgtaagtt ttatacattt atgtgttttc   1085880
     acgatggtag agatcatcct tttgcttcca agtatagaac tcccttaagc atttcttgta   1085940
     aggccaatat agtggtgatg aatttcccca ggctccagtg gctcacatag gtgctggctg   1086000
     tggtgggcag gatgggatga tcctttgccc cacccagtaa cagtagcagt gtgacgggag   1086060
     agcctgtcct cagggcatgt gaagtgcaca gtggccctgc tatggggtca ctgcccgtgg   1086120
     catgtacttc agccctggaa gcagcagcag cttcatcata gcttatttaa ttcagcggac   1086180
     caagagcata aacacctgag cttcctcttt ttatctgctc attctgcttc ccaagttctc   1086240
     aaggacttat gtggacagtc ttcccgcact tgccaaaaac tcagttcatt ctgttataaa   1086300
     tctcagtgcc tctacctgct acagacacgg agctcacccc gtcagtggca ccccttctga   1086360
     cccagaagtg tccctaggtg tcttcgtggc ctctgccatg ggtgctggta tgccattgtg   1086420
     ggaggtgggg ggcagtggga gtcaggaggt tgctcctctg ggtggagctg cctgccaatc   1086480
     aagatcctga ctctacctcc accccatgtg accaggctgc aggggcaggc cttagtgggc   1086540
     accggtggtg ctctggtcac aagacaccct ggaggtgaag ggggccttcc aattctaaag   1086600
     ctgtagctgc agacttgggc agtgcaaatc ctgtcaggac caaacaccaa attctatcac   1086660
     ccactcaagc ttgaagttaa gtgggaggga gagagtccct gcaggaccct tgctatagtt   1086720
     ggatgtttgt cccccaagac ttcatgttga aattcgaccc ccagtgttgg aggtggagcc   1086780
     tggtagaagg tgtttggatt ttgggtgcag atcccaaaat gctctccttt gagggtgagt   1086840
     tctcactctt attcctgcta gagctggctg gtgaaaggag cctggcatct cccctctttc   1086900
     ttgcttcctc tctccccatg tgatctctgc acataccatg gttcttgtac agcctgcaga   1086960
     accatgagcc aaataaacct cttttcttta taaattaccc agcttcagga attcccttat   1087020
     agcaacttaa tggacaaaga caacacctga cagccactct cccacatgcc tccagcaatg   1087080
     ctctgtgggt ttgcacctcc aactaagtca tttaggcagg atttaatgga aggacacctt   1087140
     gctgacacag atctgggctc agtgcaagag cccctgggac actgaggact ccagagctgg   1087200
     ctccggcaga gtgaagagta gcagaatatg ccactttcaa acaagaataa ttttgagccg   1087260
     aagacaatta aaaagaagca tacccaagaa aaaaatccct gcccactccc tttctaccag   1087320
     gaaaagcaga ggattcttag tccttggaga caactctaga tgcttatcag cccagagaag   1087380
     gcactagagg aatctacaca acaaactagc aagctatctt ctgttagctt cccctatata   1087440
     tttacttccc agaatttgct gccctacagg ttgaaagccc ctttcctttg tcttgccaat   1087500
     tctgtaaaaa tgattgccca tttgctaaga tgctccgtaa gctcacgttc caaccgcccc   1087560
     tttgaattcc tcatctctga cggctcccac atagaggcat aatgcacatg ttagtaaact   1087620
     tttgtttgtt tttctcatgt tagtatcttg ttaatctaat tgacaagacc ccagctaatg   1087680
     aacctaagat gggtagaagg gggaaaaatg tttcttcccc tacaagagca agcacctccc   1087740
     cacacactgg aggaggagag agagccgctc cgagatctgc aggaacagag gctccttggg   1087800
     ctgtctgttg aggaaggcaa taaggtccag gcaaccctgc aaggagagag ccagggtcac   1087860
     cacttagcat cagcatcccc ttcctctgat ctcctgcagg cagccagccc acccaaagag   1087920
     ctgacccatc cagcacccaa ggagtgaggc aggccagagt ccactccaag ggcagaggct   1087980
     gtggggcagg tggggagcca gcctggagga tgttagcaga gccaggtggg tcatggcacc   1088040
     cacagcctcg gggatccagc ttgagagaga ccctgagggc caggctgata acagatgagt   1088100
     tgtgatgaca gggctcattg tcagacactg taggctgtca ggctgtcaac ctgagcagac   1088160
     taccaagtct tcccatccgc aaaacagctc accccagcta tcaggggtgg gcacccagag   1088220
     gagagaccat tctcatctcc acctgaactc ttttggtgct cccagaacat tctgatgctg   1088280
     aagaggagtg tagaatcaga tagaaacaat tcctcttgta gcttccatga tggttggatg   1088340
     gtaaagtaca actggctttg ttagttcatt ttgcattgct gtagaggaat acctgaggct   1088400
     cagtaactta taaagaaaag aggtttattt ggctcagggt tttgcaggcc ataaaagaac   1088460
     cctggtgcca gcatctgctt ctggtgaggg catcaaggag cttacaacca tggcagaagt   1088520
     tgaaggggga gagagcatgt aacatggtga gagagagagc aagagaggga aagaggaggt   1088580
     gccaagctct tttaaacaac cagctcttgt gtgaaccagt agagcaaaac tcactcatca   1088640
     ccaagaggat ggcagcaagg ccattcatag ggatccatgg catgacccaa acatttccac   1088700
     taggccccac ctccaacatt ggggatcaca tttcaacagg agatttggag gggacaaata   1088760
     tccaaaccat gtcactccac actgtccccc caaatctcat gtccctctca cattgcaaaa   1088820
     tataatcatc ccttcccaat agtgcccaaa aaatctcaac ttgttctagg atctactcaa   1088880
     tcaaaactcc gaagtttcat ctgagactca aggcatgccc cttccaccta tgaggctgta   1088940
     agatccaaaa caagttgcta cttccaagat acagtgatgg tacaggcatt gggtaaacat   1089000
     ttcccttcct tccaaaagga ataaattggc caaaagaagg agggggaaga agcaacaggc   1089060
     cccacacaat tctgagagaa agacattaaa ctttaaagct ccaaaataat ccttgactcc   1089120
     atgtcctgca tccagggcac actggtgtga ggagcgggtt cccaaggcct tgggctggtc   1089180
     tgcccccatg gctttgcaga gtgcagccca catggctgct ctcatgggtt gtagttgagt   1089240
     gcctgaatct tttccaggct ctgggcgcaa gctgctggtg gctctaccat tcttgggtat   1089300
     ggagggcagc agccccattc ccagagctcc actaggcagt accccactgg ggactctgtg   1089360
     aaggggctcc aaccctacat tttccctcca cactgcccta ctggaggctc tgtgggagaa   1089420
     ctccacccct gcggcaggtt tctgtctggg ctcccaggct ttcttttttg tctctctctt   1089480
     tttttatttt tatttcaata gttttaggtt tttggttaca tagataagtt ctttcatggt   1089540
     aatttctgag atcttggcac acttgtcacc caagcagtgt acgctgtacc caatatgtaa   1089600
     tcttttatcc ctcacctcca tcccccactt gccctcaagt atccagagtc cattatatca   1089660
     ttcttatgcc tttgcatcct tatagtttag ctcccactta taagtgagaa catatgatgt   1089720
     ttgtttttcc attcctgagt tatttcactt agaataatgg cctccaactc catccagttt   1089780
     gctgcaaatg ccactatttt attccttttt atggctgagt agtatcccat ggtgtatata   1089840
     taccacattt tttatccact cattggtcaa tgggcattta ggctggctcc atatttttgc   1089900
     aattacaagg agttgcaatt gcactgctct aaatatgtgt gtgcaagtgt ctttttcata   1089960
     taatgactta tttttttctg gatatcgaat agtgggactg ctggatcaaa tggtgattct   1090020
     acttttagtt atttaaggaa tctccatact gttttccaaa gtggttgtac tagtttacat   1090080
     tcccaaccag gagtgtaaaa ctgttccctt ttcaccatat ccatgacaac atctgttagt   1090140
     ttttaatttt taattatggt cattcttaca gaagtgaggt ggtatctcat tgcagtttta   1090200
     atttgcattt ccccaaaaat tagtgatatt gagcatttct ttatatgttt gttggccatt   1090260
     tgtatatctt cttttgagaa ttgtctattc atgtcgttcg cccacttttt gatggtattt   1090320
     tttttattga tgatttgttt gaattccttg tagattctgg atattagtcc tttatcagat   1090380
     gcatagtttg caaatatttt ctcttattct gtgggctgtc tgtttactct ctgattattt   1090440
     cttttgctgt gcagaagttt tttttgtcta attaggtcac atttatttat ttatttattt   1090500
     atttttggtt ttgttgcact tgcttttggg ttcttgatca tgaactcttt gcctaagcca   1090560
     atgtctagaa gagttttacc aatgttatct tctagaattt tcatggtttc aggtcttaga   1090620
     tttaagcctt tcatccatct tgagttgatt tttgtataaa gtgagagatg aggatccagc   1090680
     ttcattcttc tacatgtggc ttgccaatta tcccagcacc atttgttgaa taggacatct   1090740
     tttccccact ttacattttt gtttgctttg tcaaagatca gttggatata agtatttgac   1090800
     ttcatttctg gattctctgt tctgttccat tggtctaatg cctattttta tacaagtacc   1090860
     gtgctatttt ggtaactata gccttgtagt atagtatgaa tttgggtaat gtaatgcctc   1090920
     cagatttgtt caccttgctt agtcttgctt tggctatgtg gggtcttttt tggttccaaa   1090980
     tgaattttag aattgttttt tatatttctg tgaagaatga tggtggcact ttgatgggaa   1091040
     ttgcattaaa tctgtagatt gtttttggca gtatggtcat tttcacaata ttgattctac   1091100
     ccatccaaga gcatgggatg tgtctccatt tgtttgcgtc attgatgatt gctttcagca   1091160
     gtgttttgta gtttttcttg tacagatctt ttacctcctt ggttaggtat gttcctaagt   1091220
     attttatttt ttgcaggtgt tgtaaaaggg attgagttct tgatttgttt ctcagcttgg   1091280
     tcattgttgg tgtatagcag tgctgataat ttatgtacat tgattttgta tcctgaaatt   1091340
     ttactgaatt aatttaacag atctagaagc tttttggatg agctttaggg ttttctaggt   1091400
     atgtgatcat atcatcagtg aacagcaaca gtctgacttc ctttttactg atttggatgg   1091460
     gcacccagac tttcctatac accctctgaa atgtaggtag gagctgccat gcctccttca   1091520
     ctcttgtatt ctgcccacct gtgggcttag caccatgtgg aagctaccaa ggcttccagc   1091580
     ttgcaccttc tggagtggca gcctgagctg cacctgggac tctttgagcc ttggctgaac   1091640
     ctggagcagc aacctcccaa gataccacag ggaagcaagg ccccagccct gacccccata   1091700
     acaatttcat cctcctaggc ctctgggcct gtgatgggag agactgtccc aaagacttct   1091760
     gaaatgcctt taagaccttt ttctcattgt ctttgctatt agcatttagt tgccttttag   1091820
     ttatgctaat ctctctagca agtggttgct ccataaggca tttgggttcc ttgcctgaaa   1091880
     aggctctttt cttctcaacc tcatggccag cctgcaaatt ttccaaatgt ttatgttctg   1091940
     cttacctttt aattataaat tctaatttta agtcattttt tttgctccca tatttgattt   1092000
     aggccattca aagcagccag gttacttctt ggatgctctg ctgctttgaa atttcttcca   1092060
     ccagatacct taggtcatca ctgtcaatct cagcctttca gaaagccctg gaacatggac   1092120
     acaatacagc caagctcctt gctgggggca taacaagcat cacctttact ccagttccta   1092180
     ataaattcct catttctacc tgagacctca tcggcctggc ctttactgtc catatttcta   1092240
     tcagcacttt gatcacaact atgtaaccag tctctaaaaa gttccaaatt tcccctcatc   1092300
     ttcctgtctt cttcctgggc cctccaaact cttccaacct ctgcccatta cccagttcca   1092360
     aagccacttc cacattttca ggtatcttta tagcaacacc gcactccttg ctaccaattt   1092420
     tctgttttca tccattttgc attgctataa aagaatacct gaggctggat aatttgtaaa   1092480
     gaaaaggaag tttatttggc tcactacaag ctgtacaaga agcataatgc cagtatctgc   1092540
     ttctggtgag gcctctggaa gtttacaatc aaggcagaag gcaaaaaagg agccagcata   1092600
     ggacaggcat ggtggctcat gcctgtaatc ccagcacttt gggaggccaa ggcaggtgga   1092660
     tcacttgagg tcaggagttc gagaccagcc tgaccaacat ggtgaaaccc catctctact   1092720
     aaaaatacaa aattatccgg gtatggtggt gcacacctgt agtcccagct actagggagg   1092780
     ctgaggcagg agaattgctt gaacccagga gacagaggtt gcagtgagtg agccgagatc   1092840
     acaccattgc actccagcct gggcaacaag agtgaaactc catctcaaaa aaaaaaaaaa   1092900
     aaaggagcca gcatgtcaca tggtgagaat ggtgagaggg agaaagagag ataagaagga   1092960
     agtgtcaggt ttttttaaac aaccagctct tgtgtgaact aatagagtga gaaatcaccc   1093020
     atcaccaagg ggatggcacc aaaccattca tgagagatct ggccttatga ccccaatacc   1093080
     tcccaccagg tctcacctcc aacactgggg atcatatttc aatgtgaaat ttggagggga   1093140
     caaacatcta aatcatatca ggaactccat ggtactgcta acccccaaat ctctaggggt   1093200
     gatattgagt gacatgcatg gtactgaacg atacttggtc aggttctccc agtgaagccc   1093260
     aagcaccctc agggtacagg gctgggaggc ccaatcacac cagctcagcc caggacttgg   1093320
     ctgagtctgc ccggtattag aaggtagcag acctagaact gggttgaaca agatcaggcc   1093380
     actttgtgca tgttgccaaa ggagaaactg aaggatcaga agtgggcagt aagttttcag   1093440
     tgagcccagg ggtcataagt agcaaaatgt ggaattcagt aaggggtaga ggctggaaga   1093500
     gttttgaggt gtgtgttaga aaaagcctag gtttccttga agactatgtt agaaatttgg   1093560
     acattaaagg tgatcctggt gggggctcag aaagaaagag gagagctata gagaaaactc   1093620
     ctgtcatctt ggagaataca taaattgtca cgaacagaat gttgctagaa acgtgaatgt   1093680
     taaagatgcc tctgctgagg ctccagacag aaataaggaa ggtgttattg aaaacgaagg   1093740
     tgacccttgt catacagtgg caaagcactt ggctgaactg tgttccgttt tgtggaaagt   1093800
     agaaattata catgatgaac ttggatgttt agctgacgaa gttcctaaga aaattgttga   1093860
     aggcattctt gatttctcct tgctgcttac agtaaaatat gagaagagag agatcagctg   1093920
     aagagggaag tgctgaggtg gctgggtcag ctgacccaag ggcccttcaa gctgatttag   1093980
     gctgctcacc ttggtctcag aatcaccacg gccatggccg gtcagtcaca ggtgatccag   1094040
     cagctgctgc aggccgagaa gcaggatgcc aagaaggtgt ctggggccca caagcaaaag   1094100
     aaccagaggc tgaagcagcc caaaggagca gcttaggctg aaattgaaca gcactgcctg   1094160
     cagaaggaga aagagttcaa ggccaaggaa gctgcagcac taggatctcg cagacccagg   1094220
     ataaccatcc tccagaccta cttccagcag aacagggaag aagtcttaga taaccttttg   1094280
     gcctttgtct gcagcatcca gccagaaatc cctgaaaact atcgcataag tagatattaa   1094340
     aagagagaag cacctgttaa atggactggc actttagatg ccctcatgga ataagaagct   1094400
     ctagttatat tcttataaga agacattaac ttatctctgt atattataga gtaggcccat   1094460
     tcacttttcg gagagaagca aatccagttt ctttgtacag acttagaatt tatctaggct   1094520
     gggcatggtg gttcacgcct gtaatcccag cattttcggg ggccaaggcg ggcagatggc   1094580
     ttgagctcag gagtttgaga cctgcctgaa caacacagtg agactgtgtc tctacaagga   1094640
     aatataaaaa ttagccagat atggtggtac atgcttatag tcctacctac ttggaagagt   1094700
     gaggcaggag gatcacttgg gcccggaagg cagaggttat agtgagccaa gattgtgcca   1094760
     ctgcactcca gcctgggtgc tagagtgaga ccctgtctca aaaaaaaaaa acttaaagat   1094820
     ttcatctttt tacctcatgt ttcttaggaa tataatgggt aaatattgtc tattttatta   1094880
     tgccttttgg ctcaagcaat atatatacat atatatatat cactgttgat gttttctttc   1094940
     ttgtatcaag tttaaaagaa aaagcaaaac aatcctttga aaaaaggaag gaattaaatc   1095000
     attttttcct taatgctttt ttgaaggtca ggggctttat ctatgaaaaa gtagtagttt   1095060
     ctgtaacctg tgtgaagcat cagccagcct taaagtagtc cattgctgct aataattaga   1095120
     acagtgaata ttactagtat aattgtttca gcttcttaat caaaataact agatgataga   1095180
     attcaagaac ttgttacatg ttattactcg gtgtactact aatcatttaa aagtaaagcc   1095240
     tatcatgcaa ataaataaat aaataaataa ataaatacat gagaccagat aaaatgaagg   1095300
     aagaatgact ttgagatcag agccacaggt gaggaggctg ttggggctgc tgttgccacc   1095360
     ttgggcccag agtgtggggc caccccagtg ggcctggaag atggagcatt aaccgaggag   1095420
     gatgaatctt gagacttaaa tctaataaag ggccaactag aagcccctag tgctcatcat   1095480
     cctcataaag aaagccagaa aagtcaatag acaactatat tttaacgaaa ataactgagg   1095540
     gagagcactg agtgcatcag aggagtaaca gaaaccctga taagcacaga aatgtgggct   1095600
     ggccacatag aaaacagaag gaaacacacg gcctccacca ctccatcccc caaccaggat   1095660
     tatctgggaa tcaggaggaa tttctgacat gaggaggtaa gcaagaggat cccagtggca   1095720
     cccactaaca cctcggacac ctgcagacct cgccactggg gtcccctgca gtttgcactg   1095780
     gagggagttg cctggaggcc acatagctgt gctctccaaa aaaggtgctg acaccgtacc   1095840
     ccactcccac atggctagtg cttcaccaca ctggaatagg aactatggct ggagtgtgtc   1095900
     ttacccaagg agtgagtagt cacagctccc cttcatctct gaggctaagc tgccactgaa   1095960
     caacccagac tagtggtccc acgtccccaa gtcaagctat gggcatctat taaacccttc   1096020
     cccacagagc caagtggatc agctctaccc actcctcccc ctgacccttt gagtccaagc   1096080
     taaagcagtt tcttgcctcc tagcaaaaca ggactttggc catgcctttc tagtgtctac   1096140
     gttgaagcag caccctgcat tccaggaact aatgccttgg ccacccagaa ggtcacacac   1096200
     tgcagtgctt aggctgaagc agcaacctgt atcctacaga aatggtgcct gggccactca   1096260
     gaacaatcac accctctgat acataaatgc aggactttcc ttacagcaga gaaggcctga   1096320
     gctgaagcaa cacatcacct tctagggaat cagtgccctg gcttaagctg agcagcagca   1096380
     catcccaagg ctgagctgat atagtaccct atgccccagg aaaacagagc agtggctaag   1096440
     ctgagacatc tcagcctaca ggccaaacaa ctgtagtgcc ctgcttccct ggagcttgcc   1096500
     taggccccta gggtctgagc tgctgaggca ccctctccct gggaagtaga atcagcactg   1096560
     tgctgttccc tgcctactcc ccaccaaggc ccaaattaca gctatgctct gccatcttgg   1096620
     gatacttgct gacactgtac ctggtctcac atagtctggg atcctgctga gccctaccat   1096680
     cctagggtct agagtcaata ccatacagtg cctcatcccc tgggaccaaa gtttccattg   1096740
     tgccctattg gctcagcttc ccaaattgca gccataccat actccccagg tccacacctc   1096800
     cagaacaccc cttcttcccc agagttggac aaatgctgca ccccccagga atagactcac   1096860
     agctacaacc tggccccttg agcaaaacct gttaaagggt acctcagagt cacagatcct   1096920
     agcactgtga gccacctaat ccaaacctgc cacagaaact gaacctacac cccaagaccc   1096980
     agatgccaca gtagattctt gaaaccagga gactaggact ccagttgcac agctactcta   1097040
     attttctgca cctggaatcc agcaccattg cagctgcacg ttggccacat tagacctgct   1097100
     accaagagag agccccttga ctaaatctcc tcattgtggg gaaacaagaa gaggaggact   1097160
     acaaaagccc ttgacactga gtacattaac ctgtgccacc actacatcca caaacttcta   1097220
     cagcctagtc cactgaggtg cccacagtta ttgctgaggt tgaatgcagt tgaagaagac   1097280
     gcatggaagc tatgccactg cacctattca gaaacagagt catcacactt tcccaaccaa   1097340
     aacactaaac ccaactgcag gtgaaattgt ttctctaaaa aacccactct tgaaagtttg   1097400
     gaagaggtga ctatttcgcc agatgcacag acatcaatgc aggaacacaa taaacatgag   1097460
     aaagcaagga aatatgacac caccaaagga acataactct cattaataga cctcaatgga   1097520
     aaaagaaatc aatgtattgc tggaaaagga attcaaaata atgatcttaa gataactcaa   1097580
     taagatacaa gaaaacagat aaacaattca atgaattcag gaaagtaatt tacagtatga   1097640
     atgaaaattt caacagagat agaaataaaa aagaagcaaa ctaaaattct gcaggtgaag   1097700
     aattcaatga ataaaattaa aaaatacagt aatgagcttc aacagcagat atgatgaagc   1097760
     agaagaaagg aagagataga tcccaataca ataatagttg ataacttcaa caccactctc   1097820
     agcactggac agataatcca gacagaaagt caacaaagaa gcattcaatt taaaaagcac   1097880
     tttagaccaa atgcatctaa cagatattta cacaacattt catccaacag ctgtgaaata   1097940
     cacattcttt tcatcagcac atggaatatt ctccaggata aaccatacag taagtcacaa   1098000
     aacaagtctc aacaaatttg aaagaattaa aattatatca aatatcattt atgaccacaa   1098060
     taaaactcga aatcaataag aggaactttt aagactgtac aaagacatgg gaaattaaat   1098120
     aatgtactcc tgagtgacca ataggaatta agaagggaat tttagaatgt caacaaagaa   1098180
     aatgaaaata gaaatgcaac gtactagaat ttataagata tagcaaaaag cagcattaag   1098240
     atggaagttt atggcaataa atacctacat caaaaaagca gagagatttc aaataaatct   1098300
     catccttgaa aatgcatgtc aaggaactag gaaatcaaga ataaaccaaa cccaaaatta   1098360
     gtagaaagaa agaaataata aagatcagag cagaaataaa tgaaattcag actaaaaaaa   1098420
     aaaaagatca acaaaatgaa aaattgaatt tttcaaaaga taaacaaaaa tgacaaataa   1098480
     ttagctagac taaacaaaaa agacccaaaa gaatgaaatc agaaatgaaa acagaaacat   1098540
     tacaactcat atcacagaaa cacaaaggat aattagagac catacaccaa cagattagaa   1098600
     aacctagcag aaacaggtat atttctggac aatacaacct accaagactg aacccagaag   1098660
     aaatagaaag cctgcacaaa ccagtcatga gcattgagat tgaatctgta acaaaaagtc   1098720
     tcccatcaaa ggaaagccca aagaaattcc tgttgaaata ccaatgacat tcctatcaaa   1098780
     atgccaattc tacagtaatc caatggcttc agtggggaat tctaccaaac actaaaaaaa   1098840
     ctaataccta ttcttctgaa aatcttcccg aaaattgaag aggagggaat tcttctaaat   1098900
     tccttgtatg aggccactat tatggtaatt atgattatga ttatgattat gtgattatta   1098960
     ttatgattcc aaaaccagac aaggacacaa caaaaaaagc aaactatagg ctaaatactc   1099020
     tgatgaagat aatagatgaa aaccttcaac caaatactag caaattgaat ccagcagcat   1099080
     attaaaaaca ttatttatca tgatcaagtg agatttatcc caggaatgca aggatggttc   1099140
     aacatatgca aatcacatca acagaatgaa ggacaaaaac cgtattatca tcataacaga   1099200
     cacagaaaaa gtatttgata aaatccaaca tcccttcata aaaattctca gcaagttagg   1099260
     tatgaaagga atatacctca acacaataag agctatatgt gacaaaccca cagccaacat   1099320
     catactgaac atgcaagagt tgaaagcttt tctctaaaat ctggaacaag ccaatgatat   1099380
     caggccagat gtagtggctc atgcctctaa ttctagcact ttgggaggcc aaggcaggag   1099440
     gatcacttga gcccaacaaa ggaaacaatc aacagagtga aaagacaatc tacagaatgt   1099500
     tagaaaatat ttgcaaactc tccatccaac aagggactaa tatttagaat acattggaaa   1099560
     ctcaaacaac taaacagaaa agaaaacaaa tttttttatt aaaaataggc aaatgaaaaa   1099620
     atatttaaaa gtttttaaaa ccaaaaaagg aactgaatag acgtctctaa aaggaagata   1099680
     tacgaatggc caacagacat atgagaaaat gctcaacatt actaatcata aggaaaatgc   1099740
     aaattgaaac cacaataaga tataatctca ccccagttaa aatggctatt attaaaaaag   1099800
     gcaaaaaaca attgctgtca aagattcaga gaaaggggag ctttaatgca ttattggttg   1099860
     aaatcttgtc tttggaatta gcacagccat tgaaaaacaa taaagagctt cctcaaaaaa   1099920
     tggaaatgag tactgcccca tgatcccacc atcctctacc gggtatttat ccaaaggaaa   1099980
     tctatgtcaa agagacatct acactacatg ttcattgcag cactattcac aatgccagtg   1100040
     ttggaatcaa cctacgtgtc catcaacaga tgaatgcata aagaaaatgt agtgtttata   1100100
     cacaaacaca cacagagaga gagagagaga ggaattctat tctgccatga aaaagaatga   1100160
     aatcttgtca tctgaggtaa catggatgag cttggaggac atgttaagtg aaataaagct   1100220
     agacacagac agccaaatat cacatgttct cactcatata tggaagccaa ataagttgat   1100280
     ctcatagaaa tagggagtag aatagtggta accagaggct gggaatggga ggggatagga   1100340
     tagctagaag tcgattaata aataaaaaat tacagtcagg taggaggaat aggccccggt   1100400
     gttctctaac accatagggt gactataact aacaacaatt tattgtatat tttcatacgg   1100460
     ctagaagagc aaattttgat tgttcccagc acaaagaagt gataaatgtt tgaggtgatg   1100520
     gatatgctaa ttgttgcagg aagtcaggga ccccgaatgg agggaccagc tggagccatg   1100580
     gcagaggaac ataaattgtg aagatttcat ggacatttat cagttcccaa ataatacttt   1100640
     tataatttct tatgcctgtc tttactttaa tctcataatc ctgttatctt cataagctga   1100700
     ggatgtacgt cacctcagga ccactgggat aattgtgtta actgtacaaa ttgattgtaa   1100760
     aacatatgtg tttgaacaat atgaaatcag tgcaccttga aaaagacaga ataacagcga   1100820
     tttttaggga ataagggaag acaaccataa ggtctgactg cctgaggggt tgggcaaaaa   1100880
     gagccatatt tttcttcttg cagaaagcct ataaatgaac gtgcaagtag ggaagatatc   1100940
     gctaaattat tttcctagca aggaatatta atactaatac cctgggaaag gaatgcatcc   1101000
     ctgggggagg tctataaacg gccgctctgg gaatgtctgt cttatgtggt tgagataagg   1101060
     actgagataa ggactgagat acgccctggt ctcctgcagt accctcagct tattaggggg   1101120
     gtgaaaaact ccaccctggt aaatttgtgg tcacactggt tctctgctct caaactctgt   1101180
     tttctgttgt ttaagatgtt tatcaagata atatatgcac tgctgaacat agacccttat   1101240
     cagtagttct gtttttgccc tttgccttgt gatctttgtt ggacccttat cagtggttct   1101300
     gcttttgccc tttgtcctgt tccctcagaa gcatgtgatc tttgttagac ccttattagt   1101360
     agttctgctt tttgcctttg aagcatgtga tctttgtacc cactccctgt gcttacaccc   1101420
     cctccctttt tgaaaccctt aacaaaaaac ctgctggttt gaggctcagg cggtcatcac   1101480
     agtcctactg atatgtgatg tcacccccgg ctgcccagct gtaaaatcct ctctttatac   1101540
     tgtctctctt tatttctcag ctggccaaca attatggaaa acagaaagaa cctacattga   1101600
     aatattgggg gcaggttccc ccaatactaa ttatcctgat ttgatcatca cccattgtat   1101660
     atatgtatcc aaatatcaca atgtacccca aaatatatac aattattatg tgtcaattaa   1101720
     aaacaatcat aaaactttta aacagctaaa ataaaagtat attgttttct tcaaaaaaaa   1101780
     tctaatgcag ttttccctac taggttttgg gcttgcttag gacccatggc tcctctctcc   1101840
     cttccaatgt atcccttttg gaataggaat gtccatccta tgcctgcccc atcattgtac   1101900
     tttggaagca gataacttct tgtcaagtta caaaggtcca cagatggaga ggaatttcac   1101960
     cccagaatga atcaccctgc atttctccca cacttgatgc aggtgatatg tcgctgagat   1102020
     tgtggactaa gagttggtgc tggaaggggt tagccatcgt ggagatgttg ctatgggatg   1102080
     cagggatttt gcctgtgaga aggacatgat tatgggggga gcggagggca aactgtcatg   1102140
     ggttaaaatg tgtcccctat aaattcatgt gttgaagtcc taacccccag gaccacagaa   1102200
     tgtgaccttg tttggaaaca gtctttgcag ctgcaatcaa gttcggatga ggtcaccctg   1102260
     gagtagggca agcctctgat ccaatatgac tgctgtcctc atgaaaaggg ggaatctggg   1102320
     cacagacgca cgtgtggaga acgccctgtg aagatggtgc tgcttccaca agccaagagc   1102380
     agcagagacg gccggcaaag cccagcagca aggagagagc ctggaacaga gtctccatga   1102440
     cacagaggag ccagccccac cgagacctcc atcccagatg accggcctcc agaaccagga   1102500
     cggaataaac gtctgttgtt taagccacgc agtctggggt gcagtgttgc cagggccgca   1102560
     cttaacggat acgagtgttg tcctgagctg ccagccccac agactgcaca aggcctccct   1102620
     gccccagcca agtgcagtct ccccagcccc ctgggtgtgc catgggcagt gtggggccca   1102680
     tcactccgtc ctcccccagg ctgggaggtt gagcccatta tgagctccat ggggtgaagc   1102740
     tggagcgaga ggctgggagc cgactgggag cccgcggctg gaggatggat ttccccaggg   1102800
     acccacacgt gcacctccac ctgtctcctg gacgttctct ctgagggcag ggctggtgcc   1102860
     agctcaggga tccagcaggg acagaagggc gggccgggtc cttgtggaga gcacatttag   1102920
     tgggagggac atgatttccc ctcacaagtg tccattcttc ctgttccttg ctggacgctt   1102980
     cctcttccat tctggacact tcctgtgcga cacctcctcg ggctttcccg aggccctctg   1103040
     gcctcattcc gttccctgct acctcccact tccacgtacg tccttgccca gctcttccct   1103100
     ctatccagag cttctgcctg gcaaggtccc tgctgagatc agtccaggct cccccagcac   1103160
     aggtaggagc cttgcacatg cccttggacc tccccaccct gcatgatgcc agcatcccca   1103220
     ggccccaggg aggccccatt tctctctctg ctggtagtcc agtggccctg gagtcccact   1103280
     gcaggtgggg tgtgcccctg aactctgagg aagctaagta ccctgccctc agacaggcta   1103340
     tcccccctgc tcagccccag ggccctgccc cctacccctt cccctcacct gcaccacagg   1103400
     ctctggccaa ctctgcccag gccctgaatg ggcccctctg gctcccctct gctgctacac   1103460
     tgccctgcac cacctccact cagcctcagt gtgttcatcc gcctgtccca cgtcccctcg   1103520
     gcccccagga gcacagctgg tggccctggc tcctcgcagc ccatcttgtt ccttctggag   1103580
     caccagcctc agaggccttc ctgtgcaggg tccactcggc cagccctggg accctcctgg   1103640
     tctcaagcac acacattctc cctgcagcca gacctgcccc tgcctgtgag ctcagacctg   1103700
     agccttggaa cgccttccct tctccatccc agctcgcctt tgccagctgc tcagcaggat   1103760
     gaactcacac tcccctccct gcaccatgag tgagagtcag ctggagagat gcccaggcaa   1103820
     aagcagccac cagggcccag tgggggccag aagcttcaga tgagaggccc aggtattgag   1103880
     aggctgagac cacgggcaga atggtcataa tcgttgccag tctcagtcca gccccaggga   1103940
     ctcagagaca gagaaaagag cagcacacaa ggtccgggct ccccaccttc tcccgtgagt   1104000
     acgggggagt atgggggcag ccaccacccc catccccaca cacccatgag gcagcctcag   1104060
     ctgtgtctgg actccccctc gccctctgac acagaaacca ccagaagaaa agggaacttc   1104120
     aggaagtaag tggtgccgcc ggtttcaatc ctgttcttag tgtttgcagc gtggagttca   1104180
     cacccctggg gacctggggg ccgagctgtg atttcctagg aagacaagtg gcagctgaca   1104240
     gcgtgggcaa ggctgcccac atgtacctcg ccacaacagg aagggctgag acccccacct   1104300
     cggtgagtgg ggtcagcaca gggcaggagc acaggctcag gagaaggaca gagcctgggc   1104360
     gcagccatcg gcgcttctgg acctgagctg ctgaacaggc tgcaagaggc tggggagagg   1104420
     ctggggcgag gccagcccca catggaagcc taagcggagc cagcacaggg gaggtgggca   1104480
     gccttcaggc accgatgccc acccagtgcg agacgacagg gaccgtgggc aggggcttcc   1104540
     aagccaacag ggcaggacac accagaggct gactgaggcc tccaggacga ccgggctggg   1104600
     agtgtgagga acatgacggg atggggcaga gccagccatg gggtgatgcc aggatgggca   1104660
     tgaccgacct gagctcagga ggcagcagag agagggagga ggagaggccc caggtgaacc   1104720
     gaggggcttg tccaggccgg cagcatcacc ggagcccagg gcagggtcag cagagctggc   1104780
     cgtagggccc tcctctcagc caggaccaag gacagcaggt gagctgggag cagagcaggg   1104840
     agggtgagtg tggcagcagg acaggagggt ggaagccaag gagcccagag gcagaggcag   1104900
     ggacagggga ggcacagggg ctgggctcag agccagctga tggggttggg gcacctgctg   1104960
     gccgggagca gggctgtggt cagcagcgga gaggagggga gagctgtgct gagtgcacgg   1105020
     gcgggaggag ggaagagtcc agggaggccc agaaaggccc agagtgcagc aggcctgggg   1105080
     cgaggggagg ggctgaggct ccgtgcgttc agggaactga cccagcagag cagaggccac   1105140
     tgaggagctg aggttctgga gaggcttcca gagcaggagc agtgcaggga cgggaggatc   1105200
     cgggagctca tccaggaggg gcacatgggc aagggcaagg ggctctgttg gggagacctg   1105260
     actggacact ggggctgctc cacagcatag ggaacacgcc aagtgctgca aaaacaaaaa   1105320
     tgaggcaaga aaaacagcct aaacctggac agagggtgcc aggacaggca ggggggcaac   1105380
     agtggaccga gtgacgttgc tgccccaggt tgagggaggg cagagtgagc agggggcagg   1105440
     cattggagtt caggggacca ggaccaagca gccacaggtg cacagggcag gtgggaggca   1105500
     ggaagaacag ggggcagctc ctggagctca ggggaccacg ggagagcatc tgaaggtgaa   1105560
     caggagccgg tggaggggca ggatgagcag gaggaagacc ctggagctca ggggaccagg   1105620
     gcagagcagc cacaggtgag gaggcagcca caggtgcgca gggcaggtgg gaggcaggac   1105680
     cagcaggggg cagcccctgg agctcataga gccagggcag agcagttgca ggtgagcagg   1105740
     gctcagtgga agatgggcaa gcagggggca gctcctggag ctcagggcac cagggcagtg   1105800
     tagccacagg tgagcagggg ctggtgggag gcaggacaag caggcggcag ctcccggagc   1105860
     tcagggaacc agggcagtgt agctgcaggt gagcaggggc tggtggaagg caggacaagc   1105920
     aggtggcagc tcctggagct cagggaacca gggcagtgta gctgcaggtg agcaggggct   1105980
     ggtgggaggc aggacgagca gggggcagcc cctggagctc aggggaccag gggagggcat   1106040
     cagaaggtga gcaggggctg gtgggaggca ggatgagtaa ggggcagccc ctttagctca   1106100
     ggggaccagg gcagagcagc ctcaggtgag caggggcagg tgaggggcag gacgagcagg   1106160
     gggcagtcct ggagctcagg ggatcagggc agagcatcag aaggtgagca tggctggtgg   1106220
     gaggtgggcg agcagggggc agcccctgga gctcagggga ccagggcaga gcagtcgcag   1106280
     gtgagcacgg gttggtggga ggtagaacga gtagggggca gctcctggag ctcagggaac   1106340
     aggggagaac atcagaagat gagaagggcc ggtgggaggt tgcagagcag ggggcagctt   1106400
     ctgcagctct gccagccagg gcagagcagc tgcaggtaag cagggctggt gggaagcagg   1106460
     ggtagcaggt gggcagcccc tggagctcag agagccgggg aagatcatcg gtaggtgagc   1106520
     aggggctggt ggaaagcagg aggagcaagg ggcagctcat ggagctcaga ggaccaggga   1106580
     agagcagcca caggtgaaca ggggcaggtg ggcggcagaa tgagcagggg cagctcctgg   1106640
     agctcagggg acaagggcag agcagccata ggcaaacagg agcagggtca ggggacagga   1106700
     ggagcagggg gcagctcttg gagctcaggg gaccagggca gagccgctgc aggtgagcag   1106760
     gggcaggtgg ggggcaggag gagcaggggg catctcctgg agctcagagc accagggcag   1106820
     agcagccaca ggtgagcagg gacggtggga ggcagcacgc agctcctaga ctttggcagg   1106880
     agctgggtag ttgccggcag cagacagctg agggctggtg aaagtgcagt gcagcctcct   1106940
     ggtgccggga agggagtgtg agtccatccc actgagcagt tggcaagggc gagctgggat   1107000
     ggagaaggga aggcgttcca gggctcgggg ctgagctctc aggcaggggc aggtgtggct   1107060
     gcagggggaa cgtgtgcttg agaccaggag ggtcccaggg ctggccccag cggaccctgg   1107120
     gcaggaaggc ctctgaggct ggtgccccag aaggagcaag atgggctgcc aggagccagg   1107180
     accatcagca caatgaagct gagtggaggt ggtgcagggc agtgtagcag cagagggcag   1107240
     ccagaggggc ccattcaggg cctgggcaga gtcagccaga gcctgtggtg caggtgaggg   1107300
     gaaggggtgg tgagcggggc cctggggctg agcagagggg atggcctggc tgagggcagg   1107360
     gcgcttagct tcctcagagg tcaggggcac accccacctg cagtgggact ccagggccac   1107420
     tgggccagca gcagagagaa atggggcctc cctgtggcct gggggtcctg gcaccacgca   1107480
     gggtggggag ggccaagggc aggtgcaagg ctcctacctg tgctgggggg cctgggttga   1107540
     gcccagcagg gaccttgccg ggggaagctc tggagagagg gaggaggtgg gctggtggct   1107600
     gagaaggcca ggccagggct gggagggtga cggtgtggtg actgagcctc cagaagtaat   1107660
     gcaggacact gggaggcagg gggcatccag gcactcaggg ccctgacctg ggctgctgca   1107720
     cactggggct aaggggaaag gaggggagag gctgaggagg aggctcccgg ggcgatattc   1107780
     caaggcaggg ggttccgggg ccctggggct gaagggcgcc gaccctatgc agtgtctggc   1107840
     ccctctgctg cacagaagaa aagggccttg gagggcagag ggcaggctat gaccagggcc   1107900
     ctgggcaagt caggcccact cactagcgga gggccacgct ggggcggcag ggtcaggagc   1107960
     ttcaggggac tcaggggacc cacgagaagc catctgagaa cagtgtccac tggtcaagcc   1108020
     aggcacccat aaaaggctgg agtggggcca atgggcatga gccgtccctg aggtggcacc   1108080
     gatggccaga gctgaggcca agctagaggc cctggactgt gctgactccc ggcagacaca   1108140
     gagcgctgac ctggctgccg agccccgcct cctaggctgc aggggtgcct gcagaagggc   1108200
     accacagggc caccggtcct gcaagctttc tggggcgggc cgggcctgac cttggctttg   1108260
     gggcagggag ggggctaagg tgaggcaggt ggcgccagcc aggcgcacac ccaatgcccg   1108320
     tgagcccaga cactggaccc tgcctggacc ctcgtggata gacaagaacc gaggggcctc   1108380
     tgcgccctgg gcccagctct gtcccacacc gcagtcacat ggcgccatct ctcttgcagc   1108440
     ttccaccaag ggcccatcgg tcttccccct ggcgccctgc tccaggagca cctctggggg   1108500
     cacagcggcc ctgggctgcc tggtcaagga ctacttccca gaaccggtga cggtgtcgtg   1108560
     gaactcaggc gccctgacca gcggcgtgca caccttcccg gctgtcctac agtcctcagg   1108620
     actctactcc ctcagcagcg tggtgaccgt gccctccagc agcttgggca cccagaccta   1108680
     cacctgcaac gtgaatcaca agcccagcaa caccaaggtg gacaagagag ttggtgagag   1108740
     gccagcgcag ggagggaggg tgtctgctgg aagccaggct cagccctcct gcctggacgc   1108800
     atcccggctg tgcagtccca gcccagggca ccaaggcagg ccccgtctga ctcctcaccc   1108860
     ggaggcctct gcccgcccca ctcatgctca gggagagggt cttctggctt tttccaccag   1108920
     gctccgggca ggcacaggct ggatgcccct accccaggcc cttcacacac aggggcaggt   1108980
     gctgcgctca gagctgccaa gagccatatc caggaggacc ctgcccctga cctaagccca   1109040
     ccccaaaggc caaactctct actcactcag ctcagatacc ttctctcttc ccagatctga   1109100
     gtaactccca atcttctctc tgcagagctc aaaaccccac ttggtgacac aactcacaca   1109160
     tgcccacggt gcccaggtaa gccagcccag gcctcgccct ccagctcaag gcgggacaag   1109220
     agccctagag tggcctgagt ccagggacag gccccagcag ggtgctgacg catccacctc   1109280
     catcccagat ccccgtaact cccaatcttc tctctgcaga gcccaaatct tgtgacacac   1109340
     ctcccccgtg cccacggtgc ccaggtaagc cagcccaggc ctcgccctcc agctcaaggc   1109400
     aggacaagag ccctagagtg gcctgagtcc agggacaggc cccagcaggg tgctgacgcg   1109460
     tccacctcca tcccagatcc ccgtaactcc caatcttctc tctgcagagc ccaaatcttg   1109520
     tgacacacct cccccatgcc cacggtgccc aggtaagcca gcccaggcct cgccctccag   1109580
     ctcaaggcgg gacaagagcc ctagagtggc ctgagtccag ggacaggccc cagcagggtg   1109640
     ctgacgcatc cacctccatc ccagatcccc gtaactccca atcttctctc tgcagagccc   1109700
     aaatcttgtg acacacctcc cccgtgccca aggtgcccag gtaagccagc ccaggcctcg   1109760
     ccctccagct caaggcagga caggtgccct agagtggcct gcatccaggg acaggtccca   1109820
     gtcgggtgct gacacatctg cctccatctc ttcctcagca cctgaactcc tgggaggacc   1109880
     gtcagtcttc ctcttccccc caaaacccaa ggataccctt atgatttccc ggacccctga   1109940
     ggtcacgtgc gtggtggtgg acgtgagcca cgaagacccc gaggtccagt tcaagtggta   1110000
     cgtggacggc gtggaggtgc ataatgccaa gacaaagccg cgggaggagc agtacaacag   1110060
     cacgttccgt gtggtcagcg tcctcaccgt cctgcaccag gactggctga acggcaagga   1110120
     gtacaagtgc aaggtctcca acaaagccct cccagccccc atcgagaaaa ccatctccaa   1110180
     aaccaaaggt gggacccgcg gggtatgagg gccacatgga cagaggccag cttgacccac   1110240
     cctctgccct gggagtgacc gctgtgccaa cctctgtccc tacaggacag ccccgagaac   1110300
     cacaggtgta caccctgccc ccatcccggg aggagatgac caagaaccag gtcagcctga   1110360
     cctgcctggt caaaggcttc taccccagcg acatcgccgt ggagtgggag agcagcgggc   1110420
     agccggagaa caactacaac accacgcctc ccatgctgga ctccgacggc tccttcttcc   1110480
     tctacagcaa gctcaccgtg gacaagagca ggtggcagca ggggaacatc ttctcatgct   1110540
     ccgtgatgca tgaggctctg cacaaccgct tcacgcagaa gagcctctcc ctgtctccgg   1110600
     gtaaatgagt gcgacggccg gcaagccccc gctccccggg ctctcggggt cgcgcgagga   1110660
     tgcttggcac gtaccccgtg tacatacttc ccgggcaccc agcatggaaa taaagcaccc   1110720
     agcgctgccc tgggcccctg cgagactgtg atggttcttt ccacgggtca ggccgagtct   1110780
     gaggcctgag tggcatgagg gaggcagagc gggtcccact gtccccacac tggcccaggc   1110840
     tgtgcaggtg tgcctgggcc gcctagggtg gggctcagcc aggggctgcc ctcggcaggg   1110900
     tgggggattt gccagcgtgg ccctccctcc agcagcagct gccctgggct gggccacggg   1110960
     aagccctagg agcccctggg gacagacaca cagcccctgc ctctgtagga gactgtcctg   1111020
     tcctgtgagc gccctgtcct ccgacccgca tgcccactcg ggggcatgcc tagtccatgt   1111080
     gcgtagggac aggccctccc tcacccatct acccccacgg cactaacccc tggcagccct   1111140
     gcccagcctc gcacccgcat ggggacacaa ccgactccgg ggacatgcac tctcgggccc   1111200
     tgtggagaga ctggtccaga tgcccacaca cacactcagc ccagacccgt tcaacaaacc   1111260
     ccgcactgag gttggccggc cacacggcca ccacacacac acgtgcacgc ctcacacacg   1111320
     gagcctcacc cgggcgaacc gcacagcacc cagaccagag caaggtcctc gcacacgtga   1111380
     acactcctcg gacacaggcc cccacgagcc ccacgcggca cctcaaggcc cacgagccgc   1111440
     tcggcagctt ctccacatgc tgaccagctc agacaaaccc agccctcctc tcacaaggtg   1111500
     cccctgcagc cgccacacac acacaggccc ccacacacag gggaacacac gccacgtcgc   1111560
     gtccctggca ctggcccact tcccaataca gcccttccct gcagctgggg tcacatgagg   1111620
     tgtgggcttc accatcctcc tgccctctgg gcctcaggga gggacacggg agacggggag   1111680
     tgggtcctgc tgagggccag gtcgctatct agggccgggt gtgtggctga gtcccggggc   1111740
     caaagctggt gcccagggcg ggcagctgtg gggagctgac ctcaggacac tgttggccca   1111800
     tcccggccgg gccctacatc ctgggtcctg ccacagaggg aatcaccccc agaggcccga   1111860
     gcccagcagg acacagcact gaccaccctc ttcctgtcca gagctgcaac tggaggagag   1111920
     ctgtgcggag gcgcaggacg gggagctgga cgggctgtgg acgaccatca ccatcttcat   1111980
     cacactcttc ctgttaagcg tgtgctacag tgccaccgtc accttcttca aggtcggccg   1112040
     cacgttgtcc ccagctgtcc ttgacattgt cctccatgct gtcacacact gtccctgaca   1112100
     ctgtccccag gctgtcccca cctgtccctg acactgtccc ccacgctctc acaaactgtc   1112160
     cctcacactg tcccccatgc tgtcacaaac tgtcactgac actgtccccc atgctatccc   1112220
     cacctgtccc tgacactgtc cctgacactg tctctcatgc tgtccccact catctgcgac   1112280
     actgtacccc acgctgtccc cacttgtcct caacaatgtc ccccatgctg tccccacctg   1112340
     tccctgatgc tgtcccccac actgtcccaa tctgtcccca ccactctccc ccacgctgtc   1112400
     cccacctgtc cctgacactg tcccccatgc catccccatc tgtcccgaca atgtccccag   1112460
     ggtgtcccca gctgtccctg atgctgtccc ccacactgtc cccacctctc cctgacgctg   1112520
     tcccccacgt ggtccccact tgtccctgat gctgtccccc acactgtccc cacctgtccc   1112580
     tgacactgtc ccccatgcca tccccatctg tcccgacaat gtccctatgg tgtccccagc   1112640
     tgtccctgat gctgtccccc acactgtccc cacctgtccc tgacgctgtc ccccacactg   1112700
     tccccacctc cccctgacac tgtcccccac actgtcccca cctctcccta acactgtccc   1112760
     acacactgtc ccctcctgtc cccaacactt tcccccatgc tgtccccacc agtccccaac   1112820
     actgtacacc atgcttttcc cacctgtccc caacactgtc ccccatgctg tcccctcctg   1112880
     tccccaacaa tgtcccccat gctgtttcct cctgtcccca acactgtccg ccactctgtt   1112940
     tcctcctttc cctgacactg tcccccactc tgtccccacc tgtagccaac actatcccct   1113000
     acgctgtctc cacctgtccc tgatgctgtc ccccacactg tccccactcc tccctgacac   1113060
     tgtcccctat gctgtcccca ccggttccta acactgtccc ccacactgtc cctacctgtc   1113120
     cccgacactt tctcccatgc tgttcccacg tgtctccaac actgtccccc acacagtctc   1113180
     cacctgtccc tgacactgtc ccccatgctg tcctcaccca tctctgacac tgtacacata   1113240
     ctgtccccac ctgtccctga tgctgtcctc catgatgtcc ccacctctcc ctgacactgt   1113300
     cacccatgct gtccccacct gcccctgaca ctctcctcca cgctgttctc acctgtcccc   1113360
     aacactctcc cccacactgt ctccacctgt ccctgacact gtcctccacg ctgtccccac   1113420
     ctatccctga cactgtcccc catgctgtcc tcacctgtcc ccaacactct cctccacact   1113480
     gtcctcacct gtccccaaca ctctcccccc acactgtctc aacctgtccc tgacactgtc   1113540
     ccccatgctg tcctcacctg tccctgacac tgtcccccat gctgtcctca cctgtctctg   1113600
     acactgtccc ccgtgctgtc cccacctgac actatcttct gtgctgtcca catgctgttg   1113660
     ctgccctggc tctgctctcc atgtccaggc ctcagagcag gcagtggtga ggccctggca   1113720
     catgggtggc atgaggggcc ggataggcct caggggcagg gctgtggcct gggtggcctg   1113780
     aggggtgagc aggcctcggg ggcagggctg tggcctcgct cacccctgtg ctgtgccttg   1113840
     cctacaggtg aagtggatct tctcctcggt ggtggacctg aagcagacca tcatccccga   1113900
     ctataggaac atgattgggc agggggccta gggccaccct ctgcggggtg tccagggcca   1113960
     cccagatccc acacacgagc cgtgggccat gctcagccac cacccaggcc acaactgccc   1114020
     ccgacctcac cgccctcaac cccatggctc tctgtctttg cagtcgccct ctgagccctg   1114080
     acacgccccc cttccagacc ctgtgcatag caggtctacc ccagacctcc gctgcttggt   1114140
     gcatgcaggg agctggggac caggtgtccc ctcagcagga tgtccctgcc ctccagaccg   1114200
     ccagatgctc acacaaaagg aggcagtgac cagcatccga ggcccccacc caggcaggag   1114260
     ctggccctgg agccaacccc gtccacgcca gcctcctgaa cacaggcgtg gtttccagat   1114320
     ggtgagtggg agcatcagcc gccaaggtag ggaagccaca gcaccatcag gccctgttgg   1114380
     ggaggcttcc gagagctgcg aaggctcact cagacggcct tcctcccagc ccgcagccag   1114440
     ccagcctcca ttccgggcac tcccgtgaac tcctgacatg aggaatgagg ttgttctgat   1114500
     ttcaagcaaa gaacgctgct ctctggctcc tgggaacagt ctcggtgcca gcaccacccc   1114560
     ttggctgcct gcctacactg ctggattctc gggtggaact ggacccgcag ggacagccag   1114620
     ccccagagtc cgcactgggg agagaagggg ccaggcccag gacactgcca cctcccaccc   1114680
     actccagtcc accgagatca ctcagagaag agcctgggcc atgtggccac tgcaggagcc   1114740
     ccacagtgca agagtgagga tagcccaagg aagggctggg catctgccca gacaggcctc   1114800
     ccagagaagg ctggtgacca ggtcccaggc gggcaagact cagccttggt ggggcctgag   1114860
     gacagaggag gcccaggagc atcggggaga gaggtggagg gacaccggga gagccaggag   1114920
     cgtggacaca gccagaactc atcacagagg ctggcgtcca gccccgggtc acgtgcagca   1114980
     ggaacaagca gccactctgg gggcaccagg tggagaggca agatgccaaa gagggtgccc   1115040
     gtgttcttgc gaaagcgggg ctgctggcca cgagtgctgg acagaggccc ccacgctctg   1115100
     ctgcccccat cacgccgttc cgtgactgtc acgcagaatc cgcagacagg aagggaggct   1115160
     cgagcgggac tgcggccagc gcctgcctcg gccgtcaggg aggactcccg ggctcactcg   1115220
     aaggaggtgc caccatttca gctttggtag cttttcttct tcttttaaat tttctaaagc   1115280
     tcattaattg tctttgatgt ttcttttgtg atgacaataa aatatccttt ttaagtcttg   1115340
     tacttcgtga tgggagccgc cttcctgtgt ccacgcgcct cctgcccccg gtgggaagca   1115400
     cattcaggag gaggctggtc cagctgcacc tcgggggctc cctgcatacg ccccccgcct   1115460
     cctgcagcca cacgcattgc ccgagcgacc ctccctggcc cctgtcgcta catggacccc   1115520
     cggggcttct cctcttttct acatggatgc agtttctcct cctgctgggc acggtgctgc   1115580
     ctgccctggt cactctgcgg gggacagggc ctccagggaa agctgggtcg aggctgggag   1115640
     ctggctcagg ctggccaggc agagccacag ggagggcctt ccagaaccaa ccatggtccg   1115700
     aagcgagagg tgggtgtcag atctgtgtgc gtcagctcag gaccacagcg gggcggctcc   1115760
     cacagcagac atggatcctc ccaggcctag agaccaggaa tctgagatca ggatgcaggc   1115820
     agggctggtt tctctcaagc cctctctcct tggcttgtag acaccgtctc ctccctggtc   1115880
     ctcacgtggc catccctctg tgtgcccgtg tcctaagctt ctcttcttat aagaacacac   1115940
     atcagattag attagtgacc ccctatgaac ttaatgacct ctgtaaagac cccatctcca   1116000
     aatagtcaca ttgtgaggcc agggattaag acttgaatat atgaatttgt aggggccacg   1116060
     atttaaccca tcacagtcca gactctggcc cccaaaattc atgttcttct cacatgcaga   1116120
     atacattcat cctgtctcag catccccctg ggcactaggt catgtagcaa ggacggattt   1116180
     tcaacagaaa taactattgc aacagaagaa agagtccggc atgacctgga ctcaccttca   1116240
     tctgtgcaga ggccacagcc ttgtaaaggg aggtggtagg gggagcaggg tgggtgctcg   1116300
     gggctcagtc gtcggggaag ggaaaagttg cccagcgctg gtcagcgtcc ccgggatggg   1116360
     acccgctgtg tccgtgccgg ccactgttga ggtcaggatt ctgtcctccc agagcctgga   1116420
     gacacaggcc ccatccttcc caatggggac acttcaggga gcggctctca ggtcccgaga   1116480
     aagaccctcc tgggtcacag gaaatgcaca gacatcggga acagacagaa ggacgtgtgg   1116540
     ttgcggccct ctcagcagat accctgagaa agggaggtcg gggttggtcc aaacggtgag   1116600
     ttctggtgca cggagctttc tcaggcaggt gttgacgggg caggggtcgg cctaggggta   1116660
     cggccagaag ctgttagaaa ctgttagtgt ctgctcaagt ctttacaagc caaggttgag   1116720
     gccgagtgga gaggctccga ggagcctggc tggaactcag tcaaggacag ggtcttgtta   1116780
     ctgcagtggc tgcggtggct gcggtggctg gaaaatgccg tcggagttgc ctgtggcagg   1116840
     agagagacca tctcacccag gaaggaggag tggttggatt cgtttgtgtg gcatcgagca   1116900
     gctggagctt caccaaacac agagttgggg actaaatccc cagactccag gccctgccat   1116960
     gccgtgggaa ggctcgccac tggagggtgg gctccagggg gcctggcctg aactgggtgc   1117020
     tgaagcccag ccctttaact ctcaggacac gctgctgcag ccccgcgggg ggtgagggag   1117080
     acagcacctg gggtgcaggg cgggcagctg ctgcatcacc ggctctatcc caagcccaag   1117140
     gatggcgtcc cagagatgca ggagagcttt gtccagagaa ggtgccagcc ctcagggacc   1117200
     ctgctggaga gatctccacc ctctgccctt caaggggccc tacgggcttc cgggtgccct   1117260
     ggtggggtgg gctccagtcc actgtctgag gatggacggc ctggccagga taaggaaagg   1117320
     aaacccagga cggtgccggg ctccgggtca ttccgtgcac tgagcaggct gagttgggaa   1117380
     gaagcagatg cttcctgcag ctcctgcccc tgcagggcct ggcgcctgga ccaggttccc   1117440
     ctggggaaat tgggcccctc cctgagccac ccggggccca ccgccccctt tccacctggg   1117500
     accgagcatc ctccagaggg tcagccctcc tgagggaaca ccatgcccag ccccaggacc   1117560
     ctccctcaac tctccagcaa ggctgcactg tgggcggccc ctgcacatac cccagcagtc   1117620
     catgctgtga tgtaacatga catggtgtga cgtggtgtga tgtctctggt gtgacgtgga   1117680
     tgtcatgtgg catgatgcga caggacatgg tgtgatgttg tatgatgtgg tatgacggtg   1117740
     tgatatccga ggggtgatgt gatgtggtgt gacatggtgt aatgtccctg gtgtgaggtg   1117800
     gcgttggaca tgctgggatg tggtgtgaca cagtgtgaca tccctggtgt gatgtggtgt   1117860
     gatgaagcgt aacaggctgt gacatagtgt gacgtggtgt gatataatgt gatgtggtgt   1117920
     gaggtggcgt gacatggtgg gatgtggtgt gacatggtat aatgtccctg gtgtgaggtg   1117980
     gtgtgatgtg gtatgatggg gtgtggcagg gtacgacatc cctagtgtga tgataagttt   1118040
     catggtatga tgtggcgtga catccctggc gtgacataat gagatgtggt gtgacatggt   1118100
     gtggcattcc tggtgtgact cctgtgttct gacatttgtg gccaccccag gatacagagg   1118160
     tccctgtagc caagggaagg gggagaatag aaccatctga gcatgttgac ctggaggaat   1118220
     tggtggccct tgagtccacg aagcccaccc tgccaggtgc ccctgcccca cgtgacccaa   1118280
     gtgggcttgc agagcagcaa gcaggactct ggttagactg caagaaggac ctgccaccac   1118340
     gtggccttgt gaggagacac agagcaaggc agtgacctca gcgtccaccc agcacagggt   1118400
     gctgctgaag ctcctcctgt cctctcagca gcggtctcag agcaaggccc aaggcaggct   1118460
     gaagagaggg gcagagggag gatgctgggg aggcaggggt gaggggagtg agagcccagg   1118520
     tttcagctga gcccctgcac agggaaggag gctagctgaa cacccatctc cccacacact   1118580
     cccaaccctg cctctgcccg aacacctccc agaaggcacc tcaaactctg ctacccacac   1118640
     tcagcaaggg gtatggtgtc cccaccgagt ccagccagtg aggcccggca cagccatgcc   1118700
     tgtgccgacc actcccatgg ccaagctccc tgctaacatg gtaggacaga gccaggcctg   1118760
     gaggagacag aacatcagtc ccatggggaa gctccctgct cacatggcag ggccgggcct   1118820
     ggaggagaca gaacgccccg tctggtatgg tactcaggct gcacatgcct gccacgcacg   1118880
     ggggccatgt ggcacacacg ggtactcaca cggtcctcac acagggacac acgcacttgc   1118940
     aaccccagca cccacccagt gcactcatgc acgggctccc caggtgggtc acagtctcac   1119000
     gccttgagct actccatgtg ccaggccctt cacccacatt cccgtccccg gtgccagctg   1119060
     ttgagccaca ccgtcttctc tgtggatccc tcccagcccg ctcagcacaa tggacatgct   1119120
     ctctccagtc cagcaactgc actggcctct tcccctcaga atccacgtct ggccatctcc   1119180
     caggaaaccc ggaccaccac gggcagggac caccttctca ctgtccatgt ggaccacccc   1119240
     acacctgacc ccaggcagag tcacactacg acctgcttca atattgaaaa ggggaaaagc   1119300
     tggaggaggg gaaagatgaa agagaaaaaa gcaagagggg agggtcacat tcttgtgagg   1119360
     ctttgattag agttcactga gcccccacgt tgcatgaaaa ggaggggtgg agggagcaat   1119420
     tacgcattcg ccttgtgctc agtaaatctg cactttataa gcaaataaac agagtagagg   1119480
     aagaagtcaa atatgcattc gtctcagggg taggaggaac gatttcttgt ctcattttgt   1119540
     cccatgtcat gaagaccggc ctgttaattt atattctcag ggtgagggag gccacctggg   1119600
     tagacctggc ctatctgctg ctgctatcac tttggaaaca aaaggaaagg catgactttt   1119660
     tttttttttc atgactcagc ttcccacctc aactgttcat tttggcatag tgagtttgga   1119720
     gtcctgagat tttattttcc tttcacaatg gtcaaggtcg caccctcagt attcaggaga   1119780
     gctggtccaa cccagacccc tgccgtccct ggacgctttt caaatacgtg ctgcagtctc   1119840
     tgacactctc tcaccatggt agaaactgag gtcttggtga gcctgcaagc ccctgctcat   1119900
     ggggacaatg caggctgaaa cccgcatttc tgaccccaaa gctcctgttc ctttcactca   1119960
     cccccacacc agctctttga gtccagagct ttgtccttgc ctgagtccta ccctcaggga   1120020
     caggggccca acccagacac caacacatca taccctgaga gggtgccagg agcccagaga   1120080
     tgtttggaga gcacagaagc cctggaggct ctgtggagat gctgcacatt tctctattca   1120140
     acagatactc accaggtggc cagcggcaga gatgccacat ggcatggagc tcgccttggc   1120200
     caacaggaca ggtgtggggt tgggaggcct tcccagggca ctccttgaag cagagctgtg   1120260
     gggaaaagtt ggggccaacc tcagatctcc tcatcaacac cggttctcct gccctcctgg   1120320
     gccacagaaa actaagctcc ctggatactg cggctgggtg gggccatgag ggagaagaaa   1120380
     tcacaacgag ttaaaagatc attttctaaa actgttatga tcaggactca cataaacata   1120440
     tgacgacaca tttcagagat gctctttatc tcattaatta aggtgtcgta accagttcaa   1120500
     agtggaattc taagtactac acttacataa ttgattcagg aatgctaaaa ggagttcata   1120560
     gatagatgca aaactggcct tttccctgga agatgaggag caattcattg tccttccaaa   1120620
     ggtgagaact tgaatttcta ccaactcaaa gagcttttgc attgctatca attatgtaca   1120680
     acttagagca gtagtcccca acatttttgg caccaggaac cagttccatg gaagacaatt   1120740
     tttccacaga ccaggatcgg gggatggctt ggggacaaag ctgttctacc tcagatcatt   1120800
     aggcattagg atttcacgag gagtgtgcaa cttagatctg ggaatgtgca gctcgcaata   1120860
     gggttcgctc ctatgagaat ctaatgctgc cactgatctg acgggaggtg gagctcaggc   1120920
     aggaatgctc acacacccct cacctcctgc tctgtggccc agttcctaac aggccatgag   1120980
     cagattccag tgcatgaccc aggggttggg gacccctggc ttatagaggt gtaaaatagt   1121040
     tcaaaggaaa taaaagatgc agagctccat agaatgaaat aacttggaag agtgtacaag   1121100
     acgatgcctt gctttccatg gaaggcacct actaatcttt tggtccattt cagttcttct   1121160
     taatgcttcc tataaacata tataactgac tgacacaaac agatccataa tataaagaag   1121220
     accactgtaa accaatgaga aaaaaaatca aataatccaa caaggaatag gcaagagaat   1121280
     tgaacagatg ttttacagaa gatatccaaa tagccactaa acatatgaaa agatgttgaa   1121340
     ccacactagt caacagggaa atgaaaatga aaaaccacat gagagaaagt agttctgatt   1121400
     ccaataattc tggagcagct aatatcagac tagccctttg gcagatggca attataaaca   1121460
     ctagaaacgc tgtaagcaca cacaacaccc acacacacca attgcaggca ctggaacatg   1121520
     accagaagta ggcaaacact agtaaggatt attccgttaa atattcgtct gaagtcacac   1121580
     tccagggcat gtaatgggta cagctagagt tcaagcagga aactgcagcc ctcctggtga   1121640
     ggagtgggat gccgggctac attttcagag cagctggaaa tgaagagaag atgtccataa   1121700
     aggagaaggt caccgaaggg aaaccccaca atctgcaagt aaactccact gaaacctctg   1121760
     gctgatccct taggtgtgca tgggtaggga aaactccaaa gggcccagca gaaagcaaca   1121820
     cgtgtaaggt caagagaact gagattccag ctactgccaa ctgccaggca gacagactgg   1121880
     ggagtttgag tcaactcaag ctaactgctt gctaacattg aaaaaaacaa ttaatgttct   1121940
     gctaaggaag aatgcaaacc ccatagcctg tacacatgtt aacaacatca ggtgcacatc   1122000
     caaaattacc aggtatgcaa agaaatatga aaatgtgatc catagttaaa agaaaaaggg   1122060
     atcaatggag atcaactcca agatgaccta gatgcagata cagttatcag acaaggactt   1122120
     taaagaagtt atgttaaata tgttcaaaga cttaaaggaa aatatggtta tcatgagtga   1122180
     ctagatgagg tatctctata aaaaactgaa aatagttaca agaagcaaat gaaaattcta   1122240
     gaactaagag tatgattcgg aaatgaaaaa catcatttga ccaggcccag tggctcatac   1122300
     ctgtaatcct agcactctgg gaggccaaac atggaaggat cacttgatct caggagtttg   1122360
     agaccaccct gggcaacatg gtgaaacccc atctctattt tattaaagaa gaaaagaaag   1122420
     agaaaaacat tacccaagtt taataacaga ttggacaaga cataagaagg aactgaaaac   1122480
     ttaaagatgc atcaatagaa tatgtcaatt ctttttaatc caatccaaag aagagagaga   1122540
     aaaatactaa aaaatgaaaa gagactcatc ctgctggaca aggtgaagca ttctaaaaca   1122600
     tgtgaaactg gagtccagag ggggatgtaa tacttccatg ggaaaggtca accctaagct   1122660
     atatgcaaat gtacctgcgt aggctggtgt ggtgggctga tggcatctcc ctggccaaat   1122720
     gtatgcccac ctggaacctc agaatgtgac cttatttgga aatggggtct ttacaaatta   1122780
     ggttaaggac ttgagatgat atcatactaa attttggatg gatcccaagt ccaaacactg   1122840
     gtgtcctcat aagaggagag gacaaagaca tacatggagg taagccatat gatgacaaag   1122900
     gagaagatga gcggtgtgtc aggagccacc tgtgtttgtc catttgcatt actgtaaaga   1122960
     aatacctgag gctgagtaac ttatgaagga aagaggttca attggctcac agttctgcag   1123020
     gctgtatagg aagtatggca ccagcatctg cttctggcga ggacctcagg aaacttacag   1123080
     tcatagcaga aggtgaaggg agagcaggta tgccacatgg caagaatggg agcaagaggc   1123140
     tgaggggggg ccccagactt aaacaactag actgtgtgtg agctgactgg ggcagaattc   1123200
     actcatcacc aaggggatga ttctaaatca ttcatgaggg atcttccccc ttgatctaat   1123260
     cacctcctac caggccccac ttccaacact gggaatcata ttacaacatg agatttggag   1123320
     gggacaaata tataaaccat atccttccac ccctggtccc ccaaatctca tgttgcaaaa   1123380
     tacaatcatg ccttctcaac agtcccccaa agtcttacct cattccagca tcaacccaaa   1123440
     tttcccaagt cccaagtctc atctgaagat gagtcccttc cacctatcag cctatgaaat   1123500
     caaacacaaa ttattgactc ccaaaataca atgagagtgc agacattgcg taaacattcc   1123560
     cattccacaa gggagaagtt ggcaaaaaga aaggatctac atgccccata tatgtcagaa   1123620
     atccagcagg gcagtcatta aatcttaaag ctccaaggta atctcctttg acttcatgtc   1123680
     ccatatccag ggcacattgg tgcagggggt gggctcccaa ggccttggaa aactctaccc   1123740
     ttgtggcttt ggaggatgtt gcccctgtgg cttctctcac atgatagagt tgagtgcctg   1123800
     tggcttttcc agattcaggg tgcaaactgc cagtggatcc accattctgg ggtctggagg   1123860
     acagtggccc ccttcccaca gctccactag gcagtgccct ggtggggaca ttatatgggg   1123920
     gttcaacccc acattctctt tggcactgct ctagtagagg ttctctgtga gggctccatg   1123980
     ctggcaggag gcttctgtct gggcacctag gctttctcat acatttctga aatctagagg   1124040
     gaagatgcca agcttccttc actcgtgtat tctgggggcc tacaggctta acaccagatg   1124100
     gaagtcacca aggcttatag tggcttgcac tctccaaaga agcagcctaa gctatacttg   1124160
     gggccctttg agccaaggct ggagccagag gaaccaggat gtggagagca gtgtcccaag   1124220
     gctgcacagg cagcagtggc catggccatg gcccacaaaa ccatcctttc cctcttggcc   1124280
     tctgggcctg tgatgggaaa gaccacctca gagatttcca aaatgccttc aaggccttct   1124340
     tccctttgtt ctggatatga gcacttagct cccttttagt tatgctaatc tctctagcaa   1124400
     ctggctgctc catagcctac ttatatttgt ctcctgaaaa taccttttct tttctaccat   1124460
     atggccaggc catgaatttt ccaaattttt tatgctctgc ttccctttgt tgatgaaaaa   1124520
     agccaaactc tataaaacac ttgacgagat tgattctgag ccacatgtga gagccatgaa   1124580
     ctgtggcaca gcctcaaaag gtcctgagaa catgtgtgca aggtgcttgg gttgcggcct   1124640
     ggttttatgt tttagggaga catgagacat caatcaatac atgtaaagta tacattcgtt   1124700
     tggtttggaa tggcaggaca attcaaagtg gggaattcca aatcataggt ggatttgaag   1124760
     attttctgat tggcaattgg ctgaaaaatt taaattatct aaaaagttga agtcagcaaa   1124820
     aagcaatgct taagataaag caggttgtgg aagccaaggt tcttgttatg tagatgaagc   1124880
     ctccacgttc cagagagaag agatggtcaa tgtctcttat cagaacctaa aaggtgccag   1124940
     actcttggat aaatctctcc tgaatcagga agagacctgg aaagggaaaa agattctcga   1125000
     cagaatatac atttcctcca caagagacgg ctttgcaggg ccattccaaa atgtgtcaga   1125060
     gaaatatatt ctggcataaa atactttaat ttccttccat gcctgccaac tgtcatgtga   1125120
     tgctatacca gagtcaggtt ggaatttgat atcttattgc tacaaagagt ctgctttgtc   1125180
     agtcttaagc tccctgtttt aatgttaaca ccggtcagct gagcctaaac tccaacaggg   1125240
     agagggtata gtgagggagt ccaaaccacc ctccccttcc tgtcatggcc tgaactggtt   1125300
     ttccaggttt ctttggaatc cccttgcatt agcattttat tttttcattt gcacctttta   1125360
     aatatcagtt ccaactttaa gtcattcctt tgctcccata tcttaccata gactgttaga   1125420
     agcaaccagg tcacacctta gaaatgtctg cttagaagta tcttccacca gatgtcctaa   1125480
     gtcatcactc ttaaggtcaa acttccacag atccctaggg catgaataca atgcagccaa   1125540
     gttgtttctt aggatttaac aagcgtgacc tttactccag ttcccaataa gttcctcatt   1125600
     tccatctgag atttcatcag cctggctttc acagtccata tgtctatcag cattttggtg   1125660
     acaaccattt aatcagtctc taagaaattc caaactttcc ctcatcttcc tgtcttcttc   1125720
     tgagtcctcc aaattcttct aacttctctg cctgttacct gattccaaag ctgcttccaa   1125780
     attttcaggt atctttatag caatgttcca ctcctcaata ccaaatttct gtgttagttt   1125840
     gtgttactag aaaaaaaaat acatgaggct agtaatttat aaagaaaaga ggtttaggcc   1125900
     aggcactgtg gctcatacct gtcatcctag cattttggga ggcagaggca ggaggatcac   1125960
     ttgagctcag aagtttgaga ccaacctggg caacatagtg agaacttgtc ttgaaaagac   1126020
     aagaagaagg gaagggtggg gaagagcaga agggcagaag ggcagggcag aagggaaggg   1126080
     cagggaaggc agaagagaag gaaggaaggg aggtttaatt agttcatatt tctgcaggcc   1126140
     gttctggaaa cacggccctg gcatctgctt ctggcgaggc ctcagggaga ttgcaatcac   1126200
     agcagaaggc gaagggggag caggcatgtc acatggcaag agggagcaag agaagaaagt   1126260
     ggcacgtccc agacttttaa acaaccagct gtcacaggga ctaactgagg gagaactcgc   1126320
     tcatcacctg ggagatggtg ctaggccatt catgaggggt ccagccccat gatctaatca   1126380
     cctcccacca ggcccacctc caacactggg aatcacatct caacatgaaa tttggagaga   1126440
     cgcacatcca aactattgca ccacaggaag ctggaagagg tggacggtcc tcccctggag   1126500
     cactcagaga cagccaccaa caccttgatc tcagatttct gactcccaga actctgaaaa   1126560
     aatttttgtt gttttaagcc acatttgtga tattttgtta cggcagccac agcacggata   1126620
     tagctggtaa cagagttctt tagcctccaa aatacaagcc tggggtaaat aactattgaa   1126680
     tttggccaca tggaaatcag gaagaatgta ataagcatca tcgatggacg ggatgacctg   1126740
     ggagatcgtg gcaagtttaa agctaattgg aagttggcaa ccaaaaaatc ctgtcttgca   1126800
     aatggccagg aaattgccaa gaaaaccttt agaaaacatg gttaaagatg gagaaacccg   1126860
     gatgactccc tggtttctga agaggtgctt aggcattgaa gtgagactga tgagacttca   1126920
     cactgtaccc ttcagaaggg cagacactca cgagataatc aaccacggct ccctcgctgg   1126980
     gaggcagaca cagagctgga ggcggcggcc acaaactagg tgggcagaga ggcgtcagca   1127040
     tcgggacttg cgggaggccc tgggaaggaa caggcagcgg tggggcctgg acagccctgg   1127100
     ggaaaggaag ttacttggag gagaccaggg gcttgcattt gggccaaaac caaggacaag   1127160
     agatcagctg gtcccaggga actctggagc ccaacacccc accacgggtg caggggcctg   1127220
     acaacccaac agagggctgt gggcaccggg caccagcctg tggggggtct gaaggctgct   1127280
     gcccagccac acatgtcctg gttctgaaca aagggttcaa gagacacctg gaaacctacc   1127340
     cttctgagag cagcctctta gcctcagctg gtattggccc ctggtcaccc cacatgcccc   1127400
     gacagtgggg cctggcctca gaaaggggcc cctccatttg tactttctat ctgatccttg   1127460
     acacagtgct aacaccaaag accaaacccg aatcttggtt cacatactct gcaaagaaat   1127520
     gactaagaca agttcttccg gaggtcagtt ttagaacatt attctgactt aattctgcct   1127580
     tatctctgtg caacaagaaa actatctgca catctgtagt aaaatgtgaa ggtaccttca   1127640
     ttttgtttgt atactaaact tttaagaaat acctcacttt ttaaaacttg gttttgtgtt   1127700
     ttgtatgctt ataatatttt ccataataga tcattttaat gataaactct gaatcttctg   1127760
     acagaatgca gatcaatgga ttcctggcca tcagggaggc gttgattgca aaggggcctg   1127820
     gtggaatatt tggagagatt ctacatcttc ttgcaactgt tcatctggat tacggtggtg   1127880
     gttgcatgag tgtgcatttg ttgaaactca catgtcacag gatggattat actgaatgta   1127940
     aactacactt gcgtaaatat gacttttaaa actttaaaaa gttgggggat ggttcactgc   1128000
     cccggctcga agccccctgg ccacgctgcc tggccagccc acccgcatcc ctgccagcgc   1128060
     ttgcctccag cgtcccaaca gcctcctgac cccctgggct gctctaaacc ctcaggagcg   1128120
     cagccatccg ggatcagctg gatggagatg gggagcccga gactcgtgcc acaccacgtc   1128180
     ctcccacccc caccagccac acgcagacgt caaagcagca ctgtcctcgc acgcgctgct   1128240
     cccacccacc ctggcatggc cattccaggc ctggggcagg aaggcagatg ctccccctgc   1128300
     ccccagacac aagcattcct gcacataccc ccagcacaca cacacactcc catgcgcaca   1128360
     ctgacacaca caggtgtgtg cagctgagac acagcccatt cccaggcagt ccagcccccg   1128420
     tcactgaggg aaaggggcag caccacgggg ccacaggagt ggcagctgga cacagagcca   1128480
     ggcgctggcg aaggcccagg ccactgaggc gggctgccgg catggctggg cgtgaaggcc   1128540
     agaagagggc aggaggggcc gtaggcactg tgcctattgg gcctaggtgg gcacacgcct   1128600
     ggcaggagag gaacagccca gcccctgagg aaggggtggg ggcaggggcc atttctggag   1128660
     gccagggcag ggccagcacc ccaaggaaaa gcagagcagg gtgagaacgg acatggggct   1128720
     cagagctgag caggcctgct gggccccagg agggagacac agacgaccgg ggatctcaag   1128780
     gctggcagag gccagagatg gagccccacc tgggatgcca tcctccttcc tgggggccca   1128840
     ccctgcccgg cccctccagc ccagcacagc ttggggcatt ggatagaacc gggagagagc   1128900
     cgaccaggca ctgaggcccc tgccccaaat gcccacagcc tggggaaaat gagcaggtac   1128960
     atgggagggg caagtggagc cccaggcaca cccacacagt gcacacggcc tcacctgggc   1129020
     cggagggggc aggaggctcg ccaccccgct gtggtttctc tcctaatctc accctgggtt   1129080
     tctgccacac ttgatgcagg tgatgtttct ctgacattgt ggactaagag ttggtgctgg   1129140
     aaggggttag ccatcttgga gatgttgcta tggggtgcag ggattttgcg tgtgagaagg   1129200
     acatgattat ggggggagcg gagggcaaac tgtcatgggt taaaatgtgt cccctataaa   1129260
     ttcatgtgtt gaagtcctaa cccccaggac cacagaatgt gaccttgttt ggaaacagtc   1129320
     tttgcagctg caatcaagtt cagatgaggt caccctggag tagcgcaagc ctctgatcca   1129380
     atatgactgc tgtcctcatg aaaaggggga atctgggcac agacagcacg tggggagaac   1129440
     gccctgtgaa gatggtgctg cttccataag ccaagagcag cagagacggc cggcaaagcc   1129500
     cagcagcagg gagagagcct gggacagagt ctcccatgac acagaggtgc cagccccacc   1129560
     gaggcctcca tcccagatgc ccggcctcca gaaccaggac ggaataaacg tctgttgttt   1129620
     aagccacgca gtctggggtg ctgtgttgcc agggccacag ttaacggata cgagtgttgt   1129680
     cctgagctgc cagccccaca ggctgcacga ggcctccctg ccccagccca gtgcagactc   1129740
     cccagccccc tgggtgtgcc atgggcagtg cggggacccc tcactccgtc ctcccccagc   1129800
     ctgggaggtt gagcccatta tgagctccat ggggtgaagc tggaacgaga ggctgggagc   1129860
     cgactgggag cctgcggctg gaggatggat ttccccaggg acccacacgt gcacctccac   1129920
     ctgtctcctg gacattctct ctgagggcag ggctggtgcc agctcaggga tccagcaggg   1129980
     tcacaagggc aggccgggtc cttgtggaga gcacatttag tgggagggac atgatttccc   1130040
     ttcaaagtgc ccattctgga cgcttcccgt tccatgctgg acgcttcctc ttccacgctg   1130100
     gatgcttcct gttccacact ggatgcttcc tgttccacgc tggatgtttc ctgttacact   1130160
     ctggatgctt cctgttccac actggatgct tcctgttcca tcctggatgc ttcctgttcc   1130220
     atgctggaca tttcctgttc cactctggat gctccctgtt ccatgctgga tgcttcctgt   1130280
     tccatgctgg atgcttcctg ttccatgctg gacatttcct gttccactct gcatgcttcc   1130340
     tgttccactc tggatgcttc ctgttccatg gtggacgttt cctgttccac tctgcatgct   1130400
     tcctgttcca tgctggatgc ttccttttcc attctggatg cttcctgttc catgttggat   1130460
     gtttcttgtt ccactctgga tgcttcctat tccattctgg atgcttcctg ttccatgctg   1130520
     gacatttcct gttccatgct ggatgctttc tgttacatcc tggatgcttc ctgttccatg   1130580
     ctggatgttt tttgtttgac tctggatgct tccagttcca ttctggatgc ttcctgttcc   1130640
     atgctggatg cttccttttc cattccgcac aattcctatt ccattctgga cacttcctgt   1130700
     gcgacacctc cttgggtttt ctgtctgccc agtccctcta tcctcatccc gttccctgct   1130760
     acctcccacc tccacaatcg tccttgccca gctcctccct ctctctagag cttcggcctg   1130820
     gcaaggtccc tcctgatctc agtccaggct cccccagcac aggtaggagc ctagcacctg   1130880
     cccttggacc tccccaccct gcatggtgcc agcatccccc ggtccccagg gaggccccat   1130940
     ttctctctct gcttgtagtc cagtggccct ggagtcccac tgcaactcgg gtgtgcccct   1131000
     gacctctgag gaagttaagt gtcctgtccc tagccaggct atcccgtctg ctcagcccca   1131060
     gggccctgcc cccaacccct tcccctcacc tgcaccacag gctctggcca actctgccca   1131120
     ggccctgaat gggcccctct ggctcccctc tgctgctaca ctgccctgca ccacctccac   1131180
     tcagcttcag tgtgttcatc cacctgtccc aagtcccctc ggcccccagg agcacagctg   1131240
     gtggccctgg ctcctggcag cccatcttgt tccttctgga gcaccagcct cagaggcctt   1131300
     cctgtgcagg gtccacttgg ccagccctgg gaccctcctg gtctcaagca cacacgttct   1131360
     ccctgcagcc agacctgccc ctgcctgtga gctcagccct gagccttgga atgccttccc   1131420
     atctccatcc cagctcgcct ttgccagctg ctcagcagga tgaactcaca ctcccctccc   1131480
     tgcaccatga gtcagagcca gctggagaca cgcccaggcc aaagcagcca ccagggccta   1131540
     gtgggggcca gaagcttcag atgagaggcc caggtattga gaggctgaga tcacgggcag   1131600
     aatggtcata atcgctgcca gtatcagtcc agccccaggg actcagagac agagaaaaga   1131660
     gcagcacaca aggtctgggc tccccacctt ctcccgtgag tacgggggag tatgggggca   1131720
     gccaccaccc ccatccccac acacccatga ggcagcctcg gctgtgtctg gactccccct   1131780
     cgccctctga cccagaaacc accagaagaa aagggaactt caggaagtaa gtggtgccgc   1131840
     cggtttcaat cctgttctta gtctttgcag cgtggagttc acacacctgg ggacctgggg   1131900
     gccgagctgt gatttcctag gaagacaaat agcggctgac ggcaggggcg gggctgccca   1131960
     catgtacctc gccagaacag gaagggctga gacccccacc tcggtgagtg gggtcagcac   1132020
     agggcagggg cacaggctcg ggaggaggac agagcctggg ggcagccgtg ggcgctcctg   1132080
     gacctgagat gctgaacagg ctccaagagg ctggggagac atggggtcga ggccggcccc   1132140
     acatggaggc ccaagcggag ccagcacggg ggaggtgggc agccttcagg caccgatgcc   1132200
     cacccagtgc gagacgacgg ggaccgtggg caggggcttc caagccaaca gggcaggaca   1132260
     caccagaggc tgactgaggc ctccaggacg accgggctgg gagcacgagg aacatgactg   1132320
     gatgcggcag agccggccgt ggggtgatgc caggatgggc acgaccgacc tgagctcagg   1132380
     aggcagcaga gcgagggagg aggagaggcc ccaggtgaac ggaggggctt gtccaggccg   1132440
     gcagcatcac cggagcccag ggcagggtca gcagtgctgg ccgtggggcc ctcctctcag   1132500
     ccaggaccaa ggacagcagg tgagccggga gcagagcagg gagggtgagt gtggcagcag   1132560
     gacaggaggg tggaagccaa ggagcccaga ggcagaggca gggacagggg aggcacaggg   1132620
     gctgggctca gagccagctg atggggttgg ggcacctgct ggcggggagc agggctgtgg   1132680
     tcagcagtgg agaggagggg agagctgtgc tgagtgcacg ggcgggagaa gggaagagtc   1132740
     cagggaggcc cagaaaggcc cagagtgcag caggcctggg gcgaggggaa gggctgaggc   1132800
     tccgtgcgtt cagggaactg acccagcaga gcagaggcca ctgaggagct gaggttccag   1132860
     agaggcttcc agagcaggag cagtgcaggg acaggaggat ccgggagctc attcaggagg   1132920
     ggcacatggg caagggcaag gggctctgtt ggggagacct gactggacac tggggctgct   1132980
     ccacagcata gggaacacgc caagtgctgc aaaatcaaaa atgagggcag aaaaacagcc   1133040
     caaacctgga cagagggtgc caggacaggc aggggggcaa cagtgacctg agtgacattg   1133100
     ctgcccgggt tgagggaggg cagagtgagc agggagcagg cattggagct cagggaccag   1133160
     gaccaagcag ccacaggtga gcagggcagg tgggggcaga aggagcaggg ggcacctcct   1133220
     ggagctcagg ggaccagggc agagcagcct caggtgagca ggggctggtg ggcggcagga   1133280
     tgagcagggg gaagaccctg gagctcaggg gaccagggca gagcatcaga aggtgagcat   1133340
     ggctagtggg agatgggcaa gcagggggca gcccctggag ctcaggggac caggacagag   1133400
     catcaggagg tgagcatggc tagtgggagg tgggcgagca gggggcagcc cctggaactc   1133460
     aggggaccag ggcagagcag ccgcaggtga gcacgggctg gtgggaggcg ggaggaacag   1133520
     ggggcagctc ctggacttca ggggaccagg gagggcatct gaaggtgaac aggggccagt   1133580
     ggggggcagg atgagcaggg ggaagctcct ggagctcagg gagccaaggc agagcagccg   1133640
     caggtcagca ggggcaggtg ggaagcatgg ggagcaggtg ggcagcccct ggagctcaga   1133700
     gagccagggc agatcatcca caggagagca ggggctggta ggaagcagga ggagcaagtg   1133760
     ggcagctttt ggagctcaga gcaccagggc agaagagcct caggtgagaa ggggcaggtg   1133820
     ggaggcagaa taagcagggg acagcccctg gacctcagga gaccagggca gagcatcaca   1133880
     acgtcagcat ggctggtggg aggtgggcga gcagggggca gcccctggac ctcagagagc   1133940
     cagggcagat ctgcaggtga gcaggggcag gtgggaggca ggaagagcag gaggcagctc   1134000
     ctggagctca ggggatcagg gcagagcagc cacaggtgag caggggcagg taggaagcag   1134060
     aaagatcagg ggtcagcccc tggagctcag gggacaaggg gagagcatca gaaggtgagc   1134120
     aggactgagg ctcagcctca gggagccagg gcagagcagc tgcaggtgag cagggccggt   1134180
     gggaagcagg aggagcaggt gggcagcccc tggagctcag agagccaggg aagatcatcc   1134240
     gcaggtgagc aggggctggt gggaagcagg aggagcaagg ggcagctcct ggagctcagg   1134300
     ggaccagggc agagcagtcg caggtgaaca ggggcaggtg gggggcagga ggagcaagga   1134360
     gcagctcctg gagctcaggg gaccagggca gagcagtcgc aggtgaacag gggcaggagg   1134420
     agcaaggggc agctcctgga gctcagggga ccagggcaga gcagccgcag gtgagcaggt   1134480
     gcaggtgggg ggcaggagga gcagggggca gctcctggag ctcaggggac cagggcagag   1134540
     cagccgcagg tgagcagggg caggtggggt gcaggaggag cagggggcag gcactggagc   1134600
     tcaggggacc agggcagagc agtcgcaggt gaacaggggc aggtgggggg caggagtagc   1134660
     aaggggcagc tcctggagct caggggacca gggcagagca gtcgcaggtg aacaggggca   1134720
     ggtggggggc aggaggagca gggggcagct cctggagctc aggggaccag ggcagagcag   1134780
     ccgcaggtga gcaggtgcag gtggggggca ggaggagcag gggtcaggca ctggagctca   1134840
     ggggaccagg gcagagcagc cgcaggtgag caggggcagg tggggggcag gaggagcagg   1134900
     gggcaggcac tggagctcag gggaccaggg cagagcagcc gcaggtgagc aggggcaggt   1134960
     ggggggcagg aggagcaggg ggcaggcact ggagctcagg ggaccagggc agagcagccg   1135020
     caggtgagca ggggcaggtg gggggcagga ggagcagggg gcaggcactg gagctcaggg   1135080
     gaccagggca gagcagccgc aggtcagcag ggccggtggg aggcaggacg agcaggggac   1135140
     aggcactaga gctcagggca aggcagccac aggtgagcag ggctggtggg aggcatcact   1135200
     cagctcctag actttggcag gagctgggta gttgctggca acagacagct gagggctggt   1135260
     gaaagtgcag tgcagcctcc tggtgccggg aagggagtgt gagtccatcc cactgagcag   1135320
     ttggcaaggg cgagctggga tggagaaggg aaggcgttcc agggctcagg gctgagctct   1135380
     caggcagggg caggtgtggc tgcaggggga acgtgtgctt gagaccagga gggtcccacg   1135440
     gctggtccca gcggaccctg ggcaggaagg cctctgaggc tggcgcccca gaaggagcaa   1135500
     gatgggctgc caggagccag ggccaccagc acaatgaagc tgagtggagg tggtgcaggg   1135560
     cagtgtagca gcagagggca gccagagggg cccattcagg gcctgggcag agtcagccag   1135620
     agcctgtggt gcaggtgagg ggaaggggtg gtgagcgggg ccctggggct gagcagaggg   1135680
     gatggcctgg ctgagggcag ggcgcttagc ctcctcagag gtcaggggca caccccacct   1135740
     gcagtgggac tccagggcca ctgggccagc ggcagagaga aatggggcct ccctgtggcc   1135800
     tgggggtcct ggcaccatgc agggtgggga gggccaaggg caggtgcaag gctcctacct   1135860
     gtgctggggg gcctgggttg agcccagcag ggaccttgcc gggggaagct ctggagagag   1135920
     ggaggaggtg ggctggtggc cgagaaggcc aggccagggc tgggagggtg acggtgtggt   1135980
     gactgagcct ccagaagtaa tgcaggacac tgggaggcag ggggcatcca ggcactcagg   1136040
     gccctgacct gggctgctgc acactggggc taaggggaaa ggaggggaga ggctgaggag   1136100
     gaggctccag gaggctattc caaggcaggg ggttccgggg ccctggggct gaagggcgcc   1136160
     gaccctatgc agtgtctggc ccctctgctg cacagaagaa aagggccttg gagggcagag   1136220
     ggcaggctat gaccagggcc ctgggcaagt caggccaact cactagggga gggccacgct   1136280
     ggggcggcag ggtcaggggc ttcagggggc tcgggggacc cacgagaagc catctgagaa   1136340
     cagtgtccac tggtcaagcc aggcacccat aaaaggctgg agtggggcca atgggcatga   1136400
     gccgtccctg aggtggcacc gatggccaga gctgaggcca agctagaggc cctggactgt   1136460
     gctgactccc ggcagacaca gagcgctgac ctggctgccg agccccgcct cctaggctgc   1136520
     aggggtgcct gcagaagggc accacagggc caccggtcct gcaagctttc tggggcaggc   1136580
     cgggcctgac cttggctttg gggcaggggg tgggctaagg tgacgcaggt ggcgccagcc   1136640
     aggcgcacac ccaatgcccg tgagcccaga cactggacgc tgaacctcgc ggacagttaa   1136700
     gaacccaggg gcctctgcgc cctgggccca gctctgtccc acaccgcggt cacatggcac   1136760
     cacctctctt gcagcctcca ccaagggccc atcggtcttc cccctggcac cctcctccaa   1136820
     gagcacctct gggggcacag cagccctggg ctgcctggtc aaggactact tccccgaacc   1136880
     ggtgacggtg tcgtggaact caggcgccct gaccagcggc gtgcacacct tcccggctgt   1136940
     cctacagtcc tcaggactct actccctcag cagcgtggtg accgtgccct ccagcagctt   1137000
     gggcacccag acctacatct gcaacgtgaa tcacaagccc agcaacacca aggtggacaa   1137060
     gaaagttggt gagaggccag cacagggagg gagggtgtct gctggaagcc aggctcagcg   1137120
     ctcctgcctg gacgcatccc ggctatgcag ccccagtcca gggcagcaag gcaggccccg   1137180
     tctgcctctt cacccggagg cctctgcccg ccccactcat gctcagggag agggtcttct   1137240
     ggctttttcc ccaggctctg ggcaggcaca ggctaggtgc ccctaaccca ggccctgcac   1137300
     acaaaggggc aggtgctggg ctcagacctg ccaagagcca tatccgggag gaccctgccc   1137360
     ctgacctaag cccaccccaa aggccaaact ctccactccc tcagctcgga caccttctct   1137420
     cctcccagat tccagtaact cccaatcttc tctctgcaga gcccaaatct tgtgacaaaa   1137480
     ctcacacatg cccaccgtgc ccaggtaagc cagcccaggc ctcgccctcc agctcaaggc   1137540
     gggacaggtg ccctagagta gcctgcatcc agggacaggc cccagccggg tgctgacacg   1137600
     tccacctcca tctcttcctc agcacctgaa ctcctggggg gaccgtcagt cttcctcttc   1137660
     cccccaaaac ccaaggacac cctcatgatc tcccggaccc ctgaggtcac atgcgtggtg   1137720
     gtggacgtga gccacgaaga ccctgaggtc aagttcaact ggtacgtgga cggcgtggag   1137780
     gtgcataatg ccaagacaaa gccgcgggag gagcagtaca acagcacgta ccgtgtggtc   1137840
     agcgtcctca ccgtcctgca ccaggactgg ctgaatggca aggagtacaa gtgcaaggtc   1137900
     tccaacaaag ccctcccagc ccccatcgag aaaaccatct ccaaagccaa aggtgggacc   1137960
     cgtggggtgc gagggccaca tggacagagg ccggctcggc ccaccctctg ccctgagagt   1138020
     gaccgctgta ccaacctctg tccctacagg gcagccccga gaaccacagg tgtacaccct   1138080
     gcccccatcc cgggatgagc tgaccaagaa ccaggtcagc ctgacctgcc tggtcaaagg   1138140
     cttctatccc agcgacatcg ccgtggagtg ggagagcaat gggcagccgg agaacaacta   1138200
     caagaccacg cctcccgtgc tggactccga cggctccttc ttcctctaca gcaagctcac   1138260
     cgtggacaag agcaggtggc agcaggggaa cgtcttctca tgctccgtga tgcatgaggc   1138320
     tctgcacaac cactacacac agaagagcct ctccctgtct ccgggtaaat gagtgccacg   1138380
     gccggcaagc ccccgctccc caggctctcg gggtcgcgcg aggatgcttg gcacgtaccc   1138440
     cgtgtacata cttcccaggc acccagcatg gaaataaagc acccagcgct tccctgggcc   1138500
     cctgcgagac tgtgatggtt ctttccacgg gtcaggccga gtctgaggcc tgagtggcat   1138560
     gagggaggca gagtgggtcc cactgtcccc acactggccc aggctgtgca ggtgtgcctg   1138620
     ggccgcctag ggtggggctc agccaggggc tgccctcggc agggtggggg atttgccagc   1138680
     gtggccctcc ctccagcagc agctgccctg ggctgggcca cgagaagccc taggagcccc   1138740
     tggggacaga cacacagccc ctgcctctgt aggagactgt cctgttctgt gagcgccctg   1138800
     tcctccgacc cgcatgccca ctcgggggca tgcctagtcc atgtgcgtag ggacaggccc   1138860
     tccctcaccc atctaccccc acggcactaa cccctggcag ccctgcccag cctcgcaccc   1138920
     gcatggggac acaaccgact ccggggacat gcactctcgg gccctgtgga gagactggtc   1138980
     cagatgccca cacacacact cagcccagac ccgttcaaca aaccccgcac tgaggttggc   1139040
     cggccacacg gccaccacac acacacgtgc acgcctcaca cacggagcct cacccgggcg   1139100
[truncated: 208,704 more chars]
