# Supplementary material for: Genotyping of selected germline adaptive immune system loci using short-read sequencing data
Source: Genome Res. 2025 Sep;35(9):2076–86. doi: 10.1101/gr.280314.124 (PMC12401057; doi:10.1101/gr.280314.124)
Supplement: Supplement 1 [file Supplemental_Code.zip › ImmunoTyper2-methods/HPRC-assembly-benchmarking/pileup-example/mview-I20250417-165536-0417-23955503-p1m.html]

MView


```
Reference sequence (1): HG00438_maternal-IGLV2-14_05_c=a010000050127948463-28872075_s=320422_e=320706_-
Identities normalised by aligned length.
Colored by: identity
```

```
                                                                                              cov    pid   1 [        .         .         .         .         :         .         .         . 80 
 1 HG00438_maternal-IGLV2-14_05_c=a010000050127948463-28872075_s=320422_e=320706_-         100.0% 100.0%     --CCTTATCAGTCCTGTTACATCTTCTCAGCATCTGAAACCACTGGAGAGGTGGTCACCATCTCCTGTGCTG-ACACAGC    
 2 HG00438_paternal-IGLV2-14_05_c=hg004381jahbcb010000195129541-926378_s=260553_e=260837_+ 100.0%  99.6%     --CCTTATCAGTCCTGTTACATCTTCTCAGCATCTGGAACCACTGGAGAGGTGGTCACCATCTCCTGTGCTG-ACACAGC    
 3 HG00438_maternal-IGLV2-14_03_c=a010000050127948463-28872075_s=507469_e=507761_-          96.5%  36.6%     GGCAGTCAGGGCTCACTTGGTGGCCTGC--ACAGTTTGTCTGTGACTAAACCAGCCACCATCTCCTGCACTGGAAACAGC    
 4 HG00438_paternal-IGLV2-14_03_c=hg004381jahbcb010000195129541-926378_s=73795_e=74087_+    96.5%  36.3%     GGCAGTCAGGGCTCACTTGGTGGCCTGC--ACAGTTTGTCTGTGACTAAACCAGCCACCATCTCCTGCACTGGAAACAGC    
 5 IGLV2-14*05                                                                              98.9%  51.7%     --CAGTCTGCCCTGACTCAGCCTGCCTCCGTGTCTGGGTCTCCTGGACAGTCGATCACCATCTCCTGCACTGGAACCAGC    
 6 HG00438_maternal-IGLV2-14_01_c=a010000050127948463-28872075_s=174329_e=174625_-          98.9%  50.3%     --CAGTCTGCCCTGACTCAGCCTGCCTCCGTGTCTGGGTCTCCTGGACAGTCGATCACCATCTCCTGCACTGGAACCAGC    
 7 IGLV2-14*03                                                                              98.9%  50.0%     --CAGTCTGCCCTGACTCAGCCTGCCTCCGTGTCTGGGTCTCCTGGACAGTCGATCACCATCTCCTGCACTGGAACCAGC    
 8 HG00438_paternal-IGLV2-14_01_c=hg004381jahbcb010000195129541-926378_s=406753_e=407049_+  98.9%  50.7%     --CAGTCTGCCCTGACTCAGCCTGCCTCCGTGTCTGGGTCTCCTGGACAGTCGATCACCATCTCCTGCACTGGAACCAGC    
 9 IGLV2-14*01                                                                              98.9%  50.7%     --CAGTCTGCCCTGACTCAGCCTGCCTCCGTGTCTGGGTCTCCTGGACAGTCGATCACCATCTCCTGCACTGGAACCAGC    
10 IGLV2-14*04                                                                              90.9%  52.7%     -------------------------CTCCGTGTCTGGGTCTCCTGGACAGTCGATCACCATCTCCTGCACTGGAACCAGC    
   consensus/100%                                                                                            .........................ssC..ssssTssusCssssusssAussuusCACCATCTCCTGsuCTG.AssCAGC    
   consensus/90%                                                                                             ..CssTssssssssssTsussssssssC..ssssTssusCssssusssAussuusCACCATCTCCTGsuCTG.AssCAGC    
   consensus/80%                                                                                             ..CssTssssssssssTsussssssCTCsGsuTCTGuGTCTsCTGGAsAGsCGuTCACCATCTCCTGCACTGGAAsCAGC    
   consensus/70%                                                                                             ..CAGTCTGssCTsACTsAGssssCCTCsGsuTCTGGGTCTsCTGGAsAGsCGuTCACCATCTCCTGCACTGGAAsCAGC    

                                                                                              cov    pid  81          .         1         .         .         .         .         :         . 160
 1 HG00438_maternal-IGLV2-14_05_c=a010000050127948463-28872075_s=320422_e=320706_-         100.0% 100.0%     AGTGACATTGGGGGTTATAATTCTGTCTCTTGGTAACAAAAGCAACAGGGAAGAGCCCCATATG-ACTGAGGGGTGAGAT    
 2 HG00438_paternal-IGLV2-14_05_c=hg004381jahbcb010000195129541-926378_s=260553_e=260837_+ 100.0%  99.6%     AGTGACATTGGGGGTTATAATTCTGTCTCTTGGTAACAAAAGCAACAGGGAAGAGCCCCATATG-ACTGAGGGGTGAGAT    
 3 HG00438_maternal-IGLV2-14_03_c=a010000050127948463-28872075_s=507469_e=507761_-          96.5%  36.6%     AGCAATAAC---GTCCTTGGAATTGTGTCCTGGTACCAACAATGAACAGGAAGTGTCCTTAAACCTCTGATCTGTGGAT-    
 4 HG00438_paternal-IGLV2-14_03_c=hg004381jahbcb010000195129541-926378_s=73795_e=74087_+    96.5%  36.3%     AGCAATAAC---GTCCTTGGAATTGTGTCCTGGTACCAACAATGAACAGGAAGTGTCCTTAAACCTCTGATCTGTGGAT-    
 5 IGLV2-14*05                                                                              98.9%  51.7%     AGTGACGTTGGTGGTTATAACTATGTCTCCTGGTACCAACAGCACCCAGGCAAAGCCCCCAAACTCATGATTTATGAGGT    
 6 HG00438_maternal-IGLV2-14_01_c=a010000050127948463-28872075_s=174329_e=174625_-          98.9%  50.3%     AGTGACGTTGGTGGTTATAACTATGTCTCCTGGTACCAACAGCACCCAGGCAAAGCCCCCAAACTCATGATTTATGATGT    
 7 IGLV2-14*03                                                                              98.9%  50.0%     AGTGACGTTGGTGGTTATAACTATGTCTCCTGGTACCAACAACACCCAGGCAAAGCCCCCAAACTCATGATTTATGATGT    
 8 HG00438_paternal-IGLV2-14_01_c=hg004381jahbcb010000195129541-926378_s=406753_e=407049_+  98.9%  50.7%     AGTGACGTTGGTGGTTATAACTATGTCTCCTGGTACCAACAGCACCCAGGCAAAGCCCCCAAACTCATGATTTATGAGGT    
 9 IGLV2-14*01                                                                              98.9%  50.7%     AGTGACGTTGGTGGTTATAACTATGTCTCCTGGTACCAACAGCACCCAGGCAAAGCCCCCAAACTCATGATTTATGAGGT    
10 IGLV2-14*04                                                                              90.9%  52.7%     AGTGACGTTGGTGGTTATAACTATGTCTCCTGGTACCAACAGCACCCAGGCAAAGCCCCCAAACTCATGATTTATGATGT    
   consensus/100%                                                                                            AGsuAsuss...GssssTuusssTGTsTCsTGGTAsCAAsAususssuGGsAusGsCCsssAss.ssTGAsssuTGuss.    
   consensus/90%                                                                                             AGsuAsuss...GssssTuusssTGTsTCsTGGTAsCAAsAususssuGGsAusGsCCsssAss.ssTGAsssuTGuss.    
   consensus/80%                                                                                             AGTGACuTTGGsGGTTATAAsTsTGTCTCCTGGTACCAACAuCAsCCAGGsAuAGCCCCsAAACsssTGATsTuTGAsuT    
   consensus/70%                                                                                             AGTGACuTTGGsGGTTATAAsTsTGTCTCCTGGTACCAACAGCAsCCAGGsAuAGCCCCsAAACsssTGATsTuTGAuuT    

                                                                                              cov    pid 161          .         .         .         2         .         .         .         . 240
 1 HG00438_maternal-IGLV2-14_05_c=a010000050127948463-28872075_s=320422_e=320706_-         100.0% 100.0%     CAGGAAAAAGCCTCAGAGATTCCA-GTTCTAATCTCAGGCCGTTCTTTGTATTCTTTTATGAGCTAGTTAGTCAGCTGGT    
 2 HG00438_paternal-IGLV2-14_05_c=hg004381jahbcb010000195129541-926378_s=260553_e=260837_+ 100.0%  99.6%     CAGGAAAAAGCCTCAGAGATTCCA-GTTCTAATCTCAGGCCGTTCTTTGTATTCTTTTATGAGCTAGTTAGTCAGCTGGT    
 3 HG00438_maternal-IGLV2-14_03_c=a010000050127948463-28872075_s=507469_e=507761_-          96.5%  36.6%     -GTAAATATCCACCTGCAGATCCAGAACTGATTCTTGAGTGA---TCAGTCTGGCAAGGAGGCCTTCCTGAGCATATCTG    
 4 HG00438_paternal-IGLV2-14_03_c=hg004381jahbcb010000195129541-926378_s=73795_e=74087_+    96.5%  36.3%     -GTAAATATCCACCTGCAGATCCAGAACTGATTCTTGAGTGA---TCAGTCTGGCAAGGAGGCCTTCCTGAGCATATCTG    
 5 IGLV2-14*05                                                                              98.9%  51.7%     CAGTAATCGGCCCTCAGGGGTCCCTGATCGCTTCTCTGGCTC---CAAGTCTGGCAACACGGCCTCCCTGACCATCTCTG    
 6 HG00438_maternal-IGLV2-14_01_c=a010000050127948463-28872075_s=174329_e=174625_-          98.9%  50.3%     CAGTAAGCGGCCCTCAGGGGTTTCTAATCGCTTCTCTGGCTC---CAAGTCTGGCAACACGGCCTCCCTGACCATCTCTG    
 7 IGLV2-14*03                                                                              98.9%  50.0%     CAGTAATCGGCCCTCAGGGGTTTCTAATCGCTTCTCTGGCTC---CAAGTCTGGCAACACGGCCTCCCTGACCATCTCTG    
 8 HG00438_paternal-IGLV2-14_01_c=hg004381jahbcb010000195129541-926378_s=406753_e=407049_+  98.9%  50.7%     CAGTAATCGGCCCTCAGGGGTTTCTAATCGCTTCTCTGGCTC---CAAGTCTGGCAACACGGCCTCCCTGACCATCTCTG    
 9 IGLV2-14*01                                                                              98.9%  50.7%     CAGTAATCGGCCCTCAGGGGTTTCTAATCGCTTCTCTGGCTC---CAAGTCTGGCAACACGGCCTCCCTGACCATCTCTG    
10 IGLV2-14*04                                                                              90.9%  52.7%     CAGTAATCGGCCCTCAGGGGTTTCTAATCGCTTCTCTGGCTC---CAAGTCTGGCAACACGGCCTCCCTGACCATCTCTG    
   consensus/100%                                                                                            .ussAAssssCssssusuusTsss.ussssssTCTssuGsss...sssGTsTssssssusGusCTsssTuusCAssTsss    
   consensus/90%                                                                                             .ussAAssssCssssusuusTsss.ussssssTCTssuGsss...sssGTsTssssssusGusCTsssTuusCAssTsss    
   consensus/80%                                                                                             CAGsAAssuGCCCssuuGGuTssssuATCGsTTCTCsGGCss...ssAGTCTGGCAAsAsGGCCTsCCTGAsCATCTCTG    
   consensus/70%                                                                                             CAGsAATsuGCCCssuuGGuTssssAATCGsTTCTCsGGCss...ssAGTCTGGCAAsAsGGCCTsCCTGAsCATCTCTG    

                                                                                              cov    pid 241          :         .         .         .         .         3  ] 303
 1 HG00438_maternal-IGLV2-14_05_c=a010000050127948463-28872075_s=320422_e=320706_-         100.0% 100.0%     CAGTCAGCTGTGGCCACAAGAAGAAATGGAAGAGAGGTTCAGAA------AACAGC-------    
 2 HG00438_paternal-IGLV2-14_05_c=hg004381jahbcb010000195129541-926378_s=260553_e=260837_+ 100.0%  99.6%     CAGTCAGCTGTGGCCACAAGAAGAAATGGAAGAGAGGTTCAGAA------AACAGC-------    
 3 HG00438_maternal-IGLV2-14_03_c=a010000050127948463-28872075_s=507469_e=507761_-          96.5%  36.6%     GGCTCCAGGCTGAGGACAAGGCTGATCACTAACGTTGGATTTGAACAGTTCTCTGGAGGCCCC    
 4 HG00438_paternal-IGLV2-14_03_c=hg004381jahbcb010000195129541-926378_s=73795_e=74087_+    96.5%  36.3%     GGCTCCAGGCTGAGGACAAGGCTGATCATTAACGTTGGATTTGGACAGTTCTCTGGAGGCCCC    
 5 IGLV2-14*05                                                                              98.9%  51.7%     GGCTCCAGGCTGAGGACGAGGCTGATTATTACTGCAGCTCATATACAAGCAGCAGCACTCTC-    
 6 HG00438_maternal-IGLV2-14_01_c=a010000050127948463-28872075_s=174329_e=174625_-          98.9%  50.3%     GGCTCCAGGCTGAGGACGAGGCTGATTATTACTGCAGCTCATATACAAGCAGCAGCACTTTC-    
 7 IGLV2-14*03                                                                              98.9%  50.0%     GGCTCCAGGCTGAGGACGAGGCTGATTATTACTGCAGCTCATATACAAGCAGCAGCACTCTC-    
 8 HG00438_paternal-IGLV2-14_01_c=hg004381jahbcb010000195129541-926378_s=406753_e=407049_+  98.9%  50.7%     GGCTCCAGGCTGAGGACGAGGCTGATTATTACTGCAGCTCATATACAAGCAGCAGCACTCTC-    
 9 IGLV2-14*01                                                                              98.9%  50.7%     GGCTCCAGGCTGAGGACGAGGCTGATTATTACTGCAGCTCATATACAAGCAGCAGCACTCTC-    
10 IGLV2-14*04                                                                              90.9%  52.7%     GGCTCCAGGCTGAGGACGAGGCTGATTATTACTGCAGCTCATATACAAGCAGCAGCACTCTC-    
   consensus/100%                                                                                            susTCsusssTGussACuAGussuAssussAssGssGsssssus......ssCsGs.......    
   consensus/90%                                                                                             susTCsusssTGussACuAGussuAssussAssGssGsssssus......ssCsGs.......    
   consensus/80%                                                                                             GGCTCCAGGCTGAGGACuAGGCTGATTAsTAssGsuGsTCATAsACAussAuCAGCAssssC.    
   consensus/70%                                                                                             GGCTCCAGGCTGAGGACuAGGCTGATTATTAssGsuGsTCATAsACAussAuCAGCAssCsC.
```

MView 1.67, Copyright © 1997-2020 Nigel P. Brown
