# Supplementary material for: Genotyping of selected germline adaptive immune system loci using short-read sequencing data
Source: Genome Res. 2025 Sep;35(9):2076–86. doi: 10.1101/gr.280314.124 (PMC12401057; doi:10.1101/gr.280314.124)
Supplement: Supplement 1 [file Supplemental_Code.zip › ImmunoTyper2-methods/HPRC-assembly-benchmarking/receptor_utils/docs/_build/html/_modules/receptor_utils/novel_allele_name.html]

receptor\_utils.novel\_allele\_name — receptor-utils documentation


receptor-utils

Contents:

- Introduction
- Using AIRR Community Reference Sets with IgBLAST
- Using AIRR Community Reference Sets with MiXCR
- Using custom databases with IgBlast
- Command-Line Utilities
- receptor\_utils

receptor-utils

- Module code
- receptor\_utils.novel\_allele\_name

---

# Source code for receptor\_utils.novel\_allele\_name

```
# Provide a name for a novel allele sequence, using the VDJbase approach as outlined
# at https://wordpress.vdjbase.org/index.php/vdjbase_help/airr-seq-data-allele-names/
# For V sequences, the sequence must be full-length at the 5' end, or IMGT-gapped.

# Copyright (c) 2021 William Lees

# This source code, and any executable file compiled or derived from it, is governed by the European Union Public License v. 1.2,
# the English version of which is available here: https://perma.cc/DK5U-NDVE


from receptor_utils import simple_bio_seq as simple
from receptor_utils import number_v
from collections import namedtuple
from Bio import Align


# Make a name for the novel allele, given its gapped or ungapped sequence
# returns a tuple (name, gapped_sequence)
#
# novel_seq: the full-length sequence to name, which may be either gapped or ungapped in the case of a V-gene
# ref_set: dict of reference genes (gapped in the case of v-genes), in the format returned by read_fasta
# v_gene: True if we are naming a v_gene
# gaps in V-sequences are '.', as in the IMGT reference set

[docs]def name_novel(novel_seq: str, ref_set: dict, v_gene: bool = True):

    """Make a name for the novel allele, given its gapped or ungapped sequence.
    The name conforms to the description `here <https://wordpress.vdjbase.org/index.php/vdjbase_help/airr-seq-data-allele-names/>`_ .
    The sequence must be full-length at the 5 prime end, or gapped

    :param novel_seq: the full-length sequence to name, which may be either gapped or ungapped in the case of a V-gene
    :type novel_seq: str
    :param ref_set: dict of reference genes (gapped in the case of v-genes), in the format returned by read_fasta
    :type ref_set: dict
    :param v_gene: True only if we are naming a v_gene
    :type v_gene: bool
    :return: tuple consisting of three strings: novel_name, novel_seq, notes. novel_seq is gapped in the case of a V-gene
    :rtype: tuple
    """

    novel_seq = novel_seq.upper()
    notes = ''

    # Check for truncation at 5' end and construct ambiguous reference set if necessary

    if novel_seq[0] == '.':
        for i in range(0, len(novel_seq)):
            if novel_seq[i] != '.':
                break
        ref_set = build_ambiguous_ref(ref_set, i)

        # TODO - handle ungapped sets that are truncated at the 5' end
    else:
        # gap the sequence (even if already gapped) so that we get the notes
    
        if v_gene:
            if '.' in novel_seq:
                novel_seq = novel_seq.replace('.', '')
    
            ungapped_ref_set = {}
            for k, v in ref_set.items():
                ungapped_ref_set[k] = v.replace('.', '')
            novel_seq, aa, notes = number_v.gap_sequence(novel_seq, ref_set, ungapped_ref_set)


    closest_ref_name = closest_aligned_ref(novel_seq, ref_set)[0]
    closest_ref_seq = ref_set[closest_ref_name]

    diffs = {}
    insertions = []
    class Diff:
        pass

    # check for indels

    n_diffs = simple.nt_diff(closest_ref_seq, novel_seq)
    if n_diffs > 0:
        ungapped_closest_ref = closest_ref_seq.replace('.', '')
        fixed_seq, insertions = find_indels(ungapped_closest_ref, novel_seq.replace('.', ''))
        fixed_diffs = simple.nt_diff(ungapped_closest_ref, fixed_seq)
        if fixed_diffs < n_diffs:
            # thread the gaps through the fixed sequence and adjust insertion positions
            novel_seq = ''
            fixed_ind = 0
            for i in range(len(closest_ref_seq)):
                if closest_ref_seq[i] != '.':
                    novel_seq += fixed_seq[fixed_ind]
                    fixed_ind += 1
                else:
                    novel_seq += '.'
                    for insertion in insertions:
                        if insertion[0] > i:
                            insertion[0] += 1
                            
            if fixed_ind < len(fixed_seq):
                for i in range(fixed_ind, len(fixed_seq)):
                    novel_seq += fixed_seq[i]
                            
    if len(closest_ref_seq) < len(novel_seq):
        closest_ref_seq += '.' * (len(novel_seq) - len(closest_ref_seq))

    for i in range(0, len(novel_seq)):
        if closest_ref_seq[i] != novel_seq[i]:
            d = Diff()
            d.pos = i
            d.ref = closest_ref_seq[i]
            d.novel = novel_seq[i]
            diffs[i] = d

    # trim any diffs that are past the end of the novel sequence

    for i in range(len(novel_seq) - 1, -1, -1):
        if novel_seq[i] != '.':
            break

    for i in list(diffs.keys()):
        if i >= len(novel_seq):
            del diffs[i]

    # add diffs if the closest ref is longer than the novel seq

    if len(closest_ref_seq) > len(novel_seq):
        for j in range(len(novel_seq), len(closest_ref_seq)):
            d = Diff()
            d.pos = j
            d.ref = closest_ref_seq[j]
            d.novel = '-'
            diffs[j] = d


    # consolidate any adjacent diffs

    prev_i = None
    for i, d in list(diffs.items()):
        if prev_i and diffs[prev_i].pos + len(diffs[prev_i].novel) == i:
            diffs[prev_i].ref += closest_ref_seq[i]
            diffs[prev_i].novel += novel_seq[i] if i < len(novel_seq) else '-'
            del diffs[i]
        else:
            prev_i = i

    # split diffs that are too short to consolidate

    for i, d in list(diffs.items()):
        if 1 < len(d.novel) < 4 and '.' not in d.novel and '.' not in d.ref:
            for j in range(1, len(d.novel)):
                diffs[i + j] = Diff()
                diffs[i + j].pos = d.pos + j
                diffs[i + j].ref = d.ref[j]
                diffs[i + j].novel = d.novel[j]
            d.ref = d.ref[0]
            d.novel = d.novel[0]

    # form the suffix

    all_positions = list(diffs.keys())
    all_positions.extend([i[0] for i in insertions])
    all_positions = list(set(all_positions))
    all_positions.sort()

    suffs = []

    # create the name suffix parts
    
    for i in all_positions:
        if i in diffs:
            d = diffs[i]
            if len(d.novel) == 1 and d.ref != '.' and d.novel != '.':
                suffs.append('%s%d%s' % (d.ref.lower(), d.pos + 1, d.novel.lower()))
            else:
                suffs.append('%d%s%d' % (d.pos + 1, d.novel.lower(), d.pos + len(d.novel)))

        insertions_at_i = [insertion for insertion in insertions if insertion[0] == i]
        if len(insertions_at_i) > 0:
            suffs.append('i%d%s' % (i + 1, ''.join([insertion[1].lower() for insertion in insertions_at_i])))

    novel_name = closest_ref_name + '_' + '_'.join(suffs) if len(suffs) else closest_ref_name

    # put the insertions into the sequence. 

    for i in range(len(insertions) - 1, -1, -1):
        novel_seq = novel_seq[:insertions[i][0] + 1] + insertions[i][1] + novel_seq[insertions[i][0] + 1:]

    return novel_name, novel_seq, notes


[docs]def aligned_diff(novel_seq: str, ref_seq: str):
    aligner = Align.PairwiseAligner()
    aligner.mode = 'global'
    aligner.open_gap_score = -5
    aligner.extend_gap_score = -1
    aligner.match_score = 2
    aligner.mismatch_score = 0
    aligner.target_end_gap_score = 1

    best_alignment = aligner.align(novel_seq, ref_seq)[0]

    # return 0-based start, end and score
    return best_alignment[0], best_alignment[1], best_alignment.score

# Find the closest reference sequence to a query sequence using a global alignment
# Returns a list of equally close reference names

[docs]def closest_aligned_ref(seq: str, ref: dict):
    """Given an input sequence, find the closest entry or entries in an IMGT or other reference set as determined by a global alignment.

    :param seq: the input sequence
    :type seq: str
    :param ref: the reference set
    :type ref: dict
    :return: the name of the closest entry, entries, if >1 sequences in the reference set were equally close
    :rtype: list
    """

    scores = [(seq_name, aligned_diff(seq, ref_seq)[2]) for seq_name, ref_seq in ref.items()]
    max_score = max(scores, key=lambda x: x[1])[1]
    closest_names = [seq_name for seq_name, score in scores if score == max_score]

    return closest_names

# Align novel sequence against the reference sequence and check for indels

[docs]def find_indels(closest_ref_seq, novel_seq):
    insertions = []
    fixed_novel = ''        # this will be the novel without any insertions, and now aligned so that there is a '-' for any deletion

    seqA, seqB, _ = aligned_diff(closest_ref_seq, novel_seq)
    seqA = list(seqA)

    for i in range(len(seqA)-1, -1, -1):
        if seqA[i] == '-':
            del seqA[i]
        else:
            break
    seqA = ''.join(seqA)

    for c in zip(list(seqA), list(seqB)):
        if c[0] != '-':
            fixed_novel += c[1]
        else:
            insertions.append([len(fixed_novel) - 1, c[1]])

    if len(seqB) > len(seqA):
        for i in range(len(seqA), len(seqB)):
            fixed_novel += seqB[i]

    return fixed_novel, insertions

# Build (and name) a reference set for sequences truncated at the 5' end
# Start is 0-based index of first available bp

[docs]def build_ambiguous_ref(full_ref, start):
    rev_ref = {}
    for name, seq in full_ref.items():
        seq = '.' * start + seq[start:]
        if seq not in rev_ref:
            rev_ref[seq] = []
        rev_ref[seq].append(name)

    ref = {}
    for seq, names in rev_ref.items():
        if len(names) == 1:
            ref[names[0]] = seq
        else:
            genes = {}
            for name in names:
                gene, allele = name.split('*')
                if gene not in genes:
                    genes[gene] = []
                genes[gene].append(allele)
            if len(genes) == 1:
                name = gene + '*' + '_'.join(sorted(genes[gene]))
                ref[name] = seq
            else:
                gene_list = sorted(genes.keys())
                name = gene_list[0] + '*' + '_'.join(sorted(genes[gene_list[0]]))
                fam_0 = gene_list[0].split('-')[0]
                for i in range(1, len(gene_list)):
                    name += '_'
                    fam = gene_list[i].split('-')[0]
                    num = gene_list[i].replace(fam + '-', '')
                    if fam == fam_0:
                        alleles = [num + '.' + a for a in genes[gene_list[i]]]
                        name += '_'.join(alleles)
                    else:
                        fam = gene_list[i][4:]
                        alleles = [fam + '.' + a for a in genes[gene_list[i]]]
                        name += '_'.join(alleles)
                ref[name] = seq

    return ref


[docs]def run_tests():
    ref_set = simple.read_fasta('Homo_sapiens_IGHV_gapped.fasta')
    test_novels = simple.read_fasta('IGHV3-20.04.fasta')

    for name, seq in test_novels.items():
        novel_name = name_novel(seq, ref_set)[0]
        if novel_name != name:
            print('Error: %s != %s' % (novel_name, name))

if __name__ == "__main__":
    run_tests()
```

---

© Copyright 2022, William Lees.

Built with Sphinx using a
theme
provided by Read the Docs.
