# Supplementary material for: Genotyping of selected germline adaptive immune system loci using short-read sequencing data
Source: Genome Res. 2025 Sep;35(9):2076–86. doi: 10.1101/gr.280314.124 (PMC12401057; doi:10.1101/gr.280314.124)
Supplement: Supplement 1 [file Supplemental_Code.zip › ImmunoTyper2-methods/HPRC-assembly-benchmarking/receptor_utils/docs/_build/html/_modules/receptor_utils/number_v.html]

receptor\_utils.number\_v — receptor-utils documentation


receptor-utils

Contents:

- Introduction
- Using AIRR Community Reference Sets with IgBLAST
- Using AIRR Community Reference Sets with MiXCR
- Using custom databases with IgBlast
- Command-Line Utilities
- receptor\_utils

receptor-utils

- Module code
- receptor\_utils.number\_v

---

# Source code for receptor\_utils.number\_v

```
# Copyright (c) 2021 William Lees

# This source code, and any executable file compiled or derived from it, is governed by the European Union Public License v. 1.2,
# the English version of which is available here: https://perma.cc/DK5U-NDVE

import re
from receptor_utils import simple_bio_seq as simple

[docs]def nt_diff(s1, s2):
    diffs = 0
    row = list(zip(s1.upper(), s2.upper()))

    for p in range(len(row)):
        if row[p][0] != row[p][1] and (row[p][0] not in ['X', 'N'] and row[p][1] not in ['X', 'N']):
            diffs += 1

    diffs += abs(len(s1) - len(s2))
    return diffs

# De novo IMGT-gapping of IGHV germline genes, using guidance from http://www.bioinf.org.uk/abs/info.html#cdrid to confirm CDR1 and 2
# This is experimental. Not used in the examples and specific to IGHV. Maybe a recourse if there is no suitable gapped reference template
# to rely on

[docs]def number_ighv(seq):
    gapped = ''
    # find the conserved Cys at 23
    if 'C' not in seq[21:23]:
        return None, 'First conserved cysteine not found'

    if seq[21] == 'C':
        gapped += seq[:9]
        gapped += '.'
        gapped += seq[9:25]
        next_p = 25
    else:
        gapped += seq[:26]
        next_p = 26

    # find the conserved Trp

    if 'W' not in seq[33:42]:
        return None, 'Conserved Trp not found'

    trp_pos = [pos.start()+33 for pos in re.finditer('W', seq[33:42])]

    max_conf = -1
    best_result = ''
    for pos in trp_pos:
        (confidence, ret) = number_from_trp(seq, next_p, gapped, pos)
        if confidence is not None and confidence > max_conf:
            best_result = ret
            max_conf = confidence

    if max_conf >= 0:
        return best_result, ''
    else:
        return None, 'CDR2 or 2nd Cys not found'


[docs]def number_from_trp(seq, next_p, gapped, trp_pos):
    # add the CDR1 residues
    gapped += distribute_cdr(seq[next_p:trp_pos-2], 12)
    gapped += seq[trp_pos-2:trp_pos+1]
    next_p = trp_pos+1

    # allocate FR2 residues
    gapped += seq[next_p:next_p + 14]
    next_p = next_p + 14        # seq[next_p] is now the first aa of cdr2

    # look for motif trailing cdr2 (should start at position 75)

    found_motif = False
    motif_range = seq[next_p+2+8:next_p+10+8+3]    # allow for cdr2 min length 2, max length 10 and motif length 3
    for match in match_score(motif_range, [['K', 'R'], ['L', 'I', 'V', 'F', 'T', 'A'], ['T', 'S', 'I', 'A']], 2):
        # check for conserved cysteine at position 104
        cys_range = seq[next_p+2+10+match[0]+26:next_p+2+10+match[0]+29]
        if 'C' in cys_range:
            c_index = cys_range.index('C')
            match = next_p + 2 + 10 + match[0]     # now an absolute index of the motif in seq
            found_motif = True
            break

    if not found_motif:
        return (None, None)

    # Build a confidence score based on leading residue sequence
    cdr2_leader = gapped[49:54]
    confidence = match_score(cdr2_leader, [['L'], ['E'], ['W'], ['I'], ['G']], 0)
    if len(confidence) > 0:
        confidence = confidence[0][1]  # only one result, because string lengths are the same

    # add the CDR2 residues
    cdr2 = seq[next_p:match - 10]
    gapped += distribute_cdr(cdr2, 10)

    # add the remaining v-region residues
    gapped += seq[match - 10:]

    # insert gaps in FR3
    if c_index < 2:
        gaps = '.' * (2 - c_index)
        gapped = gapped[:72] + gaps + gapped[72:]

    return (confidence, gapped)


[docs]def match_score(target, match_list, thresh):
    res = []
    for i in range(len(target)-len(match_list) + 1):
        score = 0
        for j in range(len(match_list)):
            if target[i+j] in match_list[j]:
                score += 1

        if score >= thresh:
            res.append((i, score))

    return res

# distribute aas in the cdr according to the numbering scheme

[docs]def distribute_cdr(cdr, length):
    cdr_div = int(len(cdr)/2)

    if 2 * cdr_div == len(cdr):
        return cdr[:cdr_div] + '.' * (length - len(cdr)) + cdr[cdr_div:]
    else:
        return cdr[:cdr_div+1] + '.' * (length - len(cdr)) + cdr[cdr_div+1:]

pretty_header = """
           FR1-IMGT          CDR1-IMGT       FR2-IMGT     CDR2-IMGT                 FR3-IMGT
            (1-26)            (27-38)        (39-55)       (56-65)                  (66-104)
1       10        20         30         40        50         60         70        80        90        100     
.........|.........|...... ...|........ .|.........|..... ....|..... ....|.........|.........|.........|...."""


# insert space before the position pos (zero numbering)

[docs]def insert_space(seq, pos):
    return seq[:pos] + ' ' + seq[pos:]

# pretty print of gapped sequence

[docs]def pretty_gapped(seq):
    seq = insert_space(seq, 65)
    seq = insert_space(seq, 55)
    seq = insert_space(seq, 38)
    seq = insert_space(seq, 26)

    print(pretty_header)
    print(seq)

# Given a gapped AA sequence and an ungapped nt sequence, produce a gapped nt sequence

[docs]def gap_nt_from_aa(nucleotide_seq, peptide_seq):
    codons = [codon for codon in simple.chunks(nucleotide_seq, 3)]  #splits nucleotides into codons (triplets)
    remains = nucleotide_seq[3*len(codons):] if 3*len(codons) != len(nucleotide_seq) else ''
    gapped_codons = []
    codon_count = 0

    for aa in peptide_seq:  #adds '---' gaps to nucleotide seq corresponding to peptide
        if aa not in ('-', '.'):
            gapped_codons.append(codons[codon_count])
            codon_count += 1
        else:
            gapped_codons.append('...')

    while codon_count < len(codons):                # account for trailing nt, eg fragment of a codon
        gapped_codons.append(codons[codon_count])
        codon_count += 1

    return ''.join(gapped_codons) + remains


[docs]def gap_align(seq, ref):
    seq_i = iter(seq)

    res = ''
    started = False

    for r in ref:
        if r != '.':
            started = True
            try:
                res += next(seq_i)
            except StopIteration:
                res += '.'
        else:
            if started:
                res += '.'
            else:
                try:
                    res += next(seq_i)
                except StopIteration:
                    res += '.'

    while True:
        try:
            res += next(seq_i)
        except StopIteration:
            break

    # remove trailing gaps

    i = len(res) - 1

    while i and res[i] == '.':
        i -= 1

    res = res[:i+1]


    return res


[docs]def gap_align_aa(seq, ref):
    seq_i = iter(seq)

    res = ''
    started = False

    for r in ref:
        if r != '.':
            started = True
            try:
                res += next(seq_i)
            except StopIteration:
                break
        else:
            if started:
                res += '.'
            else:
                try:
                    res += next(seq_i)
                except StopIteration:
                    break

    while True:
        try:
            res += next(seq_i)
        except StopIteration:
            break

    return res


[docs]def gap_align_aa_from_nt(aa_seq, nt_gapped):
    aa_i = iter(aa_seq)
    gapped_aa = ''

    started = False

    for codon in simple.chunks(nt_gapped, 3):
        if '.' in codon:
            if started:
                gapped_aa += '.'
            else:
                try:
                    gapped_aa += next(aa_i)
                except StopIteration:
                    break

        else:
            started = True

            try:
                gapped_aa += next(aa_i)
            except StopIteration:
                break

    return gapped_aa

# gap using the closest reference

[docs]def gap_sequence(seq, gapped_ref, ungapped_ref):
    differences = [(k, nt_diff(seq, v)) for k, v in ungapped_ref.items()]
    closest = min(differences, key=lambda x: x[1])[0]

    # print(f'gapping with {closest}')
    res = gap_align(seq, gapped_ref[closest])
    aa = simple.translate(seq)
    aa = gap_align_aa_from_nt(aa, res)

    notes = ''
    # checks
    if 'V' in closest:
        notes = check_conserved_residues(aa)

    return (res, aa, notes)


[docs]def check_conserved_residues(aa):
    notes = []

    if len(aa) < 104:
        return 'Sequence truncated before second cysteine'

    # allow stop codon in final nucleotides without warning
    if '*' in aa:
        pos = aa.index('*')
        if pos <= 103:
            notes.append('Stop codon in V-REGION before 2nd cysteine')

    if aa[22] != 'C':
        notes.append('First cysteine not found')

    if aa[40] != 'W':
        notes.append('Conserved Trp not found')

    if aa[103] != 'C':
        notes.append('Second cysteine not found')

    return ', '.join(notes)


[docs]def run_tests():
    failed = 0

    print('1-64')
    query = 'EMQLVQSEAEVKKPGASVKISCKASGYTFTYRYLHWLRQTPGQGLEWMGWITPYNGNTNYAQKFQDRATITRDRSMSTAYMELSSLRSEDTAVYYCAR'

    (seq, status) = number_ighv(query)

    if seq is not None:
        pretty_gapped(seq)
        if seq == 'EMQLVQSEA.EVKKPGASVKISCKASGYTF....TYRYLHWLRQTPGQGLEWMGWITPY..NGNTNYAQKFQ.DRATITRDRSMSTAYMELSSLRSEDTAVYYCAR':
            print('passed')
        else:
            print('FAILED')
            failed += 1
    else:
        print(query + '->')
        print(status)
        failed += 1


    print('2 -143')
    query = 'QVTLKESGPALVKPTQTLTLTCTFSGFSLTTSGMGVGWIRQPPGKALEWLALIYWDDDKRYSTSLKSRLTISKDTSKNQVVLTMTNMDPMDTATYYCARG'

    (seq, status) = number_ighv(query)

    if seq is not None:
        pretty_gapped(seq)
        if seq == 'QVTLKESGP.ALVKPTQTLTLTCTFSGFSLT..TSGMGVGWIRQPPGKALEWLALIYWD...DDKRYSTSLK.SRLTISKDTSKNQVVLTMTNMDPMDTATYYCARG':
            print('passed')
        else:
            print('FAILED')
            failed += 1
    else:
        print(query + '->')
        print(status)
        failed += 1

    print('3-8')
    query = 'EVQLVESGGGLVQPGRSLRPSCAASGFTFSSYGMHWVRQAPEEGLVWVSYIGSSTMYYADSVKGRFTISRDNAKNSLYLQMNSLRAEDTAVYYCVR'

    (seq, status) = number_ighv(query)

    if seq is not None:
        pretty_gapped(seq)
        if seq == 'EVQLVESGG.GLVQPGRSLRPSCAASGFTF....SSYGMHWVRQAPEEGLVWVSYIGS....STMYYADSVK.GRFTISRDNAKNSLYLQMNSLRAEDTAVYYCVR':
            print('passed')
        else:
            print('FAILED')
            failed += 1
    else:
        print(query + '->')
        print(status)
        failed += 1

    print('4-86')
    query = 'QVQLQESGPGLVKPSETLSLTCAVSGGSISSSNWWSWIRQPPGKGLEWIGRISGSGGSTSDNPSLKSRVTISKDTSKNQFSLKLSSVTAADTAVYYCAR'

    (seq, status) = number_ighv(query)

    if seq is not None:
        pretty_gapped(seq)
        if seq == 'QVQLQESGP.GLVKPSETLSLTCAVSGGSIS...SSNWWSWIRQPPGKGLEWIGRISGS..GGSTSDNPSLK.SRVTISKDTSKNQFSLKLSSVTAADTAVYYCAR':
            print('passed')
        else:
            print('FAILED')
            failed += 1
    else:
        print(query + '->')
        print(status)
        failed += 1

    print('5-16')
    query = 'EVQLVQSGAEVKRPGESLKISCKTSGYSFTSYWISWVRQMPGKGLEWMGAIDPSDSDTRYNPSFQGQVTISADKSISTAYLQWSRLKASDTATYYCAK'

    (seq, status) = number_ighv(query)

    if seq is not None:
        pretty_gapped(seq)
        if seq == 'EVQLVQSGA.EVKRPGESLKISCKTSGYSF....TSYWISWVRQMPGKGLEWMGAIDPS..DSDTRYNPSFQ.GQVTISADKSISTAYLQWSRLKASDTATYYCAK':
            print('passed')
        else:
            print('FAILED')
            failed += 1
    else:
        print(query + '->')
        print(status)
        failed += 1

    print('6-1')
    query = 'QVQLQESGPGLVKPSQTLSLTCAISGDSVSSNSATWNWIRQSPSRGLEWLGRTYYRSKWYNDYAQSVQNRISINPDTSKNQFSLQLNSVTPEDMAVYYCAR'

    (seq, status) = number_ighv(query)

    if seq is not None:
        pretty_gapped(seq)
        if seq == 'QVQLQESGP.GLVKPSQTLSLTCAISGDSVS..SNSATWNWIRQSPSRGLEWLGRTYYRS.KWYNDYAQSVQ.NRISINPDTSKNQFSLQLNSVTPEDMAVYYCAR':
            print('passed')
        else:
            print('FAILED')
            failed += 1
    else:
        print(query + '->')
        print(status)
        failed += 1

    print('7-108')
    query = 'QVQLVQSGAEVKQPGASVKVSCKASGYTFTSYGMNWVRQAHGQRLEWMGWINTDTGNPTYAQGFKERFTFSMDTSISTAYLQISSLKAEDTAVYYCAR'

    (seq, status) = number_ighv(query)

    if seq is not None:
        pretty_gapped(seq)
        if seq == 'QVQLVQSGA.EVKQPGASVKVSCKASGYTF....TSYGMNWVRQAHGQRLEWMGWINTD..TGNPTYAQGFK.ERFTFSMDTSISTAYLQISSLKAEDTAVYYCAR':
            print('passed')
        else:
            print('FAILED')
            failed += 1
    else:
        print(query + '->')
        print(status)
        failed += 1


    if failed == 0:
        print('All tests passed.')
    else:
        print('%d tests failed.' % failed)

if __name__ == "__main__":
    run_tests()
```

---

© Copyright 2022, William Lees.

Built with Sphinx using a
theme
provided by Read the Docs.
