# Supplementary material for: Genotyping of selected germline adaptive immune system loci using short-read sequencing data
Source: Genome Res. 2025 Sep;35(9):2076–86. doi: 10.1101/gr.280314.124 (PMC12401057; doi:10.1101/gr.280314.124)
Supplement: Supplement 1 [file Supplemental_Code.zip › ImmunoTyper2-methods/HPRC-assembly-benchmarking/receptor_utils/docs/_build/html/_modules/receptor_utils/simple_bio_seq.html]

receptor\_utils.simple\_bio\_seq — receptor-utils documentation


receptor-utils

Contents:

- Introduction
- Using AIRR Community Reference Sets with IgBLAST
- Using AIRR Community Reference Sets with MiXCR
- Using custom databases with IgBlast
- Command-Line Utilities
- receptor\_utils

receptor-utils

- Module code
- receptor\_utils.simple\_bio\_seq

---

# Source code for receptor\_utils.simple\_bio\_seq

```
"""Wrapper around BioSeq functions for simple applications
principles:
- store sequences as strings, use dicts for collectiopns
- all sequences are coerced to upper case on input
- iterators are coreced into lists for ease of debugging
basically just make things simple for cases where we don't need to do more
"""

# Copyright (c) 2021 William Lees
__author__ = 'William Lees'

# This source code, and any executable file compiled or derived from it, is governed by the European Union Public License v. 1.2,
# the English version of which is available here: https://perma.cc/DK5U-NDVE

from warnings import warn
from Bio import SeqIO
from Bio.Seq import Seq
from Bio.SeqRecord import SeqRecord
from Bio.Align import MultipleSeqAlignment, AlignInfo
import random
import csv
from collections import Counter

[docs]def chunks(l: str, n: int):
    """ Yield successive n-sized chunks from l.
    """
    for i in range(0, len(l), n):
        yield l[i:i+n]


[docs]def read_fasta(infile: str):
    """Read a FASTA file into a dict

    :param infile: Pathname of the file
    :type infile: str
    :returns: A dictionary indexed by FASTA ID, containing the sequences (in upper case)
    :rtype: dict
    """
    res = {}
    recs = SeqIO.parse(infile, 'fasta')
    for rec in recs:
        res[rec.id] = str(rec.seq).upper()
    return res


[docs]def write_fasta(outfile: str, seqs: dict):
    """Write a dict into a FASTA file. The dict should be indexed by sequence name

    :param outfile: Pathname of the file to be written
    :type outfile: str
    :param seqs: The sequences to write
    :type seqs: dict
    :returns: the number of records written
    :rtype: int
    """
    try:
        SeqIO.write(toSeqRecords(seqs), outfile, 'fasta')
    except:
        SeqIO.write(toSeqRecords(outfile), seqs, 'fasta')
        warn("write_fasta(seqs, outfile) is deprecated. Please use  write_fasta(outfile, seqs) instead", DeprecationWarning, stacklevel=2)


[docs]def read_single_fasta(infile):
    """Read a single sequence from a FASTA file

    :param infile: Pathname of the file
    :type infile: str
    :returns: The first (or only) sequence in the file
    :rtype: str
    """
    recs = SeqIO.parse(infile, 'fasta')
    for rec in recs:
        return str(rec.seq).upper()

# read V,D,J regions from one or more species from an IMGT reference file
# species should be a ***list*** of species
# returns a nested dict: [species][chain]

[docs]def read_imgt_fasta(infile: str, species: str, chains=('IGHV', 'IGHD', 'IGHJ', 'CH'), functional_only: bool =False, include_orphon: bool =False):
    """read V,D,J regions from one or more species from an IMGT reference file

    :param infile: The IMGT reference file
    :type infile: str
    :param species: Species as specified in the file, with spaces replaced by underscore
    :type species: str
    :param chains: List of chains to read (e.g. ['IGHV', 'IGHJ'])
    :type chains: list
    :param functional_only: If True, returns only sequences marked as functional (F)
    :param functional_only: If True, includes orphons
    :return: A dict containing the serquences, indexed by name
    :rtype: dict
    """
    res = {}
    for sp in species:
        res[sp] = {}
        for chain in chains:
            res[sp][chain] = {}

    recs = SeqIO.parse(infile, 'fasta')

    for rec in recs:
        for sp in species:
            for chain in chains:
                if ('-REGION' in rec.description or chain == 'CH' or 'EX' in rec.description) and sp in rec.description and chain in rec.description:
                    if not functional_only or '|F|' in rec.description:
                        if 'V' not in chain or len(rec.seq) > 280:       # strip out obviously incomplete Vs
                            name = rec.description.split('|')[1]
                            if include_orphon or '/OR' not in name:
                                seq_type = rec.description.split('|')[4]

                                if chain == 'CH' or ('EX' in rec.description and chain in rec.description):
                                    gene, allele = name.split('*')
                                    res[sp][chain][gene + '_' + seq_type + '*' + allele] = str(rec.seq).upper()
                                else:
                                    res[sp][chain][name] = str(rec.seq).upper()
    return res


[docs]def sample_fasta(seqs: dict, number: int):
    """Return a random sample of sequences stored in a dict. Sequences are not resampled.
    Returns a ValueError if the dict does not contain enough sequences

    :param seqs: The sequences to sample
    :type seqs: dict
    :param number: The number of sequences to return
    :type number: int
    :return: The sampled sequences
    :rtype: dict
    """
    keys = seqs.keys()

    if len(keys) <= number:
        return seqs

    sample = random.sample(keys, number)
    sample_seqs = {}
    for key in sample:
        sample_seqs[key] = seqs[key]
    return sample_seqs


[docs]def translate(seq: str, truncate: bool = True, ignore_partial_codon: bool = True):
    """Translate a nucleotide sequence to amino acid

    :param seq: the sequence to translate
    :type seq: str
    :param truncate: If True, truncate the sequence so that it terminates on a codon boundary. Otherwise pad with N if necessary
    :type truncate: bool
    :param ignore_partial_codon: If True, if any position in a codon contains - or ., set the entire codon to --- ensuring it gets translated as -
    :type ignore_partial_codon: bool
    :return: Amino acid string
    :rtype: str
    """
    seq_len = len(seq)
    residue = seq_len % 3

    if residue != 0:
        if truncate:
            seq = seq[:seq_len - residue]
        else:
            seq += 'N' * (3 - residue)
            
    if ignore_partial_codon:
        codons = [codon for codon in chunks(seq, 3)]
        i = 0

        for i in range(len(codons)):
            if '-' in codons[i] or '.' in codons[i]:
                codons[i] = '---'
            
        seq = ''.join(codons)

    return str(Seq(seq).translate())


[docs]def reverse_complement(seq: str):
    """Return the reverse complement of a nucelotide sequence

    :param seq: the nucleotide sequence
    :type seq: str
    :return: the reverse complement
    :rtype: str
    """
    return str(Seq(seq).reverse_complement())


[docs]def nt_diff(s1: str, s2: str):
    """Returns a count of the positions in which the input sequences differ. If the sequences are of different lengths, the count only covers
    positions in the shorter sequence.

    :param s1: the first sequence
    :type s1: str
    :param s2: the second sequence
    :type s2: str
    :return: The number of positions at which the sequences differ
    :rtype: int
    """
    diffs = 0
    row = list(zip(s1.upper(), s2.upper()))

    for p in range(len(row)):
        if row[p][0] != row[p][1] and (row[p][0] not in ['X', 'N'] and row[p][1] not in ['X', 'N']):
            diffs += 1

    diffs += abs(len(s1) - len(s2))
    return diffs

# convert our dict format to SeqRecords

[docs]def toSeqRecords(seqs: dict):
    """Convert a dict of sequences to a list of BioPython SeqRecords

    :param seqs: the sequences to convert
    :type seqs: dict
    :return: the BioPython SeqRecords
    :rtype: list
    """
    recs = []
    for name, seq in seqs.items():
        recs.append(SeqRecord(Seq(seq), id=name, description=''))
    return recs


[docs]def dumb_consensus(seqs: dict, threshold: float = 0.7):
    """Return a dumb consensus on a dict of sequences, using Bio.Align.AlignInfo.SummaryInfo.dumb_consensus. All sequences should be the same length.

    :param seqs: the sequences to analyse
    :type seqs: dict
    :param threshold:  the threshold value that is required to add a particular atom
    :type threshold: float
    :return: the consensus sequence
    :rtype: str
    """
    msa = MultipleSeqAlignment(toSeqRecords(seqs))
    summary_align = AlignInfo.SummaryInfo(msa)
    return str(summary_align.gap_consensus(threshold=threshold))


[docs]def scored_consensus(seqs: dict, threshold: float = 0.7):
    """Return a dumb consensus on a dict of sequences, using Bio.Align.AlignInfo.SummaryInfo.gap_consensus. All sequences should be the same length.
    The output is modified to provide, as well as the consensus string, the mimimum score achieved at any position

    :param seqs: the sequences to analyse
    :type seqs: dict
    :param threshold:  the threshold value that is required to add a particular atom
    :type threshold: float
    :return: (consensus sequence, min_score)
    :rtype: str
    """
    msa = MultipleSeqAlignment(toSeqRecords(seqs))
    summary_align = AlignInfo.SummaryInfo(msa)
    return scored_gap_consensus(msa, threshold=threshold)

# Adapted from BioPython.Bio.ALign
# return min_score: this is the lowest proportion of reads that 'won' at any position

[docs]def scored_gap_consensus(alignment, threshold=0.7, ambiguous="X", require_multiple=False):
    """Adapted from BioPython.Bio.ALign. This function is called by receptor_utils.scored_consensus.
    """
    consensus = ""

    # find the length of the consensus we are creating
    con_len = alignment.get_alignment_length()
    con_rows = len(alignment)

    # go through each seq item
    min_score = 1.0
    for n in range(con_len):
        # keep track of the counts of the different atoms we get
        atom_dict = Counter()
        num_atoms = 0

        for record in alignment:
            # make sure we haven't run past the end of any sequences
            # if they are of different lengths
            try:
                c = record[n]
            except IndexError:
                continue
            atom_dict[c] += 1

            num_atoms += 1

        max_atoms = []
        max_size = 0

        for atom in atom_dict:
            if atom_dict[atom] > max_size:
                max_atoms = [atom]
                max_size = atom_dict[atom]
            elif atom_dict[atom] == max_size:
                max_atoms.append(atom)

        if require_multiple and num_atoms == 1:
            consensus += ambiguous
            min_score = min(min_score, 1/con_rows)
        elif (len(max_atoms) == 1) and (
            (float(max_size) / float(num_atoms)) >= threshold
        ):
            consensus += max_atoms[0]
            min_score = min(min_score, float(max_size) / float(con_rows))
        else:
            consensus += ambiguous
            min_score = min(min_score, float(max_size) / float(con_rows))

    return (Seq(consensus), min_score)

# Find the closest reference sequence to a query sequence
# Returns a list of equally close reference names

[docs]def closest_ref(seq: str, ref: dict):
    """Given an input sequence, find the closest entry or entries in an IMGT or other reference set as determined by nt_diff.

    :param seq: the input sequence
    :type seq: str
    :param ref: the reference set
    :type ref: dict
    :return: the name of the closest entry, entries, if >1 sequences in the reference set were equally close
    :rtype: list
    """
    closest_diff = 99999
    closest_names = []
    for ref_name, ref_seq in ref.items():
        diff = nt_diff(seq, ref_seq)
        if diff < closest_diff:
            closest_diff = diff
            closest_names = [ref_name]
        elif diff == closest_diff:
            closest_names.append(ref_name)
    return closest_names

# Read csv file into a list of dicts

[docs]def read_csv(file: str, delimiter: str = None):
    """Read a delimited file into a list of dicts (as produced by DictReader)

    :param file: filename of the file
    :type file: str
    :param delimiter: the delimiter (',' by default)
    :type delimiter: str
    :return: the list of dicts
    :rtype: list
    """
    ret = []
    with open(file, 'r') as fi:
        if delimiter:
            reader = csv.DictReader(fi, delimiter=delimiter)
        else:
            reader = csv.DictReader(fi)
        for row in reader:
            ret.append(row)

    return ret

# Write csv file given a list of dicts. Fieldnames are taken from the first row

[docs]def write_csv(file: str, rows: list, delimiter: str = None, scan_all: bool = False):
    """Write a list of dicts to a delimited file. The header row is determined from the keys of the first item

    :param file: filename of the delimited file to create
    :type file: str
    :param rows: the rows to write
    :type rows: list
    :param delimiter: the delimiter (',' by default)
    :type delimiter: str
    :param scan_all: If True, scan all rows to find the full set of keys
    :type scan_all: bool
    :return: None
    :rtype: None
    """
    if not rows:
        return

    if scan_all:
        fieldnames = list()
        for row in rows:
            for key in row.keys():
                if key not in fieldnames:
                    fieldnames.append(key)       # use lists rather set so we can retain ordering 
    else:
        fieldnames = rows[0].keys()

    with open(file, 'w', newline='') as fo:
        if delimiter:
            writer = csv.DictWriter(fo, fieldnames=list(fieldnames), delimiter=delimiter)
        else:
            writer = csv.DictWriter(fo, fieldnames=fieldnames)

        writer.writeheader()
        
        for row in rows:
            writer.writerow(row)
```

---

© Copyright 2022, William Lees.

Built with Sphinx using a
theme
provided by Read the Docs.
