# Supplementary material for: Genotyping of selected germline adaptive immune system loci using short-read sequencing data
Source: Genome Res. 2025 Sep;35(9):2076–86. doi: 10.1101/gr.280314.124 (PMC12401057; doi:10.1101/gr.280314.124)
Supplement: Supplement 1 [file Supplemental_Code.zip › ImmunoTyper2-methods/HPRC-assembly-benchmarking/receptor_utils/docs/_build/html/airrc_sets_with_igblast.html]

Using AIRR Community Reference Sets with IgBLAST — receptor-utils documentation


receptor-utils

Contents:

- Introduction
- Using AIRR Community Reference Sets with IgBLAST
  - Prerequisites
  - 1. Download the germline set for human IGHV genes and additional IgBLAST files
  - 2. Log in to the Immcantation container so that we can use its installed tools
  - 3. Build IgBLAST databases for the germline set
  - 4. Annotate the sample sequences using IgBLAST
  - 5. Convert the IgBLAST output to Change-O format
- Using AIRR Community Reference Sets with MiXCR
- Using custom databases with IgBlast
- Command-Line Utilities
- receptor\_utils

receptor-utils

- Using AIRR Community Reference Sets with IgBLAST
- View page source

---

# Using AIRR Community Reference Sets with IgBLAST

The download\_germline\_set utility is designed to make it as easy as possible to use AIRR Community germline reference sets from the Open Germline Receptor Database (OGRDB) with IgBLAST.
The utility can download the germline reference set in a format that is compatible with IgBLAST, and create the necessary auxiliary files for IgBLAST to use the germline reference set. In this section we will describe how to
annotate a small example dataset using the germline reference set for human IGHV genes, and the accompanying auxiliary files.

For simplicity, we will use the Immcantation Docker/Singularity container. IgBLAST is installed in the container, together with other Immcantation tools.
If you are not already familiar with the container, you can read about it in the Immcantation documentation. Alternatively, you can install IgBLAST on your local machine and skip the ‘log in’ step below.
To install locally, use the instructions provided on the IgBLAST website. Please make sure that you use a recent version of IgBLAST, as the procedure for using custom databases has been significantly simplified from version 1.20 onwards.

We will annotate a sample set of sequences provided on the Immcantation website.
The sequences have been preprocessed and quality-filtered from Illumina paired-end reads and are present in a FASTA file called `HD13M.fasta`. The process required to create such a file from sequencing reads, will depend on the sequencing protocol.
You can consult the Presto documentation on the Immcantation website, or other sources, to determine a suitable approach for you sequencing data.

## Prerequisites

Before you start, you will need to have the following installed on your machine:

> - Docker or Singularity
> - Python 3.9 or above
> - The receptor-utils package (see Introduction for installation instructions)
> - The file `HD13M.fasta`, extracted from the tarball which you can download here.

The steps we shall follow are as follows:

1. Download the germline set for human IGHV genes and additional IgBLAST files.
2. Log in to the Immcantation container so that we can use its installed tools.
3. Build IgBLAST databases for the germline set.
4. Annotate the sample sequences using IgBLAST.
5. Convert the IgBLAST output to Change-O format.

## 1. Download the germline set for human IGHV genes and additional IgBLAST files

In a suitable directory to use for this test, use the download\_germline\_set utility to download the germline set for human IGHV genes in a format that is compatible with IgBLAST:

```
$ download_germline_set "Homo sapiens" IGH -f MULTI-IGBLAST
https://ogrdb.airr-community.org/api_v2/germline/species
Homo sapiens: 9606
9606.IGH_VDJ
FASTA files saved to Homo_sapiens_IGH_V.fasta, Homo_sapiens_IGH_D.fasta, Homo_sapiens_IGH_J.fasta, Homo_sapiens_IGH_V_gapped.fasta
IgBLAST ndm file saved to Homo_sapiens_IGH.ndm
IgBLAST aux file saved to Homo_sapiens_IGH.aux
```

Finally, extract the file `` HD13M.fasta` `` from the downloaded tarball and copy it to the directory.

## 2. Log in to the Immcantation container so that we can use its installed tools

Log in to the container, mounting the current local directory as /data.

From Linux using Docker:

```
docker run -it -v $(pwd):/data:z immcantation/suite:4.5.0 bash
```

From Windows using Docker:

```
docker run -it -v %cd%:/data:z immcantation/suite:4.5.0 bash
```

For further options, e.g. use with Singularity, please refer to the Immcantation documentation.

Once in the container, cd to /data and check that the reference set files are present:

```
[root@b426e4d7c0ae /]# cd /data
[root@b426e4d7c0ae data]# ls
HD13M.fasta           Homo_sapiens_IGH.ndm      Homo_sapiens_IGH_J.fasta  Homo_sapiens_IGH_V_gapped.fasta
Homo_sapiens_IGH.aux  Homo_sapiens_IGH_D.fasta  Homo_sapiens_IGH_V.fasta
[root@b426e4d7c0ae data]#
```

## 3. Build IgBLAST databases for the germline set

Use IgBLAST’s makeblastdb tool to build the germline databases:

```
makeblastdb -parse_seqids -dbtype nucl -in Homo_sapiens_IGH_V.fasta -out Homo_sapiens_IGH_V
makeblastdb -parse_seqids -dbtype nucl -in Homo_sapiens_IGH_D.fasta -out Homo_sapiens_IGH_D
makeblastdb -parse_seqids -dbtype nucl -in Homo_sapiens_IGH_J.fasta -out Homo_sapiens_IGH_J
```

After these commands have run, you will see many more files in the directory, for example

```
[root@b426e4d7c0ae data]# ls Homo_sapiens_IGH_V.*
Homo_sapiens_IGH_V.fasta  Homo_sapiens_IGH_V.nhr  Homo_sapiens_IGH_V.njs  Homo_sapiens_IGH_V.nos  Homo_sapiens_IGH_V.nsq  Homo_sapiens_IGH_V.nto
Homo_sapiens_IGH_V.ndb    Homo_sapiens_IGH_V.nin  Homo_sapiens_IGH_V.nog  Homo_sapiens_IGH_V.not  Homo_sapiens_IGH_V.ntf
[root@b426e4d7c0ae data]#
```

## 4. Annotate the sample sequences using IgBLAST

Annotate the sequences in `` HD13M.fasta` `` with IgBLAST. We use the verbose output format `7 std qseq sseq btop`. The IGDATA environment variable needs
to be set for IgBLAST to run, but in fact the files in that directory are not used, as we override them with command-line options in order
to use the files downloaded from OGRDB:

```
export IGDATA=/usr/local/share/igblast
igblastn \
    -germline_db_V Homo_sapiens_IGH_V \
    -germline_db_D Homo_sapiens_IGH_D \
    -germline_db_J Homo_sapiens_IGH_J \
    -auxiliary_data Homo_sapiens_IGH.aux \
    -custom_internal_data Homo_sapiens_IGH.ndm \
    -domain_system imgt \
    -outfmt '7 std qseq sseq btop' \
    -num_threads 20 \
    -query HD13M.fasta \
    -out HD13M.fmt7
```

Once IgBLAST has run, you can examine the output file HD13M.fmt7` witn more or another suitable tool, to confirm it contains sequence annotations.

## 5. Convert the IgBLAST output to Change-O format

Run the Immcantation tool MakeDb to create a TSV database of the IgBLAST output. Note that we use the –failed and –log options to capture details of any
sequences that fail annotation or import. Please refer to Using custom databases with IgBlast for some guidance on how to check for errors.

```
MakeDb.py igblast -i HD13M.fmt7 -s HD13M.fasta --failed --log HD13M.log \
    -r Homo_sapiens_IGH_V_gapped.fasta Homo_sapiens_IGH_D.fasta Homo_sapiens_IGH_J.fasta \
    --extended
```

The output should be similar to this:

```
OUTPUT> HD13M_db-pass.tsv
PASS> 7887
FAIL> 1402
END> MakeDb
```

You can review `HD13M_db-pass.tsv` to see the resulting output.

Previous
Next

---

© Copyright 2022, William Lees.

Built with Sphinx using a
theme
provided by Read the Docs.
