# Supplementary material for: Genotyping of selected germline adaptive immune system loci using short-read sequencing data
Source: Genome Res. 2025 Sep;35(9):2076–86. doi: 10.1101/gr.280314.124 (PMC12401057; doi:10.1101/gr.280314.124)
Supplement: Supplement 1 [file Supplemental_Code.zip › ImmunoTyper2-methods/HPRC-assembly-benchmarking/receptor_utils/docs/_build/html/airrc_sets_with_mixcr.html]

Using AIRR Community Reference Sets with MiXCR — receptor-utils documentation


receptor-utils

Contents:

- Introduction
- Using AIRR Community Reference Sets with IgBLAST
- Using AIRR Community Reference Sets with MiXCR
  - Prerequisites
  - 1. Download the germline set for human IGHV genes
  - 1. Log in to the MiXCR container so that we can use its installed tools
  - 3. Build MiXCR database for the germline set
  - 4. Annotate the sample sequences using MiXCR
- Using custom databases with IgBlast
- Command-Line Utilities
- receptor\_utils

receptor-utils

- Using AIRR Community Reference Sets with MiXCR
- View page source

---

# Using AIRR Community Reference Sets with MiXCR

For simplicity, we will use the MiXCR Docker/Singularity container.
If you are not familiar with the container, you can read about it in the MiXCR documentation. Alternatively, you can install MiXCR on your local machine,
using the instructions provided on the MiXCR website. In this case just skip the ‘log in to Docker’ step in the walkthrough.

We will annotate a sample set of sequences provided on the Immcantation website.
The sequences have been preprocessed and quality-filtered from Illumina paired-end reads and are present in a FASTA file called `HD13M.fasta`. The process required to create such a file from sequencing reads, will depend on the sequencing protocol.
You can consult the Mixcr documentation, or other sources, to determine a suitable approach for you sequencing data.

## Prerequisites

Before you start, you will need to have the following installed on your machine:

> - Docker
> - Python 3.9 or above
> - The receptor-utils package (see Introduction for installation instructions)
> - The file `HD13M.fasta`, extracted from the tarball which you can download here.

The steps we shall follow are as follows:

1. Download the AIRR-C germline set for human IGHV genes
2. Log in to the MiXCR container so that we can use its installed tools.
3. Build a MiXCR database for the germline set.
4. Annotate the sample sequences using MiXCR.

## 1. Download the germline set for human IGHV genes

In a suitable directory to use for this test, use the download\_germline\_set utility to download the germline set for human IGHV genes:

```
$ download_germline_set "Homo sapiens" IGH -f MULTI-F
https://ogrdb.airr-community.org/api_v2/germline/species
Homo sapiens: 9606
9606.IGH_VDJ
FASTA files saved to Homo_sapiens_IGH_V.fasta, Homo_sapiens_IGH_D.fasta, Homo_sapiens_IGH_J.fasta, Homo_sapiens_IGH_V_gapped.fasta
```

Finally, extract the file `HD13M.fasta` from the downloaded tarball and copy it to the directory.

## 1. Log in to the MiXCR container so that we can use its installed tools

Log in to the container, mounting the current local directory as /data.

From Linux using Docker:

```
docker run -it -v $(pwd):/work ghcr.io/milaboratory/mixcr/mixcr:latest bash
```

From Windows using Docker:

```
docker run -it -v %cd%:/work ghcr.io/milaboratory/mixcr/mixcr:latest bash
```

Once in the container, cd to /work and check that the reference set files are present:

```
bash-4.2# cd /work
bash-4.2# ls
HD13M.fasta  Homo_sapiens_IGH_D.fasta  Homo_sapiens_IGH_J.fasta  Homo_sapiens_IGH_V.fasta  Homo_sapiens_IGH_V_gapped.fasta
bash-4.2#
```

## 3. Build MiXCR database for the germline set

Set the MiXCR license to match your key:

```
bash-4.2# MI_LICENSE="...your licence key here..."
bash-4.2# export MI_LICENSE
```

Use MiXCR to build the database:

```
bash-4.2# mixcr buildLibrary --debug \
    --v-genes-from-fasta Homo_sapiens_IGH_V.fasta --v-gene-feature VRegion \
    --j-genes-from-fasta Homo_sapiens_IGH_J.fasta \
    --d-genes-from-fasta Homo_sapiens_IGH_D.fasta \
    --chain IGH --taxon-id 9606 --species human \
    human-IGH.json.gz
```

You may see warnings during the build process that stop codons were found in some sequences. This is expected, as some pseudogenes are included in the AIRR-C set. No action is required.

After these commands have run, the database human-IGH.json.gz will be present in the directory.

## 4. Annotate the sample sequences using MiXCR

Annotate the sequences in `HD13M.fasta` with MiXCR. As the sequences are in plain FASTA format, we will use the ‘generic pacbio’ template.

```
bash-4.2# mixcr analyze generic-pacbio -s human \
--library human-IGH \
--assemble-clonotypes-by FR1+CDR1+FR2+CDR2+FR3+CDR3+FR4 \
HD13M.fasta \
HD13M
```

Once MiXCR has run, you can view `HD13M.clones_IGH.tsv` to see the resulting annotations.

Previous
Next

---

© Copyright 2022, William Lees.

Built with Sphinx using a
theme
provided by Read the Docs.
