# Supplementary material for: Genotyping of selected germline adaptive immune system loci using short-read sequencing data
Source: Genome Res. 2025 Sep;35(9):2076–86. doi: 10.1101/gr.280314.124 (PMC12401057; doi:10.1101/gr.280314.124)
Supplement: Supplement 1 [file Supplemental_Code.zip › ImmunoTyper2-methods/HPRC-assembly-benchmarking/receptor_utils/docs/_build/html/annotate_j.html]

annotate\_j — receptor-utils documentation


receptor-utils

Contents:

- Introduction
- Using AIRR Community Reference Sets with IgBLAST
- Using AIRR Community Reference Sets with MiXCR
- Using custom databases with IgBlast
- Command-Line Utilities
  - Naming and Aligning V, D and J Sequences
  - Working with IgBLAST
    - download\_germline\_set
    - make\_igblast\_ndm
    - annotate\_j
      - Positional Arguments
      - Named Arguments
  - Convenience Tools
- receptor\_utils

receptor-utils

- Command-Line Utilities
- annotate\_j
- View page source

---

# annotate\_j

Annotate a set of J sequences, finding the frame alignment and 1-based index of the first nucleotide of the conserved PHE or TRP

```
usage: annotate_j [-h] [--verbose] seq_file out_file
```

## Positional Arguments

`seq_file`
:   J sequences to annotate (fasta or AIRR-C JSON format)

`out_file`
:   Annotation output in aux format for use with IgBlast

## Named Arguments

`--verbose, -v`
:   Verbose output

    Default: False

Previous
Next

---

© Copyright 2022, William Lees.

Built with Sphinx using a
theme
provided by Read the Docs.
