# Supplementary material for: Genotyping of selected germline adaptive immune system loci using short-read sequencing data
Source: Genome Res. 2025 Sep;35(9):2076–86. doi: 10.1101/gr.280314.124 (PMC12401057; doi:10.1101/gr.280314.124)
Supplement: Supplement 1 [file Supplemental_Code.zip › ImmunoTyper2-methods/HPRC-assembly-benchmarking/receptor_utils/docs/_build/html/at_coords.html]

at\_coords — receptor-utils documentation


receptor-utils

Contents:

- Introduction
- Using AIRR Community Reference Sets with IgBLAST
- Using AIRR Community Reference Sets with MiXCR
- Using custom databases with IgBlast
- Command-Line Utilities
  - Naming and Aligning V, D and J Sequences
  - Working with IgBLAST
  - Convenience Tools
    - download\_germline\_set
    - extract\_refs
    - fix\_macaque\_gaps
    - identical\_seqs
    - rev\_comp
    - at\_coords
      - Positional Arguments
      - Named Arguments
    - merge\_fasta
- receptor\_utils

receptor-utils

- Command-Line Utilities
- at\_coords
- View page source

---

# at\_coords

> Extract the sequence at the specified 1-based co-ordinates in the file

```
usage: at_coords [-h] [-n N] [-r] [-xr] input_file start end
```

## Positional Arguments

`input_file`
:   File containing the reference sequence (FASTA)

`start`
:   Start coord (1-based)

`end`
:   End coord (1-based)

## Named Arguments

`-n`
:   Label of sequence in file (must specify if there is more than one sequence in the file)

`-r`
:   Reverse-complement the result

    Default: False

`-xr`
:   Extract the result from the reverse-complement of the sequence (coords refer to the reverse complement sequence)

    Default: False

Previous
Next

---

© Copyright 2022, William Lees.

Built with Sphinx using a
theme
provided by Read the Docs.
