# Supplementary material for: Genotyping of selected germline adaptive immune system loci using short-read sequencing data
Source: Genome Res. 2025 Sep;35(9):2076–86. doi: 10.1101/gr.280314.124 (PMC12401057; doi:10.1101/gr.280314.124)
Supplement: Supplement 1 [file Supplemental_Code.zip › ImmunoTyper2-methods/HPRC-assembly-benchmarking/receptor_utils/docs/_build/html/custom_igblast.html]

Using custom databases with IgBlast — receptor-utils documentation


receptor-utils

Contents:

- Introduction
- Using AIRR Community Reference Sets with IgBLAST
- Using AIRR Community Reference Sets with MiXCR
- Using custom databases with IgBlast
- Command-Line Utilities
- receptor\_utils

receptor-utils

- Using custom databases with IgBlast
- View page source

---

# Using custom databases with IgBlast

Local installations of IgBlast can be configured to support ‘custom organisms’ - either an organism for which no databases are provided with
the tool, or a modified database for a supported organism. The procedure is significantly simplifed in version 1.20, and upgrading to at least this version is encouraged. For full
annotation, two files are required, in addition to the germline database: the auxiliary\_data file, which describes details of the J-genes such as frame orientation, and the ndm file,
which describes the position of the CDRs in V-genes.

If you just wish to add alleles to existing V-genes, or add D-genes or alleles, all that is necessary is to create a custom blast sequence database using the procedure linked above. The ndm and aux files provided
with IgBlast will support this use case. If you wish to add V-genes that are not in the IgBlast database, you will need to create a new ndm file and provide it to IgBlast using the
`--custom_internal_data` command-line parameter. If you wish to modify or add to the J-gene database, you will need to provide a new aux file, using the `--auxiliary_data` command-line
parameter.

make\_igblast\_ndm will make an ndm file from a set of IMGT-gapped V-sequences. You should include all V-sequences in the reference set (if necessary, you can gap them using
gap\_sequences if you have at least a partial or near-matching set for your organism, or for a closely-related organism).

annotate\_j will create an aux file from a set of J-sequences. You should include all sequences in your J gene database.

It is very important to test the annotation carefully when using a custom database. If there are issues with the configuration - particularly issues with the two files above - IgBlast
may fail to annotate a substantial proportion of submitted sequences. My own approach is as follows:

- Annotate a test repertoire using IgBlast output format `-outfmt '7 std qseq sseq btop'`
- Convert to Changeo format using MakeDb. Use the `--failed` option to create a database of failed records. Use the `--log` option to create a log file.
- Examine the count of passed and failed records. Typically, you might expect under 10% of records to fail, possibly many fewer than that, depending on the quality of the reads. A higher count of failed records is a warning sign.
- Open db\_fail, the failed database. Reads that were found to contain a stop codon can be discounted. How many others are there, and what proportion do they represent? If this is more than a few percent, it is a warning sign. Are they obviously short sequences, i.e. not full-length? Again, these can be discounted. Take a sample of reads that look as though they are full-length, and analyse them in IMGT V-Quest, either using your organism of interest, or a closely related one. Does V-Quest find them to be non-functional? If a substantial number of sequences in the failed database are found to be functional by V-Quest, you should strongly suspect a problem in the IgBlast configuration.
- Open the error log, and check the report for any sequences in the failed database that you suspect from the above analysis may have been mis-annotated. You may see the following errors in the log file:

  - **IgBlast does not provide a junction analysis or a J assignment** - this means that the junction analysis and/or J assignment are not in the IgBlast annotation file. If you open the file
    and look at the analysis of the read, you will see that indeed that part of the analysis is missing! There is no error or warning, it is simply not included! This is not an error if the read is
    too short to cover the entire junction, but, if V-quest annotates the sequence as functional, it is likely that the aux file incorrectly specifies one or more J-genes, or is empty.
  - **MakeDb.py: Junction does not match the sequence starting at position 310 in the IMGT numbered V(D)J sequence** - Again this can occur with pseudogenes or corrupted sequences, but if
    V-Quest annotates the sequence as functional, it is likely to indicate a problem in the ndm file, such that the sequence is incorrectly aligned. Another circumstance under which it
    can occur is if MakeDb does not recognise the v, d or j-call as being in the canonical format, i.e. something along the lines of IGHV1-69\*02. Some latitude is allowed, but if you
    are using allele names that depart from this format, check that they are not causing issues. In particular, a name without an allele suffix will cause this problem.
- Once any problems identified in the above steps are resolved, open the ‘pass’ database in Excel or something similar. Check that a reasonable proportion of reads are annotated as functional, and that all V-gene and J-gene families result in some functional arrangements - investigate any exceptions
- Continue to exercise caution as you extend your analysis to more repertoires, being conscious of the possibility of annotation errors in your results.

I hope that this section doesn’t put you off! But it should serve as a warning that you need to be systematic, and on the lookout for problems that are not well flagged
by the tools, and also have an understanding of the underlying data in your repertoire, and what to expect from it.

Previous
Next

---

© Copyright 2022, William Lees.

Built with Sphinx using a
theme
provided by Read the Docs.
