# Supplementary material for: Genotyping of selected germline adaptive immune system loci using short-read sequencing data
Source: Genome Res. 2025 Sep;35(9):2076–86. doi: 10.1101/gr.280314.124 (PMC12401057; doi:10.1101/gr.280314.124)
Supplement: Supplement 1 [file Supplemental_Code.zip › ImmunoTyper2-methods/HPRC-assembly-benchmarking/receptor_utils/docs/_build/html/download_germline_set.html]

download\_germline\_set — receptor-utils documentation


receptor-utils

Contents:

- Introduction
- Using AIRR Community Reference Sets with IgBLAST
- Using AIRR Community Reference Sets with MiXCR
- Using custom databases with IgBlast
- Command-Line Utilities
  - Naming and Aligning V, D and J Sequences
  - Working with IgBLAST
    - download\_germline\_set
      - Positional Arguments
      - Named Arguments
      - Format Options
      - Examples
    - make\_igblast\_ndm
    - annotate\_j
  - Convenience Tools
    - download\_germline\_set
      - Positional Arguments
      - Named Arguments
      - Format Options
      - Examples
    - extract\_refs
    - fix\_macaque\_gaps
    - identical\_seqs
    - rev\_comp
    - at\_coords
    - merge\_fasta
- receptor\_utils

receptor-utils

- Command-Line Utilities
- download\_germline\_set
- View page source

---

# download\_germline\_set

This utility will download reference sequences from the Open Germline Receptor Database (OGRDB).

Download germline sets from the Open Germline Receptor Database (OGRDB)

```
usage: download_germline_set [-h] [-n NAME] [-v VERSION] [-f {AIRRC-JSON,SINGLE-FG,SINGLE-FU,MULTI-F,MULTI-IGBLAST}] [-u URL] [-p PREFIX] species locus
```

## Positional Arguments

`species`
:   Species (e.g. “Homo sapiens”)

`locus`
:   Locus (IGH, IGK, IGL, TRA, TRB, TRD, TRD)

## Named Arguments

`-n, --name`
:   germline set name (the utility will attempt to determine the name, if none is specified)

`-v, --version`
:   Specific version to download, otherwise the latest version will be downloaded

    Default: “latest”

`-f, --format`
:   Possible choices: AIRRC-JSON, SINGLE-FG, SINGLE-FU, MULTI-F, MULTI-IGBLAST

    Format to download

    Default: “AIRRC-JSON”

`-u, --url`
:   URL to use

    Default: “https://ogrdb.airr-community.org/api\_v2”

`-p, --prefix`
:   Prefix for filenames. Default prefix is species\_locus (with \_ substituted for space). If PREFIX is NONE, no prefix will be used for multi files, and the default prefix will be used for single files.

## Format Options

- JSON format: AIRR-C JSON format (default)
- Single FASTA file: all V(D)J sequences in a single FASTA file
- Multiple FASTA files: V, D, J, and gapped V sequences in separate FASTA files
- IgBLAST format: Multiple fasta files, plus IgBLAST germline configuration files

By default, germline sets are downloaded in AIRR-C JSON format. The advantage of this format
is that it contains full information on the germline set including delineation of the V-sequence CDRs and delieation of the J-sequences. This means that it can,
in principle, be loaded into an annotation tool without needing any additional information to be provided to the tool. The json format is also recommended for use
with annotate\_j and make\_igblast\_ndm utilities, which create germline configuration files for IgBLAST. The AIRR-C JSON format can also be
specified with the argument -f AIRRC-JSON.

Another option is to download all V(D)J sequences into a single FASTA file. This can be specified by the -f SINGLE-FG argument, which will download gapped V sequences, or
-f SINGLE-FU, which will download ungapped V sequences. The filename, by default, is, respectively, <species>\_<locus>\_gapped.fasta, or <species>\_<locus>.fasta.
The -p argument can be used to replace the <species>\_<locus>\_ prefix with a custom prefix.

The sequences can also be downloaded into four FASTA files. These are named, by default, <species>\_<locus>\_V.fasta, <species>\_<locus>\_D.fasta, <species>\_<locus>\_J.fasta,
<species>\_<locus>\_V\_gapped.fasta. This is specified by the -f MULTI\_F argument. The -p argument can be used to replace the <species>\_<locus>\_ prefix with a custom prefix.

Finally, the -f MULTI-IGBLAST option will download the four multi files as above, and also create .ndm and .aux files for use with IgBLAST. For further details
on use with IgBLAST please see Using AIRR Community Reference Sets with IgBLAST.

## Examples

Download the latest version of the human IGK germline set in AIRR-C JSON format:

```
download_germline_set -"Homo sapiens" IGK
```

Download the latest version of the human IGH germline set in single FASTA format, with gapped V sequences:

```
download_germline_set "Homo sapiens" IGH -f SINGLE-FG
```

Download the latest version of the mouse C57BL/6 IGH germline set in multiple FASTA format:

```
download_germline_set "Mus musculus" IGH -n "C57BL/6 IGH" -f MULTI-F
```

Note that the utility will try to provide helpful information in the event of a command error:

```
>download_germline_set "Mus musculus" IGH -n "C57BL/6" -f MULTI-F
https://ogrdb.airr-community.org/api_v2/germline/species
Mus musculus: 10090
Error: set C57BL/6 not found for species 10090 locus IGH. Available sets:
BALB/c IGHV, C57BL/6 IGH, C57BL/6 IGHV, CAST/EiJ IGH, LEWES/EiJ IGH, MSM/MsJ IGH, NOD/ShiLtJ IGH, PWD/PhJ IGH, BALB/c IGH
```

Download human IGH files for IgBLAST, using the custom filename prefix AIRRC\_IGH:

```
download_germline_set "Homo sapiens" IGH -f MULTI-IGBLAST -p AIRRC_IGH
```

Previous
Next

---

© Copyright 2022, William Lees.

Built with Sphinx using a
theme
provided by Read the Docs.
