# Supplementary material for: Genotyping of selected germline adaptive immune system loci using short-read sequencing data
Source: Genome Res. 2025 Sep;35(9):2076–86. doi: 10.1101/gr.280314.124 (PMC12401057; doi:10.1101/gr.280314.124)
Supplement: Supplement 1 [file Supplemental_Code.zip › ImmunoTyper2-methods/HPRC-assembly-benchmarking/receptor_utils/docs/_build/html/extract_refs.html]

extract\_refs — receptor-utils documentation


receptor-utils

Contents:

- Introduction
- Using AIRR Community Reference Sets with IgBLAST
- Using AIRR Community Reference Sets with MiXCR
- Using custom databases with IgBlast
- Command-Line Utilities
  - Naming and Aligning V, D and J Sequences
  - Working with IgBLAST
  - Convenience Tools
    - download\_germline\_set
    - extract\_refs
      - Positional Arguments
      - Named Arguments
    - fix\_macaque\_gaps
    - identical\_seqs
    - rev\_comp
    - at\_coords
    - merge\_fasta
- receptor\_utils

receptor-utils

- Command-Line Utilities
- extract\_refs
- View page source

---

# extract\_refs

This utility will extract sections from an IMGT gapped reference file, or download the file and extract. The latest file can also be
downloaded here.

Extract reference files for nominated species from a gapped IMGT reference file

```
usage: extract_refs [-h] [-r REF] [-L LOCUS] [-F] species_name
```

## Positional Arguments

`species_name`
:   species name for IMGT file in quotes, e.g. “Homo sapiens”, “Macaca mulatta”

## Named Arguments

`-r, --ref`
:   gapped imgt reference file. If not specified, the current file will be downloaded from http://www.imgt.org/download/GENE-DB/IMGTGENEDB-ReferenceSequences.fasta-nt-WithGaps-F+ORF+inframeP

`-L, --locus`
:   locus identifier, e.g. “IGH” (default), “IGK”, “TRB”, “TRG”

    Default: “IGH”

`-F, --functional_only`
:   Default: False

Previous
Next

---

© Copyright 2022, William Lees.

Built with Sphinx using a
theme
provided by Read the Docs.
