# Supplementary material for: Genotyping of selected germline adaptive immune system loci using short-read sequencing data
Source: Genome Res. 2025 Sep;35(9):2076–86. doi: 10.1101/gr.280314.124 (PMC12401057; doi:10.1101/gr.280314.124)
Supplement: Supplement 1 [file Supplemental_Code.zip › ImmunoTyper2-methods/HPRC-assembly-benchmarking/receptor_utils/docs/_build/html/fix_macaque_gaps.html]

fix\_macaque\_gaps — receptor-utils documentation


receptor-utils

Contents:

- Introduction
- Using AIRR Community Reference Sets with IgBLAST
- Using AIRR Community Reference Sets with MiXCR
- Using custom databases with IgBlast
- Command-Line Utilities
  - Naming and Aligning V, D and J Sequences
    - name\_allele
    - gap\_sequences
    - fix\_macaque\_gaps
      - Positional Arguments
  - Working with IgBLAST
  - Convenience Tools
    - download\_germline\_set
    - extract\_refs
    - fix\_macaque\_gaps
      - Positional Arguments
    - identical\_seqs
    - rev\_comp
    - at\_coords
    - merge\_fasta
- receptor\_utils

receptor-utils

- Command-Line Utilities
- fix\_macaque\_gaps
- View page source

---

# fix\_macaque\_gaps

IMGT’s gapped FASTA sequences for rhesus macaque IGHV, IGLV and IGKV contain inserted codons relative to the sequences for
most other species. These additional codons in macaque sequences cause problems for downstream tools such as Change-o
that expect features such as CDRs and conserved residues to be present at specific co-ordinates. This utility restores
the ‘standard’ alignment by removing the inserted codons. A small number of sequences in the IMGT sets use these
codons: the tool lists those sequences and removes them from the set. Personally I have not observed these sequences
in rhesus repertoires, but this is something that you may wish to check before using the set produced by the tool.

Remove unwanted inserted codons in IMGT alignment of rhesus macaque IG sequences

```
usage: fix_macaque_gaps [-h] infile outfile chain
```

## Positional Arguments

`infile`
:   gapped file from IMGT

`outfile`
:   gapped file confirming to the customary alignment (without inserted codons)

`chain`
:   IGH, IGK or IGL

Previous
Next

---

© Copyright 2022, William Lees.

Built with Sphinx using a
theme
provided by Read the Docs.
