# Supplementary material for: Genotyping of selected germline adaptive immune system loci using short-read sequencing data
Source: Genome Res. 2025 Sep;35(9):2076–86. doi: 10.1101/gr.280314.124 (PMC12401057; doi:10.1101/gr.280314.124)
Supplement: Supplement 1 [file Supplemental_Code.zip › ImmunoTyper2-methods/HPRC-assembly-benchmarking/receptor_utils/docs/_build/html/gap_sequences.html]

gap\_sequences — receptor-utils documentation


receptor-utils

Contents:

- Introduction
- Using AIRR Community Reference Sets with IgBLAST
- Using AIRR Community Reference Sets with MiXCR
- Using custom databases with IgBlast
- Command-Line Utilities
  - Naming and Aligning V, D and J Sequences
    - name\_allele
    - gap\_sequences
      - Positional Arguments
    - fix\_macaque\_gaps
  - Working with IgBLAST
  - Convenience Tools
- receptor\_utils

receptor-utils

- Command-Line Utilities
- gap\_sequences
- View page source

---

# gap\_sequences

Gap the sequences in a FASTA file, using the closest sequence in a gapped reference set as template.

The tool will only use as a template those sequences in the reference set that are complete at the 5’ end and extend at least to the
second cysteine. Any others will not be used as a template, and neither will any that are apparently non-functional.
Warnings will be given of any sequences with these issues. In most circumstances (that is, if they are only a small
fraction of the total sequences in the reference set), these warnings can be ignored.

Gap the sequences in a FASTA file, using the closest sequence in a gapped reference set as template

```
usage: gap_sequences [-h] inferred_file ref_file out_file
```

## Positional Arguments

`inferred_file`
:   ungapped inferred sequences (fasta)

`ref_file`
:   gapped reference set (fasta)

`out_file`
:   output file containing gapped inferred sequences

Previous
Next

---

© Copyright 2022, William Lees.

Built with Sphinx using a
theme
provided by Read the Docs.
