# Supplementary material for: Genotyping of selected germline adaptive immune system loci using short-read sequencing data
Source: Genome Res. 2025 Sep;35(9):2076–86. doi: 10.1101/gr.280314.124 (PMC12401057; doi:10.1101/gr.280314.124)
Supplement: Supplement 1 [file Supplemental_Code.zip › ImmunoTyper2-methods/HPRC-assembly-benchmarking/receptor_utils/docs/_build/html/genindex.html]

Index — receptor-utils documentation


receptor-utils

Contents:

- Introduction
- Using AIRR Community Reference Sets with IgBLAST
- Using AIRR Community Reference Sets with MiXCR
- Using custom databases with IgBlast
- Command-Line Utilities
- receptor\_utils

receptor-utils

- Index

---

# Index

**A**
| **B**
| **C**
| **D**
| **F**
| **G**
| **I**
| **M**
| **N**
| **P**
| **R**
| **S**
| **T**
| **W**

## A

- aligned\_diff() (in module receptor\_utils.novel\_allele\_name)

## B

- build\_ambiguous\_ref() (in module receptor\_utils.novel\_allele\_name)

## C

|  |  |
| --- | --- |
| - check\_conserved\_residues() (in module receptor\_utils.number\_v) - chunks() (in module receptor\_utils.simple\_bio\_seq) | - closest\_aligned\_ref() (in module receptor\_utils.novel\_allele\_name) - closest\_ref() (in module receptor\_utils.simple\_bio\_seq) |

## D

|  |  |
| --- | --- |
| - distribute\_cdr() (in module receptor\_utils.number\_v) | - dumb\_consensus() (in module receptor\_utils.simple\_bio\_seq) |

## F

- find\_indels() (in module receptor\_utils.novel\_allele\_name)

## G

|  |  |
| --- | --- |
| - gap\_align() (in module receptor\_utils.number\_v) - gap\_align\_aa() (in module receptor\_utils.number\_v) | - gap\_align\_aa\_from\_nt() (in module receptor\_utils.number\_v) - gap\_nt\_from\_aa() (in module receptor\_utils.number\_v) - gap\_sequence() (in module receptor\_utils.number\_v) |

## I

- insert\_space() (in module receptor\_utils.number\_v)

## M

- match\_score() (in module receptor\_utils.number\_v)
- module
  - receptor\_utils
  - receptor\_utils.novel\_allele\_name
  - receptor\_utils.number\_v
  - receptor\_utils.simple\_bio\_seq

## N

|  |  |
| --- | --- |
| - name\_novel() (in module receptor\_utils.novel\_allele\_name) - nt\_diff() (in module receptor\_utils.number\_v)   - (in module receptor\_utils.simple\_bio\_seq) | - number\_from\_trp() (in module receptor\_utils.number\_v) - number\_ighv() (in module receptor\_utils.number\_v) |

## P

- pretty\_gapped() (in module receptor\_utils.number\_v)

## R

|  |  |
| --- | --- |
| - read\_csv() (in module receptor\_utils.simple\_bio\_seq) - read\_fasta() (in module receptor\_utils.simple\_bio\_seq) - read\_imgt\_fasta() (in module receptor\_utils.simple\_bio\_seq) - read\_single\_fasta() (in module receptor\_utils.simple\_bio\_seq) - receptor\_utils   - module - receptor\_utils.novel\_allele\_name   - module | - receptor\_utils.number\_v   - module - receptor\_utils.simple\_bio\_seq   - module - reverse\_complement() (in module receptor\_utils.simple\_bio\_seq) - run\_tests() (in module receptor\_utils.novel\_allele\_name)   - (in module receptor\_utils.number\_v) |

## S

|  |  |
| --- | --- |
| - sample\_fasta() (in module receptor\_utils.simple\_bio\_seq) | - scored\_consensus() (in module receptor\_utils.simple\_bio\_seq) - scored\_gap\_consensus() (in module receptor\_utils.simple\_bio\_seq) |

## T

|  |  |
| --- | --- |
| - toSeqRecords() (in module receptor\_utils.simple\_bio\_seq) | - translate() (in module receptor\_utils.simple\_bio\_seq) |

## W

|  |  |
| --- | --- |
| - write\_csv() (in module receptor\_utils.simple\_bio\_seq) | - write\_fasta() (in module receptor\_utils.simple\_bio\_seq) |

---

© Copyright 2022, William Lees.

Built with Sphinx using a
theme
provided by Read the Docs.
