# Supplementary material for: Genotyping of selected germline adaptive immune system loci using short-read sequencing data
Source: Genome Res. 2025 Sep;35(9):2076–86. doi: 10.1101/gr.280314.124 (PMC12401057; doi:10.1101/gr.280314.124)
Supplement: Supplement 1 [file Supplemental_Code.zip › ImmunoTyper2-methods/HPRC-assembly-benchmarking/receptor_utils/docs/_build/html/identical_seqs.html]

identical\_seqs — receptor-utils documentation


receptor-utils

Contents:

- Introduction
- Using AIRR Community Reference Sets with IgBLAST
- Using AIRR Community Reference Sets with MiXCR
- Using custom databases with IgBlast
- Command-Line Utilities
  - Naming and Aligning V, D and J Sequences
  - Working with IgBLAST
  - Convenience Tools
    - download\_germline\_set
    - extract\_refs
    - fix\_macaque\_gaps
    - identical\_seqs
      - Positional Arguments
    - rev\_comp
    - at\_coords
    - merge\_fasta
- receptor\_utils

receptor-utils

- Command-Line Utilities
- identical\_seqs
- View page source

---

# identical\_seqs

List identical sequences and sub-sequences in a fasta file

```
usage: identical_sequences [-h] input_file
```

## Positional Arguments

`input_file`
:   gapped imgt reference file

Previous
Next

---

© Copyright 2022, William Lees.

Built with Sphinx using a
theme
provided by Read the Docs.
