# Supplementary material for: Genotyping of selected germline adaptive immune system loci using short-read sequencing data
Source: Genome Res. 2025 Sep;35(9):2076–86. doi: 10.1101/gr.280314.124 (PMC12401057; doi:10.1101/gr.280314.124)
Supplement: Supplement 1 [file Supplemental_Code.zip › ImmunoTyper2-methods/HPRC-assembly-benchmarking/receptor_utils/docs/_build/html/introduction.html]

Introduction — receptor-utils documentation


receptor-utils

Contents:

- Introduction
  - Installation
  - Naming and aligning V, D and J sequences
  - Using custom databases with IgBlast
  - Convenience Tools
  - simple\_bio\_seq API
- Using AIRR Community Reference Sets with IgBLAST
- Using AIRR Community Reference Sets with MiXCR
- Using custom databases with IgBlast
- Command-Line Utilities
- receptor\_utils

receptor-utils

- Introduction
- View page source

---

# Introduction

This package contains utilities and code which are useful in working with IG/TR receptor sequences.

## Installation

`pip install receptor-utils`

The module requires Biopython, which needs to be installed separately.

## Naming and aligning V, D and J sequences

name\_allele - Provide a name for a given sequence. The name includes the nearest
germline allele (taken from a reference set supplied to the tool) and
incorporating any SNPs. The naming scheme that it uses is described
here

gap\_sequences - Gap a supplied set of sequences according to the IMGT alignment, warning of any missing conserved residues. It employs a gapped reference set, which must be provided. For each sequence to be gapped, the tool identifies the closest reference sequence, and uses it as a template.

fix\_macaque\_gaps - The IMGT alignment for macaque IG sequences has inserted codons relative to the alignment used for most other species. This can cause problems for downstream tools. This utility removes the additional codons in macaque IG, reverting to the standard alignment

## Using custom databases with IgBlast

These tools are covered in detail in the next section.

make\_igblast\_ndm - Create a custom ndm file for IgBlast

annotate\_j - Create a custom auxiliary data file for IgBlast

## Convenience Tools

download\_germline\_set - Download reference sequences from the Open Germline Receptor Database (OGRDB)

extract\_refs - Download reference sequences from IMGT

identical\_seqs - Report cases where, in a FASTA file, the same sequence is listed more than once with different IDs

rev\_comp - Reverse-complement a nucleotide sequence

at\_coords - Find the sequence at specific co-ordinates within the sequence in a fasta file

merge\_fasta - Merge sequences in two FASTA files, removing duplicates

## simple\_bio\_seq API

This is a set of simple wrappers around commonly-used functions in Biopython which suit my use case and are used in the tools above. They support, for example,
one-line reading and writing of FASTA files and comma/tab-separated files, and manage sequences as strings in a dict, which I find simplifies code compared to the
Biopython functions, at the expense of flexibility which I rarely need. They are documented under API.

Previous
Next

---

© Copyright 2022, William Lees.

Built with Sphinx using a
theme
provided by Read the Docs.
