# Supplementary material for: Genotyping of selected germline adaptive immune system loci using short-read sequencing data
Source: Genome Res. 2025 Sep;35(9):2076–86. doi: 10.1101/gr.280314.124 (PMC12401057; doi:10.1101/gr.280314.124)
Supplement: Supplement 1 [file Supplemental_Code.zip › ImmunoTyper2-methods/HPRC-assembly-benchmarking/receptor_utils/docs/_build/html/make_igblast_ndm.html]

make\_igblast\_ndm — receptor-utils documentation


receptor-utils

Contents:

- Introduction
- Using AIRR Community Reference Sets with IgBLAST
- Using AIRR Community Reference Sets with MiXCR
- Using custom databases with IgBlast
- Command-Line Utilities
  - Naming and Aligning V, D and J Sequences
  - Working with IgBLAST
    - download\_germline\_set
    - make\_igblast\_ndm
      - Positional Arguments
      - Named Arguments
      - Notes
    - annotate\_j
  - Convenience Tools
- receptor\_utils

receptor-utils

- Command-Line Utilities
- make\_igblast\_ndm
- View page source

---

# make\_igblast\_ndm

Make IMGT-delineated igblast ndm file from an IMGT-gapped variable-region reference set or AIRR-C JSON format file.

```
usage: make_igblast_ndm [-h] [--cdr_coords CDR_COORDS] ref_file chain ndm_file
```

## Positional Arguments

`ref_file`
:   gapped reference set (.fasta, .json)

`chain`
:   chain as required by igblast (eg VH)

`ndm_file`
:   output file (ndm)

## Named Arguments

`--cdr_coords, -c`
:   comma-separated list of 1-based co-ordinates for CDR1, CDR2 and CDR3-start (default 79,114,166,195,313)

## Notes

If the reference set is available in AIRR-C JSON format, it is recommended to use this format as it will ensure correct placement of the CDRs using coordinates for each sequence from the file.
All germline sets on OGRDB are available in this format.

If the fasta format is used, V sequences must be IMGT gapped. By default, make\_igblast\_ndm assumes that the gapped alignment follows the IMGT numbering scheme
with no inserted codons. Please note that some reference sets available from IMGT, such as those for rhesus macaque, do contain insertions in the alignment. In this case, you can use the `--cdr_coords` option to
specify coordinates of the CDRs. If in doubt, please analyse one sequence from the gapped set in IMGT V-QUEST and review the codon alignment: if there are insertions, denoted by a codon number followed by A, B, C, etc.,
you will need to specify the CDR coordinates using the `--cdr_coords` option. An alternative is to review the sequences in the reference set and remove the insertions. This may be an option, depending upon
the application, the number of germline sequences that have the insertion, and their usage in your samples. We offer a specific tool, fix\_macaque\_gaps to conduct this realignment for IMGT rhesus macaque sets.

Please note that downstream analysis tools may assume that IMGT-gapped sequences follow the default alignment with no inserted codons. If you use a tool that requires a gapped set of V sequences and there is no
provision to specify CDR coordinates, this is likely to be the case.

Previous
Next

---

© Copyright 2022, William Lees.

Built with Sphinx using a
theme
provided by Read the Docs.
