# Supplementary material for: Genotyping of selected germline adaptive immune system loci using short-read sequencing data
Source: Genome Res. 2025 Sep;35(9):2076–86. doi: 10.1101/gr.280314.124 (PMC12401057; doi:10.1101/gr.280314.124)
Supplement: Supplement 1 [file Supplemental_Code.zip › ImmunoTyper2-methods/HPRC-assembly-benchmarking/receptor_utils/docs/_build/html/merge_fasta.html]

merge\_fasta — receptor-utils documentation


receptor-utils

Contents:

- Introduction
- Using AIRR Community Reference Sets with IgBLAST
- Using AIRR Community Reference Sets with MiXCR
- Using custom databases with IgBlast
- Command-Line Utilities
  - Naming and Aligning V, D and J Sequences
  - Working with IgBLAST
  - Convenience Tools
    - download\_germline\_set
    - extract\_refs
    - fix\_macaque\_gaps
    - identical\_seqs
    - rev\_comp
    - at\_coords
    - merge\_fasta
      - Positional Arguments
- receptor\_utils

receptor-utils

- Command-Line Utilities
- merge\_fasta
- View page source

---

# merge\_fasta

This utility will merge sequences from two fasta files. The output will contain all sequences from file1, and any sequences
from file2 that are not present in file1 (the sequences themselves are compared, rather than the sequence ids).

Merge two fasta files, keeping records in file 2 that do not match sequences in file 1

```
usage: merge_fasta [-h] file_1 file_2 outfile
```

## Positional Arguments

`file_1`
:   first file to merge

`file_2`
:   second file to merge

`outfile`
:   output file

Previous
Next

---

© Copyright 2022, William Lees.

Built with Sphinx using a
theme
provided by Read the Docs.
