# Supplementary material for: Genotyping of selected germline adaptive immune system loci using short-read sequencing data
Source: Genome Res. 2025 Sep;35(9):2076–86. doi: 10.1101/gr.280314.124 (PMC12401057; doi:10.1101/gr.280314.124)
Supplement: Supplement 1 [file Supplemental_Code.zip › ImmunoTyper2-methods/HPRC-assembly-benchmarking/receptor_utils/docs/_build/html/modules.html]

receptor\_utils — receptor-utils documentation


receptor-utils

Contents:

- Introduction
- Using AIRR Community Reference Sets with IgBLAST
- Using AIRR Community Reference Sets with MiXCR
- Using custom databases with IgBlast
- Command-Line Utilities
- receptor\_utils
  - receptor\_utils package

receptor-utils

- receptor\_utils
- View page source

---

# receptor\_utils

- receptor\_utils package
  - Submodules
  - receptor\_utils.novel\_allele\_name module
    - `aligned_diff()`
    - `build_ambiguous_ref()`
    - `closest_aligned_ref()`
    - `find_indels()`
    - `name_novel()`
    - `run_tests()`
  - receptor\_utils.number\_v module
    - `check_conserved_residues()`
    - `distribute_cdr()`
    - `gap_align()`
    - `gap_align_aa()`
    - `gap_align_aa_from_nt()`
    - `gap_nt_from_aa()`
    - `gap_sequence()`
    - `insert_space()`
    - `match_score()`
    - `nt_diff()`
    - `number_from_trp()`
    - `number_ighv()`
    - `pretty_gapped()`
    - `run_tests()`
  - receptor\_utils.simple\_bio\_seq module
    - `chunks()`
    - `closest_ref()`
    - `dumb_consensus()`
    - `nt_diff()`
    - `read_csv()`
    - `read_fasta()`
    - `read_imgt_fasta()`
    - `read_single_fasta()`
    - `reverse_complement()`
    - `sample_fasta()`
    - `scored_consensus()`
    - `scored_gap_consensus()`
    - `toSeqRecords()`
    - `translate()`
    - `write_csv()`
    - `write_fasta()`
  - Module contents

Previous
Next

---

© Copyright 2022, William Lees.

Built with Sphinx using a
theme
provided by Read the Docs.
