# Supplementary material for: Genotyping of selected germline adaptive immune system loci using short-read sequencing data
Source: Genome Res. 2025 Sep;35(9):2076–86. doi: 10.1101/gr.280314.124 (PMC12401057; doi:10.1101/gr.280314.124)
Supplement: Supplement 1 [file Supplemental_Code.zip › ImmunoTyper2-methods/HPRC-assembly-benchmarking/receptor_utils/docs/_build/html/name_allele.html]

name\_allele — receptor-utils documentation


receptor-utils

Contents:

- Introduction
- Using AIRR Community Reference Sets with IgBLAST
- Using AIRR Community Reference Sets with MiXCR
- Using custom databases with IgBlast
- Command-Line Utilities
  - Naming and Aligning V, D and J Sequences
    - name\_allele
      - Positional Arguments
      - Named Arguments
    - gap\_sequences
    - fix\_macaque\_gaps
  - Working with IgBLAST
  - Convenience Tools
- receptor\_utils

receptor-utils

- Command-Line Utilities
- name\_allele
- View page source

---

# name\_allele

Name an allele by comparing to a reference set. Construct a novel name containing SNPs if necessary. Use extract\_refs to create suitable reference sets.

```
usage: name_allele [-h] [-g GENE_NAME] [-r] ref_set sequence
```

## Positional Arguments

`ref_set`
:   reference set containing sequences from a single locus (e.g. IGH, TRB) (FASTA) (V genes should be IMGT-gapped)

`sequence`
:   input sequence

## Named Arguments

`-g, --gene`
:   only consider reference sequences from the specified gene

`-r, --rev_comp`
:   reverse-complement sequence before processing

    Default: False

Previous
Next

---

© Copyright 2022, William Lees.

Built with Sphinx using a
theme
provided by Read the Docs.
