# Supplementary material for: Genotyping of selected germline adaptive immune system loci using short-read sequencing data
Source: Genome Res. 2025 Sep;35(9):2076–86. doi: 10.1101/gr.280314.124 (PMC12401057; doi:10.1101/gr.280314.124)
Supplement: Supplement 1 [file Supplemental_Code.zip › ImmunoTyper2-methods/HPRC-assembly-benchmarking/receptor_utils/docs/_build/html/py-modindex.html]

Python Module Index — receptor-utils documentation


receptor-utils

Contents:

- Introduction
- Using AIRR Community Reference Sets with IgBLAST
- Using AIRR Community Reference Sets with MiXCR
- Using custom databases with IgBlast
- Command-Line Utilities
- receptor\_utils

receptor-utils

- Python Module Index

---

# Python Module Index

**r**

|  |  |  |
| --- | --- | --- |
|  |  |  |
|  | **r** |  |
|  | `receptor_utils` |  |
|  | `receptor_utils.novel_allele_name` |  |
|  | `receptor_utils.number_v` |  |
|  | `receptor_utils.simple_bio_seq` |  |

---

© Copyright 2022, William Lees.

Built with Sphinx using a
theme
provided by Read the Docs.
