# Supplementary material for: Genotyping of selected germline adaptive immune system loci using short-read sequencing data
Source: Genome Res. 2025 Sep;35(9):2076–86. doi: 10.1101/gr.280314.124 (PMC12401057; doi:10.1101/gr.280314.124)
Supplement: Supplement 1 [file Supplemental_Code.zip › ImmunoTyper2-methods/HPRC-assembly-benchmarking/receptor_utils/docs/_build/html/receptor_utils.html]

receptor\_utils package — receptor-utils documentation


receptor-utils

Contents:

- Introduction
- Using AIRR Community Reference Sets with IgBLAST
- Using AIRR Community Reference Sets with MiXCR
- Using custom databases with IgBlast
- Command-Line Utilities
- receptor\_utils
  - receptor\_utils package
    - Submodules
    - receptor\_utils.novel\_allele\_name module
      - `aligned_diff()`
      - `build_ambiguous_ref()`
      - `closest_aligned_ref()`
      - `find_indels()`
      - `name_novel()`
      - `run_tests()`
    - receptor\_utils.number\_v module
      - `check_conserved_residues()`
      - `distribute_cdr()`
      - `gap_align()`
      - `gap_align_aa()`
      - `gap_align_aa_from_nt()`
      - `gap_nt_from_aa()`
      - `gap_sequence()`
      - `insert_space()`
      - `match_score()`
      - `nt_diff()`
      - `number_from_trp()`
      - `number_ighv()`
      - `pretty_gapped()`
      - `run_tests()`
    - receptor\_utils.simple\_bio\_seq module
      - `chunks()`
      - `closest_ref()`
      - `dumb_consensus()`
      - `nt_diff()`
      - `read_csv()`
      - `read_fasta()`
      - `read_imgt_fasta()`
      - `read_single_fasta()`
      - `reverse_complement()`
      - `sample_fasta()`
      - `scored_consensus()`
      - `scored_gap_consensus()`
      - `toSeqRecords()`
      - `translate()`
      - `write_csv()`
      - `write_fasta()`
    - Module contents

receptor-utils

- receptor\_utils
- receptor\_utils package
- View page source

---

# receptor\_utils package

## Submodules

## receptor\_utils.novel\_allele\_name module

receptor\_utils.novel\_allele\_name.aligned\_diff(*novel\_seq: str*, *ref\_seq: str*)[source]

receptor\_utils.novel\_allele\_name.build\_ambiguous\_ref(*full\_ref*, *start*)[source]

receptor\_utils.novel\_allele\_name.closest\_aligned\_ref(*seq: str*, *ref: dict*)[source]
:   Given an input sequence, find the closest entry or entries in an IMGT or other reference set as determined by a global alignment.

    Parameters
    :   - **seq** (*str*) – the input sequence
        - **ref** (*dict*) – the reference set

    Returns
    :   the name of the closest entry, entries, if >1 sequences in the reference set were equally close

    Return type
    :   list

receptor\_utils.novel\_allele\_name.find\_indels(*closest\_ref\_seq*, *novel\_seq*)[source]

receptor\_utils.novel\_allele\_name.name\_novel(*novel\_seq: str*, *ref\_set: dict*, *v\_gene: bool = True*)[source]
:   Make a name for the novel allele, given its gapped or ungapped sequence.
    The name conforms to the description here .
    The sequence must be full-length at the 5 prime end, or gapped

    Parameters
    :   - **novel\_seq** (*str*) – the full-length sequence to name, which may be either gapped or ungapped in the case of a V-gene
        - **ref\_set** (*dict*) – dict of reference genes (gapped in the case of v-genes), in the format returned by read\_fasta
        - **v\_gene** (*bool*) – True only if we are naming a v\_gene

    Returns
    :   tuple consisting of three strings: novel\_name, novel\_seq, notes. novel\_seq is gapped in the case of a V-gene

    Return type
    :   tuple

receptor\_utils.novel\_allele\_name.run\_tests()[source]

## receptor\_utils.number\_v module

receptor\_utils.number\_v.check\_conserved\_residues(*aa*)[source]

receptor\_utils.number\_v.distribute\_cdr(*cdr*, *length*)[source]

receptor\_utils.number\_v.gap\_align(*seq*, *ref*)[source]

receptor\_utils.number\_v.gap\_align\_aa(*seq*, *ref*)[source]

receptor\_utils.number\_v.gap\_align\_aa\_from\_nt(*aa\_seq*, *nt\_gapped*)[source]

receptor\_utils.number\_v.gap\_nt\_from\_aa(*nucleotide\_seq*, *peptide\_seq*)[source]

receptor\_utils.number\_v.gap\_sequence(*seq*, *gapped\_ref*, *ungapped\_ref*)[source]

receptor\_utils.number\_v.insert\_space(*seq*, *pos*)[source]

receptor\_utils.number\_v.match\_score(*target*, *match\_list*, *thresh*)[source]

receptor\_utils.number\_v.nt\_diff(*s1*, *s2*)[source]

receptor\_utils.number\_v.number\_from\_trp(*seq*, *next\_p*, *gapped*, *trp\_pos*)[source]

receptor\_utils.number\_v.number\_ighv(*seq*)[source]

receptor\_utils.number\_v.pretty\_gapped(*seq*)[source]

receptor\_utils.number\_v.run\_tests()[source]

## receptor\_utils.simple\_bio\_seq module

Wrapper around BioSeq functions for simple applications
principles:
- store sequences as strings, use dicts for collectiopns
- all sequences are coerced to upper case on input
- iterators are coreced into lists for ease of debugging
basically just make things simple for cases where we don’t need to do more

receptor\_utils.simple\_bio\_seq.chunks(*l: str*, *n: int*)[source]
:   Yield successive n-sized chunks from l.

receptor\_utils.simple\_bio\_seq.closest\_ref(*seq: str*, *ref: dict*)[source]
:   Given an input sequence, find the closest entry or entries in an IMGT or other reference set as determined by nt\_diff.

    Parameters
    :   - **seq** (*str*) – the input sequence
        - **ref** (*dict*) – the reference set

    Returns
    :   the name of the closest entry, entries, if >1 sequences in the reference set were equally close

    Return type
    :   list

receptor\_utils.simple\_bio\_seq.dumb\_consensus(*seqs: dict*, *threshold: float = 0.7*)[source]
:   Return a dumb consensus on a dict of sequences, using Bio.Align.AlignInfo.SummaryInfo.dumb\_consensus. All sequences should be the same length.

    Parameters
    :   - **seqs** (*dict*) – the sequences to analyse
        - **threshold** (*float*) – the threshold value that is required to add a particular atom

    Returns
    :   the consensus sequence

    Return type
    :   str

receptor\_utils.simple\_bio\_seq.nt\_diff(*s1: str*, *s2: str*)[source]
:   Returns a count of the positions in which the input sequences differ. If the sequences are of different lengths, the count only covers
    positions in the shorter sequence.

    Parameters
    :   - **s1** (*str*) – the first sequence
        - **s2** (*str*) – the second sequence

    Returns
    :   The number of positions at which the sequences differ

    Return type
    :   int

receptor\_utils.simple\_bio\_seq.read\_csv(*file: str*, *delimiter: Optional[str] = None*)[source]
:   Read a delimited file into a list of dicts (as produced by DictReader)

    Parameters
    :   - **file** (*str*) – filename of the file
        - **delimiter** (*str*) – the delimiter (‘,’ by default)

    Returns
    :   the list of dicts

    Return type
    :   list

receptor\_utils.simple\_bio\_seq.read\_fasta(*infile: str*)[source]
:   Read a FASTA file into a dict

    Parameters
    :   **infile** (*str*) – Pathname of the file

    Returns
    :   A dictionary indexed by FASTA ID, containing the sequences (in upper case)

    Return type
    :   dict

receptor\_utils.simple\_bio\_seq.read\_imgt\_fasta(*infile: str*, *species: str*, *chains=('IGHV', 'IGHD', 'IGHJ', 'CH')*, *functional\_only: bool = False*, *include\_orphon: bool = False*)[source]
:   read V,D,J regions from one or more species from an IMGT reference file

    Parameters
    :   - **infile** (*str*) – The IMGT reference file
        - **species** (*str*) – Species as specified in the file, with spaces replaced by underscore
        - **chains** (*list*) – List of chains to read (e.g. [‘IGHV’, ‘IGHJ’])
        - **functional\_only** – If True, returns only sequences marked as functional (F)
        - **functional\_only** – If True, includes orphons

    Returns
    :   A dict containing the serquences, indexed by name

    Return type
    :   dict

receptor\_utils.simple\_bio\_seq.read\_single\_fasta(*infile*)[source]
:   Read a single sequence from a FASTA file

    Parameters
    :   **infile** (*str*) – Pathname of the file

    Returns
    :   The first (or only) sequence in the file

    Return type
    :   str

receptor\_utils.simple\_bio\_seq.reverse\_complement(*seq: str*)[source]
:   Return the reverse complement of a nucelotide sequence

    Parameters
    :   **seq** (*str*) – the nucleotide sequence

    Returns
    :   the reverse complement

    Return type
    :   str

receptor\_utils.simple\_bio\_seq.sample\_fasta(*seqs: dict*, *number: int*)[source]
:   Return a random sample of sequences stored in a dict. Sequences are not resampled.
    Returns a ValueError if the dict does not contain enough sequences

    Parameters
    :   - **seqs** (*dict*) – The sequences to sample
        - **number** (*int*) – The number of sequences to return

    Returns
    :   The sampled sequences

    Return type
    :   dict

receptor\_utils.simple\_bio\_seq.scored\_consensus(*seqs: dict*, *threshold: float = 0.7*)[source]
:   Return a dumb consensus on a dict of sequences, using Bio.Align.AlignInfo.SummaryInfo.gap\_consensus. All sequences should be the same length.
    The output is modified to provide, as well as the consensus string, the mimimum score achieved at any position

    Parameters
    :   - **seqs** (*dict*) – the sequences to analyse
        - **threshold** (*float*) – the threshold value that is required to add a particular atom

    Returns
    :   (consensus sequence, min\_score)

    Return type
    :   str

receptor\_utils.simple\_bio\_seq.scored\_gap\_consensus(*alignment*, *threshold=0.7*, *ambiguous='X'*, *require\_multiple=False*)[source]
:   Adapted from BioPython.Bio.ALign. This function is called by receptor\_utils.scored\_consensus.

receptor\_utils.simple\_bio\_seq.toSeqRecords(*seqs: dict*)[source]
:   Convert a dict of sequences to a list of BioPython SeqRecords

    Parameters
    :   **seqs** (*dict*) – the sequences to convert

    Returns
    :   the BioPython SeqRecords

    Return type
    :   list

receptor\_utils.simple\_bio\_seq.translate(*seq: str*, *truncate: bool = True*, *ignore\_partial\_codon: bool = True*)[source]
:   Translate a nucleotide sequence to amino acid

    Parameters
    :   - **seq** (*str*) – the sequence to translate
        - **truncate** (*bool*) – If True, truncate the sequence so that it terminates on a codon boundary. Otherwise pad with N if necessary
        - **ignore\_partial\_codon** (*bool*) – If True, if any position in a codon contains - or ., set the entire codon to — ensuring it gets translated as -

    Returns
    :   Amino acid string

    Return type
    :   str

receptor\_utils.simple\_bio\_seq.write\_csv(*file: str*, *rows: list*, *delimiter: Optional[str] = None*, *scan\_all: bool = False*)[source]
:   Write a list of dicts to a delimited file. The header row is determined from the keys of the first item

    Parameters
    :   - **file** (*str*) – filename of the delimited file to create
        - **rows** (*list*) – the rows to write
        - **delimiter** (*str*) – the delimiter (‘,’ by default)
        - **scan\_all** (*bool*) – If True, scan all rows to find the full set of keys

    Returns
    :   None

    Return type
    :   None

receptor\_utils.simple\_bio\_seq.write\_fasta(*outfile: str*, *seqs: dict*)[source]
:   Write a dict into a FASTA file. The dict should be indexed by sequence name

    Parameters
    :   - **outfile** (*str*) – Pathname of the file to be written
        - **seqs** (*dict*) – The sequences to write

    Returns
    :   the number of records written

    Return type
    :   int

## Module contents

Previous

---

© Copyright 2022, William Lees.

Built with Sphinx using a
theme
provided by Read the Docs.
