# Supplementary material for: Genotyping of selected germline adaptive immune system loci using short-read sequencing data
Source: Genome Res. 2025 Sep;35(9):2076–86. doi: 10.1101/gr.280314.124 (PMC12401057; doi:10.1101/gr.280314.124)
Supplement: Supplement 1 [file Supplemental_Code.zip › ImmunoTyper2-methods/HPRC-assembly-benchmarking/receptor_utils/docs/_build/html/scripts.html]

Command-Line Utilities — receptor-utils documentation


receptor-utils

Contents:

- Introduction
- Using AIRR Community Reference Sets with IgBLAST
- Using AIRR Community Reference Sets with MiXCR
- Using custom databases with IgBlast
- Command-Line Utilities
  - Naming and Aligning V, D and J Sequences
    - name\_allele
    - gap\_sequences
    - fix\_macaque\_gaps
  - Working with IgBLAST
    - download\_germline\_set
    - make\_igblast\_ndm
    - annotate\_j
  - Convenience Tools
    - download\_germline\_set
    - extract\_refs
    - fix\_macaque\_gaps
    - identical\_seqs
    - rev\_comp
    - at\_coords
    - merge\_fasta
- receptor\_utils

receptor-utils

- Command-Line Utilities
- View page source

---

# Command-Line Utilities

## Naming and Aligning V, D and J Sequences

- name\_allele
- gap\_sequences
- fix\_macaque\_gaps

## Working with IgBLAST

- download\_germline\_set
- make\_igblast\_ndm
- annotate\_j

## Convenience Tools

- download\_germline\_set
- extract\_refs
- fix\_macaque\_gaps
- identical\_seqs
- rev\_comp
- at\_coords
- merge\_fasta

Previous
Next

---

© Copyright 2022, William Lees.

Built with Sphinx using a
theme
provided by Read the Docs.
