# Supplementary material for: Genotyping of selected germline adaptive immune system loci using short-read sequencing data
Source: Genome Res. 2025 Sep;35(9):2076–86. doi: 10.1101/gr.280314.124 (PMC12401057; doi:10.1101/gr.280314.124)
Supplement: Supplement 1 [file Supplemental_Code.zip › ImmunoTyper2-methods/HPRC-assembly-benchmarking/receptor_utils/docs/_build/html/search.html]

Search — receptor-utils documentation


receptor-utils

Contents:

- Introduction
- Using AIRR Community Reference Sets with IgBLAST
- Using AIRR Community Reference Sets with MiXCR
- Using custom databases with IgBlast
- Command-Line Utilities
- receptor\_utils

receptor-utils

- Search

---

Please activate JavaScript to enable the search functionality.

---

© Copyright 2022, William Lees.

Built with Sphinx using a
theme
provided by Read the Docs.
