# Supplementary material for: Genotyping of selected germline adaptive immune system loci using short-read sequencing data
Source: Genome Res. 2025 Sep;35(9):2076–86. doi: 10.1101/gr.280314.124 (PMC12401057; doi:10.1101/gr.280314.124)
Supplement: Supplement 1 [file Supplemental_Code.zip › ImmunoTyper2-methods/HPRC-assembly-benchmarking/vquest/test_vquest/data/test_vquest/TestVquestInvalid/response.dat]

IMGT/V-QUEST


http://www.imgt.org

# WELCOME ! to IMGT/V-QUEST

## IMGT®, the international ImMunoGeneTics information system®

**Citing IMGT/V-QUEST**:  
Brochet, X. et al., Nucl. Acids Res. 36, W503-508 (2008).
PMID: 18503082.
  
Giudicelli, V., Brochet, X., Lefranc, M.-P., Cold Spring Harb Protoc. 2011 Jun 1;2011(6). pii: pdb.prot5633. doi: 10.1101/pdb.prot5633.  
PMID: 21632778
Abstract
also in *IMGT booklet with generous provision from
Cold Spring Harbor (CSH) Protocols*
 (high res)
 (lower res)

IMGT/V-QUEST program version: 3.5.21 (1 December 2020) - IMGT/V-QUEST reference directory release: 202049-2 (1 December 2020)

## Analyse your IG (or antibody) or TR nucleotide sequences

The list of the IMGT/V-QUEST reference directory sets to which your sequences can be compared is available
in here.

Human sequence sets to test IMGT/V-QUEST are available here

### Your selection

- The receptor type or locus is not available for this species

Species
---
Homo sapiens (human)
Mus musculus (house mouse)
Aotus nancymaae (Ma's night monkey)
Bos taurus (bovine)
Camelus dromedarius (Arabian camel)
Canis lupus familiaris (dog)
Capra hircus (goat)
Chondrichthyes
Danio rerio (zebrafish)
Equus caballus (horse)
Felis catus (domestic cat)
Gadus morhua (Atlantic cod)
Gallus gallus (chicken)
Ictalurus punctatus (Channel catfish)
Macaca fascicularis (crab-eating macaque)
Macaca mulatta (Rhesus monkey)
Mustela putorius furo (ferret)
Nonhuman Primates
Oncorhynchus mykiss (rainbow trout)
Ornithorhynchus anatinus (platypus)
Oryctolagus cuniculus (rabbit)
Ovis aries (sheep)
Rattus norvegicus (Norway rat)
Salmo salar (Atlantic salmon)
Sus scrofa (pig)
Teleostei
Tursiops truncatus (Bottlenose dolphin)
Vicugna pacos (alpaca)
Receptor type or locus
---

### Sequence submission

Type (or copy/paste) your nucleotide sequence(s) in
FASTA format
>IGKV2-ACR\*02
291GACATTGTGATGACCCAGACTCCACTCTCCCTGCCCGTCACCCCTGGAGAGCCAGCC
TCCATCTCCTGCAGGTCTAGTCA292GAGCCTCTTGGATAGTGACGGGTACACCTGTTTG
GACTGGTACCTGCAGAAGCCAGGCCAGTCTCCACAGCTCCTGATCT293ATGAGGTTTCC
AACCGGGTCTCTGGAGTCCCTGACAGGTTCAGTGGCAGTGGGTCAGNCACTGATTTCACA
CTGAAAATC294AGCCGGGTGGAAGCTGAGGATGTTGGGGTGTATTACTGTATGCAAAGT
ATAGAGTTTCCTCC

Or give the path access to a local file containing your sequence(s)
in FASTA format

### Display results

A. Detailed view

HTML

Text

Nb of nucleotides per line in alignments:

60
90
120
150
10000
Nb of aligned reference sequences:

1
2
3
4
5
6
7
8
9
10
11
12
13
14
15
16
17
18
19
20

1. Alignment for V-GENE
2. Alignment for D-GENE
3. Alignment for J-GENE
4. Results of IMGT/JunctionAnalysis
     
   with full list of eligible D-GENE
     
   without list of eligible D-GENE
5. Sequence of the JUNCTION ('nt' and 'AA')

6. V-REGION alignment
7. V-REGION translation
8. V-REGION protein display
9. V-REGION mutation and AA change table
10. V-REGION mutation and AA change statistics
11. V-REGION mutation hotspots

12. Sequences of V-, V-J- or V-D-J- REGION ('nt' and 'AA') with gaps in FASTA
      
    and access to IMGT/PhyloGene for V-REGION ('nt')
13. Annotation by IMGT/Automat
14. IMGT Collier de Perles
      
    link to IMGT/Collier-de-Perles tool
      
    IMGT/Collier de Perles (for a nb of sequences < 5)
      
    no IMGT/Collier-de-Perles

B. Synthesis view

HTML

Text

Nb of nucleotides per line in alignments:

60
90
120
150
10000
Summary table sequence order:

V-GENE and allele name
input

1. Alignment for V-GENE
2. V-REGION alignment
3. V-REGION translation
4. V-REGION protein display

5. V-REGION protein display (with AA class colors)
6. V-REGION protein display (only AA changes displayed)
7. V-REGION most frequently occurring AA
8. Results of IMGT/JunctionAnalysis

C. Excel file
Open in a spreadsheet
Download in a zip archive
Display 1 CSV file in your browser
Download AIRR formatted results

1. Summary
2. IMGT-gapped-nt-sequences
3. nt-sequences
4. IMGT-gapped-AA-sequences
5. AA-sequences
6. Junction

7. V-REGION-mutation-and-AA-change-table
8. V-REGION-nt-mutation-statistics
9. V-REGION-AA-change-statistics
10. V-REGION-mutation-hotspots
11. Parameters
12. scFv (only for option "Analysis of single chain Fragment variable (scFv)")

### Advanced parameters

Selection of IMGT reference directory set

F+ORF
F+ORF+ in-frame P
F+ORF including orphons
F+ORF+ in-frame P including orphons

With all alleles
With allele \*01 only

Search for insertions and deletions in V-REGION
Yes
No

Parameters for IMGT/JunctionAnalysis

Nb of accepted D-GENE in IGH (default is 1), TRB (default is 1) or TRD (default is 3) JUNCTION

default
0
1
2
3

Nb of accepted mutations:

default
0
1
2
3
4
5
6
7
8
9
10
in 3'V-REGION

default
0
1
2
3
4
5
6
7
8
9
10
in D-REGION

default
0
1
2
3
4
5
6
7
8
9
10
in 5'J-REGION

---

Parameters for "Detailed view"
Nb of nucleotides to exclude in 5' of the V-REGION for the evaluation of the nb of mutations (in results 9 and 10)

Nb of nucleotides to add (or exclude) in 3' of the V-REGION for the evaluation of the alignment score (in results 1)

### Advanced functionalities

Analysis of single chain Fragment variable (scFv)
Yes
No

Clinical application: search for CLL subsets #2 and #8
Yes
No

IMGT/V-QUEST Documentation
IMGT/V-QUEST Search page

IMGT Home page |
IMGT Repertoire (IG and TR),
(MH),
(RPI) |
IMGT Index |
IMGT Scientific chart |
IMGT Education
IMGT Latest news

  
  
  

© Copyright 1995-2021 IMGT®, the international ImMunoGeneTics information system®
| Terms of use | About us | Contact us | Citing IMGT
